# ﻿Monograph of wild and cultivated chili peppers (*Capsicum* L., Solanaceae)

**DOI:** 10.3897/phytokeys.200.71667

**Published:** 2022-06-14

**Authors:** Gloria E. Barboza, Carolina Carrizo García, Luciano de Bem Bianchetti, María V. Romero, Marisel Scaldaferro

**Affiliations:** 1 Instituto Multidisciplinario de Biología Vegetal (CONICET-Universidad Nacional de Córdoba), Casilla de Correo 495, 5000 Córdoba, Argentina Instituto Multidisciplinario de Biología Vegetal Córdoba Argentina; 2 Empresa Brasileira de Pesquisa Agropecuária—Centro Nacional de Pesquisa de Recursos Genéticos e Biotecnologia (EMBRAPA—Recursos Genéticos e Biotecnologia), PqEB Parque Estação Biológica, Av. W/5 final, Brasília-DF, CEP 70770–917, Caixa Postal 02372, Brazil Centro Nacional de Pesquisa de Recursos Genéticos e Biotecnologia Brasília Brazil; 3 Facultad de Ciencias Exactas, Físicas y Naturales, Universidad Nacional de Córdoba, Córdoba, Argentina Universidad Nacional de Córdoba Córdoba Argentina

**Keywords:** America, *Capsicum*, chili peppers, cytogenetics, morphology, phylogeny, taxonomy

## Abstract

*Capsicum* L. (tribe Capsiceae, Solanaceae) is an American genus distributed ranging from the southern United States of America to central Argentina and Brazil. The genus includes chili peppers, bell peppers, ajíes, habaneros, jalapeños, ulupicas and pimientos, well known for their economic importance around the globe. Within the Solanaceae, the genus can be recognised by its shrubby habit, actinomorphic flowers, distinctive truncate calyx with or without appendages, anthers opening by longitudinal slits, nectaries at the base of the ovary and the variously coloured and usually pungent fruits. The highest diversity of this genus is located along the northern and central Andes. Although *Capsicum* has been extensively studied and great advances have been made in the understanding of its taxonomy and the relationships amongst species, there is no monographic treatment of the genus as a whole. Based on morphological and molecular evidence studied from field and herbarium specimens, we present here a comprehensive taxonomic treatment for the genus, including updated information about morphology, anatomy, karyology, phylogeny and distribution. We recognise 43 species and five varieties, including *C.mirum* Barboza, **sp. nov.** from São Paulo State, Brazil and a new combination *C.muticum* (Sendtn.) Barboza, **comb. nov.**; five of these taxa are cultivated worldwide (C.annuumL.var.annuum, C.baccatumL.var.pendulum (Willd.) Eshbaugh, C.baccatumL.var.umbilicatum (Vell.) Hunz. & Barboza, *C.chinense* Jacq. and *C.frutescens* L.). Nomenclatural revision of the 265 names attributed to chili peppers resulted in 89 new lectotypifications and five new neotypifications. Identification keys and detailed descriptions, maps and illustrations for all taxa are provided.

## ﻿Introduction

*Capsicum* L., with 43 species, is placed in the tribe Capsiceae (subfam. Solanoideae, Solanaceae) together with *Lycianthes* (Dunal) Hassl. It is native to temperate, subtropical and tropical regions of the Americas, growing from the southern United States of America to central Argentina and Brazil, with the primary centre of diversity in the Andes.

*Capsicum* is an important crop genus, comprising the chili peppers, bell peppers, ajíes, habaneros, jalapeños, ulupicas or pimientos, with five main domesticated species: *C.annuum* L., *C.chinense* Jacq. and *C.frutescens* L., now widely cultivated throughout the world and *C.baccatum* L. and *C.pubescens* Ruiz & Pav., cultivated predominantly in South America. The genus comprises a diverse group of sweet and hot chili peppers, which have been used as spices since 6000–6500 BCE (Perry et al. 2007; Kraft et al. 2014). Today, they are of great commercial interest and are consumed by one fourth of the global population (Masud Parvez 2017).

Chili peppers are known for their high nutritional value, health benefits and medicinal properties (Saleh et al. 2018). The fruits are highly appreciated not only for their taste and colour (Thampi 2003; Jarret et al. 2019), but also because of their essential oils and the presence of capsaicinoids (Pruthi 2003; Meckelmann et al. 2013). Capsaicinoids are the pungent principles of hot chili peppers, of which capsaicin is the most abundant (Mazourek et al. 2009). These bioactive compounds have various pharmacological effects, such as the treatment of chronic neuropathic pains and many other ailments (Papoiu and Yosipovitch 2010; Aza-González et al. 2011; Al-Snafi 2015; Saleh et al. 2018). In addition, the ornamental use of chili peppers as potted or bedding plants, particularly in the case of the most widely-cultivated species *C.annuum*, is gaining popularity in the ornamental plant market in many countries (Stommel and Griesbach 2005; Costa et al. 2019).

The genus is characterised by a distinctive truncate calyx, without or with appendages (vein prolongations) borne below the entire margin and by variously coloured and usually pungent berries (Hunziker 2001). Traditionally, *Capsicum* was placed in tribe Solaneae Miers (Wettstein 1891; Hunziker 1979, 2001), based on morphological grounds (valvate aestivation, straight filaments, dorsifixed to basifixed anthers, juicy fruits and annular to coiled embryos). Emphasising calyx vasculature and structure (D’Arcy 1986), D’Arcy and Averett (1996) proposed a new tribe Capsiceae D’Arcy, with *Capsicum* and *Lycianthes* as the core group of this tribe, but they also suggested the inclusion of another eight genera (*Acnistus* Schott, *Dunalia* Kunth, *Aureliana* Sendtn., *Iochroma* Benth., *Saracha* Ruiz & Pav., *Tubocapsicum* (Wettst.) Makino, *Vassobia* Rusby and *Witheringia* L’Hér.), based on possession of calyx features at least superficially similar to *Capsicum* (D’Arcy 1991). Hunziker (2001) maintained *Capsicum* within tribe Solaneae, which he divided into five subtribes, placing *Capsicum* within subtribe Capsicinae T.Yamaz. and *Lycianthes* within subtribe Solaninae Wetts., a view that has not been accepted by later workers.

In the molecular phylogenetic reconstructions of Solanaceae (Olmstead et al. 2008; Särkinen et al. 2013), *Capsicum* and *Lycianthes* are resolved as sister taxa and are now the only members of tribe Capsiceae (subfam. Solanoideae). Although acceptance of *Capsicum* as a monophyletic group has not been questioned, its relationship with *Lycianthes* is still unresolved (Carrizo García et al. 2016). However, a recent genomic study analysing transcriptome sequences in members of both genera suggests possible paraphyly of *Lycianthes* with respect to *Capsicum* (Spalink et al. 2018). If upheld with further study, this result has important implications for the taxonomy of tribe *Capsiceae*; either circumscription of *Capsicum* should be enlarged to include *Lycianthes* or *Lycianthes* should be split into different genera that are monophyletic (Spalink et al. 2018).

Although *Capsicum* has been extensively studied and great advances have been made in the understanding of its taxonomy and the relationships amongst the species, there is no taxonomic monograph of the genus as a whole. As part of ongoing projects to revise the genera *Capsicum* and *Lycianthes*, we present here a comprehensive taxonomic treatment of *Capsicum*, including updated information about morphology, anatomy, karyology, phylogeny and distribution and a revision of the nomenclature and typification of the 265 names in the genus. An identification key and descriptions of wild and domesticated taxa, together with distribution maps and illustrations for each, are provided.

## ﻿Circumscription and infrageneric classification of *Capsicum*

Since Linnaeus (1753), there has been no agreement about the circumscription of *Capsicum*. *Capsicum* species have been moved back and forth between several genera: *Acnistus*, *Athenaea* Sendtn., *Brachistus* Miers, *Bassovia* Aubl., *Withania* Pauq., *Witheringia* and others. Dunal was the first botanist to confuse generic concepts of *Capsicum* by describing some species under *Witheringia* (Dunal 1816), an error perpetuated by Kunth (1818) and Walpers (1844) or under *Bassovia* (Dunal 1852). Hiern (1877), Johnston (1905) and Rusby (1927) also described *Capsicum* species under *Bassovia.*Miers (1849) was mistaken in interpreting the deeply 5-lobed calyx of *Brachistus* or the slightly 3–5-lobed calyx of *Witheringia* (Bohs 2015) as homologous with the entire calyx with appendages of *Capsicum* (Hunziker 2001) and he named some species now recognised as *Capsicum* under *Brachistus* (newly-described species or transferred from *Witheringia*). Other authors also added species recognised today as *Capsicum* under *Brachistus* (Watson 1890; Greenman 1903; Stewart 1911; Rusby 1912; Svenson 1946), *Acnistus* (Dammer 1905), *Fregirardia* Dunal ex Delile (Delile 1849; Dunal 1852) or *Solanum* L. (Van Heurck and Müller Argoviensis 1870). Conversely, a number of present-day species, placed in a variety of Solanaceae genera (i.e. *Vassobia*, *Witheringia*, *Athenaea*) and even in other families (*Pombalia* Vand., Violaceae), were originally described as members of *Capsicum* (see Excluded names). The circumscription of the genus began to stabilise with the publication of complete treatments for morphologically similar genera, such as *Lycianthes* (Bitter 1919; Dean 1995), *Witheringia* (Hunziker 1969a; Sousa-Peña 2001), *Brachistus* (D’Arcy et al. 1981), *Acnistus* (Hunziker 1982), *Cuatresia* Hunz. (Hunziker 1987), *Aureliana* (Hunziker and Barboza 1990) and *Tubocapsicum* (Zhang et al. 1994; Zhang and Lu 1999). The information provided in these taxonomic works allowed Hunziker (2001) to propose a convincing circumscription of *Capsicum*, based on the diagnostic characters of an entire calyx with or without appendices, staminal plaque (= stapet) with auricles, anthers with longitudinal dehiscence, annular nectary at the base of the ovary, usually piquant (pungent) berries, a pericarp usually possessing giant cells and berries lacking stone cells.

There has been little agreement on an infrageneric classification of *Capsicum*. Kuntze (1891) proposed three sections: 1. *Bassovia* (Aubl.) Kuntze, 2. *Eucapsicum* Kuntze and 3. *Poecilochroma* (Miers) Kuntze, creating a heterogeneous group where *Aureliana* (= *Athenaea*), *Witheringia*, *Saracha*, *Brachistus*, *Lycianthes* and *Capsicum* species were mixed. This classification was based on flower size and the ratio of anther to filament length. At the same time, Wettstein (1891) divided *Capsicum* into two sections according to corolla shape: *Eucapsicum* Wettst. (30 American species) and the monospecific section Tubocapsicum Wettst. that included only *C.anomalum* Franch. & Sav., a species native to Japan (= *Tubocapsicumanomalum* (Franch. & Sav.) Makino).

Bitter (1919, 1921, 1922) placed species of Capsicum with 10 linear calyx appendages in his section Decameris Bitter (*C.dusenii* Bitter, *C.brachypodum* (Dunal) Kuntze, *C.eggersii* Bitter), but he never proposed an infrageneric classification across the whole of *Capsicum*.

The taxonomic division of *Capsicum* was re-examined in Hunziker’s *Capsicum* synopsis (Hunziker 1956), presented in the International Botanical Congress held at Paris in 1954. He recognised three sections, comprising sect. Tubocapsicum (today, a valid genus with two species, D’Arcy et al. 2001), the monotypic sect. Pseudocapsicum Hunz. for *C.breviflorum* (Sendtn.) Hunz. (presently *Vassobiabreviflora* (Sendtn.) Hunz.) and sect. Capsicum including 24 wild species, one variety and three cultivated species. Eshbaugh (1980) accepted this sectional classification suggesting other members in sect. Capsicum (22 wild species and five domesticated, with their varieties), in the light of new discoveries.

The amount of new evidence produced in recent years has allowed considerable progress in the characterisation of infrageneric groups in *Capsicum*. Some attempts to group the species were made, based on cytogenetic studies (Moscone et al. 2007 and references therein), as well as a combination of data from enzyme, crossing and molecular studies (e.g. Walsh and Hoot 2001; Carrizo García et al. 2016).

**Figure 1.**
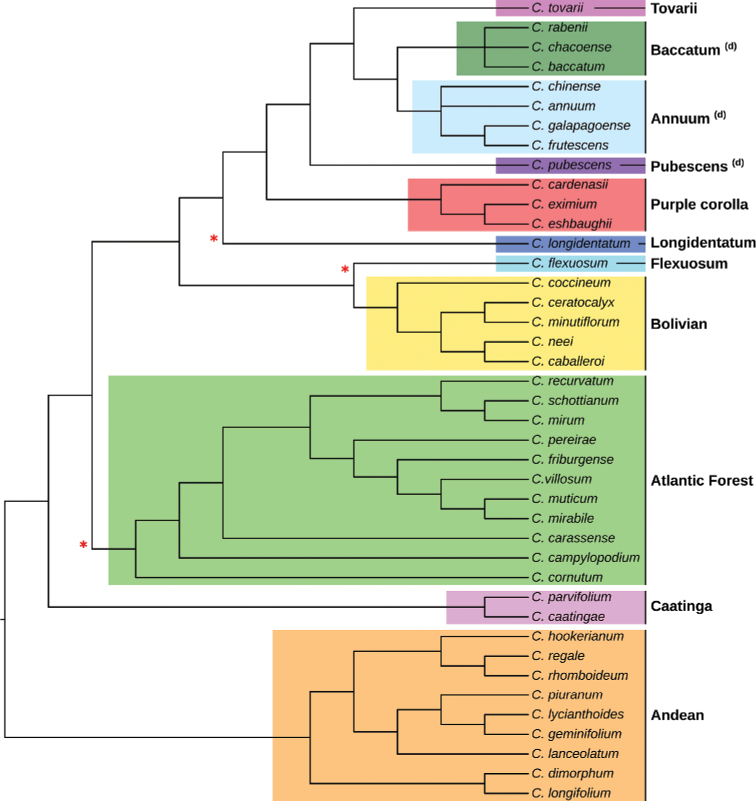
*Capsicum* phylogeny. Cladogram summarising findings from Carrizo García et al. (2016) and Barboza et al. (2019, 2020b). Only one sample per species has been included; species names have been updated. Clades distinguished are labelled. Asterisks indicate branches with moderate/low support, letters ‘d’ clades with domesticated taxa.

Species relationships in *Capsicum* have been analysed following a phylogenetic approach using a range of molecular data. Several early phylogenetic hypotheses involved and primarily concerned domesticated species, although they also included a small number of wild ones (Walsh and Hoot 2001; Jarret and Dang 2004; Guzmán et al. 2009). A comprehensive phylogenetic analysis that included 34 of the 35 species recognised at that time was published in 2016 (Carrizo García et al. 2016). Based on that study and subsequent additions (Barboza et al. 2019, 2020b, eleven major clades are resolved within *Capsicum* and given informal clade names: Andean, Caatinga, Flexuosum, Bolivian, Longidentatum, Atlantic Forest, Purple Corolla, Pubescens, Tovarii, Baccatum and Annuum (Fig. 1). Two main lineages can be distinguished in the genus: one formed by the early diverging Andean clade that includes species from Central America and north-western South America and the other including the remaining species of the genus (Table 1). The domesticated species and their closest relatives are resolved in this second lineage and contained within the Annuum, Baccatum and Pubescens clades, which form the most derived branches (Fig. 1). Data on phylogenetic affinities and clades to which each species have been assigned are detailed in the species descriptions and in Table 1.

**Table 1.** *Capsicum* clades and species composition (after Carrizo García et al. 2016, Barboza et al. 2019, 2020b; *placement unassigned, **placement suggested in this work, based on morphology).

Clade	Species
**Unassigned**	*C.benoistii* Hunz. ex Barboza *
**Andean**	*C.dimorphum* (Miers) Kuntze
	*C.geminifolium* (Dammer) Hunz.
	*C.hookerianum* (Miers) Kuntze
	*C.lanceolatum* (Greenm.) C.V.Morton & Standl.
	*C.longifolium* Barboza & S.Leiva
	*C.lycianthoides* Bitter
	*C.piuranum* Barboza & S.Leiva
	*C.regale* Barboza & Bohs
	*C.rhomboideum* (Dunal) Kuntze
**Atlantic Forest**	*C.campylopodium* Sendtn.
	*C.carassense* Barboza & Bianch.
	*C.cornutum* (Hiern) Hunz.
	*C.friburgense* Bianch. & Barboza
	*C.hunzikerianum* Barboza & Bianch. **
	*C.mirabile* Mart.
	*C.mirum* Barboza
	*C.muticum* (Sendtn.) Barboza
	*C.pereirae* Barboza & Bianch.
	*C.recurvatum* Witasek
	*C.schottianum* Sendtn.
	*C.villosum* Sendtn.
**Flexuosum**	*C.flexuosum* Sendtn.
**Caatinga**	*C.caatingae* Barboza & Agra
	*C.parvifolium* Sendtn.
**Longidentatum**	*C.longidentatum* Agra & Barboza
**Bolivian**	*C.caballeroi* M.Nee
	*C.ceratocalyx* M.Nee
	*C.coccineum* (Rusby) Hunz.
	*C.minutiflorum* (Rusby) Hunz.
	*C.neei* Barboza & X.Reyes
**Purple Corolla**	*C.cardenasii* Heiser & P.G.Sm.
	*C.eshbaughii* Barboza
	*C.eximium* Hunz.
**Pubescens**	*C.pubescens* Ruiz & Pav.
**Tovarii**	*C.tovarii* Eshbaugh, P.G.Sm. & Nickrent
**Baccatum**	*C.baccatum* L.
	*C.chacoense* Hunz.
	*C.rabenii* Sendtn.
**Annuum**	*C.annuum* L.
	*C.chinense* Jacq.
	*C.frutescens* L.
	*C.galapagoense* Hunz.

## ﻿Taxonomic history of *Capsicum*

The quest of Europeans for the “Indies” (namely the Americas) was accompanied by the discovery of new aromatic plants that extensively enriched cuisines around the world; amongst these were the chili peppers (reviewed in Andrews 1984). The first written documentation of these spices is that of Diego Alvárez Chanca, physician on Columbus’ second voyage to the West Indies. In a letter he wrote in 1494 to the Town Council of Seville, Spain, describing the principal events that occurred during the voyage, he referred to the ‘agi’ (word in Spanish) as a spice that the native Indians of Hispaniola employed to season their foods (American Journeys Collection 2003).

Fifty years after Columbus’ first voyage to the West Indies, Leonhard Fuchs, a German physician and botanist, published the first three scientific illustrations of chili peppers (Fuchs 1542). His illustrations (Calechutifcher Pfeffer, Langer Indianifcher Pfeffer and Greyter Indianifcher Pfeffer) were all given Pliny’s name of ‘Siliquastrum’ (large, elongate fruit), because he did not know the geographic origin of the plants (Bostock and Riley 1856) and he associated them with the spices native to India that were known at that time (Piperitis and Siliquastrum).

The word ‘capsicum’ was coined in the pre-Linnaean literature for the first time by Matthias de L’Obel (1576: 173) for the “Piper indicum longioribus siliquis”. L’Obel also provided two illustrations of fertile plants (*C.annuum* in its wild and domesticated forms). Pre-Linnaean botanists (e.g. Dodoens 1554, 1583; Clusius 1611; Bauhin 1623; Parkinson 1640; Morison 1669) proposed many polynomials for the peppers emphasising the variable fruit characters (colour, shape, size, position or pungency). Joseph Pitton de Tournefort (1719), in his influential work *Institutiones*, used the name *Capsicum*, gave an original description for the genus and listed 27 polynomial species that corresponded to his concept of the genus. He also mentioned the etymology of the word *Capsicum*, either from the Greek word δάγκωμα (= to bite), on account of the burning strength of the seeds or from the Latin voice *capsa* (= box), on account of the boxy shape of the cultivated fruits.

Linnaeus (1753) took Tournefort’s *Capsicum* as the generic name of peppers and reduced the number of species to two, the herbaceous annual *C.annuum* and the shrubby perennial *C.frutescens.* In subsequent works, Linnaeus (1767) added two more species, *C.baccatum* and *C.grossum* L., Jacquin (1776) described *C.chinense*, Ruiz and Pavón (1799)*C.pubescens* and Willdenow (1809) proposed 10 species, amongst them *C.pendulum* Willd. By the beginning of the 19^th^ century, the five currently accepted domesticated *Capsicum* species had already been described.

Subsequently the taxonomy of the genus has been complicated by both generic circumscription (see above) and by differing species concepts. Opinions as to the number of taxa that belong to *Capsicum* range from as many as 61 species (plus infraspecific taxa) in the genus (e.g. Dunal 1852) to as few as two (Irish 1898) or one (Dierbach 1829; Bailey 1923; Shinners 1956). Understandably, there has been underlying disagreement amongst taxonomists about whether to accept emerging names as well as the different intricate infraspecific classifications proposed for the domesticated species (e.g. see Dierbach 1829; Terpó 1966). Further complications included: 1) the majority of the new taxa were described, based on the very wide range of variation observed in the fruit morphology of cultivated plants, which is highly influenced by human selection; 2) most herbarium specimens of *Capsicum* were inadequate, many consisting only of fruits and lacking flowers, the most critical and useful organ for identification purposes; and 3) type specimens for many names were difficult to locate or did not exist.

### ﻿Taxonomy of the domesticated species

After Linnaeus, the British gardener Philip Miller (1768) recognised 10 domesticated species, five of them new species described by him. The descriptions of all these species were almost certainly made from living plants cultivated in the Chelsea Physic Garden in London.

The German botanist, Johann Heinrich Dierbach (1829), proposed a particularly confusing infraspecific classification. He accepted a single species and validated the pre-Linnaean name “*Capsicumindicum*” of L’Obel (1576), as *C.indicum* Dierb. He recognised infraspecific unranked taxa designated by numbers (1. macrocarpon, 2. pachycarb[p]on, 3. cerasocarpon, 4. elaeocarpon, 5. microcarpon), based on fruit shape; none of these numbered names had a direct reference to the author or a specimen. Within each numbered category, the different taxa were designated with a letter (a to e; e.g. *C.indicum* Lobel. 1. macrocarpon b. longum) and, in addition, before the designation of letters, the taxa were grouped according to the pericarp colour (e.g. *pericarpiis rubris*, *pericarpiis flavis*). Heiser and Pickersgill (1975) attempted to interpret Dierbach’s classification system, concluding that it “… should be ignored for nomenclatural purposes”. However, Dierbach based the lettered infraspecific taxa on the names of the chili peppers known at that time, with the result that many of them are now synonyms (especially under *C.annuum*) or as doubtful names (see Doubtful names).

The first illustrated monograph of *Capsicum* was published by the German botanist, Karl Anton Fingerhuth (1832), in which he included 32 species. He recognised the taxa already described by previous authors and described only three new species (*C.cumanense* Fingerh., *C.strictum* Fingerh. and *C.ceratocarpum* Fingerh.). He also recognised 29 varieties, most of which were newly described. Although he did not propose a formal infrageneric classification, he grouped the species into two groups (A and B) depending on the position of the fruits (A. Fruits erect; B. Fruits pendent). Within each group, he further separated the species into two subgroups (a. Fruits oblong; b. Fruits subglobose). The distinctions of his many varieties were based on the broad range of different fruit shapes. None of the taxa proposed by Fingerhuth is an accepted name in this treatment (see synonymy under the domesticated species).

In his monumental treatment of Solanaceae, Michel-Felix Dunal (1852) recognised 61 *Capsicum* species (16 newly described by him), together with 51 varieties (16 new), 11 species requiring further study and three doubtful names. From these, 44 species and 40 varieties referred to the domesticated species. Dunal did not establish an infrageneric classification, but grouped the species in a similar way as had Fingerhuth (1832), first by the position of the fruits (erect or pendent) and then by shape (oblong or globose-ovate).

In extreme contrast, other authors tended to reduce the number of accepted species to two (Irish 1898) or only one (Bailey 1923; Erwin 1929; Shinners 1956; and others), with very complex infraspecific hierarchical systems, based on the extreme variation in fruit characters of the domesticated taxa and their cultivars. Terpó (1966) summarised some of these elaborate systems, while Eshbaugh (1980) expressed the difficulties inherent in developing a useful and rational system that satisfied everyone. *Capsicumannuum* and *C.frutescens* and, to a lesser extent for *C.chinense*, have been the taxa most debated by taxonomists and horticulturists in classifications below the species level. From an evolutionary perspective, the relationships between these three species have been the most debated (Pickersgill 1988), whereas there was a general agreement that *C.pubescens* and C.baccatumvar.pendulum were two well-defined domesticated taxa (Eshbaugh 1980; Pickersgill 1988).

As a crop genus, *Capsicum* has inspired researchers to follow a number of in-depth approaches since the mid-1900s, such as classical and molecular cytogenetic analyses, crossing experiments, biochemical and protein electrophoretic studies, molecular characterisation through genotypic markers (restriction fragment length polymorphism, RFLP, amplified fragment length polymorphism, AFLP, random amplified polymorphic DNA, RAPD, microsatellite or simple sequence repeat, SSR, random amplified microsatellite polymorphism, RAMPO and direct amplification of minisatellite DNA, DAMDPCR), phytogeographic and phylogeographic analyses, chloroplast and nuclear DNA and whole-genome sequencing studies (see Moscone et al. 2007; Aguilar-Meléndez et al. 2009; Carvalho et al. 2014; Carrizo García et al. 2016; Raveendar et al. 2017; Scaldaferro et al. 2018; Tripodi et al. 2019; and references in each). This evidence has contributed to a better understanding of the complex taxonomy of the domesticated (and wild) species, as well as to determine the origin of the crop species. Most current taxonomic works and breeding programmes recognise five domesticated species of *Capsicum* (Pickersgill 2016).

### ﻿Taxonomy of the wild species

Taxonomic work on wild *Capsicum* species began in the 19^th^ century. Initially, some authors (Cavanilles 1802; Willdenow 1809; Dunal 1816; Kunth 1818; Roemer and Schultes 1819; and others) described isolated species. The *Capsicum* treatment by Sendtner (1846) in “Flora Brasiliensis” is the first significant work from an extended geographical area; he accepted seven wild species and five infraspecific taxa in addition to several cultivated species. Later, Dunal (1852) made an important contribution with the addition of seven other novel wild species and three varieties.

During the early 20^th^ century, sporadic descriptions of new *Capsicum* taxa continued (Chodat 1902; Chodat and Hassler 1903; Witasek 1910; Bitter 1919, 1921, 1922, 1924). Later, a new generation of taxonomists interested in this genus, led by Paul Smith and Charles Heiser, Jr. (in the United States) and Armando T. Hunziker (in Argentina), made great advances in the understanding of the generic limits of *Capsicum* (see above). These authors not only provided new names, but also new evidence to establish the relationships between species. Hunziker, especially, contributed greatly to a comprehensive understanding of the taxonomy of the genus (Hunziker 1950, 1956, 1961, 1969b, 1971, 1998, 2001). In his book “Genera Solanacearum”, Hunziker (2001) proposed *Capsicum* as a natural group with “ca. 20 species and a few varieties”.

In the last two decades, field explorations across South America, mainly in the central Andean countries and Brazil, have enabled us to gain a better understanding of the genus as a whole. Thirteen new wild species have been described and partial keys for the identification of the species for particular areas have been provided (Barboza and Bianchetti 2005; Nee et al. 2006; Barboza et al. 2011, 2019; Barboza et al. 2020a, Barboza et al. 2020b). We recognise here 43 species of *Capsicum* (including the domesticates), with new species still awaiting discovery in herbaria and in the field (especially in Peru and Bolivia).

## ﻿Morphology

### ﻿Habit and stems

Members of *Capsicum* plants are erect (e.g. *C.schottianum*, *C.geminifolium*), compact (e.g. *C.chacoense*, C.annuumvar.annuum) or somewhat prostrate (e.g. C.annuumvar.glabriusculum). They are subshrubs or shrubs, rarely trees (e.g. *C.rhomboideum*), short-lived perennials (e.g. *C.chinense*) or annual herbs (e.g. C.annuumvar.annuum). *Capsicumcoccineum* is unusual in being sprawling vines or scrambling shrubs. Stems are woody at the base (1.5–2.5 cm in diameter, rarely more) and some species have fissured bark and lenticels (e.g. *C.rhomboideum*, *C.hookerianum*); young stems are angular, herbaceous, usually hollow and weak and, occasionally, somewhat scrambling, range from glabrous to densely pubescent and may have anthocyanin along their length. The nodes are inflated and commonly green or purple.

**Figure 2.**
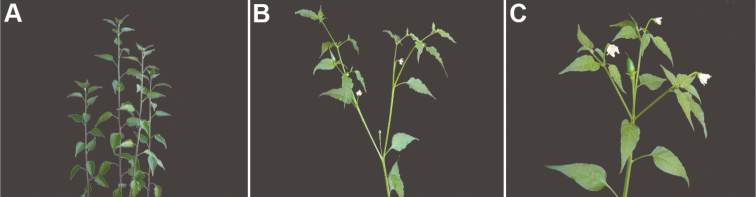
Plant development in *C.chacoense***A** monopodial vegetative growth **B** first dichotomy of the main stem and start of sympodial growth **C** initial branching with three branches. Photos by G.E. Barboza.

*Capsicum* plants have typical solanaceous sympodial growth, giving the stems a “zig-zag” appearance. Initially, the vegetative growth is monopodial and the first stem to emerge has 8–39 leaves (*C.annuum* cultivars, Dorland and Went 1947; Patel and Shah 1977; Bosland and Votava 2000; *C.chacoense*, Barboza, pers. obs.) before the onset of sympodial ramification and flowering (Fig. 2A); the alternate leaves are arranged in a 2/5 phyllotaxic spiral. The main stem ends in a solitary flower or in a flower and one or two leaves (Fig. 2B, C). The number of leaves in a sympodial unit can be unifoliate or difoliate. In difoliate sympodial units, the leaves are geminate with the leaf pair slightly dissimilar in shape or size (e.g. *C.cardenasii*) or markedly dissimilar (e.g. *C.dimorphum*, *C.lycianthoides*) in size and/or shape.

### ﻿Leaves

Species of *Capsicum* have simple leaves that are generally membranous or less frequently coriaceous (e.g. *C.hunzikerianum*, *C.longifolium*, *C.pereirae*), concolourous to discolourous, ovate to elliptical, rarely lanceolate or narrowly elliptical (*C.longifolium*, *C.carassense*) in outline; in taxa with geminate leaves, the minor leaves can be orbicular and sessile (*C.dimorphum*, *C.lycianthoides*). Leaf margins are always entire, rarely slightly revolute (*C.caballeroi*, *C.ceratocalyx*, *C.hunzikerianum*) and the leaf base is asymmetric, attenuate or truncate and sometimes decurrent on to the petiole (*C.piuranum*, *C.rabenii*). Leaf apices are obtuse or acute to acuminate or long-acuminate in few species (e.g. *C.benoistii*, *C.piuranum*, *C.hunzikerianum*). Petioles are longer in the domesticated species and in the major leaves of geminate leaf pairs in wild species.

Leaf and petiole anatomy of *Capsicum* members were investigated in domesticated species (Idu and Ogbe 1997; Weryszko-Chmielewsk and Michałojć 2011; Dias et al. 2013; Wahua et al. 2014) and, more extensively, in 13 wild species from different biogeographic environments (Palchetti et al. 2014). Most of the studied species have amphistomatic leaves, but leaves are hypostomatic in some *C.frutescens* and *C.annuum* cultivars, *C.caatingae*, *C.cornutum* and *C.geminifolium* (Idu and Ogbe 1997; Palchetti et al. 2014). Anisocytic, anomocytic and hemiparacytic stomatal types were reported; this variation was observed within a same leaf, a common feature in Solanaceae (Metcalfe and Chalk 1957; Bessis and Guyot 1979; Karatela and Gill 1986). Palchetti et al. (2014) described leaf blades with dorsiventral mesophyll, sparse to abundant calcium oxalate crystals (druses, solitary crystals and/or crystal sands), bicollateral vascular bundles surrounded by parenchyma, fibres or collenchyma and petioles with arch- or U-shaped vascular bundles. Leaf epidermal variables and the internal structural differences of leaf and petiole were not significantly different in the 13 wild species from different environments (Palchetti et al. 2014), but some anatomical characters were useful for the identification (stomata position and types, type of trichomes, type of cells surrounding the vascular bundle in the leaf, presence/absence and abundance and types of crystals and others).

### ﻿Pubescence

**Figure 3.**
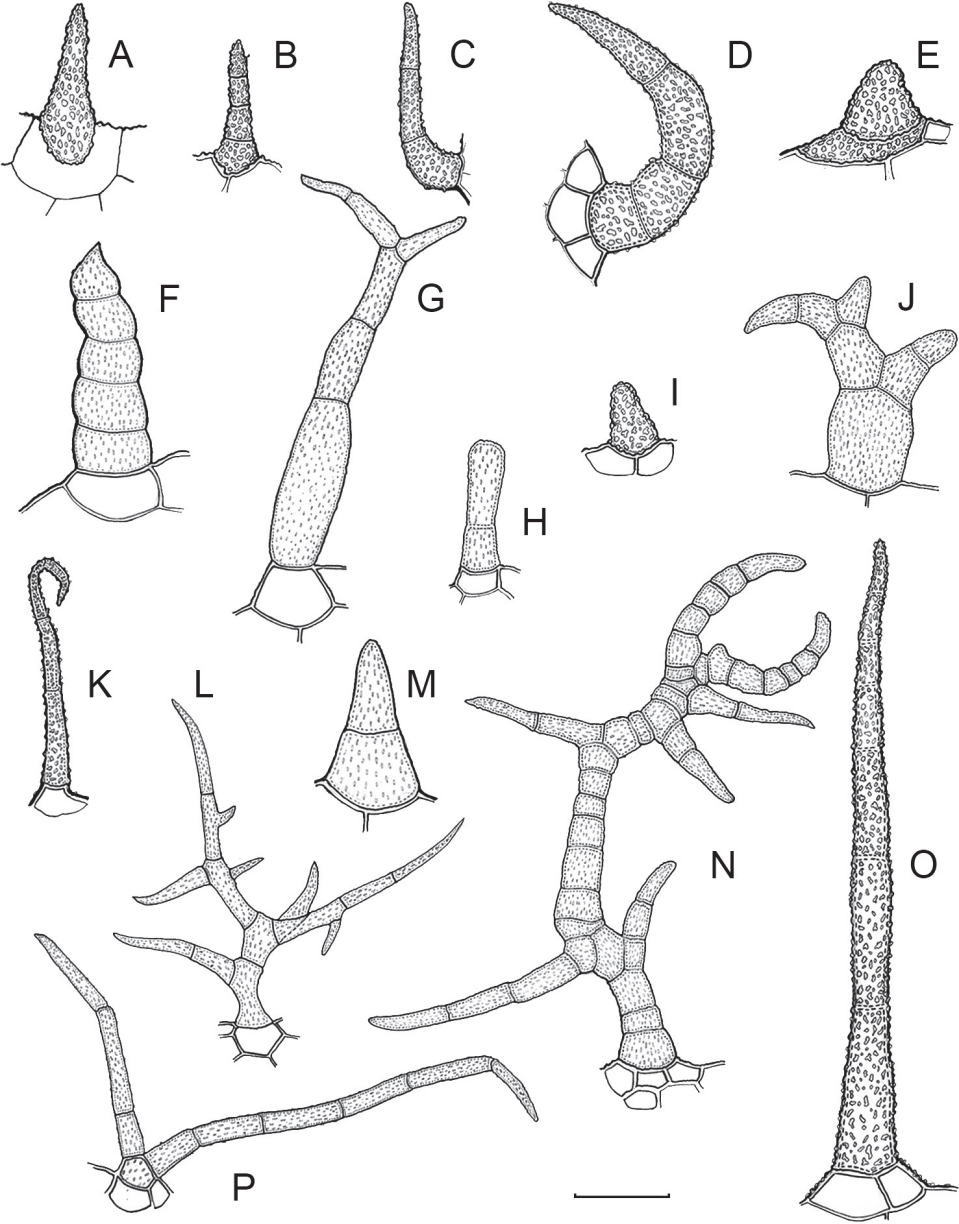
Eglandular trichomes of *Capsicum* species **A–D, F, H, K, M, O** simple trichomes **E, I** papillae **G, J, L, N, P** branched trichomes. Scale bar: 50 μm (**A, B, E, H–J**); 100 μm (**C, D, F, G, K–P**).

Trichomes in *Capsicum* are mostly eglandular and simple, although branched trichomes can also occur (e.g. *C.longidentatum*, *C.rhomboideum* and *C.parvifolium*). Simple trichomes are uniseriate and usually 1–11-celled (Fig. 3A–D, F, K, O), with slightly (Fig. 3F–H) to strongly (Fig. 3A–D, O) minutely warty cuticle (Dias et al. 2013; Palchetti et al. 2014). Trichomes can be straight (Fig. 3A, B, O) or curved (Fig. 3C, D), cylindrical (Fig. 3B, O) or conical (Fig. 3A, M), flexible or somewhat rigid, antrorse or spreading. The distal cell of the trichomes can be acute (Fig. 3A, D, F), obtuse (Fig. 3H) or strongly curved resulting in a hook-shaped trichome (Fig. 3K). Branched trichomes can be furcate (forked) (Fig. 3G, P) to many times branched (Fig. 3J, L, N, see also Barboza et al. 2011). Papillae (Fig. 3E, I) are common at the margin and the apex of the corolla lobes in most species.

**Figure 4.**
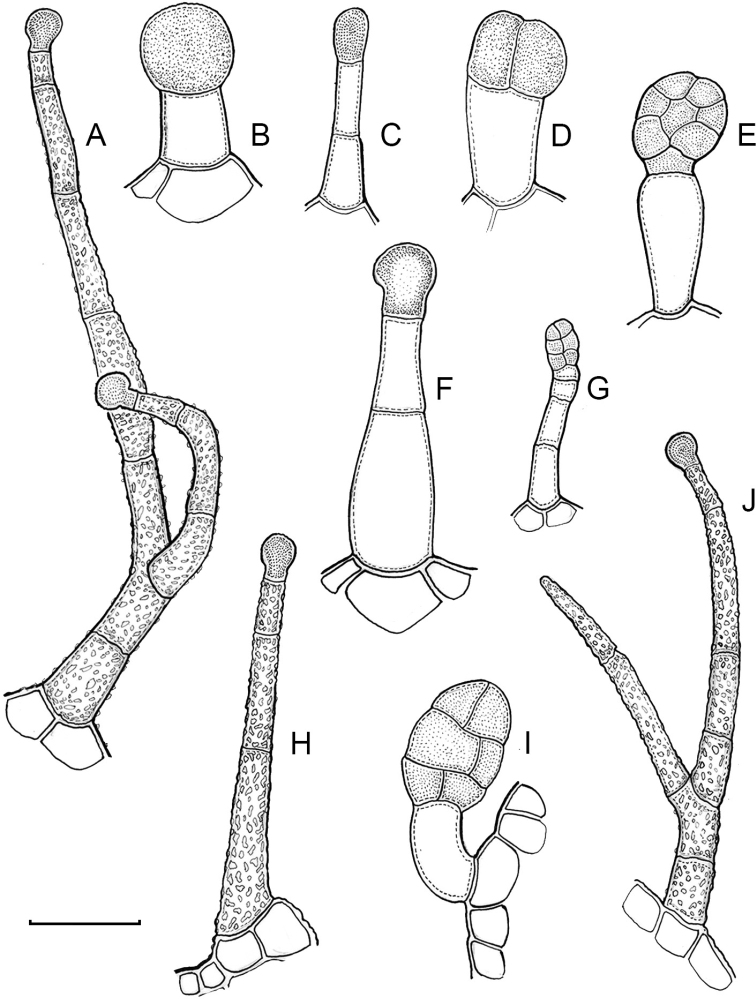
Glandular trichomes of *Capsicum* species. Scale bar: 50 μm (**A–G, I**); 100 μm (**H, J**).

Glandular trichomes are common in *Capsicum* species. In most species, glandular trichomes are simple, with short uni- or bicellular stalks and globose to ellipsoid multicellular heads (Fig. 4D, E, G, I). These are usually distributed on stems, leaves, pedicels and the outer calyx surface of some species (e.g. *C.caatingae*, *C.cardenasii*, *C.galapagoense*, *C.tovarii*) and on the inner calyx surface in all *Capsicum* species. Glandular trichomes with (1–) 2–3-celled stalks and globose or globose-peltate unicellular heads (Fig. 4B, C, F) are found on the interior surface of the corolla in many species. The most unusual glandular trichomes are found in *C.eshbaughii* (Fig. 4A, H, J) which has a distinctive branched glandular indument (Barboza 2011).

Density of pubescence is highly variable both within and between species. Sometimes, this variability has been considered diagnostic at the infraspecific level. For example, the densely pubescent plants of *C.chacoense*, *C.baccatum*, *C.eshbaughii* or *C.annuum* have been recognised at the infraspecific level (see descriptions and synonymy of those species).

### ﻿Inflorescences

*Capsicum* species have axillary flowers; they are solitary only in *C.chacoense* (Fig. 2C) or may be in 1–2 (–3)-flowered inflorescences in some species (e.g. *C.baccatum*, *C.friburgense*, *C.piuranum*) or in 4–13-flowered inflorescences in most of the species. *Capsicumceratocalyx* and *C.caatingae* may have more than 18 flowers per inflorescence. The inflorescences are either epedunculate and unbranched or borne on a short (e.g. *C.ceratocalyx*, *C.coccineum*) or somewhat elongate rachis that is occasionally forked (e.g. *C.regale*). Flowers are usually deciduous, falling off the plant gradually and leaving conspicuous or obscure pedicel scars on the rachis.

**Figure 5.**
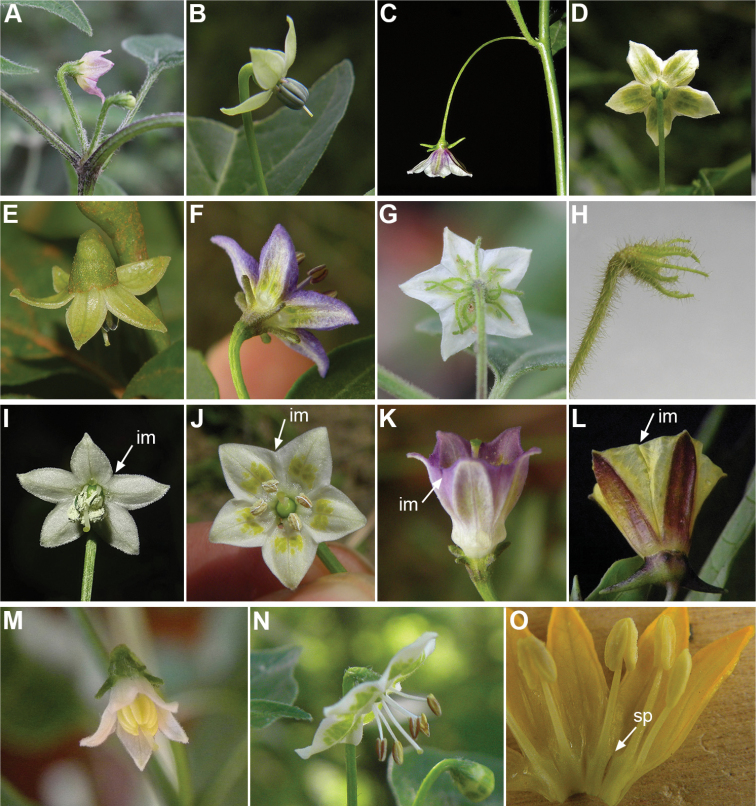
Flower morphology in *Capsicum* species **A***C.rabenii***B**C.annuumvar.glabriusculum**C***C.lanceolatum***D***C.schottianum***E***C.frutescens***F***C.eximium***G***C.eshbaughii***H***C.cornutum***I***C.galapagoense***J***C.recurvatum***K***C.cardenasii***L***C.lycianthoides***M***C.chacoense***N**C.baccatumvar.baccatum**O***C.caballeroi*. Abbreviations. *im* interpetalar membrane *sp* staminal plaque.

**Figure 6.**
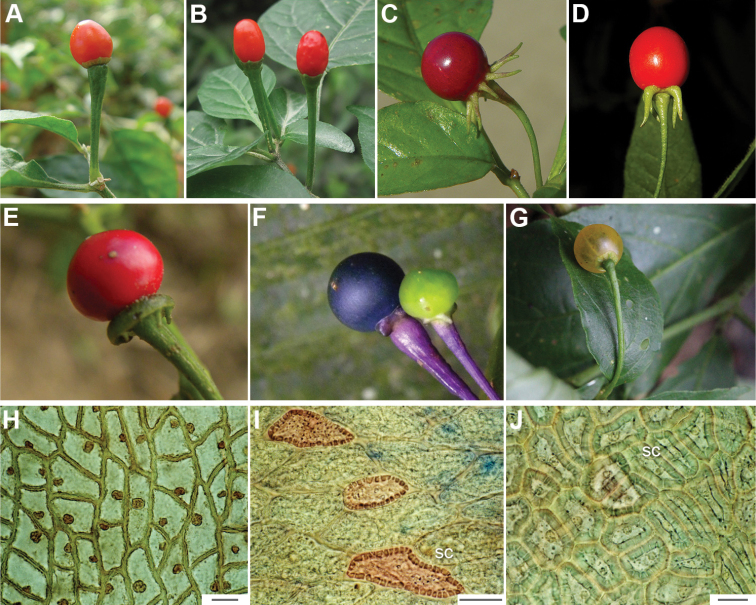
**A–G** Fruit morphology in *Capsicum* species **H, J** epicarp structure **A***C.chinense***B**C.baccatumvar.baccatum**C***C.hookerianum***D***C.lanceolatum***E***C.coccineum***F***C.regale***G***C.schottianum***H** epicarp with regular epidermal cells **I** epicarp with some sclereids amongst the regular epidermal cells **J** epicarp exclusively with sclereids. Abbreviation. *sc* sclereids. Scale bar: 10 μm (**H, I, J**).

All *Capsicum* flowers have distinct, usually pubescent, pedicels that may be terete (Fig. 5B), angled (Fig. 5A) or, rarely, almost winged (*C.ceratocalyx*). Pedicel/flower position is a useful identification character. Pedicels (and flowers) can be deflexed (termed pendent in this treatment; for example, *C.pereirae*, *C.lanceolatum*, Fig. 5C) or erect to spreading. In some cases, the pedicels are distally abruptly twisted forming a right or acute angle with the longitudinal axis of the flower (termed geniculate in this treatment). Flowers with geniculate pedicels may be orientated with their longitudinal axis in horizontal position (e.g. *C.rabenii*, Fig. 5A) or nearly parallel to the pedicel axis (e.g. C.annuumvar.glabriusculum, Fig. 5B). In fruits, pedicels are usually green (Fig. 6A, B, G) or green with purple stripes (e.g. *C.eximium*, *C.longifolium*) or completely purple (*C.regale*, Fig. 6F). They are normally widened distally and can be pendent (e.g. *C.recurvatum*, *C.flexuosum*) or erect (e.g., C.baccatumvar.baccatum, *C.chacoense*, *C.frutescens*).

### ﻿Calyces

The calyx in *Capsicum* species is usually 5-merous (4–8-merous in domesticated taxa) and entirely synsepalous (Fig. 5E). The calyx tube is generally cup-shaped or campanulate and 5–10 nerved, with the margin always entire (Fig. 5E, L). Below the margin, up to10 calyx appendages may emerge (Fig. 5A, F–H, L), a character only shared with *Lycianthes* (Dunal 1852; Bitter 1919; D’Arcy 1986; Hunziker 2001). In calyces without appendages, the calyx outline can be circular with the five main veins completely immersed in the calyx tube and ending very near its margin (e.g. *C.campylopodium*, *C.galapagoense*) (Fig. 5E). In species such as *C.schottianum*, the calyx outline may be pentagonal (Fig. 5D) with the main veins ending in five tiny lateral umbos or lumps that barely emerge over the calyx margin. Calyx appendages are lateral elongate expansions of the calyx tube (Fig. 5C, F–H, L) with vasculature formed by each main or secondary vein arching outwards into the appendages and returning to the calyx tube to end in the margin of the calyx (see figs 1–4 in D’Arcy 1986). The calyx sleeve, which is a ring of tissue immediately below the margin, is rudimentary (e.g. *C.recurvatum*, *C.hookerianum*, *C.coccineum*, *C.lycianthoides*, Fig. 5L) or non-existent in *Capsicum* (Fig. 5F, H). This sleeve is well developed in many *Lycianthes* species (D’Arcy 1986; Dean et al. 2017, 2020) and, thus, serves as a trait to differentiate the two genera. The number, shape, size and orientation of the appendages are important identification characters within *Capsicum*. The number of appendages is consistent (5 or 10) in most species, but in others, it can vary (2–10) within a species and, sometimes, even within an individual (e.g. *C.recurvatum*). Appendages shape ranges from subulate (wider at the base and pointed at the tip, for example, *C.lycianthoides*, Fig. 5L), to cylindrical (e.g. *C.eximium*, Fig. 5C, F) and linear (e.g. *C.eshbaughii*, *C.cornutum*, Fig. 5G, H). In some species, the appendages are thick and strongly laterally flattened like triangular-compressed wings (e.g. *C.longifolium*, *C.regale*). Appendages are usually equal or subequal (e.g. *C.eximium*, *C.piuranum*), but when there are more than five in number, the main appendages (those with vasculature formed by the main veins) are longer than the secondary appendages (those with vasculature formed by secondary veins) (e.g. *C.hookerianum*, *C.longidentatum*). The longest main appendages (ca. 8.5 mm) are found in *C.cornutum* (Fig. 5H). Calyx appendages can be erect and appressed to the corolla (e.g. *C.eximium*, Fig. 5F), spreading (e.g. *C.dimorphum*, *C.lycianthoides*, Fig. 5L), recurved (e.g. *C.recurvatum*), reflexed (e.g. *C.lanceolatum*, Fig. 5C) or incurved and horn-like (*C.ceratocalyx*); orientation may change in the transition between flowering and fruiting (e.g. *C.coccineum*). An annular constriction at the junction between the fruiting calyx and pedicel is present in a few wild species (e.g. *C.caatingae*, *C.regale*) and it is a key character used in the identification of *C.chinense* (Fig. 6A). Fruiting calyces are persistent and not accrescent or only slightly accrescent (domesticated species). They are discoid in most species (Fig. 6G), but can be cup-shaped (e.g. *C.frutescens*) and are strongly reflexed in *C.coccineum* (Fig. 6E).

### ﻿Corollas

*Capsicum* species have 5-merous (6–8-merous in domesticated taxa) sympetalous corollas. Corollas are usually of intermediate size (6–14 mm long), the smallest measuring 4–5 mm long (e.g. *C.galapagoense*) and the largest ones reaching 17–18 mm long (e.g. *C.caballeroi*, *C.piuranum*). Most species have stellate corollas (Fig. 5D–G, I, J), but campanulate (e.g. *C.lanceolatum*, Fig. 5C, *C.cardenasii*, Fig. 5K), broadly campanulate (*C.lycianthoides*, Fig. 5L, *C.rhomboideum*), campanulate-urceolate (*C.friburgense*), rotate-stellate (*C.flexuosum*, C.baccatumvar.baccatum, Fig. 5N) or rotate (e.g. *C.pubescens*) corollas are also present. The corolla tube can be very short (*C.benoistii*), short (e.g. *C.eximium*, Fig. 5F) or long (e.g. *C.caballeroi*, *C.cardenasii*, Fig. 5K, *C.piuranum*). The lobes are connected by a thinner interpetalar membrane nearly completely (e.g. *C.lycianthoides*, Fig. 5K, L), partly (e.g. C.baccatumvar.baccatum Fig. 5J) or just near the base (C.annuumvar.glabriusculum, *C.galapagoense*, Fig. 5I); in very few species, interpetalar membrane is absent (e.g. *C.caballeroi*).

Corolla colour is highly variable in *Capsicum* species. Corollas can be entirely white (e.g. *C.chacoense*, *C.galapagoense*, C.annuumvar.annuum), dull white or greenish-white (*C.chinense*, *C.frutescens*), light yellow (*C.neei*), yellow (e.g. *C.piuranum*, *C.caballeroi*) or violet or fuchsia (*C.friburgense*). Other species have corollas with a predominant primary colour (white, yellow or purple), as well as markings (spots) with diverse pigmentation. The abaxial surface (outer surface) of the corollas may have the same colouration as the adaxial surface (inner surface) (e.g. *C.geminifolium*, *C.regale*) or it may have a faded or a different colouration (e.g. *C.tovarii*, *C.pereirae*). In many species, the co-occurrence of different pigments results in multi-coloured corollas, for example, white corollas with purple (or variations) spots at the base of the lobes and limb and green or greenish-yellow centre (e.g. *C.villosum*, *C.schottianum*, *C.pereirae*). Descriptions in literature or on specimen labels usually refer to the colour of the inner (adaxial) corolla surface. In this monograph, description of the corolla colour of both surfaces is provided for each species; corolla colour can be very difficult to see on herbarium specimens.

Corolla pigmentation is due to anthocyanins which produce violet or purple shades (e.g. *C.lycianthoides*, *C.lanceolatum*, Fig. 5C, F, K, L) and chlorophyll which produces green colouring (*C.baccatum*, *C.flexuosum*, Fig. 5D, J).

The adaxial surfaces of the corollas are glabrous (many Andean species) or may be covered by sparse glandular trichomes (e.g. *C.ceratocalyx*, *C.tovarii*) or have a continuous ring of glandular trichomes (Fig. 4C, F) at the base of the lobes and in the throat (the Bolivian and Brazilian species). The abaxial surfaces of corolla lobes are mostly papillate, with minutely warty papillae (Fig. 3E, I) or with short simple trichomes on the margins and tips. The lobes are spreading in most species (Fig. 5D, F, G, I, J), erect in *C.tovarii* or slightly (e.g. *C.cardenasii*, Fig. 5K) to strongly recurved in some species (*C.caballeroi* and *C.friburgense*).

### ﻿Androecium

*Capsicum* species have usually five (sometimes 6–8 in domesticated taxa) equal stamens; unequal stamens have only been observed in three species: *C.campylopodium* (and sometimes also *C.lycianthoides*), which has two stamens longer than the other three (Hunziker 2001) and *C.regale*, which has one longer than the other four (Barboza et al. 2020b). The free portion of the filaments is always distinct and glabrous and it is usually longer than the anthers (Fig. 5N). Each filament broadens at its base forming a staminal plaque (called a stapet, Hunziker 2001), with two short lateral auricles fused to the corolla base. In *C.chacoense*, these lateral auricles are long and are not fused to the corolla. The anthers are longitudinally dehiscent and are usually ellipsoid and yellow or cream in colour (Fig. 5M); in domesticated taxa and some wild species (e.g. *C.dimorphum*, *C.carassense*, *C.regale*), the anthers are blue (Fig. 5B), bluish-grey or purple. In pre-anthesis and early anthesis, the anthers are connivent (Fig. 5M), but they become separated during anthesis (Fig. 5N), remaining somewhat connivent in some species (Fig. 5B). Anther size is variable, ranging from 0.9–1.3 mm long in *C.galapagoense*, to 1–3 mm long in most species, to up to 4 mm in *C.hunzikerianum*.

Pollen is yellow or white, trizonocolporate, spheroidal, prolate, prolate-spheroidal or oblate-spheroidal, with a triangular to circular outline in polar view. It is usually small, from 15 µm in *C.rhomboideum* (Bo and Carrizo García 2015) to 18–24.73 µm in some domesticated species (Dharamadhaj and Prakash 1978; Martins et al. 2013; Adedeji and Akinniyi 2015; Bo and Carrizo García 2015; Kayani et al. 2018; Yang et al. 2018; Song et al. 2019), but in other domesticated and wild species, sizes range from 25.75 to 38 µm (Murray and Eshbaugh 1971; Quagliotti 1979; Martins et al. 2013). Pollen grains are shed in monads and at the binucleate stage (Cochran 1938; Yaqub and Smith 1971; Dharamadhaj and Prakash 1978; Kim et al. 2004; Bo and Carrizo García 2015) or trinucleate stage (e.g. a *C.frutescens* cultivar, Lengel 1960). Information on the ornamentation of the exine is variable in literature depending on whether observations were made with a light microscope (LM) or a scanning electron microscope (SEM). The exine appears to be clearly microechinate and perforate under SEM in *C.annuum* (Halbritter 2016a; Song et al. 2019) and *C.pubescens* (Bo and Carrizo García 2015; Halbritter 2016b). The exine has been described as reticulate (Murry and Eshbaugh 1971; Roubik and Moreno 1991), foveolate (Martins et al. 2013), psilate or faintly granulate (Kayani et al. 2018) and scabrate (Barth and Duarte 2008), based on LM observations for some *Capsicum* species, which should be corroborated with SEM. Pollen morphology is lacking for most wild *Capsicum* species and more information is needed to evaluate the usefulness of pollen to distinguish species.

### ﻿Gynoecium

The gynoecium in *Capsicum* species is usually bicarpellate (2–5-carpellate in domesticated species). The ovary is superior with axile placentation, glabrous and usually subglobose to ovoid, rarely ellipsoid (e.g. *C.frutescens*). The style is simple, straight or slightly curved, cylindrical (the same width from the proximal to distal end, Fig. 5N) or clavate (broadened gradually from its base to the apex), glabrous, white, cream, lilac or purple and commonly exserted beyond the anthers. Most species have homostylous flowers, but some species (*C.annuum*, *C.baccatum*, *C.benoistii*, *C.tovarii*, *C.pubescens*) have heterostylous flowers, with different flowers on the same plant bearing short, medium or long styles. In domesticated species, style length variations have been observed amongst cultivars (Bosland and Votava 2000; Peña-Yam et al. 2019). The stigma is pale green or cream, globose or discoid, sometimes slightly bilobed (e.g. *C.longifolium*, *C.lanceolatum*) and finely papillate. The ovules are usually numerous, anatropous, tenuinucellate, with a single integument (Cochran 1938; Munting 1974; Dharamadhaj and Prakash 1978; Pérez-Pastrana et al. 2018). The nectary is located at the base of the ovary and is easily observed in fresh material, but sometimes can be very difficult to see in herbarium specimens; it is an inconspicuous annular disc, paler in colour than the rest of the ovary, variable in thickness and produces copious nectar. The nectar is colourless and is exposed in five nectar droplets on the corolla limb (see more detail in Floral biology and pollination).

### ﻿Fruits

**Figure 7.**
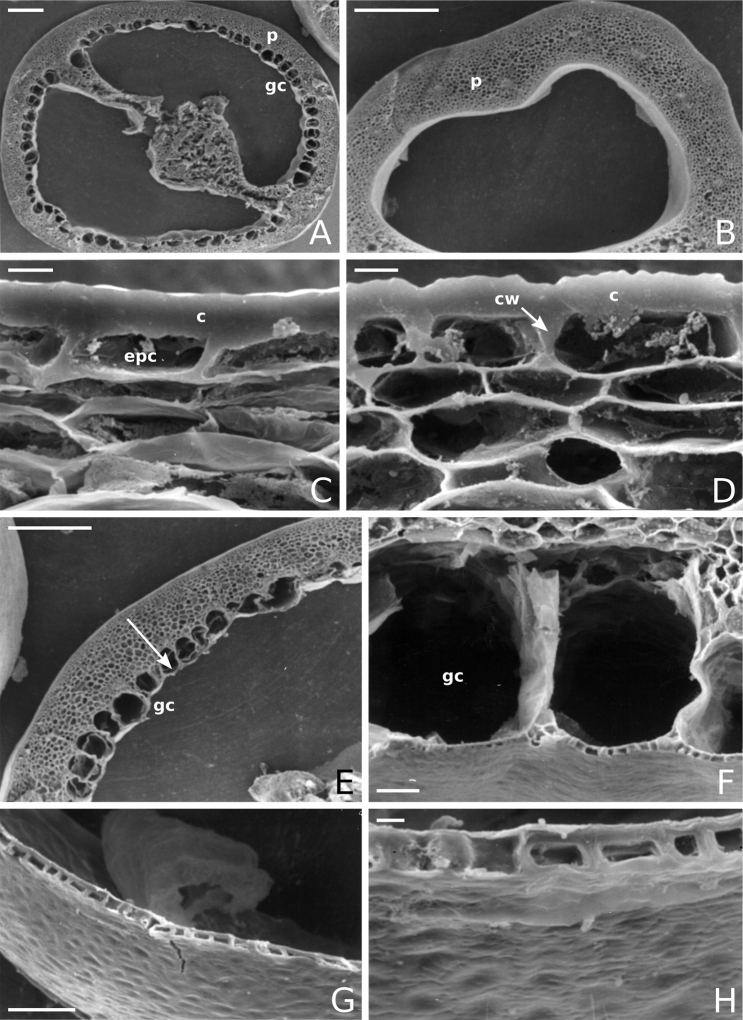
Fruit anatomy in *Capsicum* species **A, D–F**C.baccatumvar.pendulum**B**C.baccatumvar.umbilicatum**C, G, H***C.pubescens***A** fruit, in cross section (note giant cells in the pericarp) **B** one locule of a fruit, in cross section (note the absence of giant cells in the pericarp) **C, D** epicarp and some layers of mesocarp (in D, observe cuticular wedges) **E** sector of pericarp (the arrow indicates the increase of the cell size ending in the giant cells) **F** detail of two adjacent giant cells **G** sector of homogeneous endocarp **H** sclereids of the endocarp. Abbreviations. *c* cuticle, *cw* cuticular wedge, *epc* epidermal cells, *gc* giant cells, *p* pericarp. Scale bars: 1 mm (**A, B, E**); 10 μm (**C, D, H**); 100 μm (**F, G**).

The fruit is usually a bicarpellate berry (Fig. 7A) that can also be 3–5-carpellate in domesticated species (e.g. C.annuumvar.annuum, *C.chinense*, *C.pubescens*) or some wild species (e.g. *C.tovarii*). The berries are extremely diverse in shape, size and colour in domesticated species and more homogeneous in wild (and semi-domesticated) species. The fruits are juicy, fleshy, opaque (most species) or translucent (some Brazilian species). Wild species have globose or subglobose berries < 15 mm in diameter (Fig. 6A, C, E–G) or short ellipsoid or ovoid (e.g. C.baccatumvar.baccatum, *C.chacoense*) berries 0.6–14 mm long (Fig. 6B). The fruits in domesticated species are much longer and wider, with the most elongate fruits (300 mm long or more) in C.annuumvar.annuum (Bosland and Votava 2000). The great variation of the fruit shapes has resulted in the proposal of more than 100 names applied to the domesticated species (here assigned to any of the five recognised domesticated taxa). Within each of the domesticated taxon, a system of different fruit types (i.e. characterised by a defined fruit shape and colour, pungency level, aroma and/or flavour and uses) is used by horticulturists or plant breeders (Bosland et al. 1988); nearly 100 fruit types are known, the most frequent of which are described in Bosland and Votava (2000) and DeWitt and Bosland (1997, 2009). Red is the most frequent fruit colour in wild *Capsicum* species (Fig. 6B–E), with colours ranging from orange-red (e.g. *C.geminifolium*), dark burgundy (*C.rhomboideum*) to dark blue or purple-blue (*C.regale*, Fig. 6F). Some Brazilian species have greenish-golden yellow, translucent fruits (e.g. *C.parvifolium*, *C.schottianum*, Fig. 6G), a character state considered to be derived within *Capsicum* (Carrizo García et al. 2016).

Fruiting pedicels are usually green (Fig. 6A, B, G) or green with purple stripes (e.g. *C.eximium*, *C.longifolium*) or completely purple (*C.regale*, Fig. 6F). They are normally widened distally and can be pendent (e.g. *C.recurvatum*, *C.flexuosum*) or erect (e.g., C.baccatumvar.baccatum, *C.chacoense*, *C.frutescens*). Pedicels and ripe fruits are persistent in domesticated species, staying attached to the plant; in most wild species, the mature berries are usually deciduous and are easily detached from the calyx, leaving only pedicels and fruiting calyces on the plant (Carvalho et al. 2014, GEB, pers. obs.). In just a few species (e.g. *C.muticum*, *C.coccineum*), mature berries fall from the plant with pedicels attached, leaving conspicuous scars.

### ﻿Pericarp structure

The development of the pericarp in *Capsicum* species is the typical of a true berry (Roth 1977), with the epicarp and endocarp originating respectively from the outer and inner epidermis of the ovary wall and the mesocarp derived from the middle layers between the two epidermal layers (Roth 1977; Filippa and Bernardello 1992); this structure is typical of most Solanaceae (Roth 1977; Bernardello 1983; Filippa and Bernardello 1992). The taxonomic significance of the pericarp structure in *Capsicum* has been rarely considered with some observations made on varietals or cultivars of the domesticated species (Tschirch and Oesterle 1900; Augustin 1907; Tschirch 1925; Winton and Winton 1939; András 1966; Fridvalszky and Nagy 1966; Konecsni 1971; Munting 1974; Dave et al. 1979; Dave 1986; Weryszko-Chmielewska and Michałojć 2011; Dias et al. 2013) and a few observations made on wild species (Filippa and Bernardello 1992). In this monograph, we include the most relevant taxonomic characters observed in nearly all the species of *Capsicum* (see Suppl. material 1: Appendix 1, for full details of each species).

The epicarp consists of a uniseriate epidermis covered by a smooth (e.g. *C.annuum*, *C.chacoense*, *C.pubescens*, Fig. 7C) or striate cuticle (e.g. *C.campylopodium*, *C.mirabile*). The cuticle can be thin (4.5–11 µm, for example, *C.flexuosum*, *C.campylopodium*) or thick (11.5–19 µm, for example, *C.eximium*, *C.galapagoense*) and usually projects towards the anticlinal walls of two adjacent epidermal cells forming conspicuous cuticular wedges (Fig. 7D). The cuticular wedges can be shallow to very deep, reaching the first layer of the mesocarp, with the cuticle extending below the inner periclinal wall of the epidermal cells (e.g. *C.caatingae*); these cuticular wedges vary from 7.5 to 54 µm thick. The epicarp is formed by regular epidermal cells (Fig. 6H, I), scarce stomata and sometimes sclereids (Fig. 6I, J). In cross section, epidermal cells are elongate tangentially (rectangular, Fig. 7C, D), isodiametric or flask-shaped (e.g. *C.hookerianum*) and in superficial view, they are polygonal, with straight (Fig. 6H) or slightly sinuous cell walls; pits appear frequently in thick- and thin-walled cells (Fig. 6I). The epicarp may have scattered sclereids amongst the epidermal cells (e.g. C.baccatumvar.pendulum, Fig. 6I) or it can consist exclusively of sclereids (C.baccatumvar.baccatum, Fig. 6J). Ventilating clefts are found in the epicarp of many species (Dave et al. 1979; Filippa and Bernardello 1982; this work), but their presence is not a constant feature within a species (Dave 1986).

The mesocarp consists of (5–) 7–26 layers, with up to 30 layers found in the thick pericarp of a sweet C.annuumvar.annuum cultivar known as “calahorra”. The mesocarp can be homogeneous or heterogeneous; a homogeneous mesocarp is formed exclusively by parenchyma (thin-walled cells) or collenchyma (thick-walled cells) (e.g. *C.rhomboideum*, *C.hookerianum*), whilst a heterogeneous mesocarp consists of both collenchyma and parenchyma with a variable number of layers for each tissue, depending on the species (see Suppl. material 1: Appendix 1). The collenchymatous layers are always placed underneath the epicarp and both together have been referred as the “exocarp” (Roth 1977). The parenchymatous layers are underlying the collenchyma and the number of layers is usually greater than those of the collenchyma. Crystal sand and vascular bundles are found in the collenchyma or parenchyma (e.g. *C.rhomboideum*, *C.dimorphum*). A gradual increase of cell size occurs from the periphery inwards (Fig. 7E). In a few *Capsicum* species (e.g. the Andean clade species), the cells of the innermost layer of the mesocarp are equal in size and similar in shape to the adjacent mesocarp cells (Fig. 7B); thus, the pericarp surface facing the locule (endocarp) is smooth. In contrast, in most species, the inner surface of the pericarp is blistered due to the extensive development of the inner hypodermis (Fig. 7A, E, F); the inner hypodermis develops as a single layer of ‘giant cells’ that are initially large in the ovary wall (Augustin 1907; Munting 1974) and then increase tremendously in size towards fruit maturity (Fig. 7A, E). These giant cells are occupied by scanty cytoplasm, a large watery vacuole and a large nucleus pushed to the periphery by the vacuole. The size of the giant cells varies amongst the species; they can be nearly as long as wide, ranging from 390–1380 µm long and 300–1050 µm wide (most species) or 2–6 times longer than wide, varying from 540–2500 µm long and 150–490 µm wide (e.g. some Brazilian species). Each giant cell is attached to its neighbouring cell and the endocarp and they are surrounded by a triangular multiseriate wedge-shaped cluster of parenchymatous cells between their anticlinal cell walls (“bridge”, fide Roth 1977). The development of giant cells in the mesocarp is a derived anatomical trait in *Capsicum*, absent in the Andean clade and with a single reversion to the ancestral state in C.baccatumvar.umbilicatum (Fig. 7B) (Carrizo García et al. 2016).

Hard inclusions of sclereids (stone cells) are developed in the mesocarp of some species (5 spp., see Suppl. material 1: Appendix 1); the stone cells can be completely immersed in the mesocarp and then only visible under microscope (*C.piuranum*) or they are easily seen in the pericarp surface in dried fruits (e.g. *C.geminifolium*). There are never more than six per fruit. The presence, number and position of the stone cells are not consistent within a single species; thus, these are not useful traits in the identification of *Capsicum* species, as they may be in other Solanaceous genera, such as *Lycianthes* (Bitter 1919) or *Solanum* (Särkinen et al. 2018; Knapp et al. 2019).

The endocarp develops from the inner epidermis of the ovary wall; it consists of one layer of polygonal or irregular cells (surface view) with straight or sinuate cell walls. The endocarp can be homogeneous, that is entirely with thin (e.g. *C.recurvatum*) or pitted thick-walled cells (sclereids) (e.g. *C.rhomboideum*, *C.pubescens*, Fig. 7G, H) or it may consist of a mixture of cells with thin and thick walls forming a heterogeneous tissue (e.g. *C.annuum*); in this latter endocarp type, groups of pitted sclereids lie on the inner periclinal walls of the giant cells that alternate with groups of thin-walled cells placed on the multiseriate wedge (“bridge”) of parenchymatous cells (Konecsni 1971; Dave 1986).

Some structural features of the pericarp useful in species-level taxonomy are detailed in Suppl. material 1: Appendix 1.

### ﻿Septum and placenta

The presence of the pungent principles (capsaicinoids) of *Capsicum* fruits within cells of the interlocular septum and placenta has been demonstrated by histochemical (Ohta 1962d) and radioisotopic (Iwai et al. 1979) evidence, structural and ultrastructural microscopic analyses (Suzuki et al. 1980; Zamski et al. 1987; Stewart et al. 2007) and analytical chemical procedures (Manirakiza et al. 2003; Stewart et al. 2007; Nugroho 2016; Guillen et al. 2018). The histological characteristics of the septum during fruit development have been investigated extensively in some cultivars of *C.annuum* and *C.frutescens* (Tschirch and Oesterle 1900; Ohta 1962d; Munting 1974; Dave et al. 1979; Suzuki et al. 1980), as well as some wild *Capsicum* species (Filippa and Bernardello 1992). In general, the septum consists of epidermal cells and a loose parenchymatous tissue with intercellular spaces. The epidermal cells are responsible for the synthesis, accumulation (in pockets or blisters) and secretion of capsaicinoids. In early stages of fruit development (about 10 days after flowering), some of these cells become secretory (Ohta 1962d; Suzuki et al. 1980; Filippa and Bernardello 1992), but they fully develop the characteristic traits of a glandular tissue (i.e. large elongate size, abundant cytoplasm, a large vacuole, many small vesicles containing electron-dense granules and a large nucleus) nearly 30 days after flowering (Suzuki et al. 1980).

An ultrastructural study demonstrated that the major reservoir of capsaicinoids is in the capsisome, a specific capsaicinoid biosynthesising and accumulating vacuole, different from the vacuoles regarded as reservoirs of organic acids (Fujiwake et al. 1980, 1982).

Capsaicinoids have also been found in the pericarp and seeds in significant or low amounts in some species and cultivars (e.g. *C.chinense*, *C.baccatum*) (Pandhair and Sharma 2008; Bosland et al. 2015; Guillen et al. 2018).

### ﻿Seeds

**Figure 8.**
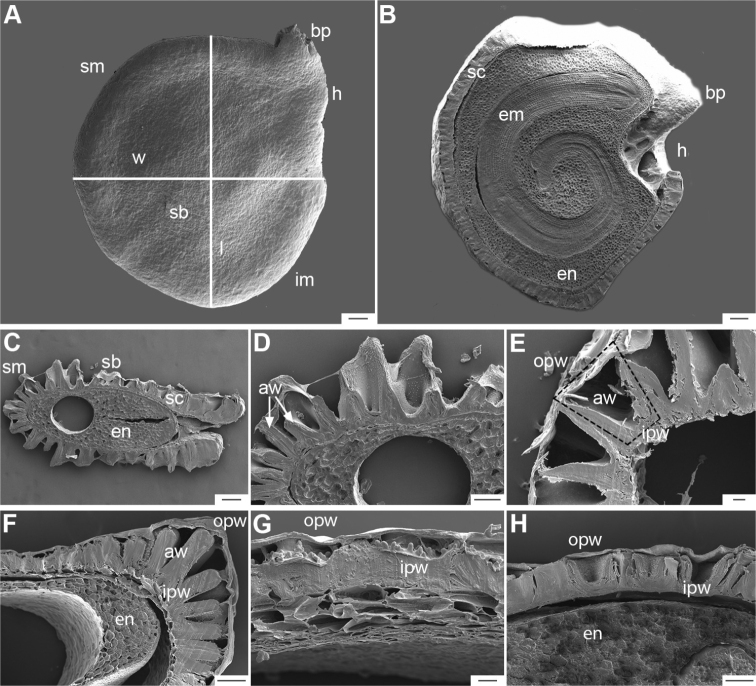
Seed morphology and seed coat structure **A***Capsicumchinense***B***C.chacoense*, longitudinal section **C, D***C.schottianum*, cross section (**D** detail of the seed coat structure) **E**C.annuumvar.annuum, detail of seed coat structure (the rectangle indicates a cell of the seed coat) **F, G***C.eximium*, cross sections at the seed margin and seed body, respectively **H***C.dimorphum*, cross section at the seed body. Abbreviations. *aw* anticlinal cell wall, *bp* beak prominence, *em* embryo, *en* endosperm, *h* hilum, *im* inferior seed margin, *ipw* inner periclinal wall, *opw* outer periclinal wall, *sb* seed body, *sc* seed coat, *sm* superior seed margin, *w* seed width, *l* seed length. Scale bars: 200 μm (**A–C**); 100 μm (**D, F, H**); 20 μm (**E, G**).

The gross morphology of the seeds and details of the sculpturing of the seed coat for all *Capsicum* species are summarised in Suppl. material 2: Appendix 2. The attributes used in species descriptions are illustrated in Fig. 8.

Seed shape (and size) is influenced by the position in the berry. The seeds are flattened to slightly angled, mostly C- or D-shaped (Gunn and Gaffney 1974), subglobose or ellipsoid, rarely reniform or teardrop-shaped. Seed shape is sometimes difficult to define in the domesticated species due to the presence of a protrusion on the seed coat (a beak-like prominence) in a vertical or nearly vertical direction above the hilum (Fig. 8A, B). This protrusion can also occur in a lateral position in some wild species with the hilum placed on the protrusion edge (e.g. *C.cornutum*). Seed size varies from small (1.5–2.5 mm long) and medium (2.6–3.9 mm long) to large (4–7 mm long). *Capsicumlycianthoides* has the smallest seeds (1.5–1.8 mm long), whereas the largest seeds are found in the domesticated *C.pubescens* (5.5–7 mm long). Seed colour is usually uniform and is slightly shiny to shiny when observed dry under a stereomicroscope. Seeds can be grouped in three categories based on their colour: (1) pale yellow or nearly white (*C.neei*) to yellow (mostly domesticated species and their close relatives); (2) brownish-yellow to brown (many Bolivian species); and (3) brownish-black to black (many Andean and Brazilian species). Seed number per berry in *Capsicum* species is usually 10–45, with only four seeds found in *C.campylopodium* and more than 50 in *C.lanceolatum*, *C.piuranum* and some domesticated taxa.

**Figure 9.**
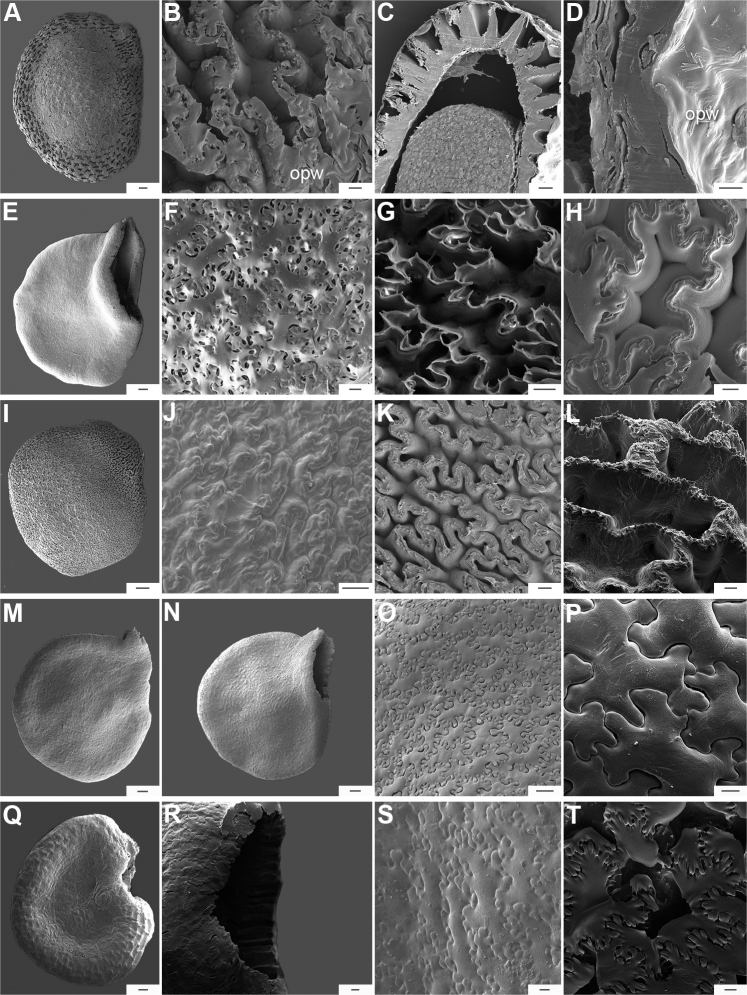
Seeds and seed coat morphology in species of the Annuum Clade **A–D**C.annuumvar.annuum**E–H**C.annuumvar.glabriusculum**I–L***C.frutescens***M–P***C.chinense***Q–T***C.galapagoense***A, I** seeds with testa partly digested **B** seed coat with the external periclinal cell wall partly removed **C, D** cross section of the seed at the seed margin and seed body, respectively; **E, M, N, Q** untreated seeds showing subterminal hilum (**E, N**) and medial hilum (**Q**) **F, J, O, P, S, T** detail of a non-digested portion of the seed coat **G, K** testa pattern with the external periclinal cell wall removed **H, L** detail of testa cells **R** hilum. Abbreviation. *opw* outer periclinal cell wall. Scale bars: 200 μm (**A, E, I, M, N, Q**); 20 μm (**B, F–H, K, L, P, S, T**); 50 μm (**C, J, O, R**); 10 μm (**D**).

**Figure 10.**
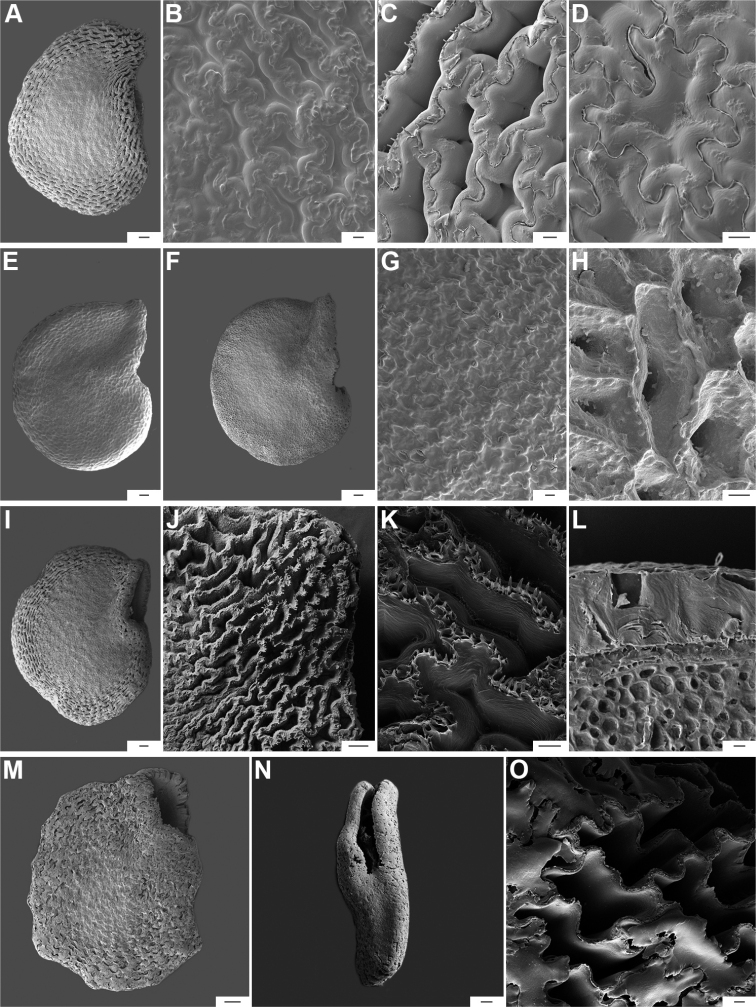
Seeds and seed coat morphology in species of the Baccatum Clade **A–D**C.baccatumvar.baccatum**E**C.baccatumvar.pendulum**F–H**C.baccatumvar.umbilicatum**I–L***C.chacoense***M–O***C.rabenii.***A, I, M** seeds with testa partly digested **B, G** detail of a non-digested portion of the seed coat **C, H, J, K, O** testa pattern of treated seeds showing anticlinal cell walls with fibrils (**C, K**), papillae (**H**) and ridge (**O**) **D** detail of a testa cell **E, F** untreated seeds **L** cross section of the seed at the seed body **N** seed showing the subterminal elliptical hilum. Scale bars: 10 μm (**H**); 20 μm (**B–D, G, K, L, O**); 100 μm (**J**); 200 μm (**A, E, F, I, M, N**).

The hilum is always marginal (on the inferior margin, Fig. 8A) and its position may be medial (Fig. 9Q), subterminal (Figs 9N, 10N) or terminal (Fig. 9I). The hilum area may be inconspicuous (minute to small) or conspicuous (medium to large). The hilum area shape may be elliptical, linear, ovoid or more rarely triangular (*C.minutiflorum*, *C.pubescens*) or circular (*C.tovarii*); some species may have two or three different hilar area shapes.

*Capsicumannuum* (and its varieties) has been the most frequently studied species (Lohde 1875; Moeller 1886, 1905; Hanausek 1888; Hartwich 1894; Wojciechowska 1972; Krstić et al. 2001, Kong et al. 2011) before our studies reported here. Some other authors focused on the development and the structure and sculpture of the seed coat of many Solanaceae species, including some of the domesticated capsicums (Souèges 1907; Gunn and Gaffney 1974; Al-Nowaihi and Mourad 1999; Dias et al. 2013). More recently, Chiou and Hastorf (2014) provided a morphometric approach, based on 27 qualitative and quantitative seed attributes for the five domesticated species. They recovered some useful traits that can be used to compare features of modern *Capsicum* seeds to archaeological seeds. The gross morphology and the sculpture of the seed coat provided here for each *Capsicum* species (Suppl. material 2: Appendix 2) highlight the importance of some characters for delimiting species, species groups or clades. Carrizo García et al. (2016) found that seed colour is useful for identifying species or small clades (e.g. brownish-black or black seeds in the Atlantic Forest and in Andean clades or the pale yellow seeds in the Annuum and Baccatum clades). The seeds of species within the same clade are also characterised by similar sculpture and the structure of the outer layer. For example, species of the Atlantic Forest clade all have a seed coat that is reticulate-tuberculate/reticulate with pillar-like structures, has deep cells in the margin and seed body and has a thin cellulosic outer periclinal cell wall in the outer layer that is easily removed (naturally or by enzyme etching).

**Figure 11.**
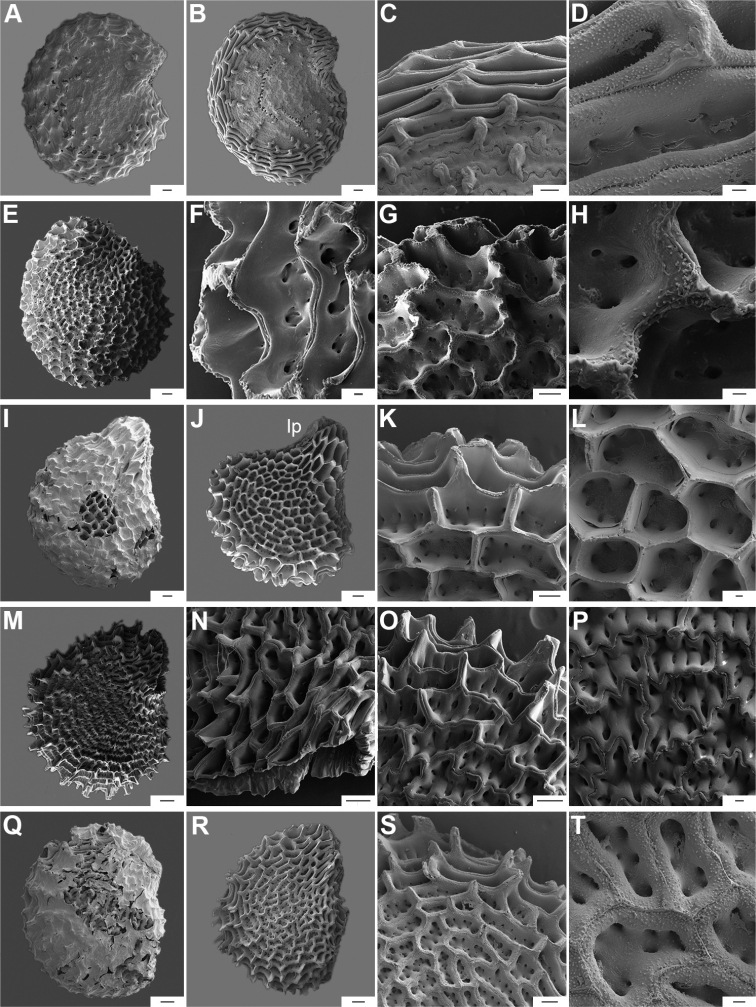
Seeds and seed coat morphology in species of the Atlantic Forest Clade **A–D***C.campylopodium***E–H***C.carassense***I–L***C.cornutum***M–P***C.friburgense***Q–T***C.mirabile***A** untreated seed **B, I, Q** seeds with testa partly digested **C, F, K, O, S** marginal testa pattern of treated seeds (note pillar-like outgrowth in **K, O, S**) **D, G, H, L, P, T** testa pattern at the seed body of treated seeds showing anticlinal cell walls papillate and punctate **E, J, M, R** treated seeds (in J showing lateral prominence) **N** hilar zone with a linear hilum. Abbreviation. *lp* lateral prominence. Scale bars: 200 μm (**A, B, E, I, J, M, Q, R**); 100 μm (**C, G, K, N, O, S**); 20 μm (**D, F, H, L, P, T**).

**Figure 12.**
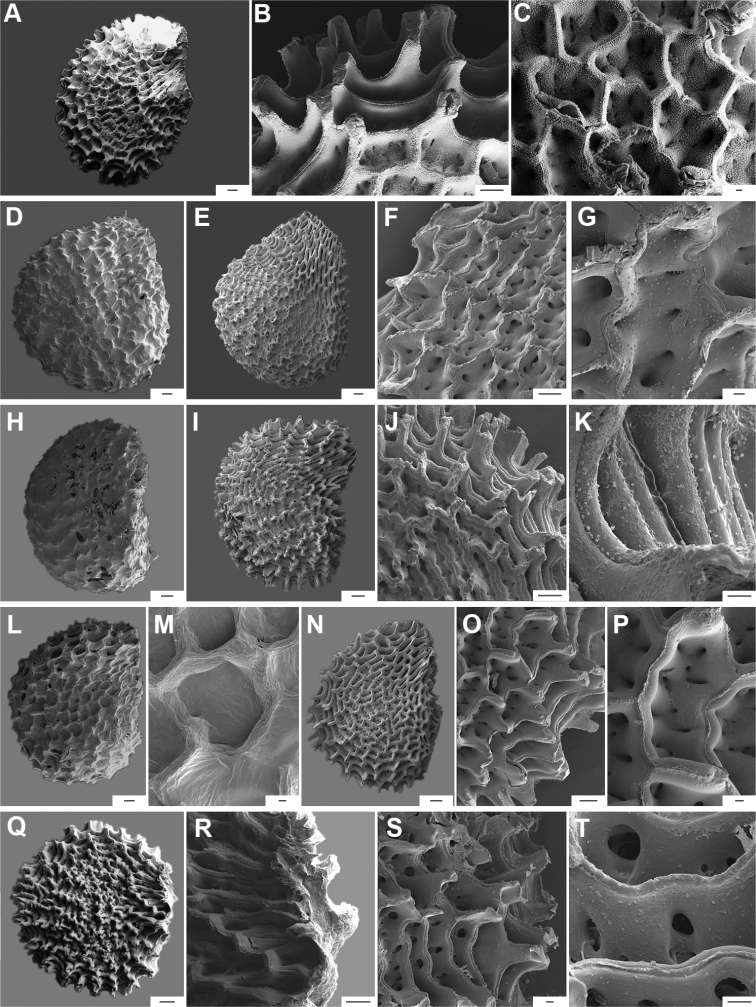
Seeds and seed coat morphology in species of the Atlantic Forest Clade **A–C***C.muticum***D–G***C.pereirae***H–K***C.mirum***L–P***C.schottianum***Q–T***C.recurvatum***A, E, I, N, Q** treated seeds **B, F, J, O, S** marginal testa pattern of treated seeds with pillar-like outgrowths **C, G, P, T** testa pattern at the seed body of treated seeds showing anticlinal cell walls papillate and punctate **D, H, L** untreated seeds **K** detail of papillate anticlinal cell walls **M** detail of a non-digested portion of the seed coat **R** hilar zone with a linear hilum. Scale bars: 200 μm (**A, D, E, H, I, L, N, Q**); 100 μm (**B, F, J, O, R**); 20 μm (**C, G, K, M, P, S, T**).

**Figure 13.**
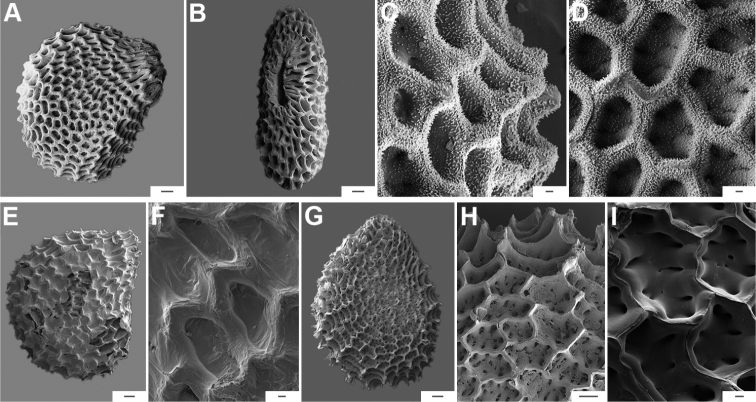
Seeds and seed coat morphology in species of the Atlantic Forest Clade **A–D***C.hunzikerianum***E–I***C.villosum***A, G** seeds with testa digested **B** seed showing medial and linear hilum **C, H** marginal testa pattern of treated seeds **D, I** testa pattern at the seed body of treated seeds showing anticlinal cell walls papillate (**D**) and punctate (**I**) **E** untreated seed **F** detail of a non-digested portion of the seed coat. Scale bars: 200 μm (**A, B, E, G**); 20 μm (**C, D, F, I**); 100 μm (**H**).

**Figure 14.**
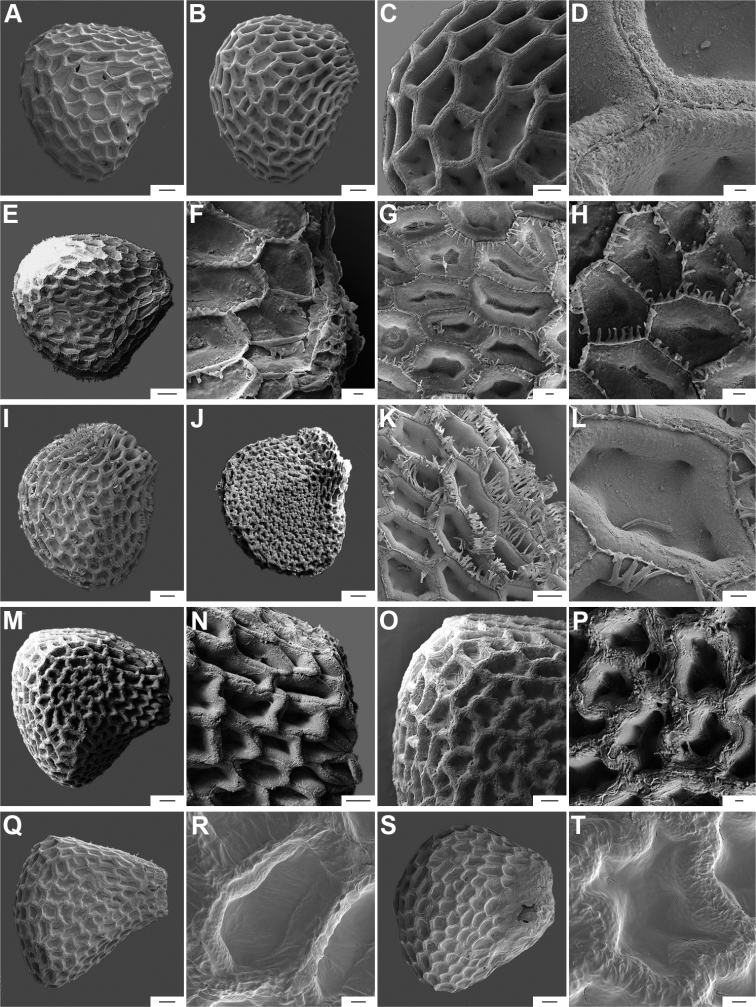
Seeds and seed coat morphology in species of the Andean Clade **A–D***C.dimorphum***E–H***C.geminifolium***I–L***C.lanceolatum***M–P***C.longifolium***Q–T***C.lycianthoides***A, Q, S** seeds untreated **B, E, I, J, M** seeds with testa digested **C, G, K, O** testa pattern at the seed body of treated seeds showing anticlinal cell walls punctate (**C, J, O**) and with fibrils (**G, K, O**) **D** detail of ridge **H, L, P** detail of testa cells **F, N** hilar zone **R, T** detail of a non-digested portion of the seed coat. Scale bars: 200 μm (**A, B, E, I, J, M, Q, S**); 100 μm (**C, K, N, O**); 20 μm (**D, F, G, H, L, P, R, T**).

**Figure 15.**
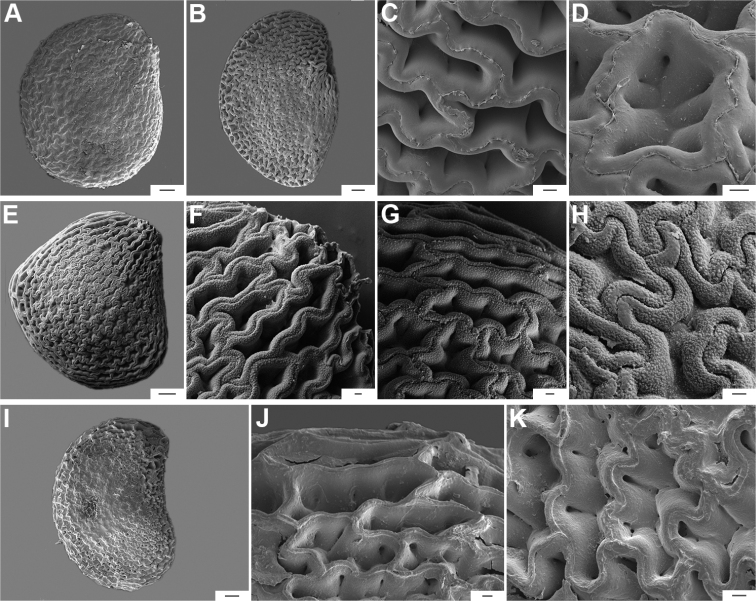
Seeds and seed coat morphology in species of the Andean Clade **A–D***C.hookerianum***E–H***C.piuranum***I–K***C.rhomboideum***A** untreated seed **B, E** treated seeds **C, D, G, J, K** testa pattern of treated seeds showing anticlinal cell walls punctate (**C, D, J, K**) and papillate (**G**) **F** hilar zone **H** detail of testa cells densely papillate **I** seed partly digested. Scale bars: 200 μm (**A, B, E, I**); 20 μm (**C, D, F, G, H, J, K**).

**Figure 16.**
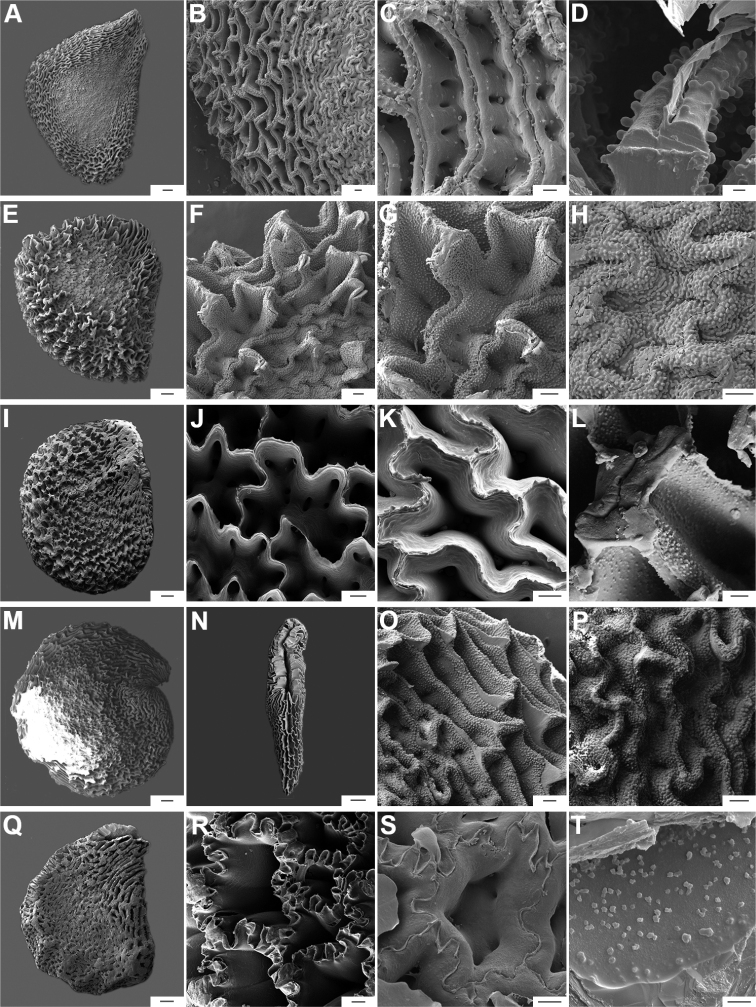
Seeds and seed coat morphology in species of the Bolivian Clade **A–D***C.caballeroi***E–H***C.ceratocalyx***I–L***C.minutiflorum***M–P***C.neei***Q–T***C.coccineum***A, Q** seeds with testa partly digested **B, F, G, J, O, R** marginal testa pattern of treated seeds showing anticlinal cell walls punctate and densely papillate (**G, O**), punctate (**J**) and with ridge (**R**) **C, H, K, P, S** detail of testa cells **D, L, T** detail of papillae **E, I, M** treated seeds **N** linear hilum. Scale bars: 300 μm (**A, E, I, M, N, Q**); 50 μm (**B, F, G, H, O, P**); 20 μm (**C, J, K, L, R, S**); 5 μm (**D, T**).

**Figure 17.**
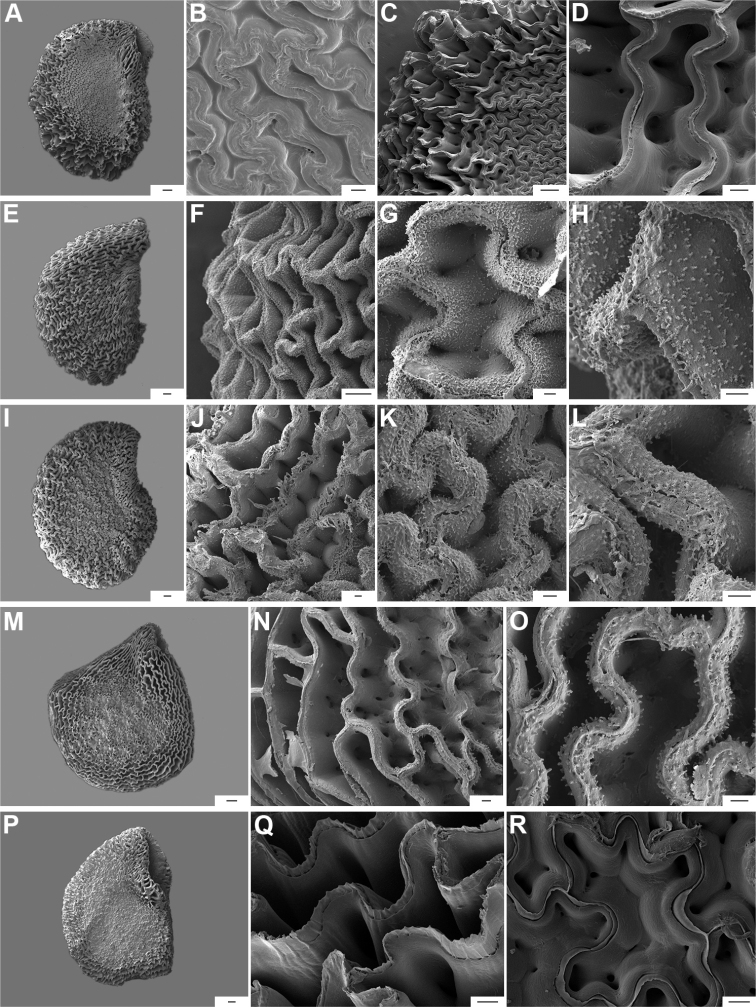
Seeds and seed coat morphology in species of the Caatinga, Longidentatum, Flexuosum and Tovarii Clades **A–D***C.caatingae***E–H***C.parvifolium***I–L***C.longidentatum***M–O***C.flexuosum***P–R***C.tovarii***A, M, P** seeds with testa partly digested (in A hilum in terminal position, P hilum subterminal) **B** detail of a non-digested portion of the seed coat **C, F, J, N, Q** marginal testa pattern of treated seeds showing anticlinal cell walls papillate (**F**), with fibrils (**J**), punctate (**N**) and with fringe (**Q**) **D, G, K, O, R** detail of testa cells **E, I** treated seeds **H, L** papillae on anticlinal cell walls Scale bars: 200 μm (**A, E, I, M, P**); 20 μm (**B, D, G, H, J, K, L, N, O, Q, R**); 100 μm (**C, F**).

**Figure 18.**
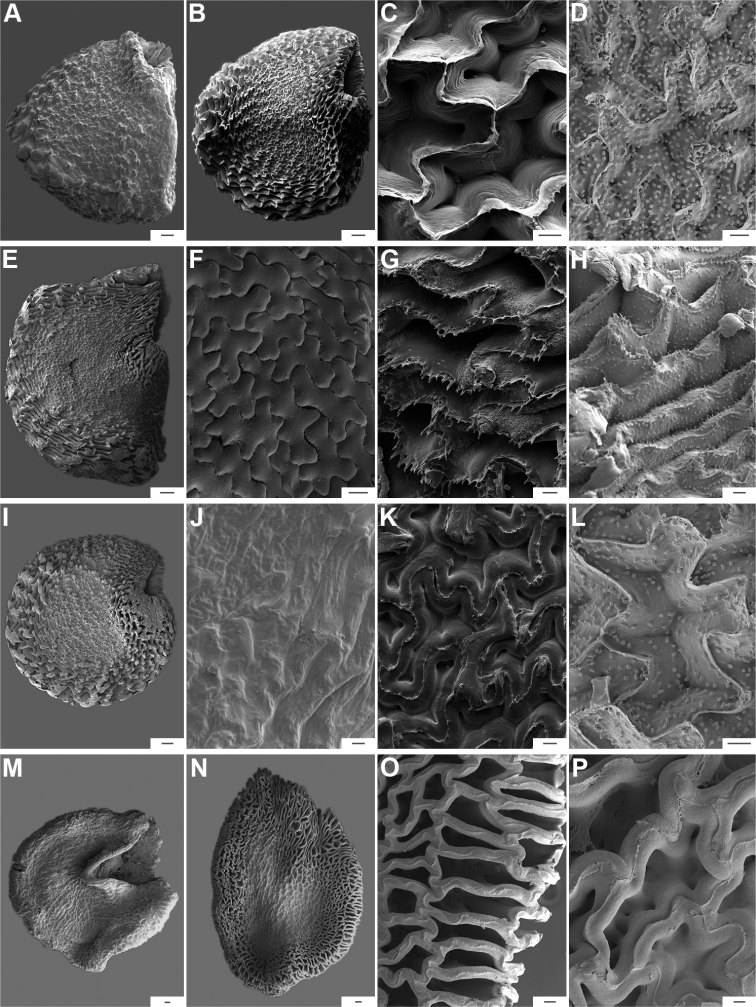
Seeds and seed coat morphology in species of the Purple corolla and Pubescens Clades **A–D***C.cardenasii***E–H***C.eshbaughii***I–L***C.eximium***M–P***C.pubescens***A, M** untreated seeds (in **A** hilum terminal, **M** hilum medial) **B** treated seed **C, G, K, O** testa pattern of treated seeds showing anticlinal cell walls with fringe (**C**) and fibrils (**G**) **D, H, L, M** detail of testa cells **E, I, N** seeds with testa partly digested (in **I** hilum subterminal) **F, J** detail of a non-digested portion of the seed coat. Scale bars: 200 μm (**A, B, E, I, M, N**); 20 μm (**C, D, G, H, J, K, L, P**); 50 μm (**F, O**).

Seed ornamentation refers to the appearance of the seed coat (testa) (Figs 9–18). The testa appearance varies in the majority of the species, depending on the magnification (naked eye, stereomicroscope or SEM) and whether or not the seeds are untreated or treated with a technique, such as enzyme etching. By removing the outer periclinal wall with enzyme etching, the ornamentation of the seed coat (sculpture) can be observed in detail under SEM. From the SEM data obtained of all treated seeds, the sculpture of the seed coat and variations in the anticlinal (lateral) walls of the outer epidermal layer vary between species and provide useful diagnostic features.

Five major types of seed coat sculpture were observed after the outer periclinal wall was removed by enzymatic digestion: (1) reticulate, with straight to wavy cell walls (Fig. 14B, C, M, O, for example, *C.dimorphum*); (2) cerebelloid, with sinuate to strongly sinuate cell walls (Fig. 15B, E, F, K, for example, *C.hookerianum*); (3) reticulate-cerebelloid, reticulate at the seed margins and cerebelloid in the seed body (Fig. 16A, B, for example, *C.caballeroi*); (4) reticulate with pillar-like outgrowths at margins (Fig. 11M, R, S, for example, *C.cornutum*, *C.mirabile*), these outgrowths due to unequal height of the anticlinal walls thickenings (Fig. 8C, D); and (5) cerebelloid with pillar-like outgrowths at margins (Fig. 11B, C, for example, *C.regale* and some seeds of *C.campylopodium* and *C.schottianum*). The most common combinations in the ornamentation of untreated/treated seeds in each species are: smooth to obscurely reticulate/cerebelloid (Figs 9I, K, 10A, C); reticulate, marginally tuberculate/reticulate with pillar-like outgrowths at margins (Figs 11I–K, Q–S, 12L–O, 13E–H); reticulate/reticulate (Fig. 14A–C); smooth and reticulate at margins or completely reticulate/reticulate-cerebelloid (Fig. 15I–K) (see Suppl. material 2: Appendix 2 for each species).

The anticlinal cell walls observed with SEM are either: (1) papillate, with papillae 2.5–4 µm in diameter on the cell walls (Figs 10H, 11D, H, T, 12C, G, K, T, 15H, 16D, H, L, P, T, 17H, L, O, 18D, H, L); and (2) punctate, with perforations (holes, ca. 35 µm in diameter) at the bottom (Figs 9L, 11G, H, L, T, 12C, G, 14L, 16S) or uniformly distributed on the cell walls (Figs 11O, P, 12P, T, 14P, 15D, K, 17G, N).

The distal end of the anticlinal cell walls may have three different types of appendages: (1) a thin ridge (Figs 9K, 10D, H, O, 11K, L, O, P, T, 12P, T, 14D, 15K), which is very common in the genus; (2) a more or less wide fringe (Figs 16K, L, R, S, 17O, Q, R, 18C); and (3) strands of thickenings differentiated as finger-like laciniations or fibrils (hair-like structures, Figs 9G, 10C, K, 14G, H, K, L, P, 17J, 18G, K).

### ﻿Embryo

The embryo in *Capsicum* is usually imbricate, meaning that the cotyledon tips are parallel or almost parallel to the radicle (Gunn and Gaffney 1974) and, less frequently, annular (cotyledon tips point toward the radicle, for example, *C.dimorphum*) or coiled (cotyledons coiled twice, Fig. 8B; for example, *C.chacoense*). The endosperm is firm, whitish and relatively abundant (Fig. 8B, C). Germination in *Capsicum* is epigeal.

## ﻿Floral biology and pollination

Most work on pollination and floral biology in *Capsicum* has been done with the domesticated species used for their fruits. *Capsicum* species are generally reported to be self-compatible, although the studied cases concerned mainly domesticated species and a few wild relatives (e.g. *C.annuum* and *C.galapagoense* from the Annuum clade or *C.baccatum* and *C.chacoense* from the Baccatum clade) (Onus and Pickersgill 2004; Cauich et al. 2006). Self-incompatibility has only been documented in *C.cardenasii*, as well as in some accessions of *C.pubescens* (Yaqub and Smith 1971; CCG, pers. obs). Successful self-pollinations have also been achieved in *C.cardenasii* (CCG pers. obs.), suggesting a variable degree of self-(in)compatibility.

Flowering phenology, with particular attention to the timing of gynoecium and androecium maturity, has been studied to improve pollination, fertilisation and, ultimately, the fruit set, as well as to analyse the chances of doing targeted crosses (e.g. through bud pollination). Anther dehiscence has been registered to occur after flower opening, whereas the stigma is receptive before anther dehiscence, even in the buds and receptivity is maintained throughout the lifespan of the flower (Alleemullah et al. 2000; Crispim et al. 2017; Peña-Yam et al. 2019). Anthers open by a longitudinal slit; the anther walls extend outwards completely, fully exposing the pollen grains, which are held together in clumps on the anther walls by means of pollenkitt. Pollen viability is highest at the time of anther dehiscence, gradually decreasing towards the end of the flower anthesis (Peña-Yam et al. 2019; CCG, pers. obs.). Temperature has proven to be a key factor for pollen and pollen tube development, as well as for fertilisation, with high temperature being unfavourable (Aloni et al. 2001; Erickson and Markhart 2002; Reddy and Kakani 2007; Kafizadeh et al. 2008).

*Capsicum* species have served as models to examine unilateral self-incompatibility using reciprocal interspecific crosses mainly between the domesticated species and their closest relatives, both within and between clades that include domesticated species (e.g. Tong and Bosland 2003). Interspecific hybridisations can be successful amongst closely-related species (e.g. *C.annuum* × *C.frutescens*, *C.pubescens* × *C.eximium*), but crosses are not always successful or are only so in one non-reciprocal sense (e.g. *C.chinense*-male × *C.baccatum*-female, *C.annuum*-male × *C.pubescens*-female) (Emboden 1961; Peter and McCollum 1984; Tong and Bosland 2003; Onus and Pickersgill 2004; Vilagelim Costa et al. 2009; da Silva Monteiro et al. 2011; Kamvorn et al. 2014; Martins et al. 2015).

Nectar production and its presentation in *Capsicum* is due to the formation of nectar ducts, structures also found in other Solanaceae genera, such as *Jaltomata* Schltdl., *Physalis* L. (Vogel 1998; Cocucci 1999) and *Mellissia* Hook.f. (Fay et al. 2007). The base of the ovary contains a nectary disc that is enveloped by the narrow basal tube formed by the corolla and the adnate filaments of the stamens. The nectar passes through individual ducts formed between adjacent filaments and is exposed on the corolla limb as isolated droplets that are radially arranged at the base of the limb and alternating with the filaments (Fig. 5I). Even though nectar ducts are elaborate systems, such mechanism of nectar presentation, as an easily accessible reward, would lead to generalist pollinators (Vogel 1998), such as bees. Bees have been documented as effective pollinators of *Capsicum* (Kristjansson and Rasmussen 1991; Rabinowitch et al. 1993; Cauich et al. 2006; Cruz and Olivera de Campos 2007; Palma et al. 2008).

## ﻿Fruit and seed dispersal

Wild *Capsicum* species typically produce colourful, juicy, fleshy, conspicuous, many-seeded berries that are attractive to their consumers. The fruits in most *Capsicum* species contain capsaicinoids (mainly capsaicin), the chemical principles responsible for their pungency, which are highly concentrated in the placental and septum tissues. Some authors (Mason et al. 1991; Norman et al. 1992; Tewksbury and Nabhan 2001) have experimentally proved that capsaicin repels rodents (house mice: *Musmusculus*; deer mice: *Peromyscusmaniculatus*; Norway rats: *Rattusnorvegicus*; cactus mice: *Peromyscuseremicus*; packrats: *Neotomalepida*). This contrasts to the insensitivity of birds to capsaicin (European starlings: *Sturnusvulgaris*; parrots: *Amazona* spp.; pigeons; red-winged blackbirds: *Agelaiusphoeniceus*; cedar waxwings: *Bombycillacedrorum*; house finches: *Carpodacusmexicanus*; curve-billed thrashers: *Toxostomacurvirostre*; northern cardinal: *Cardinaliscardinalis*, northern mockingbird: *Mimuspolyglottos*), which may reflect basic differences in trigeminal chemoreception (Mason et al. 1991 and literature therein; Norman et al. 1992; Tewksbury et al. 1999; Levey et al. 2006). Tewksbury and Nabhan (2001) proposed the directed deterrence by capsaicin hypothesis which predicts that this compound in ripe peppers fruits functions to selectively discourage fruit consumption by non-seed dispersing vertebrates without deterring beneficial seed dispersers. Levey et al. (2006) tested this hypothesis in the field with C.annuumvar.glabriusculum (North America: Arizona) and *C.chacoense* (South America: Bolivia), both of which have upright, pungent, red fruits. Their studies support the hypothesis that *Capsicum* fruit consumption is diurnally facilitated by bird communities (Passeriformes: Fam. Tyrannidae, Turdidae, Mimidae, Cardinalidae and others), whereas mammals avoid consumption of the spicy fruits because of aversion to capsaicin (Jordt and Julius 2002). In addition, the gut passage and retention time of pepper seeds in birds enhance seed germination rates (Tewksbury and Nabhan 2001; Levey et al. 2006; Tewksbury et al. 2008a; Fricke et al. 2013). Frugivorous birds would be the legitimate seed dispersers of chili peppers. Rodents appear to be only significant as *Capsicum* seed predators, first when they directly consume the non-pungent fruits of *C.chacoense*, resulting in the destruction of the seeds and 0% germination (Tewksbury and Nabhan 2001) and second, when they consume the post-dispersal seed defecated by birds (Noss and Levey 2014).

It is expected that the non-pungent red or orange fruits of the Andean *Capsicum* species are also dispersed by birds. It would be would be interesting to test in nature if the directed deterrence hypothesis functions in a similar way in the Brazilian Atlantic forest species whose greenish-golden yellow fruits are not as showy as the red-fruited species; they are pendent and are somewhat masked amongst the copious green foliage of the plant, perhaps attractive to fauna moving underneath the plants.

## ﻿Cytogenetics

*Capsicum* species are mostly diploid, with two chromosome numbers: 2n = 24 (x = 12) and 2n = 26 (x = 13), the latter appearing only in wild species (Heiser and Smith 1958; Pickersgill 1971, 1977, 1991; Eshbaugh et al. 1983; Moscone 1990, 1992, 1993, 1999; Moscone et al. 1996a, 2007; Tong and Bosland 2003; Pozzobon and Schifino-Wittmann 2006; Pozzobon et al. 2006; Barboza et al. 2011, 2019, 2020b; Scaldaferro et al. 2013, 2016; Scaldaferro and Moscone 2019). The number of species belonging to the x = 13 group has increased as new taxa have been discovered and now almost equals those with x = 12 (Table 2). In addition, although polyploidy is very rare in *Capsicum*, Greenleaf (1947), Pickersgill (1977) and Jha et al. (2012) described what they believed to be natural polyploids, triploids and tetraploids with 2n = 3x = 36 and 2n = 4x = 48, respectively. Polyploid forms can also be induced as has been shown (Gyorffy 1939; Nishiyama 1939, 1940; Pal and Ramanujam 1939; Pal et al. 1941; Aleksic 1960; Palfi et al. 1961; Ohta 1962c; Siskovic 1962; Raghuvanshi and Joshi 1964; Indira and Susan 1977; Panda et al. 1984; Rao 1987; Ishikawa et al. 1997; Ishikawa 2001; Kumar and Raja Rao 2003; Kumar et al. 2004; Takizawa et al. 2008; Kulkarni and Borse 2010).

**Table 2.** Cytogenetic information on *Capsicum* species.

Taxon and voucher number	n	2n	Haploid karyotype formula	Chromosomes with active NORs	Hc amount (HKL in µm)	1C DNA co ntent in pg	References
C.annuumvar.annuum							
No voucher cited	-	24	-	-	-	-	Pickersgill 1971, 1977, 1991
cytotype 1 EAM 193, 251, 203	-	24	10 m + 1 sm + 1 st	11 sm	1.80 (68.51)	3.41*	Moscone et al. 1993, 1995, 1996a, 2003*, 2007
cytotype 2 EAM 204, 252; NMCA 10544, 10272	-	24	10 m + 1 sm + 1 st	11 sm, 12 st	2.88 (70.40)	3.32*	Moscone et al. 1993, 1995, 1996a, 2003*, 2007
Cuneo w.no. Doux Long des Landes w.no.	-	-	-	-	-	3.83†	Belletti et al. 1998†
C.annuumvar.glabriusculum							
No voucher cited	-	24	-	-	-	-	Pickersgill 1971 (as C.annuumvar.minimum), 1977, 1991
cytotype 1 NMCA 10955	-	24	10 m + 1 sm + 1 st	11 sm	2.26 (59.53)	-	Moscone et al. 2007
cytotype 2 NMCA 10983	-	24	11 m + 1 st	1 m, 5 m	3.54 (51.95)	-	Moscone et al. 2007
cytotype 3 LQ w. no	-	24	11 m + 1 st	11 m	2.33 (55.13)	-	Scaldaferro et al. 2013
cytotype 4 YSG w. no	-	24	11 m + 1 st	5 m, 12 st	6.33 (53.56)	-	Scaldaferro et al. 2013
cytotype 5 Neth 804750009	-	24	11 m + 1 sm	12 sm	3.37 (55.43)	-	Scaldaferro et al. 2013
cytotype 6 PI 511885	-	24	11 m + 1 st	1 m, 5 m, 6 m, 12 st	2.97 (80.38)	-	Scaldaferro et al. 2013
cytotype 7 PI 511886	-	24	11 m + 1 st	1 m, 2 m, 5 m, 8 m	3.83 (70.05)	-	Scaldaferro et al. 2013
C.baccatumvar.baccatum							
Vouchers not cited	-	24	-	-	-	-	Pickersgill 1977
GEB 163	-	24	11 m + 1 st	1 m, 3 m, 10 m, 12 st	7.45 (66.84)	3.71*	Moscone et al. 2003*, 2007
Tuscia University, Italy w. no	-	-	-	-	-	4.22^†^	Belletti et al. 1998 ^†^
C.baccatumvar.pendulum							
No voucher cited	-	24	-	-	-	-	Pickersgill 1971, 1977
cytotype 1 EAM 192, 209	-	24	11 m + 1 st	1 m, 3 m, 12 st	7.30 (75.53)	3.71*	Moscone et al. 1993, 1995, 1996a, 2003*, 2007
cytotype 2 EAM 205, 206, 247; ATH 25382; EAM & RN 211	-	-	11 m + 1 st	1 m, 3 m, 10 m, 12 st	7.56 (74.31)	3.68*	Moscone et al. 1993, 1995, 1996a, 2003*, 2007
Tuscia University, Italy w. no, Sao Paulo University w. no	74.92	-	-	-	-	4.20^†^	Belletti et al. 1998 ^†^
C.baccatumvar.umbilicatum							
EAM 197, 253	-	24	11 m + 1 st	1 m, 3 m, 10 m, 12 st	9.06 (74.27)	3.76*	Moscone 1999; Moscone et al. 2003*
*C.caatingae*							
LBB 1560	12	-	-	-	-	-	Pozzobon and Schifino- Wittmann 2006
LBB 1560	12	24	-	-	-	-	Pozzobon et al. 2006 (as *C.parvifolium*)
cytotype 1 ATH 25233	-	24	11 m + 1 st	12 st	5.52 (82.40)	-	Moscone 1993; Moscone et al. 1993a, 1995; 2007
cytotype 2 ATH 25233 bis	-	24	12 m	12 m	7.47 (77.60)	5.77*	Moscone et al. 2003* (as *C.parvifolium*)
*C.caballeroi*							
GEB et al. 3655	-	24	-	-	-	-	this monograph
*C.campylopodium*							
LBB 1566	13	-	-	-	-	-	Pozzobon and Schifino- Wittmann 2006
LBB 1566	13	26	-	-	-	-	Pozzobon et al. 2006
cytotype 1 ATH 25116	-	26	5 m + 6 sm + 1 st + 1 t	7 sm	32.49 (88.30)	5.74*	Moscone et al. 2003*, 2007
cytotype 2 ATH 25128, 25130, 25136	-	26	10 m + 2 sm + 1 st	11 sm	20.41 (87.95)	4.53*	Moscone et al. 2003*, 2007
*C.cardenasii*							
Heiser 4196	12	-	-	-	-	-	Heiser and Smith 1958
No voucher cited	-	24	-	-	-	-	Pickersgill 1977
CORD 135	-	24	-	-	-	-	Moscone et al. 2007
cytotype 1 Neth 904750136	-	24	11 m + 1 sm	7 m, 12 sm	6.91 (62.00)	-	Scaldaferro et al. 2016
cytotype 2 AAC w. no; GEB w. no	-	24	11 m + 1 sm	7 m, 12 sm	10.41 (76.01)	-	Scaldaferro et al. 2013, 2016
Budapest, Hungary w. no	-	-	-	-	-	4.49^†^	Belletti et al. 1998 ^†^
*C.chacoense*							
Argentina (Córdoba), no voucher cited	12	-	-	-	-	-	Schnack and Covas 1947 (as *C.microcarpum*)
No voucher cited	-	24	-	-	-	-	Pickersgill 1977
LB et al. 498	12	-	-	1 m, 12 st	-	-	Moscone 1992
cytotype 1 EAM 104, 195, 207, 250; AAC et al. 973	-	24	11 m + 1 st	1 m, 12 st	2.94 (65.02)	3.34*	Moscone 1990; Moscone et al. 1993, 1995, 2003*, 2007; [Bibr B427][Bibr B307]; Moscone et al. 1993, 1995, 2003*, 2007
cytotype 2 LB & LG 525	-	24	11 m + 1 st	-	2.44 (71.25)	3.36*	
Tuscia University, Italy w. no	-	-	-	-	-	3.83^†^	Belletti et al. 1998 ^†^
*C.chinense*							
C 323, C 324	12	-	-	-	-	-	Pickersgill 1966
No voucher cited	-	24	-	-	-	-	Pickersgill 1977
cytotype 1 GEB et al. 797; GEB 807	-	24	11 m + 1 st	12 st	3.91 (61.31)	3.43*	Moscone et al. 2003*, 2007
cytotype 2 EAM 201	-	24	11 m + 1 st	12 st	5.52 (61.36)	3.41*	Moscone et al. 2003*, 2007
LBB 1720	12	-	-	-	-	-	Pozzobon et al. 2006
MVR 9	-	24	11 + 1 st	12 st	-	-	Romero da Cruz and Forni Martins 2015
Sao Paulo University w. no, Reading University U. K. w. no, I.N.R.A. France w. no	-	-	-	-	-	4.02^†^	Belletti et al. 1998 ^†^
*C.cornutum*							
LBB 1542, 1546	13	-	-	-	-	-	Pozzobon and Schifino-Wittmann 2006
LBB 1546	-	26	-	-	-	-	Pozzobon et al. 2006
*C.eshbaughii*							
CCG 91	-	24	-	-	-	-	Carrizo García et al. 2020
*C.eximium*							
No voucher cited	12	-	-	-	-	-	Heiser and Smith 1958
No voucher cited	-	24	-	-	-	-	Pickersgill 1977
cytotype 1 EAM 254	-	24	11 m + 1 sm	7 m, 12 sm	4.90 (68.89)	4.06*	Moscone et al. 2003*, 2007
cytotype 2 EAM 255	-	-	11 m + 1 sm	7 m, 12 sm	2.10 (69.65)	-	Moscone et al. 2007; Scaldaferro et al. 2013; Debat et al. 2017
University of Reading, U. K. w. no, Budapest, Hungary w. no	-	-	-	-	-	4.35^†^	Belletti et al. 1998 ^†^
*C.flexuosum*							
RSu, EAM 4133	12	-	-	-	-	-	Moscone 1992
LBB 1552	12	-	-	-	-	-	Pozzobon and Schifino-Wittmann 2006
LBB 1552	-	24	-	-	-	-	Pozzobon et al. 2006
GEB et al. 3631	-	24	-	-	-	-	this monograph
GEB et al. 1034; JD & AIH 599	-	24	11 m + 1 st	2 m, 5 m	16.82 (103.69)	-	Moscone et al. 2007; Scaldaferro et al. 2013; Debat et al. 2016
No voucher cited	-	-	-	-	-	7.2^¤^	Jarret et al. 2019 ^¤^
*C.friburgense*							
LBB 1565	13	-	-	-	-	-	Pozzobon and Schifino-Wittmann 2006; Pozzobon et al. 2006
*C.frutescens*							
No voucher cited	-	24	-	-	-	-	Pickersgill 1977
GEB et al. 795; EAM 200	-	24	11 m + 1 st	1 m, 12 st	5.55 (66.63)	3.40*	Moscone et al. 1996; 2003*, 2007
Tuscia University, Italy w. no, Sao Paulo University w. no	-	-	-	-	-	3.97^†^	Belletti et al. 1998 ^†^
*C.galapagoense*							
No voucher cited	12	-	-	-	-	-	Heiser and Smith 1958
No voucher cited	-	24	-	-	-	-	Pickersgill 1977
PI 639682	-	24	11 m + 1 st	12 st	2.24 (48.66)	-	Moscone et al. 2007; Scaldaferro et al. 2013
*C.hunzikerianum*							
GEB et al. 5041	-	26	-	-	-	-	this monograph
*C.lanceolatum*							
NMCA 90016	13	-	-	-	-	-	Tong and Bosland 2003
*C.longidentatum*							
MFA & GEB 7086	-	24	12 m	12 m	-	-	Barboza et al. 2011
*C.longifolium*							
GEB & SLG 4821	-	26	9 m + 3 sm + 1 st	10 sm	3.77 (23.86)	-	Barboza et al. 2019
*C.lycianthoides*							
GDB 85	-	26	9 m + 3 sm + 1 st	10 sm	-	-	Scaldaferro and Moscone 2019
*C.mirabile*							
NMCA 50029	13	-	-	-	-	-	Tong and Bosland 2003
LBB 1559, 1564, 1568	13	-	-	-	-	-	Pozzobon and Schifino-Wittmann 2006 (as *Capsicum* sp 6)
LBB 1550, 1554	13	-	-	-	-	-	Pozzobon and Schifino-Wittmann 2006 (as *C.buforum*)
cytotype 1 ATH 25238, 25251	-	26	9 m + 2 sm + 1 st + 1 t	7 m	29.64 (83.81)	-	Moscone et al. 2007
cytotype 2 ATH 25238, 25255	-	26	8 m + 3 sm + 1 st + 1 t	7 m	29.25 (93.72)	-	Moscone et al. 2007
cytotype 3 ATH 25238, 25267	-	26	9 m + 3 sm + 1 t	9 m	30.93 (103.4)	-	Moscone et al. 2007
*C.parvifolium*							
MFA & GEB 7075	-	24	11 m + 1 sm	12 sm	-	-	Barboza et al. 2011; Romero da Cruz et al. 2017
*C.pereirae*							
LBB 1558	13	-	-	-	-	-	Pozzobon and Schifino-Wittmann 2006
LBB 1558	-	26	-	-	-	-	Pozzobon et al. 2006 (as *Capsicum* sp 7)
cytotype 1 ATH 26137	-	26	9 m + 1 sm + 2 st + 1 t	4 m, 11 st	11.42 (74.52)	-	Moscone et al. 2007
cytotype 2 ATH 25249	-	26	10 m + 2 st + 1 t	6 m, 11 st	16.04 (75.85)	-	Moscone et al. 2007
*C.piuranum*							
GEB & SLG 4841	-	26	9 m + 3 sm + 1 st	10 sm	2.84 (22.97)	-	Barboza et al. 2019
*C.pubescens*							
No voucher cited	-	24	-	-	-	-	Pickersgill 1977, 1991
GEB 79; EAM 198, 202, 208, 256, 257	-	24	11 m + 1 st	10 m, 12 st	18.95 (80.53)	4.47*	Moscone et al. 1993, 1995, 1996a, 2003*, 2007
Budapest, Hungary w. no	-	-	-	-	-	4.86^†^	Belletti et al. 1998 ^†^
*C.rabenii*							
No voucher cited	12	-	-	-	-	-	Heiser and Smith 1958
No voucher cited	-	24	-	-	-	-	Pickersgill 1977
LBB 1553, 1555	12	-	-	-	-	-	Pozzobon and Schifino-Wittmann 2006
LBB 1524, 1553, 1555	-	24	-	-	-	-	Pozzobon et al. 2006 (as C.baccatumvar.praetermissum)
cytotype 1 PI 441654	-	24	11 m + 1 st	7 m, 12 st	10.96 (72.55)	-	Moscone et al. 2007
cytotype 2 EFM 05-17	-	24	11 m + 1 sm	6 m, 12 sm	14.92 (76.20)	-	Grabiele et al. 2014; Scaldaferro et al. 2016
Budapest, Hungary w. no, Gatersleben, Germany w. no	-	-	-	-	-	4.57^†^	Belletti et al. 1998 ^†^
*C.recurvatum*							
LBB 1523	13	-	-	-	-	-	Pozzobon and Schifino- Wittmann 2006
LBB 1523	-	26	-	-	-	-	Pozzobon et al. 2006 (as *Capsicum* sp. 2)
GEB et al. 915; GEB et al. 1629, 1632	-	26	10 m + 2 sm + 1 st	12 sm	5.73 (68.55)	-	Moscone et al. 2007; Scaldaferro and Moscone 2019
*C.regale*							
AOR et al. 3034	-	26	-	-	-	-	Barboza et al. 2020b
*C.rhomboideum*							
No voucher cited	-	26	-	-	-	-	Pickersgill 1977 (as *C.ciliatum*)
YSG 19, 20	-	26	10 m + 1 sm + 2 st	9 m	4.88 (42.13)	-	Moscone et al. 2007; Scaldaferro et al. 2013; Aguilera et al. 2014
No voucher cited	-	-	-	-	-	2.08^¤^	Jarret et al. 2019 ^¤^
*C.schottianum*							
LBB 1535, 1536, 1540, 1544, 1545	13	-	-	-	-	-	Pozzobon and Schifino-Wittmann 2006
LBB 1535, 1540	-	26	-	-	-	-	Pozzobon et al. 2006
ATH 25160	-	26	9 m + 2 sm + 1 st + 1 t	11 sm	23.28 (93.71)	-	Moscone et al. 2007
*C.tovarii*							
No voucher cited	12	-	-	-	-	-	Eshbaugh et al. 1983
cytotype 1 ATH & GEB 25653	-	24	11 m + 1 sm	10 m, 12 sm	38.91 (70.32)	-	Moscone et al. 2007
cytotype 2 NMCA 90008	-	24	11 m + 1 sm	6 m, 7 m, 12 sm	4.89 (67.02)	-	Scaldaferro et al. 2016
The Netherlands w. no	-	-	-	-	-	3.97 ^†^	Belletti et al. 1998 ^†^
*C.villosum*							
LBB 1538, 1539, 1543, 1557	13	-	-	-	-	-	Pozzobon and Schifino-Wittmann 2006
LBB 1538, 1543, 1557	-	26	-	-	-	-	Pozzobon et al. 2006
ATH 25169; GEB et al. 1653	-	26	9 m + 3 sm + 1 t	12 sm	9.74 (75.89)	-	Moscone et al. 2007; Scaldaferro and Moscone 2019

Abbreviations of collector’s name: MFA MF Agra; AA A Anton; GEB GE Barboza; LBB L de Bem Bianchetti; GDB GD Beltrán; LB L Bernardello; LB L Bohs; CCG C Carrizo García; AAC AA Cocucci; JD J Daviña; EFM E Forni Martins; LG L Galetto; AIH AI Honfi; ATH AT Hunziker; SLG S Leiva González; LQ Llatas Quiroz; EAM EA Moscone; RN R Neumann; AR A Romanutti; MVR María V. Romero; AOR A Orejuela; YSG Y Sanchez García; GS G Sierra; RSu R Subils; Neth collection number of Nijmegen University, the Netherlands; NMCA collection number of the College of Agriculture, New Mexico State University; PI collection number of the United States Department of Agriculture (Griffin, GA). Abbreviations: - unknown data; Hc heterochromatin amount; HKL haploid karyotype length; var. variety; w. no. without number; m metacentric; sm submetacentric; st subtelocentric; t telocentric; w.no. without number.

Cytogenetics provides a valuable and irreplaceable source of information for solving taxonomic, evolutionary and applied questions (Poggio 1996; Guerra 2012). To this end, a comprehensive cytogenetic characterisation has been carried out in a large number of species in the genus. Techniques have included classical staining to indicate chromosome number and karyotype formula, silver staining to expose chromosomes with active nucleolar organiser regions (NORs), fluorescent banding to show heterochromatin amount (Hc) and distribution, fluorescence *in situ* hybridisation (FISH) to denote number and distribution of rDNA sites and flow cytometry and Feulgen densitometry to estimate nuclear DNA content (Pickersgill 1971, 1977, 1991; Moscone 1990, 1993, 1999; Moscone et al. 1993, 1995, 1996a, b, 1999, 2003, 2007; Belletti et al. 1998; Park et al. 2000; Pozzobon et al. 2006; Scaldaferro et al. 2006, 2013, 2016; Barboza et al. 2011, 2019, 2020b; Romero-da Cruz et al. 2016; Jarret et al. 2019; Scaldaferro and Moscone 2019; Carrizo García et al. 2020).

Almost half of the taxa that have been cytogenetically studied exhibit intraspecific karyotype variation, differing in karyotype formulas, number and location of active NORs, heterochromatin content and banding patterns (Moscone et al. 2007; Scaldaferro et al. 2013, 2016; Scaldaferro and Moscone 2019). The chromosome number of 2n = 26 for one species of the Atlantic Forest clade, *C.hunzikerianum*, is reported in this monograph for the first time, as is the number of 2n = 24 for *C.caballeroi* of the Bolivian clade (Table 2).

*Capsicum* disploidy (the presence of two basic chromosome numbers) has been examined in relation to genome size evolution and species diversification. The chromosome number 2n = 24 is dominant across the recognised *Capsicum* clades, whereas the 2n = 26 taxa are restricted to Andean and Atlantic Forest clades only. These last two clades are the more species-rich and include almost one-half of wild species of the genus.

Species with 2n = 24 chromosomes show rather uniform and comparatively the most symmetrical karyotypes, with the 11 m + 1 st karyotype formula, although 11 m + 1 sm is also frequent. In contrast, species with 2n = 26 karyotype formulas have more asymmetric, with nine different karyotypes amongst ten taxa. Out of these, the species of the Atlantic Forest clade are the most asymmetric, with seven different karyotype formulas found amongst them (Table 2).

It has been suggested that species with 13 chromosome pairs are derived from species with 12 pairs, since the latter have more symmetrical karyotypes (Pickersgill 1991; Moscone et al. 1993, 1995, 1996b, 2007; Tong and Bosland 2003; Barboza et al. 2011, 2019; Scaldaferro et al. 2013, 2016; Scaldaferro and Moscone 2019). However, the opposite scenario has also been proposed by Pozzobon et al. (2006), who hypothesised that x = 13 is the ancestral chromosome number of the genus and that the reduction in chromosome number is the result of the loss of the small 13^th^ chromosome pair. Even though the origin and fate of this chromosome pair is not known, the occurrence of x = 13 taxa in distinct clades from x = 12 taxa (Carrizo García et al. 2016) suggests that the extra chromosome(s) arose and/or was lost on more than one occasion. It is conceivable that two 2n = 26 subgroups with asymmetrical karyotypes have arisen via a centric fission in a metacentric chromosome (Moscone et al. 2007; Scaldaferro et al. 2013). One subgroup of 2n = 26 is composed by all species of the Andean clade, which have smaller genomes, single heterochromatic banding patterns and one nucleolar organiser region (NOR) per haploid complement (Scaldaferro pers.obs.). The other subgroup includes *C.mirabile*, *C.campylopodium*, *C.cornutum*, *C.friburgense*, *C.pereirae*, *C.recurvatum*, *C.schottianum* and *C.villosum*, all representatives of the Atlantic Forest clade. These taxa have larger genomes and rich heterochromatic regions with complex banding patterns. They also contain AT-rich, GC-rich and moderately GC-rich satellite DNA in addition to one or two NORs (Moscone et al. 2007; Scaldaferro et al. 2013; Scaldaferro and Moscone 2019). The 13^th^ chromosome pair shows distinctive characteristics amongst the subgroups.

Heterochromatin amount (Hc), indicated as percentage of haploid karyotype length (HKL), is quite variable in the genus (from 1.80 to 38.91) and correlates positively with the HKL in most of the taxa. *Capsicumannuum* and *C.tovarii* have the lowest and highest Hc, respectively, but across clades, the Annuum clade has the lowest Hc, whereas the highest Hc is found in the Atlantic Forest clade (Table 2).

DNA content analysis and characterisation of the 5S and 18S-5.8S-26S (45S) rDNA by FISH has been completed for only a few species of *Capsicum* (Belletti et al. 1998; Park et al. 2000; Moscone et al. 2003; Scaldaferro et al. 2006, 2016; Romero-da Cruz et al. 2016; Jarret et al. 2019; Scaldaferro and Moscone 2019). Although information is still lacking for many species/clades (e.g. the complete Atlantic Forest clade), genome sizes are highly variable between *Capsicum* species. This range of variation may be caused by different components of the genome that can modify the structure and composition of DNA; for example, more than 81% of the genome is composed of transposable elements in *C.annuum* (Qin et al. 2014).

## ﻿Domestication

Five *Capsicum* species, *C.annuum*, *C.chinense*, *C.frutescens*, *C.baccatum* and *C.pubescens*, were independently domesticated for their fruits in different areas of Central and South America (Eshbaugh 1970, 1979; Pickersgill and Heiser 1977; Aguilar-Meléndez et al. 2009; Albrecht et al. 2012a, Albrecht et al. 2012b; Kraft et al. 2014; Scaldaferro et al. 2018). Archaeological records from ca. 8000 BCE indicate the presence of *Capsicum* species remains associated with human settlements in northern Peru (Dillehay et al. 2017). At least three *Capsicum* species (*C.frutescens*, *C.chinense* and *C.baccatum*) would have been cultivated/consumed along the Andes from around 4000–4500 BCE (Perry et al. 2007; Clement et al. 2010; Kraft et al. 2014). Paleobiolinguistic data suggest that Mesoamerican cultures had names for chili peppers by ca. 4500 BCE, such as in the Proto-Otomanguean language (Brown et al. 2013; Kraft et al. 2014), evidence that chilies were recognised and possibly consumed by the local people at that time. Different sources of evidence reveal that *Capsicum* species were used by Meso- and South American peoples as spices due to their pungent fruits (Yacovleff and Herrera 1935; Powis et al. 2013). On the whole, archaeological remains show that *Capsicum* species, together with beans (*Phaseolus* spp.) and several pumpkins (*Cucurbita* spp.), were amongst the first cultivated plants in the Americas (Pickersgill 1969b) that were domesticated by humans. Domesticated chili peppers spread outside the Americas after Columbus’ expeditions in the 15^th^ century, first introduced into Europe and subsequently into Africa and Asia (Powis et al. 2013). Since that time, thousands of landraces have been developed in different regions around the world, some of which are currently recognised as secondary diversification centres (Nicolaï et al. 2013).

Domestication processes typically modify a few genes that affect domestication traits (Guerra-García and Piñero 2017). *Capsicum* species are cultivated for their fruits and are likely to have low quantities of proteins, carbohydrates and fats, then it is the presence of capsaicinoids, the compounds responsible for fruit pungency, the valuable and desired feature that may have led to its domestication. In cases like *Capsicum*, in which the target of selection has been the dispersal unit, modifications directed to reduce dispersal efficiency would increase the yield. For *Capsicum*, selection was orientated towards traits that would diminish fruit natural dispersal, as well as their consumption by birds, such as non-deciduous fruits and larger sizes. In addition to the loss of natural dispersal mechanisms, plants subjected to domestication can present other modifications regarded as part of the domestication syndrome in *Capsicum*, that is: changes in the reproductive system, increased morphological variability, changes in habit, loss of seed dormancy, loss of chemical or mechanical protection, as well as a variable degree of fruit pungency (Gepts 2004; Pickersgill 2007; Luna-Ruiz et al. 2018). All or some of these traits can be observed in the different domesticated species/varieties that belong to the Annuum (e.g. C.annuumvar.annuum), Baccatum (e.g. C.baccatumvar.pendulum) and Pubescens (*C.pubescens*) clades.

## ﻿Distribution and habitat

**Figure 19.**
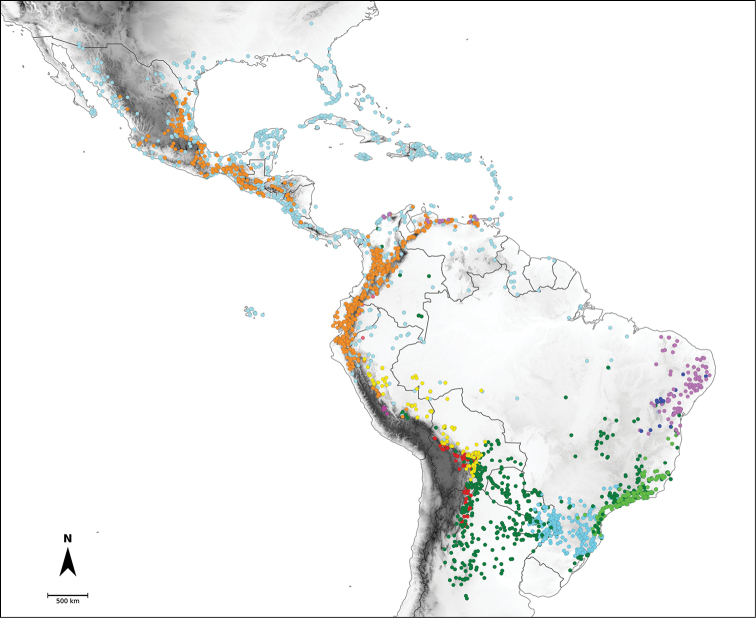
*Capsicum* geographic distribution. Georeferenced collection points of all wild *Capsicum* species/varieties. Circles are coloured by clades (Andean: orange; Atlantic Forest: bright green; Flexuosum: bright light blue; Caatinga: lilac; Longidentatum: dark blue; Bolivian: yellow; Purple corolla: red; Tovarii: fuchsia; Baccatum: dark green; Annuum: pale light blue).

*Capsicum* species are widely distributed across the Americas, from central Argentina and southern Brazil to the southern extreme of the United States of America, although most of the clades recognised here correspond to a particular geographic region (Fig. 19). Approximately half of the species are country-level endemics in South America: 14 species in Brazil (*C.caatingae*, *C.campylopodium*, *C.carassense*, *C.cornutum*, *C.friburgense*, *C.hunzikerianum*, *C.longidentatum*, *C.mirabile*, *C.mirum*, *C.muticum*, *C.pereirae*, *C.recurvatum*, *C.schottianum* and *C.villosum*), six species in Bolivia (*C.caballeroi*, *C.cardenasii*, *C.ceratocalyx*, *C.eshbaughii*, *C.minutiflorum* and *C.neei*) and two species in each of Ecuador (*C.benoistii* and *C.galapagoense*) and Peru (*C.piuranum* and *C.tovarii*). Another five species are native to only two countries: *C.longifolium* and *C.hookerianum* to Ecuador and Peru, *C.lycianthoides* to Ecuador and Colombia, *C.eximium* to Argentina and Bolivia and *C.rabenii* to Brazil and Paraguay. The remaining species have wider distributions, the most widely distributed are C.annuumvar.glabriusculum, C.baccatumvar.baccatum, *C.chacoense* and *C.rhomboideum*. Species richness is highest in the Andean Region (22 species), concentrated in Bolivia and Peru, each with 14 species.

Wild *Capsicum* species are found in a wide variety of habitats, from xeric shrublands to rainforests (Suppl. material 3: Appendix 3). These habitats represent around 120 ecoregions, which belong to 10 different biomes (Suppl. material 3: Appendix 3). The high number of ecoregions represented is mainly due to the broad distribution of C.annuumvar.glabriusculum (Fig. 24), spanning a total of ca. 100 ecoregions (particularly diverse in the southern United States of America, Mexico and Central America, including both continental and insular ranges) from nine out of the 10 possible biomes registered in *Capsicum* (Suppl. material 3: Appendix 3). Most species are found in habitats that correspond to the ‘tropical and subtropical moist broadleaf forest’ biome, except for *C.cardenasii* and *C.galapagoense*, the first of which occupies ‘montane grasslands and shrublands’ and ‘tropical and subtropical dry broadleaf forests’ biomes and the second of which occupies ‘deserts and xeric shrublands’ (Suppl. material 3: Appendix 3). The ‘tropical and subtropical dry broadleaf forests’, as well as the ‘tropical and subtropical grasslands, savannahs and shrubland’ biomes are next in number of species and include representatives of most clades (except for *C.tovarii* from the Tovarii clade; Suppl. material 3: Appendix 3). The remaining biomes are less represented, which encompass forests, shrublands and/or grasslands, ranging from desert to flooded conditions, including mangroves, as well as both tropical and temperate climates. The richest and most diverse clades in terms of biomes (and ecoregions) are found amongst the Baccatum and Annuum clades that include the domesticated species and their allies.

## ﻿Materials and methods

### ﻿Trichomes

To analyse the trichomes, temporary preparations of the epidermis of leaves, stems, calyx and corolla were made by making direct peels of the epidermis or cross sections; observations were made under light microscope and drawings were done with the help of a camara lucida.

### ﻿Fruit anatomy

Mature fruits were used to analyse the anatomy of the pericarp. Fresh fruits were collected in the wild, bought at various markets (domesticated species) or obtained from plants cultivated at the University of Cordoba (Argentina) (see Suppl. material 1: Appendix 1). Fruits were fixed in formalin-acetic acid-ethanol (FAA; 3.7% formaldehyde; 5% glacial acetic acid; 50% ethanol) for at least 48 hours and then small pieces of 3–5 × 3–5 mm from the middle part of the fruit were examined. For light microscopy, fixed material was dehydrated through an ascendant ethanol series (50°, 60°, 70°, 80°, 90°, 95°, 100°), clarified in pure xylene and embedded in Paramat^(TM)^. The samples were sectioned at 12 μm thick with a Minot rotary microtome. Sections were double-stained using Astra Blue/Basic Fuchsin (Kraus et. al. 1998; Zarlavsky 2014). Cross-sections were also made by hand. These were stained with safranin that stains intensely lignified cell walls (sclereids), which can then be easily differentiated from the cellulose-walled parenchyma cells. The cuticle was detected with Sudan IV. Outer and inner epidermal peels were obtained from the upper, middle and lower part of the fruit and were stained with safranin. The stained sections and peels were mounted in a drop of 50% glycerine solution. Measurements of the thickness of the cuticle and cuticular wedges, as well as the giant cells’ size, were made using a stage micrometer. Camera lucida drawings of pericarp sections are shown in many of the figures. Only dried fruits from herbarium specimens could be analysed for some species. Here, fruits were hydrated and kept in water at 90 °C for the necessary time (1–2 hours) to soften the tissues to be able to perform sectional cuts or pericarp peels.

### ﻿Seeds

Mature seed samples were taken from herbarium material or collected from wild or cultivated sources (see Suppl. material 2: Appendix 2). All seeds were selected and analysed in three ways for: (1) stereomicroscope (SM), (2) scanning electron microscopy (SEM) and (3) light microscope (LM) observations. The processing of the seeds for each case was as follows: (1) seeds were removed from fresh or hydrated fruits (untreated seeds) and gross morphology observations were made on dried seeds with a Zeiss Stemi 2000-C stereomicroscope (SM) at 32× magnification; (2) for scanning electron microscopy (SEM), seeds were prepared following the enzyme etching technique (Lester and Durrands 1984) to dissolve the outer cell walls, affixed to aluminium stubs with double-sided adhesive tape, then coated with gold and examined using either a JEOL JSM 35 CF SEM (LABMEM, National University of San Luis, Argentina) or a FE-SEM Sigma (LAMARX, National University of Córdoba, Argentina; (3) for sectioning for light microscopy, seeds were soaked in boiling water, washed with a diluted sodium hypochlorite solution (10%) to remove possible fungal hyphae and rinsed in distilled water; cross sections were free-hand cut with glass knives and then stained with Astra Blue and safranin (Johansen 1940). Terminology for seed (and embryo) morphology largely follows Gunn and Gaffney (1974), Zhang et al. (2005) and Chiou and Hastorf (2014).

### ﻿Cytogenetics

Chromosome counts, cytogenetic information and DNA content are based on voucher specimens for which we were able to verify their correct identifications (vouchers cited in Table 2) or based on studies with taxonomic identity, verifiable from the descriptions material (vouchers not cited in Table 2). We do not cite all the extant cytogenetic literature in Table 2 for the domesticated species and some related wild species, since these works are not comparable with the karyotyping nomenclature systems (e.g. Levan et al. 1964; Moscone et al. 1996a) currently used; this means that some works have been excluded (Huskins and La-Cour 1930; Dixit 1931; Yamamoto and Sakai 1932; Tokunaga 1934; Raghavan and Venkatasubban 1940; Chennaveeraiah 1947; Sinha 1950; Vazart 1950, 1951; Eshbaugh 1964; Chennaveeraiah and Habib 1966; Shopova 1966; Datta 1968; Carluccio and Saccardo 1977; Limaye and Patil 1989; Alcorcés de Guerra 2001; Nascimento Sousa et al. 2015).

### ﻿Taxonomy

The monograph is based on results from many years of herbarium study and field work to collect these taxa across South America. Fresh material was preserved in FAA (formaldehyde–acetic acid–ethanol) or ethanol (70°) to perform measurements of reproductive organs using a Zeiss Stemi 2000-C stereomicroscope at 6.5–50× magnification or trichomes using a Leitz light microscope at 10–40× magnification. Descriptions were based on living plants observed during fieldwork and examination of ca. 6,900 herbarium specimens loaned from or inspected at 213 herbaria (acronyms follow Thiers 2021): A, AAU, AC, ALCB, ANDES, AS, ASE, ASSAM, B, BA, BAA, BAB, BAF, BHCB, BHZB, BKL, BM, BOLV, BR, C, CAL, CAS, CDS, CEN, CEPEC, CESJ, CHEP, CM, CNMT, COAH, COL, COLO, CONC, CONN, CORD, CR, CRI, CTES, CUVC, CUZ, DAV, DUKE, E, EAC, EAP, EFC, ENCB, ESA, ESAL, F, FCQ, FLAS, FLOR, FMB, FPS, FSU, FUEL, FURB, G, GA, GB, GBH, GH, GL, GOET, GUA, GUAY, HAL, HAJB, HAMAB, HAO, HAS, HB, HBG, HBR, HCF, HEH, HEPH, HFSL, HJ, HOXA, HRB, HRCB, HSB, HST, HSTM, HUA, HUAZ, HUCP, HUCS, HUEFS, HUEM, HUFU, HUFSJ, HULE, HURB, HURB, HUSA, HUSU, HUT, HVC, IAC, IAN, IEB, IBN, ICN, IND, INPA, IPA, ITIC, JAU, JAUM, JPB, K, KFTA, L, LAGU, LE, LINN, LP, LPB, LIL, LOJA, LUSC, M, MA, MAC, MCNS, MBM, MBML, MEDEL, MEL, MERL, MEXU, MICH, MISS, MNES, MO, MOL, MPU, MU, MY, NA, NDG, NY, OUPR, OXF, P, PACA, PEL, PEUFR, PH, PMSP, PR, PSO, PUL, PY, PYO, Q, QAP, QCA, QCNE, R, RB, REG, RIOC, RSA, RUSU, S, SAMES, SEV-H, SF, SMDB, SI, SJRP, SP, SPF, SPSF, TEFH, TUB, TULV, U, UB, UC, UDBC, UEC, UFG, UFP, UFRN, UNA, UNAH, UNOP, UNR, UOJ, UPCB, UPS, US, USCG, USF, USM, USZ, UT, VEN, VIC, VIES, VT, W, WAG, WU, WIS, XAL, YU and Z. Digital images were also accessed via different repositories, such as Global Plants (https://plants.jstor.org/), INCT Herbário Virtual (http://inct.splink.org.br), Herbário Virtual Reflora (http://reflora.jbrj.gov.br/reflora/herbarioVirtual), Atlas of the Florida Plants (http://florida.plantatlas.usf.edu) or Botanical Collections Databases of the following herbaria: B (http://ww2.bgbm.org/herbarium/default.cfm), COL (http://www.biovirtual.unal.edu.co/es/colecciones/search/plants/), F (http://fieldmuseum.org/explore/department/botany/collections), G (http://www.ville-ge.ch/cjb/bd.php), GH (https://huh.harvard.edu/pages/digital-resources), MA (http://colecciones.rjb.csic.es/), MPU (https://collections.umontpellier.fr/collections/botanique/herbier-mpu/base-herbier-mpu), NY, P (https://science.mnhn.fr/institution/mnhn/collection/p/item/search/form), S (https://herbarium.nrm.se/), US (https://collections.nmnh.si.edu/search/botany/), W (http://herbarium.univie.ac.at/database/search.php) and Z (http://www.herbarien.uzh.ch/index.html).

Measurements of dried material were made from dissections of flowers or fruits rehydrated in hot water, supplemented by measurements from living materials. Information about flower, fruit and seed colour was taken mainly from our own field observations and, in a few cases, flower colour was described from herbarium label data (e.g. *C.hookerianum*). The terminology used in the mature fruit descriptions of the domesticated species is based on the list of descriptors for *Capsicum* (IPGRI et al. 1995); pungency of immature and mature fruits was tested by tasting them in the field.

Distribution maps were produced using QGIS 3.16.0-Hannover (QGIS Development Team 2019) and were based on georeferenced data of all the herbarium collections analysed. Maps with boundaries of countries were imported from Natural Earth (www.naturalearthdata.com); elevation maps were obtained from the DIVA-GIS (http://diva-gis.org/gdata) spatial data. Specimens with latitude and longitude data on the labels were mapped directly; collections without geographical coordinates were georeferenced using different georeferencing tools, such as the point-radius method (determination of latitude and longitude and the maximum error distance), search for localities in Google Maps, Google Earth and GraphHopper Maps or in available databases with localities already georeferenced.

Preliminary conservation status was assessed using IUCN (2022) criteria B (for the relatively widespread species) and C (for some species with very restricted occurrences). We have given more weight to the extent of occurrence (EOO) in the threat assessments than to the area of occupancy (AOO), since the EOO is very sensitive to georeferencing bias and collecting effort (Knapp et al. 2020). The extent of occurrence and area of occupancy were calculated using the Geospatial Conservation Assessment Tool GeoCAT (Bachman et al. 2011; GeoCAT 2020).

Typification of cultivated taxa has been a particularly difficult task. For many taxa, the authors did not cite specimens or locality of the type. We searched for original material in potential herbaria and when we succeeded or duplicates were found, we designated lectotypes. In other taxa, lectotypifications were based on an illustration cited by the author in the protologue (e.g. Fingerhuth’s illustrations). For taxa recognised only as synonyms, we have cited the taxa in synonymy and indicated that duplicates have not been found rather than neotypifying these taxa. In cases where taxa were described from collections of living material cultivated in botanical gardens from unknown origin or the original material was destroyed in World War II, we designated neotypes when probable original material could be found (e.g. in *C.ovatum*) or using a modern collection (*C.flexuosum*). However, for the species described by Philip Miller (1768) in his“ Gardener’s Dictionary”, we have postponed typifications. Specimens made from plants grown by Miller at Chelsea Physic Garden in London (UK) are found in several different herbaria, mostly at BM and its associated historical herbaria; since we were unable to visit these herbaria for searching Miller’s original material due to restrictions for the current pandemic caused by Covid-19, we do not typify them here, leaving that for a separate study when these materials, including any non-digitised specimens, can be studied in detail.

In cases where specific herbaria have not been cited in protologues, we designate lectotypes rather than assuming holotypes exist (McNeill 2014). For cases in which the collection type was based on syntypes, we have examined as many as possible, choosing the best-preserved specimen as the lectotype; in all cases, we give the reasons for our choice in the species discussions.

Type specimens are cited with their barcodes in square brackets after each herbarium acronym, according to the style used in each herbarium (e.g. GOET003420 or MO-562486); we also indicate the sheet number after the barcode when available (i.e. IND-0153285, acc. # 139721). When barcodes are missing, we indicate only the sheet number (i.e. LIL acc. # 173409). In a few cases, we do not cite barcode or accession number (e.g. some type material from LE).

Identities of all numbered collections seen are presented in the List of Exsiccatae. Numbered and un-numbered collections are presented in Suppl. material 4: Appendix 4 where full citations of all the specimens examined for this treatment are presented in PDF format. We have not cited specimens of any *Capsicum* collected outside the Americas, with the exception of types.

Common names were taken from herbarium label data and reliable literature if we could verify the identity of taxa, but the list of common names for the cultivars of *C.annuum*, *C.frutescens* and *C.chinense* is not complete since we did not comprehensively examine the vast amount of literature where this information appears. Indigenous names are given in a separate paragraph for clarity indicating in brackets the indigenous language (if given). We cite only one specimen by provenance of the common and indigenous names per administrative division of each country. Uses as foods, spices or in ritual practices are cited in the species treatment and folk medicinal uses are summarised in Table 3, where the organ used and the medicinal properties, as well as the source of information, are indicated (verified specimens or literature).

**Table 3.** Medicinal uses attributed to *Capsicum* species.

Taxa	Organ	Use	Country	*Voucher*/Reference
*C.annuum var. annuum*			Colombia	
	Leaf, fruit	Medicinal (no specification)	Amazonas	*Alvarado C. 198*
			Ecuador	
	Fruit	For snake bite	Morona-Santiago	*Evans 4384*
			Peru	
	Leaf (juice)	For pregnant women to help birth easily	Loreto	*Williams et al. 10922*
C.annuumvar.glabriusculum			Brazil	
	Leaf	To cure acne	Amapá	Pereira et al. 2011
			Colombia	
	Fruit	To increase body temperature and for the skin fungi	Huila	*Buendía 2*
	Fruit	To soothe haemorrhoid pains	Valle del Cauca	*Duque Jaramillo 4083-A*
			Ecuador	
	Leaf	To reduce body temperature	Orellana	*Carrillo & Reyes 434*
	Fruit	Stomach medicine (for sore belly)	Napo	*Davis & Yost 994*
	Fruit	To kill parasites	Morona-Santiago	*Bedoya 2*
	Fruit	For skin diseases (measles, pox)	Zamora-Chinchipe	*Santín et al. 100*
	Fruit	For conjunctivitis	Guatemala	*Kufer 99*
			Mexico	
	Leaf	To relieve rashes in children (warm bath)	Quintana Roo	*Serralta P. 104*
	Fruit	For infected wounds	Querétaro	*Martínez Torres 82*
	Fruit	To treat ulcers	Tabasco	*Orozco-Segovia 368*
	Fruit	For skin wounds	Yucatán	*Ucan 4617*
	Fruit	Medicinal	Yucatán	*Simá 517*
			U.S.A.	
	Fruit	Stimulant	Texas	*Chávez Jr. s.n.*
*C.chacoense*	Fruit	Anti-rheumatic	Argentina	Martínez Crovetto (1981); Del Vitto et al. (1997)
	Fruit	Digestive		Scarpa (2002)
	Fruit	Anti-spasmodic, vermifuge, stomach pain		Filipov (1994)
	Fruit extract	Anti-inflammatory activity (mice)		López et al. (2012)
	Fruit	Anti-parasitic	Paraguay	Friesen Ratzlaff (2017)
*C.chinense*			Colombia	
	Seedling	For haemorrhoids	Meta	*Quevedo et al. 1816*
			Ecuador	
	Leaf	To treat joint pains	Sucumbíos	*Reyes & Moya 234*
	-	Anti-parasitic	Napo	*Bolotin 21*
	Fruit	For stomach ache	Napo	*Davis & Yost 993*
	Fruit	For eye infections and coughing	Napo	*Miller et al. 2404*
	Fruit	For dysentery	Orellana	*Herrera & Guerrero 186*
	Fruit	Medicinal: cardiotonic	Sucumbíos	*Moya & Reyes 20*6
			Mexico	
	Leaf	To treat wounds	Yucatán	*Ucan Ek 4652*
*C.coccineum*			Bolivia	
	Entire plant	In baths to relieve stomach pain	La Paz	*Vargas 1310*
*C.frutescens*			Brazil	
	Leaf (infusion)	Used for dizziness	Minas Gerais	*Pereira 3219*
	Immature fruits	For flu	Minas Gerais	*Pereira 3219*
*C.frutescens*			Colombia	
	Root (infusion)	To facilitate childbirth	Guaviare	*Garzón et al. 3214*
	Buds	To cure hand infection	Guaviare	*Garzón et al. 3214*
	Fruit	Medicinal	Cundinamarca	*García Barriga 20315A*
			Ecuador	
	Leaf	For fungal diseases	Esmerlada	*Kvist 40356*
	Leaf, fruit	To facilitate the fall of the baby’s umbilical cord	Napo	*Siquihua 4*
	Fruit	For snake bites	Morona-Santiago	*van Asdall 82-59*
	Fruit	For snake bites	Zamora-Chinchipe	*Ortega 51*
			Guatemala	
	Fruit	To treat conjunctivitis	Chiquimula	*Kufer 100*
*C.pubescens*			Ecuador	
	Leaf	For bites of dogs	Loja	*Ellemann 66689*
	Fruit	For headache, weakness and cold	Loja	*Ellemann 66689*
	Fruit?	Veterinary: to treat “moquillo” (catarrhal disease in dogs and cats) and “tos de nermo” (cough in horses)	Peru	
			Oxapampa	*Chuck 137*
*C.rhomboideum*			Ecuador	
	-	To heal skin eruptions	Pichincha	*Cerón 6953*

All species are illustrated with line drawings, colour illustrations or both; photos were taken by the authors of this treatment or were provided by other colleagues (credits are cited in each case). For some species (*C.caatingae*, *C.friburgense*, *C.hunzikerianum*, *C.mirum*), photos were provided and taken in the field by members of the Associazione PepperFriends (Verona, Italy); these photos have no herbarium voucher, but the identification was verified by the senior author of this treatment (GEB).

### ﻿Biomes and ecoregions

Ecoregions were determined according to Olson et al. (2001). To determine the ecoregions and biomes occupied by the different species, maps were generated using QGIS 3.16.0-Hannover (QGIS Development Team 2019) superimposing layers of georeferenced data (as for the distribution maps, see above) with a layer of ecoregions obtained from Ecoregions2017^©Resolve^ (https://ecoregions.appspot.com/).

## ﻿Taxonomic treatment

### ﻿ Capsicum

Taxon classificationPlantae

L., Sp. pl. 1: 188. 1753.

F5AB4123-9EB5-5B43-BB98-48F9EFFB7F44

Capsicum section Decameris Bitter, Abh. Naturwiss. Vereins Bremen 24(2): 293. 1919. Type: C.dusenii Bitter
Capsicum section Capsicum , Huitième Congr. Int. Bot. Paris. Comptes Rend. Séances Rapp. & Commun. 1954, sect.4: 73. 1956. Type: C.annuum L.

#### Type.

*C.annuum* L. (lectotype, designated by Britton 1918, pg. 338).

#### Description.

Shrubs, subshrubs, rarely trees, vines or short-lived perennials or annuals, occasionally with a thick lignified xylopodium, glabrous or glabrescent or sparsely to densely pubescent with simple, branched, eglandular or glandular, uniseriate trichomes. Stems woody at the base, sometimes with fissured bark and lenticels; young stems angled, herbaceous, usually weak and fragile and occasionally somewhat scrambling. Sympodial units difoliate or unifoliate, the leaves usually geminate, blades simple, entire, concolorous or discolorous, glabrous to densely pubescent with eglandular and/or glandular simple or branched uniseriate trichomes; petioles generally well-developed. Inflorescences axillary, usually unbranched (rarely branched), with few to many (up to 20 or more) flowers clustered or, more rarely, on short rachis or spaced along an elongate rachis, sometimes with flowers solitary or paired. Flowers 5-merous (4–8-merous in domesticated species), actinomorphic, all perfect. Pedicels erect, slightly spreading or pendent, geniculate at their distal end or non-geniculate. Calyx truncate, entire, circular or five-angled in outline, often with 3–10 appendages. Corolla stellate, rotate-stellate, campanulate or campanulate-urceolate, entirely white, yellow, violet or fuchsia or with greenish-yellow and/or maroons or purple spots within, rarely entirely greenish-white or mostly purple, the lobes spreading or reflexed at anthesis, usually with interpetalar membrane. Stamens five (up to eight in domesticated species), usually equal (rarely unequal), the filaments glabrous and broadened at the base to form a staminal plaque fused to the corolla base, each plaque with two short lateral auricles, the anthers dorsifixed, ellipsoid or ovoid, yellow, cream or blue to purple, connivent in pre-anthesis, usually free when mature, dehiscent by longitudinal slits. Gynoecium usually bicarpellate, rarely 3–4-carpellate; ovary superior, glabrous, subglobose to ovoid (rarely ellipsoid), with an annular nectary at the base; styles straight or slightly curved, cylindrical or clavate, glabrous, commonly exserted beyond the anthers, sometimes heteromorphic (long, medium and short styles); stigma globose or discoid, sometimes somewhat bilobed, finely papillate. Fruit glabrous berry, globose, subglobose or somewhat elongate, the mesocarp juicy, the pericarp red, orange-red, greenish-golden yellow or, rarely, dark burgundy or purple-blue at maturity (in domesticated species, fruits of various shapes and colours), pungent or not; fruiting pedicels erect or deflexed; fruiting calyx discoid or campanulate, not accrescent or slightly accrescent. Seeds flattened to slightly angled, mostly C- or D-shaped, subglobose or ellipsoid (rarely reniform or teardrop-shaped), pale yellow to yellow, brownish-yellow to brown or brownish-black to black, seed coat smooth, reticulate or reticulate marginally tuberculate. Stone cells absent or present, if present, not more than six. Embryo usually imbricate (less frequently annular or coiled); endosperm firm, whitish and relatively abundant. Chromosome number: 2n = 24, 26 (see Table 2).

#### Distribution

**(Fig. 19).** Species of *Capsicum* are native to temperate, subtropical and tropical regions in the Americas, growing from southern United States of America to central Argentina and Brazil. Five taxa are widely cultivated elsewhere.

### ﻿Artificial key to *Capsicum*

**Table:** 

1	Stem and mature leaf blades completely glabrous, if trichomes present, sparsely distributed on the veins and margins	**2**
–	Stem and mature leaf blades variously pubescent	**10**
2	Calyx appendages (3–) 5, strongly incurved; flowering pedicels slightly winged and conspicuously winged in fruit; leaves coriaceous; Bolivia	***C.ceratocalyx***
–	Calyx appendages absent or up to 10, straight; flowering and fruiting pedicels not winged; leaves coriaceous or membranous	**3**
3	Corolla tubular-campanulate to broadly campanulate, lobed less than 1/3 of the way to the base	**4**
–	Corolla usually stellate or stellate-campanulate, lobed 1/3 up to nearly halfway to the base	**6**
4	Calyx appendages 10, unequal (5 long, 5 short); corolla lobes recurved; fruits pungent; seeds 3–4 (–5) mm long, pale yellow to nearly white; Bolivia	***C.caballeroi***
–	Calyx appendages 2–5, equal or subequal; corolla lobes erect; fruits non-pungent; seeds 1.5–2.5 mm long, brown to black	**5**
5	Leaves coriaceous; calyx appendages (2–) 3–5, spreading or reflexed; filaments 1–2.5 mm long; corolla broadly campanulate; Colombia and Ecuador	***C.lycianthoides***
–	Leaves membranous; calyx appendages 5, erect; filaments 3–5 mm long; corolla tubular-campanulate; Peru	***C.piuranum***
6	Inflorescences with 5–13 flowers on an elongate rachis, sometimes rachis forked; fruiting calyx with a conspicuous annular constriction at the junction with the pedicel; fruiting pedicels erect, brilliant dark purple; fruits dark blue to purple; Colombia, Ecuador and Peru	***C.regale***
–	Inflorescences with 3–7 (–9) axillary flowers, rarely on a very short unbranched rachis or flowers solitary; fruiting calyx without an annular constriction at the junction with the pedicel; fruiting pedicels pendent or rarely curved, green or greenish-purple; fruits of other colours	**7**
7	Calyx appendages absent or 5, minute (< 0.5 mm long); corolla tube and base of the lobes with a sparse but continuous ring of glandular trichomes adaxially; fruits greenish-golden yellow; Brazil	**8**
–	Calyx appendages 2–10, longer (1–5 mm long); corolla tube and base of the lobes glabrous adaxially; fruits orange or greenish-golden yellow	**9**
8	Leaves membranous, elliptic to ovate; flowering pedicels 9–14 mm long, erect, geniculate at anthesis; corolla small, 4.5–6.5 (–8) mm long; stamens unequal (3+2); ovules 2 per locule; fruits 4-seeded; Brazil (Rio de Janeiro, Minas Gerais, Espírito Santo)	***C.campylopodium***
–	Leaves coriaceous, elliptic to narrowly elliptic, flowering pedicels 15–30 mm long, pendent, non-geniculate at anthesis; corolla larger, 9–10 mm long; stamens equal; ovules more than 2 per locule; fruits multi-seeded (up to 20 seeds); Brazil (Bahía, Espírito Santo, Minas Gerais, São Paulo)	***C.pereirae***
9	Major leaves narrowly elliptic; calyx appendages 2–3, triangular-compressed wings; flowering pedicels 3–8 mm long, pendent, non-geniculate; corolla 6–8.5 mm long, 8–11 mm in diameter, entirely yellow or with red-brown pigmentation within; fruits orange, non-pungent; seeds 1.7–2.3 mm long; Peru and Ecuador	***C.longifolium***
–	Major leaves ovate to elliptic; calyx appendages 5 (6–10), cylindrical; flowering pedicels (13–) 20–38 (–48) mm long, erect to spreading, geniculate; corolla 10–14 (–16) mm long, (10–) 15–23 mm in diameter, white with diffuse brown-purple spots and a greenish-yellow centre within; fruits greenish-golden yellow, pungent; seeds 2.5–3.2 mm long; Brazil	***C.hunzikerianum***
10	Pubescence mostly of branched eglandular or glandular trichomes, few simple trichomes	**11**
–	Pubescence mostly of simple eglandular trichomes, rarely furcate eglandular trichomes or simple glandular trichomes	**13**
11	Dense pubescence of long furcate and simple glandular trichomes; calyx appendages usually 10 (rarely 5 or up to 12); filaments 2.5–3 mm long; flowering and fruiting pedicels erect; fruits pungent; Bolivia	***C.eshbaughii***
–	Dense pubescence of furcate to dendritic eglandular trichomes mixed with few simple eglandular trichomes; calyx appendages usually 5 (rarely 3–4 or 6); filaments 1.2–2.3 mm long; flowering and fruiting pedicels pendent; fruits non-pungent	**12**
12	Calyx appendages (4.5–) 5–8.5 mm long; corolla stellate, lobed almost halfway to the base, white with dark greenish-yellow spots within, with small glandular trichomes adaxially; style exserted ca. 1 mm beyond the anthers; seeds < 20 per fruit, 3–3.7 mm long, 2.5–2.8 mm; inflorescences usually (1–) 2–5-flowered; Brazil	***C.longidentatum***
–	Calyx appendages 0.9–3 mm long; corolla campanulate or campanulate-rotate, shallowly lobed, entirely yellow or sometimes tinged greenish within, glabrous adaxially; style barely exserted beyond the anthers; seeds > 20 per fruit, 2.4–2.8 mm long, 1.8–2.2 mm wide; inflorescences usually (1–) 3–8 (–13)-flowered; Mexico to Peru	***C.rhomboideum***
13	Corolla nearly lobed to the base, the tube 4–4.5 times shorter than the lobes; Ecuador	***C.benoistii***
–	Corolla shallowly lobed or lobed halfway or to 2/3 of the way to the base, the tube as long as the lobes or 1.5 times shorter than the lobes	**14**
14	Staminal plaques with conspicuous auricles not fused to the corolla at the point of insertion of the filaments; Bolivia, Argentina and Paraguay	***C.chacoense***
–	Staminal plaques with inconspicuous auricles fused to the corolla at the point of insertion of the filaments	**15**
15	Major leaves narrowly elliptic to lanceolate, the length/width ratio 5–10 (–16)	**16**
–	Major leaves ovate or elliptic, if elliptic the length/width ratio (2–) 2.5–4 (–4.5)	**17**
16	Calyx appendages 5, erect, green; corolla stellate, white with large purple spots and a cream centre within, with glandular trichomes in the throat and lobes adaxially; flowering pedicels erect to spreading, geniculate at anthesis; anthers blue; berry 6–7 mm in diameter, greenish-golden yellow, pungent; seeds 3.5–4 mm long, 2.5–3 mm wide; Brazil	***C.carassense***
–	Calyx appendages (2–) 3–5, spreading or erect, green, greenish-purple or purple; corolla campanulate, entirely yellow or yellow with smaller maroon or purple spots within, glabrous adaxially; flowering pedicels pendent, non-geniculate at anthesis; anthers cream, yellow or rarely white; berry 7–12 mm in diameter, pale orange or orange, non-pungent; seeds 1.8–2.3 mm long, 1.3–1.5 mm wide; Colombia, Ecuador and Peru	***C.geminifolium***
17	Flowering pedicels pendent, non-geniculate at anthesis	**18**
–	Flowering pedicels erect to slightly spreading, geniculate at anthesis	**29**
18	Flowers 5–7-merous; calyx thick, strongly 5–10-nerved; style heteromorphic (included at the same level as the stamens or exserted); corolla usually entirely white, dull white or greenish-white, rarely entirely purple or pale yellow; seeds pale yellow or nearly white, the seed coat smooth; fruits of various shape, size and colour; widely cultivated in the Americas	**19**
–	Flowers 5-merous (rarely 4-merous); calyx usually thin, comparatively weakly 5–10-nerved; style usually homomorphic, rarely dimorphic (included and exserted); corolla entirely yellow, yellow with maroon or purple spots within, white with greenish-yellow spots within or primarily purple or lilac; seeds usually brown or brownish-black to black, rarely yellow or pale yellow, the seed coat uniformly reticulate or reticulate and tuberculate at margins; fruits usually globose or subglobose, not more than 16 mm in diameter, orange to red or greenish-golden yellow; wild species, mostly from South America	**20**
19	Flowers solitary, rarely in pairs or more; petioles up to 10 cm long; corolla 8–15 mm long, entirely white, rarely entirely purple or pale yellow; fruiting calyx without a prominent annular constriction at junction with the pedicel	**C.annuumvar.annuum**
–	Flowers 2–4 (–5); petioles up to 3.5 cm long; corolla (5–) 6.5–8 mm long, entirely dull white or greenish-white (occasionally with purple spots); fruiting calyx with a prominent annular constriction at junction with the pedicel	***C.chinense***
20	Corolla tubular-campanulate, 14.5–17 mm long; calyx appendages 5; stone cells 2; northern Peru	***C.piuranum***
–	Corolla campanulate, campanulate-stellate, rotate-stellate or stellate, 4.5–12 (–15) mm long; calyx appendages absent or up to 10; stone cells absent or up to 6	**21**
21	Calyx appendages absent or up to 5, equal and minute, < 1 mm long	**22**
–	Calyx appendages 2–10, subequal or unequal, > 1 mm long	**25**
22	Young stems, leaves and calyx with simple eglandular trichomes mixed with small dark glandular trichomes	**23**
–	Young stems, leaves and calyx only with simple eglandular trichomes, glandular trichomes absent	**24**
23	Corolla campanulate to campanulate-stellate, lobed less than ⅓ of the way to the base; style dimorphic, short style 1–1.6 mm, long style 4.1–4.2 mm long; flowering pedicels 3–10 mm long; inflorescences few-flowered (2–8 flowers); fruiting pedicels erect, green; central Peru	***C.tovarii***
–	Corolla stellate, lobed nearly halfway to the base; style homomorphic, 4.3–4.8 mm long; flowering pedicels longer, 7–21 (–28) mm long; inflorescences multi-flowered (5–13 flowers or up to 20 or more); fruiting pedicels pendent, green or purple; north-eastern Brazil	***C.caatingae***
24	Leaf pair strongly dissimilar in shape and size; flower buds ovoid, purple or yellowish; fruits non-pungent; seeds 1.9–2.7 mm long, 1.8–2.1 mm wide; corolla entirely yellow or with purple or maroon spots within; Colombia, Ecuador and Peru	***C.dimorphum***
–	Leaf pair similar or dissimilar in size, similar in shape; flower buds globose, white with green spots; fruits pungent; seeds 2.8–3.4 mm long, 2.2–3 mm wide; corolla white with greenish-yellow spots, rarely also with purple spots; Argentina, Paraguay and Brazil	***C.flexuosum***
25	Calyx appendages 8–10, unequal	**26**
–	Calyx appendages 2–5 (–7), subequal	**27**
26	Leaves coriaceous; inflorescences (1–) 2-flowered; flowering pedicels 20–40 (–50) mm long; corolla ≥ 10 mm long, 4–6 mm in diameter; filaments 4–6 mm long; style 7–9 mm long; fruits pungent; fruiting calyx appendages appressed to the berry; Bolivia	***C.caballeroi***
–	Leaves membranous; inflorescences with (1–) 2–7 flowers; flowering pedicels shorter, 8–15 mm long; corolla 7.5–9 mm long, 8–10 mm in diameter, filaments (1.5–) 1.8–2 mm long; style ca. 4 mm long; fruits non-pungent; fruiting calyx appendages spreading or reflexed; Ecuador and Peru	***C.hookerianum***
27	Leaf pair dissimilar in size, similar in shape; major leaves ovate; corolla stellate, lobed nearly halfway to the base; fruits greenish-golden yellow, pungent; seeds 3–3.8 mm long, 2.7–3 mm wide; Colombia, Venezuela and Brazil	***C.parvifolium***
–	Leaf pair markedly dissimilar in size and shape, rarely similar in shape; major leaves elliptic to lanceolate; corolla campanulate, lobed ⅓ of the way to the base; fruits orange to orange-red, non-pungent; seeds 1.8–2.8 mm long, 0.8–1.8 mm wide	**28**
28	Mature leaves glabrous adaxially; flowers solitary, rarely 2 per node; calyx appendages (4–) 5, spreading or strongly reflexed; corolla purple with white interpetalar membrane; North America (Mexico) and Central America (Guatemala and Honduras)	***C.lanceolatum***
–	Mature leaves sparse to densely pubescent adaxially; flowers 2–5 (–6) per node; calyx appendages 2–3 (–5), erect or spreading; corolla entirely yellow or with purple or maroon pigmentation within and yellow interpetalar membrane; South America: Colombia, Ecuador and Peru	***C.geminifolium***
29	Corolla broadly campanulate, campanulate-urceolate, rotate or rotate-stellate, lobed 1/3 or less of the way to the base	**30**
–	Corolla stellate, lobed more than 1/3 up to 2/3 of the way to the base	**36**
30	Stems and mature leaves with minute dark simple glandular trichomes mixed with sparse eglandular trichomes; corolla broadly campanulate or campanulate-urceolate	**31**
–	Stems and mature leaves lacking minute dark glandular trichomes mixed with abundant or sparse eglandular trichomes; corolla rotate or rotate-stellate	**32**
31	Major leaves (5.5–) 8.5–13 (–21) cm long; corolla campanulate-urceolate, entirely fuchsia or violet, glabrous adaxially, the lobes spreading to strongly recurved; fruits greenish-golden yellow, slightly pungent; seeds brownish-black to black; Brazil	***C.friburgense***
–	Major leaves 3–5 (–6) cm long; corolla campanulate, almost completely violet or lilac and a greenish-yellow to white centre within, with short glandular trichomes adaxially, the lobes erect or spreading; fruits red, pungent; seeds pale yellow to brownish-yellow; Bolivia	***C.cardenasii***
32	Flowers 4–8-merous; corolla 8.5–15 mm long; fruiting pedicels pendent; fruits large, > 10 mm in diameter, persistent, variously coloured; cultivated in the Americas	**33**
–	Flowers 5-merous (rarely 4-merous); corolla 4–7 mm long; fruiting pedicels erect; fruits small, < 10 mm in diameter, deciduous, orange or red; wild or semi-domesticated in South America	**35**
33	Leaves densely pubescent, rarely glabrescent; flower buds dark purple on pendent pedicels; style clavate; seeds 5.5–7 mm long, 4.8–6 mm wide, brownish-black to black, the seed coat reticulate; corolla dark purple or violet with a white or yellowish-green centre within	***C.pubescens***
–	Leaves glabrous to sparsely pubescent; flower buds greenish-white or purple on geniculate pedicels; style cylindrical; seeds 3–5.2 mm long, 3–4 mm wide, pale yellow to yellow, the seed coat smooth to slightly reticulate; corolla white with large greenish-yellow spots and white centre within	**34**
34	Fruits pungent, rarely non-pungent, usually elongate, endocarp alveolate, pericarp with giant cells	**C.baccatumvar.pendulum**
–	Fruits non-pungent, campanulate-umbilicate, endocarp smooth, pericarp without giant cells	**C.baccatumvar.umbilicatum**
35	Leaves with dense pubescence, especially abaxially; corolla marginally purple with greenish-yellow centre; Brazil and Paraguay	***C.rabenii***
–	Leaves mostly glabrescent, more rarely moderately pubescent; corolla white with greenish-yellow spots within, purple pigmentation absent; widely distributed across South America	**C.baccatumvar.baccatum**
36	Flowers 4–8-merous; style usually heteromorphic (three different lengths), when homomorphic carpels 2; fruits of various size, shape and colours; mostly cultivated or semi-domesticated plants across the Americas	**37**
–	Flowers always 5-merous; style homomorphic; fruits small, not more than 15 mm in diameter, globose or subglobose, most rarely ellipsoid or ovoid, usually red or red-orange or greenish-golden yellow; wild plants from South America	**41**
37	Calyx appendages absent or if present, minute, ≤ 0.5 mm long	**38**
–	Calyx appendages > 0.5 mm long	**40**
38	Flowers solitary, rarely in pairs or more; petioles up to 10 cm long; corolla 8–15 mm long, entirely white, rarely entirely purple or pale yellow; fruits usually large, up to 300 mm long, pungent or non-pungent	**C.annuumvar.annuum**
–	Flowers (1–) 2–5; petioles up to 3.5 cm long; corolla 3.75–8 mm long, entirely dull white or greenish-white (occasionally with purple spots); fruits small to medium-sized, up to 100 mm long, pungent, rarely non-pungent	**39**
39	Corolla glabrous adaxially; style heteromorphic; fruits highly variable in shape (domesticated), with the base obtuse or truncate (fruits subglobose in wild populations); fruiting calyx discoid or shallowly cup-shaped, with a prominent annular constriction at junction with the pedicel; ovary subglobose, 2–2.5 mm long, 2.5–3.5 mm in diameter	***C.chinense***
–	Corolla with small glandular trichomes adaxially; style homomorphic; fruits usually elongate and narrowly triangular, with the base abruptly narrowed; fruiting calyx deeply cup-shaped lacking a constriction at junction with the pedicel; ovary oblong-ovoid, 2.5–4 mm long, 1.3–1.8 mm in diameter	***C.frutescens***
40	Young leaves rugose; flowers 4–8-merous; corolla 10–15 mm long, 15–22 (–25) mm in diameter; style heteromorphic; fruiting pedicels pendent; fruits > 10 mm in diameter, persistent; seeds 5.5–7 mm long, 4.8–6 mm wide, brownish-black to black, the seed coat reticulate; cultivated from Mexico to Bolivia	***C.pubescens***
–	Young leaves plane; flowers 5-merous, rarely 4-merous; corolla 6–7 mm long, (9–) 12–15 mm in diameter; style homomorphic; fruiting pedicels erect; fruits < 10 mm in diameter, deciduous; seeds 3–4.2 mm long, 2.5–2.8 mm wide, yellow, the seed coat smooth. Wild or semi-domesticated; Brazil and Paraguay	***C.rabenii***
41	Androecium heterodynamous (three long and two short filaments); ovules two per locule; fruits 4-seeded; corolla white or cream with golden yellow or ochraceous spots within; Brazil	***C.campylopodium***
–	Androecium homodynamous (filaments equal or slightly subequal); ovules more than two per locule; fruits many-seeded; corolla variously coloured	**42**
42	Calyx appendages absent or five, minute, < 0.5 mm long	**43**
–	Calyx appendages 2–10, the main appendages ≥ 0.5 mm long	**47**
43	Filaments < 2 mm long; fruiting pedicels erect; seeds pale yellow, yellow or brownish-yellow; fruits yellow, red-orange or red	**44**
–	Filaments ≥ 2 mm long; fruiting pedicels pendent; seed brownish-black to black; fruits greenish-golden yellow	**46**
44	Flowers 4–13 (–18) on a short rachis; style clavate; seeds 4–4.6 mm long, (–2.8) 3.2–3.75 mm wide, yellow to brownish-yellow; sprawling vines or scrambling shrubs, with stems to 7 m long; Peru, Bolivia and Brazil	***C.coccineum***
–	Flowers 1–2 per axil, rarely up to 3; style cylindrical; seeds 3–4 mm long, 2.5–3.2 mm wide, pale yellow to yellow; perennial herbs or low shrubs or subshrubs up to 2 m, rarely larger	**45**
45	Calyx circular in outline; corolla 4–5 mm long, ca. 6 mm in diameter, with glandular trichomes adaxially; style 2.25–2.5 mm long, exserted 0.5–0.8 mm beyond the anthers; seed coat smooth; plants densely pubescent, the trichomes spreading; Ecuador: Galapagos Islands	***C.galapagoense***
–	Calyx pentagonal in outline; corolla (5–) 6–8 mm long, 8–10 (–12) mm in diameter, glabrous adaxially; style 4–4.8 mm long, exserted 1.5–2 mm beyond the anthers; seed coat reticulate to obscurely reticulate; plants glabrescent to densely pubescent, the trichomes appressed-antrorse, sometimes spreading; widespread in the Americas	**C.annuumvar.glabriusculum**
46	Stems, leaves, pedicels and calyx densely pubescent with long spreading eglandular trichomes 0.5–2 mm long; major leaves elliptic to narrowly elliptic; corolla white with large greenish-yellow spots and sparse diffuse purple or brown spots within, the lobes widely triangular; Brazil (Rio de Janeiro)	***C.muticum***
–	Stems, leaves, pedicels and calyx glabrescent to moderately pubescent, with short antrorse eglandular trichomes 0.25–0.5 mm long; major leaves elliptic to ovate; corolla white with large or small purple or brownish spots and large greenish-yellow spots within (in some populations purple or brownish pigmentation completely absent); the lobes triangular or ovate; Brazil (Minas Gerais, Rio de Janeiro and São Paulo)	***C.schottianum***
47	Leaves coriaceous; calyx appendages strongly incurved, flattened laterally; Bolivia	***C.ceratocalyx***
–	Leaves membranous; calyx appendages straight or recurved, filiform or cylindrical	**48**
48	Fruiting pedicels usually erect, rarely pendent; fruits red; seeds nearly white or yellow to brown	**49**
–	Fruiting pedicels pendent; fruits greenish-golden yellow; seeds brownish-black to black	**52**
49	Calyx strongly 10-nerved with prominent venation; seeds pale yellow to nearly white, the seed coat smooth and reticulate at margins; Bolivia	***C.neei***
–	Calyx slightly 5-nerved with inconspicuous venation; seeds yellow to brown, the seed coat faintly reticulate and slightly tuberculate at margins	**50**
50	Inflorescences many-flowered (4–18 flowers); pedicel scars prominent, corky; filaments < 2 mm long; sprawling vines or scrambling shrubs; Peru, Bolivia and Brazil	***C.coccineum***
–	Inflorescences few-flowered (up to 5 flowers); pedicels scars inconspicuous; filaments ≥ 2 mm long; erect shrubs or subshrubs	**51**
51	Calyx appendages 5, 1–1.5 mm long; filaments 2–2.5 mm long; corolla yellow with small and faint greenish-yellow spots within; seeds 4–4.5 mm long, 3–3.5 mm wide, dark brown; Bolivia	***C.minutiflorum***
–	Calyx appendages (4–) 5, 1.2–2.7 (–3) mm long; filaments 2.7–3.8 mm long; corolla lilac, purple or magenta with a continuous greenish-yellow or ochre tube within, sometimes the corolla white with greenish-yellow spots; seeds 2.5–4 (–4.2) mm long, 2.1–3 mm wide, brownish-yellow; Bolivia and Argentina	***C.eximium***
52	Calyx appendages five	**53**
–	Calyx appendages 10 or 6–10, rarely five	**54**
53	Plants densely pubescent, the stem trichomes spreading; Brazil: Rio de Janeiro, Minas Gerais and São Paulo	***C.villosum***
–	Plants glabrescent to moderately pubescent, the stem trichomes antrorse; Brazil: Bahia, Espírito Santo, Minas Gerais, Rio de Janeiro and São Paulo	***C.mirabile***
54	Calyx appendages 10, subequal; filaments 3–3.2 mm long; style barely exserted beyond the anthers; anthers lilac or pale blue; corolla almost entirely purple; Brazil (São Paulo)	***C.mirum***
–	Calyx appendages ranging from 5 to 10, unequal; filaments 1.4–2.5 mm long; style exserted 0.8–1.3 mm beyond the anthers; anthers yellow, light green or grey; corolla white with greenish-yellow or purple spots	**55**
55	Flowering calyx appendages cylindrical or triangular-compressed, glabrous to moderately pubescent with antrorse trichomes, the longest appendages 1–2.5 mm; corolla 6–7 mm long, ca. 11 mm in diameter, white with greenish-yellow spots within; style 3.2–3.5 mm long; fruiting calyx appendages strongly recurved; fruiting pedicels 18–25 mm long; Brazil (Minas Gerais, Paraná, Rio de Janeiro, Santa Catarina, and São Paulo)	***C.recurvatum***
–	Flowering calyx appendages linear or subulate, densely pubescent with spreading trichomes, the longest appendages 2.5–5 (–6) mm long; corolla (8–) 9–14 mm long, 18–22 mm in diameter, white with purple or reddish-brown spots within; style 4–6.8 mm; fruiting calyx appendages spreading; fruiting pedicels (25–) 30–38 mm long; Brazil (São Paulo and Rio de Janeiro)	***C.cornutum***

### ﻿Key to the wild *Capsicum* species from North America, Central America and the Caribbean

**Table:** 

1	Leaf pair subequal in size and shape; calyx appendages absent or five, minute, < 0.5 mm long; corolla (5–) 6–8 mm long, stellate, lobed nearly halfway or up to 2/3 of the way to the base, entirely white or almost pale yellow, rarely greenish-white; style cylindrical; flowering and fruiting pedicels erect; fruits pungent; seeds pale yellow to yellow; perennial herbs or prostrate subshurb; southern United States of America, Mexico, Central America and the Caribbean islands	**C.annuumvar.glabriusculum**
–	Leaf pairs markedly dissimilar in size and shape; calyx appendages (3–4) 5, > 0.5 mm long; corolla 5–14 mm long; campanulate or campanulate-rotate, lobed not more than 1/3 of the way to the base, yellow or purple marginally white; style clavate; flowering and fruiting pedicels pendent; fruits non-pungent; seeds brown or brownish-black to black; erect shrubs or shrubs, rarely trees	**2**
2	Plants glabrescent to densely pubescent with simple, furcate or dendritic trichomes; major leaves ovate, elliptic or rhomboid-ovate; inflorescences of 3–8 (–13) flowers, rarely flowers solitary; calyx appendages erect or spreading, 0.9–3 mm long; corolla (5–) 6–10 mm long, entirely yellow or with diffuse greenish spots within; Mexico, Guatemala, Honduras, El Salvador, Nicaragua and Costa Rica (also South America)	***C.rhomboideum***
–	Plants glabrous or glabrescent only with simple eglandular trichomes; major leaves elliptic to lanceolate; inflorescence of a solitary flower, rarely two; calyx appendages spreading or strongly reflexed, (2–) 3–5 mm long; corolla 9.8–14 mm long, purple with white margin within; Mexico, Guatemala and Honduras	***C.lanceolatum***

### ﻿Key to the wild Andean and adjacent Andean (Venezuela, Colombia, Ecuador, Peru, Bolivia and Argentina) *Capsicum* species

**Table:** 

1	Stem and mature leaf blades completely glabrous, if trichomes present sparsely distributed on the veins and margins	**2**
–	Stem and mature leaf blades variously pubescent	**7**
2	Calyx appendages (3–) 5, strongly incurved; flowering pedicels slightly winged and conspicuously winged in fruit; leaves coriaceous; Bolivia	***C.ceratocalyx***
–	Calyx appendages absent or up to 10, straight; flowering and fruiting pedicels not winged; leaves coriaceous or membranous	**3**
3	Corolla tubular-campanulate to broadly campanulate, lobed less than 1/3 of the way to the base	**4**
–	Corolla stellate or stellate-campanulate, lobed between 1/3–2/3 of the way to the base	**6**
4	Calyx appendages 10, unequal (five long, five short); corolla lobes recurved; fruits pungent; seeds 3–4 (–5) mm long, pale yellow to nearly white; Bolivia	***C.caballeroi***
–	Calyx appendages 2–5, equal or subequal; corolla lobes erect; fruits non-pungent; seeds 1.5–2.5 mm long, brown to black	**5**
5	Leaves coriaceous; calyx appendages (2–) 3–5, spreading or reflexed; filaments 1–2.5 mm long; corolla broadly campanulate; Colombia and Ecuador	***C.lycianthoides***
–	Leaves membranous; calyx appendages five, erect; filaments 3–5 mm long; corolla tubular-campanulate; Peru	***C.piuranum***
6	Leaves coriaceous; major leaves narrowly elliptic (length/width ratio 6–10.8); calyx tube membranaceous; stamens equal, 2–2.6 mm long; fruits 8–13 mm in diameter, orange at maturity; fruiting pedicels 10–16 mm long, green, pendent; fruiting calyx green-purple or green; seeds 1.7–2.3 mm long, 1.7–2.2 mm wide, seeds D or teardrop-shaped, the surface reticulate; Peru and Ecuador	***C.longifolium***
–	Leaves membranaceous; major leaves elliptic (length/width ratio 2.5–4); calyx tube fleshy; stamens subequal (one longer), (2–) 3–4.3 mm long; fruits 6–9 mm in diameter, dark blue to purple at maturity; fruiting pedicels ca. 18 mm long, brilliant dark purple, erect; fruiting calyx entirely brilliant purple; seeds 2.75–3.40 mm long, 2.25–2.70 mm wide, C-shaped, the surface smooth and tuberculate at margins; Colombia, Ecuador, and Peru	***C.regale***
7	Pubescence mostly of branched eglandular or long forked glandular trichomes, few simple trichomes	**8**
–	Pubescence mostly of simple eglandular trichomes, rarely furcate eglandular trichomes or simple long glandular trichomes	**9**
8	Dense glandular pubescence of long furcate and simple trichomes; calyx appendages usually 10 (rarely 5 or up to 12); corolla stellate, lobed nearly halfway to the base, white with greenish-yellow spots within (sometimes nearly white or with purple spots in the lobes); flowering and fruiting pedicels erect; fruits pungent; inflorescences usually 2–3 (–4)-flowered; Bolivia	***C.eshbaughii***
–	Dense eglandular pubescence of simple, furcate and dendritic trichomes; calyx appendages usually five (rarely 3–4); corolla campanulate or campanulate-rotate, shallowly lobed, entirely yellow or sometimes tinged greenish within; flowering and fruiting pedicels pendent; fruits non-pungent; inflorescences usually 3–8 (–13)-flowered; Venezuela to Peru (also in Mexico and Central America)	***C.rhomboideum***
9	Corolla nearly lobed up to the base, tube 4–4.5 times shorter than the lobes; Ecuador	***C.benoistii***
–	Corolla shallowly lobed or lobed halfway or up to 2/3 of the way to the base, tube as long as the lobes or 1.5 times shorter than the lobes	**10**
10	Calyx appendages (3–) 5, strongly incurved; flowering pedicels nearly winged and conspicuously winged in fruit; leaves coriaceous; Bolivia	***C.ceratocalyx***
–	Calyx appendages absent or up to 10, straight; flowering and fruiting pedicels not winged; leaves coriaceous or membranous	**11**
11	Young stems, lower surface of the leaves and calyx with small dark glandular trichomes mixed with simple eglandular trichomes; corolla primarily purple, violet or lilac; fruits pungent	**12**
–	Young stems, leaves and calyx only with simple eglandular trichomes, glandular trichomes absent; corolla entirely yellow or nearly white or primarily purple and usually with maroon, purple or greenish-yellow pigmentation within; fruits pungent or non-pungent	**13**
12	Calyx appendages absent or five, minute ≤ 0.5 mm long; leaves moderately to densely pubescent; flowering pedicels usually pendent, non-geniculate at anthesis, 3–10 mm long; style dimorphic; fruiting pedicels erect; Peru	***C.tovarii***
–	Calyx appendages five, 1–2 mm long; leaves glabrescent; flowering pedicels usually erect, geniculate at anthesis, 8–18 (–22) mm long; style homomorphic; fruiting pedicels pendent; Bolivia	***C.cardenasii***
13	Corolla tubular-campanulate or campanulate, lobed less than 1/3 of the way to the base	**14**
–	Corolla stellate or rotate-stellate, lobed between 1/3–2/3 of the way to the base	**17**
14	Calyx appendages 8–10, unequal	**15**
–	Calyx appendages 2–5, equal or subequal	**16**
15	Leaves coriaceous; inflorescences 2-flowered or flowers solitary; flowering pedicels 20–40 (–50) mm long; corolla ≥ 10 mm long, 4–6 mm in diameter; filaments 4–6 mm long; style 7–9 mm long; fruits pungent; fruiting calyx appendages appressed to the berry; seeds pale yellow to nearly white; Bolivia	***C.caballeroi***
–	Leaves membranous; inflorescences with 2–7 flowers, rarely flowers solitary; flowering pedicels shorter, 8–15 mm long; corolla 7.5–9 mm long, 8–10 mm in diameter, filaments (1.5–) 1.8–2 mm long; style ca. 4 mm long; fruits non-pungent; fruiting calyx appendages spreading or reflexed; seeds yellow or brown; Ecuador and Peru	***C.hookerianum***
16	Calyx appendages five, erect; corolla tube narrow, ca. 6 mm in diameter; filaments 3–5 mm long; mature leaves glabrescent adaxially; stone cells two; northern Peru	***C.piuranum***
–	Calyx appendages 2–3 (–5), erect or spreading; corolla tube broad, 8–10 mm in diameter; filaments 2–3 mm; mature leaves sparse to densely pubescent adaxially; stone cells 1–5 or absent; Colombia, Ecuador and Peru	***C.geminifolium***
17	Flowers 4–13 (–18) on a short rachis; pedicel scars prominent, corky; fruiting calyx usually recurved; sprawling vines or scrambling shrubs; Peru, Bolivia (also Brazil)	***C.coccineum***
–	Flowers 2–6 (–8) per axil, rarely flowers solitary; pedicel scars usually inconspicuous, rarely prominent; fruiting calyx not recurved; usually scandent or erect shrubs or subshrubs, rarely perennial herbs	**18**
18	Calyx appendages absent or minute, ≤ 1 mm long	**19**
–	Calyx appendages (4–) 5–10, > 1 mm long	**21**
19	Leaf pair strongly dissimilar in shape and size; flower buds ovoid, purple or yellowish; fruits non-pungent; seeds 1.9–2.7 mm long, 1.8–2.1 mm wide, brownish-black to black; corolla entirely yellow or with purple or maroon spots within; Colombia, Ecuador and Peru	***C.dimorphum***
–	Leaf pair similar or dissimilar in size, similar in shape; flower buds globose or ovoid, white cream or greenish-white; fruits pungent; seeds 3.2–4 mm long, 2.5–4 mm wide, pale yellow to yellow; corolla entirely white or pale yellow, rarely greenish-white	**20**
20	Calyx circular in outline; corolla 4–5 mm long, ca. 6 mm in diameter, with glandular trichomes adaxially; style 2.25–2.5 mm long, exserted 0.5–0.8 mm beyond the anthers; seed coat smooth; plants densely pubescent, the trichomes spreading; endemic; Ecuador, Galapagos Islands	***C.galapagoense***
–	Calyx pentagonal in outline; corolla (5–) 6–8 mm long, 8–10 (–12) mm in diameter, glabrous adaxially; style 4–4.8 mm long, exserted 1.5–2 mm beyond the anthers; seed coat reticulate to obscurely reticulate; plants glabrescent to densely pubescent, the trichomes appressed-antrorse, sometimes spreading; widespread; Colombia, Venezuela, Ecuador, Peru, Bolivia (also in North and Central America, the Caribbean Islands and Brazil	**C.annuumvar.glabriusculum**
21	Flowers solitary; corolla entirely white; filaments with conspicuous auricles not fused to the corolla at the point of insertion; Bolivia and Argentina (also Paraguay)	***C.chacoense***
–	Flowers 2–8 per axil, rarely solitary; corolla white, purple or yellow with greenish-yellow spots or greenish-yellow centre within; filaments with inconspicuous auricles fused to the corolla at the point of the insertion	**22**
22	Flowering pedicels pendent, non-geniculate at anthesis; calyx appendages 5–10	**23**
–	Flowering pedicels erect, geniculate at anthesis; calyx appendages (4–) 5	**24**
23	Calyx appendages always 10; calyx tube strongly 10-nerved with prominent venation; pedicels scars inconspicuous; corolla entirely yellow or with small greenish-yellow spots within; fruits red; seeds 4–5 mm long, 3–4.25 mm wide, pale yellow to white; Bolivia	***C.neei***
–	Calyx appendages 5 (–7); calyx tube strongly 5-nerved with prominent venation; pedicels scars prominent; corolla purple with a narrow white margin and yellowish-green centre; fruits greenish-golden yellow; seeds 3–3.8 mm long, 2.7–3 mm wide, brownish-black; Colombia and Venezuela (also Brazil)	***C.parvifolium***
24	Corolla rotate-stellate; white with greenish-yellow spots within; seeds pale yellow to yellow; fruits globose, subglobose or ellipsoid; Colombia to Argentina (also Paraguay and Brazil)	**C.baccatumvar.baccatum**
–	Corolla stellate; primarily yellow or lilac, purple or magenta; seeds brownish-yellow or dark brown; fruits globose	**25**
25	Calyx appendages five, 1–1.5 mm long; filaments 2–2.5 mm long; corolla yellow with small and faint greenish-yellow spots within; seeds 4–4.5 mm long, 3–3.5 mm wide, dark brown; Bolivia	***C.minutiflorum***
–	Calyx appendages (4–) 5, 1.2–2.7 (–3) mm long; filaments 2.7–3.8 mm long; corolla lilac, purple or magenta with a continuous greenish-yellow or ochre tube within, sometimes the corolla white with greenish-yellow spots; seeds 2.5–4 (–4.2) mm long, 2.1–3 mm wide, brownish-yellow; Bolivia and Argentina	***C.eximium***

### ﻿Key to the wild *Capsicum* species occurring in Brazil, Paraguay and Argentina

**Table:** 

1	Stem and mature leaf blades completely glabrous, if trichomes present, sparsely distributed on the veins and margins	**2**
–	Stem and mature leaf blades variously pubescent	**4**
2	Calyx appendages five (6–10), unequal, 1–5 mm long; corolla 10–14 (–16) mm long; Brazil (São Paulo)	***C.hunzikerianum***
–	Calyx appendages absent or five, minute, < 0.5 mm long; corolla 4.5–10 mm long	**3**
3	Leaves membranous, elliptic to ovate; flowering pedicels 9–14 mm long, erect, geniculate at anthesis; corolla small, 4.5–6.5 (–8) mm long; stamens unequal (3+2); ovules 2 per locule; fruits 4-seeded; Brazil (Rio de Janeiro, Minas Gerais, Espírito Santo)	***C.campylopodium***
–	Leaves coriaceous, elliptic to narrowly elliptic, flowering pedicels 15–30 mm long, pendent, non-geniculate at anthesis; corolla larger, 9–10 mm long; stamens equal; ovules more than 2 per locule; fruits multi-seeded (up to 20 seeds); Brazil (Bahía, Espírito Santo, Minas Gerais, São Paulo)	***C.pereirae***
4	Pubescence of furcate to dendritic eglandular trichomes mixed with few simple eglandular trichomes; fruits non-pungent; calyx appendages long, (4.5–) 5–8.5 mm long; Brazil (Bahía, Minas Gerais, Pernambuco)	***C.longidentatum***
–	Pubescence of simple eglandular trichomes, rarely furcate trichomes; fruits usually pungent; calyx appendages absent or, if present, up to 6 mm long	**5**
5	Corolla campanulate-urceolate, rotate or rotate-stellate, lobed less than 1/3 of the way to the base	**6**
–	Corolla stellate, lobed more than 1/3 up to 2/3 of the way to the base	**8**
6	Corolla campanulate-urceolate, entirely fuchsia or violet, glabrous adaxially, the lobes spreading to strongly recurved; fruiting pedicels pendent; fruits greenish-golden yellow, slightly pungent; seeds brownish-black to black; Brazil (Rio de Janeiro)	***C.friburgense***
–	Corolla rotate or rotate-stellate, white or primarily purple or lilac with greenish-yellow pigmentation within, with glandular trichomes adaxially; the lobes spreading; fruiting pedicels erect; fruits usually red, pungent; seeds pale yellow to yellow	**7**
7	Leaves with dense pubescence, especially underneath; corolla marginally purple or lilac with greenish-yellow centre; Brazil and Paraguay	***C.rabenii***
–	Leaves mostly glabrescent, more rarely moderately pubescent; corolla white with greenish-yellow spots within, purple pigmentation absent; widely distributed across South America	**C.baccatumvar.baccatum**
8	Calyx appendages absent or up to five, minute, < 0.5 mm long	**9**
–	Calyx appendages 2–10, > 0.5 mm long	**16**
9	Flowering pedicels pendent, non-geniculate at anthesis	**10**
–	Flowering pedicels erect, geniculate at anthesis	**11**
10	Young stems, leaves and calyx with simple eglandular trichomes mixed with small dark glandular trichomes; inflorescences multi-flowered (5–13 flowers or up to 20 or more); corolla purple with a white margin within; fruiting pedicels green or purple, with a constriction at the junction with the calyx; seeds pale yellow; Brazil	***C.caatingae***
–	Young stems, leaves and calyx only with simple eglandular trichomes, glandular trichomes absent; inflorescences few-flowered (2–6 flowers), rarely solitary flowers; corolla white with greenish-yellow spots, rarely also with purple spots; fruiting pedicels green, without a constriction at the junction with the calyx; seeds brownish-black; Argentina, Paraguay and Brazil	***C.flexuosum***
11	Flowers 4–13 (–18) on a short rachis; pedicels scars prominent, corky; fruiting calyx usually recurved; seeds 4–4.6 mm long, (–2.8) 3.2–3.75 mm wide, yellow to brownish-yellow; sprawling vines or scrambling shrubs, with stems to 7 m long; Brazil (also Peru and Bolivia)	***C.coccineum***
–	Flowers 2–5 (–7) per axil, rarely solitary; pedicels scars inconspicuous; fruiting calyx not recurved; seeds 2–4 mm long, 2.5–3.5 mm wide, pale yellow or brownish-black to black; erect shrubs or subshrubs up to 2.5 m tall, rarely low perennial herbs or small trees	**12**
12	Stamens unequal (3+2); ovules two per locule; fruits 4-seeded; Brazil (Rio de Janeiro, Minas Gerais, Espírito Santo)	***C.campylopodium***
–	Stamens equal; ovules more than two per locule; fruits multi-seeded (up to 20 seeds)	**13**
13	Filaments short, < 2 mm long; fruiting pedicels erect; seeds pale yellow to yellow; fruits yellow, red-orange or red	**14**
–	Filaments longer, 2.4–4 mm long; fruiting pedicels pendent; seeds brownish-black to black; fruits greenish-golden yellow	**15**
14	Fruiting calyx without a prominent annular constriction at junction with the pedicel	**C.annuumvar.glabriusculum**
–	Fruiting calyx with a prominent annular constriction at junction with the pedicel	***C.chinense***
15	Stems, leaves, pedicels and calyx densely pubescent with long spreading eglandular trichomes (0.5–2 mm long); major leaves elliptic to narrowly elliptic; corolla white with large greenish-yellow spots and sparse diffuse purple or brown spots within, the lobes widely triangular; Brazil (Rio de Janeiro)	***C.muticum***
–	Stems, leaves pedicels, and calyx glabrescent to moderately pubescent, with short antrorse eglandular trichomes (0.25–0.5 mm long); major leaves elliptic to ovate; corolla white with large or small purple or brownish spots and large greenish-yellow spots within (in some populations, purple or brownish pigmentation absent entirely); the lobes triangular or ovate; Brazil (Minas Gerais, Rio de Janeiro and São Paulo)	***C.schottianum***
16	Flowers solitary; corolla entirely white; staminal plaques with auricles not fused to the corolla at the point of insertion; Argentina and Paraguay (also Bolivia)	***C.chacoense***
–	Flowers 2–18, rarely flowers solitary; corolla yellow, purple or white tinged of different colours within; staminal plaques with auricles fused to the corolla at the point of insertion	**17**
17	Corolla usually yellow with yellowish-green or purple-brown spots within; fruiting calyx usually recurved; seeds 4–4.6 mm long, (–2.8) 3.2–3.75 mm wide, yellow to brownish yellow; sprawling vines or scrambling shrubs, with stems to 7 m long; western Brazil (also Peru and Bolivia)	***C.coccineum***
–	Corolla primarily purple or white; fruiting calyx not recurved; seeds (2–) 2.2–3.5 (–4) mm long, (–1.8) 2–3 (–3.5) mm wide, yellow or brownish-black to black; erect shrubs or subshrubs, up to 5 m tall	**18**
18	Calyx appendages 10 or 6–10, rarely five	**19**
–	Calyx appendages five (very rarely up to seven)	**21**
19	Calyx appendages 10, subequal; filaments 3–3.2 mm long; style barely exserted beyond the anthers; anthers lilac or pale blue; corolla almost entirely purple; Brazil (São Paulo)	***C.mirum***
–	Calyx appendages ranging from 6 to 10, rarely five, unequal; filaments 1.4–2.5 mm long; style exserted 0.8–1.3 mm beyond the anthers; anthers yellow, light green or grey; corolla white with greenish-yellow or purple spots within	**20**
20	Flowering calyx appendages cylindrical or triangular-compressed, glabrous to moderately pubescent with antrorse trichomes, the longest appendages 1–2.5 mm; corolla 6–7 mm long, ca. 11 mm in diameter, white with greenish-yellow spots within; fruiting calyx appendages strongly recurved; fruiting pedicels 18–25 mm long; Brazil (Minas Gerais, Paraná, Rio de Janeiro, Santa Catarina and São Paulo)	***C.recurvatum***
–	Flowering calyx appendages linear or subulate, densely pubescent with spreading trichomes, the longest appendages 2.5–5 (–6) mm long; corolla (8–) 9–14 mm long, 18–22 mm in diameter, white with purple or reddish-brown spots within; fruiting calyx appendages spreading; fruiting pedicels (25–) 30–38 mm long; Brazil (São Paulo and Rio de Janeiro)	***C.cornutum***
21	Major leaves ovate; corolla primarily purple with greenish-yellow or cream centre within	**22**
–	Major leaves elliptic or narrowly elliptic, more rarely ovate	**23**
22	Pedicels scars prominent; flowering and fruiting pedicels pendent; fruits greenish-golden yellow; seeds brownish-black, the seed coat reticulate and tuberculate at margins; Brazil (also Colombia and Venezuela)	***C.parvifolium***
–	Pedicels scars inconspicuous; flowering and fruiting pedicels erect; fruits orange or red; seeds pale yellow or yellow, the seed coat smooth; Paraguay and Brazil	***C.rabenii***
23	Plants densely pubescent on stems, petioles, pedicels and sometimes also on the leaf nerves beneath, the trichomes spreading; Brazil (Rio de Janeiro, Minas Gerais, São Paulo and Espírito Santo)	***C.villosum***
–	Plants glabrescent to densely pubescent on stems, leaves and pedicels, the trichomes appressed-antrorse; calyx appendages up to 5 mm long	**24**
24	Plants glabrous to sparsely pubescent; major leaves elliptic to ovate, rarely narrowly elliptic (length/width ratio: (2–) 2.5–4 (–4.9), apex acuminate to long acuminate; calyx appendages (0.4–) 0.5–1.5 (–3) mm; flower buds purple; corolla (6–) 7.5–12 mm long, (9–) 10–13 mm in diameter; Brazil (Bahia, Espírito Santo, Minas Gerais, Rio de Janeiro and São Paulo)	***C.mirabile***
–	Plants moderately to densely pubescent; major leaves narrowly elliptic to lanceolate (length/width ratio: (4–) 5–10 (–16), apex acute to obtuse; calyx appendages (2.8–) 3–4 (–5) mm; flower buds cream with greenish and purple spots; corolla (8–) 10–12 mm long, 13–20 mm in diameter; Brazil (Minas Gerais)	***C.carassense***

### ﻿Key to the domesticated *Capsicum* species

**Table:** 

1	Calyx appendages absent or minute, ≤ 0.5 mm long	**2**
–	Calyx appendages > 0.5 mm long	**4**
2	Flowers solitary, rarely in pairs or more; petioles up to 10 cm long; corolla 8–15 mm long, entirely white, rarely entirely purple or pale yellow; fruits usually large, up to 300 mm long, pungent or non-pungent	**C.annuumvar.annuum**
–	Flowers 2–5, rarely solitary; petioles up to 3.5 cm long; corolla 3.75–8 mm long, entirely dull white or greenish-white (occasionally with purple spots); fruits small to medium-sized, up to 100 mm long, pungent, rarely non-pungent	**3**
3	Corolla glabrous adaxially; style heteromorphic; fruits subglobose to highly variable in shape, with the base obtuse or truncate; fruiting calyx discoid or shallowly cup-shaped, with a prominent annular constriction at junction with the pedicel; ovary subglobose, 2–2.5 mm long, 2.5–3.5 mm in diameter	***C.chinense***
–	Corolla with small glandular trichomes adaxially; style homomorphic; fruits usually elongate, narrowly triangular, with the base abruptly narrowed; fruiting calyx deeply cup-shaped lacking a constriction at junction with the pedicel; ovary oblong-ovoid, 2.5–4 mm long, 1.3–1.8 mm in diameter	***C.frutescens***
4	Leaves densely pubescent, rarely glabrescent, the youngest leaves rugose; flower buds dark purple on pendent pedicels; corolla dark purple or violet with a white or yellowish-green centre within; style clavate; seeds 5.5–7 mm long, 4.8–6 mm wide, brownish-black to black, the seed coat reticulate	***C.pubescens***
–	Leaves glabrous to sparsely pubescent; the youngest leaves even; flower buds greenish-white (rarely purple) on geniculate pedicels; corolla white with large greenish-yellow spots and white centre within, style cylindrical; seeds 3–5.2 mm long, 3–4 mm wide, pale yellow to yellow, the seed coat smooth to slightly reticulate	**5**
5	Fruits pungent, rarely non-pungent, usually elongate, endocarp alveolate, pericarp with giant cells	**C.baccatumvar.pendulum**
–	Fruits non-pungent, campanulate-umbilicate, endocarp smooth, pericarp without giant cells	**C.baccatumvar.umbilicatum**

### ﻿Species descriptions

### ﻿1. Capsicum annuum

Taxon classificationPlantaeSolanalesSolanaceae

L., Sp. Pl. 1: 188. 1753.

0123B844-DD8C-5B6D-B2D1-753D9878FCCD

#### Type.

“Habitat in America meridionali” Herb. Clifford: 59, *Capsicum* 1 (lectotype, designated by D’Arcy 1974 [’1973’], pg. 591: BM [BM000558022]).

### ﻿1a. Capsicum annuum L. var. annuum

Taxon classificationPlantaeSolanalesSolanaceae

E53925CD-35EB-559E-B5F8-01EEBEFEA28B

Figs 20 , 21

Capsicum grossum L., Mant. Pl.: 47. 1767. Type. “Habitat in India … *H.U.*” HU [Horto Upsaliensis]: Fructu vario crasso. Caulis biennis, Herb. Linn. N° 249.5 (lectotype, designated here: LINN [LINN-HL249-5]).
Capsicum cordiforme Mill., Gard. Dict. ed. 8, no. 2. 1768. Type. Cultivated at the Chelsea Physic Garden (no specimens cited; no original material located).
Capsicum tetragonum Mill., Gard. Dict. ed. 8, no. 3. 1768. Type. Cultivated at the Chelsea Physic Garden (no specimens cited; no original material located).
Capsicum angulosum Mill., Gard. Dict. ed. 8, no. 4. 1768. Type. Cultivated at the Chelsea Physic Garden (no specimens cited; no original material located).
Capsicum olivaeforme Mill., Gard. Dict. ed. 8, no. 6. 1768. Type. Cultivated at the Chelsea Physic Garden, seeds from “Barbadoes” (no specimens cited; no original material located).
Capsicum pyramidale Mill., Gard. Dict. ed. 8, no. 7. 1768. Type. Cultivated at the Chelsea Physic Garden, seeds from Egypt (no specimens cited; no original material located).
Capsicum conicum Lam., Tabl. Encycl. 2: 26. 1794. Type. “Ex Indiis” Herb. Lamarck s.n. (lectotype, designated here: P-LAM [P00357734]).
Capsicum bicolor Jacq., Fragm. Bot. 66, tab 99, fig. 1. 1809. Type. “Patriam ignoro” (no specimens cited; lectotype, designated here: Jacquin, Fragm. Bot.: 66, tab 99, fig. 1. 1809).
Capsicum grossum Willd., Enum. Pl. [Willdenow] 1: 241. 1809, nom. illeg., not Capsicumgrossum L. (1767). Type. “*Habitat in* India *orientali*” Capsicumgrossum [sheet] 2, Herb. *Willdenow* (lectotype, designated here: B [B-W04425-02-0]).
Capsicum sphaericum Willd., Enum. Pl. [Willdenow] 1: 241. 1809. Type. “Habitat…. ” (lectotype, designated here: B [B-W04426-01-0, F neg. 2886]).
Capsicum nigrum Willd., Enum. Pl. [Willdenow] 1: 242. 1809, nom. illeg. superfl. Type. Based on Capsicumbicolor Jacq. (cited in synonymy).
Capsicum purpureum Vahl ex Hornem., Hort. Bot. Hafn. 1: 224. 1813. Type. [Denmark]. Hort. Haf., 1802, Herb. Vahl s.n. (lectotype, designated here: C [C10019148]).
Capsicum ovatum DC., Cat. Pl. Horti Monsp.: 86. 1813. Type. “Habitat….” (no specimens cited; no original material located; Capsicumovatum, Anonymous s.n. (neotype, designated here: G-DC [G00200072]).
Capsicum longum DC., Cat. Pl. Horti Monsp.: 86. 1813. Type. “Hab... in hortis frequens” (no specimens cited; lectotype, designated here [illustration]: “Piper Calecuticum sive Capsicum oblongius, Bauhin et al., Hist. pl. 2: 943, f. I. 1651).
Capsicum globiferum G.Mey., Prim. Fl. Esseq.: 113. 1818. Type. “In plantationibus”, no specimens cited; [Guyana]. Río Essequibo, *E.K. Rodschied 29* (lectotype, designated here: GOET [GOET003420]).
Capsicum purpureum Roxb., Fl. Ind., ed. Carey & Wall. 2: 259. 1824, nom. illeg., not Capsicumpurpureum Vahl ex Hornem. (1813). Type. “Most likely from the Molucca Islands” (no specimens cited; neotype, designated here: “C.purpureum, H.B.C.” [Horto Botanici Calcutta]: K [K001132446]).
Capsicum indicum Dierb. var. vulgatum Dierb., Arch. Apotheker-Vereins Nordl. Teutschl. 30: 22. 1829, nom. illeg. superfl. Type. Based on Capsicumannuum L. (cited in synonymy).
Capsicum indicum Dierb. var. longum (DC.) Dierb., Arch. Apotheker-Vereins Nordl. Teutschl. 30: 23. 1829. Type. Based on Capsicumlongum DC.
Capsicum indicum Dierb. var. tetragonum (Mill.) Dierb., Arch. Apotheker-Vereins Nordl. Teutschl. 30: 23. 1829. Type. Based on Capsicumtetragonum Mill.
Capsicum indicum Dierb. var. angulosum (Mill.) Dierb., Arch. Apotheker-Vereins Nordl. Teutschl. 30: 25. 1829. Type. Based on Capsicumangulosum Mill.
Capsicum indicum Dierb. var. cordiforme (Mill.) Dierb., Arch. Apotheker-Vereins Nordl. Teutschl. 30: 25. 1829. Type. Based on Capsicumcordiforme Mill.
Capsicum indicum Dierb. var. grossum (L.) Dierb., Arch. Apotheker-Vereins Nordl. Teutschl. 30: 26. 1829. Type. Based on Capsicumgrossum L.
Capsicum indicum Dierb. var. sphaericum (Willd.) Dierb., Arch. Apotheker-Vereins Nordl. Teutschl. 30: 27. 1829. Type. Based on Capsicumsphaericum Willd.
Capsicum indicum Dierb. var. ovatum (DC.) Dierb., Arch. Apotheker-Vereins Nordl. Teutschl. 30: 27. 1829. Type. Based on Capsicumovatum DC.
Capsicum indicum Dierb. var. pyramidale (Mill.) Dierb., Arch. Apotheker-Vereins Nordl. Teutschl. 30: 28. 1829. Type. Based on Capsicumpyramidale Mill.
Capsicum indicum Dierb. var. olivaeforme (Mill.) Dierb., Arch. Apotheker-Vereins Nordl. Teutschl. 30: 28. 1829. Type. Based on Capsicumolivaeforme Mill.
Capsicum indicum Dierb. var. nigrum (Willd.) Dierb., Arch. Apotheker-Vereins Nordl. Teutschl. 30: 29. 1829. Type. Based on Capsicumnigrum Willd.
Capsicum axi Vell., Fl. Flumin.: 61. 1829 (“1825”); Fl. Flumin. Icon. 2: t. 6. 1831 (“1827”). Type. Brazil. [Rio de Janeiro]: “Colitur hortis” (lectotype, designated by Knapp et al. 2015, pg. 824: [illustration] Original parchment plate of Flora Fluminensis in the Manuscript Section of the Biblioteca Nacional, Rio de Janeiro [cat. no.: mss1198651_009] and later published in Vellozo, Fl. Flumin. Icon. 2: t. 6. 1831).
Capsicum silvestre Vell., Fl. Flumin. 60. 1829 (“1825”); Fl. Flumin. Icon. 2: t. 1. 1831 (“1827”). Type. Brazil. [Rio de Janeiro]: “Ad declivium Alpium Fluminensium” (lectotype, designated by Knapp et al. 2015, pg. 824: [illustration] Original parchment plate of Flora Fluminensis in the Manuscript Section of the Biblioteca Nacional, Rio de Janeiro [cat. no.: mss1198651_004] and later published in Vellozo, Fl. Flumin. Icon. 2: t. 1. 1831).
Capsicum annuum L. var. rugosulum Fingerh., Monogr. Capsic.: 13. 1832. Type. No locality cited (no specimens cited; lectotype, designated here [illustration]: Fingerhuth, Monogr. Capsic. Tab. II b. 1832).
Capsicum annuum L. var. acuminatum Fingerh., Monogr. Capsic.: 13. 1832. Type. No locality cited (no specimens cited; lectotype, designated here [illustration]: Fingerhuth, Monogr. Capsic. Tab. II c. 1832).
Capsicum annuum L. var. subangulosum Fingerh., Monogr. Capsic. 13. 1832. Type. No locality cited (no specimens cited; lectotype, designated here [illustration]: Fingerhuth, Monogr. Capsic. Tab. II d. 1832).
Capsicum annuum L. var. ovoideum Fingerh., Monogr. Capsic.: 14. 1832. Type. No locality cited (no specimens cited; lectotype, designated here [illustration]: Fingerhuth, Monogr. Capsic. Tab. II e. 1832).
Capsicum annuum L. var. abbreviatum Fingerh., Monogr. Capsic.: 14. 1832. Type. No locality cited (no specimens cited; lectotype, designated here [illustration]: Fingerhuth, Monogr. Capsic. Tab. II f. 1832).
Capsicum annuum L. var. olivaeforme Fingerh., Monogr. Capsic.: 14. 1832. Type. “Crecit in America meridionali et India oriental” (no specimens cited; lectotype, designated here [illustration]: Fingerhuth, Monogr. Capsic. Tab. II g. 1832).
Capsicum bicolor Jacq. var. purpureum (Vahl ex Hornem.) Fingerh., Monogr. Capsic.: 16. 1832. Type. Based on Capsicumpurpureum Vahl ex Hornem.
Capsicum strictum Fingerh., Monogr. Capsic.: 21. 1832. Type. “Patria…..” (no specimens cited; lectotype, designated here [illustration]: Fingerhuth, Monogr. Capsic. Tab. V a. 1832).
Capsicumgrossum Willd. var. pomiforme Fingerh., Monogr. Capsic.: 22. 1832. Type. No locality cited (no specimens cited; lectotype, designated here [illustration]: Fingerhuth, Monogr. Capsic. Tab. V c. 1832).
Capsicum grossum Willd. var. ovatum Fingerh., Monogr. Capsic.: 22. 1832. Type. No locality cited (no specimens cited; lectotype, designated here [illustration]: Fingerhuth, Monogr. Capsic. Tab. V d. 1832).
Capsicumgrossum Willd. var. cordatum Fingerh., Monogr. Capsic.: 22. 1832. Type. No locality cited (no specimens cited; lectotype, designated here [illustration]: Fingerhuth, Monogr. Capsic. Tab. VI a. 1832).
Capsicumgrossum Willd. var. angulosum Fingerh., Monogr. Capsic.: 22. 1832. Type: “Patria India orientalis (Herb. Wight et Herb. Hamilt.)” (no specimens found; lectotype, designated here [illustration]: Fingerhuth, Monogr. Capsic. Tab. VI d. 1832).
Capsicum ceratocarpum Fingerh., Monogr. Capsic.: 22. 1832. Type. “Patria….” (no specimens cited; lectotype, designated here [illustration]: Fingerhuth, Monogr. Capsic. Tab. VI c. 1832).
Capsicum longum DC. var. incrassatum Fingerh., Monogr. Capsic.: 24. 1832. Type. No locality cited (no specimens cited; lectotype, designated here [illustration]: Fingerhuth, Monogr. Capsic. Tab. VII a. 1832).
Capsicum longum DC. var. latum Fingerh., Monogr. Capsic.: 25. 1832. Type. No locality cited (no specimens cited; lectotype, designated here [illustration]: Fingerhuth, Monogr. Capsic. Tab. VII b (as - - [Capsicumlongum] luteum). 1832).
Capsicum longum DC. var. rectum Fingerh., Monogr. Capsic.: 25. 1832. Type. “Cresit in Indiis et America meridionali” (no specimens cited; lectotype, designated here [illustration]: Fingerhuth, Monogr. Capsic. Tab. VII c. 1832).
Capsicum pendulum Willd. var. torulosum Fingerh., Monogr. Capsic.: 26. 1832. Type. [Indonesia] “in Amboina” (no specimens cited; lectotype, designated here [illustration]: Capsicumrubrumminus Rumphius, Herbarium Amboinense 5, Tab. LXXXVIII, fig. 1, 1747, cited in synonymy).
Capsicum angulosum Mill. var. conicum Fingerh., Monogr. Capsic.: 28. 1832. Type. No locality cited (no specimens cited, no original material located).
Capsicum angulosum Mill. var. ovale Fingerh., Monogr. Capsic.: 28. 1832. Type. “Patria….?” (no specimens cited; lectotype, designated here [illustration]: Fingerhuth, Monogr. Capsic. Tab. VIII b. 1832).
Capsicum hamiltonii G.Don, Gen. Hist. 4: 447. 1838. Type. [Caribbean Islands] “Native of the Island of Nevis, in gardens” (no specimens cited, no original material located).
Capsicum annuum L. var. longum (DC.) Sendtn., Fl. Bras. (Martius) 10(6): 144. 1846. Type. Based on Capsicumlongum DC.
Capsicum annuum L. var. grossum (Willd.) Sendtn., Fl. Bras. (Martius) 10(6): 147. 1846. Type. Based on Capsicumgrossum Willd.
Capsicum annuum L. var. cordiforme (Mill.) Sendtn., Fl. Bras. (Martius) 10(6): 148. 1846. Type. Based on Capsicumcordiforme Mill.
Capsicum abyssinicum A.Rich., Tent. Fl. Abyss 2: 96. 1850. Type. [Ethiopia] “Abyssinia, Ouedjerate”, R. Quartin Dillon s.n. (lectotype, designated here: P [P00329903]; isolectotypes: P [P00329904, P00329905]).
Capsicum annuum L. var. oblongum Dunal, Prodr. [A. P. de Candolle] 13(1): 412. 1852. Type. “Capsicumannuum α oblongum fructibus rubris”, 1844, *Herb. Dunal* (lectotype, designated here: G-DC [G00131768]).
Capsicum pyramidale Mill. var. longicorne Dunal, Prodr. [A. P. de Candolle] 13(1): 414. 1852. Type. [Indonesia] Java, 1843, *H. Zollinger 489* (lectotype, designated here: G-DC [G00131841]; isolectotypes: G [G00390281], LE).
Capsicum bicolor Jacq. var. purpureum (Vahl ex Hornem.) Dunal, Prodr. [A. P. de Candolle] 13(1): 414. 1852. Type. Based on Capsicumpurpureum Vahl ex Hornem.
Capsicum testiculatum Vis. ex Dunal, Prodr. [A. P. de Candolle] 13(1): 424. 1852. Type. In Hort. Montpellier [seeds sent by R. de Visiani], 1837, Anonymous s.n. (lectotype, designated here: G-DC [G00200067]; isolectotype: MPU [MPU023039]).
Capsicum angulosum Mill. var. macrocarpum Dunal, Prodr. [A. P. de Candolle] 13(1): 426. 1852. Type. No locality cited (no specimens cited; lectotype, designated here [illustration]: Fingerhuth, Monogr. Capsic. Tab. VIII a (as Capsicumangulosum M.). 1832).
Capsicum leucocarpon Dunal, Prodr. [A. P. de Candolle] 13(1): 429. 1852. Type. Cultivated in England “Capsicumamericanumlatifolium, fructu oblongo erecto candido” (Miller 1752) (no specimens cited, no original material located).
Capsicum dulce Dunal, Prodr. [A. P. de Candolle] 13(1): 428. 1852. Type. Cultivated in Montpellier, France, “In hortis botanicis cultum” (no specimens cited; no original material located).
Capsicum annuum L. var. cordiforme (Mill.) Alef., Landw. Fl.: 132. 1866. Type. Based on Capsicumcordiforme Mill.
Capsicum annuum L. var. angulosum (Mill.) Alef., Landw. Fl.: 132. 1866, as ‘*angulatum*’. Type. Based on Capsicumangulosum Mill.
Capsicum annuum L. var. pyramidale (Mill.) Alef., Landw. Fl.: 132. 1866. Type. Based on Capsicumpyramidale Mill.
Capsicum annuum L. var. globiferum (G.Mey.) Alef., Landw. Fl.: 132. 1866. Type. Based on Capsicumglobiferum G.Mey.
Capsicum annuum L. var. longum (DC.) Alef., Landw. Fl.: 132. 1866. Type. Based on Capsicumlongum DC.
Capsicum annuum L. var. tetragonum (Mill.) Alef., Landw. Fl.: 133. 1866. Type. Based on Capsicumtetragonum Mill.
Capsicum annuum L. var. tetragonum (Mill.) Alef., Landw. Fl.: 133. 1866. Type. Based on Capsicumtetragonum Mill.
Capsicum annuum L. var. purpureum (Roxb.) Alef., Landw. Fl.: 134. 1866. Type. Based on Capsicumpurpureum Roxb.
Capsicum annuum L. var. ceratocarpum (Fingerh.) Alef., Landw. Fl.: 134. 1866. Type. Capsicumceratocarpum Fingerh.
Capsicum annuum L. var. bicolor (Jacq.) Alef., Landw. Fl.: 134. 1866. Type. Capsicumbicolor Jacq.
Capsicum fasciculatum Sturtev., Bull. Torrey Bot. Club 15(5): 133. 1888. Type. No locality cited (no specimens cited; lectotype, designated here [illustration]: “Tenjikumamori, Capsicumannuum L. (Solaneae)”, Tanaka & Motoyoshi, Sô-Mokou-Zoussets, vol. 3, Tab. 38. 1874).
Capsicum annuum L. var. longum (DC.) Kuntze, Revis. Gen. Pl. 2: 449. 1891. Type. Based on Capsicumlongum DC.
Capsicum annuum L. var. erectum Kuntze, Revis. Gen. Pl. 2: 449. 1891. Type. “Java, cult.” (no specimens cited, no original material located).
Capsicum annuum L. var. grossum (L.) Kuntze, Revis. Gen. Pl. 2: 449. 1891. Type. Based on Capsicumgrossum L.
Capsicum annuum L. var. fasciculatum (Sturtev.) Irish, Rep. (Annual) Missouri Bot. Gard. 9: 68, pl. 9, f. 4. 1898. Type. Based on Capsicumfasciculatum Sturtev.
Capsicum frutescens L. var. lanicaule Greenm., Proc. Amer. Acad. Arts 39: 88. 1903. Type. Mexico. Jalisco: along Ave. Vallarta in Ciudad Granja, on the western outskirts of Guadalajara, 31 Dec 1886, *E. Palmer 639* (lectotype, designated here: US [00816554, acc. # 92534], isolectotype: BM [BM000775827]).
Capsicum velutinum De Wild., Pl. Bequaert. 1: 413. 1922. Type. [Democratic Republic of the Congo]. Basankusu, Mar 1913, *O. Lamboray 22* (lectotype, designated here: BR [BR000000649909]).
Capsicum frutescens L. var. fasciculatum (Sturtev.) L.H.Bailey, Gentes Herbarum 1: 129. 1923. Type. Based on Capsicumfasciculatum Sturtev.
Capsicum frutescens L. var. grossum (Willd.) L.H.Bailey, Gentes Herbarum 1: 129. 1923. Type. Based on Capsicumgrossum Willd.
Capsicum annuum L. forma erectum Makino, J. Jap. Bot. 3(8): 29. 1926, as “Capsicumannuumvar.fasciculatumf.erectum”. Type. “Hab. JAPAN, cultivated” (no specimens cited, no original material located).
Capsicum annuum L. forma pendulum Makino, J. Jap. Bot. 3(8): 29. 1926, as “Capsicumannuumvar.fasciculatumf.pendulum”. Type. “Hab. JAPAN, cultivated, rare” (no specimens cited, no original material located).
Capsicum petenense Standl., Publ. Carnegie Inst. Wash. 461(4): 84. 1935. Type. Guatemala. Distr. Peten, La Libertad, Jun 1933, *C. L. Lundell 3754* (holotype: F [v0072800F, acc. # 685329]; isotypes: CORD [CORD00101764 fragment ex MICH], MICH [1109873]).
Capsicum sonitpurense J.Sarma & G.Dutta, Bangladesh J. Pl. Taxon. 24(2): 215. 2017. Type. India. Assam, Sonitpur, Tezpur, 49 m, 22 Oct 2016, *J. Sarma & G. Dutta 394* (holotype: ASSAM [acc. # 95893, sheet 394A]; isotypes: TUH [Tezpur University Herbarium, 3 sheets 394 B, C, D]).

#### Description.

Annual herbs or short-lived, compact, low subshrubs, 1–1.5 m tall, the main stem 0.5–1 cm in diameter at base, branched from near the base. Young stems 3–4-angled, fragile, green to brownish-green, sometimes with purple lines, glabrous, glabrescent to moderately pubescent, rarely densely pubescent, with appressed-antrorse, simple, uniseriate, (5–) 8–13)-celled, eglandular trichomes 0.5–1 (–2) mm long; nodes green or with purple spots; bark of older stems light brown or brown, glabrous to sparsely pubescent; lenticels absent or few. Sympodial units difoliate, the leaves geminate; leaf pair similar in size and shape. Leaves membranous, concolorous, pale to dark green, glabrous to moderately pubescent on both sides, especially on the main veins abaxially, the trichomes similar to those of the stems; blades of all leaves 3–7 (–15.5) cm long, 2.5–5 (–8) cm wide, ovate to elliptic, the major veins (3–) 5–8 on each side of mid-vein, the base truncate to cordate or cuneate to attenuate, the margins entire, the apex acuminate or long-acuminate; petioles (0.5–) 4–7 (–10) cm, with the same pubescence as the stems. Inflorescences axillary, 1 (– 2) flowers per axil, rarely more; flowering pedicels (6–) 10–40 mm long, angled, erect and geniculate at anthesis or pendent and non-geniculate, green or purple, glabrous to moderately pubescent, the eglandular trichomes usually short, antrorse; pedicels scars inconspicuous. Buds globose, white or purple. Flowers 5–7-merous. Calyx 1–4 mm long, 3–5 mm wide, cup-shaped, green, strongly 5–10-nerved, glabrous to moderately pubescent with similar short or long eglandular trichomes as the stems, the calyx appendages usually 5 (–7), minute, 0.3–0.5 mm long. Corolla 8–15 mm long, (8–) 10–22 mm in diameter, entirely white, rarely entirely pale yellow or purple, stellate with narrow interpetalar membrane, lobed ca. halfway or 2/3 of the way to the base, glabrous adaxially and abaxially, the tube 3–8 mm long, the lobes 5–7 mm long, 3.5–5.5 mm wide, ovate, spreading, the margins finely ciliate, the tips acute, papillate. Stamens 5–7, equal; filaments 1–3 mm long, white or cream, sometimes purple, inserted on the corolla 1–1.5 mm from the base, with auricles fused to the corolla tube at the point of insertion; anthers 2–3 mm, ellipsoid or ovoid, pale blue to purplish, very rarely yellow, connivent or not connivent at anthesis. Gynoecium with ovary 1.5–3 mm long, 1.2–2.5 mm in diameter, ovoid or globose, green; nectary ca. 0.5 mm tall, pale green; style heteromorphic, short style 2.2–2.5 mm, not exceeding the anthers, medium style nearly the same height as the anthers, long style 3–5.1 mm, exserted 1.3–2.3 mm beyond the anthers, cylindrical, white or purple; stigma 0.1–0.2 mm long, ca. 0.4 mm wide, discoid or capitate, pale green or yellow. Berry highly variable in shape, size and colour, usually blocky or elongate, less commonly globose, up to 300 mm long, 6–65 mm in diameter, green, yellow or purple when immature, yellow, red, brown, purple or purple-black at maturity, persistent, pungent or non-pungent, the pericarp thick, opaque, with giant cells (endocarp alveolate); stone cells absent; fruiting pedicels 25–50 (–70) mm, erect or pendent, rigid, angled, uniformly widened, green; fruiting calyx 15–25 mm in diameter, slightly accrescent, discoid or rather cup-shaped, green. Seeds more than 50 per fruit, 3.8–4.4 mm long, 3.2–3.6 mm wide, C-shaped, pale yellow, the seed coat smooth to slightly reticulate (SM), cerebelloid (SEM), the cells irregular in shape, the lateral walls sinuate; embryo imbricate.

**Figure 20.**
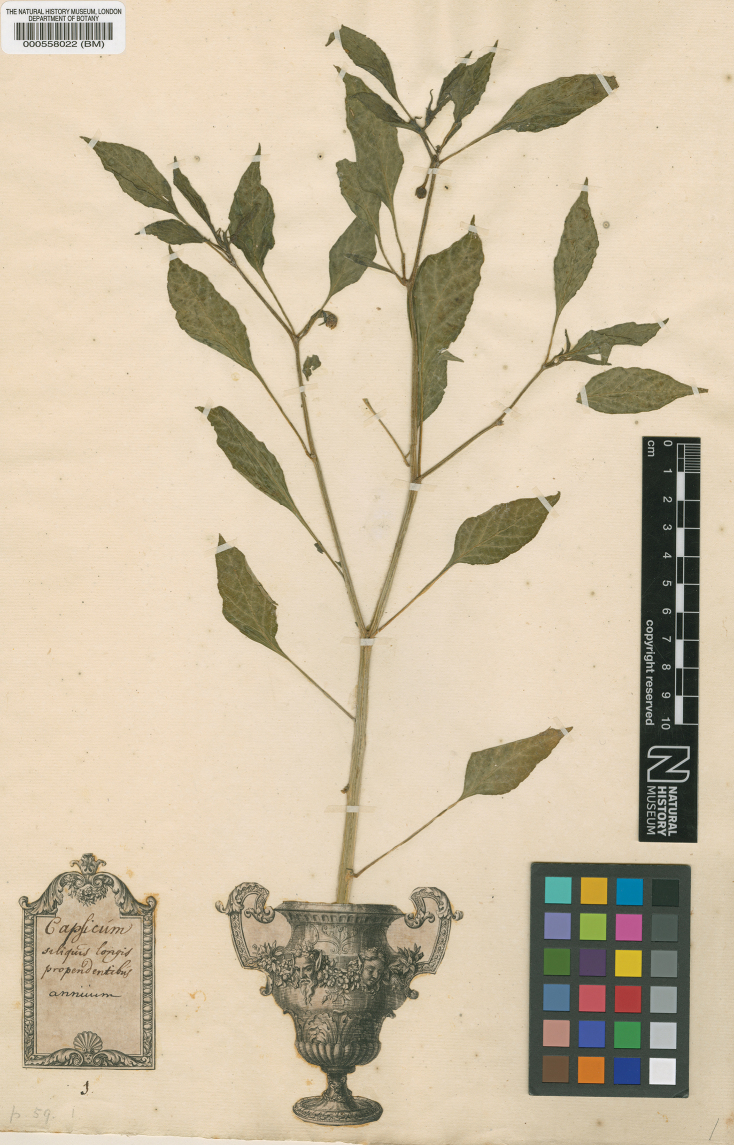
Capsicumannuumvar.annum. Lectotype (BM). Copyright The Trustees of the Natural History Museum, London. Reproduced with permission.

**Figure 21.**
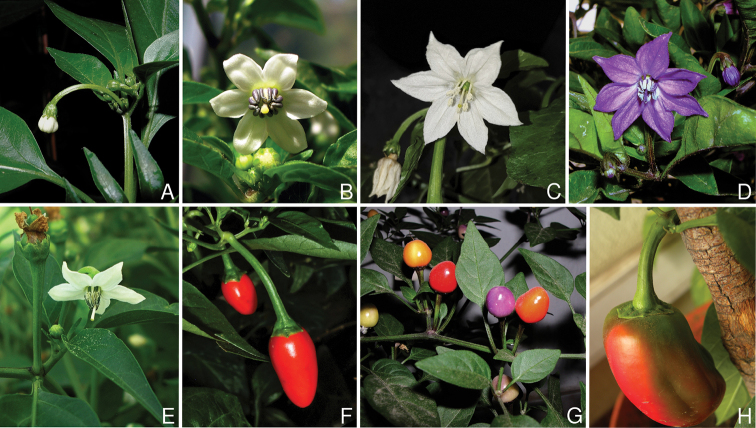
Capsicumannuumvar.annuum**A** flower bud on pendent pedicel **B** flower with connivent anthers **C** flower with hexamerous corolla (note nectar droplets on the limb) and style near the same length as the anthers **D** flower with heptamerous purple corolla **E** flower with pentamerous corolla and style exceeding the anthers **F, H** mature fruits on pendent pedicels **G** mature fruits on upright pedicels **A–H** no specimen vouchers, photos by G.E. Barboza and C. Carrizo García taken at different greenhouses.

#### Distribution.

Capsicumannuumvar.annuum is the most extensively cultivated pepper worldwide.

#### Ecology.

Capsicumannuumvar.annuum is found in diverse habitats throughout its wide distribution and is well adapted to the highlands environments (0–2,600 m elevation).

#### Phenology.

Flowering and fruiting all year.

#### Chromosome number.

*2n* = 2x = 24 (Pickersgill 1971, 1977, 1991; Moscone et al. 2007).

#### Common names.

**Argentina**: Ají balita (Jujuy, *Moscone 204*), Pimiento (Corrientes, *Anzótegui & Benitez 237*; Salta, *Hunziker 25498*), Serrano (Salta, *Hunziker 25492*), Pimiento Calahorra (Córdoba, *Hunziker 29428*); **Bolivia**: Ají (Beni, *Balderrama 10*; Santa Cruz, *Saldías P. 759*), Urubibi (Beni, *Ticona & Saravia May 10*), Pimentón colorado (Santa Cruz, Libreros et al. 2014), **Brazil**: Pimentão (Roraima, Barbosa et al. 2006; São Paulo, *Duth s.n.*), Pimenta-americana, Pimentão-vermelho, Pimentão-indigena, Pimenta-de-mesa, Pimenta-ornamental, Pimentão-bola, Pimenta-doce (Roraima, Barbosa et al. 2006), Pimenta-jalapeño, Pimenta-cayenne, Pimenta-serrano (Carvalho et al. 2006); **Chile**: Ají (Concepción, *Junge 5755*); **Colombia**: Ají (Amazonas, *Cordero P. 683*; Chocó, *La Rotta & Martínez 737*; Guainía, *Espina et al. 189*; Vichada, *Rodríguez 165*), Pimentón (Amazonas, *Torres & Morales 139*; Caquetá, *Cárdenas et al. 9310*; Cundinamarca, *Correa N. 006*), Ají amarillo (Caquetá, *Cárdenas et al. 9375*, Vichada, *Rodríguez 1*), Ají bravo (Amazonas, *Posada 2580*), Ají dulce (Amazonas, *Cárdenas et al. 9438*; Cundinamarca, *Duque-Jaramillo 3555*), Ají grande (Amazonas, *Cárdenas et al. 9432*), Ají pajaa (Vaupes, *Rodríguez 86*), Ají picante (Amazonas, *Posada 2581*), Ají pimentón (Amazonas, *Torres et al. 4011*), Ají yunga (Nariño, *de Benavides 4673*), Ají yuquitania (Vaupes, *Plowman 11986*), Pimentón dulce (Caquetá, *Cárdenas et al. 9360*), Pimentón quisquis (Meta, *Álvarez & Montañez 1*), Ají de agua (Vaupes, *Rodríguez 102*), Ají de blanco (Amazonas, *Henao & Kuiru 172*), Ají de curripaco (Vaupés, *Rodríguez 105*), Ají de gente (Amazonas, *Cárdenas et al. 9424*), Ají mas picante (Vaupes, *Rodríguez 83*), Diente de chucha (Caquetá, *Cárdenas et al. 9319*), Pipí de perro (Caquetá, *Cárdenas et al. 9306*), Ají largo de blanco (Amazonas, *Henao 316*), **Ecuador**: Ají (Chimborazo, *Lara s.n.*; Pichincha, *Mejía 001*), Pimiento (Guayas, *Bonifaz & Cornejo 4158*; Pichincha, *Narváez 018*), Ají colorado (Tungurahua, *Acosta Solís 8848*), Ají manzana (Guayas, *Valverde 392*), Ají patateño (Chimborazo, *Ganchozo 007*), Manzanita de Eva (Guayas, *Valverde 35*); **El Salvador**: Chile de relleno (San Salvador, *Calderón* 523); **Guatemala**: Chile (Quezaltenango, *Steyermark 34462*); **Honduras**: Chile fuerte (Morazán, *Molina R. & Molina 34535*), Chile picante (Copán, *Molina R. & Molina 33572*; Morazán, *Molina R. 34008*), Chile de gallina (Cortes, *Chevez 40*); **Mexico**: Chile (Chiapas, *Calzada et al. 3774*; Michoacán, *Miranda et al. 1519B*; Tamaulipas, *Rodríguez & Lira 63*; Veracruz, *Baizabal & Zola B. 12*), Picante (Veracruz, *Baizabal & Zola B. 11*), Pimentón (Michoacán, *Bye et al. QD 246*), Chile ancho (Guanajuato, *Benítez 689*; Tamaulipas, *Rodríguez & Lira 63b*), Chile bolita (Zacatecas, *Benítez 738*), Chile camote (Tamaulipas, *Hernández 1944*), Chile cimarrón (México, *Hinton 4336*), Chile chaua (Yucatán, *Simá 610*), Chile cora (Zacatecas, *Benítez 734*), Chile costeño (Campeche, *Ramírez A. 56*), Chile chilaca (Guanajuato, *Benítez et al. 386*), Chile delgado (Quintana Roo, *Gutiérrez 85-27*), Chile dulce (Michoacán, *Bye et al. 90*; Tabasco, *Ortíz 01*; Yucatán, *Simá 606*), Chile gordo (Oaxaca, *Hernández Ortega 484*; Veracruz, *Vázquez 673*), Chile guajon (Zacatecas, *Benítez 724*), Chile kat (Yucatán, *Ucan et al. 3519*), Chile largo (Quintana Roo, *Gutiérrez 85-41*), Chile pableño (Guanajuato, *Vieyra s.n.*), Chile pasilla (Tamaulipas, *Rodríguez & Lira 63ª*), Chile pimienta (Mexico, *Rodin 40*), Chile pimiento (Mexico, *Linares 846*), Chile serrano (Hidalgo, *Villa 71*; Oaxaca, *Martínez Calderón 1643*; Veracruz, *Zola & Baizabal 1439*), Chile uñepicho (Veracruz, *Díaz Rico 48*), Chile verde, chile de huerta (Michoacán, *Soto Núñez et al. 5442*), Chile xalapeño, *Calzada 2367*), Fruto azul (Guerrero, *Díaz Rico 221*), Pimiento grande (Oaxaca, *Bamonte 77*), Chile de agua (Oaxaca, *Acosta Castellanos 9417*), Chile de árbol (Michoacán, *Soto N. 14302*), Chile de vida o chilar (Oaxaca, *Moreno 29*), Chile mira parba (Tabasco, *Ortíz 07*), Chile pico paloma (Tabasco, *Ortíz 20*), Chile de árbol de bola (Michoacán, *Soto Núñez 14047*), Chile de uña de perro (Veracruz, *Vázquez 998*); **Nicaragua**: Chile (León, *Guzmán et al. 1011*); **Panamá**: Ají (Canal Zone, *Standley 28523*), Sweet pepper, pimiento morrón, ají (Canal Zone, *Standley 29880*); **Peru**: Encarnado (Lima, *Velarde Nuñez 20*), Pimiento (Lima, *Vilcapoma S. 84*), Aji amarillo (Lima, *Vilcapoma S. 85*), Ají cerezo (Lambayeque, Libreros et al. 2013), Ají dulce (Loreto, *Hormia 2228*), Ají limón (Trujillo, *Plowman 14541*), Ají tomate (Junín, *Ridoutt 11714*), Bobo panca (Lima, *Velarde Nuñez 19*), Cerezo triangular (Lambayeque, Libreros et al. 2013), Conico amarillo (Lima, *Velarde Nuñez 26*), Cónico panca (Lima, *Velarde Nuñez 18*), Tambo Tacna (Lima, *Velarde Nuñez 22*), Aji Acarí Moquegua (Lima, *Velarde Nuñez 7*); **United States of America**: Black Chile or Chile negro (New Mexico, *Wooton 48*), Red Chile or Chile rojo (New Mexico, *Wooton 49*). **Venezuela**: Ají caribe (Portuguesa, *Aymard 5108*).

#### Indigenous names.

**Bolivia**: Ta (Beni, *Ticona & Saravia May 10*); **Colombia**: Aati (Curripaco, Guainía, *Espina et al. 189*), Aii (Cauca, *Plowman & Vaughan 5370*), Asi (Piapoco, Vichada, *Rodríguez 177*), Azi (Piapocos, Vichada, *Rodríguez 165*), Biaá (Tanimuka, Amazonas, *Cárdenas et al. 9406*), Coc (Puinabe, Vichada, *Rodríguez 169*), Curripaati (Tucano, Guainía, *Marín & Rodríguez 502*), Fecogɨ (Bora, Amazonas, *Torres et al. 4020*), Fekorai (Huitoto-Mɨnɨka, Amazonas, *Henao* 167), Jipujou (Caquetá, *Cárdenas et al. 9330*), Jumerien (Sukuare, Vichada, *Rodríguez 1*), Mèe (Colona, Amazonas, *Torres & Rodríguez 2021*), Munɨ (Huitoto, Amazonas, *Posada 2577*), Nubata (Andoque, Amazonas, *Torres et al. 4047*), Pidá (Emberá, Chocó, *La Rotta & Martínez 737*), Rɨairai (Huitoto-Mɨnɨka, Amazonas, *Henao & Kuiru 172*), Arera rɨairai (Huitoto-Mɨnɨka, Amazonas, *Henao 316*), Yicane (Miraña, Caquetá, *Cárdenas et al. 9375*), Jeba gayebá (Mui, *Castro et al. 238*), Masan via (Amazonas, *Cárdenas et al. 9424*), Pipita deé (Mui, Amazonas, *Castro 305*), Viahoracá carunoje (Tanimuka, Amazonas, *Cárdenas et al. 9432*); **Ecuador**: Aatyu (Chapalaachi, *Yañez et al. 1485*), Uchu (Quichua, Napo, *Kohn 1225*), Ahí bia (Siona & Secoya Indians, Napo, *Vickers 211*), Suara pia (Siona & Secoya Indians, Napo, *Vickers 227*), Soa horo bia (Siona & Secoya Indians, Napo, *Vickers 200*); **Mexico**: Cants (Huave, Oaxaca, *Zizumbo & Colunga 145*), Chaunik (Yucatán, *Vargas 66*), Guiin-cànár (Zapateco, Oaxaca, *Hunn OAX-1345*), Guiin-ló-yág (Zapateco, Oaxaca, *Hunn OAX-1341*), Guiin-ló-ngÚbidz (Zapateco, Oaxaca, *Hunn OAX-1343*), Guiin-nàl-zhàb (Zapateco, Oaxaca, *Hunn OAX-1342*), Guiin-txxtlé (Zapoteco, Oaxaca, *Hunn OAX-1344*), Moo-o-re (Oaxaca, *Hernández Ortega 482*), Moo-o-qui (Oaxaca, *Hernández Ortega 481*), Niiy (Oaxaca, *Antonio B. GUI 201*), Xcatic (Maya, Quintana Roo, *Villanueva 591*), X-mash ik (Quintana Roo, *Gutiérrez 85-26*), X-mehen (Quintana Roo, *Gutiérrez 85-27*), Xkat-ik (Maya, Quintana Roo, *Gutiérrez 26*), Ya Jimia (Morona-Santiago, *Evans 4384*), Ya’axik (Maya, Quintana Roo, *Gutiérrez 109*), Chaua ik (Yucatán, *May 39*), Ixa nadun (Guerrero, *Wagenbreth 130*), Kat ik (Yucatán, *Ucan et al. 3529*), Nadam kanc (Huave, Oaxaca, *Bamonte 77*), Namis kanc (Huave, Oaxaca, *Bamonte 79*), Yaá dia (Mixteco, Guerrero, *Díaz Rico 221*), Yak ik (Quintana Roo, *Gutiérrez 85-71*), Ixe dun xkuiya smidi (Guerrero, *Wagenbreth 687*); **Peru**: Iwiá (Mayna Jívaro, Loreto, *Lewis et al. 10922*), Kistian jima (Amazonas, *Ancuash 297*), Mun hima (Amazonas, *Berlín 1572*), Tsitikana ogat-santsakarioni (Machiguenga, Cuzco, *Johnson 70*).

#### Uses.

Capsicumannuumvar.annuum is the economically most important member of the genus. The fruits are widely used in international cuisine in a broad spectrum of meals and preparations, because of their aroma, flavour, texture and level of pungency. Some cultivars have good acceptance as ornamental plants due to the colour of the leaves and the brightness of the colourful and usually erect fruits (e.g. Christmas peppers, Bolivian rainbow, Fig. 21). There are few instances where medicinal uses have been recorded on herbarium labels (Table 3), but the medical and nutritional importance, as well as the pharmacological properties and therapeutic effects of the active compounds, of *C.annuum* fruits have been extensively highlighted (Al-Snafi 2015; Srinivasan 2016; Masud Parvez 2017; Sricharoen et al. 2017; Saleh et al. 2018; Sanati et al. 2018; Roman et al. 2020).

#### Preliminary conservation assessment.

Capsicumannuumvar.annuum is not under threat.

#### Discussion.

The domesticated taxon C.annuumvar.annuum belongs to the Annuum clade, together with *C.chinense*, *C.frutescens* and *C.galapagoense* (Carrizo García et al. 2016). The three domesticated species and their conspecific wild populations constitute the *Capsicumannuum* primary gene pool (van Zonneveld et al. 2015).

The vast majority of the modern landraces, varietals and hybrids of chili peppers belong to this variety (Bosland and Votava 2000) and it is consequently the most intensively studied species of *Capsicum* with regard to diversity, domestication and genetics (OECD 2006; Pickersgill 2016; Acquadro et al. 2020 and references therein). Conversely, a full comprehension of its taxonomy has not been achieved in the last 50 years. Research indicates that its domestication could have been initiated in central-east Mexico over 6,500 years ago (Kraft et al. 2014).

Due to the selective pressure for domestication and diversification, defining a characteristic group of traits for var. annuum is difficult; however, the most distinctive features are its herbaceous to shrubby, annual or perennial habit, the solitary axillary flowers (rarely two or more), the strongly 5–10-nerved calyx, the large white (or purple) corollas (up to nearly 22 mm in diameter) and the usually persistent and pendent fruits, which are highly variable in size, form, colour and pungency. Some of these traits contrast with those of var. glabriusculum which has a shrubby habit, 5-nerved calyx, smaller corollas (≤ 12 mm in diameter) and small (< 10 mm in diameter), globose, ellipsoid or ovoid, erect, red or red-orange, deciduous fruits.

Philip Miller was the curator of the Chelsea Physic Garden in London in the late 18^th^ century. Many of the plants he grew there were new taxa in his “Gardener’s Dictionary” (1768). He described several *Capsicum* species (*C.cordiforme*, *C.tetragonum*, *C.angulosum*, *C.olivaeforme* and *C.pyramidale*), based on cultivated specimens obtained from seeds of different provenance. As was the practice at the time, he did not cite specimens and is likely to have based his descriptions on living plants. Most of these plants were described as annuals with white flowers and a variety of fruit sizes, shapes (heart-shaped, angular-obtuse, oval-shaped, pyramidal), colours (yellow, scarlet, red), textures and positions (pendent or upright), characters that are highly variable due to human selection in these domesticated species. Specimens made from plants grown by Miller are found in several different places, mostly at BM and its associated historical herbaria. As these names are almost certainly described from living plants and, thus, will need neotypification, we do not typify them here, but leave that for a separate study when these materials, including any non-digitised specimens, can be studied in detail.

We found a collection in the Lamarck Herbarium with a label indicating that it belongs to *C.conicum* (P00357734) which we designate here as the lectotype.

*Capsicumbicolor* was probably described only from living material cultivated in the gardens of Schönbrunn Palace near Vienna (Austria). Jacquin (1809) cited no specimens for *C.bicolor* and gave no place of origin for his species. The description is quite complete and includes a colourful illustration (Tab. 99, fig. 1); both fall within our circumscription of *C.annuum*. Since no specimens have been found, we designate the illustration here as the lectotype.

There are two sheets of original material labelled *C.grossum* in Willdenow’s Herbarium held at Berlin. Both contain reproductive branches; one of these (B-W 04425 -02 0) consists of two fruiting branches that exactly match Willdenow’s description (Willdenow 1809) and is, therefore, designated the lectotype here.

Willdenow (1809) cited neither specimen nor locality when describing *C.sphaericum*. We found at B original material labelled “C.sphaericum, Hort. Bot. Berol. W” (B-W04426-01-0) and select it here as the lectotype.

In the protologue of *C.purpureum*, Hornemann (1813) stated “Herb. Vahlii … Hab. - -”, referring to material cultivated at Horto Hafniensis (Hafnia = Haunia = Copenhagen). A specimen held in C [C10019148], with good flowering material and bearing a label (on the verso of the sheet) with data matching the protologue, is here selected as the lectotype. Additional original material is found at C (C10019147, upper stem only), which corresponds to a Herb. Hornemann specimen cultivated in Copenhagen; this specimen is sterile.

De Candolle (1813) described *C.ovatum*, based on a living specimen of unknown origin cultivated at the Montpellier Botanical Garden (hort Bot. Monspeliensis), but he cited no herbarium material. We found no original material at MPU; Dunal (1852) expanded the description in the *Prodromus* with specimens seen in the De Candolle Herbarium, now held in G-DC. At G-DC, there are two elements: a small fruiting fragment possibly from Montpellier (“h.m.” [my herbarium] on the label) and a more complete specimen with a label stating “*Capsicumovatum* D.C.” in Dunal’s hand; we designate here this latter specimen (G00200072) as the neotype.

When coining the name *C.longum*, De Candolle (1813) cited in synonymy pre-Linnean works, some with illustrations. We examined the illustrations and the one from J. Bauhin et al. (1651) best illustrates and corresponds to the original diagnosis and, therefore, is selected here as the lectotype.

Meyer (1818) based his description of *C.globiferum* on collections made by Ernst Karl Rodschied in what is now Guyana. In Göttingen, where Meyer worked, we found two Rodschied specimens labelled as *C.globiferum*, both from Rio Essequibo, that are certainly original material. One (GOET003420) has what appears to be a collecting number, *Rodschied 29*, while the other lacks any indication of a number (GOET003419). We designate here the former and most complete specimen (*Rodschied 29*) as the lectotype.

*Capsicumpurpureum* is based on a single plant found in the Botanic Garden of Calcutta (India), whose exact origin is unknown, but Roxburgh (1824) suggested the seeds came from the “Molucca Islands”. A specimen at Kew (K001132446), labelled as “Capsicumpurpureum” and with a faint annotation of “H.B.C.” at the bottom of the sheet, is possible original material, but we cannot be sure it was used by Roxburgh or when it was prepared. We, therefore, designate this sheet as the neotype of *Capsicumpurpureum*.

Dierbach (1829) referred to his C.indicumvar.vulgatum as “Capsicumannuum Auctor. plurimor.” making this name superfluous. His species was characterised by its red, oblong and straight fruits, one of the most common forms of the domesticated *C.annuum*.

Fingerhuth (1832) described many domesticated chili pepper taxa (see above); he did not mention specimens and, if he made specimens, the fate of his herbarium is unknown (Stafleu and Cowan 1976). Fingerhuth provided a set of ten plates, each one with figures in colour that illustrated almost all of the taxa included in his monograph. These figures are of good quality and match the protologues, which allowed us to assign the species to which they belong and to use them as lectotypes for Fingerhuth’s names. The names *Capsicumstrictum*, *C.ceratocarpum*, *C.angulosum* (var. macrocarpum and *ovale*), some varieties of *C.annuum* (var. rugosulum, *acuminatum*, *subangulosum*, *ovoideum*, *abbreviatum*, *olivaeforme*), *C.grossum* (var. pomiforme, *ovatum*, *cordatum*, *angulosum*) and *C.longum* (var. incrassatum, *latum*, *rectum*) are here lectotypified, based on the Fingherhuth figures indicated above.

In the protologue of *C.abyssinicum*, Richard (1850) cited two collections from “Abyssinia” (Ethiopia), one made by León Richard Quartin Dillon and the other by Antoine Petit, botanists on the Lefebvre expedition to the mountains of Africa. Quartin Dillon’s collection is housed at P and consists of three sheets (P00329903, P00329904, P00329905), all of them with complete flowering and fruiting branches. We were unable to find the second collection at P, but a duplicate of the original Petit collection is at MEL (MEL 2442182) and is also a well-preserved specimen. We designate here the best-preserved collection and that which Richard is likely to have seen and used (P00329903) as the lectotype.

Dunal (1852) coined C.annuumvar.oblongum with a direct citation to a polynomial and illustration in “Fingerh. l.c. t. 2 f.a” (Fingerhuth 1832); he also stated “v.s. in h. DC”. A sheet in G-DC (G00131768) is labelled “Capsicumannuum α oblongum fructibus rubris Fingerh.” from “Herb. Dunal 1844”, both in Dunal’s hand; we select this specimen as the lectotype.

In his description of C.pyramidalevar.longicorne, Dunal (1852) cited the collection *Zollinger 489* that he had seen in “h. Boiss. et h. DC.” (now G and G-DC). Both specimens consist of fertile branches, but that in G-DC (G00131841) has an immature fruit which confirms the identity of this name and is selected here as the lectotype.

In the protologue of *Capsicumtesticulatum*, Dunal (1852) stated “v.s. in h. Dc. et herb. meo”. The original material came from plants grown in the Botanical Garden in Montpellier from seeds sent by R. de Visiani. The sheet in G-DC (G00200067) is a more complete fruiting branch which we select here as the lectotype.

In the protologue of C.angulosumvar.macrocarpum, Dunal (1852) cited no specimens, but referred his variety to the illustration “tab. 8, fig. a” provided by Fingerhuth (1832) which is selected as the lectotype.

Dunal based *C.leucocarpum* on Miller’s (1752) polynomial “Capsicum americanum latifolium, fructu oblongo erecto candido”, referring to a sort of white-coloured *C.annuum* fruit; Fingerhuth (1832) also transcribed Miller’s polynomial exactly, but he did not provide a formal name, as appears in Index Kewensis (*Capsicumleucocarpon* Fingerh., accessed on 20 April 2020) or IPNI (*Capsicumleucocarpon* Mill. ex Fingerh. (accessed on 20 April 2020).

Sturtevant (1888b) described *C.fasciculatum*, based on his own cultivated living material known as Bouquet rouge (French garden name) or Red Cluster (American name). He distinguished this species from *C.annuum* primarily by its peculiarly clustered leaves and fruits at the summit of the plant. We found no herbarium material corresponding to this name, but Sturtevant stated in the protologue that *C.fasciculatum* is “well figured under the name *Tenjikumamori* in a Japanese botanical work…” referring to Tanaka and Ono (1874). The figure cited by Sturtevant (1888b) is very accurate and represents very well one of the many variations of the domesticated *C.annuum* and is here selected as the lectotype.

Greenman (1903) based C.frutescensvar.lanicaule on four syntypes and characterised it as a more pubescent variant of *C.frutescens*. We studied all the syntypes (*Palmer 639, 640, 642* and *González 975*). Palmer’s collections are flowering and fruiting branches, while González’s specimen has flowers and very young fruits. The fruiting calyx is critical in assigning the correct placement of this name and, amongst the fruiting Palmer specimens, the most informative one is *Palmer 639* which has mature fruits typical of C.annuumvar.annuum and is designated here as the lectotype.

*Capsicumvelutinum* (De Wildeman 1922) was described based on five syntypes from different localities and collectors in what is now the Democratic Republic of the Congo, all of them well preserved at BR (*Blommaert s.n.*, *Jespersen s.n*., *Lamboray 22* and *Lescrauwaet 315*). All the specimens consist of strikingly pubescent flowering or fruiting branches or both. We designate *Lamboray 22* (BR 000000649909) as the lectotype for this name, since it matches the protologue most closely.

### ﻿1b. Capsicum annuum L. var. glabriusculum

Taxon classificationPlantaeSolanalesSolanaceae

(Dunal) Heiser & Pickersgill, Baileya 19 (4): 156. 1975.

DAF42A7A-D34A-5D65-BCA6-3BAAF56DBAD5

Figs 22 , 23

Capsicum hispidum Dunal var. glabriusculum Dunal, Prodr. [A. P. de Candolle] 13(1): 420. 1852. Type. [United States of America. Texas: Bexar Co., San Antonio]: “Mexico, circa Bejar”, Sep 1828, *J.L. Berlandier 1863* (lectotype, designated by Barboza 2011, pg. 27: P [P00410138]; isolectotypes, BM [BM000775839], F [F0072795F, acc. # 680282], G [G00390278], NY [000138591], P [P00409852], YU [YU.065273]).
Capsicum minimum Mill., Gard. Dict. ed. 8, no. 10. 1768. Type. “Cultivated in England” (no specimens cited; no original material located).
Capsicum havanense Kunth, Nov. Gen. Sp. [H.B.K.] 3: 38. 1818. Type. [Cuba]. “in arenosis maritimis, prope Havanam (Insulae Cubae)” [Havana] s.d., *F.W.H.A. von Humboldt* & *A.J.A. Bonpland 4518* (lectotype, designated here: P [P00670653]).
Capsicum indicum Dierb. var. aviculare Dierb., Arch. Apotheker-Vereins Nördl. Teutschl. 30(1): 30. 1829. Type. Based on Capsicumminimum Mill. and C.microcarpon DC. (cited in synonymy), PANAMA: Coclé, 10 mi. E of Nata at Rio Grande, 4 Jan 1969, *E.L. Tyson 5222* (neotype, designated here: MO [MO-562584, acc. # 1980106]; isoneotype, FSU [000064909, acc. # 119808]).
Capsicum frutescens L. var. minus Fingerh., Monogr. Capsic.: 17. 1832. Type. “Crecit in India orientali et America meridionali” (no specimens cited; lectotype, designated here [illustration]: “Capsicumrubrumminimum” Rumphius, Herbarium Amboinense 5, Tab. 88, fig. 2, 1747]).
Capsicum pendulum Willd. var. minus Fingerh., Monogr. Capsic.: 25. 1832. Type. Based on Capsicumhavanense Kunth.
Capsicum chlorocladum Dunal, Prodr. [A. P. de Candolle] 13(1): 415. 1852. Type. Mexico. Tamaulipas: “Tampico da Tamaulipas”, 1827, *J.L. Berlandier 97* (lectotype, designated here: G-DC [G00131884]; isolectotypes: BM [BM000775807, BM000775821], G [G00342805], F [v0072794F, acc. # 680277], LE [LE01072484], MPU [MPU023049], P [P00410031, P00410147, P00409849]).
Capsicum laurifolium Dunal, Prodr. [A. P. de Candolle] 13(1): 418. 1852. Type. Brazil. Bahia: “partie mérid. de la prov. de Bahia”, 1840, *J.S. Blanchet 3098 A* (lectotype, designated here: G-DC [G00131882]; isolectotypes: MPU [MPU023046, MPU023047).
Capsicum hispidum Dunal, Prodr. [A. P. de Candolle] 13(1): 419. 1852. Type. Mexico. Tamaulipas: circa Tupan et Tampico de Tamaulipas, 1827, *J.L. Berlandier 152* (lectotype, designated here: G-DC [G00131880]; isolectotypes: BM [BM000775838], G [G00390276], G [G00390277], MO [MO-562486, acc. # 1690380], MPU [MPU013437], P [P00409850, P00410137]).
Capsicum angustifolium Dunal, Prodr. [A. P. de Candolle] 13(1): 420. 1852. Type. “In Indiâ utrâque colitur”. Capsicumbaccatum hort. Geneve, 1836, Anonymous 1414/6 (lectotype, designated here: G-DC [G00131878]).
Capsicum microphyllum Dunal, Prodr. [A. P. de Candolle] 13(1): 421. 1852. Type. [Cuba]: La Havanna, 1828, R. de la Sagra 3 (lectotype, designated by D’Arcy and Eshbaugh 1974, pg. 99: G-DC [G00131975]; isolectotype: MPU [MPU023042]).
Capsicum pendulum Willd. var. minus Dunal, Prodr. [A. P. de Candolle] 13(1): 425. 1852, nom. illeg., not C.pendulumvar.minus Fingerh. (1832). Type. Based on Capsicumhavanense Kunth.
Capsicum annuum L. var. minus (Fingerh.) Shinners, Baileya 4: 82. 1956. Type. Based on CapsicumfrutescensL.var.minus Fingerh.
Capsicum annuum L. var. minimum (Mill.) Heiser, Ci. & Nat. 7: 52. 1964. Type. Based on Capsicumminimum Mill.
Capsicum annuum L. var. aviculare (Dierb.) D’Arcy & Eshbaugh, Phytologia 25(6): 350. 1973. Type. Based on CapsicumindicumDierb.var.aviculare Dierb.
Capsicum frutescens L. var. glabriusculum (Dunal) M.R.Almeida, Fl. Maharashtra 3B: 356. 2001. Type. Based on CapsicumhispidumDunalvar.glabriusculum Dunal.

#### Type.

Based on CapsicumhispidumDunalvar.glabriusculum Dunal.

#### Description.

Perennial low herbs or somewhat prostrate subshrubs, (1–) 1.5–2 (–3) tall, the main stem woody, 0.5–1 cm in diameter at base, much branched from near the base, the branches dichotomously spreading in a typical “zig-zag” appearance above. Young stems angled, fragile, green to greenish-grey or purple-striped, glabrescent to densely pubescent, with appressed-antrorse to spreading, simple, uniseriate, (2–) 3–8 (–12)-celled, eglandular trichomes 0.3–0.9 (–2) mm long, rarely furcate trichomes; nodes solid, green or purple; bark of older stems light brown or brown, glabrous to sparsely pubescent; lenticels absent. Sympodial units difoliate, the leaves geminate; leaf pair subequal in size and shape. Leaves membranous, discolorous, dark green above, light green beneath, glabrescent to densely pubescent on both sides, if glabrescent with an evident tuft of trichomes in the vein axils beneath, the trichomes similar to those of the stems; blades of all leaves 2.5–6 (–8.5) cm long, 1.15–2.5 (–3.4) cm wide, ovate to elliptic, the major veins 4–5 on each side of mid-vein, the base attenuate or truncate and rather unequal, the margins entire, the apex acuminate; petioles (0.5–) 1.5–2.5 (–3) cm, glabrous to moderately pubescent. Inflorescences axillary, 1–2 flowers per axil, more rarely 3 flowers; flowering pedicels 7.5–27.8 mm long, angled, erect, geniculate at anthesis, green, glabrous to moderately pubescent, the eglandular trichomes short, antrorse; pedicels scars inconspicuous. Buds globose, white, cream or greenish-white. Flowers 5-merous. Calyx 1.5–2.5 (–3) mm long, 2–3.8 mm wide, cup-shaped, green, pentagonal in outline, glabrous to moderately pubescent with similar short or long eglandular trichomes as the stems, without appendages or with five minute appendages less than 0.5 mm long. Corolla (5–) 6–8 mm long, 8–10 (–12) mm in diameter, entirely white or almost pale yellow, rarely greenish-white, stellate with narrow interpetalar membrane, lobed ca. halfway or 2/3 of the way to the base, glabrous adaxially and abaxially, the tube (2–) 3–3.5 mm long, the lobes 3–4.5 mm long, 2–2.5 mm wide, triangular, spreading, the margins slightly involute and finely ciliate, the tips acute to long-cucullate, densely papillate. Stamens five, equal; filaments 1–1.25 mm long, white, cream or purple, sometimes lilac at the apex, inserted on the corolla 1–1.3 mm from the base, with auricles fused to the corolla tube at the point of insertion; anthers 0.95–2.55 mm, broadly ellipsoid or ellipsoid, blue, bluish-grey or purple, very rarely yellow, connivent at anthesis. Gynoecium with ovary 1.2–1.5 (–2.5) mm long, 1–2 mm in diameter, green or cream, ovoid or globose; nectary ca. 0.3 mm tall, pale yellow; style homomorphic, 4–4.8 mm, exserted 1.5–2 mm beyond the anthers, cylindrical, white or pale lilac; stigma 0.1–0.2 mm long, ca. 0.3 mm wide, discoid or bilobed, pale bright green or white. Berry 6–8.5 mm in diameter, globose (larger in semi-domesticated specimens, 9–11 mm in diameter) or ellipsoid or ovoid with acute to slightly obtuse apex, 9–13 mm long, 5–6.5 mm in diameter (larger in semi-domesticated specimens, 15–25 mm long, 7–12 mm in diameter), green or green and partly dark purple or purple when immature, bright lemon-yellow, bright red-orange or red at maturity, deciduous, very pungent, the pericarp thick, opaque, with giant cells (endocarp alveolate); stone cells absent; fruiting pedicels 16–28 (–35) mm, erect, rigid, angled, widened distally, green; fruiting calyx 4–4.5 mm in diameter, persistent, not accrescent, discoid or rather cup-shaped, green. Seeds (6–) 8–26 per fruit, 3.2–4 mm long, 2.5–3.2 mm wide, C- or D-shaped, pale yellow to yellow, the seed coat reticulate to obscurely reticulate (SM), cerebelloid (SEM), the cells irregular in shape, the lateral walls strongly sinuate; embryo imbricate.

**Figure 22.**
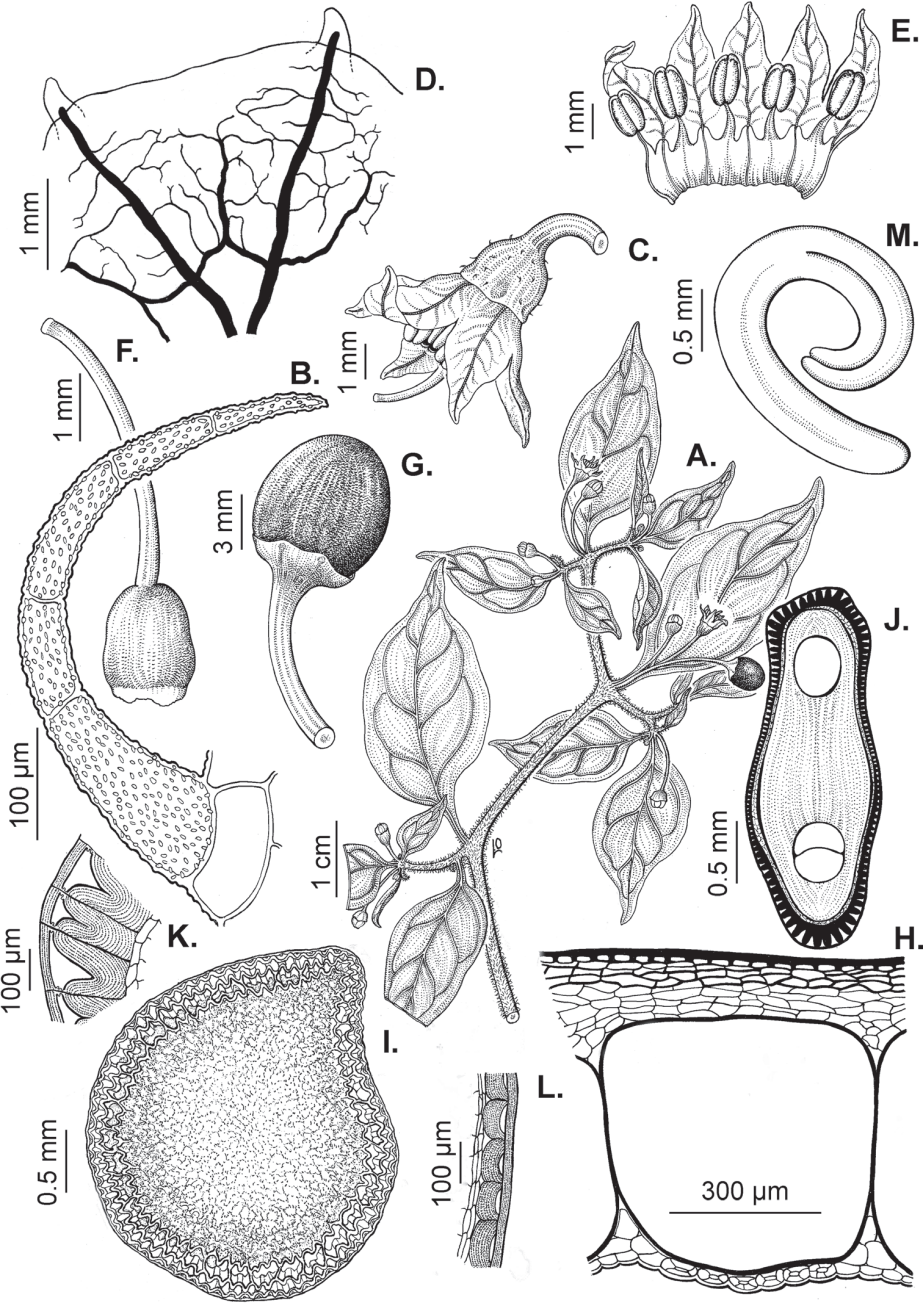
Capsicumannuumvar.glabriusculum**A** reproductive branch **B** eglandular trichome of the leaf **C** flower **D** section of the calyx showing the venation **E** opened corolla **F** gynoecium **G** fruit **H** anatomical detail of the pericarp (note the giant cell in the mesocarp) **I** seed **J** seed, in cross section **K** structure of seed coat at the seed margin **L** structure of seed coat at the seed body **M** embryo. **A–H** from *Singleton 195***I–M** from *Scolnik 19An329*. Drawn by L. Ochoa. Published in Hunziker (2001), reproduced with permission.

**Figure 23.**
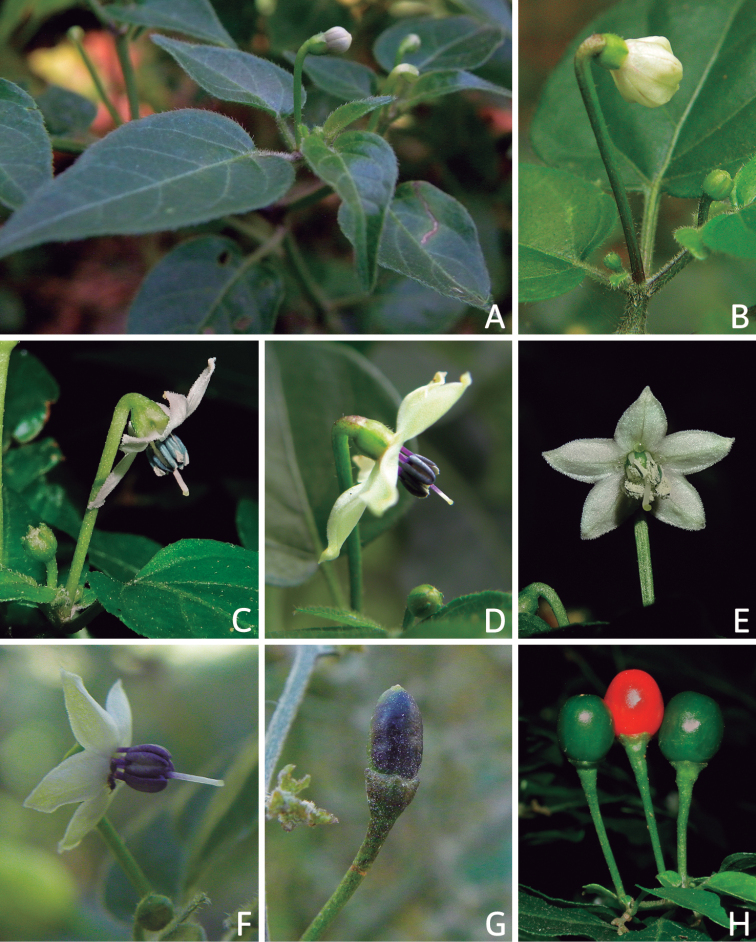
Capsicumannuumvar.glabriusculum**A** apex of a reproductive branch **B** flower bud on geniculate pedicel **C–F** flowers in anthesis showing variations in corolla, stamens and style colouration **G** immature fruit **H** mature and immature fruits **A, B, D, F** from *Barboza et al. 5049*, photos by G.E. Barboza **C, E, H***Carrizo García 102*, photos by C. Carrizo García **G***Leiva González et al. 2105*, photo by S. Leiva González.

#### Distribution.

**Figure 24.**
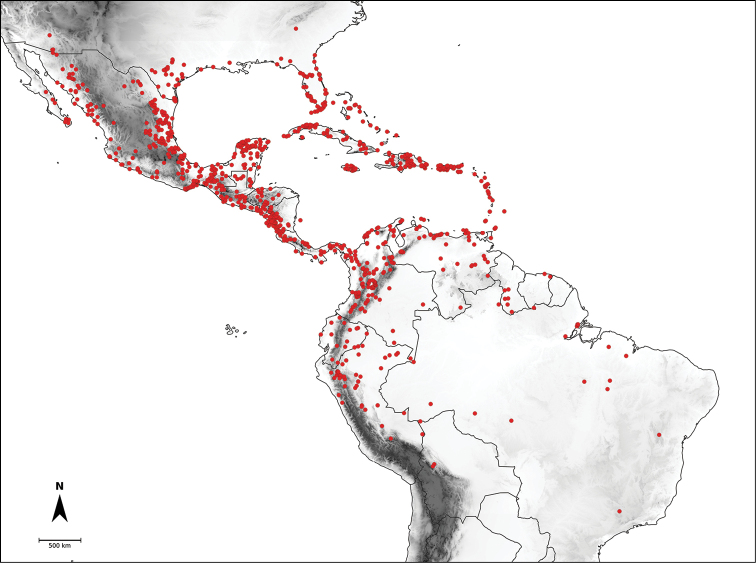
Distribution of C.annuumvar.glabriusculum.

Capsicumannuumvar.glabriusculum is the most widely distributed member of the genus, from southern United States of America to northern Bolivia and northern Brazil (Fig. 24). It is more common in Mexico, Central America, the Caribbean, Colombia and Venezuela. In eastern Australia, it is reported as a weed (Symon 1981; Purdie et al. 1982).

#### Ecology.

Capsicumannuumvar.glabriusculum occupies a wide variety of habitats throughout its wide distribution, including tropical deciduous, semi-deciduous and evergreen forests, less frequently in dry tropical or subtropical forests or in thorny scrub, from sea level to ca. 2,500 m elevation. It is found in shade along roadsides, stream banks, meadows near shores or as a weed in pastures or on the edges of cultivated lands. Indigenous communities and rural people cultivate C.annuumvar.glabriusculum for self-consumption and it is often found escaped from cultivation.

#### Phenology.

Flowering and fruiting all year.

#### Chromosome number.

*2n* = 2x = 24 (Pickersgill 1971, 1977, 1991; Moscone et al. 2007; Scaldaferro et al. 2013).

#### Common names.

**Bahamas**: Bird pepper (Long Island, *Richey 98-355*), Pepper bush (Bimini, *Howard & Howard 10055*); **Boliva**: Ají (Pando, *Beck et al. 19150*); **Brazil**: Pimenta-de-mesa, Pimenta-peito-de-moça, Pimenta-ova-de-tamuata (Pereira et al. 2011), Pimenta açaí (Amapá, *Pereira & Severino 1853*), Pimenta chumbinho (Amapá, *Pereira et al. 1807*), Pimenta de cheiro vermelha (Amapá, *Pereira et al. 1747*), Pimenta ova de aruanã (Amapá, *Pereira et al. 1867*); **Colombia**: Ají (Amazonas, *Henao & Padd 168*; Guainía, *Espina et al. 191*; Huila, *Llanos & Camacho 1827*; Valle del Cauca, *Cuatrecasas 22800*), Ajicito (Valle del Cauca, *Cuatrecasas 22800*), Ajijito (Valle del Cauca, *Lehmann 4730*), Cimarrón (Bolívar, *Killip & Smith 14251*), Ají chilca (Guainía, *Augusto 4847*), Ají chiquito (Norte de Santander, *Carvajalino & Díaz 11*; Santander, *Tochoy & Garzón 675*), Ají chirel (Antioquia, *Santa María 733*), Ají chivato (Meta, *García Barriga 5056*; Valle del Cauca, *Duque Jaramillo 4083-A*), Ají guagua (Bolívar, *Espina 577*), Ají pajarito (Antioquia, *Barkley & Gutiérrez V. 1776*; Caldas, *Galán & Cárdenas 12*; Norte de Santander, *Garganta F. s.n.*), Ají perfumado (Amazonas, *Henao 169*), Ají picante (Bolívar, *Killip & Smith 14534*; Norte de Santander, *Garganta F. 818*), Ahipique (Valle del Cauca, *Dryander 2178*), Ají pimienta (Bolívar, *Romero Castañeda 9255*; Santander, *Betancur et al. 10158*), Ají pique (Cundinamarca, *Dumont et al. 43*; Nariño, *de Benavides 4693*, Valle del Cauca, *Soukup 1851*), Ají piquicho (Huila, *Buendía S. 2*), Ají del monte (Atlántico, *Bro. Elías 1461*), Aji amarillo de culebra (Amazonas, *Henao & Z*ɨ*uec 247*), Ají ojo de charapa (Amazonas, *Henao & Kuiru 173*), Ají ojo de sapo (Amazonas, *Henao 170*), Chicha e gato (Santander, *Tochoy & Garzón 675*), Pimiento (Norte de Santander, *Carvajalino & Díaz 11*); **Costa Rica**: Chile, Chile congo, Chiltepe (Bohs 2015); **Cuba**: Ají guaguao (La Habana, *García Cañizares 127*; Villa Clara, *Luna 356*); **Dominican Republic**: Ají (Altagracia, *Zanoni & Mejía 17077 A*), Ajicito montecino (Azua, *Zanoni et al. 22115*; Monte Cristi, *Valeur 476*; Santiago, *Valeur 275*); **Ecuador**: Ají (Napo, *Alarcón 102*), Veneno de perro (Esmeraldas, *Freire & Ruales 2931*); **El Salvador**: Chiltepe (Ahuachapán, *Standley 19872*; Sonsonate, *Calderón 1658*), Chile de zope (Ahuachapán, *Standley 19872*; La Unión, *Standley 20689*), Chile chocolate pequeño (San Salvador, *Calderón 1197*); **Guadeloupe**: Piment Moka (Basse-Terre, *Duss 3575*), Piment grives (Basse-Terre, *Duss 3681*); **Guatemala**: Chiltepe, chile chiltepe (Chimaltenango, *Porter 1299*; Chuiquimula, *Kufer 99*; Huehuetenango, *Steyermark 51277*), Chiltep, chile de monte (Huehuetenango, *Steyermark 51364*), Chile de montaña (Huehuetenango, *Steyermark 51277*); **Honduras**: Chilpepe (Atlantida, *Standley 54485*), Chiltepe (Morazán, *Standley 26254*), Chiltepin (Atlantida, *Standley 53628*), Chile bravo (Atlantida, *Standley 53392*); **Jamaica**: Bird pepper (Kingston, *Harris 10051*; Saint Mary, *Yuncker 18460*); **Leeward Islands**: Piment café (*Duss 149*); **Martinica**: Piment rond, Piment sauvage (*Duss 349*); **Mexico**: Chile (Colima, *Eyerdanm & Beetle 8720*; Hidalgo, *Blanco-Macías 1559*; Puebla, *Villalobos C. & Guerrero 182*; Veracruz, *Olivares Hernández 1*), Chigundo (Oaxaca, *Torres et al. 199*), Chilegole (Oaxaca, *Salas M. et al. 1851*), Chilepepina (Baja California, *Carter 4921*), Chiletepin (Puebla, *Sarukhán et al. 3581*), Chilillo (Oaxaca, *González Olivares 494*), Chilpalla (Veracruz, *Martínez C. 1287*), Chilpitín (Baja California, *León de Luz 2019*; Coahuila, *Wendt* & *Riskind 1611*), Chiltapin (Chihuahua, *Gentry 949* & *1541*), Chiltepín (Puebla, *Bye et al. 16386*; Sonora, *Reina G. 1012*; Veracruz, *Cortés 552*), Chiltipen (Chihuahua, *Bye Jr. 1875*, Sonora, *Joyal 1815*), Chiltipin (Tamaulipas, *Nee 32699*; Veracruz, *Acosta et al. 39*), Chipilin (Chiapas, *Calzada et al. 3990*), Chirripitín (Michoacán, *Madrigal Sánchez 4875*), Pinchitle (Veracruz, Nee 1986), Piquín (Querétaro, *Martínez Torres 57*; San Luis Potosí, *Edwards 592*; Veracruz, *Vázquez 611*), Quipín (Querétaro, *Martínez Torres 57*), Tempenchile (Chiapas, *Arcos Vernet 40*), Tempinchile (Chiapas, *Trujillo Eslava 75*), Tepechile (Chiapas, *Calzada et al. 9679*), Chile bola (Veracruz, Nee 1986), Chile congo (Chiapas, *Castillo C. et al. 4136*), Chile chichalaco (Guerrero, *Guízar Nolazco & Pimentel B. 2884*), Chile chiguado (Oaxaca, *Nava Zafra et al. 1812*), Chile chilpalla (Veracruz, *Ibarra Manríquez 3616*), Chile Gole (Oaxaca, *Vásquez & Ortega 865*), Chiltepin grande (Puebla, *Villalobos C. & Guerrero 205*), Chile maxito (Campeche, *Ramírez A. 57*), Chile piquín (Chihuahua, *Torres C.* & *Tenorio L. 3751*; Veracruz, Nee 1986), Chile de árbol (Veracruz, *Ortega T. 88*), Chile de bolita (Veracruz, *Martínez C. 2011*), Chile Amash, chile mashito (Tabasco, *Orozco-Segovia 368*), Chile amachito (Tabasco, *Guadarrama et al. 865*), Chile chigol (Oaxaca, *Gopar Vásquez 122*), Chile garbanzo (Tabasco, *Escolastico 165*), Chile gordo (Veracruz, *Castillo C. et al. 214*), Chile machito (Tabasco, *Ortega O. 870*), Chile Pekin (Veracruz, *Calzada 5577*), Chili pequin (Sinaloa, *Ferris & Mexia 5130*), Chile piquín (Campeche, *Alvaro M. 397*; Nueva León, *Cano s.n.*; S. L. Potosí, *Gómez-Lorence 877*; Sinaloa, *Vega A. 1297*; Tamaulipas, *Rodríguez 104*; Veracruz, *Robles G. 886*), Chile silvestre (Campeche, *Álvarez 89*), Chile de monte (Jalisco, *Pérez J. 521*; Oaxaca, *Gopar Vásquez 122*; Yucatán, *Ordonez 239*), Chilote de monte (Tamaulipas, *Martínez Ojeda 263*), Jonnihui, tempen-chile (Chiapas, *Palacios E. 636*), Max, Chile max (Campeche, *Álvarez 89*; Quintana Roo, *Serralta P. 104*; Yucatán, *Estrada 43*), Max-hic (Campeche, *Bacab W. 115*), Siete Cardo (Chiapas, *Matuda 17590*), Chilitos de monte (Guerrero, *Kruse 1906*); **Nicaragua**: Chile (Carazo, *Aranda et al. 94*; Managua, *Guzmán et al. 396*; Masaya, *Guzmán et al. 1307*), Chile congo (Chontales, *Nee 28288*; Estelí, *Nee 27745*; Managua, *Araquistain 5*; Rivas, *Araquistain 278*), Chile montero (Managua, *Grijalva 706*); **Peru**: Ají (Loreto, *Lewis et al. 11196*), Ají del monte o charapilla (San Martín, *Woytkowski 35161*), Ají charapilla (Loreto, *de Jong 53*), Ají charapita (Ucayali, *Graham & Schunke V. 460*), Ají del trueno (Cajamarca, *Campos & Díaz 2293*), Pipi de mono (Huánuco, *Becerra González & Perea 1152*); **Puerto Rico**: Ahi caballero (Adjuntas, *Stimson 3922*), Ají caballos (Ponce, *Britton & Britton 7344*); **Surinam**: Spaanse pepper (Marowijne/Sipaliwini, *Rombouts 735*), Busi peper (Commewijne, *Heilbron & Sanredjo 6*); **United States of America**: Chillipiquin (Florida, *Cory 51407*), Bird pepper (Florida, *Kral 1884*), Cayenne Pepper (Florida, *Bishop & B. Holst CC0048*); **Venezuela**: Pajarito (Lara, *Tamayo 2618*), Ají corito (Mérida, *Trujillo 6351*), Ají pajarito (Carabobo, *Hunziker 9032*; Mérida, *Pittier 12837*), Ají de mono (Bolívar, *Liesner & González 5417*), Chirel del Mono (Bolívar, *Knab-Vispo et al. 1288*).

#### Indigenous names.

**Colombia**: Aati (Curripaco, Guainía, *Espina et al. 191*), Aiyo borarede jairai (Huitoto-Nɨpode, Amazonas, *Henao & Z*ɨ*ueche 247*), Beeakxtú (And, Amazonas, *Castro & Andoke 607*), Jɨgɨngo uijɨ (Huitoto-Mɨnɨka, Amazonas, *Henao 170*), Jimorai (Huitoto-Mɨnɨka, Amazonas, *Henao & Padd 168*), Kupirapa’ajiné (Amazonas, *Castro & Matapí 523*), Meniño uijɨ (Huitoto-Mɨnɨka, Amazonas, *Henao 173*), Wainpiraicha (Guajira, *Betancur et al. 11258*), Ziorai (Huitoto-Mɨnɨka, Amazonas, *Henao 169*); **Ecuador**: Bula uchu (Napo, *Irvine 773*), Giimo (Oncaye, Napo, *Davis & Yost 994*), Jimiea (Achuar Jívaro, Pastaza, *Lewis et al. 14010*), Jimia (Shuar, Zamora-Chinchipe, *Van den Eynden et al. 700*), Sampíajimia (Shuar, Zamora-Chinchipe, *Santín et al. 100*), Uchu (Quichua, Napo, *Alarcón 102*), Uchumuyu (Quichua/Spanish, Pastaza, *Lewis et al. 14010*); **Guatemala**: Chi-ik (Alto Verapaz, *Standley 90936*), Tamut ich (Ch’orti’, Chuiquimula, *Kufer 99*); **Mexico**: Guiiña (Zapateco, Oaxaca, *Sánchez L. et al. 1190*), Skapin (Totonaco, Veracruz, *Cortés-Vásquez 552* & *143*), Guiiña dxuladi (Zapateco, Oaxaca, *Sánchez L. & Trujillo V. 874*), Guien guiix (Oaxaca, *Ruiz Núñez 7*), Guiinya xigundu (Zapateco, Oaxaca, *Sánchez L. 317*), Kulum its (Huastec, S. Luis Potosí, *Alcorn 2369*), Max ik (Campeche, *Álvarez 89*; Yucatán, *Ucan et al. 3527* & *3893*), Lak’su pin (Tot, Puebla, *Villalobos C. & Guerrero 205*), Tsakam its (Huastec, S. Luis Potosí, *Alcorn 1406*; Veracruz, *Alcorn 1903*), Xmax ik (Yucatán, *Ucan* 4617), Aj max iik (Yucatán, *Ucan 5058*); **Peru**: Ají (Quichua, Loreto, *Lewis et al. 12906*), Cusharu’ nu’ca” (Chayahuita, Loreto, *Odonne 561*), Imiá (Achual Jívaro, Loreto, *Lewis et al. 11196*), Nuca (Loreto, *Odonne 25*), Nu’ca (Loreto, *Odonne 626*), Uchu (Loreto, *Lewis et al. 12552*), Yaa Jimia (Amazonas, *Salaün 185*), Yampit jima (Amazonas, *Berlin 2016*), Yanco nu’ca” (Chayahuita, Loreto, *Odonne 563*); **Surinam**: Lombo riwit (Ja, Commewijne, *Heilbron & Sanredjo 6*), Lombo kusti ‘pepper of god’ (Commewijne, *Heilbron & Sanredjo 6*).

#### Uses.

This taxon is used as an ornamental, for food and for medicine. The fruits are harvested by local people and are widely used and much prized throughout its distribution as a hot seasoning; they are also eaten fresh, dry or in vinegar, raw or toasted. Some medicinal properties have been attributed to the leaves and fruits in different countries (see Table 3). In some communities, fruits are used against evil spirits in ritual practices of the Day of the Dead (Mexico).

#### Preliminary conservation assessment.

EOO (37,301,728.615 km^2^); AOO (4,496 km^2^). Capsicumannuumvar.glabriusculum is not under threat for the time being.

#### Discussion.

Capsicumannuumvar.glabriusculum, better known as ‘chiltepin’ or ‘chilipequin’ (with some variations of these names) in Mexico and Central America (see common names) or ‘bird pepper’ in the United States and the Caribbean, belongs to the Annuum clade (Carrizo García et al. 2016). It is considered the wild progenitor of the cultivated C.annuumvar.annuum from which it can easily be differentiated by its fragile-stemmed somewhat prostate habit, very small flowers, calyx with 0–5 inconspicuous appendages, short filaments and small globose, ellipsoid or ovoid red-orange or red fruits.

In herbaria or in literature, many names have been misapplied to the specimens of this variety, such as *C.baccatum*, *C.frutescens*, *C.conoides*, C.annuumvar.conoides, C.annuumvar.baccatum, C.frutescensvar.baccatum and so on. Based only on the morphology of the fruits, this variety is sometimes confused with wild C.baccatumvar.baccatum. While the fruiting calyx of C.annuumvar.glabriusculum has 0–5 inconspicuous appendages and the fruits are generally more ovoid with an acute to slightly obtuse apex (rarely truncate), in C.baccatumvar.baccatum, the calyx has five appendages up to 2 mm in length and the fruits are generally more globose or subglobose to ellipsoid with a truncate or flattened apex (very rarely acute to slightly obtuse). In addition to the differences in the fruits, C.annuumvar.glabriusculum has solitary or paired flowers (rarely three flowers), stellate corollas that are entirely white to greenish-white without spots within and connivent blue, bluish-grey or purple anthers at anthesis (Fig. 23C–F), whereas C.baccatumvar.baccatum has usually 2–3 flowers (rarely one), white rotate or rotate-stellate corollas with greenish-yellow spots within and not connivent, usually white or pale yellow anthers at anthesis (Fig. 26D–F).

Kunth (1818) did not cite a specific specimen from Cuba in the protologue of *C.havanense*. At P, we found a sheet labelled *C.havanense* with the number ‘4518’ (P00670653) in the Herbarium of Humboldt and Bonpland; this sheet is selected as the lectotype.

Dierbach (1829) based Capsicumindicumvar.aviculare on *C.minimum* Mill. and *C.microcarpum* DC. *Capsicumminimum* applies to the wild specimens of *C.annuum* while *C.microcarpon* refers to the wild *C.baccatum* form which is, in fact, very similar to the wild *C.annuum* in the fruiting stage. D’Arcy and Eshbaugh (1973) proposed C.annuumvar.aviculare, based on C.indicumvar.aviculare, a name that was frequently used in literature to refer to the widespread spontaneous variety of *C.annuum*. In the interest of fixing the application of the basionym, we are designating a neotype using the modern collection *E.L. Tyson 5222* (MO-562584, acc. # 1980106) that D’Arcy used to illustrate this taxon (figure 4 in D’Arcy 1973) which shows its diagnostic characters.

Fingerhuth (1832) gave a very brief description for C.frutescensvar.minus (“fructu ovato obtuso minori”) and based this name on an illustration in the pre-Linnean Rumphius’ Herbarium Amboinense (1747: Tab. 88, fig. 2, as *Capsicumrubrumminimum*). This figure is of a complete flowering and fruiting branch that unequivocally matches with our concept of C.annuumvar.glabriusculum. As no collections were cited and no specimens with internal evidence of being part of the original material were found, Tab. 88, fig. 2 of Rumphius’ work is selected as the lectotype.

In describing *C.chlorocarpum*, Dunal (1852) cited in the protologue “Berland. n. 97, in h. DC. et Boiss”. We did not find the duplicate that Dunal saw in the “h. Boiss” (now part of the general herbarium at G). Therefore, we select here the duplicate at G-DC (G00131884) as the lectotype.

*Capsicumlaurifolium* was described, based on two different specimens in G-DC, both mounted on the same sheet. The right hand specimen (*Anonymous 67*, G00131902) comes from the Island of Guadeloupe in the Leeward Islands, part of the Lesser Antilles in the Caribbean. The left hand specimen is that of Blanchet from Bahia (Brazil). Of these two collections, we have selected the most complete specimen that most closely matches the data in the protologue (*Blanchet 3098 A*) as the lectotype (G00131882).

Dunal (1852) based the description of *C.hispidum* on three specimens of *Berlandier 152* today housed at G. The one in G-DC (G00131880) is the best-preserved and is here selected as the lectotype.

Dunal (1852) described *C.angustifolium*, based on specimens from plants cultivated in the Botanic Gardens in Geneva (“hort. Geneve 1836”). We have selected as the lectotype a sheet in G-DC (G00131878) with the same data as the protologue (“*Capsicumbaccatum* hort. Geneve, 1836”) and a handwritten label with “*Capsicumangustifolium* Dun., janvier 1845” in Dunal’s hand.

Barboza’s (2011) lectotypification for *C.microphyllum* using the collection *Berlandier 1907* from “Béjar” (Texas, United States of America) must be set aside, since D’Arcy and Eshbaugh (1974) explicitly lectotypified this name earlier with the collection of *Sagra* from Cuba at G-DC.

#### Specimens examined.

See Suppl. material 4: Appendix 4.

### ﻿2. Capsicum baccatum

Taxon classificationPlantaeSolanalesSolanaceae

L., Syst. Nat., ed. 12, 2: 174. 1767; Mant. Pl.: 47. 1767.

565EC4AE-E89D-53FE-9197-B3E958EC2D16

#### Type.

“Habitat in Indiis” Herb. Linn. N° 249.3 (lectotype, designated by D’Arcy and Eshbaugh 1974, pg. 95: LINN [LINN-HL249-3]).

### ﻿2a. Capsicum baccatum L. var. baccatum

Taxon classificationPlantaeSolanalesSolanaceae

84EC7D82-DB0D-5926-8109-427443E4BAC5

Figs 25 , 26

Capsicum pulchellum Salisb., Prodr. Stirp. Chap. Allerton: 134. 1796, nom. illeg. superfl. Type. Based on Capsicumbaccatum L. (cited in synonymy).
Capsicum microcarpum Cav., Descr. Pl. (Cavanilles): 371. 1802. Type. Cultivated in the Royal Botanical Garden in Madrid, Spain “H.R.M. [Hortus Regis Matritensis]. Se cría en la Havana... y se cultiva en el Jardín botánico” (lectotype, designated here: MA [MA-307276]).
Capsicum ciliare Willd., Enum. Pl. [Willdenow] 1: 243. 1809. Type. Cultivated in Berlin, Germany, of unknown origin “Cult. in Hort. Bot. Berol.”, C.L. Willdenow s.n. (lectotype, designated here: B [B-W04430-01-0]).
Capsicum indicum Dierb. var. ribesium Dierb., Arch. Apotheker-Vereins Nördl. Teutschl. 30 (1): 29. 1829. Type. Based on C.baccatum L.
Capsicum comarim Vell., Fl. Flumin.: 60. 1829 (“1825”); Fl. Flumin. Icon. 2: t. 2. 1831 (“1827”). Type. Brazil. [Rio de Janeiro]: “Colitur hortis, et sponte undequaque crescit” (lectotype, designated by Knapp et al. 2015, pag. 284: [illustration] Original parchment plate of Flora Fluminensis in the Manuscript Section of the Biblioteca Nacional, Rio de Janeiro [cat. no.: mss1198651_005] and later published in Vellozo, Fl. Flumin. Icon. 2: t. 2. 1831).
Capsicum cumanense Fingerh., Monogr. Capsic.: 17. 1832, nom. illeg. superfl. Type. Based on (renaming of) “Capsicumbaccatum Kunth” [= C.baccatum L.] (cited in synonymy).
Capsicum microcarpum DC. forma fruticosum Sendtn., Fl. Bras. (Martius) 10(6): 146. 1846. Type. Brazil “In Brasilia”, Pohl s.n. (lectotype, designated here: M [M-0171544]).
Capsicum microcarpum DC. forma herbaceum Sendtn., Fl. Bras. (Martius) 10(6): 146. 1846. Type. Brazil. “Martius Mss. in Itinerario n. 132”, Prope Polafoco, Sept., *C.F.P. Martius 132* (lectotype, designated here: M [M-0171543]; isolectotype, CORD [CORD00101765]).
Capsicum annuum L. var. microcarpum (DC.) Alef., Landw. Fl.: 133. 1866. Type. Based on Capsicummicrocarpum DC.
Capsicum annuum L. var. baccatum (L.) Kuntze, Revis. Gen. Pl. 2: 449. 1891. Type. Based on Capsicumbaccatum L.
Capsicum annuum L. var. microcarpum (Cav.) Voss, in Vilm. Blumengärtn., ed. 3. 1: 723. 1894. Type. Based on Capsicummicrocarpum Cav.
Capsicum frutescens L. var. baccatum (L.) Irish, Rep. (Annual) Missouri Bot. Gard. 9: 99. 1898. Type. Based on Capsicumbaccatum L.
Capsicum microcarpum Cav. var. glabrescens Hassl., Repert. Spec. Nov. Regni Veg. 15: 244. 1918. Type. Paraguay. Canindeyú: “Iter ad Yerbales montium Sierra de Maracayu, in regione cursus superioris fluminis Jejui guazú”, Dec. 1898-99, *É Hassler 5703* (lectotype, designated by Barboza 2011, pg. 28, second step designated here: G [G00390268]; isolectotypes: BM [BM000074084, acc. # 5447772; BM000074084a, acc. # 4575837; G [G00390266 two sheets with same barcode, G00390267], GH [00936720], K [K000585896], MO [MO-503802, acc. # 1574551], NY [00138600], P [P00410160, P00410161, P00482076], UC [UC944854], W [acc. # 1902-0002869]).
Capsicum annuum L. subsp. baccatum (L.) Terpó, Feddes Repert. 72: 173. 1966. Type. Based on Capsicumbaccatum L.

#### Description.

Erect shrubs or perennial herbs 0.50–3 (–3.5) m tall, rarely small trees, the main stem 2-2.5 cm in diameter at base, much branched from near the base and above, the branches spreading in a typical “zig-zag” appearance. Young stems 3–4-angled, fragile, green, sometimes the ridges purple, mostly glabrous to sparsely or moderately pubescent with appressed-antrorse, simple, uniseriate, 4–7-celled, eglandular trichomes 0.2–1.2 mm long; nodes usually purple; bark of older stems fissured, dark brown, glabrous; lenticels abundant. Sympodial units difoliate, the leaves geminate; leaf pair unequal in size, similar in shape. Leaves membranous, slightly discolorous, dark green above, light green beneath, glabrescent to moderately pubescent with appressed-antrorse trichomes like those of the stems on both surfaces and margins; blades of major leaves 4.5–10 cm long, 2.5–6 cm wide, ovate, the major veins 5–8 on each side of mid-vein, the base somewhat asymmetric and attenuate, the margins entire, the apex acute; petioles (1.5–) 2–4 cm long, moderately to densely pubescent; blades of minor leaves 3.5–4.5 cm long, 2–3 cm wide, ovate, the major veins 4–5 on each side of mid-vein, the base rounded or truncate, the apex acute; petioles 0.5–1 cm long, moderately to densely pubescent. Inflorescences axillary, 2–3 flowers per axil, rarely flowers solitary; flowering pedicels (17–) 20–35 mm long, angled, erect or slightly spreading, geniculate at anthesis, glabrescent to moderately pubescent, the eglandular trichomes short, spreading or antrorse; pedicel scars inconspicuous. Buds globose, white with greenish-yellow spots, occasionally purple. Flowers 5-merous. Calyx 1.5–2 (–2.5) mm long, ca. 2–2.5 mm wide, cup-shaped, thick, green, pubescent with the same trichomes as pedicels and some glandular trichomes, the calyx appendages 5, (0.3–) 0.5–2 mm long, 0.2 mm wide, subequal, thick, erect, cylindrical, inserted close to the margin, pubescent with the same trichomes as calyx tube. Corolla 4.5–7.5 mm long, 10–13 mm in diameter, thick, white with greenish-yellow spots and white centre outside and within, rotate or rotate-stellate, with interpetalar membrane, lobed 1/3 or less of the way to the base, pubescent adaxially with short glandular trichomes (stalk 1–3-celled; head globose, peltate, unicellular) in the throat and base of the lobes, glabrous abaxially, the tube 4–5 mm long, the lobes 2.5–2.7 mm long, 3.4–3.5 mm wide, broadly triangular, spreading, the margins with very short eglandular trichomes, the tips acute, papillate. Stamens five, equal; filaments 2.5–3.5 mm long, white, inserted on the corolla 1–1.1 mm from the base, with auricles fused to the corolla at the point of insertion; anthers 1.5–1.8 mm long, ellipsoid, white or pale yellow, more rarely greyish, not connivent at anthesis. Gynoecium with ovary 2.5–2.7 mm long, ca. 2 mm in diameter, ovoid, green; nectary 0.3–0.5 mm tall; styles dimorphic, short style 2–2.5 mm long, not exceeding the anthers length, long style ca. 3.5 mm long, exserted 1.4–1.7 mm beyond the anthers, cylindrical, white; stigma 0.3 mm in diameter, globose or discoid, pale green. Berry 6–8 (–10) mm in diameter, globose or subglobose, less frequently ellipsoid with truncate or flattened apex, 10–20 mm long, 4–7 mm in diameter, green when immature turning to greenish-black and bright red at maturity, deciduous, pungent, the pericarp thick, opaque, with giant cells (endocarp alveolate); stone cells absent; fruiting pedicels 20–35 mm long, erect, strongly angled, widened distally, green; fruiting calyx 3–4.5 mm in diameter, persistent, not accrescent, cup-shaped or discoid, green, the appendages 1.5–2.3 mm long, appressed to the berry or spreading. Seeds 12–15 per fruit, 2.5–4 mm long, 2.3–3 mm wide, ovoid, subglobose or C-shaped, pale yellow to yellow, the seed coat smooth or slightly reticulate (SM), cerebelloid (SEM), the cells irregular in shape, the lateral walls sinuate strongly sinuate; embryo imbricate.

**Figure 25.**
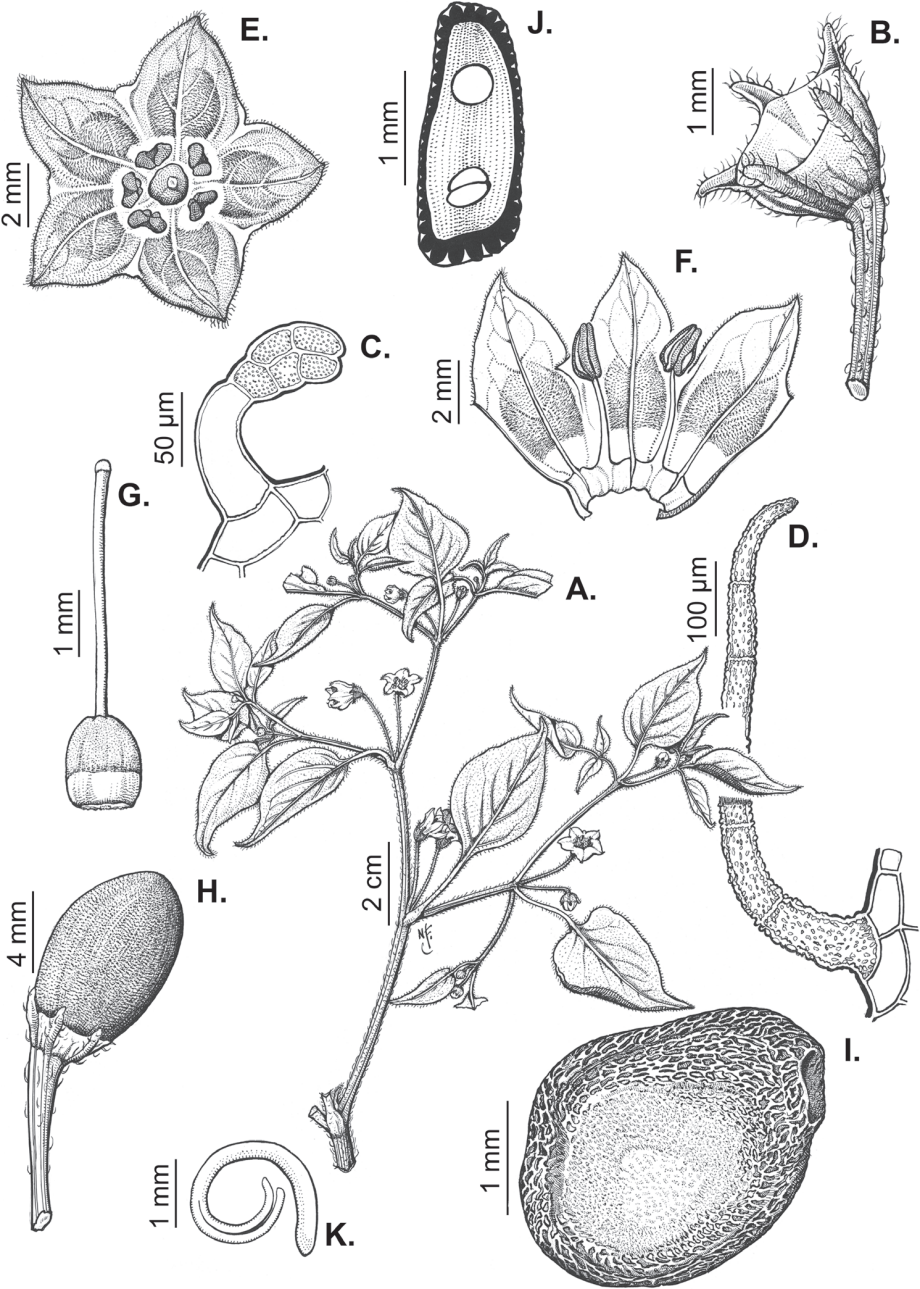
Capsicumbaccatumvar.baccatum**A** flowering branch **B** calyx **C** glandular trichome of the calyx **D** eglandular trichome of the calyx **E** flower, upper view **F** sector of opened corolla **G** gynoecium **H** fruit **I** seed **J** seed, in cross section **K** embryo **A–G** from *Hunziker 7350***H–K** from *Hunziker 1579*. Drawn by N. de Flury. Published in Barboza (2013), courtesy of the Board of the Instituto Darwinion (San Isidro, Buenos Aires, Argentina), reproduced with permission.

**Figure 26.**
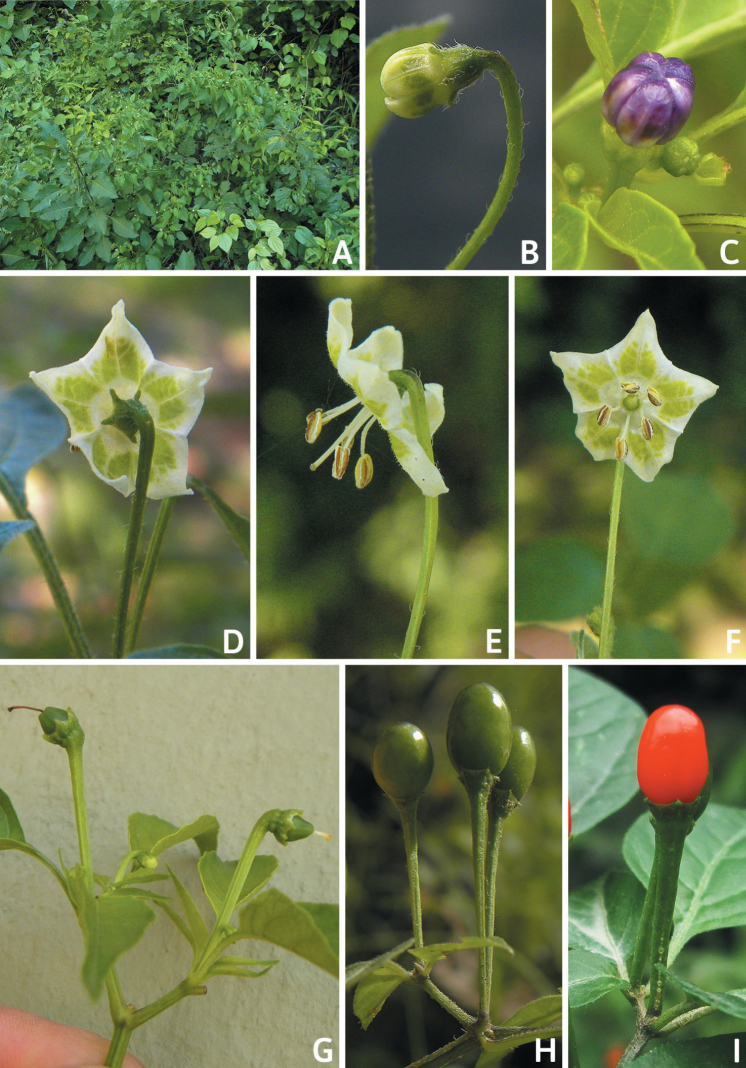
Capsicumbaccatumvar.baccatum**A** plant **B** flower bud **C** purple flower bud **D, E, F** flowers at anthesis, different views **G** young fruiting branch showing ovary with long and short styles **H** immature fruits **I** mature fruit **A, C** from *Barboza 4913***B, D–F, H** from *Barboza 5038***G** from *Barboza 2431 bis*, **I** from *Barboza et al. 3419*. Photos by G.E. Barboza.

#### Distribution.

**Figure 27.**
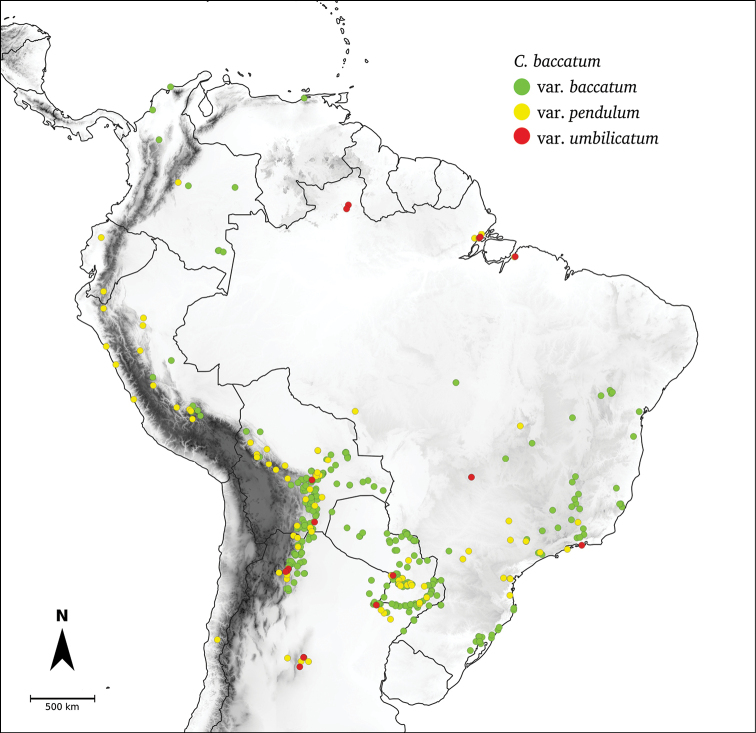
Distribution of C.baccatumvar.baccatum, C.baccatumvar.pendulum and C.baccatumvar.umbilicatum.

Capsicumbaccatumvar.baccatum is widely distributed in South America from northern Venezuela and Colombia through Peru, Bolivia, Paraguay, northern and north-eastern Argentina to south and eastern Brazil (Fig. 27).

#### Ecology.

Capsicumbaccatumvar.baccatum occurs in dry or humid subtropical or tropical forests with semi-deciduous or deciduous vegetation, between 150 and 1,900 m elevation; it is quite common in Chaco scrub forests, in gallery forests and in the margins or interior of secondary forests. It is frequently a ruderal in disturbed areas.

#### Phenology.

Flowering from October to May. Fruiting from late November to September.

#### Chromosome number.

2*n* = 2x = 24 (Pickersgill 1977; Moscone et al. 2003, 2007).

#### Common names.

**Argentina**: Coincho (Jujuy, *Fabris 3454*), Cumbarí (Misiones, *Montes 15164*), Quitucho (Salta, *Hunziker 1985*), Puta-parió (Corrientes, *Martínez Crovetto 11125*), Ají quitucho (Salta, *Hunziker 1579*), Ají del campo, Ajitucho (Salta, *West 8389*), Ají del monte (Salta, *Rial Alberti s.n.*), Pimenta del monte (Misiones, *Montes 15164*), Pimentón del monte, ají cumbarí (Misiones, *Montes 15202*); **Bolivia**: Arabibi (Santa Cruz, *Zenteno-R 12798*), Aribibe (Santa Cruz, *Hurtado 296*), Aribibi (Chuquisaca, *Debouck 3019*; Santa Cruz, *de Michel 159*), Arivivi (Chuquisaca, *Serrano 1903*; Santa Cruz, *Cárdenas 4702*), Cobincho (Tarija, *Krapovickas & Schinini 39010*), Ají aribibi (Cochabamba, *Thomas 705*), Aribibi silvestre (Beni, *Rivero 218*), Ají del campo (Tarija, *Krapovickas & Schinini 39010*), Arivivi grande o cumbarito (Fundación PROINPA 2007), Arivivi last’a o miska (Fundación PROINPA 2007), Arivivi morado (Fundación PROINPA 2007), Arivivi tuna árbol grande (Fundación PROINPA 2007), Arivivi tuna con flor blanca (Fundación PROINPA 2007); **Brazil**: Cumari (Espírito Santo, *Crepaldi 59*), Pimenta-cumari (Goiás, *Mendonça et al. 5971*), Pimenta de passarinho (Bahia, *Mori 11603*); **Colombia**: Ají (Vichada, *Rodríguez 164*), Ají de babilla (Amazonas, *Cárdenas 9423*), Ají de la capitania (Amazonas, *Cárdenas 9403*); **Peru**: Aji Ayucllo (Junín, Bosland and Votava 2000).

#### Indigenous names.

**Bolivia**: Pochetii (Trinitario, Cochabamba, *Thomas 705*), Winno, Sachimi (Yuracare, Cochabamba, *Thomas 705*); **Colombia**: Azi (Piapocos, Vichada, *Rodríguez 164*), Gugsobia (Amazonas, *Cárdenas 9423*), Kulana (And, Amazonas, *Castro & Matapí 564*), Kulana (Yucuna, Amazonas, *Cárdenas 9403*), Kuraraka (Letuama, Amazonas, *Cárdenas 9401*); **Paraguay**: Hõmpita (Ayoreo, Boquerón, *Gragson 124*), Nuuhá (Alto Paraguay, *Schmeda 1584*).

#### Uses.

As fruits are generally extremely pungent, they are collected and stored for use as a food condiment by native populations. Some accessions of this wild pepper (“arivivi”) in Bolivia have been considered promising for their interesting agro-morphological and biochemical characteristics with potential for the development of high value products for different markets (Fundación PROINPA 2007; van Zonneveld et al. 2015). However, commercialisation is still marginal and fruits are cultivated in home gardens or gathered from nature for self-consumption or to be distributed locally (Jäger et al. 2013).

#### Preliminary conservation assessment.

EOO (11,809,545.422 km^2^); AOO (1,212 km^2^). Capsicumbaccatumvar.baccatum is considered Least Concern (LC) for the time being.

#### Discussion.

Capsicumbaccatumvar.baccatum is a member of the Baccatum clade and is related to *C.rabenii* and *C.chacoense* (Carrizo García et al. 2016). This entity is considered to be the wild progenitor of the cultivated C.baccatumvar.pendulum and is widespread in South America. Its main centre of domestication is thought to be in the Bolivian Amazonia and inter-Andean valleys (Scaldaferro et al. 2018).

In an effort to clarify the taxonomy of *C.baccatum*, Eshbaugh and collaborators (Eshbaugh 1968, 1970, 1976; D’Arcy and Eshbaugh 1974; Jensen et al. 1979; Pickersgill et al. 1979; McLeod et al. 1983a, 1983b; Mitchell et al. 1989) and others (Kuriachan 1981; Egawa and Tanaka 1984; Jarret 2007; Scaldaferro et al. 2018) have studied the wild and domesticated forms of this species with a multitude of techniques (morphological, breeding, cytogenetic, biochemical, molecular, phylogeographical); the results support the wild progenitor-domesticate association proposed by Eshbaugh (1968).

Capsicumbaccatumvar.baccatum typically exhibits 2–3 flowers per node, rarely solitary flowers, geniculate pedicels that are erect or declining at anthesis, 5-merous flowers with white rotate or rotate-stellate corollas with greenish-yellow spots, dimorphic styles and small, globose, subglobose or ellipsoid, erect, deciduous, red fruits (Fig. 26). The domesticated genetic lines mainly differ in having larger, 5–8-merous flowers, an ovary with 2–5 locules, quite diverse fruits that vary in size, colour (green, yellow, brown, orange, red) and shape (pendent, usually elongate or of different forms) and larger seeds.

Fruiting specimens of C.baccatumvar.baccatum are very similar to *C.rabenii* and it is sometimes impossible to distinguish the two, especially if there are no annotations about the corolla colour (in *C.rabenii*, corolla lobe margins are purple). However, C.baccatumvar.baccatum usually has glabrescent to moderately pubescent leaves in contrast to the densely lanose pubescence found abaxially along the main veins in *C.rabenii* (Fig. 106B).

Capsicumbaccatumvar.baccatum differs from *C.chacoense*, with which it is sympatric in some localities of Argentina, Bolivia and Paraguay, by usually having five calyx appendages, a larger and rotate or rotate-stellate corolla with greenish-yellow pigmentation within, staminal plaques with auricles fused to the corolla and long and short styles. In contrast, *C.chacoense* has a calyx with 5–10 unequal appendages, entirely white and smaller corollas (4–6 mm long), staminal plaques with auricles not fused to the corolla and homomorphic styles (Fig. 46). Fruiting specimens of C.baccatumvar.baccatum sometimes are difficult to distinguish from *C.chacoense*. The fruiting pedicels of C.baccatumvar.baccatum are usually 2–3 per node and the fruiting calyces have five subequal appendages, without any evidence of a mucro below the calyx margin, which differs from the solitary fruiting pedicels and 5–10 unequal calyx appendages in *C.chacoense*.

Some earlier botanists submerged the epithet *baccatum* under *C.annuum* (Kuntze 1891) or *C.frutescens* (Irish 1898) to name specimens belonging to C.annuumvar.glabriusculum or wild forms of *C.baccatum*, an interpretation followed by other researchers (Smith and Heiser 1957; Emboden 1961; Terpó 1966).

Although the pungency of *C.baccatum* is regarded as low-mild (Libreros et al. 2014; Tanaka et al. 2017), some accessions of C.baccatumvar.baccatum from Bolivia have been reported as non-pungent (e.g. Chuquisaca: *Manchego CBNP 04, 05 & 06*; Santa Cruz: *Manchego CBNP 01, 02 & 03*; also see Tewksbury et al. 2006).

Cavanilles (1802) described *C.microcarpum*, based on material cultivated at the Real Jardín Botánico de Madrid from seeds sent from Cuba. We found two Cavanilles specimens at MA gathered in the Real Jardín Botánico de Madrid in 1802: MA-307276, separated as type material in the *Cavanillesii Typi* collection (Garilleti 1993; https://plants.jstor.org/stable/viewer/10.5555/al.ap.specimen.ma307276?loggedin=true) and MA-307278, which is filed in the general collection. Both specimens are labelled as *Capsicummicrocarpum*. According to Garilleti (pers. comm.), the original writing on the sheet label MA-307278 (the left label) is not from Cavanilles, but from Demetrio Rodríguez; since this plant was grown in the Real Jardín Botánico, Rodríguez could have collected it later and Cavanilles might not have seen it. Therefore, the only unequivocal original material is that with the label in Cavanilles’ handwriting (MA-307276), which is here selected as the lectotype; this sheet consists of three branches with flowers and fruit.

Willdenow (1809) based the description of *C.ciliare* on a specimen of unknown origin cultivated in the Botanical Garden of Berlin. At B, there is one sheet in Willdenow’s Herbarium with the script “C.ciliare, Hort. Bot. Berol. W.”; this sheet is selected as the lectotype.

Sendtner (1846) described C.microcarpumformafruticosum, based on two collections, one from Corego de Jaragua, São Paulo (“leg. Pohl”) and the other from “prov. Sebastianopolitana, ad Lagoa de Freitas” [Rio de Janeiro], but he cited no herbaria. We have found only the specimen collected by Pohl (M-0171544), which is selected as the lectotype.

For C.microcarpumformaherbaceum, Sendtner (1846) mentioned the specimen “Mart. Mss. in Itinerario n. 132”, but cited no herbaria. We found a collection at M (M-0171543) with a handwritten description that is in agreement with Sendtner’s diagnosis and has the number 132. Therefore, this sheet is designated the lectotype.

When Kuntze (1891) proposed the combination C.annuumvar.baccatum (L.) Kuntze, he was referring to specimens that actually correspond to the spontaneous forms of *C.annuum* (cfr. *Kuntze s.n.*, NY barcode 01008231), but he incorrectly used the epithet *baccatum* L. which corresponds to a different species (*C.baccatum* L.).

When Terpó (1966) proposed the combination C.annuumsubsp.baccatum, based on *C.baccatum* L., he clearly stated “Mexikanischer Wildpaprika” which actually belongs to the spontaneous forms of *C.annuum*.

#### Specimens examined.

See Suppl. material 4: Appendix 4.

### ﻿2b. Capscium baccatum L. var. pendulum

Taxon classificationPlantaeSolanalesSolanaceae

(Willd.) Eshbaugh, Taxon 17: 51. 1968.

B7A3FEA5-FA8C-5687-B9FE-9EF7962C503C

Fig. 28

Capsicum pendulum Willd., Enum. Pl. [Willdenow]: 242. 1809. Type. Cultivated in the Berlin Botanic Garden, Germany “Habitat ... [Country unknown]. Cult. in Hort. Bot. Berol”., C.L. Willdenow s.n. (lectotype, designated here: B [B-W04431-01-0]).
Capsicum frutescens L. var. pendulum (Willd.) Besser, Cat. Jard. Bot. Krzemieniec: 29. 1816. Type. Based on Capsicumpendulum Willd.
Capsicum indicum Dierb. var. pendulum (Willd.) Dierb., Arch. Apotheker-Vereins Nördl. Teutschl. 30: 28. 1829. Type. Based on Capsicumpendulum Willd.
Capsicum pendulum Willd. var. majus Dunal, Prodr. [A. P. de Candolle] 13(1): 425. 1852. Type. No locality cited (no specimens cited; no original material located; Dunal may have considered this the typical variety).

#### Type.

Based on *Capsicumpendulum* Willd.

#### Description.

Erect shrubs or perennial herbs (0.60–) 1–2.5 m tall, with the main stem 1.5–2.5 cm in diameter at base, much branched from near the base and above, the branches spreading in a typical “zig-zag” appearance. Young stems 3–4-angled, fragile, dark green or green, mostly glabrous to sparsely or moderately pubescent with appressed-antrorse, short to long, simple, uniseriate, 4–9-celled, eglandular trichomes 0.5–1.3 mm long; nodes green; bark of older stems green with light brown fissures, glabrous; lenticels absent or few. Sympodial units difoliate, the leaves geminate; leaf pair unequal in size, equal or subequal in shape. Leaves membranous, discolorous, dark green above, light green beneath, glabrous to glabrescent with short eglandular trichomes in margins and long, spreading, 5–9-celled, eglandular trichomes along the primary veins and in the vein axils beneath; blades of major leaves 5–12 (–14.5) cm long, 3–5 (–7) cm wide, ovate, the major veins 5–7 on each side of mid-vein, the base asymmetric and attenuate or symmetric and rounded, the margins entire, the apex acute or acuminate; petioles 2.5–7.5 cm long, sparsely pubescent; blades of minor leaves 3–5.8 cm long, 1.3–2.5 cm wide, ovate or elliptic, the major veins 3–4 on each side of mid-vein, the base rounded, the margins entire, the apex acute; petioles 1.7–2 cm long, sparsely to moderately pubescent. Inflorescences axillary, 2–3 flowers per axil or flowers solitary; flowering pedicels 20–50 mm long, terete or angled, erect, sometimes curved, geniculate at anthesis, glabrous, glabrescent to moderately pubescent, the trichomes short, antrorse; pedicels scars inconspicuous. Buds globose, white with greenish-yellow spots, occasionally purple. Flowers 5–8-merous. Calyx 2–3 mm long, 3–4.2 mm wide, cup-shaped, thick, strongly 10-nerved, green, glabrous or glabrescent, the calyx appendages 5–6, rarely up to 8, 0.9–2.5 mm long, 0.2 mm wide, subequal, thick, erect, cylindrical, inserted close to the margin, with the same pubescence as calyx tube. Corolla 8.5–15 mm long, 12–16 (–20) mm in diameter, thick, white with greenish-yellow to tan spots and a white centre outside and within, rotate to rotate-stellate, with interpetalar membrane, lobed less than 1/3 of the way to the base, pubescent adaxially with short glandular trichomes (stalk 1–3-celled; head globose, peltate, unicellular) in the throat and base of the lobes, glabrous abaxially, the tube 4–5 mm long, the lobes 3–3.3 (–5) mm long, 3.5–6 mm wide, triangular to broadly triangular, spreading, the margins finely ciliate, the tips acute, papillate. Stamens 5–8, equal; filaments (2.5–) 3–4 mm long, white, inserted on the corolla 1.2–1.5 mm from the base, with auricles fused to the corolla at the point of insertion; anthers 2–2.6 mm long, ellipsoid or ovoid, yellow, brownish post-dehiscent, not connivent (rarely connivent) at anthesis. Gynoecium with ovary 2.5–3 mm long, 1.6–2.5 mm in diameter, 2–5-carpelar, light green, ovoid; ovules more than two per locule; nectary ca. 1.2 mm tall, yellowish-green; styles dimorphic, short style 1.3–2 mm long, not exceeding the anthers length, long style 2.5–3.5 mm long, at the same level or slightly exserted beyond the anthers, yellowish-white, cylindrical; stigma ca. 0.2 mm long, 0.7–0.8 mm wide, discoid or bilobed, pale green. Berry 20–180 mm long, (10–) 20–40 (–50) mm in diameter, usually elongate or elongate-curved, triangular or campanulate, rarely subglobose, the base truncate or obtuse, the apex rounded, blunt or pointed, dark green or green when immature, green, bright yellow, orange, brown or red at maturity, persistent, pungent, the pericarp thick, opaque, with giant cells (endocarp alveolate); stone cells absent; fruiting pedicels (35–) 50–95 mm long, pendent, sometimes strongly curved, terete or slightly angled, widened distally, green; fruiting calyx 9–18 (–20) mm in diameter, persistent, slightly accrescent, campanulate, thick, somewhat corrugated or not, green, the appendages 0.5–2 mm long, appressed to the berry. Seeds 30–80 per fruit, (3–) 4–5.2 mm long, 3–3.8 (–4) mm wide, reniform or C-shaped, pale yellow to yellow, the seed coat smooth or slightly reticulate (SM), minutely reticulate (SEM), the cells polygonal to irregular in shape, the lateral walls straight to wavy; embryo imbricate.

**Figure 28.**
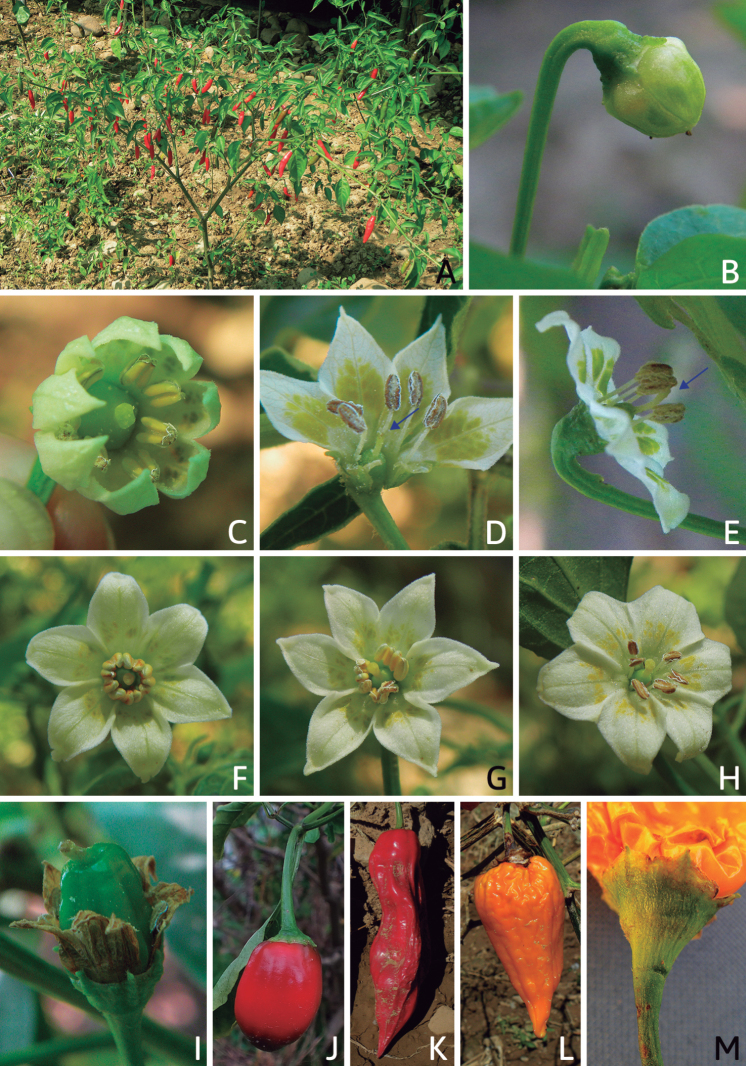
Capsicumbaccatumvar.pendulum**A** plant **B** flower bud **C** flower, in pre-anthesis **D** flower short-styled, longitudinal section **E** flower long-styled, lateral view **F–H** flowers hexamerous showing connivent anthers and not connivent anthers **I** immature fruit **J–M** mature fruit **A, B, E** from *Barboza 4886***C, D, I, K, L, M** no specimen vouchers (cult. Pairumani, Cochabamba-Bolivia) **F, G, H** from *Palombo 3***J** from *Barboza et al. 4824*. Photos by G.E. Barboza.

#### Distribution.

Capsicumbaccatumvar.pendulum is found from low to mid-Andean elevations, mainly in Bolivia and Peru, extending to Ecuador and Colombia in the north and reaching Argentina, Paraguay and south-western Brazil in the south (Fig. 27). Introductions to North and Central America (D’Arcy and Eshbaugh 1974; Thampi 2003), northern Brazil (Roraima, Barbosa et al. 2006), Europe (Rodríguez-Burruezo et al. 2009), Japan (D’Arcy and Eshbaugh 1974) and India (Thampi 2003; Rudrapal and Sarwa 2020) are recent.

#### Ecology.

Capsicumbaccatumvar.pendulum is a cultivated plant adapted to many different ecological conditions between 150 and 3,400 m elevation.

#### Phenology.

Flowering and fruiting all year.

#### Chromosome number.

2*n* = 2x = 24 (Pickersgill 1977; Moscone et al. 2003, 2007).

#### Common names.

**Argentina**: Ají (San Juan, *Ariza Espinar 3214*; Corrientes, *Benítez 76*); Varita (Salta, *Krapovickas & Schinini 28134*), Ají picante (Salta, *Hunziker 25496*); Ají vainilla (Salta, *Hilgert 1363*), Puta parió (Corrientes, *Martínez Crovetto 11125*), Varita larga (Salta, *Krapovickas & Schinini 28132*), Ají huevo de gallo (Salta, *Hilgert 1374*); **Bolivia**: Ají (Santa Cruz, *Williams 696*; Tarija, *Krapovickas & Schinini 39321*), Aribibe (Santa Cruz, *Hurtado 296*), Aribibi (Santa Cruz, *Heiser C281a*, La Paz, *Debouck et al. 3016*), Ulupica (Tarija, *Krapovickas & Schinini 31056*), Aji acabeche (La Paz, *Hinojosa & Wásra 1133*), Ají amarillo (Cochabamba, *Moscone 205*), Aji Picante (Santa Cruz, *Krapovickas & Schinini 32133*), Locato largo (Santa Cruz, *Heiser C290*), Ají churcu, Ají rojo, Ají redondo, Pimentón colorado, Ají colorado gigantón (Santa Cruz, Libreros et al. 2014), Asta de buey amarillo, Huacareteño Duraznal, Huacareteño naranjo, Pa púca, Plomadita amarillo, Plomadita rojo, Chicotillo grueso, Huacarateño ancho amarillo, Cola de ratón amarillo, Cola de ratón naranjo (Chuquisaca, Libreros et al. 2014), Chicotillo, Huacareteño, Huacareteño amarillo, Ají amarillo, Ají colorado, Cumbaru rojo, Asta de buey, Asta de toro, Astay toro amarillo, Astay toro rojo, Astay toro anaranjado, Puntay lanza rojo, Puntay lanza anaranjado, Astay buey rojo (Chuquisaca, Jäger et al. 2013), Lata y Toro (Cochabamba, *Moscone 209*); **Brazil**: Pimenta (Rio de Janeiro, *Krapovickas et al. 23428*), Pimenta ardida (São Paulo, *Alves de Paiva 01*), Chifre de Veado (São Paulo, *Heiser BGH 952*), Dedo de moça (Paraná, *Freire de Carvalho s.n.*; Roraima, Barbosa et al. 2006), Pimenta de Passarinho (Espírito Santo, *Mori et al. 11603*), Pimenta dedo-de-moça (Amapá, *Pereira et al. 1819*; Minas Gerais, *Vianna s.n.*; Paraná, *Leitão s.n.*), Pimenta de cheiro amarela (Amapá, *Pereira et al. 1830*), Pimenta de cheiro vermelha (Amapá, P*ereira & Severino 1851*), Pimenta-unha-de velha (Río de Janeiro, *Borges 66*); **Chile**: Chota cabra (Santiago, *Krapovickas 22232*); **Paraguay**: Ají (Paraguarí, *Galander 1877*), Locotito (Paraguarí, *Williams et al. 127*), Locotito chico (Guairá, *Williams et al. 121*); **Peru**: Ají (San Martín, *Belshaw 3210*), Ají escabeche (Ancash, *Francia 42*); Amarillo Moquegua (Lima, *Velarde Nuñez 6*), Amarillo Tacna (Lima, *Velarde Nuñez 23*), Amarillo Trujillo (Lima, *Velarde Nuñez 10*), Cilindro amarillo (Lima, *Velarde Nuñez 13*), Cilindro colorado (Lima, *Velarde Nuñez 15*), Colorado Tacna (Lima, *Velarde Nuñez 24*), Escabeche amarillo (Lima, *Velarde Nuñez 11*), Escabeche intermedio (Lima, *Velarde Nuñez 27*), Escabeche Lurín (Lima, *Velarde Nuñez 2*), Escabeche Moquegua (Lima, *Velarde Nuñez 3*), Extra Lurín (Lima, *Velarde Nuñez 21*), Escabeche colorado ENA (Lima, *Velarde Nuñez 1*), Ají Amarillo, Ají escabeche (Bosland and Votava 2000), Ayuyo, Challuaruro (San Martín, Libreros et al. 2013), Ají uña de gallina (Lambayeque, Libreros et al. 2013).

#### Indigenous names.

**Argentina**: Keuí (= picante) (Corrientes, *Hunziker 7339*); **Bolivia**: Kîî (Guaraní, Santa Cruz, *Roca 0689*); **Paraguay**: Ky y’ (Guaraní, Cordillera, *Williams et al. 135*), Pimenta í (Guaraní, Guairá, *Williams et al. 121*).

#### Uses.

This domesticated variety, mostly known as ‘Ají’, ‘Ají amarillo’ or ‘Ají escabeche’, is an important component of the diet of the Bolivian and Peruvian native population, less so in Argentina, Brazil, Ecuador and Colombia. In Bolivia and Peru, many different pod types occur that differ both in morphological (shape, colour, size) and biochemical attributes (e.g. capsaicinoids, vitamin E, flavonoids and quercetin content and antioxidant capacity). These forms are consumed in regional cuisines as spices and vegetables, fresh or dehydrated and ground (Libreros et al. 2013, 2014).

#### Preliminary conservation assessment.

EOO (11,296,813 km^2^); AOO (356 km^2^). Capsicumbaccatumvar.pendulum is a very widespread cultivated plant and can be assigned a category of Least Concern (LC).

#### Discussion.

Capsicumbaccatumvar.pendulum is a member of the Baccatum clade (Carrizo García et al. 2016). Morphologically, the corollas are similar in shape and colour to those of its wild progenitor, but they are larger, reaching 20 mm in diameter at anthesis; more consistent differences are the position of the fruiting pedicels and fruits (pendent), fruits that vary in size, shape and colour, the number of seeds (up to 80 per fruit) and the larger seeds (3–5.2 mm long, 3–4 mm wide). The presence of heterostylous flowers is more frequent in var. pendulum than in var. baccatum (Barboza, pers. obs.). This is an uncommon character within *Capsicum* that has been observed in both domesticated (Perera and Poulos 1993; Peña-Yam et al. 2019) and wild species (*C.benoistii* and *C.tovarii*).

Many studies have been carried out that demonstrate the potential of this domesticated form in crop improvement. Capsicumbaccatumvar.pendulum encompasses a wide range of fruit morphology (e.g. fruit weight, size, shape and ripe colour), health-related compounds (flavonoids, polyphenols, quercetins, vitamin E, ascorbic acid, fat, amongst others) and capsaicinoids content (low to mild) (Rodríguez-Burruezo et al. 2009; Kollmannsberger et al. 2011; Libreros et al. 2013, 2014; van Zonneveld et al. 2015). Furthermore, a particularly strong vegetable-like aroma has been detected in the fruits, due to diverse volatiles in accessions from Ecuador and Peru, with additional fruity/exotic notes in some genotypes (Kollmannsberger et al. 2011).

Willdenow (1809) provided the description of *C.pendulum*, based on cultivated material of unknown provenance. In Willdenow’s Herbarium housed at B, we found a sheet with original material (B-W04431-01-0) consisting of a fruiting specimen with the inscription “C.pendulum, Hort. bot. Berol. W.”; this sheet is designated the lectotype.

#### Specimens examined.

See Suppl. material 4: Appendix 4.

### ﻿2c. Capsicum baccatum L. var. umbilicatum

Taxon classificationPlantaeSolanalesSolanaceae

(Vell.) Hunz. & Barboza, Kurtziana 26: 27. 1998.

FB7F27D0-A36C-58EC-9877-A59A371E6462

Figs 29 , 30

Capsicum umbilicatum Vell., Fl. Flumin.: 61. 1829. Type. Brazil. [Rio de Janeiro]: “Colitur hortis” (lectotype, designated by Knapp et al. 2015, pg. 825: [illustration] Original parchment plate of Flora Fluminensis in the Manuscript Section of the Biblioteca Nacional, Rio de Janeiro [cat. no.: mss1198651_010] and later published in Vellozo, Fl. Flumin. Icon. 2: t. 7. 1831).

#### Type.

Based on *Capsicumumbilicatum* Vell.

#### Description.

Erect shrubs 1.50–2 m tall, with the main stem 1–1.5 cm in diameter at base, much branched from near the base, the branches spreading in a typical “zig-zag” appearance. Young stems 3–4-angled, fragile, green, mostly glabrous to sparsely pubescent with appressed-antrorse, short, simple, uniseriate, 4–5-celled, eglandular trichomes 0.3–0.6 mm long; nodes green or purple; bark of older stems green with light brown fissures, glabrous; lenticels absent. Sympodial units difoliate, the leaves geminate; leaf pair unequal in size, similar in shape. Leaves membranous, concolorous or slightly discolorous, dark green above, light green beneath, glabrous to glabrescent adaxially and in margins with 5–6-celled eglandular trichomes and with long, spreading, 8–12-celled, eglandular trichomes along the primary veins and in the vein axils abaxially; blades of major leaves 5–14 cm long, 2.5–5 cm wide, ovate, the major veins 6–7 on each side of mid-vein, the base asymmetric and attenuate, the margins entire, the apex acute; petioles 2.5–7.5 (–9.5) cm long, sparsely pubescent; blades of minor leaves 4–4.5 cm long, 1.5–2.5 cm wide, ovate, the major veins 4–5 on each side of mid-vein, the base rounded, the apex acute; petioles 0.8–1.5 cm long, sparsely pubescent. Inflorescences axillary, 1–2 flowers per axil, rarely 3-flowered; flowering pedicels 25–35 mm long, angled, erect, geniculate at anthesis, rarely slightly curved and pendent, glabrescent, the trichomes short, antrorse; pedicels scars inconspicuous. Buds globose, white with greenish-yellow spots. Flowers 5–6-merous. Calyx 2–2.5 mm long, 3–3.8 mm wide, subequal, cup-shaped, thick, strongly 5–10-nerved, green, glabrescent to moderately pubescent with simple and some forked eglandular trichomes, the calyx appendages 5 or up to 8, 0.8–1.2 mm long, 0.2 mm wide, subequal, thick, erect, cylindrical, inserted close to the margin, with the same pubescence as calyx tube. Corolla 10–13 mm long, 12–14 mm in diameter, thick, white with greenish-yellow spots and a white centre outside and within, rotate-stellate with interpetalar membrane, lobed ⅓ or less than of the way to the base, pubescent adaxially with short glandular trichomes (stalk 1–3-celled; head globose, peltate, unicellular) in the throat and base of the lobes, glabrous abaxially, the tube 4 mm long, the lobes 5–6 mm long, ca. 3 mm wide, triangular or broadly triangular, spreading, sometimes with sparse eglandular trichomes abaxially, the margins finely ciliate, the tips acute, papillate. Stamens five, equal; filaments 2.5–2.7 long, white, inserted on the corolla 1–1.2 mm from the base, with auricles fused to the corolla at point of insertion; anthers 2.5–2.7 mm long, ellipsoid, yellow, not connivent at anthesis. Gynoecium with ovary 2–2.2 mm long, ca. 1.5 mm in diameter, 3 (–4)-carpelar, light green, ovoid; ovules more than two per locule; nectary ca. 1.2 mm tall, yellowish-green; styles dimorphic, short style 1.25–1.5 mm long, not exceeding the anthers length, long style 2.5–2.8 mm long, at the same level of the anthers or slightly exserted, white, cylindrical; stigma ca. 0.2 mm long, 0.6–0.7 mm wide, discoid, yellowish-green. Berry 25–40 mm long, 30–55 mm in diameter, campanulate-umbilicate, the base truncate or obtuse, the apex rounded, blunt or pointed, light green when immature, orange-red or bright red at maturity, persistent, pungent, the pericarp thick, opaque, without giant cells (endocarp smooth); stone cells absent; fruiting pedicels 25–35 mm long, pendent, sometimes strongly curved, strongly angled, widened distally, green; fruiting calyx 10–16 mm in diameter, persistent, slightly accrescent, slightly campanulate, thick, strongly nerved, green, the appendages 1–2 mm long, spreading or slightly recurved. Seeds 30–68 per fruit, 3.5–4.2 mm long, 3–4 mm wide, C-shaped, pale yellow to yellow, the seed coat slightly reticulate (SM), minutely reticulate (SEM), the cells polygonal to irregular in shape, the lateral walls straight to wavy; embryo imbricate.

#### Distribution.

Capsicumbaccatumvar.umbilicatum is a cultigen and has been reported from Brazil, Colombia, Peru, Paraguay, Bolivia, Argentina and the Caribbean Islands (Fig. 27). The commercialisation of the fruits has increased enormously in different South American (Hunziker and Barboza 1998; Barboza, pers. obs.) and European markets (Hazen-Hammond 1993).

#### Ecology.

Capsicumbaccatumvar.umbilicatum is a cultivated plant adapted to wet and semi-shaded places.

#### Phenology.

Flowering from October to June; fruiting from December to July.

#### Chromosome number.

2*n* = 2x = 24 (Moscone 1999; Moscone et al. 2003).

#### Common names.

**Argentina**: Campanita (Salta, *Barboza 164*), Farolito, mitra (Distrito Federal, *Melchiore s.n.*); **Brazil**: Pimiento pitonga (Rio de Janeiro, *Scaldaferro 57*), Pimenta-de-cheiro-amarela (Amapá, *Pereira et al. 1830*), Chapéu-de-frade, Cabeça-de-frade, Chapéu-de-bispo, Pimenta-chapéu, Pimenta-de flor, Pimenta-de-cheiro (Roraima, Barbosa et al. 2006; São Paulo, Carvalho et al. 2006), Pimenta-chapeu-de-padre (São Paulo, *Bernacci 2816*), Pimentao fundo de garraba (Sturtevant 1888a); **Jamaica and other Caribbean Islands**: Jamaica hot (Miller and Harrison 1991); **Peru**: Rosasuchu (Andrews 1984); **Western Hemisphere**: Scallop pepper (Halsted et al. 1912), Rocotillo or Red squash (Andrews 1984).

#### Uses.

The mildly hot fruits of this cultivated variety are valued for their use in dishes with tropical fruits, sauces, Caribbean fish stews, curries and chutneys (Miller and Harrison 1991) or fresh as a condiment (Andrews 1984). The fruiting plant is very showy and so it is also grown as an ornamental (Barboza, pers. obs.).

#### Preliminary conservation assessment.

EOO (6,070,048 km^2^); AOO (64 km^2^). Capsicumbaccatumvar.umbilicatum is a cultigen in the Least Concern (LC) category.

#### Discussion.

**Figure 29.**
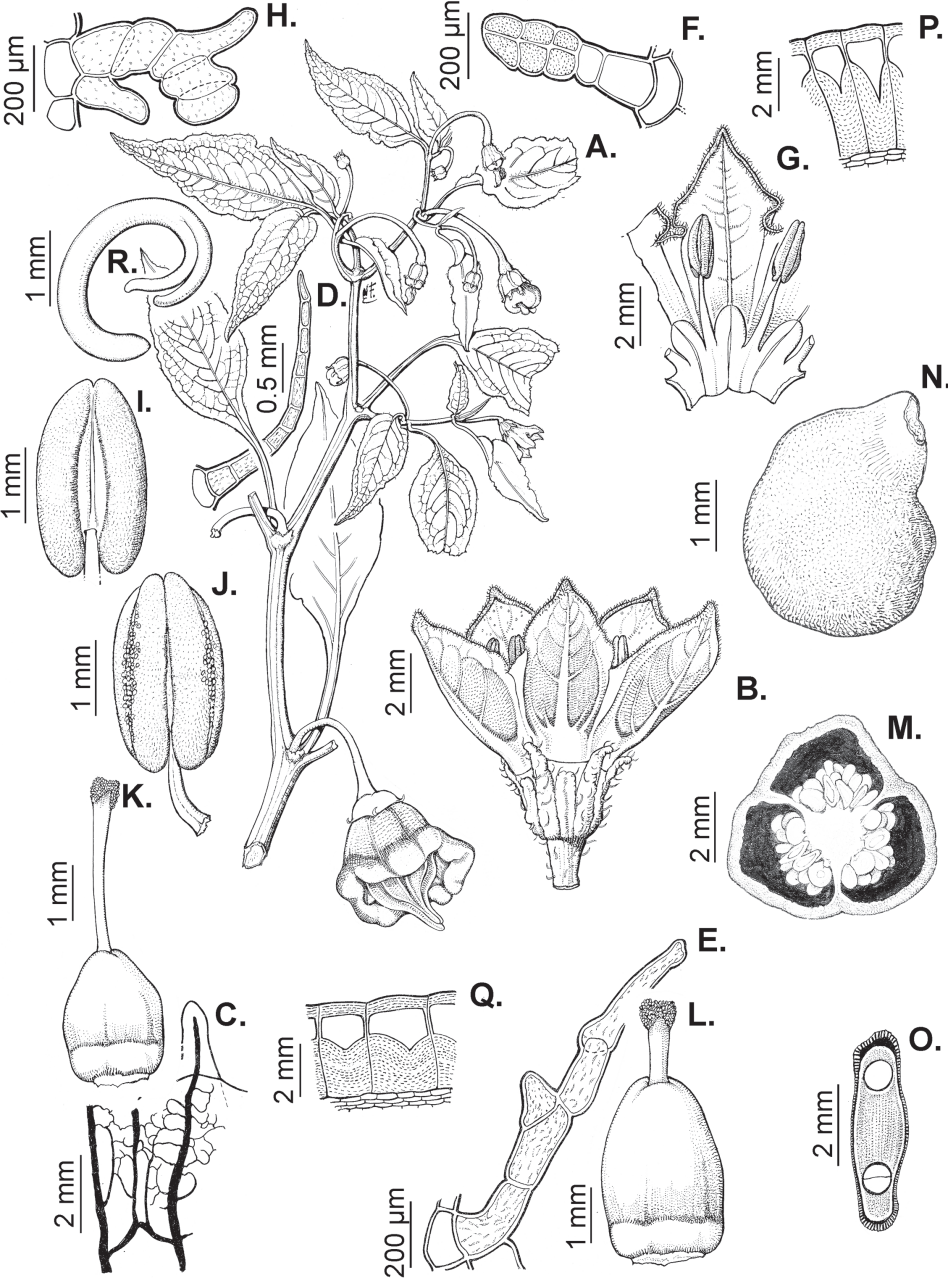
Capsicumbaccatumvar.umbilicatum**A** reproductive branch **B** flower **C** section of the calyx showing the venation **D, E** eglandular trichomes of the abaxial surface of the calyx **F** glandular trichome of the adaxial surface of the calyx **G** sector of opened corolla **H** eglandular trichome of the corolla lobes **I, J** anthers, in dorsal and ventral views, respectively **K** gynoecium with long style **L** gynoecium with short style **M** ovary trilocular, in cross section **N** seed **O** seed, in cross section **P** structure of seed coat at the seed margin **Q** structure of seed coat at the seed body **R** embryo. From *Rodríguez s.n.* (CORD 241). Drawn by N. de Flury. Published in Hunziker (1998), reproduced with permission.

**Figure 30.**
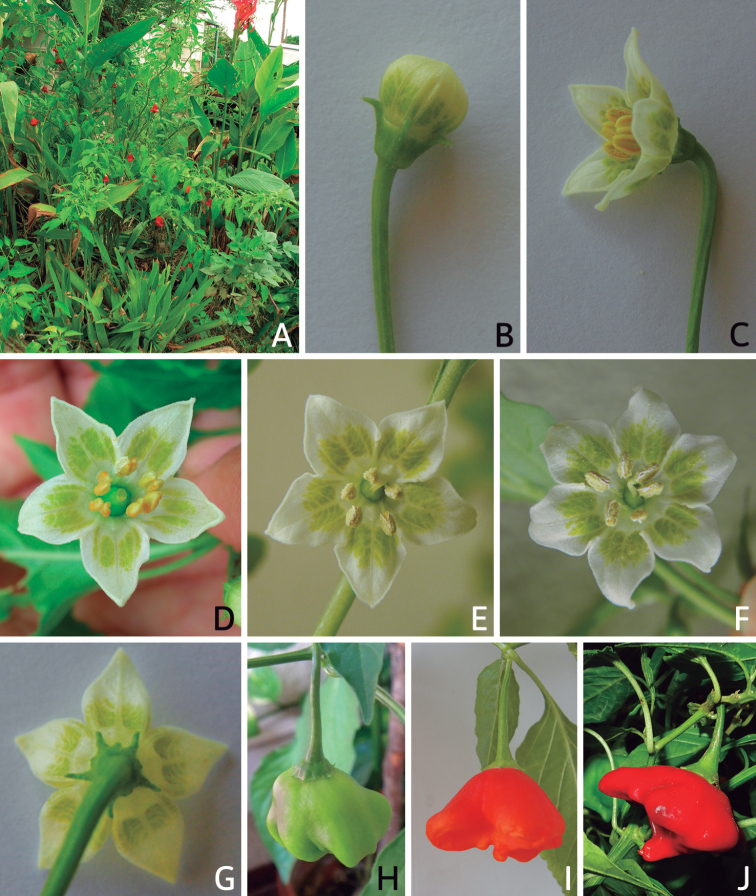
Capsicumbaccatumvar.umbilicatum**A** plant **B** flower bud **C** flower, lateral view **D** flower short-styled **E** flower long-styled **F** flower hexamerous **G** flower, seen from behind **H** immature fruit **I, J** mature fruits **A–I f**rom *Barboza 5163*, photos by G.E. Barboza **J** from *Carrizo García 101*, photo by C. Carrizo García.

Capsicumbaccatumvar.umbilicatum belongs to the Baccatum clade (Carrizo García et al. 2016). Amongst the broad morphological variation of the fruits within the domesticated *C.baccatum*, this cultigen was described from eastern Brazil (Rio de Janeiro), based on its noteworthy and unusual morphotype, consisting in a campanulate-umbilicate, red or orange-red, pendent fruit (Hunziker and Barboza 1998), the character on which the original description was based (Vellozo 1829). The campanulate-umbilicate shape refers to the fruit having an apex with an acute protrusion arising from a central depression at the middle height of the fruit (Figs 29D, 30I, J). Due to this unusual fruit shape and bright red colour, this plant has been repeatedly discussed in the chili pepper literature. After Vellozo (1829), Sendtner (1846) mentioned *C.umbilicatum* as a synonym of C.annuumvar.grossum. Then Irish (1898) submerged it under the ‘Red Wrinkled’ cultivar of *C.annuum*. Other authors illustrated and named this taxon as “Scallop pepper” (Halsted et al. 1912, Lam. 19, fig. 2) or as *C.chinense* cv. ‘rocotillo’ (Andrews 1984, pl. 31), respectively. It was Sturtevant (1888a) who provided a detailed description for this cultigen, based on plants obtained from seeds from Rio de Janeiro (Brazil) and stated that it was “an extremely well marked variety”. Hunziker and Barboza also grew plants of this cultivar from seeds from Paraguay and observed that the flowers were identical to the Andean domesticated *C.baccatum* populations. Hunziker and Barboza (1998) considered that the fruit shape was a particular variant within this taxon and proposed the name C.baccatumvar.umbilicatum. In addition to the shape of the fruits, the typical giant cells of the pericarp, a derived character present in most of the *Capsicum* species (Carrizo García et al. 2016), are missing in this cultivar. In addition, the presence of sclereids in the epicarp and endocarp distinguish this variety from the wild and other domesticated *C.baccatum* (Hunziker and Barboza 1998 and see Suppl. material 1: Appendix 1). Although molecular studies (Albrecht et al. 2012b; Carrizo García et al. 2016; Scaldaferro et al. 2019) demonstrated the close affinities between domesticated lines of *Capsicumbaccatum*, they were not sufficiently conclusive to support combining the domesticated varieties into one entity. Therefore, for those individuals that fit this distinctive and unusual fruit morphology and anatomy, we prefer to maintain the name C.baccatumvar.umbilicatum.

#### Specimens examined.

See Suppl. material 4: Appendix 4.

### ﻿3. Capsicum benoistii

Taxon classificationPlantaeSolanalesSolanaceae

Hunz. ex Barboza, PLoS ONE 14(1): 4. 2019.

9B31C9D2-BAD1-59F7-B333-9944CE1BD701

Fig. 31

#### Type.

Ecuador. Tungurahua: Baños, 3 Apr 1931, *M.R. Benoist 4204* (holotype: P [P04023406]).

#### Description.

Erect shrubs with few branches. Young stems 3-angled, light brown, glabrous or sparsely pubescent with appressed-antrorse, simple, uniseriate, 3–5-celled, eglandular trichomes 0.3–1.1 mm long; bark of older stems dark brown, angled, glabrous; lenticels absent. Sympodial units difoliate, the leaves geminate; leaf pair markedly unequal in size, subequal in shape. Leaves membranous, discolorous, dark green above, light green beneath, glabrous or with sparse trichomes adaxially and abaxially, similar to those of the stems, more abundant on main veins; blades of major leaves 8.5–12 cm long, 2.8–6 cm wide, ovate or elliptic, the major veins 4–5 (–6) on each side of mid-vein, the base asymmetric and attenuate, the margins entire, the apex long-acuminate; petioles 0.5–1 cm long, glabrous or glabrescent; blades of minor leaves 2.4–6 cm long, 1.7–4 cm wide, ovate or elliptic, the major veins 3–4 on each side of mid-vein, the base asymmetric and rounded, the margins entire, the apex acute or rounded; petioles 0.1–1 cm long, glabrescent or sparsely pubescent. Inflorescences axillary, 3–6 flowers per axil; flowering pedicels 13–20 mm long, angled, filiform, pendent, non-geniculate at anthesis, moderately to densely pubescent, the eglandular trichomes long, spreading to antrorse; pedicels scars inconspicuous. Buds ovoid, colour unknown. Flowers 5-merous. Calyx 2–2.5 mm long, ca. 5 mm wide, cup-shaped, thick, colour unknown, moderately pubescent with the same trichomes as pedicels, the calyx appendages 5, 2.5–3.5 mm long, ca. 0.5 mm wide, equal or subequal, thick, erect, subulate, inserted close to the margin, with the same pubscence as calyx tube. Corolla ca. 12–13 mm long, thick, deeply stellate without interpetalar membrane, lobed nearly to the base, glabrous adaxially and abaxially, the tube ca. 3 mm long, the lobes ca. 9 mm long, ca. 2 mm wide, narrowly triangular, erect, the margins and the tips pubescent. Stamens five, equal; filaments 3–3.2 mm long, inserted on the corolla 1.5 mm from the base, with auricles fused to the corolla at the point of insertion; anthers ca. 3 mm long, ellipsoid, not connivent at anthesis. Gynoecium with ovary 1.3–1.7 mm long, 1.2–1.5 mm in diameter, subglobose; ovules more than two per locule; nectary ca. 0.3 mm tall; styles dimorphic, long style ca. 6.5 mm, short style ca. 3.6 mm, clavate; stigma 0.3 mm long, 0.5 mm wide, globose. Berry and seeds unknown.

**Figure 31.**
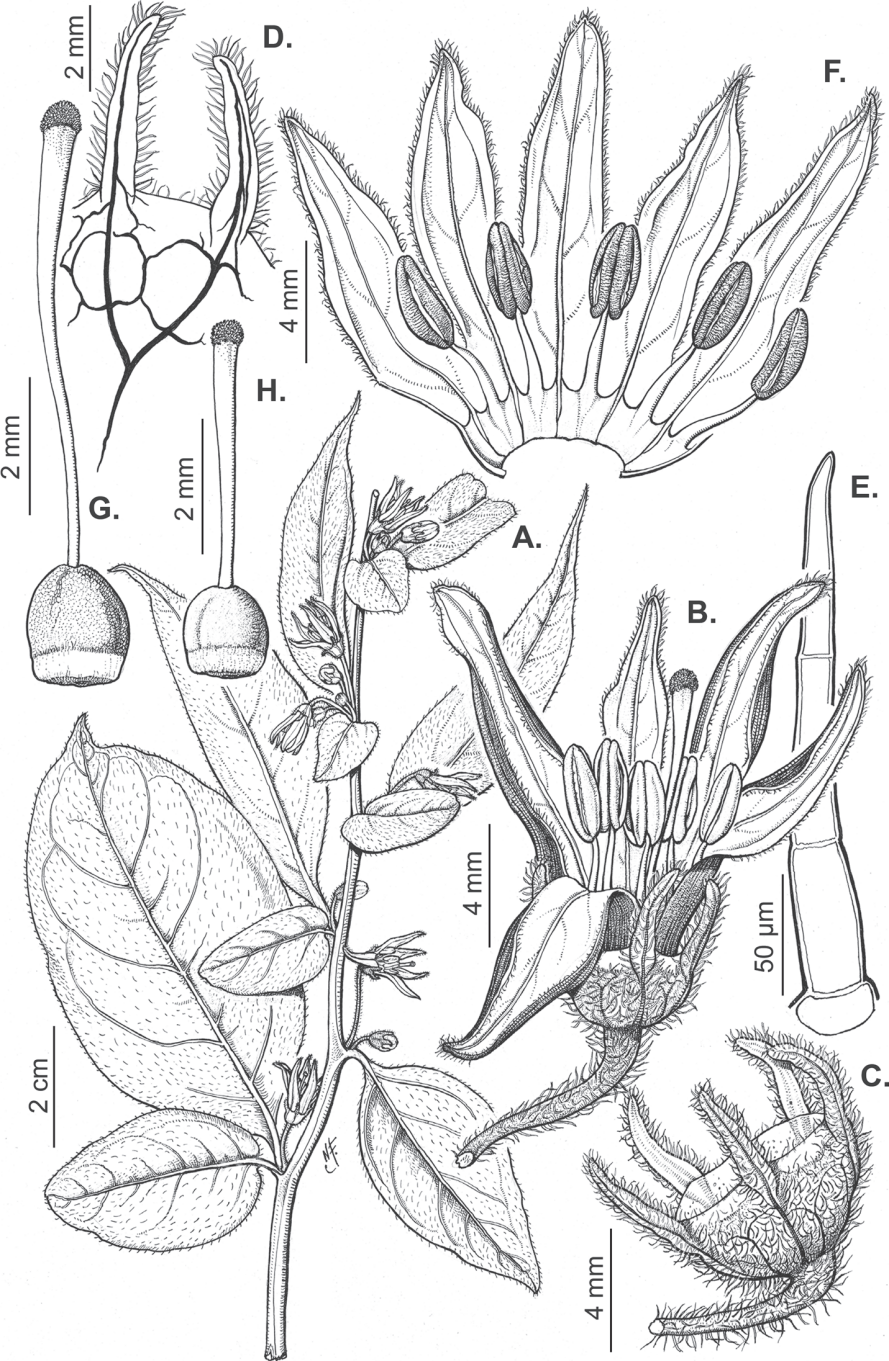
*Capsicumbenoistii***A** flowering branch **B** flower **C** calyx **D** section of the calyx showing the venation **E** eglandular trichome of the calyx **F** opened corolla **G** gynoecium with long style **H** gynoecium with short style. From *Benoist 4204.* Drawn by N. de Flury. Published in Barboza et al. (2019), reproduced with permission.

#### Distribution.

**Figure 32.**
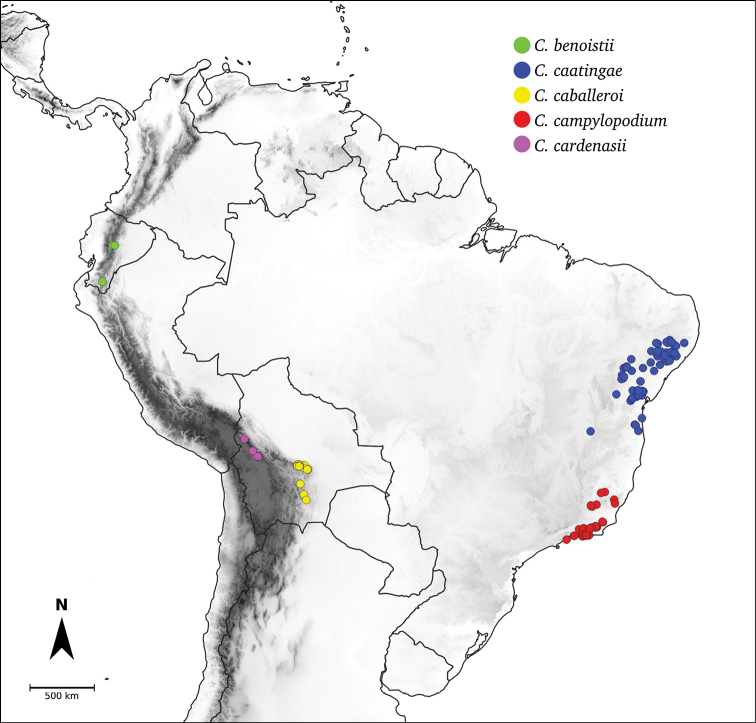
Distribution of *C.benoistii*, *C.caatingae*, *C.caballeroi*, *C.campylopodium* and *C.cardenasii*

*Capsicumbenoistii* is endemic to a restricted area in central-southern Ecuador (Provinces of Tungurahua and Loja) (Fig. 32).

#### Ecology.

*Capsicumbenoistii* grows in thickets in montane forests, between 1,500 and 2,600 m elevation.

#### Phenology.

Flowering from March to May. Fruiting time unknown.

#### Chromosome number.

Not known.

#### Common names.

None recorded.

#### Uses.

None recorded.

#### Preliminary conservation assessment.

EOO (2,627.651 km^2^); AOO (12 km^2^). Considering the extent of occurrence, the area of occupancy, the few localities (3) where it was collected and the decline observed in its geographic range, we assign *C.benoistii* the Endangered (EN; B1+2ab(i,ii) category. The species has not been collected since 1978 despite recent intensive field explorations in the same locations (Barboza et al. 2019).

#### Discussion.

The affinities of *C.benoistii* have not yet been explored and, due to the lack of data on some morphological characters, it is not assigned to any of the recognised clades (Barboza et al. 2019). This species is poorly known and information about corolla colour, fruit and seed characters and chromosome number are still lacking. However, *C.benoistii* is distinctive in its deeply-lobed stellate corolla (lobes three times longer than the tube, Fig. 31B, F) and in the presence of heterostylous flowers (Fig. 31G, H). These features, plus the short flowering pedicels (13–20 mm long), distinguish *C.benoistii* from *C.geminifolium*, a morphological similar species, which has campanulate corollas that are lobed less than 1/3 of the way to the base and homostylous flowers.

The presence of heterostylous flowers in *C.benoistii* is unusual amongst *Capsicum* species. It has been reported in *C.chinense* (Peña-Yam et al. 2019), *C.baccatum* (Hunziker 2001; Barboza 2013), in many cultivars (Perera and Poulos 1993) and observed in *C.pubescens* (see Fig. 103I–K).

#### Specimens examined.

See Suppl. material 4: Appendix 4.

### ﻿4. Capsicum caatingae

Taxon classificationPlantaeSolanalesSolanaceae

Barboza & Agra, Syst. Bot. 36 (3): 769. 2011.

B362E069-19B4-54C6-9A91-96EF6CE36333

Figs 33 , 34

#### Type.

Brazil. Bahía: Cachoeira, Estação da EMPASA, Vale dos Rios Paraguaçu e Jacuipe, 12°32'39"S, 39°05'00"W, 40–120 m elev., Jun 1980, *P. do Cavalo et al. 162* (holotype: HUEFS [HUESF000001216, acc. # 00920]; isotypes: ALCB [ALCB000131, acc. # 07938], RB [RB00461411, acc. # 263323]).

#### Description.

Small trees or erect shrubs 2–4 (–6) m tall, the main stem thick, 2.5–5 cm in diameter at base and with indefinite growth up to 5 m high, few branched, the branches slender or scandent. Young stems 3–4-angled, rigid, grey, glabrescent or, more rarely, sparsely pubescent with antrorse, simple, uniseriate, 3–7-celled, eglandular trichomes 0.2–0.9 mm long, sometimes furcate trichomes 0.7–0.9 mm long or minute, simple, glandular trichomes, ca. 0.1 mm long; nodes solid, green; bark of older stems grey or brown, glabrous; lenticels light brown. Sympodial units difoliate, the leaves geminate; leaf pair unequal in size and similar or dissimilar in shape. Leaves membranous or papery, slightly discolorous, glabrescent to moderately pubescent on both sides, with antrorse, 5–8-celled, eglandular trichomes 0.4–1.2 mm long, sometimes with branched trichomes 0.7–0.9 mm long and small glandular hairs (stalk short, 2–3-celled; head multicellular or unicellular), especially on veins abaxially; blades of major leaves 4–11.5 (–20) cm long, 1.5–2.4 (–8.5) cm wide, ovate to elliptic, the major veins 4–5 on each side of mid-vein, the base unequal and short-attenuate, the margins entire, the apex acute or somewhat acuminate; petioles (0.5–) 0.7–2.5 cm long, moderately pubescent adaxially; blades of minor leaves 1.5–2 cm long, 0.7–1.3 cm wide, ovate, the major veins 3–4 on each side of mid-vein, the base short-attenuate or truncate, the margins entire, the apex acute; petioles 0.2–0.5 cm long, moderately pubescent adaxially. Inflorescences axillary, congested, 5–13 (–20 or more) flowers per axil; flowering pedicels 7–21 (–28) mm long, terete, pendent, non-geniculate at anthesis, green with violet tones, glabrescent to moderately pubescent, the eglandular trichomes short, antrorse; pedicels scars conspicuous, somewhat corky. Buds globose to ellipsoid, cream, greenish-white or purple at the apex. Flowers 5-merous. Calyx 1.2–1.7 (–2) mm long, 2–2.5 mm wide, cup-shaped, circular in outline, thin, weakly 5-nerved, green, greenish-purple or purple, sparsely pubescent with the same antrorse eglandular and glandular trichomes of the leaves, without appendages. Corolla 4.5–6 (–8) mm long, lilac or purple, greenish-yellow at the base outside, with different tones of purple and a narrow white marginal band in the lobes and greenish-yellow to yellowish-white centre within, stellate with narrow interpetalar membrane, lobed nearly halfway to the base, pubescent adaxially with a continuous ring of small glandular trichomes (stalk short, 1–2-celled; head globose, unicellular) in the throat and base of the lobes, glabrous abaxially, the tube 2.8–3.2 mm long, the lobes 2.9–3.5 mm long, 1.7–2.4 mm wide, broadly triangular, spreading, the margins involute and finely ciliate, the tip cucullate, papillate. Stamens five, equal; filaments (0.8–)1.1–1.75 mm long, greenish-white or white, inserted on the corolla ca. 2 mm from the base, with auricles fused to the corolla at the point of insertion; anthers 1.4–2.1 mm long, ellipsoid, light green or yellow, not connivent at anthesis. Gynoecium with ovary 1.1–1.4 in diameter, pale green, subglobose; ovules more than two per locule; nectary ca. 0.3–0.4 mm tall; styles homomorphic, (4.3–) 4.6–4.8 mm, exserted ca. 1 mm beyond the anthers, pale yellow or cream, clavate; stigma ca. 0.6 mm in diameter, globose or 0.15 mm long, 0.6 mm wide, discoid, light green. Berry (5–) 7–11 mm in diameter, globose, slightly flattened at the apex, green or yellowish-green when immature, red at maturity, deciduous, pungent, the pericarp thick, opaque, with giant cells (endocarp alveolate); stone cells absent; fruiting pedicels (15–) 20–25 mm long, pendent, terete, inflated, strongly widened distally and with a constriction at the junction with the calyx, green or purple; fruiting calyx 3–4 mm in diameter, persistent, not accrescent, discoid, 5 (–10)-nerved, the margin entire or sometimes easily torn, green or purple. Seeds (9–) 11–17 per fruit, 3.2–3.7 mm long, 2.2–2.8 mm wide, C-shaped, pale yellow, the seed coat reticulate and tuberculate at margins (SM), cerebelloid (SEM), the cells irregular in shape, the lateral walls wavy at margins and strongly sinuate in the central zone; embryo imbricate.

#### Distribution.

*Capsicumcaatingae* is endemic to the north-eastern States of Brazil (Alagoas, Bahia, Pernambuco Sergipe and northern Minas Gerais States, Fig. 32). A beautiful specimen has been cultivated on the campus of the Federal University of Viçosa (Minas Gerais) for more than 30 years (seeds from Bahia).

#### Ecology.

*Capsicumcaatingae* is a xerophytic species usually found in the margins of arid open Caatinga forests (Seasonal Deciduous and Seasonal Residual Forests) and in the anthropogenic Caatinga, more rarely in degraded humid forests (Floresta Estacional Decidual or Semidecidual). It is quite common in savannahs or amongst granitic and gneissic outcrops (‘inselbergs’), growing with thorny, deciduous arboreal and shrubby vegetation, between 100 and 950 m elevation.

#### Phenology.

Flowering from December to August; fruiting from February to October.

#### Chromosome number.

*n* = 12 (Pozzobon and Schifino-Wittmann 2006, as *C.parvifolium*); 2*n* = 2x = 24 (Moscone 1993; Pozzobon et al. 2006; Moscone et al. 2007; all count as *C.parvifolium*).

#### Common names.

**Brazil**. Caraibera (Alagoas, *Silva & Moura 1586*), Murta (Sergipe, *Silva 287*), Pimenta brava (Bahía, *Pinto & Bautista 104*), Semente-de-macaco (Alagoas, *Oliveira 7*).

#### Uses.

None recorded.

#### Preliminary conservation assessment.

EOO (270,442.695 km^2^); AOO (276 km^2^). The large extent of occurrence and the number of localities where *C.caatingae* was collected indicate Least Concern (LC) category. Given its highly specialised habitat limited to the Caatinga Biome, some subpopulations may be adversely affected because deforestation has intensified rapidly in recent years due to the consumption of native firewood for domestic and industrial purposes, over-grazing and changes in the ecosystem due to pasture and agricultural expansion (MMA-Brazil 2020).

#### Discussion.

*Capsicumcaatingae* is a member of the Caatinga Clade (Carrizo García et al. 2016) and is closely related to *C.parvifolium*, with which it is sympatric in Brazil; the two species have been confused in literature (Moscone 1993; Hunziker 2001; Barboza and Bianchetti 2005; Pozzobon and Schifino-Wittmann 2006; Pozzobon et al. 2006; Moscone et al. 2007) and in herbaria (Barboza, pers. obs.). Barboza et al. (2011) clarified this confusion and re-circumscribed *C.parvifolium*.

**Figure 33.**
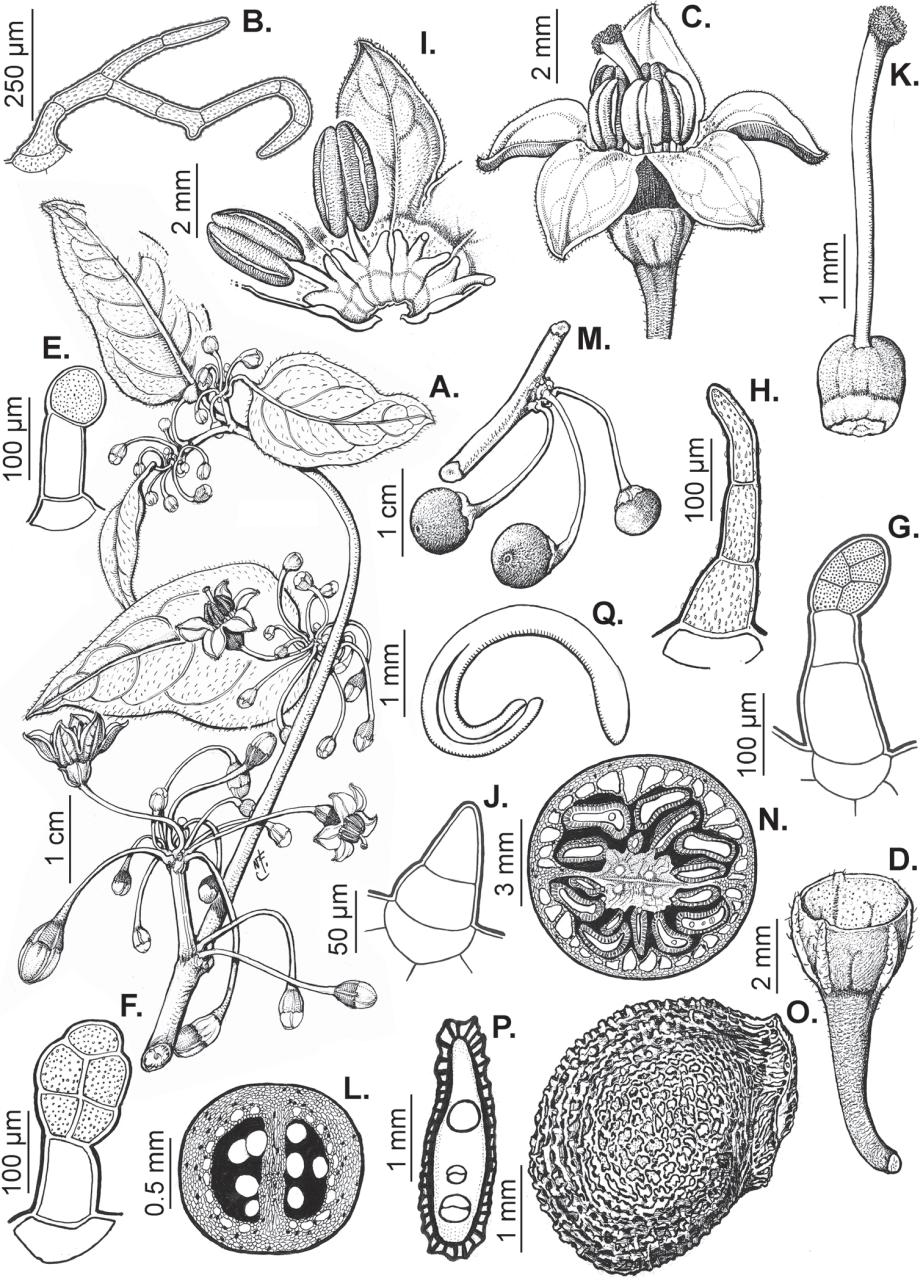
*Capsicumcaatingae***A** flowering branch **B** branched trichome from leaf **C** flower **D** calyx **E, F, G** glandular trichomes of the calyx and leaf venation **H** eglandular trichome of the calyx **I** sector of opened corolla **J** eglandular trichome of the corolla lobes **K** gynoecium **L** ovary, in cross section **M** node of a fruiting branch **N** fruit, in cross section **O** seed **P** seed, in cross section **Q** embryo. From *Hunziker 25233*. Drawn by N. de Flury. Published in Barboza et al. (2011), reproduced with permission.

**Figure 34.**
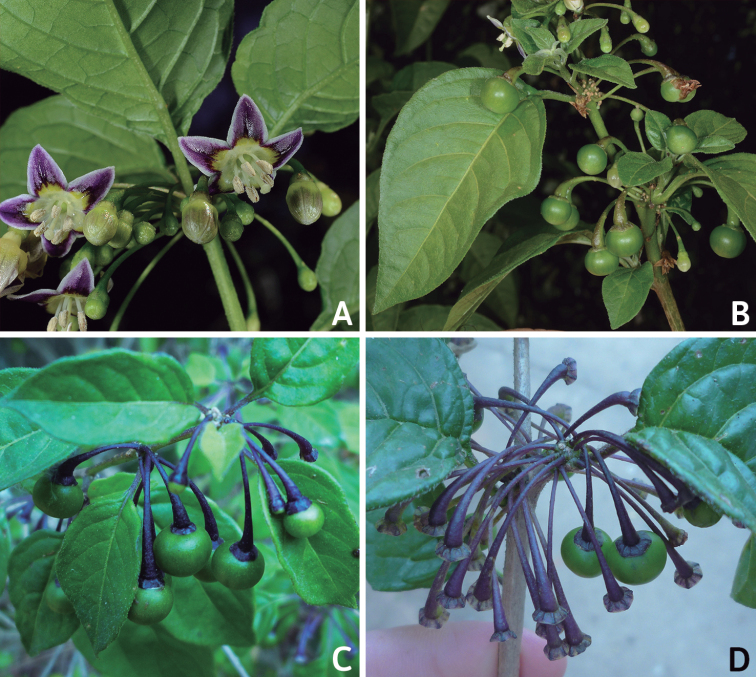
*Capsicumcaatingae***A** flowering branch **B** young fruiting branch **C** immature fruits (note the purple pedicels) **D** node of a fruiting branch, some fruits already fallen down. No specimen voucher. Photos taken in Federal University of Viçosa (Minas Gerais) by C. dal Zovo (Associazione PepperFriends).

*Capsicumcaatingae* is an unusual species with a combination of uncommon features rarely found amongst its congeners: arborescent habit, with indefinite growth of the main stem reaching up to 6 m high (15 m fide *Bautista & Pinto 1023*), very congested inflorescences with up to 20 or more flowers per node, corolla with varied colours (lobes white edged, then deep purple or variations of purple colour, tube with greenish-yellow to yellowish-white centre) and terete and inflated fruiting pedicels with a strong annular constriction at the junction with the calyx base (Fig. 34), similar to *C.chinense* and a few other species (*C.minutiflorum*, *C.lanceolatum* and *C.regale*).

*Capsicumcaatingae* differs from *C.parvifolium* in the absence of calyx appendages (five appendages in *C.parvifolium*), the number of flowers per node (up to 20 or more flowers vs. not more than seven flowers in *C.parvifolium*) and the fruit and seed colour (red berry with pale yellow seeds vs. greenish-golden yellow translucent berry with brownish-black seeds in *C.parvifolium*). *Capsicumcaatingae* is sometimes sympatric with *C.longidentatum* from which it differs by its arborescent growth (vs. shrubby growth), the indumentum mostly of simple trichomes (vs. indumentum of branched and dendritic trichomes), the lack of calyx appendages (vs. five, rarely six, calyx appendages), the mostly purple corolla (vs. corolla white with greenish-yellow spots) and the pungent red fruit with pale yellow seeds (vs. non-pungent probably yellowish-green fruit with brown to brownish-black seeds).

#### Specimens examined.

See Suppl. material 4: Appendix 4.

### ﻿5. Capsicum caballeroi

Taxon classificationPlantaeSolanalesSolanaceae

M.Nee, Brittonia 58 (4): 323. 2006.

64187248-09A4-59C8-9EAA-3E5423A958B8

Figs 35 , 36

#### Type.

Bolivia. Santa Cruz. Prov. Caballero: Parque Nacional Amboró, Cerro Bravo, 10 km al N de Comarapa, 17°49.5'S, 64°32.5'W, 2400–2500 m elev., 7–10 Apr 1994, *I. Vargas C. & J.M. Camacho 3118* (holotype: USZ; isotypes: CORD [CORD00003917], MO [MO-1921597, acc. # 5959888], NY [00745836], US [00902045, acc. # 3520370]).

#### Description.

Erect shrubs or more rarely small trees 0.70–5 (–7) m tall, with the main stem thick, 2.5–3 cm in diameter at base, few to much branched above. Young stems angled, rigid, green, glabrous to sparsely pubescent, with appressed-antrorse, simple, uniseriate, 3–4-celled, eglandular trichomes 0.07–0.2 mm long; nodes solid, green; bark of older stems brown or brownish-grey, glabrous; lenticels few, light brown. Sympodial units difoliate, the leaves geminate; leaf pair unequal in size, similar or dissimilar in shape. Leaves coriaceous, slightly discolorous, glabrous on both sides or sparsely pubescent along the mid-vein abaxially, with simple, eglandular trichomes 0.2–0.4 mm long; blades of major leaves 4–13.5 cm long, 1.5–4 cm wide, elliptic, the major veins 4–5 on each side of mid-vein, the base acute to short-attenuate, the margins entire slightly revolute, the apex acuminate; petioles 0.2–0.8 cm long, glabrous; blades of minor leaves 2–4.5 cm long, 1–2 cm wide, elliptic or ovate, major veins 2–3 on each side of mid-vein, the base short-attenuate, the margin entire slightly revolute, the apex acute; petioles 0.3–0.5 cm long, glabrous. Inflorescences axillary, 1–2 flowers per axil; flowering pedicels 20–40 (–50) mm long, terete, slightly pendent to pendent, non-geniculate at anthesis, green, glabrous; pedicels scars inconspicuous. Buds ellipsoid, yellow. Flowers 5-merous. Calyx 1.5–3 mm long, ca. 3 mm wide, cup-shaped, thick, pale green, sparsely pubescent with the same antrorse eglandular trichomes of the young stems, the calyx appendages 10, unequal, the five main appendages 1–3.2 mm long, the five secondary appendages 0.8–2 mm long, erect, subulate, inserted close to the margin. Corolla (10–) 11–14 (–18) mm long, 4–6 mm in diameter, thick, entirely pure yellow or lemon-yellow, campanulate without interpetalar membrane, shallowly 5-lobed; glabrous adaxially and abaxially, the tube 7–10 mm long, the lobes 3–4 mm long, ca. 2 mm wide, narrowly triangular, recurved, the margins involute and finely ciliate, the tips deeply acute, papillate. Stamens five, equal; filaments 4–6 mm long, cream, inserted on the corolla 1–2 mm from the base, with auricles fused to the corolla at the point of insertion; anthers 2–3 mm long, ellipsoid, yellow, not connivent at anthesis. Gynoecium with ovary ca. 2 mm long, 1.5 mm in diameter, pale green, ovoid; ovules more than two per locule; nectary ca. 0.4–0.5 mm tall; styles homomorphic, 7–9 mm, scarcely exserted beyond the anthers, cream, clavate; stigma ca. 1 mm in diameter, whitish, capitate. Berry (9–) 10–16 mm in diameter, globose, slightly flattened at the apex, pale green or white when immature, bright red at maturity, persistent, pungent, the pericarp thick, opaque, with giant cells (endocarp alveolate); stone cells absent; fruiting pedicels 25–50 mm long, pendent, curved, terete, strongly widened distally and with a constriction at the junction with the calyx, green; fruiting calyx 3–5 mm in diameter, persistent, not accrescent, discoid, green, the appendages (1–) 3–5 mm long, appressed to the berry. Seeds 5–21 per fruit, 3–4 (–5) mm long, 3.8–5 mm wide, C-shaped or teardrop-shaped, pale yellow or nearly white, the seed coat smooth and reticulate at margins (SM), cerebelloid-reticulate (SEM), the cells irregular and polygonal in shape, the lateral walls strongly sinuate to nearly straight at the seed margin; embryo imbricate or coiled.

#### Distribution.

*Capsicumcaballeroi* is an endemic species from central Bolivia (Santa Cruz, Cochabamba and Chuquisaca Departments, Fig. 32).

#### Ecology.

*Capsicumcaballeroi* is a rare element of montane cloud forests (Yungas and Bosque Tucumano-Boliviano Montano), found in very moist shaded wooded quebradas or at the margin of the forest between 1,000 and 2,600 m elevation.

#### Phenology.

Flowering and fruiting probably all year long; a peak of flowering was observed in November to early January and fruiting from January to June.

#### Chromosome number.

2*n* = 2x = 24 (*Barboza et al. 3655*, see Table 2).

#### Common names.

**Bolivia.** Aribibi (Cochabamba, *Fernández T. et al. 2007*), Ají de monte (Santa Cruz, *Vargas C. & Prado 1282*), Ulupica de yunga (Santa Cruz, *Vargas C. et al. 1343*).

#### Uses.

None recorded.

#### Preliminary conservation assessment.

EOO (14,005.388 km^2^); AOO (84 km^2^). *Capsicumcaballeroi* grows mostly in the cloud forest of the Amboró National Park and peripheral areas where the population consists of few individuals; although found in a relatively large geographical area, we observed a severe decline of both the EOO and the AOO of this species due to the continuing indiscriminate deforestation occurring in the last years; in this way, we consider *C.caballeroi* under threat and assign the Vulnerable category (VU; B1ab(ii,iii)).

**Figure 35.**
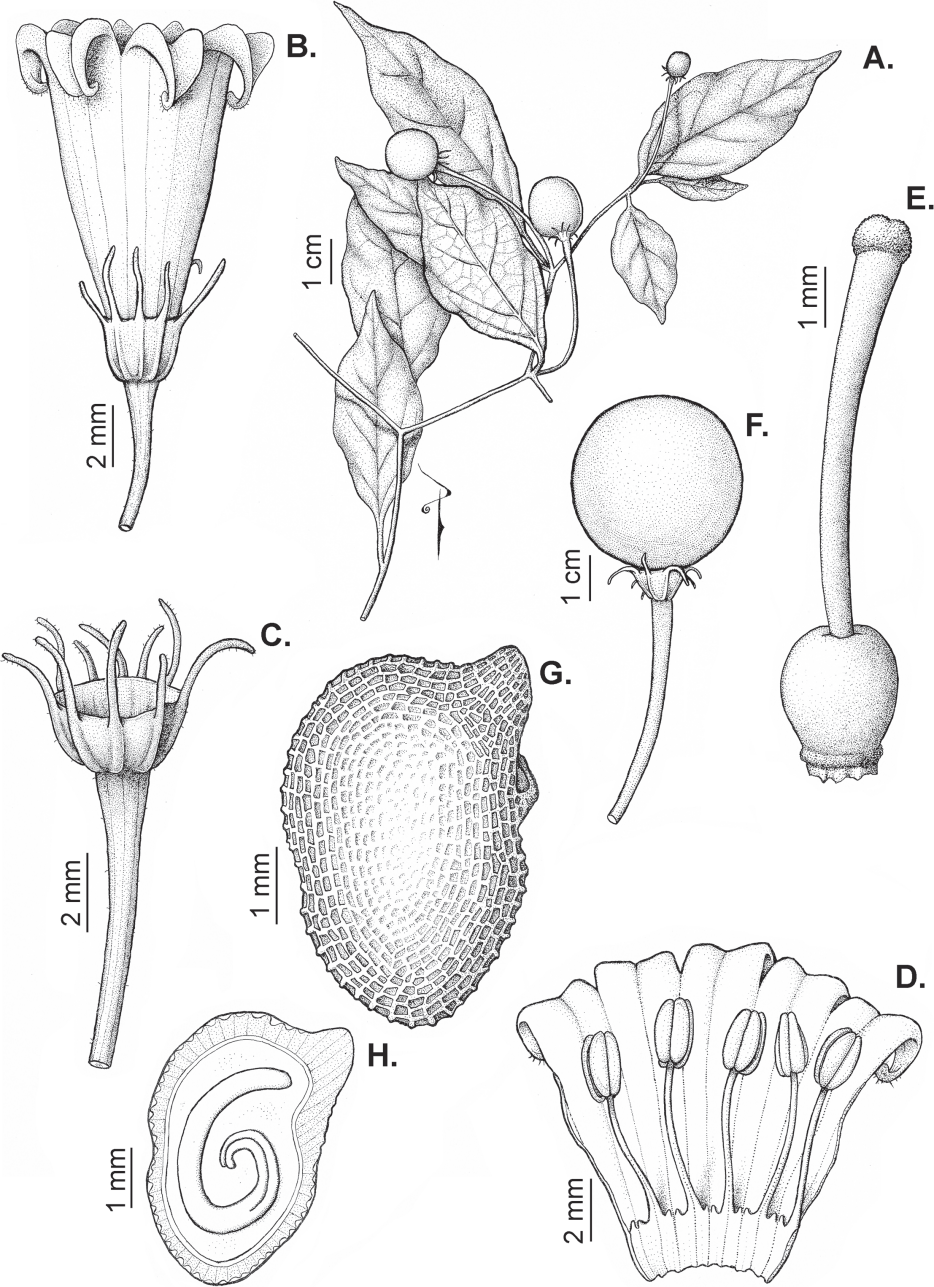
*Capsicumcaballeroi***A** fruiting branch **B** flower **C** calyx **D** opened corolla **E** gynoecium **F** fruit **G** seed **H** seed, in longitudinal section. From *Wood 11102*. Drawn by P. Peralta.

#### Discussion.

**Figure 36.**
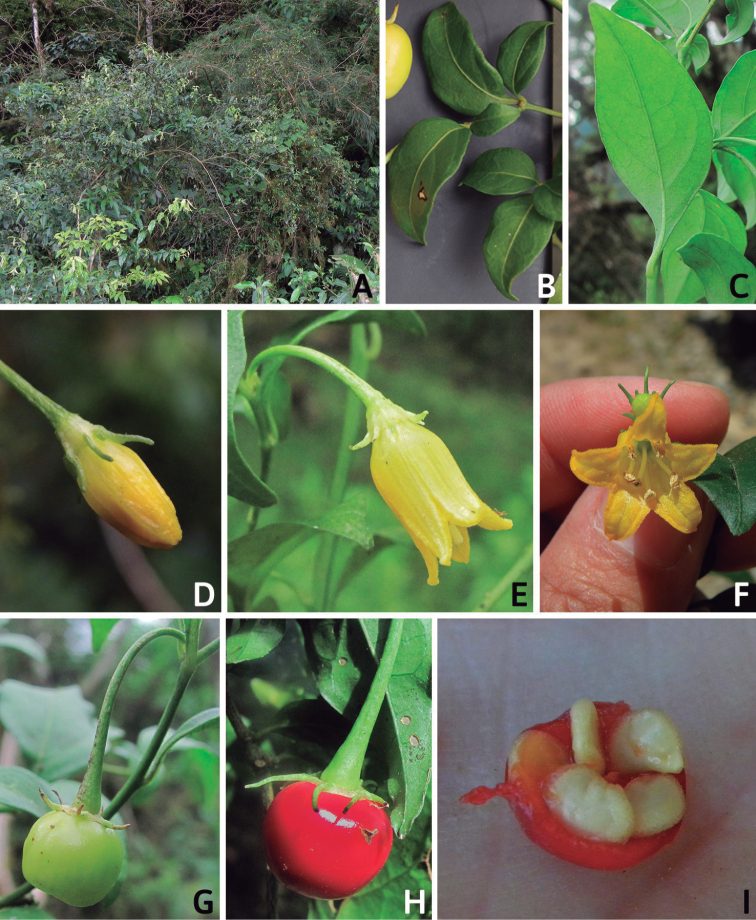
*Capsicumcaballeroi***A** plant **B** leaf pairs **C** major leaf **D** flower bud **E** flower **F** flower, in front view **G** immature fruit **H** mature fruit **I** mature fruit, in cross section, showing the seeds **A** from *Barboza et al. 4907***B, C, E, F, H***Barboza et al. 3655***D, G, I** from *Barboza et al. 4908*. Photos by G.E. Barboza and S. Leiva González.

*Capsicumcaballeroi* belongs to the Bolivian clade (Carrizo García et al. 2016; Barboza et al. 2019). It is easily distinguishable from any other Bolivian species in its calyx with 10 unequal appendages, its yellow tubular-campanulate pendent corollas, its long flowering and fruiting pedicels and its large, bright red fruits and pale seeds (Fig. 36D, I).

*Capsicumcaballeroi* is morphologically most similar to *C.piuranum*, an endemic species from northern Peru and can be distinguished from that species in its calyx (green calyx with 10 unequal linear appendages vs. purple or greenish-purple calyx with five equal subulate appendages), its corolla lobes (recurved vs. spreading) and its fruits and seeds (red pungent fruits with pale yellow or nearly white seeds vs. orange to red non-pungent fruits with dark brown to black seeds). The fruits and the seeds of *C.caballeroi* are larger than those of *C.piuranum*. *Capsicumcaballeroi* is sympatric with *C.minutiflorum*; they share yellow corollas and red fruits, but are distinguished by the coriaceous leaves, calyx with 10 unequal appendages, campanulate and larger corollas (10–14 mm long) and fruits (9–16 mm in diameter) of *C.caballeroi* vs. the membranous leaves, calyx with five equal or subequal appendages, stellate, smaller corollas (6.5–8.5 mm long) and fruits (7–10 mm in diameter) of *C.minutiflorum* (Fig. 87).

#### Specimens examined.

See Suppl. material 4: Appendix 4.

### ﻿6. Capsicum campylopodium

Taxon classificationPlantaeSolanalesSolanaceae

Sendtn., Fl. Bras. (Martius) 10: 144. 1846.

38E71A26-17B6-5352-BCA8-7A1E8E49203E

Figs 37 , 38

Capsicum gracilipes Dunal, Prodr. [A. P. de Candolle] 13(1): 418. 1852. Type. [Brazil]. Rio de Janeiro, 1834, *C. Gaudichaud 513* (lectotype, designated here: G-DC [G00131901]; isolectotypes: F [F neg. F0BN002869], MPU [MPU013436 fragment], P [P00410015, P00410016]).
Capsicum salicifolium Dunal, Prodr. [A. P. de Candolle] 13(1): 418. 1852. Type. Brazil. Rio de Janeiro: “In provinciã Rio de Janeiro, Serra dos Órgãos”, Oct. 1833, *A.-C. Vauthier 528* (lectotype, designated here: G-DC [G00131881]; isolectotypes: CORD [CORD00006953], F [F neg. 6846 ex G-DC + F0093724F fragment, acc. # 644821], GH [GH00077007], MPU [MPU023040 fragment], P [P00410013, P00410014]).

#### Type.

Brazil. “Brasilia”, [no date], *F. Sellow 6* (lectotype, designated by Barboza 2011, pg. 29: P [P00410022]; isolectotypes: B [destroyed, F neg. 2865], CORD [CORD00006952, fragment of lectotype], K [K000585891 right plant], F [v0076865F, acc. # 648993, fragment of holotype], LE [LE01072483]).

#### Description.

Erect subshrubs or shrubs, 0.5–2 m tall, with the main stem thick, up to 2.5 cm in diameter at base, much branched above, the branches dichotomously spreading in a typical “zig-zag” appearance. Young stems striate, fragile, green, glabrous to glabrescent, with antrorse, curved, simple, uniseriate, 3–4-celled eglandular trichomes 0.3–0.5 mm long; nodes solid, green; bark of older stems dark brown, glabrous; lenticels few, light brown. Sympodial units difoliate, the leaves geminate; leaf pair unequal in size, similar in shape. Leaves membranous, slightly discolorous, green or dark green above, light green beneath, glabrous or sparsely pubescent with appressed-antrorse, 3–5-celled, eglandular trichomes 0.2–0.4 mm long on both surfaces; blades of major leaves 4–11.5 (–20) cm long, 1.5–2.4 (–8.5) cm wide, elliptic to ovate, the major veins 5–7 on each side of mid-vein, the base attenuate and unequal, the margins entire, the apex acuminate to long-acuminate; petioles 0.5–1.7 cm, glabrescent or glabrous; blades of minor leaves 1.7–3.5 (–4.5) cm long, 0.5–2 cm wide, elliptic to ovate, the major veins 3–4 on each side of mid-vein, the base attenuate, the margins entire, the apex acute; petioles 0–0.5 cm, glabrous. Inflorescences axillary, 2–5 (–7) flowers per axil, rarely flowers solitary; flowering pedicels 9–14 mm, very thin, delicate, terete to slightly striate, erect to slightly spreading, geniculate at anthesis, entirely green or reddish basally, glabrescent, the eglandular trichomes short, antrorse; pedicels scars inconspicuous. Buds ovoid, cream or with greenish-yellow spots. Flowers 5-merous. Calyx (1–) 1.2–1.6 mm long, 1.4–1.5 mm wide, hemispherical, circular in outline, very thin, green, glabrous or rarely glabrescent, without appendages. Corolla 4.5–6.5 (–8) mm long, (6–) 6.4–7.5 (–11) mm in diameter, mostly cream outside, white or cream with two large golden yellow or ochraceous spots on each lobe and part of the limb and cream or white centre within, stellate with narrow interpetalar membrane, lobed more than 1/3 to nearly halfway to the base, pubescent adaxially with a continuous ring of glandular trichomes (stalk long, 2–3-celled; head globose, peltate, unicellular) in the throat and base of the lobes, glabrous abaxially, the tube 2–4 mm long, the lobes 1.8–3.5 (–4.3) mm long, 2–4 mm wide, triangular or broadly triangular, spreading, the margins finely ciliate, the tips acute, cucullate, papillate. Stamens five, unequal in length; three filaments short 1.5–2.3 mm long, the two longer 1.9–3 mm long, white or cream, inserted on the corolla 0.6–1 mm from the base, with auricles fused to the corolla at the point of insertion; anthers 0.8–1.7 mm, ellipsoid, yellow, not connivent at anthesis. Gynoecium with ovary 0.7–1 mm long, 0.9–1.3 mm in diameter, light green, ovoid; ovules two per locule; nectary ca. 2.2 mm tall; styles homomorphic, 2.3–4 mm, somewhat exserted beyond the anthers, cream, clavate, slightly curved; stigma 0.1–0.2 mm long, 0.4 mm wide, discoid, pale green. Berry 3–5 mm long, 5–7 mm in diameter, globose-depressed, green when immature, greenish-golden yellow at maturity, deciduous, pungent, the pericarp thin, translucent, with giant cells (endocarp alveolate); stone cells absent; fruiting pedicels 14–25 mm, pendent, angled, slightly widened distally, green; fruiting calyx 3–4.5 mm in diameter, persistent, not accrescent, discoid, green. Seeds 4 (–6) per fruit, 3.7–3.9 (–4) mm long, 3–3.3 mm wide, C-shaped or reniform, brownish-black, the seed coat smooth or faintly reticulate and tuberculate at margins (SM), reticulate-cerebelloid with pillar-like outgrowths at margins (SEM), the cells rectangular or polygonal at margin and irregular in seed body, the lateral walls straight to wavy at margins and sinuate in the central zone; embryo imbricate.

#### Distribution.

*Capsicumcampylopodium* is an endemic species from south-eastern Brazil (Rio de Janeiro, Minas Gerais and Espírito Santo States, Fig. 32).

#### Ecology.

*Capsicumcampylopodium* is a typical component of the coastal Atlantic Forest (Mata Atlântica) and of some remnants of interior forests of the same biome. It is found in small colonies of a few individuals in shady or semi-shady places, sometimes also in sun, along roadsides or trails of the Ombrophilous Forest (Floresta Ombrófila Densa Submontana and Montana), between 100 and 1,200 m elevation.

#### Phenology.

Flowering from late September to March; fruiting from December to April.

#### Chromosome number.

*n* = 13 (Pozzobon and Schifino-Wittmann 2006), 2*n* = 2x = 26 (Moscone et al. 2003, 2007; Pozzobon et al. 2006).

#### Common names.

**Brazil.** Pimenta da Serra (Rio de Janeiro, *Kuhlmann 6288*).

#### Uses.

None recorded.

#### Preliminary conservation assessment.

EOO (58,586.312 km^2^); AOO (212 km^2^). *Capsicumcampylopodium* is a relatively widespread species that occurs in many formally protected areas, such as Parque Nacional da Tijuca, Parque Estadual da Pedra Branca, Estação Ecologica Estadual de Paraiso, Reserva Ecologica de Rio das Pedras, Parque Municipal Ecológico da Prainha, Estação Biológica Caratinga, amongst others (see Suppl. material 4: Appendix 4). Based on the extent of occurrence and the number of localities (ca. 50), we assign a category of Least Concern (LC). However, most collections are from a much-visited place in Rio de Janeiro (Parque Nacional da Tijuca) and from Serra dos Órgãos, with a serious problem of forest fragmentation in some areas (Freitas et al. 2006; Nehren et al. 2009) which may represent a threat to this species.

#### Discussion.

**Figure 37.**
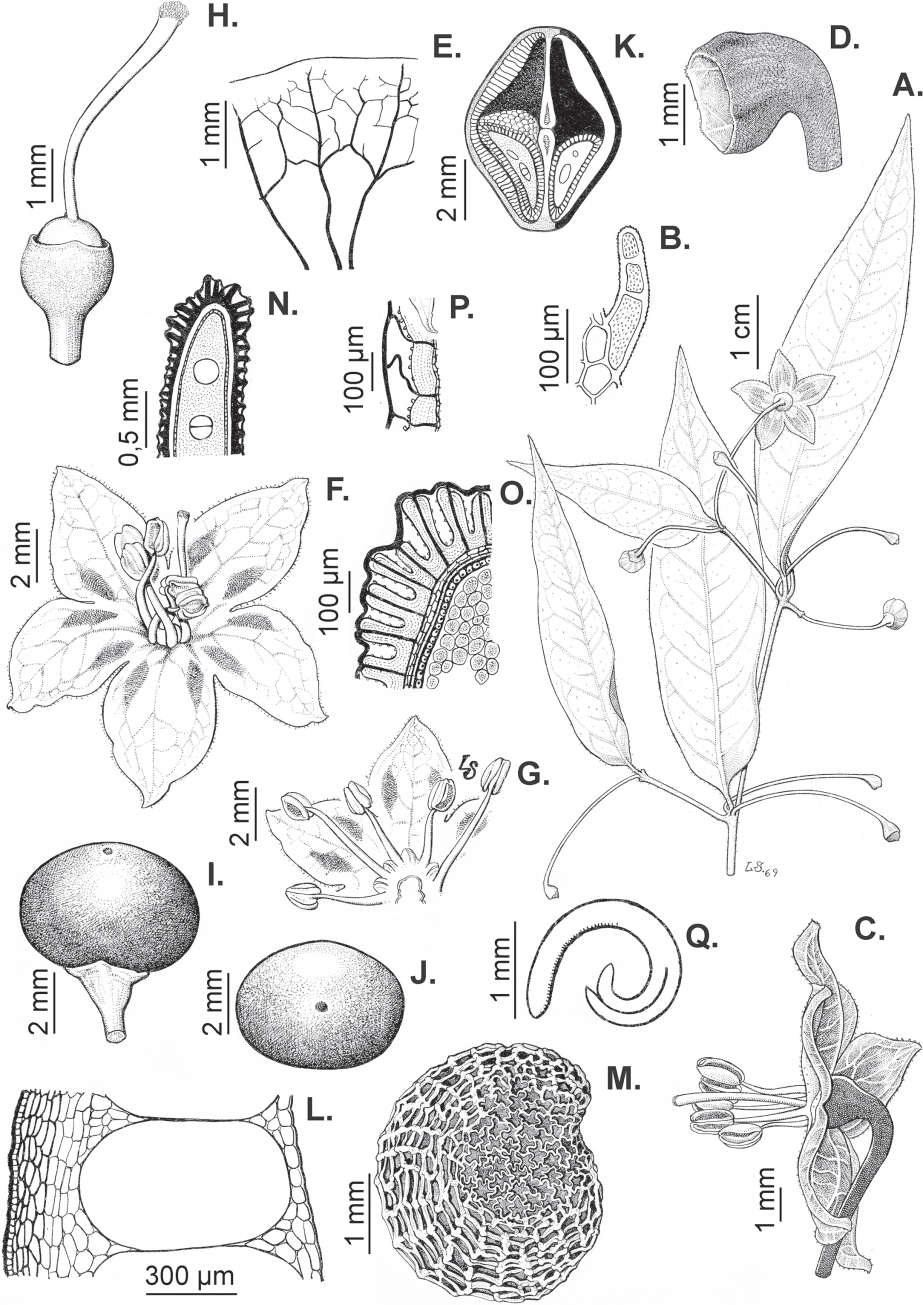
*Capsicumcampylopodium***A** flowering branch **B** eglandular trichome of the leaf **C** flower **D** calyx **E** section of the calyx showing the venation **F** flower, upper view **G** sector of opened corolla **H** gynoecium **I** fruit **J** fruit, upper view **K** fruit, in cross section **L** anatomical detail of the pericarp (note the giant cell in the mesocarp) **M** seed **N** seed, in cross section **O** structure of seed coat at the seed margin **P** structure of seed coat at the seed body **Q** embryo. From *Hunziker 25116*. Drawn by L. Sánchez. Published in Hunziker (2001), reproduced with permission.

**Figure 38.**
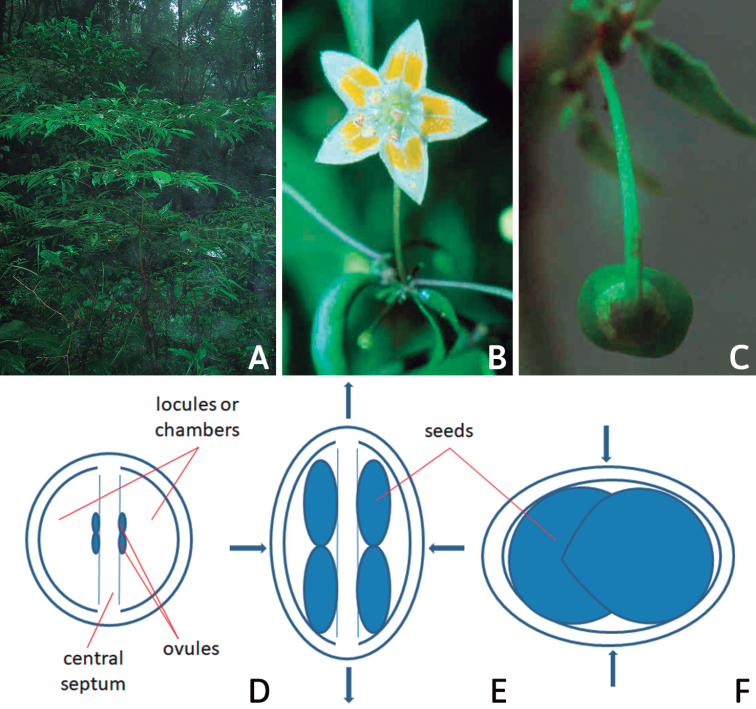
*Capsicumcampylopodium***A** plant **B** flower **C** immature fruit **D–F** diagrams of different stages of fruit development **D** ovary, in cross section, showing the locules and the number of ovules **E** young fruit, in cross section (the lateral arrows indicate the fruit is flattened around the centre) **F** mature depressed fruit (one locule), in longitudinal section, showing the two seeds occupying the whole locule **A** from *Barboza et al*. *2057*, photo by G.E. Barboza **B, C** from *Bianchetti et al. 511*, photos by L. Bianchetti.

*Capsicumcampylopodium* belongs to the Atlantic Forest clade (Carrizo García et al. 2016). It is characterised by its small flowers, hemispherical calyx with a circular outline, white corolla with golden yellow or ochraceous spots, locules with only two ovules and depressed fruits with not more than four seeds (Fig. 38). In herbarium material, the morphology of the fruits is not always preserved. However, in living material, the number of seeds per locule (2), the size of the seeds (large in proportion to the size of the fruit) and the arrangement of the seeds (parallel, but not touching) results in a slightly depressed fruit, rather than spherical (Figs 37I, J, 38C), which is a more common fruit shape in other species.

*Capsicumflexuosum*, *C.schottianum* and *C.campylopodium* all lack calyx appendages and are sometimes extremely difficult to distinguish from one another due to some characters being poorly preserved in herbarium specimens. *Capsicumcampylopodium* and *C.schottianum* both have clearly geniculate pedicels, a calyx with five evident nerves and greenish-golden yellow fruits with brownish-black to black seeds. The distinction of *C.campylopodium* from *C.schottianum* is based on calyx shape and size (hemispherical and ≤ 1.5 mm in diameter vs. cup-shaped and > 2 mm in diameter in *C.schottianum*), corolla colour (mostly white with large golden yellow spots within vs. white usually with purple and greenish-yellow spots within), fruit shape (globose-depressed vs. globose or subglobose) and number of seeds (four, very rarely six vs. ≥ six). The separation of *C.flexuosum*, the most distinctive species of the three, is based primarily on its lack of geniculate pedicels and its having a calyx with ten evident nerves, white corolla with yellowish-green spots within and red fruits (Fig. 63).

Dunal (1852) described *C.gracilipes*, based on three Gaudichaud specimens citing “v.s. in h. DC et h. Mus. Paris”; these are now housed at G-DC (G00131901) and P (P00410015, P00410016). All three collections are fruiting specimens, the one at G-DC (G00131901) is the best-preserved and here is designated lectotype.

For *C.salicifolium*, Dunal (1852) mentioned in the protologue the *Vauthier 528* collection he saw in G-DC and P. We found these specimens and other duplicates. We select the best-preserved specimen (G00131881) as the lectotype.

#### Specimens examined.

See Suppl. material 4: Appendix 4.

### ﻿7. Capsicum carassense

Taxon classificationPlantaeSolanalesSolanaceae

Barboza & Bianch., PhytoKeys 140: 127. 2020.

6A62452A-16E1-5232-9551-8CCEBE904458

Figs 39 , 40

#### Type.

Brazil. Minas Gerais: Catas Altas, RPPN Serra do Caraça, trilha da gruta de Lourdes, após a capelinha, 20°05'41"S, 43°28'52"W, 1386 m elev., 26 Oct 2014, *J.R. Stehmann, L.L. Giacomin, G.E. Barboza & S. Knapp 6347* (holotype [two sheets]: BHCB acc.#174038 [BHCB0019940_1, BHCB0019940_2]; isotypes: CORD [CORD00006968], RB [RB01220059, acc. # 674586], MBM).

#### Description.

Erect shrubs (0.8–) 1–2 (–3) m tall, with the main stem somewhat thick and sparsely branched, the branches dichotomous and spreading horizontally. Young stems 3–4-angled, fragile, green, moderately to densely pubescent with uncinate and antrorse, simple, uniseriate, 3–5 (–6)-celled, eglandular trichomes 0.2–0.7 mm long, yellowish-brown when dried; nodes green or purple; bark of older stems brown, striate, pubescent; lenticels absent. Sympodial units difoliate, the leaves geminate; leaf pair unequal in size, similar in shape. Leaves membranous to chartaceous, discolorous, dark green above, paler beneath, moderately pubescent especially on the veins, with simple trichomes like those of the stem and sparse or frequent glandular trichomes (stalk unicellular; head multicellular) adaxially and abaxially; blades of major leaves 6–16 cm long, 0.9–2.5 cm wide, narrowly elliptic to lanceolate, the major veins 6–8 on each side of mid-vein, the mid-vein prominent and the secondary veins obscure, the base attenuate, the margins entire, the apex acute to obtuse; petioles 0.2–0.6 cm long, moderately pubescent; blades of minor leaves 2.9–3.9 cm long, 0.5–0.8 cm wide, narrowly elliptic, the major veins 2–3 (–4) on each side of mid-vein, the base attenuate, the margins entire, the apex obtuse; petioles 0.2–0.4 cm long, moderately pubescent. Inflorescences axillary, 2–4 flowers per axil; flowering pedicels (12–) 15–20 (–22) mm long, slightly angled, erect to spreading, geniculate at anthesis, green, moderately pubescent, the eglandular trichomes short or long, antrorse to spreading; pedicels scars inconspicuous. Buds ellipsoid, cream with greenish-yellow spots. Flowers 5-merous. Calyx 1.2–1.6 mm long, 2.5–3 mm wide, cup-shaped, thin, light green to cream, moderately pubescent with antrorse, curved, 3–5-celled, eglandular trichomes and sparse short glandular trichomes (stalk short, unicellular; head dark, elongate, multicellular), the calyx appendages five, (2.8–) 3–4 (–5) mm long, subequal, thick, erect, cylindrical, inserted very close to the margin. Corolla (8–) 10–12 mm long, 13–20 mm in diameter, thick, white with greenish-yellow spots outside, mostly with large purple spots on the lobes and the throat and cream centre within, stellate with abundant interpetalar membrane, lobed halfway or less of the way to the base, pubescent adaxially with a continuous ring of long glandular trichomes (stalk 2–3-celled; head globose, peltate, unicellular) in the throat and base of the lobes, glabrous abaxially, the tube 4.5–5 mm long, the lobes 4.5–6.5 mm long, 5–8 mm wide, broadly triangular to triangular, the margins densely pubescent, the tips cucullate. Stamens five, subequal; filaments 2.7–3.1 (–4.1) mm long, white, inserted on the corolla ca. 1 mm from the base, with auricles fused to the corolla at the point of insertion; anthers 1.5–1.9 mm long, ellipsoid, blue, not connivent at anthesis. Gynoecium with ovary 1.3–1.5 mm long, ca. 1.2 mm in diameter, light green, subglobose to ovoid; ovules more than two per locule; nectary ca. 0.3 mm tall; styles homomorphic, 4.3–5 (–7) mm long, barely exserted beyond the anthers, white, clavate; stigma ca. 0.2 mm long, ca. 0.7 mm wide, discoid, cream. Berry 6–7 mm in diameter, globose-depressed, green when immature, greenish at maturity, deciduous, pungent, the pericarp with giant cells (endocarp alveolate); stone cells absent; fruiting pedicels 18–25 mm long, pendent and slightly curved, slightly angled, widened at the apex, green; fruiting calyx ca. 4 mm in diameter, persistent, not accrescent, discoid, yellowish-green, the appendages spreading, green. Seeds 7–13 per fruit, 3.5–4 mm long, 2.5–3 mm wide, ellipsoid to reniform, brownish-black to black, the seed coat deeply reticulate and slightly tuberculate at margins (SM), reticulate with small pillar-like outgrowths at margins (SEM), the cells polygonal in shape, the lateral walls straight to wavy; embryo imbricate.

**Figure 39.**
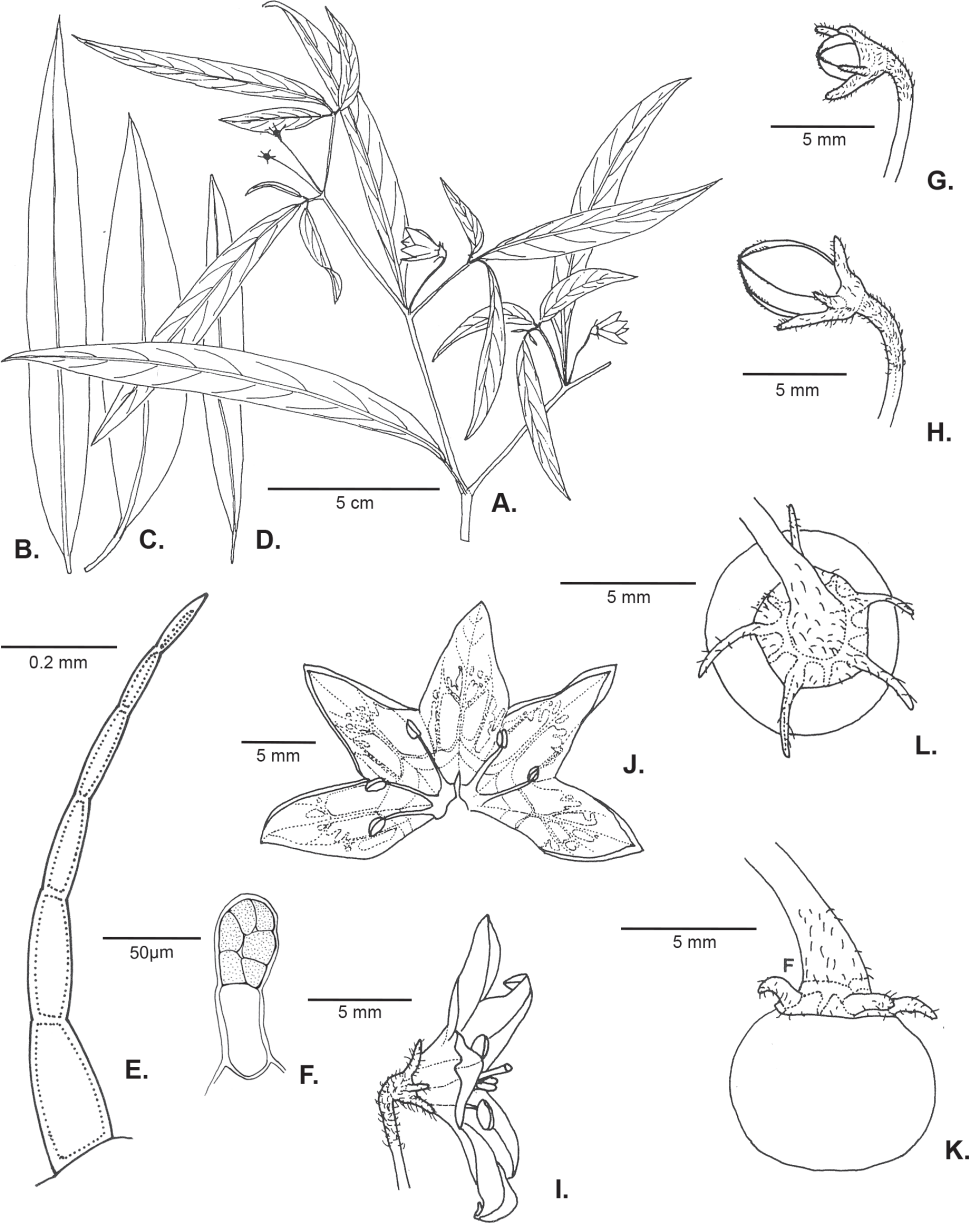
*Capsicumcarassense***A** flowering branch **B–D** leaf morphology **E** eglandular trichome of the stem **F** glandular trichome of the calyx **G, H** flower buds in different stages of development **I** flower **J** opened corolla **K** fruit **L** fruiting calyx. From *Bianchetti et al. 1364*. Drawn by L. Bianchetti. Published in Barboza et al. (2020a), reproduced with permission.

**Figure 40.**
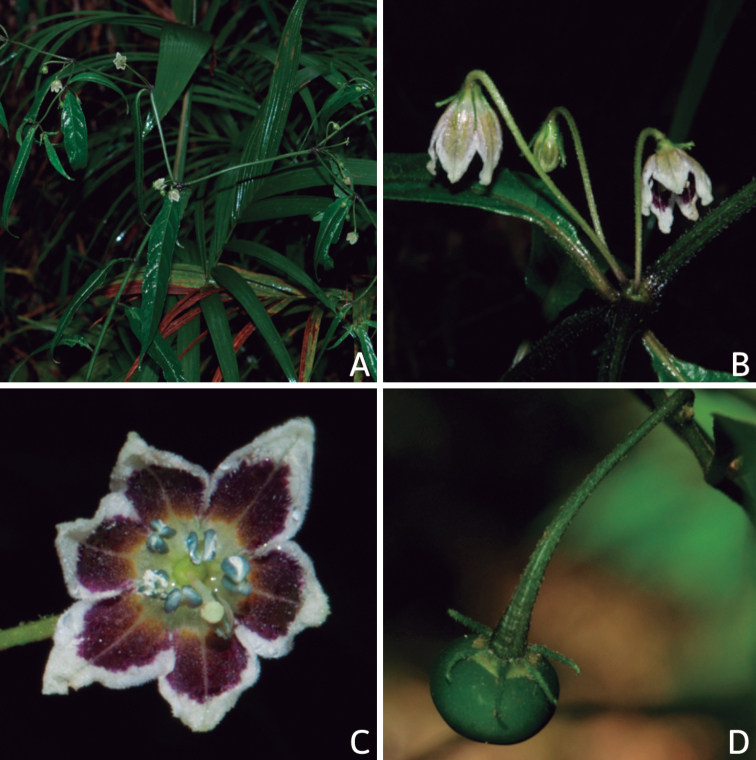
*Capsicumcarassense***A** plant, showing the typical lanceolate leaves **B** inflorescence with geniculate pedicels **C** flower, in front view **D** fruit **A–C** from *Stehmann 6344***D** from *Agra 7268*. Photos by J.R. Stehmann. Published in Barboza et al. (2020a), reproduced with permission.

#### Distribution.

**Figure 41.**
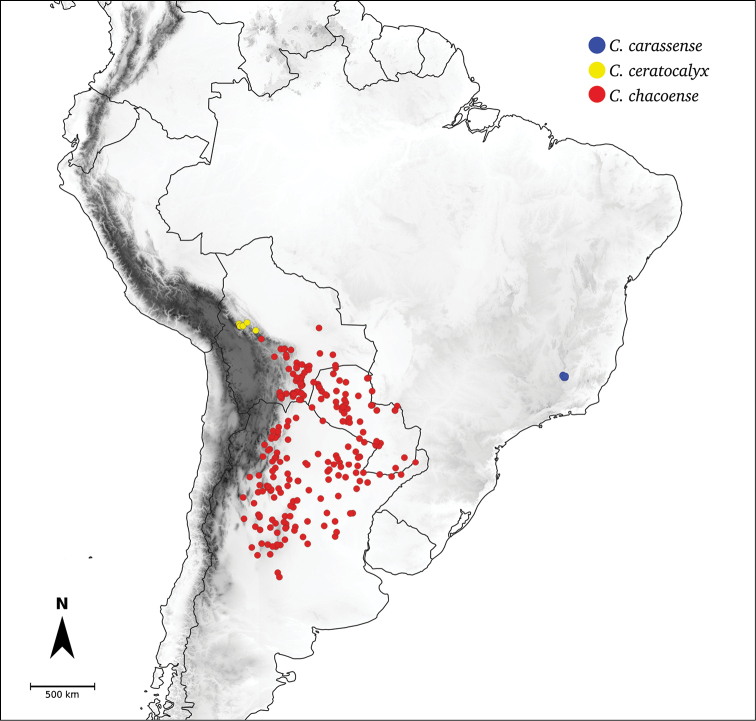
Distribution of *C.carassense*, *C.ceratocalyx* and *C.chacoense*

*Capsicumcarassense* is endemic to south-eastern Minas Gerais State (Brazil), growing mainly in the Serra do Caraça and other nearby mountainous areas (Fig. 41).

#### Ecology.

*Capsicumcarassense* inhabits the understorey of the semi-deciduous montane Atlantic Forest, in a shaded and moist environment, between 1,000 and 1,390 m elevation.

#### Phenology.

In flower from October to January, also in May; fruiting in December, February and April.

#### Chromosome number.

Not known.

#### Common names.

None recorded.

#### Uses.

None recorded.

#### Preliminary conservation assessment.

EOO (483.4 km^2^); AOO (32 km^2^). *Capsicumcarassense* is considered Endangered (EN, B1ab(iii,iv)). We suggest this because of its very restricted geographic distribution, as well as the increasingly degraded habitat quality, especially associated with the extensive iron mining activities in the region (Barboza et al. 2020a).

#### Discussion.

*Capsicumcarassense* belongs to the Atlantic Forest clade (Barboza et al. 2020a). This species is morphologically very similar to *C.mirabile* with which it has been confused in herbaria. Both species share a similar habit, the geniculate pedicels at anthesis, the number of calyx appendages, the shape and colour of the corolla, the colour and pungency of the fruits and the blackish seeds. They can be easily distinguished by the moderate to dense indumentum, the narrowly elliptic to lanceolate major leaves with apex acute to obtuse, the shorter petioles (up to 0.6 cm long), the cream buds with greenish-yellow pigmentation, the longer calyx appendages (up to 5 mm) and the larger corolla (13–20 mm in diameter) in *C.carassense*, compared to *C.mirabile* which has sparse pubescence (young stems sometimes glabrous), major leaves elliptic (rarely narrowly elliptic) to ovate with apex acuminate to long-acuminate, longer petioles (up to 2.5 cm), purple or greenish-purple buds, shorter calyx appendages (up to 3 mm) and smaller corolla (9–13 mm in diameter) (see also Table 4).

**Table 4.** Differences between *C.mirum*, *C.cornutum*, *carassense* and *C.mirabile*.

Character	*C.mirum*	*C.cornutum*	*C.carassense*	*C.mirabile*
Indument/trichomes (stems and leaves)	Densely pubescent/trichomes antrorse	Densely pubescent/trichome spreading	Moderately to densely pubescent/trichomes antrorse	Glabrate to sparsely pubescent/trichomes antrorse
Major leaf shape	Mostly elliptic, apex acute to acuminate	Ovate to widely elliptic, apex acuminate	Narrowly elliptic to lanceolate, apex acute to obtuse	Elliptic to ovate, rarely narrowly elliptic, apex acuminate to long- acuminate
Major leaf length/width ratio	2.5–3	2.4–5	(4–) 5–10 (–16)	(2–) 2.5–4 (–4.9)
Petioles length	0.8–2.5 cm	0.3–0.8 cm	0.2–0.6 cm	0.7–2.5 cm
Pedicels length	12–17 mm	(22–) 25–35 mm	(12–) 15–20 (–22) mm	(13–) 16–25 mm
Buds colour	Purple	White with green and purple spots	Cream with greenish-yellow spots	Purple or greenish-purple
Calyx appendages	10, subequal, spreading, long, (1.7–) 2–3.2 mm	(5–) 7–10, unequal, erect or spreading, short to long, 0.5–6 mm	5, subequal, long, erect, (2.8–) 3–4 (–5) mm	5, subequal, erect, short to long, (0.4–) 0.5–1.5 (–3) mm
Corolla size	6–8 mm long, 11–14 mm in diameter	(8–) 9–14 mm long, 18–22 mm in diameter	(8–) 10–12 mm long, 13–20 mm in diameter	(6–) 7.5–12 mm long, (9–) 10–13 mm in diameter
Corolla colour	Almost entirely purple and a thin white border within	White with small purple or reddish-brown spots within	Mostly with large purple spots and a thin white border within	Mostly with large purple spots and a thin white border within

#### Specimens examined.

See Suppl. material 4: Appendix 4.

### ﻿8. Capsicum cardenasii

Taxon classificationPlantaeSolanalesSolanaceae

Heiser & P.G.Sm., Brittonia 10(4): 195. 1958.

EE7328AC-CCAE-5683-A1B4-AEE6C901AEA2

Figs 42 , 43

#### Type.

Cultivated at Indiana University greenhouse from seeds sent by M. Cárdenas from market in La Paz, Bolivia, 15 Aug 1956, *C.B. Heiser Jr. 4196* (Paul Smith Ac.-1793) (lectotype, designated here: IND [IND1000063, acc. # 139347]; isolectotype: IND [IND1000064, acc. # 139348]).

#### Description.

Erect shrubs or subshrubs, 0.8–2 (–2.5) m tall, with the main stem 1–1.5 cm in diameter at base, much branched from near the base, the fragile branches in a typical “zig-zag” appearance above. Young stems strongly angled, green, glabrescent with sparse appressed-antrorse, simple, uniseriate, 4–6 (–7)-celled eglandular trichomes 0.08–0.6 mm long and minute, simple, glandular trichomes (stalk short; head dark); nodes green; bark of older stems greyish-white or with light brown-green fissures, glabrescent; lenticels absent. Sympodial units difoliate, the leaves geminate; leaf pair subequal in size and shape. Leaves membranous, slightly discolorous, glabrescent, with sparse eglandular trichomes similar to those on stems and many small glandular trichomes (stalk unicellular; head dark, multicellular) on both surfaces, the glandular trichomes more abundant along mid-vein abaxially; blades of major leaves 3–5 (–6.5) cm long, 1.4–2.5 cm wide, narrowly ovate or ovate-lanceolate, the major veins 3–4 on each side of mid-vein, the base attenuate, the margins entire, the apex acute; petioles 1.2–2 cm long, glabrous or glabrescent; the blades of minor leaves 2–3.5 cm long, 0.9–1.2 cm wide, narrowly ovate or ovate-lanceolate, the major veins 2–3 on each side of mid-vein, the base attenuate, the margins entire, the apex acute or obtuse; petioles 0.5–0.8 cm long. Inflorescences axillary, 2–3 flowers per axil or flowers solitary; flowering pedicels 8–18 (–22) mm long, angled, erect to slightly spreading, geniculate at anthesis, entirely green, or purple distally, with moderate small glandular trichomes (stalk transparent, uni-bicellular; head dark, multicellular) and sparse short, antrorse eglandular trichomes; pedicels scars inconspicuous. Buds ellipsoid or ovoid, lilac or violet. Flowers 5-merous. Calyx 1–3 mm long, 2–3 mm wide, cup-shaped, thick, green or green with violet spots, moderately pubescent with the same glandular and eglandular trichomes as the pedicels, the calyx appendages five, 1–2 mm long, 0.3 mm wide, subequal, thick, erect or spreading, cylindrical, inserted close to the margin, sparsely pubescent with the same trichomes as the calyx tube. Corolla (6–) 6.5–12 mm long, 8–11 (–13) mm in diameter, thick, almost completely violet or lilac, but white at the base and along the main veins outside and within, sometimes greenish-yellow spots near the base within, campanulate with interpetalar membrane, lobed 1/3 or less of the way to the base, the tube 7–9 mm long, pubescent adaxially with short glandular trichomes (stalk 1–2-celled; head globose, unicellular) up to near its base, glabrous abaxially, the lobes (1.5–) 3–3.2 mm long, 2–2.4 mm wide, triangular, erect or spreading, alternating with five minute interlobes, glabrous adaxially and abaxially, the margins papillate, the tips acute, papillate. Stamens five, equal; filaments (4–) 6–7 mm long, whitish or lilac, inserted on the corolla 1.5–2 mm from the base, with auricles fused to the corolla at the point of insertion; anthers 1.5–1.8 mm long, ellipsoid, lilac or bluish, not connivent at anthesis. Gynoecium with ovary 1.5–1.85 mm long, 1.2–1.6 mm in diameter, green, ovoid or pear-shaped; ovules more than two per locule; nectary 0.4–0.6 mm tall, light green; styles homomorphic, 4.5–5.7 mm long, exserted ca. 1 mm beyond the anthers, lilac or purple, clavate; stigma ca. 0.2 mm long, 0.8 mm wide, discoid or globose, pale green. Berry 6–10 mm in diameter, globose or subglobose, green when immature, orange-red to bright red at maturity, deciduous, pungent, the pericarp thick, opaque, with giant cells (endocarp alveolate); stone cells absent; fruiting pedicels 10–24 mm long, pendent, strongly angled, slightly widened distally, usually green; fruiting calyx 2–4 mm in diameter, persistent, not accrescent, discoid, green, the appendages 1–2.5 mm long, ca. 0.3 mm wide, appressed to the berry, spreading or reflexed. Seeds (4–) 5–13 per fruit, (2.5-) 3–4.2 mm long, (2.2–) 2.5–2.8 mm wide, C-shaped or subglobose, pale yellow to brownish-yellow, the seed coat reticulate to obscurely reticulate (SM), mostly cerebelloid (SEM), the cells irregular in shape, the lateral walls strongly sinuate in the central zone, rectangular to subpolygonal at margins; embryo imbricate.

#### Distribution.

*Capsicumcardenasii* is a narrow endemic species restricted mainly to the highlands of La Paz Department (Bolivia, Fig. 32). Only one collection from Tarija, probably introduced.

#### Ecology.

*Capsicumcardenasii* is a typical component of the warm and dry hillsides and remnants of forests in the inter-Andean valleys, growing preferentially in open places between cactus and *Cassia*, at 2,400–3,000 m elevation. It is cultivated by local people on small farms for local or family use of the fruits (Barboza, pers. obs.).

#### Phenology.

Flowering from December to March; fruiting from February to April.

#### Chromosome number.

*n* = 12 (Heiser and Smith 1958); 2*n* = 2x = 24 (Pickersgill 1977; Moscone et al. 2007; Scaldaferro et al. 2013, 2016).

#### Common name.

**Bolivia.** Ulupica (La Paz, *Heiser & Smith 4196*).

#### Indigenous name.

**Bolivia.** Uaika (Aymará, La Paz, *Barboza 4881*).

#### Uses.

The fruits are harvested directly from wild plants and marketed locally on a small scale (Jäger et al. 2013), mainly in La Paz, Bolivia. People consume dehydrated or fresh fruits in the preparation of a hot sauce called ‘Jallpa huayka’ (in aymara), ‘uchu llajfua’ (in quechua) (Heiser and Smith 1958; Cárdenas 1969), ‘llajwa’ (in quechua) or ‘llaswa’ (most popular), a mix of ‘tomato’, ‘onion’, ‘ulupica’ or other ‘chiles’ and aromatic herbs. Fruits are also preserved in vinegar or in oil and used as pickle (‘escabeche’) or they are cooked in boiling water before being sold (National Research Council 1989; Barboza, pers. obs.).

#### Preliminary conservation assessment.

EOO (1,032.864 km^2^); AOO (32 km^2^). *Capsicumcardenasii* is a geographically isolated species from the dry valleys of Luribay (Prov. Loayza), not far from La Paz; based on its extent of occurrence and the number of localities (6), it is assigned a status of Endangered (EN; B1ab(iii,iv)). It is harvested by local people; its area of distribution is poorly known.

**Figure 42.**
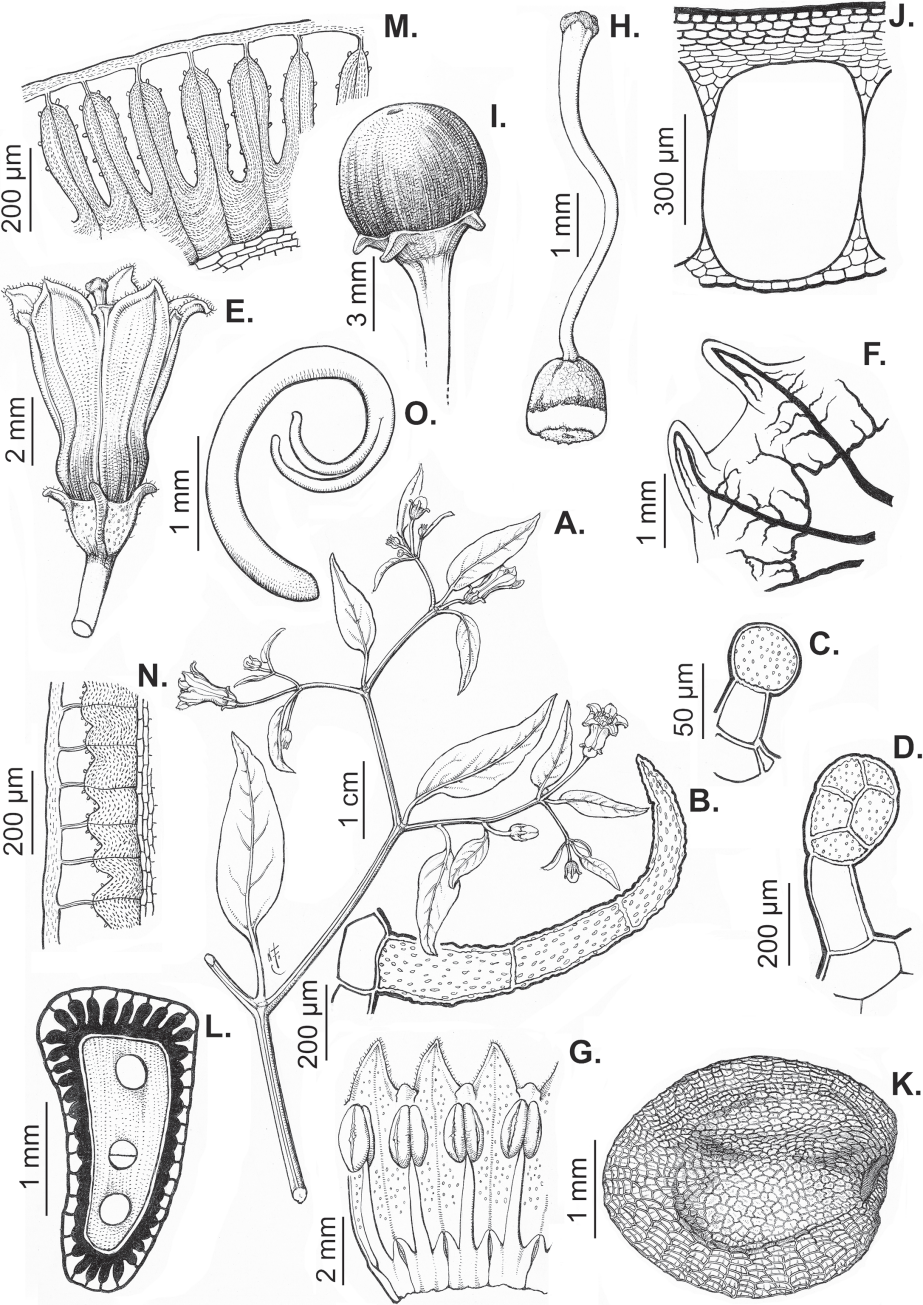
*Capsicumcardenasii***A** flowering branch **B** eglandular trichome of the calyx **C** glandular trichome of the corolla **D** glandular trichome of the adaxial surface of the calyx **E** flower **F** section of the calyx showing the venation **G** sector of opened corolla **H** gynoecium **I** fruit **J** anatomical detail of the pericarp (note the giant cell in the mesocarp) **K** seed **L** seed, in cross section **M** structure of seed coat at the seed margin **N** structure of seed coat at the seed body **O** embryo **A–H** from *Eshbaugh 1527***I–O** from *Eshbaugh 2046 J*. Drawn by N. de Flury.

#### Discussion.

**Figure 43.**
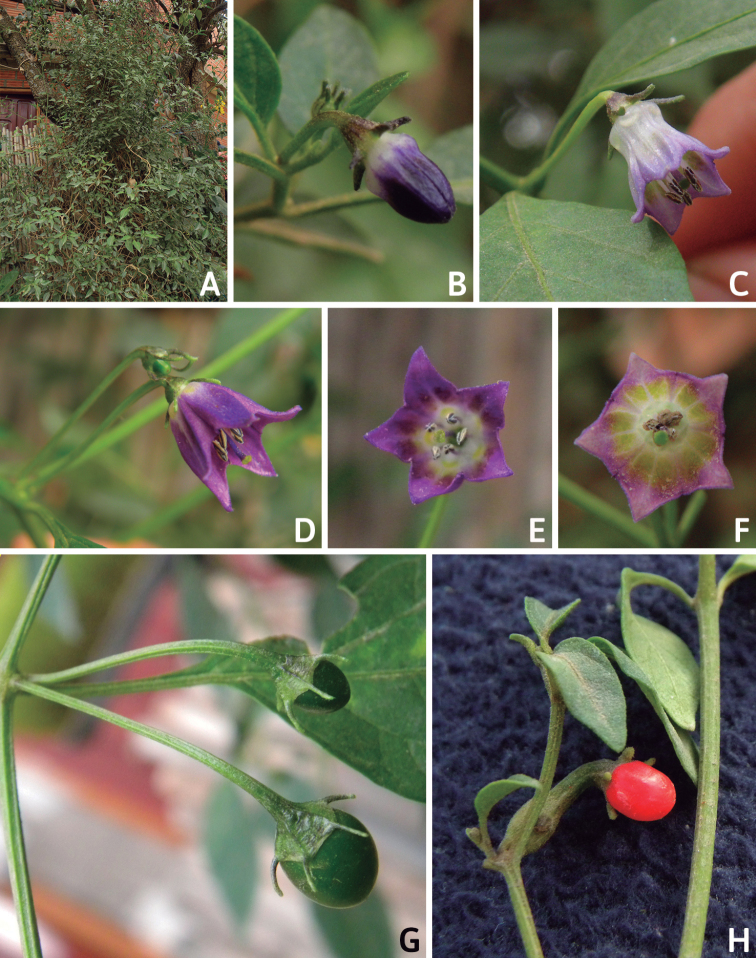
*Capsicumcardenasii***A** plant **B** flower bud **C, D** flower, in lateral view **E, F** flower, in front view (note the different colouration inside the corolla) **G** immature fruits **H** mature fruit **A, B, D, E, G** from *Barboza 4882*, C, F, H from *Barboza 4881*. Photos by G.E. Barboza.

*Capsicumcardenasii* is resolved within the Purple corolla clade (Carrizo García et al. 2016). More recent preliminary phylogenetic evidence showed that *C.pubescens* is sister to this clade (Carrizo García et al. 2019; CCG, pers. obs.), thus circumscription of these taxa is under revision (CCG, pers. obs.; see under *C.pubescens* description). The fruits of *C.cardenasii* are very similar to those of *C.eximium* and *C.eshbaughii*, both also known as “ulupica”. The three species can be differentiated by their general pubescence and corollas. *Capsicumeshbaughii* (Fig. 58) has a dense pubescence of long furcate glandular trichomes and stellate white corollas with greenish-yellow spots around the throat (rarely purple lines in the lobes). In contrast, *C.cardenasii* and *C.eximium* have sparse to moderate pubescence of eglandular simple trichomes and minute simple glandular trichomes. In addition, *C.cardenasii* has lilac to purple, broadly campanulate corollas (Fig. 43C–F), while *C.eximium* has mostly purple, lilac or magenta, stellate corollas with greenish-yellow pigmentation within (Fig. 60H–J).

Phytogeographically, Eshbaugh (1979) suspected that *C.cardenasii* and *C.eximium* could be sympatric on the eastern margin of the range of *C.cardenasii*. He had observed some intermediate plants between both taxa in the Luribay Valley (Eshbaugh 1976, 1979, but specimens not cited) and, at that time, no collections of the typical *C.eximium* were known from that area (Eshbaugh 1979). We recently collected *C.eximium* (e.g. *Barboza 4885*) in Luribay, very near to the sites where *C.cardenasii* grows abundantly. Luribay Valley deserves to be explored intensively to document the presence of natural hybrids in the area and to find out if their level of fertility is as high as in experimental crosses (Heiser and Smith 1958; Eshbaugh 1976). Evidence of hybridisation was found while attempting to identify and cytogenetically characterise these *Capsicum* species, using a molecular cytogenetic approach (seeds from Gene Bank, Nijmegen University, The Netherlands); however, the fertility of the hybrid has not been possible to ascertain (Scaldaferro 2019).

Eshbaugh and Smith (1971) also obtained successful crosses between *C.cardenasii* and *C.eshbaughii* (e.g. IND 139349), though F1 and F2 hybrids were less fertile than with *C.eximium*. The narrow distributions of *Capsicumcardenasii* and *C.eshbaughii* are allopatric, with the first species in north-western Bolivia (La Paz: Luribay) and the second concentrated in central-eastern Bolivia (mainly Santa Cruz: Samaipata). Their distributions are separated by nearly 700 km of distance, making hybridisation in the wild highly unlikely.

Although the number of collections of *C.cardenasii* obtained in the field are scarce (8), the ease with which the seeds of this species germinate and produce fertile plants explains the large numbers (> 20) of specimens (and duplicates) gathered from plants in cultivation that are housed in many herbaria (see Specimens Examined).

The type collection of *C.cardenasii* consists of flowering specimens (two sheets dated 15 Aug 1956 at IND) obtained from seeds bought at the La Paz (Bolivia) marketplace; it is supposed that fruits came from warm, dry places along the Río Abajo, near La Paz, at 2400 m altitude (Heiser and Smith 1958). Of the two specimens in IND, that with barcode 1000063 is the most complete, is labelled type and is here designated as the lectotype. There are specimens distributed in other herbaria (e.g. CORD, IND, LIL, US) with the same collection number as the lectotype (*Heiser 4196*, Paul Smith Acc. 1793), but these have different dates of collection and should not be considered as duplicates of the lectotype.

#### Specimens examined.

See Suppl. material 4: Appendix 4.

### ﻿9. Capsicum ceratocalyx

Taxon classificationPlantaeSolanalesSolanaceae

M.Nee, Brittonia 58 (4): 326. 2006.

D837310E-F6C0-56E8-9497-D36DFD62E0D6

Fig. 44

#### Type.

Bolivia. La Paz: Prov. Sud Yungas: 7.5 km (by road) from Huancané on road to San Isidro, 16°21'S, 067°30'W, 2225 m elev., 10 May 2001, *M. Nee, L. Bohs, S. Knapp & J.M. Mendoza F. 51778* (holotype: LPB [LPB0003514]; isotypes: CORD [CORD00004289], MO [MO-2078805, acc. # 5959885], NY [01085523], USZ).

#### Description.

Erect shrubs 0.80–3 m tall, much branched above. Young stems angled, green, glabrous to sparsely pubescent, with antrorse, curved, simple, uniseriate, 2–4 (–5)-celled, eglandular trichomes 0.09–0.5 mm long; nodes solid, green; bark of older stems brown, glabrous; lenticels absent. Sympodial units difoliate, the leaves geminate; leaf pair markedly unequal in size, similar in shape. Leaves coriaceous, slightly discolorous, glabrescent on both sides with sparse eglandular trichomes similar to the ones of the stems, mainly along the mid-vein abaxially; blades of major leaves 10–22 cm long, 4–7 cm wide, elliptic, the major veins 6–7 on each side of mid-vein, the base attenuate and unequal, the margin slightly revolute, the apex long-acuminate; petioles 1–2 (–3) cm long, glabrous; blades of minor leaves (3–) 5.5–7.5 cm long, 2–2.7 cm wide, elliptic, the major veins 3–4 on each side of mid-vein, the base short-attenuate, the margin slightly revolute, the apex acute; petioles 0.5–0.6 cm long, glabrous. Inflorescences axillary, congested, (4–) 8–10 (–12) flowers on a short rachis; flowering pedicels 10–23 mm long, strongly angled and nearly winged, erect, geniculate at anthesis, green, glabrous; pedicels scars prominent and corky. Buds ovoid, yellow. Flowers 5-merous. Calyx 1.8–2 mm long, ca. 3 mm wide, cup-shaped, slightly 5-nerved, sparsely pubescent, with the same antrorse eglandular trichomes of the young stems and small glandular trichomes (stalk unicellular; head multicellular), the calyx appendages (3–) 5, 0.25–2.5 mm long, subequal, thick, notoriously incurved, spreading, flattened laterally, glabrescent, inserted close to the margin. Corolla 6–8.5 mm long, ca. 5 mm in diameter, yellow with green spots within, stellate to broadly campanulate with interpetalar membrane, lobed nearly or more than the halfway to the base, the tube 3–4 mm long, pubescent adaxially with sparse glandular trichomes (stalk uni-bicellular; head unicellular), glabrous abaxially, the lobes 3.2–3.7 mm long, 2–3 mm wide, triangular, erect, glabrous adaxially and abaxially, the margins involute, the tips acute, papillate. Stamens five, equal; filaments 1–2 mm long, inserted on the corolla 1–1.5 mm from the base, with auricles fused to the corolla at the point of insertion; anthers 1.4–1.8 mm long, ovoid, not connivent at anthesis. Gynoecium with ovary 1.8–2 mm long, 1.3–1.5 mm wide, ovoid; ovules more than two per locule; nectary ca. 0.3 mm tall; styles homomorphic, 5.8–6.5 mm, clavate; stigma 0.1–0.2 mm long, ca. 0.5 mm wide, discoid. Berry 8–11 mm in diameter, globose, slightly flattened at the apex, green when immature, bright red at maturity, persistent, pungent, the pericarp thick, opaque, with giant cells (endocarp alveolate); stone cells absent; fruiting pedicels (15–) 25–30 mm long, erect, conspicuously angled, winged and widened distally; fruiting calyx 3–5 (–7) mm in diameter, persistent, not accrescent, discoid, the appendages 2–5 mm long, incurved. Seeds 13–26 per fruit, 4–5 mm long, (2.6–) 3–4 mm wide, C-shaped or teardrop-shaped, brownish-yellow to brown, the seed coat reticulate (SM), reticulate-cerebelloid (SEM), the cells irregular in shape, the lateral walls strongly sinuate in the seed body, wavy at margins to nearly straight near hilum; embryo coiled.

**Figure 44.**
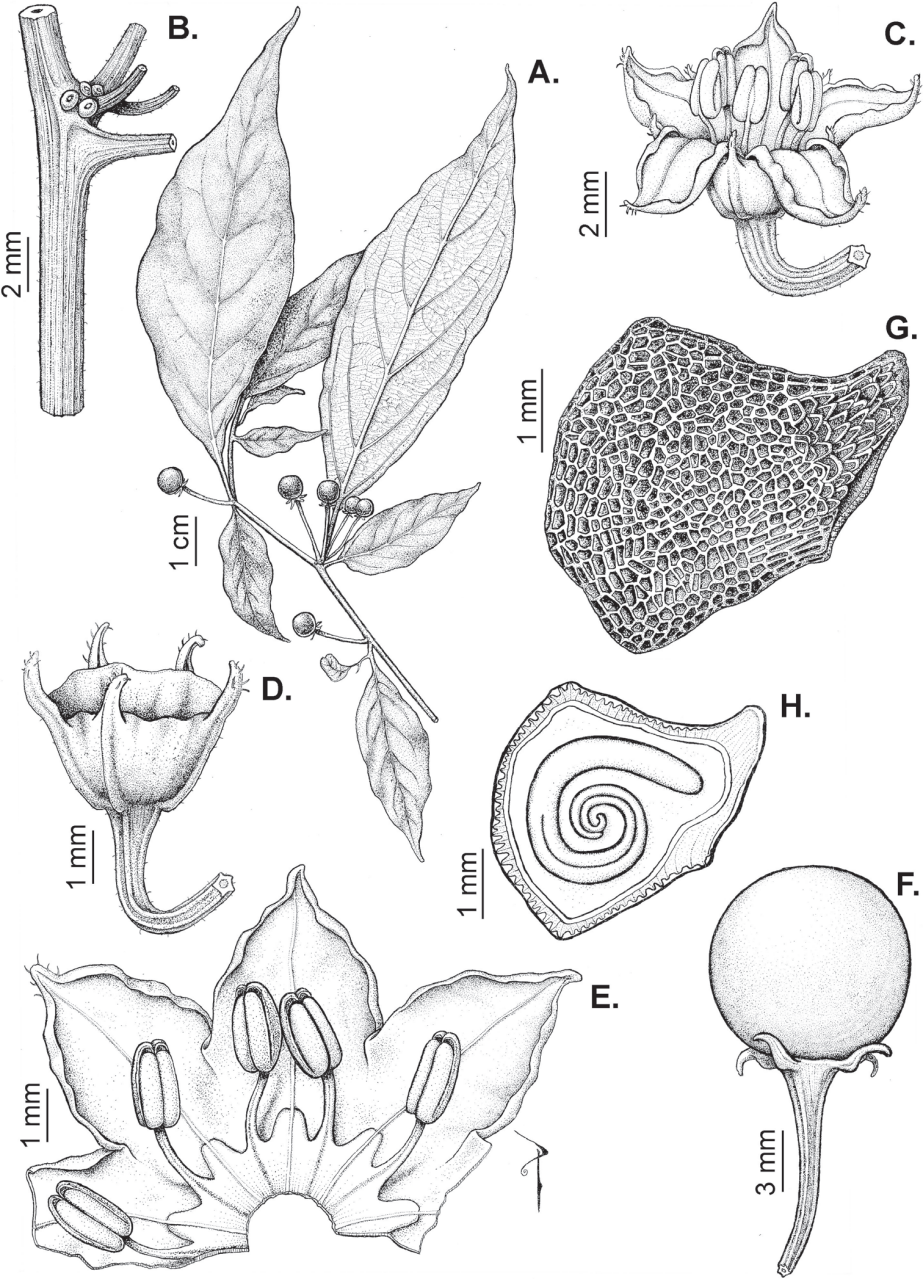
*Capsicumceratocalyx***A** fruiting branch **B** node with pedicels scars and base of two pedicels **C** flower **D** calyx **E** opened corolla **F** fruit **G** seed **H** seed, in longitudinal section **A–E** from *Beck 28089*, **F–I** from *Seidel & Hinojosa 1267*. Drawn by P. Peralta.

#### Distribution.

*Capsicumceratocalyx* is endemic to the Bolivian Departments of La Paz and Cochabamba (Fig. 41).

#### Ecology.

*Capsicumceratocalyx* is known from few collections, all from moist montane forest (Yungas) with little disturbance, between 700 and 2,500 m elevation.

#### Phenology.

Flowering and fruiting from November to July.

#### Chromosome number.

Not known.

#### Common names.

None recorded.

#### Uses.

None recorded.

#### Preliminary conservation assessment.

EOO (3,612.392 km^2^); AOO (32 km^2^). *Capsicumceratocalyx* is an endemic with relatively small EOO and AOO from the montane forests of the Bolivian Yungas, an ecoregion that deserves urgent special conservation efforts (Mueller et al. 2002). The few collections (9) come from unprotected areas and the populations are threatened by expanding agriculture. For these reasons, it is assigned a status of Endangered (EN; B1ab(iii,iv)).

#### Discussion.

*Capsicumceratocalyx* has been assigned to the Bolivian clade (Carrizo García et al. 2016; Barboza et al. 2019), with *C.minutiflorum* as its sister species. *Capsicumceratocalyx* is a poorly known component of the mid-altitudes of the Bolivian Yungas. It has large coriaceous leaves, a calyx with 3–5 incurved and spreading appendages, yellow corollas and winged fruiting pedicels. The affinities of this species need to be further studied, since new preliminary data, based on genome-wide DNA sequences, suggest this species may form an isolated lineage (CCG, pers. obs.).

#### Specimens examined.

See Suppl. material 4: Appendix 4.

### ﻿10. Capsicum chacoense

Taxon classificationPlantaeSolanalesSolanaceae

Hunz., Darwiniana 9(2): 228. 1950.

E74BE3F5-2313-5313-8BCB-3C28A34262D1

Figs 45 , 46

Capsicum chacoense Hunz. var. tomentosum Hunz., Darwiniana 9(2): 235. 1950. Type. Argentina. Chaco: [Dept. Independencia], Colonia J.J. Mármol, 31 Dec 1946, *F. Buratovich 117* (holotype: LIL [acc. # 173409); isotype: B [B10-1067916]).

#### Type.

Argentina. Chaco: [Dept. Primero de Mayo] entre Colonia Benítez y Resistencia, 12 Mar 1945, *A.T. Hunziker 7340* (lectotype, designated here: CORD [CORD00003920]; isolectotypes: CORD [CORD00003919, CORD00087962]).

#### Description.

Low compact shrubs or subshrubs 0.40–1 (–2.5) m tall, much branched from a thick basal rootstock, with the main stem 2–3 cm in diameter at base, the branches expanded and divaricated, in a typical “zig-zag” appearance. Young stems strongly 3–4-angled, fragile, green or purple, sparsely to densely pubescent with antrorse and more or less rigid or spreading and flexuous, simple, uniseriate, 3–7-celled, eglandular trichomes 0.03–0.8 (–1.4) mm long, rarely branched trichomes 1–7 mm long; nodes green; bark of older stems brown, glabrescent to glabrous; lenticels sparse. Sympodial units unifoliate or difoliate, the leaves geminate; leaf pair more or less similar in shape and size. Leaves membranous, slightly discolorous or concolorous, glabrescent to densely pubescent with eglandular trichomes similar to the stems on both surfaces and margins; blades of all leaves 2–6 (–8) cm long, 1–3.5 (–5) cm wide, ovate, narrowly ovate or rarely elliptic, the major veins 3–4 on each side of midvein, the base attenuate and asymmetric, the margins entire, the apex long-acuminate; petioles 0.5–2 (–3.5) cm long, glabrescent to densely pubescent. Inflorescences axillary, flower solitary; flowering pedicels (5–) 10–25 (–40) mm long, strongly angled, erect, geniculate at anthesis, green, scarcely to moderately pubescent; pedicels scars inconspicuous. Buds globose or ovoid, white. Flowers 5-merous. Calyx 1.2–2 mm long, 2–2.5 mm wide, cup-shaped, thick, green, moderately pubescent with the same eglandular trichomes as stems, the calyx appendages 7–10 (rarely 5), unequal, rarely subequal, the five main appendages longer, 0.5–1.5 mm long, 0.3 mm wide, alternating with 2–5 shorter secondary appendages up to 0.7 mm long, thick, erect or slightly spreading, cylindrical or slightly compressed, inserted close to the margin, sparsely pubescent with the same trichomes as calyx tube. Corolla 4–6 (–9) mm long, 9–11 mm in diameter, thick, entirely white, stellate with interpetalar membrane, lobed 1/2 or less of the way to the base, glabrous adaxially and abaxially, the tube 2–4 mm long, the lobes 2–2.6 (–3.1) mm long, 2–2.4 mm wide, triangular, spreading, the margins papillate, the tips acute, papillate. Stamens five, equal; filaments 0.8–1.5 mm long, white, inserted on the corolla 1–1.3 mm from the base, with conspicuous auricles free, not fused to the corolla at the point of insertion; anthers (0.9–) 1.2–1.5 mm long, ellipsoid, yellow or cream, not connivent at anthesis. Gynoecium with ovary 1.5–2.3 mm long, ca. 1.7 mm in diameter, light green, ovoid; ovules more than two per locule; nectary ca. 0.4 mm tall, light green; styles homomorphic, 2.4–2.8 (–4) mm long, exserted ca. 1–1.5 mm beyond the anthers, white, cylindrical; stigma ca. 0.2 mm long, 0.2 mm wide, cream or light green, globose. Berry 7–10 mm in diameter, globose or (6–) 8–14 mm long, 5–8 mm in diameter, ellipsoid, green or green with blackish spots when immature, orange to bright red at maturity, deciduous, pungent, in some populations, non-pungent, the pericarp thick, opaque, with giant cells (endocarp alveolate); stone cells absent; fruiting pedicels 15–20 mm long, erect, strongly angled, widened distally, green; fruiting calyx 4–5 mm in diameter, persistent, not accrescent, discoid, green, the appendages 0.5–1.6 mm long, spreading or slightly recurved. Seeds 14–25 per fruit, 3.4–4 mm long, 2.8–3 mm wide, C-shaped, rarely subglobose or reniform, pale yellow, the seed coat smooth to reticulate (SM), reticulate-cerebelloid (SEM), the cells irregular in shape, the lateral walls strongly sinuate in seed body, nearly straight at margin; embryo imbricate or coiled.

#### Distribution.

*Capsicumchacoense* is a widespread species usually confined to Chaco vegetation, which extends from Bolivia and Paraguay to central Argentina (Fig. 41).

#### Ecology.

*Capsicumchacoense* is a typical component of the dry and subhumid Gran Chaco forests ranging from the inter-Andean valleys at higher elevations to the lower Chaco forests in northern Argentina and Paraguay. *Capsicumchacoense* is found in the undergrowth, beneath the shade of nurse shrubs or trees, from 50–2,700 m elevation.

#### Phenology.

Flowering from late October to March and April, fruiting from January to May or June.

#### Chromosome number.

n = 12 (Moscone 1992); 2n = 24 (Pickersgill 1977; Moscone 1990; Moscone et al. 1993, 1995, 2007).

#### Common names.

**Argentina**: Ají (Catamarca, *Brizuela 1127*; Chaco, *Aguilar 568*; Córdoba, *Fernández 17*; Corrientes, *Nicora s.n.*; Santiago del Estero, *Soriano & Barret 3592*), Bolita (Santiago del Estero, *Perrone s.n.*), Cumbaré (Córdoba, *Castellanos 217*), Cumbarí (Catamarca, *de Ance 81*; Corrientes, *Ibarrola 2988*; La Rioja, *Hunziker 4807*), Lají (Córdoba, *Lorentz s.n.*), Pipí (Formosa, *Morel 910*), Putaparió (Chaco, *Gaillard s.n.*; Córdoba, *Cocucci 4966*; Tucumán, *Schreiter 1927*), Putaqueteparió (Córdoba, *Hawkes et al. 3308*), Uchuca (Catamarca, *Vervoorst 3535*), Ají cumbarí (Catamarca, *Schickendantz 74*; Chaco, *Schulz 2*; Córdoba, *Castagnino s.n.*; La Rioja, *Giacomelli 130*), Ají chuca (Chaco, *de Ance 81*), Ají fuerte (Córdoba, *Villafañe 400*), Ají picante (Córdoba, *Botta 115*), Ají quitucho (Catamarca, *Capparelli 158*), Ají uchiquita (La Rioja, *Hunziker 4807*), Ají uchuco (Catamarca, *Falcone & Castellanos* 262), Ají del campo (Chaco, *Schulz 92*; Córdoba, *Castellanos 217*; La Rioja, *Hunziker 5088*; San Luis, *Anderson 1447*; Santa Fe, *Krapovickas 762*), Ají del monte (Catamarca, *Cabrera 1132*; Chaco, *Fortunato 1445*; Córdoba, *Spegazzini s.n.*; Salta, *Ayarde & Sidán 299*; Santiago del Estero, *Bartlett 20435*; Tucumán, *Schreiter 1927*), Ají mala palabra (Santiago del Estero, *Crespo s.n.*), Ají puta parió (San Juan, *Cortez 156*), Ají quitucho dulce (Salta, *Hunziker 1578*), Picante del monte (Jujuy, *Joergensen s.n.*), Pimiento del monte (Chaco, *Meyer 8561*); **Bolivia**: Aribibi (Santa Cruz, *Navarro* & *Vargas C. 261*), Ají del zorro (Chuquisaca, *Saravia Toledo 10343*); **Paraguay**: Ají (Boquerón, *August 18*), Cambarí (Central, *Rojas 10821*), Cumbary (Capital, *Rojas 14325*).

#### Indigenous names.

**Argentina**: Atéshuk (Chorote, Scarpa 2007), Awarañink¨i’¨i’ (Tapieté, Montani and Scarpa 2016), Chemmak’-raík’ (= picante) (Mocoví, Scarpa and Rosso 2014), Ke-ig (Guaraní, Corrientes, *Ibarrola 2988*), Kodae (Pilagá, Formosa, *Martínez Crovetto 31, Arenas 3043*), Ko’rae (Pilagá, Filipov 1994), Ko’rai’ (Pilagá, Formosa, *Arenas 1956*), Pájanak (Chorote, Scarpa 2007), Pa:nãn (Salta, *Arenas 2121*), Quihiy (Chaco, *Escobar 19*); **Bolivia**: Aguara keu (Santa Cruz, *Bourdy 2000*), Kî mi’ (Santa Cruz, *de Michel 2568*); **Paraguay**: Atés (Boquerón, *Arenas 1688*), Atic (Presidente Hayes, *Arenas 1546*), Ciaq taqatic (Presidente Hayes, *Arenas 2377*), Hũpita (Boquerón, *Arenas 1853*), Naatikgit (Boquerón, *Arenas 436*), Yemade (Toba, Presidente Hayes, *Williams et al. 140*).

#### Uses.

The pungent fruits are locally used as spicy food additives for both local and indigenous people (National Research Council 1989; Arenas and Scarpa 2007; Biurrun et al. 2007; Scarpa 2007; Martínez Crovetto 2014; Montani and Scarpa 2016; Saur Palmieri et al. 2018). In regional markets, fruits are sold fresh or as pickles in oil or vinegar (Barboza, pers. obs.). Fruits are also used in traditional medicine (see Table 3).

#### Preliminary conservation assessment.

EOO (1,724,002 km^2^); AOO (952 km^2^). *Capsicumchacoense* is widespread across subtropical Chaco forests from Bolivia to Paraguay and is assigned the Least Concern (LC) category.

#### Discussion.

*Capsicumchacoense* belongs to the Baccatum clade (Carrizo García et al. 2016). It has a peculiar dwarf habit, profusely branched from the thick rootstock with small leaves, solitary flowers, entirely white corollas, filaments with conspicuous free auricles at point of insertion to the corolla and abundant red mature fruits per plant. Its most variable character is pubescence. Typically, plants are sparsely pubescent, but densely tomentose populations grow in north-eastern Argentina (Chaco) and Paraguay (Alto Paraguay), which, in the past, were recognised as a separate variety, C.chacoensevar.tomentosum (Hunziker 1950). Some of these tomentose populations were included in Hunziker’s circumscription of C.microcarpumvar.tomentosum (= *C.rabenii*) (Hunziker 1950), leading to confusion between the two taxa.

A second variable feature is the number and degree of development of the calyx appendages. The most common condition is the presence of calyces with 10 unequal appendages, the five main ones longer and alternating with five shorter, all of them usually well-developed (that is, appendages exceed the truncate calyx edge). In some cases, the shorter appendages vary from 2–5, with some exceeding the calyx edge and others scarcely noticeable, reduced to a mucro. Calyces with this second pattern create confusion in the identification of fruiting specimens, especially when all short appendages are not well-developed. Often, these specimens are annotated in herbaria as *C.baccatum* from which *C.chacoense* can be distinguished by its smaller and entirely white corollas.

The fruits of *C.chacoense* are locally very appreciated for their flavour and high pungency (see references in Uses). However, *C.chacoense* is naturally polymorphic for the production of capsaicinoids, such that completely pungent and completely non-pungent individuals co-occur in some Bolivian populations (Tewskbury et al. 2006); non-pungent fruits have also been recorded in Argentinean populations (e.g. Salta, *Hunziker 1578*). Tewksbury et al. (2008b) demonstrated that the pungency variation found in *C.chacoense* may be an adaptive response to selection by a microbial pathogen. Thus, capsaicinoids protect the fruits and seeds from fungal pathogens (*Fusarium*) that severely reduce seed viability.

There is little information about which birds, the most effective dispersers of *Capsicum* seeds, eat *C.chacoense* fruits. Information from a herbarium label (Bolivia, *Debouck 3016*) suggests that small parrots eat the fruits of this species. Tewskbury et al. (2006) mentioned *Elaeniaparvirostris* ‘fiofío pico corto’ (Fam. Tyrannidae) and *Turdusamaurochalinus* ‘zorzal chalchalero’ (Fam. Turdidae) as the major dispersers of *Capsicum* seeds in Bolivia.

In the protologue of *C.chacoense*, Hunziker (1950) indicated “Typus speciei. (ATH)” [Armando Teodoro Hunziker] referring to his own Herbarium now housed at CORD. In CORD, there are three mounted sheets of *Hunziker 7340*, two of them (sheets A and B) are labelled as “holotype” (barcodes CORD00003919, CORD00003920) and the third one (CORD00087962) has a label in Hunziker’s hand, indicating it is a duplicate specimen (probably with the intention to be donated elsewhere, but now mounted and accessioned at CORD). Sheet B contains a complete fruiting young individual with a label where the word Typus was handwritten by Hunziker as an indication that it should be the holotype; we are designating this sheet B (CORD00003920) as the lectotype.

**Figure 45.**
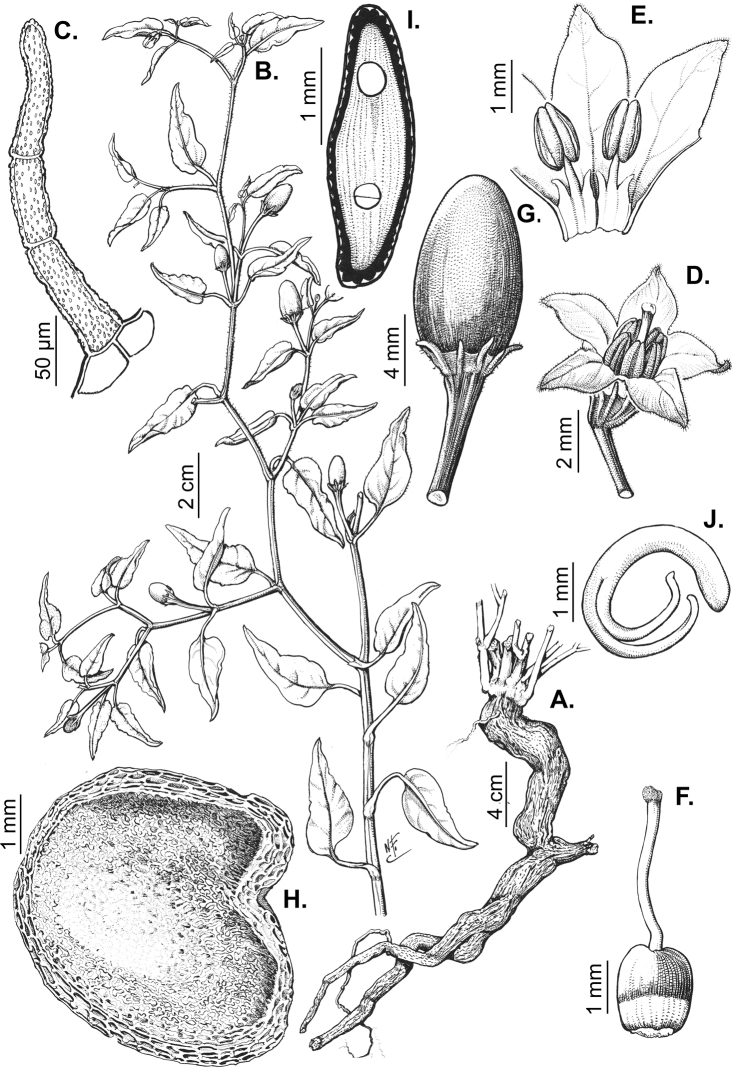
*Capsicumchacoense***A** root **B** fruiting branch **C** eglandular trichome of the leaf **D** flower **E** sector of opened corolla **F** gynoecium **G** fruit **H** seed **I** seed, in cross section **J** embryo **A, C–F** from *Hunziker 18572***B, G–J** from *Hunziker et al. 25388*. Drawn by N. de Flury. Published in Barboza (2013), courtesy of the Board of the Instituto Darwinion (San Isidro, Buenos Aires, Argentina), reproduced with permission.

**Figure 46.**
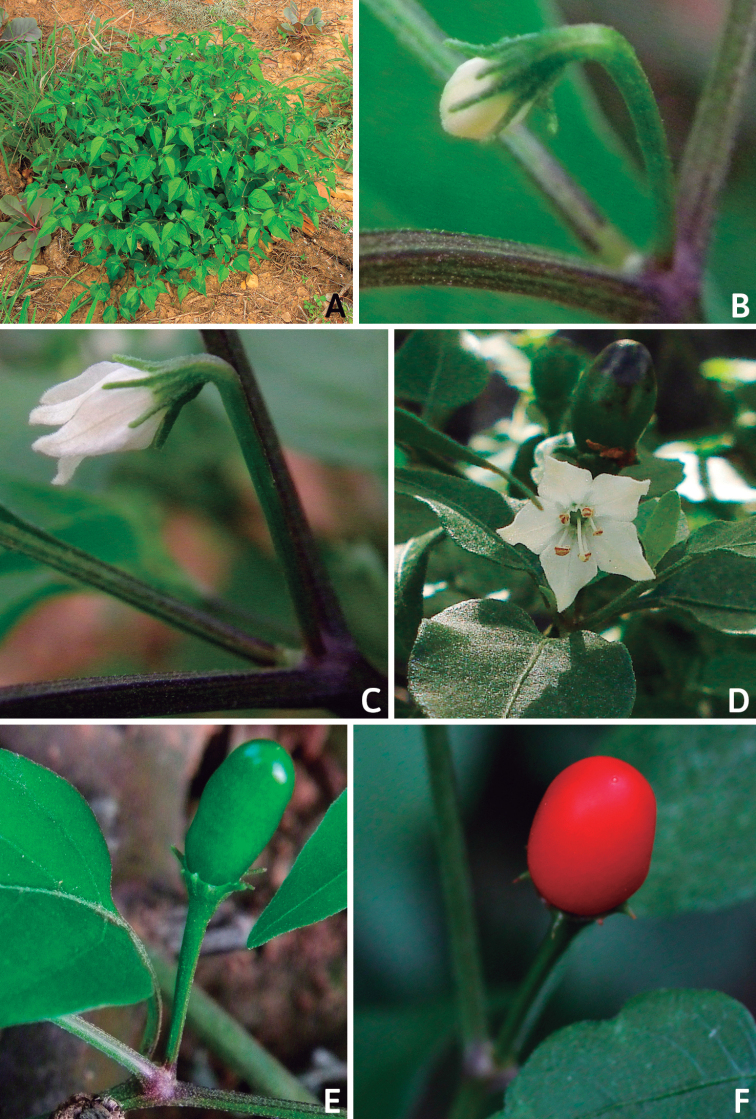
*Capsicumchacoense***A** plant **B** flower bud **C** flower, in pre-anthesis **D** flower, in front view **E** immature fruit **F** mature fruit **A–F** from *Barboza et al. 4910*. Photos by G.E. Barboza.

#### Specimens examined.

See Suppl. material 4: Appendix 4.

### ﻿11. Capsicum chinense

Taxon classificationPlantaeSolanalesSolanaceae

Jacq., Hort. Bot. Vindob. 3: 38. t. 67. 1776.

6FE51356-7657-5E38-8E42-2495E49F9526

Fig. 47

Capsicum cerasiforme Mill., Gard. Dict. ed. 8, no. 5. 1768. Type. Cultivated at the Chelsea Physic Garden, London, seeds from Spanish West Indies (no specimens cited).
Capsicum cerasiforme Lam., Tabl. Encycl. 2: 26. 1794, nom. illeg., not Capsicumcerasiforme Mill. (1768). Type. Brazil. “E Brafilia” (lectotype, designated here: P [P00357733]).
Capsicum luteum Lam., Tabl. Encycl. 2: 26. 1794. Type. India. “Ex India. Sonnerat” P. Sonnerat s.n. (lectotype, designated here: P [P00357731]).
Capsicum cerasiforme Willd., Enum. Pl. [Willdenow] 1: 242. 1809, nom. illeg., not Capsicumcerasiforme Mill. (1768). Type. Cultivated in the Berlin Botanic Garden, Germany, “Habitat…..” Capsicumcerasiforme [sheet] 1, *herb. Willdenow* (lectotype, designated here: B [B-W04427-01-0]).
Capsicum milleri Roem. & Schult., Syst. Veg., ed. 15 bis [Roemer & Schultes] 4: 563. 1819, nom. illeg. superfl. Type. Based on Capsicumcerasiforme Mill. (cited in synonymy).
Capsicum indicum Dierb. var. luteum (Lam.) Dierb., Arch. Apotheker-Vereins Nordl. Teutschl. 30: 24. 1829. Type. Based on Capsicumluteum Lam.
Capsicum indicum Dierb. var. humifusum Dierb., Arch. Apotheker-Vereins Nordl. Teutschl. 30: 26. 1829. Type. Based on Capsicumcerasiforme Mill. (cited in synonymy).
Capsicum indicum Dierb. var. ochranthum Dierb., Arch. Apotheker-Vereins Nordl. Teutschl. 30: 27. 1829. Type. Based on Capsicumcerasiforme Willd. (cited in synonymy).
Capsicum dichotomum Vell., Fl. Flumin.: 61. 1829 (“1825”); Fl. Flumin. Icon. 2: t. 9. 1831 (“1827”), as “Capsicumconicum”. Type. Brazil. [Rio de Janeiro]: “Colitur hortis” (lectotype, designated by Knapp et al. 2015, pg. 824: [illustration] Original parchment plate of Flora Fluminensis in the Manuscript Section of the Biblioteca Nacional, Rio de Janeiro [cat. no.: mss1198651_012] and later published in Vellozo, Fl. Flumin. Icon. 2: t. 9. 1831).
Capsicum odoriferum Vell., Fl. Flumin.: 61. 1829 (“1825”); Fl. Flumin. Icon. 2: t. 8. 1831 (“1827”). Type. Brazil. [Rio de Janeiro]: “Colitur hortis” (lectotype, designated by Knapp et al. 2015, pg. 824: [illustration] Original parchment plate of Flora Fluminensis in the Manuscript Section of the Biblioteca Nacional, Rio de Janeiro [cat. no.: mss1198651_011] and later published in Vellozo, Fl. Flumin. Icon. 2: t. 8. 1831).
Capsicum conicum Vell., Fl. Flumin. Icon. 2: t. 9. 1831. (“1827”), nom. illeg., not Capsicumconicum G.Mey. (1818). Type. Based on Capsicumdichotomum Vell.
Capsicum toxicarium Poepp., Not. Natur- Heilk. 32: 228. 1832. Type. No locality cited (no specimens cited, no original material found). Peru. “Peruvian hispan., Maynas alto”, Jan 1830, *E.F. Poeppig 2220* (neotype, designated here: W [acc. # 0102196].
Capsicum cereolum Bertol., Hort. Bonon. Pl. Nov. 1: 6. t. 2. 1838. Type. Cultivated in Bologna, Italy “Nascitur in Brasilia unde semina ad nos attulit, et comiter dedit Eq. NUNNEZIUS” (lectotype, designated here [illustration]: Bertoloni, Hort. Bonon. Pl. Nov. 1: 6. t. 2. 1838).
Capsicum cordiforme Mill. var. subangulosum Fingerh., Monogr. Capsic.: 29. 1832. Type. No locality cited (no specimens cited; lectotype, designated here: [Fingerhuth, Monogr. Capsic. Tab. IX d. 1832]).
Capsicum cordiforme Mill. var. majus Fingerh., Monogr. Capsic.: 30. 1832. Type. No locality cited (no specimens cited; lectotype, designated here [illustration]: Fingerhuth, Monogr. Capsic. Tab. IX e. 1832).
Capsicum cordiforme Mill. var. minus Fingerh., Monogr. Capsic.: 30. 1832. Type. No locality cited (no specimens cited; lectotype, designated here [illustration]: Fingerhuth, Monogr. Capsic. Tab. X a. 1832).
Capsicum cordiforme Mill. var. olivaeforme Fingerh., Monogr. Capsic.: 30. 1832. Type. No locality cited (no specimens cited; lectotype, designated here [illustration]: Fingerhuth, Monogr. Capsic. Tab. X b. 1832).
Capsicum cordiforme Mill. var. globosum Fingerh., Monogr. Capsic.: 30. 1832. Type. “Crescit in Indiis et America meridionali” (no specimens cited; lectotype, designated here [illustration]: Fingerhuth, Monogr. Capsic. Tab. X c. 1832).
Capsicum ustulatum Paxton, Paxton’s Mag. Bot. 5: 197. 1838. Type. Cultivated at Chatwsworth House, England (no specimens cited; lectotype, designated here: [illustration], Paxton, Paxton’s Mag. Bot. 5: [un-numbered plate opposite] 197. 1838).
Capsicum cerasiforme Willd. var. maurocarpum Dunal, Prodr. [A. P. de Candolle] 13(1): 422. 1852. Type. No locality cited (no specimens cited; lectotype designated here: [illustration] “SolanumCapsicum dictum perenne minuserectum” Weinemann, Phytanthoza Iconogr. 4: 349, t. 930 c. 1745).
Capsicum oxycarpum Dunal, Prodr. [A. P. de Candolle] 13(1): 426. 1852. Type. Brazil. Rio de Janeiro, 1831, *M. Blanchet 90* (holotype: G-DC [G00200070]; isotype: MPU [MPU023041]).
Capsicum cordiforme Mill. var. subsulcatum Dunal, Prodr. [A. P. de Candolle] 13(1): 427. 1852. Type. No locality cited (no specimens cited; lectotype designated here: [illustration] Fingerhuth, Monogr. Capsic. Tab. IX c. 1832).
Capsicum annuum L. var. chinense (Jacq.) Alef., Landw. Fl.: 132. 1866. Type. Based on Capsicumchinense Jacq.
Capsicum annuum L. var. luteum (Lam.) Alef., Landw. Fl.: 132. 1866. Type. Based on Capsicumluteum Lam.
Capsicum annuum L. var. milleri (Roem. & Schult.) Alef., Landw. Fl.: 133. 1866. Type. Based on Capsicummilleri Roem. & Schult.
Capsicum grossum Willd. var. cerasiforme (Lam.) C.B.Clarke, Fl. Brit. India 4: 239. 1883. Type. Based on Capsicumcerasiforme Lam.
Capsicum annuum L. var. cerasiforme (Mill.) Irish, Rep. (Annual) Missouri Bot. Gard. 9: 92. 1898. Type. Based on Capsicumcerasiforme Mill.
Capsicum frutescens L. var. cerasiforme (Mill.) L.H.Bailey, Gentes Herbarum 1: 129 1923. Type. Based on Capsicumcerasiforme Mill.
Capsicum assamicum J.Purkay. & Lok.Singh, Ozean J. Appl. Sci. 5(1): 56. 2012. Type. India. Assam: Tezpur, 157 m, 25 May 2008, *Purkayastha* & *Singh*, DRLT 12 (holotype: CAL [by error, BSI in protologue, n.v.]; isotype: CAL [by error, DRLT and BSI in protologue, n.v.]).

#### Type.

Cultivated in Vienna, Austria [“Hort. Bot. Vindob.”], *N.J. von Jacquin s.n.* (lectotype, designated here: W [acc. # 0080115]).

#### Description.

Low, erect, short-lived subshrubs or rarely shrubs, 0.5–1.5 (–2.5) m tall, with the main stem (0.5–) 0.8–1.5 cm in diameter at base, few to much branched from near the base. Young stems 4-angled, fragile, green or greenish-brown, glabrous to glabrescent, with spreading, simple, uniseriate, (2–) 4–5-celled, eglandular trichomes 0.07–0.7 mm long; nodes solid, green or purple; bark of older stems light brown or green with light brown stripes, glabrous to sparsely pubescent; lenticels absent. Sympodial units difoliate, the leaves geminate; leaf pair unequal in size, similar in shape. Leaves membranous, concolorous or slightly discolorous, dark green above, pale green below, glabrescent to sparsely pubescent, with simple, 4–9-celled, eglandular trichomes 0.6–1.2 mm long, especially along the mid-vein or with a tuft of trichomes in the basal vein axils abaxially; blades of major leaves (4–) 5.25–10 (–15.5) cm long, 2.4–4 (–7) cm wide, ovate to elliptic, the major veins 4–5 (–6) on each side of mid-vein, the base attenuate or truncate and rather unequal, the margins entire, the apex short-acuminate, acuminate or acute; petioles 0.5–3 (–3.5) cm, glabrous to sparsely pubescent; blades of minor leaves similar in shape, 4–5.5 cm long, 2–2.5 cm wide; petioles 0.7–1.5 cm long, with the same pubescence as the major leaves. Inflorescences axillary, 2–4 (–5) flowers per axil, occasionally solitary flower; flowering pedicels 12–20 (–30) mm long, angled, erect or slightly spreading, geniculate at anthesis (wild forms) or pendent and non-geniculate (domesticated forms), green or green with purple lines, glabrous to moderately pubescent, the eglandular trichomes short, antrorse; pedicels scars conspicuous, slightly corky. Buds ellipsoid, cream or greenish-white. Flowers 5–6-merous. Calyx 1–2.5 (–3) mm long, 3–4 mm wide, cup-shaped, green, pentagonal or hexagonal in outline, the main veins strongly marked, the calyx appendages absent or with 5–6 mucro-like appendages, glabrous to moderately pubescent with similar short or long eglandular trichomes as the stems. Corolla 5 (–6)-merous, (5–) 6.5–8 mm long, 10–15 (–20) mm in diameter, dull white or greenish-white, occasionally with purple spots outside and within, stellate with interpetalar membrane, lobed nearly 2/3 of the way to the base, glabrous adaxially and abaxially, the tube (2–) 2.5–3 mm long, the lobes 3.5–5 mm long, 2–3.5 mm wide, triangular, spreading, the margins papillate, the tips acute, cucullate, papillate. Stamens 5 (–6), equal; filaments 1–1.3 mm long, white, cream or purple, inserted on the corolla 1–1.5 mm from the base, with auricles fused to the corolla at the point of insertion; anthers (1–) 1.38–2.05 mm long, blue or bluish-grey, very rarely yellow or greenish-white, broadly ellipsoid or ellipsoid, connivent or not connivent at anthesis, the connective sometimes wide and clearer. Gynoecium with the ovary 2–2.5 mm long, 2.5–3.5 mm in diameter, 2–3 (–4)-carpelar, light green, subglobose; ovules more than two per locule; nectary ca. 0.5 mm tall; styles heteromorphic, 3–4.1 mm, included, at the same level to the stamen length or exserted ca. 2 mm beyond the anthers, lilac or white, cylindrical; stigma ca. 0.15 mm long, ca. 0.5 mm wide, minute, discoid, pale green or pale yellow. Berry < 10 mm in diameter, subglobose and orange to red (in wild populations), highly variable in size, shape and colour (in semi-domesticated or domesticated cultivars): subglobose or triangular, 10–20 mm in diameter, to long-triangular or campanulate, 30–60 (–100) mm long, 20–30 mm in diameter, some blocky, with the apex pointed, blunt or long-acuminate and upcurved and the base obtuse or truncate, green, yellow, brown or purple when immature, pale yellow, yellow, dark brown, orange, red or vermilion-scarlet at maturity, deciduous or persistent, very pungent (sometimes non-pungent), the pericarp thick, opaque, with giant cells (endocarp alveolate); stone cells absent; fruiting pedicels 15–45 (–55) mm long, thick, angled, strongly widened distally, erect and rigid (wild) or pendent and curved (domesticated); fruiting calyx 5–10 mm in diameter, persistent, not accrescent, discoid or shallowly cup-shaped, sometimes reflexed, with a strong annular constriction at junction with the pedicel (wild and domesticated), sometimes the margin ripped, green. Seeds 14–35 per fruit, (2.7–) 3–4 mm long, (2.5–) 3–3.5 mm wide, C-shaped or subglobose, pale yellow or nearly white, the seed coat smooth (SM), cerebelloid (SEM), the cells irregular in seed body, polygonal at margins, the lateral walls sinuate in the seed body, straight at margins; embryo imbricate.

**Figure 47.**
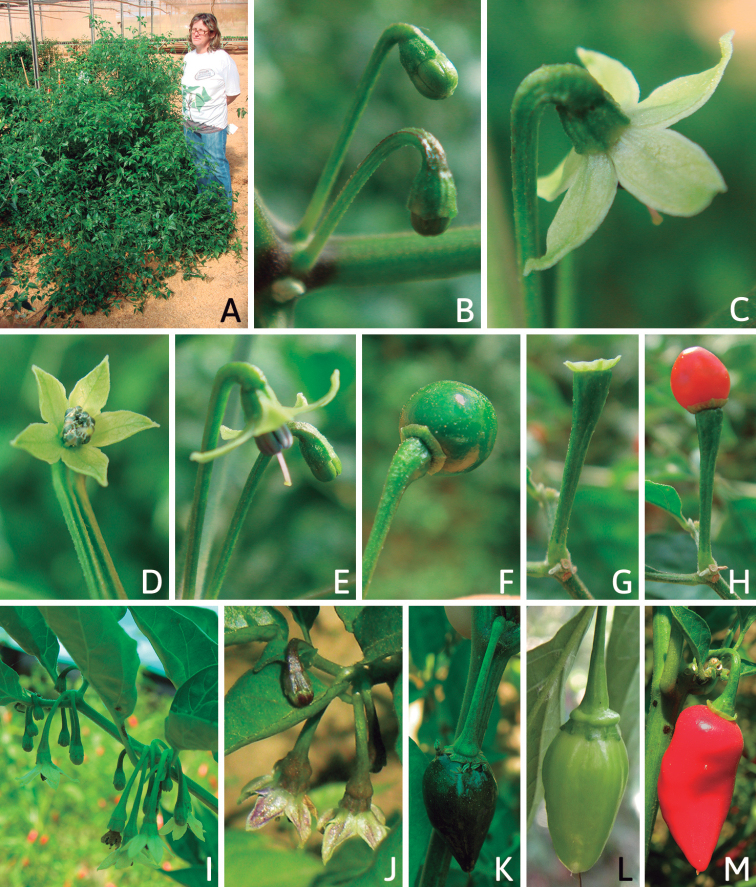
*Capsicumchinense* (**A–H** from wild plants **I–M** from domesticated plants) **A** plant **B** flower buds **C** flower on geniculate pedicel **D** flower, in front view **E** flower, in full anthesis **F** immature fruit **G** fruiting pedicel **H** mature fruit **I** flowering branch **J** flowers on pendent pedicels **K, L** immature fruits **M** mature fruit **A–H** no specimen vouchers (cult. in Banco de Germoplasma de Hortaliças, Embrapa/Hortaliças, Brasília-DF, Brazil), photos taken *in situ* by L. Bianchetti **I–M** no specimen vouchers (cult. in Pairumani, Cochabamba-Bolivia), photos taken *in situ* by G.E. Barboza.

#### Distribution.

**Figure 48.**
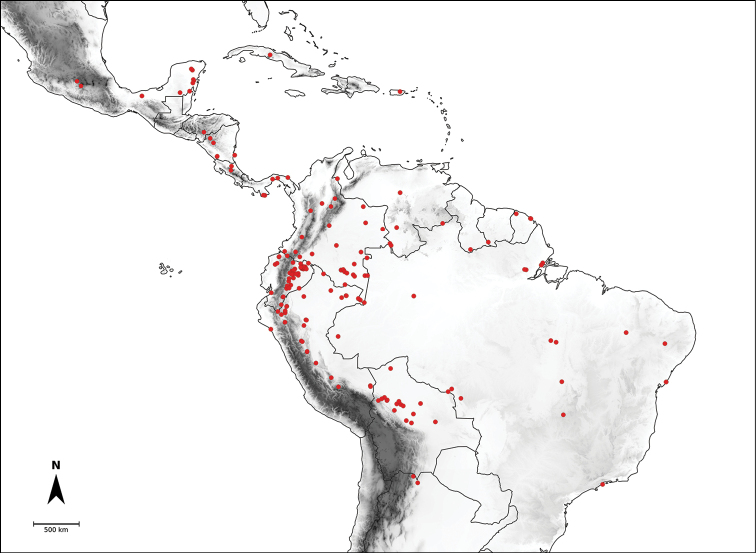
Distribution of *C.chinense*.

*Capsicumchinense* wild forms were thought to originally occur in the north-central Amazon Basin lowlands (Brazil), where domestication is thought to have occurred (Pickersgill 1971; Eshbaugh 1993). Pickersgill (1984) stated that wild *C.chinense* is confined to the lowlands of the Amazon, Orinoco and eastern Brazil, while Moses et al. (2014) suggested that populations in Central America and the Caribbean may have been primarily derived from progenitors from the Upper Amazon Region and later diverged through geographical isolation. Wild populations can still be found in the nature, but they are difficult to locate or are restricted to remote areas. Wild *C.chinense* (indigenous name: Pimi’ró) have been found in Roraima State (Brazil) (Barbosa et al. 2002, 2008; Busso et al. 2003; Nascimento-Filho et al. 2007; Romero da Cruz and Forni Martins 2015), which confirms the occurrence of probable ancestors in the eastern lowlands of Amazonia (Fig. 48).

*Capsicumchinense* has been introduced into United States of America (Bosland and Votava 2000), Mexico through Cuba (González Estrada et al. 2010; Ruiz-Lau et al. 2011; López Castilla et al. 2019) and Central and South America where is found cultivated or escaped from cultivation; it has also been introduced outside the Americas (Eastern Europe, Africa, Asia: China, Japan, Taiwan, India and others) mainly by Portuguese explorers (Eshbaugh 1983; Andrews 1993; Meghvansi et al. 2010).

#### Ecology.

*Capsicumchinense* grows in wet tropical and subtropical forests where native communities cultivate it around their chakras or in-home gardens, between 100 and 800 (–1,800) m elevation. Wild populations in northern Brazil grow in lowlands and disperse spontaneously in fallow agriculture areas, before the beginning of the rainy period (Barbosa et al. 2002).

#### Phenology.

Flowering and fruiting all year in most parts of its range.

#### Chromosome number.

n = 12 (Pickersgill 1966; Pozzobon and Schifino-Wittman 2006); 2n = 2x = 24 (Pickersgill 1977; Moscone et al. 2003, 2007; Pozzobon et al. 2006; Romero da Cruz and Forni Martins 2015).

#### Common names.

**Argentina**: Ají bolita (Salta, *Hilgert 2060*). **Belize**: Jabanero pepper (Corozal, *Balick 2211*); **Bolivia**: Ají (Beni, *Williams 937*; La Paz, *Williams 774*), Aji mote (Santa Cruz, *Krapovickas & Schinini 32488*), Ají soliman (Cochabamba, *Thomas 1093*), Ají trompillo (Cochabamba, *Thomas 704*), Ají grande rojo (Beni, *Rivero 428*); **Brazil**: Baianinha (Rondônia, *Teles Walter 579*), Canaimé, Chumbinho, Malaguetão, Morangão, Moranguinho, Murici, Murupi, Pimentãozinho, Peão-amaerlo, Peão-vermelho, Peixe-boi, Pimenta amarela, Pimenta-moranga, Pimenta murici, Pimenta ornamental, Pimenta-roxa, Chifre-de-carneiro, Chifre-de veado, Cumari-do-Pará, Esporão-de-galo, Olho-de-cará, Olho-de-carangueijo, Olho-de-mutum, Olho-de-peixe (falsa), Olho-de-peixe (verdadeira), Pimenta-vermelha longa, Unha-de-gato, Pimenta-de-cherio amarela (ardosa), Pimenta-de-cheiro longa, Pimenta-de mesa-ardosa (Roraima, Barbosa et al. 2006), Pimenta (Goiás, *Amaral 139*; São Paulo, *Hoehne s.n*.), Habanero vermelha (São Paulo, *Lélis et al. 12*), Pimenta açaí (Amapá, *Pereira et al. 1918*), Pimenta acerola (Amapá, *Pereira et al. 1816*), Pimenta-biquinho (Mina Gerais, Goiás, Carvalho et al. 2006), Pimenta boliviana (Rondonia, *Teles Walter et al. 572*), Pimenta camapú (Amapá, Pereira et al. 2011), Pimenta cambuí (Bahía, *Medeiros et al. 196*), Pimenta-doce (Rondônia, *Teles Walter 567*), Pimenta habanero (Carvalho et al. 2006), Pimenta-murupi (Região Norte, Carvalho et al. 2006), Pimenta queimosa (Amapá, *Pereira et al. 1905*), Pimenta do Curupira (Roraima, Barbosa et al. 2002; Nascimento Filho et al. 2007), Pimenta amarela ardosa (Amapá, *Pereira et al. 1908*), Pimenta de bode (Região Centro-Oeste, Carvalho et al. 2006), Pimenta de cheira (*Pickersgill RU72-175*), Pimenta de cheiro (Bahía, *Medeiros et al. 182*; Rio de Janeiro, *Gonçalves & Kaketru 2*; Paraíba, *Gadelha Neto 1748*), Pimenta queimosa amarela (Amapá, *Pereira et al. 1904*), Pimenta roxa alongada (São Paulo, *Hoehne s.n.*), Pimenta de cheiro amarela (Amapá, *Pereira et al. 1901*), Pimenta de cheiro legítimo (Río de Janerio, *Freire de Carvalho s.n.*), Pimenta de cheiro redondinha (Pereira et al. 2011), Pimenta de cheiro tacacá (Amapá, Pereira et al. 2011), Pimenta-de-cheiro vermelha (Amapá, *Pereira et al. 1831*; Roraima, Barbosa et al. 2006), Pimenta-dedo-de-papagaio (Bahía, *Medeiros et al. 200*), Pimenta de cheiro de panela (Amapá, Pereira et al. 2011); **Colombia**: Ají (Meta, *Quevedo et al. 1816*, Santander, *Rosado 01*), Trompito (Vichada, *García Guzmán s.n.*), Aji amarillo (Amazonas, *Cárdenas et al. 9439*), Ají pecas (Caquetá, *Cárdenas et al. 9311*), Aji trompito (Caquetá, *Cárdenas et al. 9356*), Ojito de Lucía (Caquetá, *Cárdenas et al. 9331*), Ají de babilla (Amazonas, *Cárdenas et al. 9431*); **Costa Rica**: Chile, Chile chiricano, Chile panameño (Bohs 2015); **Ecuador**: Ají (Napo, *Ríos 429*; Morona-Santiago, *Villegas & Meneses 16*), Ají botoncillo (Guayas, *Holmgren & Heilborn s.n.*); **French Guiana**: Péppé (Cayenne, *Fleury 124*); **Jamaica**: Scotch bonnet (Jamaica, Bosland and Votava 2000); **Mexico**: Habanero (Quintana Roo, *Palma Gutiérrez 85-69*), Rosita (Yucatán, López Castilla et al. 2019), Chile cimarrón (México, *Hinton 4336*), Chile costeño (Campeche, *Ramírez A. 56*), Chile habanero (Yucatán, *Ucan 4652*), Chile xalapeño (Quintana Roo, *Palma Gutiérrez 85-25*), Chile de árbol (Michoacán, *Soto Núñez & Silva R. 3842*); **Panamá**: Ají Chombo (Bosland and Votava 2000); **Peru**: Ají (Lambayeque, *Llatas Quiroz 3454*; San Martín. *Plowman & Schunke V. 11695*), Arequipeño (Lima, *Velarde Núñez* 8), Charapita (Iquitos, Bosland and Votava 2000), Chinchi-uchu (Peru, Andrews 1984), Esticana (Cuzco, *Ferreyra 16384*), Limos (Lima, *Velarde Núñez* 16), Pucunuchu (San Martín, *Plowman & Schunke 11497*), Ají amarillo (Pasco, *Huamán et al. 0317*), Ají bravo (Loreto, *Plowman 2457*), Aji Charapa (Iquitos, Bosland and Votava 2000), Ají Dulce (Loreto, *Plowman 2458*), Ají mutecillo (Amazonas, *Campos* & *Vargas 6945*), Ají rojo o ají chanchamayo (Pasco, Oxapampa, *Huamán et al. 0320*), Ají Panca (Bosland and Votava 2000), Ají Pucomucho (Bosland and Votava 2000), Ajuju aji (Loreto, *Hormia 2194*), Colorado Cuzco (Lima, *Velarde Núñez 4*), Colorado Trujillo (Lima, *Velarde Núñez* 9), Munición uchu (Loreto, *Williams 5225*), Rojo Arequipa (Lima, *Velarde Núñez 12*), Ají corazón de pollo (Madre de Dios, *Pesha Baca 95*); **Puerto Rico**: Rocotillo (Bosland and Votava 2000); **Trinidad and Tobago**: Congo (Bosland and Votava 2000); **United Stated of America**: Datil (Florida, Bosland and Votava 2000), Rocotillo (Texas, Andrews 1984).

#### Indigenous names.

**Bolivia**: Bidó (Tacana, La Paz, *Williams 1177*), Cheti (Trinitario, Cochabamba, *Thomas 704*), Ta’ (Beni, *Davis 1066*), Hchetgi (Trinitario, Cochabamba, *Thomas 1092*), Tata (Yuqui, Cochabamba, *Martínez et al. 246*), Uchu (Quechua, La Paz, *Williams 772*), Winno (Yuracare, Cochabamba, *Thomas 1093*), Winno manera (Yuracare, Cochabamba, *Thomas 704*); **Brazil**: Pimi’ró (Macuxi, Roraima, Barbosa et al. 2002; Nascimento Filho et al. 2007); **Colombia**: Aati (Curripaco, Guainía, *Espina et al. 182*); Chamouiju (Colono Huitoto, Caquetá, *Cárdenas et al. 9331*), Doehoe (Amazonas, *Cárdenas et al. 9439*), Duaó (Caquetá, *Cárdenas et al. 9385*), Gubsovia (Makuna, Amazonas, *Cárdenas et al. 9431*), Kukunarí (And, Amazonas, *Castro & Matapí 565*), Jade deé (Mui, Amazonas, *Castro & Ramírez 132*), Kukunari (Amazonas, *Castro & Matapí* 565), Fakiki deé (Mui, Amazonas, *Castro & Rodríguez 309*), Lole-ra (Norte de Santander, *Solange 8*), Tada tarre (Sáliba, Casanare, *Camargo 020*), Wijichi-taku (And, Amazonas, *Castro & Matapí 561*); **Ecuador**: Atalba ucho (Kichwa, Sucumbíos, *Moya & Reyes 207*), Ampi (Shuar, Zamora-Chinchipe, *Santín et al. 102*), Ccoma (Cofán, Sucumbios, *Cerón 20848*), Giimo (Oncaye, Napo, *Davis & Yost 993*), Giimo (Huaorani, Napo, *Miller & Yépez 651*), Giimoñabu (Huaorani, Napo, *Miller & Yépez 651*), Guimo (Huaorani, Orellana, *Naranjo & Freire 329*), Giimohue (plant) (Huaoroni, Napo, *Miller & Yépez 651*), Guimuwe (Huaorani, Orellana, *Freire & Naranjo 612*), Hueapia (Secoya, *Cerón 62013*), Imiá (Shuar Jívaro, Pastaza, *Lewis et al. 13960*), Jinia (Shuar, Morona Santiago, *Pujupet RBAE 1005*), Juminialinae ([Guahibo?] Casanare, *Camargo 020*), K^h^oma (Kofán, Napo, *Pinkley 230*), Munisión (Kichwa, Orellana, *Carrillo & Reyes 434*), Ochabia (Siona, Sucumbíos, *Miranda & Moya 437*), Piujimia (Orellana, *Herrera & Guerrero 186*), Ucho (Quechua, Napo, *Miller et al. 2404*), Úchu (Achuar Jívaro, Pastaza, *Lewis et al. 13960*), Aji ucho (Quechua, Orellana, *Reyes & Carrillo 773*), Anya bia (= snake pepper) (Napo, *Vickers 178*), Arara uchu (Quechua, Orellana, *Kohn 1613*), Araya ucho (Quechua, Sucumbíos, *Reyes & Moya 234*), Araray-uchu (Quechua, Napo, *Balslev & Irvine 4598*), Biruti uchu (Quechua, Orellana, *Kohn 1611*), Butun uchu (Quechua, Napo, *Ríos & Oña 430*), Chipiri k^h^uma (Kofán, Napo, *Pinkley 256*), Cua K^h^oma (Kofán, Napo, *Pinkley 544*), Palanda uchu (Quechua, Orellana, *Kohn 1612*), Puca uchu (Quechua, Orellana, *Kohn 1896*), Sasi uchu (Quechua, Orellana, *Kohn 1611*), Tiupi tio (Cayapa, Esmeraldas, *Kvist 40566*), Tɛsi k^h^uma (Kofán, Napo, *Pinkley 257*), t^h^ot^h^ao k^h^uma (Kofán, Napo, *Pinkley 259*), Tota cu’ma (Kofán, Napo, *Cerón et al. 5849*), Uchu muyu (Quechua, Napo, *Ríos 429*), Wea bia (Siona, Napo, *Vickers 115*), Bula muyu uchu (Quechua, Orellana, *Kohn 1910*), Dio tape fin chuno (Cayapa, Esmeralda, *Kvist 40586*); **Guyana**: Há-ka-pu-tá (Cuyuni-Mazaruni, *Tillett & Tillett 45424*); Tor-tor-oi-ma (Cuyuni-Mazaruni, *Tillett & Tillett 45627*); **Mexico**: Habanero ik (Maya, Quintana Roo, *Gutiérrez 24*), Maax-ik (Maya, Quintana Roo, *Gutiérrez 388*), X-Kat ik (Quintana Roo, *Palma Gutiérrez 85-24*); **Peru**: Imia (Achual Jívaro, Loreto, *Lewis et al. 11207*), Tots (Yanesha, Pasco, *Huamán et al. 0317*), Yaájima (Amazonas, *Kayap 652*); **Surinam**: Ah-se-se (Kaxuyana, Kwamala, *Plotkin 139*), Pepra (Sranam, Kwamala, *Plotkin 139*), Pom-we (Tirio, Kwamala, *Plotkin 139*).

#### Uses.

*Capsicumchinense* has medicinal, ornamental and food uses. Indigeneous communities and rural people widely cultivated this species around their homes to consume the fruits raw, cooked or used as a spice (Barbosa et al. 2002). The consumption of the fruits of this species has spread globally and are highly appreciated (sweet and pungent) due to its fleshiness and distinct aroma (Kurian and Starks 2002; Basu and De 2003; Gahungu et al. 2011) in sauces and powders.

In addition to medicinal properties indicated in Table 3, *C.chinense* has spiritual uses which have sometimes been recorded by researchers on herbarium specimen labels. For example, in Ecuador (Comunidad Oncaye, *Davis & Yost 993*), fruits were used by a shaman’s wife in the termination of an ayahuasa session, (Comunidad Bataburo, *Freire & Naranjo 612*), the entire plant is used to cure “espanto” (fearfulness) and (Comunidad Indillama, *Reyes 502*) and the plant is used to cure illness caused by the “mal viento”, an evil spirit of the air. In Peru (Comunidad Palcazú, *Huamán et al. 317*), the smoke of a single dried fruit is used to help children when they are tearful. In Brazil (Roraima; Comunidad Macuxi, Barbosa et al. 2002), the common name of the species is an allusion to ‘curupira’, a supernatural being, guardian of the Amazon forests in Tupi mythology (Gomez Platero and Palma Ehrichs 2011). In addition, outside the Americas, *C.chinense* has a broad spectrum of ethnopharmacological applications and medicinal properties (e.g. see Meghvansi et al. 2010).

#### Preliminary conservation assessment.

EOO (18,657,305 km^2^); AOO (712 km^2^). *Capsicumchinense* is a very widespread cultivated species worldwide; we assign the status of Least Concern (LC).

#### Discussion.

*Capsicumchinense* belongs to the Annum clade (Carrizo García et al. 2016). It is most popularly known as Habanero (Mexico and United States of America) or Scotch Bonnet (Caribbean Islands), but it has numerous common and indigenous names in South America (see above and Baba et al. 2016), as well as landrace and cultivar names (e.g. Carolina Reaper, Bhut Jolokia, Red Savina, Congo pepper, NuMex Suave, NuMex Trick-or-Treat). Many of the names refer to differences in the fruit’s pungency, shape or colour (Bosland and Votava 2000; Votava and Bosland 2004; Bosland and Baral 2007; Bosland et al. 2012; Bosland and Coon 2015).

Ever since Smith and Heiser (1957) first recognised *C.chinense* as a cultivated species in the genus, its recognition as an independent entity and as a member of the *C.annuum* complex (*C.annuum*-*C.frutescens*-*C.chinense*) has been debated in literature (see Baral and Bosland 2004; Eshbaugh 2012 and references therein), with arguments based on morphological, cytogenetic, biochemical (isoenzymes), phylogenetic and reproductive evidence. Recently, Raveendar et al. (2017) established the phylogenetic relationships of 13 complete chloroplast genome sequences belonging to the Solanaceae (five *Capsicum* species included). This study showed that the *C.chinense* cp genome is much closer to C.annuumvar.glabriusculum, wild progenitor of *C.annuum*, than any other sampled *Capsicum* species (C.annuumvar.annuum, *C.frutescens*, *C.baccatum* and *C.lycianthoides*). This indicates that members of the *C.annuum* complex share a common gene pool as was suggested by Eshbaugh (1983), Ibiza et al. (2012) and others.

An unequivocal morphological delimitation for *C.chinense* is difficult for two reasons: first, intermediate well-established types occur between wild and domesticated forms, probably due to intraspecific hybridisation (Pickersgill et al. 1979) and second, some specimens are hardly indistinguishable from their closest relatives (*C.frutescens* and *C.annuum*) at flowering or fruiting stage (Smith and Heiser 1957; Eshbaugh 1976; Baral and Bosland 2004; Pickersgill 2016). *Capsicumchinense* is a short-lived subshrub or rarely shrub with 2–4 (–5) flowers per node, flowering pedicels that can be erect or spreading and geniculate at anthesis (mostly in wild forms) or pendent and non-geniculate (mostly in domesticated cultivars), a calyx with strongly-marked main veins that lacks appendages or is pentagonal or hexagonal in outline with 5–6 mucro-like appendages, a corolla that is dull white or greenish-white, fruits that are variable in shape, size and colour, deciduous or persistent, a fruiting calyx with a strong annular constriction at the junction with the pedicel and a clearly discoid fruiting calyx. The discoid calyx with a distinct annular constriction is the most conspicuous and consistent feature of *C.chinense* (Fig. 47).

Morphologically, Baral and Bosland (2004) found *C.chinense* produces primarily pendent flowers and calyces with a circular constriction, while *C.frutescens* produces erect flowers and calyces without a constriction. Another character found in literature to distinguish these species is the degree to which the style is exserted beyond the anthers (D’Arcy 1973; Eshbaugh 2012). It is most difficult to distinguish these two species at the flowering stage. The great number of specimens of *C.chinense*, analysed in this treatment, showed that the position of the flowering pedicels and whether or not the style is exserted more than 1 mm beyond the anthers can be quite variable and prevents confident species assignment (Peña-Yam et al. 2009). Calyx characters can be helpful in the identification of flowering specimens. The flowering calyx in both *C.chinense* and *C.frutescens* is deeply cup-shaped, but in *C.chinense*, the main nerves protrude remarkably from the calyx surface (Fig. 47C, J, L), while in *C.frutescens*, they are often completely immersed in the calyx surface (Fig. 68C, G) or may protrude distally. In both species, these nerves may slightly exceed (less than 1 mm) the calyx edge. In fruit, these species differ clearly. *Capsicumchinense* has erect and uniformly widened pedicels (wild/domesticated forms, Fig. 47F–H) or pendent pedicels with one or two swellings distally (domesticated/cultivated forms, Fig. 47L–K), but in both cases, a noticeable circular constriction at the junction with the fruiting calyx is present. In addition, the calyx flattens completely (discoid calyx) and remains appressed or reflexed to the fruit base (Fig. 47H, M). In contrast, in *C.frutescens*, the pedicels are usually erect and widen in a similar way to the wild form of *C.chinense*, but the circular constriction is totally absent and the fruiting calyx remains deeply cup-shaped, housing the narrowed base of the elongate fruit (Fig. 68G, H).

The differentiation of *C.chinense* from *C.annuum* (wild or domesticated specimens) is sometimes also difficult, especially at the fruiting stage. Wild forms of *C.chinense* with small red fruits and erect pedicels could be confused with some domesticated (or semi-domesticated) specimens of C.annuumvar.glabriusculum, which share the same pedicel position and fruit characteristics (size, shape and colour), although wild forms of this variety have smaller fruits than the typical *C.chinense*. The presence/absence of the constriction in the pedicel/calyx junction (lacking in C.annuumvar.glabriusculum) allows the assignment to one or other taxon. Furthermore, *C.chinense* is a more robust plant with larger leaves and flowers than C.annuumvar.glabriusculum.

During its long history of cultivation, *C.chinense* fruits have developed a broad range of variation due to active selection by growers, differing cultivation methods and adaptation to the environment (Barbosa et al. 2010); in addition, there may have been historical and geographical isolation of subpopulations (Bharath et al. 2013). This variation has been observed along its distribution (Bharath et al. 2013; Baba et al. 2016; Moreira et al. 2018), where elongate, campanulate and blocky fruit shapes are more predominant than triangular or rounded shapes. Similarly, red fruits, followed by orange or yellow ones, are the most common, with rare occurrences of white, orange-yellow, black or brown fruits.

The pungency and aroma of this species are also remarkable. The “Carolina Reaper” cultivar is reputed to be the world’s hottest chile, which rates at an average of 1,641,183 Scoville Heat Units (SHU) (https://www.guinnessworldrecords.com/world-records/hottest-chili, accessed on 29 October 2019) and is a favourite amongst hot-pepper lovers. At the other extreme, the fruits of the low pungency varieties (pimenta-doce, pimenta-de-cheiro longa, pimenta-de-cheiro amarela) found in Brazil (Roraima) are used by indigenous and non-indigenous communities and are preferentially used in stews or salads, for preparing jellies or liqueurs or for food dish decoration (Barbosa et al. 2006). The fruits are also distinguished by their distinct volatile profiles (depending on the cultivars), which give them a powerful exotic-fruity aroma (Rodríguez-Burruezo et al. 2010; Garruti et al. 2013 and references therein; Patel et al. 2016).

Baral and Bosland (2004) provided morphological, phylogenetic (RADP) and reproductive (sexual compatibility) evidence to differentiate *C.chinense* from *C.frutescens*, while a study of genetic diversity (SSR and AFLP) (Ibiza et al. 2012) has shown that, although *C.chinense* and *C.frutescens* are closely related, the molecular characterisation obtained is sufficient to differentiate the individual members of these species. Intermediate phenotypes and intermediate genetic accessions were explained by hybridisation, since gene flow could be occurring in sympatric areas of these two species (Baral and Bosland 2004; Ibiza et al. 2012).

Jacquin (1776) described *C.chinense*, based on cultivated material. In the protologue, he stated that he saw this species cultivated on the Island of Martinique (Lesser Antilles), a small island in the Caribbean Sea, but he called it *C.chinense*, alluding to the region to which he thought the plant was native. We found probable original material only at W (W-acc. # 0080115). This sheet has two labels, one smaller at the lower left corner with the script “Capsicum sinense”, probably in Jacquin’s filius writing (see D’Arcy 1970) and another at the lower right corner where “Capsicum sinense” and “Hort. bot. Vind.” are handwritten by an unknown hand; this sheet is also annotated “Hb. Jacq.” in a different hand and clearer ink. We designate this sheet (W-acc. # 0080115) as the lectotype for this species.

*Capsicumcerasiforme* was published by Lamarck (1794) with only the type locality “E Brafilia” cited. At P, we found a collection in Lamarck’s Herbarium with a label indicating that it belongs to “*Capsicumcerasiforme*” (P00357733) which we designate as the lectotype.

For *C.luteum*, Lamarck (1794) cited in the protologue “Ex India. Sonnerat” and the vernacular names ‘piment jaune’ and ‘Le piment de Mozambique’. At P, we found a sheet in Lamarck’s Herbarium with original material and the same data (P00357731). The label has a description and pencil drawings that likely were used in preparing the description. We select this sheet as the lectotype.

There are three sheets of original material labelled *C.cerasiforme* in Willdenow’s Herbarium held at Berlin. All of them contain reproductive branches; one of these (B-W04427-01-0) consists of a fruiting branch that matches Willdenow’s description exactly (Willdenow 1809) and is, therefore, designated the lectotype.

In describing *C.toxicarium*, Poeppig (1832) cited no herbarium material. Poeppig’s Herbarium is housed at W (Stafleu and Cowan 1983) where two specimens from Peru with the handwritten name *C.toxicarium* are held. On one of the specimens is a note referring to the poisonous effect of this plant known as “aji de veneno”, a vernacular name cited in the protologue. This sheet consists of four fruiting branches and a fifth sterile branch (acc. # 0102196). The second sheet is a poorer specimen (one branch with only one fruit, acc. # 0102195) and has a different collection date from the other sheet. We designate W acc. # 0102196 as the neotype of *C.toxicarium*.

Bertoloni (1838) described *C.cereolum*, based on material cultivated in the Botanical Garden of Bologna (Italy), from Brazilian seeds. We could find no specimens, but Bertoloni provided a colour plate for *C.cereolum* that consists of a flowering and a fruiting branch; both description and plate match with our concept of *C.chinense* and we designate this illustration as the lectotype.

Paxton (1838) described *C.ustulatum*, based on cultivated plants grown at Chatsworth (England) obtained from seeds of unknown origin, sent by J. Bateman, Esq., of Knypersly, under the name “True Chili Capsicum”. Paxton is not known to have made herbarium material (Stafleu and Cowan 1983), but he provided a plate opposite page 197 in Paxton (1838) labelled only as “Capsicum” although clearly associated with the protologue; this plate is here designated as the lectotype.

In the protologue of C.cerasiformevar.maurocarpum, Dunal (1852) cited no specimens, but referred this variety to a pre-Linnaean colour figure published by Weinemann (1745), which is here selected as the lectotype. Fruit characters depicted on the plate leave no doubt that this variety corresponds to *C.chinense*.

Dunal (1852) based C.cordiformevar.subsulcatum on Fig. IX c (as *C.cordiforme*) published by Fingerhuth (1832); as Dunal did not mention any specimens, we designate Fingerhuth’s figure as the lectotype.

The original material of *C.assamicum* could not be found and is apparently lost. According to the protologue (Purkayastha et al. 2012: 56) “Voucher specimens of the taxon are lodged at the Herbarium of Defence Research Laboratory (DRDO), Tezpur, Assam (DRLT, unregistered herbarium acronym), India and was also sent to the Herbarium of Botanical Survey of India, Eastern Circle, Shillong (BSI), India”. The authors were wrong when they assigned the acronym BSI to the Eastern Circle, Shillong; it is should be ASSAM. BSI stands for Botanical Survey of India, Western Circle, Pune, India (Thiers 2021). An annotation in The International Plant Name Index (https://www.ipni.org/n/60460283-2, accessed on 13 December 2019) “Purkayastha (pers. comm.) admitted that the citation of BSI was an error for CAL”. After consulting the respective CAL, BSI and ASSAM curators (Barboza *in litt*.), neither holotype nor isotypes have been located in these Herbaria.

#### Specimens examined.

See Suppl. material 4: Appendix 4.

### ﻿12. Capsicum coccineum

Taxon classificationPlantaeSolanalesSolanaceae

(Rusby) Hunz., Huitième Congr. Int. Bot. Paris. Comptes Rend. Séances Rapp. & Commun. 1954, sect.4: 73. 1956.

95AF79C2-1599-5F61-B2A7-E6E849C3EF3E

Figs 49 , 50

Brachistus coccineus Rusby, Bull. New York Bot. Gard. 8(28): 117. 1912. Type. Bolivia. La Paz: [Prov. A. Iturralde]. San Buena Ventura, 1400 ft, 18 Nov 1901, *R.S. Williams 634* (lectotype, designated by Barboza 2011, pg. 25: NY [00138552]; isolectotypes: BM [BM000884131], CORD [CORD00006961 fragment ex NY], K [K000648541]).
Lycianthes coccinea (Rusby) Rusby, Bull. Torrey Bot. Club 53: 210. 1926. Type. Based on Brachistuscoccineus Rusby

#### Type.

Based on *Brachistuscoccineus* Rusby.

#### Description.

Sprawling vines or scrambling shrubs, (1–) 1.5–7 m tall, with the main stem thick, 2–3 cm in diameter at base, few to much branched, the branches scandent. Young stems strongly angled, fragile, brown or green, glabrous, glabrescent or moderately pubescent, with antrorse, curved, simple, uniseriate, 4–6-celled, eglandular trichomes 0.3–0.9 (–1.2) mm long; nodes solid, green; bark of older stems green or brown with lighter stripes, glabrescent; lenticels absent. Sympodial units difoliate, the leaves geminate, rarely solitary; leaf pair unequal in size, similar in shape. Leaves membranous, concolorous, sometimes brilliant, glabrous to moderately pubescent on both sides and especially along the main veins abaxially, with simple trichomes like those of the stems, sometimes trichomes in tufts in the vein axils beneath; blades of major leaves 4.7–13 (–15) cm long, (1.7–) 2.1–5 cm wide, ovate to elliptic, the major veins 5–7 on each side of mid-vein, prominent abaxially, the base attenuate or short-attenuate and barely unequal, the margins entire, the apex acuminate; petioles 1–1.8 cm long, moderately pubescent; blades of minor leaves 3.2–4.8 cm long, 1.4–1.6 cm wide, elliptic or ovate, the major veins 3–4 on each side of mid-vein, the base short-attenuate, the margins entire, the apex acute; petioles 0.3–0.4 cm long, moderately pubescent. Inflorescences axillary, 4–13 (–18)-flowers on a short rachis 1.5–3 mm long; flowering pedicels 5–15 mm long, angled, erect, geniculate at anthesis, green, glabrescent to moderately pubescent, the eglandular trichomes short, antrorse; pedicel scars prominent, corky. Buds ovoid, colour unknown. Flowers 5-merous. Calyx 1–2.2 mm long, ca. 2 mm wide, cup-shaped, thick, slightly 5-nerved, green, sparsely pubescent with the same eglandular trichomes as the stems, calyx appendages absent or 2–10 unequal, the five longer appendages, 0.8–1.3 (–2) mm long, the shorter appendages 0.2–0.5 mm, filiform, erect, sparsely pubescent with the same trichomes as the calyx tube. Corolla (5–) 6.5–8 mm long, ca. 7–8 mm in diameter, yellow outside, usually yellow with yellowish-green or purple-brown spots within, stellate with interpetalar membrane, lobed nearly halfway to the base, pubescent adaxially with abundant long glandular trichomes (stalk 2–4-celled; head peltate, unicellular) in the throat and the lobes, the tube 2.5–3.5 mm, glabrous abaxially, the lobes 2.3–3.8 mm long, 2.4–3.4 mm wide, broadly triangular or ovate, the margins involute, sparsely pubescent (2–3-celled eglandular trichomes) abaxially, the tips cucullate and papillate. Stamens five, equal; filaments 0.9–1.9 mm long, purple, lilac or greyish, inserted on the corolla 1.2–1.4 mm from the base, with auricles fused to the corolla at the point of insertion; anthers 1.3–2 mm long, ellipsoid, dull yellow or light brown, not connivent at anthesis. Gynoecium with ovary ca. 1.5 mm long, 1 mm in diameter, light green, ovoid; ovules more than 2 per locule; nectary ca. 0.3 mm tall; styles homomorphic, 3–4 mm, barely exserted beyond the anthers, purple or white, clavate; stigma ca. 0.2 mm long, 0.6 mm wide, discoid or globose, light green. Berry (6–) 7–9 mm in diameter, globose, green to brown when immature, turning to orange, glossy red or reddish-orange at maturity, only 2–4 fruits per inflorescence, deciduous, pungent, pericarp thick, opaque, with giant cells (endocarp alveolate); stone cells 0–4; fruiting pedicels 10–18 mm long, erect, faintly angled, widened distally, green; the fruiting calyx 4–5.5 mm in diameter, persistent, not accrescent, discoid, rotate or reflexed, green, margin entire or sometimes ripped, the appendages up to 2.2 mm long, spreading or reflexed. Seeds (2–) 4–9 (–13) per fruit, 4–4.6 mm long, (2.8–) 3.2–3.75 mm wide, C-shaped or teardrop-shaped, yellow to brownish-yellow, the seed coat faintly reticulate (SM), reticulate-cerebelloid (SEM), the cells irregular and elongate near hilum, the lateral walls sinuate to straight at seed margin; embryo annular or imbricate.

#### Distribution.

*Capsicumcoccineum* grows from eastern Peru (Ayacucho, Cuzco, Huánuco, Junín, Loreto, Madre de Dios, San Martín and Ucayali Departments) to north-central Bolivia (Beni, Cochabamba, La Paz, Pando and Santa Cruz Departments) and western Brazil (Acre, Amazonas and Rondônia States) (Fig. 51).

#### Ecology.

*Capsicumcoccineum* is a common species of the Amazon Basin lowlands, found in tropical evergreen moist forests or in deciduous subtropical forests along the eastern Andes. It grows on scrubby banks of roads through disturbed forests, along river edges and streams or in flooded areas, usually in the sun, between 150 and 800 m elevation.

#### Phenology.

Flowering mainly from September to March; fruiting all year.

#### Chromosome number.

Not known.

#### Common names.

**Bolivia**: Pimienta (Santa Cruz, *Steinbach 5373*), Ají del monte (Beni, *Killen et al. 3415*; Cochabamba, *Thomas & Berdeja 1428*; Sud Yungas, *Seidel & Vaquiata 7689*); **Brazil**: Pimentinha (Acre, *Daly 9979*); **Peru**: Ají silvestre (Loreto, *Mc Daniel 14075*; Huánuco, *Woytkowski 7516*), Ajicillo (Huánuco, *Schunke V. 10620*), Ajisillo (San Martín, *Schunke V. 3415*), Charapilla (Huánuco, *Schunke V. 9984*), Charapillo (Huánuco, *Schunke V. 12469*), Chintillo (San Martín, *Plowman & Schunke V. 11523*), Sacha Ají (Huánuco, *Schunke V. 9984*), Sacha charapillo (Ucayali, *Schunke & Graham 16612*), Ají silvestre liana (Loreto, *Mc Daniel & Santiago 2556*).

#### Indigenous names.

**Bolivia**: Meñu winno (Yuracaré, Cochabamba, *Thomas & Berdeja 1428*), Hpochetgi (Trinitario, Cochabamba, *Thomas & Berdeja 1428*); Tá yejti (Mosetén, La Paz, *Vargas et al. 1310*), Neshita’mo (Beni, *Guareco 457*).

#### Uses.

The fruits are used as condiments in Bolivia (*Steinbach 5373*, *Rivero 218*), but on a lesser scale than other wild species (‘ulupica’ or ‘aribibi’). In Peru, fruits are used to prepare curare (*Woytkowski 7516*). The entire plant is used as a medicine (see Table 3).

#### Preliminary conservation assessment.

EOO (987,169.745 km^2^); AOO (336 km^2^). *Capsicumcoccineum* occupies a large range along the lowlands of the Amazon Basin in well preserved areas, as well as in fragmented forests. The species inhabits in reduced subpopulations along the main effluents of the Amazon drainage basin (Rivers Beni, Ucayali, Purús, Huallaga, Apurimac, Madre de Dios, Río Acre, Surutú and others) and in many conservation units where is expected that it is not in a serious risk of threat. However, due to the continuing decline of the quality of the habitat outside of natural reserves, we assign *C.coccineum* the Near Threatened (NT) category.

#### Discussion.

*Capsicumcoccineum* has been placed in the Bolivian clade, although such placement is only moderately supported (Carrizo García et al. 2016; Barboza et al. 2019). The relationships of *C.coccineum* should be analysed in depth, especially with regard to its phenotypic variability (see below) and wide geographic distribution. This shrubby species is the only one in the genus with a notable scrambling vine habit; its very fragile branches are supported on the trunks of other trees or shrubs. This habit, along with the inflorescence, with nearly 18 flowers on a short rachis, the short, strongly ridged pedicels, the corky scars of the pedicels, the small corollas and the reflexed fruiting calyx, are the most diagnostic characters for this species.

Two morphs of *C.coccineum* can be distinguished, based on the number of calyx appendages. The first morph, which includes the type collection, has a calyx with 10 unequal appendages or 2–10 appendages; the second morph has a calyx lacking appendages. Populations from Bolivia generally have calyx appendages, while populations from Peru and Brazil exhibit both morphs. Further fieldwork throughout the range of *C.coccineum* is needed to elucidate if the variability observed in the calyx morphology is an intraspecific variation or if a cryptic species is involved. Similarly, corolla colour is also variable in this species and deserves more attention in the field. Some collector labels mention a uniform corolla colour (yellow, cream, brilliant greenish-yellow), while others indicate dull yellow corollas with yellowish-green spots within or with purple-brown or violet centre. Variations in corolla colour and degree of development of the calyx appendages are observed in other species from the Brazilian Atlantic Forest, for example, *C.schottianum*.

In Bolivia, *C.coccineum* is sympatric with *C.minutiflorum* and C.baccatumvar.baccatum. All three of these entities share red globose pungent fruits on erect pedicels. *Capsicumcoccineum* differs from *C.minutiflorum* in its scrambling vine habit vs. erect shrubby habit, multi-flowered inflorescence (up to 18 flowers) vs. few-flowered inflorescence (up to five flowers) and calyx appendages absent or 2–10 vs. usually five appendages. Capsicumbaccatumvar.baccatum differs from *C.coccineum* in the same set of contrasting characters and in its corolla colour that is white (vs. yellow or cream) and the dimorphic styles (vs. homomorphic styles). The fruits of C.baccatumvar.baccatum can also be ellipsoid.

**Figure 49.**
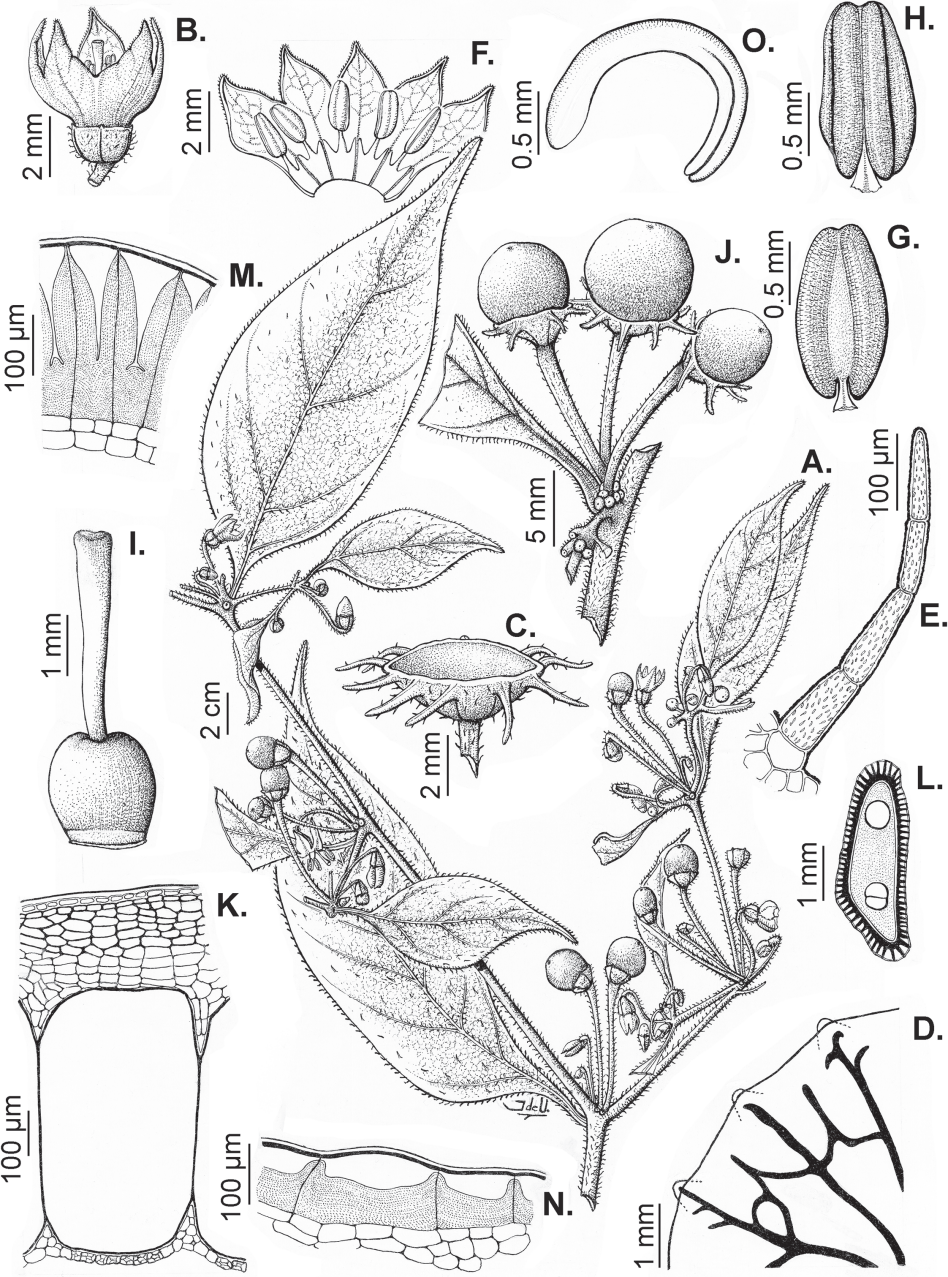
*Capsicumcoccineum***A** reproductive branch **B** flower **C** calyx with appendages **D** section of the calyx showing the venation **E** eglandular trichome of the calyx **F** opened corolla **G, H** anthers, dorsal and ventral views, respectively **I** gynoecium **J** node with fruits **K** anatomical detail of the pericarp (note the giant cell in the mesocarp) **L** seed, in cross section **M** structure of seed coat at the seed margin **N** structure of seed coat at the seed body **O** embryo **A, B, D–I, K** from *Woytkowski 1176***C, J** from *Nee & Saldías 35956***L–O** from *Nee 44503.* Drawn by J. de Ugarte.

**Figure 50.**
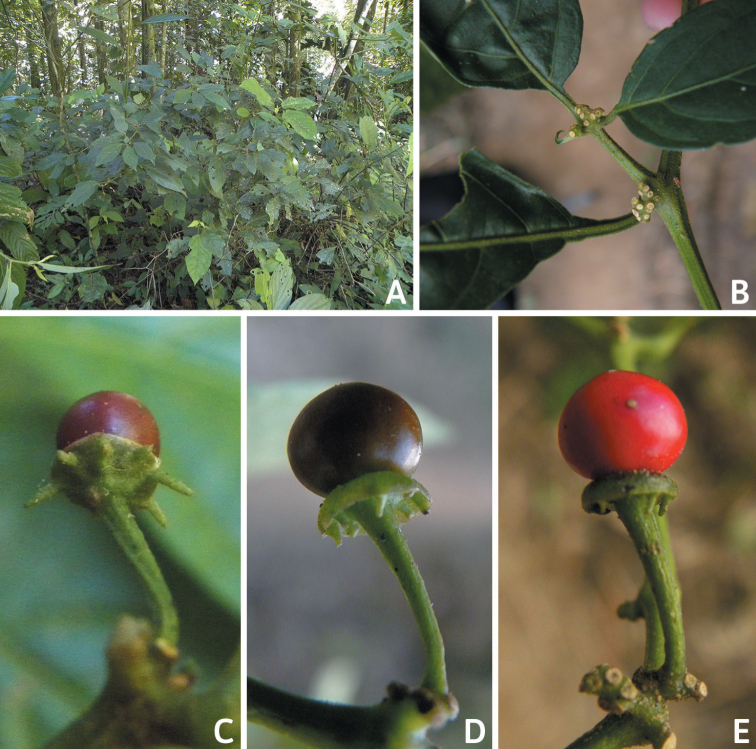
*Capsicumcoccineum***A** plant **B** fruiting node with many pedicels scars **C** young immature fruit **D** immature fruit with calyx reflexed **E** mature fruit with calyx reflexed. From *Barboza et al. 4921.* Photos by G.E. Barboza.

#### Specimens examined.

See Suppl. material 4: Appendix 4.

### ﻿13. Capsicum cornutum

Taxon classificationPlantaeSolanalesSolanaceae

(Hiern) Hunz., Huitième Congr. Int. Bot. Paris. Comptes Rend. Séances Rapp. & Commun. 1954, sect.4: 73. 1956.

4E24F7C6-B18E-5A26-9BAB-D31EF19F0F9E

Figs 52 , 53

Bassovia cornuta Hiern, Vidensk. Meddel. Naturhist. Foren. Kjøbenhavn: 59. 1877. Type. Brazil. Rio de Janeiro: Rio de Janeiro, [no date], A.A.W. Lund s.n. (lectotype, designated here: C [C10019145]; isolectotypes: [K000585893], P [P00410056]).
Capsicum dusenii Bitter, Abh. Naturwiss. Vereine Bremen 24: 520. 1919. Type. Brazil. São Paulo: Serra do Mar, Alto da Serra, 30 Sep 1912, *P.K.H. Dusén 14227* (lectotype, designated here: S [acc. # S04-2813]; isolectotypes: CORD [CORD00006948 fragment ex S], RB [RB01413389], S [acc. # S18-42558]).

#### Type.

Based on *Bassoviacornuta* Hiern.

#### Description.

Erect shrubs or subshrubs, (0.80–) 1–3.5 m tall, with the main stem thick, ca. 2.5 cm in diameter at base, much branched above, the branches dichotomously spreading in a typical “zig-zag” appearance. Young stems angled, fragile, green or brown, densely pubescent, with spreading, more or less rigid, brilliant and ferruginous, simple, uniseriate, (3–) 5–9-celled, eglandular trichomes 0.5–3 mm long, rarely glabrescent; nodes solid, green; bark of older stems dark brown, sparsely pubescent; lenticels absent. Sympodial units difoliate, the leaves geminate; leaf pair unequal in size, similar in shape. Leaves membranous, slightly discolorous, green above, light green beneath, densely pubescent adaxially with appressed-antrorse eglandular trichomes similar to those of the stems and abundant spreading, uniseriate, 5–8-celled, eglandular trichomes abaxially, especially on the veins and margins; blades of major leaves 6–16 (–18) cm long, 4–8 cm wide, ovate or widely elliptic, the major veins 6–8 on each side of mid-vein, the base attenuate, the margins entire, the apex acuminate; petioles 0.3–0.5 (–0.8) cm long, densely pubescent with spreading eglandular trichomes; blades of minor leaves (3–) 4–6 cm long, 1.5–3.2 cm wide, elliptic or ovate, the major veins 3–5 on each side of mid-vein, the base attenuate, the margins entire, the apex acute; petioles 0.2–0.4 cm long, same pubescence as the major leaves . Inflorescences axillary, 2–5 (–7) flowers per axil or flowers solitary; flowering pedicels (22–) 25–35 mm long, delicate, angled, erect, geniculate at anthesis, green or green with purple lines, moderately pubescent, the eglandular trichomes long, spreading; pedicels scars inconspicuous. Buds ovoid, inflated, white with green and purple spots. Flowers 5-merous. Calyx 1–2 mm long, 4–5 mm wide, cup-shaped, thin, green, densely pubescent with spreading eglandular trichomes, the calyx appendages (5–) 7–10 unequal, the five main appendages 2.5–5 (–6), the five secondary appendages shorter 0.5–1.5 (–2) mm long, thin, erect or spreading, linear or subulate, inserted very close to the margin. Corolla (8–) 9–14 mm long, 18–22 mm in diameter, white with purple and yellowish-green spots outside, white with intense small purple or reddish-brown spots amongst the veins and in the throat and yellowish-cream centre within, stellate with thin interpetalar membrane, lobed halfway or less of the way to the base, the tube 4–5 mm long, pubescent adaxially with a continuous ring of glandular trichomes (stalk long, 2–3-celled; head globose, peltate, unicellular), glabrous abaxially, the lobes 3.5–6.8 mm long, 3–5.5 mm wide, broadly triangular, spreading, glabrous adaxially and with eglandular trichomes 3–6-celled on the veins abaxially, the margins papillate, the tips acute, cucullate, papillate. Stamens five, equal; filaments 1.4–2.2 (–2.5) mm long, white or cream, inserted on the corolla ca. 1.75 mm from the base, with auricles fused to the corolla at the point of insertion; anthers 1.2–2.3 mm long, ellipsoid, pale grey or grey, not connivent at anthesis. Gynoecium with ovary ca. 2 mm in diameter, light green, globose; ovules more than two per locule; nectary ca. 0.3 mm tall; styles homomorphic, 4–6.8 mm long, exserted ca. 1.3 mm beyond the anthers, white or cream, clavate; stigma 0.3 mm long, 0.6–0.8 mm wide, globose or discoid, pale green. Berry 7–10 mm in diameter, globose, green when immature, greenish-golden yellow at maturity, deciduous, slightly pungent, the pericarp thin, translucent, with giant cells (endocarp alveolate); stone cells absent; fruiting pedicels (25–) 30–38 mm long, pendent and curved, angled, widened distally, green; the fruiting calyx 4–5 mm in diameter, persistent, not accrescent, discoid, green, the appendages 1–6 mm long, spreading. Seeds (4–) 6–18 (–20) per fruit, 3–3.5 mm long, 2.5–3.5 mm wide, teardrop-shaped, black, the seed coat reticulate and tuberculate at margins (SM), reticulate with pillar-like outgrowths at margins (SEM), the cells polygonal in shape, the lateral lateral walls straight; embryo imbricate.

#### Distribution.

**Figure 51.**
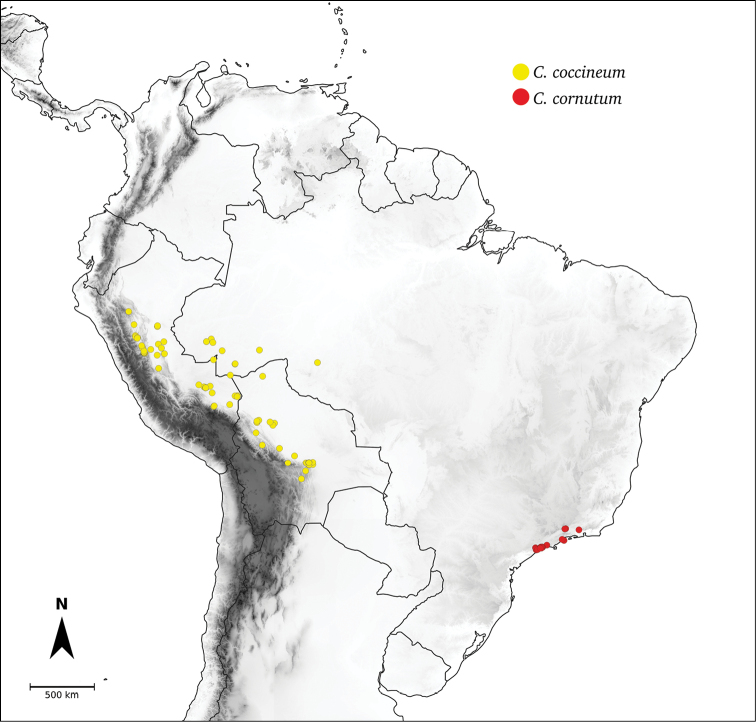
Distribution of *C.coccineum* and *C.cornutum*.

*Capsicumcornutum* is endemic to south-eastern Brazil, confined to small areas in São Paulo and Rio de Janeiro States (Fig. 51).

#### Ecology.

*Capsicumcornutum* occurs in the Atlantic Forest (Mata Atlântica), in the Dense Ombrophilous Forest (Floresta Ombrófila Densa), in semi-shade, on the edge of steep, open ravines or along forest roadsides, between 500 and 900 m elevation.

#### Phenology.

Flowering from September to May. Fruiting from December to May and June.

#### Chromosome number.

*n* = 13 (Pozzobon and Schifino-Wittmann 2006); 2*n* = 2x = 26 (Pozzobon et al. 2016).

#### Common name.

**Brazil**. Pimentinha-do-mato (Rio de Janeiro, *Bovini & Giordano 363*).

#### Uses.

None recorded.

#### Preliminary conservation assessment.

EOO (17,682.480 km^2^); AOO (72 km^2^). *Capsicumcornutum* occurs exclusively in the Brazilian Atlantic Forest, one of the world’s biological diversity hotspots that is increasingly threatened by the rapid destruction (deforestation) and fragmentation of its natural areas. This species inhabits the Serra do Mar, in much reduced subpopulations mainly in officially protected areas, such as Parque Nacional do Itatiaia and APA-Cairuçu (Rio de Janeiro), Parque Estadual da Serra do Mar and Reserva Biológica do Alto da Serra de Paranapiacaba (São Paulo). Based on the EOO, the continuing decline observed in the quality of its habitat outside of the natural reserves and decline in number of locations, we assign the threat status of Vulnerable (VU; B1ab(iii,iv)).

#### Discussion.

*Capsicumcornutum* is a member of the Atlantic Forest clade (Carrizo García et al. 2016). It is rarely collected, but easily recognised by its dense pubescence, usually 7–10 unequal calyx appendages and white, stellate corollas with purple and yellowish-green spots abaxially and intense small purple or brownish-red spots adaxially (Table 4). Some specimens from the State of Rio de Janeiro (Paraty) lack the typical dense pubescence.

*Capsicumvillosum* is superficially similar to *C.cornutum* in its pubescence, geniculate pedicels and corolla and fruit colour, but differs in having five subequal calyx appendages, a smaller corolla (7–9 mm long, 14–14.5 mm in diameter vs. 8–14 mm long, 18–22 mm in diameter in *C.cornutum*), a different design of purple pigmentation in the corolla lobes and throat (two large spots at the base of each lobe forming a more or less ring-like purple centre within the corolla vs. many small purple or reddish-brown spots amongst the veins in *C.cornutum*) and filaments 1.6–2.4 longer than the anthers (vs. filaments somewhat longer than the anthers in *C.cornutum*).

*Capsicumcornutum* has also been confused in herbaria with another species of the Atlantic Forest clade, *C.recurvatum*. *Capsicumcornutum* differs in having dense pubescence of long spreading trichomes, longer and erect or spreading calyx appendages and purple pigmentation within the corolla; *C.recurvatum* is much less pubescent with shorter antrorse trichomes, has shorter and recurved calyx appendages and has a corolla with greenish-yellow spots within and no purple pigmentation. *Capsicumcornutum* is sympatric, but cannot be confused, with *C.schottianum*. The presence of calyx appendages, long trichomes and a larger corolla distinguish *C.cornutum* from *C.schottianum*.

Carrizo García et al. (2013) mentioned a population named *Capsicum* ‘cunha’ (from the area around Cunha in São Paulo, Brazil, no voucher cited) with entirely white corollas and with a combination of traits matching both *C.recurvatum* (calyx morphology) and *C.cornutum* (pubescence pattern). It is probable that *Capsicum* ‘cunha’ belongs to *C.recurvatum*, in light of the fact that they share the characters of the calyx (which are very consistent in *C.recurvatum*) and they lack pigmentation in the corolla (which occurs also in *C.recurvatum*). *Capsicumcornutum* is distinctive in the density, orientation and length of the trichomes, so it will be important to ascertian if those characters are also present in *Capsicum* ‘cunha’ or if these plants are more similar to *C.recurvatum*. Geographically, *C.recurvatum* has also been collected in the same locality of *Capsicum* ‘cunha’.

Hiern (1877) described *C.cornutum* under *Bassovia* (now a synonym of *Solanum*, cfr. Solanaceae Source, www.solanaceasource.org), but he clearly stated in the protologue “calyce parvulo cupulari truncato dentibus accesoriis 10 subulatis... antheris … lateraliter dehiscentibus ...” both unequivocal characters for *Capsicum* (Hunziker 2001). Bitter (1921: 333) doubted its position in Capsicumsect.Decameris, a section he established to place *Capsicum* species with 10-toothed calyces (Bitter 1919: 293).

When describing *B.cornuta*, Hiern (1877) did not cite specific herbarium in the protologue; we designate lectotype the best-preserved of Lund’s collections held at Copenhaven.

In his description of *C.dusenii*, Bitter (1919) described a calyx with 10 linear, unequal appendages and a white corolla with numerous violet maculations, these features being the same diagnostic traits used by Hiern (1877) for *B.cornuta*. He also cited *Dusén 8255* (State of Paraná, Brazil) as belonging to *C.dusenii*, but this specimen corresponds to *Athenaeawettsteiniana* (Witasek) I.M.C.Rodrigues & Stehmann (Hunziker and Barboza 1990, as *Aurelianawettsteiniana* (Witasek) Hunz. & Barboza). In describing *C.dusenii*, Bitter (1919) indicated that he had seen the collection *Dusén 14227*, but also that Dusén’s notes stated that his collections *599a*, *6559* and *8255* also corresponded to the same taxon; Bitter cited no herbaria in the protologue. We have found duplicates of *Dusén 14227* at S and RB. In S, the collection is mounted on two sheets. All of them are very good flowering branches. We designate S04-2813 that is annotated by Bitter as the lectotype for this name.

**Figure 52.**
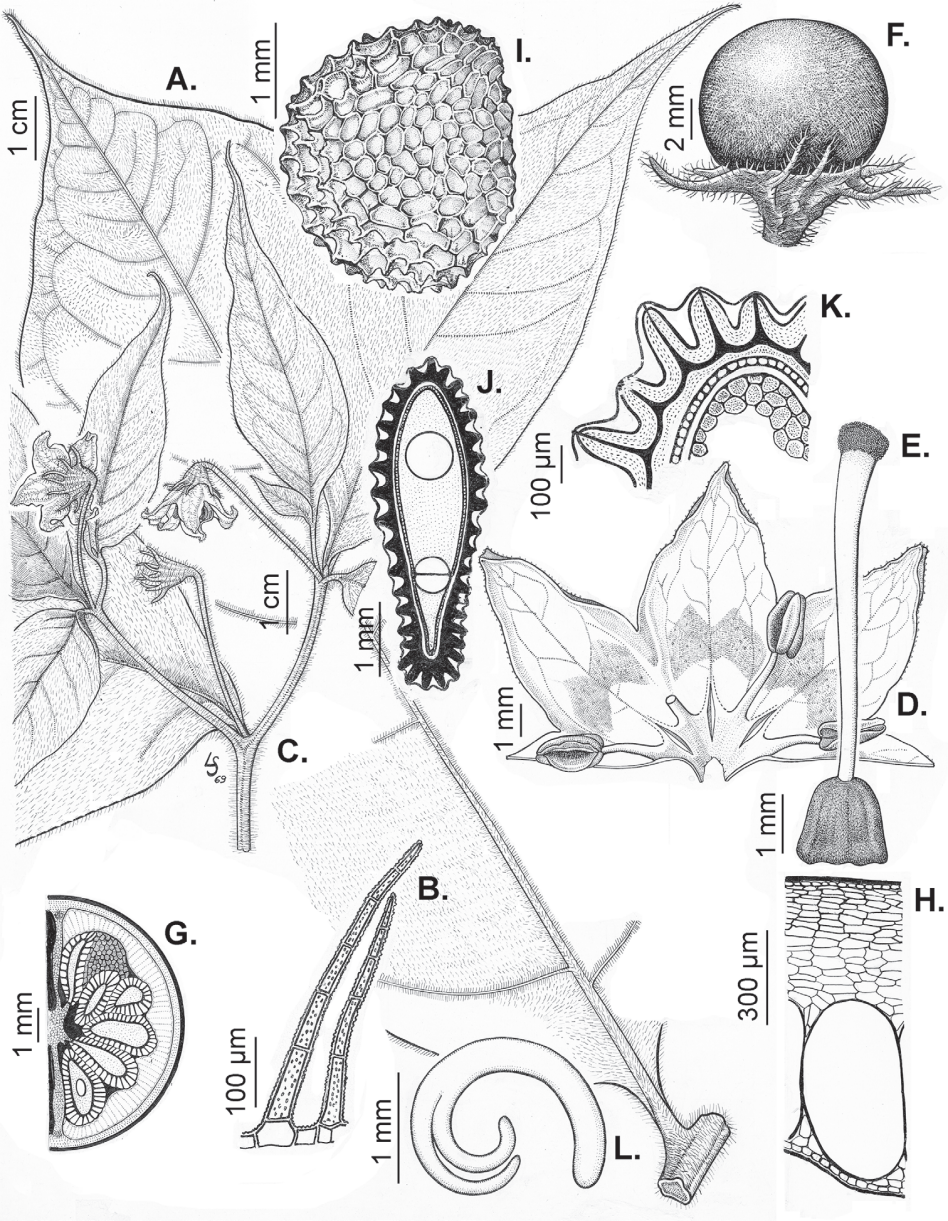
*Capsicumcornutum***A** leaf **B** eglandular trichomes of the leaf **C** flowering branch **D** sector of opened corolla **E** gynoecium **F** fruit **G** fruit (one carpel), in longitudinal section **H** anatomical detail of the pericarp (note the giant cell in the mesocarp) **I** seed **J** seed, in cross section **K** structure of seed coat at the seed margin **L** embryo **A–G** from *Hunziker* 19557 **H–L** from *Kuhlmann 4321.* Drawn by L. Sánchez. Modified from Hunziker (1971), reproduced with permission.

**Figure 53.**
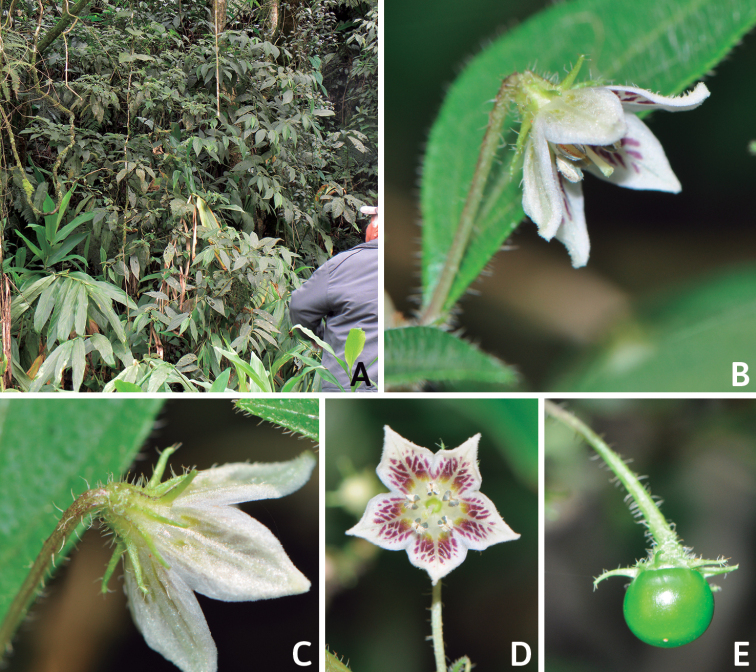
*Capsicumcornutum***A** plant **B** flower on geniculate pedicel **C** flower, in lateral view, showing the calyx **D** flower, in front view **E** immature fruit. From *Barboza & Cosa 2525*. Photos by G.E. Barboza.

#### Specimens examined.

See Suppl. material 4: Appendix 4.

### ﻿14. Capsicum dimorphum

Taxon classificationPlantaeSolanalesSolanaceae

(Miers) Kuntze, Revis. Gen. Pl. 2: 449. 1891.

FD22D704-0B40-5AC1-8536-A7689DE7FB5F

Figs 54 , 55

Brachistus dimorphus Miers, Ann. Mag. Nat. Hist., ser. 2, 3(16): 267. 1849. Type. Colombia. Quindio: Region froid en Las Tapias, Dec 1814, J. Goudot s.n. (holotype: K [K000585926]; isotypes: BM [BM000777291, pro parte only fragments at bottom right side], P [P00410061]).

#### Type.

Based on *Brachistusdimorphus* Miers.

#### Description.

Erect or scandent shrubs or subshrubs, (0.4–) 0.8–2 (–3) m tall, profusely branched above, the branches dichotomously spreading in a typical “zig-zag” appearance. Young stems terete or slightly angled, fragile, greenish-purple or maroon, densely pubescent with ochraceous or white, spreading or antrorse, flexuous or hirsute, simple, uniseriate, 2–8-celled, eglandular trichomes 0.3–1 (–1.5) mm long, sometimes glabrous or glabrescent; nodes green; bark of older stems pale brown or golden-brown; lenticels light brown, sometimes absent. Sympodial units difoliate, the leaves geminate; leaf pair markedly unequal in size and shape. Leaves membranous, rarely coriaceous, with sparse or abundant antrorse eglandular trichomes adaxially and abaxially, the trichomes spreading on the veins abaxially; blades of major leaves 4.2–12 (–17) cm long, 0.8–4 (–8) cm wide, elliptic, the major veins 4–6 on each side of mid-vein, sometimes purple-coloured abaxially, the base attenuate, asymmetric or not, the margins entire, the apex long-acuminate; petioles 0.5–1.2 cm long, moderately pubescent or glabrescent; blades of minor leaves sessile, 0.5–4 (–8) cm long, 0.4–1.7 (–5) cm wide, orbicular or ovate or rarely elliptic, the major veins 2–3 on each side of mid-vein, the base short-attenuate or rounded, asymmetric, the margins entire, the apex obtuse or rounded, with same pubescence as major leaves. Inflorescences axillary, 2–5 flowers or flowers solitary; flowering pedicels (3–) 5–14 mm, angled, green or green with purple lines, curved to pendent, non-geniculate at anthesis, glabrescent to moderately pubescent, the eglandular trichomes short or long, spreading or antrorse; pedicels scars inconspicuous. Buds ovoid, purple or yellowish. Flowers 5-merous. Calyx 1.75–2.6 mm long, 3–3.3 mm in diameter, cup-shaped, circular or pentagonal in outline, fleshy, green, greenish-purple or purple, calyx appendages absent or 1–3, as mucro-like structures, 0.5–1 mm long, spreading and laterally flattened, emerging 0–0.2 mm below the margin, glabrescent to densely pubescent with antrorse or spreading trichomes. Corolla 6–9.5 (–11) mm long, 8–12 mm in diameter, entirely yellow or yellow with dark purple or maroon spots outside and within, stellate with interpetalar membrane, lobed nearly halfway to the base, glabrous adaxially and abaxially, the tube 2–3.5 mm long, the lobes (1.75–) 2–4 mm long, 1.5–2.8 (–3) mm wide, triangular or ovate, spreading or slightly reflexed at anthesis, the margins and tips papillose. Stamens five, equal; filaments (1.5–) 2–2.5 mm long, cream or light lilac, inserted on the corolla ca. 2 mm from the base, with auricles fused to the corolla at the point of insertion; anthers 1.5–2.3 mm long, ovoid, slightly apiculate, pale purple, bluish-purple, dark yellow or cream, connivent at anthesis. Gynoecium with ovary 1.37–1.6 mm long, 1.3–1.5 mm in diameter, cream, ovoid; ovules more than two per locule; nectary ca. 0.6 mm tall; styles homomorphic, 4.5–6.7 mm long, exserted 1.3–2 mm beyond the anthers, white near the base and purple or pale lilac to the apex, clavate, slightly curved distally; stigma 0.2–0.3 mm long, 0.6 mm wide, usually discoid, green or yellowish-green. Berry 5–11 mm in diameter, globose or subglobose, bright or opaque light green when immature, bright reddish-orange or bright red at maturity, deciduous, non-pungent, the pericarp thick, opaque, lacking giant cells (endocarp smooth); stone cells absent; fruiting pedicels 6–15 mm long, usually erect, rarely curved to pendent, sometimes angled, widened distally, green or purple; fruiting calyx 4–5 (–7) mm in diameter, persistent, not accrescent, discoid, sometimes ripped at the margin, greenish-white or purple, the appendages if present, reflexed or spreading, purple. Seeds (6–) 10–36 per fruit, 1.9–2.7 mm long, 1.8–2.1 mm wide, C-shaped or teardrop-shaped, brownish-black to black, the seed coat reticulate (SM, SEM), the cells polygonal in shape, the lateral walls straight; embryo annular.

**Figure 54.**
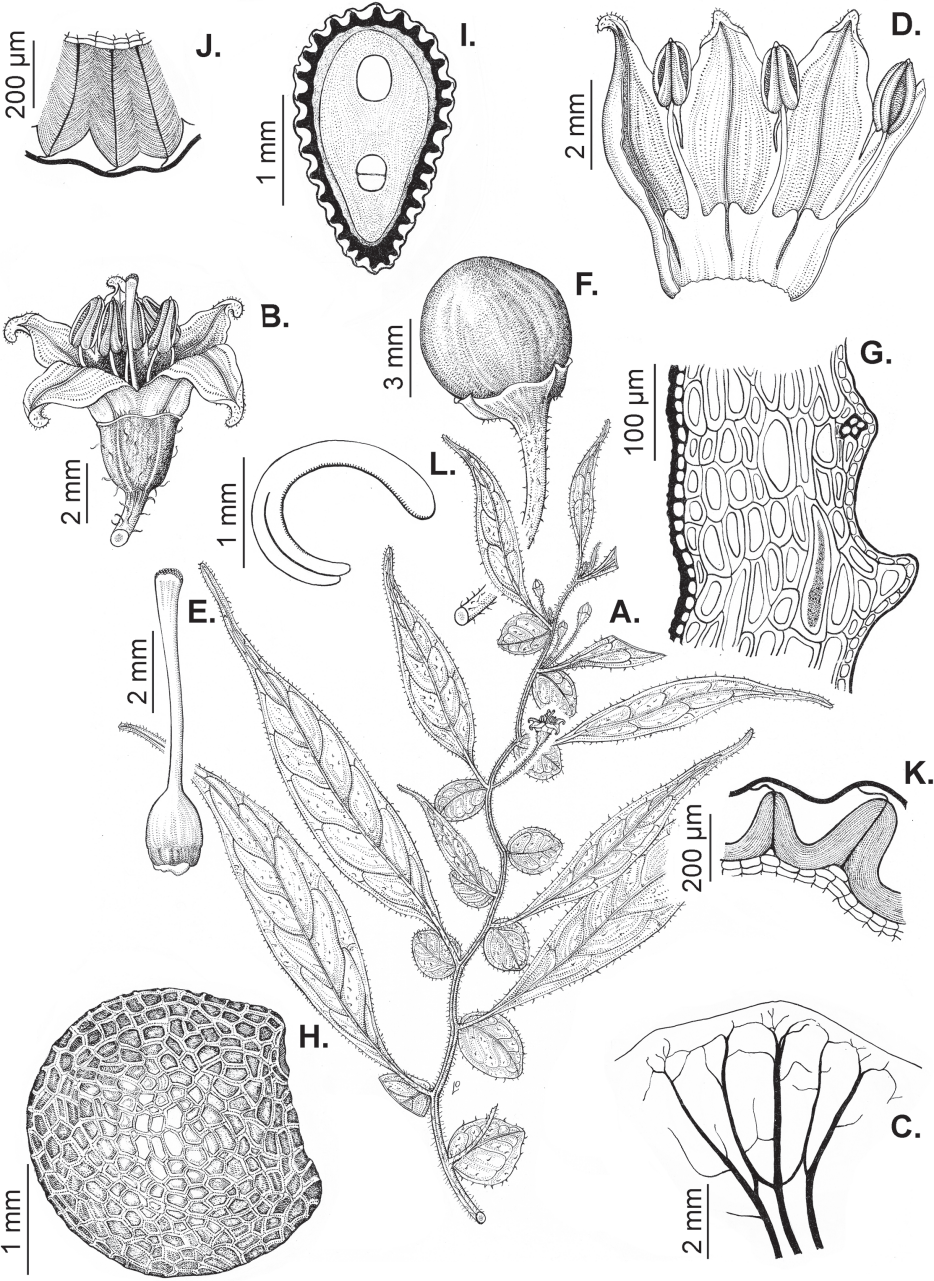
*Capsicumdimorphum***A** flowering branch **B** flower; **C** section of the calyx showing the venation; **D** sector of opened corolla; **E** gynoecium; **F** fruit **G** anatomical detail of the pericarp (note the absence of giant cells in the mesocarp) **H** seed; **I** seed, in cross section **J** structure of seed coat at the seed margin **K** structure of seed coat at the seed body **L** embryo **A, B, D, E** from *Jaramillo et al. 2698***C** from *Sneidern 3044***F–L** from *Cuatrecasas 8572*. Drawn by L. Ochoa.

**Figure 55.**
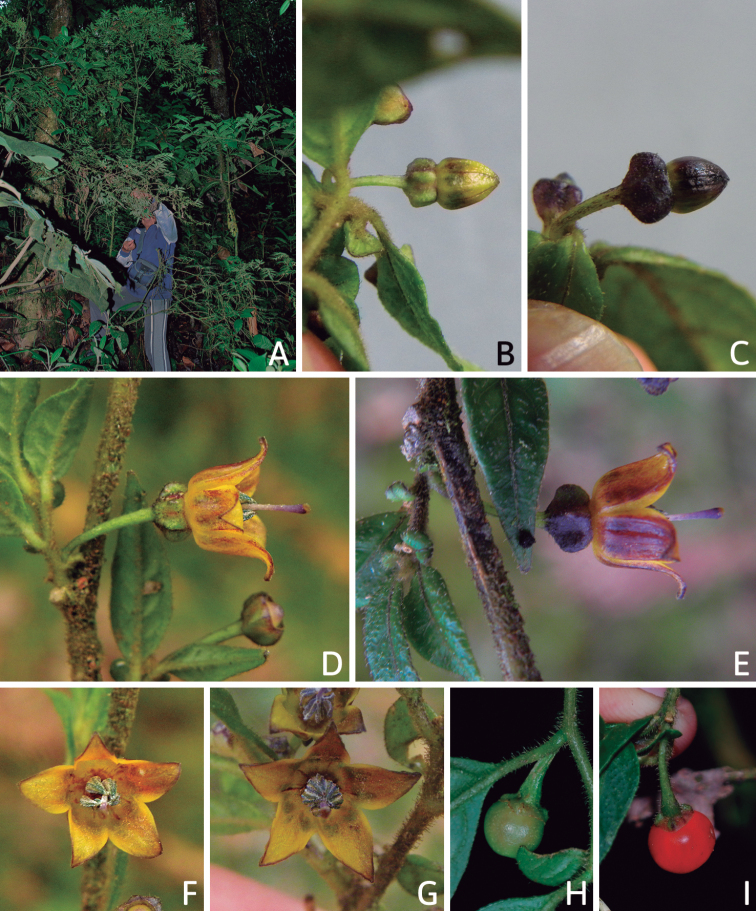
*Capsicumdimorphum***A** plant **B, C** flower buds of different colour **D, E** flowers, in lateral view, with different corolla colour outside **F, G** flowers, in front view, with different colouration patterns in the corolla within **H** immature fruit **I** mature fruit **A, H, I** from *Beltrán 140*, photos by G. Beltrán **B–G** from *Orejuela R. et al. 2685*, photos by G.E. Barboza.

#### Distribution.

**Figure 56.**
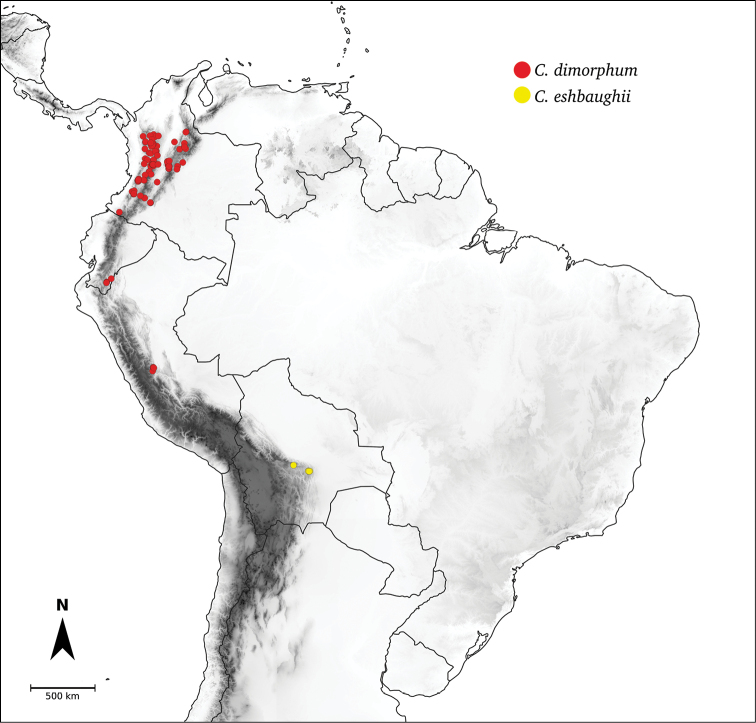
Distribution of *C.dimorphum* and *C.eshbaughii*.

*Capsicumdimorphum* is endemic to north-western South America and is most common in Colombia and Ecuador and reaching central Peru (Fig. 56).

#### Ecology.

*Capsicumdimorphum* is an Andean species of premontane or montane moist forests. It is found in the margins, understorey or interior of primary or secondary (sometimes disturbed) forests, between 950 and 3,000 m elevation.

#### Phenology.

Flowering and fruiting all year.

#### Chromosome number.

Not known.

#### Common names.

**Colombia**: Ahuyamo (Quindío, *Bernal 1828*), Mirtico de monte (Cauca, *Pittier 737*; Cundinamarca, *Duque-Jaramillo 3330*).

#### Uses.

None recorded.

#### Preliminary conservation assessment.

EOO (738,784.034 km^2^); AOO (452 km^2^). *Capsicumdimorphum* is a widespread Andean species in northern South America; considering the large EOO and its presence in officially protected areas in Colombia, Ecuador and Peru, we suggest a status of Least Concern (LC).

#### Discussion.

*Capsicumdimorphum* is a member of the Andean clade, recovered as sister to *C.longifolium* (Carrizo García et al. 2016; Barboza et al. 2019). *Capsicumdimorphum* is variable in the degree of general pubescence, leaf shape and presence or absence of purple or maroon spots on the corolla (Fig. 55B–G). It is morphologically most similar to *C.longifolium* (Barboza et al. 2019) with which it shares short pedicels and similar shape and colour of the corolla, fruit and seeds. *Capsicumdimorphum* can be distinguished by having usually pubescent vegetative organs and calyces (vs. glabrous), membranous, longer and wider major leaves, flowers that are solitary or up to five per axil (vs. 3–9 flowers on a short rachis) and a calyx without appendages or with three minute appendages (vs. 2–3 winged triangular appendages).

*Capsicumdimorphum* is sympatric with *C.geminifolium* which is distinguished by having long-acuminate leaves, 2–5 long and thin calyx appendages and campanulate corollas. Populations of *C.dimorphum* from Peru (Department Pasco) are glabrescent to glabrous plants and the leaves are somewhat coriaceous, but the flowers and fruits match those of the pubescent populations of Colombia and Ecuador.

#### Specimens examined.

See Suppl. material 4: Appendix 4.

### ﻿15. Capsicum eshbaughii

Taxon classificationPlantaeSolanalesSolanaceae

Barboza, PhytoKeys 2: 32. 2011.

C0D4EAED-E212-565D-BBF2-BFD7FF4683E7

Figs 57 , 58

Capsicum eximium Hunz. var. tomentosum Eshbaugh & P.G.Sm., Baileya 18: 15. 1971. Type. Cultivated in the Indiana University greenhouses, from seeds collected in Bolivia (Santa Cruz: Prov. Florida: 158 km W of Santa Cruz, on road to Cochabamba, 1300 m elev., *P.G. Smith C281*), 29 Nov 1960, *C.B. Heiser C301* (lectotype, designated here: IND [IND-0105969, acc. # 139720]; isolectotypes: IND [IND-0105968, acc. # 113590, IND-0153285, acc. # 139721]).

#### Type.

Based on CapsicumeximiumHunz.var.tomentosum Eshbaugh & P.G.Sm.

#### Description.

Erect shrubs or subshrubs, 1–3 m tall, with the main stem 1.5–3 (–4) cm at base, few or much branched from near the base and with fragile and tangled branches. Young stems strongly 3–4-angled, fragile, pale green, densely pubescent with white or ochraceous, brilliant, spreading, simple, glandular trichomes (stalk 3–7-celled, head unicellular, stipitate or not stipitate) 0.1–0.8 mm long or furcate trichomes with both branches ending in an unicellular head or one branch eglandular and the other longer and glandular; nodes green; bark of older stems greyish-white or pale brown, fissured, glabrescent; lenticels absent. Sympodial units difoliate, the leaves geminate; leaf pair more or less similar in size and shape. Leaves membranous, concolorous or discolorous, intense green adaxially and whitish-grey abaxially, densely pubescent on both surfaces, especially along the veins, with the same glandular trichomes like those on the stems and a few simple 3–4-celled eglandular trichomes; blades of major leaves 3.7–5.7 (–6.5) cm long, (1.6–) 2–4 cm wide, ovate, major veins 3–4 on each side of mid-vein, the base attenuate or cuneate and somewhat asymmetric, the margins entire, the apex acuminate; petioles 1.3–2.5 cm long, densely pubescent; blades of minor leaves 3–4.3 cm long, 1.4–2 cm wide, ovate, the major veins 2–3 on each side of mid-vein, the base attenuate, the margins entire, the apex acute; petioles 0.6–0.7 cm long, densely pubescent. Inflorescences axillary, 2–3 (–4) flowers per axil or more rarely flowers solitary; flowering pedicels 8–17 mm long, strongly angled, erect, geniculate at anthesis, densely glandular pubescent, the trichomes spreading; pedicel scars inconspicuous. Buds globose, white or white-purple. Flowers 5-merous. Calyx ca. 2 mm long, ca. 2 mm wide, cup-shaped, thick, green, densely glandular pubescent as stems and leaves, calyx appendages (5–) 10 (–12), 1.5–2.7 (–3) mm long, 0.1 mm wide, unequal, thin, erect, linear, inserted close to the margin, densely pubescent with the same trichomes as calyx tube. Corolla (5–) 6–7 mm long, 6–7 mm in diameter, white with greenish-yellow spots outside and within, sometimes corollas nearly white or with pale purple spots in the lobes, stellate with interpetalar membrane, lobed nearly halfway to the base, pubescent adaxially with short glandular trichomes (stalk 1–2-celled; head globose, unicellular) in the throat and base of the lobes, the tube 2–4 mm long, glabrous abaxially, the lobes 3–3.5 mm long, 3.5–4 mm wide, triangular or widely ovate, spreading, with eglandular trichomes abaxially, the margins finely ciliate, the tips acute, papillate. Stamens five, equal; filaments 2.5–3 mm long, white, inserted on the corolla 0.9–1.2 mm from the base, with auricles fused to the corolla at the point of insertion; anthers 1.5–1.7 mm long, ellipsoid, light yellow, bluish post-dehiscent, not connivent at anthesis. Gynoecium with ovary 1–1.2 mm long, ca. 1.5 mm in diameter, green, ovoid; ovules more than two per locule; nectary ca. 0.4 mm tall; styles dimorphic, 3.25–4.3 mm long, at the same level or sparsely exserted beyond the anthers, purple, clavate; stigma ca. 0.2 mm long, 0.35 mm wide, discoid, pale green. Berry (4–) 6–8 mm in diameter, globose or subglobose, dark green turning to dark brown when immature, bright red at maturity, pungent, pericarp thick, opaque, with giant cells (endocarp alveolate); stone cells absent; fruiting pedicels (10–) 15–20 mm long, erect, strongly angled, widened distally, brownish-green; the fruiting calyx 4–5 mm in diameter, persistent, not accrescent, discoid, green, the appendages 1.5–3.5 mm long, spreading. Seeds 8–20 per fruit, 3–3.5 mm long, 2.5–3.2 mm wide, C-shaped, yellow to brownish-yellow, the seed coat faintly reticulate (SM), reticulate-cerebelloid (SEM), the cells irregular in seed body, polygonal at margins, the lateral walls sinuate in seed body, straight to wavy at margins; embryo imbricate.

#### Distribution.

*Capsicumeshbaughii* is an endemic species restricted mainly to central Bolivia (Santa Cruz Department), at mid-elevation (1,300–1,800 m), with only one collection in the Department of Cochabamba, at 3,000 m (Fig. 56).

#### Ecology.

*Capsicumeshbaughii* is an uncommon species in dry deciduous and degraded marginal forests close to urbanised areas.

#### Phenology.

Flowering from November to April. Fruiting from February to April.

#### Chromosome number.

2*n* = 2x = 24 (Carrizo García et al. 2020).

#### Common name.

**Bolivia.** Ulupica (Santa Cruz, *Eshbaugh 1943*; *Nee 43483*).

#### Uses.

Fruits are used as condiments (*Nee 43483*).

#### Preliminary conservation assessment.

EOO (365.297 km^2^); AOO (24 km^2^). *Capsicumeshbaughii* has a small geographical extent and area of occupancy and is known from only 10 collections, the majority of them from Samaipata and surroundings. As far as we know, this species is only found in anthropogenically disturbed areas (Carrizo García et al. 2020) in small subpopulations, thus we assign *C.eshbaughii* the threat status of Endangered (EN; B1ab(ii,iii)). Recently, *C.eshbaughii* was found in new sites around Samaipata (dal Zovo, pers. comm. 2019).

**Figure 57.**
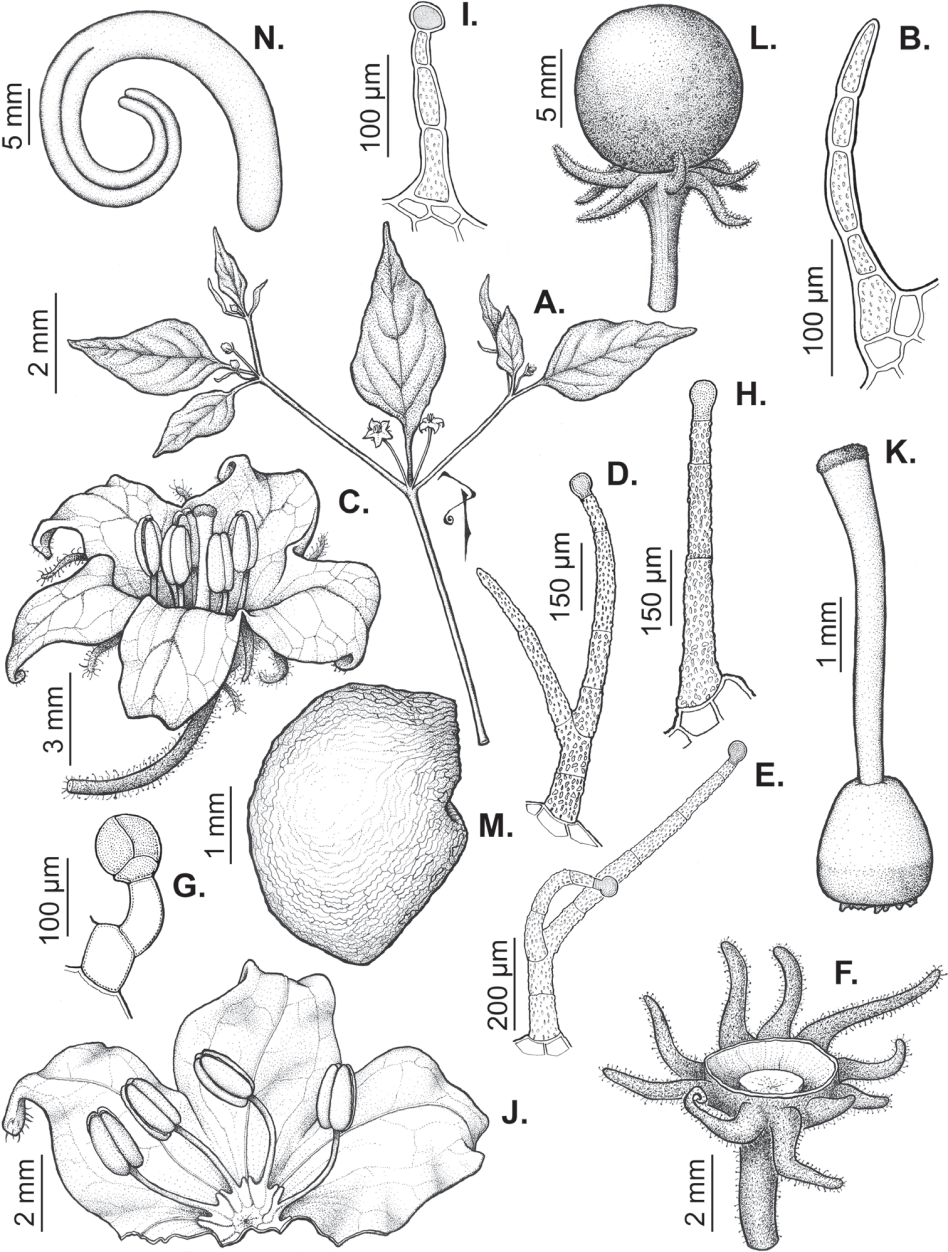
*Capsicumeshbaughii***A** flowering branch **B** eglandular trichome of the leaf **C** flower **D, E** furcate glandular trichomes of the pedicel **F** fruiting calyx **G** glandular trichome of the abaxial surface of the calyx **H, I** glandular trichomes of the adaxial surface of the calyx **J** sector of opened corolla **K** gynoecium **L** fruit **M** seed **N** embryo **A–E, G–K** from *Eshbaugh 1943 b***F, L–N** from *Nee 36164.* Drawn by P. Peralta. Published in Barboza (2011), reproduced with permission.

#### Discussion.

**Figure 58.**
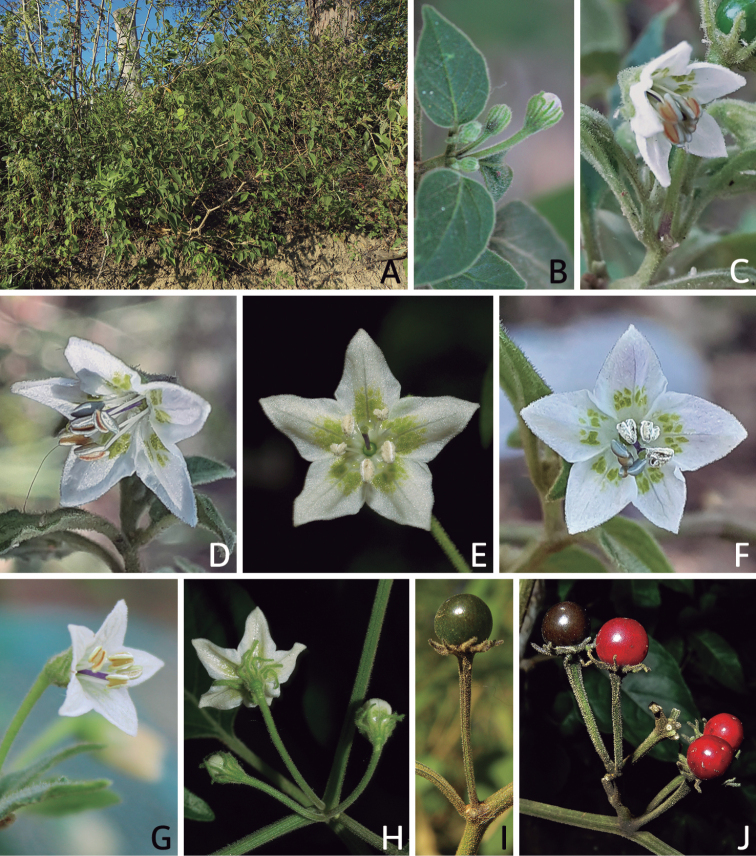
*Capsicumeshbaughii***A** plant **B** flower buds **C** flower, in pre-anthesis **D–G** stellate corollas with different colouration patterns **H** flower buds and flower in anthesis, seen from behind **I** immature fruit **J** mature fruits **A, E, H–J** from *Carrizo García 67*, photos by G.E. Barboza and C. Carrizo García **B, C, D, F, G** from *Palombo 19*, photos by N. Palombo.

*Capsicumeshbaughii* was assigned to the Purple corolla clade (sister to *C.eximium*, Carrizo García et al. 2016).). As mentioned above for *C.cardenasii*, the circumscription of this clade is being revised (CCG, pers. obs.); more details are presented under *C.pubescens* description. This is a poorly known species from not more than 10 collections, the majority of them in fruit. *Capsicumeshbaughii* can be distinguished from other species by its combination of dense, glandular pubescence covering the vegetative organs, pedicels and calyx, the presence of (5–) 10 (–12) linear unequal calyx appendages and the lack of purple pigmentation in the corollas (usually) (Fig. 58). The indumentum consists of different types of glandular trichomes (Barboza 2011): long simple trichomes with multicellular stalks and unicellular stipitate or not stipitate heads are the most common; short trichomes with bicellular stalks and multicellular heads appear in the calyx and are rare; and furcate trichomes with both branches ending in a unicellular head or one eglandular branch and the other longer and glandular are also common. Eglandular simple or furcate trichomes also appear sparsely intermixed amongst the glandular trichomes.

Experimental studies have shown that *C.eshbaughii* hybridises freely with *C.eximium* and *C.cardenasii*, the others ‘ulupicas’, producing highly fertile hybrids (Eshbaugh and Smith 1971). However, natural hybrids between *C.eshbaughii* and *C.eximium*, this latter a more or less sympatric species (Carrizo García et al. 2016), have not been found to date.

Barboza (2011), following the protologue, cited IND as the location of the holotype of C.eximiumvar.tomentosum. In IND, however, there are three specimens, so a lectotypification is needed. The sheets at IND are flowering branches with the collection date cited in the protologue of 29 November 1960; these are all syntypes. The two specimens housed at MU were gathered on different dates, one of them is dated 8 June 1960 (MU000020803, acc. # 153648) and the other (MU000020804, acc. # 153649) lacks a collection date; these are, therefore, from a different gathering and are not type material as cited in Barboza (2011). In DAV, there are another nine specimens grown from the same Smith seed collection in the University of California at Davis greenhouse with unknown dates of harvest. None of these specimens is type material. We designate IND-0105969 (acc. # 139720) as the lectotype, because it is the most well-preserved of the specimens at IND.

#### Specimens examined.

See Suppl. material 4: Appendix 4.

### ﻿16. Capsicum eximium

Taxon classificationPlantaeSolanalesSolanaceae

Hunz., Darwiniana 9(2): 235. 1950.

E1C7FF75-FA67-55B5-8911-23D61B2B8BD7

Figs 59 , 60

#### Type.

Argentina. Salta: Dpt. Guachipas, Quebrada de San Antonio, Pampa Grande, 1600 m elev., semillas del ejemplar A. T. Hunziker 1907, cultivadas en el Jardín Botánico de la Facultad de Agronomía y Veterinaria de Buenos Aires, 4 Mar 1943, *A.T. Hunziker 7346* (lectotype, designated by Barboza 2011, pg. 30: CORD [CORD00006579]).

#### Description.

Erect shrubs or subshrubs, 0.5–3 (–4) m tall, the main stem thick, up to 6 cm in diameter at base, much branched from near the base, the branches fragile, flexuous, fragile in a typical “zig-zag” appearance above. Young stems strongly angled, fragile, green, moderately pubescent with antrorse, flexuous, simple, uniseriate, 4–7-celled, eglandular trichomes 0.5–1.6 mm long and sparse minute glandular trichomes (stalk short, translucent, 1–2-celled; head dark, multicellular); bark of older stems greyish-white, brown or dark green, fissured, glabrous; lenticels absent. Sympodial units difoliate, the leaves geminate; leaf pair more or less similar in size and shape. Leaves membranous, slightly discolorous or concolorous, glabrescent on adaxial and abaxial surfaces and margins with sparse eglandular trichomes like those on stems, but abundant in a tuft of trichomes in the basal vein axils and spreading along the mid-vein abaxially; blades of major leaves 3.4–12.5 cm long, 2.1–5 (–6) cm wide, ovate or elliptic, the major veins 4–5 on each side of mid-vein, the base attenuate or cuneate and asymmetric, the margins entire, the apex acuminate; petioles 1–2.5 cm long, glabrescent or glabrous; blades of minor leaves 3–4 cm long, 1.3–2 cm wide, ovate or elliptic, the major veins 3–4 on each side of mid-vein, the base attenuate, the margins entire, the apex acute; petioles 0.3–0.5 cm long, with the same pubescence as the major leaves. Inflorescences axillary, 2–5 flowers per axil or flowers solitary; flowering pedicels 6–18 mm long, strongly angled, erect, geniculate at anthesis, green, scarcely to moderately pubescent with eglandular and glandular trichomes; the eglandular trichomes short, antrorse; pedicels scars inconspicuous. Buds globose or ovoid, fairly inflated, lilac, purple or yellowish-green. Flowers 5-merous, occasionally perianth 4-merous. Calyx 2–2.5 mm long, ca. 3 mm wide, cup-shaped, thick, green, green with purple spots or purple, moderately pubescent with the same eglandular and glandular trichomes as the stem, the calyx appendages (4–) 5, 1.2–2.7 (–3) mm long, subequal, thick, erect or slightly spreading, cylindrical or laterally compressed, inserted close to the margin, sparsely pubescent with the same trichomes as calyx tube or glabrescent. Corolla 5–8.5 mm long, 9–11 mm in diameter, lilac or purple or white with lilac and greenish-yellow spots outside, lobes marginally or completely lilac, purple or magenta, tube greenish-yellow or ochre and white centre within, sometimes the purple pigmentation is lacking, stellate with interpetalar membrane, 5 (4–)-lobed, halfway or less of the way to the base, pubescent adaxially with a continuous ring of long glandular trichomes (stalk 2–3-celled; head globose, unicellular) in the throat and up to near the base of the lobes, glabrous abaxially, the tube 3–4 mm long, the lobes 3–4.4 mm long, 2.4–3.6 mm wide, triangular, spreading, the margins finely ciliate, the tips acute, papillate. Stamens five, equal; filaments 2.7–3.8 mm long, white or lilac, inserted on the corolla 1–1.5 mm from the base, with auricles fused to the corolla at the point of insertion; anthers 1.7–2.1 mm long, ellipsoid or ovoid, yellow or purplish, not connivent at anthesis. Gynoecium with ovary 1.3–1.8 mm long, 0.9–1.5 mm in diameter, green, ovoid; ovules more than two per locule; nectary ca. 0.4 mm tall; styles homomorphic, 3.5–4.5 mm long, barely exserted beyond the anthers, lilac or white, clavate; stigma ca. 0.2 mm long, 0.5 mm wide, discoid, pale green. Berry 7–10 mm in diameter, globose, green or green with black or violet spots turning to dark brown when immature, bright red at maturity, deciduous, pungent (in some populations non-pungent), the pericarp thick, opaque, with giant cells (endocarp alveolate); stone cells absent; fruiting pedicels 14–24 mm long, erect, strongly angled, widened distally, green or purplish-green; fruiting calyx 3–5 mm in diameter, persistent, not accrescent, discoid, green or purple, the appendages 1–3.3 mm long, spreading or reflexed. Seeds 7–17 per fruit, 2.8–4.2 mm long, 2.1–3 mm wide, C-shaped or subglobose, brownish-yellow, the seed coat faintly reticulate (SM), reticulate-cerebelloid (SEM), the cells irregular in seed body and polygonal at margins, the lateral walls sinuate in seed body, straight to wavy at margins; embryo imbricate.

**Figure 59.**
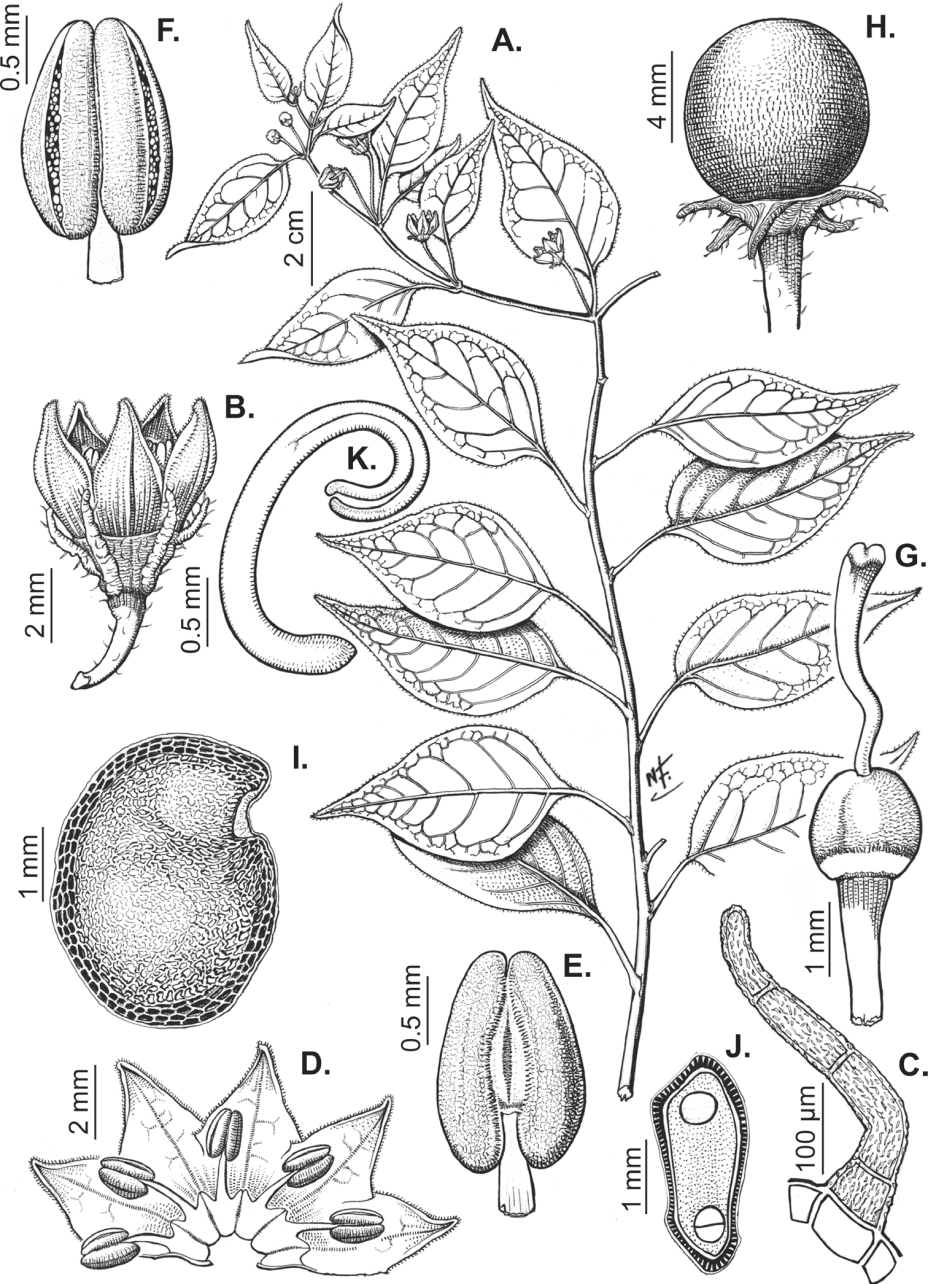
*Capsicumeximium***A** flowering branch **B** flower **C** eglandular trichome of the calyx **D** opened corolla **E, F** anthers, dorsal and ventral views, respectively **G** gynoecium **H** fruit **I** seed **J** seed, in cross section **K** embryo **A** from *Hunziker 7346***B–K** from *Hunziker 1907*. Drawn by N. de Flury. Published in Barboza (2013), courtesy of the Board of the Instituto Darwinion (San Isidro, Buenos Aires, Argentina), reproduced with permission.

**Figure 60.**
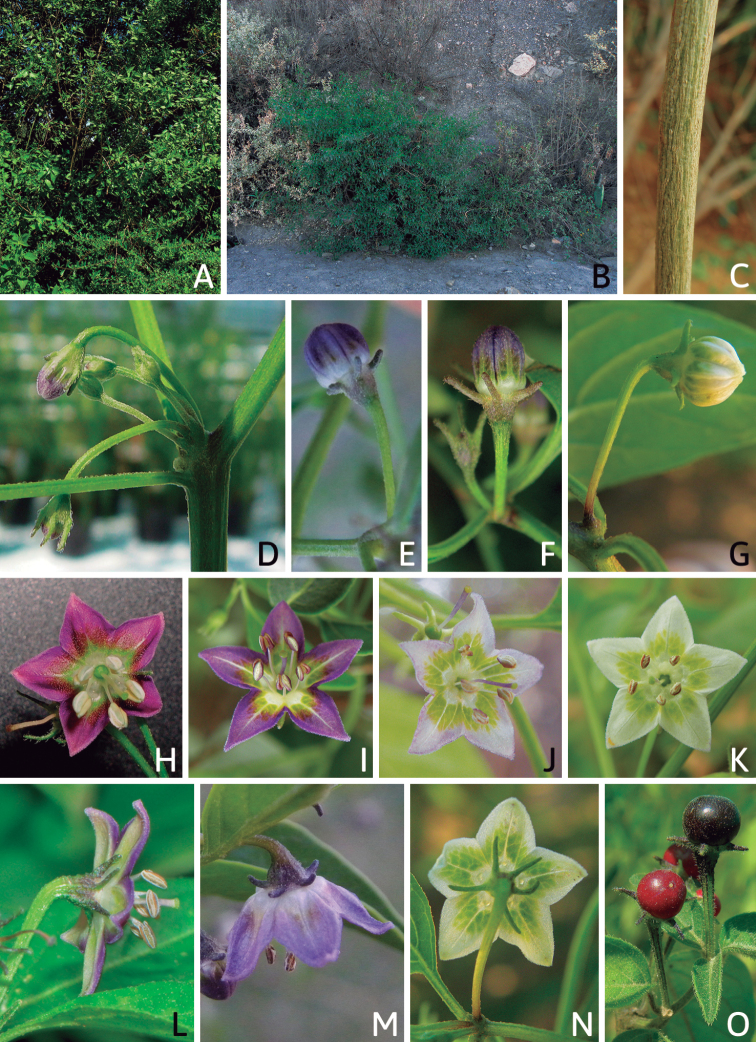
*Capsicumeximium***A** plant (wet habitat) **B** plant (dry habitat) **C** main fissured stem **D** young inflorescence **E–G** flower buds with different colouration **H–K** flowers, in front view, with different corolla colouration within **L** flower, in lateral view **M, N** flowers seen from behind **O** mature fruits **A, G, N** from *Barboza et al. 4914***B, E, M** from *Barboza et al. 4885***C, K** from *Barboza et al.* 4903 **D** from Wageningen Netherlands University germplasm collection **F, I** from *Barboza et al. 4895***H, O** from *Barboza 1919* (cult.) **J** from *Barboza et al. 4896***L** from *Barboza et al. 3543***A–C, E–O** photos by G.E. Barboza, **D** photo by P. Bosland.

#### Distribution.

**Figure 61.**
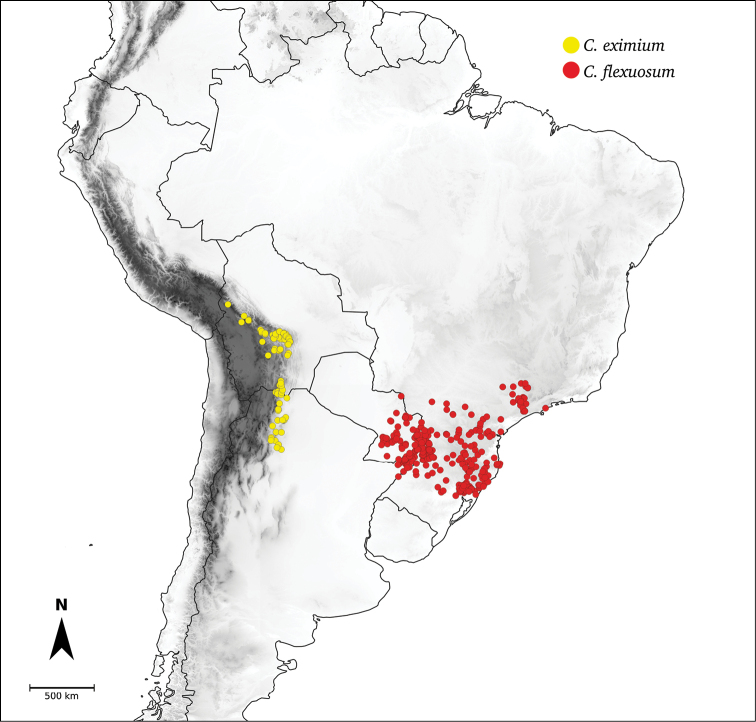
Distribution of *C.eximium* and *C.flexuosum*.

*Capsicumeximium* occupies a continuous area from northern Bolivia (La Paz, Potosí, Cochabamba, Santa Cruz, Chuquisaca and Tarija Departments) to northern Argentina (Jujuy, Salta and Tucumán Provinces) (Fig. 61). Its southern-most distribution (Argentina, Tucumán) needs to be re-confirmed since the two extant collections date from a hundred years ago.

#### Ecology.

*Capsicumeximium* grows preferentially in dry mesothermic and sub-Andean valleys with deciduous forest and scrub (Chaco), in pastures or on the edge of cultivated fields; it is often found on steep or gentle slopes, along dried watercourses or in remnant of forests dominated by Mimosoideae, *Schinopsis* or cacti, between 1,000 and 3,000 m elevation.

#### Phenology.

Flowering from November to April; fruiting from late December to May.

#### Chromosome number.

*n* = 12 (Heiser and Smith 1958); 2*n* = 2x = 24 (Pickersgill 1977; Moscone et al. 2003, 2007).

#### Common names.

**Argentina**: Ulapuca (Salta, *Lahitte s.n.*), Ulupica (Salta, *Schinini et al.34774*), Ají cobincho (Salta, *Hilgert 2061*); **Bolivia**: Ulupica (Cochabamba, *Peñaranda 458*; La Paz, *Beck 25261*; Potosí: *Zamora 193*; Santa Cruz, *Vargas C. 1382*), Ají ulupica (Tarija, *Manchego CEP T21*), Ulupica muruchi, Ulupica negra semiosca, Ulipica grande hosca, Ulupica tuna, Ulupica negra neta, Ulupica blanca, Ulupica verde, Ulupica camba, Ulupica negra con flor blanca (Chuquisaca, Libreros et al. 2014).

#### Uses.

The fruits are very pungent and are used as spices or in pickles in the Bolivia (National Research Council 1989; Jäger et al. 2013) and Argentina (Eshbaugh and Smith 1971; Barboza, pers. obs.). Fresh or dry fruits are powdered to prepare a sauce known as “llaswa” (see details in *C.cardenasii*) that people add to “empanadas” (little meat pies) in Argentina; ‘ulupica’ is not only an ingredient in “llaswa”, but also in many recipes in Bolivia.

#### Preliminary conservation assessment.

EOO (278,222.889 km^2^); AOO (396 km^2^). *Capsicumeximium* is found over a large extent of occupancy from northern Bolivia to northern Argentina and is common in the inter-Andean valleys. We suggest the status of Least Concern (LC). It is a species of open areas, forming small populations and is sometimes cultivated on farms for self-consumption of the fruits.

#### Discussion.

*Capsicumeximium* is a member of the Purple corolla clade (Carrizo García et al. 2016). New preliminary evidence on the affinities of species in this clade is discussed under *C.pubescens*. *Capsicumeximium* is the most widespread ‘ulupica’ with corolla colour as its most variable character (Fig. 60H–N). Eshbaugh (1982) pointed out that, within the same population, he could observe individuals with white, cream or purple corollas; he also associated this variation with the distribution of the species, indicating that the forms with white corollas were restricted to the northern part of its range while populations with purple corollas occurred in southern Bolivia (Tarija). Data from modern collections reinforce that there is high inter- and intrapopulation variability in corolla colour (e.g. *Nee 37575*, *Barboza et al. 4895 & 4896*); this deserves further field studies to better understand if populations with white corollas (as stated in many herbaria labels, for example, *Vargas C. 36 & 816*, *Cárdenas 4237*, *Saravia Toledo 12126, Wood 20218*) refer to entirely white corollas or white corollas with greenish-yellow centres (e.g. *Mendoza 801, Novara 8346, Barboza 4914*).

The pungency of the fruits is polymorphic in *C.eximium* as it is in *C.chacoense* and *C.baccatum* (Tewksbury et al. 2006), *C.flexuosum* and some cultivars of the domesticated species of the *C.annuum* complex (Carrizo García et al. 2016). In Bolivia (Tarija), individuals of *C.eximium* from the same locality have been observed to have pungent and non-pungent berries (*Manchego CEP T21* & *Manchego CENP T22*).

*Capsicumeximium* is a self-compatible species (Eshbaugh 1979; Onus and Pickersgill 2004). Its affinity with *C.cardenasii* has been confirmed by breeding studies (Heiser and Smith 1958; Eshbaugh 1976, 1979; Onus and Pickersgill 2004), chemotaxonomic and cytological work (Jensen et al. 1979; McLeod 1979a, 1979b, Moscone et al. 2007) and molecular evidence (Carrizo García et al. 2016, 2020). Morphologically, they can be distinguished by leaf size (smaller in *C.cardenasii*) and corolla shape (campanulate in *C.cardenasii* and stellate in *C.eximium*). Eshbaugh (1976) has demonstrated that *C.eximium* and *C.cardenasii* interbreed freely and produce fertile hybrids; he also stated that it is possible to find intermediates between the two taxa (no vouchers cited, Eshbaugh 1982). *Capsicumeximium* also hybridises naturally with *C.pubescens*, a domesticated species with purple corollas (Eshbaugh 1979, 1982; Barboza, pers. obs.). The collection *Barboza et al. 1849* from La Paz (Bolivia) is an intermediate with the pubescence and corolla shape (stellate) of *C.eximium* and the corolla size and colour (purple) and fruit size of *C.pubescens*; no seeds were observed. Similar specimens have been collected recently from experimental crossings between the two taxa made at a rural farm in Chuquisaca Department, Bolivia (*Barboza et al. 4925* & *4926*).

#### Specimens examined.

See Suppl. material 4: Appendix 4.

### ﻿17. Capsicum flexuosum

Taxon classificationPlantaeSolanalesSolanaceae

Sendtn., Fl. Bras. (Martius) 10(6): 143. 1846.

EA12C8C6-89DC-5EB9-9B4F-889051A18992

Figs 62 , 63

Capsicum schottianum Sendtn. var. leptophyllum Dunal, Prodr. [A. P. de Candolle] 13(1): 416. 1852. Type. Brazil. “In Brasiliâ australiore. (Sellow e Sendtn. l.c.)” (no herbaria cited; no original material found).
Capsicum parvifolium Sendtn. var. sellowianum Dunal, Prodr. [A. P. de Candolle] 13(1): 419. 1852. Type. Brazil. “In Brasilia australiore. (Sellow.)” (no herbaria cited; no original material found).
Capsicum campylopodium Sendtn. forma magis-puberula Chodat, Bull. Herb. Boissier ser. 2, 2: 815. 1902. Type. Paraguay. [Canindeyú]: Sierra de Maracayú, in silva Ipé hú, Oct 1898–1899, *É. Hassler 5134* (lectotype, designated by Barboza 2011, pg. 26 [G], second step designated here: G [G00390263]; isolectotypes: A [00936719], BM [BM000074110], CORD [CORD00087947, fragment ex G], G [G00390264], G [G00390265], K [K000648540], NY [04206101], P [P00410080, P 00410081], S [S16-28249], UC [UC-944853]).
Capsicum campylopodium Sendtn. forma laurifolium Chodat, Bull. Herb. Boissier ser. 2, 2: 815. 1902. Type. Paraguay. [Itapuá]: in silva pr. fl. Capibary, 5 Dec 1898–1899, *É. Hassler 5893* (lectotype, designated here: G [G00390270]; isolectotypes: BM [BM000074083], G [G00390269], K [K000648539], P[P00410127], S [S16-27826], UC [UC950167]).
Capsicum mositicum Toledo, Arq. Bot. Estado São Paulo 3(2): 64, f. 2. 1953. Type. Brazil. São Paulo: Mun. de Amparo, Monte Alegre, margem da estrada para Socorro, 740 m elev., 17 Dec 1942, *M. Kuhlmann 144* (holotype: SP [001631, acc. # 47939]; isotypes: CORD [CORD00006628, CORD00006629]).
Capsicum ramosissimum Witasek, Denkschr. Kaiserl. Akad. Wiss., Wien. Math.-Naturwiss. Kl. 79(2): 320. 1910. Type. Brazil. “Prov. São Paulo: Prope “Fazenda bella vista”, in districtu urbis Santa Cruz ad flumen Rio Pardo, ca. 500 m elev., VII, leg. *v. Wettstein et Schiffner*”, Jul 1901, *R. v. Wettstein & V. Schiffner* [*338*] (lectotype, designated here: WU [acc. # 0037945]; isolectotypes: CORD [CORD00006633], F [v0093723F, acc. # 871103, fragment], W [1922-0001510], WU [acc. # 0037946], Z [Z-000038683]).
Capsicum schottianum Sendtn. var. flexuosum (Sendtn.) Hunz., Huitième Congr. Int. Bot. Paris. Comptes Rend. Séances Rapp. & Commun. 1954, sect.4: 73. 1956. Type. Based on Capsicumflexuosum Sendtn.

#### Type.

Brazil. “In Brasilia australiore: Sellow” (no original material located; Brazil. Paraná: Mun. Curitiba, Parque Iguaçu, 27 Dec 1979, *R. Kummrow 1307*; neotype, designated here: MBM [MBM064252]; isoneotype, CORD [CORD00101762]).

#### Description.

Erect subshrubs or shrubs, (0.3–) 0.9–2 m tall, more rarely small trees 2–2.5 m tall, with the main stem thick 2–2.5 cm in diameter at base, much branched above, the branches dichotomously spreading in a typical “zig-zag” appearance. Young stems terete or slightly ridged, fragile, green, glabrous to moderately or densely pubescent, with antrorse simple, uniseriate, 2–6-celled, eglandular trichomes 0.2–0.9 mm long; nodes solid, green; bark of older stems dark brown, glabrous, rarely pubescent; lenticels absent. Sympodial units difoliate, the leaves geminate; leaf pair unequal or more or less equal in size, similar in shape. Leaves membranous, discolorous, dark green above, light green or greenish-grey beneath, moderately pubescent on both sides or, if glabrescent, with an evident tuft of trichomes in the basal vein axils abaxially, the trichomes appressed-antrorse similar to those of the stems; blades of major leaves 5.2–14 cm long, 1.5–4.5 (7.5–) cm wide, ovate to elliptic, the major veins 4–7 on each side of mid-vein, the base attenuate or short-attenuate, the margins entire, the apex acuminate; petioles 0.5–1.5 cm, glabrous to moderately pubescent; blades of minor leaves 1.7–3.5 (–4.5) cm long, 0.5–2 cm wide, ovate to elliptic, the major veins 3–4 on each side of mid-vein, the base attenuate, the margins entire, the apex acute to acuminate; petioles 0–0.5 cm, with same pubescence as the major leaves . Inflorescences axillary, 2–3 (–6)-flowers per axil or flowers solitary; flowering pedicels 10–21 mm long, terete, pendent, more rarely spreading, non-geniculate at anthesis, glabrous to moderately pubescent, the eglandular trichomes short, antrorse; pedicels scars inconspicuous. Buds globose, inflated, white with light green spots. Flowers 5-merous. Calyx 1.3–2 mm long, 3–3.5 mm wide, cup-shaped, green or greenish-yellow, hyaline at the margin, glabrous to moderately pubescent, the calyx appendages five, inconspicuous, umbo-like and the calyx pentagonal in outline with a tuft of short uniseriate eglandular trichomes on each angle, if the calyx appendages absent, the calyx circular in outline. Corolla 6–11 mm long, ca. 13 mm in diameter, white with yellowish-green pigmentation outside, white with variously yellowish-green spots in the base of lobes and throat within, exceptionally also with purple spots, stellate or rotate-stellate, with thin interpetalar membrane, lobed less than or up to nearly halfway to the base, the tube 3–6 mm long, pubescent adaxially with a continuous ring of glandular trichomes (stalk long 1–3-celled; head globose, peltate, unicellular), glabrous abaxially, the lobes 3–5 mm long, 2–4.5 (–5) mm wide, broadly triangular, spreading, glabrous adaxially and abaxially, the margins involute, papillate, the tips cucullate, densely papillate. Stamens five, equal, filaments 2.5–3.5 mm long, cream, inserted on the corolla 1–1.3 mm from the base, with auricles fused to the corolla at the point of insertion; anthers 1.1–1.9 mm, ellipsoid or ovoid, yellow, not connivent at anthesis. Gynoecium with ovary 1.3–2 mm long, 1.2–1.4 mm in diameter, light green, ovoid; ovules more than two per locule; nectary ca. 0.4 mm tall; styles homomorphic, (3.8–) 4–5.7 mm, exserted 0.8–1 mm beyond the anthers, white, clavate; stigma 0.2 mm long, 0.8 mm wide, discoid, pale green. Berry 5–8 mm in diameter, subglobose or globose, brilliant green when immature, reddish-orange or red at maturity, deciduous, pungent, the pericarp thick, opaque, with giant cells (endocarp alveolate); stone cells absent; fruiting pedicels 15–26 mm long, pendent, terete, widened distally, green; fruiting calyx 4–5 mm in diameter, persistent, not accrescent, discoid, light green, sometimes the margin ripped. Seeds (4–) 5–25 per fruit, 2.8–3.4 mm long, 2.2–3 mm wide, C-shaped, subglobose or teardrop-shaped, brownish-black, the seed coat faintly reticulate (SM), reticulate (SEM), the cells polygonal to irregular in shape, the lateral walls sinuate in the seed body and straight to wavy at margins; embryo imbricate or coiled.

#### Distribution.

*Capsicumflexuosum* is a widespread species occupying a more or less continuous range from north-eastern Argentina (Corrientes and Misiones Provinces) and southern Paraguay (Alto Paraná, Amambay, Caaguazú, Caazapá, Canindeyú, Central, Guairá, Itapuá, Paraguarí and San Pedro Departments) to south and south-eastern Brazil (Minas Gerais, Paraná, Rio Grande do Sul, Santa Catarina and São Paulo States) (Fig. 61).

#### Ecology.

*Capsicumflexuosum* is very common in the interior and margins of primary and secondary forests, in remnants of forests or disturbed areas. In Brazil, it occurs in a wide range of vegetation types, primarily in the Atlantic Rainforest (Floresta Estacional Decidual, Floresta Estacional Semidecidual Montana and Submontana, Floresta Ombrófila Densa Submontana and Montana, Floresta Ombrófila Mista Montana, Floresta Ombrófila Densa das Terras Baixas), at low and medium altitudes, between 30 and 1,300 m.

#### Phenology.

Flowers nearly all year around; fruiting occurs from January to July and from October to December.

#### Chromosome number.

*n* = 12 (Moscone 1992; Pozzobon and Schifino-Wittmann 2006); 2*n* = 2x = 24 (Pozzobon et al. 2006; Moscone et al. 2007).

#### Common names.

**Argentina**: Cumbarí (Misiones, *Montes 2139*), Pimentina (Misiones, *Montes 2375*), Pimentiña (Misiones, *Montes 2375*), Ai Jesu (Misiones, *Buchinger & Rodríguez 3222*), Ají Cumbarí (Corrientes, *Bonpland s.n.*), Pimenta silvestre (Misiones, *Montes 2140*), Pimienta del monte (Misiones, *Schwindt 4310*), Pimiento del monte (Misiones, *Buchinger & Rodríguez 3222*), Pimentón del monte (Misiones, *Schwindt 1871*); **Brazil**: Pimenteira (Rio Grande do Sul, *Santos et al. 2869*), Pimenta-braba (Rio Grande do Sul, *Abruzzi 576*), Pimenta de passarinho (Paraná, *Lleras Pérez et al. 1947*), Pimenta do bugni (Santa Catarina, *Neubert 216*), Pimento-do-mato, pimenta-braba (Santa Catarina, *Schwirkowski 1519*); **Paraguay**: Locote (Canindeyú, *Montes 3280*), Pimiento silvestre (Canindeyú, *Montes 3280*).

#### Indigenous names.

**Argentina**: Guachu ky’i (Guabyrá poty, Misiones, *Keller & Ferreira 1349*), Guach ky’yi (Takuapi, Misiones, *Keller 2957*), ‘Ka’a ete’y’ (Guabyrá poty, Misiones, *Keller & Ferreira 288*); **Paraguay**: Ke-jiú (Canindeyú, *Montes 3280*).

#### Uses.

Herbarium labels record that fruits are used as condiments in Argentina and Paraguay due to their high pungency. There are no records of uses from Brazil.

#### Species conservation assessment.

EOO (778,640.645 km^2^); AOO (1,348 km^2^). *Capsicumflexuosum* is quite abundant throughout its distribution. Based on the EOO, the AOO and its presence in many conservation units, we consider *C.flexuosum* is not under risk and assign the category of Least Concern (LC).

#### Discussion.

*Capsicumflexuosum* is the single member of the Flexuosum clade (Carrizo García et al. 2016). It is a highly variable species in its pubescence density that varies from absent (plants glabrous) to moderate or dense on young branches, leaves and calyx; on leaves, the simple eglandular trichomes are located mainly on the abaxial surface of the lamina. The name *C.mositicum* was applied to glabrous or glabrescent specimens with a tuft of trichomes in the abaxial leaf vein axils. Another striking aspect of *C.flexuosum* is the profuse, dichotomous-divaricate branching at the apex of young branches that give the plant a messy aspect, with crowded leaves, flowers and fruits; *C.ramosissimum* was described, based on this peculiar trait. It is possible that this growth pattern is caused by a plant/pathogen (bacterium, fungus, virus or insect).

**Figure 62.**
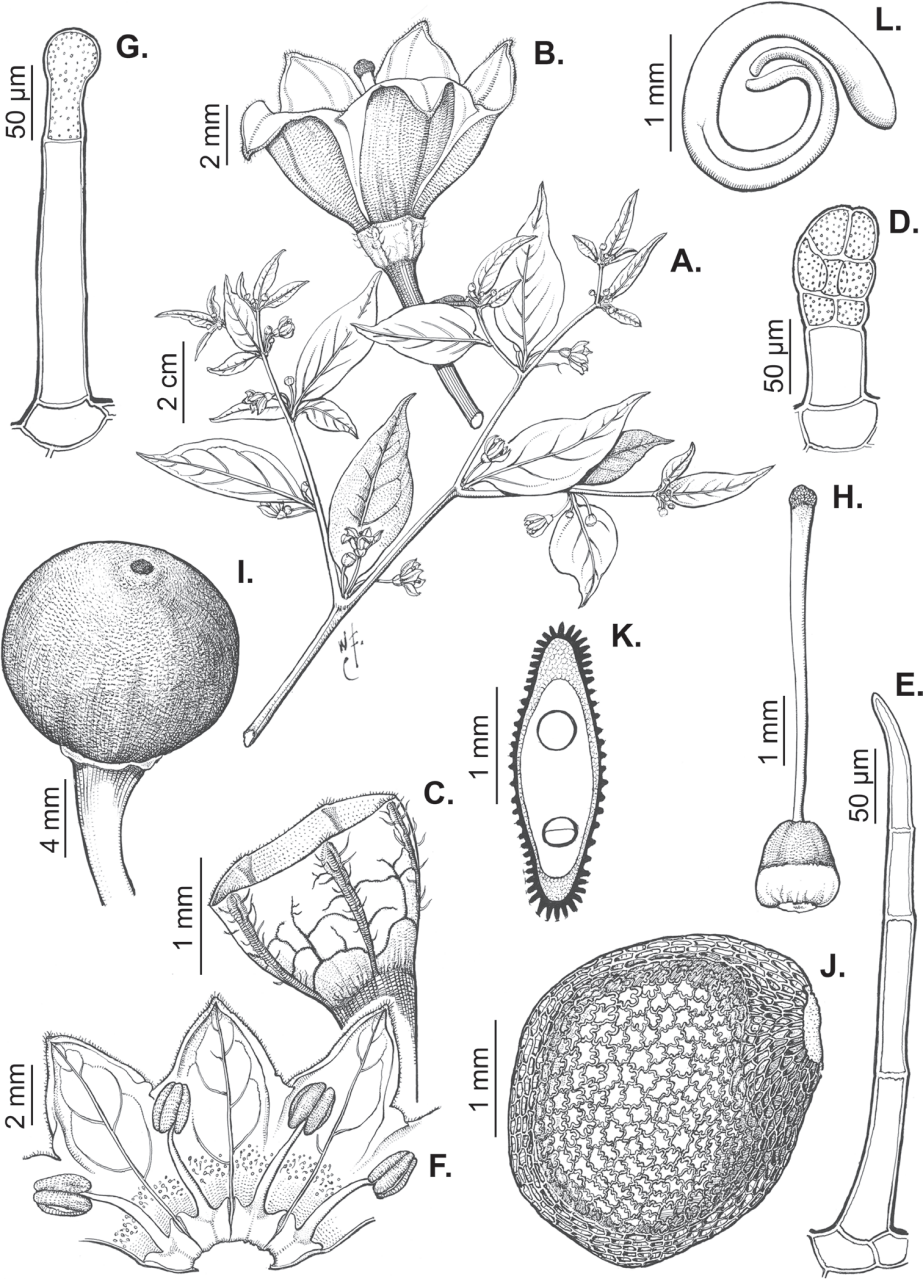
*Capsicumflexuosum***A** flowering branch **B** flower **C** calyx **D** glandular trichome of the abaxial surface of the calyx **E** eglandular trichome of the adaxial surface of the calyx **F** sector of opened corolla **G** glandular trichome of the abaxial surface of the corolla **H** gynoecium **I** fruit **J** seed **K** seed, in cross section **L** embryo **A** from *Hatschbach 18030***B–H** from *Subils & Moscone 4273***I–L** from *Hunziker et al. 24993*. Drawn by N. de Flury. Published in Barboza (2013), courtesy of the Board of the Instituto Darwinion (San Isidro, Buenos Aires, Argentina), reproduced with permission.

**Figure 63.**
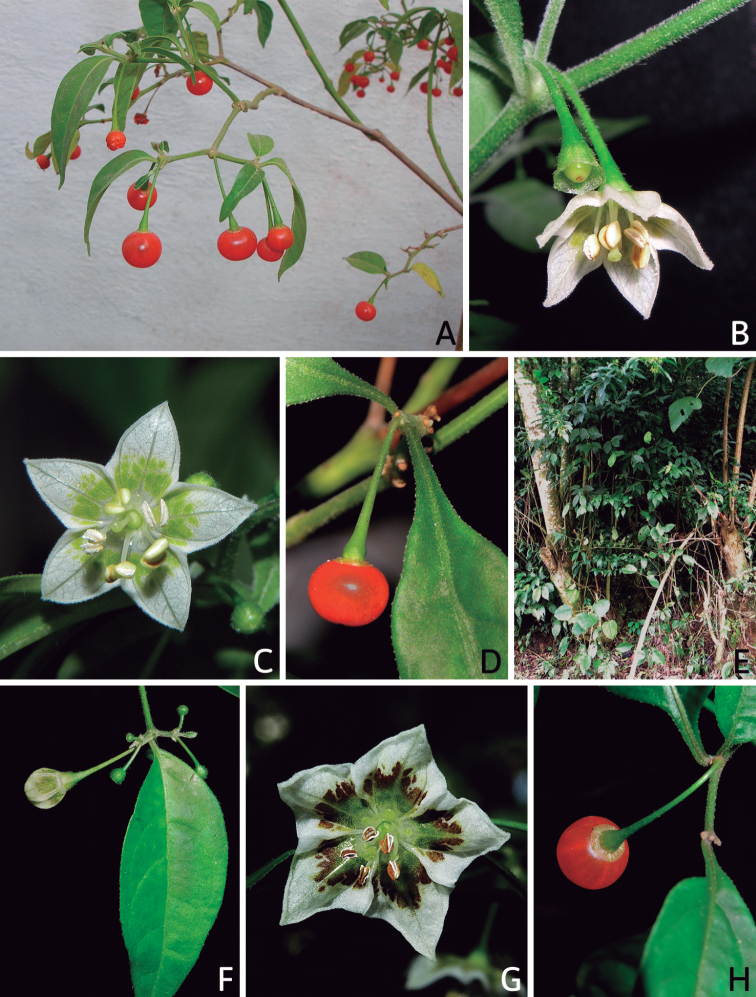
*Capsicumflexuosum***A** fruiting branch **B** flowering node **C** stellate corolla with greenish spots **D, H** mature fruits **E** plant **F** flower bud **G** rotate-stellate corolla with purple and greenish spots **A–D** from *Barboza et al. 1034***E***Barboza et al. 3631***F–H** from *Carrizo García 84*. Photos by C. Carrizo García.

Another variable trait is the greenish-yellow pigmentation of the white corolla; this colouration can be present as many small spots interrupted by white lines covering the throat and the base of the lobes or it can be present as five large spots covering the surface (Fig. 63C) or the greenish-yellow pigmentation can extend over the lobes, leaving a thin white edge.

A striking population from around Monteiro Lobato (São Paulo State, Brazil) is morphologically very similar to *C.flexuosum* in its habit, pendent, non-geniculate flowering pedicels, pentagonal calyx, orange-red globose pungent fruits and black seeds. However, unlike other *C.flexuosum* populations in which the corolla is clearly stellate and white with greenish-yellow spots (Fig. 63E–H), the population in Monteiro Lobato has a rotate-stellate white corolla with greenish-yellow pigmentation in the throat and purple spots in the lobes (similar to *C.schottianum*). An accession of this population (as Capsicumaff.flexuosum) was included in the molecular phylogeny (Carrizo García et al. 2016) and was recovered within the Flexuosum clade. In addition, its chromosome number is 2n = 24 (see Table 2). Since this population occurs within other typical populations of *C.flexuosum* (Pozzobon et al. 2006; Moscone et al. 2007), is treated here as a local variant of *C.flexuosum*.

*Capsicumschottianum* and *C.campylopodium* are the most morphologically similar species to *C.flexuosum*. All three of these species lack calyx appendages and are extremely difficult to distinguish from one another in herbarium material, when the corolla colour is not stated on the labels or only fruit is present. *Capsicumflexuosum* differs from *C.campylopodium* and *C.schottianum* in its non-geniculate (vs. geniculate) pedicels, yellowish-green spots on the adaxial surface of the corolla (absent in *C.campylopodium* and in combination with purple spots in *C.schottianum*) and edible reddish-orange or red fruit (vs. greenish-golden yellow fruits).

For over 100 years, *C.flexuosum* has been misinterpreted in both literature and herbaria. Chodat (1902) and Chodat and Hassler (1903) cited specimens of *C.flexuosum* from Paraguay under *C.campylopodium*, a Brazilian endemic. In addition, these authors described infraspecific names, such as formamagis-puberula and formalaurifolium. Hunziker (1950) confused *C.flexuosum* with *C.schottianum*, but later amended the error (Hunziker 1998) and restricted the distribution of *C.schottianum* to Brazil (Hunziker 2001). Finally, in floristic Brazilian works, some specimens of *C.recurvatum* have been referred to as *C.flexuosum* (Freire de Carvalho 1985).

Sendtner (1846) described *C.flexuosum* and cited a single un-numbered collection made by Friedrich Sellow from “Brasilia australiore”, but as was usual at the time, he did not cite a herbarium. Despite searching in the many herbaria (e.g., BM, F, G, K, M, W) where Sellow’s collections are held today, the only Sellow collection we have seen that corresponds to *C.flexuosum* was held in Berlin (F neg. 2868) and was destroyed in World War II. In the interests of fixing the application of this name, we are designating a neotype using a modern collection that shows all the diagnostic characters of the species (*R. Kummrow 1307*: MBM064252; CORD00101762).

Dunal (1852) proposed C.schottianumvar.leptophyllum for the unnamed var. β of Sellow’s *C.schottianum* (Sellow 1846: 144). This name refers to plants with narrower leaves and fruiting pedicels that are not curved, but arched throughout their length in contrast to the typical fruiting pedicels that are initially rigid, geniculate (curved) and erect to spreading in *C.schottianum* (pedicels pendent in mature fruits). As no original material was found, we propose the varietal name *leptophyllum* under *C.flexuosum*, based on the pedicel features that fit exactly with that species (i.e. pedicels never curved in flower and fruit).

Dunal (1852) based the name C.parvifoliumvar.sellowianum on a previous Sellow’s description (Sellow 1846: 146) given for a fruitful specimen with “foliis glabrioribus, ... calyci deplanato 5-angulari ...”. As no original material was found, we propose this name as a synonym of *C.flexusoum* due to glabrous leaves and pentagonal calyx (calyx appendages minute) described in the protologue which fit best with *C.flexuosum*. *Capsicumparvifolium* has pubescent leaves and five well-developed calyx appendages.

Chodat (1902) based the description of C.campylopodiumformalaurifolium on the collection *Hassler 5893*, without citing a herbarium. We found two sheets of this gathering at G, both consisting of very well-preserved reproductive branches. We select the better of these, G00390270, as the lectotype.

Witasek (1910) described *C.ramosissimum* on the basis of two Wettstein & Schiffner specimens, both from “Fazenda bella vista” (São Paulo, Brazil). We found the two syntypes at WU where the collections she used are held; the specimen numbered *Wettstein & Schiffner 338* (WU 0037945) is selected as the lectotype, because it is well-preserved and has duplicates housed at CORD, F, W, WU and Z.

#### Specimens examined.

See Suppl. material 4: Appendix 4.

### ﻿18. Capsicum friburgense

Taxon classificationPlantaeSolanalesSolanaceae

Bianch. & Barboza, Syst. Bot. 30(4): 865. 2005.

F1C8339C-182B-52D7-8150-DC0CADD55BE3

Figs 64 , 65

#### Type.

Brazil. Rio de Janeiro: Mun. Nova Friburgo, subindo o Morro da Caledônia, a 6.45 km do Camping Club do Brasil (RJ.2), 22°17'S, 42°32'W, 1800 m elev., 6 Apr 1986, *L. Bianchetti, A.T. Hunziker, V. Casali & G.P. Silva 393* (holotype: CEN [CEN00010214]; isotypes: CORD [CORD00003939, CORD00003940, CORD00004164]).

#### Description.

Erect shrubs 0.8–2.5 (–3) m tall, with the main stem 0.8–1.5 cm in diameter at base, few to much branched above, the branches pendulous dichotomously spreading in a typical “zig-zag” appearance. Young stems angled, fragile, green, glabrescent, with sparse appressed-antrorse, simple, uniseriate, 2–3-celled, eglandular trichomes 0.2–0.5 mm long and abundant minute glandular trichomes (stalk translucent, unicellular, head dark and multicellular); nodes solid, green or slightly purple; bark of older stems dark brown, smooth, glabrous; lenticels absent. Sympodial units difoliate, the leaves geminate; leaf pair unequal in size, similar in shape. Leaves membranous, concolorous to slightly discolorous, glabrescent on both sides, especially on the margins and veins, with antrorse, curved, 3–5-celled, eglandular trichomes 0.3–0.7 mm long; blades of major leaves (5.5–) 8.5–13 (–21) cm long, (1.5–) 2.5–4.5 (–7.5) cm wide, ovate to elliptic, the major veins 6–7 on each side of mid-vein, the base short-attenuate or attenuate, unequal, the margins entire, the apex acuminate; petioles 0.6–1.2 (–1.5) cm, glabrescent; blades of minor leaves (1.8–) 2.2–3.3 (–5) cm long, 0.9–2 (–2.5) cm wide, elliptic or ovate, the major veins 3–5 on each side of mid-vein, the base rounded, the margins entire, the apex acute; petioles 0.2–0.5 cm, glabrescent. Inflorescences axillary, 2-flowered or flowers solitary; flowering pedicels (17–) 21–49 (–65) mm long, angled, erect or slightly spreading, geniculate at anthesis, green, glabrous or glabrescent, the eglandular trichomes minute, antrorse; pedicels scars inconspicuous. Buds ovoid, entirely purple or fuchsia. Flowers 5-merous. Calyx 2–3 mm long, 3–4 mm wide, cup-shaped, green, thin, strongly 5-nerved, glabrescent with short, uniseriate, eglandular trichomes on the margin and minute glandular trichomes on the tube similar to those of the stem and leaves, the calyx appendages five, 1.2–3 (–3.5) mm long, subequal, erect or spreading, glabrescent. Corolla (7–) 9–12 (–15) mm long, 7.5–10.5 mm in diameter, entirely violet or fuchsia at anthesis; campanulate-urceolate without interpetalar membrane, lobed at the apex, glabrous adaxially and abaxially, the tube 4–6 mm long, the lobes (1.5–) 2–3 (–4) mm long, (1.5–) 2–3 (–4) mm wide, broadly triangular, spreading to strongly recurved at anthesis, the margins and tips densely papillate or with short eglandular trichomes, the tips cucullate. Stamens five, equal; filaments (4–) 5–6 (–7) mm long, lilac, inserted on the corolla 1.5–1.75 mm from the base, with auricles fused to the corolla at the point of insertion; anthers 1.5–2 (–2.5) mm, ellipsoid, yellowish-white, grey-purple post-dehiscent, not connivent at anthesis. Gynoecium with ovary ca. 1.8 mm long, 1.2 mm in diameter, light green, globose to ovoid; ovules more than two per locule; nectary ca. 0.3 mm tall; styles homomorphic, (5–6) 8–11 mm, barely exserted beyond the anthers, white, clavate; stigma ca. 0.3 mm long, 1 mm wide, discoid-depressed, pale green. Berry (4–) 6–10 mm in diameter, globose or globose-depressed, light green and pungent when immature, dark green to greenish-golden yellow and scarcely pungent to rather sweet at maturity, deciduous, the pericarp thin, translucent, with giant cells (endocarp alveolate); stone cells absent; fruiting pedicels 35–65 mm long, pendent, angled, widened distally, green, the receptacle inflated and forming a weak annular constriction in the limit with the calyx; fruiting calyx 4–4.5 mm in diameter, persistent, not accrescent, green, discoid, the appendages 2–4 mm long, spreading or slightly reflexed. Seeds (2–) 4–9 per fruit, 2.5–3.2 mm long, 2–2.5 mm wide, C-shaped, brownish-black to black, the seed coat reticulate and tuberculate at margins (SM), reticulate-cerebelloid with pillar-like outgrowths at margins (SEM), the cells polygonal in seed body and elongate at hilum zone, the lateral walls straight, sometimes wavy in seed body; embryo imbricate.

**Figure 64.**
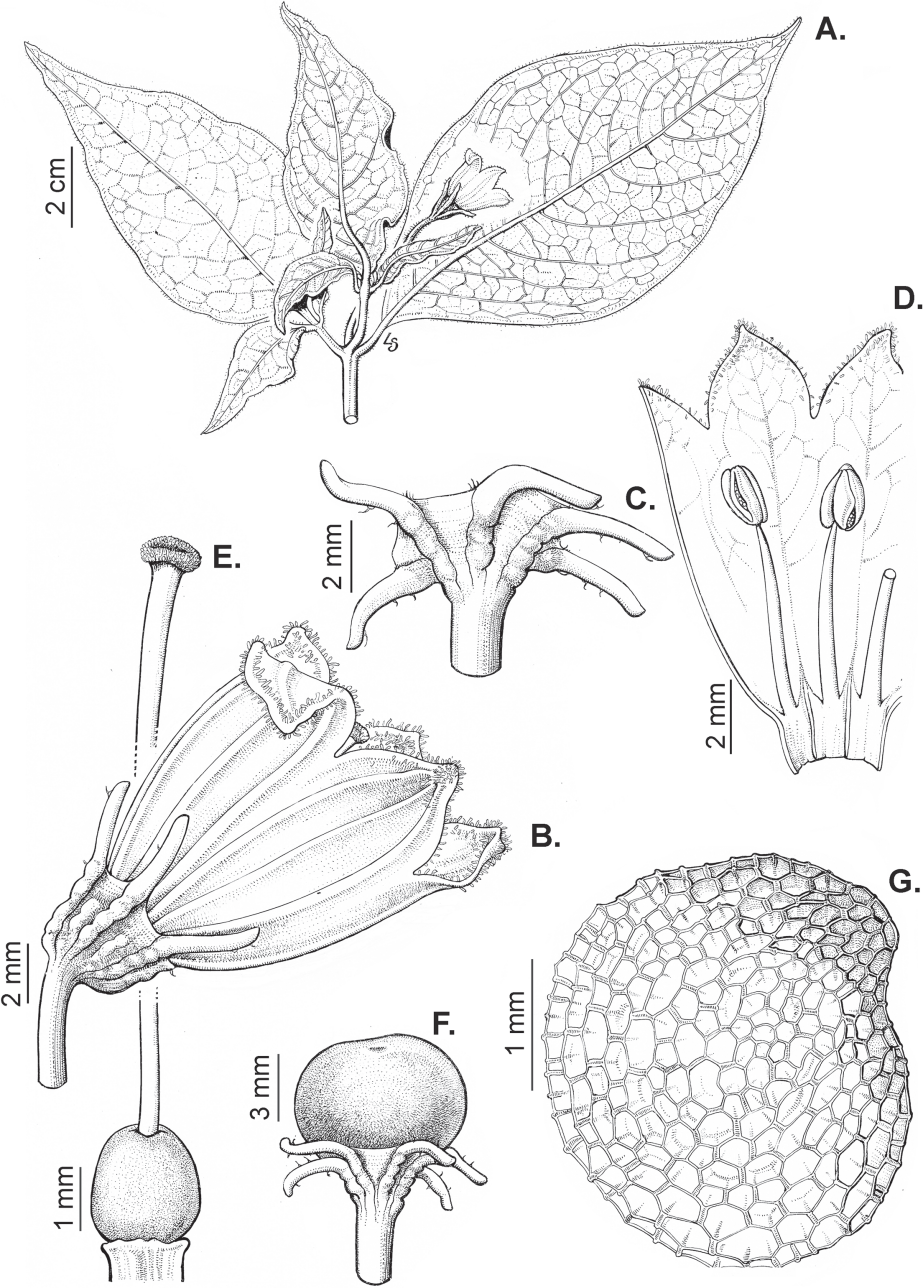
*Capsicumfriburgense***A** flowering branch **B** flower **C** calyx **D** sector of opened corolla **E** gynoecium **F** fruit **G** seed. From *Bianchetti et al. 393*. Drawn by L. Sánchez. Published in Barboza and Bianchetti (2005), reproduced with permission.

**Figure 65.**
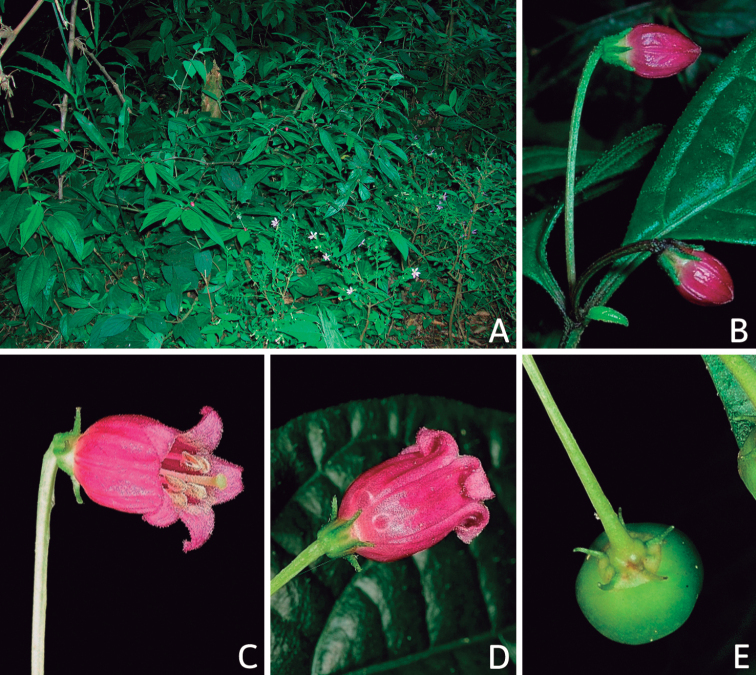
*Capsicumfriburgense***A** plant **B** flower buds **C** flower, in lateral view **D** flower, seen from behind **E** immature fruit **A, B** from *Barboza et al. 2048*, photo by G.E. Barboza **C–E** no specimen vouchers, photos taken *in situ* by C. dal Zovo (Associazione PepperFriends).

#### Distribution.

**Figure 66.**
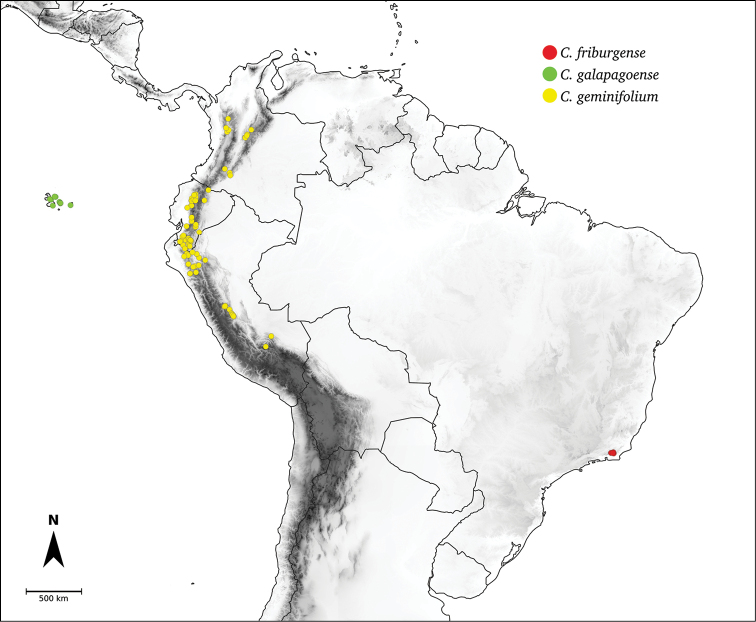
Distribution of *C.friburgense*, *C.galapagoense* and *C.geminifolium*.

*Capsicumfriburgense* is an endemic species restricted to a narrow area in the centre of Rio de Janeiro State (Brazil) (Fig. 66).

#### Ecology.

*Capsicumfriburgense* grows in one of the most protected areas of the Atlantic Forest (Mata Atlântica), which has the highest elevations of the Serra do Mar. It is found in the understorey of primary forests of the Floresta Pluvial Montana (Floresta Ombrófila Densa Altomontana), from 1,750 to 1,950 m elevation.

#### Phenology.

Flowering from December to May. Fruiting from February to May.

#### Chromosome number.

*n* = 13 (Pozzobon and Schifino-Wittmann 2006; Pozzobon et al. 2006, *as Capsicum* sp. 8).

#### Common names.

None recorded.

#### Uses.

None recorded.

#### Preliminary conservation assessment.

EOO (123.163 km^2^); AOO (24 km^2^). *Capsicumfriburgense* grows in two forested areas that have been set aside as Environmental Protection Areas in Rio de Janeiro, the APA do Caledônia (Maciço da Caledônia) and the Parque Estadual dos Três Picos. It has a small population (< 250 mature individuals) with not more than 10 subpopulations per site. Observers are seeing a continuing decline of subpopulations size, due to serious environmental pressures caused by deforestation, development and changes in hydrology. In recent years, these protected areas faced important natural disasters, including forest fires (2007, 2011 and 2019), great floods and mudslides (2011, see Rosi et al. 2019), all of which caused a loss of biodiversity. Due to all these factors, we assign *C.friburgense* the threat status of Critically Endangered (CR; C2a(i)).

#### Discussion.

*Capsicumfriburgense* is a beautiful species of the Brazilian Atlantic Forest clade (Carrizo García et al. 2016) with one of the narrowest ranges in the genus. It stands out from other species of the genus in its combination of a distinctive, campanulate-urceolate, entirely pink or lilac corollas with recurved lobes at anthesis and fruiting pedicels up to 65 mm long, the longest of all the Brazilian species (Fig. 65C–E).

*Capsicumfriburgense* is sympatric in Nova Friburgo (Rio de Janeiro) with *C.mirabile*; both species have a calyx with five appendages and geniculate pedicels and they have similar colour and morphology of fruit and seeds. *Capsicumfriburgense* differs from *C.mirabile* in having one or two flowers per node, campanulate-urceolate corollas without spots and fruiting pedicels up to 65 mm long (vs. 2–5 flowers per node, multi-coloured stellate corollas and fruiting pedicels up to 32 mm long in *C.mirabile*).

#### Specimens examined.

See Suppl. material 4: Appendix 4.

### ﻿19. Capsicum frutescens

Taxon classificationPlantaeSolanalesSolanaceae

L., Sp. Pl. 1: 189. 1753.

D685CB65-1634-56E0-9AB6-E9319899CE83

Figs 67 , 68

Capsicum conoide Mill., Gard. Dict. ed. 8, Capsicum no. 8. 1768. Type. Cultivated at the Chelsea Physic Garden (England), seeds from Antigua (Antilles) (no specimens cited).
Capsicum conicum G.Mey., Prim. Fl. Esseq.: 112. 1818, nom. illeg., non Capsicumconicum Lam. (1794). Type. “In plantationibus” (no specimens cited) [Guyana]. Rio Essequebo, Sept, *E.K. Rodschied 30* (lectotype, designated here: GOET [GOET003418]).
Capsicum minimum Roxb., Fl. Ind., ed. Carey & Wall. 2: 261. 1824, nom. illeg., not C.minimum Mill. (1768). Type. India. “East India” (no specimens cited), 18 Dec 1814, W. Roxburgh s.n. [Wallich Cat. No. 2641A) (lectotype, designated here: K [K001116724]).
Capsicum fastigiatum Blume, Bijdr. Fl. Ned. Ind. 13: 705. 1826. Type. “Crescit: in hortis et locis incultis … *Nomen*: Tjabe rawiet” [Indonesia]. Java, C.L. Blume s.n. (lectotype, designated here: L [L 0003564, acc. # 202560]).
Capsicum indicum Dierb. var. conoide (Mill.) Dierb., Arch. Apotheker-Vereins Nördl. Teutschl. 30(1): 28. 1829. Type. Based on Capsicumconoide Mill.
Capsicum indicum Dierb. var. berberideum Dierb., Arch. Apotheker-Vereins Nördl. Teutschl. 30(1): 29. 1829. Type. Based on Capsicumfrutescens Mill. (= C.frutescens L.)
Capsicum baccatum Vell., Fl. Flumin.: 60. 1829 (“1825”); Fl. Flumin. Icon. 2: t. 3. 1831 (“1827”), nom. illeg., non Capsicumbaccatum L. (1753). Type. Brazil. [Rio de Janeiro]: “Sponte crescit, et colitur hortis” (lectotype, designated by Knapp et al. 2015, pg. 824: [illustration] Original parchment plate of Flora Fluminensis in the Manuscript Section of the Biblioteca Nacional, Rio de Janeiro [cat. no.: mss1198651_006] and later published in Vellozo, Fl. Flumin. Icon. 2: t. 3. 1831).
Capsicum conoide Mill. var. sulcatum Fingerh., Monogr. Capsic.: 15. 1832. Type. No locality cited (no specimens cited; lectotype, designated here: [illustration] Fingerhuth, Monogr. Capsic. Tab. III c. 1832]).
Capsicum conoide Mill. var. chordale Fingerh., Monogr. Capsic.: 15. 1832. Type. “Crescit in America et Indiis”; no specimens cited; lectotype, designated here: [illustration] Fingerhuth, Monogr. Capsic. Tab. III d. 1832]).
Capsicum flexuosum Sendtn. var. perrottetti Dunal, Prodr. [A. P. de Candolle] 13(1): 413. 1852. Type. French Guiana. Sin. loc., 1820, *S. Perrottet 218* (holotype: G-DC [G00131855]).
Capsicum frutescens L. var. multilobatum Dunal, Prodr. [A. P. de Candolle] 13(1): 413. 1852. Type. No locality indicated, possibly from plants in cultivation (holotype: G-DC [G00131856]).
Capsicum conicum G.Mey. var. orientale Dunal, Prodr. [A. P. de Candolle] 13(1): 415. 1852. Type. Sudan. [Kurdufan] “ad pagum Cordofan in Milbes”, 4 Dec 1839, *G.C.T. Kotschy 292* (lectotype, designated here: G-DC [G00131905]; isolectotypes: BM [BM001016438, BM001016449], E [E00687052], G [G00442768], LE).).
Capsicum conicum G.Mey. [unranked “variat”] latifolium Dunal, Prodr. [A. P. de Candolle] 13(1): 415. 1852. Type. Sudan. “Senaar”, 1831, G. Acerbi s.n. (lectotype, designated here: G-DC [G00131885]).
Capsicum conicum G.Mey. [unranked “variat”] angustifolium Dunal, Prodr. [A. P. de Candolle] 13(1): 415. 1852. Type. Oman. Muscat “in horto Mascate”, *P.M.R. Aucher-Eloy 5039* (lectotype, designated here: G [G00390279]; isolectotype: G [G00390280]).
Capsicum crispum Dunal, Prodr. [A. P. de Candolle] 13(1): 415. 1852. Type. Republic of Mauritius. “Cult. au Jardin des Pamplemousses”, 1839, L. Bouton s.n. (holotype: G-DC [G00131904]; isotype: MPU [MPU023045]).
Capsicum crispum Dunal var. piper-rabiosum Dunal, Prodr. [A. P. de Candolle] 13(1): 416. 1852. Type. France. Île de Bourbon [= Réunion Island], 1821, Anonymous s.n. (holotype: G-DC [G00131903]; isotype: MPU [MPU023044]).
Capsicum conoide Mill. var. oblongoconicum Dunal, Prodr. [A. P. de Candolle] 13(1): 415. 1852. Type. No locality cited (no specimens cited; lectotype designated here: [illustration] Fingerhuth, Monogr. Capsic. Tab. III, b. 1832).
Capsicum curvipes Dunal, Prodr. [A. P. de Candolle] 13(1): 423. 1852. Type: French Guiana. “In Guianâ (h. Moric.)”, Anonymous s.n. (holotype: G [G00390275]; isotype: MPU [MPU023048].
Capsicum annuum L. var. frutescens (L.) Kuntze, Revis. Gen. Pl. 2: 449. 1891. Type: Based on Capsicumfrutescens L.
Capsicum annuum L. var. conicum (G.Mey.) Alef., Landw. Fl.: 132. 1866. Type: Based on Capsicumconicum G.Mey.
Capsicum annuum L. var. subconicum Alef., Landw. Fl.: 133. 1866. Type: Based on Capsicumconoide Mill.
Capsicum annuum L. var. conoide (Mill.) Irish, Rep. (Annual) Missouri Bot. Gard. 9: 65. 1898, as “conoides”. Type: Based on Capsicumconoide Mill.
Capsicum frutescens L. var. conoide (Mill.) L.H.Bailey, Gentes Herbarum 1: 129. 1923. Type: Based on Capsicumconoide Mill.
Capsicum annuum L. var. parvo-acuminatum Makino, J. Jap. Bot. 3(8): 30. 1926. Type. Cultivated in Japan “Hab. JAPAN, cultivated” (no specimens cited, no original material located).
Capsicum frutescens L. var. queenslandicum Domin, Biblioth. Bot. 89: 572. 1928. Type: Australia. Queensland: “Nordost-Queensland: Harweys Creek”, Jan 1910, *K. Domin 8247* (holotype: PR [acc. # 530853]).
Capsicum annuum L. var. oblongo-conicum (Dunal) Cufod., Bull. Jard. Bot. État Bruxelles 33 (Suppl.): 860. 1963. Type: Based on CapsicumconoideMill.var.oblongo-conicum Dunal.

#### Type.

“Habitat in India” Herb. A. van Royen n. 908.244–150 (lectotype, designated by Heiser and Pickersgill 1969, pg. 280: L [L 0053043, acc. # 902560, branch in the lower half of the sheet]).

#### Description.

Low subshrubs or shrubs, herbaceous or woody at the base, 0.3–1.5 (–2.5) m tall, much branched from near the base. Young stems slightly angled, fragile, green or purple or green with purple ridges, glabrous to sparsely pubescent with simple, uniseriate, 4–9 (–11)-celled, eglandular trichomes 0.3–1.4 mm long; nodes solid, green or purple; bark of older stems brown; lenticels absent. Sympodial units difoliate, the leaves geminate; leaf pair more or less similar in size and shape. Leaves membranous, concolorous or slightly discolorous, glabrescent or glabrous on both surfaces, sometimes with eglandular trichomes on the main veins or a tuft of trichomes in the vein axils abaxially; blades of all leaves (4–) 4.4–8.2 (–12.5) cm long, 2–4.5 (–6) cm wide, ovate or narrowly elliptic, the major veins 5–7 on each side of mid-vein, the base asymmetric, cuneate or attenuate, the margins entire, the apex acuminate to long-acuminate; petioles 0.5–3 cm, glabrescent or glabrous. Inflorescences axillary, 2–4 (–5) flowers per axil, rarely flowers solitary; flowering pedicels 9–30 (–40) mm long, erect, geniculate at anthesis, glabrous or sparsely pubescent; the eglandular trichomes minute, antrorse; pedices scars inconspicuous. Buds ovoid, cream or greenish-white. Flowers 5–7-merous, spreading or pendent. Calyx 1.2–2.5 mm long, cup-shaped, thick, green, glabrous or sparsely pubescent, the calyx appendages absent or five, ca. 0.5 mm long. Corolla 3.75–6.5 mm long, 5–15 mm in diameter, usually dull white or greenish-white outside and within, stellate with interpetalar membrane, lobed nearly the halfway to the base, pubescent adaxially with small glandular trichomes (stalk 1–2-celled; head unicellular) in the throat and base of the lobes, glabrous abaxially, the tube 3–4 mm long, the lobes 3–5 mm long, 2–3.5 mm wide, the margins finely ciliate, the tips acute, papillate. Stamens five (–seven), equal; filaments 1–1.5 mm long, cream or purple, inserted on the corolla 1–1.6 mm from the base, with auricles fused to the corolla at the point of insertion; anthers 1.5–2.85 mm long, ellipsoid, bluish-grey or purplish or (rarely) dark green or yellow, connivent at anthesis. Gynoecium with ovary 2.5–4 mm long, 1.3–1.8 mm in diameter, oblong-ovoid, pale green; ovules more than two per locule; nectary ca. 0.3 mm tall; styles homomorphic, 3–4 mm, exserted 1.5–2 mm beyond the anthers, cream or purple, cylindrical; stigma 0.1 mm long, 0.25 mm wide, discoid, light green. Berry usually 9–30 (–60) mm long, 4–12 (–15) mm in diameter, usually elongate and narrowly triangular, with the apex pointed or blunt and the base narrowed, usually green and yellowish-green when immature with transitions to yellow, orange-red, orange-yellow, orange to red at maturity, deciduous or persistent, pungent, rarely non-pungent, pericarp thick and opaque, with giant cells (endocarp alveolate); stone cells absent; fruiting pedicels 16–35 (–50) mm long, usually erect, sometimes pendent, terete to strongly angled, widened distally, green; fruiting calyx (3–) 4–6 (–7) mm in diameter, persistent, somewhat accrescent, deeply cup-shaped, without an evident constriction in its base and the junction with the pedicel, sometimes the margin ripped, green. Seeds 10–52 per fruit, 2–4 mm long, 2.2–3.3 (–4) mm wide, C-shaped to subglobose, pale yellow, the seed coat smooth to obscurely reticulate (SM), reticulate-cerebelloid (SEM), the cells irregular in shape, the lateral walls slightly to strongly sinuate; embryo imbricate.

**Figure 67.**
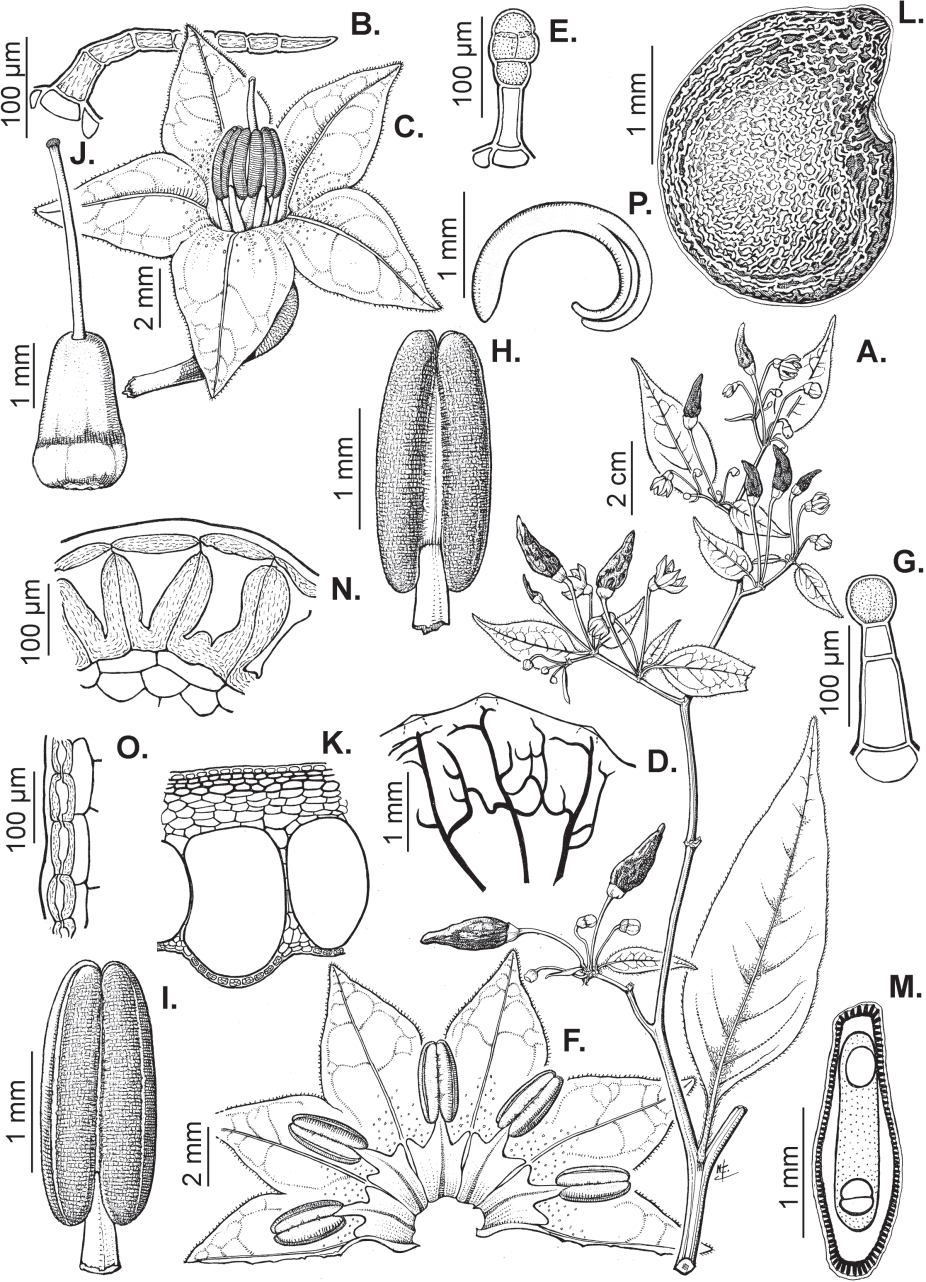
*Capsicumfrutescens***A** reproductive branch **B** eglandular trichome of the leaf **C** flower **D** section of the calyx showing the venation **E** glandular trichome of the abaxial surface of the calyx **F** opened corolla **G** glandular trichome of the abaxial surface of the corolla **H, I** anthers, dorsal and ventral views, respectively **J** gynoecium **K** anatomical detail of the pericarp (note the giant cells in the mesocarp) **L** seed **M** seed, in cross section **N** structure of seed coat at the seed margin **O** structure of seed coat at the seed body **P** embryo. From *Hunziker 25489.* Drawn by N. de Flury.

**Figure 68.**
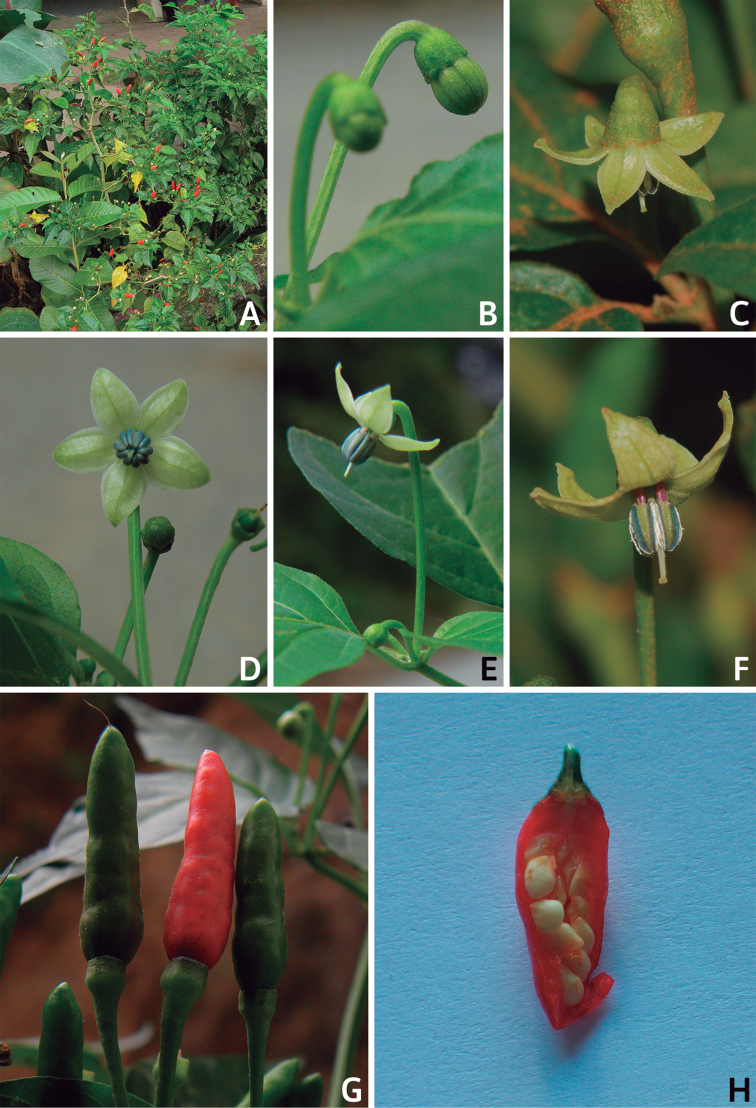
*Capsicumfrutescens***A** plant **B** flower buds **C** flower showing the calyx **D** flower, in lateral view **E** flower, in front view (note the connivent anthers) **F** flower after anthers dehiscence **G** immature (green) and mature (red) fruits **H** fruit, in longitudinal section showing the seeds **A, B, E–G** from *Barboza & Leiva González 4829***C, D, H** from *Barboza et al. 2041*. Photos by G.E. Barboza.

#### Distribution.

**Figure 69.**
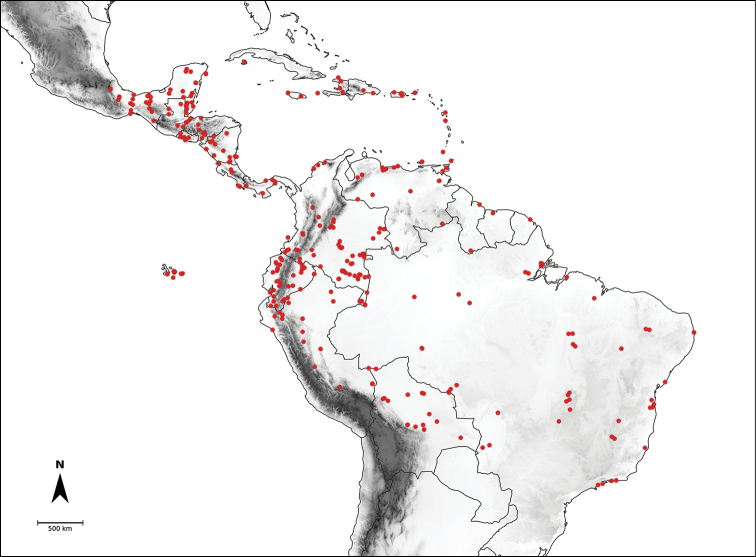
Distribution of *C.frutescens*.

*Capsicumfrutescens* has a broad distribution in lowland tropical America (Fig. 69), ranging from Brazil to Central America and the West Indies. It is not found in Chile, Argentina and Uruguay in the field, but it is sold in local markets (Peralta and Liverotti 2018). The species has increased its worldwide distribution more as a weed than as a cultivated plant. In the Old World, it has been introduced and established as a weedy component or it has escaped from cultivation into the flora of Papua New Guinea (Pickersgill 1989), Nepal (Baral and Bosland 2002), Philippines (Yamamoto and Nawata 2009) and Africa. It is cultivated in many countries around the world (Bosland and Votava 2000; Yamamoto and Nawata 2005, 2009), especially in the United States of America (Louisiana), where the cultivar Tabasco is grown commercially (Pickersgill 1971; DeWitt and Bosland 1997) and in Brazil, where the cultivar Malagueta is very popular (Bosland and Votava 2000).

#### Ecology.

*Capsicumfrutescens* grows in low semi-deciduous forests or in disturbed areas and agricultural clearings; it is commonly cultivated near homesites and in the chakras of many local communities, between 10 and 1,500 (2,000) m elevation.

#### Phenology.

Flowering and fruiting all year in different parts of its range.

#### Chromosome number.

2*n* = 2x = 24 (Pickersgill 1977, Moscone et al. 1996, 2007).

#### Common names.

**Argentina**: Tabasco (Salta, *Hunziker 25489*); **Antilles**: Bird pepper (Pickersgill 1984); **Belize**: Bird pepper (Cayo, *Atha et al. 1022*), Chile (Cayo, *Balick et al. 2272*), Chili del monte (Cayo, *Arvigo et al. 148*); **Bolivia**: Ají (Beni, *Williams 933*), Arivivi (Santa Cruz: *Krapovickas & Schinini 36348*), Aribibi chico (La Paz, *Williams 764*), Aribibi silvestre (Beni, *Scolnik & Luti 672*); **Brazil**: Malagueta (Amazonas, *Hill et al. 12996*; Bahia, *Thomas et al. 9080*; Goiás, *Vieira 709*; Mato Grosso do Sul, *Pott et al. 5562*; Piauí, *Pickersgill RU72-140*; Rio Grande do Norte, *Pickersgill RU72-357*; Rondonia, *Walter et al. 581*), Malaguetinha, malaguetão (Roraima, Barbosa et al. 2006), Olho-de-caranguejo (Paraná, *Leitão s.n.*), Pimenta camari (Minas Gerais, *Grandi 261*), Pimenta malaguêta (Amazonas, *Hill 12996*; Bahia, *André et al. 6863*; Espírito Santo, *Folli 2995*; Mato Grosso do Sul, *Bortolotto & Rodrigues B-615*; Minas Gerais, *Pereira 3219*; Paraíba, *Agra 625*; Tocantins, *Eiten & Eiten 10140*), Pimenta malaguetinha (Amapá, *Pereira & Severino 1855*), Pimenta malaguetão (Amapá, *Pereira et al. 1836*), Pimenta malagueta doce (Rondonia, *Walter et al. 575*); **Colombia**: Ají (Amazonas, *Henao 167*; Bolívar, *Bro. Heriberto 231*; Guainía, *Triana 18*; Magdalena, *de Romero 72*; Vaupes, *Zarucchi 2173*), Chivatillo (Nariño, *de Benavides 4692*), Ají Ajuja (Guainía, *Marín & Rodríguez 508*), Ají clavito (Amazonas, *Torres & Rodríguez 2020*), Ají chivatillo (Nariño, *Caballero 40*), Ají churere (Guainía, *Marín & Rodríguez 509*), Ají malagueta (Amazonas, *Cárdenas et al. 9441*), Aji pequeñito (Caquetá, *Cárdenas 9307*; Guainía, *Rodriguez 2*), Ají largo (Bolívar, *Espina 586*), Ají Quiñapira (Guaviare, *Rodríguez & Coy s.n.*), Ají de ajoja (Vaupés, *Rodríguez 112*), Ají de blancos (Amazonas, *La Rotta 273*), Ají diente, chiche (Amazonas, *Cárdenas et al. 9413*), Ají pico de pájaro (Bolívar, *Espina 571*), Diente de chucha (Amazonas, *Cárdenas et al. 9412*; Caquetá, *Cárdenas et al. 9325*); **Costa Rica**: Chili (Puntarenas,*Tonduz 7255*); **Ecuador**: Ají (Esmeralda, *Yañez et al. 1407*; Pichincha, *Kvist 40201*), Ají chivatillo (Esmeralda, *Acosta Solías 13937*; Pichincha, *Acosta Solís 13927*), Ají clavo, ají gallinazo (El Oro, *van den Eynden & Cueva 654*; Esmeraldas, *Cerón 14561*), Ají patate (Chimborazo, *Angulo 007*), Ají silvestre (Loja, *Vivar C. 4003*), Ají de ratón (Manabí, Deanna 53); **El Salvador**: Chiltepe (El Salvador, *Velasco 8925*), Chilpepe, Chile de zope (Sonsonate, *Standley 22237*), Chile chocolate (San Salvador, *Calderón 1198*), **Guatemala**: Chili (Izabal, *Standley* 25055; Peten, *Lundell 16480*), Chile largo (Izabal, *Standley 24297*), Chile chiltepe (Izabal, *Standley 23755*); **Haiti**: Piment-chien (Ile La Tortue, *Ekman s.n.*); **Honduras**: Chile bravo (Atlántida, *Standley 53392*), Pico de Pájaro (Morazán, *Molina R. 1177*); **Mexico**: Chilpaya o Chilayate (Veracruz, *Martínez A. 346*), Chile chocolate (Chiapas, *Matuda 17642*), Chile picapalo (Tabasco, *Becerril Pérez & Ortiz Cornejo 7*), Chile tabaquero (Oaxaca, *Alcocer & Morales s.n.*), Pico Paloma (Tabasco, *Ortega et al. 876*), Chile del monte (Yucatán, *Gaumer 864*), Chile pico paloma (Tabasco, *Alegría O. 47*), Diente de perro (Chiquimula, *Kufer 100*); **Panamá**: Ají (Balboa, *Standley 25504*); **Peru**: Arnaucho, Aji arnaucho (Peru, Dunal 1852), Malageta (Loreto, *Martin & Lau-Cam 1242*), Malaguete (San Martín, Libreros et al. 2013), Ají chuncho (Junín, *Smith s.n.*), Ají mono, Ají pinguita de mono (Lambayeque, *Llatas Quiroz 3452*), Mala guctí (Huánuco, *Schunke V. 1397*), Pipí de mono (Loreto, Libreros et al. 2013); **Trinidad and Tobago**: Bird pepper (Tobago, *Broadway 4498*; Trinidad, *Heiser C 259*); **United States of America**: Tabasco (Louisiana, *Meyer & Mazzeo 11988*); **Panamá**: Ají (Balboa, *Standley 26493*); **Venezuela**: Chirel (Margarita Island, *Smith-Davis Ac 1495*), Chirere (Carabobo, *Pittier 7909*), Aji pico pájaro (Margarita Island, *Smith-Davis Ac 1498*).

#### Indigenous names.

**Belize**: Mash-ík (Mayal, Cayo, *Balick et al. 2272*), Smash-ík (Cayo, *Balick et al. 2391*); **Bolivia**: Bido (Tacana, La Paz, *Williams 764*), Naris (Chiquitano, Santa Cruz, *Del Aguila et al. 662*); **Colombia**: Bee-a’ (Tucano, Amazonas, *Schultes & Cabrera 13046*), Biaá (Yucana, Amazonas, *Cárdenas 9405*), Cog (Puinave, Guainía, *Triana 18*), Eviviaa (Tucano, Guainía, *Marín & Rodríguez 508*), Fekorai (Huitoto-Mɨnɨka, Amazonas, *Henao 167*), Ferocoi (Huitoto, Caquetá, *Cárdenas et al. 9300*), Ichiriay (Matapi, Amazonas, *Cárdenas et al. 9413*), Jibirai (Caquetá, *Cárdenas 9345*), Jichiri (Yucuna, Amazonas, *Cárdenas et al. 9441*), Lehirihay (Yucuma, Caquetá, *Cárdenas et al. 9325*), Meniray (Huitoto, Caquetá, *Cárdenas 9307*), Mèe (Colona, Amazonas, *Torres & Rodríguez 2020*), Pidootú (And, Amazonas, *Castro & Andoke 608*), Pipitatu (And, Amazonas, *Castro & Andoke 606*), Pxrxtú (And, Amazonas, *Castro & Andoke 597*), Tsirrerreji (Sikuare guahibo, Guainía, *Rodriguez 2*), Viaa (Kuboes, Guainía, *Marín & Rodríguez 509*); **Ecuador**: Aatyu (Chapalaachi, Esmeraldas, *Yañez et al. 1407*), Ampy (Shuar, Zamora-Chinchipe, *Ortega et al. 51*), Chimidu dio (Cayapa, Esmeralda, *Kvist & E. Asanza* 40456), Ma pipi pia (Siona and Secoya Indians, Napo, *Vickers 208*), Ocoma (Cofán, Napo, *Cerón 194*), Panduchu (Achuar Jívaro, Pastaza, *Lewis et al. 14011*), Pía (Secoya, Sucumbíos, *Miranda-Moyano 225*), Pipetio (Cayapa, Esmeraldas, *Kvist 40565*), Pucuitape (Cayapa, Esmeraldas, *Kvist & Asanza 40356*), Sun’nyo pipi pia (Siona & Secoya Indians, Napo, *Vickers 226*), Tun (Colorado, Pichincha, *Kvist 40201*), Uchu (Pastaza, *Lewis et al. 14011*); **Guatemala**: Ich (Ch’orti, Chiquimula, *Kufer 100*); **Paraguay**: Ki’ï (Central, *Schinini & Bordas 24511*); **Peru**: Shunaru’ca (Chayahuita, Loreto, *Odonne 641*), Tsukagka (Amazonas, *Salaün 177*), Yancuru’ca (Chayahuita, Loreto, *Odonne 664*). **Mexico**: Guiiña xcuuchu (Zapoteco, Oaxaca, *Sánchez L. et al. 953*).

#### Uses.

*Capsicumfrutescens* has medicinal, ornamental, ritual and food applications. The fresh or cooked fruits are an important component of daily meals in India (soups, sauces, jams, pepper powder). The famous hot Tabasco sauce is made from tabasco peppers, a varietal of *C.frutescens*. In the Brazilian Amazon, herbarium labels report plants with sweet fruits (*Walter et al. 575*) that are used for decorating dishes and salads and in smoking rituals (Barbosa et al. 2002, 2006). The leaves and fruits have many reported medicinal uses (see Table 3).

#### Preliminary conservation assessment.

EOO (19,757,841 km^2^); AOO (1,336 km^2^). *Capsicumfrutescens* is a widespread cultivated species across the Americas and can be assigned the Least Concern (LC) status.

#### Discussion.

*Capsicumfrutescens* belongs to the Annuum clade (Carrizo García et al. 2016); it is a shrubby plant with 2–5 flowers per node, dull white or greenish-white corollas, bluish-grey or purplish anthers, upright elongate and narrowly triangular pungent fruits, a deeply cup-shaped fruiting calyx enclosing the narrowed fruit base and smooth, pale yellow seeds. It does not have as many cultivars as *C.annuum* and *C.chinense* (Bosland and Votava 2000).

Morphoagronomic and molecular characterisation studies have been carried out on many accessions of *C.frutescens* from different germplasm banks (Carvalho et al. 2017; Tripodi and Greco 2018; Brilhante et al. 2021), as well as on commercial crops (Peñuela et al. 2020), with differing results as to the most useful morphological diagnostic descriptors for the species. Carvalho et al. (2017) identified three morphological patterns of fruits: malagueta type (the most common type with detailed characterisation), Tabasco type and malaquetinha type, based on qualitative and quantitative morphological descriptors and SSR markers in 103 accessions of *C.frutescens* from Brazil. Molecular results in the samae study recovered six groups with genetic variability between and within each group.

Many authors have recognised close affinities between *C.annuum*, *C.frutescens* and *C.chinense* (e.g. Jensen et al. 1979; McLeod et al. 1983b; Prince et al. 1995; Baral and Bosland 2004; Ibiza et al. 2012; Carrizo García et al. 2016; D’Agostino et al. 2018). Together, these species constitute the *Capsicumannuum* primary gene pool (van Zonneveld et al. 2015).

Flowering specimens of *C.frutescens* and *C.chinense* are sometimes difficult to distinguish in herbaria when fruits are missing. Venation of flowering calyx is a good distinguishing feature between both species since, in *C.frutescens*, the main nerves are often completely immersed in the calyx surface (Fig. 68C, G), while in *C.chinense*, they protrude conspicuously from the calyx (Fig. 47C, J, L) or may protrude distally. In fruit, the distinction between these two taxa is remarkable; *C.frutescens* has deeply cup-shaped calyx enclosing the narrowed base of the elongate fruit (Fig. 68G, H), the pedicels are usually erect and a constriction at the junction calyx-pedicel is totally absent. *Capsicumchinense* has a flat discoid calyx appressed or reflexed to the fruit base, the pedicels are usually pendent and a noticeable circular constriction at the junction with the fruiting calyx is present.

*Capsicumconoide* was coined by Miller (1768) for shrubby plants with conical, erect red fruits “about one half inch” long that he grew from seeds received from Antigua under the name “Hen Pepper”. The identity of *C.conoide* has been controversial in literature. Dierbach (1829) considered *C.conoide* an infraspecific category of *C.indicum* and it was submerged under C.annuumvar.glabriusculum by D’Arcy and Eshbaugh (1974), due to the size of the fruit. Fingerhuth (1832) accepted *C.conoide*, added two varietal names (C.conoidevar.sulcatumandvar.chordale) and provided illustrations for all of them. Irish (1898) recognised *C.conoide* as a variety of *C.annuum*, assuming a wide concept for this latter taxon with the inclusion of garden cultivars currently accepted in different species (Heiser and Pickersgill 1975). We found no original material for *C.conoide*, but we interpret this name (and Fingerhuth’s varieties) as synonyms of *C.frutescens*, based on the descriptions.

For *C.conicum*, Georg Meyer (1818) cited no specimens in his “Florae Essequeboensis” (Guyana). At GOET, we found original material in Herbarium Meyer (*Rodschied 30*) with ‘Capsicumfrutescens’ written in Rodschied’s hand. This matches the text stated in the protologue of *C.conicum*. We select this sheet (GOET003418) as the lectotype.

When Roxburgh used the name *Capsicumminimum* for the first time (Roxburgh 1814: 17), he provided no descriptions, but he stated that the plant was from India. Later, the name was validly published (Roxburgh 1824), accompanied by a short description and the vernacular names “East Indian Bird chilly or Cayenne-pepper capsicum”. In the Wallich Herbarium at Kew, there are two sheets, one from Francis Buchanan-Hamilton’s Herbarium (K001116724) labelled with the Wallich catalogue number 2641A and the other from “hort. Bot. Calcutta” with the Wallich catalogue number 2641B (K001116725). These are not duplicates (see discussion of Wallich’s catalogue numbering system at http://wallich.rbge.info/). The sheet from Buchanan-Hamilton’s Herbarium (K001116724) has several labels, the upper left of which is in Roxburgh’s handwriting (see Forman 1997), stating “Capsicumminimum/Botanical garden 18 Decr 1814”. The other sheet in the Wallich Herbarium (K001116725) has no specific label with either the scientific name or place of origin; this sheet is not likely to be a duplicate and should not be considered original material. A sheet in G-DC (G00131883) is a good match for the centre stem on Wallich Cat. No. 2461B and is potentially a duplicate. We consider the sheet from the Buchanan-Hamilton herbarium (K00116724) to be the only authentic original material that we have seen and we here designate it the lectotype for *C.minimum*.

The description of *C.fastigiatum* (Blume 1826) cited no herbarium material. A specimen at L collected by Blume in Java with the handwritten inscription *Capsicumfastigiatum* is here considered original material, since many of Blume’s collections are housed at L (Stafleu and Mennega 1993). Heiser and Pickersgill (1969) commented that they examined the lectotype of *C.fastigiatum*, probably alluding to the specimen at L, but they were not specific. We designate here the sheet in Leiden (L 0003564) (presumably the sheet referred to by others) as the lectotype for *C.fastigiatum*.

Capsicumconoidevar.sulcatumandvar.chordale were both coined by Fingerhuth (1832), with no specific herbarium material cited. However, illustrations were provided for each variety that depict small fruiting branches with the elongate fruit typical of *C.frutescens*. We have selected these illustrations as the lectotypes for both of these varietal names.

In describing C.frutescensvar.multilobatum, Dunal (1852) based its description on a specimen “v. s. in h. DC”, with no additional information. A sheet in G-DC (G00131856) has no locality label, but is annotated “Capsicumfrutescens β multilobatum” in Dunal’s hand; we interpret this as the holotype.

Dunal (1852) described an infraspecific category for *C.conicum* G. Mey. named β [var.] *orientale* that he distinguished by its woody branches and smaller leaves. Within it, he recognised two unranked categories (“Variat”, that is, it varies) *latifolium* and *angustifolium*, for which he cited specimens. The two specimens, cited for the unranked *latifolium* (*Kotschy 292* and *Acerbi s.n*.), are mounted on a same sheet and they unequivocally belong to *C.frutescens*, based on the shape of the fruit and morphology of the fruiting calyx. We select *Kotschy 292* (G00131905) as the lectotype for var. orientale, because it has duplicates in many herbaria. In order to avoid homotypy for [unranked “Variat”] *latifolium*, we select the other collection Dunal used for this name (*Acerbi s.n.* G00131885), mounted on the same sheet as the Kotschy collection, as the lectotype. For [unranked “Variat”] *angustifolium*, Dunal cited a collection made in Muscat by P.M.R. Aucher-Eloy in “h. Boiss.”; two sheets of *Aucher-Eloy 5039* are held in the general herbarium at G and we select the better of these (G00390279) as the lectotype.

In describing C.conoidevar.oblong-conicum, Dunal cited no herbarium material, but he referred to the Fingerhuth’s illustration “Tab 3, f. b” as the element he has seen. Therefore, he is clearly differentiating this illustration from *C.conoides* and it is the original material for the varietal name. We select this illustration as the lectotype.

#### Specimens examined.

See Suppl. material 4: Appendix 4.

### ﻿20. Capsicum galapagoense

Taxon classificationPlantaeSolanalesSolanaceae

Hunz., Huitième Congr. Int. Bot. Paris, Comptes Rend. Séances Rapp. & Commun. 1954, sect.4: 73. 1956.

B3598069-E963-575D-812A-A2754EB076F0

Fig. 70

Brachistus pubescens Stewart, Proc. Calif. Acad. Sci., ser. 4, 1: 137. 1911. Type. Ecuador. Galápagos: Albemarle Island [= Isabela], Villamil, 450–600 ft elev., 3 Jan 1906, *A. Stewart 3352* (lectotype, designated by Barboza 2011, pg. 26: CAS [0001526, acc. # 1244]; isolectotypes: B [B10-0176773], GH [00076931], US [01108008, acc. # 921602]).
Capsicum galapagense Heiser & P.G.Sm., Brittonia 10: 200. 1958, nom. illeg., not Capsicumgalapagoense Hunz. (1956). Type. Based on Brachistuspubescens Stewart

#### Type.

Based on *Brachistuspubescens* Stewart.

#### Description.

Erect low shrubs, 0.5–1 m tall, much branched from near the base. Young stems terete to slightly 2–3-angled, fragile, densely white or yellowish-white (when dry) pubescent, with spreading, simple, uniseriate, 3–7-celled, eglandular trichomes 0.3–1.5 mm long; nodes green; bark of older stems brown, glabrescent; lenticels absent. Sympodial units difoliate, the leaves geminate; leaf pair unequal in size, similar in shape. Leaves membranous, slightly discolorous, densely pubescent on both surfaces, especially on the veins, with similar eglandular trichomes to the stems, plus sparse small glandular trichomes and occasionally furcate eglandular trichomes; blades of major leaves 2–6 (–8) cm long, 0.9–2.8 (–3.5) cm wide, ovate, the major veins 3–5 on each side of mid-vein, the base cuneate or truncate and asymmetric, the margins entire, the apex slightly acuminate; petioles 0.5–1.5 cm long, densely pubescent; blades of minor leaves 1.1–1.95 cm long, 0.3–0.7 cm wide, ovate, the major veins 3–4 on each side of mid-vein, the base cuneate or truncate and asymmetric, the margins entire, the apex acute or obtuse; petioles 0.2–0.7 cm long, densely pubescent. Inflorescences axillary, 1–2-flowered; flowering pedicels 6–8 mm long, short, angled, erect to slightly spreading, geniculate at anthesis, densely pubescent, the eglandular trichomes long, spreading; pedicel scars conspicuous. Buds ovoid, white. Flowers 5-merous. Calyx 1.3–1.6 (–2) mm long, 1.8–2 mm wide, cup-shaped, circular in outline, thin, green, densely pubescent with the same trichomes as stems and leaves, the calyx appendages absent. Corolla 4–5 mm long, ca. 6 mm in diameter, entirely white or dull white outside and within, stellate without interpetalar membrane, lobed nearly halfway to the base, the tube 2–2.5 mm long, pubescent adaxially with sparse short glandular trichomes (stalk unicellular; head globose, unicellular), glabrous abaxially, the lobes 2.3–2.5 mm long, ca. 2.5 mm wide, ovate, spreading, glabrous adaxially and abaxially, the margins papillate, the tips acute, papillate. Stamens five, equal; filaments 1–1.3 mm long, white, inserted on the corolla ca. 0.7–1 mm from the base, with auricles fused to the corolla at point of insertion; anthers 0.9–1.3 mm long, ellipsoid, yellow, connivent at anthesis. Gynoeciumm with ovary 0.9–1.3 mm long, 0.8–1 mm in diameter, ovoid; ovules more than two per locule; nectary ca. 0.3 mm tall; styles homomorphic, 2.25–2.5 mm long, exserted 0.5–0.8 mm beyond the anthers, cylindrical, white; stigma 0.09 mm long, ca. 0.24 mm wide, discoid. Berry 5–7 mm in diameter, globose or somewhat ellipsoid, dark green when immature, red-orange or bright red at maturity, deciduous, pungent, the pericarp thick, opaque, with giant cells (endocarp alveolate); stone cells absent; fruiting pedicels 8–18 (–22) mm long, erect, slightly angled or terete, widened distally, green; fruiting calyx 2.8–4 mm in diameter, persistent, not accrescent, discoid, green. Seeds 8–9 per fruit, 3.5–4 mm long, 2.5–3 mm wide, flattened, C-shaped to reniform, pale yellow, the seed coat smooth (SM), cerebelloid (SEM), the cells irregular in seed body and polygonal at superior margin, the lateral walls sinuate in seed body, wavy at margins; embryo annular or imbricate.

#### Distribution.

*Capsicumgalapagoense* is endemic to the Galápagos Archipelago of Ecuador (Islands of Bartolomé, Fernandina, Isabela, Santa Cruz, Santiago, Rabida and Pinta) (Fig. 66).

#### Ecology.

*Capsicumgalapagoense* grows in shade under shrubs or small trees in the arid lowlands to moist uplands of the islands (*Pisonia*, *Scalesia* or *Croton* forests); 15–900 m elevation.

#### Phenology.

Flowering and fruiting from December to August and likely all year.

#### Chromosome number.

*n* = 12 (Heiser and Smith 1958); 2*n* = 2x = 24 (Pickersgill 1977; Moscone et al. 2007).

#### Common names.

None recorded.

#### Uses.

None recorded.

#### Preliminary conservation assessment.

EOO (ca. 8000 km^2^); AOO (56 km^2^). Based on the number of locations and the area of occupancy, these suggest an Endangered (EN; B2ab(iii)) category for *Capsicumgalapagoense*. The threats in the Galápagos, such as land-use activities, introduced alien plants in the inhabited islands and the population explosion of goats and pigs in the uninhabited ones (e.g. Islas Bartolomé, Fernandina or Rabida), have caused serious ecological problems that need to be addressed urgently to protect the rare and endemic species of the islands (Adsersen 1989).

#### Discussion.

**Figure 70.**
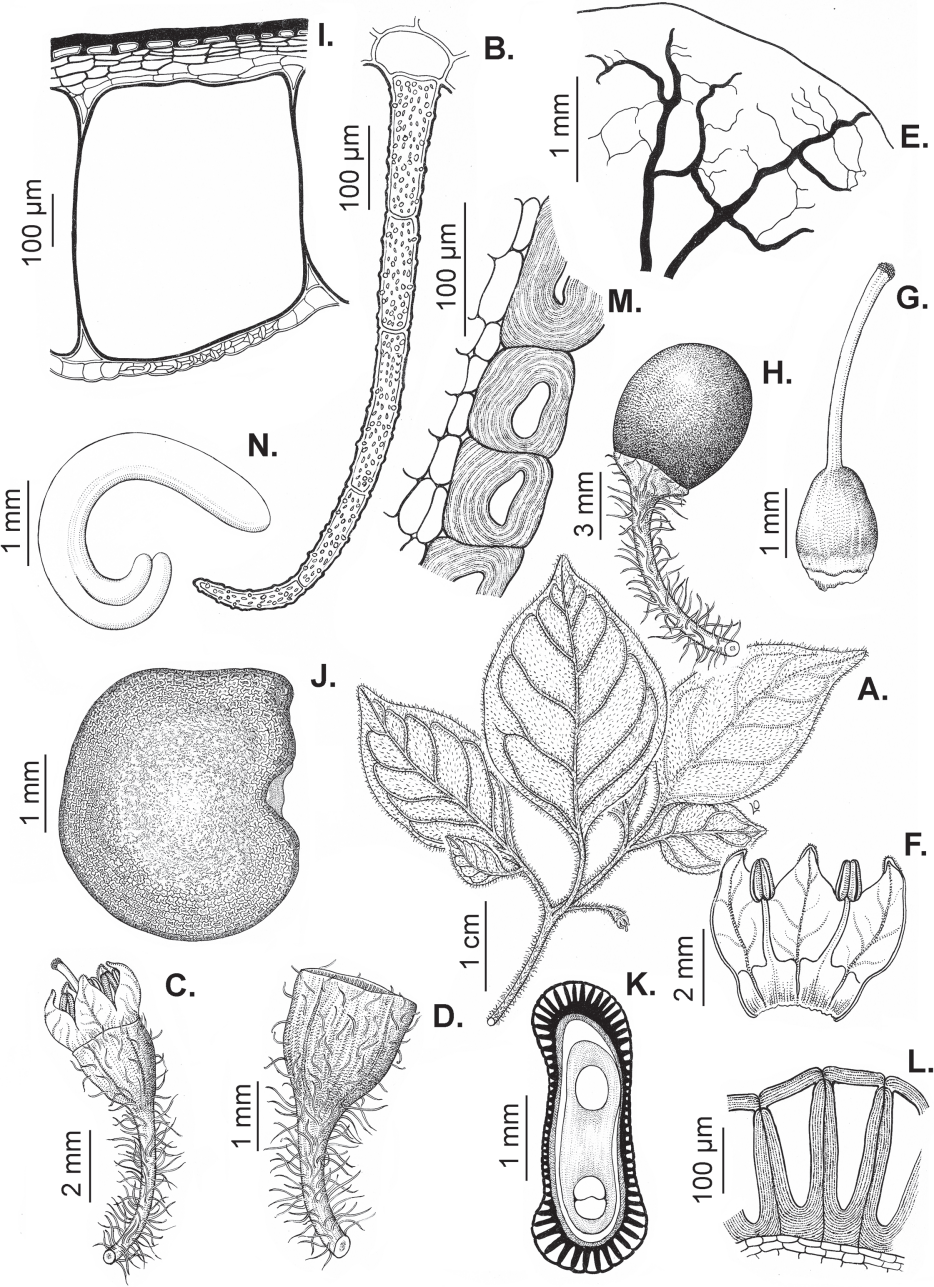
*Capsicumgalapagoense***A** flowering branch **B** eglandular trichome of the leaf **C** flower **D** calyx **E** section of the calyx showing the venation **F** sector of opened corolla **G** gynoecium **H** fruit **I** anatomical detail of the pericarp (note the giant cell in the mesocarp) **J** seed **K** seed, in cross section **L** structure of seed coat at the seed margin **M** structure of seed coat at the seed body **N** embryo **A, B** from *Stewart 3351***C–G** from *Taylor G11***H–N** from *Schimpf 20*. Drawn by L. Ochoa.

*Capsicumgalapagoense* belongs to the Annuum clade (Carrizo García et al. 2016). It is the only wild native species found in the Galapagos Islands. It is unique in having dense pubescence throughout and the smallest flowers in the genus (Fig. 70A, C). It is superficially similar to C.annuumvar.glabriusculum in its habit, lack of calyx appendages (Fig. 70C, D), white corollas, small, red, pungent fruits and pale yellow seeds. The two taxa are not sympatric. Three other *Capsicum* species grow in the Galápagos (McMullen 1999): 1) *C.frutescens* is cultivated or escaped from cultivation and can be distinguished from *C.galapagoense* in its sparse general pubescence, larger greenish-white to greenish-yellow corollas and elongate, conical fruits; 2) the commonly cultivated C.annuumvar.annuum can be distinguished by its larger white corollas and diversely-shaped fruits; and 3) C.baccatumvar.pendulum distinguished by its white corollas with greenish-yellow spots within and large pendent fruits.

*Capsicumgalapagoense* is self-compatible and has been experimentally crossed with members of the Annuum clade (*C.annuum*, *C.chinense*, *C.frutescens*) and the Baccatum clade (*C.baccatum* wild, *C.rabenii*, *C.chacoense*); no incompatibilities were found in any direction. Based on this breeding evidence, there is the potential for hybridisation between *C.annuum* or *C.frutescens* and *C.galapagoense*, although McMullen (1999) considered this unlikely.

#### Specimens examined.

See Suppl. material 4: Appendix 4.

### ﻿21. Capsicum geminifolium

Taxon classificationPlantaeSolanalesSolanaceae

(Dammer) Hunz., Huitième Congr. Int. Bot. Paris, Comptes Rend. Séances Rapp. & Commun. sect.4: 73 (1954). 1956.

5331EFDD-1C37-517E-8B46-E27AB37AD079

Figs 71 , 72

Acnistus geminifolius Dammer, Bot. Jahrb. 36(4): 384. 1905. Type. Ecuador. Pichincha: “in silv. Monte Corazón”, Sep 1873, *P.L. Sodiro 114/84* (lectotype, designated by Barboza 2011, pg. 25: P [P00410128]; isolectotype: CORD [CORD00087960, fragment ex P]).
Capsicum scolnikianum Hunz., Kurtziana 1: 213. 1961. Type. Peru. Piura: Canchaque, en el camino de Piura a Huancabamba, 1200 m elev., 1 Dec 1948, *R. Scolnik 1389* (lectotype, designated here: CORD [CORD00006647]; isolectotype: CORD [CORD00006648]).

#### Type.

Based on *Acnistusgeminifolius* Dammer.

#### Description.

Erect shrubs or subshrubs, 1–4 (–6) m tall, with the main stem 1–1.5 cm in diameter at base, profusely branched above, rarely herbs 0.30–0.80 m, the branches sometimes scandent. Young stems angled, fragile, green, sometimes with purple ridges, glabrous or sparsely to moderately pubescent with spreading or antrorse, white to yellowish-white, simple, uniseriate, 4–6-celled, eglandular trichomes 0.3–0.6 mm long, sometimes sparse branched trichomes; nodes solid, green; bark of older stems dark brown or greyish, glabrous or moderately pubescent; lenticels abundant, light brown. Sympodial units difoliate, the leaves geminate; leaf pair markedly unequal in size and similar or dissimilar in shape. Leaves membranous, concolorous or discolorous, deep green above, pale green or purple below, with sparse or abundant, simple, eglandular trichomes 0.5–1.2 mm long adaxially and abaxially, sometimes also branched trichomes along mid-vein and occasionally a tuft of simple and branched trichomes in the main vein axils abaxially; blades of major leaves 4–13 cm long, 0.4–2.2 (–3.57) cm wide, elliptic to narrowly elliptic, sometimes ovate or falcate, the major veins 4–6 on each side of mid-vein, the base attenuate, somewhat asymmetric, the margins entire, the apex acuminate or long-acuminate; petioles 0.4–0.8 cm long, moderately pubescent or glabrescent with simple trichomes; blades of minor leaves 0.8–5 (–5.5) cm long, 0.4–1.5 (–2.7) cm wide, ovate or elliptic, the major veins 3–5 on each side of mid-vein, the base short attenuate, rarely asymmetric, the margins entire, the apex obtuse; petioles 0.1–0.5 cm, glabrescent. Inflorescences axillary, 2–5 (–6) flowers per axil, rarely flowers solitary; flowering pedicels (10–) 15–25 (–27) mm, thin, terete, pendent, non-geniculate at anthesis, green or purple-tinged, glabrous to moderately pubescent, the eglandular trichomes short or long, antrorse; pedicels scars inconspicuous. Buds ovoid, yellow or dark purple. Flowers 5-merous. Calyx 1.5–3 mm long, 2–2.5 mm wide, cup-shaped, thin or somewhat fleshy, green, greenish-purple or dark purple (nearly black), glabrescent to moderately pubescent with antrorse eglandular trichomes 0.3–0.7 mm long, the calyx appendages (2–) 3–5, 3–6.5 mm long, 0.3–0.6 mm wide, subequal, erect or spreading, subulate. Corolla 7–12 (–15) mm long, 13–18 mm in diameter, dull yellow or yellow with sparse to abundant maroon or purple spots within and outside or nearly completely purple outside, campanulate, stellate in outline, with a thin interpetalar membrane connecting the lobes in the proximal half, lobed nearly 1/3 of the way to the base, glabrous abaxially and adaxially, the tube 6–7 mm long, 8–10 mm in diameter, the lobes (3–) 5–8 mm long, (3–) 4.5–5.5 mm wide, ovate, erect or spreading at anthesis, the margins and tips papillate. Stamens five, equal; filaments 2–3 mm long, pale green, white or lilac, glabrous, inserted on the corolla ca. 2 mm from the base, with auricles fused to the corolla at the point of insertion; anthers (1.6–) 2–2.5 mm long, ovoid, cream, yellow or rarely white, slightly connivent at anthesis. Gynoecium with ovary (1.5–) 1.8–2 mm long, ca. 1.5 mm in diameter, light green or cream, ovoid; ovules more than two per locule; nectary 0.5–0.8 mm tall, cream; styles homomorphic, 5–7 mm long, exserted 0.8–1.3 mm beyond the anthers, slightly curved distally, cream, clavate; stigma 0.2–0.3 mm long, ca. 0.8 mm wide, usually discoid, sometimes slightly bilobed, light green. Berry 7–12 mm in diameter, globose or globose-depressed, light green when immature, pale orange or orange at maturity, deciduous, non-pungent, the pericarp thick, opaque, lacking giant cells (endocarp smooth); stone cells 1–5 or absent, subglobose or irregular, 1–1.5 mm in diameter; fruiting pedicels 18–30 mm long, pendent, terete or distally ribbed, scarce to strongly widened distally, green, greenish-purple or purple; fruiting calyx 4–5 mm in diameter, persistent, not accrescent, discoid, greenish-purple or green, the appendages 3–8 mm long, 1 mm wide, spreading or reflexed, green, greenish-purple or purple. Seeds (8–) 12–70 per fruit, 1.8–2.3 mm long, 1.3–1.5 mm wide, teardrop-shaped, black, the seed coat reticulate (SM and SEM), the cells polygonal or irregular in shape, the lateral walls straight or wavy; embryo annular.

#### Distribution.

*Capsicumgeminifolium* is widely distributed over north-western South America, from Colombia and Ecuador to central Peru (Fig. 66).

#### Ecology.

*Capsicumgeminifolium* grows in margins or interior of primary or secondary Andean to sub-Andean montane rain forests, somewhat exposed to light, at (100–) 950–3,500 m elevation.

#### Phenology.

Flowering and fruiting all year.

#### Chromosome number.

Not known.

#### Common names.

None recorded.

#### Uses.

None recorded.

#### Preliminary conservation assessment.

EOO (1,120,997.593 km^2^); AOO (380 km^2^). *Capsicumgeminifolium* is very common across its range and it is also found in many protected areas. We assign the status of Least Concern (LC).

#### Discussion.

*Capsicumgeminifolium* is a member of the Andean clade (Carrizo García et al. 2016, as *C.scolnikianum*; Barboza et al. 2019). It is recognised by its 2–5 somewhat fleshy calyx appendages and yellow corollas that may or may not have maroon or purple spots (Fig. 72E–I). It is commonly confused with the partially sympatric species *C.lycianthoides*, both in herbaria and literature. The long flowering pedicels (15–27 mm), campanulate (stellate in outline) corollas, membranous elliptic or narrowly elliptic leaves and general pubescence distinguish *C.geminifolium* from *C.lycianthoides*, which usually has shorter pedicels (8–15 mm), broadly campanulate (pentagonal in outline) corollas, large ovate to broadly ovate coriaceous leaves and is glabrous or very sparsely pubescent (Fig. 85).

**Figure 71.**
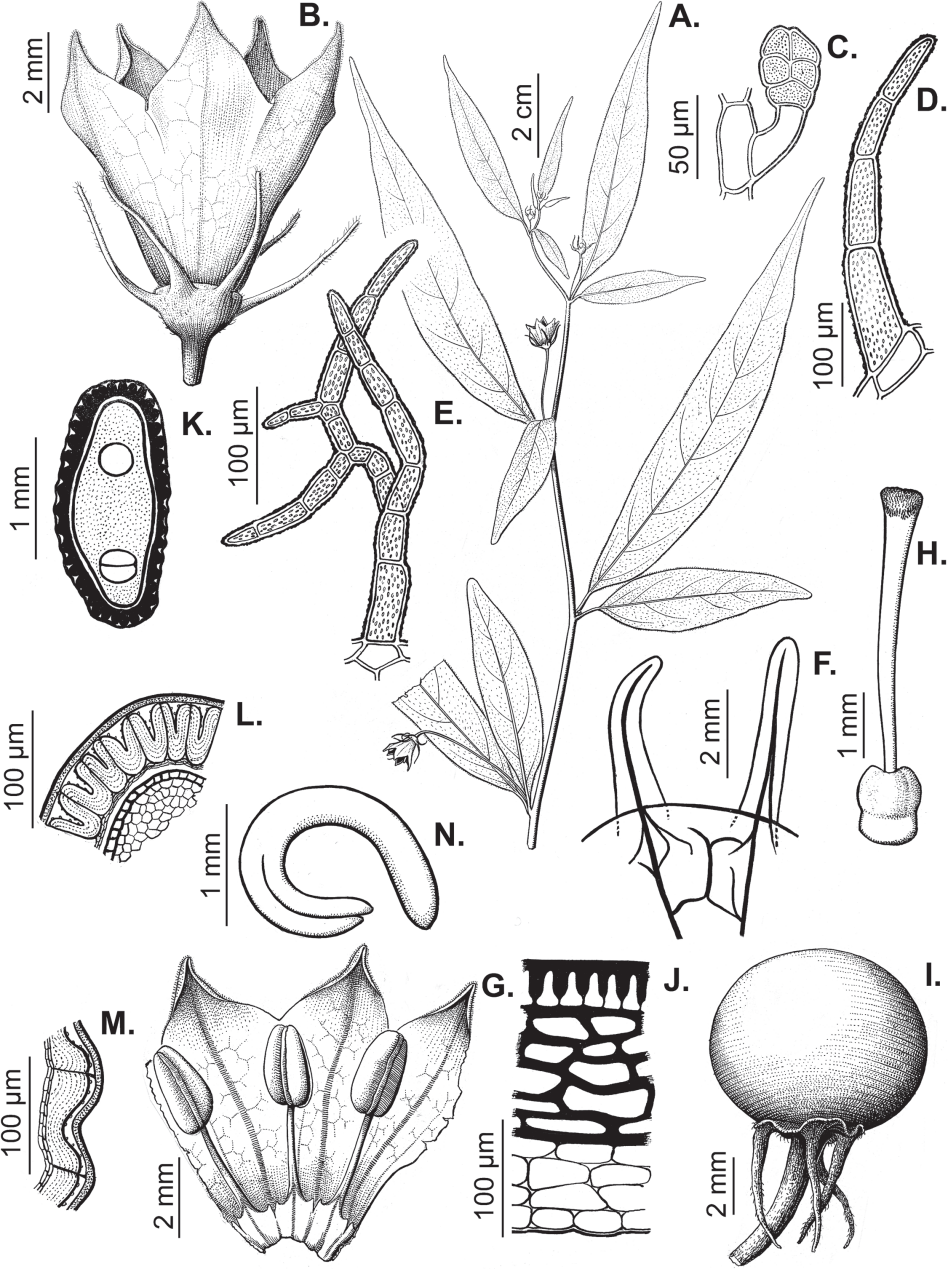
*Capsicumgeminifolium***A** flowering branch **B** flower **C** glandular trichome of the abaxial surface of the calyx **D** eglandular calyx of the leaf and calyx **E** branched trichome of the leaf and calyx **F** section of the calyx showing the venation **G** sector of opened corolla gynoecium **H** gynoecium **I** fruit **J** anatomical detail of the pericarp (note the absence of giant cells in the mesocarp) **K** seed, in cross section **L** structure of seed coat at the seed margin **M** structure of seed coat at the seed body **N** embryo. From *Scolnik 1389.* Drawn by L. Sánchez.

**Figure 72.**
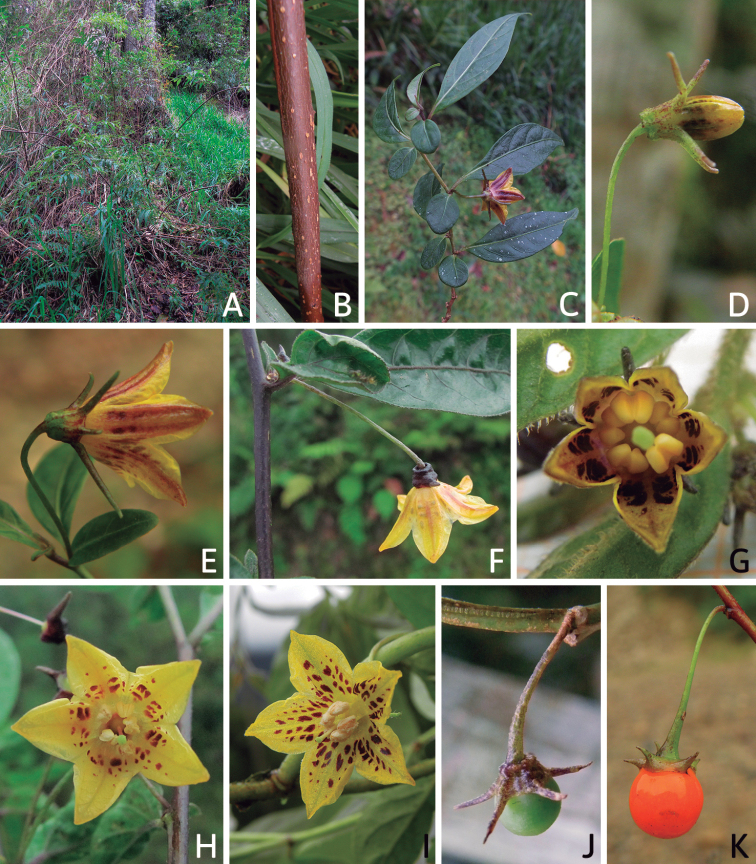
*Capsicumgeminifolium***A** plant **B** main stem with lenticels **C** flowering branch **D** flower bud **E** flower, in lateral view, with long calyx appendages **F** flower, with short calyx appendages **G** flower, in pre-anthesis **H, I** corollas, in front view, with different colouration patterns within **J** immature fruit **K** mature fruit **A, B, D, J** from *Barboza & Leiva González 4845***C, G** from *Deanna & Leiva González 3***E, I** from *Barboza & Leiva González 4852***F, H** from *Deanna & Leiva González 77***K** from *Orejuela R. 2688***A, B, D, E, I, J** photos by G.E. Barboza **C, F–H** photos by R. Deanna **K** photo by A. Orejuela.

When Hunziker (1961) described *C.scolnikianum*, he mentioned close morphological similarities with *C.geminifolium* and with other three species now recognised as members of the Andean clade (*C.hookerianum*, *C.lanceolatum* and *C.rhomboideum*). Hunziker never recognised *C.lycianthoides* as an accepted species (Hunziker 2001: 232–240) and, judging from his descriptions of corolla differences in the protologue of *C.scolnikianum*, it is obvious he misapplied the name *C.geminifolium*, referring to what is actually *C.lycianthoides*. Until recently, this confusion (accepting both *C.geminifolium* and *C.scolnikianum*, but not *C.lycianthoides*) has prevailed in literature (Moscone et al. 2007; Barboza 2016; Carrizo García et al. 2016). In Jarret et al. (2019), *C.scolnikianum* was synonymised under *C.geminifolium*, whereas *C.lycianthoides* was accepted as distinct, this treatment being supported in the updated phylogeny (Barboza et al. 2019).

The type collection of *C.scolnikianum*, housed at CORD, is mounted on two sheets (CORD00006647, CORD00006648). A label in Hunziker’s hand stating “C.scolnikianum, Typus!, Scolnik 1389” on CORD 00006647 indicates his intent to consider this sheet as the type; therefore, we here designate it the lectotype.

#### Specimens examined.

See Suppl. material 4: Appendix 4.

### ﻿22. Capsicum hookerianum

Taxon classificationPlantaeSolanalesSolanaceae

(Miers) Kuntze, Revis. Gen. Pl. 2: 449. 1891.

842619DE-48B5-528D-8F00-8BAF6B724A5F

Figs 73 , 74

Brachistus hookerianus Miers, Ann. Mag. Nat. Hist., ser. 2, 3(16): 268. 1849. Type. Ecuador. Guayaquil: “Cerro of Lantana”, Jan 1846, W. Jameson s.n. (lectotype, designated by Barboza 2011, pg. 25: K [K000585919]; isolectotypes: CORD [CORD00101767 fragment ex K], US [02827225, acc. # 1941340 photo + fragment ex K).
Bassovia brachypoda Dunal, Prodr. [A. P. de Candolle] 13(1): 411. 1852. Type. Peru. J.A. Pavon s.n. (holotype: G; isotypes: CORD [CORD00084676 fragment ex G]; MPU [MPU023058]).
Capsicum brachypodum (Dunal) Kuntze, Revis. Gen. Pl. 2: 450. 1891. Type. Based on Bassoviabrachypoda Dunal
Capsicum eggersii Bitter, Repert. Spec. Nov. Regni Veg. 18: 126. 1922. Type. Ecuador. Manabi: Agua Amarga, near El Recreo, 15 Jan 1897, *H.F.A. von Eggers 15555* (holotype: B [destroyed, F neg. 2867]; lectotype, designated here: M [M-0171548]; isolectotypes: CORD [CORD00084677 fragment ex L, CORD00087961 fragment ex K), F [F0072805F, acc. # 143187, fragment], K [K000585902], L [L.2881993, acc. # 602560], P [P00482081], US [01919835, acc. # 939121]).

#### Type.

Based on *Brachistushookerianus* Miers.

#### Description.

Climbing erect subshrubs or shrubs, (0.5–) 1–3 m tall, with the main stem 1.5–2 cm in diameter at base, profusely branched above. Young stems, angled, fragile, flexuous, glabrous to moderately pubescent with white, simple, uniseriate, 4–6-celled, eglandular trichomes 0.4–0.9 mm long; nodes green; bark of older stems longitudinally striped, dark brown, glabrous; lenticels abundant, elliptic or rounded, cream or light brown. Sympodial units difoliate, the leaves geminate; leaf pair unequal in size and shape. Leaves membranous, discolorous, deep green above, paler below, with sparse white simple eglandular trichomes adaxially and moderate to abundant trichomes abaxially, especially on main veins, the trichomes 0.4–1.2 mm long, sometimes branched trichomes 0.6–1 mm long; blades of major leaves 4–12.5 cm long, 2–5.5 cm wide, ovate or broadly ovate, the major veins 4–6 on each side of mid-vein, the base long-attenuate, strongly asymmetric, the margins entire, the apex acuminate; petioles 0–1.3 cm long, somewhat winged due to the decurrence of the base leaf, pubescent or glabrescent; blades of minor leaves 2.5–4.2 (–7) cm long, 1.2–2.7 cm wide, ovate, the major veins 3–4 on each side of mid-vein, the base short, attenuate, rarely asymmetric, the margins entire, the apex acute, obtuse or rounded; petioles 0–0.5 cm, glabrescent or pubescent. Inflorescences axillary, 2–7 flowers per axil, rarely flowers solitary; flowering pedicels 8–15 mm long, thin, pendent, non-geniculate at anthesis, glabrous to glabrescent, the eglandular trichomes antrorse; pedicels scars conspicuous, corky. Buds not seen. Flowers 5–merous. Calyx 1.3–2 mm long, 2.2–2.7 mm wide, cup-shaped, green, glabrescent to densely pubescent with eglandular trichomes, the calyx appendages usually 8–10, unequal, the five main appendages longer 4–6 (–7) mm long, the secondary appendages shorter 1.3–4 mm, erect or somewhat spreading, linear-subulate, ca. 1 mm below the margin. Corolla 7.5–9 mm long, 8–10 mm in diameter, yellow, campanulate with abundant interpetalar membrane, lobed nearly 1/3 of the way to the base, the tube 4.5–6 mm long, glabrous adaxially and abaxially, the lobes 2–2.5 (–3) mm long, ca. 3.5–5.5 mm wide, broadly triangular, glabrous adaxially and with abundant eglandular trichomes near the apex abaxially, the margins papillate, the tips cucullate, papillate. Stamens five, equal; filaments (1.5–) 1.8–2 mm long, inserted on the corolla ca. 2 mm from the base, with auricles fused to the corolla at the point of insertion; anthers (1.5–) 1.7–2.1 mm long, ellipsoid, yellow, not connivent at anthesis. Gynoecium with ovary 1.5–1.6 mm long, ca. 1.3 mm in diameter, ovoid or subglobose; ovules more than two per locule; nectary 0.5 mm tall; styles homomorphic, ca. 4 mm long, exserted less than 1 mm beyond the anthers, clavate; stigma ca. 0.5 mm long, 0.8–1 mm wide, capitate or bilobed, light green. Berry 5–9 mm in diameter, globose or subglobose, green or yellow when immature, bright red at maturity, non-pungent, the pericarp thick, opaque, lacking giant cells (endocarp smooth); stone cells 2–4, irregular in shape; fruiting pedicels 12–18 mm long, pendent, angled, widened distally, green; fruiting calyx 4–6 mm in diameter, persistent, not accrescent, discoid, greenish-white, the appendages 3–6 (–7.5) mm long, spreading or reflexed, greenish-white and purple tinged. Seeds (10–) 22–45 per fruit, 2.2–2.8 mm long, 1.5–1.9 mm wide, ellipsoid or C-shaped, yellow or brown, the seed coat reticulate (SM), cerebelloid (SEM), the cells irregular in shape, the lateral walls sinuate to strongly sinuate; embryo imbricate.

#### Distribution.

*Capsicumhookerianum* is restricted to extra-Andean western Ecuador (Guayas, El Oro, Loja and Manabí Provinces) and northern Peru (Tumbes Department) (Fig. 75).

#### Ecology.

*Capsicumhookerianum* grows in dry deciduous forests at low elevations (100–1,000 m).

#### Phenology.

Flowering from November to April (July); fruiting from late December to July.

#### Chromosome number.

Not known.

#### Common names.

None recorded.

#### Uses.

None recorded.

#### Preliminary conservation assessment.

EOO (32,802.812 km^2^); AOO (136 km^2^). Although *C.hookerianum* has a relatively large extent of occurrence and is found in many localities, some of them protected areas, we observed a continued decline in the area of occupancy outside of official natural reserves; for these reasons, we assign a category of Near Threatened (NT).

#### Discussion.

*Capsicumhookerianum* is a member of the Andean clade (Barboza et al. 2019). This species is unique in growing mostly in extra-Andean lowlands of coastal Ecuador and Peru and can be easily recognised by its dark brown stems, strongly decurrent leaves, intensely bright red fruits and conspicuous fruiting calyx with 10 unequal long appendages, which are sometimes reflexed (Fig. 74). Apparently, the corollas have no brownish or purple pigmentation, since collectors only mention dull yellow or ochre corollas on herbarium labels. We were unable to locate this species in flower in the field.

**Figure 73.**
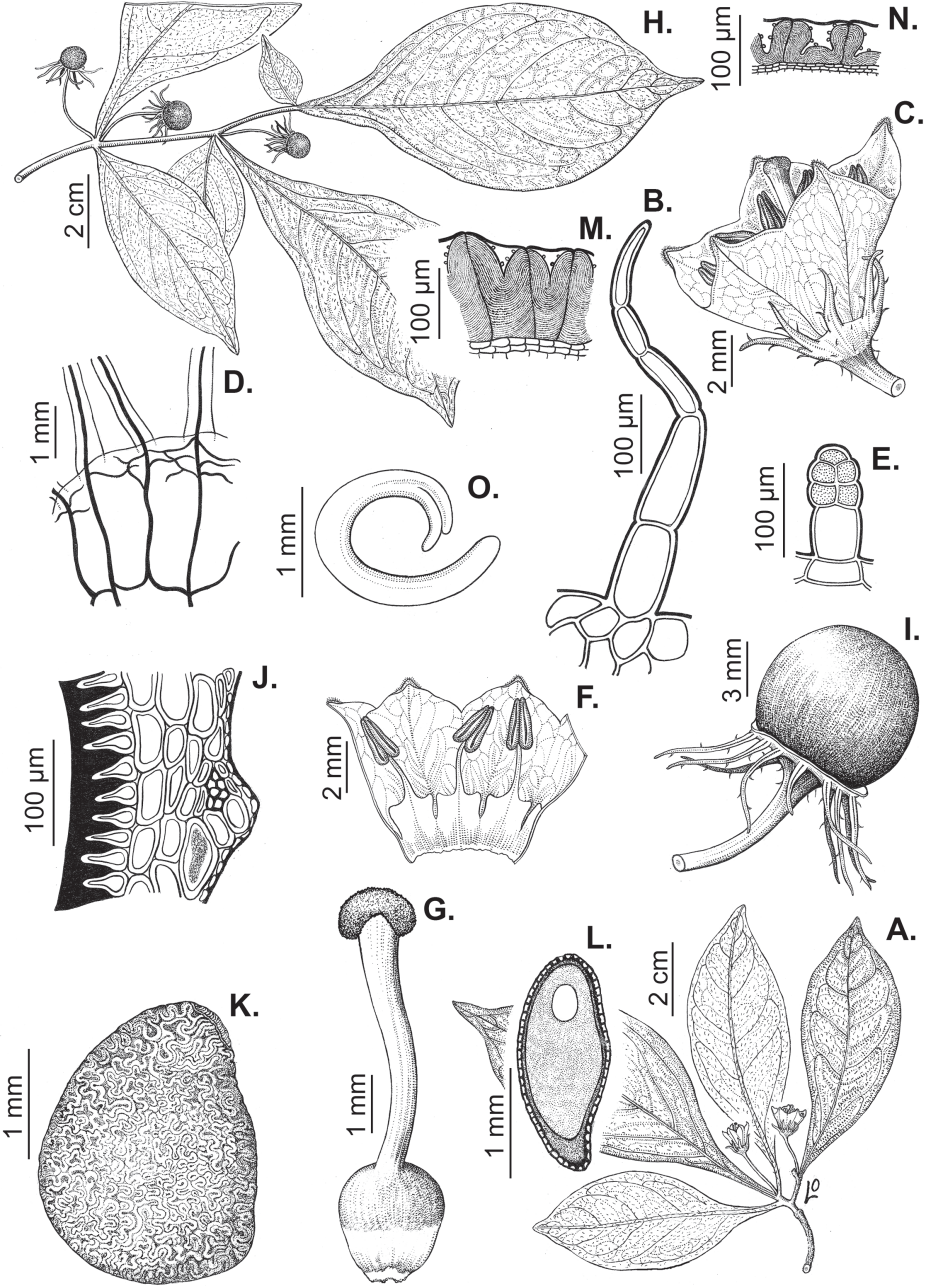
*Capsicumhookerianum***A** flowering branch **B** eglandular trichome of the leaf **C** flower **D** section of the calyx showing the venation **E** glandular trichome of the abaxial surface of the calyx **F** sector of opened corolla **G** gynoecium **H** fruiting branch **I** fruit **J** anatomical detail of the pericarp (note the absence of giant cells in the mesocarp) **K** seed **L** seed, in cross section **M** structure of seed coat at the seed margin **N** structure of seed coat at the seed body **O** embryo **A–G** from *Asplund 15241***H–O** from *Asplund 15363.* Drawn by L. Ochoa.

**Figure 74.**
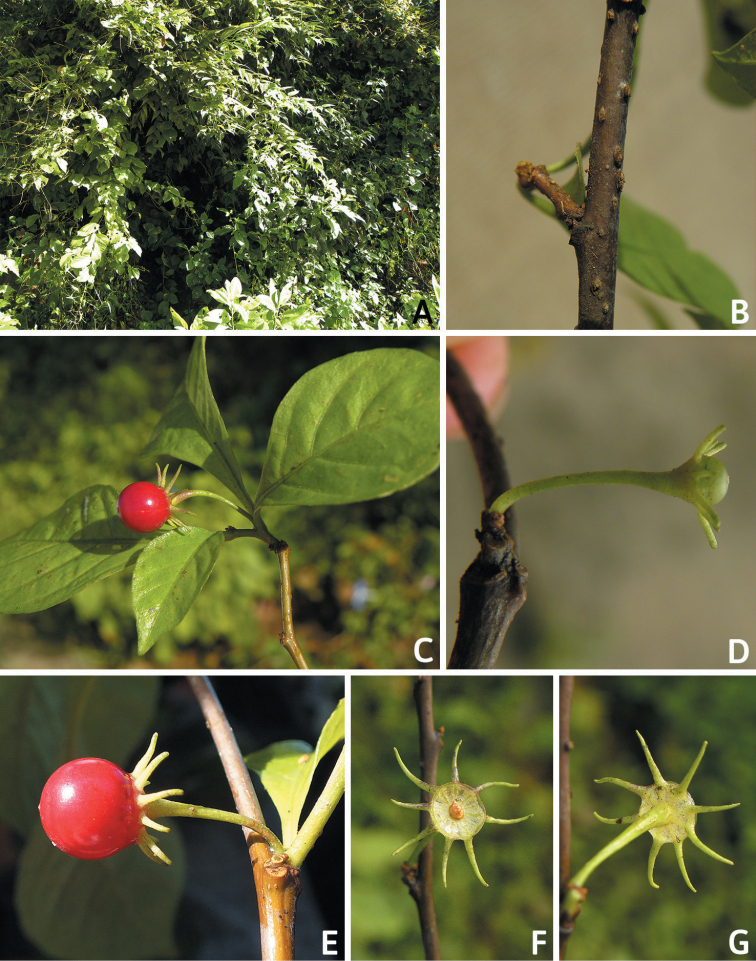
*Capsicumhookerianum***A** plant; **B** internode with lenticels; **C** fruiting branch; **D** immature fruit; **E** mature fruit; **F, G** fruiting calyx, front and from behind views, respectively. From *Barboza and Leiva González 4826*. Photos by G.E. Barboza.

**Figure 75.**
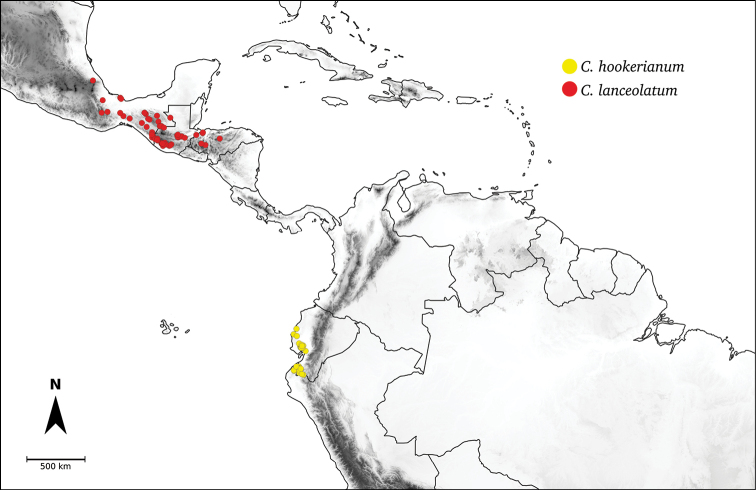
Distribution of *C.hookerianum* and *C.lanceolatum*.

*Capsicumhookerianum* is sympatric with the widespread *C.rhomboideum* which is a more vigorous plant with dense, branched pubescence and multi-flowered inflorescences with up to 12 flowers (Fig. 113); *C.hookerianum* has simple trichomes and 2–7 flowers in each inflorescence.

Duplicates of the type collection of *C.eggersii* are distributed in several Herbaria (CORD, F, K, L, M, P, US). At CORD and F, there are small fragments taken from other specimens (e.g. fragments accompanying F neg. 2867 from B and fragments in CORD taken from the duplicate in L), whereas in the remaining herbaria, the specimens consist of good fertile material. We have selected the most complete and best-preserved sheet of *Eggers 15555* (M-0171548) as the lectotype for *C.eggersii*.

#### Specimens examined.

See Suppl. material 4: Appendix 4.

### ﻿23. Capsicum hunzikerianum

Taxon classificationPlantaeSolanalesSolanaceae

Barboza & Bianch., Syst. Bot. 30(4): 867. 2005.

23D556F5-C891-5291-B68F-C7472E7536B2

Figs 76 , 77

#### Type.

Brazil. São Paulo: Salesópolis, Boracéia, 30 Nov 1951, *M. Kuhlmann 2785* (holotype [2 sheets]: CORD [CORD00003944 (sheet A) and CORD00003945 (sheet B); isotypes: CEN [CEN00109451], SP [SP001623, acc. # 79274], SPSF [acc. # 30213]).

#### Description.

Robust erect shrubs 1–3 m tall, much branched above, the branches much leafy. Young stems terete or slightly 3-angled, rigid, green with violet spots, glabrous; nodes solid, green or purple; bark of older stems fissured, brown, glabrous; lenticels absent. Sympodial units difoliate, the leaves geminate; leaf pair unequal in size, similar or dissimilar in shape. Leaves coriaceous, discolorous, dark green above, light green beneath, glabrous on both sides; blades of major leaves (7.5–) 9.5–20 (–25) cm long, 2.5–7 (–8.5) cm wide, ovate to elliptic, the major veins (4–) 5–7 on each side of mid-vein, the base attenuate and unequal, the margins entire, slightly revolute, the apex long-acuminate; petioles (0.5–) 0.8–2 (–3.5) cm long, green with dark lilac spots, glabrous; blades of minor leaves 2–4.5 cm long, (0.8–) 1–2 cm wide, ovate, the major veins 3–4 on each side of mid-vein, the base attenuate, the margins entire, the apex acute; petioles 0.2–0.5 cm long, glabrous. Inflorescences axillary, 2–4 (–5) flowers per axil, rarely flowers solitary; flowering pedicels (13–) 20–38 (–48) mm long, terete or slightly angled, erect or slightly spreading, geniculate at anthesis, green, glabrous or sparsely pubescent with minute, few glandular trichomes (stalk unicellular; head dark, multicellular) and some sparse antrorse eglandular trichomes; pedicels scars conspicuous, corky. Buds ovoid, cream or white. Flowers 5-merous. Calyx 3.5–5 (–6.5) mm, 4–4.5 mm wide, cup-shaped, membranous and cream amongst the veins, the veins thick and pale green, glabrous, the calyx appendages 5 (6–10), unequal, pale green, erect to spreading, cylindrical, inserted very close to the margin, the five main appendages longer 2.5–4.5 (–5) mm long, the 1–5 secondary appendages shorter 1–2 mm long. Corolla 10–14 (–16) mm long, (10–) 15–23 mm in diameter, thick, white with greenish-yellow lines outside, mostly white with diffuse brown-purple spots at the base of each lobe and throat and a narrow greenish-yellow centre within, stellate with thin interpetalar membrane, lobed 1/3 of the way to the base, glabrous abaxially and adaxially, the tube 5–7.5 mm long, the lobes 6–8 mm long, (3–) 3.5–5 (–6) mm wide, broadly triangular, spreading, the margins involute and finely ciliate, the tips strongly cucullate forming a hood-like structure, papillate. Stamens five, equal; filaments (1.5–) 2–3 (–3.5) mm long, white or cream, inserted on the corolla ca. 1.5–2 mm from the base, with auricles fused to the corolla at point of insertion; anthers (2–) 2.5–4 mm, ellipsoid, grey, not connivent at anthesis. Gynoecium with ovary 1–2 mm long, 1–1.5 mm in diameter, light green, subglobose; ovules more than two per locule; nectary ca. 0.3 mm tall; styles homomorphic, (4–) 5–6 mm long, exserted ca. 1 mm beyond the anthers, cream, clavate; stigma 0.3 mm long, 0.8–0.9 mm wide, somewhat discoid-bilobulate, light green or cream. Berry 6–10 mm in diameter, globose, slightly depressed, light green when immature, greenish-golden yellow at maturity, deciduous, pungent, the pericarp thin, translucent, with giant cells (endocarp alveolate); stone cells 3–5, spherical, 0.17–0.22 mm in diameter; fruiting pedicels (23–) 35–50 mm long, curved or pendent, terete, strongly widened at the apex, green; fruiting calyx 4–7 mm in diameter, persistent, not accrescent, green, discoid, the appendages 3–6 (–7) mm long, green, spreading or reflexed. Seeds 10–20 per fruit, 2.5–3.2 mm long, 2–2.5 mm wide, C-shaped or ellipsoid, black, the seed coat reticulate and tuberculate at margins (SM), reticulate with pillar-like outgrowths at margins (SEM), the cells polygonal in shape, the lateral walls straight; embryo imbricate.

**Figure 76.**
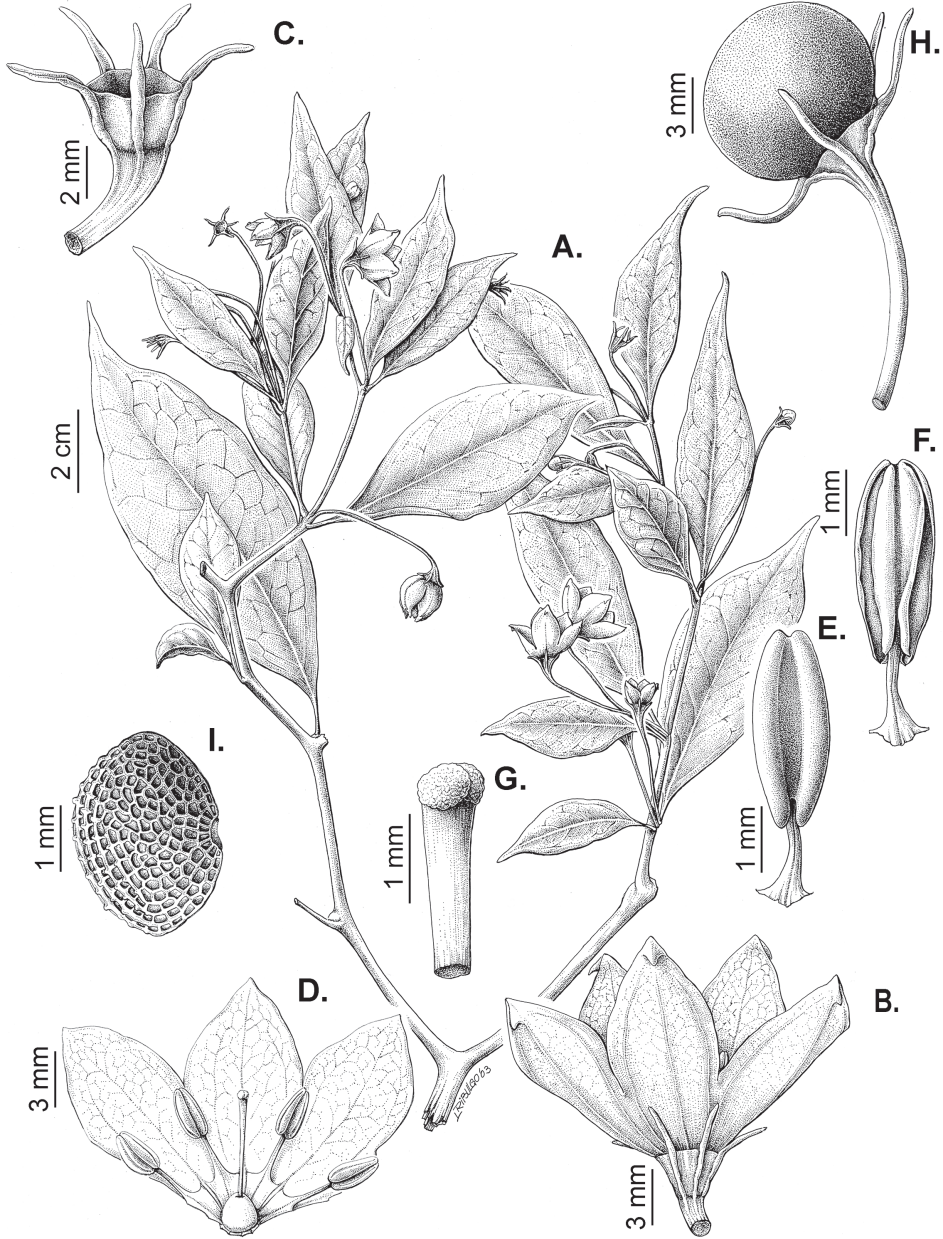
*Capsicumhunzikerianum***A** flowering branch **B** flower **C** calyx **D** sector of opened corolla **E, F** anthers, dorsal and ventral views, respectively **G** stigma **H** fruit **I** seed **A–G** from *Kuhlmann 2785*, **H, I** from *Mattos and Mattos 14254*. Drawn by L. Ribulgo. Published in Barboza and Bianchetti (2005), reproduced with permission.

**Figure 77.**
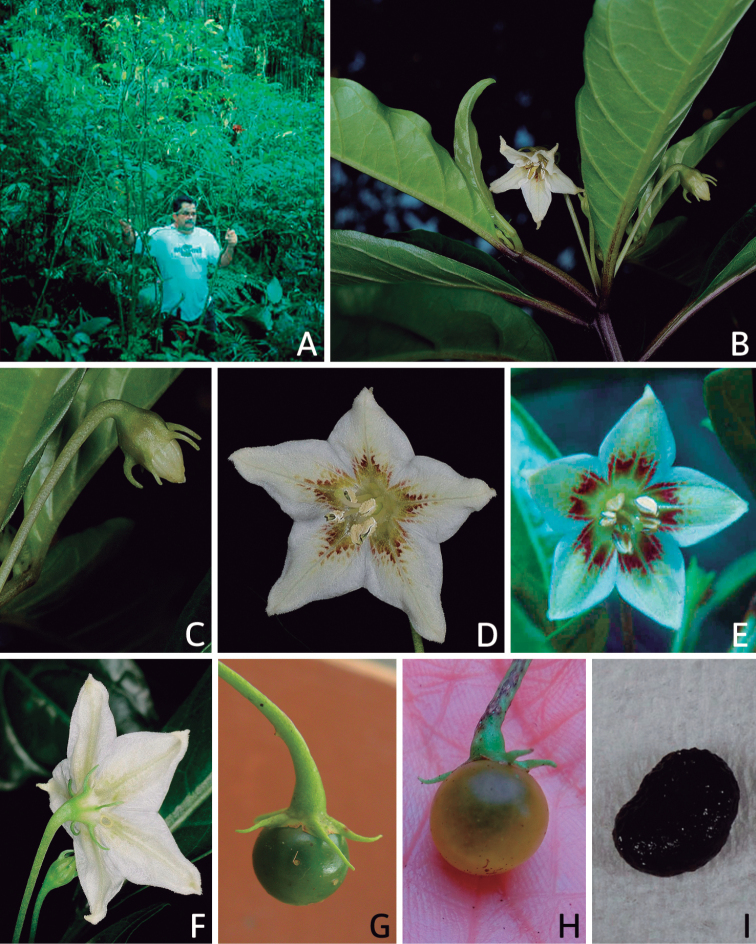
*Capsicumhunzikerianum***A** plant **B** flowering branch **C** flower bud **D, E** flowers, in front view, with different corolla colour patterns **F** flower, seen from behind **G** immature fruit **H** mature fruit **I** seed **A, E** from *Bianchetti et al. 1537*, photos by L. Bianchetti **B, C, D, F, H, I** no specimen vouchers, photos taken *in situ* by C. dal Zovo (Associazione PepperFriends) **G** from *Barboza 5041*, photo by G.E. Barboza.

#### Distribution.

**Figure 78.**
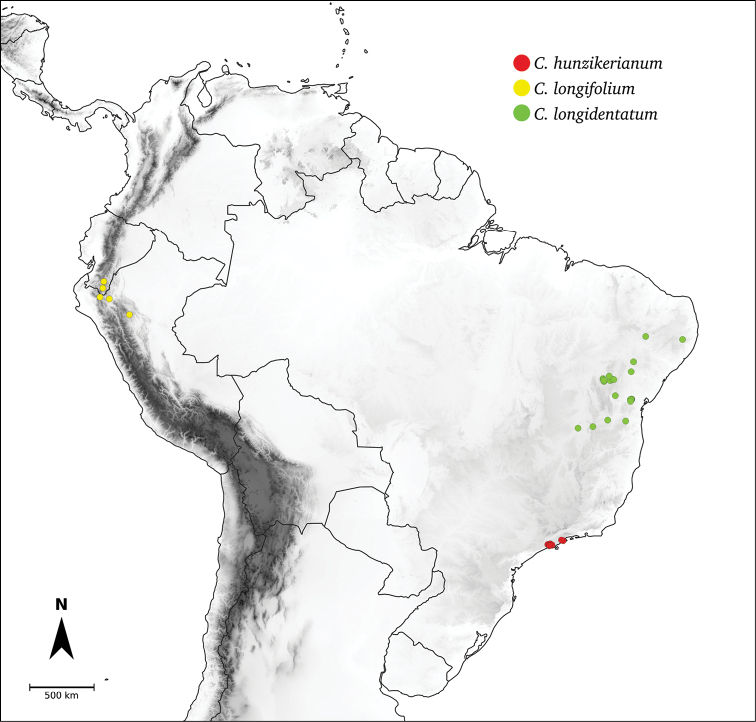
Distribution of *C.hunzikerianum*, *C.longidentatum* and *C.longifolium*.

*Capsicumhunzikerianum* is only known from a very restricted area in south-eastern Brazil (São Paulo State) (Fig. 78).

#### Ecology.

*Capsicumhunzikerianum* inhabits the montane forests of the Atlantic Forest (Mata Atlântica), in Dense Ombrophilous Forest (Floresta Ombrófila Densa), in wet, shady or semi-shady or marshy places, at medium elevations (800–1,100 m).

#### Phenology.

Flowering from November to May. Fruiting from late January to May.

#### Chromosome number.

2*n* = 2x = 26 (*Barboza et al. 5041*, see Table 2).

#### Common names.

None recorded.

#### Uses.

None recorded.

#### Preliminary conservation assessment.

EOO (1,456.094 km^2^); AOO (40 km^2^). *Capsicumhunzikerianum* grows in two rain forest reserves in the State of São Paulo, the Parque Estadual da Serra do Mar-Núcleo Cunha and the Estação Biológica da Boracéia. In both protected areas, few subpopulations have been recorded. Based on the small extent of occurrence and area of occupancy, the low number of the different locations (= 5) and a decline in the area of occupancy, we assign *C.hunzikerianum* the threat status of Endangered (EN; B1ab(ii).

#### Discussion.

*Capsicumhunzikerianum* is probably a member of the Atlantic Forest clade, but has not yet been included in phylogenetic analyses. It shares corolla shape and colour, fruit colour and pungency, seed colour, seed coat ornamentation, chromosome number, habitat and distribution of the members of this clade. It is a distinct species of the montane forest of Serra do Mar distinguished by its general glabrescence, large and coriaceous leaves and large flowers. The calyx is the largest in the genus, reaching 6.5 mm long, with five long appendages (up to 5 mm long) and 1–5 unequal, shorter appendages (1–2 mm long). The corolla is unusual for its large size and white colour with few brown-purple spots (Fig. 77).

*Capsicumhunzikerianum* is sympatric with the morphologically similar *C.schottianum*. The two species share geniculate pedicels, stellate corollas and fruit and seed features, but they differ in the corolla size (10–16 mm long in *C.hunzikerianum* vs. 7–10 mm long in *C.schottianum*), number of calyx appendages (5–10 vs. 0–5 minute appendages), consistency of the leaves (coriaceous vs. membranous) and general indumentum (plants glabrous vs. plants glabrescent to pubescent).

#### Specimens examined.

See Suppl. material 4: Appendix 4.

### ﻿24. Capsicum lanceolatum

Taxon classificationPlantaeSolanalesSolanaceae

(Greenm.) C.V.Morton & Standl., Publ. Field Mus. Nat. Hist., Bot. Ser. 22(4): 272. 1940.

2050C0C9-1E31-54DE-92CA-86C8F595D1AD

Figs 79 , 80

Brachistus lanceolatus Greenm., Bot. Gaz. 37(4): 212. 1904. Type. Guatemala. Alta Verapaz: Chucaneb, 1850 m elev., Apr 1889, *J. Donnell Smith 1837* (holotype: US [00027406, acc. # 1335158]).
Capsicum guatemalense Bitter, Repert. Spec. Nov. Regni Veg. 20: 377. 1924. Type. Guatemala. Suchitepequez: Las Nubes, Nov 1877, *C.G. Bernoulli & R. Cario 2339* (lectotype, designated here: GOET [GOET003421]; isolectotype: GOET [GOET003422]).

#### Type.

Based on *Brachistuslanceolatus* Greenm.

#### Description.

Erect shrubs or subshrubs, 1–3 (–5) m tall, much branched from near the base, the branches flexible. Young stems terete or slightly angled, fragile, green, glabrous or glabrescent with sparse white antrorse, curved, simple, uniseriate, 4–6-celled, eglandular trichomes 0.4–1.3 mm long; nodes green; bark of older stems brown, glabrous; lenticels absent. Sympodial units difoliate, the leaves geminate; leaf pair markedly unequal in size and shape. Leaves membranous to coriaceous, concolour, glabrous adaxially and with simple, 2–5-celled, eglandular trichomes abaxially, especially on veins and margins; blades of major leaves 6.5–16 cm long, 1.4–3.8 cm wide, elliptic to lanceolate, the major veins 5–7 on each side of mid-vein, sometimes more evident only on one side, the base long-attenuate or attenuate, asymmetric, the margins entire, the apex long-acuminate; petioles 0.5–0.8 cm long, glabrescent; blades of minor leaves (1–) 2–5.5 cm long, (0.6–) 0.8–2.7 cm wide, ovate or elliptic, the major veins 2–3 on each side of mid-vein, the base slightly asymmetric, the apex acute or obtuse; petioles 0–0.2 cm long, glabrescent. Inflorescences axillary, 1-flowered, rarely 2-flowered; flowering pedicels (15–) 25–43 mm long, thin, curved to pendent, non-geniculate at anthesis, green or purple, glabrous; pedicels scars inconspicuous. Buds ovoid, yellow-purple or intense purple. Flowers 5-merous, rarely perianth 4-merous. Calyx 1.2–2 mm long, 2–4 mm wide, cup-shaped, green or greenish-purple, glabrous or glabrescent, the calyx appendages (4–) 5, (2–) 3–5 mm long, ca. 0.7 mm wide, subequal, spreading or strongly reflexed, linear or subulate, 0.1–0.4 mm below the margin, glabrous or glabrescent. Corolla 9.8–14 mm long, 10–15 mm in diameter, purple and marginally white outside and within, campanulate with abundant interpetalar membrane, lobed 1/3 of the way to the base, glabrous abaxially and adaxially, the tube 8–11 mm, the lobes (1.8–) 2–3 mm long, 3.5–4 mm wide, triangular to broadly triangular, the margins and tips papillate or with short eglandular trichomes. Stamens five, equal or subequal; filaments 2.5–4.2 (–4.6) mm long, white or yellowish-white, inserted on the corolla 2–2.5 mm from the base, with auricles fused to the corolla at the point of insertion; anthers (1.2–) 1.5–2 mm long, ellipsoid, yellowish-white, connivent at anthesis. Gynoecium with ovary ca. 2 mm long, 1.5–1.8 mm in diameter, pale green, ellipsoid or ovoid; ovules more than two per locule; nectary 0.5 mm tall; styles homomorphic, 4.5–6 mm long, exserted 1–2 mm beyond the anthers, white; stigma ca. 0.2 mm long, ca. 0.8 mm wide, slightly bilobed, light green. Berry 7–13 mm in diameter, globose or globose-compressed, green when immature, orange or orange-red at maturity, non-pungent, the pericarp thick, opaque, lacking giant cells (endocarp smooth); stone cells absent; fruiting pedicels (25–) 30–55 mm long, pendent, terete or slightly angled, widened distally, green or greenish-purple; fruiting calyx 4–7 mm in diameter, persistent, not accrescent, green, discoid, with a strong annular constriction at junction with the pedicel, the appendages 4–9 mm long, 0.8–1 mm wide, green, strongly reflexed. Seeds (20–) 29–60 (–93) per fruit, 2–2.5 mm long, (0.8–) 1.2–1.8 mm wide, C-shaped or teardrop-shaped, brownish-black to black, the seed coat reticulate (SM and SEM), the cells polygonal to slightly irregular in shape, the lateral walls straight or slightly sinuate; embryo annular.

#### Distribution.

*Capsicumlanceolatum* is distributed in southern Mexico, Guatemala and Honduras (Fig. 75).

#### Ecology.

*Capsicumlanceolatum* grows in primary forest remnants or disturbed montane rain forests on steep slopes, ravines, or in stream canyons at (100–) 1,000–3,000 m elevation.

#### Phenology.

Flowering from May to December and fruiting all year.

#### Chromosome number.

2*n* = 2x = 26 (Tong and Bosland 2003).

#### Common names.

**Guatemala**: Pajarito del río (Quezaltenango, *Steyermark 33429*), Yerba de pajarito (Quezaltenango, *Steyermark 33357*).

#### Indigenous names.

**Mexico**: Tumattez (Tzeltal, Chiapas, *Méndez Girón 7745*), Chuj ch ‘ul tumaltez (Tzeltal, Chiapas, *Ton 7803*).

#### Uses.

None recorded.

#### Preliminary conservation assessment.

EOO (255,067.790 km^2^); AOO (252 km^2^). *Capsicumlanceolatum* has a large extent of occurrence; however, based on its small area of occupancy, the continuing decline of the number of locations, the extreme fluctuations observed in the number of mature individuals in the subpopulations and the demonstrated extinction of this species in many natural habitats (Bosland and González 2000), we assign this species the Endangered (EN; B2b,c(ii,iii,iv)) status.

**Figure 79.**
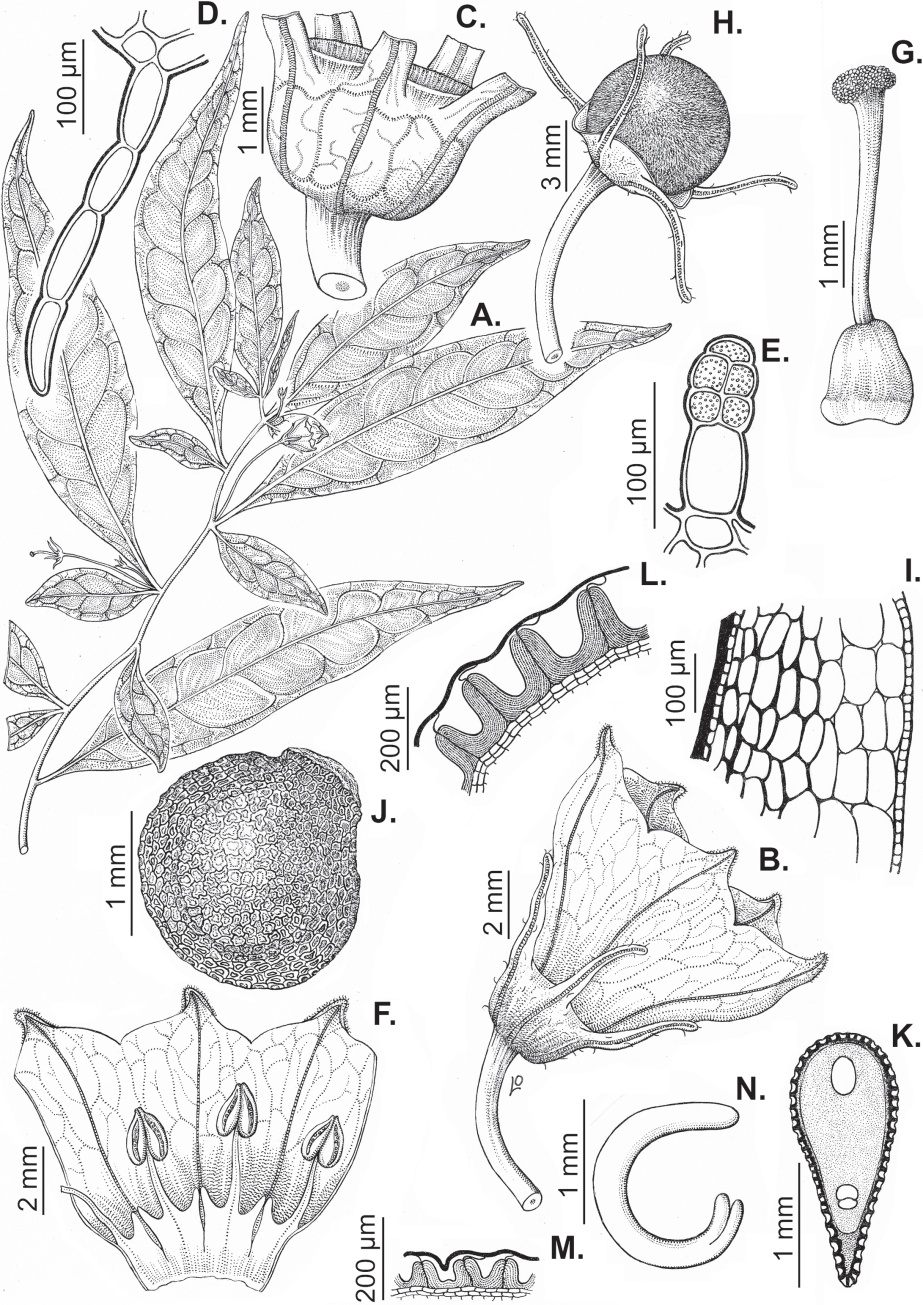
*Capsicumlanceolatum***A** flowering branch **B** flower **C** calyx (appendages cut off) **D** eglandular trichome of the adaxial surface of the calyx **E** glandular trichome of the abaxial surface of the calyx **F** sector of opened corolla **G** gynoecium **H** fruit **I** anatomical detail of the pericarp (note the absence of giant cells in the mesocarp) **J** seed **K** seed, in cross section **L** structure of seed coat at the seed margin **M** structure of seed coat at the seed body **N** embryo **A** from *Ventura A. 798***B–G** from *Skutch 1450***H–N** from *Beaman 6014*. Drawn by L. Ochoa. Published in Hunziker (2001), reproduced with permission.

#### Discussion.

**Figure 80.**
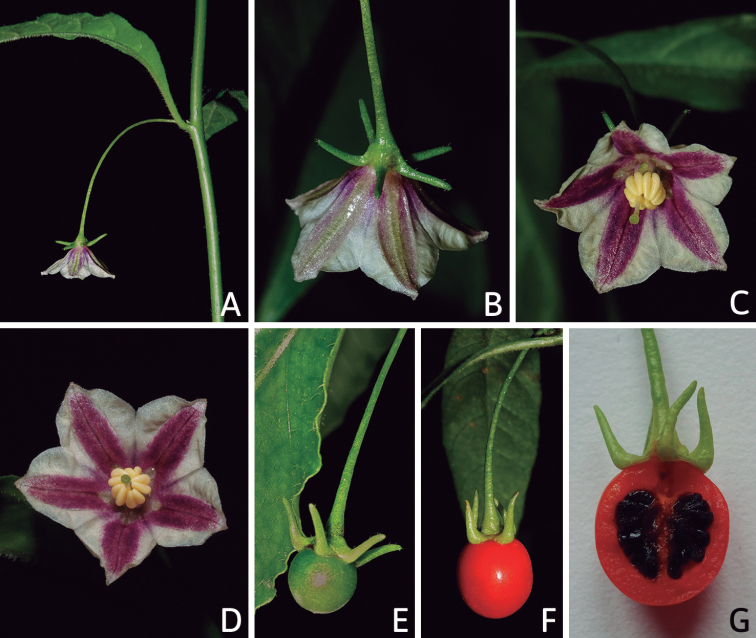
*Capsicumlanceolatum***A** pendent flower **B** flower, lateral view **C** flower, in front view **D** flower showing connivent anthers **E** immature fruit **F** mature fruit **G** fruit, longitudinal section, showing the black seeds. From *Carrizo García 73*. Photos by C. Carrizo García.

*Capsicumlanceolatum* is a member of the Andean clade (Carrizo García et al. 2016; Barboza et al. 2019). It is an uncommon Mexican and Central American species that can be distinguished from all other *Capsicum* species by its long pedicels, solitary and large flowers, campanulate white-purple corollas, relatively large fruits (up to 13 mm diameter), non-pungent fruits and long, strongly reflexed fruiting calyx appendages (Fig. 80). Tong and Bosland (2003) have shown that this species is self-compatible.

The other wild *Capsicum* species of the Andean clade that partially overlaps in distribution with *C.lanceolatum* is *C.rhomboideum*; this latter species is found in more disturbed areas. *Capsicumlanceolatum* differs from *C.rhomboideum* by having less vigorous habit, mostly glabrous (vs. densely pubescent) plant body, solitary flowers (vs. many-flowered inflorescences), white-purple corollas (vs. yellow) and larger fruits (berries 7–13 mm vs. 5–9 mm in diameter in *C.rhomboideum*).

*Capiscumguatemalense* was described, based on a collection made by Bernoulli and Cario held in GOET (Bitter 1924). Two sheets of this collection are held at GOET (GOET003421, GOET003422). The first of these (GOET003421) is the most complete and has an identification label in Bitter’s hand that we here designate as the lectotype.

#### Specimens examined.

See Suppl. material 4: Appendix 4.

### ﻿25. Capsicum longidentatum

Taxon classificationPlantaeSolanalesSolanaceae

Agra & Barboza, Syst. Bot. 36(3): 771. 2011.

79451F4B-F292-5FC7-B23E-0CF43A46D75A

Figs 81 , 82

#### Type.

Brazil. Bahia: Mun. Itatim, Morro da Pedra Grande, base do Morro, 12°42'57"S, 39°45'46"W, 285 m elev., 8 Apr 2006, *E. Melo, F. França, C.T. Lima & C. Cunha 4344* (holotype: HUEFS [HUEFS000001093, acc. # 109249]).

#### Description.

Erect, slender or semi-scandent shrubs (1–) 1.5–4 m tall, much branched from near the base. Young stems angled, fragile, greyish or brown, glabrous, the youngest stems densely pubescent with furcate and dendritic eglandular trichomes 0.2–0.9 mm long; nodes green; bark of older stems greyish, glabrous; lenticels absent. Sympodial units difoliate, the leaves geminate; leaf pair unequal in size, similar in shape. Leaves membranous, discolorous, green above, light green or yellowish-green beneath, densely pubescent on both surfaces, especially abaxially, with simple uniseriate eglandular trichomes and branched trichomes similar to those of the stems; blades of major leaves 3.5–5.4 cm long, 1.5–2.5 cm wide, ovate, the major veins 4–5 on each side of mid-vein, the base short-attenuate or truncate and unequal, the margins entire, the apex acuminate; petioles (0.3–) 0.5–1.5 cm, densely pubescent; blades of minor leaves 2.5–3 cm long, 1–1.3 cm wide, ovate, the major veins 3–4 on each side of mid-vein, the base attenuate, the margins entire, the apex acute; petioles 0.2–0.5 cm long, densely pubescent. Inflorescences axillary, 2–5 flowers, rarely flowers solitary; flowering pedicels (6.5–) 8–23 mm long, strongly angled, pendent, non-geniculate at anthesis, with abundant spreading dendritic trichomes; pedicels scars inconspicuous. Buds ovoid, whitish-green. Flowers 5-merous. Calyx 2–4 mm long, 3–3.5 mm wide, cup-shaped, thick, greenish-yellow, strongly 5 (–6)-nerved, densely pubescent with branched trichomes, the calyx appendages 5 (–6), (4.5–) 5–8.5 mm long, 0.1–0.2 mm wide, subequal or unequal, erect, greenish-yellow, linear, inserted very close to the margin, with the same indumentum as calyx tube. Corolla 6–7.5 mm long, white outside, white with greenish-yellow spots in the lobes and throat and white centre within, stellate with interpetalar membrane, lobed nearly halfway to the base, pubescent adaxially with small glandular trichomes (stalk long, 2-celled; head globose, unicellular) in the throat and base of the lobes, glabrous abaxially, the tube 3–3.5 mm long, the lobes 3.3–3.6 (–4) mm long, 2–2.5 mm wide, broadly triangular, the margins involute densely pubescent, the tips cucullate densely papillate. Stamens five, equal; filaments 1.25–2 mm long, cream, inserted on the corolla ca. 1.5 mm from the base, with auricles fused to the corolla at the point of insertion; anthers 2–2.5 mm long, ellipsoid, cream or light brown, not connivent at anthesis. Gynoecium with ovary ca. 2 mm long, 1.3–1.4 mm in diameter, greenish-white, ovoid; ovules more than two per locule; nectary ca. 0.3 mm tall; styles homomorphic, 3.5–3.75 mm, exserted ca. 1 mm beyond the anthers, greenish-white, clavate; stigma ca. 0.3 mm long, 0.6 mm wide, somewhat discoid, light green. Berry 7.5–9.5 mm long, 7–8.5 mm in diameter, globose or subglobose, slightly flattened at the apex, light green when immature, colour at maturity unknown, probably yellowish-green, deciduous, non-pungent, the pericarp with giant cells (endocarp alveolate); stone cells absent; fruiting pedicels 15–20 mm long, pendent, angled, slightly widened distally, green; fruiting calyx 5–6 mm in diameter, persistent, not accrescent, green, discoid, strongly 5(–6)-nerved, the appendages 5–8.5 mm long, ca. 0.3 mm wide, spreading, green. Seeds (3–) 5–17 per fruit, 3–3.7 mm long, 2.5–2.8 mm wide, C-shaped or ellipsoid, brown to brownish-black, faintly reticulate (SM), cerebelloid (SEM), the cells irregular in shape, the lateral walls wavy to strongly sinuate; embryo imbricate.

#### Distribution.

*Capsicumlongidentatum* is endemic to the core of the Brazilian Caatinga (Bahia, Mina Gerais, and Pernambuco States) (Fig. 78).

#### Ecology.

*Capsicumlongidentatum* is common at the base of the inselbergs, on granitic hillsides with shrubby open vegetation and in gallery forests along small rivers, between 250 and 900 m.

#### Phenology.

Flowering from October to April; fruiting from November to May.

#### Chromosome number.

2*n* = 2x = 24 (Barboza et al. 2011).

#### Common names.

None recorded.

#### Uses.

None recorded.

#### Preliminary conservation assessment.

EOO (275,449.384 km^2^); AOO (92 km^2^). *Capsicumlongidentatum* is a species restricted to the Caatinga, an ecosystem with significant human disturbance and habitat fragmentation (Antongiovanni et al. 2018). Although this species has large EOO and number of locations, the continuing decline observed in its habitat and the projected risk of loss of suitable climate conditions for the Caatinga endemic species (Silva et al. 2019), suggest this species could be seriously threatened in the future. Thus, we consider *C.longidentatum* to merit a status of Near Threatened (NT).

**Figure 81.**
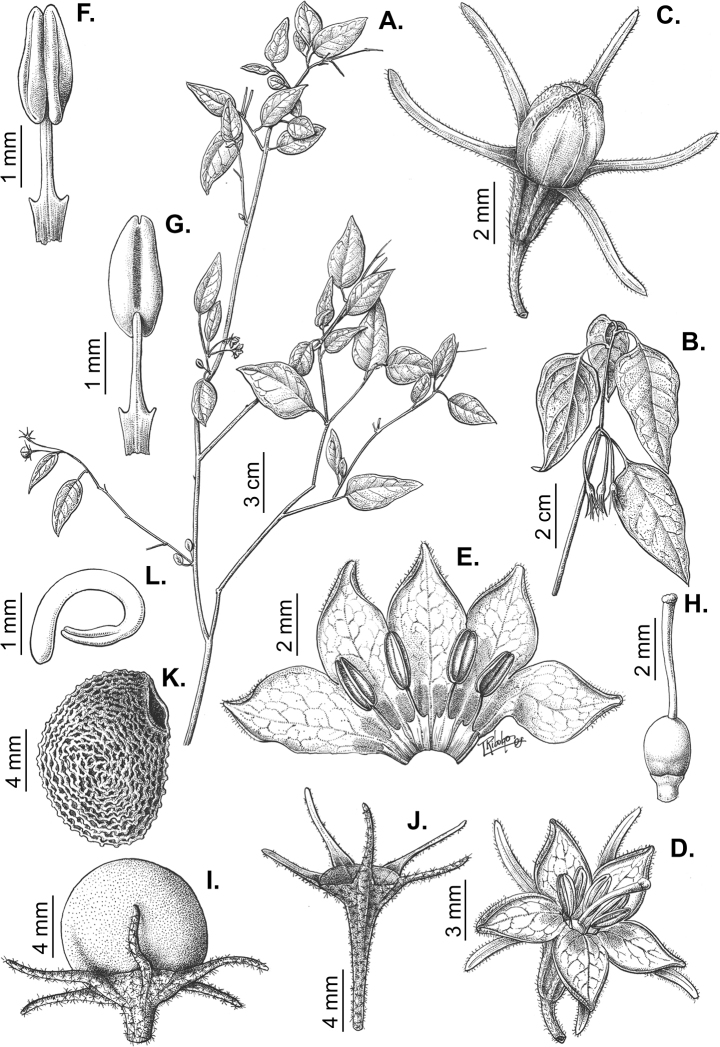
*Capsicumlongidentatum***A** reproductive branch **B** detail of a flowering node **C** flower bud **D** flower, upper view **E** opened corolla (one stamen has been removed) **F, G** anthers, ventral and dorsal views, respectively **H** gynoecium **I** fruit **J** fruiting calyx **K** seed **L** embryo **A, D, E, F–H** from *Melo et al. 4344***B, C, I–L** from *Agra & Barboza 7086*. Drawn by L. Ribulgo. Published in Barboza et al. (2011), reproduced with permission.

#### Discussion.

**Figure 82.**
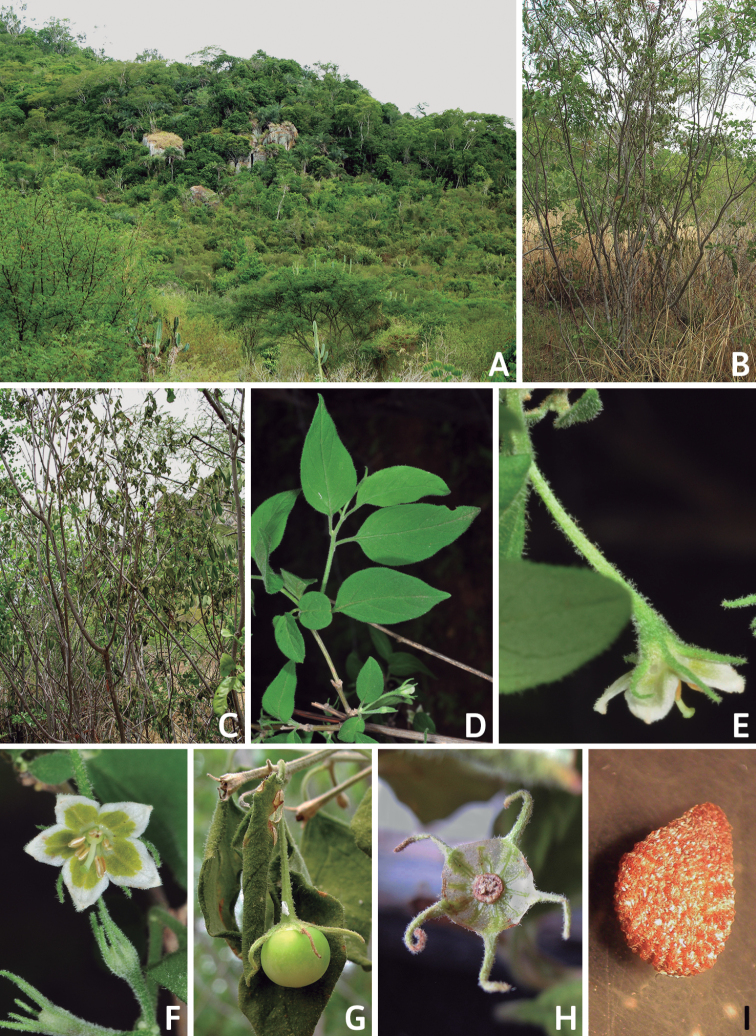
*Capsicumlongidentatum***A** habitat (Caatinga) **B** plant **C** typical aspect of the plant in the arid caatinga **D** flowering branch **E** pendent flower **F** flower, in front view **G** immature fruit **H** fruiting calyx **I** seed **A–C, G–I** from *Agra & Barboza 7086***D–F** from *Tabosa et al. 55***A–C, G–I** photos by G.E. Barboza **D–F** photos by J.R. Stehmann.

In phylogenetic analyses, *C.longidentatum* was resolved as an isolated branch and assigned to the monotypic Longidentatum clade, but the species placement is not strongly supported (Carrizo García et al. 2016). This situation could be due to the high percentage of missing data recorded for this species in the dataset analysed by Carrizo García et al. (2016), for which reason the case is being reviewed. Preliminary data, based on a dense DNA dataset, would indicate that it is related to species of the Caatinga clade (CCG, pers. obs.). *Capsicumlongidentatum* (Fig. 82) is unique and easily recognisable by its dense, branched indumentum (furcate and dendritic trichomes on young stems, leaves, pedicels and calyces) and the longest linear calyx appendages in the genus, reaching 8.5 mm in length (Barboza et al. 2011). The mature fruits are non-pungent.

*Capsicumlongidentatum* is distinguishable from *C.parvifolium* and *C.caatingae*, the other members of the Caatinga clade, in its long calyx appendages (4.5–8.5 mm long), white corolla with greenish-yellow spots within, densely branched pubescence (though furcate or dendritic trichomes appear occasionally in *C.caatingae* and *C.parvifolium*, respectively) and non-pungent fruits. *Capsicumcaatingae* and *C.parvifolium* have corollas mostly purple, usually simple pubescence and pungent fruits. *Capsicumcaatingae* lacks of calyx appendages (vs. five, rarely six, in *C.longidentatum*) and has arborescent habit and multi-flowered inflorescences (vs. shrubby habit and few-flowered inflorescences in *C.longidentatum*), while *C.parvifolium* has similar habit and inflorescences as *C.longidentatum*, but the 5 (–7) calyx appendages are shorter (0.7–2.2 mm long).

There are few reports on labels of the fruit colour of *Capsicumlongidentatum* at full maturity; it may be yellow (*Gonçalves et al. 175*, *Pastore & Harley 2603*, *Socorro 150*, *Melo 4693*) or orange (*Pastore & Harley 2603*). Both colour and consistency of the pericarp need to be checked in the field to observe whether it is similar to Brazilian species with greenish-golden yellow gelatinous fruits (Atlantic forest species and also *C.parvifolium*) or to the red fruited species, such as *C.caatingae*.

#### Specimens examined.

See Suppl. material 4: Appendix 4.

### ﻿26. Capsicum longifolium

Taxon classificationPlantaeSolanalesSolanaceae

Barboza & S.Leiva, PLOS One 14(1): 7. 2019.

9D5F7F56-B82E-5F44-B8E5-FCB9A852600A

Figs 83 , 84

#### Type.

Ecuador. Zamora-Chinchipe: Area of Estación Científica San Francisco, road Loja-Zamora, ca. 35 km from Loja, transect Q2, 03°58'S, 79°04'W, 1900 m elev., 12 Jun 2005, *F.A.Werner 1548* (holotype: QCA [160608]; isotypes: LOJA, NY [01130066]).

#### Description.

Erect, scandent shrubs (0.60–) 1.40–3 m tall, laxly branched above, the branches bending down. Young stems angled, fragile, green, glabrous; nodes green or purplish-green; bark of older stems striate, dark green, glabrous; lenticels ovoid, whitish to light brown. Sympodial units difoliate, the leaves geminate; leaf pair markedly unequal in size and shape. Leaves coriaceous, slightly discolorous, adaxial surface dark green and shiny, abaxial surface light green and opaque, glabrous on both surfaces and margins; blades of major leaves (7–) 8.5–17 (–18) cm long, (0.8–) 1–2.5 cm wide, narrowly elliptic, the major veins (11–) 13–17 on each side of mid-vein, the base asymmetric and attenuate, the margins entire, the apex acuminate; petioles (0.2–) 0.5–1.4 cm long, glabrous; blades of minor leaves 2.5–5.7 cm long, 1–2 cm wide, ovate or broadly elliptic, the major veins 4–5 on each side of mid-vein, the base short-attenuate, sometimes asymmetric, the margins entire, the apex obtuse; petioles 0.1–0.5 cm long, glabrous. Inflorescences axillary, 3–7 (–9) flowers per axil or sometimes on a short rachis, rarely flowers solitary; flowering pedicels 3–8 mm long, filiform, terete, pendent, slightly curved, non-geniculate at anthesis, green, glabrous; pedicels scars inconspicuous. Buds ovoid, yellow or purplish-yellow. Flowers 5-merous. Calyx 2.5–3 mm long, 2.8–3 mm wide, cup-shaped, membranous, translucent, light green or greenish-purple, glabrous, the calyx appendages 2–3, 2–2.5 mm long, 1.8–2.2 mm wide, subequal, thick, green or purple, oblique or spreading, triangular-compressed, wing-like, glabrous. Corolla 6–8.5 mm long, 8–11 mm in diameter, thick, entirely yellow or yellow with red-brown pigmentation in the throat or at margin lobes within, campanulate-stellate, without or with a thin interpetalar membrane, lobed 1/3 to nearly halfway to the base, glabrous adaxially and abaxially, the tube (3–) 4–5 mm long, the lobes 3–3.5 (–4) mm long, ca. 3 mm wide, broadly ovate, erect or spreading, the tips cucullate and papillate. Stamens five, equal; filaments 2–2.6 mm long, white or red-brown, inserted on the corolla ca. 2 mm from the base, with auricles fused to the corolla at the point of insertion; anthers 2–2.75 mm long, ellipsoid, purplish-white or brown, connivent at anthesis. Gynoecium with ovary 1.6–1.8 mm long, 1.2 mm in diameter, white or light green, subglobose; ovules more than two per locule; nectary 0.3–0.5 mm tall; styles homomorphic, 5–5.8 mm long, exserted 1–1.4 mm beyond the anthers, white and lilac at the apex or red-brown, clavate; stigma 0.3 mm long, 0.2–0.4 mm wide, somewhat bilobed, light green. Berry 8–13 mm in diameter, globose, slightly flattened at the apex, green when immature, orange at maturity, deciduous, non-pungent, the pericarp thick, opaque, lacking giant cells (endocarp smooth); stone cells absent; fruiting pedicels 10–16 mm long, pendent, terete, widened distally, green; fruiting calyx 4–5.5 mm in diameter, persistent, not accrescent, discoid, green-purple or green, the appendages short and wide (2–2.8 mm long, 2.4–2.6 mm wide at base) or long and more slender (4.5–5.5 mm long, ca. 1.5 mm wide at base), spreading or reflexed, green-purple or green. Seeds ca. 24 per fruit, 1.7–2.3 mm long, 1.7–2.2 mm wide, D- or teardrop-shaped, black, the seed coat reticulate (SM and SEM), the cells rectangular or polygonal in shape, the lateral walls straight or slightly sinuate; embryo annular.

#### Distribution.

*Capsicumlongifolium* is endemic to northern Peru (Amazonas, Cajamarca, Junín and Piura Departments) and southern Ecuador (Zamora-Chinchipe Province) (Fig. 78).

#### Ecology.

*Capsicumlongifolium* is a plant of Andean montane wet forests, in the interior of primary forests in shady areas at mid-elevations (1,800–2,200 m).

#### Phenology.

Flowering and fruiting from December to August (probably all year).

#### Chromosome number.

2*n* = 2x = 26 (Barboza et al. 2019).

#### Common names.

None recorded.

#### Uses.

None recorded.

#### Preliminary conservation assessment.

EOO (67,266.225 km^2^); AOO (24 km^2^). Although *C.longifolium* has been collected many times in San Francisco Biological Reserve (SFBR, Zamora-Chinchipe, Ecuador), it is known from only five other locations in areas not included in the National System of Protected Areas where the quality of its habitat probably will decline. Based on this, we suggest a status of Vulnerable (VU; B2ab(iii).

#### Discussion.

*Capsicumlongifolium* is strongly resolved within the Andean clade (Barboza et al. 2019). It is unique in the genus in having the longest and narrowest leaves and striking calyx appendages that arise from the calyx tube as laterally compressed thick expansions or wings (Fig. 84D, E, I). Variation in corolla colour and length of the fruiting calyx appendages can be observed in the field in individuals growing under the same environmental conditions. The corolla is usually entirely yellow (Fig. 84E, H, I), but occasional plants have corolla lobes that are red- to brown-edged (Fig. 84J, K) or have a red-brown ring within the corolla throat (Fig. 84F); in this latter case, the filaments and the style are also red-brown. In general, the calyx appendages do not enlarge considerably in fruit (Fig. 4L, N), but some specimens have long appendages (Fig. 84M).

**Figure 83.**
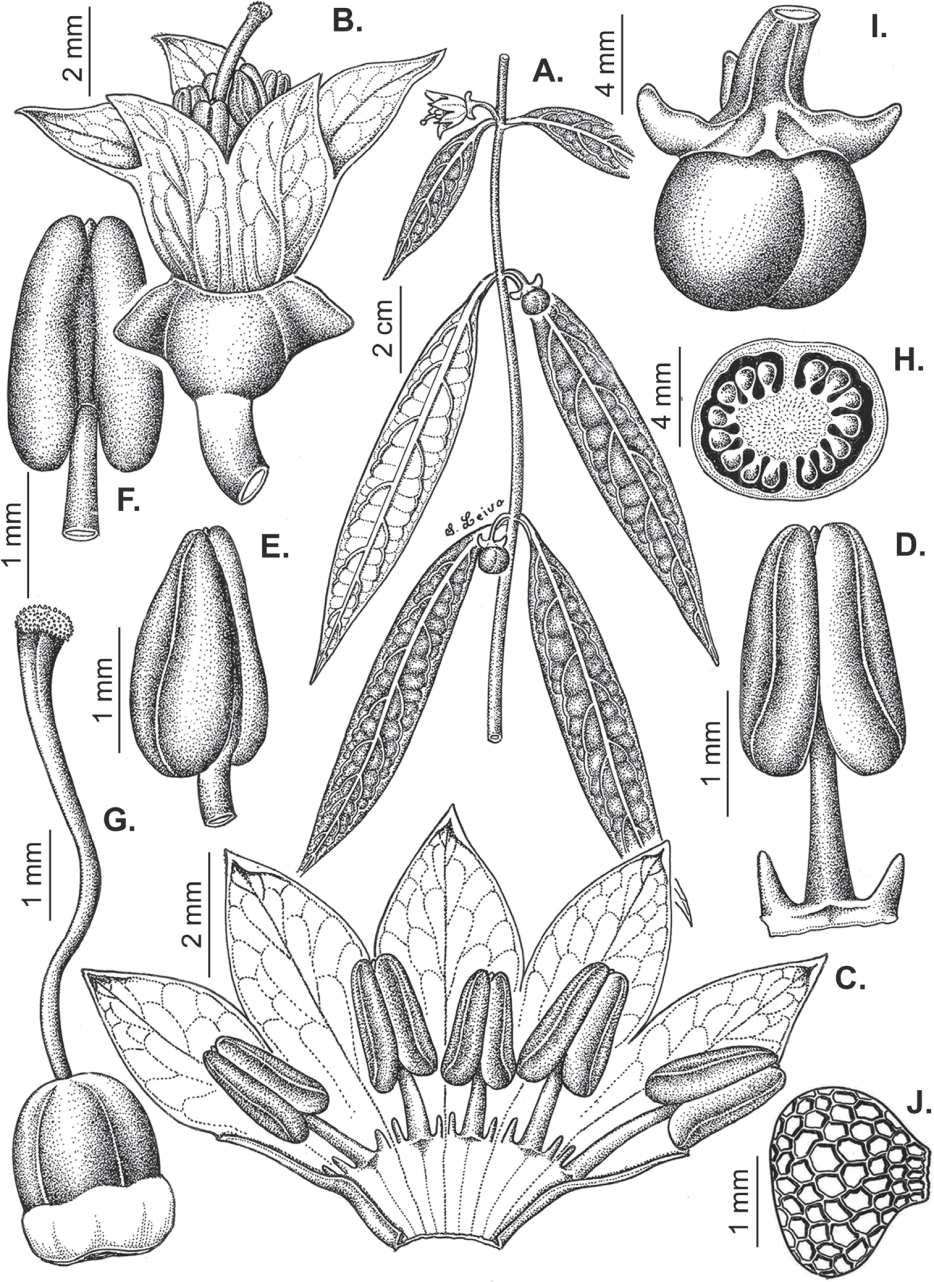
*Capsicumlongifolium***A** reproductive branch **B** flower **C** opened corolla **D, E, F** anthers, ventral, lateral and dorsal views, respectively **G** gynoecium **H** ovary, in cross section **I** fruit **J** seed. From *Barboza & Leiva González 4849.* Drawn by S. Leiva González. Published in Barboza et al. (2019), reproduced with permission.

**Figure 84.**
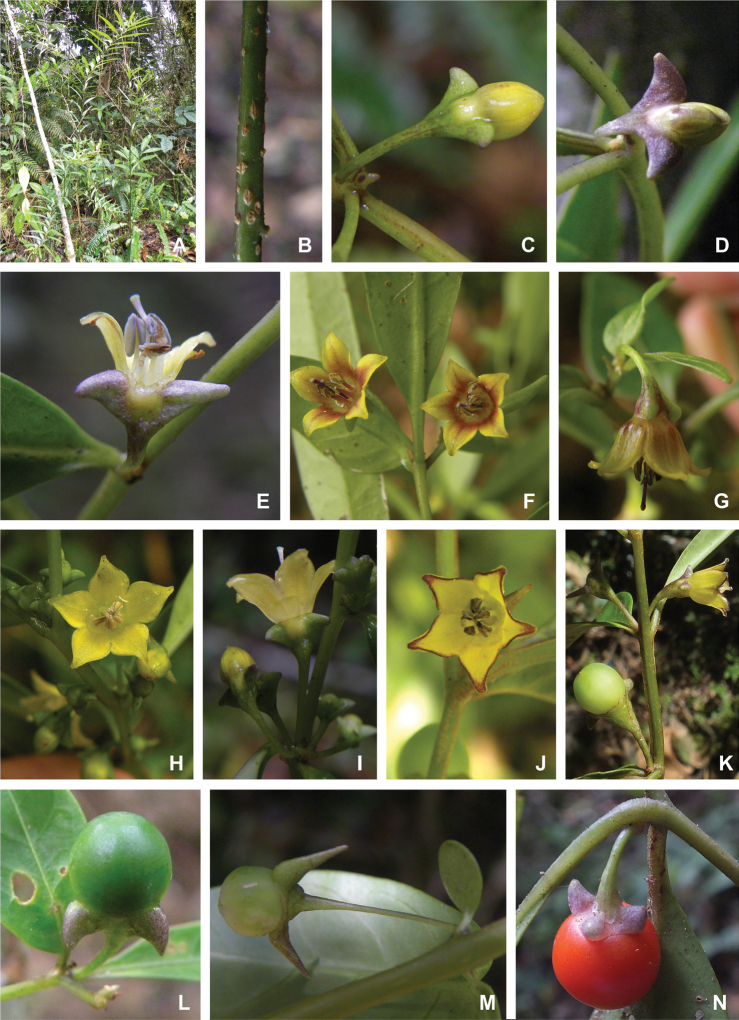
*Capsicumlongifolium***A** plant **B** internode with lenticels **C, D** flower buds **E** Flower, in longitudinal section **F** flowers showing corolla yellow with brownish centre **G** flower, in lateral view **H, I** flowers with completely yellow corollas, upper and lateral views, respectively **J, K** flowers with yellow corollas and red-brown edges, upper and lateral view, respectively **L, M** immature fruits **N** mature fruit **A, C, H, I** from *Barboza & Leiva González 4821***B, D, E, L, N** from *Barboza & Leiva González 4846***F, G** from *Barboza & Leiva González 4850***J, K, M** from *Barboza & Leiva González 4851.* Photos by S. Leiva González and G.E. Barboza. Published in Barboza et al. (2019), reproduced with permission.

*Capsicumlongifolium* is morphologically most similar to *C.dimorphum*, its sister species and to *C.regale* with which it shares the stellate yellow corollas, the non-pungent fruits and the black seeds. *Capsicumlongifolium* can be distinguished from *C.dimorphum* by having completely glabrous vegetative organs and calyces, long and narrow (ratio 6–10.8), coriaceous, major leaves, flowers 3–7 (–9) on a short rachis and calyces with 2–3 thick triangular-compressed appendages that look like wings. In contrast, *C.dimorphum* has pubescent vegetative organs and calyces, shorter and wider (ratio 4–5.25), membranous, major leaves, solitary or up to five axillary flowers and no calyx appendages or, if appendages are present, they are three and minute (Fig. 55). *Capsicumregale* differs from *C.longifolium* in its occasionally forked inflorescence (vs. unbranched in *C.longifolium*), elliptic (vs. narrowly elliptic) major leaves, dark purple pedicels in fruit (vs. green) and purple (vs. orange) berries. Another species of *Capsicum* sympatric with *C.longifolium* (especially in SFBR, Ecuador) is *C.geminifolium*; that species has a dense indumentum, long-acuminate leaves, thin calyx appendages and campanulate yellow corollas with many purple or maroon spots within (Fig. 72).

#### Specimens examined.

See Suppl. material 4: Appendix 4.

### ﻿27. Capsicum lycianthoides

Taxon classificationPlantaeSolanalesSolanaceae

Bitter, Repert. Spec. Nov. Regni Veg. 17: 332. 1921.

E61B2FD7-F34A-5838-B269-D7EDA9B66A11

Fig. 85

#### Type.

Ecuador. Chimborazo: secus v. Chasuán, Jul 1860, *R. Spruce s.n*. (holotype: W [acc. # 1889-0222993]; isotypes: CORD [CORD00101757, fragment ex K], K [K000201917]).

#### Description.

Erect slender or scandent shrubs or subshrubs, (0.50–) 1–3 (–4) m tall, few branched above, the branches dichotomously spreading in a typical “zig-zag” appearance. Young stems angled, fragile, flexuous, dark green or purple, glabrous, the new growth with few antrorse, simple, uniseriate, 3–4-celled, eglandular trichomes 0.1–0.3 mm long; nodes solid, green or purple; bark of older stems brown, glabrous; lenticels absent. Sympodial units difoliate, the leaves geminate; leaf pair markedly unequal in size and shape. Leaves coriaceous, discolorous, dark green above, pale green or purple or green with purple spots below, glabrous on both surfaces, sometimes with some antrorse eglandular trichomes on mid-vein abaxially; blades of major leaves (10–) 11–22.5 cm long, (3–) 4–8.5 cm wide, ovate or broadly ovate, rarely elliptic, the major veins (6–) 7–10 on each side of mid-vein, the base attenuate to strongly asymmetric, the margins entire, the apex acuminate or long-acuminate; petioles 0.8–2.3 (–3.5) cm long, glabrous; blades of minor leaves 2.5–5.5 (–8) cm long, 1.8–4.5 cm wide, ovate or orbicular, the major veins 3–5 on each side of mid-vein, the base truncate or rounded, the margins entire, the apex acute or obtuse; petioles 0–0.4 cm, glabrous. Inflorescences axillary, (2–) 3–8 (–10) flowers on a short rachis; flowering pedicels 8–15 mm, thin, terete, curved to pendent, non-geniculate at anthesis, green or purple, glabrous or moderately pubescent, the eglandular trichomes short, antrorse; pedicels scars conspicuous, corky. Buds ovoid, yellow or yellow-purple. Flowers 5-merous. Calyx 2–4 mm long, 3–5 mm in diameter, cup-shaped, thin or somewhat fleshy, green, greenish-purple or purple, the calyx appendages (2–) 3–5, 2–3.5 mm long, subequal, linear or subulate, sometimes like horns, spreading or reflexed, 0.3–0.5 mm below the margin, tube and appendages glabrescent with antrorse trichomes 0.3–0.5 mm long. Corolla 8–15 mm long, 15–18 mm in diameter, yellow or yellow with a purple star or dark brown spots outside and within, broadly campanulate, with a thin and wide interpetalar membrane connecting the lobes to the distal end, pentagonal in outline, shallowly lobed, glabrous adaxially and abaxially, the tube 7.5–14.5 mm long, the lobes ca. 0.5 mm long, the margin and the tips papillate. Stamens five, subequal or filaments unequal, with three filaments 1.5–2.5 mm long and two filaments 2.5–4 mm long, pale yellow or yellow-brown or whitish, inserted on the corolla ca. 2 mm from the base, with auricles fused to the corolla at the point of insertion; anthers (2.2–) 2.5–2.7 mm long, ellipsoid, whitish, yellow or light brown, not connivent at anthesis. Gynoecium with ovary 1.5–2 mm long, ca. 1.2 mm in diameter, greenish-yellow, ovoid or ellipsoid; ovules more than two per locule; nectary 0.5–0.7 mm tall; styles homomorphic, 6–7.2 mm long, exserted ca. 1.5 mm beyond the anthers, white or cream, clavate; stigma 0.3–0.4 mm long. 0.7 mm wide, usually discoid, sometimes slightly bilobed, light green or cream. Berry 7–12 mm in diameter, globose or globose-depressed, white or light green when immature, bright orange or red at maturity, deciduous, non-pungent, the pericarp thick, opaque, lacking giant cells (endocarp smooth); stone cells 1–6 or absent, ellipsoid, 0.8–1.1 mm long, 0.5–1.2 m in diameter; fruiting pedicels 13–30 mm long, pendent, terete, slightly widened distally, green or greenish-purple; fruiting calyx 5–6 mm in diameter, persistent, not accrescent, greenish-purple or purple, discoid, the appendages 3–6 mm long, 1.5 mm wide, reflexed, greenish-purple or purple. Seeds 34–75 per fruit, 1.5–1.8 mm long, 1.1–1.4 mm wide, teardrop-shaped, brownish-black to black, the seed coat reticulate (SM and SEM), the cells polygonal in shape, the lateral walls straight; embryo annular.

**Figure 85.**
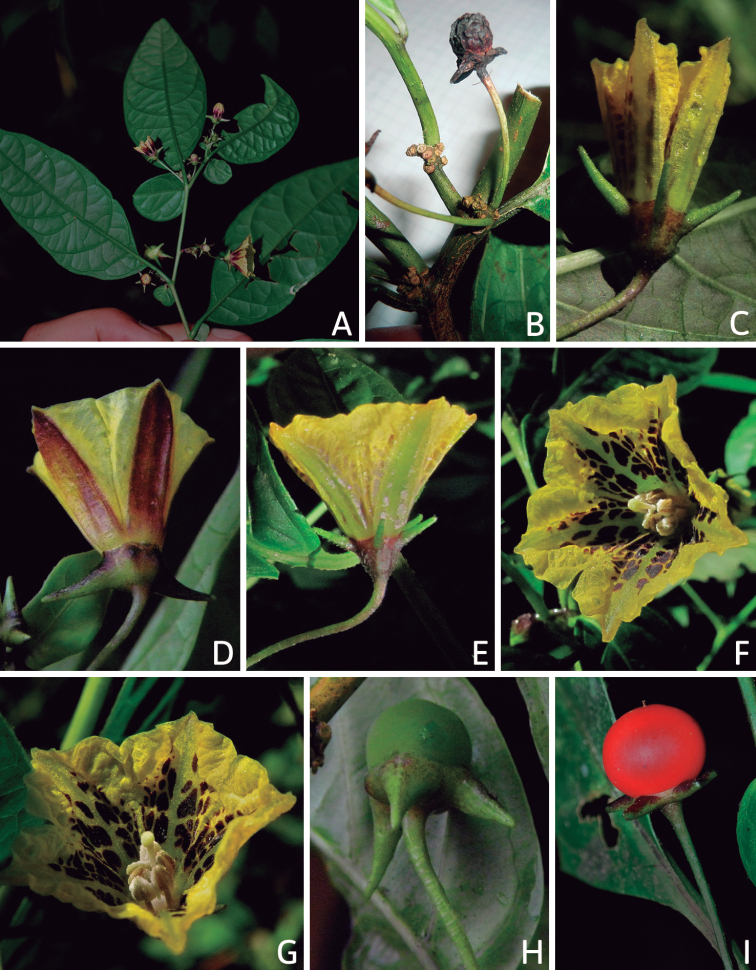
*Capsicumlycianthoides***A** flowering branch **B** reproductive nodes with pedicel scars **C** flower, in pre-anthesis **D, E** flowers, in lateral view, with variations in calyx morphology and corolla colouration **F, G** flowers, in front view **H** immature fruit **I** mature fruit **A, I** from *Beltrán 85*, photos by G. Beltrán **B, D, H** from *Deanna et al. 144***C, E–G** from *Deanna et al. 133*, photos by R. Deanna.

#### Distribution.

**Figure 86.**
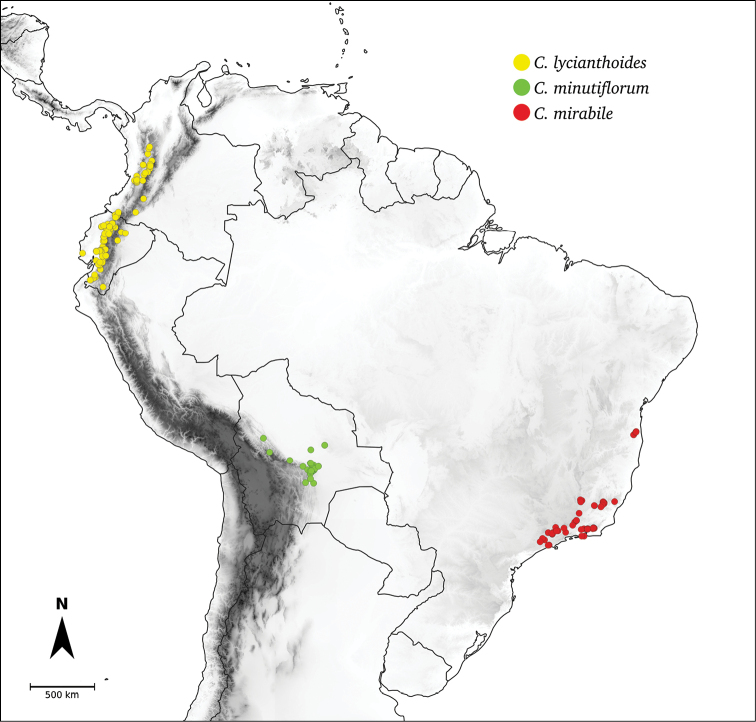
Distribution of *C.lycianthoides*, *C.minutiflorum* and *C.mirabile*.

*Capsicumlycianthoides* is endemic to the Andean Region of Colombia and Ecuador (Fig. 86).

#### Ecology.

*Capsicumlycianthoides* is a typical element of tropical montane rain forests growing in the margins or interior of primary or secondary forests in sunny or shady areas at (300–) 500–3,500 m elevation.

#### Phenology.

Flowering and fruiting all year.

#### Chromosome number.

2*n* = 2x = 26 (Scaldaferro and Moscone 2019).

#### Common names.

None recorded.

#### Uses.

None recorded.

#### Preliminary conservation assessment.

EOO (224,147.836 km^2^ – LC); AOO (492 km^2^ – EN). *Capsicumlycianthoides* is widely distributed along the Colombian and Ecuadorian Andes and is a common species where it occurs. Based on the EOO and AOO and that many collections have been made in different official and private protected areas in recent years, we assign this species the Least Concern (LC) status.

#### Discussion.

*Capsicumlycianthoides* is a member of the Andean clade (Carrizo García et al. 2016, as *C.geminifolium*; Barboza et al. 2019). It is easily recognised by its glabrous stems and leaves, large ovate to broadly ovate leaves, 2–5 calyx appendages that are sometimes horn-like, showy, broadly campanulate, yellow (often spotted dark brown) corollas and orange or red non-pungent fruits (Fig. 85).

The name *C.lycianthoides* has been ignored in literature and the species has been confused with *C.geminifolium* (Moscone et al. 2007; Barboza 2016; Carrizo García et al. 2016), a sympatric species. Both species share pendent flowering pedicels, yellow corollas and orange to red non-pungent fruits, but *C.geminifolium* is a pubescent plant with smaller membranous, elliptic or narrowly elliptic leaves, longer flowering pedicels (up to 27 mm vs. 15 mm in *C.lycianthoides*) and campanulate (stellate in outline) corollas (vs. broadly campanulate, pentagonal in ouline, in *C.lycianthoides*).

#### Specimens examined.

See Suppl. material 4: Appendix 4.

### ﻿28. Capsicum minutiflorum

Taxon classificationPlantaeSolanalesSolanaceae

(Rusby) Hunz., Huitième Congr. Int. Bot. Paris, Comptes Rend. Séances Rapp. & Commun. 1954, sect.4: 74. 1956.

3B241AA5-E2E2-530C-A58D-E3C518E75C77

Fig. 87

Bassovia minutiflora Rusby, Mem. New York Bot. Gard. 7: 343. 1927. Type. Bolivia. La Paz: Prov. Sud Yungas, Vic. Huachi, head of Beni River, 3000 ft elev., 21 Aug 1921, *H.H. Rusby 680* (holotype: NY [00138548]; isotypes: GH [GH00077005], MICH [1109880], MO [MO-1642901, acc. # 5468836], US [US00027424, acc. # 1284151]).

#### Type.

Based on *Bassoviaminutiflora* Rusby.

#### Description.

Erect slender shrubs or subshrubs (1–) 1.5–3 (–5) m tall, with the main stem thick 3–4 cm in diameter at base, few to much branched above. Young stems angled, fragile, green, sparsely pubescent, with antrorse, curved, simple, uniseriate, 3–4-celled, eglandular trichomes 0.05–0.6 mm long; nodes solid, green; bark of older stems fissured, light brown, glabrous; lenticels absent. Sympodial units difoliate, the leaves geminate; leaf pair unequal in size, similar or dissimilar in shape. Leaves membranous, concolorous or slightly discolorous, glabrous or sparsely pubescent on both surfaces, with simple, eglandular trichomes like those of the stems on margins and especially along the main veins abaxially, sometimes trichomes in tufts in the vein axils abaxially; blades of major leaves 3.5–9 (–10.5) cm long, (0.8–) 2.5–3.5 (–4.5) cm wide, ovate to elliptic, the major veins 3–4 (–6) on each side of mid-vein, the base attenuate and rather asymmetrical, the margins entire, the apex acuminate or acute; petioles 0.25–0.8 cm long, sparsely pubescent; blades of minor leaves 2.5–3.2 cm long, 0.65–1.6 cm wide, ovate, the major veins 2–3 on each side of mid-vein, the base short-attenuate, the margins entire, the apex acute; petioles 0.2–0.3 cm long, sparsely pubescent. Inflorescences axillary, 4–5-flowers per axil; flowering pedicels 1–17 (–25) mm long, terete, slightly striate, erect, geniculate at anthesis, green, sparsely pubescent, the eglandular trichomes short, antrorse; pedicels scars inconspicuous. Buds ovoid, greenish-yellow. Flowers 5-merous. Calyx 1.75–2 mm long, 2.3–2.5 mm wide, cup-shaped, thick, green, sparsely pubescent with the same eglandular trichomes as those of stems, the calyx appendages five (rarely absent), 1–1.5 mm long, equal or subequal, spreading to reflexed, erect, usually cylindrical. Corolla 6.5–8 (8.5–) mm long, 8.5–9 mm in diameter, thin, yellow outside, pale or dull yellow slightly spotted with yellowish-green within, stellate with interpetalar membrane, lobed less than halfway to the base, pubescent adaxially with sparse glandular hairs (stalk 2–3-celled; head peltate, unicellular) in the throat and the lobes; the tube 3.5–5 mm, with sparse short eglandular trichomes abaxially, the lobes 3–3.5 mm long, 1.8–2 mm wide, triangular, with sparse eglandular trichomes abaxially and in the margins, the margins involute, the tips cucullate, papillate. Stamens five, equal; filaments 2–2.5 mm long, white, inserted on the corolla ca. 1.2 mm from the base, with auricles fused to the corolla at the point of insertion; anthers 1.6–2 mm long, ellipsoid, yellow, not connivent at anthesis. Gynoecium with ovary ca. 1.2 mm long, 1.5 mm in diameter, dark green, subglobose; ovules more than two per locule; nectary ca. 0.2–0.3 mm tall; styles homomorphic, 3–3.5 mm, exserted ca. 1 mm beyond the anthers, white, clavate; stigma ca. 0.2 mm long, 0.5 mm wide, discoid, light green. Berry 7–10 mm in diameter, globose, green when immature, bright red at maturity, deciduous, pungent, the pericarp thick, opaque, with giant cells (endocarp alveolate); stone cells absent; fruiting pedicels 1.8–2.3 cm long, erect, faintly angled, widened distally, green; fruiting calyx 2.5–3 mm in diameter, persistent, not accrescent, green, discoid, with a strong annular constriction at junction with the pedicel, the appendages up to 1.4 mm long, spreading or reflexed. Seeds 5–19 per fruit, 4–4.5 mm long, 3–3.5 mm wide, C-shaped, ellipsoid or subglobose, dark brown, the seed coat diffusely reticulate and slightly tuberculate at margins (SM), reticulate or reticulate-cerebelloid (SEM), the cells irregular in seed body and polygonal at margins, the lateral walls sinuate in the seed body and straight and wavy at margins; embryo imbricate.

#### Distribution.

*Capsicumminutiflorum* is endemic to central Bolivia (Cochabamba, La Paz, Santa Cruz Departments) (Fig. 86).

#### Ecology.

*Capsicumminutiflorum* is an uncommon plant that grows in the subtropical semi-deciduous forest (Chiquitano Forest) and transitional forests to the Yungas on slopes or along streams and occasionally on road-sides, between 350 and 1,500 (–2,800) m elevation.

#### Phenology.

Flowering from October to May; fruiting from November to July.

#### Chromosome number.

Not known.

#### Common names.

None recorded.

#### Uses.

None recorded, but it is likely that the fruits are used as spices due to their similarities in colour and pungency with C.baccatumvar.baccatum.

#### Preliminary conservation assessment.

EOO (101,632.300 km^2^); AOO (108 km^2^). *Capsicumminutiflorum* has a relatively large extent of occurrence growing in more than ten localities; it can be assigned the preliminary Least Concern (LC) category. However, this species is of some conservation concern because of its relatively small AOO and the small number of records in protected areas (Amboro National Park, Santa Cruz).

#### Discussion.

*Capsicumminutiflorum* is a member of the Bolivian clade (Carrizo García et al. 2016). This poorly-known species has few collections and is one of the five Bolivian species (with *C.caballeroi*, *C.ceratocalyx*, *C.coccineum* and *C.neei*) with yellow corollas. The calyx normally has five short appendages, but in some specimens (*Buchtien 2231* and *Vargas C. et al. 2173*), they are lacking.

**Figure 87.**
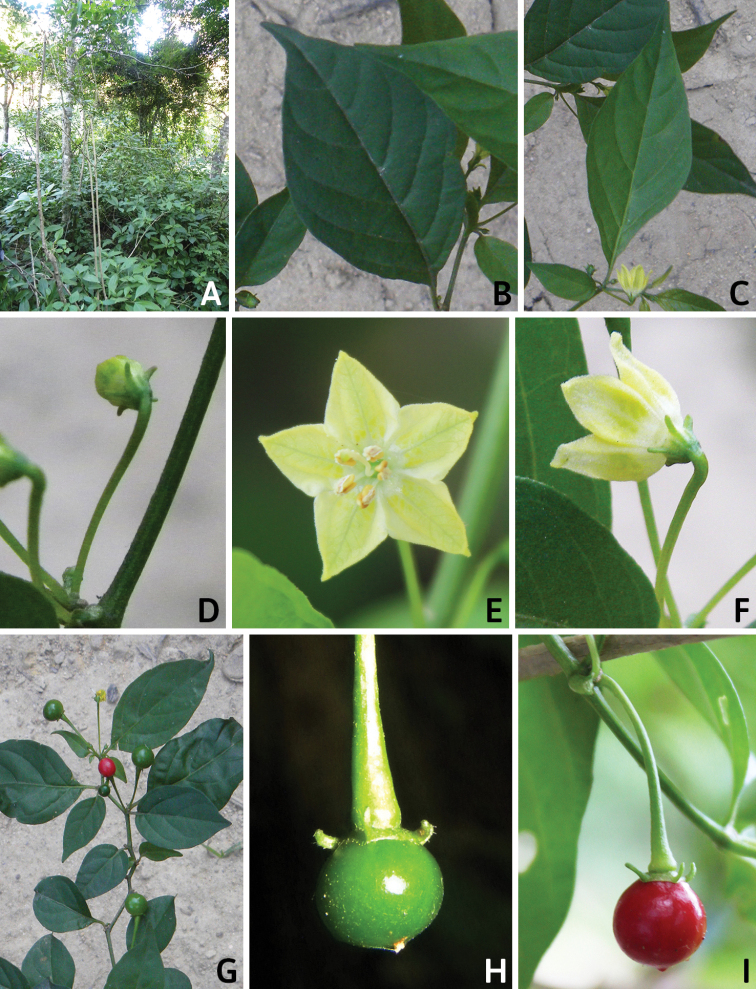
*Capsicumminutiflorum***A** plant **B** major leaf **C** flowering branch **D** flower bud on geniculate pedicel **E** flower, in front view **F** flower, in lateral view **G** fruiting branch **H** immature fruit **I** mature fruit. From *Barboza et al. 4918.* Photos by G.E. Barboza.

*Capsicumminutiflorum* is often confused with the sympatric C.baccatumvar.baccatum and *C.neei.* All these three taxa share the few-flowered inflorescences and the red pungent fruits. *Capsicumminutiflorum* and *C.neei* can be distinguished by its yellow corollas with greenish-yellow spots within (vs. white corollas with greenish-yellow spots within in C.baccatumvar.baccatum), this character not being evident in herbarium specimens. In addition, *C.minutiflorum* (Fig. 87) and *C.neei* (Fig. 95) plants are few-branched taller shrubs with globose fruits. In contrast, *C.baccatum* plants can be highly-branched perennial herbs, subshrubs or shrubs growing up to 1.5 m (rarely more) and have globose to ellipsoid fruits (Fig. 26). *Capsicumminutiflorum* differs from *C.neei* in its geniculate and erect flowering pedicels and a weakly nerved calyx with five equal, short appendages, while *C.neei* has non-geniculate, pendent flowering pedicels and strongly-nerved calyx with 10 unequal appendages. Another sympatric species is the more widespread *C.coccineum*, which differs from *C.minutiflorum* by its peculiar sprawling or scrambling habit, its showy multi-flowered inflorescences and its calyx with 0–10 unequal appendages (Fig. 50).

#### Specimens examined.

See Suppl. material 4: Appendix 4.

### ﻿29. Capsicum mirabile

Taxon classificationPlantaeSolanalesSolanaceae

Mart., Fl. Bras. (Martius) 10(6): 144. 1846.

3C4656A4-1B05-589B-9090-C37A677B4F1E

Figs 88 , 89

Capsicum mirabile Mart. var. grandiflorum Sendtn., Fl. Bras. (Martius) 10(6): 144. 1846. Type. [Brazil]. Brasilia, *F. Sellow 209* (possible original material B, destroyed [F neg. 2871], no additional material found).
Capsicum buforum Hunz., Kurtziana 5: 394. 1969. Type. Brazil. São Paulo: Eugênio Lefévre, a unos 26 km de Campos do Jordão, ca. 1400 m elev., 5 Dec 1967, *A.T. Hunziker 19562* (lectotype, designated here: CORD [CORD00006616]; isolectotypes: CORD [CORD00006617, CORD00006618]).

#### Type.

Brazil. “Habitat in sylvis fere ubique per Provinciam Sebastianopol. et Paulinam”, Dec., *C.F.P. von Martius s.n.* (lectotype, designated by Barboza 2011, pg. 30: M [M-0171538]).

#### Description.

Erect shrubs or subshrubs (0.70–) 1.2–2.5 (–3) m tall or rarely small trees, with the main stem thick 2.5–4 cm in diameter at base, much branched above, the branches dichotomously spreading in a typical “zig-zag” appearance. Young stems angled, fragile, green, glabrous or glabrescent, with antrorse, curved, simple, uniseriate, 3–4-celled, eglandular trichomes 0.1–0.3 mm long, new growth with sparse whitish pubescence; nodes purple, slightly purple or green; bark of older stems dark brown, angled, glabrous; lenticels absent. Sympodial units difoliate, the leaves geminate; leaf pair unequal in size, similar in shape. Leaves membranous to coriaceous, slightly discolorous to discolorous, green above, light green beneath, glabrous adaxially, glabrescent abaxially and margins, especially on the veins, with sparse, 2–3-(–6)-celled, eglandular trichomes 0.2–0.5 mm long; blades of major leaves 5–13.5 cm long, (1–) 1.3–3 (–5.5) cm wide, elliptic or narrowly elliptic to ovate, the major veins 4–6 (–7) on each side of mid-vein, the base asymmetric and attenuate, the margins entire, the apex acuminate to long-acuminate; petioles 0.7–2 (–2.5) cm long, glabrous; the blades of minor leaves 2–3.3 (–4) cm long, 0.9–1.7 cm wide, elliptic or ovate, the major veins 3–4 on each side of mid-vein, the base attenuate, the margins entire, the apex acute; petioles 0.2–0.4 cm long, glabrous. Inflorescences axillary, 2–5 (–7) flowers per axil, rarely flowers solitary; flowering pedicels (13–) 16–25 mm long, terete or slightly angled, erect or slightly spreading, geniculate at anthesis, green or greenish-purple, glabrous or glabrescent, the eglandular trichomes short, antrorse; pedicels scars inconspicuous. Buds globose to ovoid, inflated, greenish-purple or dark purple or clearer near anthesis. Flowers 5-merous. Calyx 1.5–2.2 mm long, ca. 2–2.5 mm wide, cup-shaped, thin, yellowish-green or green, glabrous or glabrescent, the calyx appendages five, (0.4–) 0.5–1.5 (–3) mm long, subequal, thick, green, erect or spreading, cylindrical, inserted very close to the margin. Corolla (6–) 7.5–12 mm long, (9–) 10–13 mm in diameter, thick, purple with white margin and yellowish-green at the base outside, similar colour within, but sometimes the purple or reddish-brown spots diffuse up to absent, stellate with interpetalar membrane, lobed halfway or less of the way to the base, the tube 3.5–5 mm long, with a continuous ring of glandular trichomes (stalk long, 1–3-celled; head globose, peltate, unicellular) adaxially, glabrous abaxially, the lobes 3–5 mm long, 3.2–4 mm wide, broadly triangular, spreading, glabrous abaxially and adaxially, the margins papillate, the tips cucullate, papillate. Stamens five, equal; filaments (2.8–) 3–3.8 (–4) mm long, equal, light green or greenish-white, inserted on the corolla 1–1.8 mm from the base, with auricles fused to the corolla at the point of insertion; anthers 1.5–2 mm long, ellipsoid, yellow when young and reddish-brown or grey-purple post-dehiscent, not connivent at anthesis. Gynoecium with ovary 1.1–1.5 mm long, 1–1.2 mm in diameter, green, globose to ovoid; ovules more than two per locule; nectary ca. 0.3 mm tall; styles homomorphic, 3.5–4.7 mm long, at the same level or barely exserted beyond the anthers, yellowish-white or light green, clavate; stigma 0.2 mm long, 0.6–0.8 mm wide, discoid or globose, pale green. Berry 7–9 mm in diameter, globose, green when immature, greenish-golden yellow, deciduous, pungent, the pericarp thin, translucent, with giant cells (endocarp alveolate); stone cells absent; fruiting pedicels 20–32 mm long, pendent and curved, angled, widened and with a slight constriction distally, green; fruiting calyx 3–3.5 mm in diameter, persistent, not accrescent, green, discoid, the appendages 0.7–3.5 mm long, 0.3–0.4 mm wide, spreading. Seeds (2–) 3–8 (–10) per fruit, 2.5–3.2 mm long, 2–2.5 mm wide, C-shaped, brownish-black to black, the seed coat reticulate and tuberculate at margins (SM), reticulate with pillar-like outgrowths at margins (SEM), the cells polygonal in shape, the lateral walls straight; embryo coiled.

#### Distribution.

This species is endemic to east and south-eastern Brazil (Bahia, Espírito Santo, Minas Gerais, Rio de Janeiro and São Paulo States) (Fig. 86).

#### Ecology.

*Capsicummirabile* is found in the understorey, edges and interior of the wet Atlantic Forest (Mata Atlântica), primarily in the Floresta Ombrófila Densa Montana, at 800–1,900 m elevation.

#### Phenology.

Flowering from November to April and fruiting from December to May.

#### Chromosome number.

*n* = 13 (Tong and Bosland 2003; Pozzobon and Schifino-Wittmann 2006, as *Capsicum* sp 6 and *C.buforum*); 2*n* = 2x = 26 (Moscone et al. 2007).

#### Common name.

**Brazil**: Pimenta do sapo (São Paulo, *Hunziker 19562*).

#### Uses.

None recorded.

#### Preliminary conservation assessment.

EOO (190,248.624 km^2^); AOO (224 km^2^). Based on the large extent of occurrence, as well the many collections made in several protected areas (Parque Nacional Serra das Lontras, PN do Caparaó, Parque Estadual Serra do Brigadeiro, PN Itatiaia, Parque Estadual Pico do Itacolomi, Reserva Florestal Uaimi, Parque Estadual Três Picos, PN da Serra dos Órgãos, Parque Estadual Maciço da Pedra Branca, Estação Biológica de Boracéia and PN Serra do Bocaina), we assign *Capsicummirabile* the Least Concern (LC) category. Subpopulations are not rare in the interior of forests, but this species could be at risk due to the fragmentation and loss of its primary forest habitat outside the legal conservation units.

#### Discussion.

*Capsicummirabile* is an enigmatic and morphologically variable species of the Brazilian Atlantic Forest clade (Carrizo García et al. 2016) and its interspecific relationships are still not well understood. Smith and Downs (1966) confused plants of *C.recurvatum* (e.g. *Smith 7974*, *Reitz & Klein 5766*, *5943* and *Klein 2232*) and *Athenaea* spp. (e.g. *Reitz 4417*, *Ule 699*, *Klein 2417* and others) from Santa Catarina, Brazil, with *C.mirabile*, a region where this latter species does not occur. Furthermore, Hunziker’s use of the epithet *mirabile* in herbaria has been erratic, making it difficult to understand its real circumscription. Initially, Hunziker (*in litt.*) had a broad and inclusive concept for this species, which motivated him to annotate specimens he considered different, but related to *C.mirabile* with the imprecise name “*C.mirabile* var.”. Later, Hunziker (2001) accepted six species for eastern Brazil (C.baccatumvar.praetermissum, *C.campylopodium*, *C.dusenii* (here a synonym of *C.cornutum*), *C.mirabile*, *C.schottianum* and *C.villosum*), but he did not mention *C.buforum* which he described in 1969 and here is treated as a synonym of *C.mirabile*. Both names, *C.mirabile* and *C.buforum* have been used in literature for accessions used in cytogenetic studies (Tong and Bosland 2003; Pozzobon and Schifino-Wittmann 2006; Pozzobon et al. 2006; Moscone et al. 2007). Barboza and Bianchetti (2005) and Barboza et al. (2011, 2020a) provided identification keys for the Brazilian species, finally resolving the ambiguities surrounding *C.mirabile*.

**Figure 88.**
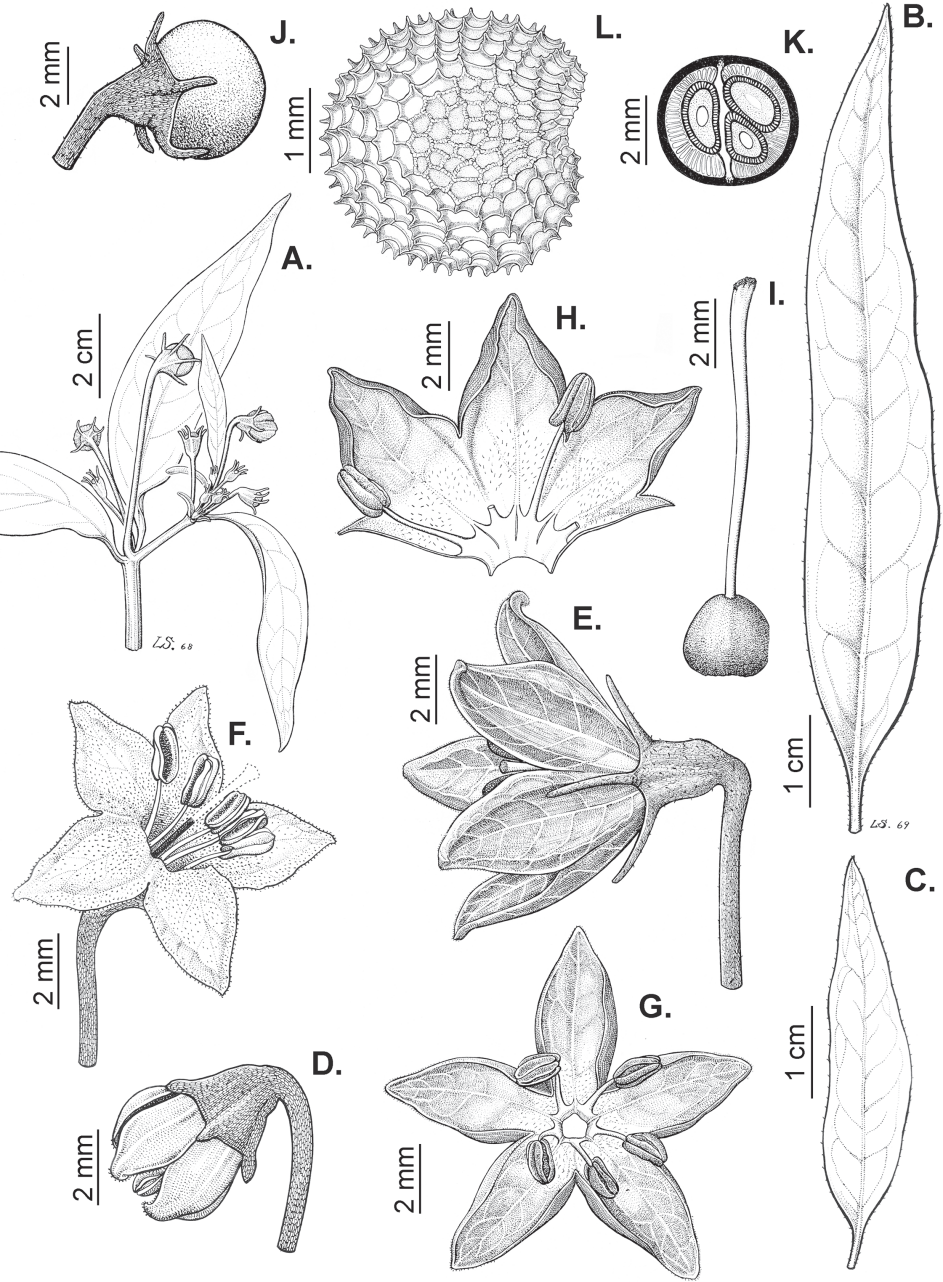
*Capsicummirabile***A** flowering branch **B, C** major and minor leaf **D** flower bud **E** flower, in lateral view **F, G** flowers, upper views **H** sector of opened corolla **I** gynoecium **J** fruit **K** fruit, in cross section **L** seed. From *Hunziker 19562.* Drawn by L. Sánchez.

**Figure 89.**
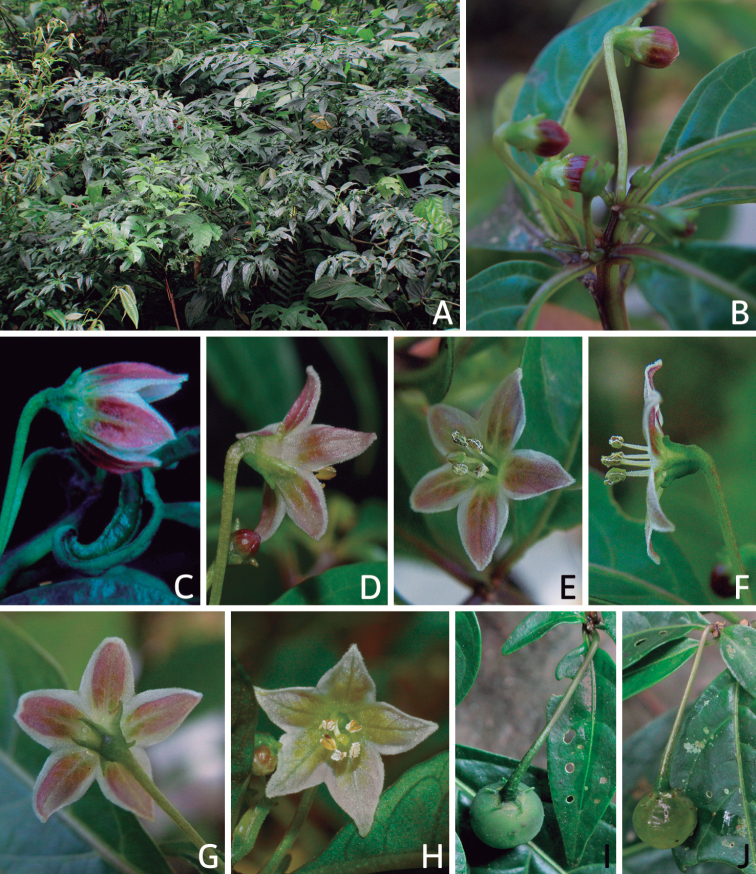
*Capsicummirabile***A** plant **B** flower buds on geniculate pedicels **C** flower, in pre-anthesis **D** flower, seen from behind **E, H** flowers, in front view, showing corollas with different colouration **F** flower, in full anthesis **G** flower, seen from behind **I** immature fruits **J** mature fruit **A, B, D–J** from *Barboza & Deanna 5024***C** from *Bianchetti & Bustamante 1518***A, B, D–H** photos by G.E. Barboza **C** photo by L. Bianchetti **I, J** photos by R. Deanna.

*Capsicummirabile* is characterised by shrubby habit with dichotomous branching, glabrous or glabrescent pubescence, short petioles, elliptic to ovate leaves, few flowers (2–7) per node, five short to long calyx appendages, purple corolla with white margin and yellowish-green or yellowish-white centre and greenish-golden yellow fruits (Table 4, Fig. 89).

The distinctive corolla spots of this species were not mentioned in the protologue (Martius 1846), although the other diagnostic characters were clearly stated; this may have introduced confusion into the understanding of this species. Variations in some of the morphological features have been observed in the field, such as the length of the calyx appendages, which can range from very short (< 1 mm long, Rio de Janeiro, Espírito Santo and Minas Gerais States) to longer (up to 3 mm, São Paulo and Minas Gerais States) and the shape of the leaves (usually elliptic, but also ovate). The most variable trait is the extent and intensity of tones of the reddish-brown spots in the corolla (Fig. 89C–H), covering partially or almost completely the lobes within (Carrizo García et al. 2013 and see photographs in http://www.pepperfriends.org/dbpf/capsicum-mirabile_001.asp and http://www.pepperfriends.org/dbpf/capsicum-mirabile_003.asp). Variation in the corolla colour has also been observed in specimens in cultivation at the Federal University of Viçosa (seeds from Espírito Santo). Each corolla lobe had a unique purplish spot in some specimens (*Hunziker 25235*) or two intense purple spots (*Hunziker 25243*) or the spots were scarcely purple or nearly lacking all together (*Hunziker 25236*).

*Capsicummirabile* is sympatric with *C.friburgense* near the top of Cerro Calêdonia (Nova Friburgo, Rio de Janeiro) where the latter is endemic. Both species share geniculate pedicels, a calyx with five appendages, greenish-golden yellow fruits and blackish-brown or black seeds. They are easily told apart by the shape and colour of the corolla (stellate and multi-coloured in *C.mirabile* vs. campanulate-urceolate and entirely lilac or pink in *C.friburgense*) and the leaf shape (elliptic to ovate in *C.mirabile* vs. ovate in *C.friburgense*).

The two species similar to *C.mirabile* are *C.villosum* and *C.muticum*. The main difference amongst the three species is the pubescence, since *C.mirabile* is almost completely glabrous (or sparsely pubescent) and the other two species are densely pubescent on stems, leaves, pedicels and calyx. *Capsicumvillosum* has five calyx appendages (as *C.mirabile*), but the corolla is smaller and the lobes are more white than purple and the greenish-yellow centre is more extensive in comparison with *C.mirabile*. *Capsicummuticum* lacks calyx appendages and the purple pigmentation is very scarce, as lines or small spots bordering the greenish-yellow centre of the corolla.

When Hunziker (1969b) described *C.buforum*, he stated that *Hunziker 19562* was “Typus speciei (CORD)”. Three sheets of this collection are held in CORD, but none of them has an annotation indicating intent by Hunziker; we select here the best of these (CORD00006616) as the lectotype.

**Figure 90.**
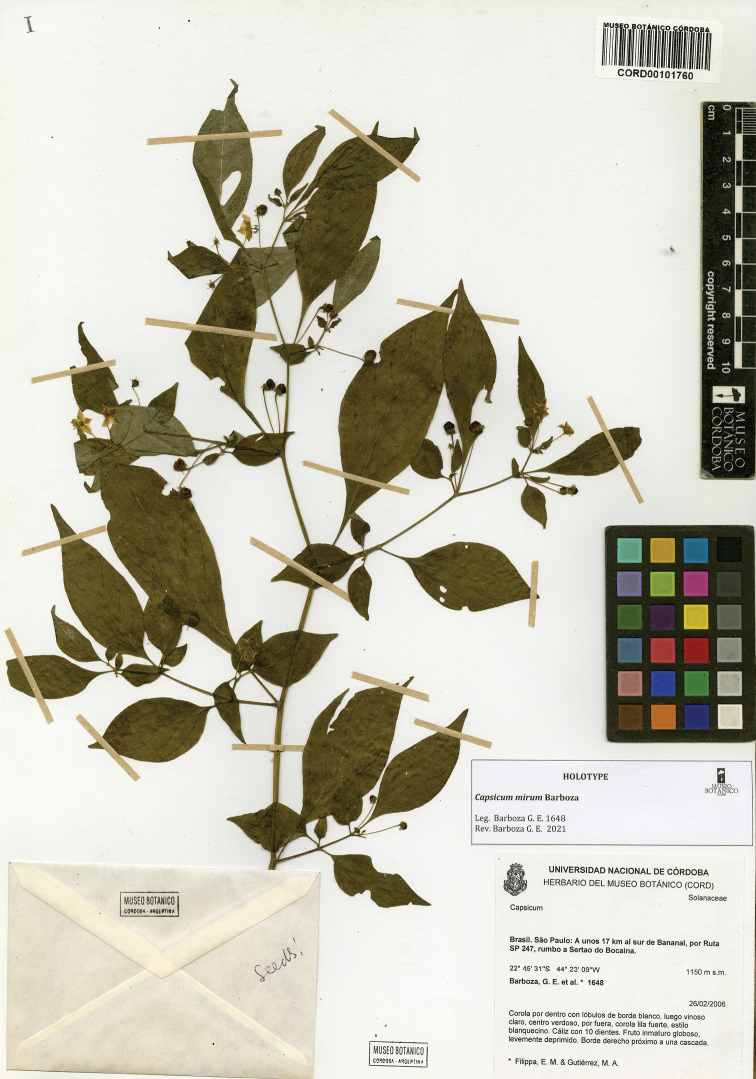
*Capsicummirum*. Holotype (CORD). From *Barboza et al. 1648*. Copyright Universidad Nacional de Córdoba, Argentina. Reproduced with permission.

#### Specimens examined.

See Suppl. material 4: Appendix 4.

### ﻿30. Capsicum mirum

Taxon classificationPlantaeSolanalesSolanaceae

Barboza sp. nov.

2E1483E4-7999-57A3-92D4-38B644FCDC94

urn:lsid:ipni.org:names:77299337-1

Fig. 91

#### Diagnosis.

*Capsicummirum* is morphologically most similar to *C.cornutum* (Hiern) Hunz., but differs in having antrorse pubescence on stems and leaves, longer petioles, a 2–3-flowered inflorescence, shorter pedicels, purple buds, subequal calyx appendages, smaller corollas almost entirely purplish outside and within and longer filaments.

#### Type.

Brazil. São Paulo: Mun. Bananal, a unos 17 km al sur de Bananal, por ruta SP 247, rumbo a Sertão do Bocaina, 22°45'31"S, 44°23'09"W, 1150 m elev., 26 Feb 2006, *G.E. Barboza, E.M. Filippa, A. Gutierrez & G. Bertone 1648* (fl, fr) (holotype: CORD [CORD 00101760], isotypes: BHCB [BHCB 195615], CORD [CORD00101760]).

#### Description.

Erect to compact shrubs 1.5–2 (–3) m tall, much branched above, the branches dichotomous, much spreading horizontally and leafy. Young stems angled, fragile, green, densely pubescent with flexuous, antrorse, curved, simple, uniseriate, 4–7-celled, eglandular trichomes 0.3–0.8 mm long; nodes solid, purple; bark of older stems brown, moderately pubescent; lenticels absent. Sympodial units difoliate, the leaves geminate, the leaf pair unequal in size and similar or dissimilar in shape. Leaves membranous, slightly discolorous, green adaxially, pale green with the mid-vein and the secondary veins prominent abaxially, moderately pubescent on both surfaces and margins, abaxial veins densely pubescent with antrorse, curved, 3–6-celled, eglandular trichomes; blades of major leaves 7–10.5 cm long, 2.5–3.5 cm wide, elliptic or ovate, the major veins 5–6 on each side of mid-vein, the base unequal and attenuate, the margins entire, the apex acute to acuminate; petioles 1.4–2.5 cm long, green adaxially and abaxially, moderately pubescent, the trichomes antrorse; blades of minor leaves 4.5–5.7 cm long, 2.5–2.7 cm wide, ovate, the major veins 3–4 on each side of mid-vein, the base attenuate or rounded, the margins entire, the apex acute; petioles 0.8–1 cm long, green, moderately pubescent, the trichomes antrorse. Inflorescences axillary, 2–3 flowers per axil or flowers solitary; flowering pedicels 12–17 mm long, thin, angled, erect to slightly spreading, geniculate at anthesis, green or with purple lines, the youngest purple, densely pubescent, the eglandular trichomes long and spreading, the glandular trichomes sparse, small; pedicels scars inconspicuous. Buds globose to ovoid, purple to strongly purple and green at the base. Flowers 5-merous. Calyx 1.8–2 mm long, ca. 2.5 mm wide, cup-shaped, circular in outline, fleshy, green, strongly 10-nerved, the veins purple, densely pubescent with short antrorse eglandular trichomes, the calyx appendages 10, (1.7–) 2–3.2 mm long, subequal, thin, spreading, cylindrical, green, inserted very close to the margin, densely pubescent with spreading, long, eglandular trichomes. Corolla 6–8 mm long, 11–14 mm in diameter, almost entirely purple, with a thin white margin and yellowish-green centre outside and within, stellate with thin interpetalar membrane, lobed halfway to the base, pubescent adaxially with glandular trichomes (stalk long, 2–3-celled; head globose, peltate, unicellular) in the throat and base of the lobes, glabrous abaxially, the tube 2–3 mm long, the lobes 4–5 mm long, 3.5–4 mm wide, triangular, the margins with short eglandular trichomes, the tips papillate or with short eglandular trichomes. Stamens five, equal; filaments 3–3.2 mm long, white, inserted on the corolla ca. 1 mm from the base, with auricles fused to the corolla at the point of insertion; anthers 1.3–1.6 mm long, ellipsoid, lilac or pale blue, with a broad cream connective before opening, not connivent at anthesis. Gynoecium with ovary ca. 1.2 mm in diameter, light green, globose; ovules more than two per locule; nectary ca. 0.5 mm tall, paler than the ovary; styles homomorphic, 3.5–5 mm long, barely exserted beyond the anthers, cream, clavate; stigma 0.2–0.3 mm long, ca. 0.8–0.9 mm wide, discoid, cream. Berry globose, green when immature (mature berries not seen), pungent, the pericarp with giant cells (endocarp alveolate), stone cells absent; fruiting pedicels 20–30 mm long, pendent and curved, angled, slightly widened at the apex, green; fruiting calyx 3–4 mm in diameter, persistent, not accrescent, discoid, green or purple, the appendages 2.5–3.5 mm long, spreading, straight or slightly curved. Seeds 9–12 per fruit, 2.4–3.2 mm long, 1.8–2.3 mm wide, C-shaped, brownish-black to black, the seed coat reticulate and tuberculate at margins (SM), reticulate with pillar-like outgrowths at margins (SEM); the cells irregular in shape, the lateral walls sinuate, polygonal at margins; embryo imbricate.

**Figure 91.**
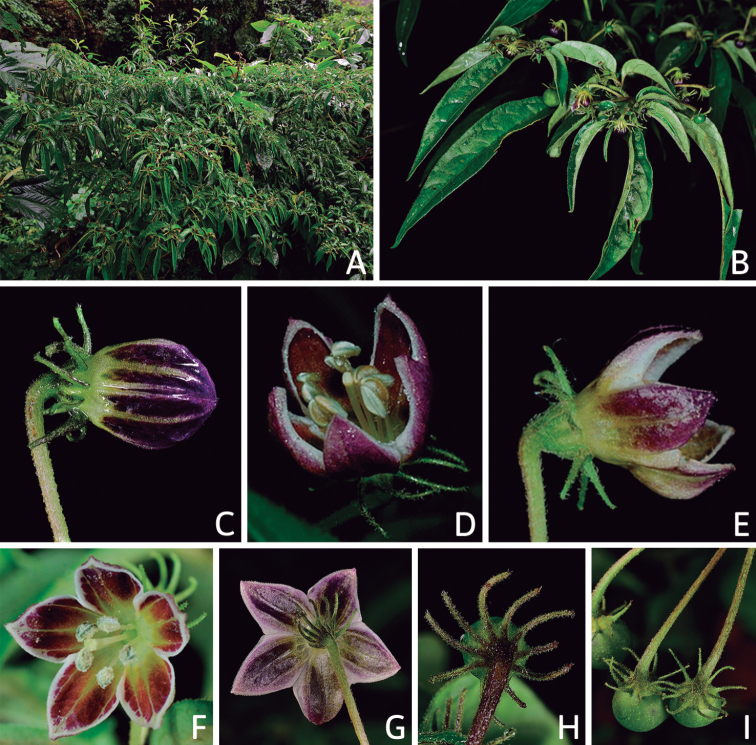
*Capsicummirum***A** plant **B** flowering branch **C** bud on geniculate pedicel **D** flower in pre-anthesis **E** flower, lateral view **F** flower, in front view **G** flower, seen from behind **H** fruiting calyx **I** immature fruits. No specimen vouchers. Photos taken *in situ* by J. Lackey (Associazione PepperFriends).

#### Distribution.

**Figure 92.**
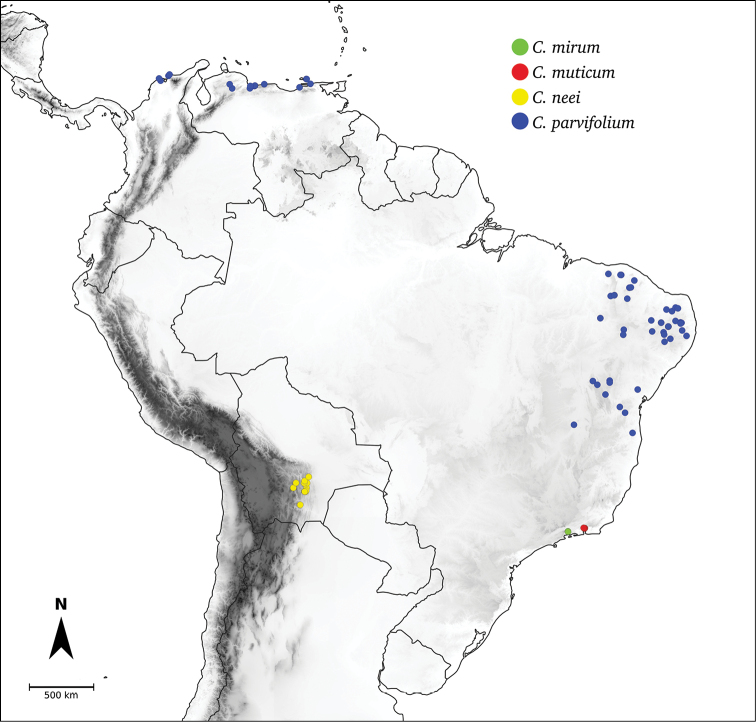
Distribution of *C.mirum*, *C.muticum*, *C.neei* and *C.parvifolium*.

*Capsicummirum* occurs in a very restricted area in south-eastern Brazil (São Paulo State) (Fig. 92).

#### Ecology.

*Capsicummirum* occurs in small populations in the coastal Atlantic Forests (Mata Atlântica), at the margins of the forest usually in the sun, between 1,100 and 1,300 m elevation.

#### Phenology.

The species has been collected in flower and in immature fruits (but mature seeds) in February and April.

#### Chromosome number.

Not known.

#### Etymology.

The specific epithet comes from the Latin *mirus* (wonderful, extraordinary), referring to the remarkable beauty of this species given by the colourful corolla and the showy calyx.

#### Common names.

None recorded.

#### Uses.

None recorded.

#### Preliminary conservation assessment.

EOO (1.205 km^2^); AOO (8 km^2^). *Capsicummirum* is the species with the narrowest extent of occurrence in the genus and with only three collections probably belonging to a single population. The species has been collected in an unprotected clearing of the Atlantic Forest where a continuing decline of the mature individuals has been observed. According to IUCN criteria, *C.mirum* is proposed as a Critically Endangered species (CR; B1ab; C2a(i)).

#### Discussion.

*Capsicummirum* belongs to the Atlantic Forest clade (Carrizo García et al. 2016, as *C.hunzikerianum*). It is a beautiful species due to its profuse flowering branches (Fig. 91A), the intensely purple buds and corollas (Fig. 91C–G) and the long and spreading calyx appendages (Fig. 91E, H, I). Although *C.mirum* is a very attractive plant, it has been poorly collected.

*Capsicummirum* is morphologically similar to *C.cornutum*, *C.mirabile* and *C.carassense* with which it shares geniculate flowering pedicels at anthesis, stellate corollas, pungent fruits, brownish-black to black seeds with seed coat reticulate and tuberculate at margins and similar habitat. Differences between these species are provided in Table 4.

*Capsicummirum* (as *C.hunzikerianum*) resolved as sister to *C.schottianum* (Carrizo García et al. 2016). The two species have an overlapping range in São Paulo and their habitats are close enough for the two species to interact. *Capsicumschottianum* is easily differentiated from *C.mirum* by its glabrescent to moderate pubescence, greenish-white buds, lack of calyx appendages (or five minute appendages) and mostly white corolla with a different intensity of the purple spots within (Fig. 116B–H). No intermediate individuals have been found in the area, but intensive field explorations are needed to better understand the range of *C.mirum* and if it hybridises with *C.schottianum*.

#### Paratypes.

Brazil. São Paulo: Mun. Bananal, a unos 17 km al sur de Bananal, por ruta SP 247, rumbo a Sertão do Bocaina, 22°45'50"S, 44°23'35"W, 1150 m elev., 26 Feb 2006 (fl, fr), *G.E. Barboza et al. 1649* (CORD); do cruzamento de Bananal rumo à Estacão Ecológica de Bananal, 22°46'55.8"S, 44°22'41.1"W, 1333 m elev., 5 Apr 2018 (fl, fr), *J.R. Stehmann et al. 6474* (= *G.E. Barboza & R. Deanna 5028*) (BHCB).

### ﻿31. Capsicum muticum

Taxon classificationPlantaeSolanalesSolanaceae

(Sendtn.) Barboza comb. nov.

194B06AE-625C-5490-B306-230585B1C458

urn:lsid:ipni.org:names:77299338-1

Fig. 93

Capsicum villosum Sendtn. var. muticum Sendtn., Fl. Bras. (Martius) 10(6): 144. 1846. Type. [Brazil. Rio de Janeiro]: “Serra d’Estrella,” [no date], *H.W. Schott 5416* (lectotype designated by Barboza 2011, pg. 31: W [acc. # 0074661]); isolectotype: CORD [CORD00006937]).
Bassovia leptopoda Dunal, Prodr. [A. P. de Candolle] 13(1): 411. 1852. Type. [Brazil. Rio de Janeiro]: Rio de Janeiro, 1819, F. Sellow s.n. (holotype: BM [BM000798808]; isotypes: G-DC [G00131651], MPU [MPU023056]).
Capsicum leptopodum (Dunal) Kuntze, Revis. Gen. Pl. 2: 449. 1891. Type. Based on Bassovialeptopoda Dunal.
Capsicum villosum forma vimineum Wawra, Itin. Princ. S. Coburgi 1: 100. 1883. Type. Brazil. Rio de Janeiro: “Petropolis; an gerodeten Stellen (Benod). *Coll II 10*”, 1879 [coll. Princes August and Ferdinand of Saxe-Coburg] (no material found).

#### Type.

Based on CapsicumvillosumSendt.var.muticum Sendtn.

#### Description.

Erect shrubs or subshrubs (0.80–) 1–2.5 m tall, with the main stem ca. 1.5 cm in diameter at base, much branched above, the branches dichotomously spreading in a typical “zig-zag” appearance. Young stems angled, fragile, green, densely pubescent with spreading, flexuous to rigid, white or slightly ferruginous (dried specimens), simple, uniseriate, (3–) 4–8-celled, eglandular trichomes 0.5–2 mm long; nodes solid, green or light purple; bark of older stems brown or dark brown, with ferruginous pubescence; lenticels few. Sympodial units difoliate, the leaves geminate; leaf pair markedly unequal in size, similar or dissimilar in shape. Leaves membranous, slightly discolorous, dark green above, light green beneath, moderately pubescent adaxially, with appressed-antrorse trichomes similar to those of the stems, densely pubescent abaxially and margins, with appressed (on the lamina) or spreading (on the veins), flexuous, 4–6-celled, eglandular trichomes 0.5–1.7 mm long; blades of major leaves 4.5–11 cm long, (1.5–) 2–3.6 cm wide, elliptic or narrowly elliptic, the major veins 5–8 on each side of mid-vein, the base attenuate, slightly unequal, the margins entire, the apex acuminate; petioles 0.5–1.5 (–2) cm long, densely pubescent; blades of minor leaves 2–4.3 cm long, 1–1.4 cm wide, elliptic, the major veins 3–4 on each side of mid-vein, the base attenuate, the margins entire, the apex acute; petioles 0.2–0.5 (–1.5) cm long, densely pubescent. Inflorescences axillary, 2–5 flowers per axil, rarely flowers solitary; flowering pedicels 1–17 mm long, angled, erect, geniculate at anthesis, green, densely pubescent, with long spreading eglandular trichomes and small sparse glandular trichomes (stalk unicellular; head dark multicellular); pedicels scars conspicuous. Buds globose, inflated, yellowish-green. Flowers 5-merous. Calyx ca. 2 mm long, 2 mm wide, cup-shaped, pentagonal in outline, thick, green, strongly 5-nerved, densely pubescent with the same trichomes as the pedicels, calyx appendages five, up to 0.2 mm long, erect, green. Corolla 6–9 mm long, 12–14 mm in diameter, white with wide greenish-yellow pigmentation outside, white with predominance of greenish-yellow spots, sparse and diffuse purple or brown spots and a cream centre within, stellate with interpetalar membrane, lobed halfway or less of the way to the base, pubescent adaxially with a continuous ring of glandular trichomes (stalk long, 2–3-celled; head globose, peltate, unicellular) in the throat and base of the lobes, the tube 3–5 mm long, glabrous abaxially, the lobes 3–4 mm long, 3–4.3 mm wide, widely triangular, spreading, with eglandular trichomes especially on the veins abaxially, the margins papillate, the tips cucullate, papillate. Stamens five, equal; filaments 2.5–3.2 mm long, white, inserted on the corolla ca. 1.2 mm from the base, with auricles fused to the corolla at the point of insertion; anthers 1–1.5 mm long, ellipsoid, cream when young, purplish post-dehiscent, not connivent at anthesis. Gynoecium with ovary ca. 1.2 mm long, 1 mm in diameter, light green, subglobose; ovules more than two per locule; nectary ca. 0.4 mm tall, pale yellow; styles homomorphic, 4–4.5 mm long, exserted 0.5–0.7 mm beyond the anthers, white, clavate; stigma 0.1 mm long, 0.5 mm wide, discoid, pale green. Berry 7–8 mm in diameter, globose, green when immature and pungent, mature fruit colour not seen; fruiting pedicels ca. 20 mm long, pendent and curved, angled, widened distally, green; fruiting calyx 4–4.5 mm in diameter, persistent, not accrescent, discoid, green. Seeds (4–) 8–11 per fruit, 3.25–3.75 mm long, 2.5–2.75 mm wide, C-shaped, brownish-black to black, the seed coat reticulate and tuberculate at margins (SM), reticulate with pillar-like outgrowths at margins (SEM), the cells rectangular to polygonal in shape, the lateral walls straight; embryo imbricate.

#### Distribution.

*Capsicummuticum* is confined to central-eastern Rio de Janeiro State (Brazil) (Fig. 92).

#### Ecology.

*Capsicummuticum* is a component of the montane forests in the Atlantic Forest (Mata Atlântica); it was found growing in the Serra dos Órgãos mountain range (Serra d’Estrella, Serra dos Órgãos, Alto da Serra), mainly in the Dense Ombrophilous Forest (Floresta Ombrófila Densa) in full sun or semi-shade, between 1,000 and 1,300 m elevation.

#### Phenology.

Flowering from December to April. Fruiting from February to May.

#### Chromosome number.

Not known.

#### Common names.

None recorded.

#### Uses.

None recorded.

#### Preliminary conservation assessment.

EOO (15.520 km^2^); AOO (16 km^2^). *Capsicummuticum* is one of the rarest species. The few collections are mostly historical, date from the early 1800s to 1900s and have imprecise locations for the Serra dos Órgãos (Rio de Janeiro). The species was rediscovered in 1986 in Alto da Serra (Petropolis) and gathered several times at the same location. In this site, *Capsicummuticum* is severely threatened since a progressive decline of the habitat has been observed due to anthropogenic disturbance and serious deforestation of the area. Based on the extent of occurrence, the advanced fragmentation, loss of habitat and the few known records for *C.muticum*, we assign this species a threat status of Critically Endangered (CR; B1ab(iii,iv)).

#### Discussion.

*Capsicummuticum* is a member of the Atlantic Forest clade (Carrizo García et al. 2016, as C.villosumvar.muticum). When Sendtner (1846) described *C.villosum*, he also proposed two varietal names: β *latifolium*, a synonym of *C.villosum* (Hunziker, 1971; this treatment) and γ *muticum*. Sendtner recognised var. muticum by its 5-angled, truncate calyx. Hunziker (1971) accepted this varietal name and distinguished var. villosum by its long calyx appendages vs. var. muticum with very short or no calyx appendages. Hunziker also stated that the varieties might not be distinct, based on the specimen *Löfgren 5878* (US, SP), which he identified as var. muticum, even though it has well-developed appendages; this specimen is considered *C.villosum* in this treatment. Modern field explorations (Bianchetti and Hunziker in 1986; Barboza and Carrizo García in 2013; Barboza et al. in 2018) have provided more morphological data for *C.muticum* and, based on recent phylogenetic results that show that it is more closely related to C.mirabilethan tovar.villosum (Carrizo García et al. 2016), we recognise this varietal name at species level.

**Figure 93.**
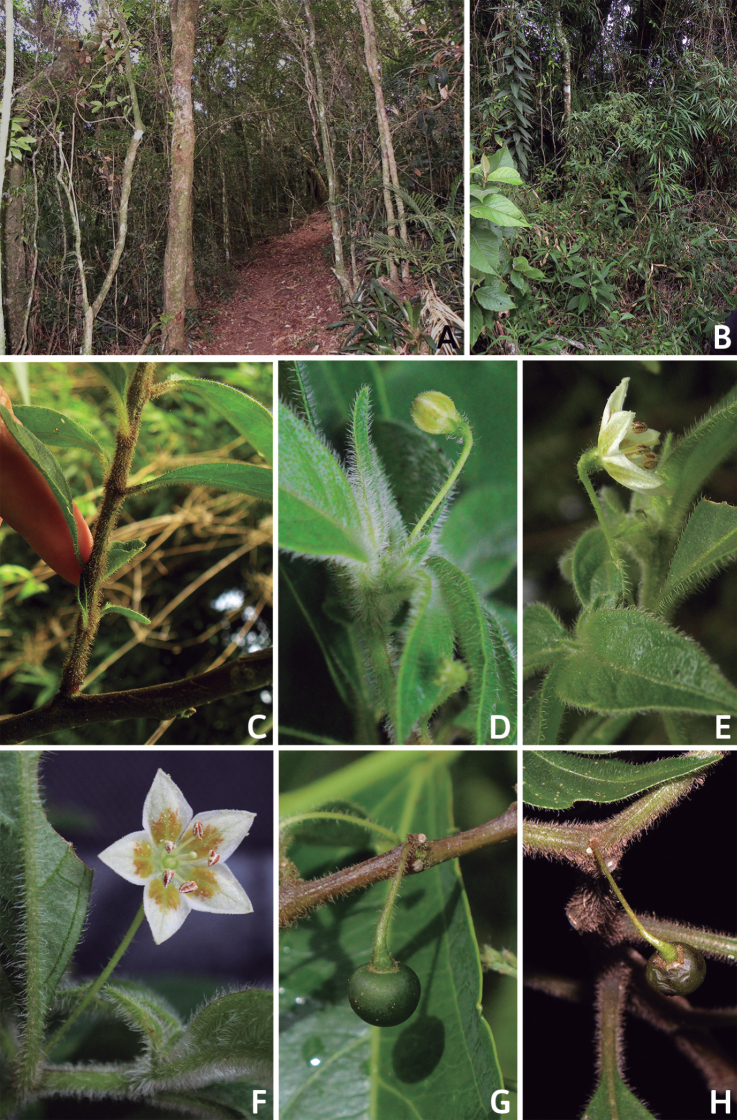
*Capsicummuticum***A** habitat (degraded Atlantic Forest) **B** plant **C** stem and leaves showing the dense indument **D** flower bud on geniculate pedicel **E** flower, in lateral view **F** flower, in front view **G** immature fruit **H** fruit near maturity. From *Barboza et al*. *5032*. Photos by G.E. Barboza.

*Capsicumvillosum* and *C.muticum* are densely pubescent shrubs with long, spreading, flexuous, eglandular trichomes, geniculate flowering pedicels and stellate corollas (Fig. 93D–F); it is also likely that *C.muticum* has the same greenish-golden yellow mature fruits and brownish-black seeds (mature fruits were not found in field explorations) as *C.villosum. Capsicummuticum* is distinguished by having a truncate calyx without appendages or with five minute appendages (not more than 0.2 mm long) delimiting a 5-angular calyx (Fig. 93E, G, H) and white corolla with a predominantly greenish-yellow centre (Fig. 93F); in contrast, *C.villosum* has a calyx with five well-developed linear to subulate appendages (0.3–2.5 mm long) and a white corolla with predominantly purple pigmentation on the lobes and throat (Fig. 121B–E).

#### Specimens examined.

See Suppl. material 4: Appendix 4.

### ﻿32. Capsicum neei

Taxon classificationPlantaeSolanalesSolanaceae

Barboza & X.Reyes, PLOS One 14(1): 19. 2019.

DF0D92EC-D596-5FFD-B253-43FE1FE75F7C

Figs 94 , 95

#### Type.

Bolivia. Chuquisaca: Prov. Hernando Siles, a 4.1 km del puente nuevo de Monteagudo viniendo desde Monteagudo, sobre mano derecha, -19.804617°S, -64.019923°W, 16 Dec 2017, *G.E. Barboza 4927* (holotype: LPB [LPB0003513]; isotypes: CORD [CORD00006935, CORD00006956], NY [04206098]).

#### Description.

Low erect shrubs 0.70–2 (–3) m tall, with the main stem 1–1.5 cm in diameter at base, laxly branched above, the branches thin. Young stems slightly angled, fragile, green, glabrescent; bark of older stems light brown, glabrous; lenticels few. Sympodial units difoliate, the leaves geminate; leaf pair unequal in size, similar in shape. Leaves membranous, concolorous, glabrous or glabrescent on both surfaces and margins, with antrorse, simple, 4–7-celled, eglandular trichomes 0.2–0.5 mm long; blades of major leaves (5.5–) 6.7–11 cm long, 2.1–4 (–4.5) cm wide, elliptic or ovate, major veins 3–5 on each side of mid-vein, the base attenuate, the margins entire, the apex acute; petioles 0.3–0.8 (–1.5) cm long, moderately pubescent; blades of minor leaves 2.7–4.6 (–6) cm long, 1.2–1.8 (–2.3) cm wide, elliptic or ovate, the major veins 3–4 on each side of mid-vein, the base short-attenuate, the margins entire, the apex obtuse or acute; petioles 0.2–0.5 (–0.8) cm long, with similar pubescence as in major leaves. Inflorescences axillary, 2–4-flowers per axil, rarely flowers solitary; flowering pedicels (6.5–) 8–15 mm long, filiform, angled, pendent, slightly curved, non-geniculate at anthesis, green, with sparse antrorse eglandular trichomes and small dark glandular trichomes (stalk unicellular; head multicellular); pedicels scars inconspicuous. Buds ovoid, greenish-yellow. Flowers 5-merous. Calyx 1.7–2.5 mm long, 2–3 mm wide, cup-shaped, with 10 veins clearly evident, green, moderately pubescent with eglandular trichomes, the calyx appendages 10, unequal, the five main appendages longer (0.7–) 0.9–1.75 (–2) mm long, emerging very close from the margin, the five secondary appendages shorter 0.2–0.8 (–1.2) mm long, emerging 0.8–1 mm below the margin, linear, green, with the same non-glandular trichomes of the calyx tube. Corolla (6–) 8–10 mm long, 6–8 (–12) mm in diameter, entirely yellow or with small greenish spots in the base of the lobes and tube outside and within, stellate with a thin interpetalar membrane, lobed nearly halfway to the base, the tube 3–4.5 mm long, pubescent adaxially with small glandular trichomes (head and stalk one-celled each), glabrescent abaxially, the lobes 3.5–5.5 mm long, ca. 2 mm wide, ovate or triangular, spreading, glabrous adaxially and with sparse eglandular trichomes abaxially, the margins finely ciliate, the tips cucullate and papillate. Stamens five, slightly subequal; filaments 1.4–1.75 mm long, cream, inserted on the corolla ca. 1.2 mm from the base, with auricles fused to the corolla at the point of insertion; anthers (1.5–) 1.8–2 mm long, ellipsoid, light yellow, not connivent at anthesis. Gynoecium with ovary ca. 1.2 mm long, 1.3 mm in diameter, light green, ovoid or subglobose; ovules more than two per locule; nectary ca. 0.3 mm tall; styles homomorphic, 3.75 mm long, 0.8–1 mm beyond the anthers, cream, clavate; stigma ca. 0.2 mm long, 0.3 mm wide, somewhat bilobed, light green. Berry 4–9 mm in diameter, globose, slightly apiculate, green when immature turning to orange-coloured or red at maturity, pungent, the pericarp thick, opaque, with giant cells (endocarp alveolate); stone cells absent; fruiting pedicels (13–) 18–34 mm long, pendent, angled or terete, widened distally, green; the fruiting calyx ca. 4 mm in diameter, persistent, not accrescent, discoid, green, with an annular constriction at junction with the pedicel, the appendages 1–2 mm long, spreading or reflexed. Seeds 5–10 per fruit, 4–5 mm long, 3–4.25 mm wide, subglobose, pale yellow to white, the seed coat smooth and reticulate at margins (SM), reticulate-cerebelloid (SEM), the cells polygonal in shape, the lateral walls straight to wavy or sinuate; embryo not seen.

#### Distribution.

*Capsicumneei* is endemic to central and southern Bolivia (Chuquisaca and Santa Cruz Departments) (Fig. 92).

#### Ecology.

*Capsicumneei* is most commonly collected in the Boliviano-Tucumano Forest from the understorey of deciduous cloud forests, between 1,100 and 1,750 m elevation.

#### Phenology.

Flowering and fruiting from October to May.

#### Chromosome number.

Not known.

#### Preliminary conservation assessment.

EOO (13,880.224 km^2^); AOO (48 km^2^). *Capsicumneei* has been collected in more than 10 localities and many times in the last 23 years, in a recently Protected Area (National Park and Integrated Management Natural Area “Serranía Inao”) and in nearby areas, which suggests that both the geographic range (EOO and AOO) and the population size are likely not to be significantly affected in the forthcoming years. Due to this, *C.neei* can be assessed as Least Concern (LC).

#### Discussion.

In phylogenetic analyses, *Capsicumneei* was recovered within the Bolivian clade, resolved as sister to *C.caballeroi* (Barboza et al. 2019). The demonstrated pungency of the fruits and the presence of giant cells in the pericarp are characters also found in the remaining species of the Bolivian clade, but, to clarify the other species not yet included in a phylogeny (i.e. *C.ceratocalyx*), the affinities of *C.neei* deserve to be studied in more detail.

*Capsicumneei* is morphologically most similar to the Bolivian *C.minutiflorum* in having stellate, yellow corollas and red fruits at maturity (Fig. 95I, J). It is distinguished by its non-geniculate, pendent flowering pedicels and strongly-nerved calyx with 10 unequal appendages (Fig. 95C, E, F). In contrast, *C.minutiflorum* has geniculate and erect flowering pedicels and a weakly-nerved calyx with five equal, short appendages (Fig. 87D, F).

**Figure 94.**
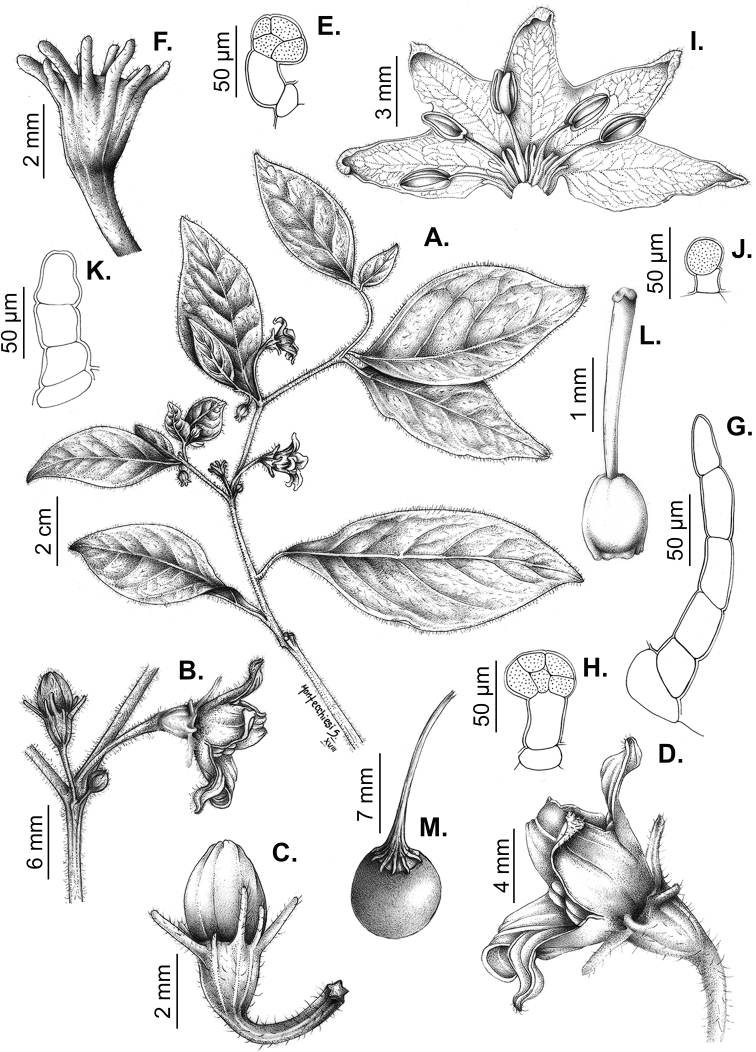
*Capsicumneei***A** flowering branch **B** inflorescence **C** flower bud **D** flower **E** glandular trichome of the pedicels **F** calyx **G** eglandular trichome of the outer surface of the calyx **H** glandular trichome of the inner surface of the calyx **I** opened corolla **J** glandular trichome of the inner surface of the corolla **K** eglandular trichome of the inner surface of the corolla lobes **L** gynoecium **M** fruit. From *Barboza 4927.* Drawn by S. Montecchiesi. Published in Barboza et al. (2019), reproduced with permission.

**Figure 95.**
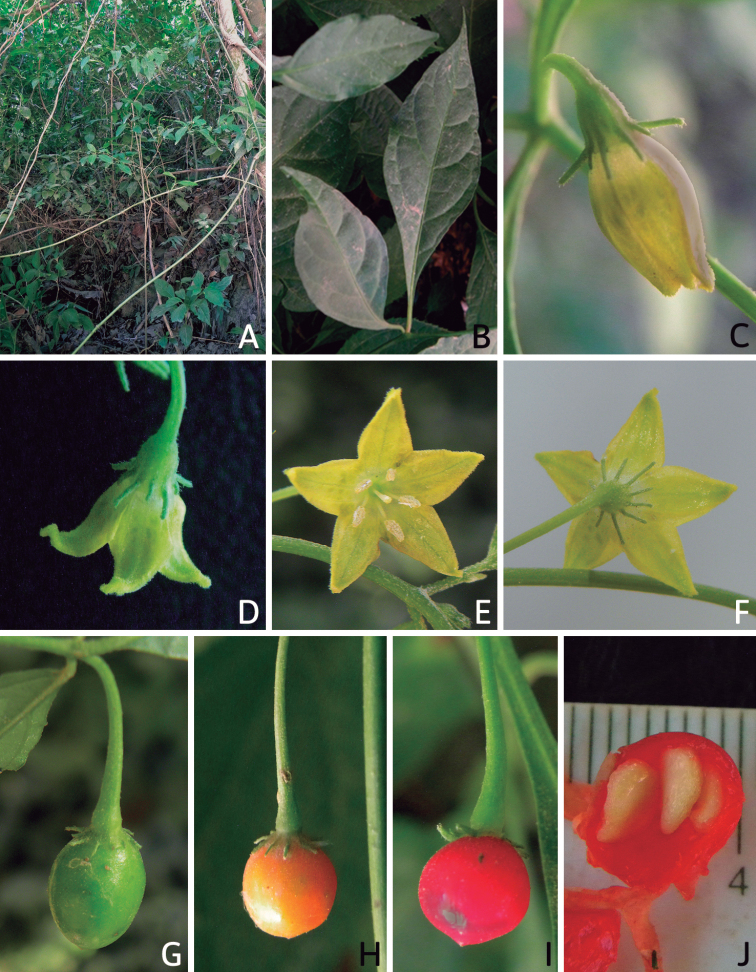
*Capsicumneei***A** plant **B** leaves, abaxial surface **C** flower bud **D** flower, in lateral view **E** flower, in front view **F** flower, seen from behind **G** immature fruit **H, I** mature fruits **J** fruit, longitudinal section, showing the seeds **A–G** from *Barboza et al. 4927***H–J** from *Barboza & Reyes 5040*. Photos by G.E. Barboza. Modified from Barboza et al. (2019), reproduced with permission.

*Capsicumneei* is sympatric with C.baccatumvar.baccatum, which has geniculate pedicels, a calyx with five subequal appendages, white corollas with greenish-yellow spots within and globose to ellipsoid upright red fruits.

#### Specimens examined.

See Suppl. material 4: Appendix 4.

### ﻿33. Capsicum parvifolium

Taxon classificationPlantaeSolanalesSolanaceae

Sendtn., Fl. Bras. [Martius] 10(6): 145. 1846.

369A8687-DCF7-5773-826E-F7E097A29F02

Figs 96 , 97

Fregirardia leptoclada Dunal, Prodr. [A. P. de Candolle] 13(1): 505. 1852. Type. Brazil. Bahia: “le bas des montagnes. Certam du Rio Fco [Rio San Francisco]”, 1838, *J.S. Blanchet 2823* (lectotype, designate here, G-DC [G00200561]; isolectotypes: BM [BM001016675, BM000617948], CORD [CORD00087945, CORD00087948], F [v0043297F, acc. # 646814], G [G00390274], K [K000585899, K000585900], LE, MPU [MPU026995 fragment ex G-DC, MPU023035 fragment ex G, MPU023036 fragment ex G], P [P00410185], W [acc. # 0001348, acc. # 1889-0118245]).
Capsicum leptocladum (Dunal) Kuntze, Revis. Gen. Pl. 2: 450.1891. Type. Based on Fregirardialeptoclada Dunal.
Bassovia ciliata J.R.Johnst., Proc. Amer. Acad. Arts 40: 694. 1905. Type. Venezuela. Nueva Esparta: Isla Margarita El Valle, 30 Aug 1903, *J.R. Johnston 75* (lectotype, designated by Barboza et al. 2011, pg. 773: GH [00936715]; isolectotype: US [00027416, acc. # 531916)].

#### Type.

[Brazil]. Bahia: Serra Açuruá, Rio San Francisco, 1838, *J.S. Blanchet 2823* (lectotype, designated by Barboza et al. 2011, pg. 773: W [acc. # 1889-0118245]; isolectotypes: BM [BM001016675, BM000617948], CORD [CORD00087945, CORD00087948], F [v0043297F, acc. # 646814], G-DC [G00200561], G [G00390274], K [K000585899, K000585900], LE, MPU [MPU026995 fragment from G-DC, MPU023035 fragment from G, MPU023036 fragment from G], P [P00410185], W [acc. # 0001348]).

#### Description.

Erect shrubs 1–4 (–5) m tall, with an almost fastigiate habit, much branched above. Young stems angled, slender, fragile, greyish-brown, moderately pubescent with antrorse, simple, uniseriate, 4–6-celled, eglandular trichomes 0.3–1.1 mm long; nodes light brown; bark of old stems grey or brown, glabrous; lenticels absent. Sympodial units difoliate, the leaves geminate; leaf pair unequal in size, similar in shape. Leaves membranous, slightly discolorous, dark green above, paler beneath, sparsely to moderately pubescent on both surfaces, the trichomes similar to those of the stems, rarely few dendritic trichomes on the veins; blades of major leaves 3–8 (–9) cm long, 1–4.2 cm wide, ovate, the major veins 5–7 on each side of mid-vein, the base short-attenuate and unequal, the margins entire, the apex acuminate; petioles 0.5–2.4 cm, moderately to densely pubescent; blades of minor leaves 1.5–2.5 cm long, 0.8–1.5 cm wide, ovate, the major veins 3–4 on each side of mid-vein, the base short-attenuate, the margins entire, the apex acute; petioles 0.3–0.5 cm, moderately to densely pubescent. Inflorescences axillary, 3–6 (–8) flowers per axil; flowering pedicels 9–18 (–20) mm long, angled, pendent, non-geniculate at anthesis, green, densely pubescent, with short antrorse eglandular trichomes; pedicels scars conspicuous, corky. Buds subglobose, pale violet at the apex and cream near the base. Flowers 5-merous. Calyx (1.2–) 1.5–2.4 mm long, 2–3 mm wide, cup-shaped, thick, strongly 5-nerved, green, densely pubescent like the pedicels, the calyx appendages five or, exceptionally, up to seven, 0.7–2 (–2.2) mm long, subequal, erect or spreading, green. Corolla 4.5–7.3 (–8) mm long, 1–2 cm in diameter, purple with a narrow white margin and a yellowish-green centre outside and within, stellate with interpetalar membrane, lobed nearly halfway to the base, pubescent adaxially with small glandular trichomes (stalk 2-celled; head unicellular) in the throat and base of the lobes, glabrous abaxially, the tube (2.4–) 3–4 (–4.5) mm long, the lobes (2.1–) 2.9–3.5 mm long, 2–3.5 mm wide, broadly triangular, spreading, the margins involute, papillate or with long simple trichomes, the tips cucullate, densely papillate. Stamens five, equal; filaments 1.5–2.5 mm long, greenish-white, inserted on the corolla 1.3–1.5 mm from the base, with auricles fused to the corolla at the point of insertion; anthers 1.7–2.3 mm long, ellipsoid, yellow or violet, not connivent at anthesis. Gynoecium with ovary 1–1.4 mm long, 1 mm in diameter, greenish-white, ovoid; ovules more than two per locule; nectary 0.3–0.4 mm tall; styles homomorphic, 3.5–5.1 mm long, exserted ca. 1.5 mm beyond the anthers, whitish-green, clavate; stigma ca. 0.3 mm long, ca. 1–1.1 mm wide, somewhat discoid, green. Berry 8.5–9.5 mm in diameter, globose, slightly flattened at the apex, dark green when immature, greenish-golden yellow at maturity, deciduous, pungent, the pericarp thin, translucent, with giant cells (endocarp alveolate); stone cells absent; fruiting pedicels 10–18 mm long, angled, pendent, curved, widened distally, green; fruiting calyx 4–6 mm in diameter, persistent, not accrescent, discoid, strongly 5(–10)-nerved, green, the appendages spreading or reflexed. Seeds (2–) 4–13 per fruit, 3–3.8 mm long, 2.7–3 mm wide, C-shaped, brownish-black, the seed coat reticulate and slightly tuberculate at margins (SM), reticulate-cerebelloid (SEM), the cells irregular in shape, the lateral walls sinuate in the seed body, straight and wavy at margins; embryo imbricate.

#### Distribution.

*Capsicumparvifolium* has a disjunct distribution in South America (Fig. 92). It is found in the Brazilian Caatinga (Bahia, Ceará, Minas Gerais, Paraíba, Pernambuco, Piauí and Rio Grande do Norte States), as well as along the coast of Venezuela (Distrito Federal, Aragua, Carabobo, Lara, Sucre, Nueva Esparta States) and Colombia (Atlántico and Magdalena Departments).

#### Ecology.

*Capsicumparvifolium* is a common element in seasonally dry tropical forest, mainly in the outcrops (‘inselbergs’) of the arborescent-shrubby Caatinga, at 500–1,200 m. In Venezuela and Colombia, it is found in coastal areas with higher humidity at lower elevations (0–350 m).

#### Phenology.

Flowering from August to April and fruiting from February to June in the Caatinga; flowering from April to October and fruiting from May to October in Venezuela and Colombia.

#### Chromosome number.

2*n* = 2x = 24 (Barboza et al. 2011).

#### Common names.

**Brazil**: Alecrim-quebrado (Piauí, *Mendes et al. 509*), Jiriquiti (Piauí, *Mendes et al. 539*), Murta (Sergipe, *Oliveira et al. 552*), Pimentinha (Pernambuco, *Lucena 91*).

#### Uses.

None recorded.

#### Preliminary conservation assessment.

EOO (4,493,957.435 km^2^); AOO (228 km^2^). *Capsicumparvifolium* is under threat in its fragmented habitat. Although in Brazil it is common, its habitat is restricted to the Caatinga, the least protected of all the major ecoregions in the country (Fonseca et al. 2017; Silva et al. 2019). The species is more seriously threatened along the coast of Colombia, since only four records, which all date from the early 1900s, exist from areas that are currently highly urbanised (the cities of Santa Marta and Barranquillas). Few collections are known (13) in Venezuela and none of them is from protected areas. Considering the severely-fragmented habitat (Antongiovanni et al. 2018) and the projected decline of the area of occupancy by the impact of climate change (Silva et al. 2019), we assign a threat status of Vulnerable (VU; B2ab(iii) for *C.parvifolium*.

#### Discussion.

*Capsicumparvifolium* belongs to the Caatinga clade (Carrizo García et al. 2016). Together with *C.longidentatum* and *C.caatingae*, these are the only *Capsicum* species growing in the arid Brazilian Caatinga (although *C.parvifolium* also extends into northernmost Venezuela and Colombia). The close affinity between *C.parvifolium* and *C.caatingae* was discussed and supported by morphological and karyological data by Barboza et al. (2011). *Capsicumparvifolium* resembles *C.caatingae*; nevertheless, it is clearly distinguishable by the five calyx appendages (vs. absence of appendages in *C.caatingae*), the few-flowered (vs. multi-flowered) inflorescences, the greenish-golden yellow (vs. red) fruits and the brownish-black (vs. yellow) seeds. Specimens of *C.parvifolium* from Venezuela have been confused in herbaria with *C.rhomboideum*, due to their similarities in calyx structure. However, they can be easily differentiated by corolla shape and colour (campanulate and yellow in *C.rhomboideum*) and fruit colour at maturity (red in *C.rhomboideum*).

Dunal (1852) described *Fregirardialeptoclada*, based on three *Blanchet 2823* collections kept at G (G-DC and general collection). We found two specimens at G and duplicates of the three G collections at MPU. We select the more complete specimen housed at G-DC [G00200561] as the lectotype. Duplicates are widely distributed in other herbaria.

**Figure 96.**
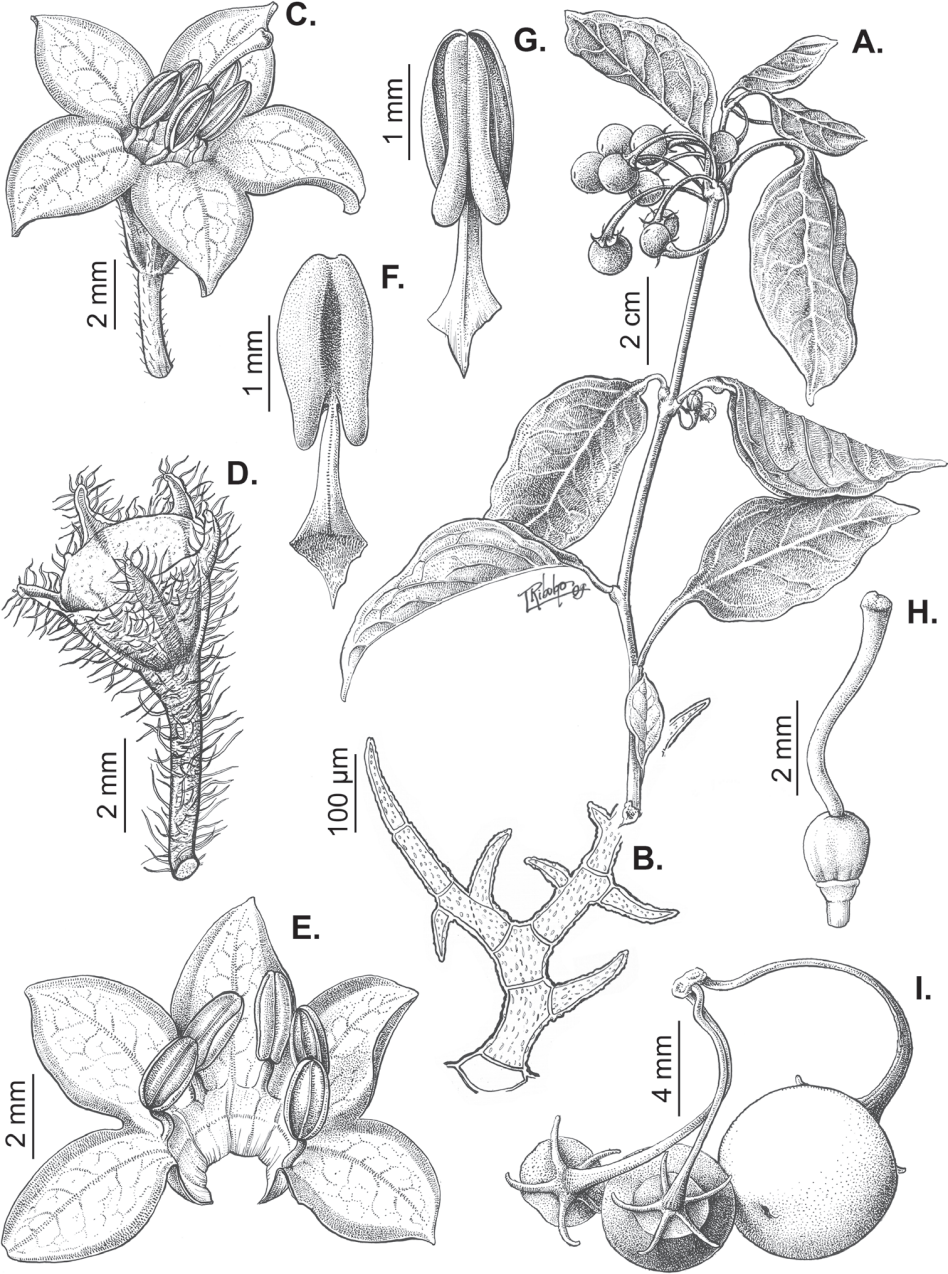
*Capsicumparvifolium***A** fruiting branch **B** branched trichome of the leaf **C** flower **D** calyx **E** opened corolla **F, G** anthers, dorsal and ventral views, respectively **H** gynoecium **I** fruits **A, C, E–I** from *Agra & Barboza 7075***B, D** from *Pickersgill 72–366*. Drawn by L. Ribulgo. Published in Barboza et al. (2011), reproduced with permission.

**Figure 97.**
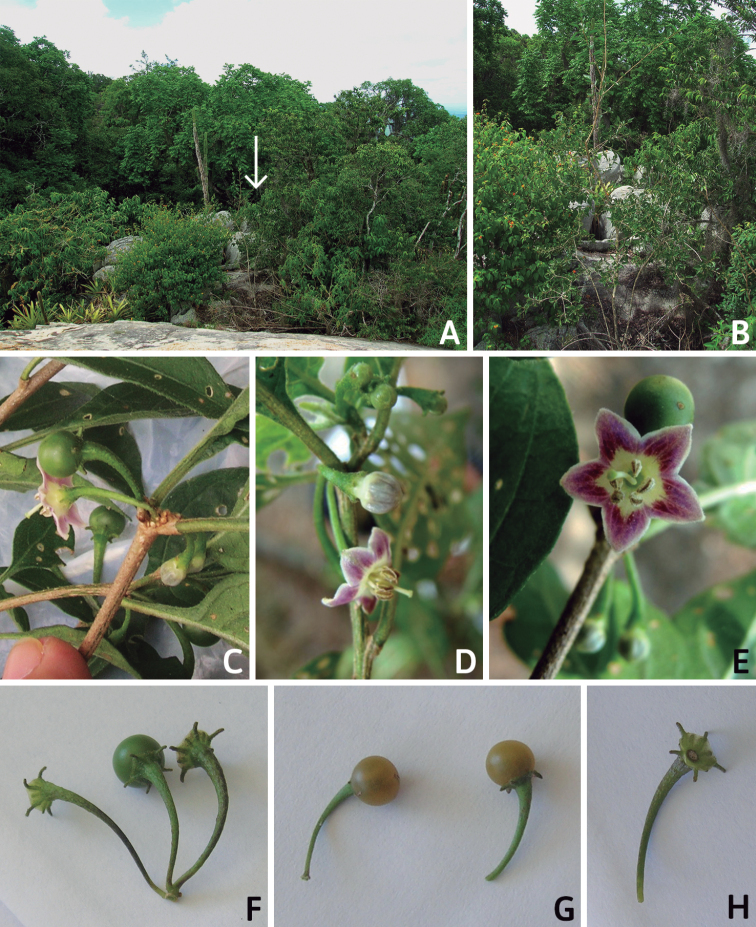
*Capsicumparvifolium***A** habitat (Caatinga) **B** plant **C** reproductive branch **D** flower bud and flower on pendent pedicels **E** flower, in front view **F** fruiting calyces and immature fruit **G** mature fruits **H** fruiting calyx, inside view. From *Agra & Barboza 7075.* Photos by G.E. Barboza. Modified from Barboza et al. (2011), reproduced with permission.

#### Specimens examined.

See Suppl. material 4: Appendix 4.

### ﻿34. Capsicum pereirae

Taxon classificationPlantaeSolanalesSolanaceae

Barboza & Bianch., Syst. Bot. 30(4): 863. 2005.

DC7EDF6E-600D-5981-8795-6875102AFEB1

Figs 98 , 99

#### Type.

Brazil. Espírito Santo: Mun. Castelo, Castelo-Forno Grande, 6 Dec 1956, *E. Pereira 2245* (holotype: CORD (CORD00006630); isotypes: CORD [CORD00006631, CORD00006632], HB [acc. # 7060], RB [RB00722054, acc. # 96194]).

#### Description.

Erect shrubs (0.5–) 0.8–3 (–8?) m tall, the branches slender and horizontal in a typical “zig-zag” appearance. Young stems slightly angled, fragile, green, white mottled, glabrous; nodes solid, purple; bark of older stems brown, glabrous; lenticels absent. Sympodial units difoliate, the leaves geminate; leaf pair unequal in size, similar in shape. Leaves coriaceous, discolorous, bright dark green above, opaque light green beneath, glabrous on both surfaces, with the abaxial nerves white mottled; blades of major leaves (6.1–) 9–15 (–20) cm long, 2–4 (–7) cm wide, elliptic to narrowly elliptic, the major veins (7–) 8–12 on each side of mid-vein, the base asymmetric, short-attenuate or attenuate, the margin entire, the apex acute to acuminate; petioles 0.5–1 (–1.5) cm, glabrous; blades of minor leaves 2.5–3 (–5.4) cm long, 0.75–1.5 cm wide, elliptic to narrowly elliptic, the major veins 4–7 on each side of mid-vein, the base short-attenuate, the margin entire, the apex acute; petioles 0.3–0.5 cm, glabrous. Inflorescences axillary, 2–6 flowers per axil, rarely flowers solitary; flowering pedicels 15–30 mm long, slightly striate, pendent, sometimes curved, non-geniculate at anthesis, green or green-purple, glabrous; pedicels scars inconspicuous. Buds subglobose, white with green or greenish-yellow spots at the base. Flowers 5-merous. Calyx 1.5–2 (–3) mm, (2.5–) 3–3.2 mm wide, cup-shaped, circular or pentagonal in outline, thin and translucent, green or light green, glabrescent, with short eglandular trichomes on the margin, the calyx appendages absent or five, minute, less than 0.5 mm. Corolla 9–10 mm long, 8–19 mm in diameter, entirely white or white with greenish-yellow spots outside, white with purple and greenish-yellow pigmentation in the lobes and throat and a cream centre within, stellate with interpetalar membrane, lobed nearly halfway to the base, the tube ca. 4 mm long, with a continuous ring of small glandular trichomes (stalk long, 1–2-celled; head globose, peltate unicellular) adaxially, glabrous abaxially, the lobes 5–6 mm long, 4.5–5 (–6) mm wide, broadly triangular, spreading, glabrous adaxially and abaxially, the margins involute and papillate, the tips strongly cucullate, densely papillate. Stamens five, equal; filaments 3–4 (–5) mm long, equal, cream, inserted on the corolla ca. 1 mm from the base, with auricles fused to the corolla at the point of insertion; anthers 1.8–2.1 (–3) mm, ellipsoid, dark green or greyish, not connivent at anthesis. Gynoecium with ovary ca. 1.5 mm long, ca. 1.2 mm in diameter, light green, subconic; ovules more than two per locule; nectary ca. 0.3 mm tall; styles homomorphic, (3.7–) 4–6 (–7) mm long, barely exserted beyond the anthers, cream, clavate, slightly curved; stigma 0.2–0.3 mm long, ca. 0.7 mm wide, discoid, somewhat bilobulate, light green. Berry (6–) 8–10 mm in diameter, globose, hardly depressed, green when immature, greenish-golden yellow at maturity, deciduous, pungent, the pericarp thin, translucent, with giant cells (endocarp alveolate); stone cells absent; fruiting pedicels (20–) 23–28 (–35) mm, slightly angled, pendent, slightly widened distally, green; fruiting calyx 5–6 mm in diameter, persistent, not accrescent, discoid, green. Seeds (3–) 5–20 per fruit, 3–3.7 mm long, 2.5–3.4 mm wide, subglobose or teardrop-shaped, brownish-black to black, the seed coat reticulate and tuberculate at margins (SM), reticulate with pillar-like outgrowths at margins (SEM), the cells irregular in shape, the lateral walls sinuate, straight to the hilum; embryo imbricate.

**Figure 98.**
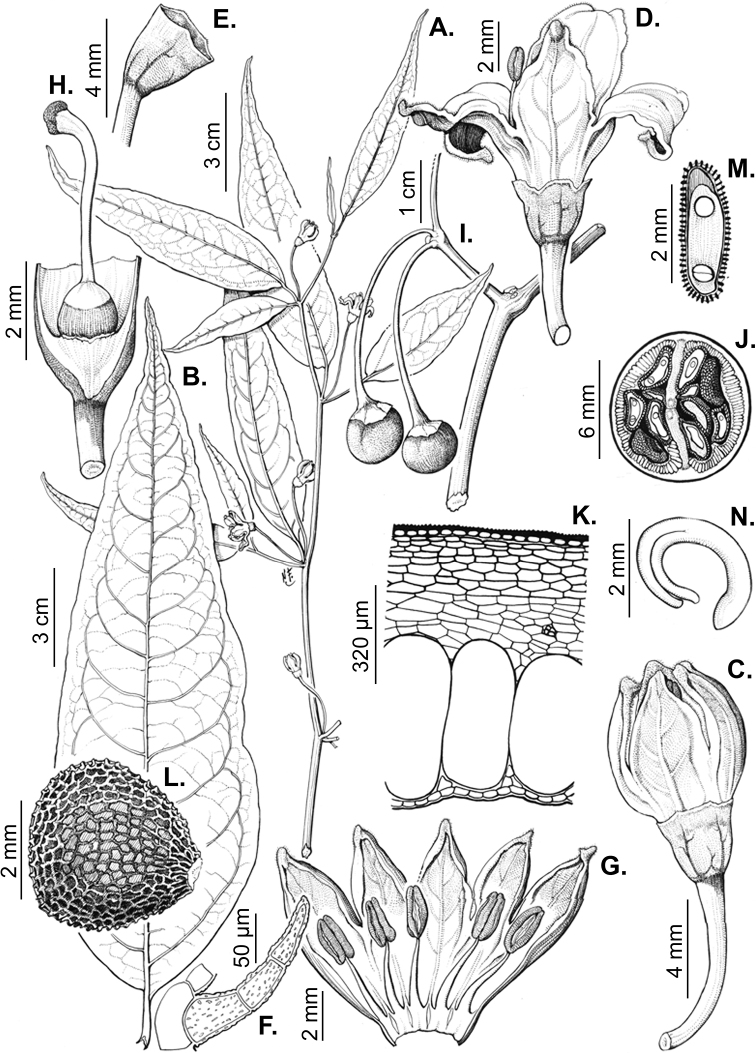
*Capsicumpereirae***A** flowering branch **B** leaf **C** flower bud **D** flower **E** calyx **F** eglandular trichome of the calyx **G** opened corolla **H** gynoecium **I** fruiting branch **J** fruit, in cross section **K** anatomical detail of the pericarp (note the giant cells in the mesocarp) **L** seed **M** seed, in cross section **N** embryo **A–J, L–N** from *Pereira 2245***K** from *Hunziker 25248*. Drawn by N. de Flury. Published in Barboza and Bianchetti (2005), reproduced with permission.

**Figure 99.**
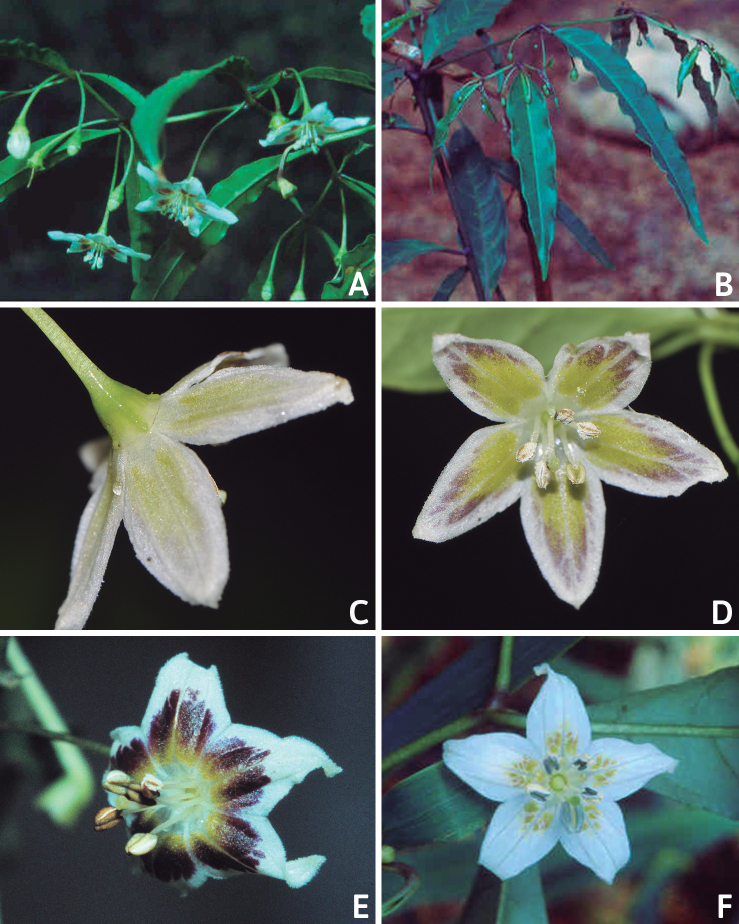
*Capsicumpereirae***A** flowering branch **B** young branch showing lanceolate leaves and buds on pendent pedicels **C** flower showing mutic calyx **D, E** flowers, in front view, with different patterns of purple colouration **F** flower without purple spots **A, B, E** from *Bianchetti et al. 1558***F** from *Bianchetti et al. 1567*, photos by L. Bianchetti **C, D** no specimen vouchers, photos taken *in situ* by C. dal Zovo (Associazione PepperFriends). Modified from Barboza and Bianchetti (2005), reproduced with permission.

#### Distribution.

**Figure 100.**
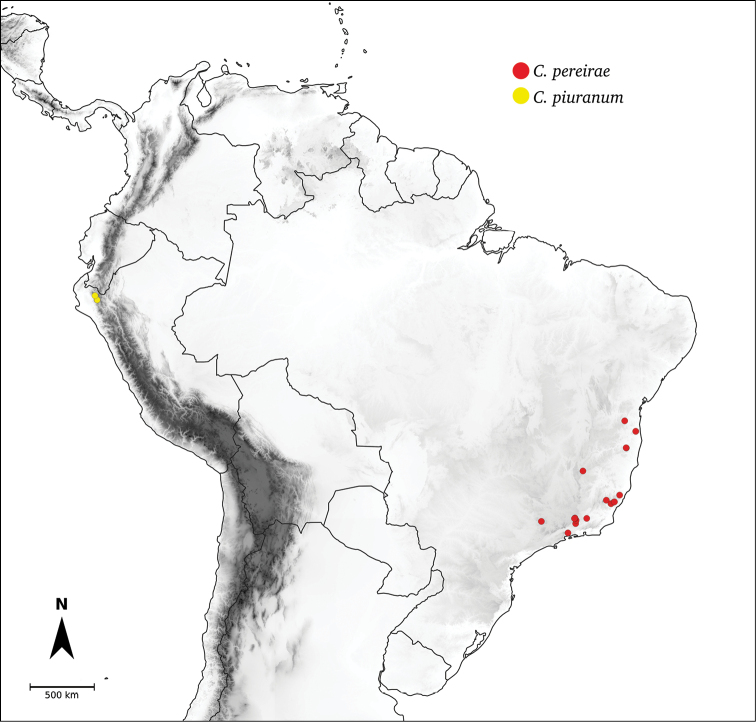
Distribution of *C.pereirae* and *C.piuranum*.

*Capsicumpereirae* is endemic to north- and south-eastern Brazil (Bahía, Espírito Santo, Minas Gerais and São Paulo States) (Fig. 100).

#### Ecology.

*Capsicumpereirae* inhabits the Atlantic Forest (Mata Atlântica), mainly in the Dense Ombrophilous Forest (Floresta Ombrófila Densa), between 500 and 1,650 m elevation. It is found in marginal or interior forests or in aquatic depressions usually in shady places. In the Parque Estadual do Ibitipoca (Minas Gerais), the species is found close to wet caves in the Nanofloresta Nebular (Mata de Grota) formation.

#### Phenology.

Flowering from late October to March. Fruiting from February to May.

#### Chromosome number.

*n* = 13 (Pozzobon and Schifino-Wittmann 2006); 2*n* = 2x = 26 (Pozzobon et al. 2006, *as Capsicum* sp 7; Moscone et al. 2007).

#### Common names.

None recorded.

#### Uses.

None recorded.

#### Preliminary conservation assessment.

EOO (271,639.782 km^2^); AOO (92 km^2^). *Capsicumpereirae* has a relatively large extent of occurrence and is found in more than 10 localities, the majority of them in protected areas (Bahia: RPPN Palmeira, PN de Boa Nova; Espírito Santo: Estação Biológica de Santa Lúcia; Mina Gerais: APA Felício, Parque Estadual do Ibitipoca and RPPN Loredano Aleixo). For these reasons, we assign the preliminary LC category. However, subpopulations outside conservation areas within the Atlantic Forest may be threatened.

#### Discussion.

*Capsicumpereirae* belongs to the Atlantic Forest clade (Carrizo García et al. 2016) and is a member of the group of species with white corollas tinged with purple and greenish-yellow spots within (Fig. 99D, E), also growing in eastern Brazil (e.g. *C.mirabile*, *C.schottianum*, *C.hunzikerianum*). In some specimens from the Parque Estadual do Ibitipoca, anthocyanin pigmentation of the corolla is paler (Fig. 99D) or, more rarely, the purple spots are lacking completely (Fig. 99F), a peculiarity also observed in *C.schottianum* and *C.mirabile.*

*Capsicumpereirae* also shares with *C.schottianum* the absence of evident calyx appendages, but the two species can be distinguished by general pubescence (plants glabrous in *C.pereirae* vs. plants glabrescent to pubescent in *C.schottianum*), consistency of the leaves (coriaceous vs. membranous) and geniculation of the flowering pedicels (non-geniculate vs. geniculate). Stems and leaves of *C.pereirae* are glabrous like *C.hunzikerianum* from which it can be differentiated by the lack of appendages in the calyx, the smaller flowers and the shorter fruiting pedicels. The presence of membranous leaves and calyx with five conspicuous appendages distinguish *C.mirabile* (Figs 88, 89) from *C.pereirae* with its coriaceous leaves and lack of appendages.

#### Specimens examined.

See Suppl. material 4: Appendix 4.

### ﻿35. Capsicum piuranum

Taxon classificationPlantaeSolanalesSolanaceae

Barboza & S.Leiva, PLOS One 14(1): 14. 2019.

93AFBB74-CAD3-5C19-9957-6A648FFD46A3

Figs 101 , 102

#### Type.

Peru. Piura: Prov. Huancabamba, borde de carretera y riachuelo, 5°22'46"S, 79°33'47"W, 2311–2459 m elev., 22 Mar 2011, *T. Mione 812* (holotype: CORD [CORD00006936]; isotype: NY [03231447]).

#### Description.

Erect shrubs 2–2.20 (–3) m tall, densely branched above, the branches scandent. Young stems angled, fragile, flexuous, green, glabrous and shiny; bark of older stems green to dark brown, angled, glabrous; lenticels absent. Sympodial units difoliate, the leaves geminate; leaf pair markedly unequal in size and similar or dissimilar in shape. Leaves membranous, discolorous, dark green and shiny adaxially, light green and opaque abaxially, the mid-vein and primary veins raised abaxially, glabrous or both surfaces with sparse antrorse, simple, 4–5-celled, eglandular trichomes 0.5–1.2 mm long, occasionally trichomes more abundant on main veins and margins; blades of major leaves (8–) 12–17.7 cm long, (2–) 2.5–4.5 cm wide, elliptic, the major veins 7–9 on each side of mid-vein, the base asymmetric and attenuate, the margins entire, the apex long-acuminate; petioles 0.7–1.4 (–1.7) cm long, slightly winged from the decurrent leaf bases, glabrous or glabrescent; blades of minor leaves 2.5–4.5 cm long, 1.5–2.6 cm wide, ovate or elliptic, the major veins 3–4 on each side of mid-vein, the base short-attenuate or rounded and asymmetric, the margins entire, the apex acute or slightly rounded; petioles 0.2–0.5 cm long, glabrescent or moderately pubescent. Inflorescences axillary, 1–3-flowered; flowering pedicels 19–26 mm long, filiform, terete, pendent, slightly curved, non-geniculate at anthesis, usually green, sometimes with purple spots, glabrous or glabrescent, the eglandular trichomes short, antrorse; pedicels scars inconspicuous. Buds ovoid, yellow or pale yellow. Flowers 5-merous. Calyx 1.5–2.6 (–3) mm long, 3–4 mm wide, cup-shaped, thick, purple or greenish-purple, glabrescent to moderately pubescent, the calyx appendages five, (0.9–) 2.5–3 mm long, 0.5–0.8 mm wide, equal, thick, erect, subulate, inserted close to the margin, glabrous or glabrescent with the same trichomes as pedicels and calyx tube. Corolla 14.5–17 mm long, 12–17 mm in diameter, thick, entirely yellow outside and within, tubular-campanulate with interpetalar membrane, lobed less than 1/3 of the way to the base, glabrous adaxially and abaxially, the tube 11–12 mm long, ca. 6 mm in diameter, the lobes 3.5–5 mm long, 4.5–5 mm wide, broadly ovate, erect, the margins finely ciliate, the tips papillate. Stamens five, subequal; filaments 3–5 mm long, greenish-white, inserted on the corolla 3–4 mm from the base, with auricles fused to the corolla at the point of insertion; anthers 2–2.5 (–2.8) mm long, ellipsoid, yellowish-white, slightly connivent at anthesis. Gynoecium with ovary 1.25–1.5 mm long, 1.5 mm in diameter, white, subglobose; ovules more than two per locule; nectary ca. 0.5 mm tall; styles homomorphic, 7.5–8 mm long, barely exserted beyond the anthers, white, clavate; stigma 0.5 mm long, 0.8–1 mm wide, somewhat bilobed, green. Berry 9–12 mm in diameter, globose, slightly flattened at the apex, green or white when immature, orange to red at maturity, non-pungent, the pericarp thick, opaque, lacking giant cells (endocarp smooth); stone cells two, polyhedral, yellowish-white; fruiting pedicels 28–36 mm long, pendent, slightly angled, widened distally, green; fruiting calyx ca. 4 mm in diameter, persistent, not accrescent, discoid, green-purple or green, the appendages 5–6.1 mm long, 0.8–1 mm wide at base, reflexed. Seeds ca. 50–80 per fruit, ca. 2.5 mm long, 2–2.2 mm wide, subglobose or teardrop-shaped, dark brown to black, the seed coat reticulate (SM), reticulate-cerebelloid (SEM), the cells polygonal or irregular in shape, the lateral walls straight or slightly sinuate; embryo imbricate.

#### Distribution.

*Capsicumpiuranum* is endemic to a restricted area in northern Peru (Piura Department) (Fig. 100).

#### Ecology.

*Capsicumpiuranum* grows in misty rain montane forests, in margins or near streams, between 2,300 and 2,860 m elevation.

#### Phenology.

Flowering from November to May, with a peak of fruiting in March–May.

#### Chromosome number.

2*n* = 2x = 26 (Barboza et al. 2019).

#### Common names.

None recorded.

#### Uses.

None recorded.

#### Preliminary conservation assessment.

EOO (10.195 km^2^); AOO (8 km^2^). *Capsicumpiuranum* has a very restricted distribution (EOO and AOO) and a small population size (< 250 individuals). This species is proposed as Critically Endangered (CR; C2a(i)) because of the inferred continuing decline in the number of mature individuals in each subpopulation (< 50).

**Figure 101.**
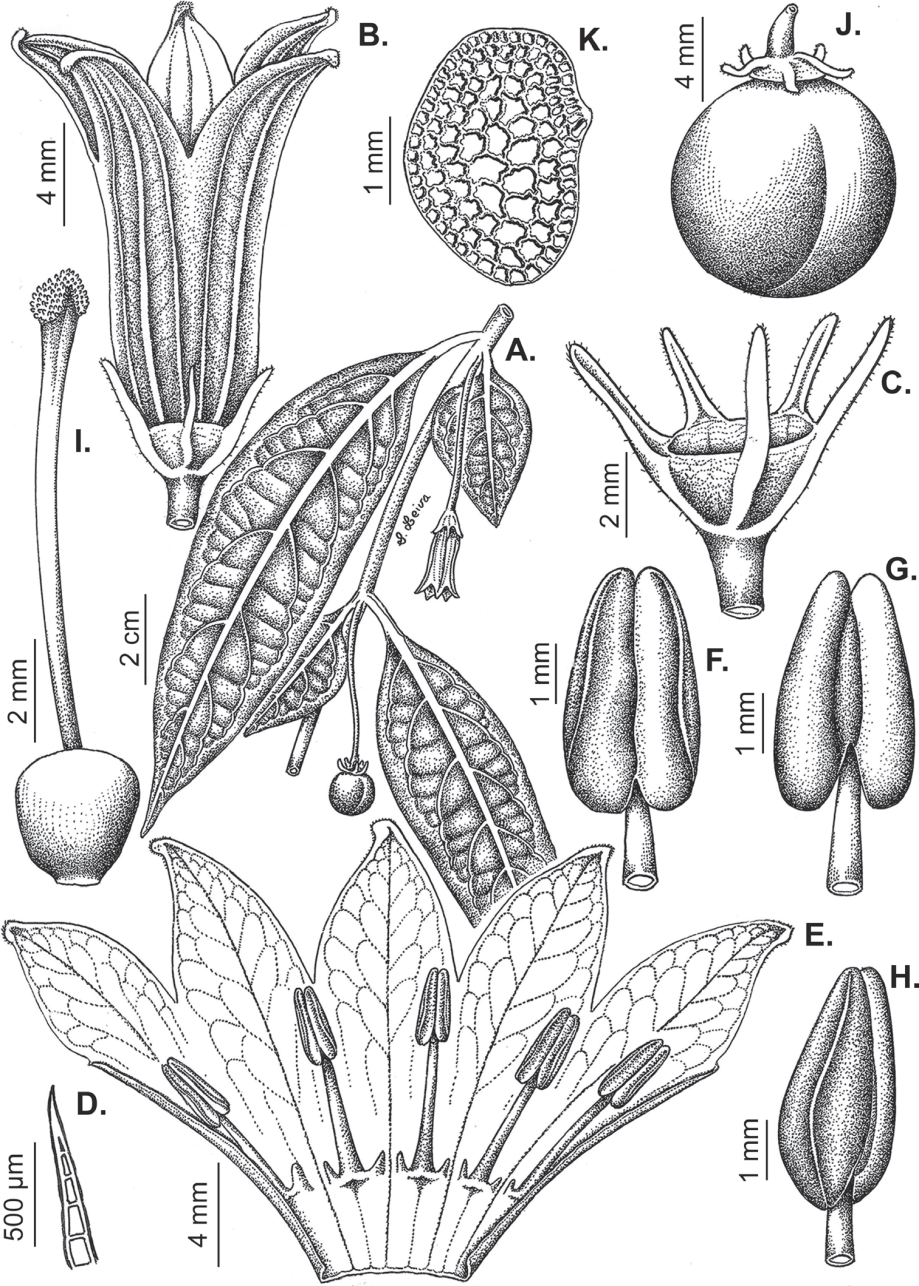
*Capsicumpiuranum***A** reproductive branch **B** flower **C** calyx **D** eglandular trichome of the outer surface of the calyx **E** opened corolla **F, G, H** anthers, ventral, dorsal and lateral views, respectively **I** gynoecium **J** fruit **K** seed. From *G.E. Barboza & S*. *Leiva González 4841.* Drawn by S. Leiva González. Published in Barboza et al. (2019), reproduced with permission.

#### Discussion.

**Figure 102.**
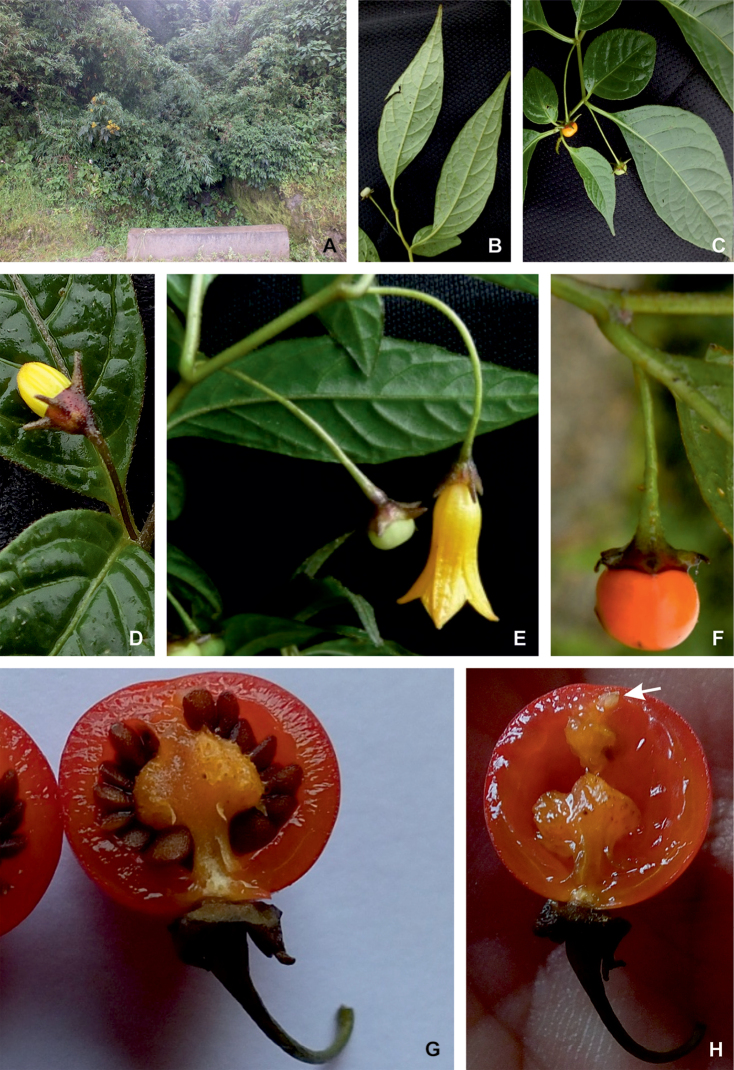
*Capsicumpiuranum***A** plant **B** leaves, abaxial surface **C** Fruiting branch with leaf pair dissimilar in shape and size **D** flower bud **E** flower and immature fruit on pendent pedicels **F** mature fruit **G** fruit, in cross section, showing placenta and seeds **H** fruit, in cross section, showing a stone cell at the apex (arrow). From *Barboza & Leiva González 4841.* Photos by S. Leiva González and G.E. Barboza. Published in Barboza et al. (2019), reproduced with permission.

*Capsicumpiuranum* belongs to the Andean clade (Barboza et al. 2019). It is morphologically most similar to *C.caballeroi* of the Bolivian yungas (Santa Cruz and Cochabamba Departments), based on their campanulate yellow corollas, but they can be easily distinguished. *Capsicumpiuranum* has a purple or greenish-purple calyx with five equal subulate appendages, corolla lobes spreading, smaller fruits (up to 12 mm in diameter), orange to red and non-pungent, pericarp with two stone cells and dark brown to black smaller seeds (Fig. 102D–H). In contrast, *C.caballeroi* has a green calyx with 10 unequal linear appendages, corolla lobes recurved, larger fruits (up to 16 mm), bright red and pungent, stone cells absent and larger seeds pale yellow or nearly white (Fig. 36E–I). The species are not sympatric.

*Capsicumpiuranum* is sympatric with other two Andean species, *C.geminifolium* and *C.rhomboideum*, both of which also have yellow corollas and non-pungent fruits, but a moderate to dense pubescence on stems and leaves. *Capsicumgeminifolium* differs in having longer calyx appendages (3–6.5 mm long) compared to *C.piuranum* (2.5–3 mm long) and campanulate generally purple spotted yellow corollas (tubular-campanulate and entirely pure yellow in *C.piuranum*, Fig. 102E). *Capsicumrhomboideum* has ovate to rhomboid-ovate leaves, up to 13 axillary flowers, campanulate or campanulate-rotate smaller corollas (5–10 mm long) and smaller (up to 9 mm in diameter) bright red to dark burgundy fruits (Fig. 113) in contrast to *C.piuranum*, in which leaves are elliptic (sometimes the minor leaves are ovate, Fig. 102B), the flowers are solitary or up to three (Fig. 102E), the corolla is tubular-campanulate and longer (14.5–17 mm long, Fig. 102E) and the orange to red fruits are larger (9–12 mm in diameter) (Fig. 102F).

#### Specimens examined.

See Suppl. material 4: Appendix 4.

### ﻿36. Capsicum pubescens

Taxon classificationPlantaeSolanalesSolanaceae

Ruiz & Pav., Fl. Peruv. [Ruiz & Pavon] 2: 30. 1799.

A9017D92-FA3D-5D2D-9DB5-9ED1F251A931

Figs 103 , 104

Capsicum violaceum Kunth, Nov. Gen. Sp. [H.B.K.] 3: 49. 1818. Type. Ecuador. Pichincha: Quito, [no date], *F.W.H.A. von Humboldt* & *A.J.A. Bonpland 3027* (holotype: P [P00670654]).
Capsicum quitense Willd. ex Roem. & Schult., Syst. Veg., ed. 15 bis [Roemer & Schultes] 4: 809. 1819. Type. Ecuador. Pichincha: In Quito (holotype: B [B-W04433-01-0]).
Brachistus? lanceifolius Miers, Ann. Mag. Nat. Hist. ser. 2, 3(16): 267. 1849, as “*lanceaefolius*”. Type. Ecuador. Loja: Loja, Aug 1847, *B.C. Seemann 879* (lectotype, designated here: K [K000585923]; isolectotype: BM [BM000992131].
Capsicum maximowiczii Regel & Rach, Index Seminum [St. Petersburg (Petropolitanus)]: 40. 1859. Type. Cultivated in St. Petersburg, Russia [protologue “Cultum in hortis circa Valparaiso sub nomine “Agi dulce”. Semina misit Maximowicz. (Rch.)”] “Ex horto bot. Petropolitano”, 27 May 1858, *L.T. Rach* (no specimens cited; lectotype, designated here: LE).
Capsicum pubescens Ruiz & Pav. var. oviforme Hassk., Bonplandia (Hanover) 8(6): 95. 1860. Type. “Ab incolis Peruviae” (no specimens cited; no original material found).
Capsicum lanceifolium (Miers) Kuntze, Revis. Gen. Pl. 2: 449. 1891, as “*lanceaefolium*”. Type. Based on Brachistus? lanceifolius Miers.
Capsicum annuum L. var. violaceum (Kunth) Alef., Landw. Fl.: 134. 1866. Type. Based on Capsicumviolaceum Kunth.

#### Type.

Peru. Pasco: “Ex Pozuzo” [protologue - “Habitat affatim in Peruviae cultis, praesertim ad Panatahuarum Provinciam et in Andium nemoribus”], *H. Ruiz & J. Pavón s.n.* (lectotype, designated here: MA [MA-815154]; isolectotypes: CORD [CORD 00101751, fragment from G], G, MA [MA-815153, MA-815155]).

#### Description.

Erect and scandent shrubs or perennial herbs, 1–3 (–4) m tall, with the main stem ca. 3 cm in diameter at base, much branched above, the branches in a typical “zig-zag” appearance. Young stems angled, fragile, green or green with purple spots and dark brown ridges, glabrescent to densely pubescent with a soft whitish pubescence of long, spreading, simple, uniseriate, 4–11-celled, eglandular trichomes (some forked) 0.5–1.5 mm long; nodes frequently dark purple; bark of older stems dark brown, smooth or striate, glabrescent to densely pubescent; lenticels absent. Sympodial units difoliate, the leaves geminate; leaf pair markedly unequal in size, similar or dissimilar in shape. Leaves membranous, concolorous or discolorous, green above, light green beneath, rugose (the youngest leaves) or somewhat smooth with the mid-vein and primary veins raised abaxially, glabrescent to densely pubescent on both surfaces and margins, with similar trichomes like those on stems; blades of major leaves 6–12 (–16) cm long, 2.4–4 (–7) cm wide, ovate or more rarely elliptic, the major veins 4–6 on each side of mid-vein, the base asymmetric and attenuate or cuneate, the margins entire, the apex acuminate; petioles 1.5–2 cm long, densely pubescent to glabrescent; blades of minor leaves (2.5–) 3.5–5 (–6) cm long, 1.8–2.4 cm wide, ovate, the major veins 3–4 on each side of mid-vein, the base rounded, the margins entire, the apex acute or acuminate; petioles 0.5–0.7 cm long, densely pubescent to glabrescent. Inflorescences axillary, 1–2 flowers per axil, rarely up to four flowers; flowering pedicels 15–25 mm long, angled, erect, geniculate at anthesis, green or purple-ribbed, moderately to densely pubescent, the eglandular trichomes long, spreading; pedicels scars conspicuous. Buds globose or ovoid, dark purple. Flowers 4–8-merous. Calyx 2–3 mm long, ca. 4–4.3 mm wide, cup-shaped, thick, green, moderately to densely pubescent with the same trichomes as pedicels, sometimes sparse forked trichomes, the calyx appendages 4–8, (0.3–) 0.5–1.5 (–1.7) mm long, subequal or somewhat unequal, thin, erect, cylindrical, green, inserted close to the margin, pubescent with the same trichomes as calyx tube. Corolla 10–15 mm long, 15–22 (–25) mm in diameter, thick, dark purple or violet with a white centre outside and within (sometimes with a weak yellowish-green centre within), rotate to stellate, with thin interpetalar membrane, lobed 1/3 or a little more of the way to the base, pubescent adaxially with short glandular trichomes (stalk 1–2-celled; head globose, unicellular) in the throat and base of the lobes, the tube 5–8 mm long, glabrous abaxially, the lobes 3.5–6.5 (–7.2) mm long, 4.7–7.4 (–8.5) mm wide, broadly triangular, spreading, with eglandular trichomes abaxially especially on the veins, the margins pubescent with very short purple eglandular trichomes, the tips acute or obtuse, cucullate or not, sometimes papillate. Stamens 4–8, equal; filaments 2–3.3 (–4.25) mm long, purple or lilac, inserted on the corolla 1.4–1.6 mm from the base, with auricles fused to the corolla at the point of insertion; anthers 2–2.8 mm long, ellipsoid or ovoid, purple with a wide cream connective, not connivent at anthesis. Gynoecium with ovary 2–3-carpelar, 2–2.5 mm long, ca. 2.5 mm in diameter, light green, ovoid or pear-shaped; ovules more than two per locule; nectary ca. 1.2 mm tall; styles heteromorphic, short, 3–3.5 mm, not exceeding the anthers, medium near the same length as the anthers or long 4.5–5.2 mm, exserted 1–1.5 mm beyond the anthers, lilac or purple, clavate; stigma 0.3 mm long, 0.7 mm wide, discoid or slightly globose, light green. Berry 20–40 mm long, 17–25 mm in diameter (semi-domesticated plants) or larger up to 50 mm long, 55 mm in diameter (cultivated plants), round, blocky or elongate-curved or not, the base obtuse, truncate or lobate, sometimes narrowed forming a neck-like, the apex blunt or sunken, rarely pointed, green when immature, brightly coloured at maturity (from red to light yellow or blackish), persistent, very pungent, the pericarp thick, opaque, with giant cells (endocarp alveolate); stone cells absent; fruiting pedicels 35–50 (–55) mm long, pendent, stout, curved or not, strongly angled, widened distally, usually green; fruiting calyx 8–13 mm in diameter, persistent, slightly accrescent, discoid, green, the appendages 0.8–2.1 mm long, ca. 0.3–0.4 mm wide, spreading. Seeds 15–45 per fruit, 5.5–7 mm long, 4.8–6 mm wide, C-shaped, subglobose or irregular, brownish-black to black, the seed coat reticulate (SM), reticulate-cerebelloid (SEM), the cells polygonal or irregular in shape, the lateral walls wavy to sinuate in the seed body, straight at margins; embryo imbricate.

**Figure 103.**
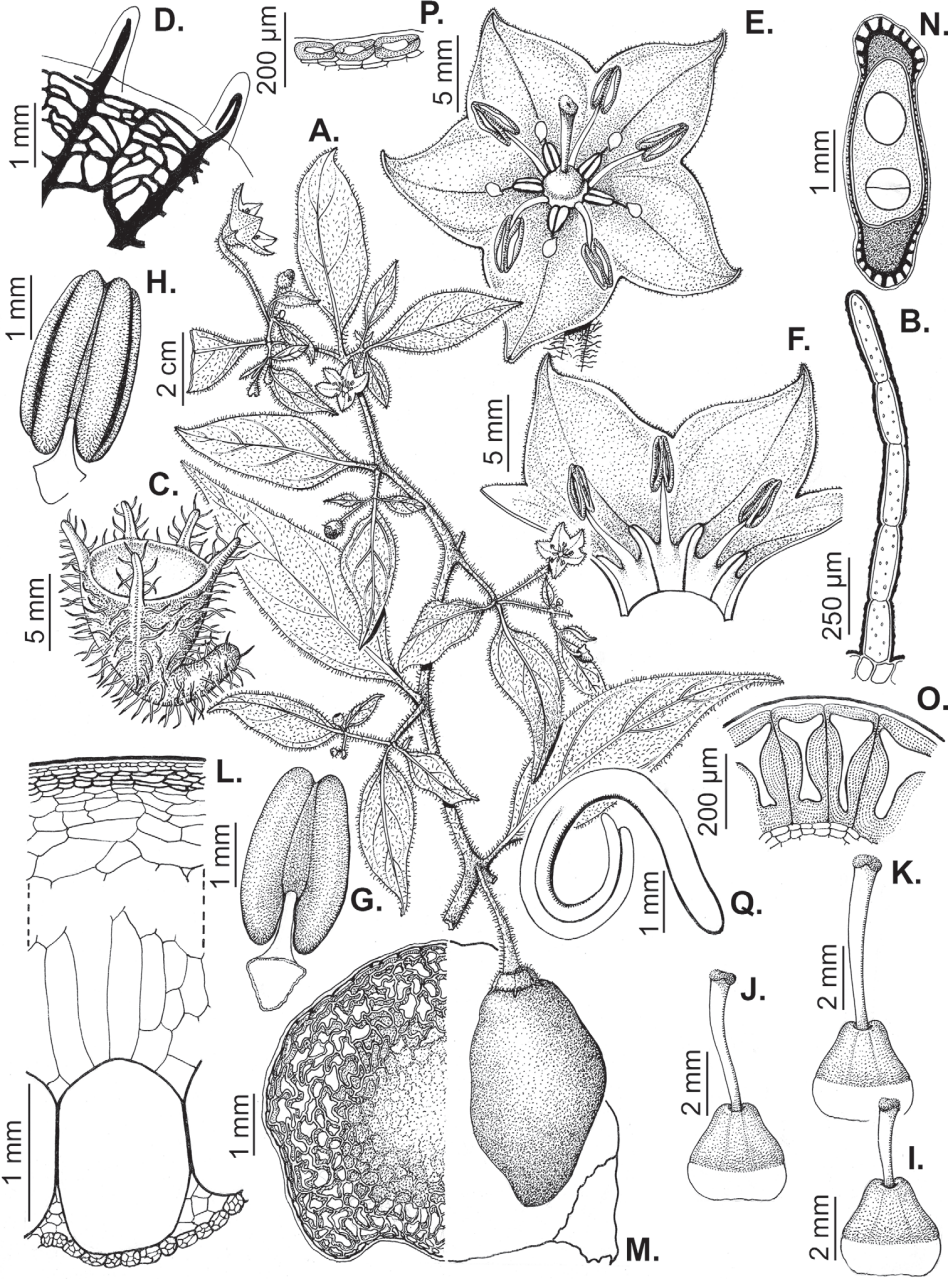
*Capsicumpubescens***A** reproductive branch **B** trichome of the leaf **C** calyx **D** section of the calyx showing the venation **E** flower, upper view **F** sector of opened corolla **G, H** anthers, dorsal and ventral views, respectively **I, J, K** gynoecium with short, medium and long style, respectively **L** anatomical detail of the pericarp (note the giant cell in the mesocarp) **M** seed **N** seed, in cross section **O** structure of seed coat at the seed margin **P** structure of seed coat at the seed body **Q** embryo. From *Hunziker 25484*. Drawn by J. de Ugarte.

**Figure 104.**
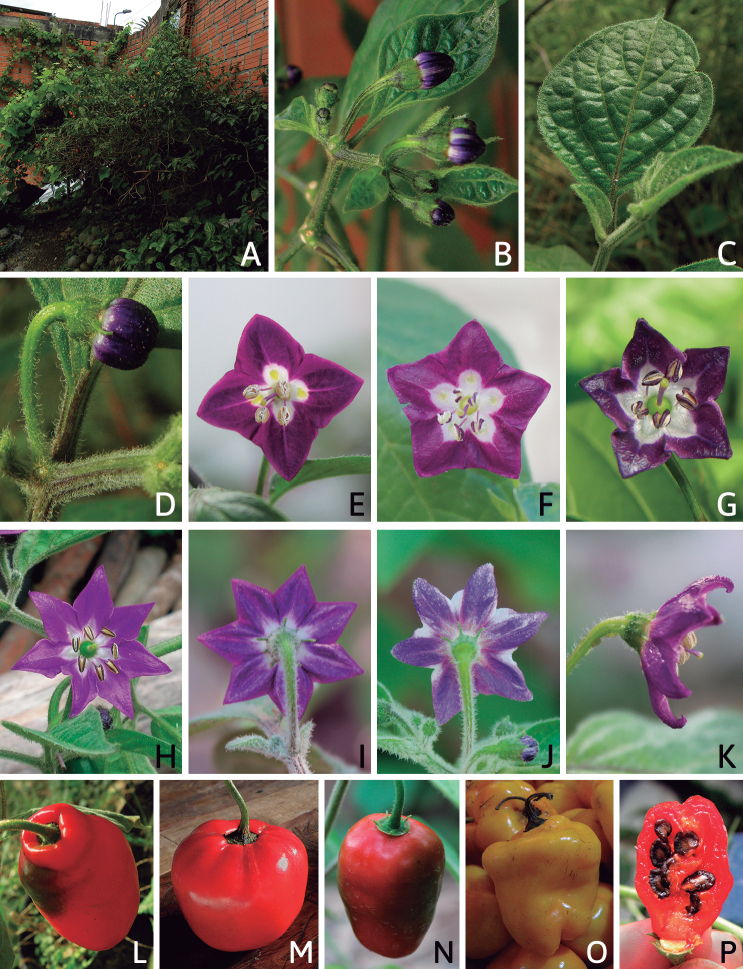
*Capsicumpubescens***A** plant **B** reproductive nodes **C** young leaves, adaxial surface **D** flower bud on geniculate pedicel **E–H** flowers, in front view, showing 4-7-merous corollas **I, J** flowers, seen from behind **K** flower, in lateral view **L–O** mature fruits from different provenance (**L, N** from Bolivia, **M** from Peru, **O** from Mexico) **P** fruit, in longitudinal section, showing the black seeds **A–D, G***Barboza et al. 4889***E, P** from *Barboza et al. 4890***F** from *Palombo 21***H** from *Barboza et al. 1847***I** from *Palombo 22***J** from *Palombo 23***K, L** no specimen vouchers (cult. Córdoba, Argentina) **M** no specimen voucher (cult. Huancayo-Peru) **N** from *Carrizo García et al. 35***O** no voucher specimen (bought in Mexico) **A–D, G, H, L, M, O, P** photos by G.E. Barboza **E, F, I–K, N** photos by N. Palombo.

#### Distribution.

**Figure 105.**
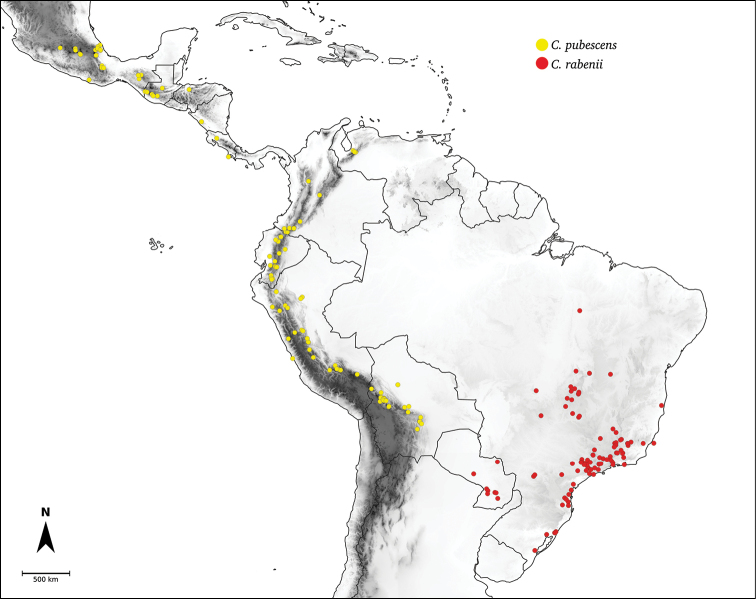
Distribution of *C.pubescens* and *C.rabenii*.

*Capsicumpubescens* is native to Bolivia and Peru (Fig. 105), probably originating in mid-elevation southern Andes (Eshbaugh 1976, 1993; Pickersgill 1984), but confirmed wild populations have not been recorded. Currently, this species is confined to the Americas being moderately cultivated in North America (mainly in Mexico) and Central America and more intensively along the Andean Region of South America, especially from Colombia to Bolivia and less in northern Argentina (Salta and Jujuy), Chile (Arica and Parinacota, cf. Tapia and Campos 2016) and Venezuela. Cultivation of *C.pubescens* outside the Americas is very restricted. A few populations have been reported for Asia (north-western China and Tibetan mountains, Djian-Caporalino et al. 2007) and an introduction was confirmed in Java, Indonesia (Yamamoto et al. 2013). Recent introductions as cash crops were recorded in Nagano, Japan (Matsushima et al. 2010) and a mention as a potential crop in the United Kingdom (Samuels 2014).

#### Ecology.

*Capsicumpubescens* is frequently found in the Andean mid-elevations to highlands from (800–) 1,200–3,500 m and rarely below 500 m elevation. In Indonesia, the cultivation of *C.pubescens* occurs also in highlands, over 1,400 m elevation (Yamamoto et al. 2013).

#### Phenology.

Probably flowering and fruiting all year, depending on the cultivation area.

#### Chromosome number.

2*n* = 2x = 24 (Pickersgill 1977, 1991; Moscone et al. 1993, 1995, 1996a, 2007).

#### Common names.

**Bolivia**: Locato (La Paz, *Heiser C272*), Locoto (La Paz, *Lewis 88629*; Santa Cruz, *Saldías P. 563*), Locote (La Paz, *Duke & Winters 17330*), Locotito (Santa Cruz, *Vargas C. 932*); **Colombia**: Ají (Nariño, *López Jurado* & *Riascos 613*; Putumayo, *Bristol 1115*), Ají rocoto (Huila, *Romero Castañeda 6674*); **Ecuador**: Ají (Chimborazo, *Moina Z 23*; Loja, *Ellemann 66689*; Tungurahua, *Cascante 6*), Ají rocoto (Azuay, *Steyermark 52690*; Pichincha, *Mejía 002*); **Guatemala**: Siete caldos, Caballo (Meckelmann et al. 2015), Chile cuadrocaldo (San Marcos, *Steyermark 36930*), Chile caballo (San Marcos, *Steyermark 36930*); **Honduras**: Chile garrapata (Alta Verapaz, *Standley 91227*), Chile petenero (Morazán, *Valerio R. 3237*); **Mexico**: Chile (Morelos, *Aguilar P. s.n.*), Jalapeño, Perón, Manzano, Ciruelo (Laborde et al. 1982), Chile cera (Veracruz, *Ventura A. 9739*), Chile manzano (México, *Monsalvo J. 12*; Michoacán, *Soto Núñez et al. 6369*), Chile pimiento (México, *Bonilla Beas 346*), Chile canario (Oaxaca, *García R. & Montaño M. 348*; Veracruz, *Castillo C. et al. 1757*), Chile de cepa (Veracruz, *Chazaro B. 2558*), Chile de cera (Veracruz, *Calzada 10856*), Chile gordo (Veracruz, *Castillo C. et al. 214*), Morrón (Veracruz, *Castillo C. et al. 1757*), Moro Ich (Chiapas, *Sánchez León 1139*); **Peru**: Alú, ahi (Loreto, *Killip & Smith 28864*), Ají (Loreto, *Williams 3405*), Locoto (Junín, *Ochoa 602*), Rocobo (Pasco, Ruiz 1940), Rocoto (Ancash, *Gamarra 439*; Cajamarca, *Campos* & *Nuñez 4266*; Cuzco, *Cook* & *Gilbert 1017*; Lima, *Cerrate de Ferreyra 7644*; San Martín, *Quipuscoa* & *Bardales 979*; Pasco, *Chuck 137*), Roccoto (Ruiz and Pavón 1799), Ají rocoto (Andahuaylas, *West 3739*); **Venezuela**: Ají (Mérida, *Pittier 12707*), Ají vocato (*Humboldt* & *Bonpland & 3027*)

#### Indigenous names.

**Bolivia**: Uchu rocoto (= ají globoso) (*Rentzell s.n.*); **Colombia**: Totsha (Kamsá, Putumayo, *Bristol 1115*); **Ecuador**: Huchu (Quechua, Loja, *Ellemann 66689*).

#### Uses.

The fruits of *Capsicumpubescens* are one of the most appreciated in the Andean cuisine for their unique aroma, flavour, meatiness, juiciness and pungency (Meckelmann et al. 2015). Fruits are used fresh, cooked or in powder (as condiment) in different ways in traditional and popular meals (“rocoto relleno”, “ceviche”, “picante de gallina”, “pique macho”, cfr. Meckelmann et al. 2015; Tapia and Campos 2016; Barboza, pers. obs.). In Mexico, fruits are also consumed as a spice and in a great variety of industrial products (Montes Hernández 2010). In South America, indigenous communities (Saraguro people, Ecuador) have attributed medicinal properties to leaves and fruits; in Peru, fruits are used in veterinary practice (see Table 3 for details). In Indonesia, immature and mature fruits are consumed as vegetables and spices (Yamamoto et al. 2013).

#### Preliminary conservation assessment.

EOO (7,150,643 km^2^); AOO (536 km^2^). *Capsicumpubescens* is a widespread cultivated species across the Americas and can be assigned the Least Concern (LC) status.

#### Discussion.

*Capsicumpubescens* belongs to the Pubescens clade (Carrizo García et al. 2016). The origin and affinities of *C.pubescens* are being analysed in depth and preliminary results (using genome-wide SNP data obtained through RAD-sequencing) show *C.pubescens* is sister to a clade formed by *C.eximium*, *C.eshbaughii* and *C.carde­nasii* (Carrizo García et al. 2019; CCG, pers. obs.). Therefore, the circumscription of the Pubescens and Purple corolla clades (after Carrizo García et al. 2016) is being re-assessed (Carrizo García and Palombo 2019; CCG, pers. obs.). *Capsicumpubescens* was domesticated and has been highly appreciated by early Peruvian peoples for 4,000 years before the present (Perry et al. 2007), while its introduction in Central America and Mexico has occurred in the twentieth century (Laborde et al. 1982; McLeod et al. 1982). *Capsicumpubescens* is very distinctive in the combination of the following characters, with minor differences in cultivars: habit, general pubescence, shape, size and colour of flowers and seeds and heteromorphic style (Figs 103, 104). *Capsicumpubescens* is an erect to scandent shrub up to 4 m high, with a dense white, soft pubescence covering stems, leaves, pedicels and calyx (sometimes plants are glabrescent), with rugose young leaves, large rotate or rotate-stellate 4–8-merous corollas that are usually deep purple, heteromorphic styles with three different lengths and the largest brownish-black to black seeds (5.5–7 mm long, 4.8–6 mm wide) in the genus. Variations in corolla colour have been observed throughout its distribution, from dark purple to lighter tonalities (near rose colour) or completely lacking purple pigmentation (Eshbaugh 1979). Pure white corollas (filaments and style white) have also been observed as a rare mutant in cultivated *C.pubescens* plants in Indonesia (Yamamoto et al. 2013).

The fruit is the most variable character in this species and its different common names refer to this (Heiser and Smith 1948; Rick 1950; Eshbaugh 1979). In the Andean highlands, the most popular names (and some deviations of these words), are “locoto”, an Aymara word (luqutu), meaning ‘piquant’, used in Bolivia (also in Argentina and Chile) and “rocoto”, a Quechua word (rukutu) meaning ‘pepper very piquant’ as is mostly known in Peru and Ecuador. In Central America and Mexico, the common names refer to fruit shapes and colours, to the sensation caused by capsaicinoids in humans or to a particular use in the cuisine (DeWitt and Bosland 2009; Meckelmann et al. 2015). Fruits are large as in the other cultivated species and very attractive at maturity because of their shape and bright colours (red, orange-red, orange, orange-yellow, yellow, light yellow, nearly black). They can be more or less spherical, blocky or elongate (Fig. 104L–P); in the first case, they are depressed with the apex truncate or rounded, such as in the “canario” (bird canary, fruit roundish and yellow), “manzano” (apple-shaped and red) and “peron” (large pear-shaped and yellow fruit) varietals (DeWitt and Bosland 2009); the elongate fruits can be ovoid or elliptic, curved or not, with the apex truncate, rounded or acute and the base sometimes narrowed forming a conspicuous neck-like shape. In Mexico, *C.pubescens* fruits are also called “chile de cera” (cera = wax) in allusion to the soft and brilliant pericarp.

In the protologue of *C.pubescens*, Ruiz and Pavón (1799) stated “Habitat affatim in Peruviae cultis, praesertim ad Panatahuarum Provinciam et in Andium nemoribus”. The three specimens of *C.pubescens* in the Ruiz and Pavón Herbarium at MA (MA-815153, MA-815154, MA-815155) are labelled as being collected in Pozuzo (“Ex Pozuzu”) and are not in exact agreement with the protologue of *C.pubescens*. In his posthumously published journals, however, Ruiz (1940) clearly states the record of the travels of the expedition “Among the plants that I described while we remained in Puzuzo are the following [p. 175] … *Capsicumfrutescens* L., arnaucho and *pubescens*, rocobo; both species very abundant in Peru” [p. 176]. We, therefore, assume that all of these specimens labelled as coming from “Pozuzu” belong to the type gathering of *C.pubescens*. From amongst these three sheets, we have selected the best fertile specimen as the lectotype (MA-815154). A sheet at G from “herb. Pavon” appears to be a duplicate of MA-815154 and a fragment taken from the sheet at G is housed at CORD.

Miers (1849) cited two collections in the protologue of Brachistus?lanceifolius: *Seemann 879* & *McLean s.n.* that he saw in “herb. Hook.”, now held at Kew. The Seemann collection (K000585923) consists of two flowering branches and has Seemann’s original label with the collecting locality; the McLean collection (K000585921) has mounted on it another branch collected near Lima by W. Nation (K000585922) that also fits within the circumscription of *C.pubescens*, but is not part of the type material. We select the Seemann collection (K000585923) as the lectotype of *B.lanceifolius* as it is the most complete and best-preserved of the specimens cited by Miers.

The protologue of *C.maximowiczii* (Kuester et al. 1859) provides a complete description for this species, based on a specimen grown in St. Petersburg from seeds sent to Karl Maximowicz (at the time curator of the Herbarium in St. Petersburg) of a plant cultivated in Valparaiso (Chile). No specimens are indicated and it is likely the description was based on living plants. The corollas are said to be hexamerous and violet and the seeds black, which matches with *C.pubescens*. However, the common name “agi dulce” referring to a sweet pepper is not usually used in reference to *C.pubescens* and could be an error. We found a sterile branch with the script “v.v. Rach” [seen alive by Rach] dated 27 May 1858 at LE; this is certainly original material and we designate it the lectotype.

#### Specimens examined.

See Suppl. material 4: Appendix 4.

### ﻿37. Capsicum rabenii

Taxon classificationPlantaeSolanalesSolanaceae

Sendtn., Fl. Bras. (Martius) 10(6): 145. 1846.

05D722DE-3C21-5775-891A-48FBEF352A16

Fig. 106

Capsicum microcarpum Cav. var. tomentosum Chodat & Hassl., Bull. Herb. Boissier 2,4: 80. 1903. Type. Paraguay. Paraguarí: In regione collium, Cerros de Paraguay, Dec 1900, *É. Hassler 6498* (lectotype, designated by Barboza 2011, pg. 27, second step designated here: G [G00390271]; isolectotypes: BM [BM000087632, acc. # 5447784; BM000087632a, acc. # 4575838], CORD [CORD00006947 fragment from G], G [G00390272, G00390273], K [K000585894], P [P00410166], MO [MO-2530203, acc. # 1575002], MPU [MPU023043], S [S16-28128], UC [UC944377]).
Capsicum praetermissum Heiser & P.G.Sm., Brittonia 10(4): 198. 1958. Type. Brazil. Minas Gerais: Viçosa, road to São Miguel, near km 4, 700 m elev., 3 Jan 1930, *Y. Mexia 4205* (holotype: F [F-0093991F]; isotypes: BM [BM000992134], CAS [CAS0002376], CORD [CORD00086136], GH [GH00077006], K [K001073038], NY [00138603], MICH [1109872], MO [MO-503526, acc. # 1164250], U [U.1736360], UC [UC509516], S [S-04-2815], US [US00027398], Z [Z000028484]).
Capsicum baccatum L. var. praetermissum (Heiser & P.G.Sm.) Hunz., Kurtziana 6: 242. 1971. Type. Based on Capsicumpraetermissum Heiser & P.G.Smith

#### Type.

Brazil. Rio de Janeiro: “In prov. Sebastianopolitana: Com. de [F. C.] Raben (n. 301)” (lectotype, designated here: BR [BR 0000005529261].

#### Description.

Erect shrubs or scandent subshrubs (0.50–) 0.70–2.5 (–3) m tall, with the main stem 2–2.5 cm in diameter at base, rarely perennial herbs, much branched from near the base and above. Young stems angled, somewhat rigid, green, pubescent with appressed-antrorse or spreading, simple, uniseriate, 4–8-celled, eglandular trichomes 0.3–1.5 mm long, the new growth with a dense whitish pubescence; nodes green or purple; bark of older stems striate, dark brown, glabrescent; lenticels absent. Sympodial units difoliate, the leaves geminate; leaf pair subequal in size and similar or dissimilar in shape. Leaves membranous, slightly discolorous, green above, light green and with the primary veins raised beneath, glabrescent to moderately pubescent, with appressed-antrorse trichomes like those on stems on adaxial surface and margins, sparsely pubescent on the lamina, but with tufts of eglandular trichomes in the vein axils and with long spreading trichomes along the veins giving a woolly appearance abaxially; blades of major leaves 5.3–10 cm long, 2.4–6.5 cm wide, ovate, the major veins 4–6 (–7) on each side of mid-vein, the base asymmetric and attenuate, the margins entire, the apex acuminate or long-acuminate; petioles (1.5–) 2.5–3.5 cm long, winged distally for the decurrent base of the lamina, moderately to densely pubescent; blades of minor leaves 3–4 cm long, 1.4–2 cm wide, ovate or elliptic, the major veins 2–3 on each side of mid-vein, the base rounded or truncate, the margins entire, the apex acute or acuminate; petioles 0.8–1.2 cm long, moderately to densely pubescent. Inflorescences axillary, 2–4 flowers per axil; flowering pedicels (8–) 12–18 (–25) mm long, angled, erect, geniculate at anthesis, moderately to densely pubescent, the eglandular trichomes spreading or antrorse; pedicels scars inconspicuous. Buds globose, whitish-green or with green spots at the base. Flowers 5-merous, rarely 4-merous. Calyx 1.5–2 mm long, ca. 2–2.5 mm wide, cup-shaped, thick, green, pubescent with the same trichomes as pedicels, the calyx appendages 5, 0.8–1.1 mm long, 0.3 mm wide, subequal, thick, erect, cylindrical, inserted close to the margin, moderately pubescent with the same trichomes as calyx tube. Corolla 6–7 mm long, (9–) 12–15 mm in diameter, thick, white or lilac and greenish-yellow outside, mostly purple or lilac with greenish-yellow or green spots and cream centre within, rotate to stellate with interpetalar membrane, lobed less than 1/3 to nearly halfway to the base, pubescent adaxially with short glandular trichomes (stalk 1–3-celled; head globose, peltate, unicellular), glabrous abaxially, the tube 4–4.5 mm long, the lobes 2–2.5 mm long, 2.2–4 mm wide, broadly triangular, spreading, the margins with very short eglandular trichomes, the tips acute, papillate. Stamens five, rarely four, equal; filaments (0.8–) 1.2–1.5 (–2) mm long, purple or lilac, inserted on the corolla 0.8 mm from the base, with auricles fused to the corolla at the point of insertion; anthers 1.2–1.5 (–1.8) mm long, ellipsoid, white, cream or yellow, not connivent at anthesis. Gynoecium with ovary 1.3–1.5 mm long, 1.4–1.5 mm in diameter, green, ovoid; carpels two, ovules more than two per locule; nectary ca. 0.3 mm tall; styles homomorphic, 2.6–2.8 mm long, exserted 0.8–1.2 mm beyond the anthers, yellowish-white, cylindrical; stigma 0.5 mm long, 0.7–0.75 mm wide, discoid, pale green. Berry globose or subglobose, 5–7.5 mm in diameter or ellipsoid, 7–11 mm long, (4–) 6–8 mm in diameter, green when immature, orange-coloured or bright red at maturity, deciduous, pungent, the pericarp thick, opaque, with giant cells (endocarp alveolate); stone cells absent; fruiting pedicels (14–) 20–28 mm long, erect, strongly angled, widened distally, green; fruiting calyx 4–4.5 mm in diameter, persistent, not accrescent, cup-shaped or discoid, green, the appendages 1–2 mm long, ca. 0.3–0.4 mm wide, erect or spreading. Seeds (5–) 7–15 per fruit, 3–4.2 mm long, 2.5–2.8 mm wide, C-shaped or subglobose, pale yellow or yellow, the seed coat smooth (SM), reticulate-cerebelloid (SEM), the cells irregular in shape, the lateral walls sinuate in seed body, straight at margins; embryo imbricate.

#### Distribution.

*Capsicumrabenii* is native to eastern and central Brazil, occupying a large range in Brazil from Bahia to Rio Grande do Sul States and into central Paraguay (Fig. 105).

#### Ecology.

*Capsicumrabenii* occurs in a wide variety of vegetation types, from the Atlantic Forest (Semi-deciduous Seasonal Forest, Ombrophilous Forest) to xerophytic forests in the Cerrado and Caatinga; it can be found in disturbed gallery forest, calcareous outcrops vegetation, creek and river margins and as ruderal around anthropogenic and cultivated areas, between (35–) 100 and 1,300 m elevation. It is also frequent as an escape from cultivation or in cultivation around houses and growing commercially in small farms.

#### Phenology.

Flowering from November to May. Fruiting from December to May and June extending to September when in cultivation.

#### Chromosome number.

*n* = 12 (Heiser and Smith 1958, as *C.praetermissum*; Pozzobon and Schifino-Wittmann 2006 as C.baccatumvar.praetermissum); 2*n* = 2x = 24 (Pickersgill 1977, as *C.praetermissum*; Pozzobon et al. 2006; Moscone et al. 2007; Scaldaferro et al. 2016, counts as C.baccatumvar.praetermissum).

#### Common names.

**Brazil**: Comarim (Minas Gerais, *Krieger 5212*), Pimenta (Mina Gerais, *Mexia 4205*; São Paulo, *Briske s.n*.), Pimentinha (Santa Catarina, *Reitz 4749*), Pimenta brava (Minas Gerais, *Anonymous s.n*.), Pimenta comary (São Paulo, *Handro 860*), Pimenta camari (São Paulo, *Coelho s.n.*), Pimenta cumarí (Goiás, *Vieira 700*; Minas Gerais, *Krieger 5212*; São Paulo, *Hoehne & Gehrt s.n.*), Pimenta cumarina (Minas Gerais, *Tavares et al. 17*), Pimenta-cumari-verdadeira (Santa Catarina, *Schwirkowski 885*), Pimenta Jorobinha (Rio de Janeiro, *Glaziou 8841*), Pimenta Passarinho (Southeast Brazil, Ferreira Pinto et al. 2016), Pimenta de combari (Minas Gerais, *Lindberg 177*), Pimenta do mato (Rio Grande do Sul, *Costa Sacco 1332*; Espírito Santo, *Sucre & Soderstrom 8987*).

#### Uses.

In Brazil, fresh fruits are very appreciated in markets (Ferreira Pinto et al. 2016), used as a condiment and consumed as pickles and in hot sauces.

#### Preliminary conservation assessment.

EOO (2,746,764 km^2^); AOO (404 km^2^). *Capsicumrabenii* is well-adapted to different environments including highly disturbed anthropogenic areas and is easily cultivated in farms, which suggest that it is not under threat. This species is proposed as Least Concern (LC).

#### Discussion.

*Capsicumrabenii* is a member of the Baccatum clade (Carrizo García et al. 2016, as *C.praetermissum*). The species has a wide distribution in Brazil where it is mostly known as ‘pimenta comari’ or “Cumari” (as are also *C.baccatum*, *C.chacoense* and *C.flexuosum*) in allusion to its pungent red fruits. It grows spontaneously in many areas but can also be found semi-domesticated and under cultivation in small gardens or in larger scale in farms for commercial use (Pickersgill 1969a; Eshbaugh 1993; Ferraz et al. 2016). In Paraguay, this species is less common and the fruits appear to be not so appreciated and consumed as in Brazil.

**Figure 106.**
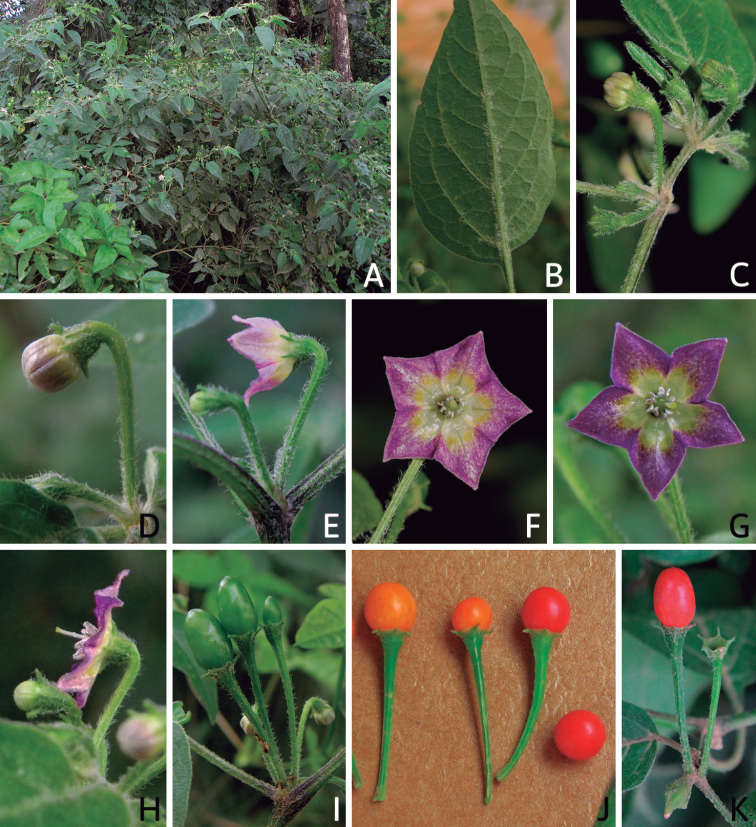
*Capsicumrabenii***A** plant **B** leaf, abaxial surface showing pubescence on the veins **C** reproductive nodes **D** bud on geniculate pedicel **E** flower, lateral view **F** rotate corolla **G** stellate corolla **H** flower showing the exserted style **I** immature fruits **J** globose mature fruits **K** ellipsoid mature fruit and fruiting calyx **A–I, K** from *Barboza & Deanna 5003***J** from *Barboza et al. 1646.* Photos by G.E. Barboza.

Flowering and fruiting specimens of *C.rabenii* are very similar to C.baccatumvar.baccatum on herbarium specimens, it sometimes being impossible to distinguish one from another if there are no annotations of corolla colour or if specimens are only in fruit. Both species have long petiolate leaves, rotate or rotate-stellate corollas, orange to red globose or elliptic upright fruits and pale yellow seeds. *Capsicumrabenii* is distinguished, however, not only for its purple-margined corollas (Fig. 106E–H), but also for the dense woolly pubescence of the leaves abaxially with spreading trichomes along the main veins and a conspicuous tuft of trichomes in the vein axils (Fig. 106B); these features distinguish *C.rabenii* from C.baccatumvar.baccatum that lacks anthocyanin pigmentation in the corollas and has usually glabrescent leaves (Hunziker 1998; Barboza 2013). Only one individual of *C.rabenii* has been found with completely white corollas in São Paulo State (Brazil, *Hunziker 19564*) growing in the same environmental conditions with other specimens with purple-margined corollas (*Hunziker 19563*); this lack of pigmentation may be due to a recessive gene. Similarly, some specimens from Goias State (Brazil) lack dense pubescence along the veins but the tuft of trichomes on the secondary vein axils and the typical corollas with the purple band at the margins are present in these populations.

*Capsicumrabenii* differs from *C.chacoense*, with which is sympatric in Paraguay, in its rotate, coloured corollas and the characteristic pubescence of the leaves beneath, while *C.chacoense* has stellate, entirely white corollas and a variable pubescence, but is never woolly (Fig. 46).

It is possible that *C.rabenii* hybridises naturally with *C.baccatum* and *C.frutescens* in Brazil as has already been shown through reciprocal crosses that produced some fruits with viable seeds (Heiser and Smith 1958), but intermediates amongst these species have not been observed in field. Our observations indicate that the three species are usually distinguishable using the corolla colour, the pubescence (see above) and calyx features.

When Heiser and Smith (1958) described *C.praetermissum*, they stated that none of the species of “Flora Brasiliensis” (Sendtner 1846) matched with their new species; they did not realise that the description of *C.rabenii* fitted very well with *C.praetermissum* in the pubescence of leaves and shape of the corolla, characters clearly observable in the type collections of both species. Hunziker (1971) proposed *C.praetermissum* as a variety of *C.baccatum* and he recognised *C.rabenii* as synonym of his new varietal name. Hunziker preferred to use the epithet *praetermissum* because of the valuable and unequivocal information that Heiser and Smith (1958) provided on this plant. According to the ICN (Art. 11.2, Turland et al. 2018), Hunziker could use the name *praetermissum* because *rabenii* was in a different rank and, consequently, the epithet *rabenii* passed into oblivion. Since then, many authors have spread both names *C.praetermissum* and C.baccatumvar.praetermissum in literature and on herbarium specimens. Enzymatic (McLeod et al. 1983b), cytogenetic (Moscone et al. 2007) and molecular studies (Kochieva et al. 2004; Ibiza et al. 2012; Carvalho et al. 2014; Carrizo García et al. 2016), plus the clear morphological characters discussed above, provide more than enough evidence that *C.baccatum* and *C.praetermissum* are distinct species. As *C.rabenii* has priority over *C.praetermissum*, the first becomes the correct name for this species, despite the previous more common usage of the latter in both literature and on herbarium sheets.

Sendtner (1846) cited a single collection in the description of *C.rabenii*, “Com. de Raben (n.301)” (Danish Count Frederik Christian Raben). A specimen at BR labelled “Com. Raben” with the number 301 written on it in another hand is the only original material we have found and we designate it the lectotype. A specimen in C (C10019144) labelled as “Herbarium Fr. Chr. Comitis Raben” and “Lago de Rodriguez. R. de Janeiro. 1835”, has been suggested as original material by Olof Ryding on an additional annotation slip on this sheet stating “… It matches the holotype of *Capsicumrabenii* Sendtn. at BR. The two [BR and C] specimens apparently belonged to the same collection … O. Ryding 1999” (see also Ryding 2002 for further information on Raben collections). We compared these specimens and they are clearly not the same. The lectotype at BR has leaves that are densely pubescent abaxially, matching Sendtner’s (1846) description, while the specimen at C has leaves that are glabrous or glabrescent on both surfaces, belonging to C.baccatumvar.baccatum.

#### Specimens examined.

See Suppl. material 4: Appendix 4.

### ﻿38. Capsicum recurvatum

Taxon classificationPlantaeSolanalesSolanaceae

Witasek, Denkschr. Ak. Wissensch. Wien, Mathem. Naturw. Kl. 79(2): 321. 1910.

98B54609-CD82-5EB9-BB8A-62351F149F76

Figs 107 , 108

#### Type.

Brazil. São Paulo: Apiahy, Nov 1888, [*G.L.T. Puiggari*] *3577* (lectotype, designated here: WU [acc. # 0037948]; isolectotype: CORD [CORD00006951 fragment from WU]).

#### Description.

Erect shrubs (1–) 1.5–4 m high, with the main stem thick, 2–2.5 cm in diameter at base, much branched above, the branches dichotomously spreading in a typical “zig-zag” appearance. Young stems angled, fragile, green or light green, glabrescent to moderately pubescent, with antrorse, curved, simple, uniseriate, 3–6 (–8)-celled, eglandular trichomes 0.3–0.9 mm long; nodes solid, purple or green; bark of older stems dark brown, striate, glabrous; lenticels few. Sympodial units difoliate, the leaves geminate; leaf pair unequal in size, similar or dissimilar in shape. Leaves membranous, discolorous, dark green above, light green beneath, glabrous to moderately or densely pubescent on both surfaces and margins, especially on the veins beneath, with uniseriate, 2–5-celled, eglandular trichomes 0.9–1.2 mm long; blades of major leaves 5–16 (–20) cm long, 1.4–4.8 cm wide, elliptic or narrowly elliptic to ovate, the major veins 4–6 (–7) on each side of mid-vein, the base asymmetric and attenuate, the margins entire, the apex acute or acuminate; petioles 0.3–1.5 (–2) cm long, moderately pubescent; blades of minor leaves 3.2–4.7 cm long, 1.4–2.2 cm wide, elliptic or ovate, the major veins 3–4 on each side of mid-vein, the base attenuate, the margins entire, the apex acute or obtuse; petioles 0.2–0.4 cm long, glabrous or moderately pubescent. Inflorescences axillary, 2–4 flowers per axil, rarely flowers solitary; flowering pedicels 9–20 mm long, terete, sometimes slightly striate, erect, geniculate at anthesis, green, with sparse antrorse eglandular trichomes; pedicels scars inconspicuous. Buds globose, inflated, white with greenish-yellow spots. Flowers 5-merous. Calyx 1.5–2 mm long, ca. 2–2.7 mm wide, cup-shaped, thick, green, glabrous to moderately pubescent, with 2–4-celled eglandular trichomes and small glandular trichomes (stalk one-celled; head dark, multicellular), the calyx appendages 5–9 (–10), the five main appendages longer and subequal 1–2.5 mm long, 0.2–0.5 mm wide, the 1–4 (–5) secondary appendages shorter and unequal 0.1–0.7 mm long, 0.2–0.5 mm wide, spreading or recurved, cylindrical or more commonly triangular, laterally compressed, green, inserted 0.1–0.3 mm below the margin leaving a rudimentary membranous hyaline sleeve portion. Corolla 6–7 mm long, ca.11 mm in diameter, thick, white with green spots outside, white with different patterns of greenish-yellow spots in the base of the lobes and in the throat and a white centre within, stellate without interpetalar membrane, lobed more than 1/3 to nearly halfway to the base, the tube 3–3.5 mm long, pubescent adaxially with a continuous ring of glandular trichomes (stalk long, 1–2-celled; head globose peltate, unicellular), glabrous abaxially, the lobes 3–3.7 mm long, 3.5–4 mm wide, triangular, spreading, glabrous adaxially and abaxially, the margins papillate, the tips cucullate, papillate. Stamens five, equal; filaments 2–2.5 mm long, white, inserted on the corolla 1–1.2 mm from the base, with auricles fused to the corolla at the point of insertion; anthers 1–1.8 (–1.9) mm long, ellipsoid, yellow, light green or grey, brownish post-dehiscent, not connivent at anthesis. Gynoecium with ovary 1–1.8 mm long, 1–1.3 mm in diameter, green, subglobose; ovules more than two per locule; nectary 0.3–0.5 mm tall; styles homomorphic, 3.2–3.5 mm long, exserted 0.8–1 mm beyond the anthers, white, clavate; stigma 0.2 mm long, 0.5–0.9 mm wide, discoid, pale green. Berry 7–9 mm in diameter, globose, green when immature, greenish-golden yellow at maturity, deciduous, very pungent when immature and rather sweet at maturity, the pericarp thin, translucent, with giant cells (endocarp alveolate); stone cells absent; fruiting pedicels 18–25 mm long, pendent, angled, widened distally, green; fruiting calyx (3–) 4–4.5 mm in diameter, persistent, not accrescent, discoid, green, the appendages 0.4–2.6 mm long, 0.3–0.5 mm wide, strongly recurved and compressed. Seeds (4–) 8–25 per fruit, 2.5–2.8 mm long, 2–2.5 mm wide, C-shaped, reniform or subglobose, dark brown or black, the seed coat reticulate-tuberculate (SM), reticulate with pillar-like outgrowths (SEM), the cells irregular or polygonal in shape, the lateral walls wavy to sinuate in the seed body and straight at margins; embryo imbricate.

**Figure 107.**
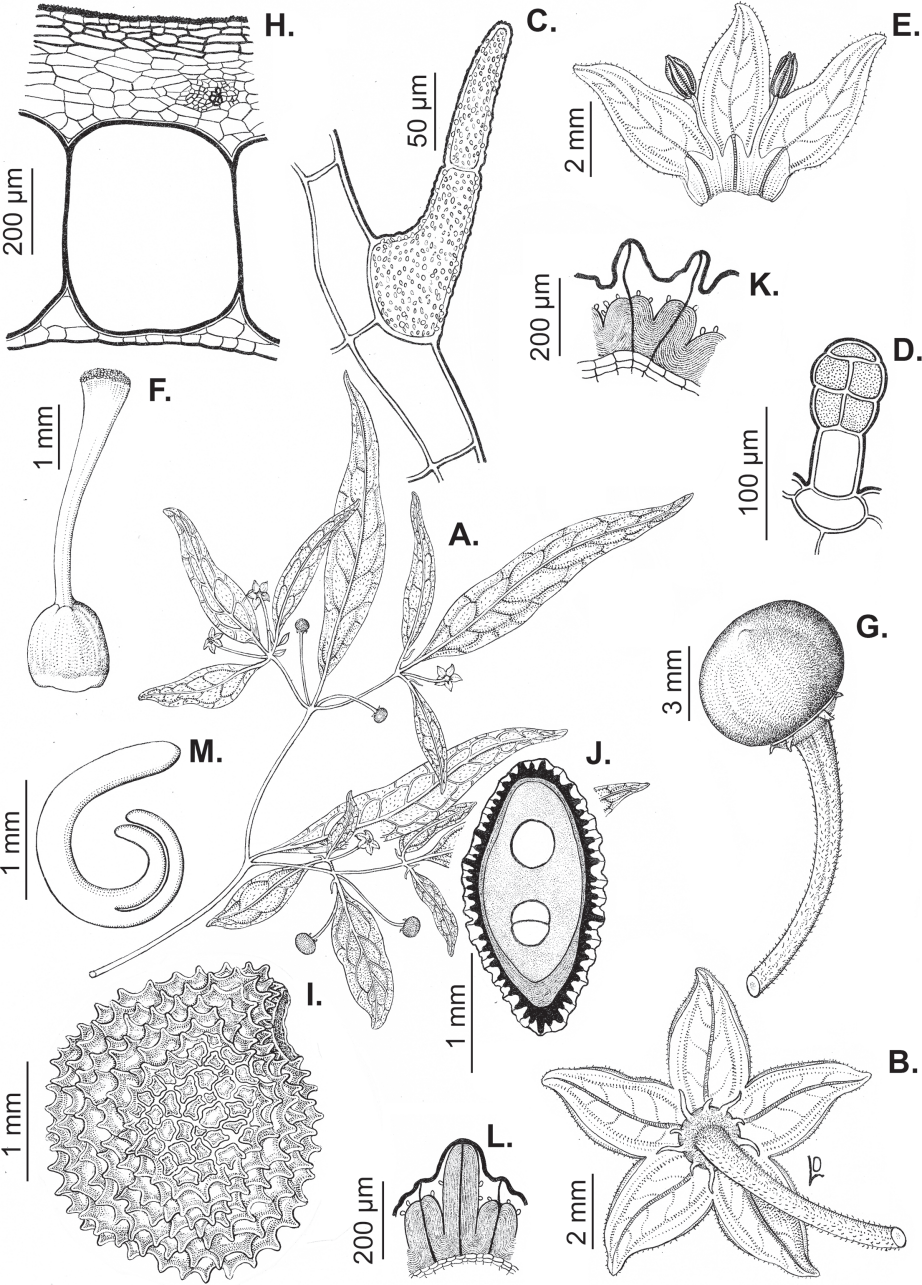
*Capsicumrecurvatum***A** reproductive branch **B** flower, seen from behind **C** eglandular trichome of the outer surface of the calyx **D** glandular trichome of the inner surface of the calyx **E** sector of opened corolla **F** gynoecium **G** fruit **H** anatomical detail of the pericarp (note the giant cells in the mesocarp) **I** seed **J** seed, cross section **K** structure of seed coat at the seed margin **L** structure of seed coat at the seed body **M** embryo **A, C, D, G, H** from *Hunziker 20775***B, E, F** from *Hunziker 19546***I–M** from *Barboza et al. 2023*. Drawn by L. Ochoa.

**Figure 108.**
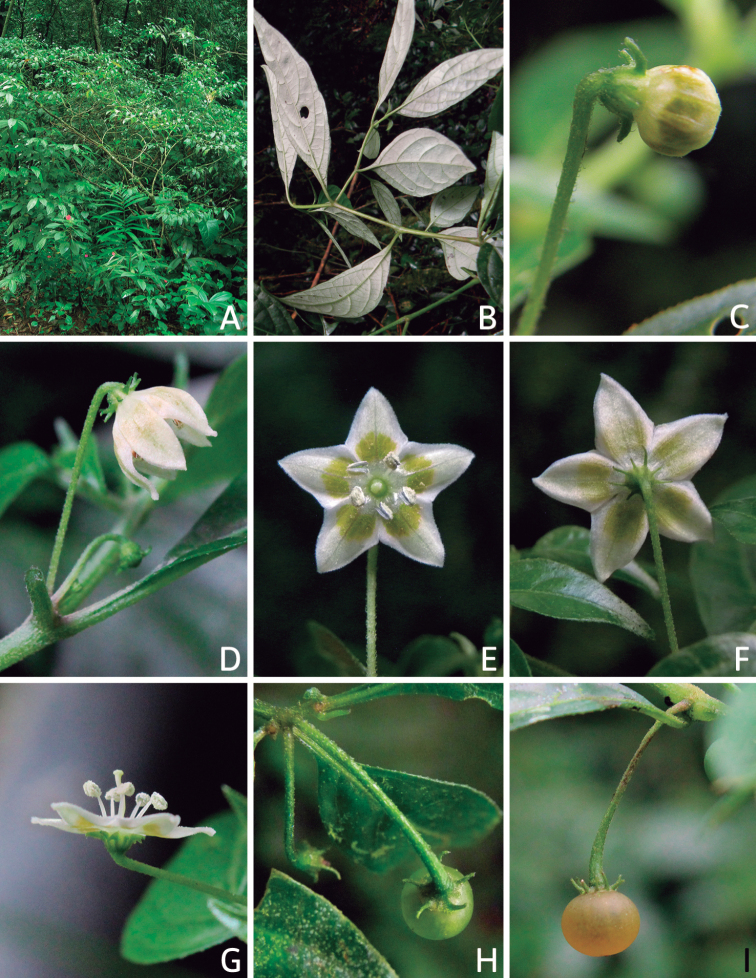
*Capsicumrecurvatum***A** plant **B** apical branch, showing leaves abaxial surface **C** flower bud **D** flower, in pre-anthesis **E** flower, in front view **F** flower, seen from behind **G** flower showing exserted style **H** immature fruit **I** mature fruit. From *Barboza & Deanna 5004*. Photos by G.E. Barboza.

#### Distribution.

**Figure 109.**
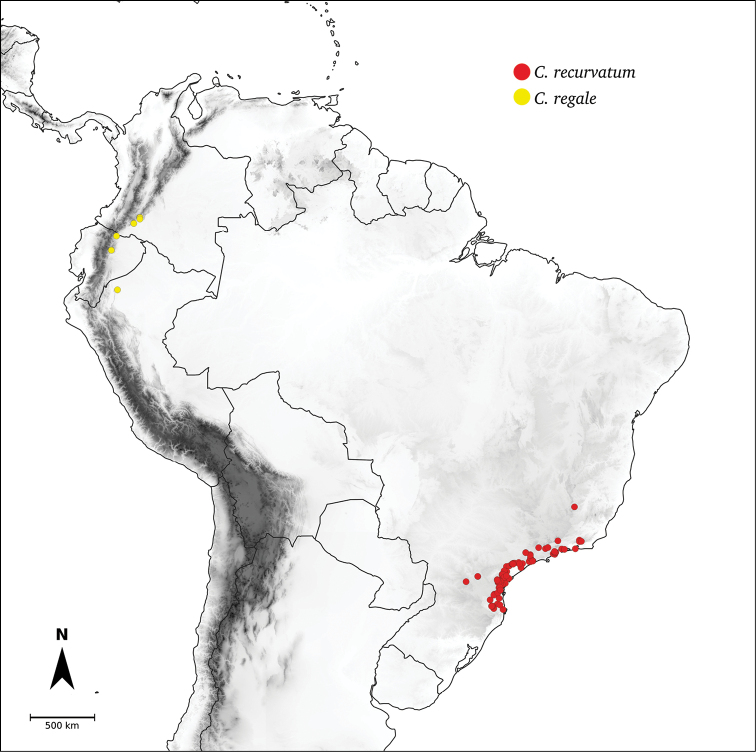
Distribution of *C.recurvatum* and *C.regale*.

*Capsicumrecurvatum* is endemic to south and south-eastern Brazil (Minas Gerais, Paraná, Rio de Janeiro, Santa Catarina and São Paulo States) (Fig. 109).

#### Ecology.

*Capsicumrecurvatum* is quite common along the Serra do Mar, in different formations of the coastal Atlantic Forest (Floresta Ombrófila Densa Montana, Floresta Ombrófila Densa Submontana, Floresta Estacional Semidecídual, Floresta Ombrófila Mista, Floresta Tropical Pluvial Atlântica de Encosta and Floresta Tropical de Encosta). It is found growing on moist ground mainly in semi-shade in primary or secondary forests, between 50 and1,800 m elevation.

#### Phenology.

Flowering from late October to April; in fruit from January to June.

#### Chromosome number.

*n* = 13 (Pozzobon and Schifino-Wittmann 2006, as *Capsicum* sp. 2); 2*n* = 2x = 26 (Pozzobon et al. 2006, as *Capsicum* sp. 2; Moscone et al. 2007).

#### Common names.

**Brazil**: Pimenta silvestre (São Paulo, *Hoehne s.n*.), Pimenta de bugre (Santa Catarina, *Reitz C 924*), Pimenta-do-mato (Santa Catarina, *Schwirkowski 139*), Pimenta do pasarinho (São Paulo, *Barboza et al. 2023*).

#### Uses.

None recorded.

#### Preliminary conservation assessment.

EOO (288,689.164 km^2^); AOO (380 km^2^). *Capsicumrecurvatum* occupies a large range along the Serra do Mar and is very frequent in many conservation units. Based on these criteria and the number of locations, we consider this species in the Least Concern (LC) category.

#### Discussion.

*Capsicumrecurvatum* belongs to the Atlantic Forest clade (Carrizo García et al. 2016). Its most remarkable character is the calyx which is extremely variable in the number, length, shape and orientation of the appendages; the main appendages are five and each flower also bears 1–5 secondary appendages of different lengths amongst them; appendages are cylindrical or most commonly triangular and laterally compressed, spreading or curved backwards in flowering calyx and strongly recurved in fruiting calyx. The corolla is white with a variable patterning of the greenish-yellow pigmentation within, having a large more or less extended greenish-yellow spot in each lobe (e.g. *Hunziker 20230*) or two smaller spots separated by a white line (main vein) (e.g. *Barboza 5004*) or many small spots (e.g. *Hunziker 25183*) in the base of the lobes and in the throat; completely white corollas have been occasionally observed (e.g. *Hunziker 25186*).

Smith and Downs (1966) identified plants of *C.recurvatum* from Santa Catarina, Brazil as *C.mirabile*. It can be distinguished from that species in the number and orientation of the appendages (5–10 of varying lengths in *C.recurvatum* vs. five subequal appendages in *C.mirabile*), corolla colour (white in *C.recurvatum* vs. purple with white margins in *C.mirabile*) and its distribution (*C.mirabile* does not reach Santa Catarina). In herbaria, it is quite common to find specimens of *C.recurvatum* identified as *C.cornutum* from which it differs in its white corollas with greenish-yellow spots (vs. white corollas with purple spots in *C.cornutum*), the recurved (vs. erect or spreading) calyx appendages and the antrorse (vs. spreading and denser) pubescence.

In the protologue of *C.recurvatum*, Witasek (1910) cited no specific herbarium for the type specimen; the collections she used are at WU and we selected a specimen with the same data as the protologue as the lectotype.

### ﻿39. Capsicum regale

Taxon classificationPlantaeSolanalesSolanaceae

Barboza & Bohs, PhytoKeys 167: 16. 2020.

424895FD-B6C4-53A5-A42C-3A489EB715C1

Figs 110 , 111

#### Type.

Colombia. Caquetá: Mun. Florencia, Corregimiento El Caraño, Finca de don Isauro, camino al río, en interior de bosque fuertemente inclinado, 01°44'10.6"N, 75°40'78.3"W, 1004 m elev., 22 Aug 2019, *A. Orejuela, L. Bohs, G.E. Barboza, P. González, R. Deanna, J. Urdampilleta, J. Valencia & G. Sierra 3034* (holotype: COL; isotypes: COAH, CORD, HUAZ [to be distributed]).

#### Description.

Erect slender shrubs (1–) 1.8–2.5 (–3) m tall, with the main stem somewhat thick, ca. 0.8 cm in diameter at base, sparsely branched towards apex, the branches dichotomous, weak, spreading horizontally. Young stems angled, fragile, glossy, pale green; nodes green; bark of older stems dark brown, glabrous; lenticels present. Sympodial units difoliate, the leaves geminate; leaf pair unequal in size and shape. Leaves membranous, slightly discolorous, green adaxially, pale green with the mid-vein prominent and purple and the secondary veins lilac or green abaxially, glabrous on both surfaces; blades of major leaves 17–20 (–24) cm long, 4.7–8 (–9.2) cm wide, elliptic, the major veins 6–8 on each side of mid-vein, the base unequal and attenuate, the margins entire, the apex acuminate to long-acuminate; petioles (0.8–) 1.5–2.3 cm long, green adaxially and purple abaxially, glabrous; blades of minor leaves 2–5 cm long, 1–3 cm wide, ovate, the major veins 3–5 on each side of mid-vein, the base unequal, the margins entire, the apex obtuse; petioles 0–0.4 cm long, green, glabrous. Inflorescences axillary, ca. 10 mm long, unbranched or rarely forked, with 5–13 flowers; rachis elongate, 4.5–6 mm long; peduncle 0–5.5 mm; flowering pedicels 12–14 mm long, thin, angled, erect to spreading, non-geniculate at anthesis, purple to green, glabrous; pedicels scars conspicuous, corky. Buds ellipsoid, green. Flowers 5-merous. Calyx 2–3 mm long, ca. 2 mm wide, cup-shaped, circular in outline, fleshy, green or greenish-purple, glabrous, the calyx appendages absent or 4–5, 1–1.8 mm long, 0.8–1.1 mm wide, subequal, thick, purple, triangular-compressed, wings-like, reflexed, inserted very close to the margin. Corolla 7–8 mm long, ca. 10 mm in diameter, thick, pure yellow or yellow with maroon pigmentation outside and greenish-yellow with lobes marginally maroon inside, deeply stellate with narrow interpetalar tissue, lobed 2/3 of the way to the base, glabrous adaxially and abaxially, the tube 2–2.5 mm long, the lobes 5–5.5 mm long, ca. 2 mm wide, triangular, the margins with short eglandular trichomes, the tips papillate. Stamens five, subequal; one filament longer than the others, long filament 3.5–4.3 mm long, shorter filaments (2–) 3–3.2 mm long, white, inserted on the corolla ca. 1 mm from the base, with auricles fused to the corolla at the point of insertion; anthers ca. 2 mm long, ellipsoid, lilac or pale bluish, not connivent at anthesis. Gynoecium with ovary ca. 1.3 mm long, ca. 1 mm in diameter, light green, ovoid; ovules more than two per locule; nectary ca. 0.4 mm tall; styles homomorphic, 4.3–4.5 mm long, barely exserted beyond the anthers, white, clavate; stigma ca. 0.1 mm long, ca. 0.8 mm wide, globose or somewhat discoid, light green. Berry 6–9 mm in diameter, globose, green when immature, becoming nearly white and translucent and then dark blue to purple at maturity, non-pungent, the pericarp thick, opaque, without giant cells (endocarp smooth); stone cells absent; fruiting pedicels ca. 18 mm long, 1.8–2 mm in diameter proximally, 2.5–2.6 mm in diameter distally, erect, fleshy, slightly angled and strongly thickened distally, brilliant dark purple; fruiting calyx 3.75–4.25 mm in diameter, persistent, not accrescent, discoid, brilliant purple, with a conspicuous annular constriction at the junction with the swollen pedicel, the appendages reflexed, brilliant purple, fleshy and laterally compressed. Seeds 7–17 per fruit, 2.75–3.40 mm long, 2.25–2.70 mm wide, C-shaped, black, the seed coat smooth, tuberculate at margins (SM), cerebelloid with pillar-like outgrowths (SEM), the cells irregular in shape to polygonal at seed margins, the lateral walls sinuate to straight; embryo annular.

#### Distribution.

*Capsicumregale* is endemic to southern Colombia (Caquetá Department), eastern Ecuador (Morona-Santiago, Napo and Sucumbío Provinces) and northern Peru (Loreto Department), known mainly on the eastern slopes of the Andes (the Andean-Amazonian Piedmont) (Fig. 109).

#### Ecology.

*Capsicumregale* occurs in small populations in the understorey of the premontane or montane humid tropical forests of the Amazonian slopes of the Andes, between 700 and 1,900 m elevation.

#### Phenology.

Flowering and fruiting in April and from August to December.

#### Chromosome number.

2*n* = 2x = 26 (Barboza et al. 2020b).

#### Common names.

None recorded.

#### Uses.

None recorded.

#### Preliminary conservation assessment.

EOO (47,806.378 km^2^); AOO (32 km^2^). Based on the EOO and AOO, *C.regale* is considered Endangered (EN B2a,b iii). Although this species has been collected in some protected areas, the habitat quality outside of these reserves is susceptible to human disturbance, such as crop planting and high levels of deforestation (Barboza et al. 2020b).

#### Discussion.

**Figure 110.**
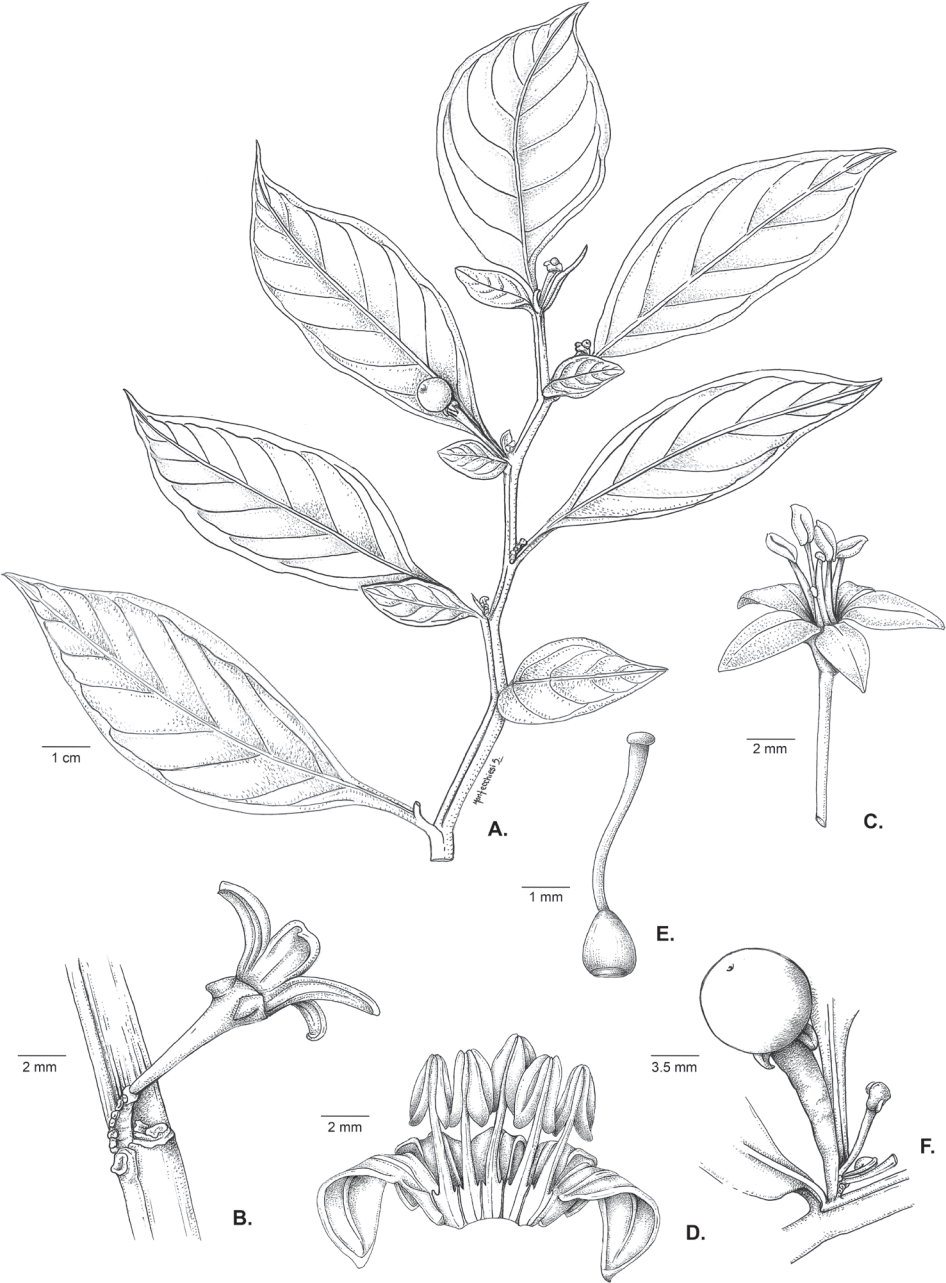
*Capsicumregale***A** fruiting apical branch **B** unbranched inflorescence **C** flower **D** opened corolla **E** gynoecium **F** fruit. From *Orejuela R. et al. 3034*. Drawn by S. Montecchiesi. Published in Barboza et al. (2020b), reproduced with permission.

**Figure 111.**
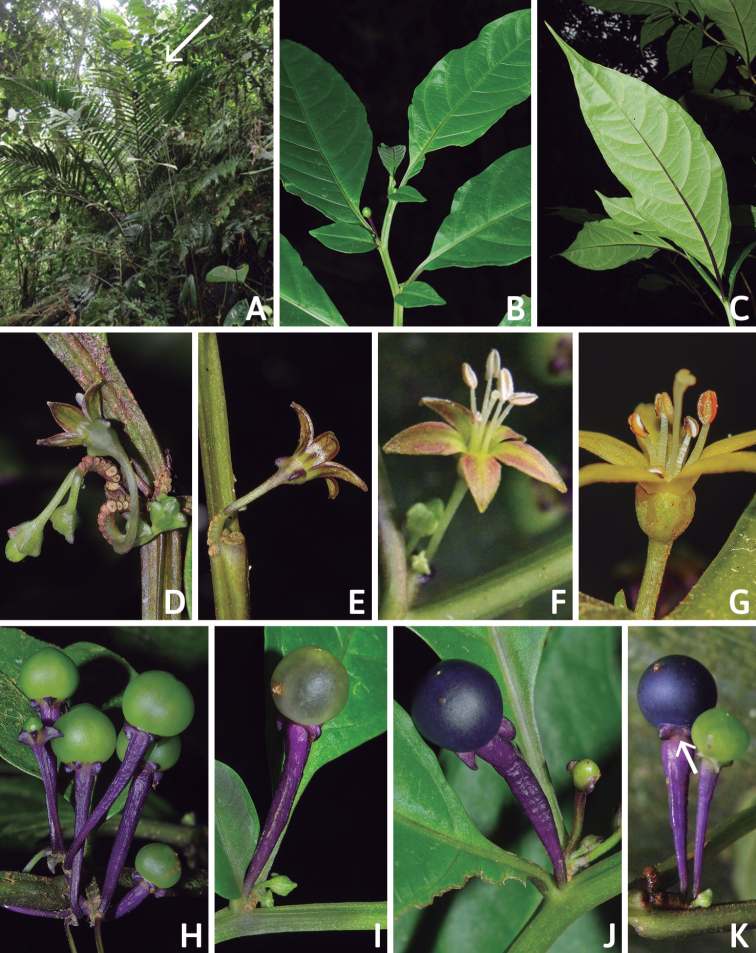
*Capsicumregale*. **A** habitat **B** apical branch, showing leaf pairs dissimilar in shape and size **C** abaxial surface of leaf with purple main vein **D** forked inflorescence; note the scars of the deciduous flowers **E** flower, in lateral view, on an unbranched elongate inflorescence **F, G** flowers with and without pigmentation, respectively **H–K** various stages of fruit maturity **K** mature fruit showing the constriction between the pedicel and the berry (arrow) **A–F, H–K** from *Orejuela R. et al. 3034*, photos by A. Orejuela, P. Gonzáles and G.E Barboza **G** from *Hoyos et al. 127*, photo by L. Coca. Published in Barboza et al. (2020b), reproduced with permission.

*Capsicumregale* belongs to the Andean clade (Barboza et al. 2020b). It is the most striking species of the genus with a set of unique traits, such as its unbranched (Figs 110B, 111E, J) or forked inflorescence (Fig. 111D) with 5–13 deciduous flowers on an elongate rachis, fleshy and laterally compressed calyx appendages (Fig. 111D, E), strongly thickened and brilliant purple fruiting pedicels (Fig. 111H–K), dark blue to purple fruits (Fig. 111J, K) and black seeds. This species is morphologically most similar to *C.longifolium* (Barboza et al. 2019) with which it shares lack of pubescence, multi-flowered inflorescences, yellow corollas, laterally compressed calyx appendages and black seeds. It differs from *C.longifolium* in its occasionally forked inflorescence (vs. unbranched in *C.longifolium*), elliptic (vs. narrowly elliptic) leaves, dark purple pedicels in fruit (vs. green) and purple (vs. orange) berries. *Capsicumregale* can be distinguished from *C.dimorphum*, which also has pure yellow corollas or with maroon pigmentation, calyx lacking appendages or with short appendages and black seeds, in its glabrescence, its larger major leaves and ovate minor leaves, its unbranched or forked 5–13-flowered inflorescence and its blue or purple fruits. *Capsicumdimorphum* is usually a pubescent plant, with smaller major leaves and orbicular or ovate (rarely elliptic) minor leaves, unbranched inflorescence of 1–5 flowers and orange to red fruits.

#### Specimens examined.

See Suppl. material 4: Appendix 4.

### ﻿40. Capsicum rhomboideum

Taxon classificationPlantaeSolanalesSolanaceae

(Dunal) Kuntze, Revis. Gen. Pl. 2: 450. 1891.

D2F5198A-71C3-5B18-96C7-67E6E95D17A9

Figs 112 , 113

Witheringia rhomboidea Dunal, Solan. Syn. 1. 1816. Type. Colombia. Quindio: “Mont Quindiu”, F.W.H.A. von Humboldt & A. Bonpland s.n. (holotype: P-Bonpl. [P00670657]).
Witheringia dumetorum Dunal, Solan. Syn. 1. 1816. Type. Ecuador. Pasto: Pasto, F.W.H.A. von Humboldt & A. Bonpland s.n. (holotype: P-Bonpl. [P00670658]).
Witheringia ciliata Kunth, Nov. Gen. Sp. [H.B.K.] (quarto ed.) 3: 11. 1818. Type. Ecuador. Pasto: “Crescit in Andibus frigidis Pastoensium prope Tulcan, alt. 1580 hex.” [474 m], F.W.H.A. von Humboldt & A. Bonpland s.n. (holotype: P [not found]; isotype: B destroyed, photo F neg. 2875]).
Witheringia mollis Kunth, Nov. Gen. Sp. [H.B.K.] (quarto ed.) 3: 12. 1818. Type. Peru. Cajamarca: “Crescit in Regno Peruviano prope urbem Caxamarca, alt. 1500 hex” [450 m], F.W.H.A. von Humboldt & A. Bonpland s.n. (lectotype, designated here: P-Bonpl. [P00670656]; isotype: B [destroyed, photo F neg. 2877]).
Capsicum aggregatum Willd. ex Roem. & Schult., Syst. Veg., ed. 15 bis [Roemer & Schultes] 4: 809. 1819. Type. No locality information given “Habitat …”, “Capsicumaggregatum, herb. Willd.)” (holotype: B [B-W04434-01-0]).
Witheringia diversifolia Klotzsch ex Walp., Repert. Bot. Syst. (Walpers) 3: 29. 1844. Type. Cultivated in Berlin Botanical Garden, Germany “In Mexico- (Floruit in horto Berolinensi botanico mense Aprili anni 1841)” (no specimens cited; no original material found).
Brachistus ciliatus (Kunth) Miers, Ann. Mag. Nat. Hist. ser. 2, 3(16): 263. 1849. Type. Based on Witheringiaciliata Kunth.
Brachistus rhomboideus (Dunal) Miers, Ann. Mag. Nat. Hist. ser. 2, 3(16): 264. 1849. Type. Based on Witheringiarhomboidea Dunal.
Brachistus mollis (Kunth) Miers, Ann. Mag. Nat. Hist. ser. 2, 3(16): 264. 1849. Type. Based on Witheringiamollis Kunth.
Brachistus dumetorum (Dunal) Miers, Ann. Mag. Nat. Hist. ser. 2, 3(16): 265. 1849. Type. Based on Witheringiadumetorum Dunal.
Brachistus diversifolius (Klotzsch ex Walp.) Miers, Ann. Mag. Nat. Hist. ser. 2, 3(16): 268. 1849. Type. Based on Witheringiadiversifolia Klotzsch ex Walp.
Fregirardia luteiflora Dunal ex Delile, Ind. Sem. Hort. Monsp.: 7. 1849. Type. Cultivated in Montpellier, France. “Hort. Montpellier”, 17 Feb 1849, M.F. Dunal s.n. (lectotype, designated by Hunziker 1971, p. 253: MPU, second step designated here: MPU [MPU023032]); isolectotypes: HBG [HBG-511320, G-DC [G00200559], MPU [MPU023031]).
Fregirardia rhomboidea (Dunal) Dunal, Prodr. [A. P. de Candolle] 13(1): 504. 1852. Type. Based on Witheringiarhomboidea Dunal.
Fregirardia dumetorum (Dunal) Dunal, Prodr. [A. P. de Candolle] 13(1): 504. 1852. Type. Based on Witheringiadumetorum Dunal.
Fregirardia mollis (Kunth) Dunal, Prodr. [A. P. de Candolle] 13(1): 505. 1852. Type. Based on Witheringiamollis Kunth.
Fregirardia vargasii Dunal, Prodr. [A. P. de Candolle] 13(1): 505. 1852. Type. Venezuela. Distrito Federal: Circa Caracas, 1830, *J.M. Vargas 249* (holotype: G-DC [G00200549; isotype: MPU [MPU023030]).
Solanum mendax Van Heurck & Müll.Arg., Observ. Bot. (Van Heurck): 61. 1870. Type. Ecuador. Tungurahua: Baños, Aug 1857, *R. Spruce 5050* (lectotype, designated by Barboza 2011, pg. 29: K [K000201915]; isolectotypes: BM [BM000777290], E [E00202462], G (G0342803, G00342804], GH [GH00077720], K [K000201792, K000201915], MO [MO-1287475, acc. # 1691274, MO-1287476, acc. # 1781034], P [P00410209], W [W-Rchb. 1889-0294613, W-Rchb. 1889-0223014]).
Brachistus pringlei S.Watson, Proc. Amer. Acad. Arts 25: 159. 1890. Type. Mexico. Nuevo León: in the Sierra de la Silla near Monterrey, 28 May 1889, *C. G. Pringle 2544* (holotype: GH [00936714]; isotypes: AC [AC00320467], BKL [BKL00004329], BR [BR0000005530854, BR0000005531189], CM [CM1923, acc. # 260433], COLO [00352195], E [E00570143], EAP [EAP71917], F [v0072759F], G [G00342807], GOET [GOET003410], K [K000585889], KFTA [KFTA0002452, KFTA0002453], M [M-0171536], MEL [MEL2442144], MEXU [00028827], MO [MO-022273, acc. # 3727961; MO-153287, acc. # 1768532], NA [NA-0026220], NDG [NDG44985], NY [00138549, 00138550], P [P00410033, P00410034], PH [PH00008049], PUL [PUL00000045], RSA [RSA0006263, acc. # 102428], S [acc. # S-G-1004], UC [UC548235], US [00027409, acc. # 48601], VT [UVMVT026402], W [acc. # 1890-0000820], WU [acc. # 0033958]).
Capsicum ciliatum (Kunth) Kuntze, Revis. Gen. Pl. 2: 450. 1891. Type. Based on Witheringiaciliata Kunth.
Capsicum diversifolium (Klotzsch ex Walp.) Kuntze, Revis. Gen. Pl. 2: 450. 1891. Type. Based on Witheringiadiversifolia Klotzsch ex Walp.
Capsicum dumetorum (Dunal) Kuntze, Revis. Gen. Pl. 2: 450. 1891. Type. Based on Witheringiadumetorum Dunal.
Capsicum molle (Kunth) Kuntze, Revis. Gen. Pl. 2: 450. 1891. Type. Based on Witheringiamollis Kunth.
Capsicum vargasii (Dunal) Kuntze, Revis. Gen. Pl. 2: 450.1891. Type. Based on Fregirardiavargasii Dunal.
Capsicum mendax (Van Heurck & Müll.Arg.) J.F.Macbr., Candollea 5: 402. 1934. Type. Based on Solanummendax Van Heurck & Müll. Arg.
Brachistus haughtii Svenson, Amer. J. Bot. 33: 481, t. 19, f. 2. 1946. Type. Peru. Piura: summit of Cerro Prieto, 4°45'S, 81°15'W, 2500 ft elev., 28/30 Mar 1941, *O. Haught & H.K. Svenson 11621* (holotype: BKL [BKL00004328]).
Capsicum pringlei (S.Watson) J.F.Macbr. & Standl., Publ. Field Mus. Nat. Hist., Bot. Ser. 11: 173. 1936. Type. Based on Brachistuspringlei S.Watson.
Brachistus vargasii (Dunal) Pittier, Cat. Fl. Venez. [Pittier] 2: 358. 1947. Type. Based on Fregirardiavargasii Dunal.
Capsicum haughtii (Svenson) J.F.Macbr., Publ. Field Mus. Nat. Hist., Bot. Ser. 13 (V-B, 1): 72. 1962. Type. Based on Brachistushaughtii Svenson.

#### Type.

Based on *Witheringiarhomboidea* Dunal.

#### Description.

Erect, slender and sprawling shrubs or rarely trees (0.5–) 2–3 (–5) m tall, with the main stem 1.8–2.5 cm in diameter at base, profusely branched above, the branches erect or decumbent. Young stems terete or slightly angled, somewhat rigid, glabrous or sparsely to densely pubescent with white or rarely ochraceous, antrorse or spreading, simple, uniseriate, eglandular trichomes 0.3–0.9 mm long or furcate to 3–5-branched (or more) trichomes 0.2–0.8 (–1.5) mm long; nodes green; bark of older stems longitudinally ridged, grey, dark brown or brownish-purple; lenticels abundant, white or light brown. Sympodial units difoliate, the leaves geminate; leaf pair unequal in size and similar or dissimilar in shape. Leaves membranous, discolorous, dark green, slightly glossy above, dull light green, with the primary vein raised beneath, moderately pubescent to glabrous adaxially, moderately to densely pubescent abaxially, with similar antrorse trichomes like those of the stems, sometimes a tuft of trichomes in the vein axils beneath; blades of major leaves (4–) 4.8–12 cm long, 2–5 cm wide, ovate, elliptic or rhomboid-ovate, the major veins 4–6 on each side of mid-vein, the base attenuate or truncate, sometimes asymmetric, the margins entire, the apex acute or obtuse-acuminate; petioles 0.5–1.5 (–1.8) cm, with moderate to dense pubescence of spreading trichomes; blades of minor leaves 2–3 cm long, 1.5–2.5 cm wide, ovate or rhomboid-ovate, the major veins 3–4 on each side of mid-vein, the base short-attenuate, the margins entire, the apex acute or obtuse; petioles 0.3–0.8 cm with similar pubescence as major leaves. Inflorescences axillary, 3–8 (–13)-flowers per axil, rarely solitary; flowering pedicels (8–) 11–28 mm, terete or slightly striate, pendent, non-geniculate at anthesis, green, glabrescent to densely pubescent, the eglandular trichomes short or long, spreading or antrorse; pedicels scars conspicuous, corky. Buds globose, yellow or green. Flowers 5-merous. Calyx 2.7–3.8 mm long, 5–6 mm wide, cup-shaped, green, glabrescent to densely pubescent with antrorse or spreading simple, furcate or dendritic trichomes 0.3–0.8 mm long, the calyx appendages usually five (rare 3–4), 0.9–3 mm long, subequal, erect or spreading, linear-subulate, 0.0–0.3 mm below the margin, with the same pubescence as the calyx tube. Corolla (5–) 6–10 mm long, (8–) 10–12 mm in diameter, yellow, sometimes tinged greenish outside and within, campanulate or campanulate-rotate with a wide interpetalar membrane connecting the lobes up to the distal end, hardly lobed, glabrous adaxially and abaxially, the tube (4.4–) 5.4–9 mm long, the lobes 0.6–1 mm long, 1.6–1.9 mm wide, broadly ovate, the margins finely ciliate, the tips cucullate, papillate. Stamens five, equal; filaments 1.2–2.3 mm long, pale yellow or light green, inserted on the corolla 1–1.3 mm from the base, with auricles fused to the corolla at the point of insertion; anthers (1.4–) 1.6–2.3 mm long, ellipsoid, pale yellow or yellow, not connivent at anthesis. Gynoecium with ovary 1.3–1.6 mm long, 1.2–1.3 mm in diameter, greenish-white, ovoid; ovules more than two per locule; nectary 0.5–0.6 mm tall; styles homomorphic, 3.9–5.8 mm long, barely exserted beyond the anthers, pale yellow, clavate; stigma 0.25–0.5 mm long, 0.4–0.6 mm wide, usually bilobed, light green. Berry 5–9 mm in diameter, globose or globose-depressed, green when immature, bright red turning dark burgundy at maturity, non-pungent, the pericarp thick, opaque, lacking giant cells (endocarp smooth); stone cells absent; fruiting pedicels 15–35 mm long, pendent, terete or angled, widened distally, green; fruiting calyx 4–5 mm in diameter, persistent, not accrescent, discoid, green, the appendages 3–6 mm long, spreading or reflexed. Seeds 24–60 per fruit, 2.4–2.8 mm long, 1.8–2.2 mm wide, C-shaped, reniform, rarely teardrop-shaped, dark brown, rarely light brown, the seed coat reticulate (SM), reticulate-cerebelloid (SEM), the cells irregular in shape, the lateral walls mostly sinuate, wavy at margins; embryo annular or imbricate.

**Figure 112.**
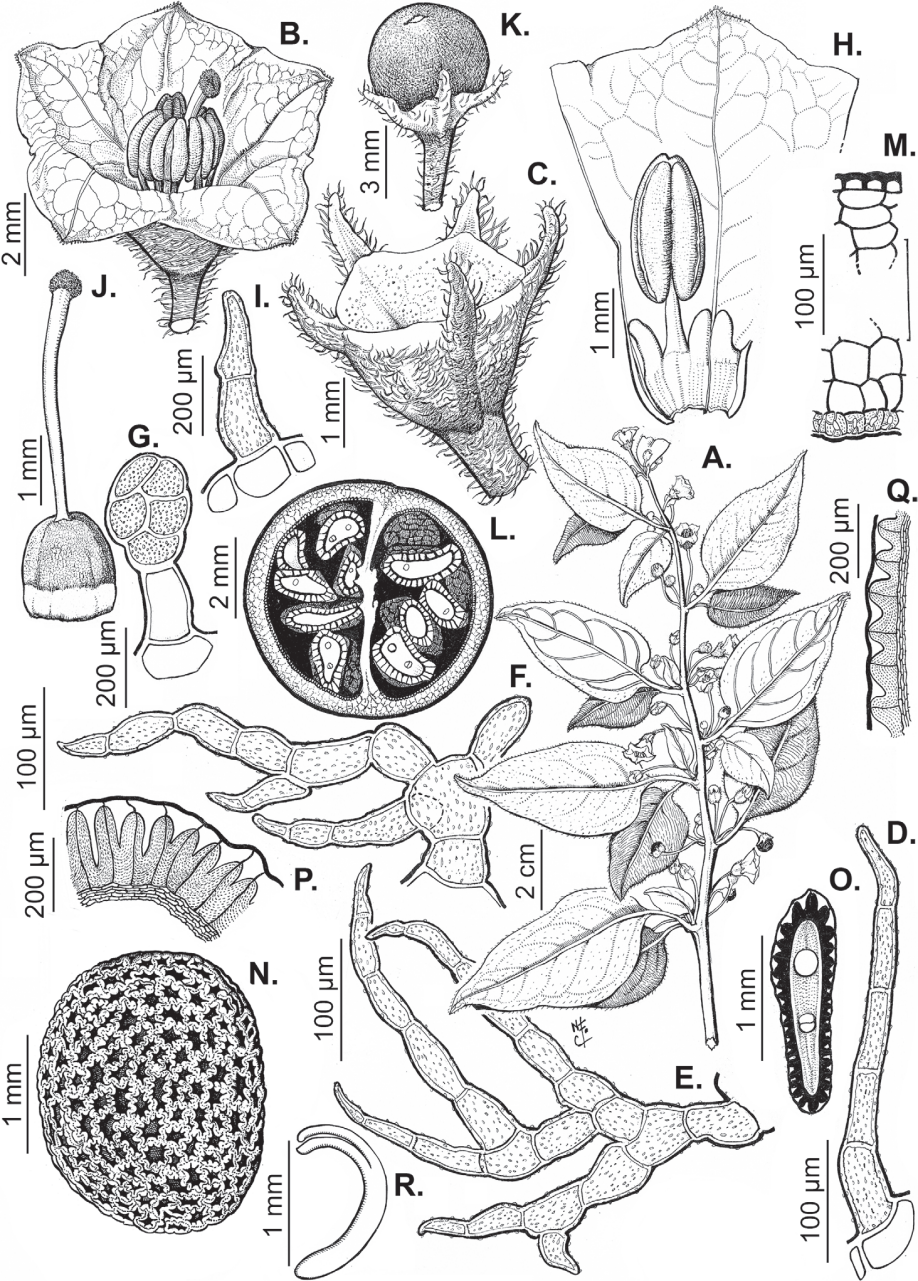
*Capsicumrhomboideum***A** reproductive branch **B** flower **C** calyx **D** eglandular trichome of the calyx **E, F** branched trichomes of the calyx **G** glandular trichome of the abaxial surface of the calyx **H** sector of opened corolla **I** eglandular trichome of the corolla lobes **J** gynoecium **K** fruit **L** fruit, in cross section **M** anatomical detail of the pericarp (note the absence of giant cells in the mesocarp) **N** seed **O** seed, in cross section **P** structure of seed coat at the seed margin **Q** structure of seed coat at the seed body **R** embryo **A** from *Parra V. 089***B–D, G–J** from *Moore 3831***E, F** from *Haught & Svenson 11621***K–R** from *Acosta Solís 14234*. Drawn by N. de Flury. Published in Hunziker (2001), reproduced with permission.

**Figure 113.**
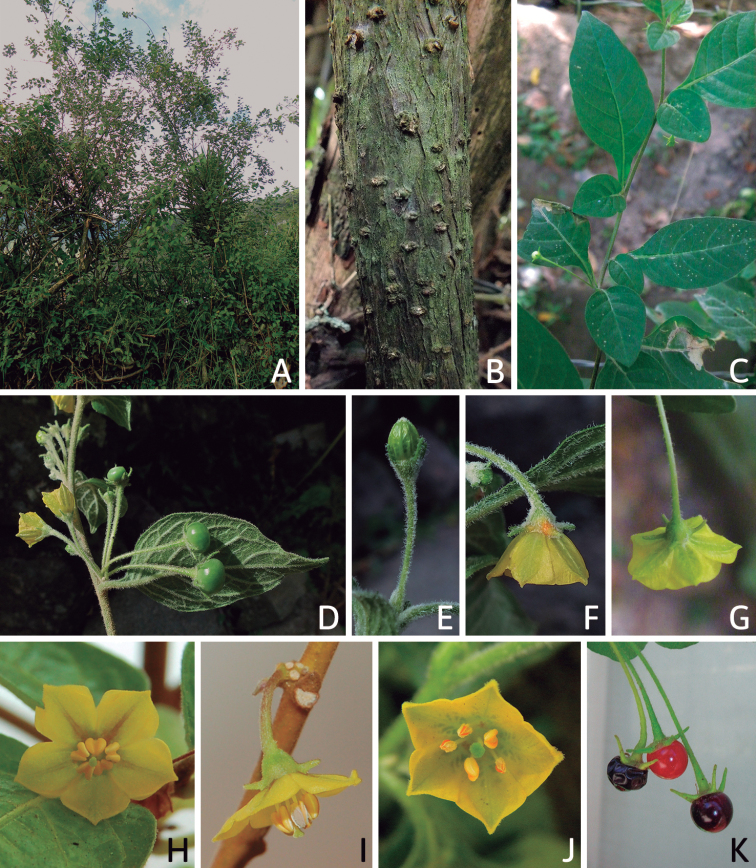
*Capsicumrhomboideum***A** plant **B** main stem with lenticels flower buds **C** branch showing leaf pair dissimilar in shape and size **D** reproductive branch **E** flower bud **F** flower, in lateral view **G** flower, seen from behind **H, J** flower, in front view, showing connivent and not connivent anthers, respectively **I** node with a pendent flower and pedicels scars **K** mature fruits **A, J** from *Barboza & Leiva González 4854***B, D–F** from *Leiva González 6585***C, G** from *Barboza et al. 5050***H, I, K** from *M. Scaldaferro 73*. Photos by G.E. Barboza.

#### Distribution.

**Figure 114.**
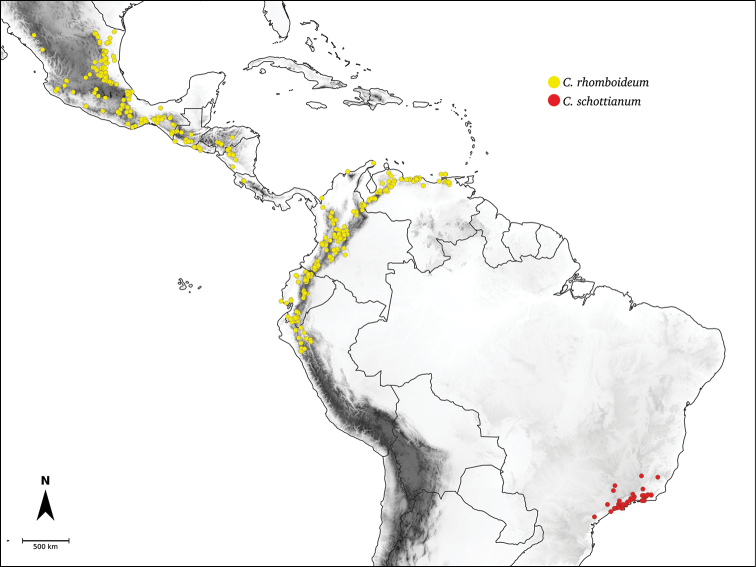
Distribution of *C.rhomboideum* and *C.schottianum*.

*Capsicumrhomboideum* is widely distributed from Mexico and Central America to northern Peru (Fig. 114).

#### Ecology.

*Capsicumrhomboideum* is ecologically adapted to different vegetation formations, but grows mostly in xerophytic thickets and dry forests or in tropical deciduous forests, at 250–2,500 (–3,000) m elevation.

#### Phenology.

Flowering and fruiting all year.

#### Chromosome number.

2*n* = 2x = 26 (Pickersgill 1977, as *C.ciliatum*; Moscone et al. 2007; Scaldaferro et al. 2013).

#### Common names.

**Colombia**: Tinto (Norte de Santander, *Garganta 857*); **Ecuador**: Arrayán (Chimborazo, *Cerón 15714*), Motupe (Imbabura, *Vivar C. s.n.*), Caspi morocho (Chimborazo, *Scolnik 1594*), Hierva dura (Carchi, *Cerón 11092*), Hierba mora (Pichincha, *Cerón 6953*), 7 varas (Pichincha, *Cerón 13171*); **El Salvador**: Mora silvestre (San Miguel, *Villacorta et al. 2819*), Veranera silvestre (Ahuachapán, *Sandoval 308*), Arito de niña (Ahuachapán, *Sandoval 550*); **Venezuela**: Cachimbito (Distrito Federal, *Buschel s.n.*),

#### Indigenous name.

**Mexico.** Tumaltez (Tzeltal, Chiapas, *Alonso Méndez 7709*).

#### Uses.

**Ecuador**. Long stems are used to make drum hoops (*Cerón 13171*). Used in medicine (see Table 3).

#### Preliminary conservation assessment.

EOO (7,747,997.619 km^2^); AOO (1,964 km^2^). *Capsicumrhomboideum* is widespread from Mexico to Peru; we assign the Least Concern (LC) status.

#### Discussion.

*Capsicumrhomboideum* belongs to the Andean clade (Carrizo García et al. 2016). This species is one of the most polymorphic and widespread and has the largest synonymy of all the wild species of *Capsicum*. *Capsicumrhomboideum* is distinctive by its habit (shrubs or trees up to 5 m tall), the dark brown or brownish-purple bark with abundant lenticels, inflorescences up to 13-flowered, campanulate or campanulate-rotate yellow corollas, relatively small (5–9 mm in diameter) bright red turning dark burgundy non-pungent fruits, small dark brown seeds (2.4–2.8 mm long) and a mixture of simple and branched pubescence (Fig. 113). While the reproductive characters are usually consistent along its wide distribution, the pubescence and leaf shape are the most variable features in *C.rhomboideum*, this variation being responsible for the many different names given to this species. Some populations are densely pubescent with only simple eglandular trichomes or with both simple and branched trichomes, while others vary from moderately pubescent to glabrescent (a few, completely glabrous). Simple eglandular trichomes range from 3–8 (–11) cells, whereas branched trichomes can be furcate or variously 3–5 (or more)-branched (many branches in different directions). Highly branched trichomes are uncommon in the genus, this character also occurring in a few Brazilian species (e.g. *C.longidentatum*, *C.parviflorum*; Barboza et al. 2011). Leaf shape ranges from ovate, elliptic to rhomboid-ovate and, in some cases, the dissimilarity in size of the leaf pair is not obvious.

Amongst the various names coined by Kunth (1818) under *Witheringia* (two of them here recognised as synonyms of *C.rhomboideum*) is *W.ciliata*, on which *Capsicumciliatum* was based (Kuntze 1891). Although the name *C.ciliatum* has been widely used in literature (Hunziker 1956, 1971; D’Arcy and Eshbaugh 1974; Molina 1975; Nee 1986; Mitchell et al. 1989), *C.rhomboideum* has priority and is the correct name for this entity (Hunziker 2001).

We found in the P-Bonpland Herbarium a sheet (P00670656) that seems to be original material of *Witheringiamollis* and this is selected as the lectotype.

#### Specimens examined.

See Suppl. material 4: Appendix 4.

### ﻿41. Capsicum schottianum

Taxon classificationPlantaeSolanalesSolanaceae

Sendtn., Fl. Bras. (Martius) 10(6): 143. 1846.

E7D8C769-5466-5FA5-A214-DEE4B6922A30

Figs 115 , 116

#### Type.

[Brazil. Rio de Janeiro]: Serra d’Estrella, [no date], *H.W. Schott [5425*] (lectotype, designated here: W [acc. # 0074666]; isolectotypes: CORD [CORD00006649], F [v0072906F, acc. # 874709], W [acc. # 0074665, acc. # 0074667]).

#### Description.

Erect shrubs 1–2.5 m tall or small trees 3–5 m tall, with the main stem (1.5–) 2–3 (–6) cm in diameter at base, much branched above, the branches dichotomously spreading in a typical “zig-zag” appearance. Young stems 3–4-angled, rigid, green or light purple, moderately pubescent, with antrorse, curved, simple, uniseriate, 2–6-celled, eglandular trichomes 0.25–0.5 mm long; nodes solid, swollen, dark green almost black or lilac; bark of older stems dark brown, glabrous; lenticels absent. Sympodial units difoliate, the leaves geminate; leaf pair unequal in size, similar in shape. Leaves membranous, slightly discolorous to discolorous, opaque, green above, light green beneath, glabrous or sparsely pubescent, with similar trichomes as in stems on both surfaces; blades of major leaves (4–) 9.5–16.5 (–25.5) cm long, 2–4 (–6) cm wide, elliptic to ovate, the major veins 6–8 on each side of mid-vein, the base attenuate or cuneate, unequal, the margins entire, the apex acuminate; petioles 0.8–2.5 cm long, glabrescent to moderately pubescent; blades of minor leaves 3.4–4.2 cm long, 1.5–1.7 cm wide, elliptic or ovate, the major veins 3–4 on each side of mid-vein, the base attenuate, the margins entire, the apex acute or obtuse; petioles 0.1–0.4 cm long, with similar pubescence as the major leaves. Inflorescences axillary, 2–5 (–7) flowers per axil, very rarely flowers solitary; flowering pedicels 8–25 mm long, thin, angled, erect or slightly spreading, geniculate at anthesis, light green, glabrescent, the eglandular trichomes short, antrorse; pedicel scars conspicuous, corky. Buds globose-ovoid, green or greenish-yellow, rarely purple. Flowers 5-merous. Calyx (1.5–) 2–2.4 mm long, 2.25–2.5 mm wide, cup-shaped, green, circular or pentagonal in outline, sometimes the primary veins conspicuously marked on the surface, glabrescent, with sparse small glandular trichomes (stalk one-celled; head dark, multicellular) and 2–4-celled eglandular trichomes, the calyx appendages absent or with five minute appendages ca. 0.35 mm long. Corolla 7–8 (–10) mm long, 8–12 (–14) mm in diameter, mostly white with green or greenish-yellow and pale purple spots outside, white with discontinuous purple or brownish spots, greenish-yellow spots and white centre within (in some populations purple pigmentation absent); stellate without or with a thin interpetalar membrane, lobed more than 1/3 to halfway to the base, pubescent adaxially with a continuous ring of glandular trichomes (stalk long, 2-celled; head globose, peltate, unicellular) in the throat and base of the lobes, glabrous abaxially, the tube 2.4–4.2 mm long, the lobes 2–3.5 (–5) mm long, 2–4 mm wide, triangular or ovate, spreading, the margins finely ciliate or papillate, the tips acute and cucullate, papillate. Stamens five, equal; filaments (2.4–) 3–4 mm, white or cream, inserted on the corolla 1.5–2 mm from the base, with auricles fused to the corolla at the point of insertion; anthers (1.1–) 1.3–1.8 mm, ellipsoid, yellow, light green, grey or light purple, not connivent at anthesis. Gynoecium with ovary 1.25–1.7 mm long, 1.35–1.5 mm in diameter, light green, globose-ovoid; ovules more than two per locule; nectary ca. 0.5 mm tall; styles homomorphic, 3–4.5 mm long, barely exserted beyond the anthers, white, clavate, sometimes slightly curved; stigma 0.12–0.25 mm long, 0.75–0.85 mm wide, discoid or globose, pale green. Berry 7–9 mm in diameter, globose or subglobose, green when immature, greenish-golden yellow at maturity, deciduous, pungent when immature and less pungent when mature, the pericarp thin, translucent, with giant cells (endocarp alveolate); stone cells absent; fruiting pedicels 17–27 mm long, pendent, terete or angled, widened distally, green; fruiting calyx 3–4.5 mm in diameter, thin, persistent, not accrescent, discoid, light green, with a thin constriction at the junction with the calyx. Seeds (6–) 7–19 (–23) per fruit, 2.6–3.5 mm long, 2–3 mm wide, C-shaped, ellipsoid or teardrop-shaped, brownish-black to black, the seed coat reticulate and tuberculate at margins (SM), reticulate with pillar-like outgrowths (SEM), the cells irregular polygonal to irregular in shape, the lateral walls straight to sinuate; embryo imbricate.

#### Distribution.

*Capsicumschottianum* is endemic to south-eastern Brazil (Minas Gerais, Rio de Janeiro and São Paulo States) (Fig. 114).

#### Ecology.

*Capsicumschottianum* grows from the coast to the interior of the Atlantic Forest (Mata Atlântica). It is quite common, often forming large populations in the Floresta Ombrófila Densa and Floresta Estacional Semidecidual and can be found in margins and interior of primary and disturbed forests, near the watercourses, in sun or in semi-shade, between 250 and 1,700 m elevation.

#### Phenology.

Flowering from September to April; fruiting from December to June.

#### Chromosome number.

*n* = 13 (Pozzobon and Schifino-Wittmann 2006); 2*n* = 2x = 26 (Pozzobon et al. 2006; Moscone et al. 2007).

#### Common names.

**Brazil**. Pimenta bentiví (Minas Gerais, *Hunziker 25145*).

#### Uses.

None recorded.

#### Preliminary conservation assessment.

EOO (148,467.320 km^2^); AOO (216 km^2^). *Capsicumschottianum* is distributed mainly along the Serra do Mar system (Serra da Carioca, Serra da Bocaina, Serra do Paranapiacaba, Serra dos Órgãos and others) and is very frequent in many conservation units. Considering the large extent of occurrence, the high number of locations in Natural Reserves and the large population size with many highly reproductive individuals, we suggest a Least Concern (LC) category for this species.

**Figure 115.**
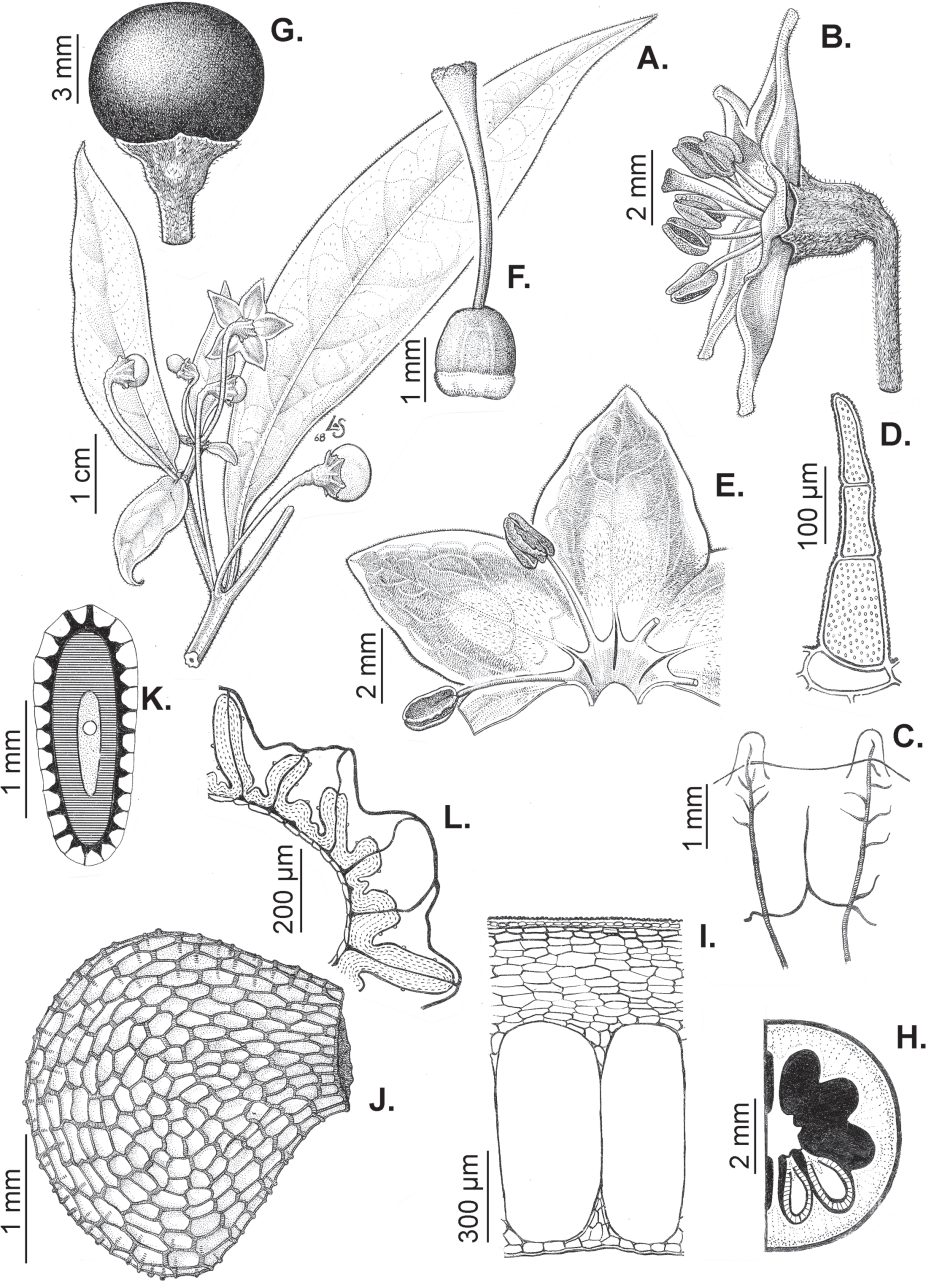
*Capsicumschottianum***A** reproductive branch **B** flower **C** section of the calyx showing the venation **D** eglandular trichome of the calyx **E** sector of opened corolla **F** gynoecium **G** fruit **H** fruit (one carpel), in cross section **I** anatomical detail of the pericarp (note the giant cells in the mesocarp) **J** seed **K** seed, cross section **L** structure of seed coat at the seed margin **A–L** from *Hunziker 19577***L** from *Macedo 3079*. Drawn by L. Sánchez.

#### Discussion.

**Figure 116.**
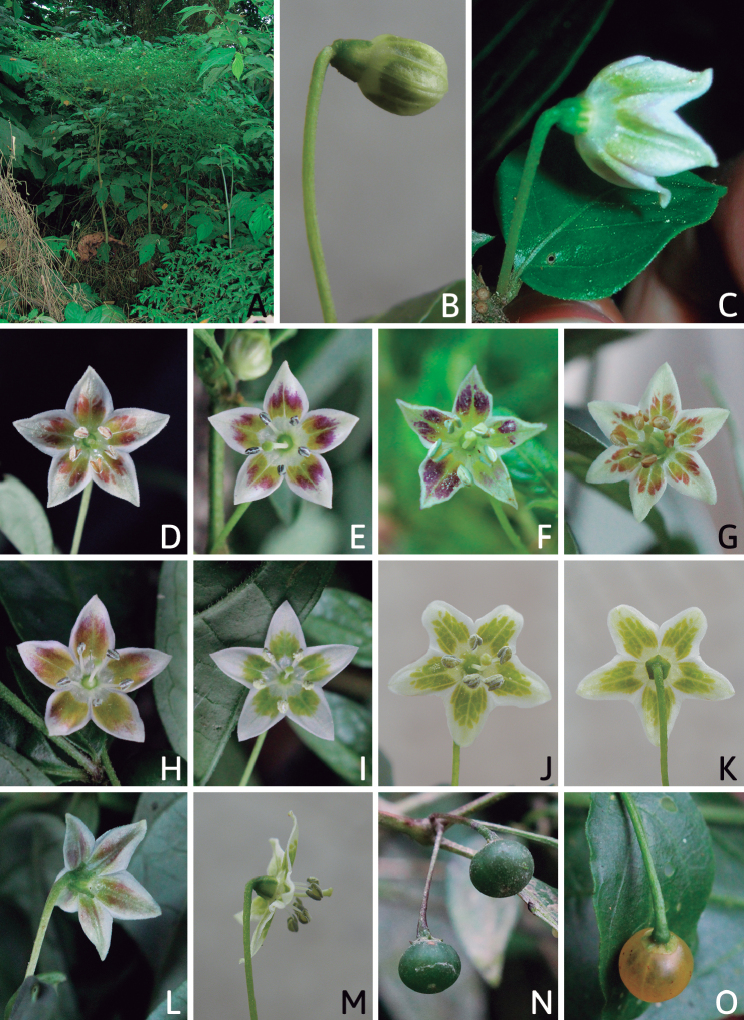
*Capsicumschottianum***A** plant **B** flower buds on geniculate pedicel **C** flower, in pre-anthesis **D–J** flowers, in front view, showing variations in the purple and greenish pigmentation of the corolla **K, L** flowers, seen from behind **M** flower in lateral view, showing not connivent anthers and style slightly exserted **N** immature fruits **O** mature fruit **A, C***Barboza et al. 1638***B, J, K, M** from *Barboza 5043***D, G, L** from *Barboza & Deanna 5019***E** from *Barboza & Deanna 5017***F** from *Barboza & Deanna 5029***H** from *Barboza & Deanna 5018***I, O** from *Barboza & Deanna 5014***N** from *Barboza & Deanna 5006*. Photos by G.E. Barboza and R. Deanna.

*Capsicumschottianum* is a member of the Atlantic Forest clade (Carrizo García et al. 2016). Its most conspicuous feature is the white corollas with a variable quantity of purple and greenish-yellow pigmentation within. The purple (or brownish) pigmentation develops as two large strong spots in the basal half of each lobe extending up to near the apex (Fig. 116D); in some cases, these spots are not equally developed on each lobe and are more or less diffuse (Fig. 116E–H) or even lacking (Fig. 116I–K). Variation in the anthocyanin pigmentation was observed in flowers of the same individual and within individuals of the same population (e.g. Ubatuba-São Paulo: *Barboza et al. 5014, 5015, 5017*). Corollas without any sign of purple colour were observed in some populations in Serra do Paranapiacaba (São Paulo). The other distinctive character for this species is the flowering calyx with very prominent main veins that lacks appendages or that has five minute appendages (not more than 0.35 mm long), giving a pentagonal outline to the calyx (Fig. 116C, K, L).

*Capsicumschottianum* is extremely difficult to distinguish from *C.campylopodium* in herbaria when the specimens have only immature fruits and there is no any indication of the corolla colour in the labels. The calyx of *C.schottianum* is generally pentagonal in outline and measures 1.5–2.4 mm long, corollas reach 7–10 mm long and 8–14 mm in diameter and have generally purple and greenish-yellow colouration within, the filaments are equal and the berry is globose to subglobose with 6–23 seeds. In contrast, the calyx of *C.campylopodium* is always circular in outline and measures (1–) 1.2–1.6 mm long, corollas reach 4.5–6.5 (–8) mm long, (6–) 6.4–7.5 (–11) mm in diameter and has golden yellow or ochraceous spots within, the filaments are unequal (3 + 2) and the berry is globose-depressed with only four (very rare six) seeds.

*Capsicumschottianum* can also be confused with *C.pereirae* which has a similar pattern of colouration in the corolla and calyx shape, but differs in the geniculation of the pedicels (geniculate in *C.schottianum* vs. non-geniculate in *C.pereirae*) and leaf morphology (membranous and opaque in *C.schottianum* vs. coriaceous and shiny in *C.pereirae*).

In describing *C.schottianum*, Sendtner (1846) cited two collections, one from “Serra d’Estrella” by Heinrich Schott and the other from “Brasilia australiore” by Friedrich Sellow. Sellow’s collection was assigned to Sendtner’s var. “β” to which he did not give a name; this was later described by Dunal (1852) as C.schottianumvar.leptophyllum, which is here considered a synonym of *C.flexuosum*. Three specimens held in the Herbarium in Vienna collected by Schott bearing the number “5425” (W, acc.# 0074666) and 5426 (W, acc. # 0074665, acc. # 0074667) and the name “Capsicumschottianum Sendt.” on the labels appear to all be original material. Only one of these has the original locality “Serra d’Estrella” on the label (W acc. # 0074666) and we select this here as the lectotype. The other sheets at W appear to be duplicates, as do sheets at F and CORD; thus, we treat these as isolectotypes.

### ﻿42. Capsicum tovarii

Taxon classificationPlantaeSolanalesSolanaceae

Eshbaugh, P.G.Sm. & Nickrent, Brittonia 35(1): 55. 1983.

F1178731-CBE4-5A54-81CD-DD65343096D8

Figs 117 , 118

#### Type.

Peru. Huancavelica: Prov. Tayacaja: Andaimarca, entre Colcabamba y Surcubamba, valle del Mantaro, 2000 m elev., 14 Apr 1954, *O. Tovar 1867* (holotype: USM [USM000895]; isotypes: MU (n.v.), US [00027402, acc. # 3048334], USM [USM248309]).

#### Description.

Erect subshrubs or shrubs, 0.5–1.5 (–2.5) m tall, with the main stem thick, 1.5–3.5 cm in diameter at base, much branched from the base, the branches fragile and scandent. Young stems 2–3-angled, green, rigid, glabrescent to moderately pubescent with appressed-antrorse or spreading, simple, uniseriate, 2–6-celled, eglandular trichomes 0.08–0.9 (–1.2) mm long and sparse small, simple, glandular trichomes (stalk short, unicellular; head dark, oblong, multicellular); nodes green, bark of older stems greenish-brown, fissured, glabrescent; lenticels absent. Sympodial units difoliate, the leaves geminate; leaf pair subequal in size and shape. Leaves membranous, discolorous, dark green above, greenish-grey below, moderately to densely pubescent, with simple eglandular trichomes and glandular trichomes similar to those of the stems, these latter especially abundant on the veins abaxially; blades of all leaves (2.5–) 4–15.5 cm long, (1–) 1.5–5.7 cm wide, ovate or lanceolate, the major veins 3–5 on each side of mid-vein, the base short-attenuate and asymmetric, the margins entire, the apex acuminate; petioles 1–3 cm long, densely pubescent. Inflorescences axillary, (2–) 4–8 flowers; flowering pedicels 3–10 mm long, short, angled or terete, spreading to pendent, non-geniculate at anthesis, green, moderately pubescent, the eglandular trichomes long, spreading; pedicel scars inconspicuous. Buds globose to ovoid, yellowish-green. Flowers 5-merous. Calyx 1.5–2 mm long, 1.8–2.5 mm wide, cup-shaped, pentagonal in outline, thin, light green with dark green veins, moderately pubescent with the same eglandular trichomes as stems and leaves, sometimes sparse glandular trichomes, the calyx appendages absent or five, equal, 0.5 mm long, inserted very close to the margin, sparsely pubescent. Corolla (4.5–) 6–8 mm long, 8–10 mm in diameter, yellowish-green with lilac interpetalar membrane outside, purple with greenish-yellow spots in the lobes and greenish-yellow centre within or lobes mostly yellowish-green and diffuse lilac pigmentation within, campanulate or campanulate-stellate, lobed less than 1/3 of the way to the base, the tube 3.8–4.62 mm long, with sparse small glandular trichomes (stalk unicellular; head globose, unicellular) adaxially, glabrous abaxially, the lobes 2.5–3.2 mm long, 2.5–3.5 mm wide, ovate, erect, glabrous adaxially and abaxially, the margins papillate, the tips acute, slightly papillate. Stamens five, equal; filaments 1.6–2.5 mm long, lilac, inserted on the corolla 0.8–1.3 mm from the base, with auricles fused to the corolla at the point of insertion; anthers 1.5–1.8 (–2) mm long, ellipsoid, purple or lilac, somewhat connivent or not connivent at anthesis. Gynoecium with ovary 1.2–2 mm long, 1–1.5 mm in diameter, 2 (–4)-carpellate, light green, ovoid or subglobose; ovules more than two per locule; nectary ca. 0.4 mm tall; styles dimorphic, short style 1–1.6 mm, not exceeding the anthers, long style 4.2–7 mm long, exserted ca. 1 mm beyond the anthers, cream with lilac pigmentation, clavate; stigma 0.2–0.37 mm long, ca. 0.75 mm wide, capitate or lobed, pale yellow. Berry 5–7.2 mm in diameter, globose, green turning nearly black when immature, bright red at maturity, deciduous, pungent, the pericarp thick, opaque, with giant cells (endocarp alveolate); stone cells absent; fruiting pedicels 7–12 (–15) mm long, erect, strongly angled, widened distally, green; fruiting calyx 4–6 mm in diameter, persistent, not accrescent, discoid, the appendages 0.0–0.6 mm long, appressed to the berry. Seeds 4–12 per fruit, 3.7–4.8 mm long, 2–3 mm wide, C-shaped or subglobose, yellow to brownish-yellow, the seed coat reticulate (SM), reticulate-cerebelloid (SEM), the cells irregular in shape, the lateral walls mostly sinuate, wavy at margins; embryo imbricate or coiled.

**Figure 117.**
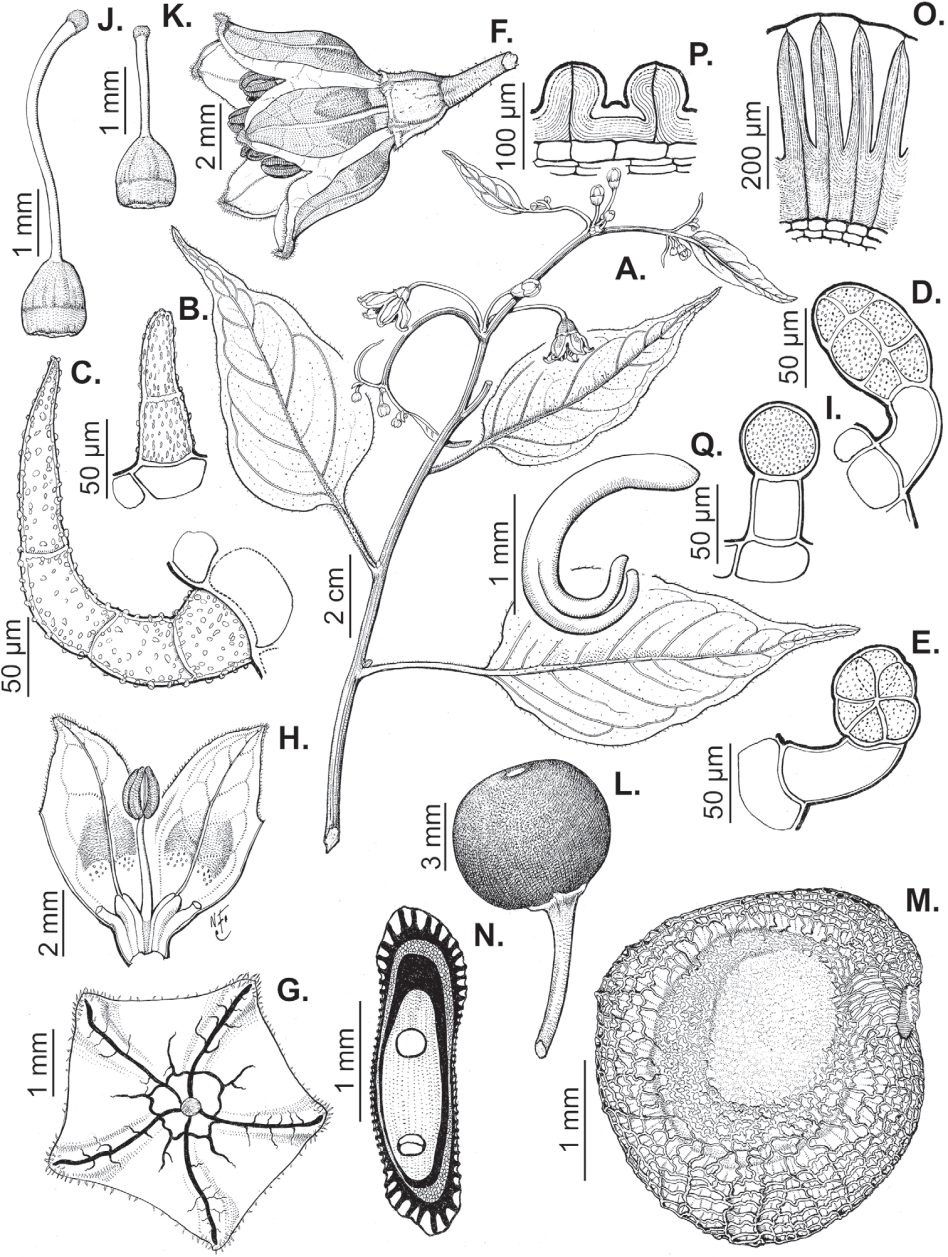
*Capsicumtovarii***A** flowering branch **B, C** eglandular trichomes of the leaf **D, E** glandular trichomes of the leaf **F** flower **G** calyx showing the venation **H** sector of opened corolla **I** glandular trichome of the abaxial surface of the corolla **J** gynoecium with long style **K** gynoecium with short style **L** fruit **M** seed **N** seed, cross section **O** structure of seed coat at the seed margin **P** structure of seed coat at the seed body **Q** embryo. From *Eshbaugh E 1137.* Drawn by N. de Flury.

**Figure 118.**
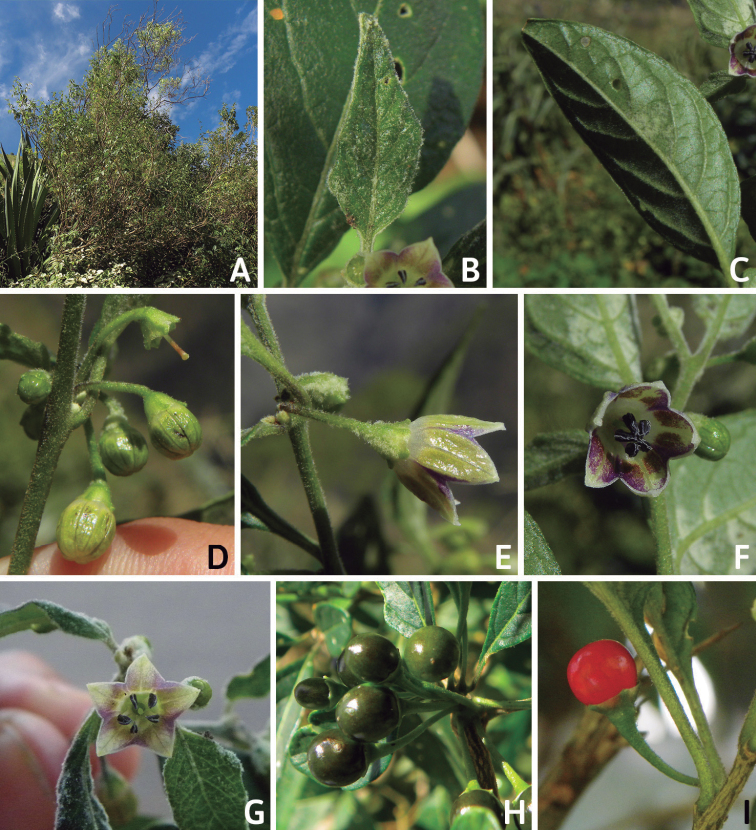
*Capsicumtovarii***A** plant **B** leaf, adaxial surface **C** leaf, abaxial surface **D** node with flower buds on pendent pedicels **E** flower, in lateral view **F, G** flower in front view, showing different pattern of purple colouration **H** immature fruits **I** mature fruit. From *Barboza 5044*. Photos by G.E. Barboza.

#### Distribution.

**Figure 119.**
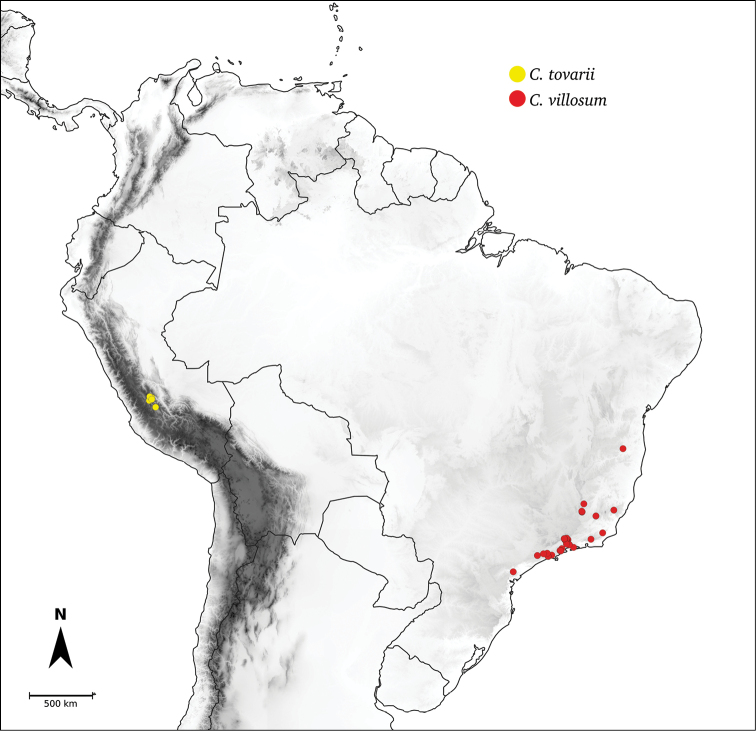
Distribution of *C.tovarii* and *C.villosum*.

*Capsicumtovarii* is an Andean endemic species from central Peru (Junín and Huancavelica Departments) (Fig. 119).

#### Ecology.

*Capsicumtovarii* grows in xerophytic to mesophytic habitats with abundant columnar cacti, *Bombaxruizii* K.Schum. (Eshbaugh et al. 1983) and *Prosopis* spp. across the Rio Mantaro Basin. It is common in full sun or protected under trees, between 850 and 2,500 m elevation.

#### Phenology.

The collections seen are from February to May, all in flower and fruit.

#### Chromosome number.

*n* = 12 (Eshbaugh et al. 1983); 2*n* = 2x = 24 (Moscone et al. 2007; Scaldaferro et al. 2016).

#### Common names.

**Peru**. Ají silvestre (Huancavelica, *Tovar 1867*), Mucuru (Huancavelica, *Tovar 1867*; Junín, *Barboza 5044*), Mucuru-uchu (Huancavelica, *Tovar 1867*).

#### Uses.

The fruits are very pungent and eaten by local people (*Tovar 1867, Barboza 5044*), used as a spice (Eshbaugh et al. 1983).

#### Preliminary conservation assessment.

EOO (1,422.442 km^2^); AOO (28 km^2^). *Capsicumtovarii* occupies a very narrow geographic range in Central Peru. Based on its EOO, AOO and the few nearby locations where it was found, none of them in protected areas and considering that the quality of the habitat will decline by the increased agricultural development, we assign the Endangered (EN; B1+B2ab(iii)) status to this species.

#### Discussion.

*Capsicumtovarii* appears as an isolated branch in the phylogeny of the genus (Carrizo García et al. 2016, see Fig. 1) and it is recognised as the distinct Tovarii clade. The closest affinities of *C.tovarii* have been the subject of debate (see Carrizo García et al. 2016 for detailed information); it has been suggested to be close to either the purple-flowered group (*C.eximium*, *C.cardenasii* or *C.pubescens*) or the white-flowered species (*C.baccatum* complex). An isolated position as sister group to these two groups (the Baccatum and Annuum clades which encompass the most important domesticated species) has recently been proposed for this species (Carrizo García et al. 2016).

This species has been rarely collected (< 10 collections) in Central Peru and the majority of the collections have been made by the late Peruvian botanist Oscar Tovar (USM). The species deserves more field observations to better understand the morphological variation in trichome type, corolla colour, heteromorphism of styles, number of carpels, breeding system and area of distribution. The pubescence varies from glabrescent with a predominance of small glandular trichomes along leaf veins, petioles and pedicels to moderately pubescent with predominance of antrorse eglandular trichomes hiding the glandular ones (but these were somewhat visible by virtue of their dark head). The corolla colour has been annotated as purple or purple-blue in Tovar´s labels (e.g. *Tovar 1867, 5012 & 5363*), but corollas that are entirely cream or cream with two green spots within are also reported in the protologue (Eshbaugh et al. 2013). Similar cream or pale yellow corollas have been observed in plants obtained from seeds from Pariahuanca-Huancayo (sent by O. Tovar in 1999 to A. T. Hunziker, CORD), in which corollas were pale yellow with two green spots at the base of each lobes and throat, while others had purple tones in the interpetalar membrane (*Hunziker 25654*). In a different plant from the same seed accession (*Hunziker 25655*), corollas displayed an evident purple pigmentation in the lobes and adjacent zone of the throat, as was seen by Barboza in another population from Potrero, Huancayo (*Barboza 5044*, Fig. 118F, G). The style is dimorphic, flowers either have short (1–1.6 mm long) or long styles (4.2–7 mm long - only a few styles measured) and the ovary is 2–4-carpellar (also needs more observations). Eshbaugh et al. (1983) mentioned that the flowers were functionally unisexual or bisexual, but more information is needed to associate this with any morphological trait.

### ﻿43. Capsicum villosum

Taxon classificationPlantaeSolanalesSolanaceae

Sendtn., Fl. Bras. (Martius) 10(6): 144. 1846.

E35CECD8-8579-5165-AF3E-E7064B0A63EC

Figs 120 , 121

Capsicum villosum Sendtn. var. latifolium Sendtn., Fl. Bras. (Martius) 10(6): 144. 1846. Type. Brazil, Rio de Janeiro: “Habitat in udis umbrosis sylvarum primaevarum, Provinciae Sebastianopolitanae, Dec.” [no year], C.F.P. von Martius s.n. (lectotype, designated here: M [M-0171535]; isotype: CORD [CORD00006950, fragment from M]).

#### Type.

Brazil, Minas Gerais: “Villa Riccam” [Ouro Preto], [no date], *J.B.E. Pohl [3674*] (lectotype, designated here: W [acc. # 0074662]; isolectotypes: CORD [CORD00006949], F [acc. # 874864], W [acc. # 0074663, acc. # 0074664).

#### Description.

Erect shrubs or subshrubs, (0.80–) 1–3 (–5) m tall, with the main stem thick, ca. 1.5 cm in diameter at base and sprouts from adventitious roots, much branched above, the branches dichotomously spread in a typical “zig-zag” appearance. Young stems 3–angled, somewhat rigid, green, densely pubescent, with spreading, rigid or flexuous, white or slightly ferruginous (dried specimens), simple, uniseriate, 5–8-celled, eglandular trichomes 0.5–1.8 mm long; nodes solid, green; bark of older stems striate, brown or dark brown, glabrescent or sparsely pubescent; lenticels absent. Sympodial units difoliate, the leaves geminate; leaf pair unequal in size, similar or dissimilar in shape. Leaves membranous, discolorous, dark green above, light green beneath, moderately pubescent with appressed-antrorse trichomes similar to those of the stems on adaxial surface, densely pubescent, with long spreading 5–9-celled, eglandular trichomes 0.3–2 mm long on abaxial surface and margins; blades of major leaves 6–17 (–24.5) cm long, (2–) 2.4–7.5 cm wide, ovate, elliptic or narrowly elliptic, the major veins 5–7 on each side of mid-vein, the base attenuate, the margins entire, the apex long-acuminate; petioles 0.4–2.3 (–3.8) cm long, densely pubescent; blades of minor leaves 2.2–2.8 (–5) cm long, 1–1.4 cm wide, ovate or elliptic, the major veins 3–4 on each side of mid-vein, the base attenuate, the margins entire, the apex acute; petioles 0.2–0.3 cm long, densely pubescent. Inflorescences axillary, 3–4 flowers per axil, rarely flowers solitary; flowering pedicels 12–17 (–20) mm long, angled, erect, geniculate at anthesis, green, sparsely to moderately pubescent, the eglandular trichomes long, spreading; pedicels scars inconspicuous. Buds ovoid or globose, inflated, yellowish-green with purple spots or almost entirely purple. Flowers 5-merous. Calyx 1.75–2 mm long, 2–2.5 mm wide, cup-shaped, thick, green, densely pubescent, the calyx appendages 5, (0.5–) 1–2 (–2.5) mm long, 0.2–0.3 mm wide, subequal, thin, erect, cylindrical or subulate, inserted very close to the margin. Corolla 7–9 mm long, 14–14.5 mm in diameter, white with yellowish-green spots and pale purple pigmentation outside, white with intense purple spots, greenish-yellow star and a white centre within, stellate without interpetalar membrane, lobed halfway or less of the way to the base, pubescent adaxially with a continuous ring of glandular trichomes (stalk long, 2–3-celled; head globose, peltate, unicellular) in the throat and base of the lobes, glabrous abaxially, the tube 3.5–4.5 mm long, the lobes (3.5–) 4.2–4.5 mm long, (3.7–) 4–4.75 mm wide, triangular or ovate, spreading, the margins finely ciliate, the tips acute or cucullate, papillate. Stamens five, equal; filaments 2.4–3.6 mm long, white or cream, inserted on the corolla ca. 1.75 mm from the base, with auricles fused to the corolla at the point of insertion; anthers 1.5–2 mm long, ellipsoid, yellow or cream, not connivent at anthesis. Gynoecium with ovary 1.3–1.5 mm in diameter, light green, subglobose; ovules more than two per locule; nectary ca. 0.3 mm tall; styles homomorphic, 3–4.5 mm long, at the same level or barely exserted beyond the anthers, clavate, cream; stigma 0.3 mm long, 0.75–0.9 mm wide, discoid, pale green. Berry 7–9 mm in diameter, globose or globose-depressed, green when immature, greenish-golden yellow at maturity, deciduous, pungent, the pericarp thin, translucent, with giant cells (endocarp alveolate); stone cells absent; fruiting pedicels (13–) 15–24 mm long, pendent, angled, widened distally, green; fruiting calyx 4–5 mm in diameter, persistent, not accrescent, discoid, green, the appendages 0.7–3 mm long, spreading, green. Seeds 8–15 per fruit, (2–) 2.4–2.8 mm long, 2.2–2.5 mm wide, C-shaped or ellipsoid, brownish-black to black, the seed coat reticulate in seed body and tuberculate at margins (SM), reticulate with pillar-like outgrowths at margins (SEM); the cells polygonal in shape, the lateral walls wavy, straight at margins; embryo imbricate.

#### Distribution.

*Capsicumvillosum* is endemic to south-eastern Brazil, most commonly in Rio de Janeiro, Minas Gerais and São Paulo States and only one collection from the State of Espírito Santo (Fig. 119).

#### Ecology.

*Capsicumvillosum* inhabits montane rain forests of the coastal and interior Atlantic Forest (Mata Atlântica), highly adapted to different formations (Floresta Ombrófila Densa, Floresta Ombrófila Mista Aluvial, Campo Rupestre). It forms small colonies along paths near rivers, streams or waterfalls and in open clearings exposed to sun or under the shade, between 500 and 1,900 m elevation.

#### Phenology.

Flowering from October to April. Fruiting from late December to July.

#### Chromosome number.

*n* = 13 (Pozzobon and Schifino-Wittmann 2006, as C.villosumvar.villosum); 2*n* = 2x = 26 (Pozzobon et al. 2006, as C.villosumvar.villosumScaldaferro et al. 2016).

#### Common names.

**Brazil**. Pimentinha (São Paulo, *Kirizawa 3288*).

#### Uses.

None recorded.

#### Preliminary conservation assessment.

EOO (216,703.381 km^2^); AOO (172 km^2^). *Capsicumvillosum* is relatively widespread in the Brazilian Atlantic Forest and occurs in several reserves or in restored and recuperating forests of this system. Although *C.villosum* has a large EOO and occurs in a number of locations, we assign the Near Threatened (NT) category, since the pressure on this ecosystem continues due to the illegal extraction of the forests and its replacement by other land uses which may adversely affect some subpopulations.

#### Discussion.

*Capsicumvillosum* is a hairy species of the Atlantic Forest clade (Carrizo García et al. 2016). This species has dense pubescence, easily visible to the naked eye on all organs of the plant, geniculate pedicels, calyx with five appendages, corolla with various degrees of purple pigmentation within, filaments much longer than anthers, greenish-golden yellow pungent fruits and brownish-black to black seeds (Fig. 121).

**Figure 120.**
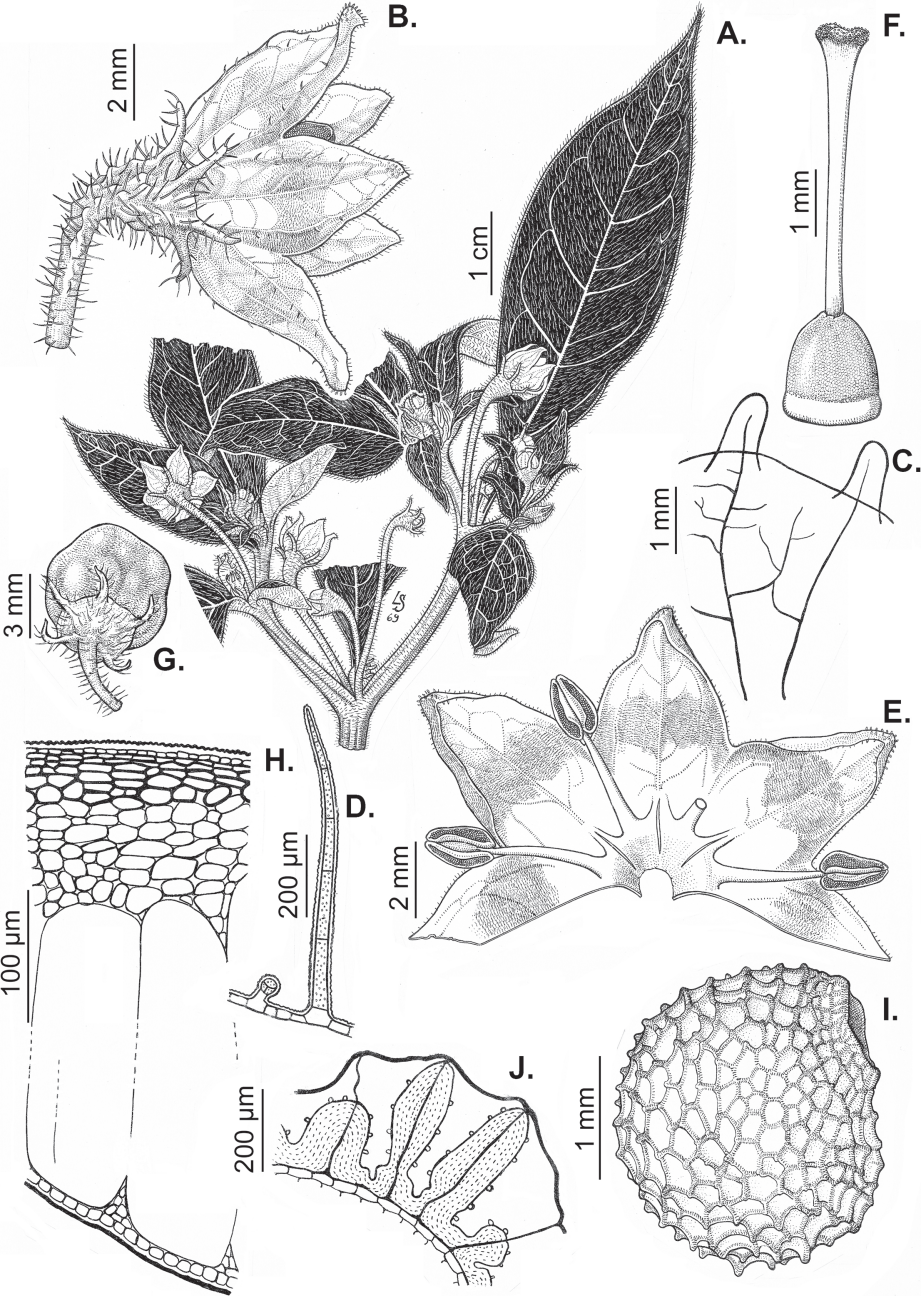
*Capsicumvillosum***A** flowering branch **B** flower **C** section of the calyx showing the venation **D** eglandular and glandular trichomes of the calyx **E** sector of opened corolla **F** gynoecium **G** fruit **H** anatomical detail of the pericarp (note the giant cells in the mesocarp) **I** seed **J** structure of seed coat at the seed margin **A, B, D–F** from *Hunziker 19566***C, H, J** from *Hunziker 25174***G, I** from *Schwacke s.n.* Drawn by L. Sánchez. Modified from Hunziker (1971), reproduced with permission.

**Figure 121.**
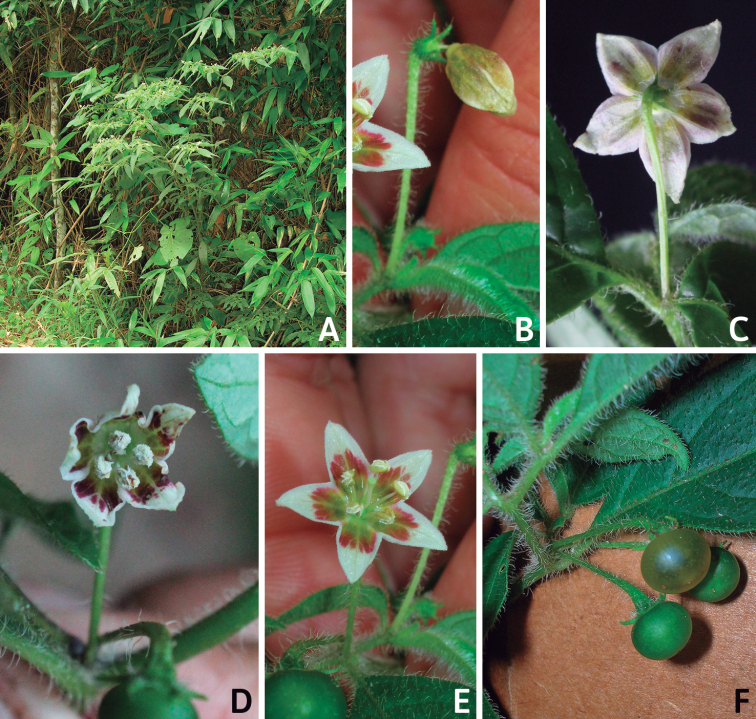
*Capsicumvillosum***A** plant **B** flower bud on a geniculate pedicel **C** flower, seen from behind **D** flower before full anthesis, front view **E** flower at full anthesis, front view **F** fruiting branch, with mature and mature fruits **A, B, E, F** from *Barboza et al. 1655*, photos by G.E. Barboza **C, D** from *Barboza & Deanna 5026 bis*, photos by R. Deanna.

The density and type of pubescence on *C.villosum* is highlighted in the specific epithet. *Capsicumcornutum*, *C.muticum* and *C.rabenii* are also variable in pubescence density. *Capsicumvillosum* can be distinguished for these other pubescent species mainly by corolla colour or size and calyx appendages. *Capsicumvillosum* and *C.rabenii* have five subequal calyx appendages and similar corolla size, but the corolla of *C.villosum* has intense purple spots inside, while in *C.rabenii*, the corolla is only purple or lilac on the margins (Fig. 106F, G). *Capsicumcornutum* has a larger corolla than *C.villosum* and up to 10 unequal calyx appendages (Fig. 53C, D), while *C.muticum* lacks calyx appendages and has no intense purple colouration within the corolla (Fig. 93E, F).

*Capsicumvillosum* is related to *C.mirabile* (Carrizo García et al. 2016); they can be differentiated by the dense pubescence and smaller corolla (7–9 mm long) in *C.villosum* vs. plants glabrescent to glabrous and larger corollas (6–12 mm long) in *C.mirabile*.

Smith and Downs (1966) identified plants of *Athenaeawettsteiniana* (Witasek) I.M.C.Rodrigues & Stehmann and *Athenaeafasciculata* (Vell.) I.M.C.Rodrigues & Stehmann as *C.villosum* in their flora of Santa Catarina State, Brazil; *C.villosum* does not grow in Santa Catarina. Hunziker (1969b) cited *Mexia 4328* as a paratype of *C.buforum* (= *C.mirabile*), stating that young stems, leaves, pedicels and calyx are pubescent, a character (amongst others) that places it within the circumscription of *C.villosum*.

In describing *C.villosum*, Sendtner (1846) cited several collections for the species (*Pohl s.n.*) and the varieties (*Martius s.n.* for var. latifolium; *Schott s.n.* and *Selllow s.n.* for var. muticum [see above under *C.muticum*]). Original material collected by Pohl from “Villa Riccam”, the cited locality and bearing the number “3674”, was found in three Herbaria (CORD, F, W). The three sheets housed at W are the best-preserved, two of these specimens (acc. #0074663, acc. #0074664) are only labelled with the number ‘3674’ on the sheet, while the other (acc. # 0074662) has an original complete label with the locality data of the protologue and, for this reason, is here designated as lectotype. Collections of Martius with the protologue locality for C.villosumvar.latifolium were found in Munich and a fragment at CORD; the one at M (M-0171535) is the best-preserved and is selected as the lectotype.

##### ﻿Doubtful names

CapsicumannuumL.var.fingerhuthi Alef., Landw. Fl.: 133. 1866.

Type. No locality cited (no specimens cited, no original material located). This variety was compared with C.annuumL.var.cordiforme (Mill.) Alef. but with scarlet fruit = C.annuumL.var.annuum or *C.chinense* Jacq.

CapsicumannuumL.var.leptoceras Alef., Landw. Fl.: 132. 1866.

Type. “So erhielt ich Exempl. aus Mexico” (no specimens cited, no original material located). The short German description for this variety refers to the size, shape and colour of the fruit and the calyx appendages shape which can apply to both CapsicumannuumL.var.annuum or C.baccatumvar.pendulum (Willd.) Eshbaugh

CapsicumannuumL.var.varians Voss in Vilm., Vilm. Blumengärtn., ed. 3. 1: 723. 1894.

Type. No locality cited (no specimens cited, no original material located). Voss (1894) described this variety alluding to the shape and the colour change of the fruit (mostly yellow or scarlet to red) = C.annuumL.var.annuum or *C.chinense* Jacq. or C.baccatumvar.pendulum (Willd.) Eshbaugh

CapsicumannuumL.var.weinmanni Alef., Landw. Fl.: 133. 1866.

Type. No locality cited (no specimens cited, no original material located). Alefeld (1866) indicated this variety was similar to C.annuumL.var.longum (DC.) Alef., but different in its vermilion fruit colour = CapsicumannuumL.var.annuum or C.baccatumvar.pendulum (Willd.) Eshbaugh

*Capsicumbauhini* Dunal, Prodr. [A. P. de Candolle] 13(1): 428. 1852.

Type. No locality cited. Dunal (1852) repeats exactly the Bauhin (1623: 103) polynomial “Piper Indicum, caule piloso, flore majore; Piper Indicum, caule pilis albis praedito, Cam.” (no type specimens cited, no original material located). = probably C.annuumL.var.annuum.

*Capsicumcerasiflorum* Link, Enum. Hort. Berol. Alt. 1: 190. 1821.

Type. “Habitat....”. (no specimens cited; original material in B, destroyed). As Link’s description is very brief and the characters mentioned are common to many species (“Petiolis junioribus ciliatis baccis erectis globosis”), its identity is uncertain and could apply to any of these domesticated species: *Capsicumannuum* L., *C.chinense* Jacq., or *C.baccatum* L.

CapsicumcerasiformeWilld.var.cerasiflorum (Link) Dunal, Prodr. [A. P. de Candolle] 13 (1): 422. 1852.

Type: Based on *Capsicumcerasiflorum* Link (a doubtful name, see above).

CapsicumcerasiformeWilld.var.minus Fingerh., Monogr. Capsic.: 20. 1832.

Type. “Patria America australis et India? orientalis” (no specimens cited; no original material located). Fingerhuth (1832) gave a very short description “fructu minori luteo vel rubro” for this variety, which can be attributed to C.annuumvar.glabriusculum (Dunal) Heiser & Pickersgill or to *C.chinense* Jacq.

*Capsicumchamaecerasus* Nees, Trans. Linn. Soc. London 17: 65. 1834. nom. illeg. superfl.

Type. Based on *Capsicumcerasiforme* Poir., Enc. Meth. 5 [in error Suppl. v.] p. 325. (nec Willd.), 1804 and *Capsicumpurpureum* Roxb. (ad partem) (both are cited in synonymy). As there is no accurate evidence of Nees concept for *C.cerasiforme* (in this treatment *C.cerasiforme* Lam. and *C.cerasiforme* Willd. are synonyms of *C.chinense*) and because *C.purpureum* Roxb. is considered here synonymy of C.annuumvar.annuum, we cannot properly assign identity of *C.chamaecerasus*.

*Capsicumconicum* Hornem., Hort. Bot. Hafn. Suppl.: 27. 1819, nom. illeg., not *C.conicum* G. Mey. (1794).

Type. “Hab. - - C. [Planta Caldarii] intr. 1813. Missum a celb. Sohradero sub hoc nomime” (no original material located). Hornemann (1819) described *C.conicum*, based on cultivated material at Horto Hafniensis (Copenhagen), with no provenance data. His short diagnosis does not allow us to determine if this name represents C.annuumL.varannuum or *C.chinense* Jacq., but we prefer not to assign it to either until original material can be found (probably in C).

CapsicumcordiformeMill.var.cerasicarpon Dunal, Prodr. [A. P. de Candolle] 13(1): 428. 1852.

Type. “v.v. in hort. Monsp. s. in h. meo.” (no original material located). Dunal (1852) described this variety with living material from the Botanical Gardens in Montpellier and from a specimen housed at MPU. We have been unable to find any material corresponding to this name. The description “fructu globoso pendulo rubro cersai colore et magnitudine” can be applied to C.annuumL.var.annuum or *C.chinense* Jacq.

*Capsicumhornemanii* Dunal, Prodr. [A. P. de Candolle] 13(1): 429. 1852.

Type. Replacement name for *C.conicum* Hornem. (see above).

*Capsicumindicum* Dierb., Arch. Apotheker-Vereins Nordl. Teutschl. 30: 22. 1829.

Type. “Capsicumindicum Lobelii”; Dierbach (1829) preferred the use of the L’Obel’s pre-Linnaean designation “Capsicumindicum” (L’Obel, 1576: 116) instead of using one of the Linnaean species names to refer to all chili peppers known by him. Dierbach’s brief description (“foliis oppositis, fructibus polymorphis, seminibus compressis”) could apply to at least four of the cultivated species (*C.annuum* L., *C.frutescens* L., *C.chinense* Jacq. or *C.baccatum* L.) and, in the absence of a type specimen, we consider *C.indicum* a doubtful name. Dierbach (1829) proposed very confusing infraspecific classification for the single species (*C.indicum*) of peppers he recognised. He designated unranked taxa by numbers (1 to 5), based on broad variation in fruit shape. None of these names (see below) has an indication to a reference or a specimen and the description of the fruits could again apply to any domesticated *Capsicum* species.

*Capsicumindicum* Dierb. [unranked category] 1) *macrocarpon*, Dierb., Arch. Apotheker-Vereins Nordl. Teutschl. 30: 22. 1829.

*Capsicumindicum* Dierb. [unranked category] 2) *pachycarpon*, Dierb., Arch. Apotheker-Vereins Nordl. Teutschl. 30: 25. 1829.

*Capsicumindicum* Dierb. [unranked category] 3) *cerasocarpon*, Dierb., Arch. Apotheker-Vereins Nordl. Teutschl. 30: 26. 1829.

*Capsicumindicum* Dierb. [unranked category] 4) *elaeocarpon*, Dierb., Arch. Apotheker-Vereins Nordl. Teutschl. 30: 28. 1829.

*Capsicumindicum* Dierb. [unranked category] 5) *microcarpon*, Dierb., Arch. Apotheker-Vereins Nordl. Teutschl. 30: 29. 1829.

*Capsicummicranthum* Link, Enum. Hort. Berol. Alt. 1: 190. 1821.

Type. “Hab. in Brasilia” (no specimens cited; no original material located); Link (1821) gave a short diagnosis for *C.micranthum*; the calyx and fruit features, as well as its origin (Brazil), make us suppose that it could be *C.frutescens* L., but as Link did not mention type specimens, we cannot unequivocally assure its identity.

CapsicumpyramidaleMill.var.torulosum (Hort.Matr. ex Hornem.) Fingerh., Monogr. Capsic.: 15. 1832.

Type. Based on *Capsicumtorulosum* Hort.Matr. ex Hornem. (a doubtful name itself).

*Capsicumtorulosum* Hort.Matr. ex Hornem., Hort. Bot. Hafn. Suppl.: 27. 1819.

Type. “Hab.- - C. [Planta Caldarii] intr. 1815”. Hornemann (1819) described *C.torulosum*, based on cultivated material at Horto Hafniensis (Copenhagen), from Horto Matritensis. In the diagnosis, he also added “An diversum a *C.pendulo*?”. As we did not find original material, we cannot exactly place this name, but judging by the diagnosis, it could be C.annuumL.var.annuum or C.baccatumvar.pendulum (Willd.) Eshbaugh.

##### ﻿Names (designations) not validly published

Designations are listed in alphabetic order; ICN Articles refer to the Shenzhen Code (Turland et al. 2018).

CapsicumsectionEucapsicum Kuntze, Revis. Gen. Pl. 2: 448. 1891, not validly published, the name of the subdivision formed from the name of the genus by adding the prefix Eu- (Art. 21.3).

CapsicumsectionEucapsicum Wettst., Nat. Pflanzenfam. 4(3b): 20. 1891, not validly published, the name of the subdivision formed from the name of the genus by adding the prefix Eu- (Art. 21.3).

CapsicumannuumL.formachlorocarpum Kuntze, Revis. Gen. Pl. 2: 449. 1891, nomen subnudum; Kuntze (1891) only says “fructus virides”; this scarce information associated with this name does not distinguish it from any other species.

CapsicumannuumL.formaleucocarpum Kuntze, Revis. Gen. Pl. 2: 449. 1891, certainly not intended as a new name, citation of “albidi (Mill. em.)” = C.annuumL.var.annuum

CapsicumannuumL.formaluteum Kuntze, Revis. Gen. Pl. 2: 449. 1891, certainly not intended as a new name, citation of “fructus aurantiaci vel rubri vel flavi (W. em.)” = C.annuumL.var.annuum

CapsicumannuumL.formaviolaceum Kuntze, Revis. Gen. Pl. 2: 449. 1891, certainly not intended as a new name, citation of “fructus violacei, atropurpurei (Brouss. em.)” = C.annuumL.var.annuum

CapsicumannuumL.var.leucocarpon Voss in Vilm., Vilm. Blumengärtn., ed. 3. 1: 723.1894, nom. subnudum; the very brief German description does not provide a character to distinguish this variety from any other and it is not validly published.

*Capsicumbaccatum* Buch.-Ham. ex Wall., Numer. List [Wallich] n. 2644. 1831, nomen nudum; a manuscript name for a specimen from Jolpighurri (India) held at K [K001116731] = *C.frutescens* L.

*Capsicumbaccatum* hort.Genev. ex Dunal, Prodr. [A. P. de Candolle] 13(1): 420. 1852, pro syn. *Capsicumangustifolium* Dunal = C.annuumL.var.annuum

*Capsicumcaerulescens* Besser, Cat. Hort. Crem.: 29. 1816, not validly published; name in a list with no diagnosis or description (Art. 38.1) and referred to other nomen nudum *Capsicumluteum* H.V. [(Schott) H. Vindebonensis] cited in Besser (1811. p. 25 [in error: 17], n. 6) = probably C.annuumL.var.annuum.

*Capsicumcerasiforme* hort. ex Dunal, Prodr. [A. P. de Candolle] 13(1): 420. 1852, pro syn. *C.baccatum* L.; based on a manuscript name “Capsicumbaccatum hort.”, at G-DC [G0000131879] = *Capsicumbaccatum* L.

*Capsicumcydoniforme* hort. ex Roem. & Schult., Syst. Veg., ed. 15 bis [Roemer & Schultes] 4: 561. 1819; pro syn. *C.tetragonum* Mill. = C.annuumL.var.annuum.

*Capsicumlongum* Bouton ex Dunal, Prodr. [A. P. de Candolle] 13(1): 414. 1852, pro syn. *Capsicumconoide* Mill.; based on a manuscript name “Capsicumlongum, Isle Mauricie, 1839, *L. Boutoun*” at G-DC [G0000131840] = *Capsicumfrutescens* L.

*Capsicumminimum* Blanco, Fl. Filip. [F.M. Blanco]: 133. 1837, not intended as a new name, citation of *Capsicumminimum* Roxb. in text (Merrill 1918) = *C.frutescens* L.

*Capsicumnarunca* Dunal, Prodr. [A. P. de Candolle] 13(1): 414. 1852, pro syn. *Capsicumbicolor* Jacq.; based on a manuscript name “Capsicum naruna” at G-DC [G00131853], = CapsicumannuumL.var.annuum.

*Capsicumodoratum* Steud., Nomencl. Bot. [Steudel], ed. 2. 1: 279. 1840, not validly published; no diagnosis or description (Art. 38.1), name in a list with only “*Arrab*. [Anton da Arrabida], *Brasil*” as reference = identity uncertain.

*Capsicumpubescens* Dunal, Prodr. [A. P. de Candolle] 13(1): 415. 1852, pro syn. *C.chlorocladum* Dunal; based on a manuscript name “Capsicumpubescens Dun. 2 Feb 1844”, at G-DC [G00131884] = C.annuumL.var.glabriusculum (Dunal) Heiser & Pickersgill.

*Capsicumtournefortii* Besser, Cat. Hort. Crem.: 30. 1816, not validly published; no diagnosis or description (Art. 38.1), name in a list referred to other nomen nudum *Capsicum* “(Tourneforti) *pomiferum* L.” (Besser 1815: 6) = probably C.annuumL.var.annuum.

*Capsicumviolaceum* Desf., Tabl. École Bot. 70. 1804; Desv. in Ham. Prod. 25. 1804, not validly published; no diagnosis or description (Art. 38.1) = probably C.annuumL.var.annuum

*Capsicumviolaceum* H.R.M.Brouss., Elench. Monsp.: 12. 1805, not validly published; no diagnosis or description (Art. 38.1), name in a list as “C. violaceum H.R.M. [Horto Regis Matritensis] = C.annuumL.var.annuum

*Solanumbrachypodum* Dunal, Prodr. [A. P. de Candolle] 13(1): 411. 1852, pro syn. *Bassoviabrachypoda* Dunal; based on a manuscript name at G; the Pavon specimen at G is annotated “Solanumbrachypodum Dun.” = *Capsicumhookerianum* (Miers) Kuntze

*Solanumdivergens* Dunal, Prodr. [A. P. de Candolle] 13(1): 411. 1852, pro syn. *Bassovialeptopoda* Dunal; based on a manuscript name at H. Banks (BM000798808) where it is ink-annotated “S. divergens Dun. ined.” and with a pencil annotation on the same label “n. 131” = *Capsicummuticum* Barboza

*Solanumleptopodum* Dunal, Prodr. [A. P. de Candolle] 13(1): 411. 1852, pro syn. *Bassovialeptopoda* Dunal; based on handwritten names at MPU (MPU023056) and G-DC (G00131651) = *Capsicummuticum* Barboza

##### ﻿Excluded names

*Capsicumanomalum* Franch. & Sav., Enum. Pl. Jap. 2: 452. 1878 = *Tubocapsicumanomalum* (Franch. & Sav.) Makino (Hunziker 2001).

*Capsicumasterotrichum* Standl., Publ. Field Mus. Nat. Hist., Bot. Ser. 4: 259. 1929 = *Witheringiaasterotricha* (Standl.) Hunz. (Bohs 2015).

*Capsicumboninense* Koidz., Fl. Symb. Orient.-Asiat.: 31. 1930 = *Tubocapsicumanoma­lum* (Franch. & Sav.) Makino (D’Arcy et al. 2001).

*Capsicumcostaricense* Standl. & C.V.Morton, Publ. Field Mus. Nat. Hist., Bot. Ser. 18: 1040. 1938 = *Witheringiaasterotricha* (Standl.) Hunz. (Bohs 2015).

*Capsicumdunalii* Kuntze, Revis. Gen. Pl. 2: 450. 1891, nom. nov. for *Fregirardiaangustifolia* Dunal = *Lyciantheslycioides* (L.) Hassl. (Barboza and Hunziker 1992).

*Capsicumglandulosum* Dunal, Prodr. [A. P. de Candolle] 13(1): 417.1852 = *Vassobiabreviflora* (Sendtn.) Hunz. (Hunziker 1984).

*Capsicumgrandiflorum* Kuntze, Revis. Gen. Pl. 3[3]: 218. 1898 = *Vassobiafasciculata* (Miers) Hunz. (Hunziker 1984).

*Capsicuminaequale* Vell., Fl. Flumin.: 61. 1829 (“1825”) = *Solanumdidymum* Dunal (Knapp et al. 2015).

*Capsicumisothrix* Standl., Publ. Field Mus. Nat. Hist., Bot. Ser. 18: 1567. 1938 = *Witheringiasolanacea* L’Her. (Bohs 2015).

*Capsicumlundellii* C.V.Morton, Contr. Univ. Michigan Herb. 4: 25. 1940 = *Witheringiaaffinis* (C.V. Morton) Hunz. (Gentry & Standley 1974).

*Capsicummacranthum* Standl. & C.V.Morton, Publ. Field Mus. Nat. Hist., Bot. Ser. 18: 1042.1938 = *Witheringiamacrantha* (Standl. & C.V.Morton) Hunz. (Bohs 2015).

*Capsicummaculatum* Standl. & C.V.Morton, Publ. Field Mus. Nat. Hist., Bot. Ser. 18: 1043. 1938 = *Witheringiamaculata* (Standl. & C.V.Morton) Hunz. (Bohs 2015).

*Capsicummalacophyllum* Standl., Publ. Field Mus. Nat. Hist., Bot. Ser. 4: 260. 1929 = *Witheringiastramoniifolia* Kunth (Nee 1986).

*Capsicummultiflorum* Standl. & C.V.Morton, Publ. Field Mus. Nat. Hist., Bot. Ser. 18: 1043. 1938 = *Witheringiaasterotricha* (Standl.) Hunz. (Bohs 2015).

*Capsicumsilvigaudens* Standl. & L.O.Williams, Ceiba 3: 57. 1952 = *Witheringiameiantha* (Donn. Sm.) Hunz. (Nelson 2001).

Capsicumsolanaceum(L’Hér.)Kuntzevar.glabrescens Kuntze, Revis. Gen. Pl. 2: 450. 1891 = *Witheringiasolanacea* L’Her. (Hunziker 1969a).

Capsicumsolanaceum(L’Hér.)Kuntzevar.pubescens Kuntze, Revis. Gen. Pl. 2: 450. 1891 = *Witheringiasolanacea* L’Her. (Hunziker 1969a).

*Capsicumstenophyllum* C.V.Morton & Standl., Publ. Field Mus. Nat. Hist., Bot. Ser. 18: 1044. 1938 = *Witheringiameiantha* (Donn. Sm.) Hunz. (Bohs 2015).

*Capsicumsubulatum* Standl. & C.V.Morton, Publ. Field Mus. Nat. Hist., Bot. Ser. 18: 1044. 1938 = *Witheringiastramonifolia* Kunth (Bohs 2015).

*Capsicumtetramerum* Standl. & C.V.Morton, Publ. Field Mus. Nat. Hist., Bot. Ser. 18: 1045. 1938 = *Witheringiasolanacea* L’Her. (Bohs 2015).

*Capsicumtorulosum* Vell., Fl. Flumin.: 60. 1829 (“1825”) = *Pombaliaatropurpureum* (St.Hil.) Paula-Souza (Violaceae) (Knapp et al. 2015).

*Capsicumvelutinum* Kuntze, Revis. Gen. Pl. 2: 448. 1891 = *Athenaeavelutina* (Sendtn.) D’Arcy (Rodrigues et al. 2019).

*Capsicumviscidum* Standl., Publ. Carnegie Inst. Wash. 461: 84. 1935 = *Witheringianelsonii* (Fernald) Hunz. (Nee 1986).

## Supplementary Material

XML Treatment for Capsicum

XML Treatment for Capsicum annuum

XML Treatment for Capsicum annuum L. var. annuum

XML Treatment for Capsicum annuum L. var. glabriusculum

XML Treatment for Capsicum baccatum

XML Treatment for Capsicum baccatum L. var. baccatum

XML Treatment for Capscium baccatum L. var. pendulum

XML Treatment for Capsicum baccatum L. var. umbilicatum

XML Treatment for Capsicum benoistii

XML Treatment for Capsicum caatingae

XML Treatment for Capsicum caballeroi

XML Treatment for Capsicum campylopodium

XML Treatment for Capsicum carassense

XML Treatment for Capsicum cardenasii

XML Treatment for Capsicum ceratocalyx

XML Treatment for Capsicum chacoense

XML Treatment for Capsicum chinense

XML Treatment for Capsicum coccineum

XML Treatment for Capsicum cornutum

XML Treatment for Capsicum dimorphum

XML Treatment for Capsicum eshbaughii

XML Treatment for Capsicum eximium

XML Treatment for Capsicum flexuosum

XML Treatment for Capsicum friburgense

XML Treatment for Capsicum frutescens

XML Treatment for Capsicum galapagoense

XML Treatment for Capsicum geminifolium

XML Treatment for Capsicum hookerianum

XML Treatment for Capsicum hunzikerianum

XML Treatment for Capsicum lanceolatum

XML Treatment for Capsicum longidentatum

XML Treatment for Capsicum longifolium

XML Treatment for Capsicum lycianthoides

XML Treatment for Capsicum minutiflorum

XML Treatment for Capsicum mirabile

XML Treatment for Capsicum mirum

XML Treatment for Capsicum muticum

XML Treatment for Capsicum neei

XML Treatment for Capsicum parvifolium

XML Treatment for Capsicum pereirae

XML Treatment for Capsicum piuranum

XML Treatment for Capsicum pubescens

XML Treatment for Capsicum rabenii

XML Treatment for Capsicum recurvatum

XML Treatment for Capsicum regale

XML Treatment for Capsicum rhomboideum

XML Treatment for Capsicum schottianum

XML Treatment for Capsicum tovarii

XML Treatment for Capsicum villosum

## ﻿List of exsiccatae

Abbott, J.R. 16182, 16431 (*baccatum* var. baccatum)

Abruzzi, M.L. 576 (*flexuosum*)

Acevedo-Rodríguez, P. 2433, 11344 (*annuum* var. glabriusculum)

Acevedo-Rodríguez, P. & D. Chinea 2653 (*annuum* var. glabriusculum)

Acevedo-Rodríguez, P. & J. Knight 15650 (*annuum* var. glabriusculum)

Acevedo-Rodríguez, P. & A. Reilly 2122 (*annuum* var. glabriusculum)

Acevedo-Rodríguez, P. et al. 646, 5092, 5554, 5652, 7223, 14065 (*annuum* var. glabriusculum)

Acevedo, S. et al. 1331 (*annuum* var. glabriusculum)

Acosta B., L.E. 187 (*annuum* var. glabriusculum)

Acosta P., R. 1405 (*lanceolatum*)

Acosta P., R. & F. Vazquez B. 753 (*rhomboideum*)

Acosta P., R. et al. 39 (*annuum* var. glabriusculum)

Acosta Castellanos, S. 9417 (*annuum* var. annuum)

Acosta Solís, M. 6435, 6899, 6968 (*lycianthoides*); 8047 (*pubescens*); 8597 (*rhomboideum*); 8848 (*annuum* var. annuum); 8916, 11252, 11295 (*rhomboideum*); 11366 (*annuum* var. glabriusculum); 12925 (*rhomboideum*); 13577 (*annuum* var. annuum); 13927 (*frutescens*); 13989 (*chinense*); 14234, 14819, 14862 (*rhomboideum*); 16208 (*baccatum* var. pendulum); 16452 (*rhomboideum*)

Adams, C.D. 5961, 8049 (*annuum* var. glabriusculum)

Adrián, C.O. 84 (*pubescens*)

Adsersen, A. & H. Adsersen 1517, 2361 (*frutescens*)

Agra, M.F. 74, 625 (*frutescens*); 658 (*parvifolium*)

Agra, M.F. & G.E. Barboza 7075 (*parvifolium*); 7086 (*longidentatum*)

Agra, M.F. & L. Bohs 7257 (*caatingae*)

Agra, M.F. et al. 2569, 2639, 4776, 4903, 5171, 5249, 5297, 5479 (*parvifolium*); 7081 (*caatingae*); 7083 (*longidentatum*); 7085 (*caatingae*); 7244 (*mirabile*); 7268 (*carassense*); 7293, 7299 (*friburgense*); 7323, 7331 (*mirabile*); 7340 (*carassense*); 7360 (*mirabile*); 7400 (*villosum*)

Aguayo, A. 75 (*flexuosum*)

Aguayo C., J.A. & H.A. Gómez R. 191 (*annuum* var. glabriusculum)

Agudelo, C.A. et al. 899 (*lycianthoides*); 1596 (*dimorphum*)

Aguiar, L. & L. Martau 339 (*baccatum* var. baccatum)

Aguilar H., M. 64, 177 (*annuum* var. glabriusculum); 199 (*frutescens*)

Aguilar, I. 46 (*rhomboideum*)

Aguilar, M.H. 78 (*annuum* var. annuum)

Aguilar, R.M. 136, 568, 721 (*chacoense*)

Aguilar Zepeda, J.A. & S. Diez Martínez y Day 38, 363 (*annuum* var. glabriusculum)

Aguinda, R. et al. 1537 (*regale*)

Aguirre-Galviz, L.E. 1099 (*frutescens*)

Agúndez, J.M. 221, 633 (*annuum* var. glabriusculum)

Ahumada, O. 5013 (*chacoense*)

Ahumada, O. & A. Castellón 9065 (*baccatum* var. baccatum)

Alarcón, E. 102 (*annuum* var. glabriusculum)

Albert de Escobar, L.K. & A. Uribe 3645 (*lycianthoides*)

Alburquerque, L.B. et al. 49 (*hunzikerianum*)

Alcorn, J.B. 1406, 1903, 2369 (*annuum* var. glabriusculum)

Alegría O., P. 47 (*frutescens*)

Aliscioni, S.S. et al. 665 (*chacoense*)

Allard, H.A. 2265, 14542, 14697, 14787, 17784 (*annuum* var. glabriusculum); 21850 (*pubescens*)

Allemão, F. 1229 (*parvifolium*)

Allen, A. & Werneck 3505 (*rabenii*)

Allen, C. 484 (*annuum* var. glabriusculum)

Allen, P.H. 2006 (*annuum* var. glabriusculum)

Almeda, F. et al. 3119 (*annuum* var. glabriusculum)

Almeida, E.C. 48 (*rabenii*)

Almeida de Jesus, J. 1959, 2064 (*campylopodium*)

Almeida Scabbia, R.J. et al. 910 (*mirabile*); 5113 (*cornutum*)

Alonso Méndez, G. 7709 (*rhomboideum*)

Alston, A.H.G. 7688 (*rhomboideum*); 8157 (*dimorphum*)

Altamirano, S. et al. 3711 (*coccineum*)

Alvarado C., A. 198 (*annuum* var. annuum)

Alvarado Flores, F. 116 (*annuum* var. glabriusculum)

Álvarez, A. et al. 1584 (*lycianthoides*)

Álvarez, D. 89, 1491, 2075 (*annuum* var. glabriusculum)

Álvarez, D. et al. 11583 (*annuum* var. glabriusculum)

Álvarez, E. & O. Montañéz 1 (*annuum* var. annuum)

Alvaro M., P. 397 (*annuum* var. glabriusculum)

Alvaro M., P. & D. Álvarez 741 (*annuum* var. glabriusculum)

Alves de Paiva, G. 01 (*baccatum* var. pendulum)

Amancio, A.M.D. et al. 188 (*flexuosum*)

Amaral Santos, A. et al. 721 (*frutescens*)

Amaral, E.V.E. 38, 62 (*rabenii*); 63 (*baccatum* var. pendulum); *65* (*baccatum* var. umbilicatum); 139 (*chinense*)

Amarilla, L. et al. 31 (*flexuosum*)

Ambrosetti, J.A. 1898 (*chacoense*)

Amorim, A.M. et al. 4429, 5371 (*mirabile*); 6317 (*caatingae*); 7235 (*mirabile*); 8292 (*longidentatum*)

Ancuash Atsut, E. 296 (*chinense*); 297 (*annuum* var. annuum)

Anderson, D.L. 1447 (*chacoense*)

Anderson, L.O. et al. 99/36 (*mirabile*)

Anderson, W.R. 11718 (*villosum*); 12556 (*annuum* var. glabriusculum)

Anderson, W.R. et al. 35734 (*frutescens*)

Andino U., R. 69 (*rhomboideum*)

Andrade, P.M. & M.A. Lopes 109 (*campylopodium*)

Andrade, P.R. & R.M. Chagas 1188 (*rabenii*)

Andrade, R. 33001B (*chinense*)

André, A. 3317 (*villosum*)

André, E.F. 701, 1780, 2520 (*rhomboideum*); 3839, 4570 (*geminifolium*)

Andreata, R. et al. 522 (*campylopodium*)

Angel, S. et al. 465 (*dimorphum*)

Ângulo, F. 007 (*frutescens*)

Anonymous 67, 130 (*annuum* var. glabriusculum); 05/17 (*rabenii*); 1414/6 (G00131878) (*annuum* var. glabriusculum)

Antezana, C.721 (*eximium*)

Anzótegui-Benitez 109, 237 (*annuum* var. annuum)

Aona, L.Y.S. et al. 1220 A (*baccatum* var. baccatum); 2046B (*pereirae*)

Apolinar María (Nicholas Seiler), Bro. 114 (*annuum* var. glabriusculum)

Araki, D.F. et al. 105 (*recurvatum*); 116 (*flexuosum*)

Aranda et al., M. 94 (*annuum* var. glabriusculum)

Araque, J. 462 (*lanceolatum*)

Araque Molina, J. & F.A. Barkley 308 (*rhomboideum*); 18N.S.140 (*annuum* var. glabriusculum); 18M.050, 19 A053 (*frutescens*)

Araquistain, M. 5, 262, 278, 394 (*annuum* var. glabriusculum)

Araquistain, M. & P.P. Moreno 69, 505, 1276, 2856 (*annuum* var. glabriusculum)

Araujo, G.B. 642 (*caatingae*)

Araujo, G.B. et al. 205 (*caatingae*)

Araujo-M., A. et al. 1638 (*frutescens*)

Arbeláez S., G. et al. 1136, 2466 (*lycianthoides*)

Arbo, M.M. 341 (*annuum* var. annuum)

Arbo, M.M. et al. 2038 (*flexuosum*)

Archer, W.A. 64 (*rhomboideum*); 93 (*annuum* var. glabriusculum); 605, 761 (*rhomboideum*); 1170, 1273 (*dimorphum*); 1404 (*chinense*)

Arcos Vernet, R. 40 (*annuum* var. glabriusculum)

Areces-Mallea, A.E. 2233 (*annuum* var. glabriusculum)

Arenas, P. 327, 436, 555, 645, 1393, 1546, 1688, 1853, 1956, 2121, 2197, 2377, 3043, 3388, 3764 (*chacoense*)

Argañaraz, J.L. 210 (*chacoense*)

Argeñal, F. 130 (*annuum* var. glabriusculum)

Argüelles, E. 889 (*rhomboideum*)

Arias-G, J.C. & J. Garzón J. 667, 668 (*frutescens*)

Arias-G., J.C. et al. 682 (*frutescens*)

Aristaguieta 3372, 4442, 5415, 6139 (*rhomboideum*)

Ariza Espinar, L. 248, 1626 (*chacoense*); 3213 (*annuum* var. annuum)

Arroyo P., L. et al. 6053 (*minutiflorum*); 6108 (*neei*)

Arroyo, L. et al. 2880, 2930 (*minutiflorum*); 3313 (*baccatum* var. baccatum), 3317, 3333 (*minutiflorum*); 5252 (*eximium*)

Arsène, G. 10587 (*chinense*)

Arvigo, R. et al. 148 (*frutescens*)

Asplund, E. 6548, 6701, 6992, 7576, 8104 (*rhomboideum*); 9713 (*geminifolium*); 12491, 12940 (*coccineum*); 13115 (*geminifolium*); 15241, 15280, 15363 (*hookerianum*); 18050 (*pubescens*); 15822, 18272 (*rhomboideum*); 19430, 19746 (*chinense*); 19939 (*rhomboideum*); 20070 (*Capsicumbenoistii*)

Atahuachi, M. et al. 773 (*eximium*); 1629 (*caballeroi*); 1632, 1634, 1635, 1636, 1666 (*baccatum* var. baccatum)

Atamp 7659 (*muticum*)

Atha, D.E. et al. 1022 (*frutescens*)

Attié, M.C.B. et al. 59 (*recurvatum*)

Atwood, J.T. 2488 (*annuum* var. glabriusculum)

August, L. 18 (*chacoense*)

Augusto, H.H. 4847 (*annuum* var. glabriusculum)

Avellaneda, M. 12 (*annuum* var. glabriusculum)

Avendaño R., S. & C. Durán E. 3143 (*annuum* var. glabriusculum)

Ávila et al., A. 783 (*annuum* var. glabriusculum); 817 (*rhomboideum*)

Ayala, F. 5786 (*annuum* var. glabriusculum)

Ayala, F. et al. 2792, 2793 (*chinense*)

Ayala, M.G. 232 (*annuum* var. glabriusculum)

Ayarde, H. & M. Sidán 299 (*chacoense*)

Aymard, G. 5108 (*annuum* var. annuum)

Aymard, G. et al. 473 (*frutescens*); 2721 (*annuum* var. glabriusculum)

Bacab W., G. 115 (*annuum* var. glabriusculum)

Bacigalupo, N.M. et al. 1136 (*chacoense*); 1163 (*baccatum* var. baccatum)

Báez, S. et al. 28 (*chinense*)

Badillo, V. 6379 (*frutescens*); 7159 (*rhomboideum*)

Bailetti, E. 207 (*eximium*)

Baitello, J.B. 547 (*villosum*)

Baizabal M., R.M. & M.G. Zola B. 11, 12, 13 (*annuum* var. annuum)

Baker, C.F. 148 (*annuum* var. glabriusculum)

Baksh, Y.S. 10 (*annuum* var. glabriusculum)

Balansa, B. 2079, 2079a (*rabenii*); 2107, 2110, 2120 (*flexuosum*); 3131 (*baccatum* var. baccatum); 4704 (*flexuosum*)

Balcazar, A. 74 (*geminifolium*)

Baldeón Malpartida, S.M. & J. Campos 5316 (*longifolium*)

Balderrama, J. 10 (*annuum* var. annuum)

Balegno, B. 1058, 1149 (*chacoense*)

Balick, M.J. 2211 (*chinense*)

Balick, M.J. et al. 1539 (*annuum* var. glabriusculum); 2272, 2391 (*frutescens*)

Balls, E. 7153 (*rhomboideum*)

Balslev, H. 4890, 4892, 4894, 4895, 4904 (*chinense*)

Balslev, H. & E. Asanza 4363 (*annuum* var. glabriusculum)

Balslev, H. & D. Irvine 4582 & 4598 (*chinense*)

Bamonte, A.G. 77, 79 (*annuum* var. annuum)

Bang, M. 200, 727 (*pubescens*); 1126 (*eximium*); 1185 (*pubescens*); 1474 (*baccatum* var. pendulum)

Banton, F.S. 6838 (*frutescens*)

Barbosa, C. 14060, 14924 (*dimorphum*)

Barbosa, M.R. et al. 2674, 2895, 3127 (*parvifolium*)

Barboza, G.E. 86 (*chacoense*); 164 (*baccatum* var. umbilicatum); 632, 638, 645 (*chacoense*); 795 (*frutescens*); 1123 (*baccatum* var. umbilicatum); 1793 (*chacoense*); 1805, 1807 (*baccatum* var. baccatum); 1901 (*chinense*); 1918 (*baccatum* var. umbilicatum); 1919 (*eximium*); 2064bis, 2271 (*frutescens*); 2431 bis (*baccatum* var. baccatum); 2504, 2505, 2506 (*chinense*); 3055 (*baccatum* var. baccatum); 4078, 4200, 4766, 4803, 4809 (*chacoense*); 4824 (*baccatum* var. pendulum); 4881, 4882, 4884 (*cardenasii*); 4885 (*eximium*); 4886 (*baccatum* var. pendulum); 4895, 4896, 4902, 4903 (*eximium*); 4907, 4908 (*caballeroi*); 4910 (*chacoense*); 4914 (*eximium*), 4927 (*neei*); 4975 (*baccatum* var. pendulum); 5038 (*baccatum* var. baccatum); 5041 (*hunzikerianum*); 5042, 5043 (*schottianum*); 5044 (*tovarii*); 5047 (*dimorphum*); 5163 (*baccatum* var. umbilicatum)

Barboza, G.E. & C. Carrizo García 3644 (*minutiflorum*); 3646 (*baccatum* var. baccatum); 3648 (*baccatum* var. umbilicatum); 3946 (*muticum*)

Barboza, G.E. & M.T. Cosa 2517 (*cornutum*); 2519 (*schottianum*); 2525 (*cornutum*); 2526 (*schottianum*); 2533 (*mirabile*)

Barboza, G.E. & R. Deanna 5000 (*recurvatum*); 5003 (*rabenii*); 5004 (*recurvatum*); 5006, 5007, 5009, 5014, 5015, 5017, 5019 (*schottianum*); 5020 (*rabenii*); 5021 (*flexuosum*); 5024 (*mirabile*); 5026 (*recurvatum*); 5026 bis (*villosum*); 5027 (*rabenii*); 5028 (*mirum*)

Barboza, G.E. & S. Leiva González 4819 (*geminifolium*); 4821 (*longifolium*); 4826 (*hookerianum*); 4829 (*frutescens*); 4831 (*hookerianum*); 4832 (*annuum* var. glabriusculum); 4841, 4842 (*piuranum*); 4845 (*geminifolium*); 4846, 4849, 4850, 4851 (*longifolium*); 4852 (*geminifolium*); 4854 (*rhomboideum*); 5053 (*dimorphum*);

Barboza, G.E. & X. Reyes 5040 (*neei*)

Barboza, G.E. et al. 415 (*baccatum* var. baccatum); 426 (*flexuosum*); 458 (*baccatum* var. baccatum); 905 (*flexuosum*); 915 (*recurvatum*); 927 (*baccatum* var. baccatum); 1027, 1034 (*flexuosum*); 1038 (*baccatum* var. pendulum); 1274 (*baccatum* var. baccatum); 1487 (*flexuosum*); 1622 (*chinense*); 1629, 1632 (*recurvatum*); 1638 (*schottianum*); 1640, 1646, 1646b, 1647 (*rabenii*); 1648, 1649 (*mirum*); 1650 (*schottianum*); 1651 (*pereirae*); 1653 (*villosum*); 1654 (*rabenii*); 1655 (*villosum*); 1656 (*rabenii*); 1657 (*flexuosum*); 1847 (*pubescens*);1849 (eximium×pubescens); 1860, 1905 (*eximium*); 1916 (*coccineum*); 1921 bis (*baccatum* var. baccatum); 2019 (*frutescens*); 2021 (*recurvatum*); 2022 (*frutescens*); 2023 (*recurvatum*); 2032, 2033, 2034, 2035, 2036, 2037 (*schottianum*); 2041 (*frutescens*); 2048 (*friburgense*); 2057 (*campylopodium*); 2058 (*mirabile*); 3419 (*baccatum* var. baccatum); 3543 (*eximium*); 3631 (*flexuosum*); 3632 (*mirabile*); 3633, 3634, 3635, 3636, 3637 (*schottianum*); 3655 (*caballeroi*); 3665 (*frutescens*); 4889, 4890 (*pubescens*); 4891 (*baccatum* var. pendulum); 4913 (*baccatum* var. baccatum); 4918 (*minutiflorum*); 4919 (*baccatum* var. baccatum); 4921 (*coccineum*); 4923 (*pubescens*); 4924, 4925, 4926 (eximium×pubescens); 5032 (*muticum*); 5049 (*annuum* var. glabriusculum); 5050 (*rhomboideum*)

Barkley, F.A. 6, 10, 262 (*dimorphum*); 13434 (*annuum* var. glabriusculum)

Barkley, F.A. & L.L. Arboleda R. 14 (*rhomboideum*)

Barkley, F.A. & C. Bostirronnois 39498 (*annuum* var. glabriusculum)

Barkley, F.A. & G. Gutiérrez V. 1765 (*annuum* var. annuum); 1776 (*annuum* var. glabriusculum)

Barkley, F.A. et al. 17C403 (*rhomboideum*); 17C405 (*annuum* var. glabriusculum)

Barlett, H.H. 10007 (*annuum* var. glabriusculum)

Barreto, K.D. et al. 980 (*baccatum* var. baccatum)

Barreto, V. et al. 218 (*caatingae*)

Barros, W.D. 728 (*villosum*)

Barth, O.M. I 214 (*villosum*)

Bartholomew, B. et al. 2719 (*annuum* var. glabriusculum)

Bartlett, H.H. 10806 (*rhomboideum*); 20417, 20435 (*chacoense*)

Bastión, E. 407 (*chacoense*); 681 (*eximium*); 779 (*chacoense*)

Bates, D. et al. 1481 (*annuum* var. glabriusculum)

Batista, E.R. et al. 129 (*recurvatum*)

Bautista, H.P. & G.C. Pinto 1023 (*caatingae*)

Beaman, J.H. 6014 (*lanceolatum*)

Becerra Gonzáles, E. & J. Perea 1152 (*annuum* var. glabriusculum)

Becerril Pérez, S. & N.L. Ortiz Cornejo 7 (*frutescens*)

Beck, H.T. et al. 87 (*annuum* var. glabriusculum); 90, 91 (*chinense*); 116 (*frutescens*)

Beck, S. 130 PG94 (*cardenasii*); 3051 (*ceratocalyx*); 4465 (*eximium*); 5362 (*frutescens*); 6255, 9789 (*eximium*); 13372 (*annuum* var. annuum); 17097 (*frutescens*); 17259 (*eximium*); 21356 (*baccatum* var. baccatum); 23451 (*minutiflorum*); 25261 (*eximium*); 28089 (*ceratocalyx*); 32066 (*baccatum* var. baccatum); 33135 (*ceratocalyx*); 33151 (*pubescens*)

Beck, S. & M. Liberman 16298 (*baccatum* var. baccatum)

Beck, S. et al. 19150 (*annuum* var. glabriusculum); 19317 (*chinense*); 22714 (*baccatum* var. baccatum)

Bedoya, M. 1, 2 (*annuum* var. glabriusculum); 5 (*lycianthoides*); 316 (*rhomboideum*)

Belinelo, R. 05/18 (*recurvatum*)

Belshaw, C.M. 3084 (*coccineum*); 3210 (*baccatum* var. pendulum)

Beltrán, G.D. et al. 24 (*rhomboideum*); 85, 87 (*lycianthoides*); 140 (*dimorphum*)

Benítez, B. & Anzotegui 76 (*baccatum* var. baccatum)

Benítez, S. 612, 688, 689, 724, 726, 732, 734, 735, 738, 744 (*annuum* var. annuum)

Benítez, S. et al. 386, 387 (*annuum* var. annuum)

Benítez B., B. 585 (*flexuosum*)

Benítez de Rojas, C. 13, 298 (*frutescens*); 330 (*rhomboideum*); 379 (*frutescens*); 970 (*rhomboideum*)

Benítez de Rojas, C. & M.J. Baldizán 4976, 4980 (*rhomboideum*)

Benítez de Rojas, C. & F. Rojas 4202 (*parvifolium*); 4786 (*rhomboideum*)

Benítez de Rojas, C. et al. 4821, 4898 (*rhomboideum*)

Bennett, B.C. & P. Gomez Andrade 3776 (*frutescens*); 3441 (*chinense*)

Bennet, B. et al. 4276 (*chinense*)

Benoist, M.R. 3478, 3580 (*rhomboideum*); 4204 (*benoistii*)

Bensman, R. 310 (*chinense*); 480 (*lycianthoides*)

Bentancur, J. 11063, 11315 (*rhomboideum*)

Bentley, P.S. 312, 312-a, 312-b, 456 (*frutescens*)

Berlandier, J.L. 97, 152, 1863, 1907, 3045 (*annuum* var. glabriusculum)

Berlin, B. 1572 (*annuum* var. annuum); 2016 (*annuum* var. glabriusculum)

Bermúdez, L.A. 34950 (*rhomboideum*)

Bermúdez, L.A. & F.A. Barkley 17C889 (*annuum* var. glabriusculum)

Bernacci, L.C. et al. 957 (*recurvatum*); 1449 (*rabenii*); 2816 (*baccatum* var. umbilicatum); 2820, 4467 (*rabenii*); 21468 (*flexuosum*)

Bernal, C. 642 (*rhomboideum*)

Bernal, C. & A. Pico 350 (*rhomboideum*)

Bernal, C. et al. 1098 (*frutescens*)

Bernal, R. 1828 (*dimorphum*)

Bernardello, G. 471 (*chacoense*); 932 (*baccatum* var. baccatum)

Bernardello, G. & L. Galetto 726 (*chacoense*)

Bernardello, G. et al. 498, 525 (*chacoense*)

Bernardi, L. 6105 (*annuum* var. glabriusculum)

Bernoulli, K.G. & O.R. Cario 2339 (*lanceolatum*); 2344, 2378, 2395 (*annuum* var. glabriusculum)

Berry, P.E. 1044, 1341 (*rhomboideum*)

Berti, H. & M. Escalante 431 (*chacoense*)

Bertoni, J.E. 400 (*flexuosum*)

Betancur, J. et al. 352 (*annuum* var. glabriusculum); 3417 (*geminifolium*); 6187 (*dimorphum*); 10158, 11258 (*annuum* var. glabriusculum); 11615 (*frutescens*)

Bianchetti, L. & P.G. Bustamante 1518 (*mirabile*)

Bianchetti, L. & G. Pereira Silva 978 (*annuum* var. glabriusculum)

Bianchetti, L. et al. 330, 331, 332, 335, 346, 347, 348, 349, 350, 351, 352, 354, 359, 363, 366, 379, 380, 381, 382, 383, 384 (*mirabile*); 391 (*friburgense*); 393 (*friburgense*); 394 (*mirabile*); 399, 400 (*campylopodium*); 406, 407, 408 (*muticum*); 409, 410, 411, 415, 416 (*schottianum*); 417, 418, 420, 422, 425, 429, 430, 431, 432, 433 (*villosum*); 435 (*rabenii*); 438, 439 (*schottianum*); 444 (*villosum*); 445, 446, 456, 457, 458, 459, 461, 462, 464, 473 (*recurvatum*); 476, 480, 481 (*schottianum*); 487 (*villosum*); 489 (*schottianum*); 490 (*recurvatum*); 491, 492, 493 (*cornutum*); 495, 496 (*mirabile*); 499, 500, 501 (*schottianum*); 504, 505 (*rabenii*); 510 (*mirabile*); 511 (*campylopodium*); 512 (*carassense*); 1270, 1273 (*pereirae*); 1299 (*friburgense*); 1333 (*cornutum*); 1346, 1347, 1348, 1349, 1350 (*pereirae*); 1363, 1364, 1367, 1368 (*carassense*); 1517, 1520, 1523 (*recurvatum*); 1524 (*rabenii*); 1526, 1527 (*recurvatum*); 1533, 1534 (*flexuosum*); 1535 (*schottianum*); 1537 (*hunzikerianum*); 1538 (*mirabile*); 1539 (*villosum*); 1540 (*schottianum*); 1542 (*cornutum*); 1543 (*villosum*); 1544, 1545 (*schottianum*); 1546 (*recurvatum*); 1549 (*villosum*); 1550, 1551 (*mirabile*); 1552 (*flexuosum*); 1553 (*rabenii*); 1554 (*mirabile*); 1555 (*rabenii*); 1556 (*mirabile*); 1557 (*villosum*);1558 (*pereirae*); 1559 (*mirabile*); 1560 (*caatingae*); 1561 (*schottianum*); 1562 (*campylopodium*); 1563 (*schottianum*); 1564 (*mirabile*); 1565 (*friburgense*); 1566 (*campylopodium*); 1567 (*pereirae*); 1568 (*mirabile*)

Bianchi, S. & C. Camardelli 951 (*chacoense*)

Bianco, C.A. 651, 1652, 3216, 3465 (*chacoense*)

Biganzoli, F. et al. 544 (*flexuosum*)

Biggs, N. et al. 38 (*baccatum* var. baccatum); 118 (*eximium*)

Billiet, F. 3183 (*chacoense*)

Bishop, A. & B. Holst CC0048, CC0053 (*annuum* var. glabriusculum)

Bittar, M. & J. Bessa 90 (*villosum*)

Biurrum, F. & E. Pagliari 3271 pp (*chacoense*)

Blanco-Macías, A. 1559 (*annuum* var. glabriusculum)

Blanchet, J.S. 2823 (*parvifolium*); 3098A (*annuum* var. glabriusculum)

Blanchet, M. 90 (*chinense*)

Bocayuva, M. & A. Calvente 91 (*campylopodium*)

Boeke, J.D. 2327 (*lycianthoides*)

Boeke, J.D. & S. Boeke 3027 (*pubescens*)

Bohs 2026, 2190, 3446 (*rhomboideum*)

Bohs, L. & M. Nee 2754 (*coccineum*)

Bohs, L. & C. Vogt 3167, 3171 (*flexuosum*)

Bohs, L. et al. et al. 2823 (*minutiflorum*); 3346, 3401, 3421 (*geminifolium*); 3929 (*annuum* var. glabriusculum)

Bolotin, D. 21 (*chinense*)

Bonifaz, C. & X. Cornejo 3126, 3249 (*lycianthoides*); 4158 (*annuum* var. annuum)

Bonilla Baes, R. & E. Monsalvo G. 346 (*pubescens*)

Bono, J. 5167, 6707 (*rhomboideum*); 6896 (*frutescens*)

Boom, B.M. & C. Brewer-Carias 10105 (*annuum* var. glabriusculum)

Boom, B.M. et al. 11158 (*annuum* var. glabriusculum)

Bordas, E. & G. Schmeda 4024 (*baccatum* var. baccatum)

Borges, R. 65 (*frutescens*); 66 (*baccatum* var. pendulum); 67 (*chinense*)

Borges, R.A.X. et al. 298 (*mirabile*); 517, 525 (*caatingae*); 707 (*mirabile*)

Bornmüller, A. 585 (*flexuosum*)

Bortolotto, I..M. & D.P. Rodrigues B-615 (*frutescens*)

Botta, S. & F. Ruiz de Angulo 115 (*chacoense*)

Botteri, M. 904 (*frutescens*); 1855 (*annuum* var. glabriusculum)

Bourdy, G. 2000 (*chacoense*)

Bovini, M.G. 1119 (*recurvatum*); 1142 (*rabenii*); 1211 (*campylopodium*);1234 (*rabenii*)

Bovini, M.G. & J.M. Braga 1566 (*campylopodium*)

Bovini, M.G. & L.C.S. Giordano 363 (*cornutum*)

Bovini, M.G. & C.M. Mynssen 2729 (*campylopodium*)

Bovini, M.G. et al. 695, 959 (*schottianum*); 1721 (*villosum*); 1993 (*schottianum*); 2100 (*campylopodium*); 2605 (*mirabile*); 4229 (*friburgense*)

Bowman, R.I. 63 (*frutescens*)

Box, H.E. 1058 (*annuum* var. glabriusculum)

Boyle, B. et al. 5020 (*annuum* var. glabriusculum)

Bradburn, A. & S. Darwin 1272 (*annuum* var. glabriusculum)

Brade, A.C. 12668, 15095 (*cornutum*); 17263, 17463 (*villosum*)

Brade, A.C. et al. 19148 (*campylopodium*)

Braem, S.W. NC0026 (*annuum* var. glabriusculum)

Braga, J.M.A. 3973 (*recurvatum*); 4361 (*campylopodium*)

Braga, J.M.A. et al. 867 (*schottianum*);1613, 2005, 2134, 3076, 3730, 3801, 3898 (*villosum*); 7273 (*campylopodium*)

Braga, P.I.S. 2441 (*cornutum*)

Braga, R. 1536 (*baccatum* var. baccatum)

Brand, J. & M. González 604 (*annuum* var. glabriusculum)

Brandegee, J. 2294 (*rhomboideum*)

Brandbyge, J. & E. Asanza 32813 (*chinense*)

Brandegee, T.S. 414 (*annuum* var. glabriusculum)

Bravo V., E. 615 (*rhomboideum*)

Breckon, G. 5574 (*annuum* var. glabriusculum)

Breedlove, D.E. 7155-A (*annuum* var. annuum); 10371 (*annuum* var. glabriusculum); 10591 (*rhomboideum*); 10689 (*frutescens*); 11248, 11249, 11716 (*annuum* var. annuum); 11805 (*annuum* var. glabriusculum); 11696 (*pubescens*); 16083 (*annuum* var. annuum); 19922 (*frutescens*); 20370 (*annuum* var. glabriusculum); 25238, 25667 (*rhomboideum*); 26097 (*lanceolatum*); 26362 (*rhomboideum*); 26482 (*annuum* var. glabriusculum); 26518 (*annuum* var. annuum); 26889, 28050, 33849 (*annuum* var. glabriusculum); 34836, 35237 (*lanceolatum*); 36628, 36682 (*annuum* var. glabriusculum); 36720 (*rhomboideum*); 37442 (*annuum* var. glabriusculum); 38789 (*lanceolatum*); 39818, 40592 (*rhomboideum*); 41082 (*lanceolatum*); . 44367 (*rhomboideum*); 48935 (*annuum* var. glabriusculum); 51520 (*rhomboideum*); 52096 (*annuum* var. glabriusculum); 53338, 56499, 68656 (*lanceolatum*); 69942, 70605, 70888 (*rhomboideum*)

Breedlove, D.E. & B. Anderson 62936 (*annuum* var. glabriusculum)

Breedlove, D.E. & P.H. Raven 13239 (*annuum* var. glabriusculum)

Breedlove, D.E. & A.R. Smith 22629, 31992 (*lanceolatum*)

Breedlove, D.E. & R.F. Thorne 31031(*lanceolatum*)

Brenckle, J.F. 47-418 (*frutescens*)

Breteler, F.J. 3207, 4059 (*rhomboideum*)

Briones V., O.L. 1866 (*annuum* var. glabriusculum)

Bristol, M.L. 702 (*frutescens*); 1115, 1335 (*pubescens*)

Britton, E.G. 2942 (*annuum* var. glabriusculum)

Britton, N.L. 350, 379, 1007, 6242 (*annuum* var. glabriusculum)

Britton, N.L. & L.J. Brace 660 (*annuum* var. glabriusculum)

Britton, N.L. & E.G. Britton 7344, 9848 (*annuum* var. glabriusculum)

Britton, N.L. & J.F. Cowell 9965 (*annuum* var. glabriusculum)

Britton, N.L. & A. Hollick 1829 (*annuum* var. glabriusculum)

Britton, N.L. & C.F. Millspaugh 3014, 5450, 6234 (*annuum* var. glabriusculum)

Britton, N.L. & W.M. Wheeler 231 (*annuum* var. glabriusculum)

Britton, N.L. & P. Wilson 256 (*annuum* var. glabriusculum)

Britton, N.L. et al. 112, 1785, 12444, 15299 (*annuum* var. glabriusculum)

Brizuela, A. 343, 627, 664, 821, 837, 936 (*chacoense*)

Brizuela, J. 70, 196, 229, 338, 410, 475, 875, 1009, 1066, 1127, 1174, 1217, 1225, 1284, 1312, 1609 (*chacoense*)

Broadway, W.E. 366, 4514-Ser. I, 4563 (*annuum* var. glabriusculum); 4498 (*frutescens*);

Brockmann, J. 103 (*annuum* var. glabriusculum)

Brooke, M.A. 5934 (*eximium*)

Brosehel, M. & J. Craig 406 (*carassense*)

Brown, S. 93 (*annuum* var. glabriusculum)

Brumbach, W.C. 7357, 7750, 7754, 8915, 8917 (*annuum* var. glabriusculum)

Brunner, D. 1569, 1692, 1741 (*chacoense*); 1804 (*flexuosum*)

Brunner, D. et al. 913 (*flexuosum*)

Bryant, P.T. & G. Bryant 94 (*annuum* var. glabriusculum)

Buchinger, M. & Rodríguez 3222 (*flexuosum*)

Buchtien, O. 337 (*baccatum* var. pendulum); 338 (*pubescens*); 2231 (*minutiflorum*); 3262 (*eximium*); 4033 (*pubescens*); 4698 (*baccatum* var. pendulum); 5542 (*pubescens*)

Buendia S., M.C. 2 (*annuum* var. glabriusculum)

Bueno, E.A. 47 (*campylopodium*)

Bueno, O. et al. 628 (*flexuosum*)

Búgola-Silva, F. 8041 (*schottianum*)

Bullock, S.H. 1231, 1937 (*annuum* var. glabriusculum)

Bunch et al., P. 685 (*annuum* var. glabriusculum)

Bünger, M.O. & M.C. Souza 635 (*campylopodium*)

Bunting, G.S. 8159 (*annuum* var. glabriusculum)

Burandt, C. et al. 27V (*annuum* var. glabriusculum); 193 (*parvifolium*)

Burandt Jr. V., C. 1003 (*rhomboideum*)

Buratovich, F. 117, 152, 303, 314, 396, 413, 477, 580, 606 (*chacoense*)

Burch, D. 2335 (*annuum* var. glabriusculum)

Burch, D. & M. Bialek 6839 (*annuum* var. annuum)

Burch, D. & N. Chevalier 3709, 3716 (*annuum* var. annuum)

Burch, D. & T. Doughty 6840 (*annuum* var. annuum)

Burch, D. & R.J. Machwart 3714 (*annuum* var. annuum)

Burch, D. & L. Schmoll 6838 (*annuum* var. annuum)

Burchell, W.J. 1319 (*baccatum* var. baccatum); 1913 (*campylopodium*); 2428 (*schottianum*); 2443 (*recurvatum*); 3460 (*rabenii*); 3472 (*recurvatum*); 3705 (*cornutum*)

Burkart, A. 7752, 12553, 13361, 13972, 20300, 21384, 22299, 23492, 25459, 26746 (*chacoense*)

Burkart, A. et al. 28424 (*annuum* var. annuum)

Búrquez, A. & E. Búrquez 548 (*annuum* var. glabriusculum)

Búrquez, A. et al. 638 (*annuum* var. glabriusculum)

Bush, B.F. 1205 (*annuum* var. glabriusculum)

Bye, R.A. et al. 13 (*pubescens*); 89 (*chinense*); QD 228, QD 246 (*annuum* var. annuum); 1875, 16386 (*annuum* var. glabriusculum)

Caballero Pardo, R. 40 (*frutescens*); 103 (*eximium*)

Caballero Serrano, V. 006 (*chinense*)

Cabrera 3365, 3366 (*chinense*)

Cabrera C., E.F. & H. de Cabrera 2939, 5819 (*lanceolatum*)

Cabrera, A.L. & A.A. Sáenz 29195 (*flexuosum*)

Cabrera, A.L. & J.M. Marchionni 12781 (*chacoense*)

Cabrera, A.L. et al. 21831, 23353 (*chacoense*); 23364, 31487 (*baccatum* var. baccatum); 31896 (*chacoense*)

Cabrera, E. & H. de Cabrera 3130, 6458, 7021, 7139, 8787, 8990, 9076, 9115, 9147, 9560, 9602, 9654, 10056, 10269, 11512, 11748, 13073, 13858, 14056, 14277, 14422 (*annuum* var. glabriusculum)

Cabrera, E. & O. Canul 5513 (*annuum* var. annuum)

Cabrera, E. & L. Cortéz 208, 427 (*annuum* var. glabriusculum)

Cabrera, E. & R. Torres 938 (*annuum* var. glabriusculum)

Cabrera, E. et al. 3001, 4578, 5643, 8523 (*annuum* var. glabriusculum)

Cabrera, I. 36 (*annuum* var. glabriusculum)

Cabrera, O. et al. 291 (*dimorphum*)

Cáceres, S. et al. 343 (*baccatum* var. baccatum)

Cadena-M., J.J. et al. 143 (*dimorphum*)

Caity, G. 190 (*annuum* var. annuum)

Calderón, S. 523 (*annuum* var. annuum); 1197 (*annuum* var. glabriusculum); 1198 (*frutescens*); 1658 (*annuum* var. glabriusculum)

Calderón, Y. 123 (*annuum* var. glabriusculum)

Calle A., J. et al. 70 (*rhomboideum*)

Callejas, R. 3151 (*dimorphum*)

Callejas, R. et al. 6373 (*dimorphum*)

Calónico S., J. 22503 (*annuum* var. glabriusculum)

Calónico S., J. et al. 5810, 21404 (*annuum* var. glabriusculum)

Calzada, J.I. 2367, 2368 (*annuum* var. annuum); 2369 (*annuum* var. glabriusculum); 04954 (*annuum* var. annuum); 5577, 10493 (*annuum* var. glabriusculum); 10856 (*pubescens*); 12646 (*lanceolatum*); 12662 (*frutescens*); 14823 (*annuum* var. annuum); 19320 (*annuum* var. glabriusculum)

Calzada, J.I. & H.V. Der Well 15483 (*annuum* var. annuum)

Calzada, J.I. et al. 3774 (*annuum* var. annuum); 3990, 9679 (*annuum* var. glabriusculum)

Calzada, J.J. 21036, 22605 (*rhomboideum*)

Calzadilla, E. et al. 111 (*caballeroi*)

Calzadilla, J.J. 2136, 2704 (*rhomboideum*)

Camargo, L.A. 7781 (*dimorphum*)

Cambar, I. 173 (*rhomboideum*)

Camp, W.H. E-1739 (*pubescens*); E-2373 (*rhomboideum*); E-3366, E-4312 (*geminifolium*); E-5031 (*pubescens*);

Campos de la Cruz, J. 4833 (*annuum* var. glabriusculum)

Campos de la Cruz, J. & L. Campos 5988 (*rhomboideum*)

Campos de la Cruz, J. & S. Corrales 3603 (*geminifolium*)

Campos de la Cruz J. & O. Díaz 2293 (*annuum* var. glabriusculum); 2333 (*annuum* var. annuum); 2339 (*frutescens*); 2486 (*annuum* var. glabriusculum)

Campos de la Cruz, J. & S. Nuñez 4266 (*pubescens*)

Campos de la Cruz, J. & W. Vargas 6945, 6947, 6981 (*chinense*)

Campos de la Cruz, J. et al. 3031 (*geminifolium*); 3732 (*rhomboideum*); 6607 (*longifolium*)

Campos V., A. 1176, 3203 (*rhomboideum*); 4107 (*annuum* var. glabriusculum)

Cândido, E.S. et al. 178, 290, 298 (*villosum*); 317, 320, 333 (*mirabile*); 404 (*villosum*); 750 (*mirabile*)

Cano, A. 374 (*annuum* var. annuum); 2278 (*geminifolium*)

Cantero, J.J. 6385, 6423, 6976, 7102, 7311 (*chacoense*)

Cantino, P. 698 (*chacoense*)

Capparelli, A. 158 (*chacoense*)

Carabia, J.P. 3633 (*annuum* var. glabriusculum)

Caranqui, J. et al. 517 (*lycianthoides*)

Carauta, P.P. & P. Maas 2730 (*campylopodium*)

Carbonó, E. 4128 (*rhomboideum*)

Cárdenas, D. 9325, 9328, 24632 (*frutescens*)

Cárdenas, D. et al. 9300 (*frutescens*); 9306 (*annuum* var. annuum); 9307 (*frutescens*); 9308 (*chinense*); 9310 (*annuum* var. annuum); 9311 (*chinense*); 9312 (*annuum* var. annuum); 9317 (*chinense*); 9319 (*annuum* var. annuum); 9331 (*chinense*); 9354 (*frutescens*); 9356 (*chinense*); 9360 (*annuum* var. annuum); 9361 (*chinense*); 9362 (*frutescens*); 9377 (*chinense*); 9380 (*annuum* var. annuum); 9384 (*frutescens*); 9385, 9393, (*chinense*); 9394 (*frutescens*); 9401, 9403 (*baccatum* var. baccatum); 9405 (*frutescens*); 9406 (*annuum* var. annuum); 9412, 9413 (*frutescens*); 9423 (*baccatum* var. baccatum); 9424 (*annuum* var. annuum); 9431 (*chinense*); 9434 (*frutescens*); 9438 (*annuum* var. annuum); 9439 (*chinense*); 9441 (*frutescens*)

Cárdenas, L. 3936 (*rhomboideum*)

Cárdenas, M. 2270, 4237 (*eximium*); 4701 (*baccatum* var. baccatum); 5114 (*eximium*); 5565 (*baccatum* var. pendulum)

Cárdenas R., R. PU. 0558 (*annuum* var. annuum)

Cardona, F.A. & H. David 1224 (*dimorphum*)

Cardoso, D. 625 (*caatingae*)

Cardoso, D. & R.M. Santos 1922 (*parvifolium*)

Cardoso, J. 150 (*mirabile*)

Cardoso, L.J.T. et al. 1130 (*recurvatum*)

Cardozo, A. et al. 231 (*baccatum* var. baccatum)

Carette, E. 28 (*chacoense*)

Carmo, F.F. & L.C. Ribeiro 3527 (*carassense*)

Carneiro, E.M. 371 (*caatingae*)

Carneiro-Torres, D.S. et al. 717 (*caatingae*)

Carnevali, G. et al. 531 (*parvifolium*)

Carnevali, R. 5290 (*chacoense*)

Caro, J.A. 1694 (*chacoense*)

Carranza González, E. 3266 (*annuum* var. glabriusculum)

Carranza, E. 736, 766, 772, 937, 2455, 3212 (*rhomboideum*)

Carranza, E. & S. Zamudio 5519 (*rhomboideum*)

Carrasco, J.A. et al. 147 (*caballeroi*)

Carrasquilla, L. et al. 281 (*chinense*)

Carrasquillo, J.A. 128 (*annuum* var. glabriusculum)

Carretero, A. et al. 824, 939, 998, 1067, 1085 (*neei*)

Carrillo, L. & D. Reyes 434, 591 (*chinense*)

Carrizo García, C. 61, 62 (*minutiflorum*); 63 (*baccatum* var. baccatum); 64 (*minutiflorum*); 67 (*eshbaughii*); 73 (*lanceolatum*); 84 (*flexuosum*); 101 (*baccatum* var. umbilicatum), 102 (*annuum* var. glabriusculum)

Carter, A. 4902, 4921 (*annuum* var. glabriusculum)

Carter, A. & R. Morán 5365 (*annuum* var. glabriusculum)

Carvajal, J. 1 (*annuum* var. glabriusculum)

Carvajalino & Díaz 11 (*annuum* var. glabriusculum)

Carvalho, B. et al. 24 (*pereirae*)

Carvalho, D.N. 283, 308, 515, 582 (*caatingae*)

Carvalho, D.N. & M.O. Matos 188 (*caatingae*)

Carvalho-Sobrinho, J.G. et al. 2518 (*parvifolium*)

Casadiego, J. 248 (*rhomboideum*)

Cascante, J. 6 (*pubescens*)

Castañeda, M.E. 79 (*rhomboideum*)

Castañeda Vega, R. 72 (*chacoense*)

Castellanos, A. 54, 212 (*lycianthoides*); 217 (*chacoense*); 239 (*lycianthoides*); 24386 (*campylopodium*)

Castiglioni, J.A. & A.E. Ragonese 7042, 7109, 8027 (*chacoense*)

Castiglioni et al. 7324 (*chacoense*)

Castillo, A. 1079 (*frutescens*)

Castillo C., G. 207 (*annuum* var. glabriusculum); 214 (*pubescens*); 4136, 9327 (*annuum* var. glabriusculum); 1757 (*pubescens*); 14287, 18139, 18164 (*annuum* var. glabriusculum)

Castillo, D. et al. 029 (*annuum* var. annuum)

Castillo, J. et al. 165 (*rhomboideum*)

Castillo Campos, G. et al. 19219 (*rhomboideum*)

Castillo Mont, J.J. 2235 (*rhomboideum*)

Castillón, L. 24, 3568, 14331 (*chacoense*)

Castro, M. & A. Magallanes 39 (*parvifolium*)

Castro, R.M. et al. 1229 (*caatingae*)

Castro, S. & N. Andoke 597, 606 (*frutescens*); 607 (*annuum* var. glabriusculum); 608 (*frutescens*)

Castro, S. & C. Bernal 32 (*dimorphum*)

Castro, S. & I. Matapí 561 (*chinense*); 562 (*frutescens*); 563, 564, 565 (*chinense*)

Castro, S. & R. Matapí 523 (*annuum* var. glabriusculum)

Castro, S. & A. Rodríguez 132, 307, 308, 309, 310, 311, 312, 313, 314 (*chinense*)

Castro, S. & Virgelina 305, 307 (*annuum* var. annuum)

Castro, S. et al. 238 (*annuum* var. annuum)

Catharino, E.L. 720 (*rabenii*)

Caxambu, M.G. & E.L. Siqueira 2890 (*flexuosum*)

Caxambu, M.G. et al. 5146, 5902 (*flexuosum*)

Cayola, L. et al. 42, 127 (*baccatum* var. baccatum)

Cedillo Trigos, R. & R. Torres C. 2309 (*rhomboideum*)

Cedillo Trigos, R. et al. 794 (*rhomboideum*)

Cerana, M.M. 823 (*baccatum* var. baccatum)

Cerón, C.E. 194 (*frutescens*); 11092 (*rhomboideum*); 14561 (*frutescens*); 15237, 15577 (*pubescens*); 15714 (*rhomboideum*); 18324 (*lycianthoides*); 18842 (*annuum* var. annuum); 18856 (*rhomboideum*); 20848 (*chinense*)

Cerón, C.E. & M. Cerón 4712 (*chinense*)

Cerón, C.E. & C. Reyes 62013 (*chinense*)

Cerón, C.E. & M. Montesdeoca 12519, 13138, 13171 (*rhomboideum*)

Cerón, C.E. & 3er Curso Biología U. Central 6953 (*rhomboideum*)

Cerón, C.E. et al. 1292, 1299, 5849 (*chinense*); 18258 (*hookerianum*)

Cerrate de Ferreyra, E. 7644, 7673 (*pubescens*)

Cervi, A.C. & F.C. Silva 2039 (*recurvatum*)

Chagas-Mota, E.C. & L.M. Leão 3144 (*caatingae*)

Chagas-Mota, E.C. 828, 4711, 4828, 6286, 7450, 7578, 8184, 9419 (*caatingae*)

Chagas-Mota, E.C. et al. 11047 (*caatingae*)

Chan, C. 4149 (*frutescens*); 7144 (*annuum* var. glabriusculum)

Chan, C. et al. 30, 253, 1406 (*annuum* var. glabriusculum)

Chandler, H. 7044 (*annuum* var. glabriusculum)

Charpin, A. & L. Ramella 21481 (*baccatum* var. baccatum)

Chase, V.H. 7075 (*rhomboideum*)

Chater, A.O. 41 (*annuum* var. glabriusculum)

Chautems, A. et al. 165 (*parvifolium*)

Chavarría, U. 16 (*annuum* var. glabriusculum)

Chaves, E.M.F. et al. 182 (*parvifolium*)

Cháves, J.L. 235 (*annuum* var. glabriusculum)

Chazaro B., M. 2558 (*pubescens*)

Chazaro B., M. et al. 5164 (*annuum* var. glabriusculum)

Chevez, S. 40 (*annuum* var. annuum)

Chiang, F. 155, 209 (*annuum* var. glabriusculum)

Chiarini, F. & G. Wahlert 875 (*flexuosum*)

Chiarini, F. 758 (*chacoense*); 545 (*annuum* var. annuum); 1223 (*baccatum* var. baccatum)

Chicharo, D. 278 (*annuum* var. glabriusculum)

Chiea, S.A.C. 223 (*cornutum*)

Chodat, R.H. 47 (*flexuosum*)

Christopher, D.K. et al. 64 (*annuum* var. glabriusculum)

Chuck, L. 137 (*pubescens*)

Clark, J.L. & C. Aulestia 12193 (*geminifolium*)

Clark, J.L. et al. 3831 (*hookerianum*); 5337 (*regale*); 7671 (*lycianthoides*); 9403 (*regale*); 9806 (*geminifolium*); 9965 (*dimorphum*)

Clark, O.M. 7135 (*rhomboideum*)

Clase, T. et al. 4656 (*annuum* var. glabriusculum)

Cleef, A.M. et al. 11091 (*rhomboideum*)

Clewell, A.F. & F.C. Craighead 543 (*annuum* var. glabriusculum)

Cocucci, A.A. 248, 477, 978, 4966 (*chacoense*)

Cocucci, A.A. & A. Sérsic 5469b, 5471 (*baccatum* var. baccatum); 5488, 5877, 5939 (*chacoense*)

Cocucci, A.A. et al. 973 (*chacoense*); 5258, 5262, 5263 (*baccatum* var. baccatum); 5969, 5977 (*chacoense*); 5992, 5996 (*baccatum* var. baccatum); 6000 (*chacoense*)

Cocucci, A.E. 77 (*chacoense*)

Colinvaux, P.A. 440 (*frutescens*)

Collares, J.E. 45 (*schottianum*)

Colonnello, G. 522 (*annuum* var. glabriusculum)

Combs, R. 149, 151 (*annuum* var. glabriusculum)

Commerson, P. 160 (*rabenii*)

Conceição, S.F. et al. 507 (*longidentatum*)

Conde, B.E. 186 (*rabenii*)

Conrad, J. & W. Dietrich 2041 (*recurvatum*)

Contreras, E. 48, 294, 7684 (*annuum* var. glabriusculum)

Conzatti, C. & V. González 1221 (*rhomboideum*)

Cordero-P., Z. 683 (*annuum* var. annuum)

Correa N., M. 006 (*annuum* var. annuum)

Cook, O.F. & G.B. Gilbert 1017 (*pubescens*); 1165 (*geminifolium*)

Coons, M.P. 77-162, 77-181, 77-182 (*rabenii*)

Cooper, G.P. 115, 177 (*annuum* var. glabriusculum)

Core, E.L. 1105 (*dimorphum*)

Cornejo S., X. 421 (*lycianthoides*); 1727 (*hookerianum*)

Cornejo S., X. & C. Bonifaz 1353, 1778 (*hookerianum*); 2584 (*frutescens*); 2958, 5009 (*hookerianum*)

Coro, M. 1016, 1126 (*baccatum* var. baccatum)

Coronado G., I. & I. Velázquez 24, 149 (*rhomboideum*)

Coronado G., I. et al. 5331 (*rhomboideum*)

Coro-Rojas 1534 (*chacoense*)

Correa A., M.D. 1124 (*dimorphum*)

Correa M. et al. 3982 (*annuum* var. glabriusculum)

Corrêa, A.M. et al. 93 (*recurvatum*)

Corrêa Gomes, J. & F. Mattos 1029 (*flexuosum*)

Correa Múnera, M.A. 858 (*dimorphum*)

Corredor, C. & E. Moncada 07 (*annuum* var. glabriusculum)

Correll, D.S. 5814, 14847, 41078, 43340, 43702, 45127, 45621, 48675, 49299 (*annuum* var. glabriusculum)

Correll, D.S. & V.L. Cory 14104 (*annuum* var. glabriusculum)

Correll, D.S. et al. 41913 (*annuum* var. glabriusculum)

Cortéz, A. 156 (*chacoense*)

Cortés, L. & R. Cortés C. 30 (*lanceolatum*)

Cortés, R. et al. 2432 (*dimorphum*)

Cortés-Vázquez, M.E. 26, 143, 552 (*annuum* var. glabriusculum)

Cory, V.L. 51407 (*annuum* var. glabriusculum)

Cosa, M.T. 50, 342, 399 (*chacoense*)

Cosentino, K. 98, 99 (*frutescens*)

Costa, A. 367, 405 (*caatingae*); 4882 (*flexuosum*)

Costa, A. & Viegas 4904 (*schottianum*)

Costa, C.B. et al. 494 (*mirabile*)

Costa, C.N.G. et al. 1650 (*flexuosum*)

Costa, G. & A.C.S. Moraes 1744 (*longidentatum*)

Costa, G. et al. 2617 (*caatingae*)

Costa, J.A. 61 (*caatingae*)

Costa, K.C. & M.J. Rodal 5 (*caatingae*)

Costa, K.C. et al. 91 (*caatingae*)

Costa, S.M. 606 (*caatingae*)

Coulleri, J.P. & S.M. Ferrucci 338 (*chacoense*)

Coulter, T. 1237 (*rhomboideum*)

Couto, A.P.L. et al. 89, 106 (*caatingae*)

Covas, G. 1184 (*chacoense*)

Cowan, C. 1722 (*annuum* var. glabriusculum); 1805 (*chinense*); 2352 (*annuum* var. glabriusculum)

Crepaldi, M.O. 59 (*baccatum* var. baccatum)

Croat, T.B. 21043 (*coccineum*); 23892, 39814 (*annuum* var. glabriusculum); 40738 (*lanceolatum*); 41934 (*rhomboideum*); 43653 (*annuum* var. glabriusculum); 49390, 50879(*lycianthoides*); 55349 (*rhomboideum*); 55459 (*dimorphum*); 55803 (*geminifolium*); 57028 (*lycianthoides*); 58309, 70550 (*annuum* var. glabriusculum); 70657 (*lycianthoides*); 70847 (*annuum* var. glabriusculum); 73052 (*frutescens*); 91102 (*dimorphum*); 96081, 101027, 104378 (*lycianthoides*)

Croat, T.B. & G. Ferry 93747 (*geminifolium*)

Croat, T.B. & J.F. Gaskin 80291 (*lycianthoides*)

Croat, T.B. & D.P. Hannon 62887 (*annuum* var. glabriusculum); 62946 (*rhomboideum*); 62956 (*annuum* var. glabriusculum); 62988 (*rhomboideum*); 62992, 63278 A, 64555 (*annuum* var. glabriusculum); 92746 (*lycianthoides*)

Croat, T.B. & J. Whitehill 82726 (*lycianthoides*)

Croat, T.B. et al. 93341, 93366, 95685, 96413, 99984 (*lycianthoides*)

Croft, M.B. 179 (*annuum* var. glabriusculum)

Crosby, M.R. & C.A. Crosby 4694 (*frutescens*)

Crosby, M.R. et al. 119 (*annuum* var. glabriusculum)

Crovetto, M. 5581, 5589 (*baccatum* var. baccatum)

Cruz, J. 27 (*campylopodium*)

Cruz D., R. & A. Reyes 5639, 5668 (*annuum* var. glabriusculum); 5674 (*annuum* var. annuum)

Cruz R., G. 92 (*rhomboideum*)

Cruz, S. 003, 004, 005, 007, 009, 011 (*annuum* var. annuum)

Cuadros V., H. & A.H. Gentry 3060 (*annuum* var. glabriusculum)

Cuadros V., H. 501 (*annuum* var. glabriusculum); 774 (*rhomboideum*); 4344, 4475 (*annuum* var. glabriusculum)

Cuatrecasas, J. 173 (*pubescens*); 3343, 3614 (*annuum* var. glabriusculum); 8254 (*rhomboideum*); 8456 (*geminifolium*); 8572, 8655 (*dimorphum*), 10519, 12926 (*annuum* var. glabriusculum); 14479 (*chinense*);14514 (*annuum* var. glabriusculum); 15354 (*rhomboideum*); 17773 (*annuum* var. glabriusculum); 17782 a, 19436 (*rhomboideum*); 19637 (*annuum* var. glabriusculum); 19689 (*frutescens*); 20456 (*rhomboideum*); 21664, 22216 (*dimorphum*); 22375, 22665 (*lycianthoides*); 22800 (*annuum* var. glabriusculum); 23690 (*rhomboideum*); 23996 (*lycianthoides*)

Cuatrecasas, J. & H. Cuadros 28926 (*annuum* var. glabriusculum)

Cuatrecasas, J. & H. García Barriga 3519 (*baccatum* var. pendulum); 3693 (*annuum* var. annuum)

Cuatrecasas, J. & R. Romero Castañeda 24894 (*annuum* var. glabriusculum)

Cuatrecasas, J. et al. 12062 (*annuum* var. glabriusculum); 12209 (*rhomboideum*); 12236 (*annuum* var. glabriusculum); 27335 (*rhomboideum*)

Cueva, M. 218 (*dimorphum*); 270 (*geminifolium*); 460, 462 (*dimorphum*); 480, 485, 569 (*geminifolium*); 628 (*dimorphum*); 2912 (*piuranum*)

Cueva, M. & R. Rivera 484 (*pubescens*)

Cueva, M. et al. 2655, 2656, 2657, 2658 (*piuranum*)

Cuevas, R. & D. Deniz 3081 (*rhomboideum*)

Cuezzo, A.R. 928, 976, 3379 (*chacoense*)

Curran, H.M. 318 (*annuum* var. glabriusculum)

Curran, H.M. & M. Haman 1181 (*parvifolium*)

Curtiss, A.H. 671, 2205, 5458 (*annuum* var. glabriusculum)

Cusato, L. 1667 (*flexuosum*)

Custódio Filho, A. 1327 (*schottianum*); 1849, 1860, 1914, 2056 (*hunzikerianum*); 2099, 2807 (*mirabile*)

Custódio Filho, A. et al. 678, 1004, 1144 (*cornutum*)

Custódio Filho, F. & A. Custódio Filho 448 (*hunzikerianum*)

Czerwenka, J. 453 (*annuum* var. glabriusculum)

D’Arcy, W. & B. Hammel 12252 (*annuum* var. glabriusculum)

D’Arcy, W. 4737, 5137, 11215, 11824, 12102 (*annuum* var. glabriusculum); 12130 (*frutescens*); 14822, 14823 (*rhomboideum*); 15777, 16423 (*lycianthoides*); 17716 (*cardenasii*)

D’Arcy, W. et al. 15644 (*dimorphum*)

d’Orbigny, A.D. 604 (*baccatum* var. baccatum)

da Costa Sacco, J. 1332 (*baccatum* var. baccatum)

da Cruz, N.D. et al. 6291 (*carassense*)

da Saldanha da Gama, J. 6075 (*rabenii*)

da Silva, M.F.B. et al. 18 (*caatingae*)

Dahlgren, B.E. & E. Sella 564 (*frutescens*)

Dalafene 2 (*frutescens*)

Dalla Tea, F. 37 (*chacoense*)

Daly, D.C. et al. 21 (*caballeroi*); 8711, 9979 (*coccineum*)

Damasceno, S.A. 56 (*caatingae*)

Daneu, L. et al. 632 (*mirabile*)

Daniel, Hno. 397, 1464, 3429 (*dimorphum*)

David, H. et al. 3119 (*annuum* var. glabriusculum)

Davidse, G. & W. D’Arcy 11227 (*flexuosum*)

Davidse, G. et al. 20126, 37476 (*annuum* var. glabriusculum)

Daviña, J. & A.I. Honfi 593 (*baccatum* var. baccatum); 597 (*flexuosum*)

Davis, E.W. 1066 (*chinense*)

Davis, E.W. & J. Yost 993 (*chinense*); 994 (*annuum* var. glabriusculum)

Dawe, J. 73-0627 (*annuum* var. glabriusculum)

Dawe, M.T. 774 (*rhomboideum*)

Dawson, G. 117 (*chacoense*)

Day, E.H. 329 (*annuum* var. glabriusculum)

de Ance, S.A. 81 (*chacoense*)

de Andrade-Lima, D. 79, 51-918, 62-4055, 71-6395 (*caatingae*); 72-7202 & 72-7203 (*villosum*); 75-8086 (*caatingae*)

de Barros, A.A.M. et al. 835, 2367 (*campylopodium*)

de Barros, F. 1230 (*schottianum*)

de Benavides, O. 2974, 3169, 3636 (*rhomboideum*); 4672 (*chinense*); 4673, 4674 (*annuum* var. annuum); 5088, 5308, 5406 (*rhomboideum*); 5698, 6387 (*annuum* var. annuum); 6463, 7157, 7332, 10603 (*rhomboideum*)

de Benavides, O. & C. Paredes 6238 (*rhomboideum*)

de Benavídez, O. 1819 (*annuum* var. glabriusculum); 1986, 2246 (*pubescens*); 4692 (*frutescens*); 5389 (*annuum* var. glabriusculum); 6190 (*pubescens*); 7875 (*annuum* var. glabriusculum); 8312, 10527 (*frutescens*)

de Campos Novaes, J. 170 (*schottianum*); 1941, 2179 (*flexuosum*)

de Camargo, C. 020 (*chinense*)

de Carvalho, A.M.V. et al. 6863 (*frutescens*)

De Egea, J. & M. Peña-Chocarro 769 (*chacoense*)

de Escobar, B. & G. Barrera 281 (*annuum* var. annuum)

de Escobar, L.A. 828 (*frutescens*)

de Garganta F., M. 818 (*annuum* var. glabriusculum); 857 (*rhomboideum*)

de Gasper, A.L. 1394 (*flexuosum*)

de Gasper, A.L. et al. 1018, 1363 (*flexuosum*); et al. 2610 (*mirabile*)

de Godoy, S.A.P. et al. 2047 (*schottianum*)

de Jeréz, Z.A. et al. 4919 C (*eximium*)

de Jong, W. 53 (*annuum* var. glabriusculum)

de la Sota, E. 104 (*baccatum* var. baccatum); 129, 747 (*chacoense*)

de Lima, H.C. et al. 2526 (*friburgense*)

de Lima, R.A.F. 85 (*recurvatum*)

de M.A. Carvalho, M.V.B. 175 (*caatingae*)

de Mello Filho, L.E. 502, 1187 (*campylopodium*)

de Mello-Silva, R. 1275 (*baccatum* var. baccatum)

de Menjivar, M.G. & E.A. Montalvo 3846 (*rhomboideum*)

de Michel, R. 159 (*baccatum* var. baccatum); 492 (*baccatum* var. pendulum); 517 (*annuum* var. annuum); 714 (*baccatum* var. baccatum); 2568 (*chacoense*)

de Moraes, P.L R. et al. 1164 (*recurvatum*); 23685 (*flexuosum*)

de Oliveira, A.A. 2881, 2911, 2916 (*flexuosum*)

de Oliveira, F. 4 (*baccatum* var. baccatum)

de Oliveira Filho, L.C. & J.C.A. Lima 131 (*longidentatum*)

de Paula, C.H.R. & R. Bacelar 203 (*campylopodium*)

de Queiroz, L.P. 12964 (*parvifolium*); 15495 (*caatingae*)

de Queiroz, L.P. et al. 1520 (*caatingae*); 5958 (*baccatum* var. baccatum); 10598 (*caatingae*)

de Romero, M. 72 (*frutescens*)

de Saint-Hilaire, A. Cat. A1-N° 651, A2-N° 58 (*campylopodium*); 584, 621 (*villosum*)

de Saldanha da Gama, J. 5163 (*muticum*); 5373 (*campylopodium*); 6075 (*baccatum* var. baccatum)

de Sampaio, A.J. 4314 (*rabenii*)

de Souza Silva, J. 608 (*caatingae*)

de T. Alvim, P. 310 (*rabenii*)

de Varela, F. & A. del Castillo 1197 (*chacoense*)

de Vásquez, M.E. 385 (*rhomboideum*)

Deam, C.C. 24 (*frutescens*); 60321 (*annuum* var. glabriusculum)

Deanna, R. 51 (*baccatum* var. pendulum); 53 (*frutescens*); 164, 168, 170 (*lycianthoides*)

Deanna, R. & S. Leiva González 3, 11, 77, 99 (*geminifolium*);

Deanna, R. et al. 133, 144 (*lycianthoides*)

Debouck, D.G. 3016 (*chacoense*)

Debouck, D.G. et al. 3016 (*baccatum* var. pendulum); 3019 (*baccatum* var. baccatum)

Degener, O. 18764 (*annuum* var. glabriusculum)

Deginani, N.B. 2093 (*chacoense*)

Deginani, N.B. et al. 918 (*eximium*); 1523, 1701 (*flexuosum*)

Del Aguila et al., M. 662 (*frutescens*)

del Castillo, A. & F. de Varela 847 (*chacoense*)

del Castillo, A. & R. Neumann 383 p.p. (*chacoense*), 383 p.p. (*eximium*)

Delgado, A. et al. 81 (*annuum* var. glabriusculum)

Delgado, L. 23, 251, 359, 505 (*rhomboideum*)

Delgado, T. & Grupo Post-Grado MO-QCNE 38 (*geminifolium*)

Delgado S., A. et al. 1542 (*annuum* var. glabriusculum)

Demuner, V. & E. Bausen 628 (*pereirae*)

Dent, H.C. 90 (*rabenii*)

Di Fulvio, E. 455 (*chacoense*)

Di Giácomo, A. 230, 607 (*chacoense*)

Dias, H.M. et al. 783 (*mirabile*)

Díaz, A.L. 246 (*rhomboideum*)

Díaz, C. et al. 10120 (*geminifolium*)

Díaz, J. et al. 106 (*frutescens*)

Díaz, S. et al. 659 (*geminifolium*)

Díaz P., S. et al. 717 (*dimorphum*); 3883, 3900 (*lycianthoides*)

Díaz Rico, A. ADR-48, ADR-221 (*annuum* var. annuum)

Díaz S., C. et al. 2548, 2767 (*geminifolium*); 3201, 4032, 6242, 7417, 7474, 7512 (*hookerianum*)

Diesel, S.1655 (*flexuosum*)

Dimitri, M.J. & B. Piccinini 56 (*chacoense*)

Diogo, J. 420 (*muticum*)

Dirección Gral. de Biodiversidad/SERNA 60 (*annuum* var. glabriusculum)

Dittrich, V.A.O. & C. Kozera 298 (*flexuosum*)

do Cavalo, P. et al. 162 (*caatingae*)

do Socorro, M. & G. Ferreira 150 (*longidentatum*)

Dodson, C.H. 9617, 12331 (*hookerianum*)

Dodson, C.H. & A.H. Gentry 6576 (*frutescens*); 12269 (*lycianthoides*)

Dodson, C.H. & K. Tan 5319 (*annuum* var. glabriusculum)

Dodson, C.H. & L.B. Thien 2101, 2131 (*lycianthoides*); 2144 (*rhomboideum*); 12778 (*lycianthoides*)

Dodson, C.H. et al. 9149 (*lycianthoides*); 9157 (*geminifolium*); 9161, 14163 (*lycianthoides*)

Domin, K. 8247 (*frutescens*)

Domínguez Cadena, R. 2606 (*annuum* var. glabriusculum)

Domínguez Mariani, A. 522, 601 (*annuum* var. glabriusculum)

Domínguez, J.A. 179 (*chacoense*)

Domínguez-Licona, E. et al. 1824 (*annuum* var. glabriusculum)

Donnell Smith, J. 1837 (*lanceolatum*); 7255 (*frutescens*)

Dorantes, J. et al. 1216 (*rhomboideum*)

Dorr, L.J. 6729 (*pubescens*)

Dorr, L.J. & L.C. Barnett 6801 (*eximium*); 7041 (*caballeroi*); 7678 (*annuum* var. glabriusculum)

Dorr, L.J. et al. 5275, 5390 (*rhomboideum*)

Dos Santos et al. 274, 275, 276, 277 (*chinense*)

dos Santos, M.B. et al. 6 (*schottianum*)

Dostert, N. 98/162 (*pubescens*)

Douglas, W.D. 6154, 6155 (*annuum* var. annuum)

Dressler, R.L. 1276, 2429 (*annuum* var. glabriusculum)

Dreveck, S. & F.E. Carneiro 1620 (*flexuosum*)

Dreveck, S. et al. 268, 403 (*flexuosum*); 1832 (*recurvatum*)

Drombrowski, L.T. & Y.S. Kuniyoshi 2817 (*recurvatum*)

Drummond, T. 243 (*annuum* var. glabriusculum)

Drushel, J.A. 2810, 4179 (*annuum* var. glabriusculum)

Dryander, E. 2178 (*annuum* var. glabriusculum)

Duarte, A.P. 5491 (*schottianum*); 7666 (*campylopodium*)

Dudey, D. 1101 (*annuum* var. glabriusculum)

Dugand, A. 478 (*annuum* var. glabriusculum)

Duke, J.A. 3815, 5080 (*annuum* var. glabriusculum); 14410 (*annuum* var. annuum)

Duke, J.A. & H.F. Winters 17330 (*pubescens*)

Dumont, K. et al. 7778 (*rhomboideum*)

Dumont, M. et al. 43 (*annuum* var. glabriusculum)

Dunlap, V.C. 178 (*annuum* var. glabriusculum)

Duque Jaramillo, J.M. 20 (*rhomboideum*); 2036 (*annuum* var. annuum); 3330 (*dimorphum*); 3549 (*annuum* var. glabriusculum); 3555 (*annuum* var. annuum); 4083-A (*annuum* var. glabriusculum)

Durán, R. et al. 2963, 3485 (*annuum* var. glabriusculum)

Durán, S. et al. 1771 (*caballeroi*)

Dusén, P. 3360 (*recurvatum*); 5072 (*campylopodium*); 7418 (*recurvatum*); 7613, 7825 (*flexuosum*); 10200 (*rabenii*); 14227 (*cornutum*); 14527, 16139 (*recurvatum*); 16337 (*flexuosum*)

Duss, A. 149, 349, 3574, 3575, 3681 (*annuum* var. glabriusculum)

Dwyer, J.D. 2559 (*annuum* var. glabriusculum)

Dziekanowski, C.T. et al. 1717 (*annuum* var. glabriusculum)

Eaton, A.A. 421 (*annuum* var. glabriusculum)

Echeverria, G. 915 (*pubescens*)

Edmundo, A 360 (*campylopodium*)

Edwards, M.T. 592 (*annuum* var. glabriusculum)

Eiten, G. & L.T. Eiten 10140 (*frutescens*)

Ekman, E. 806 (*baccatum* var. baccatum); 808 (*flexuosum*); H 2116, 11372, 16269 (*annuum* var. glabriusculum)

Elías, Bro. 570, 1461 (*annuum* var. glabriusculum)

Ellemann, L. 66689 (*pubescens*)

Ellenberg, H. 4501 (*chacoense*)

Elorsa C., M. 367, 3132, 6168, 6186 (*annuum* var. glabriusculum)

Emmons, L. 86 (*coccineum*)

Encarnación, F. 750 (*coccineum*)

Encina, J.A. et al. 2553 (*annuum* var. glabriusculum)

Enea A., G. 253 (*annuum* var. glabriusculum)

Eriksen, B. & B. Ståhl 19 (*lycianthoides*)

Ernst, W.R. 1264 (*annuum* var. glabriusculum)

Escalante, S. 627 (*annuum* var. glabriusculum)

Escobar, J.D. 19 (*chacoense*)

Escobar FM, F. 122 (*lycianthoides*)

Escolastico, R. 165 (*annuum* var. glabriusculum)

Eshbaugh, W.H. 575, 588 (*eximium*); 614 (*cardenasii*); 765 (*pubescens*); 1526, 1527 (*cardenasii*); 1943 a, 1943 C, 1943 d (*eshbaughii*); 2046 C, 2046 J (*cardenasii*); E924 (*baccatum* var. pendulum); E954 (*baccatum* var. baccatum); E1137 (MU, US) (*tovarii*)

Espejo, A. et al. 1260 (*annuum* var. glabriusculum)

Espina, J. 182 (*chinense*); 189 (*annuum* var. annuum); 276 (*chinense*); 571 (*frutescens*); 577 (*annuum* var. glabriusculum); 586 (*frutescens*); 609 (*annuum* var. glabriusculum)

Espina, J. et al. 191, 193 (*annuum* var. glabriusculum)

Espinal T., S. 521, 1059 (*rhomboideum*)

Espinal, M. 203 (*annuum* var. glabriusculum)

Espindola-Nascimento, L. & J.R.Pinheiro 13 (*chinense*)

Espindola-Nascimento, L. & L.S. Simoa 01, 02, 03 (*annuum* var. glabriusculum); 04, 05, 06 (*chinense*); 07, 08 (*annuum* var. glabriusculum)

Espinosa Jiménez, J.A. 143 (*annuum* var. glabriusculum)

Espinosa, R. 1080 (*pubescens*); 1120 (*rhomboideum*)

Estrada, E. 43 (*annuum* var. glabriusculum)

Estrada V., S. 11 (*lycianthoides*)

Estudiantes herbario MEDEL 158, 630 (*dimorphum*); 709 (*pubescens*); 976 (*annuum* var. glabriusculum)

Etter, A. & L.A. Villa 1016 (*annuum* var. glabriusculum)

Evans, D.K. 4384 (*annuum* var. annuum)

Evans, R.J. 1469 (*lanceolatum*)

Eyerdam, W.J. 4 (*annuum* var. glabriusculum); 24801 (*pubescens*); 25313 (*baccatum* var. pendulum)

Eyerdam, W.J. & A.A. Beetle 8661, 8720 (*annuum* var. glabriusculum)

Ezcurra, C. & M. Ponce 533 (*chacoense*)

Fabris, H.A. 3454 (*baccatum* var. baccatum)

Fabris, H.A. et al. 5229 (*eximium*); 5278 (*chacoense*)

Fagerlind, F. & G. Wibom 1016 (*rhomboideum*); 2632 (*lycianthoides*)

Falcao, B.F. et al. 10 (*cornutum*)

Falcão, J. 190 (*flexuosum*)

Falcone & Castellanos 262 & 5023 (*chacoense*)

Farney, C. et al. 364 (*campylopodium*)

Felger, R.S. 1502 (*annuum* var. glabriusculum)

Felger, R.S. et al. 226, 1403 (*annuum* var. glabriusculum)

Felitto, G. & V. Ariati 804 (*flexuosum*)

Félix, D.F. 71 (*baccatum* var. baccatum)

Félix, L.P. 4994 (*parvifolium*)

Fendler, A. 982 (*rhomboideum*); 1018 (*annuum* var. glabriusculum)

Fernández, E. 3998 (*minutiflorum*)

Fernández, S. 97 (*annuum* var. glabriusculum)

Fernández, A. & R. Gonto 33761 (*annuum* var. glabriusculum)

Fernández Alonso, J.L. & R. Jaramillo 5268, 5338 (*annuum* var. glabriusculum); 5401 (*rhomboideum*)

Fernández Alonso, J.L. & G. López 18335 (*rhomboideum*)

Fernández Alonso, J.L. & G. Morales 6777 (*rhomboideum*); 6793 (*annuum* var. glabriusculum)

Fernández Alonso, J.L. et al. 7542 (*frutescens*); 10725 (*lycianthoides*); 13640 (*annuum* var. glabriusculum); 16057 (*frutescens*); 20758 (*chinense*); 23798 (*dimorphum*)

Fernández Casas, F.J. & J. Molero 6063, 6131 A, 6248, 6274 (*baccatum* var. baccatum)

Fernández Casas, F.J. & R. Morales Valverde 10659 (*annuum* var. glabriusculum)

Fernández M., R. & J. Dorantes 1840 (*annuum* var. glabriusculum)

Fernández Nava, R. 2946, 3379, 3395 (*rhomboideum*)

Fernández Nava, R. & Z. Guadarrama 1395 (*annuum* var. glabriusculum)

Fernández Pérez, A. & L.E. Mora 1362 (*rhomboideum*)

Fernández T., E. et al. 1896, 2007 (*caballeroi*)

Ferrari, G. 185 (*frutescens*)

Ferraro, L. 554 (*baccatum* var. pendulum)

Ferraz, E. et al. 273 (*caatingae*)

Ferreira, C.D.M. & P. Feliz 233 (*campylopodium*); 241 (*mirabile*)

Ferreira, E.M. 31, 60, 64, 65 (*frutescens*)

Ferreira, E.V.R. 150 (*caatingae*)

Ferreyra, R. 922 (*coccineum*); 4495 (*annuum* var. glabriusculum); 10086 (*frutescens*); 13163 (*coccineum*); 16384 (*chinense*)

Ferreyra, R. & S. Sánchez 19637 (*annuum* var. glabriusculum)

Ferris, R.S. & C.D. Duncan 3188 (*annuum* var. glabriusculum)

Ferris, R.S. & Y. Mexia 5130 (*annuum* var. glabriusculum)

Fiebrig, K. 952 (*rabenii*); 1472 (*chacoense*); 2072 (*eximium*); 12649 (*baccatum* var. baccatum)

Figueiredo, C. et al. 892 (*coccineum*)

Filgueiras, T.S. 3614 (*chinense*); 3615 (*baccatum* var. baccatum)

Filskov, P. et al. 37318 (*rhomboideum*)

Fisher, G.L. 41153 (*annuum* var. glabriusculum)

Flaster, B.49, 1165, 1166 (*mirabile*)

Fleischmann, E. 143 (*coccineum*)

Fletes, E. & L. Angulo 239 (*pubescens*)

Fleury, M. 124 (*chinense*)

Florentín Peña, T. 29 (*chacoense*)

Flores, D. 89 (*eshbaughii*)

Flores, L. 23 (*rhomboideum*)

Flores, S. et al. 157 (*annuum* var. glabriusculum)

Flores M., A. & M.A. Arvizo Y. 4787, 4860 (*annuum* var. glabriusculum)

Flores M., A. & P. Ramos G., A. 2413 (*rhomboideum*)

Flügel, H. et al. 7054 (*annuum* var. glabriusculum)

Folli, D.A. 2995 (*frutescens*)

Fonnegra G., R. 2672 (*dimorphum*)

Fonnegra G., R. & F.J. Roldán 4952 (*dimorphum*)

Fonnegra G., R. et al. 4417, 4442 (*lycianthoides*); 5315 (*dimorphum*); 6243 (*annuum* var. glabriusculum)

Fonseca, W.O. & T.N. Alves 482 (*caatingae*)

Fonturbel, F. FR-17 (*pubescens*)

Forni Martins, E.R. et al. 05/01, 05/03, 05/10 (*schottianum*)

Forte, A. 6154 (*recurvatum*)

Fortunato, R. & R. Micheli 5090 (*chacoense*)

Fortunato, R. et al. 1445, 2399, 3030, 3080, 3215, 4687, 5959, 6520, 6667 (*chacoense*); 7013, 7127, 7128, 7129, 7130, 7162 (*baccatum* var. baccatum); 7184 (*chacoense*)

Forzza, R.C. et al. 3329, 3610, 4366 (*pereirae*)

Fosberg, F.R. 55377, 59385 (*annuum* var. glabriusculum)

Fosberg, F.R. & F. Prieto 22763 (*geminifolium*)

Foster, R.B. 6849, 8802 (*coccineum*)

Foster, R. & C. Augspurger 3164 (*coccineum*)

Foster, R.B. & B. d’Achille 11630, 12017 (*coccineum*)

Foster, R.B. & J. Terborgh 5043 (*coccineum*)

Foster, R.B. et al. 3484 (*coccineum*)

Fotius, G. & I.B. Sá 3769 (*parvifolium*)

Fournet, J.P. 4616, 4666 (*annuum* var. glabriusculum)

Fournet, J.P. et al. 4878 (*annuum* var. glabriusculum)

Fournier, L.A. 218 (*frutescens*)

Fraga, C.N. et al. 2877 (*villosum*)

Frame, D. et al. 217 (*coccineum*)

França, F. et al. 4955 & 4956 (*caatingae*); 5556 (*longidentatum*)

França, G.S. & R. Stehmann 289 (*mirabile*)

Francia, P. 42 (*baccatum* var. pendulum)

Franco, C. et al. 4390 (*schottianum*)

Franco Rosselli, M.P. et al. 1645, 1649 (*dimorphum*)

Fredholm, A. 5523 (*annuum* var. glabriusculum)

Freeborn, B. et al. 37 (*annuum* var. glabriusculum)

Freire, B. & D. Naranjo 612 (*chinense*)

Freire, B. & M. Ruales 2931 (*annuum* var. glabriusculum)

Freire, E. 7152 (*rhomboideum*)

Freitas, L. 268, 615 (*villosum*)

Freire Fierro, A. 1243 (*geminifolium*); 3181 (*lycianthoides*)

Freire Fierro, A. & M. Asanza 3087 (*geminifolium*)

Frías-Castro, A. et al. 673 (*rhomboideum*)

Fries, R. 1110 (*eximium*); 1224, 1547 (*chacoense*)

Froehner, C. 169 (*coccineum*)

Fryxell, P.A, 5085, 1119, 1213, 2953 (*annuum* var. glabriusculum)

Fuentes, A.F. 1385 A (*baccatum* var. baccatum)

Fuentes, Z. & E. Fuentes 480 (*rhomboideum*)

Funez, L.A. 1561, 3325 (*recurvatum*)

Funez, L.A. & A.E. Zermiani 1394 (*flexuosum*)

Furlan, A. et al. 1527 (*schottianum*)

Gadelha Neto, P. da C. 1748 (*chinense*); 1751 (*frutescens*)

Gadelha Neto, P. da C. & I.B. Lima 3993 (*parvifolium*)

Galán, S. & K. Cárdenas 12 (*annuum* var. glabriusculum)

Galander, C. 1877 (*baccatum* var. pendulum)

Galeano, G. et al. 2127 (*dimorphum*)

Galeotti, H.G. 1189 (*rhomboideum*)

Gallardo, C. et al. 115, 392, 608 (*annuum* var. glabriusculum)

Gallardo Hernández, C. & E. Pérez García 1869 (*annuum* var. glabriusculum)

Gallegos, S. 318 A (*eximium*)

Galué, N. 122 (*annuum* var. glabriusculum)

Galvão, R. 137 (*campylopodium*)

Gamarra, P. 439 (*pubescens*)

Ganchozo, W. 007 (*annuum* var. annuum)

García Barriga, H. 3119 (*rhomboideum*); 4344 (*annuum* var. glabriusculum); 4708 (*rhomboideum*); 5056, 6426 (*annuum* var. glabriusculum); 6460-6462 (*rhomboideum*); 7114 (*annuum* var. glabriusculum); 11750, 12164 (*rhomboideum*); 13265 (*annuum* var. glabriusculum); 15258 (*dimorphum*); 20144 (*rhomboideum*); 20315A (*frutescens*); 20563 (*annuum* var. glabriusculum); 21269 (*dimorphum*); 26957 (*annuum* var. glabriusculum)

García Cañizares, F. 127 (*annuum* var. glabriusculum)

García, A. 2203 (*rhomboideum*)

García, A.A. 3190 (*annuum* var. annuum)

García, L.C. 133 (*flexuosum*)

García, P. 950 (*chacoense*)

García, R. 6600 (*annuum* var. glabriusculum)

García, I. & H. Cortéz 7235 (*rhomboideum*)

García, M. & F. Ramírez 242 (*annuum* var. glabriusculum)

García, R. et al. 4464 (*annuum* var. glabriusculum)

García, R.J.F. et al. 726 (*cornutum*)

García-Bielma, M.A. & P. Domínguez H. 626 (*annuum* var. glabriusculum)

García P., J. & A. Delgado S. 827 (*annuum* var. glabriusculum)

García R., J. & I. Montaño M. 348 (*pubescens*)

García Mendoza, A. et al. 4023 (*annuum* var. glabriusculum)

Garnier, A. 665 (*pubescens*)

Garvizu, M. et al. 1156 (*eximium*)

Garzón, G. et al. 3214 (*frutescens*)

Gatti, F.E. 24 (*flexuosum*)

Gaudichaud, C. 513 (*campylopodium*)

Gaumer, G.F. 864, 1546, 1546 bis (*annuum* var. glabriusculum); 1019 (*frutescens*); 2006, 24042 (*annuum* var. glabriusculum)

Gensini, J.G. 103 (*dimorphum*); 136, 180, 199 (*lycianthoides*)

Gentry, A.H. 3761 (*annuum* var. glabriusculum); 48174 (*dimorphum*); 12638 (*rhomboideum*)

Gentry, A.H. & P.E. Berry 14774 (*rhomboideum*); 15038 (*annuum* var. glabriusculum)

Gentry, A.H. & C. Bonifaz 28785 (*rhomboideum*)

Gentry, A.H. & C. Díaz S. 58262 (*hookerianum*)

Gentry, A.H. & C. Josse 72356 (*hookerianum*)

Gentry, A.H. & H. León 20187 (*rhomboideum*)

Gentry, A.H. & A.L. Peixoto 913 (*campylopodium*)

Gentry, A.H. et al. 8976 (*annuum* var. glabriusculum); 8998 (*rhomboideum*); 12095 (*lycianthoides*); 34774 (*annuum* var. glabriusculum); 37623 (*coccineum*); 44206, 53586 (*annuum* var. glabriusculum); 63614 (*lycianthoides*); 70189 (*rhomboideum*)

Gentry, H.S. 373, 949, 1541, 2359, 14323 (*annuum* var. glabriusculum)

Germán R., M.T. et al. 736 (*pubescens*); 1005 (*annuum* var. glabriusculum)

Gerold, R. 175 (*eximium*); 284 (*chacoense*)

Giacomelli, A. 130, 4806 (*chacoense*)

Giacomin, L.L. & T.E. Almeida 1688, 1691 (*recurvatum*)

Giacomin, L. et al. 200 (*frutescens*); 373 (*villosum*); 899 (*mirabile*); 1107 (*schottianum*); 1248, 1481 (*mirabile*); 1709 (*recurvatum*); 1946, 1959 (*pereirae*); 2014 (*schottianum*); 2018 (*cornutum*)

Gil, A. et al. 310 (*dimorphum*)

Gil, M. & R.D. López 21 (*rhomboideum*)

Gillies, J. 25 (*chacoense*)

Gilman, M.F. 142 (*annuum* var. glabriusculum)

Gilmartin, A. 554 (*hookerianum*)

Ginés, Bro. (P. Mandazen Soto) 2716 (*annuum* var. glabriusculum); 3901 (*rhomboideum*)

Giraldo, L.F. et al. 667, 900, 1631 (*dimorphum*)

Giraldo-Cañas, D. et al. 3485 (*frutescens*)

Glaziou, A. 1002 (*rabenii*); 3074 pp (*recurvatum*); 3074 pp (*schottianum*); 4168 (*campylopodium*); 8841 (*rabenii*); 8872, 11385, 12108 (*campylopodium*)

Glenboski, L.L. C-51 (*annuum* var. glabriusculum), C-87 (*baccatum* var. umbilicatum)

Godoy, S.A.P. et al. 352 (*cornutum*)

Goll, G.P. 271, 273 (*annuum* var. annuum); 378 (*annuum* var. glabriusculum)

Gomes Guarino, E. de S. et al. 978 (*flexuosum*)

Gomes, P. et al. 826 (*caatingae*)

Gómez, A. 511 (*annuum* var. glabriusculum)

Gómez, P. 3 (*pubescens*); 006 (*frutescens*)

Gómez, S. 190 (*rhomboideum*)

Gómez-Lorence, F. 877 (*annuum* var. glabriusculum)

Gonçalves, F.B. 267 (*caatingae*)

Gonçalves, J.M. et al. 175 (*longidentatum*)

Gonçalves, S.L. & M. Koketsu 1, 2 (*chinense*); 3 (*frutescens*)

Gontijo, F.D. et al. 589 (*carassense*)

González 89 (*chinense*)

González, A.C. 993 (*annuum* var. glabriusculum)

González, E. 874 (*rhomboideum*)

González, M. 2398 (*rhomboideum*)

Gonzáles, P. 341 (*geminifolium*); 344 (*pubescens*)

González, S. & R. Fernández 2186 (*annuum* var. glabriusculum)

González, V. 975 (*annuum* var. annuum)

González G., F. et al. 1553 (*dimorphum*)

González M., R. et al. 13 (*dimorphum*)

González Medrano, F. 4181, 9454 (*rhomboideum*); 9692 (*annuum* var. glabriusculum); 10492, 12578, 12872 (*rhomboideum*)

González Medrano, F. & P. Hiriart 12945 (*annuum* var. glabriusculum)

González Medrano, F. et al. 1823 (*annuum* var. glabriusculum); 4148 (*rhomboideum*)

González Olivares, S. 494 (*annuum* var. glabriusculum)

Gonzalez Ortega, J. 697 (*annuum* var. glabriusculum)

González Parini, F. 170, 1176, 1533 (*flexuosum*)

González Parini, F. et al. 324, 1706 (*flexuosum*)

Gonzatti, F. & E. Valduga 1779 (*baccatum* var. baccatum)

Goodding, L.N. 405-45 (*annuum* var. glabriusculum)

Gopar Vasquez, F. 122 (*annuum* var. glabriusculum)

Gordillo, E. & L. Forero 41 (*rhomboideum*)

Goudot, J. 4 (*rhomboideum*); 55 (*lycianthoides*)

Gould, F.W. et al. 2687 (*annuum* var. glabriusculum)

Gouvêa, Y.F. 213 (*villosum*); 215 (*cornutum*); 227 (*villosum*)

Gragson, T.L. 124 (*baccatum* var. baccatum)

Graham, J. & J. Schunke V. 271, 460 (*annuum* var. glabriusculum); 4633 (*coccineum*)

Granados-Tochoy, J.C. & J. Garzón 675 (*annuum* var. glabriusculum); 691 (*dimorphum*)

Granados-Tochoy, J.C. et al. 411 (*dimorphum*)

Grandi, T.S.M. 261 (*frutescens*)

Grases, C. 10 (*rhomboideum*)

Grassi, D. 2063 (*chacoense*)

Grayum, M. 4207, 12111 (*annuum* var. glabriusculum)

Grayum, M. & R. Warner 8366 (*annuum* var. glabriusculum)

Greenman, J.M. & M.T. Greenman 5629 (*annuum* var. glabriusculum)

Gregory et al. 10508 (*frutescens*)

Grijalva, A. 706, 759 (*annuum* var. glabriusculum)

Grijalva, A. et al. 2712, 2921 (*rhomboideum*)

Grijalva, R. 259 (*lycianthoides*)

Groth, B.H.A. 40 (*annuum* var. glabriusculum)

Gruhn, J. et al. 221 (*annuum* var. glabriusculum)

Grupo P. do Cavalo 162, 331, 438, 453, 694, 771 (*caatingae*)

Guadarrama, M.A. et al. 865, 6857 (*annuum* var. glabriusculum)

Guaglianone, E.R. 2255 (*chacoense*)

Guaglianone, E.R. et al. 309 (*chacoense*)

Guareco, I. 457 (*coccineum*); 627 (*chinense*)

Guareco, I. & J. Balderrama 464 (*pubescens*)

Guedes, F. 98 (*longidentatum*)

Guedes, M.L. et al. 10847 (*caatingae*); 12268 (*parvifolium*); 13245, 13297 (*caatingae*); 16183, 16187 (*longidentatum*); 30361 (*caatingae*)

Guerrero, O.D. 408 (*rhomboideum*)

Guerrero-Nuño, J.J. 586 (*annuum* var. glabriusculum)

Guilcapi, D. 19 (*frutescens*)

Guillemin, M. 939 (*schottianum*)

Guio 80 (*baccatum* var. baccatum)

Guízar Nolazco, E. & L. Pimentel B. 2884, 2954 (*annuum* var. glabriusculum)

Gustafsson, C. & C. Bonifaz 412 & 477 (*lycianthoides*)

Gutiérrez, E. 24 (*chinense*); 25 (*annuum* var. glabriusculum); 26, 109 (*annuum* var. annuum); 388 (*chinense*)

Gutiérrez, J. 75 (*chacoense*)

Gutiérrez, J.P. 85-23, 85-26, 85-27, 85-41, 85-71(*annuum* var. annuum)

Gutiérrez, M. & T. Acero 76 (*annuum* var. glabriusculum)

Gutiérrez B., C. 2227, 4475 (*annuum* var. glabriusculum)

Gutiérrez R., J. 1004, 1072 (*neei*)

Gutierrez V., G. 414 (*annuum* var. glabriusculum); 487 (*rhomboideum*)

Gutierrez V., G. & F.A. Barkley 17C080 (*rhomboideum*)

Guzmán, L. & F.J. Santana 303 (*rhomboideum*)

Guzmán, M. et al. 396, 667 (*annuum* var. glabriusculum); 1011 (*annuum* var. annuum); 1307 (*annuum* var. glabriusculum)

Haber, W.A. & E. Bello C. 2260 (*rhomboideum*)

Haber, W.A. & W. Zuchowski 9930 (*annuum* var. glabriusculum); 10105 (*rhomboideum*)

Hage, J.L. & E. B. dos Santos 356 (*chinense*)

Hahn, W.J. 802 (*baccatum* var. baccatum); 1405 (*chacoense*); 4830 (*frutescens*)

Hahn, W.J. & J.C. Sandino 420 (*annuum* var. glabriusculum)

Hahn, W.J. et al. 937 (*flexuosum*); 1024 (*baccatum* var. baccatum); 1213, 2506 (*chacoense*)

Hall, E. 495 (*annuum* var. glabriusculum)

Hall, J.S. & S.M. Bockus 7802 (*annuum* var. glabriusculum)

Hamann, M. & O. Hamann 645, 1418 (*frutescens*)

Hamann, O. & S. Serberg 1700 (*frutescens*)

Hamblett, R.B. 1538 (*annuum* var. glabriusculum)

Hanan Alipi, A.M. et al. 1386 (*annuum* var. glabriusculum)

Handro, O. 552 (*cornutum*); 860 (*rabenii*); 1106 (*mirabile*); 2184 (*recurvatum*)

Hansen, B.F. & M. Nee 7430 (*annuum* var. glabriusculum)

Hansen, B.F. & R.P. Wunderlin 12049 (*annuum* var. glabriusculum)

Hansen, B.F. et al. 1497 (*annuum* var. glabriusculum)

Hanson, H.C. 468 (*annuum* var. glabriusculum)

Harker, M. et al. 2432 (*rhomboideum*)

Harley, R.M. 16184 (*longidentatum*); 16297 (*caatingae*)

Harley, R.M. & A.M. Giulietti 54171 (*parvifolium*)

Harley, R.M. et al. 3445 (*caatingae*); 10986 (*rabenii*); 22736 (*baccatum* var. baccatum)

Harling, G. 316 (*frutescens*); 519, 1680 (*lycianthoides*); 4272 (*rhomboideum*); 5867 (*longifolium*)

Harling, G. & L. Andersson 12250 (*lycianthoides*); 13320, 13334 (*rhomboideum*); 13680 (*geminifolium*); 14414, 15209 (*rhomboideum*); 16487 (*lycianthoides*); 18283, 18316 (*hookerianum*); 18756 (*lycianthoides*); 21464 (*longifolium*); 22180, 22344 (*geminifolium*); 21478, 21665 (*rhomboideum*); 23121, 23265 (*lycianthoides*)

Harling, G. & B. Ståhl 26441 (*geminifolium*); 26470 (*rhomboideum*)

Harling, G. et al. 9226 (*geminifolium*); 15092, 15351, 20329, 20630, 20725 (*rhomboideum*)

Harmon, W.E. & J.D. Dwyer 3543 (*annuum* var. glabriusculum)

Harris, W. 5110, 9327, 10051 (*annuum* var. glabriusculum)

Hart, J. 594 (*annuum* var. glabriusculum); 621, 953 (*pubescens*)

Hartley, W. 5H 132 (*chacoense*)

Hartweg, K.T. 1296 (*rhomboideum*)

Hassler, É. 215 (*baccatum* var. baccatum), 1607 (*flexuosum*); 1926 (*baccatum* var. baccatum), 2891 (*rabenii*); 5134 (*flexuosum*); 5703 (*baccatum* var. baccatum); 5742, 5893 (*flexuosum*); 6070 (*baccatum* var. baccatum), 6498 (*rabenii*); 8633 (*flexuosum*); 12385 (*baccatum* var. baccatum)

Hasting, J.R. & R.M. Turner 65-84, 64-229 (*annuum* var. glabriusculum)

Hatschbach, G. 52 (*recurvatum*); 3952, 7352 (*flexuosum*); 10180 (*recurvatum*); 12593 (*flexuosum*); 17491 (*recurvatum*); 18030, 18412 (*flexuosum*); 20262, 25714, 26000, 30892 (*recurvatum*); 34886 (*chinense*); 38096 (*recurvatum*); 58780 (*baccatum* var. baccatum); 61141 (*pereirae*)

Hatschbach, G. & L.Z. Ahumada 31246 (*baccatum* var. baccatum)

Hatschbach, G. & A.R. Campos 58235 (*recurvatum*)

Hatschbach, G. & D.D. Guimarães 54794, 54797 (*recurvatum*)

Hatschbach, G. & O. Guimarães 25528 (*rabenii*)

Hatschbach, G. & C. Koczicki 20077, 20877 (*recurvatum*)

Hatschbach, G. & R. Kummrow 38441 (*annuum* var. glabriusculum)

Hatschbach, G. & L. Landrum 40412 (*recurvatum*)

Hatschbach, G. & O. Ribas 61697 (*flexuosum*)

Hatschbach, G. & J.M. Silva 61582 (*villosum*)

Hatschbach, G. et al. 58241 (*rabenii*); 58827 (*baccatum* var. baccatum); 71589 (*flexuosum*); 77668 (*baccatum* var. baccatum)

Hattori, E.K.O. et al. 917 (*villosum*)

Haught, O. 2413 (*rhomboideum*); 3041, 3514 (*hookerianum*); 5148 (*rhomboideum*); 6134 (*geminifolium*); 6307 (*rhomboideum*); 6559 (*annuum* var. glabriusculum)

Haught, O. & H.K. Svenson 11621 (*rhomboideum*)

Haumán, M. et al. 0317, 320 (*chinense*)

Hawkes, J.G. et al. 2003 (*annuum* var. glabriusculum); 3308 (*chacoense*); 6558 (*eximium*)

Hawkins, T. & D. Mejía 702 (*lanceolatum*)

Haxaire, G. 5143 (*chinense*); 5144, 5147 (*annuum* var. glabriusculum)

Heilborn, O. 426 (*rhomboideum*)

Heilbron, T.J. & D. Sanredjo 6 (*annuum* var. glabriusculum)

Heinonen, S. 59 (*flexuosum*)

Heinonen, S. et al. 143, 152 (*chacoense*)

Heinrich, M. 7800 (*annuum* var. annuum)

Heinrichs, E. 182 & 184 (*rhomboideum*)

Heiser, C.B. BGH 952 (*baccatum* var. pendulum); C19 (*annuum* var. glabriusculum); C 29 (*baccatum* var. pendulum); C36, C229, C230, C256, C259, C260 (*frutescens*); C271 (*cardenasii*); C272, C273 (*pubescens*); C276a, C279a, C280a (*baccatum* var. baccatum); C281a, C282a, C290 (*baccatum* var. pendulum); C 301 (*eshbaughii*); E 1130 (*annuum* var. glabriusculum); 4196 (*cardenasii*); 4197 (*eximium*); 4675e (*annuum* var. glabriusculum); 4804, 4806 (*baccatum* var. pendulum); 4813, 4828 (*rhomboideum*); 5041, 6097, 6100, 6247, (*frutescens*); 7801 (*flexuosum*); 7802 (*coccineum*)

Heiser Jr., C.B. C1 (*pubescens*); C6 (*frutescens*); C10 (*chacoense*); 4196 (*cardenasii*)

Hekker, F. & W.H. Hekking 10014, 10023 (*rhomboideum*)

Helme, N, 80 (*chinense*)

Heller, A.A. 98, 227, 4526 (*annuum* var. glabriusculum)

Henao, A. 4373 (*annuum* var. glabriusculum)

Henao, C.I. 167 (*frutescens*); 170 (*annuum* var. glabriusculum)

Henao, C.I. & L. Buraiño 26 (*chinense*); 169 (*annuum* var. glabriusculum)

Henao, C.I. & R. Kuiru 172 (*annuum* var. annuum)

Henao, C.I. & D. Padd 168 (*annuum* var. glabriusculum)

Henao, C.I. & K. Rubiela 316 (*annuum* var. annuum)

Henao, C.I. & Zɨueche 247 (*annuum* var. glabriusculum)

Henkel, T.W. & R. James 3520 (*annuum* var. glabriusculum)

Henz, E. 35360 (*flexuosum*)

Herb. A. van Royen n. 908.244-150 (*frutescens*)

Herb. Biltmore 10175 (*annuum* var. glabriusculum)

Herb. Linn. N° 249.5 (*annuum* var. annuum)

Herb. Sag. 465 (*chinense*)

Heredia, M.D. et al. 563 (*lycianthoides*)

Heriberto, Bro. 15, 231 (*frutescens*)

Heringer, E.P. 6472 (*rabenii*)

Hernández, A. & F. Crespo 82, 83 (*annuum* var. annuum)

Hernández, L. 1206 (*rhomboideum*); 1944 (*annuum* var. annuum); 2016 (*annuum* var. glabriusculum); 3099 (*rhomboideum*)

Hernández, L. & P. Ceballos 675 (*annuum* var. glabriusculum)

Hernández A., F. & J.A. Gutiérrez G. 65 (*annuum* var. glabriusculum)

Hernández García, L. & G.J. Martín 166 (*pubescens*)

Hernández M., H.M. & R. Torres C. 413 (*rhomboideum*)

Hernández Magaña, R. 5302 (*rhomboideum*); 12093, 12130 (*annuum* var. glabriusculum)

Hernández Magaña, R. et al. 6045 (*rhomboideum*)

Hernández Najarro, F. & C. Méndez Morales 1310 (*annuum* var. glabriusculum)

Hernandez Ortega, R. 481, 482, 484 (*annuum* var. annuum)

Hernani A., L. & A. Peña C. 379 (*geminifolium*)

Herrera 543 (*chacoense*)

Herrera, D.A. 16 (*chinense*)

Herrera, G. & J. Bittner 9479 (*dimorphum*)

Herrera Ch., G. & Grupo Estudiantes de Biodiversidad 2924 (*annuum* var. glabriusculum)

Herzog, T. 2289 (*ceratocalyx*)

Heyde, E. & E. Lux 3436 (*rhomboideum*); 3439 (*annuum* var. glabriusculum); 4545 (*rhomboideum*)

Hieronymus, G. 356, 427 (*chacoense*)

Hilgert, N. 1363, 1373, 1374, 2028 (*baccatum* var. pendulum); 2060 (*chinense*); 2061 (*eximium*)

Hill, S.R. 18388 (*annuum* var. glabriusculum)

Hill, S.R. et al. 12996, 27189 (*frutescens*)

Hinojosa, I. & A. Wásra 1133 (*baccatum* var. pendulum)

Hinton, G.B. 4336 (*chinense*); 4339, 9311, 11977 (*rhomboideum*); 23896 (*annuum* var. glabriusculum)

Hinton, G.B. et al. 24206 (*annuum* var. glabriusculum)

Hinton, G.S. 21500 (*rhomboideum*)

Hinton, J.C. 17588, 17943 (*rhomboideum*)

Hiriart, P. 404, 417 (*rhomboideum*)

Hiriart V., P. & G. Ortiz C. 22 (*rhomboideum*)

Hitchcock, A.S. 242 (*annuum* var. glabriusculum); 20841 (*rhomboideum*)

Hodges, V. & J. Gorham 96 (*chinense*)

Hodge, W.H. 6882 (*annuum* var. glabriusculum)

Hoehne, W. 3539 (*frutescens*); 30971 (*annuum* var. annuum)

Holm, R.W. & H. Iltis 75, 297 (*annuum* var. glabriusculum)

Holm-Nielsen, L. 4905, 16643, 16676 (*rhomboideum*)

Holm-Nielsen, L. & J.L. Jaramillo 28836, 28876 (*rhomboideum*)

Holm-Nielsen, L. & S. Jeppesen 1240 (*geminifolium*)

Holm Nielsen, L. et al. 21109 (*chinense*)

Holston, I.F. 569 (*rhomboideum*)

Holt, E.G. & W. Gehriger 109 (*frutescens*)

Holton, I. 13 (*annuum* var. glabriusculum)

Homeier, J. 2555 (*geminifolium*)

Homeier, J. & N. Cumbicus 4060 (*dimorphum*)

Honfi, A. et al. 1284 (*baccatum* var. pendulum); 1286 (*annuum* var. annuum); 1287, 1288 (*baccatum* var. pendulum)

Hoogte, L.v.d. & C. Roersch 724 (*baccatum* var. baccatum)

Hoover, W.S. 1896, 2093, 2227 (*lycianthoides*)

Hormia, K. 2194 (*chinense*); 2195 (*annuum* var. glabriusculum); 2228 (*annuum* var. annuum)

Horta, M.B. et al. 126 (*parvifolium*)

Horton, O.B. & J.L. Morrison 8858 (*annuum* var. glabriusculum)

Hosseus, C.C. 103, 340, 424 (*chacoense*)

House, P.R. 866 (*lanceolatum*)

Howard, R.A. 4867 (*annuum* var. glabriusculum); 6278 (*chinense*); 6558, 11136, 14049 (*annuum* var. glabriusculum)

Howard, R.A. & E.S. Howard 8761, 10055 (*annuum* var. glabriusculum)

Howard, R.A. & G.R. Proctor 13367 (*annuum* var. glabriusculum)

Howell, J.T. 9009, 9175A (*frutescens*); 10289 (*annuum* var. glabriusculum)

Hoyos, D. et al. 118, 127, 146 (*regale*)

Hoyos, S.E. et al. 348 (*chinense*)

Hoyos-Gómez, S.E. et al. 355 (*annuum* var. annuum)

Huamantupa, I. 15251 (*regale*)

Huaylla, H. et al. 2178 (*neei*)

Huber, O. 252 (*annuum* var. glabriusculum)

Huft, M.J. & E. Cabrera 2436 (*annuum* var. glabriusculum)

Humbert, H. et al. 26957 (*annuum* var. glabriusculum)

Humbles, J.E. 6146 (*rhomboideum*)

Hunnewell, F.W. 18784 (*campylopodium*)

Hunn, E. OAX-1341, OAX-1342, OAX-1343, OAX-1344 & OAX-1345 (*annuum* var. annuum)

Hunziker, A.T. 1578 (*chacoense*);1579 (*baccatum* var. baccatum); 1907 (*eximium*); 1985, 1987, 2010, 2024 (*baccatum* var. baccatum); 2052 (*baccatum* var. pendulum); 2104, 2452 (*chacoense*); 2776, 2786 (*baccatum* var. baccatum); 4795, 4797, 4807, 4812, 4820, 5011, 5071, 5088, 5299, 5941, 6016, 6734, 7315 (*chacoense*); 7336, 7337, 7338 (*baccatum* var. baccatum); 7339 (*baccatum* var. pendulum); 7340, 7341(*chacoense*); 7342 (*baccatum* var. pendulum); 7343 (*baccatum* var. baccatum); 7344 (*chacoense*); 7345, 7346 (*eximium*); 7347, 7348, 7349, 7350, 7351, 7352, 7353, 7354, 7355 (*baccatum* var. baccatum); 7356, 7357, 7358, 7359, 7360 (*chacoense*); 7605 (*pubescens*); 7624, 8530, 8764, 8842, 8915, 9111, 9153, 9170, 9222, 9519, 9805 (*chacoense*); 10320 (*flexuosum*); 10376, 10381, 10642, 10700, 10816 (*chacoense*); 11491 (*baccatum* var. pendulum); 11492, 11493 (*annuum* var. annuum); 11695, 13271, 13300, 13520, 13912, 14160, 14842, 15834, 16516, 17972, 18343, 18572 (*chacoense*); 19546 (*recurvatum*); 19557 (*cornutum*); 19558 (*schottianum*); 19562 (*mirabile*); 19563, 19564 (*rabenii*); 19566, 19567 (*villosum*); 19572 (*campylopodium*); 19577 (*schottianum*); 25202, 20229 (*rabenii*); 20230 (*recurvatum*); 20232 (*flexuosum*); 20762 (*baccatum* var. pendulum); 20775 (*recurvatum*); 21016 (*chacoense*); 23481 (*baccatum* var. baccatum); 24992, 24994, 24995 (*flexuosum*); 24997, 24998, 24999, 25001, 25002, 25003 (*recurvatum*); 25013 (*flexuosum*); 25116, 25129, 25130, 25134, 25135 (*campylopodium*); 25137 (*pereirae*); 25140, 25142, 25143, 25155, 25156, 25157 (*mirabile*), 25160 (*campylopodium*); 25165 (*schottianum*); 25166 (*campylopodium*); 25167, 25168 (*schottianum*); 25169, 25173, 25174, 25175, 25176 (*villosum*); 25179, 25180 (*schottianum*); 25183, 25184, 25186, 25187 (*recurvatum*); 25190, 25192, 25193 (*schottianum*); 25196 (*recurvatum*); 25197, 25198, 25199 (*cornutum*); 25200, 25201 (*mirabile*); 25219 (*chacoense*); 25233 (*caatingae*); 25234 (*chinense*); 25235, 25236, 25237, 25238, 25239 (*mirabile*); 25240, 25241, 25242 (*schottianum*); 25243 (*mirabile*); 25244, 25245 (*campylopodium*); 25246 (*muticum*); 25247, 25248, 25249 (*pereirae*); 25250, 25251, 25252, 25253, 25254, 25255, 25260, 25264, 25267 (*mirabile*); 25380, 25381 (*baccatum* var. baccatum); 25383 (*baccatum* var. umbilicatum); 25382, 25383 (*baccatum* var. pendulum); 25481 (*annuum* var. glabriusculum); 25484 (*pubescens*); 25485, 25486, 25487 (*baccatum* var. umbilicatum); 25488 (*baccatum* var. pendulum); 25489 (*frutescens*); 25490 (*chinense*); 25491 (*baccatum* var. baccatum); 25492 (*annuum* var. annuum); 25496 (*baccatum* var. pendulum); 25498 (*annuum* var. annuum); 25505 (*frutescens*); 25550, 25501 (*annuum* var. annuum); 25502 (*chinense*); 25552, 25559 (*baccatum* var. pendulum); 25653, 25654, 25655 (*tovarii*); 29428 (*annuum* var. annuum)

Hunziker, A.T. & J.A. Caro 13495, 13610 (*chacoense*)

Hunziker, A.T. & A.E. Cocucci 14666, 14698, 14913, 15050 (*chacoense*)

Hunziker, A.T. & E. Di Fulvio 16995, 17129 (*chacoense*)

Hunziker, A.T. & P. Maldonado 16263 (*chacoense*)

Hunziker, A.T. & R. Subils 24963, 24964 (*chacoense*)

Hunziker, A.T. et al. 23390, 24346 (*chacoense*); 24993 (*flexuosum*); 25161 (*muticum*); 25181 (*villosum*); 25206 (*carassense*); 25239 (*mirabile*); 25256 (*carassense*); 25334, 25388 (*chacoense*);

Hunziker, J.H. 2450 (*annuum* var. annuum); 2532, 9031 (*frutescens*); 9032, 9034 (*annuum* var. glabriusculum)

Hunziker, J.H. et al. 12745, 13036 (*baccatum* var. baccatum)

Hürlimann, H. 6032 (*mirabile*)

Hurtado, F. 715 (*chinense*)

Hurtado, R. 698 (*pubescens*)

Hurtado, R. & R. Zenteno 1636 (*baccatum* var. baccatum)

Hutchinson, P.C. & K. von Bismarck 6396 (*geminifolium*)

Hutchinson, P.C. & J.K. Wright 3129, 3541 (*annuum* var. glabriusculum); 3549 (*rhomboideum*); 4892 (*pubescens*); 6905 (*rhomboideum*)

Hutchinson, P.C. et al. 3078 (*rhomboideum*)

Ibarra Manríquez, G. 3616 (*annuum* var. glabriusculum)

Ibarrola, T.S. 2988 (*chacoense*); 3911, 4051, 4281 (*baccatum* var. baccatum)

Idobro, J.M. 10379 (*dimorphum*)

Iltis, H.H. 257 (*baccatum* var. pendulum); 1067 (*pubescens*)

Iltis, H.H. & M.G. Iltis 29 (*annuum* var. glabriusculum)

Imaguive, N. 5276 (*flexuosum*)

Insfrán, P. 765 (*chacoense*)

Insua, R. 513 (*chacoense*)

Irvine, D. 773 (*annuum* var. glabriusculum)

Irwin, H.S. et al. 19117, 19158, 19189, 19438, 25203 (*rabenii*)

Isern, J. 8094, 8240 (*chacoense*)

Itow, S. 136 (*frutescens*)

Ivanauskas, N.M. et al. 4532 (*schottianum*); 4631 (*hunzikerianum*)

Jack, J.G. 4376, 5223 (*annuum* var. glabriusculum)

Jahn, A. 667 (*rhomboideum*)

Jaime, N.A. 23 (*chacoense*)

Jameson, W. 328 (*rhomboideum*)

Jansen-Jacobs, M.J. et al. 119 (*annuum* var. glabriusculum); 1809 (*frutescens*); 1810, 1811, 3465 (*annuum* var. glabriusculum)

Jaramillo, J. 6425 (6423?), 6426, 6774, 6965-B, 8209 (*lycianthoides*); 8926 (*pubescens*); 10265 (*geminifolium*); 26136 (*rhomboideum*); 26264 (*annuum* var. annuum)

Jaramillo, J. & F. Cohello 29118 (*rhomboideum*)

Jaramillo, J. & I. Tapia 18452 (*lycianthoides*); 18525 (*dimorphum*)

Jaramillo, J. & V. Zak 7856 (*lycianthoides*)

Jaramillo, P. et al. 1775 (*frutescens*)

Jaramillo, R. et al. 351 (*frutescens*); 2698 (*dimorphum*)

Jardim, A. et al. 1995 (*caballeroi*)

Jardim, J.G. 4435 (*parvifolium*)

Jardim, J.G. et al. 4345, 4860 (*pereirae*)

Jarenkow, J.A. 2158 (*flexuosum*)

Játiva, C. & C.C. Epling 552 (*rhomboideum*)

Jensen, E. MK0040 (*annuum* var. glabriusculum)

Jermy G. 189 (*annuum* var. glabriusculum)

Jiménez, E. et al. 1126 (*lycianthoides*)

Jiménez, I. et al. 7517 (*eximium*)

Jiménez, J. 02 (*annuum* var. annuum)

Jiménez, R. 1 (*annuum* var. glabriusculum)

Jiménez, B. & G. Marín 1651 & 5893 (*flexuosum*)

Jiménez, F. et al. 722 (*annuum* var. glabriusculum)

Jiménez L., A. 50 (*baccatum* var. baccatum)

Jiménez-Osornio, J.J. 106 (*chinense*)

Jimeno Sevilla, D. et al. 967 (*pubescens*)

Job, M.M. 874, 1210 (*chacoense*)

Jörgensen, P. 4370 (*flexuosum*); 7679 (*rabenii*)

Johns et al. 142 (*coccineum*); 201, 343 (*baccatum* var. pendulum)

Johnson, A. 70 (*annuum* var. annuum)

Johnston, I.M. & C.H. Mueller 949 (*annuum* var. glabriusculum)

Johnston, J.R. 75 (*parvifolium*)

Jönsson, G. 330 a (*flexuosum*)

Jordán, C.G. et al. 517 (*caballeroi*)

Jørgensen, P.M. 56290 (*rhomboideum*)

Jørgensen, P.M. et al. 1401, 1476, 1501 (*geminifolium*); 61307 (*rhomboideum*)

Joseph, A. 28 (*annuum* var. glabriusculum)

Josse, C. 480, 487, 539 (*rhomboideum*)

Joyal, E. 1815, 1926, 1931 (*annuum* var. glabriusculum)

Joyal, E. et al. 1761 (*annuum* var. glabriusculum)

Juárez de Varela, F. 2076 (*baccatum* var. baccatum)

Juárez García, G. 1630 (*annuum* var. glabriusculum)

Juárez García, G. et al. 2678 (*annuum* var. glabriusculum)

Judd, W.S. et al. 8388 (*annuum* var. glabriusculum)

Juncosa, A. 2018, 2228, 2236 (*rhomboideum*)

Juncosa, A. & G. Misas 1071 (*dimorphum*)

Jung, S.L. & A. Barros 374 (*recurvatum*)

Junge, C. 5755 (*annuum* var. annuum)

Jung-Mendacolli, S.L. et al. 1421 (*flexuosum*)

Jurgensen, C. 861 (*annuum* var. glabriusculum)

Kalliola, R. et al. P3-051 (*coccineum*)

Katán, T. 349 (*geminifolium*)

Kayap, R. 652 (*chinense*)

Kegler, A. 1256, 1354, 1426, 1565 (*flexuosum*)

Keller, H.A. 288, 1144, 1349, 2957, 5570 (*flexuosum*); 8340 (*baccatum* var. baccatum)

Keller, H.A. & A. Ferreira 2135 (*flexuosum*)

Keller, H.A. & M. Franco 4940 (*baccatum* var. baccatum)

Keller, H.A. & N.G. Paredes 7798 (*flexuosum*)

Keller, H.A. et al. 1945 (*flexuosum*); 5232 (*baccatum* var. baccatum); 6943 (*pubescens*); 10099 (*flexuosum*)

Kellerman, W.A. 4854, 7674 (*annuum* var. glabriusculum)

Kelly, I. 858 (*annuum* var. glabriusculum)

Kermes, E. 500 (*baccatum* var. baccatum)

Kessler, M. 2496 (*rhomboideum*); 2712 (*hookerianum*)

Kiesling, R. 8964 (*chacoense*)

Killeen, T. et al. 3415 (*coccineum*)

Killip, E.P. 93 (*annuum* var. glabriusculum); 605 (*rhomboideum*); 5395 (*annuum* var. glabriusculum); 5911, 34823 (*lycianthoides*)

Killip, E.P. & H. García 33781 (*lycianthoides*)

Killip, E.P. & T.E. Hazen 11019 (*annuum* var. glabriusculum)

Killip, E.P. & A.C. Smith 14251, 14252, 14534 (*annuum* var. glabriusculum); 20853 (*rhomboideum*); 22637 (*baccatum* var. pendulum); 23023, 26351, 26386 (*coccineum*); 27820 (*pubescens*); 28864 (*baccatum* var. pendulum); 42473 (*annuum* var. glabriusculum)

Killip, E.P. et al. 11069, 38142, 38146 (*rhomboideum*); 39889 (*dimorphum*)

King, D.O. 284 (*chacoense*)

King, R.M. 1525, 1613 (*rhomboideum*)

Kinupp, V.F. et al. 3169, 3229 (*baccatum* var. baccatum)

Kirizawa, M. 2087 (*cornutum*)

Kirizawa, M. & J.A. Correa 2164 (*recurvatum*)

Kirizawa, M. et al. 771, 2551 (*cornutum*); 3288 (*villosum*); 4321 (*cornutum*)

Klein, R.M. 2063 (*rabenii*); 2232 (*recurvatum*); 2774 (*rabenii*); 3190, 5628, 8050 (*flexuosum*)

Klein, R.M. & A. Bresolin 6447 (*frutescens*); 8346, 9216 (*baccatum* var. baccatum)

Klitgaard, B.B. & G.P. Lewis 386 (*rhomboideum*)

Klitgaard, B.B. et al. 411 (*lycianthoides*)

Klug, G. 4248 (*coccineum*)

Knab-Vispo, C. et al. 1288 (*annuum* var. glabriusculum)

Knapp, S. 5946 (*annuum* var. glabriusculum); 6164 (*rhomboideum*)

Knapp, S. & J. Mallet 3899, 6254 (*annuum* var. glabriusculum)

Knapp, S. et al. 3342 (*annuum* var. glabriusculum); 9103 (*pubescens*)

Knight, D.H. 446, 447 (*rhomboideum*); 641 (*lycianthoides*); 764 (*rhomboideum*)

Knuden, J.T. & B.Ståhl 381, 439 (*lycianthoides*)

Koch, S.D. & P.A. Fryxell 83123 (*annuum* var. glabriusculum)

Koch, S.D. et al. 79532 (*annuum* var. glabriusculum)

Køie, M.E. 5149 (*rhomboideum*)

Kohn, E. 1225 (*annuum* var. annuum); 1611, 1612, 1613, 1896, 1910 (*chinense*)

Kollmann, L. et al. 3497 (*baccatum* var. baccatum)

Korte, A. & A. Kniess 2051 (*flexuosum*)

Kozera, C. et al. 128 (*flexuosum*)

Kral, R. 1884, 61242 (*annuum* var. glabriusculum)

Krapovickas, A. 762, 1215 (*chacoense*); 1575 (*baccatum* var. baccatum); 1709, 1905, 6021, 6093, 6671 (*chacoense*); 8435 (*frutescens*); 12446 (*baccatum* var. baccatum); 15554 (*annuum* var. glabriusculum); 22180, 23686 (*annuum* var. annuum); 47426, 47449, 47466 (*eximium*)

Krapovickas, A. & C. Cristóbal 11848 (*baccatum* var. baccatum); 34271 (*baccatum* var. pendulum); 35425 (*schottianum*); 40806 (*annuum* var. annuum); 44232 (*chacoense*)

Krapovickas & A. Schinini 28514 (*baccatum* var. baccatum); 30421, 30457, 30494, 30655 (*chacoense*); 30952 (*baccatum* var. baccatum); 31054, 31056 (*baccatum* var. pendulum); 31057 (*annuum* var. annuum); 31154 (*chacoense*); 31459 (*baccatum* var. baccatum); 31659 (*baccatum* var. pendulum); 31689 (*frutescens*); 31721 (*baccatum* var. pendulum); 32132 (*frutescens*); 32133, 32141 (*baccatum* var. pendulum); 32144, 32244, 32360 (*baccatum* var. baccatum); 32488 (*chinense*); 32764 (*annuum* var. annuum); 34819, 36348 (*frutescens*); 36971 (*flexuosum*); 39010, 39012, 39018 (*baccatum* var. baccatum); 39321 (*baccatum* var. pendulum); 39244 (*baccatum* var. umbilicatum)

Krapovickas, A. et al. 13996, 14230 (*baccatum* var. baccatum); 14900 (*chacoense*); 18340, 21558 (*flexuosum*); 23427 (*frutescens*); 25312 (*flexuosum*); 25551 (*baccatum* var. baccatum); 27442, 27769, 27879, 27991, 28010, 28162, 28164 (*chacoense*); 41070 (*baccatum* var. baccatum); 45375 (*chacoense*); 47263 (*baccatum* var. baccatum); 47484 (*eximium*)

Krieger, L. 34 (*frutescens*); 7470 (*rabenii*); 13410 (*campylopodium*); 21364 (*mirabile*); 22976 (*frutescens*); 22981 pp. (*frutescens*); 22981 pp. (*chinense*)

Krukoff, B.A. 10584 (*ceratocalyx*)

Kruse, H. 1906 (*annuum* var. glabriusculum)

Kufer, J. 99 (*annuum* var. glabriusculum); 100 (*frutescens*)

Kuhlmann, J.G. 722 (*annuum* var. glabriusculum); 6288 (*campylopodium*)

Kuhlmann, M. 144 (*flexuosum*); 272 (*recurvatum*); 665 (*flexuosum*); 1710 (*schottianum*); 2643 (*recurvatum*); 2741 (*villosum*); 2785 (*hunzikerianum*); 2809 (*recurvatum*); 3026 (*baccatum* var. baccatum); 3556 (*recurvatum*); 4311 (*hunzikerianum*)

Kuhlmann, M. & S. Jimbo 80 (*annuum* var. glabriusculum)

Kummritz, S. & D. Ohashi 99 (*flexuosum*)

Kummrow, R.1307, 2449 (*flexuosum*)

Kuntz et al. 78 (*chacoense*)

Kuntze, O. 487 (*annuum* var. annuum); 1235 (*rhomboideum*); 1811 (*parvifolium*)

Kurtz, F. 192, 958, 4221, 4615, 6503, 8510, 16056 (*chacoense*)

Kvist, L.P. & E. Asanza 40201, 40356, 40456, 40565 (*frutescens*); 40566, 40586 (*chinense*)

La Rotta, C. 147, 273 (*frutescens*)

La Rotta, C. & H. Martínez 737 (*annuum* var. annuum)

La Rotta, C. et al. 435 (*chinense*)

Labiak, P.& S.R. Ziller 403 (*flexuosum*)

Lacerda, A.V. & F.M. Barbosa 415, 471, 486 (*parvifolium*)

Lakela, O.K. 25539, 27780a (*annuum* var. glabriusculum)

Lakela, O.K. & F. Almeda 30154 (*annuum* var. glabriusculum)

Lakela, O.K. & D. Laker 29097 (*annuum* var. glabriusculum)

Lakela, O.K. et al. 25447 (*annuum* var. glabriusculum)

Lamboray, O. 22 (*annuum* var. annuum)

Landrum, L.R. 2881 (*recurvatum*)

Landrum, L.R. et al. 10868 (*rhomboideum*)

Lanfranchi, A.E. 1125, 1144 (*chacoense*)

Langlassé, F. 16 (*rhomboideum*)

Lanjouw, J. 1040 (*frutescens*)

Lanjouw, L. & J.C. Lindeman 1462 (*annuum* var. glabriusculum)

Lanna, J. & A. Castellanos 26542 (*rabenii*)

Lanstyak, L. 49 (*villosum*)

Lara, M.E. 191 (*rhomboideum*)

Lasseigne, A.A. 4450 (*rhomboideum*)

Laughlin, R.M. 1116 (*annuum* var. annuum); 1296 (*annuum* var. glabriusculum); 1324 (*pubescens*); 1325, 1667, 2012 (*annuum* var. glabriusculum); 2013 (*annuum* var. annuum); 2018 (*frutescens*); 2117, 2118, 2119, 2652, 2653 (*annuum* var. annuum); 2654 (*annuum* var. glabriusculum); 2657 (*annuum* var. annuum); 2937 (*pubescens*); 3017, 3018 (*annuum* var. annuum)

Lawesson, J.E. et al. 39639, 39640, 39646 (*chinense*)

Lazor, R.L. et al. 2525 (*annuum* var. glabriusculum)

Leavenworth, W.M. & H. Hoogstrael 1339 (*annuum* var. glabriusculum)

Legname, P.R. 49, 117, 165, 204, 5786, 7218, 7316, 8186 (*chacoense*)

Legname, P.R. & A.R. Cuezzo 7204 C (*eximium*)

Legname, P.R. et al. 5322 (*chacoense*); 5901 (*baccatum* var. baccatum); 7356 (*flexuosum*)

Lehmann, F.C. 233, 234, 438 (*rhomboideum*); 2603 (*dimorphum*); 3577 (*annuum* var. annuum); 4730 (*annuum* var. glabriusculum); 4732, 4745 (*rhomboideum*); 4949 (*pubescens*)

Leisner, R.L. & V. Medina 13444 (*rhomboideum*)

Leitão, H.F. et al. 1997 (*flexuosum*)

Leitão Filho, H.F. et al. 9546, 9725 (*carassense*); 33146 (*recurvatum*)

Leite, J.E. 745 (*flexuosum*); 3792 (*mirabile*)

Leiva González, S. 6531, 6580, 6581 (*longifolium*)

Leiva González, S. & G.E. Barboza 5647 (*geminifolium*); 6555 (*annuum* var. glabriusculum); 6561, 6562 (*piuranum*); 6576 (*geminifolium*); 6585 (*rhomboideum*)

Leiva González, S. et al. 720 (*frutescens*); 834 (*annuum* var. glabriusculum); 1214, 1216 (*baccatum* var. pendulum); 1320 (*chinense*); 1427, 1429 (*pubescens*); 1740, 2018 (*geminifolium*); 2105 (*annuum* var. glabriusculum); 2788 (*geminifolium*)

Lélis, N.S. et al. 1, 2, 12 (*chinense*)

Lemes, F.O.A. & G.P. Freitas 602 (*villosum*)

León, Bro. 626, 7855 (*annuum* var. glabriusculum)

León, H.A. 20 (*chinense*); 178, 278, 591 (*annuum* var. glabriusculum)

León de Luz, J.L. 2019 (*annuum* var. glabriusculum)

León S., S. 036 (*annuum* var. annuum)

Leonard, E.C. 3318, 3461, 8495 (*annuum* var. glabriusculum); 9700 (*frutescens*); 11113 (*annuum* var. glabriusculum)

Leonard, E.C. & G.M. Leonard 12089, 14735 (*annuum* var. glabriusculum)

Leoni, L.S. 3625 (*mirabile*)

Letterman, G.W. 96 (*annuum* var. glabriusculum)

Lévy, P. 31, 1087 (*annuum* var. glabriusculum)

Lewis, G.P. & B.B. Klitgaard 3730 (*rhomboideum*)

Lewis, M. 88629 (*pubescens*)

Lewis, W.H. 2925, 2980 (*chinense*); 7187, 7407 (*annuum* var. glabriusculum); 14011 (*frutescens*)

Lewis, W.H. et al. 839, 2939 (*annuum* var. glabriusculum); 10922 (*annuum* var. annuum); 11196 (*annuum* var. glabriusculum); 11207 (*chinense*); 12552, 12906 (*annuum* var. glabriusculum); 13607, 13960, 14010 (*chinense*)

Liberman, M. et al. 1833 (*eximium*); 1883 (*chacoense*); 2051 (*baccatum* var. baccatum); 2052 (*eximium*)

Liesner, R.L. 2239, 4432 (*annuum* var. glabriusculum); 20013 (*frutescens*)

Liesner, R.L. & A. C. González 5735 (*annuum* var. glabriusculum); 10728 (*rhomboideum*); 12024 (*annuum* var. glabriusculum)

Liesner, R.L. & G. Vega 2885 (*annuum* var. glabriusculum)

Liesner, R.L. et al. 5417, 7613 (*annuum* var. glabriusculum)

Lighthipe, L.H. 738 (*annuum* var. glabriusculum)

Lillo, M. 325, 327, 610, 1949, 2533 (*chacoense*); 3850 (*baccatum* var. baccatum); 6206 (*chacoense*)

Lima, A.S. & L. da Silva 5894 (*schottianum*)

Lima, C.T. et al. 38 (*caatingae*)

Lima, I.B. & J.R. Lima 526 (*parvifolium*)

Lima, J.M. 332 (*baccatum* var. pendulum)

Lima, L.R. et al. 389 (*schottianum*)

Lima, R.A.F. 420 (*recurvatum*)

Lima, V.C. et al. 75 (*parvifolium*)

Linares, E. 846 (*annuum* var. annuum)

Linares, J.L. 556 (*frutescens*); 1840, 3856, 6186 (*rhomboideum*)

Linares, J.L. & C.A. Martínez 859 (*annuum* var. glabriusculum)

Lindberg, G.A. 176d, 177 (*rabenii*)

Lindeman, J.C. & J.H. de Haas 578, 3581, 5029 (*flexuosum*)

Lindheimer, F. 482, 1036 (*annuum* var. glabriusculum)

Linneo F., I. 476 (*baccatum* var. pendulum)

Linneo F., I. et al. 1558 (*minutiflorum*)

Lins, E. 3 (*caatingae*)

Liogier, A.H. & P. Liogier 18713, 19207, 23115, 23679, 24230 (*annuum* var. glabriusculum)

Liogier, A.H. et al. 28206, 31084, 32113, 32122, 34006, 34488 (*annuum* var. glabriusculum)

Lira Neto, J.A. et al. 444 (*campylopodium*)

Llanos, F. 421 (*rhomboideum*)

Llanos H., F. & J. Camacho 1827 (*annuum* var. glabriusculum)

Llatas Quiroz, S. 1745 (*geminifolium*); 3452 (*frutescens*); 3454 (*chinense*); 4147 (*frutescens*); 4300, 4650 (*annuum* var. annuum)

Lleras Perez, E. 2135 (*rabenii*)

Lleras Pérez, E. et al. 1942, 1944 (*rabenii*); 1947, 1956, 1957, 1961, 1964, 1970, 1973 a, 1974, 1975, 1976, 1981, 1989 (*flexuosum*); 1992, 1993, 1994, 1995 (*baccatum* var. pendulum); 2009, 2010, 2015, 2020, 2023, 2029 (*recurvatum*); 2036, 2037 (*flexuosum*); 2047 (*rabenii*); 2050, 2140 (*mirabile*); 2142 (*rabenii*); 2152, 2161, 2172 (*campylopodium*); 2179, 2181 (*pereirae*)

Lloyd, C.G. 435 (*annuum* var. glabriusculum)

Loefgren, A. 438 (*rabenii*); 4321 (*flexuosum*); 5878 (*villosum*); 15427, 15430 (*flexuosum*)

Løjtnant, B. & U. Molau 14076 (*rhomboideum*)

Lombardi, J.A. 1570 (*baccatum* var. baccatum)

Lombardi, J.A. et al. 6217 (*hunzikerianum*); 8320 (*recurvatum*); 8333 (*mirabile*)

Lomelí Sención, J.A. 2908 (*annuum* var. glabriusculum)

Long, R.W. & R.P. Wunderlin 4079 (*annuum* var. glabriusculum)

Long, R.W. et al. 2231, 2271 (*annuum* var. glabriusculum)

Lopes et al. 1422 (*caatingae*)

Lópes, M. et al. 416 (*mirabile*)

López, A. c14 (*rhomboideum*)

López, A. & E. Saravia 203 (*eximium*)

López, A. et al. 4252 (*annuum* var. glabriusculum)

López, C.A. 83 (*annuum* var. glabriusculum); 99 (*dimorphum*)

López, J.C. et al. 78 (*pereirae*)

López, R. et al. 1756, 5943, 5997 (*frutescens*)

López Figueiras, T.M. 45, 223 (*annuum* var. glabriusculum)

López Jurado, G. & J.S. Riascos 511 (*rhomboideum*); 613 (*pubescens*)

López L., L. & G.J. Martín 135 (*pubescens*)

Lopez Luna, R. 0579 (*frutescens*)

López M., A. 7873 (*annuum* var. annuum)

López Moreno, J. & E. Cedillo Portugal 29 (*annuum* var. annuum)

López-Palacios, S. 1302, 2137 (*rhomboideum*); 2143 (*annuum* var. glabriusculum)

López-Palacios, S. & J.A. Bautista B. 3473 (*pubescens*); 3557 (*rhomboideum*)

López-Palacios, S. & J.M. Idrobo 3828 (*annuum* var. glabriusculum)

Lorence, D.H. 5560 (*annuum* var. glabriusculum)

Lorence, D.H. & R. Cedillo 3078 (*annuum* var. glabriusculum)

Lorentz, P.G. 105, 1318 (*chacoense*)

Lorentz, P.G. & G. Hieronymus 240 (*baccatum* var. baccatum)

Lorenzi, H. et al. 5656 (*flexuosum*)

Lossen, W. 323 (*chacoense*)

Losser, G. & L. Aristeguieta 3438 (*rhomboideum*)

Lot, A. et al. 2075 (*annuum* var. glabriusculum)

Lott, E.J. 711 (*annuum* var. glabriusculum)

Lott, E.J. & J.A. Magallanes 659 (*annuum* var. glabriusculum)

Lozano et al. 577A (*chinense*)

Lozano, G. et al. 642 (*chinense*); 4820 (*rhomboideum*)

Lozano, P. et al. 942 (*rhomboideum*)

Lozano, R. 1207 (*neei*)

Lozano C., G. 3270, 4036 (*dimorphum*)

Lozano C., G. & R. Schnetter 2925 (*annuum* var. glabriusculum)

Lozano C., G. et al. 575, 5167 (*frutescens*)

Lucas, E.J. et al. 667 (*mirabile*)

Lucas, J. 135 (*lanceolatum*)

Lucca, C.F. et al. 18 (*caatingae*)

Lucena, R.F. & U.P. Alburquerque 91, 92 (*parvifolium*)

Lucena, R.F. & A.C. Silva 227, 228 (*parvifolium*)

Lugo S., H. 1249, 2444 (*rhomboideum*)

Luján, M.C. 327 (*chacoense*)

Luna, A. 356 (*annuum* var. glabriusculum)

Luna, F.E. 957 (*chacoense*)

Lundell, C.L. 87 (*frutescens*); 3754 (*annuum* var. annuum); 15376 (*annuum* var. glabriusculum); 16480 (*frutescens*)

Lundell, C.L. & E. Contreras 19773, 20417, 20971 (*lanceolatum*)

Lundell, C.L. & A.A. Lundell 7998 (*annuum* var. glabriusculum)

Lundell, P.H. 4766 (*frutescens*)

Luschnath de la Tour, J.B.L.T. 3098 (*annuum* var. glabriusculum)

Luteyn, J.L. 12554 (*dimorphum*)

Luteyn, J.L. & R. Callejas 11796 (*dimorphum*)

Luteyn, J.L. & O. Rangel 13146 (*dimorphum*)

Luteyn, J.L. et al. 6595 (*geminifolium*); 12547 (*lycianthoides*)

Luti, R. & E. Bücher 4004 (*chacoense*)

Lutz, B. 1007 (*mirabile*)

Lyra-Lemos, R.P. 11567 (*caatingae*)

Lyra-Lemos, R.P. & E.M. Duarte 5771 (*caatingae*)

Lyra-Lemos et al., R.P. 6827, 9402, 13147, 13177, 14027 (*caatingae*)

Maas, P.J. & P. Carauta 3275 (*campylopodium*)

Macbride, J.F. 4831 (*geminifolium*)

MacDougal, J.M. & F.J. Roldán 3547 (*lycianthoides*); 3557 (*dimorphum*)

Macedo, A. 3079 (*schottianum*)

Macedo, I.C.C. 5 (*flexuosum*)

Machado, R.F. et al. 271 (*baccatum* var. baccatum); 368 (*longidentatum*)

Machado, T.M. et al. 297 (*mirabile*)

Machado, W.J. & J.B. Jesus 738, 787, 800 (*caatingae*)

Machuca, J.A. N. 6889 (*rhomboideum*)

Madison, M.T. et al. 4675 (*annuum* var. annuum); 4967 (*annuum* var. glabriusculum)

Madrigal Sánchez, X. 4875 (*annuum* var. glabriusculum)

Madsen, E.B. 84312 (*rhomboideum*)

Madsen, J.E. 50214 (*rhomboideum*); 63565 (*frutescens*); 85610, 87010 (*rhomboideum*)

Madsen, J.E. & L. Ellemann 75101 (*dimorphum*)

Madsen, J.E. et al. 7040 (*frutescens*); 7601 (*geminifolium*)

Magallanes, J.A. 2691 (*annuum* var. glabriusculum)

Magaña, M.A. 1579 (*annuum* var. glabriusculum); 1699 (*annuum* var. annuum); 1957 (*frutescens*); 2006 (*lanceolatum*); 2326 (*annuum* var. glabriusculum)

Magaña Rueda, P. et al. 340 (*annuum* var. glabriusculum)

Magnoni 10 (*chacoense*)

Malda B., G. 111 (*annuum* var. glabriusculum)

Maldonado B., R. 504, 1250 (*chacoense*)

Malme, R. 156 (*chacoense*)

Málvarez, M.R. 583 (*chacoense*)

Mamaní M., F. 583 (*chacoense*)

Mamaní M., F. et al. 1435 (*baccatum* var. baccatum)

Manchego, C. CBP 01, 03, 04, 05, 11, 12, 13, 14, 15, 16, 17, 18 (*baccatum* var. baccatum); CBNP 01, 02, 03, 04, 05, 06 (*baccatum* var. baccatum); CBP A1 (*baccatum* var. baccatum); CBP T1, T2, T4, T7, T8, T9, T10, T16, T17, T18, T19, T20, T29 (*baccatum* var. baccatum); CBP NT2, NT3, NT5 (*baccatum* var. baccatum); CCP 01, CCP 03, CCP A1, CCP T5, CCP T6, CCP T11, CCP T13, CCP T14, CCP T25, CCP T26, CCP T27, CCP T28, CCP NT 4, CCNP A1, CCNP NT s.n., CCNP T3, CCNP T12, CCNP T15, CCNP T23, CCNP T24 (*chacoense*); CEP T21, CENP T22 (*eximium*)

Manchego, C. & M. Simon 60062B, 60065B, 60068B, 60076B, 60087B (*chacoense*)

Manchego, C. et al. 60194B, 60198B (*chacoense*)

Mandon, G. 427 (*eximium*); 428 (*pubescens*)

Maranta, A. 185 (*chacoense*)

Marchessi, E. et al. 1205 (*chacoense*)

Marín, C. et al. 2482 (*frutescens*)

Marín, C. & F. Rodríguez 504, 509 (*frutescens*); 525 (*annuum* var. annuum)

Marín, G. et al. 776 (*flexuosum*)

Markgraf, F. & A.C. Brade 3753 (*villosum*)

Marques de Lima, J. 273 (*rabenii*)

Marquete, R. et al. 260 (*campylopodium*)

Marquez R., W. 472 (*pubescens*)

Márquez, S. & B. Vecino 119 (*annuum* var. annuum)

Marrugo G., J.C. 296 (*annuum* var. glabriusculum); 1021 (*geminifolium*)

Marrugo G., J.C. & J.M. Vélez P. 55, 265 (*dimorphum*)

Marrugo G., J.C. et al. 709 (*rhomboideum*)

Marsh, E. 256 (*annuum* var. glabriusculum)

Marshall, S.A. & D.A. Neill 6624 (*annuum* var. glabriusculum)

Martin, R.T. 75, 168 (*chinense*)

Martin, R.T. & C.A. Lau-Cam 1242 (*frutescens*)

Martinelli, G. 1107 (*schottianum*)

Martinelli, G. et al. 2461, 2472, 2492 (*mirabile*); 10405 (*campylopodium*)

Martínez, G.J. 136, 190, 1185 (*chacoense*)

Martínez, M. 1145 (*annuum* var. glabriusculum); 1329, 2183, 3431 (*rhomboideum*); 5105 (*annuum* var. glabriusculum); 5332 (*annuum* var. glabriusculum); 5243 (*rhomboideum*)

Martínez, O. 85 (*rhomboideum*)

Martínez A., M.A. 346 (*frutescens*)

Martínez C., G. 1287 (*annuum* var. glabriusculum); 1643 (*annuum* var. annuum);1897, 2011, 3087, 3094 (*annuum* var. glabriusculum)

Martínez Crovetto, R. 31 (*chacoense*); 100 (*baccatum* var. baccatum); 135 (*flexuosum*); 11125 (*baccatum* var. baccatum)

Martínez Crovetto, R. & A. Schinini 10857 (*baccatum* var. baccatum)

Martínez Gallegos, J.F. 79 (*rhomboideum*)

Martínez Ojeda, E. 263 (*annuum* var. glabriusculum)

Martínez R., C. 44 (*rhomboideum*); 76 (*annuum* var. glabriusculum)

Martínez S., E. 2262 (*annuum* var. glabriusculum); 9066 (*lanceolatum*); 11240, 16073, 29417 (*frutescens*)

Martínez S., E. & A. García 22066 (*annuum* var. glabriusculum)

Martínez S., E. & A. Ibarra 40296 (*annuum* var. glabriusculum)

Martínez S., E. & F. Martínez 6316 (*frutescens*)

Martínez S., E. & L. Rico 6062 (*annuum* var. glabriusculum)

Martínez S., E. & J.C. Soto 3640 (*annuum* var. annuum)

Martínez S., E. et al. 246 (*chinense*); 4476 (*annuum* var. glabriusculum); 23039 (*lanceolatum*); 29428 (*annuum* var. annuum); 29562, 36224 (*annuum* var. glabriusculum)

Martínez Torres, H.L. 57, 82, 98 (*annuum* var. glabriusculum)

Martins, C. 242 (*caatingae*)

Marulanda, O. 395 (*dimorphum*)

Marulanda, O. & S. Churchill 490 (*chinense*)

Maruñak, V. et al. 564, 626 (*chacoense*)

Mateus Gutierrez, C. 28 (*annuum* var. glabriusculum)

Mathews 854 (*pubescens*)

Matos, F. 1076 (*rhomboideum*)

Matos, G.M.A. et al. 308 (*caatingae*)

Matos, J.R. 32 (*frutescens*)

Matthes, B. 139 (*annuum* var. glabriusculum)

Mattos, J. 11459 (*schottianum*); 12156 (*rabenii*); 12165 (*mirabile*); 12778 (*cornutum*)

Mattos, J. & M. Mattos 14254 (*hunzikerianum*); 14776 (*villosum*)

Matuda E. 247 (*annuum* var. glabriusculum); 2406 (*pubescens*); 4656, 4868 (*rhomboideum*); 5177 (*lanceolatum*); 6976 (*rhomboideum*); 17590 (*annuum* var. glabriusculum); 17642 (*frutescens*); 18120 (*annuum* var. glabriusculum)

Maturo, H. 245 (*chacoense*)

Maxon, W.R. 2160 (*frutescens*)

May, O. 39 (*annuum* var. annuum)

Mayer, M. 24 (*parvifolium*)

Mayworm, M.A. et al. 135 (*cornutum*)

McCart, W. L. 11158 (*annuum* var. glabriusculum)

McCarten, N.F. & R.L. Bittman 2585 (*rhomboideum*)

McDaniel, S. 14075 (*coccineum*); 27181 (*annuum* var. glabriusculum)

McDaniel, F. & L. Santiago 2556 (*coccineum*)

McDaniels, L.H. 698 (*pubescens*)

McDowell, T. et al. 796 (*annuum* var. annuum); 1940, 2174 (*annuum* var. glabriusculum)

McVaugh, R. 23267 (*rhomboideum*)

Meave del Castillo, J. & E. Pérez-García 1837, 2350 (*annuum* var. glabriusculum)

Medina A., M.E. & F. Vazquez B. 542 (*rhomboideum*)

Medina E. 939 (*annuum* var. glabriusculum)

Medrano, F.G. et al. 1823 (*annuum* var. glabriusculum)

Meier, W. et al. 6621 (*annuum* var. glabriusculum)

Meireles, L.D. et al. 1047 (*recurvatum*)

Mejía, F. 001 (*annuum* var. annuum); 002 (*pubescens*)

Mejía, M.M. 183 (*annuum* var. glabriusculum)

Mejía, M.M. & C. Ramírez 11039, 11137 (*annuum* var. glabriusculum)

Mejía, M.M. & T. Zanoni 6351, 6619, 7524, 8100 (*annuum* var. glabriusculum)

Mejía, M.M. et al. 8925, 10386, 10396, 10490 (*annuum* var. glabriusculum)

Mejía Pimentel, M. & T. Zanoni 7738 (*annuum* var. annuum)

Melo, E. 9364 (*longidentatum*); 11840, 11878, 13140 (*caatingae*)

Melo, E. & B. Marques da Silva 6081 (*longidentatum*)

Melo, E. et al. 1859, 1920 (*longidentatum*); 1969 (*caatingae*); 3646 (*parvifolium*); 3862, 4336 (*caatingae*); 4344 (*longidentatum*); 4693 (*longidentatum*); 5440 (*caatingae*); 5571 (*baccatum* var. baccatum); 5658 (*parvifolium*); 5664 (*baccatum* var. baccatum); 5926, 6230, 6295 (*caatingae*); 6545 (*rabenii*); 6909 (*longidentatum*); 7695 (*parvifolium*); 7813 (*flexuosum*)

Melo, G.F.A. et al. 221 (*parvifolium*)

Melo, P.H.A. 5030 (*baccatum* var. baccatum)

Melo, P.H.A. & J.A. Lombardi 362 (*baccatum* var. baccatum); 381 (*rabenii*)

Melo, P.H.A. & T.R. Peixoto 6080 (*longidentatum*)

Mencho, M. & E. Gutiérrez 586 (*baccatum* var. baccatum)

Mendes, M.R.A. & R.S. Albino 509 (*parvifolium*)

Mendes, M.R.A. et al. 292, 539 (*parvifolium*)

Méndez, M. et al. 476 (*annuum* var. glabriusculum)

Méndez Girón, A. 7745 (*lanceolatum*)

Mendonça et al. 5971 (*baccatum* var. baccatum)

Mendoza, H. 661, 814 (*lycianthoides*); 1800 (*annuum* var. glabriusculum); 17394 (*dimorphum*)

Mendoza, H. et al. 14946, 15269 (*dimorphum*)

Mendoza, J.E. & L. Quevedo 407, 525 (*dimorphum*)

Mendoza, J.E. et al. 4202 (*lycianthoides*)

Mendoza, M. 788 (*chacoense*)

Mendoza, M. & M. Balcazar 2021 (*eximium*)

Mendoza, M. & E. Calzadilla 362, 363, 801 (*eximium*)

Mendoza, M. & R. Lozano 827, 828 (*eximium*)

Mendoza, M. & D. Vidal 1265 (*eshbaughii*)

Mendoza, M. et al. 2805 (*chacoense*)

Mendoza, W. et al. 6570 (*annuum* var. glabriusculum)

Meneces, E. & W. Terceros 1004 (*eximium*)

Mentz, L.A. et al. 263 (*baccatum* var. baccatum); 278 (*flexuosum*); 289 (*recurvatum*); 290 (*flexuosum*); 309, 400, 401 (*baccatum* var. baccatum)

Mercado, M. & R. Navia 1811 (*eximium*)

Mereles, F. 2920, 5536, 8651 (*chacoense*)

Mereles, F. & R. Degen 5136 (*chacoense*)

Mereles, F. & M. Soloaga 7568 (*baccatum* var. pendulum)

Mereles, F. et al. 7498 (*flexuosum*); 7499 (*baccatum* var. pendulum); 7502 (*baccatum* var. pendulum); 7503 (*flexuosum*)

Merino, B. & T. Delgado E-1340, E- 1569 (*geminifolium*)

Messias, M.C.T. & R. M. Carrillo 2443 (*baccatum* var. baccatum)

Metz, M.C. 111 (*annuum* var. glabriusculum)

Mexia, Y. 4148, 4205 (*rabenii*); 4328 (*villosum*); 4493 (*rabenii*); 7195 (*pubescens*); 8246 (*geminifolium*)

Meyer, F.G. & P.M. Mazzeo 11988 (*frutescens*)

Meyer, R. 13848 (*chacoense*)

Meyer, T. 92, 858, 2457, 2458 (*chacoense*); 5477 (*baccatum* var. baccatum); 5506 (*flexuosum*); 5976 (*baccatum* var. baccatum); 6694 (*flexuosum*); 6790 (*baccatum* var. baccatum); 8561, 9795 (*chacoense*); 11550 (*flexuosum*); 11918 (*baccatum* var. baccatum); 13128, 13325 (*chacoense*); 15922 (*baccatum* var. baccatum); 17527 (*eximium*); 18144 (*baccatum* var. baccatum); 19927 (*coccineum*); 20304 (*chacoense*)

Meyer, T. & A.R. Cuezzo 22440 (*baccatum* var. baccatum)

Meyer, T. et al. 9170C (*chacoense*)

Meza E., P. 2 (*annuum* var. glabriusculum)

Miers, J. 4541 pp (*campylopodium*); 4541 pp (*mirabile*)

Miguel, A. 2006 (*lanceolatum*)

Mill, H. 614 (*lanceolatum*)

Miller, G.S. 149 (*frutescens*); 198 (*annuum* var. glabriusculum)

Miller, G.S. Jr. 199 (*frutescens*)

Miller, J.S. et al. 2404 (*chinense*); 5792 (*annuum* var. glabriusculum)

Miller, J. & P. Yépez 651, 652, 667 (*chinense*)

Miller, O.O. & J.R. Johnston 35 (*rhomboideum*); 81 (*frutescens*); 82 (*annuum* var. glabriusculum); 255 (*parvifolium*)

Mione, T. 466 (*geminifolium*); 812 (*piuranum*)

Miranda, A. 8 (*frutescens*)

Miranda, A. et al. 1519, 1519 A, 1519 B (*annuum* var. annuum); 1644 (*annuum* var. glabriusculum)

Miranda, A.M. 6695 (*caatingae*)

Miranda, A.M. & Félix 1642 (*caatingae*)

Miranda, A.M. et al. 6148 (*parvifolium*)

Miranda, F. 4021 (*rhomboideum*); 5482 (*annuum* var. glabriusculum); 6374 (*rhomboideum*); 8097, 8224 (*annuum* var. glabriusculum)

Miranda-Moyano, N. 225 (*frutescens*)

Miranda-Moyano, N. & G. Moya 334 (*annuum* var. annuum); 436, 437 (*chinense*)

Moina, S. Z 23 (*pubescens*)

Molas, L. 792 (*flexuosum*)

Molas, L. & V. Vera 1067 (*chacoense*)

Moldenke, H.N. 727 (*annuum* var. glabriusculum)

Molina, A.M. & J. Hilfer 2788, 2993, 3046 (*chacoense*)

Molina, A.M. et al. 1328 (*chacoense*)

Molina, D. 34, 110 (*annuum* var. glabriusculum)

Molina R., A. 262 (*annuum* var. glabriusculum); 561 (*rhomboideum*); 594 (*annuum* var. glabriusculum); 1054, 1177 (*frutescens*); 1457, 1820, 2784, 3200, 3212, 3248, 3875 (*rhomboideum*); 22452, 23062 (*annuum* var. glabriusculum); 24525 (*chinense*); 34008 (*annuum* var. annuum)

Molina R., A. & A.R. Molina 14053 (*lanceolatum*); 34535 (*annuum* var. annuum)

Molina R., A. et al. 33572 (*annuum* var. annuum)

Monetti, L. 1975 (*chacoense*); 2257 (*eximium*)

Monro, A.K. et al. 2164 (*annuum* var. glabriusculum)

Monsalvo J., C.B. 12 (*pubescens*)

Monteagudo, A. et al. 3475 (*geminifolium*); 9371(*dimorphum*); 9470 (*geminifolium*); 13706 (*dimorphum*); 15087 (*geminifolium*)

Monteiro, H. 1918, 2276 (*baccatum* var. baccatum)

Montenegro, O. 137 (*frutescens*)

Montenegro, V. 1576 (*rhomboideum*)

Montenegro Valls, J.F. et al. 8782 (*chinense*)

Montes, J.E. 398, 1635, 1689, 2139, 2140, 2155, 2213, 2305 b, 2353, 2375, 2411, 2411b, 2476, 3280, 4026, 4277, 4289, 12314, 14727 (*flexuosum*); 15164, 15188, 15202 (*baccatum* var. baccatum); 15418 (*chacoense*); 15612, 15633, 15716, 15900 (*baccatum* var. baccatum); 16310, 16536, 27474, 27476 (*flexuosum*)

Moonlight, P.W. & T. Särkinen 709 (*parvifolium*)

Moore, A.D. 1546 (*annuum* var. glabriusculum)

Moore Jr., H.E. 1820, 3831 (*rhomboideum*)

Mora, L.E. 332 (*rhomboideum*)

Moraes, A.O. et al. 159, 160 (*caatingae*); 161 (*longidentatum*)

Moraes, M.S. 18 (*campylopodium*)

Moraes, M.V. 173 (*baccatum* var. baccatum); 790 (*caatingae*)

Morales, G. 2914 (*geminifolium*)

Morales, M. et al. 015 (*annuum* var. glabriusculum)

Mora-López, J.L. 134 (*annuum* var. glabriusculum)

Moreira, H.P. et al. 84 (*campylopodium*)

Morel, I. 452, 722, 776, 910, 1746, 1845, 2867, 3375, 4171, 4417, 6746, 7127, 7691, 8909 (*chacoense*)

Morello, J. & A.E. Cuezzo 157, 174 (*baccatum* var. baccatum); 284, 402, 1004 (*chacoense*)

Moreno, P.P. 733, 763, 788, 1180, 1240, 1271, 1408, 1480, 1544, 1605, 1725, 1791, 1891, 1926, 1948, 1949, 2037, 2089, 2123, 2170, 2243, 2313, 2348, 2481, 2544, 2636, 2685, 2738, 3044, 3292, 3590, 3727, 3919, 4220, 4328 (*annuum* var. glabriusculum); 10196, 21241, 21761, 21824, 21875 (*rhomboideum*); 22007 (*annuum* var. glabriusculum)

Moreno, P.P. & J. Henrich 8445, 8793 (*annuum* var. glabriusculum); 8954 (*annuum* var. annuum)

Moreno, P.P. & J.C. Sandino 12134 (*frutescens*); 12153 (*annuum* var. annuum)

Moreno V., D.I. 34 (*rhomboideum*)

Mori, S.A. et al. 11603 (*baccatum* var. baccatum)

Morillo, G. & R. Smith 6537 (*rhomboideum*)

Morillo, G. et al. 4297, 4299, 8448 (*rhomboideum*)

Morong, T. 388 (*baccatum* var. baccatum); 961 (*chacoense*)

Morrill, S. & J. Harvey 154 (*annuum* var. glabriusculum)

Morrone, O. 780 (*flexuosum*)

Morrone, O. et al. 932, 1444 (*flexuosum*); 2884 (*baccatum* var. baccatum)

Morrow, C.F. 10, 15 (*annuum* var. glabriusculum)

Morton, C.V. 4713, 10259 (*annuum* var. glabriusculum); 10907 (*eximium*)

Moscone, E.A. 98, 104 (*chacoense*); 148 (*flexuosum*); 194, 195 (*chacoense*); 197 (*baccatum* var. umbilicatum); 198 (*pubescens*); 200 (*frutescens*); 202 (*pubescens*); 204 (*annuum* var. annuum); 207 (*chacoense*); 229 (*flexuosum*); 237 (*frutescens*)

Moscone, E.A. & R. Neumann 192, 205, 206, 209 (*baccatum* var. pendulum); 210 (*baccatum* var. baccatum); 211 (*baccatum* var. pendulum); 212, 213 (*chacoense*); 214, 215 (*baccatum* var. baccatum)

Mosén, H. 984 (*baccatum* var. baccatum); 1563 (*schottianum*); 3443 (*recurvatum*)

Mostacedo, B. 495 (*minutiflorum*)

Mostacedo C., B. 2699 (*baccatum* var. baccatum); 2975 (*chacoense*)

Mostacero, J. et al. 1768 (*rhomboideum*)

Mota, R.C. 106, 2260, 2653 (*carassense*)

Moura, S. 1016 (*caatingae*)

Moya, J.G. 84 (*rhomboideum*)

Moya, G. & D. Reyes 206, 207 (*chinense*)

Múlgura, M. et al. 1779, 2112, 3322, 3391 (*flexuosum*)

Müller, C.H. 2674 (*rhomboideum*)

Müller, C.H. & M.T. Müller 131, 647 (*annuum* var. glabriusculum)

Müller, G. & P. Gutte 8422 (*frutescens*)

Müller, J.K. 174 (*annuum* var. annuum)

Muñoz, L. 257 (*annuum* var. annuum)

Muñoz C., E. 300 (*annuum* var. glabriusculum)

Múrcia, C. 129, 191 (*dimorphum*); 371 (*lycianthoides*); 470 (*dimorphum*)

Múrcia, G. 77, 77 B (*dimorphum*)

Mutis, J.C. 2010 (*dimorphum*); 3590 (*rhomboideum*)

Mynssen, C.M. et al. 1404 (*mirabile*)

Naboulet 140 (*chacoense*)

Nadal, F.H. et al. 28 (*friburgense*); 43 (*recurvatum*)

Nadruz, W.M. et al. 2251 (*campylopodium*)

Naranjo, D. & B. Freire 329 (*chinense*)

Narváez, C. 018 (*annuum* var. annuum)

Narvaez, E. 17 (*annuum* var. glabriusculum)

Narváez Montes, M. 861 (*annuum* var. glabriusculum)

Nash, G.V. 985, 1287 (*annuum* var. glabriusculum)

Nash, G.V. & N. Taylor 1456 (*annuum* var. glabriusculum)

Nates Parra, G. 214 (*annuum* var. glabriusculum); 225 (*rhomboideum*)

Nava Zafra, A. et al. 1812 (*annuum* var. glabriusculum)

Navarro, G. & I.G. Vargas C. 261 (*chacoense*)

Navarro, J.A. et al. 288 (*dimorphum*)

Navarro Sánchez, G. 1618 (*baccatum* var. baccatum)

Nee, M. 3424, 10103 (*baccatum* var. baccatum); 6789, 6989, 6990, 23723, 26700, 27745, 28210, 28288, 32699 (*annuum* var. glabriusculum); 33493, 33715 (*minutiflorum*); 34184 (*baccatum* var. baccatum); 34412 (*annuum* var. glabriusculum); 35427 (*minutiflorum*); 36049 (*coccineum*); 36164 (*eshbaughii*); 36304, 36514 (*baccatum* var. baccatum); 37233, 37305 (*coccineum*); 37575, 37585 (*eximium*); 37759 (*baccatum* var. baccatum); 38180 (*coccineum*); 38361 (*eximium*); 38899, 39360 (*coccineum*); 39545, 40110, 40103 (*minutiflorum*); 40886 (*coccineum*); 41131 (*frutescens*); 42027 (*minutiflorum*); 42191 (*baccatum* var. baccatum); 42218 (*minutiflorum*); 43027 (*frutescens*); 43167 (*baccatum* var. baccatum); 44503 (*minutiflorum*); 44837, 45842, 47937, 48439, 48489, 48553 (*baccatum* var. baccatum); 49201 (*minutiflorum*); 51786 (*pubescens*); 52197 (*eshbaughii*); 52917 (*minutiflorum*); 55645 (*frutescens*)

Nee, M. & L. Bohs 50775 (*baccatum* var. baccatum); 50807 (*eximium*)

Nee, M. & J.I. Calzada 33201 (*annuum* var. glabriusculum)

Nee, M. & G. Coimbra S. 33969 (*baccatum* var. baccatum)

Nee, M. & G. Diggs 24413 (*annuum* var. glabriusculum)

Nee, M. & R. Flores S. 54782 (*baccatum* var. baccatum); 54790, 54863, 54882 (*chacoense*)

Nee, M. & A. Molina O. 56085 (*chacoense*)

Nee, M. & S. Mori 4270, 4271 (*rhomboideum*)

Nee, M. & M. Saldías P. 35956, 36874 (*coccineum*)

Nee, M. & Solomon 30265 (*annuum* var. glabriusculum); 30265 (*frutescens*); 32018 (*pubescens*); 36558, 36578 (*eximium*)

Nee, M. & M. Sundue 51994 (*minutiflorum*)

Nee, M. & K. Taylor 25938 (*pubescens*); 28811 (*rhomboideum*)

Nee, M. & W.R. Téllez 28018 (*annuum* var. glabriusculum)

Nee, M. & I. Vargas C. 37448, 38323 (*eximium*); 43483 (*eshbaughii*); 44747 (*eximium*)

Nee, M. & S. Vega 27926 (*annuum* var. glabriusculum)

Nee, M. et al. 19489, 27750, 28175 (*annuum* var. glabriusculum); 47215, 47257 (*rhomboideum*); 51778 (*ceratocalyx*); 52407 (*caballeroi*); 55030 (*baccatum* var. baccatum); 55070 (*eximium*); 55227 (*frutescens*); 55779 (*chacoense*)

Neill, D. & T. Núñez 10491 (*hookerianum*)

Nelson, C. 3908 (*rhomboideum*)

Nelson, C. et al. 7102 (*annuum* var. glabriusculum)

Nelson, E.B. 4343, 5379, 7809 (*annuum* var. glabriusculum)

Nesom, G. et al. 6009 (*annuum* var. glabriusculum)

Neubert, H. 51, 216 (*flexuosum*)

Nevling, L. & A. Gómez Pompa 196 (*annuum* var. glabriusculum); 645 (*annuum* var. annuum); 2317 (*rhomboideum*)

Nichols, C.E. 1139 (*annuum* var. glabriusculum)

Nicolau, S.A. 1050 (*recurvatum*)

Nicolau, S.A. et al. 1237 (*villosum*)

Noblick, L.R. 3207 (*caatingae*)

Noblick, L.R. & Lemos 4227 (*caatingae*)

Norris, D.H. 17477 (*annuum* var. glabriusculum)

Northrop, J.I. & A.R. Northrop 256 (*annuum* var. glabriusculum)

Novara, L. & S. Bruno 9295 (*baccatum* var. baccatum); 9898 (*chacoense*)

Novara, L. 1936, 3069, 3353 (*chacoense*); 5102, 8346 (*eximium*); 8423 (*chacoense*)

Novara, L. & R. Neumann 3121 (*eximium*)

Novara, L. et al. 12205 (*baccatum* var. baccatum); 12206 (*eximium*); 12504 (*chacoense*)

Núñez V., P. 6203 (*coccineum*); 7537 (*pubescens*); 8600 (*baccatum* var. pendulum); 8720 (*pubescens*); 14114 (*coccineum*)

Núñez V., P. et al. 6780 (*annuum* var. glabriusculum); 6799 (*baccatum* var. pendulum); 10024 (*coccineum*); 16906 (*frutescens*)

Núñez, T. et al. 39 (*rhomboideum*); 336 (*annuum* var. glabriusculum)

O’Donell, C.A. 351, 901, 4328, 4410, 4436 (*chacoense*)

O’Donell, C.A. & J.M. Rodríguez V. 920, 978 (*chacoense*)

Obando, S. et al. 46 (*dimorphum*)

Ochoa, C. 602 (*pubescens*); 11995 (*baccatum* var. baccatum)

Odonne, G. 25 (*annuum* var. glabriusculum); 0522 (*frutescens*); 561, 563, 626, 627 (*annuum* var. glabriusculum); 0641 (*frutescens*); 664 (*annuum* var. glabriusculum)

Olivares Hernández, D. 1 (*annuum* var. glabriusculum)

Oliveira, A.R.S. et al. 675 (*coccineum*)

Oliveira, D.G. et al. 158 (*caatingae*)

Oliveira, G.B. 360, 371, 387, 395 (*pereirae*)

Oliveira, M. 7 (*caatingae*); 5795 (*parvifolium*)

Oliveira, M. & J.R. Silva 5862 (*parvifolium*)

Oliveira, M. et al. 5408 (*caatingae*)

Oliver, R.L. et al. 1106 (*annuum* var. glabriusculum)

Øllgaard, B. & H. Balslev 9348 (*geminifolium*)

Øllgaard, B. et al. 90357 (*dimorphum*)

Omawale & R. Persaud 130 (*annuum* var. annuum)

Orcutt, C.R. 626, 1467 (*annuum* var. glabriusculum)

Ordones, J. et al. 2229 (*carassense*)

Ordonez, M.J. 53, 239 (*annuum* var. glabriusculum)

Ordóñez, M.T. & H. Valencia 10 (*annuum* var. glabriusculum)

Ordóñez, P. 016 (*frutescens*)

Orejuela R., A. & E. Calderón Sáenz 166 (*lycianthoides*)

Orejuela R., A. 109, 174 (*lycianthoides*)

Orejuela R., A. et al. 2640 (*regale*); 2685 (*dimorphum*); 2688 (*geminifolium*); 3034, 3035 (*regale*)

Orlandi, R. 251 (*caatingae*)

Orozco, C.I. & R. Kuiru 173 (*annuum* var. glabriusculum)

Orozco, C.I. et al. 3371, 3675, 3717, 3838, 3863, 3865 (*dimorphum*); 3922, 3931, 3944 (*geminifolium*); 3960, 3972, 3989 (*lycianthoides*)

Orozco, O.L. 122 (*pubescens*)

Orozco-Segovia, A.D. 368 (*annuum* var. glabriusculum)

Ortega, A. et al. 51 (*frutescens*)

Ortega, H.J. & L.R. Ortega 865 (*annuum* var. glabriusculum)

Ortega, R. et al. 876 (*frutescens*)

Ortega, R.V. 494 (*rhomboideum*)

Ortega O., R. et al. 870 (*annuum* var. glabriusculum)

Ortega-Torres, L.M. 87 (*annuum* var. annuum); 88 (*annuum* var. glabriusculum)

Ortega-Torres, L.M. & J. Tzuc 272 (*annuum* var. glabriusculum)

Ortiz, C. et al. 3 (*caballeroi*)

Ortiz, E. & R. Francis J. 736 (*geminifolium*)

Ortiz, E.V. et al. 752 (*dimorphum*)

Ortiz, F.J. 11 (*lycianthoides*)

Ortíz, G. 01, 07, 20 (*annuum* var. annuum)

Ortiz, J.J. & S. Avendaño 1055 (*annuum* var. glabriusculum)

Ortiz, M. 1283 (*flexuosum*)

Ortiz, R. et al. 591 (*rhomboideum*)

Ortíz, R.T. 341 (*annuum* var. glabriusculum)

Ortuño, T. 271 (*cardenasii*); 769 (*chacoense*)

Otero J., J.I. 284 (*annuum* var. glabriusculum)

Ovando, A. 18 (*baccatum* var. baccatum)

Pabón, M.A. E. 257, 258, 259, 277 (*chinense*)

Pabst, G. & E. Pereira 7225 (*campylopodium*)

Pachano, A. 78 (*rhomboideum*)

Padilla, I. 219 (*annuum* var. glabriusculum)

Padilla, I. et al. 3088 (*frutescens*)

Paixão, J.L. 951 (*mirabile*)

Paixão, J.L. et al. 863 (*pereirae*)

Palacios, R.A. 733 (*chacoense*)

Palacios, W. 6318 b (*geminifolium*); 6837 (*rhomboideum*)

Palacios, W.A. & D. Rubio 9956 (*hookerianum*)

Palacios, W. & M. Tirado 13256 (*dimorphum*)

Palacios, W. & H. van der Werff 3591 (*lycianthoides*)

Palacios, W. et al. 9746 (*lycianthoides*)

Palacios E., E. 636 (*annuum* var. glabriusculum)

Palma Gutiérrez, J. 85-24, 85-25, 85-69 (*chinense*)

Palmer, E. 135 (*annuum* var. annuum), 135, 136 (*chinense*); 137, 138, 139 (*annuum* var. annuum); 144 (*annuum* var. glabriusculum); 173 (*rhomboideum*); 215, 230 (*annuum* var. glabriusculum); 272 (*rhomboideum*); 394 (*annuum* var. glabriusculum); 456 (*rhomboideum*); 638 (*annuum* var. glabriusculum); 639, 640, 642 (*annuum* var. annuum); 772, 931, 1135, 10789 (*annuum* var. glabriusculum)

Palombo, N. 3 (*baccatum* var. pendulum); 19 (*eshbaughii*); 21, 22, 23 (*pubescens*)

Panero, J.L. & I. Calzada 4029 (*rhomboideum*)

Parada, G.A. et al. 2760 (*eximium*); 3523 (*neei*); 4355 (*baccatum* var. baccatum); 4456 (*minutiflorum*); 5445 (*chacoense*)

Paredes, D. 845 (*annuum* var. annuum)

Parodi, L.R. 3102, 14133 (*chacoense*)

Parra V., G. 26, 089 (*rhomboideum*)

Pascali, A.H.G. 8 (*annuum* var. annuum)

Pascual, J. 1535 (*rhomboideum*)

Pastore, J.F. & R.M. Harley 2603 (*longidentatum*)

Patzlaff, R. 15 (*frutescens*)

Paula, M.F.R. et al. 18 (*recurvatum*)

Paula-Souza, J. 7254 (*baccatum* var. baccatum); 7981, 8175 (*chacoense*)

Paula-Souza, J. et al. 7336 (*flexuosum*)

Pavajeau, L. 60, 174 (*dimorphum*)

Pavetti, C. & T. Rojas 10716 (*flexuosum*); 10821 (*chacoense*)

Pedersen, T.M. 4670 (*chacoense*); 5315 (*baccatum* var. baccatum); 5517 (*chacoense*); 7712 (*baccatum* var. baccatum); 7787 (*schottianum*); 10239 (*chacoense*); 10996 (*flexuosum*); 15395 (*chacoense*); 15791 (*flexuosum*)

Pedralli, G. 87 (*flexuosum*)

Pedralli, G. et al. 3045 (*flexuosum*)

Pedraza-Peñalosa, P. et al. 59, 2201 (*dimorphum*)

Penereiro 11062 (*chinense*)

Penland, C.W. & R.H. Summers 44, 841 (*rhomboideum*)

Pennell, F.W. 2715 (*rhomboideum*); 9318, 10316 (*dimorphum*); 10989 (*annuum* var. glabriusculum); 17941 (*rhomboideum*); 19497 (*annuum* var. glabriusculum)

Pennell, F.W. & E.P. Killip 5761 (*dimorphum*)

Pennell, F.W. & H.H. Rusby 187 (*rhomboideum*)

Penneys, D. 502 (*pubescens*)

Pennington, T.D. 60SD (*rhomboideum*)

Pensiero, J. 5245 (*chacoense*)

Pensiero, J. & J. Tivano 3261 (*chacoense*)

Pensiero, J. et al. 7434, 7511 (*chacoense*)

Peña-Chocarro, M. 2536 (*chacoense*)

Peñaloza, H. 4856 (*frutescens*)

Peñaranda, J.A. 458 (*eximium*)

Peñaranda, J.A. et al. 817 (*eximium*)

Peredo, I. 348 (*baccatum* var. baccatum)

Pereira Duarte, A. 979 (*schottianum*)

Pereira, E. 2245 (*pereirae*); 5305 (*flexuosum*)

Pereira, E. et al. 4444 (*campylopodium*)

Pereira, F.G. et al. 406 (*recurvatum*)

Pereira, L.A. et al. 1672 (*frutescens*); 1699 (*chinense*); 1701, 1747, 1780, 1807 (*annuum* var. glabriusculum); 1809 (*frutescens*); 1816, 1817 (*chinense*); 1819 (*baccatum* var. pendulum); 1820 (*frutescens*); 1821 (*chinense*);1822 (*annuum* var. glabriusculum); 1824 (*baccatum* var. umbilicatum); 1827, 1828 (*chinense*); 1829 (*frutescens*); 1830 (*baccatum* var. umbilicatum); 1831, 1833 (*chinense*); 1834 (*baccatum* var. umbilicatum); 1835 (*chinense*); 1836 (*frutescens*); 1837, 1839 (*chinense*); 1840, 1841, 1842 (*annuum* var. glabriusculum); 1846, 1851, 1852 (*chinense*);1863 (*baccatum* var. pendulum); 1864 (*chinense*); 1865, 1866 (*frutescens*); 1867 (*annuum* var. glabriusculum); 1868, 1870, 1873, 1896 (*chinense*);1899 (*baccatum* var. pendulum); 1900 (*annuum* var. glabriusculum); 1901, 1902 (*chinense*); 1903 (*annuum* var. glabriusculum); 1904, 1905, 1906, 1908, 1918, 1919, 1920 (*chinense*)

Pereira, L.A. & W.M.S. Severino 1851 (*baccatum* var. pendulum); 1853 (*annuum* var. glabriusculum); 1854 (*chinense*); 1855 (*frutescens*)

Pereira, R. et al. 115, 2607 (*caatingae*)

Pereira, S.M. 3219 (*frutescens*)

Pereira-Silva, G. 9995 (*flexuosum*); 13172 (*frutescens*)

Pereira-Silva, G. & G.A. Moreira 12751 (*annuum* var. glabriusculum)

Pereira-Silva, G. et al. 2100, 4678 (*rabenii*); 5761 (*frutescens*); 7216 (*rabenii*); 8481 (*caatingae*); 10880, 11175, 11207 (*flexuosum*); 13175 (*rabenii*)

Pérez, A.J. et al. 3833 (*chinense*); 5386 (*lycianthoides*)

Pérez, K. & D. Tea 18 (*chacoense*)

Perez Arbeláez, E. & J. Cuatrecasas 6511 (*annuum* var. glabriusculum); 8297 (*rhomboideum*); 8305 (*annuum* var. glabriusculum)

Pérez Arbeláez, E. 443 (*annuum* var. glabriusculum); 2085 (*rhomboideum*); 2491 (*annuum* var. glabriusculum); 3119 (*rhomboideum*)

Pérez C., E. & E. Carranza 2888 (*rhomboideum*)

Pérez Cruz, G. 245, 250 (*rhomboideum*)

Pérez-García, E. & B. Reyes Días 920 (*annuum* var. glabriusculum)

Pérez J., A. 521, 766 (*annuum* var. glabriusculum)

Pérez Z., J.A. et al. 1661 (*rhomboideum*)

Perrottet, S. 218 (*frutescens*)

Pesha Baca, V. 95 (*chinense*)

Pessoa, M.C. & J.R. Lima 323 (*parvifolium*)

Pessôa, S. de V.A. et al. 127 (*schottianum*)

Petersen, E. & J.P. Hjerting 947 (*chacoense*)

Petersen, P.M. 6454 (*annuum* var. glabriusculum)

Peterson, P.M. & C.R. Annable 7043 (*annuum* var. glabriusculum)

Pfeifer, H.W. 1283 (*annuum* var. glabriusculum)

Philipson, W.R. et al. 1456 (*annuum* var. glabriusculum)

Phillips, O. & P. Núñez 163 (*coccineum*)

Piccinini, B.G. & J. Hilfer 4123, 4293 (*chacoense*)

Piccinini, B.G. & C.A. Petetin 2961, 2980 (*chacoense*)

Pickel, B.J. 2492, 3892 (*parvifolium*); 3964 (*chinense*)

Pickersgill, B. Cb 4 (*annuum* var. glabriusculum); 401 (*tovarii*); 462 (*recurvatum*); 463 (*cornutum*); 464, 464-3 (*villosum*); RU72-99 (*chinense*); RU72-118, RU72-140 (*frutescens*); RU72-175 (*chinense*); RU72-244, RU72-357 (*frutescens*); RU72–366 (*parvifolium*)

Pifano, D.S. & A.S.M. Valente 266 (*schottianum*)

Pimentel, J. & R. García 390 (*annuum* var. glabriusculum)

Pinell R., N. 27, 61 (*rhomboideum*)

Pineschi, R.B. 42 (*schottianum*)

Pinheiro, M. 253 (*longidentatum*)

Pinkley, H.V. 230, 256, 257, 258, 259 (*chinense*); 359 (*pubescens*); 544 (*chinense*)

Pinto, D. et al. 48 (*frutescens*)

Pinto, G.C.P. & H.P. Bautista 103, 104 (*caatingae*)

Pinto, L.J.S. et al. 92 (*cornutum*)

Pinto, P. & M. Dumont 563 (*annuum* var. glabriusculum)

Pinto E., P. et al. 6185 (*annuum* var. annuum)

Piper, C.V. 5648 (*annuum* var. glabriusculum)

Pipoly, J.J. 1602 (*annuum* var. annuum)

Pirani, J.R. & R. García 3105 (*recurvatum*)

Pire, E.F. 520 (*chacoense*)

Pires, M.J. 392, 433 (*rabenii*)

Pirondo, A. et al. 160 (*baccatum* var. pendulum)

Pittier, H. 105 (*rhomboideum*); 614 (*annuum* var. glabriusculum); 737 (*dimorphum*); 872 (*lycianthoides*); 1372, 3810, 4299 (*annuum* var. glabriusculum); 7124 (*rhomboideum*); 7909 (*frutescens*); 9623, 10399, 11542 (*rhomboideum*); 11561 (*annuum* var. glabriusculum); 11905 (*rhomboideum*); 12199 (*frutescens*); 12585 (*rhomboideum*); 12707 (*pubescens*); 12709 (*annuum* var. annuum); 12837 (*annuum* var. glabriusculum); 13132, 13366 (*rhomboideum*); 13543 (*annuum* var. glabriusculum); 14032 (*parvifolium*); 14710 (*annuum* var. glabriusculum)

Pivetta 938 (*flexuosum*)

Plaumann, F. 229 (*flexuosum*)

Plotkin, M.J. 139 (*chinense*)

Plowman, T.C. 2457, 2458 (*chinense*); 3205 (*frutescens*); 3223 (*annuum* var. glabriusculum); 5439 (*hookerianum*); 5566 (*rhomboideum*); 5821 (*coccineum*); 5822 (*chinense*); 6048 (*annuum* var. glabriusculum); 11896 (*annuum* var. annuum); 14539 (*frutescens*)

Plowman, T.C. & P.W. Alcorn 14335 (*hookerianum*)

Plowman, T. & E.W. Davis 4164 (*annuum* var. glabriusculum); 4572 (*rhomboideum*); 4788 (*baccatum* var. baccatum); 4809 (*pubescens*); 7407 (*baccatum* var. baccatum)

Plowman, T. & J. Schunke V. 11497 (*chinense*); 11523 (*coccineum*); 11694 (*frutescens*); 11695 (*chinense*)

Plowman, T. & D. Vaughan 5366-A (*annuum* var. glabriusculum); 5370 (*annuum* var. annuum)

Plowman, T. et al. 4054, 4055 (*chinense*); 6391 (*annuum* var. annuum); 6418 (*frutescens*); 6419, 6599 (*annuum* var. glabriusculum); 6902 (*chinense*); 7075 (*annuum* var. glabriusculum); 7077, 7138, 8812 (*chinense*)

Poeppig, E.F. 1799, 1845 (*coccineum*); 2220 (*chinense*)

Pohl, J.B. 3674 (*villosum*); 5173 (*rabenii*)

Poortman, H.A.-C. 520 (*geminifolium*)

Poppleton, J.E. 933 (*annuum* var. glabriusculum)

Portal, E. et al. 108 (*neei*); 495 (*baccatum* var. baccatum)

Porter, D.M. 1299 (*annuum* var. glabriusculum)

Posada, A. 2581 (*annuum* var. annuum); 2603, 2606 (*frutescens*)

Pott, A. et al. 5562 (*frutescens*)

Poveda, K. et al. 19 (*dimorphum*)

Pozner, R. 207 (*chacoense*)

Prado, D. 10 (*chacoense*)

Prance et al., G.T. 9295 (*annuum* var. glabriusculum); 25919 (*recurvatum*); 26505 (*annuum* var. glabriusculum)

Premauer, J. & M.I. Moreno 57 (*rhomboideum*)

Prieto, F. 21 (*chinense*)

Pringle, C.G. 1912 (*annuum* var. glabriusculum); 2544 (*rhomboideum*); 2706 (*annuum* var. glabriusculum); 4057, 6505, 15620 (*rhomboideum*)

Proctor, G.R. 7507, 44888 (*annuum* var. glabriusculum)

Profice, J.R. et al. 22 (*campylopodium*)

Puch, A. 1089 (*annuum* var. glabriusculum)

Puiggari, G.L.T. 3577 (*recurvatum*)

Pujupet, J.O. RBAE 1005 (*chinense*)

Pulido, M.T. 511 (*pubescens*)

Purkayastha & Singh, DRLT 12 (*chinense*)

Purpus, C.A. 124 (*rhomboideum*); 2015 (*annuum* var. glabriusculum); 2294, 2709, 3362, 3993, 4893, 5051 (*rhomboideum*); 6976 (*lanceolatum*); 7562, 8424 (*rhomboideum*); 8499 (*annuum* var. glabriusculum)

Purseglove, J.W. 6314 (*annuum* var. glabriusculum)

Queiroz, A.S. & F. França 34 (*caatingae*)

Queiroz, A.S. et al. 55 (*caatingae*)

Quevedo, F.L. et al. 1816 (*chinense*)

Quinet, A. et al. 231 (*villosum*)

Quintana, C. & S. Valencia 441, 520 ½, 669 (*rhomboideum*)

Quintana, C. et al. 3030 (*annuum* var. glabriusculum); 843 (*rhomboideum*)

Quipuscoa S., V. & L. Bardales 979 (*pubescens*)

Quipuscoa S., V. et al. 4374 (*longifolium*)

Raben, F.C. 7 (*baccatum* var. baccatum); 301 (*rabenii*)

Ragonese, A.E. 2227 (*chacoense*)

Ragonese, A.E. & J.A. Castiglioni 7991, 8036, 8039 (*chacoense*)

Ramalho, F.B. 132 (*caatingae*)

Ramírez A., S. 56 (*annuum* var. annuum) Ramírez A., S. 56 (*chinense*)

Ramírez Delgadillo, R. & L. Portillo M. 1497 (*rhomboideum*)

Ramírez P., B.R. 295, 1399, 1905, 2174, 5891, 7476 (*rhomboideum*)

Ramírez P., B.R. 5639 (*pubescens*)

Ramírez, F. & M. García 526 (*annuum* var. glabriusculum)

Ramírez, N. 1067 (*rhomboideum*); 2548 (*parvifolium*)

Ramírez, N. & M. López 3293 & 3294 (*rhomboideum*)

Ramos, H.J. 164 (*frutescens*)

Ramos, J.E. 418 (*rhomboideum*); 440 (*annuum* var. glabriusculum); 476 (*rhomboideum*); 1794 (*lycianthoides*)

Ramos, J.E. & N. Paz 915 (*annuum* var. glabriusculum)

Ramos, J.E. & P. Silverstone-Sopkin 846 (*annuum* var. glabriusculum)

Ramos, J.E. et al. 6826, 6909, 7172 (*geminifolium*); 7438 (*rhomboideum*)

Ramos Núñez, G. et al. 18V.C.031 (*annuum* var. glabriusculum)

Rangel Ch., O. et al. 5336, 5511, 5539 (*dimorphum*)

Reales, A. 65 (*chacoense*)

Reed, H.R. 432 (*annuum* var. glabriusculum)

Regnell, A.F. Ser. III, 1001 (*flexuosum*); ser. III, 1002 (*rabenii*)

Reiche, K. 908 (*annuum* var. glabriusculum)

Reina, A.L. et al. 537, 1012, 1123 (*annuum* var. glabriusculum)

Reitz, R. C 924 (*recurvatum*); 2776, 4543 (*flexuosum*); 4749 (*rabenii*); 4750 (*annuum* var. annuum); 5484 (*flexuosum*)

Reitz, R. & R.M. Klein 3760 (*rabenii*); 5766, 5943 (*recurvatum*); 7542, 11724, 12487, 14102, 16945 (*flexuosum*); 17345 (*recurvatum*)

Reko, B.P. 41 (*pubescens*)

Renjifo, L.M. 253 (*dimorphum*)

Rennó, L. 3027 (*rabenii*)

Renolfi, R. 137, 422 (*chacoense*)

Rente, J.A. 31 (*campylopodium*)

Rentería A., E. et al. 601 (*dimorphum*)

Repizzo, A. & Z. Calle 217 (*dimorphum*)

Reyes, D. & L. Carrillo 502, 773 (*chinense*); 1048 (*annuum* var. annuum); 1049 (*frutescens*)

Reyes, D. & G. Moya 234 (*chinense*)

Reyes, J. & L.E. Arévalo 32 (*annuum* var. glabriusculum)

Reyes, S.J. 1782, 1782a, 309, 972 (*rhomboideum*)

Reyes García, A. 237 (*annuum* var. glabriusculum)

Reyes-García, A. & C. Chavarria 5066 (*rhomboideum*)

Reyes García, A. & R. Hampshire 1933 (*rhomboideum*); 1936 (*annuum* var. glabriusculum)

Reyes García, A. & G. Urquijo 753 (*rhomboideum*); 833 (*annuum* var. glabriusculum); 981 (*annuum* var. glabriusculum); 982, 1237 (*rhomboideum*)

Reyes García, A. et al. 1828, 1844 (*rhomboideum*); 5319, 5867 (*annuum* var. glabriusculum)

Reyes Santiago, J. & F. Reyes S. 594 (*rhomboideum*)

Reynel, C. et al. 5235 (*chinense*)

Rezende, M.G. et al. 100 (*pereirae*)

Ribas, O.S. et al. 4624 (*villosum*); 6004, 7150 (*recurvatum*)

Ribeiro, R. 563 (*schottianum*)

Richard 528 (*campylopodium*)

Richey, L.R. 98-355, 99-423 (*annuum* var. glabriusculum)

Ricinel 48 (*baccatum* var. baccatum)

Ricksecker, A.E. 232 (*annuum* var. glabriusculum)

Rico-Gray, V. & C. Chan 429 (*annuum* var. glabriusculum)

Ridoutt, C.A. 11714 (*annuum* var. annuum)

Riedel, L. 787 (*baccatum* var. baccatum); 1073 (*flexuosum*); 1074 (*baccatum* var. baccatum); 1080 (*schottianum*)

Riedlé, A. 1798 (*annuum* var. glabriusculum)

Rimbach, A. 170, 391 (*rhomboideum*)

Ríos, M. & A. Oña 429, 430 (*chinense*)

Rios, M. & F. Ghia 131 (*annuum* var. annuum)

Rios, M. & E. Vivanco 383 (*annuum* var. annuum)

Ritter, N. 989 (*pubescens*)

Rivas, V.M. & E. Guanín A. 113 (*geminifolium*)

Rivera C., J. L-166 (*rhomboideum*)

Rivera Díaz, O. 102 (*rhomboideum*)

Rivera Díaz, O. et al. 4217, 4270 (*dimorphum*)

Rivera H., J. et al. 371 (*annuum* var. glabriusculum)

Rivero, E. 67 (*chinense*); 218 (*baccatum* var. baccatum); 428 (*chinense*)

Robayo, C.X. 1 (*annuum* var. annuum)

Robles G., R. 886 (*annuum* var. glabriusculum)

Robleto, W. 902, 907 (*annuum* var. annuum)

Roca, A. 0689 (*baccatum* var. pendulum)

Rodrigo, A.P. 2637 (*chacoense*)

Rodrigues et al. 12335 (*frutescens*)

Rodrigues, C. et al. 03 (*baccatum* var. pendulum)

Rodrigues, F.M. & J.G. Oliveira 302 (*baccatum* var. baccatum)

Rodrigues, I.M.C. & M.M. Vieira 446, 447 (*pereirae*)

Rodrigues, I.M.C. et al. 473 (*campylopodium*); 502 (*mirabile*); 570 (*annuum* var. glabriusculum)

Rodrigues, S.T. & A.V. Carvalho 483, 490 (*baccatum* var. umbilicatum)

Rodrigues Vidal, R. 03 (*baccatum* var. pendulum)

Rodríguez, A. & L.D. Vargas 2524 (*annuum* var. glabriusculum)

Rodríguez, F. 2, 48, 49, 53, 59, 68, 71 (*frutescens*); 79 (*chinense*); 82, 112, 118, 120 (*frutescens*); 164 (*baccatum* var. baccatum); 165 (*annuum* var. annuum); 505 (*frutescens*); 1196 (*chacoense*)

Rodríguez, I. 104 (*annuum* var. glabriusculum)

Rodríguez, I. & R. Lira 63, 63a & 63b (*annuum* var. annuum)

Rodríguez, L. 461 (*annuum* var. glabriusculum)

Rodríguez, M. 168 (*baccatum* var. baccatum)

Rodríguez, M.E. & R.D. Aranda 171 (*flexuosum*)

Rodríguez, W. et al. 589 (*dimorphum*)

Rodríguez-M, G.M. & L. Olivares 272 (*annuum* var. glabriusculum)

Rodríguez R., E. 1928 (*geminifolium*)

Rodríguez R., E. & J. Campos de la Cruz 1811 (*geminifolium*)

Rodríguez R., E. & R. Cruz A. 2046 (*geminifolium*)

Rodschied, E.K. 29 (*annuum* var. annuum); 30 (*frutescens*)

Roe, K.E. 1331 (*annuum* var. glabriusculum)

Roe, K.E. & E. Roe 2223 (*rhomboideum*)

Roe, K.E. et al. 789 (*rhomboideum*)

Roic, L.D. 492 (*chacoense*)

Rojas, N. et al. 13 (*annuum* var. glabriusculum)

Rojas, R. et al. 886 (*annuum* var. glabriusculum); 1642 (*geminifolium*); 1649, 1744 (*dimorphum*);

Rojas, T. 2434 (*chacoense*); 2435 a, 2436, 2436 A (*baccatum* var. baccatum); 2440, 2605 (*chacoense*); 3356, 3363 (*flexuosum*); 3368 (*baccatum* var. baccatum); 6019 (*flexuosum*); 9529, 12452 (*baccatum* var. baccatum); 13815, 14325 (*chacoense*); 14415 (*flexuosum*)

Rojas G., R.D. 745 (*annuum* var. glabriusculum)

Roldán, F.J. & C. Cuartas 1853 (*annuum* var. glabriusculum)

Roldán, F.J. et al. 1626 (*rhomboideum*); 2332, 2350 (*dimorphum*)

Rombouts, H. 735 (*annuum* var. glabriusculum)

Romero Castañeda, R. 2101 (*annuum* var. glabriusculum); 2250 (*rhomboideum*); 2364 (*annuum* var. glabriusculum); 3797 (*chinense*); 6674 (*pubescens*); 9255, 10067 (*annuum* var. glabriusculum)

Romero, E. 64 (*annuum* var. glabriusculum)

Romero, G. et al. 1722 (*annuum* var. glabriusculum)

Romero, M.V. 1, 2, 3 (*baccatum* var. baccatum), 9 (*chinense*)

Roponen, S. & Å. Johannessen 85, 229 (*lycianthoides*)

Roque, A.A. 885, 1061 (*parvifolium*)

Rosa, E. 1697 (*chacoense*)

Rosa, M. 75 (*schottianum*); 107 (*campylopodium*); 111 (*campylopodium*); 118 (*schottianum*)

Rosa, S. 366 (*baccatum* var. baccatum)

Rosado, N. 01 (*chinense*)

Rosales, J.M. 1044 (*rhomboideum*)

Rosas R., M. 1320 (*annuum* var. glabriusculum)

Rose, J.N. 21950 (*annuum* var. glabriusculum)

Rose, J.N. & G. Rose 22258 (*geminifolium*); 23566 (*rhomboideum*)

Rosenthal, J.P. 101, 102, 104, 109 (*annuum* var. glabriusculum)

Rossi, L. & E.L.M. Catharino 1605 (*schottianum*)

Rossi, L. et al. 1474 (*recurvatum*); 2037 (*cornutum*)

Roybal, J.J. 539 (*annuum* var. glabriusculum)

Rubio, D. & G.A. Tipaz 2361 (*hookerianum*)

Rubio, H. 328 (*annuum* var. glabriusculum); 949, 1037, 2026 (*rhomboideum*)

Rueda, R.M. 12112 (*rhomboideum*)

Ruenes, R.M. 22 (*annuum* var. glabriusculum)

Rugama, R.A. 19 (*rhomboideum*)

Rugel, F. 87 (*annuum* var. glabriusculum)

Ruiz de Huidobro, M. 125 (*chacoense*)

Ruiz Núñez, N. 6, 7 (*annuum* var. glabriusculum)

Ruiz Terán, L.E. et al. 12210 (*rhomboideum*)

Ruiz, D. 185 (*rhomboideum*)

Ruiz, M. et al. 139 (*dimorphum*)

Ruiz, R. & E. Correa 929 (*dimorphum*)

Runyon, R. 285 (*annuum* var. glabriusculum); 948 (*rhomboideum*); 5449 (*annuum* var. glabriusculum)

Rusby, H.H. 680 (*minutiflorum*)

Rusby, H.H. & F.W. Pennell 503 (*annuum* var. glabriusculum); 111 (*rhomboideum*)

Russel, Padre A. 6140 (*baccatum* var. pendulum)

Rutten-Peckel Harig, C.J. 4 (*rhomboideum*)

Rzedowski, J. 6849, 7663, 10641, 10668 (*rhomboideum*); 16094 (*annuum* var. glabriusculum); 24373, 24631, 32523, 49555 (*rhomboideum*)

Saer, J. 109, 627 (*rhomboideum*)

Sagástegui A., A. 5846, 13036, 15559, 15612 (*rhomboideum*)

Sagastegui A., A. & S. Leiva González 12515 (*rhomboideum*)

Sagástegui A., A. et al. 12502 (*rhomboideum*); 12998 (*geminifolium*)

Saint Hilaire, A. 764 (*rabenii*)

Salas A., A. et al. 90 (*annuum* var. glabriusculum)

Salas M., S.H. et al. 185, 4061 (*annuum* var. glabriusculum)

Salaün 177 (*frutescens*); 185 (*annuum* var. glabriusculum)

Salazar et al., G. 8074 (*annuum* var. glabriusculum)

Saldías P., M. 110 (*coccineum*); 563, 606 (*pubescens*); 759 (*annuum* var. annuum); 760 (*baccatum* var. pendulum); 4977 (*caballeroi*)

Sales, M.F. et al. 604 (*caatingae*)

Salinas, C. & S. Benítez 240 (*annuum* var. annuum)

Salinas, N. et al. 7249 (*geminifolium*)

Salinas T., A. & E. Martínez-Correa 7357 (*rhomboideum*)

Salino, A. 3850 (*campylopodium*)

Sampaio, A. et al. 2363 (*schottianum*)

Sánchez, C. 981 (*dimorphum*)

Sánchez, D. et al. 125, 1457, 3176 (*dimorphum*)

Sánchez, F. et al. 954 (*annuum* var. glabriusculum)

Sánchez, H. et al. 579 (*annuum* var. glabriusculum)

Sánchez, M.E. 002, 7 (*frutescens*)

Sánchez, O. & C. Aguirre 358, 373 (*rhomboideum*)

Sánchez, R. & E. Linares 1479 (*dimorphum*)

Sánchez, R. et al. 1323 (*dimorphum*)

Sánchez C., J. 12 (*annuum* var. glabriusculum)

Sánchez L., F. 317 (*annuum* var. glabriusculum)

Sánchez L., F. & P. Trujillo V. 874 (*annuum* var. glabriusculum)

Sánchez L., F. et al. 258 (*annuum* var. glabriusculum); 953 (*frutescens*); 1190 (*annuum* var. glabriusculum)

Sánchez León, V.M. 1139 (*pubescens*)

Sánchez Martínez, A. & A. Nava 230, 247, 548 (*annuum* var. glabriusculum)

Sánchez Martínez, A. et al. 983 (*annuum* var. glabriusculum); 5138 (*rhomboideum*); 12745 (*frutescens*)

Sandeman, C. 2038 (*cornutum*)

Sanders, A.C. et al. 17496 (*annuum* var. glabriusculum)

Sandino, J.C. 253, 306 (*annuum* var. glabriusculum); 2975 (*annuum* var. annuum)

Sandoval, E.A. 308, 1743 (*rhomboideum*)

Sandoval, E.A. et al. 550 (*rhomboideum*)

Sanín, D. 1048 (*dimorphum*); 6236 (*regale*)

Santa et al., J. 155 (*baccatum* var. baccatum)

Santa María, E. 733 (*annuum* var. glabriusculum)

Santacruz, P. 20 (*frutescens*)

Santín, F. et al. 100 (*annuum* var. glabriusculum); 102 (*chinense*)

Santos, A.A. et al. 2663, 2829 (*flexuosum*)

Santos, E. et al. 2869 (*flexuosum*)

Santos, U.S. et al. 5 (*caatingae*)

Santos, L.A.S. 1237 (*parvifolium*)

Santos, L.B. 160 (*rabenii*)

Santos, R.M. et al. 1574 (*caatingae*)

Saravia, C. et al. 11572 (*baccatum* var. baccatum)

Saravia Toledo, C. 103, 828, 1197 (*chacoense*); 1404 (*annuum* var. glabriusculum); 1421, 4641 (*rhomboideum*); 10325 (*eximium*); 10706 (*chacoense*); 11607 (*baccatum* var. baccatum); 12126, 12245 (*eximium*); 12812, 13472, 13484, 13490 (*chacoense*)

Saravia Toledo, C. et al. 11372, 11392 (*baccatum* var. baccatum); 11495, 11496, 11544 (*chacoense*)

Saravia Toledo, C. & R. Jaramillo M. 1978 (*annuum* var. glabriusculum); 1806 (*rhomboideum*)

Saravia Toledo, C. & J. Nelson 10343, 10815 (*chacoense*)

Sargen, F.H. B69 (*frutescens*)

Särkinen, T. et al. 4805, 4813 (*longifolium*)

Sarma, J. & G. Dutta 394 (*annuum* var. annuum)

Sarukhán, J. et al. 3581 (*annuum* var. glabriusculum)

Sastre, C. 3298 (*frutescens*)

Sastre, C. et al. 2708 (*annuum* var. glabriusculum)

Sauleda, R.P. & D.S. Correll 2416 (*annuum* var. glabriusculum)

Saunes V., A. et al. 5641, 5676 (*annuum* var. glabriusculum)

Sawyer, N.W. 720 (*geminifolium*)

Sawyer, N.W. & J. Burneo 694 (*geminifolium*)

Sayago, M. B-113 (*chacoense*)

Saynes Vásquez, A. 657 (*rhomboideum*)

Saynes Vásquez, A. et al. 3263, 5601 (*annuum* var. glabriusculum)

Scaldaferro, M. 9 (*annuum* var. glabriusculum); 13 (*frutescens*); 19, 20 (*baccatum* var. pendulum); 22, 23, 24, 27, 28, 29, 32, 33 (*chacoense*); 34 (*annuum* var. annuum); 36 (*chacoense*); 37, 38, 39, 40 (*baccatum* var. pendulum); 41 (*annuum* var. glabriusculum); 42, 43 (*baccatum* var. pendulum); 44 (*chacoense*); 45, 49 (*baccatum* var. pendulum); 50 (*baccatum* var. baccatum); 57, 63 (*baccatum* var. umbilicatum); 65 (*chacoense*); 73 (*rhomboideum*)

Scalon, V.R. et al. 628 (*mirabile*)

Scariot, A.O. et al. 340 (*rabenii*); 381 (*mirabile*)

Scarpa, G.F. 649 (*chacoense*)

Schenkel, E.P. et al. 447 (*baccatum* var. baccatum)

Schickendantz, F. 74, 158, 187, 211, 299 (*chacoense*)

Schinini, A. 4140 (*baccatum* var. baccatum); 4248 (*chacoense*); 4901 (*flexuosum*); 13279 (*annuum* var. annuum); 14061 (*baccatum* var. baccatum); 19988 (*chacoense*); 23074 (*flexuosum*); 34466 (*baccatum* var. baccatum); 35123 (*baccatum* var. umbilicatum); 36578 (*baccatum* var. baccatum); 36777 (*flexuosum*)

Schinini, A. & E. Bordas 14930, 15041, 16524 (*chacoense*); 20626 (*baccatum* var. baccatum); 24511 (*frutescens*)

Schinini, A. & G. Caballero Marmori 26990 (*flexuosum*)

Schinini, A. & M. Dematteis 33242 (*flexuosum*); 33747 (*baccatum* var. baccatum)

Schinini, A. & A. Fernández 5952, 5967, 6058 (*flexuosum*)

Schinini, A. & R. Palacios 25565 (*baccatum* var. baccatum); 25726 (*chacoense*)

Schinini, A. & S.N. Pire 24216 (*chacoense*)

Schinini, A. & C.L. Quarín 11524 (*chacoense*); 11542 (*baccatum* var. baccatum)

Schinini, A. & M. Urbani 35782 (*chacoense*)

Schinini, A. et al. 11282, 17024 (*baccatum* var. baccatum); 17088 (*chacoense*); 27787 (*rabenii*); 28711, 28756 (*flexuosum*); 34778 (*eximium*); 35295 (*chacoense*)

Schipp, W.A. 1076 (*annuum* var. glabriusculum)

Schmalzel, R.J. 1211 (*annuum* var. glabriusculum)

Schmeda, C. 167, 863, 1301 (*chacoense*); 1584 (*baccatum* var. baccatum)

Schnee, L. 1183 (*annuum* var. glabriusculum)

Schneider, M. 216 (*rhomboideum*); 893 (*geminifolium*); 912 (*rhomboideum*);

Schott, H.W. 5409 (*campylopodium*); 5416 (*muticum*); 5425, 5426 (*schottianum*)

Schreiter, R. 1927, 3791, 10101 (*chacoense*)

Schultes, E.E. 3794 (*frutescens*)

Schultes, E.E. & Cabrera 13046 (*frutescens*)

Schultes, R.E. 3269 (*pubescens*); 3497, 5518, 6620 (*chinense*)

Schultes, R.E. & I. Cabrera 13138 (*chinense*); 13752 (*frutescens*)

Schultes, R.E. & M. Villareal 7598, 7958 (*pubescens*); 7863 (*rhomboideum*)

Schulz, A.F. 7155, 7204 (*flexuosum*)

Schulz, A.G. 1, 2, 87, 90 (*chacoense*); 2028 (*baccatum* var. baccatum); 2030, 2032 (*chacoense*); 2050, 2103 (*baccatum* var. baccatum); 3814, 4272, 5222 (*chacoense*); 6714 (*baccatum* var. baccatum); 6719 (*chacoense*); 7155 (*baccatum* var. baccatum); 7361, 7363 (*chacoense*); 8049 (*baccatum* var. baccatum); 8178, 8284, 8930, 9032, 9064, 10649, 15632, 15801 (*chacoense*); 15929 (*baccatum* var. baccatum); 17367, 17721, 17958, 18190 (*chacoense*)

Schulz, C.L. 845, 932, 1082, 1141, 1180 (*chacoense*)

Schunck 9711 (*chacoense*)

Schunke V., J. 821 (*coccineum*); 1397 (*frutescens*); 3415, 5199, 6442, 6554, 9984, 10620, 10777, 12469 (*coccineum*); 14492 (*annuum* var. annuum); 14518 (*chinense*)

Schuncke V., J. & J.G. Graham 15004, 16612 (*coccineum*)

Schwabe, H. 650 (*chacoense*)

Schwarz, G.J. 2875, 2938, 2939, 2986, 3414, 4023, 4160, 4389, 4458, 4690, 5763, 5991, 6900, 7037, 7038, 7589, 7755, 7882, 11868 (*flexuosum*)

Schwindt, E. 305, 947 (*flexuosum*); 996, 1362 (*chacoense*); 1548 (*baccatum* var. baccatum); 1871, 1911, 1948, 1966, 4217, 4310, 4581, 4790 (*flexuosum*)

Schwirkowski, P. 139 (*recurvatum*); 885 (*rabenii*); 1425, 1433, 1470 (*recurvatum*); 1519 (*flexuosum*); 2534 (*recurvatum*); 2713 (*rabenii*);

Scolnik, R. 821, 822 (*pubescens*); 1389 (*geminifolium*); 1594 (*rhomboideum*)

Scolnik, R. & J. Correa V. 458 (*annuum* var. glabriusculum)

Scolnik, R. & R. Luti 526 (*pubescens*); 605 (*baccatum* var. pendulum); 637 (*annuum* var. annuum); 672 (*frutescens*); 703 (*annuum* var. annuum)

Scolnik, R. et al. 19An322 (*rhomboideum*); 19An329, 19S022 (*annuum* var. glabriusculum)

Scott de Birabén, M.I. & M. Birabén 1143 (*chacoense*)

Sehnem, A. 3824, 3828, 3927 (*flexuosum*)

Seidel, R. & S. Beck 59, 361 (*baccatum* var. baccatum)

Seidel, R. & I. Hinojosa 1267 (*ceratocalyx*)

Seidel, R. & D. Vaquiata 7689 (*coccineum*)

Seigler, D. et al. 13144, 13353 (*rhomboideum*)

Seijo, G.J. 1833 (*chacoense*)

Seijo, J.G. et al. 3351, 3356 (*chacoense*); 3448, 3580, 3812 (*baccatum* var. baccatum); 3820, 3864, 3869 (*chacoense*)

Seler, E. 253, 610 (*annuum* var. glabriusculum)

Sellow, F. 6 (*campylopodium*); 79 (*muticum*); 209 (*mirabile*); 221 (*baccatum* var. baccatum)

Serberg, O. 1753 (*frutescens*)

Serralta P., L. 104 (*annuum* var. glabriusculum)

Serrano, M. 1566, 1903 (*baccatum* var. baccatum)

Serrano, M. et al. 5482 (*neei*); 6882 (*caballeroi*); 7297 (*baccatum* var. baccatum)

Sessé, M. & J.M. Mociño 1508 (*rhomboideum*); 1546 (*annuum* var. glabriusculum)

Sevilha, A.C. & A.O. Scariot 1945, 2137 (*rabenii*)

Shafer, J.A. 45, 169, 330, 1458, 2361 (*annuum* var. glabriusculum)

Shannon, W.C. 206 (*annuum* var. glabriusculum)

Shonle et al., I. 23 (*annuum* var. glabriusculum)

Sidwell, K.J. et al. 695 (*rhomboideum*)

Siles G., E.L. 30, 58, 68 (*rhomboideum*)

Silva, A.B. 1402 (*caatingae*)

Silva, A.C.C. 244, 259, 287 (*caatingae*)

Silva, A.F.B. 139 (*parvifolium*)

Silva, E.L. et al. 1 (*caatingae*); 40 (*recurvatum*)

Silva, F. et al. 19An404 (*pubescens*)

Silva, H.S.J. & M. Hernández 343 (*rhomboideum*)

Silva, J.M. & L.M. Abe 4553 (*recurvatum*)

Silva, J.M. & J. Cordeiro 6187 (*recurvatum*)

Silva, J.M. et al. 2264 (*recurvatum*); 3559 (*flexuosum*); 4218 (*recurvatum*)

Silva, J.S. 272 (*recurvatum*)

Silva, R.A. 1506, 1793 (*caatingae*)

Silva, R.A. & D. Moura 1586, 1632 (*caatingae*)

Silva, S.M. & R.M. Britez 1714 (*flexuosum*)

Silva Neto, S.J. et al. 736, 1795 (*villosum*)

Silva-Santos, A. et al. 1990 (*flexuosum*)

Silveira, M. 1080 (*coccineum*)

Silveira, M. et al. 551, 650 (*coccineum*)

Silverstone-Sopkin, P.A. 556 (*lycianthoides*); 1451, 3689, 6108 (*rhomboideum*)

Silverstone-Sopkin, P. & N. Paz 6614 (*annuum* var. glabriusculum)

Silverstone-Sopkin, P.A. et al. 2322 (*rhomboideum*); 5884 (*annuum* var. glabriusculum); 6641, 6643 (*dimorphum*); 9069, 9249, 9377, 9790, 10070 (*lycianthoides*)

Simá, P. 517 (*annuum* var. glabriusculum); 606, 610 (*annuum* var. annuum)

Simá, P. et al. 1552 (*annuum* var. glabriusculum)

Simonis, J.E. et al. 207 (*baccatum* var. baccatum); 216 (*rabenii*)

Simpson, D.R. & J. Schunke V. 469 (*hookerianum*)

Simpson, J.H. 86 (*annuum* var. glabriusculum)

Singleton W.R. 611 (*annuum* var. glabriusculum)

Sintenis, P. 461 (*annuum* var. glabriusculum); 865 (*annuum* var. annuum); 866 b (*chinense*); 3685 (*annuum* var. glabriusculum)

Siquihua, L. 4 (*frutescens*)

Skorupa, L.A. 330 (*baccatum* var. pendulum)

Skorupa, L.A. & J.N. da Silveira 328 (*frutescens*); 329, 378 (*chinense*)

Skutch, A.F. 1450 (*lanceolatum*); 1936 (*rhomboideum*)

Slanis, A.C. 72 (*eximium*)

Slanis, A.C. et al. 116, 194 (*chacoense*)

Sleumer, H. & F. Vervoorst 2349 (*chacoense*)

Small, J.K. 2121, 2772, 3867, 4604, 7468, 7686, 8740 (*annuum* var. glabriusculum)

Small, J.K. & J.J. Carter 793, 2673, 2676, 8553 (*annuum* var. glabriusculum)

Small, J.K. & C.A. Mosier 5695 (*annuum* var. glabriusculum)

Small, J.K. & E.W. Small 4864 (*annuum* var. glabriusculum)

Small, J.K. & G.K. Small 4983 (*annuum* var. glabriusculum)

Small, J.K. & P. Wilson 1773, 1892 (*annuum* var. glabriusculum)

Smith, D.N. et al. 13470 (*caballeroi*)

Smith, H.H. 1164, 1169 (*baccatum* var. baccatum); 1176, 1183 (*parvifolium*); 1479 (*baccatum* var. baccatum)

Smith, J.D. 424 (*annuum* var. glabriusculum)

Smith, L.B. 7974 (*recurvatum*)

Smith, L.B. & R.M. Klein 9108, 9968, 13238 (*flexuosum*)

Smith, L.B. & R. Reitz 8603, 8833 (*flexuosum*)

Smith, L.B. et al. 11764 (*flexuosum*)

Smith, L.C. 659 (*rhomboideum*)

Smith, P.G. SA 272, SA 276 (*eximium*); SA 281 (*eshbaughii*); SA 325, SA 351 (*eximium*); SA 374 (*frutescens*); SA 393 (*coccineum*); 406 (*cardenasii*); Ac 689 (*chacoense*); Ac. 771 (*baccatum* var. pendulum); Ac 1256 (*chacoense*); Ac. 1425 (*pubescens*); 1519 (*baccatum* var. pendulum); Ac 2017 (*tovarii*); PS 350 (*eximium*);

Smith, P.S. 149 (*pubescens*)

Smith, S.D. 203 (*lycianthoides*)

Smith, S.G. 1133 (*rhomboideum*)

Smith AC, P. 1905 (*chinense*)

Smith V., R.F. 8617 (*rhomboideum*); 9349 (*parvifolium*)

Sneidern, K. 4528 (*annuum* var. glabriusculum)

Soares, E.L.C. 83, 84 (*flexuosum*)

Sobral, M. 2989 (*flexuosum*)

Sobral, M. & I.S. Almeida 7926 (*flexuosum*)

Sobral, M. & Ganev 7646 (*caatingae*)

Sobral, M. & J.A. Jarenkow 8021 (*flexuosum*)

Sobral, M. et al. 961, 5161, 1321, 2640, 3003, 3103 (*flexuosum*); 13870 (*rabenii*)

Sodiro, L. 114/82, 114/84 (*geminifolium*)

Soejarto, D.D. & H. Cardozo 813 (*chinense*)

Soejarto, D.D. & E. Hernández 1393 (*rhomboideum*)

Soejarto, D.D. et al. 2539 (*frutescens*)

Sohn, S. 98 (*recurvatum*)

Sohns, E.R. 1203, 1384 (*annuum* var. glabriusculum)

Sol S., A. 134 (*frutescens*)

Solange, P. 8 (*chinense*)

Solbrig, O. & G.F. Pabst 6884 (*recurvatum*)

Solheim, S.L. 1654 (*rhomboideum*)

Solomon, J.C. 2417 (*annuum* var. glabriusculum); 5304 (*cardenasii*); 5305 (*pubescens*); 5306 (*annuum* var. annuum); 8844, 12086 (*ceratocalyx*); 12991, 13104 (*pubescens*); 14672 (*chinense*); 15508 (*pubescens*); 19186 (*annuum* var. glabriusculum)

Solomon, J.C. & M. Nee 18181 (*pubescens*)

Solomon, J.C. et al. 6778, 6966 (*baccatum* var. baccatum)

Solomón Maya, J. 1689, 1734 (*rhomboideum*); 2570 (*lanceolatum*); 3039, 3430 (*rhomboideum*); 3521 (*lanceolatum*); 3551 (*rhomboideum*); 3704, 3867 (*lanceolatum*)

Soria, N. 2901, 3890 (*flexuosum*); 3900 (*chacoense*)

Soriano, A. 900 (*chacoense*)

Soriano, A. & W. Barrett 3592 (*chacoense*)

Soto, A. et al. 1472 (*rhomboideum*)

Soto, C. et al. 4025 (*annuum* var. glabriusculum)

Soto, D. et al. 95 A (*minutiflorum*)

Soto, J.C. et al. 13288 (*annuum* var. glabriusculum)

Soto Núñez, J.C. 11189 (*annuum* var. glabriusculum); 14047, 14048, 14302 (*annuum* var. annuum); 14302 (*annuum* var. glabriusculum)

Soto Núñez, J.C. & S. Aureoles C. 7889 (*annuum* var. glabriusculum)

Soto Núñez, J.C. & G. Silva R. 3842 (*chinense*); 14103 (*annuum* var. annuum)

Soto Núñez, J.C. & F. Solórzano G. 12592 (*pubescens*)

Soto Núñez, J.C. & R. Torres C. 2764 (*annuum* var. glabriusculum)

Soto Núñez, J.C. et al. 5442, 5443 (*annuum* var. annuum); 6021 (*annuum* var. glabriusculum); 6369 (*pubescens*)

Soukup, J. 1851 (*annuum* var. glabriusculum)

Sousa, F.F.S. 17 (*frutescens*)

Souza, E.B. 3924 (*parvifolium*)

Souza, F.M. et al. 1073 (*flexuosum*)

Souza, G.R. et al. 1680 (*baccatum* var. baccatum)

Souza, H.O. et al. 9081 (*pereirae*)

Souza, J.P. et al. 30 (*flexuosum*); 60 (*recurvatum*); 793 (*villosum*); 970 (*hunzikerianum*); 11012, 11029 (*parvifolium*)

Souza, P.T. 21 (*pubescens*)

Souza, V.C. 2140 (*rabenii*); 11168 (*mirabile*); 40126 (*rabenii*)

Souza, V.C. & T.B. Flores 35055 (*recurvatum*)

Souza, V.C. & M.O. Pedraz 1116 (*flexuosum*)

Souza, V.C. et al. 18426 (*annuum* var. glabriusculum); 26687 (*caatingae*); 32883 (*baccatum* var. baccatum)

Souza Pedroza, D.S. et al. 658 (*campylopodium*)

Soza, D. et al. 141 (*rhomboideum*)

Sparre, B. & F. Vervoost 552 (*rabenii*)

Sparre, B. 14042, 14365, 14591 (*lycianthoides*); 14653 (*rhomboideum*); 16714, 16984 (*lycianthoides*); 19319 (*geminifolium*)

Spooner, D. et al. 5036 (*geminifolium*); 6657 (*baccatum* var. baccatum)

Spruce, R. 5050 (*rhomboideum*)

St. John, H. 20655 (*dimorphum*)

Ståhl, B. & S. Báez 7061 (*lycianthoides*)

Ståhl, B. & J.T. Knudsen 1065, 1090, 1224 (*lycianthoides*)

Standford, L.R. et al. 955, 2112 (*annuum* var. glabriusculum)

Standley, P.C. 9714, 9810, 10058 (*rhomboideum*); 14077 (*lanceolatum*); 14190 (*rhomboideum*); 19872, 20689, 20903, 21308 (*annuum* var. glabriusculum); 22237 (*frutescens*); 23453 (*annuum* var. glabriusculum); 23755 (*frutescens*); 24095 (*rhomboideum*); 24297, 25055, 25504 (*frutescens*); 25825, 25887 (*annuum* var. glabriusculum); 26247 (*rhomboideum*); 26254 (*annuum* var. glabriusculum); 26493 (*frutescens*); 26862 (*annuum* var. glabriusculum); 28523 (*annuum* var. annuum); 29336, 29640 (*annuum* var. glabriusculum); 29880 (*annuum* var. annuum); 30539 (*annuum* var. glabriusculum); 31479 (*annuum* var. annuum); 31543, 31921 (*annuum* var. glabriusculum); 32004 (*frutescens*); 36044 (*annuum* var. glabriusculum); 53392 (*frutescens*); 53628, 53913, 54485, 55021, 55721 (*annuum* var. glabriusculum); 59930 (*rhomboideum*); 60647 (*annuum* var. glabriusculum); 63153 (*lanceolatum*); 64208 (*annuum* var. glabriusculum); 64892 (*rhomboideum*); 68235, 68244, 68294 (*lanceolatum*); 74361, 78366 (*annuum* var. glabriusculum); 80793 (*pubescens*); 86930 (*lanceolatum*); 88022, 90936 (*annuum* var. glabriusculum); 91227 (*pubescens*); 91469 (*lanceolatum*)

Stearn, W.T. 588, 616 (*annuum* var. glabriusculum)

Steere, W.C. 1314 (*annuum* var. glabriusculum); 1579 (*annuum* var. annuum); 1813 (*annuum* var. glabriusculum); 7404 (*frutescens*)

Stehlé, H. 2951 (*annuum* var. glabriusculum)

Stehmann, J.R. & F.S. Faria 6269 (*carassense*)

Stehmann, J.R. & A. Ippolito 1694 (*flexuosum*)

Stehmann, J.R. et al. 1520 (*flexuosum*); 2710 (*mirabile*); 4813 (*schottianum*); 4830, 4831 (*hunzikerianum*); 5037 (*mirabile*); 6344, 6347 (*carassense*); 6467 (*flexuosum*); 6474 (*mirum*)

Steibel, P.E. & H.O. Troiani 3835 (*chacoense*)

Steinbach, J. 2044 (*baccatum* var. baccatum); 2625, 3038, 3475 (*coccineum*), 5373, 5484 (*frutescens*); 5550 (*coccineum*); 8288 (*pubescens*)

Stellfeld, C. 1677 (*baccatum* var. pendulum)

Stergios, B. & L. Delgado 13640 (*annuum* var. glabriusculum)

Stergios, B. et al. 13316 (*frutescens*)

Stern, W.L. 263, 1901 (*annuum* var. glabriusculum)

Stern, W.L. et al. 134 (*rhomboideum*); 798 (*annuum* var. glabriusculum)

Stevens, W.D. 2604, 2701, 2805, 2888, 3013, 3360, 3431, 3774, 3854, 4223, 4688, 9348, 13617 (*annuum* var. glabriusculum); 31295 (*baccatum* var. baccatum)

Stevens, W.D. & M. Araquistain 13242 (*annuum* var. annuum)

Stevens, W.D. & E. Duarte M. 29871 (*rhomboideum*)

Stevens, W.D. & O.M. Montiel 16042, 17325, 18532, 22220, 29552, 31749 (*rhomboideum*); 35819 (*annuum* var. glabriusculum)

Stevens, W.D. et al. 14899 (*annuum* var. glabriusculum); 20300 (*frutescens*); 27284, 30086 (*rhomboideum*)

Stevensen, J.A. 2464 (*annuum* var. glabriusculum)

Stewart, A. 3352 (*galapagoense*); 3354, 3355, 3356, 3357 (*frutescens*)

Stewart, R.M. 1522 (*annuum* var. glabriusculum)

Steyermark, J.A. 29933 (*lanceolatum*); 30089 (*annuum* var. glabriusculum); 33357, 33429 (*lanceolatum*); 34462 (*annuum* var. annuum); 35237, 35422 (*lanceolatum*); 36930 (*pubescens*); 38343 (*annuum* var. glabriusculum); 46797, 47328, 47911, 48958 (*lanceolatum*); 50903 (*rhomboideum*); 51277, 51364 (*annuum* var. glabriusculum); 52093 (*lanceolatum*); 52482 (*geminifolium*); 52690 (*pubescens*); 52914 (*lycianthoides*); 54146, 54149 (*geminifolium*); 56884, 62772, 62814 (*rhomboideum*); 76028, 76029 (*chinense*); 76030, 88191, 91845, 99428 (*annuum* var. glabriusculum); 108787, 111696 (*rhomboideum*); 123436 (*annuum* var. glabriusculum)

Steyermark, J. & G. Agostini 91269 (*annuum* var. glabriusculum)

Steyermark, J.A. & P. Berry 111788 (*rhomboideum*)

Steyermark, J. & G. Bunting 102309 (*annuum* var. glabriusculum)

Steyermark, J.A. & V. Carreño Espinoza 107636 (*rhomboideum*)

Steyermark, J.A. & P. Colvée 121161 (*rhomboideum*)

Steyermark, J.A. & B.J. Manara 107966 (*rhomboideum*)

Steyermark, J. et al. 108086, 108194 (*parvifolium*); 114061, 118828, 120128 (*rhomboideum*); 123045 (*annuum* var. glabriusculum);

Stimson, W.R. 3049, 3922 (*annuum* var. glabriusculum)

Stoffers, A.L. 1186 (*annuum* var. glabriusculum)

Stork, H.E. & O.B. Horton 9462, 9463 (*chinense*); 9852 (*geminifolium*)

Strong, M.T. & F. Strong 4071 (*annuum* var. glabriusculum)

Stuckert, T. 917, 1008, 1120, 1320, 2405, 1166, 4238, 4247, 5557, 5614, 6007, 9472, 9913, 11559, 12551, 13092, 13210, 13644, 14330, 15477 (*chacoense*); 19345 (*baccatum* var. baccatum); 19510, 20267, 21449, 22168 (*chacoense*)

Stutz, L.C. 364, 1348, 1720, 2095 (*flexuosum*)

Suárez M., A. & J. Jácome 32 (*rhomboideum*)

Subieta, M. 56 (*chacoense*)

Subils, R. 1437 (*chacoense*); 2064 (*annuum* var. annuum); 2107, 2335, 3876 (*chacoense*); 4569 (*baccatum* var. pendulum); 4673 (*annuum* var. annuum); 4680 (*frutescens*)

Subils, R. & L. Articó 6 (*chacoense*)

Subils, R. & G. Barboza 4611 (*annuum* var. annuum); 4612 (*pubescens*); 4645 (*annuum* var. glabriusculum)

Subils, R. & E. Moscone 4133 (*flexuosum*); 4142 (*annuum* var. annuum); 4154 (*flexuosum*); 4166 (*annuum* var. annuum); 4265 (*baccatum* var. pendulum); 4271 (*flexuosum*); 4273 (*baccatum* var. baccatum)

Subils, R. et al. 1229, 3564 (*chacoense*)

Sucre, D. 6114, 6191, 7387, 8156, 8238 (*campylopodium*); 8934 (*frutescens*)

Sucre, D. & P.I. Braga 6402 (*campylopodium*); 6511 (*schottianum*)

Sucre, D. & Pe. L. Krieger 6863 (*pereirae*)

Sucre, D. & T. Soderstron 8987 (*rabenii*)

Sucre, D. et al. 2907 (*villosum*)

Sugiyama, M. & S.A. Chiea 1094 (*cornutum*)

Sugiyama, M. et al. 230 (*cornutum*); 1293 (*schottianum*); 1399 (*cornutum*)

Sullivan, V.I. 1067 (*annuum* var. glabriusculum)

Svolenski, A.C. & G. Tiepolo 236 (*flexuosum*)

Tabosa, F.R.S. et al. 55 (*longidentatum*)

Tafur, V. 219 (*chinense*); 237 (*rhomboideum*)

Tamayo, F. 281, 2006 (*rhomboideum*)

Tamayo, T. 2618 (*annuum* var. glabriusculum)

Tavares, B.V. et al. 17, 23 (*rabenii*)

Taylor 9662 (*annuum* var. glabriusculum)

Taylor E., M. 108 (*rhomboideum*)

Taylor G., R.G. 9 (*frutescens*)

Taylor, K. 385 (*annuum* var. glabriusculum)

Taylor, N. 3 (*annuum* var. glabriusculum)

Taylor Jr., R.J. 7249 (*annuum* var. glabriusculum)

Tejero-Díez, D. 6174, 6190 (*annuum* var. glabriusculum)

Teles Walter, B.M. et al. 567, 572, 573, 576, 579 (*chinense*)

Téllex, O. 7154 (*lanceolatum*)

Téllez, O. & L. Rico 3442 (*annuum* var. glabriusculum)

Téllez V., O. et al. 3770, 8073 (*annuum* var. glabriusculum)

Tenorio L., P. 9414 (*rhomboideum*)

Tenorio L., P. & R. Hernández M. 327 (*rhomboideum*)

Tenorio L., P. & C. Romero de T. 2229, 2279 (*rhomboideum*); 2389, 6591 (*annuum* var. glabriusculum); 6631, 6821 (*rhomboideum*)

Tenorio L., P. & M. Sousa S. 19436 (*annuum* var. glabriusculum)

Tenorio L., P. et al. 4204 (*rhomboideum*); 8305, 10513 (*annuum* var. glabriusculum); 11156, 14756, 17209 (*rhomboideum*)

Tepe, E.J. & M.P. Moreno 3014 (*rhomboideum*)

Tepe, E. et al. 2318 (*geminifolium*)

Terrazas, J. 1, 2, 4, 5, 6 (*pubescens*)

Terribile, M. 407 (*chacoense*)

Tessmann, G. 3432 (*recurvatum*)

Tewksbury, J. 1007, 1009 (*baccatum* var. baccatum); 1020 (*chacoense*)

Tharp, B.C. 256 (*annuum* var. glabriusculum)

Tharp, B.C. & F.A. Barkley 13A136 (*annuum* var. glabriusculum)

Thode, V. 277, 298 (*flexuosum*)

Thomas, E. 291 (*eximium*); 705, 1092, 1093 (*chinense*)

Thomas, E. & R. Berdeja 1428 (*coccineum*)

Thomas, R.D. et al. 87043 (*annuum* var. glabriusculum)

Thomas, W.W. & S. Sant’Ana 12496 (*pereirae*)

Thomas, W.W. et al. 9080 (*frutescens*); 13660 (*villosum*)

Thompson, S.A. et al. 7626, 10651, 10775 (*annuum* var. glabriusculum)

Thorne, R.F. & E. Lathrop 40532 (*annuum* var. glabriusculum); 41699 (*lanceolatum*)

Timaná, M. 758, 818, 1007, 1930, 3670, 3673 (*coccineum*)

Timaná, M. & N. Jaramillo 2878, 3224 (*coccineum*)

Ticona, J.P. & F. Saravia May 10 (*annuum* var. annuum)

Tillett, S.S. & C.L. Tillett 45424, 45627 (*chinense*)

Tipaz, G.A. et al. 865 (*hookerianum*)

Tirado, M. et al. 1309 (*lycianthoides*)

Tolaba, J. 2843 (*baccatum* var. baccatum); 3918 (*eximium*)

Tolaba, J. et al. 1375, 4090 (*chacoense*)

Toledo, C.B. et al. 69 (*flexuosum*)

Ton, A.S. 125 (*pubescens*); 274, 275 (*annuum* var. annuum); 398 A (*pubescens*); 2744, 2956, 2996, 3158, 3261 (*annuum* var. glabriusculum); 4395 (*rhomboideum*); 7803 (*lanceolatum*)

Tonaco, A.C. & J.M. Lanna 137 (*villosum*)

Tonduz, A. 9409, 12890 (*annuum* var. glabriusculum)

Torrecillas, E. 75 (*rhomboideum*); 78, 103 (*annuum* var. glabriusculum)

Torres, C. & P. Morales 139 (*annuum* var. annuum)

Torres, M. & F. Rodríguez 2020, 2025 (*frutescens*); 2032 (*chinense*)

Torres, M. et al. 199 (*annuum* var. glabriusculum); 4015, 4055 (*frutescens*)

Torres, R. et al. 5457 (*rhomboideum*)

Torres, S. 127 (*frutescens*)

Torres C., R. 148709 (*rhomboideum*)

Torres C., R. & E.F. Cabrera C. 6173 (*rhomboideum*)

Torres C., R. & L. Cortés A. 8821 (*annuum* var. glabriusculum); 10360 (*lanceolatum*)

Torres C., R. & E. Martínez S. 11099 (*annuum* var. glabriusculum)

Torres C., R. & P. Tenorio L. 3751, 3903 (*annuum* var. glabriusculum)

Torres C., R. et al. 3549 (*rhomboideum*); 5424 (*annuum* var. glabriusculum); 9826 (*rhomboideum*)

Torres R., J.H. 965 (*dimorphum*)

Tovar, O. 1867, 3750, 4114, 5012, 5363, 7865 (*tovarii*)

Tovilla Hernández, C. & I. Escalona Luttig 480 (*annuum* var. glabriusculum)

Tracy, S.M. 6831, 8650 (*annuum* var. glabriusculum)

Traverse, A. 2523 (*annuum* var. glabriusculum)

Trécul, M. 1126 (*annuum* var. glabriusculum)

Trejo, I. 2319 (*annuum* var. glabriusculum); 2321 (*rhomboideum*); 2424 (*rhomboideum*); 2537 (*annuum* var. glabriusculum)

Tressens, S. et al. 3092, 3097 (*flexuosum*); 3116, 3873, 3991 (*baccatum* var. baccatum); 4694, 4977, 5131, 5802, 5881 (*flexuosum*); 6925 (*chacoense*)

Triana, G. 18 (*frutescens*)

Triana, J. 346, 2284 (*rhomboideum*); 3864 (*geminifolium*)

Troiani, H.O. & P.E. Steibel 4077 (*chacoense*)

Troll, C. 1292 (*eximium*)

Troncoso, N.S. 1741 (*chacoense*)

Troncoso, R. & M. Fabbroni 224 (*chacoense*)

Trujillo 6244, 6353, 6885 (*rhomboideum*)

Trujillo & Fernández 296 (*rhomboideum*)

Trujillo et al. 247 (*rhomboideum*)

Trujillo, B. 3711 (*annuum* var. glabriusculum); 4643 (*frutescens*); 6351 (*annuum* var. glabriusculum)

Trujillo, B. & Rodríguez 18026 (*frutescens*)

Trujillo, B. & M. Trujillo 388, 15605 (*frutescens*)

Trujillo, B. et al. 17177, 20576 (*annuum* var. glabriusculum)

Trujillo, W. 130 (*chinense*); 208 (*frutescens*)

Trujillo E., R. & S.D. Martínez y Day 75 (*annuum* var. glabriusculum)

Tucker, J.M. 511 (*annuum* var. glabriusculum)

Tyson, E.L. 5214, 522, 5588 (*annuum* var. glabriusculum)

Tyson, E.L. & K. Blum 1946 (*annuum* var. glabriusculum)

Ucan Ek, E. 3893, 4617 (*annuum* var. glabriusculum); 4652 (*chinense*); 5058 (*annuum* var. glabriusculum)

Ucan Ek, E. et al. 3445, 3527 (*annuum* var. glabriusculum); 3529 (*annuum* var. annuum)

Ule, E. 635 (*villosum*); 2416, 3273 (*campylopodium*); 3744 (*villosum*); 7475 (*parvifolium*); 9732, 9738 (*coccineum*)

Ulibarri, E.A. 456 (*chacoense*)

Urban, I. 308, 461, 461 b (*annuum* var. glabriusculum); 866b, 5055 (*frutescens*)

Urdampilleta, J.D. et al. 693 (*chacoense*)

Uribe U., L. 1387 (*rhomboideum*); 1655 (*geminifolium*); 2117 (*dimorphum*); 4762 (*rhomboideum*); 6059 (*dimorphum*)

Utley, J.F. & K. Burt-Utley 3934 (*annuum* var. glabriusculum)

v. Wettstein, R. 975 (*flexuosum*)

Valenzuela, L. 9902 (*annuum* var. glabriusculum); 20885 (*dimorphum*)

Valenzuela, L. & I. Huamantupa 1013 (*coccineum*)

Valenzuela, L. et al. 3117 (*baccatum* var. baccatum); 11575 (*geminifolium*); 13438 (*dimorphum*)

Valerio R., J. 3039, 3237 (*pubescens*)

Valerio, M. 151 (*frutescens*)

Valeur, E.J. 275, 476, 964 (*annuum* var. glabriusculum)

Valiente B., A. 98, 272 (*rhomboideum*)

Valiente B., A. & J.I. Solís 360 (*rhomboideum*)

Valiente B., A. & J.L. Viveros S. 179 (*rhomboideum*)

Valla, J.J. 10 (*chacoense*)

Valle, M.H. 90 (*schottianum*)

Valls, J.F.M. et al. 9014 (*frutescens*)

Valverde, F.M. 35, 392 (*annuum* var. annuum)

Valverde R., J.L. 136 (*annuum* var. glabriusculum)

van Asdall, W. 82-59 (*frutescens*)

van den Eynden, V. & E. Cueva 654 (*frutescens*)

van den Eynden, V. et al. 700 (*annuum* var. glabriusculum)

van der Berg, C. 986 (*caatingae*)

van der Werff, H.H. 909 (*frutescens*)

van der Werff, H. & E. Gudiño 10652 (*rhomboideum*); 11196 (*regale*)

van der Werff, H. & R.C. Wingfield 7444 (*rhomboideum*); 7446 (*annuum* var. glabriusculum)

van der Werff, H. et al. 14624, 15144 (*annuum* var. glabriusculum)

van Devender, T.R. et al. 1005, 1074, 1170 (*annuum* var. glabriusculum)

Van Ee, B.W. et al. 669 (*chacoense*)

van Hermann, H.A. 164, 682 (*annuum* var. glabriusculum)

van Proosdij A.S.J. et al. 1057 (*annuum* var. glabriusculum)

Vandebroek, I. 356 (*annuum* var. glabriusculum)

Vanni, R. & D. Kurtz 3708 (*chacoense*)

Vanni, R. & A. Schinini 3057 (*flexuosum*)

Vanni, R. et al. 299 (*baccatum* var. baccatum); 2081, 2282, 2561 (*chacoense*); 3289 (*baccatum* var. baccatum); 3945 (*flexuosum*)

Vara et al. 417 (*annuum* var. glabriusculum)

Varela E., L. 179 (*annuum* var. glabriusculum)

Vargas, J.M. 249 (*rhomboideum*)

Vargas, L. et al.1310 (*coccineum*)

Vargas, W.G. 3546 (*lycianthoides*)

Vargas, W.G. et al. 4238 (*dimorphum*)

Vargas C., C. 1686 (*baccatum* var. baccatum)

Vargas C., I.G. 30 (*pubescens*); 36 (*eximium*); 66 (*annuum* var. annuum); 79 (*baccatum* var. baccatum); 816, 906 (*eximium*); 932 (*pubescens*); 1382 (*eximium*); 2131 (*minutiflorum*)

Vargas C., I.G. & J.M. Camacho 3118 (*caballeroi*)

Vargas C., I.G. & C.G. Jordán 7000 (*caballeroi*)

Vargas C., I.G. & E. Prado 1282, 1286 (*caballeroi*)

Vargas C., I.G. et al. 1343 (*caballeroi*); 1979 (*pubescens*); 2173 (*minutiflorum*); 2666, 2667 (*chinense*); 4151, 7233 (*caballeroi*)

Vargas Contreras, M. 141 (*baccatum* var. baccatum); 254 (*minutiflorum*)

Vargas L., H. 5065, 6292 (*lycianthoides*)

Vasconcelo Neto, J. 05/15 (*flexuosum*); 05/16, 6707 (*rabenii*); 6813 (*flexuosum*); 20857, 20989 (*recurvatum*); 21594 (*flexuosum*)

Vásquez, B. T. 611 (*annuum* var. glabriusculum)

Vásquez, M.E. 002 (*frutescens*); 011, 012, 013 (*annuum* var. annuum)

Vásquez, R. & R. Francis 27853, 28032 (*geminifolium*)

Vásquez, R. & L. Valenzuela 35377 (*geminifolium*)

Vásquez, R. et al. 25940 (*rhomboideum*); 27256 (*geminifolium*); 28196, 30563, 31010 (*dimorphum*); 31019, 31021 (*geminifolium*)

Vattuone, J. 99 (*chacoense*)

Vauthier, A-C. 526 (*muticum*); 528 (*campylopodium*)

Vázquez, B. 673, 998 (*annuum* var. annuum)

Vázquez B., F. & O. Hernández 45 (*lanceolatum*)

Vega A., R. 1297, 4511 (*annuum* var. glabriusculum)

Vejarano, S. & G. Galeano 28 (*rhomboideum*)

Velarde Nuñez, O. 1, 2, 3 (*baccatum* var. pendulum); 4 (*chinense*); 5, 6 (*baccatum* var. pendulum); 7 (*annuum* var. annuum); 9 (*chinense*); 10, 11 (*baccatum* var. pendulum); 12 (*chinense*); 13, 15 (*baccatum* var. pendulum); 16 (*chinense*); 17 (*baccatum* var. pendulum); 18, 19, 20 (*annuum* var. annuum); 21 (*baccatum* var. pendulum); 22 (*annuum* var. annuum); 23, 24, 25 (*baccatum* var. pendulum); 26 (*annuum* var. annuum); 27 (*baccatum* var. pendulum); 326 (*hookerianum*)

Velasco, L.V. 8925 (*frutescens*)

Velasco G., K. et al. 5532 (*annuum* var. glabriusculum)

Velásquez Castillo, L.G. MA-4, MA-7 (*frutescens*); MA-8, MA-9 (*chinense*)

Velásquez D., D. 113 (*annuum* var. glabriusculum)

Velázquez L., C. 104 (*pubescens*)

Vélez, C. et al. 5014 (*dimorphum*)

Vélez, M.C. et al. 1008 (*lycianthoides*)

Vélez-Puerta, J.M. et al. 3164, 4181 (*rhomboideum*)

Véliz, M. 7079, 8126 (*annuum* var. glabriusculum); 14613 (*frutescens*); 97501 (*rhomboideum*)

Véliz, M. & F. Ramírez 12818 (*annuum* var. glabriusculum)

Velloso, H. 158 (*schottianum*); 556 (*campylopodium*)

Venda, A.K.L. et al. 54 (*mirabile*)

Ventura A., F. 798 (*lanceolatum*); 3117, 3945, 4367, 9378 (*rhomboideum*); 9739 (*pubescens*); 10619 (*rhomboideum*); 11600 (*lanceolatum*); 13411, 13678, 14331 (*rhomboideum*)

Ventura V., E. & E. López 4208, 4381, 5472 (*lanceolatum*); 6332, 7328, 8925, 9887 (*rhomboideum*)

Venturi, S. 737, 1066, 1598 (*chacoense*); 4226 (*baccatum* var. baccatum); 7860 (*chacoense*)

Vera-Sánchez, L.F. 987 (*dimorphum*)

Vervloet, R.R. & W. Pizziolo 2378 (*baccatum* var. baccatum)

Vervoorst, F. 3535 (*chacoense*)

Vianna 1509 (*campylopodium*)

Viana, G. 955 (*caatingae*)

Viana, G. et al. 1986 (*caatingae*)

Viana, P.L. & A.R. Barbosa 3500 (*schottianum*)

Viana, P.L. et al. 2253 (*pereirae*)

Vibrans, H. 2003 (*annuum* var. glabriusculum)

Vickers, W.T. 115, 178 (*chinense*); 200 (*annuum* var. annuum); 208 (*frutescens*); 211 (*annuum* var. annuum); 226 (*frutescens*); 227 (*annuum* var. annuum)

Vidal, C.V. & R.L. de Paula 1157 (*carassense*)

Vidal, J. II-308, II-5672 (*mirabile*); 01490 (*flexuosum*)

Vidal, W.N. et al. 633 (*villosum*)

Viegas, A.P. 3509 (*schottianum*)

Vieira, M.C.W. 117 (*schottianum*); 2177, 2179 (*rabenii*)

Vieira, M.F. 542 (*campylopodium*)

Vieira, M.F. et al. 475 (*villosum*)

Vieira, R.F. 700 (*rabenii*); 709 (*frutescens*)

Vieira, R.F.& I. Costa 683 (*rabenii*)

Viereck, H.W. 350, 1011 (*rhomboideum*)

Vignati, M.A. 337 (*chacoense*)

Vilar, T.S. & A.A. Santos 3 (*rabenii*)

Vilcapoma, G. 84, 85 (*annuum* var. annuum); 284, 327 (*annuum* var. glabriusculum)

Villa Carenzo, M. 424, 159, 202, 2789 (*chacoense*); 3373 (*flexuosum*); 3499 (*chacoense*)

Villa Carenzo, M. & P.R. Legname 1440 (*chacoense*); 3846 (*baccatum* var. baccatum)

Villa Kamel, A. 71, 106 (*annuum* var. annuum)

Villacorta, R. & J. Giammattei 2551 (*annuum* var. glabriusculum)

Villacorta, R. et al. 2819 (*rhomboideum*)

Villafañe, M. 348, 400 (*chacoense*)

Villalobos C., G. & E. Guerrero 182, 205 (*annuum* var. glabriusculum)

Villanueva, R. 591 (*annuum* var. annuum)

Villarreal, J.A. et al. 18036 (*annuum* var. glabriusculum)

Villarroel, D. 483 (*baccatum* var. baccatum); 1677, 1712 (*minutiflorum*)

Vimercat, J.M. 124 (*baccatum* var. baccatum)

Vincent, M.A. et al. 10583 (*annuum* var. glabriusculum)

Virillo, C.B. et al. 63 (*recurvatum*)

Vivar C., F. 4003 (*frutescens*)

Vivar C., F. & B. Merino 3027 (*frutescens*)

Vivar C., F. & Estudiantes 1066 (*benoistii*)

Vogel, S. 573 (*flexuosum*)

Vogl, C. 424 (*annuum* var. glabriusculum); 709 (*rhomboideum*)

Vogt, C. & F. Mereles 231 (*chacoense*)

von Eggers, H.F.A. 322, 322 b, 583, 1993, 2247, 2796, 4715 (*annuum* var. glabriusculum); 5302 (*chinense*); 13177 (*rhomboideum*); 15555 (*hookerianum*)

von Friedrichsthal, E. 201, 310 (*annuum* var. glabriusculum)

von Humboldt, A.J.A. & F.W. Bonpland 150 (*baccatum* var. baccatum); 3027 (*pubescens*); 4518 (*annuum* var. glabriusculum)

von Martius, C.F.P. 132 (*baccatum* var. baccatum); 3074 (*schottianum*)

von Rentzell de Atkinson, I. 11 (*frutescens*)

von Rozynski, H.W. 183, 211 (*annuum* var. glabriusculum); 467 (*rhomboideum*)

von S. Medeiros, E. et al. 176 (*frutescens*); 182, 196 (*chinense*); 198, 199 (*frutescens*); 200 (*chinense*)

von Sneidern, K. 3044, 3214 (*dimorphum*)

von Sohsten Medeiros, E. et al. 353 (*pereirae*)

von Wedel, H. 724, 1237, 2610 (*annuum* var. glabriusculum)

Voorhies, B. & A. Sanchez 61-12 (*annuum* var. glabriusculum)

Wagner, R. 1215, 1632 (*annuum* var. glabriusculum)

Wagenbreth, I. 130, 687 (*annuum* var. annuum)

Wallnöfer, B. & F.M. Tut-Tesucun 6028 (*annuum* var. glabriusculum)

Walter, B.M.T. 3628 (*rabenii*)

Walter, B.M.T. et al. 566 (*baccatum* var. pendulum); 570, 575, 581, 582, 3249, 3851 (*frutescens*); 6014 (*rabenii*)

Wanderley, M.G.L. 264 (*villosum*)

Wängler, M.S. 1569 (*mirabile*)

Wängler, M.S. & V.S. Ferreira 1027 (*mirabile*)

Ward, D.B. 2836, 5555 (*annuum* var. glabriusculum)

Warner, P. 376 (*baccatum* var. baccatum)

Warner, R.H. 215 (*annuum* var. glabriusculum)

Wasum, R. 783 (*flexuosum*)

Watson, K. et al. 39 (*lanceolatum*)

Wawra, H. & F. Maly 413 (*muticum*)

Weberbauer, A. 6059 (*rhomboideum*); 7684 (*hookerianum*)

Webster, G.L. & S. Lynch 17661 (*annuum* var. annuum)

Webster, G.L. & R. Rhode 29283 (*lycianthoides*)

Webster, G.L. & T. Thompson 27964 (*lycianthoides*)

Webster, G.L. & UREP participants 27868 (*lycianthoides*)

Webster, G.L. et al. 10450, 10791(*annuum* var. glabriusculum); 28749, 30044, 30501 (*lycianthoides*); 32023 (*rhomboideum*); 32907 (*lycianthoides*)

Webster, L. 27143 (*lycianthoides*)

Webster, L. & L. Hebert 27493 (*lycianthoides*)

Weigend, M. & B.R. Ramírez 3528 (*rhomboideum*)

Weigend, M. et al. 5285 (*geminifolium*); 7528 (*rhomboideum*)

Wendelberger, K. 170 (*neei*)

Wendt, T. & D. Riskind 1611 (*annuum* var. glabriusculum)

Wendt, T. et al. 4685, 5173 (*lanceolatum*)

Werling, L. & S. Leth-Nissen 371 (*pubescens*); 397 (*lycianthoides*)

Werner, F.A. 1548 (*longifolium*); 2594 (*geminifolium*)

West, J. 3739 (*pubescens*); 8248 (*chacoense*); 8389 (*baccatum* var. baccatum)

Weston, A.S. & J. Weston 4754 (*annuum* var. glabriusculum)

Weston, A.S. et al. 4173 (*annuum* var. glabriusculum)

Weyland Vieira, M.C. 117 (*annuum* var. glabriusculum)

White, D.A. 178 (*annuum* var. glabriusculum)

White, S.S. 1606, 3640 (*annuum* var. glabriusculum)

Whitefoord, C. 5629, 7246, 10238 (*annuum* var. glabriusculum)

Whitefoord, C. & V. Quiroz 10699 (*annuum* var. glabriusculum)

Whiting, M.G. 407 (*annuum* var. glabriusculum)

Wiggins, I.L. 7368 (*annuum* var. glabriusculum); 18643 (*frutescens*)

Wiggins, I.L. & R.C. Rollins 338 (*annuum* var. glabriusculum)

Wilke, B. 5 (*annuum* var. glabriusculum)

Williams, D.E. 696 (*baccatum* var. pendulum); 764 (*frutescens*); 772, 773 (*chinense*); 932 (*baccatum* var. baccatum); 933 (*frutescens*); 934, 1177 (*chinense*)

Williams, D.E. & A. Krapovickas 1122 (*chacoense*)

Williams, K.A. et al. 121 (*baccatum* var. pendulum); 122 (*baccatum* var. baccatum); 125 (*flexuosum*); 127 (*baccatum* var. pendulum); 129, 131 (*baccatum* var. baccatum); 133 (*flexuosum*); 134 (*baccatum* var. baccatum); 135 (*baccatum* var. pendulum); 136 (*baccatum* var. baccatum); 140 (*chacoense*); 210 (*rhomboideum*)

Williams, L. 1277 (*annuum* var. glabriusculum); 3405 (*pubescens*); 5224 (*annuum* var. glabriusculum); 5225 (*chinense*); 5785 (*annuum* var. glabriusculum); F-8202 (*chinense*); 9538 (*frutescens*); 10595 (*rhomboideum*); 10597 (*annuum* var. glabriusculum)

Williams, L.O. & A. Molina R. 14671 (*annuum* var. glabriusculum)

Williams, L.O. & R.P. Williams 18295 (*annuum* var. glabriusculum)

Williams, L.O. et al. 25674, 25950, 26924, 43125, 43612 (*lanceolatum*)

Williams, R.S. 623 (*coccineum*); 634 (*coccineum*); 715 (*annuum* var. glabriusculum)

Wilson, P. 1019, 1393, 7512, 9468 (*annuum* var. glabriusculum)

Wolff, R.A. 16 (*baccatum* var. baccatum)

Wood, C.E. et al. 9220 (*annuum* var. glabriusculum)

Wood, J.R.I. 8774 (*eximium*); 9060 (*caballeroi*); 9104 (*neei*); 11102 (*caballeroi*); 14892 (*coccineum*); 22267 (*baccatum* var. baccatum)

Wood, J.R.I. & M. Atahuachi 21581 (*eximium*)

Wood, J.R.I. & A. Carretero 19317 (*chacoense*)

Wood, J.R.I. & D.J. Goyder 15579 (*baccatum* var. baccatum); 16736 (*eximium*);

Wood, J.R.I. & J. Gutiérrez 20218 (*eximium*)

Wood, J.R.I. & H. Huaylla 21532 (*eximium*)

Wood, J.R.I. & M. Menacho 12642 (*coccineum*)

Wood, J.R.I. & M. Mendoza 19144 (*chacoense*); 19165, 19166, 21490 (*eximium*); 22105 (*caballeroi*)

Wood, J.R.I. & M. Serrano 13331 (*eximium*)

Wood, J.R.I. & D. Wasshausen 13893 (*coccineum*)

Wood, J.R.I. et al. 21229 (*eximium*); 21792 (*chacoense*); 22339 (*baccatum* var. baccatum); 22449 (*eshbaughii*); 22798 (*baccatum* var. baccatum); 22801 (*coccineum*)

Woodmansee, S.W. et al. 432 (*annuum* var. glabriusculum)

Woodson Jr., R.E. & R.W. Schery 840 (*annuum* var. glabriusculum)

Woolston, A. 165 (*baccatum* var. baccatum); 729 (*flexuosum*)

Wooton, E.O. 48, 49 (*annuum* var. annuum)

Woytkowski, F. 1176 (*coccineum*); 5678 (*rhomboideum*); 7516, (*coccineum*); 35161 (*annuum* var. glabriusculum)

Wright, C. 384, 537 (*annuum* var. glabriusculum)

Wullschlaegel, H.R. 396 (*annuum* var. glabriusculum)

Wunderlin, R.P. 5306 (*annuum* var. glabriusculum)

Wunderlin, R.P. et al. 6246 (*annuum* var. glabriusculum)

Wussow, J. & G. Landry 253 (*rhomboideum*)

Wydler, H. 264 bis (*annuum* var. glabriusculum)

Wynd, F.L. 687 (*annuum* var. glabriusculum)

Yáñez, A.P. & R. Foster 262 (*lycianthoides*)

Yañez, A.P. et al. 1407 (*frutescens*); 1485 (*annuum* var. annuum)

Yañez, M. 440, 572 (*annuum* var. glabriusculum); 783 (*rhomboideum*)

Yepes-Agredo, S. 1172 (*rhomboideum*)

Young, K. & G. Sullivan 713 (*coccineum*)

Yuncker, T. 17118, 17253, 18460 (*annuum* var. glabriusculum)

Zak, V. 836 (*lycianthoides*); 1423 (*rhomboideum*); 1433 (*lycianthoides*); 1752, 1759 (*geminifolium*); 1970 (*rhomboideum*); 9804 (*lycianthoides*)

Zak, V. & J.L. Jaramillo 2147, 2220, 3099 (*lycianthoides*)

Zamora C., P. 24, 25 (*annuum* var. annuum)

Zamora C., P. & M.G. Zola B. 4 (*annuum* var. annuum)

Zamora, V.H. 193 (*eximium*)

Zanoni, T. & M. Mejía 17002, 17077 A, 26168 (*annuum* var. glabriusculum)

Zanoni, T. & J. Pimentel 25313 (*annuum* var. glabriusculum)

Zanoni, T. et al. 12012, 21507, 22115, 30601, 32161, 42707 (*annuum* var. glabriusculum)

Zapata C., D.A. 827 (*geminifolium*)

Zardini, E.M. 1739 (*baccatum* var. pendulum); 1870 (*eximium*); 5771, 7166, 7200 (*flexuosum*); 10972 (*rabenii*); 11076 (*flexuosum*); 30803 (*rabenii*); 38869 (*chacoense*); 41733, 41772, 45402, 47751, 50492, 53141, 53151 (*flexuosum*)

Zardini, E.M. & A. Acosta 42322, 42328, 42346, 42450, 42445 (*chacoense*); 56060 (*baccatum* var. baccatum)

Zardini, E.M. & A. Aguayo 9508 (*rabenii*); 9522, 9549, 9729 (*baccatum* var. baccatum)

Zardini, E.M. & C. Balbuena 42915 (*baccatum* var. baccatum)

Zardini, E.M. & A. Benítez 47532 (*flexuosum*)

Zardini, E.M. & R. Franco 30370 (*baccatum* var. baccatum)

Zardini, E.M. & J. Godoy 50089 (*chacoense*)

Zardini, E.M. & L. Guerrero 33706 (*baccatum* var. baccatum); 37883, 37913, 37916 (*chacoense*)

Zardini, E.M. & D. Quintana 53786 (*baccatum* var. baccatum)

Zardini, E.M. & J.C. Rivas 58461 (*chacoense*)

Zardini, E.M. & T. Tillería 39351 (*chacoense*)

Zardini, E.M. & M. Velázquez 10019, 13104 (*baccatum* var. baccatum)

Zardini, E.M. & R. Velásquez 8922 (*baccatum* var. baccatum); 12460, 12920 (*flexuosum*); 13856 (*baccatum* var. pendulum); 17171 (*baccatum* var. baccatum)

Zardini, E.M. & U. Velásquez 15600 (*flexuosum*)

Zardini, E.M. et al. 1641, 1740 (*eximium*); 12774 (*baccatum* var. baccatum); 12744 (*rabenii*); 19653 (*chacoense*)

Zarucchi, J.L. 2173 (*frutescens*)

Zarucchi, J. & D. Cárdenas L. 4366 (*dimorphum*)

Zarucchi, J. et al. 4978 (*annuum* var. glabriusculum); 5476 (*rhomboideum*); 5478 (*annuum* var. glabriusculum); 5491 (*rhomboideum*)

Zelaya, L. 239 (*annuum* var. glabriusculum)

Zenteno, F. et al. 3608 (*eximium*); 12798 (*baccatum* var. baccatum)

Zizumbo, D. & P. Colunga 145 (*annuum* var. annuum); 220, 299 (*annuum* var. glabriusculum)

Zola B., M.G. 815 (*annuum* var. glabriusculum)

Zola B., M.G. & R.M. Baizabal 1459, 1467, 1496, 1500, 1503, 1506, 1511, 1514, 1548, 1550, 1599, 1620 (*annuum* var. annuum)

Zola B., M.G. & P. Zamora 1078, 1081, 1096, 1110, 1138, 1243, 1249, 1261, 1277 (*annuum* var. annuum)

Zollinger, H. 489 (*annuum* var. annuum)

Zorzanelli, J.P. 957, 968 (*pereirae*)

Zuchiwschi, E. 55 (*rabenii*)

Zuloaga, F. et al. 5171, 5431 (*flexuosum*); 6377 (*baccatum* var. baccatum); 6722 (*flexuosum*).

## Supplementary materials

Supplementary material 1 Appendix 1. Anatomical characters of *Capsicum* fruits Data type Morphological (excel file) Explanation note Description of fruit anatomical characters for each *Capsicum* species. https://binary.pensoft.net/file/695537This dataset is made available under the Open Database License (http://opendatacommons.org/licenses/odbl/1.0/). The Open Database License (ODbL) is a license agreement intended to allow users to freely share, modify, and use this Dataset while maintaining this same freedom for others, provided that the original source and author(s) are credited.Gloria E. Barboza, Carolina Carrizo García, Luciano de Bem Bianchetti, María V. Romero, Marisel Scaldaferro

Supplementary material 2 Appendix 2. Seed characters and seed coat features in *Capsicum* Data type Morphological (excel file) Explanation note Description of seed and seed coat characters for each *Capsicum* species. https://binary.pensoft.net/file/695538This dataset is made available under the Open Database License (http://opendatacommons.org/licenses/odbl/1.0/). The Open Database License (ODbL) is a license agreement intended to allow users to freely share, modify, and use this Dataset while maintaining this same freedom for others, provided that the original source and author(s) are credited.Gloria E. Barboza, Carolina Carrizo García, Luciano de Bem Bianchetti, María V. Romero, Marisel Scaldaferro

Supplementary material 3 Appendix 3. Comparative data on distribution, habitat, biomes and number of ecoregions inhabited by *Capsicum* taxa Data type Occurences (excel file) Explanation note Details of biomes and ecoregions are not applicable to domesticated taxa. https://binary.pensoft.net/file/695539This dataset is made available under the Open Database License (http://opendatacommons.org/licenses/odbl/1.0/). The Open Database License (ODbL) is a license agreement intended to allow users to freely share, modify, and use this Dataset while maintaining this same freedom for others, provided that the original source and author(s) are credited.Gloria E. Barboza, Carolina Carrizo García, Luciano de Bem Bianchetti, María V. Romero, Marisel Scaldaferro

Supplementary material 4 Appendix 4. Specimens examined Data type Occurences (PDF file) Explanation note Full information of the specimens examined for each *Capsicum* species. https://binary.pensoft.net/file/695540This dataset is made available under the Open Database License (http://opendatacommons.org/licenses/odbl/1.0/). The Open Database License (ODbL) is a license agreement intended to allow users to freely share, modify, and use this Dataset while maintaining this same freedom for others, provided that the original source and author(s) are credited.Gloria E. Barboza, Carolina Carrizo García, Luciano de Bem Bianchetti, María V. Romero, Marisel Scaldaferro
