# Supplementary material for: ﻿Monograph of wild and cultivated chili peppers (Capsicum L., Solanaceae)
Source: PhytoKeys. 2022 Jun 14;200:1–423. doi: 10.3897/phytokeys.200.71667 (PMC9881532; doi:10.3897/phytokeys.200.71667)
Supplement: Supplementary material 4 — Appendix 4. Specimens examined [file phytokeys-200-001_article-71667__-s004.pdf]

Supplementary Material. Appendix 4

Specimens examined

We list here in traditional format all specimens examined for this treatment. Countries, major divisions within them (when known), and collectors (by surname) are listed in alphabetic order. The specimens cultivated at greenhouses, botanical gardens or plants/fruits bought at different markets are cited under Cultivated.

1a. Capsicum annuum L. var. annuum

ARGENTINA. Córdoba: Oncativo, 26 Mar 1970, *C.A. Guzmán s.n.* (CORD 00029473); from seeds bought at Disco Supermarket, 9 Mar 1995, *A.T. Hunziker s.n.* (CORD 00029428); cult. entre San Pedro y el Chocolate, camino a Chancaní, 4 Jan 1956, *A.T. Hunziker 11492* & *11493* (CORD); ciudad, comprado en Supermercado Disco, 9 Mar 1995, *A.T. Hunziker 29428* (CORD); cult. Invernadero IMBIV, semillas de Mercado de Villamontes (Tarija), Bolivia, 8 Aug 2013, *M. Scaldaferro 34* (CORD); Ruta Nacional 9, Pilar, 16 Feb 1975, *R. Subils 2064* (CORD); **Corrientes**: Capital, inmediaciones Avda. Maipú, 21 Sep 1972, *Anzótegui-Benitez 109* & *237* (CTES); Concepción, Tabay, 1 Jan 1972, *M.M. Arbo 341* (CTES); Mburucuyá, Estancia San Teresa, 30 Apr 1972, *A. Krapovickas 22180* (BAA); ciudad, 1 Jun 1973, *A. Krapovickas 23686* (CTES); Santa Ana, Granja Sapucai, 4 Jul 1976, *A. Schinini 13279* (CTES); **Distrito Federal**: cult. Facultad de Agronomía-UBA, 20 Apr 1968, *A.A. García 3190* (BAF); cult. Facultad de Agronomía-UBA, *A.T. Hunziker s.n.* (CORD00040980); ciudad, 5 Apr 1948, *J.H. Hunziker 2450* (CORD); **Jujuy**: Capital, Ciudad, Mar 1957, *M. Medinaceli s.n.* (BAF 8691); Capital, 18 Mar 1992, *E.A. Moscone 204* (CORD); **Misiones**: San Ignacio, Arroyo Ñacanguazú, 19 Feb 1971, *A. Burkart et al. 28424* (CORD, SI); San Javier, San Javier, a 200 m del río Uruguay, cerca del puesto de Gendarmería, 12 Jan 2002, *F. Chiarini 545* (CORD); Iguazú, Arroyo Urugua-í y ruta 12, selva desde el puente hasta 3 km sobre la ruta 12, rumbo a Puerto Iguazú, 29 May 1987, *R. Subils & Moscone 4142* (CORD); La Península, ruta 12, a ca. 8 km del PN Iguazú, picada del obraje Mbocai, rumbo a Bosetti, 30 May 1987, *R. Subils & Moscone 4166* (CORD); **Salta**. Capital, cult. en jardín de la ciudad, 15 Feb 1943, *A.T. Hunziker s.n.* (CORD); Cerrillos, invernáculo de la Estación Experimental INTA, 28 Mar 1995, *A.T. Hunziker 25492* (CORD, LIL, SI); Cachi, Ruta provincial 33, Payogasta, cultivado en finca Ruiz de los Llanos, 29 Mar 1995, *A. T. Hunziker 25498, 25550* & *25501* (CORD); Guemes, Jan 1948, *F. Rial Alberti s.n.* (BAB); **San Juan**: Chimbas, Barrio Santo Domingo, 12 Apr 1995, *L. Ariza Espinar 3213* (CORD); Capital, ciudad de San Juan, 18 Oct 2000, *R. Subils 4673* (CORD).

BOLIVIA. Beni: Gral. Ballivián y Yacuma, Estación Biológica del Beni, comunidad Totaizal, huerta de María Caiti, 14°30'S, 66°37'W, 200 m, 8 Feb 1993, *J. Balderrama 10* (CORD, LPB, QCNE, SI); Estación Biológica del Beni, comunidad Puerto Méndez, canchón de don Daniel, 14°30'S, 66°37'W, 200 m, 18 Oct 1994, *G. Caity 190* (LPB, USZ); San Borja, comunidad Tacuaral del Matos, 14º59'50"S, 66º33'00"W, 200 m, 13 Oct 1997, *J.P. Ticona & F. Saravia May 10* (LPB); **La Paz**: Nor Yungas, del puente sobre el Rio Beni, W del Rio Beni, 9 km hacia Litoral, 30 Mar 1986, *S. Beck 13372* (CORD, MO, SI); Trinidad, 236 m, 7-11 Nov 1947, *R. Scolnik & R. Luti 637* (CORD); cult. from seeds, purchased in Mercado Lanza, la Paz, 7 Mar 1980, *J.C. Solomon 5306* (LE); **Santa Cruz**:

Cordillera, Alto Parapetí, Hacienda Yapumbia, 800 m, 8 Feb 1986, *R. de Michel 517* (LPB); A. Ibañez, La Miel, 17 km WSW (línea recta) del centro de Santa Cruz, 17°48'S, 63°21'W, 460 m, 24 Sep 1989, *M. Saldías P. 759* (USZ, NY); **Tarija**: Gran Chaco, Saladillo, 63º45'W, 21º36'W, 1000 m, 8 Apr 1977, *A. Krapovickas & A. Schinini 31057* (CTES).

BRAZIL. Paraná: Ponta Grossa, Vila Velha, alrededores del camping, 12 Jan 1987, *A. Krapovickas & C.L. Cristóbal 40806* (CTES); **Rio de Janeiro**: Horto Botánico, Sep 1924, *A. Cieri s.n.* (R 65468); Barra da Piraí, Fazenda Ponte Alta, 14 Dec 1980, *A.H.G. Pascali 8* (CORD); **Rio Grande do Sul**: Guaporé, Río Colorado, 23 Jan-2 Feb 1948, *R. Scolnik & R. Luti 703* (CORD); **Santa Catarina**: Brusque, Azambuja, 2 May 1952, *P.R. Reitz 4750* (US); **São Paulo**: Charqueada, Sítio São Sebastião, 22 Oct 1990, *P. Duth s.n.* (RB 619338); Cantareira, cul. No Horto da Cantareira, *W. Hoehne 30971* (US).

CHILE. Concepción: Valparaíso: Limache, 90 m, 1 Apr 1917, *K. Behn s.n.* (CONC 22096); Concepción, Jardín Experimental, 3 Mar 1936, *C. Junge 5755* (CONC).

COLOMBIA. Amazonas: Leticia, Monilla Amena, km 9.8 via Leticia-Tarapaca, 4º6'56''S, 69º55'38''W, 90 m, 15 Feb 2006, *A. Alvarado C. 198* (COL); margen izquierda del Río Miriti, comunidad Guayabo, chagra T. Tanimuka y H. Yucuna, 0º58'15.8''S, 70º35'13.2''W, 244 m, *D. Cárdenas et al. 9406* (COL); Margen derecha del Río Miriti, comunidad Centro Providencia, Puerto Yacuna, 1°7'19''S, 70°13'36.2''W, 244 m, *D. Cárdenas et al. 9424* (COL); corregimiento La Pedrera, comunidad Camaritawa, 1°11'27''S, 69°35'13''W, 79 m, *D. Cárdenas et al. 9438* (COL); Puerto Santander, Resguardo indígena Nonuya de Villazul, comunidad de Peña Roja, alrededores de la Estación de Tropenbos, 100-200 m, 12 Apr 2000, *S. Castro et al. 238* (COL); Isla Sumaeta, chagra de E. Moreno, 100-200 m, 17 Apr 2000, *S. Castro & Virgelina 305* & *307* (COL); La Pedrera, Resguardo Indígena Curare-Los Ingleses, comunidad Curare, ca. 17º17'S, 69º44'W, 100 m, 23 Apr 2004, *Z. Cordero-P. 683* (COL); entre los ríos Loretoyacu y Hamacayacu, orillas del Loretoyacu, 250 m, Nov 1945, *J.M. Duque-Jaramillo 2036* (COL); Igará Paraná, Milán, chagra L. Buraiño, 16 Jan 1988, *C.I. Henao & R. Kuiru 172* (COL); same locality, chagra A. Kuitu, 23 Jan 1988, *C.I. Henao & K. Rubiela 316* (COAH, COL); Puerto Nariño, *A. Posada 2581* (COAH); km 11, costado oriental, finca P. Morales, 12 Oct 1994, *C. Torres & P. Morales 139* (COL); Araracuara, sede Experimental Hortalizas, 11 Sep 1984, *M.E. Vásques 011*, *012* & *013* (COAH); **Antioquia**: en los alrededores de Dabeiba, ca. 1350 m, 20 Dec 1947, *F.A. Barkley & G. Gutierrez V. 1765* (COL, CORD, MEDEL); **Boyacá**: Togüí, 10º65'73.1''N, 114º62'00''W, 2146 m, 22 Jun 2008, *A. Hernández & F. Crespo 82* & *83* (UDBC); **Cauca**: In Popayán, 1740 m, 17 Feb 1884, *F.C. Lehmann 3577* (US); **Caquetá**: Araracuara Shuchichi, 0°40'49''S, 72°32'51''W, 244 m, *D. Cárdenas et al. 9306* (COL); Puerto Santander, margen derecha del Río Caquetá, 0°37'7.1''S, 72°23'8.4''W, 213 m, *D. Cárdenas et al. 9310* (COAH) & *9312* (COL); Rio Yari, resguardo Amenami, 0°32'34.9''S, 72°19'34''W, 244 m, *D. Cárdenas et al. 9319* (COL); Chorro del Quinche, margen derecha del rio Caquetá, 0°55'50''S, 71°46'47''W, 335 m, *D. Cárdenas et al. 9360* (COL); margen derecha del Río Caquetá, San Francisco, 1°6'21.3''S, 71°6'18''W, 183 m, *D. Cárdenas et al. 9380* (COL); **Cauca**: houseyard in Finca El Trigal midway between Rio Guachicono and Panamerican Highway, 960 m, 30 Jan 1976, *T.C. Plowman & D. Vaughn 5370* (COL); **Chocó**: Acandi, Bahía El Aguacate,

camino hacia la quebrada La Mora, 30 Jun 2005, *S.E. Hoyos-Gómez et al. 355* (COL); Alto Baudó, Resguardo Indígena Emberá, Santa María de Condoto, 280 m, 9 Jul 1985, *C. La Rotta & H. Martínez 737* (COL); Coquital, 100-150. 12 Aug 1996, *S. Márquez & B. Vecino 119* (JAUM); **Cundinamarca**: Mosquera, invernadero de Tibaitatá, 2700 m, 13 Apr 1967, *M. Correa N. 006* (COL); línea férrea entre Cachipay y Petaluma, 1700 m, 10-15 May 1946, *J.M. Duque-Jaramillo 3555* (COL); Cota, Vereda Cetime, 2547 m, 25 Nov 2005, *J. Jiménez 02* (COL); Distr. Capital, Bogotá, Plaza de mercado de Paloquemado, 2600 m, 8 Oct 2005, *C.X. Robayo 1* (COL); **Guainía**: Caserío de Karanacoa, en el Río Guainía, 150 m, 12 Oct 1977, *J. Espina et al. 189* (COL); Puerto Inirida, comunidad Matraca, margen izquierda del rio Inirida, 2º52'23''N, 69º5'36''W, *C. Marín & F. Rodríguez 525* (COAH); **Meta**: Villavicencio, Vereda Barcelona, Universidad de los Llanos, 8 Dec 2005, *E. Álvarez & O. Montañéz 1* (COL); Puerto Gaitán, Río Meta, El Porvenir, 16 Oct 1938, *J. Cuatrecasas & H. García Barriga 3693* (F, COL, US); **Valle del Cauca**, Palmira, *CIAT s.n.* (FMB 50161); Palmira, cultivos en la Universidad Nacional de Colombia, sede Palmira, *F. Rodríguez s.n.* (FMB 50163); **Nariño**: Buesaco, Vereda El Cortijo, a 13 km de la vía de Villamoreno, 2500 m, 29 Apr 1984, *O. de Benavides 4673* & *4674* (PSO); La Florida, Corregimiento de Natituy, 1800 m, 5 Sep 1985, *O. de Benavides 5698* (PSO); Sandoná, cercanías de la población, 2000 m, 12 Apr 1986, *O. de Benavides 6387* (PSO); **Vichada**: Cumaribo, margen izquierda del rio Uva, comunidad Manajuare, 3°45'42.6''N, 69°22'42.9''W, 750 ft, *F. Rodríguez 165* (COL).

COSTA RICA. Heredia: Finca La Selva, the OTS Field Station on the Río Puerto Viejo, just E of its junction with the Río Sarapiquí, 100 m, 14 Nov 1982, *T. McDowell 796* (CAS, F).

DOMINICAN REPUBLIC. **San Pedro de Macoris**, Cumayasa, S and SE of town of Boca de Cumayasa, 18º24'N, 69º06'W, 5-10 m, 31 Jul 1980, *M. Mejía Pimentel & T. Zanoni 7738* (NY).

ECUADOR. Carchi: Environs of Chical, 12 km below Maldonado on the rio San Juan, 1º04'N, 78º17'W, 1200 m, 28 May 1978, *M.T. Madison et al. 4675* (QCA); **Chimborazo**: Pallatanga, San José de Pricipamba, a 500 m del Rio Coco, Estación INIAP, 01º60'S, 78º58'W, 1630 m, 11 Jan 2003, *W. Ganchozo 007* (QCNE); Cantón Guamote, comuna Santa Lucía Bravo, 3126 m, 3 Jan 2000, *S. León S. 036* (CHEP); Penipe Cantón, 1º34'S, 78º33'W, 2500 m, 15 Jan 2004, *D. Paredes 845* (CHEP); **Esmeraldas**: Cantón Eloy Alfaro, comuna de Calle Mansa, 00º44'N, 78º53'W, 130-180 m, 12 Aug 1993, *A.P. Yañez et al. 1485* (QCNE); **Guayas**: Chongón Km 27 via Guayaquil-Salinas, 10 m, 2 Feb 2001, *C. Bonifaz & X. Cornejo 4158* (GUAY); Guayaquil, en jardín, 27 Oct 1963, *F.M. Valverde 35* (COL) & 11 Jun 1964, *F.M. Valverde 392* (COL); **Morona-Santiago**: Rio Tsuirim near Miazal, 26 Oct 1990, *D.K. Evans 4384* (QCA); **Napo**: Cantón Tena, Rio Blanco community, headwaters of Rio Huambuno, 6 km NNW of Ahuano, 01º00'S, 77º40'W, 440 m, 21-26 May 1990, *E. Kohn 1225* (QCNE); Ahuano, 1º6'S, 77º30'W, 310 m, 9 Jun 1991, *M. Rios & E. Vivanco 383* (QCA); Río Aguarico, Shushufindi, 244 m, 7 Mar 1975, *W.T. Vickers 200* (CHEP) & *211* (QCA); Río Aguarico, Shushufindi, in house garden, 4 Mar 1975, *W.T. Vickers 227* (QCA); **Orellana**: Aguarico, Parroquia Nuevo Rocafuerte, comunidad Martinica, *D. Reyes & L. Carrillo 1048* (QCNE); **Pastaza**: Parroquia Puyo, Jardín Botánico "Las Orquídeas", 01º28'56''S, 77º59'40''W, 900 m, 10 Jun 2005, *D. Castillo et al. 029* (CHEP); Villano B, en el sendero entre Villano B y márgenes del Río

Liquino, 1º27’10’’S, 77º26’32’’W, 350 m, 11 Mar 2008, *J. Jaramillo et al. 26264* (QCA); **Pichincha**: Quito, 2850 m, 20 Aug 1949, *M. Acosta Solís 13577* (F); Reserva Geobotánica del Pululahua, entre la Caldera del Volcán y la Base del Cerro Mauca Quito, 00.05N, 78.30W, 2650 m, *C.E. Cerón 18842* (QCNE); cantón Sangolqui, Barrio Selva Alegre, huerta particular, *F. Mejía 001* (QAP); Quito, Cdla. Jipijapa, 2820 m, 20 Jun 1983, *L. Muñoz 257* (QCA); Carretera Quito-Puembo, 0º10'S, 78º22'W, 2450 m, 5 Jun 1988, *C. Narváez 018* (QCA); Carretera Quito-Puerto Quito, 10 km al N de la carretera principal, km 113, 00º05'N, 79º02'W, 650-700 m, 17 Jan 1987, *M. Rios & F. Ghia 131* (QCA); **Sucumbíos**: Cuyabeno, comunidad Secoya de Centro Eno, en la ribera del Río Aguarico, a 15’ de Posa Honda, 00º11’17.7’’S, 76º29’36.6’’W, 235 m, 16-25 Jun 2004, *N. Miranda-Moyano & G. Moya 334* (QCNE); **Tungurahua**: de Ambato a Huachi, 2650-2700 m, *M. Acosta Solís 8848* (F).

EL SALVADOR. San Salvador, in gardem 1922, *S. Calderón 523* (US).

GUATEMALA. **Alto Verapaz**: Trece Aguas, 17 Apr 1905, *G.P. Goll 271* & *273* (US); **Quezaltenango**: Zunil, 2 Jan 1940, *J.A. Steyermark 34462* (F); **Petén**: La Libertad and vicinity, Aug-Nov 1933, *M.H. Aguilar* 78 (F, MICH).

GUYANA. Central Horticultural Station, East Bank Demerara, 20 Jul 1970, *Omawale & R. Persaud 130* (NY).

HONDURAS. **Copán**: vicinity of Santa Rita, 750 m, 6 Jan 1983, *A. Molina R. et al. 33572* (MEXU); **Cortes**: El Cajón Santa Cruz de Yojoa, 500 m, 8 Sep 1983, *S. Chevez 40* (MEXU); **Morazán**: on road to aldea El Jicarito, 900 m, 23 Sep 1987, *A. Molina R. 34008* (MEXU); vicinity of Zemurray Hall, on Campus of EAP El Zamorano, 23 Jun 1997, *A. Molina R. & A.R. Molina 34535* (MEXU).

MEXICO. **Campeche**: Calakmul, a 2 km al SE de Dos Naciones, 17°57’41’’N, 89°20’33’’W, 170 m, 17 Oct 1997, *E. Martínez S. et al. 29428* (MEXU); ejido Narciso Mendoza, km 33 al S de Xpujil, 18°13’40’’N, 89°27’12’’W, 240 m, 12 Oct 1997, *S. Ramírez A. 56* (MEXU); **Chiapas**: Chamula, in cornfield at Toh Tik Paraje, 6300 ft, 26 Aug 1964, *D.E. Breedlove 7155-A* (CAS); Venustiano Carranza, in the sitios of Aguacatenango, 5800 ft, 22 Jul 1965, *D.E. Breedlove 11248* & *11249* (CAS); Tenejapa, along the Tanate River, in the barrio of Habenal, paraje of Mahbenchauk, 3300 ft, 11 Aug 1965, *D.E. Breedlove 11716* (CAS, US); San Cristóbal de las Casas, 7100 ft, 22 Apr 1968, *D.E. Breedlove 16083* (CAS, MEXU); Palenque, along the ridges 6-12 km south of Palenque on the road to Ocosingo, 300 m, 27 Jul 1972, *D.E. Breedlove 26518* (CAS, MEXU); Tuxtla Chico, Rancho San Antonio, 350 m, 9 Nov 1977, *J.I. Calzada et al. 3774* (MEXU); Arriaga, Cerro Monte Bonito, 1 km al SE de Col. Alianza, 16°19’10’’N, 93°51’15’’W, 550 m, 20 Nov 2002, *R. Cruz D. & A. Reyes 5674* (MEXU); Chiapas de Corzo, El Chorreadero, 5-6 miles east of Chiapa de Corzo along Mexican Highway 190, 2500 ft, 21 Jun 1966, *R.M. Laughlin 1116* (CAS, MEXU); at Soyatitán, along the road from Pinola Las Rosas to Pugiltic, 3500 ft, 12 Sep 1966, *R.M. Laughlin 2013* (CAS); Ixtapa, in the Zinacantán paraje of Muk’tahok’, 3200 ft, 26 Sep 1966, *R.M. Laughlin 2117, 2118* & *2119* (CAS); near Rancho Carmen along the road from Acala to V. Carranza, 2500 ft, 25 Oct 1966, *R.M. Laughlin 2652* (CAS, US), *2653* (CAS, MEXU) & *2657* (CAS); same

locality, 7 Jan 1967, *R.M. Laughlin 3017* (CAS) & *3018* (MEXU, US); Tenejapa, along the Tanate River, 3200 ft, Jan-Mar 1964, *A.S. Ton 274* & *275* (CAS); **Guanajuato**: Acambaro, a orilla de la carretera de Acambaro, 10 Apr 1995, *C. Salinas & S. Benítez 240* (MEXU); Dolores Hidalgo, Rancho Paloma Prieta, 22 Jun 1995, *S. Benítez et al. 386* (MEXU); Rosalito, 22 Jun 1995, *S. Benítez et al. 387* (MEXU); Salamanca, carretera Irapuato-Celaya, km 40, 20 May 1996, *S. Benítez 688* & *689* (MEXU); Xichú, Los Pablos, 21°14’0.1’’N, 100°01’22’’W, 1514 m, 18 Aug 2013, *M.T. Vieyra s.n.* (MEXU); **Guerrero**: Ayutla de los Libres, Tepango, 600 m, 31 Oct 1999, *A. Díaz Rico ADR-221* (MEXU); Malinaltepec, Malinaltepec, 1600, 15 Aug 1989, *I. Wagenbreth 130* (MEXU); same locality, 1500 m, 24 Jun 1991, *I. Wagenbreth 687* (MEXU); **Hidalgo**: Acoxochitlan, San Francisco Atotonilco, 1750 m, 22 Aug 1984, *A. Villa Kamel 71* & *106* (MEXU); **Jalisco**: Guadalajara, Jul-Oct 1886, *E. Palmer 640* (IND, U, US); Guadalajara, 25 Sep 1886, *E. Palmer 642* (P, U, US); **Mexico**: Ecatzingo, 18° 57.3’N, 98°45.1’W, 2400 m, 14 Aug 1994, *E. Linares 846* (MEXU); Teotihuacán, en el Jardín Botánico del museo Arqueológico, en el predio de las pirámides, 24 Oct 1998, *R. Subils & G. Barboza 4611* (CORD); **Michoacán**: Morelia, Feb 1911, *F. Arséne s.n.* (BAF 8706); same locality, 24 Aug 1911, *F. Arsène s.n.* (MEXU); Zitácuaro, San Francisco Coatepec, Quinta D. Kennedy, 19.421°, 100.374°, 1900 m, 6 Aug 2011, *R. Bye et al. QD 228* (MEXU); same locality, 1 Sep 2011, *R. Bye et al. QD 246* (MEXU); Huetamo, 23 Mar 1983, *E. Martínez S. & J.C. Soto 3640* (MEXU); Tarimbaro, 17 Jun 1999, *A. Miranda et al. 1519, 1519 A* & *1519 B* (MEXU); en Guanoro, 18 km al SW de Zitácuaro, 1600 m, 10 Oct 1983, *J.C. Soto Núñez et al. 5442* & *5443* (MEXU); Huetamo, en el solar de una casa, 26 Dec 1991, *J.C. Soto Núñez 14047* & *14048* (MEXU); Huetamo, 380 m, 1 Aug 2002, *J.C. Soto & G. Silva R. 14103* (MEXU); Zitácuaro, Km 91.5 carr. Toluca-Zitácuaro, 1850 m, 1 Nov 2002, *J.C. Soto Núñez 14302* (MEXU); **Nuevo León**: Jesús Ma. de Berrones, 1600 m, *R. Cárdenas R. PU. 0558* (MEXU); **Oaxaca:** Ciénaga, 2 km al N de Zimatlan, 1500 m, 20 Jan 1993, *S. Acosta Castellanos 9417* (MEXU); Tehuantepec, al W de San Mateo del Mar, 15-22 Oct 1976, *A.G. Bamonte 77* & *79* (MEXU); San Lucas Ojitlán, Río Culebra, 17 Apr 1989, *J.I. Calzada 14823* (MEXU); Santa María Jacatepec, a 4 km del poblado La Joya, carretera a Macedonia, 150 m, 18 Jun 1990, *J.I. Calzada & H.V. Der Well 15483* (MEXU); Oaxaca, 1550 m, 5 Sep 1899, *V. González 975* (GH, K, MEXU); San Juan Guichicovi, Jardín del INI, Jul 1986, *M. Heinrich 7800* (MEXU); San Juan Bautista, Tuxtepec, Presa Cerro de Oro, 4 Mar 1989, *R. Hernandez Ortega 481*, *482* & *484* (MEXU); San Juan Mixtepec, Miahuatlán, 16°18’06’’N, 96°18’01’’W, 2070 m, 27 Jun 1997, *E. Hunn OAX-1341, OAX-1342, OAX-1343, OAX-1344* & *OAX-1345* (MEXU); Santo Domingo Yodohino, Huajuapan de León, 1653 m, 14 Sep 2003, *J. López Moreno & E. Cedillo Portugal 29* (MEXU); Chiltepec, Tux., 14 m, 19 Feb 1968, *G. Martínez Calderón 1643* (UC); San Mateo del Mar, 5 m, 20 Jan 1978, *D. Zizumbo & P. Colunga 145* (MEXU); **Quintana Roo**: Felipe Carrillo Puerto, a 10 km al NW de Vigía Chico, cerca del Rancho El Ramonal, 18 Sep 1983, *E. Cabrera & O. Canul 5513* (MEXU); Chancah Veracruz, 10 m, 23 Apr 1983, *E. Gutiérrez 26* (MEXU); km 4 carretera Vigia Chico-F. Carrillo, 10 m, 25 Jan 1983, *E. Gutiérrez 109* (MEXU); Puerto, X-Hazil, 28 Mar 1985, *J.P. Gutiérrez 85-23, 85-26* & *85-27* (MEXU); same locality, 18 May 1985, *J.P. Gutiérrez 85-41* & 19 Dec 1985, *J.P. Gutiérrez 85-71* (MEXU); km 3 carretera Vigia Chico-F. Carrillo Puerto, 19°41’N, 87°36’W, 4 m, 15 Jan 1986, *R. Villanueva 591* (MEXU); 6.5 mi S of Felipe Carrillo Puerto, 19°30’N, 88°03’W, 4 Aug 1972, *G.L. Webster & S. Lynch 17661* (MEXU); **San Luis de Potosí**: Ejido Rincón del Porvenir, 29 May 1996, *S. Benitez 612* (MEXU); **Sonora**: Guaymás, 1887, *E. Palmer 135*

(UC), *137* (MICH), *138* & *139* (UC, US); **Tabasco**: Balancán, 170 m, 7 Apr 1976, *J.I. Calzada 2367* (MEXU); desviación para el Ejido Apatzingan, 7 apr 1976, *J.I. Calzada 2368* (MEXU); Nacajuca, a 6 km carretera a Tecolutla, 26 m, 9 Oct 1978, *J.I. Calzada 04954* (MEXU); Centro, en los alrededores de la laguna Río Muerto en la Col. Las Gaviotas, 3 Sep 1986, *M.A. Magaña 1699* (MEXU); Balancán, en Rancho El Milagro, San Elpidio, 14 Jul 1984, *G. Ortíz 01*, *07* & *20* (MEXU); **Tamaulipas**: Miquihuana, Servando Canales, 3 km al W de Estanque de los Walle, 1700 m, 5 May 1986, *L. Hernández 1944* (MEXU); 12 km de la desviación hacia Villa de Reyes, 22°00'N, 100°54'W, 27 Jul 1991, *I. Rodríguez & R. Lira 63*, *63a* & *63b* (MEXU); **Veracruz**: La Antigua, Pureza, 10 May 1986, *R.M. Baizabal M. & M.G. Zola B. 11*, *12 & 13* (MEXU); San Andrés Tuxtla, Balzapote, 18°36'N, 95°05'W, 60 m, 17 Feb 1995, *A. Díaz Rico ADR-48* (MEXU); Veracruz, 1853, *J.K. Müller 174* (NY); Playa Vicente, cerca de Victoria, 80 m, 11 Apr 1969, *L.I. Nevling & A. Gomez-Pompa 645* (MEXU); Panuco, al sur de la ciudad de Panuco, 20 m, 24 Feb 1987, *L.M. Ortega T. 87* (MEXU); Hidalgotitlan, Brecha Hnos. Cedillo-Agustin Melgar, 150 m, 29 May 1974, *B. Vázquez 673* & 24 Aug 1974, *B. Vázquez 998* (MEXU); Hatillo zona arqueológica, 19º19'12''N, 96º19'48''W, 18 Mar 1986, *P. Zamora C. & M.G. Zola B. 4* (MEXU); Paso de Ovejas, Carretas, 19º18'46''N, 96º24'17''W, 26 Apr 1986, *P. Zamora C. 24* & *25* (MEXU); Tierra Blanca, 2 Apr 1986, *M.G. Zola B. & P. Zamora 1078* (MEXU); El Aguacate, 2 Apr 1986, *M.G. Zola B. & P. Zamora 1081* (MEXU); El Faisan, 4 Apr 1986, *M.G. Zola B. & P. Zamora 1096* (MEXU); La Víbora, 9 Apr 1986, *M.G. Zola B. & P. Zamora 1110* & *1138* (MEXU); Tierra Colorada, 2 May 1986, *M.G. Zola B. & P. Zamora 1243* & *1249* (MEXU); Mata Grande, 40 m, 5 Nov 1985, *M.G. Zola B. & R.M. Baizabal 1620* (MEXU); La Antigua, Pureza, 5 May 1986, *M.G. Zola B.* & *P. Zamora 1261* (MEXU); Paso Moral, 7 May 1985, *M.G. Zola B. & P. Zamora 1277* (MEXU); a 500 m del poblado La Ceiba, carretera La Ceiba-Tierra Blanca, 20 m, 1 Oct 1985, *M.G. Zola B. & R.M. Baizabal M. 1459* & *1467* (MEXU); a 500 m del poblado Guayabal, 40 m, 11 Oct 1986, *M.G. Zola B. & R.M. Baizabal 1496, 1500, 1503* & *1506* (MEXU); Guayabal, 15 Oct 1986, *M.G. Zola B. & R.M. Baizabal 1511* (MEXU); La Antigua, Pureza, 20 Oct 1985, *M.G. Zola B. & R.M. Baizabal 1514, 1548* & *1550* (MEXU); Tamarindo, 20 m, 25 Oct 1985, *M.G. Zola B. & R.M. Baizabal 1599* (MEXU); **Yucatán**: Valladolid, Xkax, 2 km al oriente de Xocén, 11 Apr 1993, *O. May 39* (MEXU); Tinum, Chichen Itzá, 25 Jun 1932, *W.C. Steere 1579* (F, MEXU, MICH); Dzitás, 20°50'22''N, 88°31'34''W, 22 m, 13 Sep 1984, *E. Ucan Ek et al. 3529* (MEXU); **Zacatecas**: col. la Rivera, 29 Aug 1996, 2385 m, *S. Benítez 724*, *732* & *734* (MEXU); Fresnillo Zacatecas, Santa Anita, 2385 m, 29 Aug 1996, 2385 m, *S. Benítez 726, 735, 738* & *744* (MEXU); Oxkutzcab, 6 km de Oxkutzcab, camino a Rancho San José, 19 Apr 1988, *P. Simá 606* & *610* (MEXU); Yaxcaba, Tixcacaltuyub, 24 m, 27 Jun 1980, *C. Vargas 66* (MEXU).

NICARAGUA. Chontales: ca. 0.8 km above (N of) Cuapa, 340 m, 22 Jan 1978, *W.D. Douglas 6154* & *6155* (MEXU); ca. 0.8 km north of Cuapa, ca. 12º16'N, 85º23'W, 340 m, 22 Jan 1978, *J.J. Pipoly 1602* (CAS); **León**: La Paz Centro, 4 km SW del pueblo, 12°18'N, 86°42'W, 60-80 m, 12 Sep 1980, *M. Guzmán et al. 1011* (MEXU); Isla Momotombito, ca. 12°21'N, 86°28'W, 40-300 m, *W.D. Stevens & M. Araquistain 13242* (MEXU); **Managua**: Volcán de Chiltepe, 12°14'N, 86°18'W, 100-200 m, 2 Jun 1981, *P. Moreno & J. Henrich 8954* (MEXU); **Nueva Segovia**: Ocotal, bosque en galería en Río Dipilto, 13°37'N, 86°27.5'W, 15 May 1982, *J.C. Sandino 2975* (MEXU); **Rivas**: Isla Ometepe, Volcán

Maderas, 11°27'N, 85°32'W, 200-500 m, 18 Jun 1984, *W. Robleto 902* & *907* (MEXU); **Zelaya**: Monkey Point, a 1 km al S sobre la playa, 11°35'N, 83°39'W, 0-5 m, 22 Oct 1981, *P.P. Moreno & J.C. Sandino 12153* (MEXU).

PANAMÁ. Canal Zone: Colón, Gamboa, 26 Dec 1923, *P.C. Standley 28523* (US); Río Paraíso above East Paraíso, 7 Jan 1924, *P.C. Standley 29880* (US); Fijoles, 17 Jan 1924, *P.C. Standley 31479* (US); **Panamá**: Chepo, Piriá, medicinal plants of the Bayano Cuna, 150 m, 24 Sep 1967, *J.A. Duke 14410* (US); en el Llano de Chepo, 16 Dec 1972, *B. de Escobar & G. Barrera 281* (F).

PARAGUAY. Alto Paraguay: Palmar Chicas, 21º45'S, 57º56'W, 28 Jun 1977, *A. Krapovickas & A. Schinini 32764* (CTES); **Presidente Hayes**: Puerto Falcón, 5 Mar 2006, *A.I. Honfi et al. 1286* (CORD).

PUERTO RICO. Mayagüez: ad Boquillas, 17 Oct 1885, *P. Sintenis 865* (LE).

PERU. **Amazonas**: Condorcanqui, Rio Cenepa, 700 m, 3 May 1973, *E. Ancuash 297* (USM); Huampami, Río Cenepa, 650 m, 14 Jul 1974, *B. Berlín 1572* (MO); **Cajamarca**: San Ignacio, Huarango, Mechinal, 5º19'S, 78º43'W, 600 m, 2 Feb 1996, *J. Campos de la Cruz & O. Diaz 2333* (USM); **Cuzco**: Convención, Shimaa, Río Kompiroshiato, Jul 1973, *A. Johnson 70* (USM); **Huanuco**: Leoncio Prado, Tingo María, Fundos Univ. Agraria, 670 m, 19 Jan 1968, *S. Cruz 003, 004, 005, 007, 009* & *011* (USM); **Ica**: Chincha, Tambo de Mora, 5 m, 30 Jun 1984, *A. Cano 374* (USM); **Junin**: Tarma, Satipo, Aug 1940, *C.A. Ridoutt 11714* (USM); **La Libertad**: Trujillo, en terreno de jardín, 45 m, Oct 1971, *A. López M. 7873* (HUT, NY); **Lambayeque**: Chiclayo, Reque, 30 m, 21 Mar 1998, *S. Llatas Quiróz 4300* (HAO†, USM); Reque, 25 m, 16 Jun 2002, *S. Llatas Quiroz 4650* (USM); **Lima**: Lima, La Molina, 300 m, 30 Jun 1954, *O. Velarde Nuñez 7, 19* (CORD); La Molina, 300 m, 5 Jun 1954, *O. Velarde Nuñez 18, 20* (CORD); La Molina, 300 m, 13 Feb 1954, *O. Velarde Nuñez 22* (CORD); La Molina, 300 m, 12 May 1954, *O. Velarde Nuñez 26* (CORD); Canta, Collo, 20 Aug 1972, 2500 m, *G. Vilcapoma S. 84* (USM); Lycahuasy, 2700 m, 20 Aug 1972, *Vilcapoma S. 85* (USM); **Loreto**: Alto Amazonas, Yurimaguas, chacra near River Shanusi, about 3 km S of the experimental station of San Ramon, 18 Sep 1984, *K. Hormia 2228* (USM); Loreto, Nueva Jerusalem and vicinity, Rio Macusari, 220-300 m, 2º55'S, 76º15'W, 10-11 Jun 1986, *W.H. Lewis et al. 10922* (USM); Maynas, Negro Urco, comunidad Huitota, 26 Mar 1987, *P. Pinto E. et al. 6185* (COL); Rio Amazonas, Isla Rondiña, opposite Leticia, 18 Mar 1977, *T. Plowman et al. 6391* (USM); San Martín: Prov. Mariscal Cáceres, Juanjui, 350-400m, 7 Mar 1998, *J. Schunke V. 14492* (NY).

UNITED STATES OF AMERICA. **California**: Santa Barbara County, Santa Barbara, Fox Canyon, 25 Aug 1956, *H.M. Pollard s.n.* (CAS 411882); cultivated organically by Community Ecological Center, Micheltorena & Garden Sts., 15 Sep 1972, *H.M. Pollard s.n.* (CAS 566263); same locality, El Mirasol Block, 1 Oct 1972, *H.M. Pollard s.n.* (CAS 566002); Ventura County, Ventura, from an abandoned garden spot on Front Street just north of the Southern Pacific R.r. depot, 18 Aug 1970, *H.M. Pollard s.n.* (CAS 556600); **Florida**: Hillsborough County, Tampa, Jul 1970, *D. Burch & N. Chevalier 3709* & *3716* (CAS); Sarasota County, Venice Gardens, 15 Jul 1970, *D. Burch & R.J. Machwart 3714* (CAS); Pinellas County, 31 Garden Way, Clearwater Beach, 6 Jul 1970, *D. Burch & L.*

Schmoll 6838 (MO); Pasco County, 412 7th Street, Zephyrhills, 19 Jun 1971, *D. Burch & M. Bialek 6839* (MO); Manatee County, Bradenton, 3 Jun 1971, *D. Burch & T. Doughty 6840* (MO); **Illinois**: Cook County, Chicago, cult. garden at 831 West Buckingham St. (original seed from the Cubeo Indians, Mitu, Vaupes, Colombia), 22 Oct 1982, *T. Plowman 11896* (COL); **New Mexico**: Experiment Station Farm at Mesilla Park, 1 Nov 1907, *E.O. Wooton 48* & *49* (CAS, LE).

VENEZUELA. Mérida: Mérida, Timotes, 2000 m, 23 Jan 1928, *H. Pittier 12709* (NY); **Portuguesa**: Guanare, caserío La Montaña, 4 km al NW de Córdoba, 10º24'N, 69º52'W, 1000 m, cult., 11 Dec 1986, *G. Aymard C. 5108* (NY).

1b. *Capsicum annuum* L. var. *glabriusculum* (Dunal) Heiser & Pickersgill

Amérique, *T.N. Baudin s.n.* (Herb. Jussieu) (P 00409839)

ANTIGUA. Antigua: Macarthy Valley, 12 Sep 1937, *H.E. Box 1058* (MO, US).

BAHAMAS. **Andros Is.**: South Andros Distr., Congo Town, Mars Bay, 2 May 1979, *R.P. Sauleda & D.S. Correll 2416* (USF); Coppice, 20-22 Jan 1910, *J.K. Small & J.J. Carter 8553* (NY, P, US); along Scott/Thompson Rd, east towards Stafford Creek Settlement from Queen's Highway, 20 May 2002, *M.A. Vincent et al. 10583* (NY); **Berry Is.**: near bridge to Bullocks Harbour, Great Harbour Cay, 17 Oct 1974, *D.S. Correll 43702* (NY); **Bimini Is.**: near center of Cat Cay, 10 Jul 1975, *D.S. Correll 45621* (NY); south Bimini, May 1948, *R.A. Howard & E.S. Howard 10055* (NY, S, US); **Cat Is.**: along a side road/trail that runs south of the long unpaved road to Greenwood Beach Resort, 24°10'93''N, 75°19'04''W, 10 Jun 1999, *L.R. Richey 99-423* (NY); **Crooked Is.**: along road between Cabbage Hill and Church Groove, 4 Jun 1977, *D.S. Correll 48675* (NY); **Eleuthera Is.**: Governer's Harbor, 19 Feb 1907, *N.L. Britton & C.F. Millspaugh 5450* (NY); North Eleuthera, west of Boiling Hole, 9 Jan 1974, *D.S. Correll 41078* (MO, NY); Eleuthera, about 1 mile south of John Millars, 15 May 1975, *D.S. Correll 45127* (NY); The Current, vicinity of the beach, 27 Dec 1968, *W.H.Lewis 7187* (MO, NY); The Current, 21 Dec 1969, *W.H.Lewis 7407* (MO); **Exuma Is.:** Great Exuma, 22-28 Feb 1905, *N.L. Britton & C.F. Millspaugh 3014* (F, NY); **Inagua Is.**: Miner's Tent to Balsam, 22 Oct 1904, *G.V. Nash 1287* (NY); **Long Island:** Clarence Town and vicinity, 16 Mar 1907, *N.L. Britton & C.F. Millspaugh 6234* (NY); south of Mangrove Bush, 23°08'00''N, 75°03'44''W, 19 Dec 1998, *L.R. Richey 98-355* (NY); **Mayaguana Is.**: Mariguana Island, Abraham Hill and vicinity, 7 Dec 1907, *P. Wilson 7512* (NY); **New Providence Is.**: along path near Winton, 9 Sep 1904, *N.L. Britton & L.J. Brace 660* (NY); edge of coppice along new road back of Northeast Point, 23 Mar 1974, *D.S. Correll et al. 41913* (MO); abandoned estate, corner of Village Road and Shirley St., Nassau, 15 Dec 1977, *D.S. Correll 49299* (NY); between Love and Gambier, 17 Febuary 1946, *O. Degener 18764* (NY); Avg Island, 1 Feb 1890, *J.I. Northrop* & *A.R. Northrop 256* (NY); near jct. of Western Rd. & Windsor Field R., 25º03.2'N, 77º30.2'W, 13 Jul 1960, *G.L. Webster et al. 10450* (US); top of Blue Hills ridge, 25°03.5'N, 77°23'W, 2 Aug 1960, *G.L. Webster et al. 10791* (S).

BARBADOS. Belle Gully, *Anonymous s.n.* (F, US); Barbados, 8 Apr 1937, *H. Stehlé 2951* (NY).

BELIZE. **Belize**: Gracie Rock, 1.5 mi. South of mile 22 on Western Highway, 4-5 Jun 1973, *T.B. Croat 23892* (WAG); **Cayo**: Chiquibul National Forest, Macal River, 1 May 2003, *L. Urban 308* (MO); Chiquibul Forest Reserve, track to Monkey Tail Branch, 500 m, 11 Apr 1998, *C. Whitefoord 10238* (MEXU); **Toledo**: Forest Home, 200 ft, 8 Dec 1932, *W.A. Schipp 1076* (GH, MO, UC, S, Z); BFREE Reserve, 45-75 m, 21 Oct 2005, *C. Whitefoord & V. Quiroz 10699* (MO).

BOLIVIA. **La Paz**: Nor Yungas, ca. 4 km SW of Yolosa, 1300 m, 14 Jan 1984, *A.H. Gentry 44206* (MO); **Pando**: N. Suárez, de Cobija unos 49 km hacia el oeste, Hacienda San Juan, 5 Oct 1989, *S. Beck et al. 19150* (LPB).

BRAZIL. Acre: Brasiléia, 9 km da ponte de ferro da cidade de Brasiléia, 10º50’S, 69º33’W, 290 m, cult., 14 Oct 1990, *L. Bianchetti & G. Pereira Silva 978* (CEN); Rio Branco, Rio Branco, Aug 1913, *J.G. Kuhlmann 722* (CORD, RB 3431); **Amapá**: Macapá, APA da Fazendinha, 26-28 May 2009, *L. Espindola-Nascimento & L.S. Simoa 01, 02, 03, 07* & *08* (HAMAB, RB); APA do Rio Curiaú, Comunidade Curiaú de Dentro, 2-5 May 2008, *L.A. Pereira et al. 1701* (HAMAB) & *1747* (HAMAB, RB); Comunidade Curiaú de Fora, 7 May 2008, *L.A. Pereira et al. 1780* (HAMAB, RB); Comunidade Curralinho, 7 May 2008, *L.A. Pereira et al. 1807* (CEPEC, HAMAB, HB, MBML, RB); distrito de Fazendinha, Minipólo Hortifruti da Fazendinha, 5 Jul 2008, *L.A. Pereira et al. 1822* (BHCB, CEN, HAMAB, RB); Santana, Ilha de Santana, 12 Jul 2008, *L.A. Pereira et al. 1840, 1841 & 1842* (HAMAB, HB, RB); Comunidade Curiaú de Fora, 14 Jul 2008, *L.A. Pereira & W.M.S. Severino 1853* (CEPEC, HAMAB, HB, MBML, RB); Ilha de Santana, 19 Jul 2008, *L.A. Pereira et al. 1867* (HAMAB, HB, RB); Minipolo Hortifruti da Fazendinha, 8 Nov 2008, *L.A. Pereira et al. 1900 & 1903* (BHCB, HAMAB, RB); **Bahía**. Bahía, *J.B.L.T. Luschnath de la Tour 3098* (P); **Maranhão**: Bom Jardim, along Rio Pindaré, ca. 15 km W of Santa Inês, 3º30’S, 45º30’W, 2 Sep 1983, *M.J. Balick et al. 1539* (CEN, RB, SP); Porto Franco, Rod. Belem-Brasilia, 28 Mar 1976, *G. Hatschbach & R. Kummrow 38441* (CORD); **Matto Grosso:** Castanheira, beira da estrada Juina-Juruena, 10°57’S, 58°44’W, 8 Jul 1997, *V.C. Souza et al. 18426* (RB); **Minas Gerais:** Monte Belo, Fazenda Lagoa, 8 Jan 1981, *M.C. Weyland Vieira 117* (RB); **Pará**: Almeirim, Estação Ecológica do Jari, miners camp, 0º75’S, 52º30’W, 13 Oct 1987, *H.T. Beck et al. 87* (INPA, NY); Rod. Belém-Brasilia, 21 Aug 1959, *M. Kuhlmann & S. Jimbo 80* (CORD, SP); Senador J. Porfirio, Basin of Rio Xingu, Gleba Bacaja, lote 88, just below mouth of Rio Bacaja, Genipapo trail, 3º22’20’’S, 50º47’50’’W, 27 Nov 1980, *G.T. Prance et al. 26505* (CORD, MO); Canaã dos Carajas, 6º27’47.86’’S, 50º19’27.01’’W, 9 Dec 2012, *I.M.C. Rodrigues et al. 570* (BHCB, NY); **Rondonia**: 21 km SE of Ariquemes on Hwy BR 364, then 1 km E on “Line 45”, 10º07’S, 62º56’W, 200 m, 17 Mar 1987, *M. Nee 34412* (GH, HFSL, INPA, MO, NY, SP, US); **Roraima**: Mun. Cantá, Dormida, foothills of Serra da Lua, 2º25-29’N, 60º11-14’W, 15 Jan 1969, *G.T. Prance et al. 9295* (F, INPA, NY, U, US). **Tocantis**: Mun. Filadelfia, Lagoa da fazenda Sítio Novo, mata ciliar do rio Tocantis, 7°19’03’’S, 47°39’04’’W, 150 m, 17 Jan 2008, *G. Pereira-Silva & G.A. Moreira 12751* (CEN).

BRITISH VIRGIN ISLANDS. George Dog Island, 9 Jul 1988, *G.R. Proctor 44888* (NY).

CENTRAL AMERICA. América Central, *H.R. Wullschlaegel 396* (W).

COLOMBIA. **Amazonas**: Puerto Santander, Meta, Chagra de R. Matapí, 100-200 m, 8 Nov 2000, *S. Castro & R. Matapí 523* (COL); Puerto Santander, Aduche, Chagra de I. Macuna, 100-200 m, 24 Nov 2000, *S. Castro & N. Andoke 607* (COL, HUAZ); cultivated in dooryard garden in Puerto Nariño, Río Loreto-Yacu, 2 Sep 1972, *L.L. Glenboski C-51* (COL); Igará Paraná, Remolinos, Chagra D. Padd, 12 Jan 1988, *C.I. Henao & D. Padd 168* (COL); Igará Paraná, Milán, Chagra L. Buraiño, 13 Jan 1988, *C.I. Henao & L. Buraiño 169* (COL); same locality, 28 Feb 1987, *C.I. Henao 170* (COAH, COL); Igará Paraná, Santa María, Chagra O. Zɨueche, 13 Nov 1987, *C.I. Henao & Zɨueche 247* (COL); Igará Paraná, Chagra A. Kuiru, 26 Oct 1987, *C.I. Orozco & R. Kuiru 173* (COL); Puerto Narino at mouth of Rio Loretoyacu, c. 100 m, cult., 8 May 1972, *T. Plowman 3223* (GH); **Antioquia**: Santa Fe de Antioquia, Finca Cotové, 700 m, 21 Mar 1974, *J. Aguirre B. s.n.* (MEDEL 16331); Medellín, 1500 m, 7 Jun 1930, *W.A. Archer 93* (US); en los alrededores de Antioquia, 550 m, 25 Oct 1947, *F.A. Barkley et al. 17C405* (CORD, COL, MEDEL); en los alrededores de Dabeiba, 1350 m, 20 Dec 1947, *F.A. Barkley & G. Gutiérrez V. 1776* (COL, CORD, MEDEL, S, US); Hacienda Montecristo, 25 km después de Apartadó, 40 m, 2 Oct 1986, *J. Betancur et al. 352* (COL, MO); Turbo, carretera Tapón del Darién, 10-20 m, 26 Nov 1983, *J. Brand & M. González 604* (COL, JAUM, MO); zona Cauchera de Villa Arteaga, 21 Dec 1952, *I. Cabrera 36* (COL); Fredonia, Vereda El Vainillo, finca Mango chiquito, 5º54'19''N, 75º38'43''W, 1350 m, 26 Jan 2010, *H. David et al. 3119* (MEDEL); Medellín, 1560 m, Jun 1940, *A. Henao 4373* (MEDEL); alrededores de Medellín, 1560 m, 10 Jul 1947, *W.H. Hodge 6882* (MEDEL, US); Río Medellín, cerca de la escuela, 7 Jun 1930, *E. Killup 93* (MEDEL); Montebello, Vereda La Merced, 56º25'5''N, 75º32'6''W, 1250 m, 22-25 May 1989, *E. Muñoz C. 300* (COL, MO, NY); Boca Regla, along Río Magdalena, 125 m, 28 Sep 1922, *F.W. Pennell 10989* (GH); Isaza, 11 Apr 1951, *R. Romero-Castañeda 2364* (MEDEL); San Luis Cocorná, 3 km S de confluencia Río Cocorná con el Magdalena, 19 Nov 1963, *R. Romero Castañeda 10067* (COL); a 1 km al oriente del puente de Occidente, 700 m, 26 mar 1949, *H. Sánchez et al. 579* (CORD); Medellín, Jul 1934, *E. Santa María 733* (MEDEL); a 1 km al oriente del Puente de Occidente, 700 m, 26 Mar 1949, *R. Scolnik & J. Correa V. 458* (CORD, MEDEL); 3 km al W de Santa Fe de Antioquia, 700 m, 26 Mar 1949, *R. Scolnik et al. 19An329* (CORD, MEDEL); ca. 15 km NE of Necoclí on road to Arboletes, 8º31'N, 76º43'W, 20 m, 24 Mar 1987, *J. Zarucchi et al. 4978* (COL, MO); along road Santa Fé de Antioquia-Anza, ca. 1 km W of crossing of Río Tonuzco at its confluence with Río Cauca, near Finca Cotové, 6º31'N, 75º50'W, 615 m, 19 Sep 1987, *J. Zarucchi et al. 5478* (COL); **Atlántico**: near Barranquilla, 9 Oct 1933, *A. Dugand 478* (F); Barranquilla and vicinity, Jul 1927, *Bro. Elías 251* (S, U); same locality, *Bro. Elías 570* (US); Barranquilla and vicinity, Jul 1936, *Bro. Elías 1461* (F, M, US); Piojó, Carretera hacia el cerro de La Vieja, 10º45'N, 75º07'W, 450-480 m, 7 Oct 2001, *J.C. Marrugo G. 296* (MEDEL); **Bolivar**: Isla de Barú, entre Santa Ana y Playa Mojana, 5-20 m, 25 Aug 1986, *H. Cuadros V. & A.H. Gentry 3060* (COL, MEXU, MO); Cartagena, Isla Tierrabomba, 10º20'N, 75º32'W, 0-20 m, 4 Sep 1989, *H. Cuadros V. 4344* (MO, US); Isla Baru, 21 Sep 1989, *H. Cuadros V. 4475* (MO); vicinity of Estrella, Caño Papayal, lands of Loba, Apr-May 1916, *H.M. Curran 318* (GH, S, US); Turbaco, Fundación J. Botánico G. Piñeres, 28 Aug 1980, *J. Espina 577* (COL); Cartagena de Indias, Corregimiento Arroyo Piedras, 10 Sep 1980, *J. Espina 609* (COL); Santa Catalina,

Hacienda El Ceibal, 10º36’24’’N, 75º17’42’’W, 34 m, 10 Jul 2000, *G.M. Rodríguez-M & L. Olivares 272* (COL); Torrecilla, near Turbaco, 150-300 m, 7-19 Nov 1926, *E.P. Killip & A.C. Smith 14252* (COL, GH, US); north of Arjona, 30-50 m, 15 Nov 1926, *E.P. Killip & A.C. Smith 14534 (*GH, NY, US*)*; Isla de Tierra Bomba, 10º21’36’’N, 75º34’11’’W, 100 m, Sep 1996, *H. Mendoza 1800* (FMB); San Martín de Loba, Corr. La Riboma, Reserva El Garcero, 5-10 m, 9º00’N, 74º05’W, 12 Jul 1992, *F.J. Roldán & C. Cuartas 1853* (NY); entre Sincelejo y Colosó, 11 Dec 1962, *R. Romero Castañeda 9255* (COL); Carthagena, 1866, *J. Triana s.n.* (G); **Boyacá**: Puerto Boyacá, 4 Oct 1975, *M. Avellaneda 12* (COL); Tipacoque, Vereda El Pozo, 1300 m, 12 Nov 1992, *A. Etter & L.A. Villa 1016* (COL); **Caldas**: Resguardo Cañamomo Lomaprieta, en jardines y cultivos, 5º25’N, 75º40’W, 1500 m, 19 Nov 2003, *S. Galán & K. Cárdenas 12* (FMB); **Caquetá**: Mun. Florencia, Barrio El Porvenir, campus universitario, 1°370238N, 75°36’383’’W, 285 m, 12 Mar 2004, *M. Correa et al. 3982* (HUAZ); Vereda Sebastopol, finca El Limonal, 30 Aug 2009, *M. Morales et al. 015* (HUAZ); **Cauca**: km 132 carretera Panamericana, Patía, 500 m, 30 May 1984, *O. de Benavides 4878* (PSO); Popayán, 22 Dec 1993, *I. Cabrera R. 22165* (PSO); NE of El Bordo, Hacienda El Trigal, basin of Río Guachicono, 800 m, 30 Jan 1976, *T. Plowman & D. Vaughan 5366-A* (COL, F, GH), around Cali, W side of Cauca valley, 1000-1200 m, Dec 1905, *H. Pittier 614* (US); **César**: Cgt. La Victoria de San Isidro, Vda. El Zumbador, 990-1400 m, 15 Mar 1996, *J.L. Fernández Alonso et al. 13640* (COL); carretera de Bucaramanga a Aguachica, entre San Alberto y Miriás, 200 m, 19 Jul 1974, *H. García Barriga 20563* (COL); **Chocó**: Vuelta Cortada (Vigía del Fuerte-Antioquia), 100-300 m, 28 Nov 1996, *R. Fonnegra G. et al. 6243* (MO); Riosucio, Parque Natural Nal, Los Katyos, por el Río Tendal, 60 m, 3-7 Jul 1976, *H.A. León 178 & 278* (COL, MO); Parque Natural Nal, Los Katyos, Santatá hacia Salto Tendal, 50-130 m, 29 Feb 1976, *H.A. León 591* (COL, MO); Río Tolo, región de Guayabal, al SE de Acaudí, 0-50 m, 28 Mar 1974, *M.T. Ordóñez & H. Valencia 10* (COL); **Cundinamarca**: Sasaima, 4 Feb 1935, *Bro. Apolinar María (Nicholas Seiler) 114* (F); Cachipay, 9 Jul 1978, *C. Corredor & E. Moncada 07* (UDBC); La Esperanza, 1500 m, 18 Apr 1932, *J. Cuatrecasas 3343* (MA); carretera Villeta Sasaima-Río Dulce, Puente de Santa Cruz, 1000 m, 27 Apr 1961, *M. Dumont et al. 43* (G); línea férrea entre Cachipay y Petaluma, 1700 m, 10-15 May 1946, *J.M. Duque Jaramillo 3549* (COL); Nariño, 340 m, 15-16 Feb 1986, *J.L. Fernández Alonso & R. Jaramillo 5268* & 5338 (COL); 15 km NW of Guaduas on road to Honda, 900 m, 5 Mar 1977, *A.H. Gentry et al. 18124* (COL, MO); La Esperanza, Granja cafeteria “E. Soto” y sus alrededores, 1280 m, 20 Oct 1943, *G. Gutierrez V. 414* (COL, CORD, GH, MEDEL); Nilo, Vereda La Esmeralda, Parque recreativo y zoológico Piscilago, 335-425 m, 1 Apr 2004, *C. Mateus Gutierrez 28* (COL); Villeta, ver. Catay, 5 Mar 1978, *G. Nates Parra 214* (COL); Tocaima, Nov 1932, *E. Pérez Arbeláez s.n.* (COL); Nariño, 500 m, Jul 1930, *E. Pérez Arbeláez 443* (COL, US); Tocaima**,** Chucundá, Dec 1932, *E. Pérez Arbeláez 2491* (COL, US); El Guayabal, cerca de Girardot, 400 m, 6 Aug 1939, *E. Pérez Arbeláez & J. Cuatrecasas 6511* (COL, F, US); San Antonio de Tena, margen derecha del Río Bogotá, 1500 m, 16 Mar 1961, *P. Pinto & M. Dumont 563* (COL, GOET); Villeta, quebrada Pune, 900-950 m, 21 Nov 1997, *J. Reyes & L.E. Arévalo 32* (COL); **Guainía**: Anolaima, Vereda La María, Jan 1954, *H.H. Augusto 4847* (MEDEL); Caserío de Karanacoa, en el Río Guainía, 150 m, 12 Oct 1977, *J. Espina et al. 191* & *193* (COL); Anolaima a La Esperanza, línea del ferrocarril a Girardot, 1280-1780 m, 10/12 Feb 1939, *H. García Barriga 7114* (COL). **Guajira**: Reserva Forestal Monte de Oca, Vereda Remedios, camino a Monte Páramo, 700 m, Feb 2009, *A. Ávila et al. 783* (UDBC); PN Natural Serranía de Makuira,

camino entre Mekijanao y El Chorro, 12º10’01.4’’N, 71º19’03.5’’W, 170-230 m, 1 Jan 2005, *J. Betancur et al. 11258* (COL); Alta Guajira, 15 Nov 1980, *P. Bunch et al. 214* (FMB); 3-5 km S of the Maicao-Riohacha road, 19 May 1981, *P. Bunch et al. 685* (FMB); **Huila**: desde Florencia por el camino viejo hacia Guadalupe, 2º0’38.3’’N, 75º45’9.4’’W, 913 m, 21 Aug 2019, *G.E. Barboza et al. 5049* (CORD); Timana, Vereda San Marcos, 1350 m, 16 May 1997, *M.C. Buendia S. 2* (COL); Agrado, Quebrada La Yaguilda, 700 m, Sep 1986, *J.L. Fernández Alonso & G. Morales 6793* (COL); km 190, Bogotá to Neivo, 400 m, 8 Jan 1974, *A.H. Gentry et al. 8976* (COL, MO); carretera a San Marcos, Río Cabrera, 700 m, 28 Sep 1990, *F. Llanos H. & J. Camacho 1827* (COL); entre Neiva y Campoalegre, 500-675 m, 19 Mar 1940, *E. Perez Arbeláez & J. Cuatrecasas 8305* (COL, F, GH); km 9 from Pitalito to San Agustín, 17 Aug 1974, *T. Plowman & E.W. Davis 4164* (COL, GH); Embalse de Betania, carr. Sta. Elena-Letrán, 580 m, 7 Jun 1987, *N. Rojas et al. 13* (COL); cordillera oriental, E of Neiva, Quebrada, 700-1550 m, 31 Jul 1917, *H.H. Rusby & F.W. Pennell 503* (GH); **Magdalena**: Rincón Hondo, 22 Aug 1924, *C. Allen 484* (MO); Hacienda Santa Marta, Globe de Maldonado Abajo y Potosí, 200 m, 29 Oct 1959, *J. Cuatrecasas & R. Romero Castañeda 24894* (COL); Cerrejón, 200 m, 30 Jul 1949, *O. Haught 6559* (COL, US); Santa Marta, PN Natural Tayrona, Ensenada de Neguanje, 21 Sep 1976, *G. Lozano C. & R. Schnetter 2925* (COL); Tucurinca, 28 Feb 1950, *R. Romero Castañeda 2101* (COL, MEDEL); San Pablo, R. Magdalena, 300 m, 1851-1857, *J. Triana s.n.* (P 00409936, W); **Meta**: Mun. Cabuyaro, Río Meta, 235 m, 14 Oct 1938, *J. Cuatrecasas 3614* (US); same locality, Jan 1937, *H. García Barriga 5056* (COL); Serranía de la Macarena, Plaza Bonita, near Río Guejar, 400 m, 17 Nov 1949, *W.R. Philipson et al. 1456* (COL, GH, US); **Nariño**: Consacá, Corregimiento de Bomboná, 1750 m, 16 May 1979, *O. de Benavides 1819* (PSO); Tumaco, km 63 Carretera Tumaco-Pasto, Llorente, 260 m, 6 May 1984, *O. de Benavides 4693* (PSO); Tambo, mina Cajabamba, 1500 m, 3 Apr 1987, *O. de Benavides 7875* (PSO); Mercaderes, 1100 m, 11 Sep 1975, *S. López-Palacios & J.M. Idrobo 3828* (COL, MO); carr. Pasto-Cali, 6-10 Dec 1962, *C. Saravia Toledo & R. Jaramillo M. 1978* (COL); **Norte de Santander**: en el valle del Río Peralonso en los alrededores de Santiago, 120 m, 21 Dec 1948, *J. Araque Molina & F.A. Barkley 18N.S.140* (COL, MEDEL); Taminango, km 77 Panamericana, Pasto-Popayán, 1300 m, 10 Apr 1985, *O. de Benavídez 5389* (PSO); Cúcuta y El Rosario, Feb 1941, *Carvajalino & Díaz 11* (COL); región del Sarare, Hoya del Río Margua, Quebrada del Río Negro, 1300 m, 9 Nov 1941, *J. Cuatrecasas 12926* (COL, US); región de Sarare, la Cabuya, 1300 m, Oct 1941, *J. Cuatrecasas et al. 12062* (COL, GH, F, US); Hoya del Río Chitagá entre Chorro Colorado y Bata, 1300 m, 14 Oct 1941, *J. Cuatrecasas et al. 12236* (COL, GH, US); **Quindio**: La Tebaida, vereda la Argentina, finca Santa Fe, 1250 m, 10 Apr 1993, *C.A. López 83* (COL); Mun. Salento, Vda. El Agrado, finca El Tesoro, 22 Sep 1999, *L.P. Romero et al. C7* (FMB); **Risaralda**: along road between Pueblo Rico and Istmina (Chocó), 14 km NW of Pueblo Rico, 22 Feb 1990, *T.B. Croat 70847* (MO); Hacienda Alejandría, km 6 carretera La Virginia-Cerritos, 10 Feb 1990, *P.A. Silverstone-Sopkin et al. 5884* (NY); **Santander**: Suaita, Vereda El Caucho, 6º09’45.3’’N, 73º24’50.5’’W, 1905 m, cult., 8 Apr 2003, *J. Betancur et al. 10158* (NY); carretera entre Barbosa y Vadorreal, 10 Feb 1989, *S. Cala L. s.n.* (MEDEL 35303); Florida Blanca, sitio El Mortiño, km 18, 1800-2060 m, 21 Jun 2004, *J.C. Granados-Tochoy & J. Garzón 675* (COL); 15 km al este de Puerto Berrío, 250 m, 13 May 1949, *R. Scolnik et al. 19S022* (CORD, MEDEL, US); **Sucre**: from Colosó to Reserva de Primata, 9º30’N, 75º30’W, 300-350 m, 17 Nov 1981, *A.H. Gentry et al. 34774* (COL, MO); **Tolima**: Valle del Río Magdalena, 400 m, 6 Oct 1940, *J. Cuatrecasas 10519* (COL,

F, US); Fresno, Cgt. Aguasclaras, 5º09'N, 75º02'W, 1200 m, 21 Sep 2007, *Estudiantes Herbario MEDEL 976* (MEDEL); Ibagué, terrenos de la Universidad de Tolima, 4º25'39''N, 75º12'50''W, 1160-1180 m, 9 Aug 2013, *A. Fernández & R. Gonto 33761* (NY); Llanos a l'est de Ibague, bassin du Río Magdalena, llanos a l'est de Ibague, 900 m, 28 Oct 1952, *H. Humbert et al. 26957* (COL, CORD, P, SI); Honda, *A. Joseph 28* (US); bei Fresno, 26 May 1961, *W. Schwabe s.n.* (B); **Valle del Cauca**: Río Cauca, 1000 m, 2 Dec 1947, *L.A. Bermúdez & F.A. Barkley 17C889* (MEDEL); Cali-Buenaventura Higway, km 12 from Cali, 13 Feb 1990, *T.B. Croat 70550* (MO); Reserva forestal El Vinculo, 1000 m, 23 May 1978, *H. Cuadros V. 501* (MO); Entre Gorgona y Puerto Cabuyo, 1000 m, 4 Jun 1943, *J. Cuatrecasas 14514* (CORD, F, GH, US); Lobo Guerrero, 610-650 m, 9-10 Sep 1944, *J. Cuatrecasas 17773* (CORD, F, US); Plana del Valle, Cabuyal, 1000 m, 20 Jun 1945, *J. Cuatrecasas 19637* (CORD, F, US); Plana del Valle, cercanías de Zaragoza, 940 m, 14-18 Nov 1946, *J. Cuatrecasas 22800* (CORD, F, US); piedemonte entre La Uribe y Astelia (Bugalagrande-Sevilla), Vuelta de Violín, 1100-1120 m, 10 Apr 1979, *J. Cuatrecasas & H. Cuadros 28926* (US); Cartago, Hacienda Las Silvia, 21 Dec 1946, *J.M. Duque Jaramillo 4083-A* (COL); Murillo, 1000 m, 28 Sep 1938, *E. Dryander 2178* (US); Santiago de Cali, 3 Apr 1935, *H. García Barriga 4344* (COL); Hacienda El Trejo entre Cerrito y Palmira, 1050 m, 5 Jan 1939, *H. García Barriga 6426* (COL, US); W of Lobo Guerrero, 3150'N, 76º45'W, 700 m, 26 Mar 1986, *A.H. Gentry et al. 53586* (COL, MO); below Dagua, 9.1 km at junction with private road to OleoDucto del Pacifico at crossing of Rio Dagua, 800 m, 19 Nov 1963, *P. Hutchison et al. 3129* (COL, F, K, M, MICH, MO, NY, P, UC, US); Dagua Valley, 700-900 m, 11 May 1922, *E.P. Killip 5395* (US); Puerto Caldas, 860-900 m, 31 Aug 1922, *E.P. Killip & T.E. Hazen 11019* (GH); Río Dagua, *F.C. Lehmann 4730* (F, GH, LE, US); Corregimiento de Mulaló, cerca a La Calera, 1000 m, 20 Oct 1985, *J.E. Ramos 440* (MO); Cerrito, hacienda San Gerardo en la Pista, 950 m, 12 Dec 1987, *J.E. Ramos & P. Silverstone-Sopkin 846* (CTES); Hacienda El Guachal, vereda de Caucaseco, ca. 2 km del Puente Paso del comercio, vía Cali-Palmira, 800 m, 11 May 1988, *J.E. Ramos & N. Paz 915* (CTES); sur de Palmira, Vereda El Cofre, 10 Nov 1948, *G. Ramos Núñez et al. 18V.C.031* (MEDEL); entre Dagua y Loboguerrero, 1050 m, 10-12 Nov. 1962, *C. Saravia Toledo 1404* (COL); Cali, Hacienda Limonar, 28 Feb 1994, *P. Silverstone-Sopkin & N. Paz 6614* (CTES, MO); Jamundi, 1000 m, Jan 1945, *K. Sneidern* 4528 (S); Hacienda El Trejo, entre El Cerrito y Palmira, 1050 m, 28 Dec-5 Jan 1939, *J. Soukup 1851* (CORD).

COSTA RICA. Calamanca, 200 m, Mar 1895, *A. Tonduz 9409* (US); **Alajuela**: vicinity of Las Pavas, *P.C. Standley 36044* (GH); Upala, between Canalete and Bijagua, *J.F. Utley & K. Burt-Utley 3934* (MO); **Cartago**: 1.5 km of Turrialba, near I.I.C.A., 500-600 m, 21 Jun 1949, *R.W. Holm & H.H. Iltis 75* (GH); Tecurrique, 1898, *A. Tonduz 12890* (P); Turrialba, near pond, Instituto Interamericana de Ciencias y Agricola, 600 m, 20 Jan 1967, *A.S. Weston et al. 4173* (UC); **Guanacaste**: ca. 15.8 km beyond the main entrance to PN Santa Rosa, 19 Jul 1977, *F. Almeda et al. 3119* (CAS); Parque Nacional Guanacaste, Estación Cacao, 1100, 2 Jun 1990, *U. Chavarría 16* (MO); Bagaces, Z.P. Miravalles, cuenca del Tempisque, sector Caralampio, 1250 m, 8 Mar 2000, *J.L. Cháves 235* (IBN); road to Liberia, 0-200 m, 1972, *J. Dawe 73-0627* (CAS); Península de Santa Elena, road along E side of Sitio Potrero Grande, 10º51'N, 85º46'30''W, 15-20 m, 24 Jun 2004, *M. Grayum 12111* (MEXU, MO); Liberia, siguiendo el camino entre Nueva Zelandia (Quebrada Grande) y Dos Ríos, Upala, San Gabriel, 600 m, 19 May 1989, *G. Herrera Ch. & Grupo*

Estudiantes de Biodiversidad 2924 (MO); Santa Rosa National Park, 1 km left of fork in road to Playa Naranjo, 0-20 m, 21 Jun 1977, *R.L. Liesner 2239* (MO); Santa Rosa National Park, along dried up stream below Nature Trail, 100-250 m, 23 Jan 1978, *R.L. Liesner 4432* (MO); 1 km E of Hacienda Palo Verde, 10°20'40"N, 85°20'28"W, 8 Jul 1976, *J. Solomon 2417* (MO); La Cruz, along road from Interamerican Hwy. to Puerto Mora and Hacienda Santa Elena, 80-100 m, 24 Aug 1979, *W.D. Stevens 13617* (MO); **Puntarenas**: Isla San Lucas, Golfo de Nicoyar, between Playa Tumbabote and Punta Barrigona, 9°57'N, 84°54'W, 0-25 m, 19 Oct 1984, *M. Grayum 4207* (MO, Z); Reserva Biológica Carara, along S side of Río Grande de Tárcoles from Carretera Costanera E to vicinity of Paso Rieles, 20 m, 3 Oct 1987, *M. Grayum & R. Warner 8366* (MO); 10 km SO, on road to Inter- American Hwy, ridge between Río Guacimal and Río Lagarto drainage, 900 m, 21 Jun 1990, *W.A. Haber & W. Zuchowski 9930* (MO); vicinity of Cascajal (25 km SE of Puntarenas), 30-100 m, Jul 1949, *R.W. Holm & H.H. Iltis 297* (G, GH, P, U); Corcovado National Park, between headquarters at Sirena and Pavo, 0-10 m, 5 Jul 1977, *R.L. Liesner & G. Vega 2885* (MO); Cuenca del Tárcoles, 9º46'13''N, 84º37'39''W, 0-20 m, 6 Oct 1997, *A. Rodríguez & L.D. Vargas 2524* (MEXU, MO); Curú, 9°46-48'N, 84°54-58'W, 0-200 m, 11 Aug 1995, *A.C. Sanders et al. 17496* (CAS, MO); Monteverde, 1700 m, 17-18 Apr 1967, *A.S. Weston & J. Weston 4754* (UC); **San José**: bord de la route pris Escazú, 21 Aug 1889, *H. Pittier 1372* (G); ca. 2 km W of Ciudad Colón, 9º55'N, 84º15'W, 600-700 m, 5 Sep 1990, *J. Solomon 19186* (MEXU).

CUBA. Cuba, *M. Guérin s.n.* (P00409987); Cuba, *R. de la Sagra s.n.* (P00409906); in Cuba Orientali, prope, Sep 1859/Jan 1860, *C. Wright 384* (G, GOET, K, LE, NY, P, S, UC); **Artemisa**: ad San Antonio, rio Seco, Feb 1889, *H.F.A. von Eggers 4715* (P, Z); **Camagüey**: Cayo Romano, Monte y Manigua, al Oeste de Caserío de Versalles, 20 Feb 1981, *A. Álvarez et al. s.n.* (HAJB 43631); vicinity of La Gloria, 30 Jan 1909, *J.A. Shafer 169* (NY); **Cienfuegos**: Cieneguitos, 6 Aug 1895, *R. Combs 149* (MO, NY, P, US) & *151* (F); Soledad, Cienfuegos, 3 Aug 1927, *J.G. Jack 5223* (P); **Guantánamo**: Imías, Lomas de Norte de la Chivera, 0-300m, 4 Feb 1976, *A. Areces et al. s.n.* (HAJB 29612); Maisí, Montes Secos, Mesa del Chivo, 10 Feb 1978, *J. Bisse et al. s.n.* (HAJB 36695); **Holguín**: Loma Silla de Gibara, 10 Feb 1971, *A. Areces et al. s.n.* (HAJB 21184); Gibara, Lomas de Cupeicillo, cerca de Gibara, 21 Apr 1987, *I. Arias et al. s.n.* (HAJB 61621); entre Gibara y Playa Caletones, 7 Nov 1971, *J. Bisse et al. s.n.* (HAJB 20893); Loma de San Marcos de Aura, 6 Nov 1971, *J. Bisse et al. s.n.* (HAJB 20691); Río Piloto, Sierra de Nipe, 18 Apr 1940, *J.P. Carabia 3633* (NY); Antilla (ad Nipe Bay), 21 May 1916, *E. Ekman s.n.* (S); **Isla de la Juventud**: camino de Punta Piedra hasta El Rincón Guanal, 5 Nov 1981, *A. Álvarez et al. s.n.* (HAJB 45672); [Isle of Pines] Caleta Cocodrilos [= Cocodilos], 8 Mar 1916, *N.L. Britton et al. 15299* (NY); **La Habana**: Valle del rio Cojímar, ca. desembocadura, 8 m, 15 Aug 1990, *A.E. Areces-Mallea 2233* (NY); Caimito, Sierra de Ana Fe, 13 Apr 1975, *J. Bisse et Meyer s.n.* (HAJB 28079); Canasí, lomas al este de Boca de Canasí, 100 m, 12 Apr 1980, *J. Bisse et al. s.n.* (HAJB 42116); Jardín Botánico Instituto, 1 Apr 1910, *F. García Cañizares 127* (HAJB); Santa Cruz del Norte, Puerto Escondido, 1 Mar 1992, *J. Gutiérrez s.n.* (NY 01284075); on hills west of Guineo, 10 Nov 1904, *H.A. van Hermann 164* (F, NY, P); Santiago de las Vegas, 3 Apr 1905, *H.A. van Hermann 682* (F, NY, P); Santa Bárbara, 31 Oct 1905, *H.A. van Hermann s.n.* (HAJB 5028); Playa de Cojimar, 16-26 Mar 1956, *A.S. Hitchcock s.n.* (F 235020); near Mariauao, 30 Jan 1909, *Bro. León 626* (NY); Mariel, desembocadura del Río Guajaibón, Sep 1971, *H. Lippold s.n.*

(HAJB 20396); Madruga, 2 Apr 1903, *J.A. Shafer 45* (NY); Santiago de Las Vegas, 6 Aug 1907, *P. Wilson 1019* (HAJB, NY); cerca de la boca del Río Almendras, 9 Sep 1907, *P. Wilson* 1393 (HAJB, US); Rio Almendares to Playa de Marianao, 22 Dec 1910, *P. Wilson 9468* (NY); **Matanzas**: Varaderos, Península de Hicacos, Rincón Francés, 22 Oct 1979, *J. Bisse et al. s.n.* (HAJB 40682); vicinity of Matanzas, Playa, 12 March 1903, *N.L. Britton et al. 112* (NY); near San Miguel, 2 Sep 1903, *N.L. Britton & P. Wilson 256* (NY); Ingenio Capitolio, 19 Apr 1981, *E.H. Day 329* (NY); entre Cárdenas y Varadero, junto al lugar llamado San Lorenzo, 25 m, 20 Jun 1988, *F.J. Fernández Casas & R. Morales Valverde 10659* (MO, NY); Canasí to Boca de Canasí, 18 Feb 1956, *C.V. Morton 10259* (US); **Mayabeque**: near Bejucal, 6 Mar 1905, *A. H. Curtiss 671* (F, HAJB, L, M, MO, NY, P, SI, US); oeste de Güines, 18 Sep 1907, *H.A. van Hermann s.n.* (HAJB 3559); **Oriente**: Antilla, 6-8 Mar 1912, *N.L. Britton et al. 12444* (MO, NY); Caunao River to Cienfuegos Bay, 22 Mar 1926, *J.G. Jack 4376* (US); bordes de camino cerca de Alto de Quemado (entre la Carretera de Alto Songo y la Loma del Gato), 1 Sep 1951, *T.M. López Figueiras 45* (HAJB, US); en la zona entre el Puente de la Mina y la Estación de Radio, carretera de Punta Gorda, bahía de Santiago de Cuba, 9 Mar 1951, *T.M. López Figueiras 223* (HAJB); along Gibara carretera north of Holguin, 18 Apr 1909, *J.A. Shafer 1458* (NY); **Pinar del Río**: Sierra del Rosario, Loma del Salón, 22º45'N, 83º10'W, 450 m, 6 Jul 1993, *P. Acevedo-Rodríguez et al. 5652* (NY, US); Sandino, Península de Guanaha Cubibes, camino entre La Jaula y La Bajada, 16 Nov 1976, *A. Areces et al. s.n.* (HAJB 33137); Sandino, camino al Cabo San Antonio, entre La Bajada y la Iguana, 17 Nov 1976, *A. Areces et al. s.n.* (HAJB 33247); Corrientes Bay, 10-12 Mar 1911, *N.L. Britton & J.F. Cowell 9965* (NY); San Antonio, 20 Mar 1956, *A.S. Hitchcock s.n.* (F 229926); **Sancti Spiritus**: Río Guaurabo (Río Negro), camino de Trinidad al Cubano, 21º50'N, 79º50'W, 1 Jul 1993, *P. Acevedo-Rodríguez et al. 5554* (NY, US); Trinidad Mountains, San Blas-Buenos Aires, Aug 1941, *R.A. Howard 6558* (S); Loma del Charcon, Banao Mts (Sta Clara), 23 Jul 1918, *Bro. León 7855* (NY); **Villa Clara**: 12 Km S of Manicaragua, 1 Jul 1953, *R.L. Dressler 1276* (LE, MEXU, MO); vicinity of Soledad, Jun 1941, *R.A. Howard 4867* (P, NY, US); Santa Clara, Feb 1920, *A. Luna 356* (NY); vicinity of Soledad, 5 Mar 1926, *W.R. Singleton 195* (CORD, L); same locality, 17 Apr 1926, *W.R. Singleton 611* (L).

CURAZAO. Christoffelberg, 26 Dec 1952, *A.L. Stoffers 1186* (NY).

DOMINICA. Saint Mark: Soufrière, 1903, *C.G. Lloyd 435* (NY); Petit Coulibir, 2 May 1992, *C. Whitefoord 7246* (MEXU); **Saint Peter**: Syndicate Estate, 29 Mar 1987, *C. Whitefoord 5629* (MEXU); **Saint Joseph**: NE of Clarke Hall Estate, Layou River Valley, ca. 400 ft, 9 May 1964, *W.R. Ernst 1264* (MO, US).

DOMINICAN REPUBLIC. **Azua**: Sierra Martín García, por el km. 28 de Azua a Barahona, 200 m, 13 Mar 1980, *M.M. Mejía 183* (MO, NY); Loma Cerro de la Vigía, ca. 4-5 km oeste de Barrero, 18º19'N, 70º56'W, 250 m, 14 Nov 1986, *J. Pimentel & R. García 390* (NY); Sierra Martin Garcia, eastern foothills, 5 km WNW of Barrero, 27 Jul 1992, *S.A. Thompson et al. 10775* (NY); Arroyo La Vaca, cerca de Sabana de Miguel Martín, 18º38'N, 70º43'W, 1400-1500 m, 28 Jul 1982, *T. Zanoni et al. 22115* (NY); en camino de Barreras a "La Cueva", 18º20'N, 70º56'W, 180 m, 14 Nov 1984, *T. Zanoni et al. 32161* (NY); **Bayaguana**: El Platano, 150 m, 27 Dec 1972, *A.H. Liogier & P. Liogier 18713* (NY); **Distrito Nacional**: vicinity of Ciudad Trujillo, caves W of Howard Allard's home, 1 mi W

of center of city, 0-25 m, 21 Jan 1946, *H.A. Allard 14787* (US); city of Santo Domingo, km 5 carretera Mella a Cancino Adentro, márgenes del Río Ozama, 30 m, 17 Feb 1981, *M.M. Mejía & C. Ramírez 11039* (MO, NY); Loma Mariana Chica, 4 km (por aire) al este de Villa Altagracía, 18º41'N, 70º08'W, 300-800 m, 19 Jun 1984, *T. Zanoni et al. 30601* (NY); **Independencia**: Sierra de Bahoruco, ca. 12 km al S de Duvergé, en Monte Palma, 18°15'N, 71°30'W, 860 m, 24 Mar 1993, *R. García et al. 4464* (B); 4 km S of Los Pinos, Loma de Vientos, 455 m, 24 Jul 1992, *S.A. Thompson et al. 10651* (NY); **La Altagracia**: Reserva del Este, 18º19'58.43''N, 68º48'42.48''W, 0 m, 27 May 2004, *P. Acevedo Rodríguez et al. 14065* (US); dry forest above Río Cumayasa, 20-100 ft, 9 Sep 1983, *S.T. McDaniel 27181* (MO); 15.1 km from Punta Cana on the new road to Otra Banda, 18°34'W, 68°29'W, 120-130 m, 14 May 1980, *M. Mejía & T.A. Zanoni 6351* (MO, NY, S); El Guanito, km 12 de la carretera de Higuey a El Seibo, camino de El Guanito a Hato de Mana, 18º35'N, 68º34'W, 80 m, 19 Feb 1981, *M.M. Mejía & C. Ramirez 11137* (NY); 24 km. SE from Otra Banda on road to Punta Cana, 110-140 m, 29 Mar 1981, *T.A. Zanoni et al. 12012* (MO, NY); Parque Nacional del Este; ca. 1 km. de Guaraguao, 18°19'05"N, 68°47'00"W, 30 m, 6 Oct 1981, *T.A. Zanoni & M. Mejía 17002* (MO, NY); PN del Este, de Las Tres Hermanas hasta ca. 2 km dentro del bosque, 18º15.5'N, 68º46'W, 5-30 m, *T.A. Zanoni & M. Mejía 17077 A* (NY, US); **La Romana**: Farallon de Cumayasa, 50 m, 19-20 May 1973, *A.H. Liogier & P. Liogier 19207* (NY); same locality, 14 Nov 1975, *A.H. Liogier & P. Liogier 24230* (NY); about half-way between Presa Chavón and Bayahibe on new La Romana-Bayahibe raod, 40-60 m, 30 Oct 1980, *M.M. Mejía et al. 8925* (MO, NY); **La Vega**: vicinity of Piedra Blanca, 200-500 m, 18 Jan 1946, *H.A. Allard 14697* (NY, US, S); same locality, 10 Dec 1947, *H.A. Allard 17784* (US); Valle de Constanza, 1170 m, 28 May 1887, *H.F.A. von Eggers 2247* (GOET); at Salto Jimenoa, 25.5 km N of El Rio on road to Jarabacoa, 19°06'N, 70°37'W, 800 m, 23 Jul 1980, *M. Mejía & T. Zanoni 7524* (MO, NY, S); near Guama de Jumunucu, km 27 from San Francisco de Macoris on raod to Bonao, 19°00'N, 70°25'W, 40 m, 28 Jan 1981, *M.M. Mejía et al. 10386* & *10396* (MO); "Joa", área rural, ca. 6 km. por aire al NO de Bayacanes, 200 m, 9 Jun 1989, *T.A. Zanoni et al. 42707* (MO, NY); **Monte Cristi**: Sabaneta, La Ceiba du Mao, 400-500 m, 16 Oct 1930, *E.J. Valeur 476* (CAS, F, MO, UC, US); Peravia: Cañada (Arroyo) La Aguita, 1 km oeste de San José de Ocoa en la carretera a El Pinar, 1600 m, 8 Jul 1982, *T.A. Zanoni et al. 21507* (MO, NY); **Pedernales**: Sierra de Bahoruco, Aceitillar, Hoyo de Pelempito, 400-600 m, 12 Jun 2007, *T. Clase et al. 4656* (MO); 17-18 km N of Cabo Rojo, 18º04'N, 71º39'W, 330-350 m, 31 Jul 1990, *S.A. Thompson et al. 7626* (NY); **Peravia**: Loma Las Tablas, 18º19'N, 70º25'W, 300 m, 14 Oct 1981, *J. Czerwenka 453* (NY); Arroyo de Parra, between Cerro de Quemada and Loma del Rancho, 18º31'N, 70º28'W, 800m, 6 Sep 1980, *M. Mejía & T. Zanoni 8100* (NY); **Puerto Plata**: Santiago-Puerto Plata road, ca. 3 km N of Benito Martínez, 24 Apr 1970, *D. Burch 2335* (MO, USF); Loma Isabel de Torres, 50 m, 7 Jul 1887, *H.F.A. von Eggers 2796* (GOET); Loma Isabel de Torres, 21-22 Jun 1975, *A.H. Liogier & P. Liogier 23115* (NY); en la ladera Oeste Isabel de Torres, 26 Jul 1975, *A.H. Liogier & P. Liogier 23679* (NY); **Samaná**: 2.3 km. S of Playa El Valle , at road crossing where Arroyo la Majagua joins Rio San Juan, 40-60 m, *M.M. Mejía & T.A. Zanoni 6619* (MO, NY); Las Canitas Mt., 1000 ft. 21 Oct 1909, *N. Taylor 3* (NY); **Santiago**: vicinity of Santiago, top of La Cumbre, near Military Barracks, 11 Jan 1946, *H.A. Allard 14542* (US); Hato del Yague, Valle del Cibao, 27 Nov 1930, *E.L. Ekman 16269* (US, S), Jaiquípicao, ca. 6 km al NO del cruce de la carretera vieja Santiago-San José de las Matas, 19°27'N, 70°54'W, 400 m, 28 Dec 1992, *F. Jiménez et al. 722* (B); San José de las Matas, Mao River

Dajao, 225 m, 23 Nov 1929, *E.J. Valeur 275* (CAS, F, MO, NY, US, S); San José, Magúa, 6 Apr 1933, *E.J. Valeur 964* (MO, US); community of Pedro Garcia, 19º35'50''N, 70º39'10''W, 28 Apr 2006, *I. Vandebroek 356* (NY); Tierra Colorada (near Higueral and LS 302), 21 Dec 1964, *M.G. Whiting 407* (US); 10 km de la Plaza Central de Janico en la carretera a Santiago, 19º23'N, 70º46'W, 320 m, 18 Feb 1983, *T. Zanoni & J. Pimentel 25313* (NY); **San Cristóbal**: Los Hoyos, Carretera hacia la represa, 125 m, 12 Jun 1997, *R. García 6600* (MO); **San Juan**: El Cercado, Juan Santiago, Hondo Valle, 1 Sep 1946, *R.A. Howard & E.S. Howard 8761*; **Santiago Ramírez**: in fruticetis ad Gurabito, 19 May 1887, *H.F.A. von Eggers 1993* (GOET); Santo Domingo, at Rio Ozama, 29 Jan 1929, *E.L. Ekman 11372* (US, S); 1 km from Hernando Alonzo on road to Palmarito; on side of loma El Diviso, 100 m, 28 Jan 1981, *M.M. Mejía et al. 10490* (MO, NY).

ECUADOR. **Esmeralda**: Cantón Quinindé, 136 m, 16 Mar 2011, *B. Freire & M. Ruales 2931* (QAP); **Guayas**: Los Manga, 8 km E of Manglar Calta, base of Cerros de Chongon (really Colonche), 60 m, 27 Jun 1977, *H.H. Iltis & M.G. Iltis 29* (WIS); **Imbabura**: Tercer Paso, on the San Lorenzo R., 0°18'N, 77°59'W, 1100 m, 7 Jun 1978, *M.T. Madison et al. 4967* (AAU, F, QCA); Río Chuchubí, 0°54'20.8"N, 78°30'50.6"W, 457 m, 31 Mar 2012, *C. Quintana et al. 3030* (QCA); **Loja**: La Argelia, 2200 m, 20 Dec 1945, *M. Acosta Solís 11366* (F); Macará, en jardines de casa particular, 4º22'59''S, 79º56'42''W, 507 m, 6 May 2017, *G.E. Barboza & S. Leiva González 4832* (CORD); ca. 7 km W of Catamayo on road to Catacocha, 3°55'S, 79°22'W, 1300 m, 6 Feb 1984, *S. Knapp & J. Mallet 6254* (QCA, QCNE, US); **Los Rios**: Rio Palenque Biological Station, km 56 Quevedo-Santo Domingo, 220 m, 16 Sep 1973, *C.H. Dodson & K. Tan 5319* (CORD, US); **Manabi**: Cerro Montecristi, carr. Manta-Jipijapa, 1°2'S, 80°41'W, 300-600 m, 11-12 Nov 1995, *T. Núñez et al. 336* (MO, QCNE); **Morona-Santiago**: Sevilla Don Bosco, 2°20'S, 78°50'W, 1100 m, 16 Oct 1993, *J. Carvajal 1* (QCA); Sevilla Don Bosco, 1100 m, 28 Nov 1993, *M. Bedoya 1 & 2* (QCA); **Napo**: Río Cuyabeno below inlet of Rio Victoriano Criollo, 0º05'S, 76º10'W, 230 m, cult. near the house, 28 Jul 1983, *H. Balslev & E. Asanza 4363* (QCA); Nuevo Rocafuerte y la orilla del Río Napo y del Río Yasuní, hasta la laguna de Jatuncocha, *E. Alarcón 102* (QCA, MO); confluence of Quiwado and Tiwaeno Rivers, Waorani, 1º50'S, 77º40'W, 18 apr 1981, *E.W. Davis & J. Yost 994* (F, GH, QCA); San José de Payamino, 40 km W of Coca, 0°30'S, 77°20'W, 300-600 m, 9 Apr 1984, *D. Irvine 773* (CHEP, F, QCNE); **Zamora-Chinchipe**: Nangaritza, comunidad Chumpias, 4°16'32''S, 78°43'56''W, 1020 m, 28 May 2001, *F. Santín et al. 100* (LOJA); Río Nangaritza, sector del Río Shamatak, 4°19'13''S, 78°42'07''W, 900 m, 15 Apr 1996, cult. huerta, *V. van den Eynden et al. 700* (LOJA, QCA).

EL SALVADOR. **Ahuachapán**: vicinity of Ahuachapán, 800-1000 m, 9-27 Jan 1922, *P.C. Standley 19872* (GH, LE); **La Unión**: vicinity of La Unión, ca. 150 m, 13-21 Feb 1922, *P.C. Standley 20689* (GH); Laguna de Maquigüe, 60 m, 18 Feb 1922, *P.C. Standley 20903* (GH); Laguna de Olomega, 13°18'N, 88°04'W, 7 Sep 1997, *R. Villacorta & J. Giammattei 2551* (MO); **Morazán**: Montecristo, ca. 15 km NE of San Miguel, 13°36'N, 88°04'W, 140 m, 11 Dec 1941, *J.M. Tucker 511* (CORD, UC, US); **Santa Ana**: entre la Hda. El Milagro y la Hda. San Cayetano, a orilla de la carretera a Metapán, 25 Oct 1993, *J.L. Linares & C.A. Martínez 859* (MEXU); **San Miguel**: Volcan Conchagua, 13°16'35"N, 87°48'50"W, 400 m, 31 Jan 1998, *A.K. Monro et al. 2164* (BM, MO); **San Salvador**: San Salvador, 17 Sep 1922, *S. Calderón 1197* (GH, US); **San Vicente**: Las Galeras, western edge of the

flood plain of the Rio Lempa, between San Vicente and San Miguel, 13°36'09''N, 88°38'40''W, 30 m, 9 Feb 1998, *G. Davidse et al. 37476* (BM, ITIC, LAGU, M, MO); vicinity of San Vicente, 2-11 Mar 1922, *P.C. Standley 21308* (US); **Sonsonate**: Acajutla, Jul 1923, *S. Calderón 1658* (GH, US); vicinity of Armenia, 18 Apr 1922, *P.C. Standley 23453* (GH, US).

FRENCH GUIANA. Iles du Salut, Aug 1854, *P.A. Sagot s.n.*, cult. (P 00409926); Karouany, cult. par les indiens de la Mana (Galibis), Jan 1859, *P.A. Sagot 856* (K, P: 3 sheets, W).

GRENADA. Grenada, [probably 1840], *E. von Friedrichsthal 201* (W); **Saint George**: Lowther Road, Jan 1905, *W.E. Broadway s.n.* (NY 01284145); near Mt. Helicoso, 28 Jul 1905, *W.E. Broadway s.n.* (NY); The Bower, Jan 1905, *W.E. Broadway 4514- Ser. I* (BR, CORD 00088595); near Mt. Itelecon, 28 Jul 1905, *W.E. Broadway s.n.* (US 849021); Grenada, 6 Jul 1906, *W.E. Broadway s.n.* (MO 808886).

GUADELOUPE. In Guadeloupe, *Anonymous 67* (G); Guadeoupe, cult. les jardines, 1892, *A. Duss 149* (P, US); Guadeloupe, 25 Aug 1941, *A. Questel s.n.* (P00409982); Mts. Carbes, Morne Grande Veute, 500 m, 25 Apr 1974, *C. Sastre et al. 2708* (MO); **Basse-Terre**, cult., 1895, *A. Duss 3574* (NY); Gourbeyre, cult., 1896, *A. Duss 3575* (NY); Pigeon, Bouillante, 1895, *A. Duss 3681* (NY); Bouillante, crête de Village, Début du Sentier des Crêtes, 10 Jun 1990, *J.P. Fournet 4616* (P); Petit Bourg, Vernou, La Glacière, 16 Aug 1990, *J.P. Fournet 4666* (P).

GUATEMALA. Alto Verapaz: vicinity of Coban, 1300 m, cult., 23 Mar-19 Apr 1941, *P.C. Standley 90936* (F); **Chimaltenango**: Finca La Esperanza, Pochuta, 23 Nov 1963, *D.M. Porter 1299* (GH); **Chiquimula**: Jucotán, Dispensario Bethania, 14°49'N, 89°23'W, 450 m, 16 Oct 2000, *J. Kufer 99* (M, MEXU, MO); Quebrada Shusho, above Chiquimula, 14 Oct 1940, *P.C. Standley 74361* (F); around Chiquimula, 400 m, 20 Oct 1939, *J. Steyermark 30089* (F); Olopa, Las Palmas, 1400 m, 28 May 1999, *M. Véliz 7079* (MEXU); **Izabal**: valley of Motagua River, 29 Mar 1940, *J. Steyermark 38343* (F); **El Progreso**: en la orilla del Río Motagua, 200-300 m, 8 Mar 2003, *M. García & F. Ramírez 242* (MEXU); Pasasagua, camino a Cobán, 200-300 m, 14º55.552'N, 90º03.532'W, 2 Apr 2003, *F. Ramírez & M. García 526* (MEXU); San Agustín Acasaguastlán, 300 m, cult. jardín, 17 Feb 2003, *M. Véliz & F. Ramírez 12818* (MEXU); **Escuintla***:* Near San José, 0 m, 30-31 Jan 1939, *P.C. Standley 64208* (US); **Huehuetenango**: canyon tributary to Río Trapichillo, between Democracia and canyon of Chamushú, 1000-1100 m, 24 Aug 1942, *J. Steyermark 51277* (GH); between Santa Ana Huista and Rancho Lucas, Sierra de los Cuchumatanes, 800-900 m, 26 Aug 1942, *J. Steyermark 51364* (UC); **Jalapa**: El Rancho, 1000 ft, 1 Jan 1908, *W.A. Kellerman 4854* (US); same locality, 12 Jan 1908, *W.A. Kellerman 7674* (US); **Petén:** La Libertad, Aug-Nov 1933, *M. Aguilar H. 64* (MICH); La Libertad and vicinity, Aug-Nov 1933, *M. Aguilar H. 177* (MEXU, MICH); Tikal, around Aguada Tikal, 29 Jul 1959, *E. Contreras 48* (CAS; MEXU, MO); Tikal National Park, 5 km on Remate road, 17 Oct 1959, *E. Contreras 294* (CAS, MEXU, MO); Tikal National Park, on new Uaxactun road, in ramonal-zapotal, 12 Apr 1968, *E. Contreras 7684* (CAS, MEXU, MO); Tikal, block 4 F, 6 Feb 1959, *C.L. Lundell 15376* (CAS, MEXU, MO); PN Tikal, Ramonal, en el camino para el Remate, 2 Oct 1969, *R.T. Ortíz 341* (MO, US); Vuelta del Escarbador, Sayaxche, 30 Apr 1993, *E. Secaira s.n.* (MEXU 1358260); sitio arqueológico La Joyanca,

100 m, 23 Mar 2000, *M. Véliz 8126* (MEXU); Umgebungdes Westufers des Lago Petén Itzá: Umbebung der Straβe am NNE-Ortsrand von Nuevo San José, 16°59’44’’N, 89°53’ W, ca. 180 m, 25 Aug 1993, *B. Wallnöfer & F.M. Tut-Tesucun 6028* (M, MO); **Quetzaltenango**: Colomba, Costa Cuce, 500 m, 13 Sep 2003, *M. Mérida s.n.* (MEXU, MO 1302019); La Unidad, Costa Cuce, 7 Mar 1920, *L. Rodríguez 461* (P); **Retalhuleu**: Champerico, Nov 1877, *K.G. Bernoulli & O.R. Cario 2344* (GOET); Retaluleu, Mar 1877, *K.G. Bernoulli & O.R. Cario 2378* (GOET, LE); Río Samalá, 1700 m, Oct 1891, *W.C. Shannon 206* (US); vicinity of Las Delicias, 200 m, 22 Feb 1941, *P.C. Standley 88022* (F, US); **Santa Rosa**: Chiapas, May 1892, *E. Heyde & E. Lux 3439* (M, US); near Oratorio, 1200 m, 21 Dec 1938, *P.C. Standley 60647* (US); near El Molino, 26 Nov 1940, *P.C. Standley 78366* (F); **Zacapa/El Progreso**: inter Rio Hondo et Acasaguastlán, 1870, *K.G. Bernoulli & O.R. Cario 2395* (GOET); **Zacapa**: 10 km W of Teculutan, 8 Apr 1970, *W.E. Harmon & J.D. Dwyer 3543* (GH, MO).

GUYANA. **Upper Takutu-Upper Essequibo Region**: Kidekperdana Creek, 8 km SE of Aishalton, 2º25’N, 59º10’W, 200 m, 26 Nov 1993, *T. W. Henkel & R. James 3520* (MO, U, US); Kanuku Mts., Rupununi R., near Witaru Falls, 3º04’N, 59º28’W, 90-120 m, 10 Feb 1985, *M.J. Jansen-Jacobs et al. 119* (NY, US); Konashen area, Essequibo River, 250 m, 25 Sep 1989, *M.J. Jansen-Jacobs et al. 1810 & 1811* (MO); Kanaku Mts., Crabwood Cr., 03°07’N, 59°06’W, 260 m, 31 Jan 1994, *M.J. Jansen-Jacobs et al. 3465* (B, MO, U, US); near Buro-Buro River along footpath from Surama village, 4º09’N, 59º04’W, 65-90 m, 19 Feb 1990, *T. McDowell et al. 1940* (NY); NE from Karasabai to Yourora Creek, 4º03’N, 59º30’W, 100 m, 7 Mar 1990, *T. McDowell et al. 2174* (CTES, MO, US).

HAITI. **Artibonite**: L'Artibonite, about 6 miles north of Atalaye Plantation, vicinity of St. Michel de l'Atalaye, 26 Dec 1925, *E.C. Leonard 8495* (NY); San Michel to Marmelade, 5 Aug 1905, *G.V. Nash & N. Taylor 1456* (NY); between St. Marc and L’Arcahaie, 8 Apr 1925, *G.S. Miller 198* (US); **North West**: vicinity of Port de Paix, 1 mi W of city, 23 Dec 1928, *E.C. Leonard* 11113 (NY, US); **West**: Port au Prince, on the coast, *E. Ekman H 2116* (NY, US, S); Morne à Cabrits, 1500 m, 3 Jul 1927, *W.J.Eyerdam 4* (P); La Fontaine de Marquisant près de Port au Prince, 6 Mar 1827, *V. Jacquemont s.n.* (P 00409976 & 00409977); vicinity of Port au Prince, 1-2 Apr 1920, *E.C. Leonard 3461* (NY); Gonave Island, vicinity of Étroite, 15 Mar 1920, *E.C. Leonard 3318* (NY, US); vicinity of Cabaret, baie des Moustiques, 17 Jan 1929, *E.C. Leonard & G.M. Leonard 12089* (GH, NY, US); Massif de la Selle, en el fondo del valle, cerca de Granier, en el arroyo de Granier, cult., 15 Jul 1983, *T.A. Zanoni & M.M. Mejía 26168* (MO, NY); **Southeast**: vicinity of Bassin Blue, road to Port de Paix, 630-1500 m, 16 Apr 1929, *E.C. Leonard & G.M. Leonard 14735* (US).

HAWAIIAN ISLANDS. **Kauai Island**: Koloa district, Lawai valley, Pacific Tropical Botanical Garden, 60-70 m, 14 Sep 1987, *D.H. Lorence 5560* (MO).

HONDURAS. **Atlantida**: along road between Tocoa ad La Ceiba at Río Sambo, 15º47’N, 86º30’W, 30 m, 9 Feb 1987, *T.B. Croat & D.P. Hannon 64555* (MEXU, MO); vicinity of Tela, 0 m, 14 Dec 1927-15 Mar 1928, *P.C. Standley 53628* (F, GH, US) & *54485* (F, US); La Fragua, 20 m, 7 Feb 1928, *P.C. Standley 55721* (GH, US); **Cortes**: Aldea El Boquerón, 10 km SE de San Pedro Sula, 17 Apr 1984, *Y. Calderón 123* (UNAH, USF); Carretera a

Armenta, 3 km al N de S.P.S., 17 Mar 1984, *D. Velásquez D. 113* (UNAH, USF); **Comayagua**: Comayagua valley, 600 m, 10 Aug 1948, *L.O. Williams & A. Molina R. 14671* (GH); vicinity of San Antonio de Flores, 29 Sep-5 Oct 1951, *L.O. Williams & R.P. Williams 18295* (GH, US); **El Paraíso**: Morocelí, camino a Moroceli-Plan Quebrada, 14º11'44''N, 86º51'5''W, 792 m, 17 Jul 2003, *E. Martínez S. et al. 36224* (MEXU); **Francisco Morazán**: near Sabana Grande, 3200 ft, 13 Sep 1969, *F.A. Barkley & C. Bostirronnois 39498* (GH); Tegucigalpa, alredores de la Col., 950 m, 5 Apr 1986, *G. Enea A. 253* (US); campo abierto de ciudad universitaria, Tega, 19 Oct 1978, *M. Espinal 203* (MEXU, MO); Ojo de Agua, 50 km SE de Tegucigalpa, 850 m, 4 Nov 1989, *S. Fernández 97* (B); near Zamorano, Río Yeguare, 800 m, Jul-Dec 1958, *J.G. Hawkes et al. 2003* (C, S); Tegucigalpa, Colonia Kennedy, 950 m, 29 Aug 1981, *D. Molina 34* (US); Zamorano, along Río de la Orilla, 850 m, 10 Jul 1947, *A. Molina R. 262* (GH, MEXU, UC); along Quebrada Suyapa, near Suyapa, 1100 m, 10 Nov 1947, *A. Molina R. 594* (GH, MEXU); Tegucigalpa, Quebrada la Orejona, detrás de la UNAH, 1000 m, 28 May 1984, *I. Padilla 219* (UNAH, USF); El Zamorano, Aug 1960, *H.W. Pfeifer 1283* (US); alrededores aldea Suyapa, quebrada La Barranca, 25 May 1978, *E. Romero 64* (MEXU, MO); vicinity of Suyapa, 1100-1200 m, Sep-Dec 1948, 1 Aug 1950, *P.C. Standley 26254* (GH); **Ocotepeque**: El Cerro, vicinity of San Antonio, 1300 m, 30 Aug 1968, *A. Molina R. 22452* (GH); **Olancho**: Sitio de Chichicazapa, 6 km S de Juticalpa, 430 m, 21 Jan 1985, *F. Alvarado Flores 116* (USF); Juticalpa, sendero de la comunidad de Pozos Abajo al Cerro El Zapotillal, 880 m, 31 Jan 2001, *Dirección Gral. de Biodiversidad/SERNA 60* (MEXU); Dulce Nombre de Culmí, 1 May 1982, *D. Molina 110* (MEXU); **Santa Bárbara**: La Ensenada Santa Bárbara a orillas del Lago de Yojoa, 600 m, 1 May 1984, *F. Argeñal 130* (MEXU); **Valle**: 3 km W of Amapala, 10 m, 17 aug 1938, *O.B. Horton & J.L. Morrison 8858* (GH, UC); Amapala, Is. de Zacate Grande, 15 m, 21 Oct 1978, *L. Zelaya 239* (GH, MEXU, MO); **Yoro**: Victoria, a orilla del Río Sulacos, 339 m, 21-23 Jan 1981, *C. Nelson et al. 7102* (MEXU, MO); Quebrada Seca, 30 m, Dec 1927, *P.C. Standley 53913* (GH, US); near Progreso, 30 m, 24 Jan 1928, *P.C. Standley 55021* (GH).

JAMAICA. Jamaica, Madeiras and all the Caribes), *H. Sloane s.n.* (BM000589956); **Kingston**: Green Valley, Aug 1893, *W. Harris 5110* (US); prope Rock Fort, 18 Dec 1907, *W. Harris 10051* (MO, NY, P, US, Z); **Manchester**: Mandeville to Spur Tree Hill, 29 Aug 1907, *N.L. Britton 1007* (NY); Mandeville, 15-26 Feb 1910, *S. Brown 93* (NY); **Saint Andrew**: Nr. Gordon Town, 1250 m, 27 Dec 1959, *C.D. Adams 5961* (MO); NE side of Long Mt. on the eastern outskirts of Kingston, 12 Jun 1963, *M.R. Crosby et al. 119* (NY UC); Blue Montains, 15-20 ft, 5 Sep 1893, *W. Harris 5110* (CORD, US); St. Andrew, 19 Oct 1957, *T. Yuncker 17118* (NY, S); **Saint Ann**: between Brownstone and St. Ann's Bay, 27 Mar 1908, *E.G. Britton 2942* (NY); "Limestone Turn Around", Reynolds mining property, 20 Dec 1953, *R.A. Howard & G.R. Proctor 13367* (USF); Reynolds Mine area near Lydford, 21 Sep 1954, *R.A. Howard 14049* (NY, US); Averham Park, NW of Caremont, *W.T. Stearn 588* (MEXU); Ocho Rios, Apr 1983, *R. Wannenmacher s.n.* (W); east of Moneague, 1000 ft, 2 Nov 1957, *T. Yuncker 17253* (NY, S); **Saint Catherine**: Fort Henderson, 2 Mar 1908, *N.L. Britton & A. Hollick 1829* (NY); Great Goat Island, 18 Jul 1906, *W. Harris 9327* (NY); east slope of the Hellshire Hills, 200-400 ft, 6 Dec 1952, *G.R. Proctor 7507* (NY); **Saint Elizabeth**: Balaclava, 4 Mar 1927, *C.R. Orcutt 626* (UC); same locality, 3 May 1927, *C.R. Orcutt 1467* (UC); Giddy Hall, Feb 1936, *T. Sangster s.n.* (MEXU 758253); **Saint James**: Half Moon Bay, 9 Sep 1960, *C.D. Adams 8049* (M); 5

miles ESE of Little River P.O., 28 Mar 1956, *W.T. Stearn 616* (MEXU); **Saint Mary**, about 4 miles east of Ocho Rios, 0-100 ft, 19 Mar 1958, *T.G. Yuncker 18460* (NY).

MARTINIQUE. Le Marin: Morne Gommier, 1880, *A. Duss 349* (US); chemin muletier entre Prêcheur et Grand Rivière, 5 Nov 1991, *J.P. Fournet et al. 4878* (P).

MEXICO. Without locality, *M. Sessé & J.M. Mociño 1546* (MA); **Baja California**: La Paz, Rancho "la Burrera", a 20 km de Todos Santos, 14 Oct 1985, *J.M. Agúndez 221*(MEXU); same locality, Oct 1987, *J.M. Agúndez 633* (MEXU); Pescadero, 20 Sep 1893, *T.S. Brandegee s.n.* (UC); Miraflores, 14 Oct 1890, *T.S. Brandegee 414* (CAS, GH, UC 103970, US); San Bartholome, 23 Oct 1890, *T.S. Brandegee 414* (UC 103971); Sierra de la Giganta, Cañón de Matancita, near foot of Cuesta de Alta Gracia, 25°41'N, 111°19.5'W, 3 Nov 1964, *A. Carter 4902* (B, MEXU, MO, UC); vicinity of Loreto, cult. in huerta, 26°N, 111°21'W, 0 m, 8 Nov 1964, *A. Carter 4921* (UC); Cañada del Encinal, south side of Valle de Los Encinos (south side of cerro Giganta), 26°3.54'N, 111°34'W, 735 m, 1 Oct 1967, *A. Carter & R. Morán 5365* (MEXU, UC); El Cañón, km 25 carr. San José de Magdalena a la Misión de Guadalupe, 26º56'N, 112º22'W, 730 m, 15 Jan 2002, *R. Domínguez Cadena 2606* (MEXU); 3 miles (by road) SW of San Antonio, 1600 ft, 10 Oct 1964, *J.R. Hasting & R.M. Turner 64-229* (CAS); Cuesta 'El Arado' del Rcho. 'La Burrera', 23°32'N, 110°04'W, 3 Sep 1986, *J.L. León de Luz 2019* (UC); La Burrera, a 27 km al E de Todos Santos, 550 m, 14 Oct 1985, *P. Tenorio L. et al. 10513* (MEXU); **Campeche**: a 1k al N de Narciso Mendoza, 18º14'50''N, 89º27'45''W, 240 m, 26 Jun 1997, *D. Álvarez 89* (COL, MEXU); a 1.3 km al W de Plan de San Luis, 18º31'46,4''N, 89º34'42,5 W, 256 m, 26 Jun 2002, *D. Álvarez 1491* (MEXU); a 1 km al W de E. Castellot Nº 2, 18º31'46''N, 89º35'23''W, 243 m, 21 Sep 2002, *D. Álvarez 2075* (MEXU); Calakmul, Zoh-Laguna, 18º35'39''N, 89º24'48''W, 290 m, cult., 19 May 1997, *P. Alvaro M. & D. Álvarez 741* (MEXU); Calakmul, km 6 rumbo a Nuevo Becal, 18º37'5''N, 89º21'10''W, 270 m, 2 Oct 1997, *G. Bacab W. 115* (MEXU); 3 km al S de Bolochen de Rjón, cerca de las Grutas de Xtacumbilxunan, 21 May 1985, *E. Cabrera et al. 8523* (MEXU); same locality, 25 Jul 1986, *E. Cabrera & H. de Cabrera 11748* (MEXU); 9 km al N de Champotón, 28 Jul 1987, *E. Cabrera & H. de Cabrera 14056* (MEXU); 4 km SO de la desviación a Lerma, carretera Campeche-Champotón, 20 Sep 1987, *E. Cabrera & H. de Cabrera 14422* (MEXU); Ruinas Balancú, 18º32'30''N, 90º56'10''W, 150 m, cult., 5 Jul 1995, *C. Gutiérrez B. 4475* (MEXU); 3 km al W de E. Echeverría Castellot I, 18º31'45''N, 89º35'21''W, 240 m, 28 Oct 1997, *E. Martínez S. et al. 29562* (MEXU); Champoton, 7-15 Jul 1932, *W.C. Steere 1813* (F); **Chiapas**: 7.5 km al E de Tapachula, 14º55' N, 92º29'W, 160 m, 21 Oct 1986, *R. Arcos Vernet 40* (MEXU); road from Chiapa de Corzo to Villa de Acala, 16º37'51''N, 92º54'51''W, 412 m, 30 Nov 2012, *L. Bohs et al. 3929* (BHCB, MEXU); 15 miles north of Ocozocoautla along road to Mal Paso, 2900 ft, 12 Jun 1965, *D.E. Breedlove 10371* (CAS, LE); Frontera Comalapa, 30 miles S of La Trinitaria, 3100 ft, 15 Aug 1965, *D.E. Breedlove 11805* (CAS); 11 miles S of La Trinitaria, 3500 ft, 14 Oct 1965, *D.E. Breedlove & P.H. Raven 13239* (CAS); 5 km E of Berriozábal along Mexican Highway 190, 800 m, 11 Oct 1971, *D.E. Breedlove 20370* (CAS, MEXU, MO); 3-5 km above Solosuchiapa, along road to Tapilula, 450 m, 26 Jul 1972, *D.E. Breedlove 26482* (CAS, MO); 6 km N of Arriaga, 250 m, 14 Aug 1972, *D.E. Breedlove 26889* (CAS, MEXU, MO); 3 Km S of Suchiapa along road to Villa Flores, 500 m, 26 Sep 1972, *D.E. Breedlove 28050* (CAS, MO); Ocosingo, at the ruins of Yaxchilán on the Banks of the Rio Usumacinta, 300 m, 26 Feb

1973, *D.E. Breedlove 33849* (CAS); near Francisco Madero, 20 km N of Cintalapa, 1250 m, 25 Aug 1974, *D.E. Breedlove 36628* (CAS); Cintalapa, 5 km W of Rizo de Oro, 900 m, 26 Aug 1974, *D.E. Breedlove 36682* (CAS, MEXU, MO); Acala, Presa La Angostura, 45 km from Tuxtla, 700 m, 9 Sep 1974, *D.E. Breedlove 37442* (CAS, MO); Tzimol, 15 km S of Comitan, on road to Tzimol and Tuxtla Gutierrez, 1200 m, 27 Dec 1980, *D.E. Breedlove 48935* (CAS, MEXU, MO); Angel Albino Corzo, above Finca Cuxtepec, 1380 m, 11 Aug 1981, *D.E. Breedlove 52096* (CAS); a 24 km al e de Pujiltic, sobre Carretera V. Carranza-Tzimol, 24 Jun 1982, *E. Cabrera et al. 3001* (MEXU, MO); a 300 m zona arqueológica Palenque, 1 Jul 1982, *E. Cabrera & H. de Cabrera 3130* (MEXU); a 25 km al S de la desviación a Chancala, carretera Palenque-Ococingo, 28 Sep 1983, *E. Cabrera et al. 5643* (MEXU); alrededores zona arqueológica de Palenque, 2 Aug 1984, *E. Cabrera & H. de Cabrera 7021* (MEXU); Mapastepec, en el poblado de Guadalupe Victoria, 180 m, 22 Nov 1977, *J.I. Calzada et al. 3990* (MEXU); Reserva del Ocote, camino al cerro La Colmena, 700 m, cult., 28 Apr 1983, *J.I. Calzada et al. 9679* (MEXU); Ocasingo, 4 km al E de Chajal, 125 m, 23 Feb 1985, *G. Castillo C. et al. 4136* (MEXU); Ejido Las Nubes, 16º18’0’’N, 93º54’3’’W, 250 m, 27 Nov 2002, *R. Cruz D. & A. Reyes 5639* (MEXU); Cerro Monte Bonito, 1 km al SE de Col. Alianza, 16º19’10’’N, 93º51’15’’W, 550 m, 29 Nov 2002, *R. Cruz D. & A. Reyes 5668* (MEXU); MEX 190, 5 mi. W of Cuauhtemoc, 20 Sep 1978, *W. D’Arcy 12102* (MEXU, MO); PN Cañón del Sumidero, Cañada Muñiz, 16º54’01.1’’N, 93º07’51.8’’W, 1323 m, 2 Jul 2007, *J.A. Espinosa Jiménez 143* (MEXU); Ruinas de Palenque, 17°29'16"N, 92°02'14"W, 55 m, 6 Mar 1983, *R. Fernández Nava & Z. Guadarrama 1395* (MO); Tuñajen, 1000 m, 27 Jul 2001, *M. Gutiérrez & T. Acero 76* (MEXU); Rancho La Suiza, 400 m al NO hacia Rancho El Ciprés, 16º2’33’’N, 93º23’38’’W, 2 Jul 2003, *F. Hernández Najarro & C. Méndez Morales 1310* (MEXU, MO); road from Palenque to Ococingo, 360 m, 8 Oct 1984, *M.J. Huft & E. Cabrera 2436* (MEXU, MO); along the Río Grijalva, 10 km S of Mexican Highway 190, road to Acala at Nandaburri, 1600 ft, 18 Jul 1966, *R.M. Laughlin 1296* (CAS, MEXU); Rancho Carmen, along the road from Acala to V. Carranza, 2600 ft, 18 Jul 1966, *R.M. Laughlin 1325* (CAS, MEXU, US); same locality, 23 Aug 1966, *R.M. Laughlin 1667* (CAS, MEXU); Soyatitán along the road from Pinola Las Rosas to Pugiltic, 3500 ft, 12 Sep 1966, *R.M. Laughlin 2012* (CAS); near Rancho Carmen, along the road from Acala to V. Carranza, 2500 ft, 25 Oct 1966, *R.M. Laughlin 2654* (CAS, US); en campo Muspac, a 3 km al NE de Puente “Cupano” de Est. Juárez a Ostuacán, 100 m, 13 Feb 1984, *E. Martínez S. & L. Rico 6062* (MEXU); a 5 km al N de J. Mujica, camino a Tzimol, 16º05’52’’N, 92º12’37’’W, 800 m, 15 Dec 1987, *E. Martínez S. & A. García 22066* (MEXU); Escuintla, cult., 5 Apr 1948, *E. Matuda 17590* (MEXU); Jalapa, Escuintla, 15 Jul 1948, *E. Matuda 18120* (MEXU); km 306, Carretera Tapachula-Tuxtla Chico, 280 m, 12 Nov 1999, *A. Miranda et al. 1644* (MEXU); Encañada a Chacona (NO Tuxtla), 21 Aug 1949, *F. Miranda 5482* (MEXU); El Zapotal, al SE de Tuxtla Gutz., 680 m, 2 Jul 1988, *E. Palacios E. 636* (CAS); 500 m al SW del entroque Aeropuerto-Ocozocoautla Cintalapa, 17 Dec 1987, *A. Reyes García 237* (MEXU, MO); 1 km SE del entroque Tzimol-Uninajab, 16º08’00’’N, 92º21’44’’W, 1110 m, 15 Sep 1988, *A. Reyes García & G. Urquijo 833* (MEXU, MO); 1 km al NW del entroque Aeropuerto-Ocozocoautla-MEX 190, 17 Sep 1988, *A. Reyes García & G. Urquijo 981* (MEXU, MO); 1 km al NW del entroque Aeropuerto-Ocozocoautla-Mexico, carretera Mexico 190, 950-1000 m, 16 Jul 1990, *A. Reyes García & R. Hampshire 1936* (MEXU, MO); Arriaga, en el puente El Toronjal, sobre carr. Mexico 190, 16º19.7’N, 93º52’16’W, 450 m, 9 Aug 2002, *A. Reyes García et al. 5319* (MEXU, MO); Ejido La Paz, 16º18’25’’N,

93º51’32’’W, 350 m, 9 Oct 2003, *A. Reyes García et al. 5867* (MEXU, MO); El Carmen, 13 km al SW de Tuxtla Gtz., 600 m, 21 Oct 1989, *J.C. Soto et al. 13288* (CAS, MEXU); en el Aguacero, al E de Cintalapa, 18 Nov 1984, *O. Téllez V. et al. 8073* (MEXU); Palenque Archeological Site, 3 mi S of Palenque, 500 ft, 27 Jun 1970, *R.F. Thorne & E. Lathrop 40532* (CAS); El Chorreadero, 5.6 miles SE of Chiapa de Corzo, along Mexican Highway # 190, 2400 ft, 12 Aug 1967, *A.S. Ton 2744* (CAS, LE, MEXU, US); same locality, 20 Sep 1967, *A.S. Ton 2956* (CAS, MEXU, US); above Finca Carmen, along the road from Acala to Pugiltik, 1800 ft, 3 Oct 1967, *A.S. Ton 2996* (CAS); Venustiano Carranza, along road from Acala to Pugiltik, 1800 ft, 7 Nov 1967, *A.S. Ton 3158* (CAS, CTES, LE); El Chorreadero, 2400 ft, 28 Nov 1967, *A.S. Ton 3261* (CAS, MEXU); La Montañita, a 5 km al NW del poblado de Acacoyahua, 100 m, 24 Aug 1988, *R. Trujillo E. & S.D. Martínez y Day 75* (MEXU); La Trinitaria, ca. 1-2 km SO of La Florida, Jan-May 1977, *B. Voorhies & A. Sanchez 61-12* (CAS); **Chihuahua**: Guimivo, 3000-3100 ft, 16 Aug 1971, *R.A. Bye 1875* (MEXU); El Limon, Rio Mayo, 25 Jul 1935, *H.S. Gentry 1541* (GH, MEXU, MO, UC, US); Guasaremos, Rio Mayo, 5 Aug 1936, *H.S. Gentry 2359* (GH, MEXU); Chihuahua, Aug/Nov 1885, *E. Palmer 230* (G, GH, L, MEXU); Orisivo, 11 km en línea recta, al SE de la cabecera municipal de Uruachi, 27º47’49,1’’N, 108º07’19,5’’W, 1005 m, 18-21 Jul 2010, *D. Tejero-Díez 6174* (MEXU); same locality, 27º48’10’’N, 108º06’53’’W, 1450 m, 18-21 Jul 2010, *D. Tejero-Díez 6190* (MEXU); Batopilas, entre Kirare y la Bufa, 600-1000 m, 18 Jul 1984, *P. Tenorio L. & C. Romero de T. 6591* (MO); Barranca de la Bufa, 141 km al SW del entronque con la carr. Guachochi-Creel, 17 Sep 1983, *R. Torres C & P. Tenorio L. 3751* (MEXU, MO); **Coahuila**: 4 km W of Musquiz, 1600 ft, 3 Oct 1970, *D. Bates et al. 1481* (NY); Rancho La Escondida, al NO de Minas de Barroterán, 27º43’03’’N, 101º26’51’’W, 634 m, 5 Dec 2007, *J.A. Encina et al. 2553* (MEXU); Sierra de San Antonio, Canyon at San Antonio de los Alamos, 2-3 Sep 1940, *I.M. Johnston & C.H. Müller 949* (CORD, GH); Muzquiz, 7 Apr 1936, *E. Marsh 256* (MEXU); Cañón de Milagro, eastern side of Sierra de los Guajes, 10 Sep 1941, *R.M. Stewart 1522* (CORD, GH); Sierra de la Gloria, Cañón El Cono, a side cyn of C. Chilpitín, 26º49’23’’N, 101º16’55’’W, 1300 m, 6 Sep 1976, *T. Wendt & D. Riskind 1611* (MEXU); Hacienda Mariposa, 17 Aug 1937, *F.L. Wynd 687* (GH, US); **Colima**: Manzanillo, 5 miles SE of town, 10 m, 8 Aug 1938, *W.J. Eyerdam & A.A. Beetle 8720* (UC); **Durango**: Topia, Rancho El Guazimal, 25º11’N, 106º34’W, 1070 m, 25 Sep 1988, *S. Acevedo et al. 1331* (MEXU); 5 km de Temoaya, 1350 m, 23 Sep 1982, *S. González & R. Fernández 2186* (MEXU); Tabahueto, brecha Tepehuanes-Tabahueto, 1330 m, 29 Aug 1983, *E. Torrecillas 78 & 103* (MEXU); **Guerrero**: a 300 m al N del caserío La Vainilla, rumbo al Calabazalito, 17º42’00’’N, 101º31’30’’W, 250 m, 23 Sep 1989, *C. Gallardo et al. 115* (MEXU); a 100 m al S del caserío La Vainilla, camino al mirador, 17º42’00’’N, 101º31’30’’W, 260 m, 23 Mar 1990, *C. Gallardo et al. 392* (MEXU); a 1.5 km al W del caserío La Vainilla, cañada de las Pozas, 17º42’00’’N, 101º31’30’’W, 300 m, 25 Jul 1990, *C. Gallardo et al. 608* (MEXU); Paraje La Cuesta del Mango Solo, camino real La Paz-La Unión, 50 m, 15 Aug 1993, *E. Guízar Nolazco & L. Pimentel B. 2884* (MEXU); poblado de La Unión, huerto familiar, 30 Nov 1993, *E. Guízar Nolazco & L. Pimentel B. 2954* (MEXU); 3 km NW of Zumpango, 1000 m, 25 May 1973, *B.F. Hansen et al. 1497* (MEXU, MO, US); 18 km al N de La Unión por la terracería a Coahuayutla de Guerrero, 300 m, 13 Nov 1983, *S.D. Koch & P.A. Fryxell 83123* (NY); La Venta (cerro), Las Piñas, frente al cerro El Peregrino, 17º7’0’’N, 99º35’W,18 Aug 1968, *H. Kruse 1906* (MEXU); en las Juntas del Río, 15 km al N de La Unión, 150 m, 24 Oct 1983, *J.C. Soto Núñez et al. 6021* (CAS, MEXU, MO); Casa

Verde, cerca de Xochipala, 800 m, 3 Sep 1962, *J. Rzedowski 16094* (CAS); El Ranchito, 13 km al NE de El Paraíso, 1100 m, 21 Mar 1985, *J.C. Soto Núñez & S. Aureoles C. 7889* (MEXU); **Hidalgo**: Calnali, 1050 m, 14 Nov 2006, *A. Blanco-Macías 1559* (MEXU); El Capulín, 6 km del Entroque de la Brecha a Pisaflores con la carretera Jacala-Tamazunchale, 680 m, 26 Oct 1982, *P. Tenorio L. & C. Romero de T. 2389* (MEXU); **Jalisco**: Est. Biol. Chamela, Vereda Tejón, 24 Sep 1985, *M.G. Ayala 232* (MEXU); Unión de Tula, ca. 50 km N of Autlan de Navarro, on Hgw 80, 1360 m, 7 Oct 1985, *B. Bartholomew et al. 2719* (CAS, MEXU, NY); Est. Biol. CHAMELA, 20 Sep 1982, *S.H. Bullock 1231* (MEXU); Est. Biol. CHAMELA, Vereda Tejon, 24 Oct 1987, *S.H. Bullock 1937* (CAS); a 8 km de las oficinas de la Fundación Cuixmala, rumbo a El Mirador, 19º28'4''N, 104º58'16''W, 300 m, 15 Nov 1997, *J. Calónico S. et al. 5810* (MEXU); brecha a Chiquilistlán, 15 Nov 1987, *M. Chazaro B. et al. 5164* (MEXU); La Huerta, Estación de Investigación, Experimentación y Difusión, Chamela-UNAM, 100 m, 18 Sep 1976, *A. Delgado et al. 81* (CAS); Ameca, a 2 km de Quexpan, camino a Las Raíces, 1110 m, 21 Sep 1995, *A. Delgado S. et al. 1542* (MEXU); unión del sendero El Buho con el arroyo el zarco, en la Est. Biol. Chamela, 19º29'57''N, 105º02'17.4''W, 40 m, *A. Domínguez Mariani 522* (MEXU); sendero Calandria, dentro de la Estación Biol. Chamela, 19º30'10.5''N, 105º01'56.4''W, 110 m, 26 Sep 1997, *A. Domínguez Mariani 601* (MEXU); San Martín Hidalgo, 200 m al N de Río Grande, 1400 m, 20 Nov 1989, *J.J. Guerrero-Nuño 586* (MEXU); Las Moras, ranchos de "Abajo", 1620 m, 10 Oct 1998, *J.A. Lomelí Sención 2908* (MEXU); La Huerta, Rancho el Paraíso, a ca. 4 km al SE de la Est. Biológica CHAMELA (UNAM), 19°30'N, 105°03'W, 28 Oct 1981, *E.J. Lott & J.A. Magallanes 659* (CAS, MEXU); Est. Biológica CHAMELA, 2 Nov 1981, *E.J. Lott 711* (MEXU); Est. Biológica CHAMELA, 28 Sep 1980, *J.A. Magallanes 2691* (MEXU); Arroyo Tapeixtes (La Mina) subiendo hacia el cerro Colorado, 16 Oct 1985, *P. Magaña Rueda et al. 340* (MEXU); Guadalajara, 21 Sep 1886, *E. Palmer 638* (U); Est. Biológica CHAMELA, 28 Oct 1971, *A. Pérez J. 521* & 2 Oct 1973, *A. Pérez J. 766* (MEXU); Est. Biológica CHAMELA, sobre sendero del área de enseñanza, marcador "Heno", 19.49995ºN, -105.0445ºW, 43 m, 2 Oct 2010, *G.A. Salazar et al. 8074* (MEXU); alrededor del km 7 del camino ciejo de terracería a Nacastillo, 19º31'51.5''N, 105º01'15''W, 100 m, *C. Soto et al. 4025* (MEXU); **Michoacán**: Tancitaro region, 5 Mar 1941, *W.M. Leavenworth & H. Hoogstrael 1339* (F, MO); Anguililla, Rancho El Seis, 830 m, 9 Oct 2005, *X. Madrigal Sánchez 4875* (MEXU); 3 km al W de Maquili, camino a Aquila, 24 Sep 1983, *E. Martínez S. et al. 4476* (MEXU); a 52 km al NO de Playa Azul, rumbo a Caleta de Campos, 130 m, 25 Mar 1981, *J.C. Soto Núñez & R. Torres C. 2764* (MEXU, MO); San Juan de Alima, 20 km al S de Coahuayana, 20 Oct 1985, *J.C. Soto Núñez 11189* (MEXU); Cerro Zinaparo, 20º07'N, 102º02'W, 490 m, 29 Aug 1992, *I. Trejo 2537* (MEXU); **Nayarit**: Tepic, Alameda Central, 1.5 m, 26 Jun 1987, *R. Ruenes M. 22* (MEXU); **Nuevo León**: Santiago, 500 m, 15 Nov 1982, *G. Cano s.n.* (CORD 00088596, MEXU); along Hwy 85, between Monterrey and Ciudad Victoria, 25º15'N, 99º59'W, 500 m, 11 Jan 1987, *T.B. Croat & D.P. Hannon 62887* (MEXU, MO); Guadalupe, 1853, *Duchassaing de Fontbressin s.n.* (P 00410140); 21.2 mi. N of Montemorelos, out of Margarita, 9 Aug 1972, *C.T. Dziekanowski et al. 1717* (MEXU, MO); between Linares and Iturbide, near Km 32, 2600 ft, 28 Nov 1969, *P.A. Fryxell 1213* (NY); La Escondida, Josecito, 1600 m, 13 Nov 1993, *G.B. Hinton 23896* (MEXU, MO); Montemorelos, Los Lirios, 315 m, 19 May 1994, *G.B. Hinton et al. 24206* (MEXU, MO); Monterrey, 6 Jul 1933, *C.H. Müller & M.T. Müller 131* (GH, MEXU); Sierra Madre Oriental, below Alamar, 15 miles SW of Galeana, 45-5000 ft, 30 May 1934, *C.H. Müller &*

M.T. Müller 647 (GH, LE, MEXU, MICH); 20 mi W of Linares, near km 35 & Los Pinos, 3600 m, 14 Sep 1967, *R.L. Oliver et al. 1106* (MEXU, MO); Campeche Hopelchén, zona arqueológica Dzibilnohcac, 17 Jul 1986, *J.J. Ortiz & S. Avendaño 1055* (MEXU, MO); near Monterrey, 9 Jul 1888, *C.G. Pringle 1912* (G, GH, LE, M, MO, P, U, UC, US, W); valley of Monterrey, 11 Jun 1889, *C.G. Pringle 2706* (MEXU); vicinity of Monterrey, 1946, *J.J. Roybal 539* (MEXU); Sierra de Picachos, Rancho el Gallo, 26º02'N, 99º48'W, 650-750 m, 12 Nov 1994, *J.A. Villarreal et al. 18036* (MEXU); Hacienda Vista Hermosa 35 mi S of Monterrey, ca. 2200 ft, 27 Jun 1939, *S.S. White 1606* (GH, LP); **Oaxaca**. Santo Domingo Tehuantepec, Los Cocos, entrada por San Pedro Huamelula, carretera por Asunción Tlacolutita, 16.04 N, 95.39W, 115 m, 30 Aug 1994, *J.I. Calzada 19320* (CAS, CTES, MEXU, MO); faldas del Cerro Indio Dormido, al S de Laollaga, 16º34'N, 95º14'W, 3 Dec 1991, *A. Campos V. 4107* (MEXU); al NE de playa de Cacaluta, 15º45'00''N, 96º10'00''W, 20 m, 3 Nov 1992, *G. Castillo C. et al. 9327* (MEXU); Sierra de Juárez, along Hwy 175 between Valle Nacional and Oaxaca, 1540 m, 30 Jun 1977, *T.B. Croat 39814* (MEXU, MO); between Esmeralda and Rio Verde, 17º10'N, 94º45'W, 100 m, 19 Jan 1987, *T.B. Croat & D.P. Hannon 63278 A* (MEXU); Santa María Huatulco, cuenca Baja del Río Cacaluta (Lomerios), 30 m, 27 Sep 2005, *E. Domínguez-Licona et al. 1824* (MEXU); Barra de la Cruz, 2.5 km NW LR, 15º51'17''N, 95º59'10''W, 175 m, 13 Jul 1998, *M. Elorsa C. 367* (MEXU); Arroyo Grande, 2 km al W de Barra de la Cruz, 15º50'30''N, 95º58'40''W, 140 m, 18 Jun 2000, 18 Jun 2000, *M. Elorsa C. 3132* (MEXU); Santiago Astata, 3 km al SE de Barra de la Cruz, 80 m, 27 Aug 2002, *M. Elorsa C. 6168* (MEXU, MO); 100 m al E del puente de Sta. María Huamelula, 15º59'53.9''N, 95º41'54.7''W, 140 m, 5 Sep 2002, *M. Elorsa C. 6186* (MEXU); Cerro "La Pedrera", a 3.2 km al NO de Nizanda, 6º40'51''N, 95º1'40''W, 400 m, 21 Jul 1996, *C. Gallardo Hernández & E. Pérez García 1869* (MEXU); Dist. Juchitán, La Pedrera, 20 km al N de La Ventosa, 120 m, 12 Aug 1988, *A. García Mendoza et al. 4023* (MEXU, MO); a 4 km al NE de San Pedro Huamelula, San Francisco, 4 Jul 1980, *S. González Olivares 494* (MEXU); camino a Cordoncillo, 15º58'34.5''N, 95º37'2.8''W, 34 m, 15 Sep 2009, *F. Gopar Vasquez 122* (MEXU); Piedra Ancha, 96º37'1.3''N, 18º0'43.5''W, 488 m, 7 Apr 2005, *G. Juárez García 1630* (MEXU); a 1.5 km al NE de la playa San Agustín, 15º41'54,2''N, 96º13'46.8''W, 30 m, 31 Jul 2006, *G. Juárez García et al. 2678* (MEXU); Sierra San Pedro Nolasco, 1843-1844, *C. Jurgensen 861* (G); Pochutla, carretera a Salina Cruz, 17.5 km al E del entronque com la carretera Pochutla-Puerto Angel, 130 m, 14 Nov 1979, *S.D. Koch et al. 79532* (CAS, MEXU, MO, NY); Juchitán, ca. 20 km al S de Matías Romero, 350 m, 12 Dec 1980, *D.H. Lorence & R. Cedillo 3078* (MEXU, MO); Tehuantepec, recorrido Hierba Santa a El Limón, 24 Jul 1985, *C. Martínez R. 76* (MEXU); Tehuantepec, 26 Jul 1936, *E. Matuda 247* (MEXU, MICH, US); faldas del Cerro Naranjo, a 5.1 km al NO de Nizanda, 16º41'53''N, 95º2'23''W, 190 m, 10 Jul 1995, *J. Meave del Castillo & E. Pérez-García 1837* (MEXU, MO); SE de Nizanda, 16º39'8''N, 95º0'13''W, 150 m, *J. Meave del Castillo & E.A. Pérez García 2350* (MEXU, MO); El Portillo, en el ejido El Porvenir, 16º37'50.6''N, 94º49'58.8''W, 110 m, 28 Nov 2006, *A. Nava Zafra et al. 1812* (MEXU); Relleno Sanitario, Santa María Huatulco, Pochutla, 24 Oct 2001, *H.J. Ortega & L.R. Ortega 865* (MEXU); a 500 m al N de Nizanda, bosque ripario hacia el "Agua Tibia", 16º40'2''N, 95º0'35''W, 215 m, *E. Pérez-García & B. Reyes Días 920* (MEXU); San Isidro Chacalapa, Las Peñas, 15º55'6''N, 95º56'39''W, 175 m, 17 Jun 1997, *J. Rivera H. et al. 371* (MEXU); Coatepec, 19º24'N, 96º48'W, 400 m, 14 Sep 1988, *J.P. Rosenthal 104* (MEXU); al sur de San Juan Guelavía, 16º56'46.8''N, 96º32'58.5''W, 1585 m, 25 Aug

2001, *N. Ruiz Núñez 6 & 7* (MEXU); San Isidro Chacalapa, 15º55’6’’N, 95º56’39’’W, 175 m, 9 Jul 1998, *A. Salas A. et al. 90* (MEXU); Zimatán, 4.5 km del puente, por la brecha a Xadani, 15º52’25’’N, 96º0’49’’W, 220 m, 21 Jul 1998, *S.H. Salas M. et al. 1851*. (MEXU); 220 m al N del Puente Sta. Ma. Huamelula, sobre la vereda al Naipu, 16º0’5.6’’N, 95º41’56.9’’W, 70 m, 26 Sep 2001, *S.H. Salas M. et al. 4061* (MEXU); entrada hacia la laguna Zanate, sobre el Sendero del Caminate, 15º43’59,7’’N, 96º9’15.1’’W, 10 m, 29 Oct 2004, *A. Sánchez Martínez & A. Nava 230* (MEXU); Laguna el Zanate, sobre el Sendero del Caminante, 15º43’50.1’’N, 96º9’29.3’’W, 10 m, 29 Oct 2004, *A. Sánchez Martínez & A. Nava 247* (MEXU); sobre el caminho viejo a Santa María Huatulco, hacia la estación de campo el Sabanal, 15º45’33’’N, 96º10’31,2’’W, 53 m, 30 Nov 2004, *A. Sánchez Martínez & A. Nava 548* (MEXU); caminho de Aguaje Anona hacia el Río Suchil, 15º46’16.2’’N, 96º12’53.4’’W, 70 m, 25 Aug 2005, *A. Sánchez Martínez et al. 983* (MEXU); Itsmo de Tehuantepec, La Ventosa, en el domicilio de la sra. Julia Rosado López, 16º33’27’’N, 94º56’48’’W, 38 m, 8 Jun 2014, *F. Sánchez et al. 954* (MEXU); en la misma localidad, en el domicilio del sr. Faustino L. Fuentes, 16º33’15’’N, 94º56’43’’W, 35 m, 7 Aug 2014, *F. Sánchez L. et al. 1190* (MEXU); Itsmo de Tehuantepec, caminho al penhasco de Nisa ndadxiña, 16º35’38’’N, 94º55’56’’W, 71 m, 7 Nov 2013, *F. Sánchez L. et al. 258* (MEXU, MO); a 100 m de la carretera Panamericana, por el puente del Río Lagartero, 16º33’05’’N, 94º56’23’’W, 32 m, 27 Nov 2013, *F. Sánchez L. 317* (MEXU); la Ventosa, cult. domicilio particular, 16º33’27’’N, 94º56’48’’W, 34 m, 2 Jun 2014, *F. Sánchez L. & P. Trujillo V. 874* (MEXU); Santa María Huatulco, 15º44’43’’N, 96º9’17’’W, 65 m, 21 Jul 2005, *A. Saunes V. et al. 5641* (MEXU); 0.35 km de la Universidad del Mar, 15º46’49,2’’N, 96º9’50,1’’W, 100 m, 24 Jul 2005, *A. Saunes V. et al. 5676* (MEXU); Asunción Ixtaltepec, 1.3 km (LR) NE de La Mata, 16º37’20,7’’N, 94º57’54.6’’W, 230 m, 14 Aug 2002, *A. Saynes Vásquez et al. 3263* (MEXU); Pochutla, 0.45 km del tante de agua Cacaluta, 15º44’33.9’’N, 96º9’16.5’’W, 50 m, 20 Jul 2005, *A. Saynes Vásquez et al. 5601* (MEXU); Tehuantepec, ladera S del Cerro Guiengola, 750 m, 16 Sep 1985, *M.L. Torres et al. 199* (MEXU); 23 km al Se de El Morro Mazatlán, carr. Salina Cruz-Pochutla, 60 m, 30 Jun 1984, *R. Torres C. et al. 5424* (MEXU, MO); 1.6 km al N de Lachiviza, caminho a Guevea de Humboldt, 280 m, 29 Aug 1986, *R. Torres C. & L. Cortés A. 8821* (MEXU, MO); Tuxtepec, bajada del prédio El Aguila a San Agustín, 17º50’N, 96º06’W, 550-270 m, 19 Jan 1988, *R. Torres C. & E. Martínez S. 11099* (MEXU, MO); Huatulco, Estero La Salina, 23 Oct 1998, *C. Tovilla Hernández & I. Escalona Luttig 480* (MEXU); El Merodio, 16º33’56’’N, 94º55’16’’W, 91 m, 1 Oct 2013, *K. Velasco G. et al. 5532* (MEXU); San Mateo del Mar, Col. Reforma, 4 m, 25 Jul 1978, *D. Zizumbo & P. Colunga 220* (CAS, MEXU); **Puebla**: Pahuatlán, S del Pueblo, 20º16.5’N, 98º08.9 W, 1050 m, 23 Jul 1988, *R. Bye et al. 16386* (MEXU); El Salto, 35 km NE de Villa Juárez, 10 Dec 1978, *M.T. Germán R. et al. 1005* (MEXU); entre el Pueblo La América y la Colonia Cafetalera “El Jonote”, 15 Jun 1962, *J. Sarukhán et al. 3581* (MEXU); Xochitlán de V. Suárez, 19º58’N, 97º44’W, 850 m, 27 Apr 1987, *G. Villalobos C. & E. Guerrero 182* (MEXU); Zapotitlán de Mendez, en huerto familiar, 20º03’N, 97º45’W, 590 m, 20 Nov 1987, *G. Villalobos C. & E. Guerrero 205* (MEXU); Jonotla, Ecatlán, 20 May 1976, *D. Zizumbo & P. Colunga 299* (MEXU); **Querétaro**: ca. 1 km al S de Arroyo Seco, 1020 m, 16 Jul 1991, *E. Carranza González 3266* (MEXU); camino de La Nopalera a La Puerta, 20º38’10,8’’N, 99º42’44,4’’W, 1971 m, 25 Aug 2004, *R. Hernández Magaña 12093* (MEXU); El Pato, cerca de San Pablo Tolimán, 20º47’32’’N, 99º52’19,5’’W, 1861 m, 22 Sep 2004, *R. Hernández Magaña 12130* (MEXU); Tolimán, comunidad de Casas Blancas, 20º55’N, 99º58’W, 1752 m, Sep 2004, *H.L. Martínez Torres*

57 (MEXU); Tancoyol, a 1 km de la desviación hacia comunidad de Zoyapilca, 21º19'N, 99º18'W, 841 m, Oct 2004, *H.L. Martínez Torres 82* (MEXU); a 1 km de la entrada principal de comunidad el Refugio, 21º33.74'N, 99º43.96W, 983 m, *H.L. Martínez Torres 98* (MEXU); Landa, 2 km al Oriente de La Peña Hidalgo, Rancho Nuevo, 800 m, 8 Dec 1988, *H. Rubio 328* (MEXU); Querétaro, em lomeríos altos, 18 Nov 1980, *J. Sánchez C. 12* (MEXU); carretera Querétaro-S. Luis Potosí, saliendo de Querétaro, 20º40'47.3''N, 100º26'1,7''W, 1890 m, 20 Oct 2001, *H. Vibrans 2003* (MEXU); **Quintana Roo**: a 0.75 km al N de V. Carranza, en la Laguna, 19º16'54.2''N, 88º45'31''W, 50 m, 23 Aug 2005, *D. Álvarez et al. 11583* (MEXU); Cobá, Nohoch-Mull, 20 m, 2 Oct 1974, *Barrera et al. s.n.* (MEXU 901450); a 6 km al N de Xel-Ha, 9 Nov 1980, *E. Cabrera & L. Cortéz 208* (MEXU, MO); a 5 km al W de F. Carrillo Puerto, rumbo a Vigía Chico, 16 Nov 1980, *E. Cabrera & L. Cortez 427* (MEXU, MO); en el antiguo aeropuerto de Cancún, 14 Jan 1981, *E. Cabrera & R. Torres 938* (CAS, MEXU); 8 km al E de Carrillo Puerto, sobre camino a Vigía Chico, 24 Mar 1983, *E. Cabrera et al. 4578* (MEXU, MO, NY); a 4 km al oeste de Playa del Carmen, 21 Jun 1984, *E. Cabrera & H. de Cabrera 6458* (MEXU); 500 m al NO de Playa Lancheros, Isla Mujeres, 25 Jan 1987, *E. Cabrera & H. de Cabrera 13073* (MEXU); F. Carrillo Puerto, a 14.2 km al E de Señor, 19º51'33''N, 88º0'1''W, 51 m, 6 Mar 2002, *J. Calónico S. 22503* (MEXU); Cozumel, ruinas de Tulum, 20°15'N, 87°30'W, 0 m, 24 Nov 1980, *C. Chan et al. 30* (UC); Laguna Bacalar, 18º35'N, 88º25'W, 0 m, 9 May 1985, *C. Chan et al. 1406* (MEXU); 12 km WSW of Akumal, 6 May 1982, *A. O. Chater 41* (MEXU, MO); 12 km WSW of Akumal, 10 m, 6 May 1982, *G. Davidse et al. 20126* (MEXU, MO); Chancah Veracruz, F. Carrillo Puerto, 10 m, huerto familiar, 23 Apr 1983, *E. Gutiérrez 25* (MEXU, MO); Coba, 20º30'N, 87º42'W, 15 m, 20 Aug 1981, *V. Rico-Gray & C. Chan 429* (MEXU); Othón P. Blanco, Reforma, 3 Sep 1989, *L. Serralta P. 104* (MEXU); a 5 km de Calderón, carretera Ucum-La Unión, 6 Sep 1980, *O. Téllez & L. Rico 3442* (MEXU, MO); Hotel de Cobá, 10 Oct 1980, *O. Téllez et al. 3770* (MEXU); **San Luis Potosí**: 30 min. walk west into sierra above Aquismon, 5 m, 24 Jul 1977, *J.B. Alcorn 1406* (MEXU); San Nicolás, 29 Dec 1978, *J.B. Alcorn 2369* (MEXU); Cerritos, cerro a SE de Montañas, Dec 2001, *D. Chicharo 278* (MEXU); Tamazunchale, 19 Jul 1937, *M.T. Edwards 592* (CAS, F, MO); 4 mi. N of Tamazunchale, near Km 200, 800 ft, 1 Oct 1969, *P.A. Fryxell 1119* (NY); Villa Hidalgo, depresión de la Joya, 1700 m, 2 Nov 1961, *A. Gómez 511* (MEXU); Tampa, Campo Experimental de la Esc. Agronomía, 23 Nov 1973, *F. Gómez-Lorence s.n.* (MEXU); ca. 2 km de El Leoncito rumbo a Peotillos, 14 Oct 1985, *F. Gómez-Lorence 877* (MEXU); ca. 10 miles west of Nuevo Morelos, on Hgw 80, Sep 1970, *D.H. Norris 17477* (CAS, MEXU, MO); near los Canos, 15-21 Oct 1902, *E. Palmer 215* (GH, US); Tanranhuitz prope Tanquian, Mar 1888, *E. Seler 253* (GH, US); between Puerta Huerta and Rioverde, in the Sierra de Alvarez, 1150-2200 m, 11 Sep 1954, *E.R. Sohns 1203* (MEXU, US); in the Sierra de Alvarez along the route Cerritos-La Joya Montaña Villar, 1100-1400 m, 21 Sep 1954, *E.R. Sohns 1384* (MEXU, US); Xilitla, camino hacia la cascada, 29 Oct 1998, *R. Subils & G. Barboza 4645* (CORD); **San Luis Potosí/Tamaulipas**: en route from San Luis Potosí to Tampico, Dec 1878-Feb 1879, *E. Palmer 1135* (GH, US); **Sinaloa**: Culiacán, a 700 m al N de Imala, 11 de Nov 1984, *J.A. Aguayo C. & H.A. Gómez R. 191* (MEXU); 3 km of Badiraguato on road to Los Pericos, 8 Sep 1983, *W.R. Anderson 12556* (CAS, MEXU, MO); vicinity of Culiacan, Sep 1904, *T.S. Brandegee s.n.* (UC); 40 km ENE of Badiraguato, S end of Sierra Surutato, 1100 m, 20 aug 1986, *D.E. Breedlove & B. Anderson 62936* (CAS); Mazatlán, 50 m, 4 Aug 1938, *W.J. Eyerdam & A.A. Beetle 8661* (GH, UC); vicinity of Labradas, 19 Sep 1925, *R.S. Ferris &*

Y. Mexia 5130 (CAS, GH); Satique, Río fuerte, 22 Nov 1933, *H.S. Gentry 949* (CAS, MICH); cerros de Navachiste about Bahia Topolobampo, 26-30 Sep 1954, *H.S. Gentry 14323* (MEXU, US); Choix, Fasajera, 420 m, *J. Gonzalez Ortega 697* (MEXU); Península de Lucenilla, a 8 km de la entrada de la península, 5 m, 28 Oct 1984, *F. Hernández A. & J.A. Gutiérrez G. 65* (MEXU); San Blas, 28 Jan 1927, *M.E. Jones s.n.* (CAS 172616, MO, RSA 0032145, UC 23119); Rancho La Tasajera, Choix, 23 Sep 1919, *M. Narváez Montes 861* (US); Hacienda Labradas, *A. Ramírez Laguna s.n.* (MEXU 28474); Culiacán, Tacuichamona, a ca. 6-7 km al SE del poblado rumbo a Chiveras Lomerios, 100-200 m, 22 Sep 1984, *J.L. Valverde R. 136* (MEXU); carretera de terracería a Ajoya, ca. 16 km al N de San Ignacio, 200 m, 21 Oct 1984, *R. Vega A. 1297* (MEXU); Rincón de los Montes, ca. carretera Pericos a Badiraguato, 250 m, 5 Nov 1992, *R. Vega A. 4511* (MEXU); **Sonora**: a 1.5 km al NW del Puerto San Javier, 28º36'10''N, 109º45'22''W, 940 m, 26 Oct 1997, *A. Búrquez & E. Búrquez 548* (MEXU); km 195 Carr. Fed. 16, a 1 km al E del Rancho La Mula, 28º28'40''N, 109º21'20''W, 950 m, 4 Sep 1996, *A. Búrquez et al. 638* (MEXU); Arroyo el Mentidero at El Chinal road, 11.3 km S of Alamos, 26º54'45''N, 108º55'05''W, 240 m, 5 Oct 1992, *T.R. van Devender et al. 1005* (CAS); Arroyo El Pilladito, near Tepoca, 28º26'20''N, 109º15'30''W, 500 m, 15 Sep 1998, *T.R. van Devender et al. 1074* (MO, NY); Cañada Adrian, 1.4 km (by air) NNW of Rancho Esmeralda headquarters, 31°13'21''N, 111°08'03''W, 1477 m, 5 Sep 2005, *T.R. van Devender et al. 1170* (CAS); El Novillo, east-central Sonora, 350 m, 24 Oct 1984, *R.S. Felger et al. 226* (MEXU); Palo Parado, Río Yaqui, 27º35'N, 110º28'30''W, 19 Nov 1985, *R.S. Felger et al. 1403* (MEXU); Cañón Nacapules, ca. 6 km N of Bahía San Carlos, 28 Dec 1985, *R.S. Felger 1502* (MEXU); 5 km al W de Sahuaripa, 29º01'30''N, 109º16'28''W, 28 Sep 1996, 720 m, *A. Flores M. & M.A. Arvizo Y. 4787* (MEXU); 2.5 km al SE del Rancho el Zacatón, carr. Hermosillo-Bacanora, 28º58'22''N, 109º39'12''W, 650 m, 29 Sep 1996, *A. Flores M. & M.A. Arvizo Y. 4860* (MEXU); Alamos, Canyon Estrella, 1 Oct 1933, *H.S. Gentry 373* (CAS); 6 miles (by road) NW of Huasavas, 3225 ft, 6 Oct 1965, *J.R. Hasting & R.M. Turner 65-84* (CAS); Rancho El Alamo, Lo Pozos drainage S into Rio Aros, 29º32'N, 108º54'W, 950-1000 m, 25 Sep 1991, *E. Joyal et al. 1761* (MEXU); Rancho el Aguilar Noria, N of Ures and Santiago, 29°33'N, 110°26'W, c. 500 m, 6 Apr 1991, *E. Joyal 1815* (CAS, MEXU); SSW side of Las Pilas (La Pirinola), ENE of Rancho El Palmar & c. 30 km E of Onavas, 28°29'N, 109°21'W, c. 1000 m, 22 Oct 1991, *E. Joyal 1926* (CAS, MEXU); Agiabampo, 10 Mar 1890, *E. Palmer 772* (U); Alamos, 300 m, 3 Aug 1935, *F.W. Pennell 19497* (US); Santa Ana de Yecora, 28º22'40''N, 109º09'W, 850 m, 9 Sep 1996, *A.L. Reina et al. 537* (MEXU); Rancho El Rincón, 3.5 km S of Sasabe, 31º26'14''N, 111º33'41''W, 1046 m, 31 Aug 2004, *A.L. Reina et al. 1012* (MEXU); Rancho La Mula, 28.2 km SE of Rio Yaqui on Mex. 16 (km 195 E of Hermosillo), 28°29'16''N, 109°21'59''W, 900 m, 21 Sep 1997, *A.L. Reina et al. 1123* (CAS); Tierras Blancas, 2 km al N de El Rincón, Brecha a Tamazula, 24º51'N, 107º00'W, 14 Mar 1985, *P. Tenorio et al. 8305* (MEXU); "Cerro Prieto", 15 km al NE de Navojoa, 2 Oct 1983, *R. Torres C. & P. Tenorio L. 3903* (MEXU, MO); San Javier, km 135 carr. 16, a 10 km al NW de la desviación al poblado, 28º35'20''N, 109º37'36''W, 500 m, 29 Aug 1996, *L. Varela E. 179* (MEXU); Río de Bavispe, Piedra Parada, 2-3 Sep 1940, *S.S. White 3640* (GH, MEXU); 11 miles northeast of Colorado on the road to Mazatlan, 6 Sep 1941, *I.L. Wiggins & R.C. Rollins 338* (GH, MO, UC, US); from Ures on road to Babiacari, 2860 m, 21 Sep 1934, *I.L. Wiggins 7368* (CAS, GH, MICH, US); **Tabasco**: 6.69 km al E de Teapa, 17º34'8''N, 92º53'29''W, 10 m, 3 Feb 2002, *J. Calónico S. et al. 21404* (MEXU); desviación al Ejido El Destino, Balancan, 7

Apr 1976, *J.I. Calzada 2369* (MEXU); Tocotalpa, al este de Puyacatengo, carretera Tacotalpa-Teapa, 18 Nov 1978, *C. Cowan 1722* (MO); Ejido Habanero, km 6 carretera de Cárdenas a Villahermosa, en huerto familiar, 10 m, 2 Jul 1979, *C. Cowan 2352* (CAS); Ejido Corregidora Ortiz del Mezcalapa, 30 m, 15 Jul 1982, *R. Escolastico 165* (MEXU); El Bajío, km 6.5 del periférico de Cárdenas, 12 Jul 1986, *M. A. Guadarrama et al. 865* (MEXU); 15 km de El Palmar, 18º15’44.4’’N, 92º11’36’’W, 20 Jan 1999, *M. A. Guadarrama et al. 6857* (MEXU); centro acuícola de Tabasco, 25 Jun 1986, *M.A. Magaña 1579* (MEXU); Paraíso, 3 Nov 1993, *M.A. Magaña 2326* (MEXU); a 12 km de Frontera rumbo a C. del Carmen Campeche, rancho Fuster, o m, 26 Dec 1976, *A.D. Orozco-Segovia 368* (MEXU); Nacajuca, 20 m, 3 Oct 1978, *R. Ortega O. et al. 870* (MEXU); 0.5 km al S de la carretera de Teapa a Tacotalpa, 0.5 km al E de la entrada a Puyacatengo, 18 Nov 1978, *G. Romero et al. 1722* (CAS); Río Samaría, Ranchería Cucuyulapa, 18º00’N, 93º16’W, 10 m, 4 Oct 1996, *P. Tenorio L. & M. Sousa S. 19436* (MEXU); **Tamaulipas**: El Gavilán, vicinity of San José, 1700 ft, 1 Jul 1930, *H.H. Barlett 10007* (CAS, F); Sierra San Carlos, Mina La Escondida, 800 m, 4 Jul 1985, *O.L. Briones V. 1866* (MEXU); along Hwy 85, between Monterrey and Ciudad Mante, 23º25’N, 99º00’W, 300 m, 11 Jan 1987, *T.B. Croat & D.P. Hannon 62992* (MEXU); along Hwy 85, between Ciudad Mante and Ciudad Valles, 22º34’N, 99º02’W, 80 m, 12 Jan 1987, *T.B. Croat & D.P. Hannon 62956* (CAS, MEXU, MO); 10 mi. SW of Cd. Victoria on MEX 101, 8 Sep 1978, *W. D’Arcy 11824* (MEXU); region of Rancho Las Yucas, ca. 40 km NNW of Adama, 23°14’N, 98°10’W, 16 Oct 1957, *R.L. Dressler 2429* (MEXU, MO, UC); 18 km al N de El Olivo, 490 m, 1 Nov 1978, *J. García P. & A. Delgado S. 827* (CAS, MEXU); Rancho Los Alacranes, 20 km al E de Casas, 200 m, Sep 1968, *F. González-Medrano et al. 1823* (MEXU); 4 km al E de la Unión, al NW de Jaumave, 15 Sep 1976, *F. González Medrano 9692* (MEXU); 4 km al O de La Oveja, camino a Camarones, 13 Sep 1982, *F. González Medrano & P. Hiriart 12945* (MEXU); Altamira, 4 km al NE de los Miradores, 23 Sep 1983, *L. Hernández & P. Ceballos 675* (MEXU); Llera de Canales, 5 km al E de Congregación Garza, camino al Picacho, 400 m, 12 Oct 1986, *L. Hernández 2016* (MEXU, MO); al pie de la Sierra CD. Victoria, 415 m, 30 Oct 1984, *G. Malda B. 111* (MEXU); Soto la Marina, Rancho de R. Dragustinovis, 23º53’38’’N, 97º48’36’’W, 8 m, 22 Jul 2008, *E. Martínez S. & A. Ibarra 40296* (MEXU); San Carlos, Cerro del Diente, 1000 m, 2 Jun 1986, *M. Martínez 1145* (MEXU, MO); San Nicolás, a 1 km de la segunda desviación hacia Flechadores, 24º32.93’N, 98º44.8’W, 690 m, 27 Nov 1998, *M. Martínez 5105* (MEXU); a 5 km del inicio de la brecha a González, 24º34.5’N, 98º41.8’W, 660 m, 28 Nov 1998, *M. Martínez 5332* (MEXU); camino de Aldama hacia Santa Juana, a 7 km SE de Aldama, 150 m, 25 Dec 1971, *E. Martínez Ojeda 263* (MEXU, MO); 20 km al este de Casas, Rancho Los Alacranes, 200 m, Sep 1968, *F.G. Medrano et al. 1823* (MEXU; UC); Abasolo, Ejido Parras de la Fuente, 7 May 1992, *J.L. Mora-López 134* (MEXU); Santander Jiménez, NE side of Lago V. Guerrero, 140 m, 16 Jun 1982, *M. Nee & G. Diggs 24413* (CORD); Casas, El Piruli, 23°34’N, 98°32’W, 350 m, 17 Aug 1986, *M. Nee 32699* (CORD, NY, UC); de Canales, along Hwy. Mex. 85 near the Microondas (microwave) tower, between Llera and Rancho Nuevo, 23°22'N, 99°00'W, 500 m, 26 Sep 1986, *M. Nee & J.I. Calzada 33201* (CORD, MO); 10.2 mi SE of Tula (centro) on road to Ocampo, 22º56.5’N, 99º36’W, 1440 m, 16 Jun 1987, *G. Nesom et al. 6009* (F, MEXU); vicinity of Victoria, 1 Feb-10 Apr 1907, *E. Palmer 144* (GH, MO, UC, US); Villa de Casas, Vado el Moro, 23º34’N, 98º35’W, 240 m, 10-14 Oct 1991, *I. Rodríguez 104* (MEXU); valley near Nogales, 30 Sep 1931, *H.W. von Rozynski 183* (UC); Tamaulipas, *H.W. von Rozynski 211* (MICH); 9 km E of Palmillas,

23°32'N, 99°27'W, 1750 m, 15 Aug 1941, *L.R. Standford et al. 955* (CAS, GH, MO, UC); 3-5 km S of Huisachal, 27 Jun 1949, *L.R. Standford et al. 2112* (CAS, G, U, US); along Río Guayalejo, downstream from Colonia Mirador, 24 Dec 1970, *R.J. Taylor Jr. 7249* (MO, US); 3.5 km al S de Las Flores, 22°48'N, 99º24'W, 450 m, 24 Jul 1992, *I. Trejo 2319* (MEXU); Cerro Victoria, 330 m, 18 Sep 1985, *M. Yañez 440* (MEXU); 15 km de Cerro Victoria, 540 m, 24 Sep 1985, *M. Yañez 572* (MEXU); **Veracruz**: Sierra M. Díaz, Cerro El Pailón, 19º33'N, 96º26'W, 100 m, 12 Oct 1984, *R. Acosta P. et al. 39* (MEXU); Silosuchil, 3 Oct 1978, *J.B. Alcorn 1903* (MEXU); Barranca de Mayatla, 2 km NE de Tenampa, 19º16'00''N, 96º53'00''W, 6 May 1993, *S. Avendaño R. & C. Durán E. 3143* (MEXU); environs d'Orizaba, *M. Botteri 1855* (P); Ramal de Chicayan, 21º39'N, 98º17'W, 150 m, 25 Aug 1979, *J.I. Calzada 5577* (MEXU, XAL); entrada del Ejido A. Melgar, a 4 km al SE del campamento H. Cedillo, 100 m, 3 Mar 1984, *J.I. Calzada 10493* (MEXU); E. Zapata, cerca del Puente de la carretera Xalapa-Veracruz, 19°25'N, 96°39'W, 220 m, 14 Nov 1977, *G. Castillo C. 207* (QCA, UC, XAL); Apazapan, 19º21'0''N, 96º42'48''W, 1 Sep 1995, *G. Castillo C. et al. 14287* (MEXU); al S de Almolonga, 19º34'24''N, 96º47'3''W, 640 m, 26 Aug 1998, *G. Castillo C. et al. 18139* (MEXU); 2 km al N de Jalcomulco 19º20'9''N, 96º45'54''W, 460 m, 2 Sep 1998, *G. Castillo C. et al. 18164* (MEXU); Cacalilao-Mendez, 8 Nov 1970, *F. Chiang 155* (MEXU); a 20 m km de Ozuluama, camino a La Puente, 6 Dec 1970, *F. Chiang 209* (F, MEXU); Papantla, 13 Jul 1983, *M.E. Cortés-Vázquez 552* (MEXU, MO); Brecha González Ortega, 1 km antes de Plan de Palmar, 190 m, 10 Nov 1981, *M.E. Cortés-Vázquez 26* (MEXU, MO); Palmar de Zapata, en huerto, 110 m, 26 Jan 1982, *M.E. Cortéz-Vázquez 143* (MEXU, MO); Plan del Río, 14 Jan 1973, *R. Fernández M. & J. Dorantes 1840* (F, MEXU, XAL); La Laguna, 1 km al N del Poblado 6, Uxpanapa, 17º46'N, 94º38'W, 15 May 1991, *M.A. García-Bielma & P. Domínguez H. 626* (MEXU); a 3 km al N de San Carlos, dirección a La Michoacana, tramo Ebano entroque a Panuco, 22º12'N, 98º17'W, 30 m, 13 Jan 1987, *C. Gutiérrez B. 2227* (MEXU); Papantla, San Pablo, 25 m, 8 Sep 1967, *L. Gutiérrez R. s.n.* (ENCB, MEXU 137238); a 8 km al S-SE de Coatzacoalcos, 14 Jul 1999, *A.M. Hanan Alipi et al. 1386* (MEXU); 1 km N of Poza Rica, 75 m, 23 Jun 1980, *B.F. Hansen & M. Nee 7430* (F, MO, USF, XAL); Est. Biol. Tropical Los Tuxtlas, lote 67, 18º34-35'N, 95º04-09'W, 200 m, 28 Oct 1991, *G. Ibarra Manríquez 3616* (MEXU); Coatepec, 4 km E Tuzamapan, 19°22'N, 96°53'W, 640 m, 3 Nov 1975, *R. Jiménez 1* (ENCB, F, NY, UC, XAL); Villa José Cardel, 29 Jan 1954, *I. Kelly 858* (UC); Laguna Verde, 0 m, 24 Jun 1972, *A. Lot et al. 2075* (MEXU); Ignacio de La Llave, Ranchería N° 1, 4 m, 14 Feb 1967, *G. Martínez C. 1287* (CAS, F, MEXU, MO, NY); Checaltianguis, 9 m, 16 May 1969, *G. Martínez C. 1897* (CAS, ENCB, F, MEXU, MO); Catemaco, 20 Sep 1969, *G. Martínez C. 2011* (MEXU, XAL); Estación Biológica de Los Tuxtlas, 10 Jul 1970, *G. Martínez C. 3087* (CAS, ENCB, F, MEXU, NY); Colonia Militar Balzapote, San Andrés de Tuxtla, Jul 1970, *G. Martínez C. 3094* (F, XAL); ca. 4 km después de Gutiérrez Zamora, 20º20'N, 97º05'W, 30 m, 28 Aug 1984, *E. Meza P. 2* (MEXU); 5 km E of Ebano, 22º12'N, 98º21'W, 50 m, 4 Jan 1981, *M. Nee et al. 19489* (F, MEXU, XAL); 10 km O of Sontecomapan, 18º35'30''N, 95º03'W, 50-150 m, 5 Dec 1981, *M. Nee 23723* (AAU, BR, CORD, F, MA, MO, NY, XAL); vicinity of Playa Escondida, 10km n. of Sentecomapan, 18°35'30"N, 95°03'00"W, 21 Apr 1983, *M. Nee 26700* (CORD, F, MO, XAL); sur de la cuidad de Panuco, 22º03'N, 98º11'W, 20 m, cult., 24 Feb 1987, *L.M. Ortega Torres 88* (MEXU); vicinity of Pueblo Viejo, 2 km south of Tampico, 10-25 Feb 1910, *E. Palmer 394* (CAS, F, G, GH, MO, NY, US); Acaxonica, Aug 1919, *C.A. Purpus 8499* (GH, MO, UC, US); Zacuapan, Río de Santa María, Aug 1906, *C.A. Purpus*

2015 (F, MO, UC, US); a 4 km al SE de Motzorongo, 18º40'N, 96º40'W, 400 m, 29 Jul 1986, *R. Robles G. 886* (MEXU); Balzapote, 50 m, 24 Aug 1968, *M. Rosas R. 1320* (CAS, F, MEXU); E. Zapata, 19º25'N, 96º40'W, 200 m, 3-5 Sep 1988, *J.P. Rosenthal 101 & 102* (MEXU); La Balsa, 2 km al s de Carrizal, 19º21'N, 96º39'W, 400 m, 15 Sep 1988, *J.P. Rosenthal 109* (MEXU); Rinconada, 1955, *G.B. Ross s.n.* (UC); Ozuluama, prope Pánuco, Apr 1888, *E. Seler 610* (US); Campamento La Laguna, along Río Las Cuevas, 17º16'N, 94º31'W, 100 m, 6 Mar 1984, *K. Taylor 385* (CORD; NY); Hidalgotitlan, brecha Hnos. Cedillo-La Escuadra, 152 m, 6 May 1974, *B. Vásquez T. 611* (MEXU, MO, XAL); Xalapa, calle Serafin Olarte, 1350 m, 27 Sep 1976, *M.G. Zola B. 815* (MEXU); **Yucatán**: Valladolid, Xocén, cult., 15 Jul 1988, *L.E. Acosta B 187* (MO); Ejido de Santa Clara, a 38 km del Munic. Tizimín, 27 Aug 1986, *J.A. Aguilar Zepeda & S. Diez Martínez y Day 363* (MEXU); comunidad de Yokdzonot Presentado, a 42 km del Municipio de Temezón, 20 m, 25 Sep 1985, *J.A. Aguilar Zepeda & S. Diez Martínez y Day* 38 (MEXU); Thien-Welden Dzibilchaltun Survey, Site VII, 3 Jul 1979, *A. Bradburn & S. Darwin 1272* (MEXU, MO); alrededores de Chichen-Itzá, 5 Aug 1984, *E. Cabrera & H. de Cabrera 7139* (MEXU); a 2 km al O de Tahmek, carretera Valladolid-Mérida, 29 Jun 1985, *E. Cabrera & H. de Cabrera 8787* (MEXU, MO); alrededores de la zona arqueológica de Dzibilchaltum, 18 Jul 1985, *E. Cabrera & H. de Cabrera 8990* (MEXU, MO); a 12 km al W de Humucma, carretera Mérida-Celestun, 20 Jul 1985, *E. Cabrera & H. de Cabrera 9076* (MEXU); alrededores de zona arqueológica de Mayapan, a 1 km al S de Telchaquillo, 21 Jul 1985, *E. Cabrera & H. de Cabrera 9115* (MEXU, MO); alrededores de Xlapac, 27 km al SW de Oxkutzcab, 22 Jul 1985, *E. Cabrera & H. de Cabrera 9147* (MEXU, MO); alrededores de la zona arqueológica de Sayil, 29 Sep 1985, *E. Cabrera & H. de Cabrera 9560* (MEXU); a 11 km al E de Maxcanú, o a 3 km al S de Cacehtok, por el camino a Muna, 30 Sep 1985, *E. Cabrera & H. de Cabrera 9602* (MEXU); a 10 km al N de Telchak Pueblo, 1 Oct 1985, *E. Cabrera & H. de Cabrera 9654* (MEXU); a 2 km al S del Cruecero Las Coloradas-San Felipe, 20 Dec 1985, *E. Cabrera & H. de Cabrera 10056* (MEXU, MO); 5 km al S de Telchac Puerto, 24 Dec 1985, *E. Cabrera & H. de Cabrera 10269* (MEXU); 8 km al SO de Xcan, tramo Cancúb-Valladolid, 21 Jul 1986, *E. Cabrera & H. de Cabrera 11512* (MEXU); 10 km al S de Puerto Progreso, 25 Jul 1987, *E. Cabrera & H. de Cabrera 13858* (MEXU, MO); c. 19 km al O de Kantunilkin, sobre el camino a Colonia Yucatán, 18 Sep 1987, *E. Cabrera & H. de Cabrera 14277* (MEXU); ruinas de Xlapak, 20°14'N, 89°37'W, 80 m, 22 Mar 1981, *C. Chan et al. 253* (UC, MEXU); del Rancho Sin-Kewek rumbo a la Costa, Celestún, 28 Oct 1984, *C. Chan 7144* (MEXU); 11.3 km al S de Chuburná Puerto a Sierra Papacal, 21º09'05''N, 89º48'10''W, 6 Sep 1997, *R. Durán et al. 2963* (MEXU, MO); 50 m E de la caseta de Vigilancia de la Reserva de Río Lagartos, 21º26'30''N, 87º42'25''W, 2 Oct 1999, *R. Durán et al. 3485* (MEXU); Oxkutzcab, ruinas de Labná, 20 m, 25 Oct 1984, *A. Espejo et al. 1260* (MEXU); Samahil, Kuchel, en huerto familiar, 24 May 1984, *E. Estrada 43* (MEXU); Piste, ca. 4 km West of Chichen-Itzá, 20°42'N, 88°37'S, 24 Nov 1981, *H. Flügel et al. 7054* (B); Yucatán, *G.F. Gaumer 864* (CAS, F, GH, MICH, MO, UC) *& 1546* (CAS); Chichankanab, *G.F. Gaumer 1546 bis* (F); Chichankanab, 38 m, *G.F. Gaumer 2006* (B, F, GH, MO); Yucatán, 1917-1921, *G.F. Gaumer 24042* (F, B); Mérida, Progreso, km 28, May-Aug 1938, *C.L. Lundell & A.A. Lundell 7998* (GH, US); Grutas de Calcetok, 24 Feb 1992, *M. Méndez et al. 476* (MEXU); cult. Titzimín, 21 Sep 1954, *F. Miranda 8097* (MEXU); Carretera Mérida-Progreso, 15 Sep 1955, *F. Miranda 8224* (MEXU); Fortín de las Flores, Posada Loma, 850 m, cult, 20 Jul 1967, *L. Nevling & A. Gómez Pompa 196* (MEXU); Amatlán de los Reyes, Peñuela, 740 m,

6 Aug 1990, *D. Olivares Hernández 1* (MEXU); zona arqueológica de Dzibilchaltun, 21°05'N, 89°26'W, 5 m, 17 Nov 1981, *M.J. Ordonez 53* (MEXU); barda este de la zona arqueológica de Dzibilchaltun, 21°05'N, 89°35'W, 5 m, 28 Aug 1981, *M.J. Ordonez 239* (MEXU, UC); Chunchuomil, 30 km al NW de Maxcanu, en huertos familiares, 20º41'N, 90º13'W, 12 m, 29 Mar 1988, *L.M. Ortega-Torres & J. Tzuc 272* (MEXU); a 3 km de Xmatkuil yendo a Molas, 20º51'N, 89º40'W, 9 m, 2 Feb 1983, *A. Puch 1089* (MEXU); Mérida Yakché, 10 Oct 1927, *K. Reiche 908* (M); 2 km S of Tekax on highway 164, 21 Aug 1965, *K.E. Roe 1331* (US); Tixcacaltuyub, 20º29'34''N, 88º54'56''W, 20 m, 25 Feb 1988, *P. Simá 517* (MEXU); San Juan Tekax camino a Iturbe, 19.4200 N, 89.26 W, 17 Nov 1992, *P. Simá et al. 1552* (MEXU); Chichen Itzá, 25 Jun 1932, *W.C. Steere 1314* (MICH); Pixoy, 20º43'N, 88º14'W, 22 m, 3 Aug 1984, *E. Ucan Ek et al. 3445* (MEXU); Dzitas, 20º47'S, 88º31'W, 22 m, en huerto, 13 Sep 1984, *E. Ucan Ek et al. 3527* (MEXU); Pixoy, Valladolid, 20°42'53''N, 88°15'45''W, 22 m, 14 May 1985, *E. Ucan Ek 3893* (MEXU, MO, W); same locality, 17 Jul 1986, *E. Ucan Ek 4617* (MEXU, MO); Xcolak, 6 Apr 1987, *E. Ucan Ek 5058* (MEXU); km 6 al NE de Yaxcabá, 20 Jul 1979, *Vara et al. 417* (MEXU); Santa Elena, road between Cooperative & Sayil, 20°14'40"N, 89°32'15"W, 50 m, 12 Jul 1982, *D.A. White 178* (MO).

MONTSERRAT. Saint Anthony: Fergus Montain, 30 Jan 1907, *J.A. Shafer 330* (NY, US).

NETHERLANDS ANTILLES. Antilles, *A. Riedlé s.n.* (P 00409863); **St. Eustatius**: Quill National Park, Crater trail into bottom of The Quill, 17º28'38''N, 17º28'43''W, 280-400 m, 1 Feb 2008, *B.M. Boom et al. 11158* (NY).

NICARAGUA. Boaco: Empalme de Boaco y Chontales, Monte Grande, 12º25'N, 85º45'W, 200 m, 30 Sep 1980, *P.P. Moreno 3292* (MEXU, MO); along Hwy 33, ca. 2.7 km SW of Rio Sacal bridge, 12º31'N, 85º34'W, 350 m, 16 Jul 1978, *W.D. Stevens 9348* (MEXU, MO, QCA); **Carazo**: Río Escalante, Estero Chacocente, 11º32'N, 86º10'W, 100-120 m, 22 Jun 1984, *M. Aranda et al. 94* (MEXU, MO); **Caribe Sur**: Corn Island, 0-30 m, 7 Mar 1971, *E.B. Nelson 4343* (CAS, MO, NY, UC); Nueva Guinea, along Zapote River, 100-200 m, 30 Mar 1971, *E.B. Nelson 5379* (MEXU, MO); ca. 1 km of El Zapote, 11º34'N, 84º24'W, 250-300 m, 8 Sep 1983, *M. Nee & S. Vega 27926* (MEXU, MO); **Chinandega**: Chinandega, 13 Jan 1903, *C.F. Baker 148* (CAS, GH, MO, UC); east base of Coseguina Volcano, 8 Jul 1932, *J.T. Howell 10289* (CAS); Potosí, península de Coseguina, 1 Dec 1973, *S.A. Marshall & D.A. Neill 6624* (MO, UC, US); falda SE del Volcán Casita, 1 km antes de Argelia, 12º40'N, 86º57'W, 650-700 m, 30 Jul 1980, *P.P. Moreno 1544* (MEXU, MO); **Chontales**: km 131 carretera al Rama, 12º08'N, 86º26'W, 100-120 m, 19 Oct 1980, *P.P. Moreno 3727* (MEXU, MO); Cerro La Bateca, at Hacienda Veracruz, 12º11'30''N, 85º22'W, 200-375 m, 21 Sep 1983, *M. Nee 28288* (MEXU, MO); ca. 4.5 km S of Hwy 7, on road to Nueva Guinea, ca. 12°57'N, 85°45'W, 200 m, 17 Jul 1977, *W.D. Stevens 2805* (MO, QCA); along road from Juigalpa NE toward La Libertad, 12º09'N, 85º19'W, 200 m, 25 Sep 1977, *W.D. Stevens 4223* (MEXU, MO); 2.0 km N of road to Betulia along road to Cerro Margarita, 12°15'34"N, 85°16'33"W, 500 m, 20 Jan 2015, *W.D. Stevens & O.M. Montiel 35819* (MO); **Estelí**: along Estanzuela creek, 8 km W of Estelí, 1000 m, 4 Nov 1968, *A. Molina R. 23062* (MO, US); valle Santa Cruz, Cerro El Picacho, lado este, 13º01'N, 86º18'W, 900-1000 m, 24 Jul 1980, *P.P. Moreno 1271* (MEXU, MO); Valle La Cascada, 13º12'N, 86º29'W, 800-900 m, 1 Sep 1980, *P.P. Moreno 1926* (MEXU, MO);

“Los Encuentros”, 13º12’N, 86º31’W, 550-600 m, 1 Sep 1980, *P.P. Moreno 1948* (MEXU, MO); Valle de San Antonio de los Platanares, 13º12’N, 86º33’W, 300-400 m, 1 Sep 1980, *P.P. Moreno 1949* (MEXU, MO); 2 km de la Had. La Grecia, 13º12’N, 86º36’W, 280-300 m, 2 Sep 1980, *P.P. Moreno 2037* (MEXU, MO); 1 km antes de llegar a San Juan de Limay, 13º11’N, 86º37’W, 280 m, 2 Sep 1980, *P.P. Moreno 2089* (MEXU, MO); Hda. La Grecia, Lomas Las Chivas, 13º11’N, 86º34’W, 400-450 m, *P.P. Moreno 2123* (MEXU, MO); Hda. La Grecia, Cerro Quiniento, 13º11’N, 86º35’W, 518 m, 2 Sep 1980, *P.P. Moreno 2170* (MEXU, MO); entre San Juan de Limay y Pueblo Nuevo, 250-300 m, 4 Sep 1980, *P.P. Moreno 2348* (MEXU, MO); along road to San Francisco with 1 km of junction with Hwy.1, 13º00’N, 86º16’W, 900 m, 1 Sep 1983, *M. Nee 27745* (MEXU, MO); along Hwy 1, at km 121, 4 km NW of San Isidro, 12º56’N, 86º13’W, 600 m, 1 Sep 1983, *M. Nee et al. 27750* (MEXU, MO); ca. 7 km from Hwy 1 (at ca. km 193) on road to Pueblo Nuevo, ca. 13°24’N, 86°27’W, 600-700 m, 3 Jul 1977, *W.D. Stevens 2604* (MO, QCA); **Granada**: camino a Charco Muerto, 3 km al E de Casa de Tejas, 100-200 m, 5 Jun 1980, *M. Araquistain & P.P. Moreno 2856* (MEXU, MO); Lake Nicaragua, 24 Dec 1968, *D. Dudey 1101* (B, MO, UC, US); Puerto Asese, small islands in Lago de Nicaragua, 11º54’N, 85º55’W, 30 m, 15 Jan 1981, *W. J. Hahn & J.C. Sandino 420* (MEXU, MO); environs de Grenade, 40 m, Jun 1869, *P. Lévy 31* (C, MO, P); Grenada, 1869, *P. Lévy 1087* (P); Valle de Cutirre, al pie del Volcán Mombacho, 11º50’N, 86º56’W, 400-450 m, 28 Jul 1980, *P.P. Moreno 1480* (MEXU, MO); valle La Cascada, 13º12’N, 86º29’W, 800-900 m, 1 Sep 1980, *P.P. Moreno 1891* (MEXU); camino a Cutirre, “San Agustín”, 85º58’N, 11º55’W, 50-70 m, 16 Sep 1980, *P.P. Moreno 2738* (MEXU, MO); 6 km NW de Pueblo Nuevo, 13º23’N, 86º32’W, 700 m, 26 Sep 1980, *P.P. Moreno 3044* (MEXU, MO); Volcán Mombacho, finca Las Delicias, 11º51’N, 85º57’W, 340-360 m, 11 May 1981, *P.P. Moreno & J. Henrich 8445* (MEXU, MO); Lake Nicaragua, 24 Dec 1968, *C.E. Nichols 1139* (CAS, MO); **León**: sector oeste de la isla Momotombito, 100 m, 19 Aug 1979, *M. Araquistain & P.P. Moreno 69* (MEXU, MO); sector izquierdo de la isla de Momotombito (Lago de Managua), 200 m, 20 Sep 1979, *M. Araquistain 262* (MEXU, MO, VEN); sector E de la isla Momotombito, 150 m, 22 Oct 1979, *M. Araquistain 394* (MEXU, MO); Lake Managua, 31 Dec 1968, *R.B. Hamblett 1538* (MO, SI, US); Laguna de Monte Galán, 12º26’N, 86º34’W, 80-100 m, 28 Aug 1980, *M. Guzmán et al. 667* (MEXU, MO); 1 km S of Punta Diablo, 18 Aug 1976, *J.S. Hall & S.M. Bockus 7802* (MEXU, MO, SI, US); Lake Managua, Momotombo, León Viejo, 31 Dec 1968, *A.D. Moore 1546* (B, MO, USF); Hda. Las Lajas, a 1 km S de San Juan de Limay, 13º10’N, 86º37’W, 300-500 m, 4 Sep 1980, *P.P. Moreno 2243* (MEXU, MO); 21 km al N de Achuapa, 13º09’N, 86º37’W, 250-300 m, 4 Sep 1980, *P.P. Moreno 2313* (MEXU, MO); El Cacao, entrada a la presa Santa Bárbara, 6 km de la carretera León-San Isidro, 11 Sep 1980, *P.P. Moreno 2481* (MEXU, MO); Soledad de la Cruz, entrada a la presa Santa Bárbara, 12º48’N, 86º16’W, 400-480 m, 11 Sep 1980, *P.P. Moreno 2544* (MEXU, MO); NE del Volcán Mombacho, empalme Santa Isabel-Cutirre, 11º51’N, 86º57’W, 340-360 m, 16 Sep 1980, *P.P. Moreno 2636* (MEXU, MO); NE del Volcán Mombacho, Hda. Las Delicias, de 8-9 km sobre la carretera a Cutirre, 11º51’N, 85º58’W, 350-400 m, 16 Sep 1980, *P.P. Moreno 2685* (MEXU, MO); 1 km S of Punta Diablo, Volcán Momotombo, 18 Aug 1976, *E.B. Nelson 7809* (B, CAS, MO); along Río Sinecapa, 12°36’N, 86°28’W, 55 m, 15 Sep 1977, *W.D. Stevens 3854* (MO, QCA); W of Quebrada Las Ruedas, NW of El Transito, 12º05’N, 86º43’W, 15-30 m, 16 Oct 1977, *W.D. Stevens 4688* (MEXU, MO, QCA); **Managua**: Reparto Belmonte, NO de Managua, 49 m, 10 Aug 1979, *M. Araquistain 5* (MEXU, MO, QCA, U); Laguna de Apoyo, el Madroñal, 11º54’N,

86º3’W, 80 m, 15 Jan 1980, *M. Araquistain & P.P. Moreno 505* (MEXU); along Route 10 between Managua and Masachapa, 2 mi S of junction with Route 12, 100 m, 18 Aug 1977, *T.B. Croat 43653* (MO); between Managua and Asososca, 24 Feb 1922, *J.M. Greenman & M.T. Greenman 5629* (MO, UC); costado SW del recinto universitario, 30 Oct 1979, *A. Grijalva 706* (MEXU, MO); costado S de Lomas de Villa Fontana, 86º16’N, 12’6’30’’W, 250 m, 6 Aug 1982, *A. Grijalva 759* (MEXU, MO); Comarca Aduana, al N del Río Aduana, 80-100 m, 21 Jul 1980, *M. Guzmán et al. 396* (MEXU, MO); Tipitapa, sobre la Carretera a Tisma, 12º10’N, 86º03’W, 40 m, 16 Jul 1980, *P.P. Moreno 1180* (MEXU, MO); entrada a la Hacienda San Jacinto, 12º21’N, 86º03’W, 90-100 m, 16 Jul 1980, *P.P. Moreno 1240* (MEXU, MO); en la base de la Cuesta del Cayol, km 56, 12º30’N, 86º04’W, 200-250 m, 25 Jul 1980, *P.P. Moreno 1408* (MEXU, MO); Península de Chiltepe, faldas de los Cerros Cuapes, 12º15’N, 86º20’W, 250-280 m, 15 Aug 1980, *P.P. Moreno 1725* (MEXU, MO); 1.5 km S de a Hda. El Tamagás, 12º16’N, 86º19’W, 100-150 m, 15 Aug 1980, *P.P. Moreno 1791* (MEXU, MO); Laguna de Apoyo, 11º56’N, 86º04’W, 100 m, 24 Oct 1980, *P.P. Moreno 3919* (MEXU, MO); cerro al sur de la casa hacienda Tamagás, 12º16’N, 86º20’W, 120 m, 4 Nov 1980, *P.P. Moreno 4220* (MEXU, MO); carretera a la Laguna de Jiloa, entre km 15 y 16, 12º12’N, 86º21’W, 60-80 m, 4 Nov 1980, *P.P. Moreno 4328* (MEXU, MO); km 36, carretera a Boaco, San Fernando, 12º19’N, 86º03’W, 60-80 m, 19 Oct 1980, *P.P. Moreno 3590* (MEXU); camino a San Francisco, 12º22’N, 86º04’W, 50-60 m, 28 May 1981, *P.P. Moreno & J. Henrich 8793* (MEXU, MO); Tipitapa, 4 Dec 1968, *E. Narvaez 17* (UC); Laguna de Jiloá, lado oeste, 250 m, 18 Nov 1980, *J.C. Sandino 253* (MEXU, MO); El Ventarrón, camino a finca Las Cuchillas, 86º20-21’N, 12º01-02’W, 400-500 m, 20 Jan 1981, *J.C. Sandino 306* (MEXU, MO); km 24 on Hwy 12, 7 km WSW of summit of Sierra de Managua, 12°04’N, 86°26’W, 200 m, 7 July 1977, *W.D. Stevens 2701* (MO, QCA); km 11 on Hwy 2 (Carretera sur), 2 km SSW of Hwy 12 intersection, 12º06’N, 86º19’W, 300 m, 23 Jul 1977, *W.D. Stevens 2888* (MEXU, MO, QCA); ca. 4 km from Hwy 12 on Hwy 10, ner bridge of Río La Aduana, 12º02’N, 86º31’W, 80-100 m, 18 Aug 1977, *W.D. Stevens 3360* (MEXU, MO, QCA); ca, 2.3 km from Hwy 12 on road along ridge of Sierra de Managua, 12º04’N, 86º22’W, 450-500 m, 20 Aug 1977, *W.D. Stevens 3431* (MEXU, MO); along Hwy 1, ca. km 58, ca. 8 km N of Las Maderas, 11°29’N, 86°03’W, 340 m, 16 Oct 1979, *W.D. Stevens et al. 14899* (MEXU, MO, VEN); **Masaya**: Masatepe del Tanque, 2 km al NE, 300-400 m, 18 Oct 1980, *M. Guzmán et al. 1307* (MEXU, MO); **Matagalpa**: Route 1, Sebaco, along bank of Río Grande, 23 Dec 1969, 200-500 m, 23 Dec 1969, *J.T. Atwood 2488* (MEXU, MO); Caserío Puertas Viejas, 6 km E sobre la carretera a S. José de los Remates, 15 Jun 1980, *P.P. Moreno 733* (MEXU, MO); 24 km al E, sobre la carretera a S. José de los Remates, 15 Jun 1980, *P.P. Moreno 763* (MEXU, MO); 1 km N de la Majada en la ribera E de la Quebrada la Majada, 400 m, 15 Jun 1980, *P.P. Moreno 788* (MEXU, MO); **Nueva Segovia**: Quebrada El Lizupo, a 3 km W de Mozonte, 13º40’N, 86º25’W, ca. 700 m, 1 Aug 1980, *P.P. Moreno 1605* (MEXU, MO); ca. km 233.5, ca. 6.2 km N of edge of Ocotal, Quebrada El Noncital, 13°41’N, 86°24’W, 700-760 m, 7 Aug 1977, *W.D. Stevens 3013* (MEXU, MO, QCA, VEN); **Río San Juan**: San Carlos, en la Isla D. Guevara, archipiélago de Solentiname, Lago Nicaragua, 31 m, 18 Sep 1982, *E. Martínez S. 2262* (MEXU); **Rivas**: entre Río Escamequita y Río Escameca, 60 m, 8 Sep 1979, *M. Araquistain 278* (MEXU, MO, QCA); Hacienda La Flor, 18 km al sur de San Juan del Sur, 10 m, 10 Feb 1980, *M. Araquistain & P.P. Moreno 1276* (MEXU, MO, U); Isla de Ometepe, 11º29’N, 85º29’W, 40-55 m, 14 Sep 1983, *P.P. Moreno 22007* (MEXU, MO); above Balgüe on facing slopes of Volcán Maderas, Isla de Ometepe, 11º28’N, 85º31’W,

600 m, 14 Sep 1983, *M. Nee & W.R. Téllez 28018* (MEXU, MO); vicinity of Moyogalpa, Isla de Ometepe, 11º32'N, 86º41'W, 50 m, 16 Sep 1983, *M. Nee et al. 28175* (MEXU, MO); beach of Lago de Nicaragua, 11º28'N, 85º47'W, 30-50 m, 17 Sep 1983, *M. Nee 28210* (MEXU, MO); along road SE from San Juan del Sur, Quebrada El Coco and small peak S of quebrada, 11°9'N, 85°47'W, 0-95 m, 11 Sep 1977, *W.D. Stevens 3774* (MO, QCA).

PANAMA. **Bocas del Toro**: region of Almirante, Jan-Mar 1928, *G.P. Cooper 115* (CAS) & *177* (MICH, P); above Altamirante on road to Changuinola, 0-200 m, 3 Apr 1977, *W. D'Arcy 11215* (CORD); Changuinola Valley, 23 Aug 1923, *V.C. Dunlap 178* (CAS, F); out Zigla road at junction of Changuinola and Tuibe Rivers, 18 Apr 1969, *R.L. Lazor et al. 2525* (FSU); Changuinola to 5 miles S at junction of Ríos Changuinola & Terebe, 100-200 ft, 17-19 Dec 1966, *W.H. Lewis et al. 839* (GH, K, MO, US, UC, US); Isla Colon, NE side of island, 30 Jan 1989, *P.M. Petersen 6454* (MO, US); 4 km S of Tiger Kwy, 50 m, 24 Feb 1989, *P.M. Peterson & C.R. Annable 7043* (MO, US); Water Valley, 11 Sep 1940, *H. von Wedel 724* (GH, MO); vicinity of Chiriqui Lagoon, 19 Oct 1940, *H. von Wedel 1237* (GH, MO); same locality, 5 Sep 1941, *H. von Wedel 2610* (GH, MO, US); **Canal Zone**: vicinity of Madden Dam, 8 Oct 1939, *P.H. Allen 2006* (GH, US); Causeway, Fort Amador, 18 Aug 1962, *J.D. Dwyer 2559* (US); vicinity of Gatuncillo, 26 Feb 1923, *C.V. Piper 5648* (US); Mamei Hill, 20-90 m, 6 Jul 1911, *H. Pittier 3810* (GH); Las Cascadas Plantation, near Summit, 2 Dec 1923, *P.C. Standley 25825* (US); same locality, 4 May 1924, *P.C. Standley 29640* (US); Darién Station, 19 Jan 1924, *P.C. Standley 31543* (US); 1 mi. N Summit Garden, 30 Oct 1965, *E.L. Tyson & K. Blum 1946* (FSU); **Coclé**: 10 mi. E of Nata at Rio Grande, 4 Jan 1969, *E.L. Tyson 5214* (FSU); **Colón**: small tributary of Quebrada Ancha, 4 km E of Buena vista, 3 km N of Cement plant , 80 m, 4 Sep 1973, *M. Nee 6789* (CORD, G, MO, P); Quebrada Bonita, 2 km NW of Salamanca, near bridge on road to Rojas, 13 km NE of Buena Vista, 90 m, 15 Sep 1973, *M. Nee 6989* (CORD, MO, US, VEN) & *6990* (CORD); **Chiriqui:** vicinity of Puerto Armuelles, 0-75 m, 28-31 Jul 1940, *R.E. Woodson Jr. & R.W. Schery 840* (GH, US); **Del Darién**: Rio Pirre, 2-5 mi. above El Real,23 Jun 1962, *J.A. Duke 5080* (GH, MO); 5 mi. W of Yaviza, 4 Nov 1982, *R.J. Schmalzel 1211* (MEXU, MO); vicinity of El Real, Río Tuira, 2 Jul 195, *W.L. Stern et al. 798* (G, LE, MO, UC, US); Boca de Cupe, 13 Apr 1908, *R.S. Williams 715* (US); **Los Santos**, vicinity of headwaters of Río Pedregal, 25 miles SW of Tomosí, 2500-3000 ft, 7 Dec 1967, *W.H. Lewis et al. 2939* (COL, UC, DUKE, K, MO); Guayabo, a place several miles west of Tonosí, 1 Mar 1963, *W.L. Stern 1901* (US); **Panama:** near Jenine, Rio Canita, 24 Sep 1961, *J.A. Duke 3815* (GH, MO, US); trail from Río Espavé toward Río Agua Clara, 23 Jan 1972, *A.H. Gentry 3761* (USF); Tocumen Marsh, SE of Airport to O. Torrijos H., 9º05'N, 78º27'W, 7 Jul 1982, *S. Knapp 5946* (MEXU, MO, QCA); Las Sabanas, 4 Dec 1923, *P.C. Standley 25887* (US); along Corazal, road near Panamá, *P. Standley 26862* (US); Río Tecumen, 3 Jan 1924, *P.C. Standley 29336* (US); Juan Díaz, 11 Jan 1924, *P.C. Standley 30539* (US); between Las Sabanas and Matías Hernández, 21 Jan 1924, *P.C. Standley 31921* (US); Saboga Island near village, Perlas Islands, 22 Jun 1969, *E.L. Tyson 5588* (FSU); **San Blas**: Ailigandi, ca. 3 mi inland, near waterfall, 7 Oct 1978, *W. D'Arcy & B. Hammel 12252* (MEXU, MO); Puerto Ovaldía, 0-50 m, Aug 1911, *H. Pittier 4299* (GH, US); by the rio Dian on the trail from Ailigandí, 0-100 m, 19 Jan 1974, *R.H. Warner 215* (F); **Veraguas:** Coclé and Herrera provinces, gallery forest along the Río Santa María near bridge of Pan American Hgw, 16 km SW of Agua Dulce, 8º10'N, 80º41'W, 0-50 m, 11 Feb

1982, *S. Knapp et al. 3342* (MEXU, MO, QCA), 3.5 miles E of Cañazas checkpoint, 8º52'N, 78º15'W, 50-100 m, 28 Feb 1982, *S. Knapp & J. Mallet 3899* (MEXU, MO, QCA).

PERU. Amazonas: Condorcanqui, 1 km S of Chavez Valdivida, garden, 31 Jul 1977, *B. Berlin 2016* (MO); Bongará, 21 km N of P. Ruiz on road between Moyobamba and Bagua, 5º52'S, 77º56'W, 1820 m, 15 Apr 1984, *T.B. Croat 58309* (MEXU, MO); Bagua, Cajaruro, cerca Bagua Grande, 14 Oct 1952, *A. Díaz s.n.* (USM); Roca San Lorenzo (Bagua-Ingenio), 700 m, 27 May 1963, *A. López et al. 4252* (HUT); Utcubamba, Yamón, caserío Chiñuña, 511 m, 30 Apr 2015, *W. Mendoza et al. 6570* (USM); Distrito de Aramango-Salinas, 6 Nov 1999, *R.D. Rojas G. 745* (NY); Galilea, 16 Jul 1991, *Salaün 185* (USM); km 289, carretera Chachapoyas-Bagua, 12 Mar 1966, *P.G. Smith s.n.* (USM); along road Bagua Grande and P. Ruiz, 500-1000 m, 10 Mar 1998, *H. van der Werff et al. 14624* (MO, USM); **Ancash**: Prov. Santa, Santa, en el propio pueblo, en jardín de casa, *S. Leiva González & G.E. Barboza 6555* (CORD, HAO); **Cajamarca**: San Ignacio, Granja Quiracas, 5°16'S, 78°46'W, 600-800 m, 30 Jan 1996, *J. Campos de la Cruz & O. Díaz 2293* (MO, USM), Chirinos, entre La Catagua y Tablón, 5º19'S, 78º47'W, 550-650 m, 9 Feb 1996, *J. Campos de la Cruz & O. Díaz 2486* (HUT, MEXU, MO, USM); Chirinos, Las Juntas, 5°22'34''S, 78°46'51''W, 550-650 m, 11 Mar 1998, *J. Campos de la Cruz 4833* (F, HUT, MO, USM); Jaén, Valle Tabacenas, *R. Ferreyra & S. Sánchez 19637* (USM); San Martín del Chinchipe, 1000 m, 5°19'16''S, 78°41'5''W, 15 Sep 1999, *S. Flores et al. 157* (USM); Pucará (on the rio Huancabamba), km 127, between Olmos and Jaén, 950 m, 12 Jan 1964, *P. Hutchinson & J.K. Wright 3541* (LE, M, MO, NY, P, UC, US, USM); Chinchipe, ca. Nambacasa (Distr. San José de Lourdes), 770 m, 7 Jan 1998, *S. Leiva González et al. 2105* (HAO†, HUT, MO); Las Juntas, 5°22'48''S, 78°46'58''W, 450 m, 1 Jun 2000, *R. Rojas et al. 886* (HUT, MO, USM); Fundo Los Arrascue, 6 km west of Pucará, along Quebrada Chaupe, 1020, 19 Jun 1993, *I. Shonle et al. 23* (MO); Jaén, 5°42'46''S, 78°48'39''W, 750 m, *H. van der Werff et al. 15144* (MO, USM); **Cuzco**: Prov. La Convención, Quintalpata, ca. 150 km from Cuzco on the road from Cuzco to Kiteni, 1210-1435 m, 29-30 Dec 1986, *P. Nuñez V. et al. 6780* (NY, USM); **Huánuco**: Leoncio Prado, Tulumayo, on Río Tulumayo, 23 km from Tingo María on highway to Pucallpa, 15 Jan 1950, *H.A. Allard 2265* (US); Inca, Codo de Pozuzo, caserío La Unión, 450 m, 3 Mar 2006, *E. Becerra Gonzáles & J. Perea 1152* (HOXA, HUT, MO, USM); road above Tingo María, 600 m, 1 Jun 1977, *J. Hart 594* (GH); **Junín**: Prov. Satipo, northern Cordillera Vilcabamba, upper Rio Poyeni, 11º33'35''S, 73º38'28''W, 1850-1950 m, 3 Jul 1997, *B. Boyle et al. 5020* (NY); **La Libertad**: Bolivar, Uchumarca, sector Pusac, Santa María, 6°59'18''S, 77°55'09''W, 1472 m, 3 Jul 2009, *J. Gruhn et al. 221* (HUT, MO); Otuzco, caserío de Platanar (al oeste de Salpo), 800 m, *S. Leiva González et al. 834* (HAO†); **Loreto**: Iquitos, Francisco de Orellana, 17 Oct 1987, *F. Ayala 5786* (MO); Río Yuvineto affluent du Putumayo, territoire des indiens Secoya, 28 Jul 1978, *G. Haxaire 5144* (F, NY) & *5147* (NY); Yurimaguas, cult., 14 Sep 1984, *K. Hormia 2195* (QCNE, USM); Prov. Requena, Yanallpa, Ucayali River, 4º55'S, 73º45'W, 16 Jun 1987, *W. de Jong 53* (MO, NY); Washintsa and vicinity, Río Huasaga, Comunidad Kapawari, 185 m, 16 Jun 1986, *W. H. Lewis et al. 11196* (MO); Río Pastaza, 2°20'S, 76°20'W, 190 m, 5 Dec 1986, *W.H. Lewis et al. 12552* (USM); 12 de Octubre, Río Tigre, 2°20'S, 75°53'W, 255 m, *W.H. Lewis et al. 12906* (USM, MO); Balsapuerto, comunidad nativa de Soledad (grupo étnico Chayahuita), 5°40'18"S, 76°37'28"W, 5 May 2007, *G. Odonne 25* (HOXA, USM); same locality, 3 Dec 2007, *G. Odonne 561 & 563* (HOXA,

USM), 19 May 2008, *G. Odonne 626* (HOXA, USM), 30 May 2008, *G. Odonne 627* & *664* (USM); Río Amazonas, Is. Rondiña, Caño Gamboa, 18 Mar 1977, *T. Plowman et al. 6419* (GH, MO); Río Ampiyacu, Puca Urquillo and vicinity, ca. 3º05'S, 71º55'W, cult., 4 Apr 1977, *T. Plowman et al. 6599* (GH, USM); Rio Ampiyacu, Pebas and vicinity, 3º10'S, 71º49'W, cult., 27 Apr 1977, *T. Plowman et al. 7075* (GH, MO, USM); Palta-Cocha on the upper Rio Nanay, Jul 1929, *L. Williams 1277* (US); Lower Rio Huallaga, 14 Nov 1929, *L. Williams 5224* (F); **Madre de Dios**: Prov. Tambopata, Dist. Las Piedras, Quebrada Gamitana, 16 Jul 2007, *L. Valenzuela 9902* (MO); **San Martín**: Mariscal Cáceres, alrededores de Juanjui, 300-400 m, 17 Aug 1948, *R. Ferreyra 4495* (CORD, USM); San Martín, road from Tarapoto to Juanjui, km 24, 300 m, 5 May 1976, *T. Plowman 6048* (F, USM); Alto Rio Huallaga, Tarapoto, 12 Jul 1929, *L. Williams 5785* (F); Tarapoto, Vaca Pozo, 830 m, 24 Feb 1947, *F. Woytkowski 35161* (CORD, G, MO, UC, USM); **Ucayali**: Purus, Río Curanja, 10º04'S, 71º06'W, 325 m, cult., 25 Oct 1997, *J. Graham & J. Schunke V. 271* (MO, NY); Coronel Portillo, Yarinachocha, camino a Pacacocha, 8º21'S, 74º34'W, 200 m, cult., 16 Jul 1998, *J. Graham & J. Schunke V. 460* (F).

PUERTO RICO. Puerto Rico, 31 Dec 1925, *E.E. Dale s.n.* (MICH); Portorico, Mar 1874, *O. Kuntze s.n.* (NY 01008231); Puerto Rico, *Léman s.n.* (P00409978); Porto-Rico, *A. Plée s.n.* (P); Puerto Rico, in sylvis, May-Jun 1829, *H. Wydler 264 bis* (F); **Adjuntas**: Barrio de Guilarte, along road 131 about 2-1/2 km below forest station, 12 Aug 1966, *W.R. Stimson 3922* (NY); **Aguadilla**: on rocky seashore at Aguadilla, 5 Feb 1900, *A.A. Heller 4526* (MO); **Bayamóm**: Mogote hill next to El Parque de Las Ciencias, 16 Apr 1990, *J.A. Carrasquillo 128* (NY); Bayamón, 11 Jan 1899, *A.A. Heller 98* (NY); Aguadilla, 5 Feb 1900, *A.A. Heller 4526* (NY); Aibonito, Bo. Azomante, 14 Nov 1937, *J.I. Otero J. 284* (MO); Bayamón, 19 May 1959, *R.O. Woodbury s.n.* (NY 01008241); Bayamón, km 15, Jun 1959, *R.O. Woodbury s.n.* (NY 1008239, NY 01008244); **Cabo Rojo**: in montibus calcareis, 31 Jan 1885, *J. Urban 461 b* (GOET, LE, M, S, US); **Caguas**: Caguas, 16 Nov 1899, *G.P. Goll 378* (NY, US); **Coamo**: vicinity of Coamo Springs, 25 Feb 1922, *N.L. Britton 6242* (NY); Coamo Arriba, 31 Dec 1931, *N.L. Britton & E.G. Britton 9848* (NY); **Culebra**: Island of Culebra, 3-12 Mar 1906, *N.L. Britton W.M. Wheeler 231* (NY); **Fajardo**: W of Fajardo, 11 Aug 1981, *A.H. Liogier et al. 32113 & 32122* (NY); Convento Beach near Fajardo, 18 Sep 1968, *R.J. Wagner 1632* (MEXU); **Guánica**: ad Montalba, 5 Feb 1886, *P. Sintenis 3685* (CORD, P); **Guayama**: Cayey-Guayama, 16 Mar 1874, *O. Kuntze 493* (NY); rte 712 ca. 1-3 km from rte 15, 18 Nov 1989, *Taylor 9662* (MO); **Isabela**: along Guajataca gourge, 15 m, 12 Jan 1995, *P. Acevedo Rodríguez et al. 7223* (NY, US); Guayataca Gorge ca 1 mi from the sea, 8 Feb 1971, *W. D'Arcy 5137* (MO); **Lares**: Callejones, 20 Oct 1980, *A.H. Liogier et al. 31084* (NY); **Luquillo**: Río Mar, 23 Jan 1979, *A.H. Liogier et al. 28206* (NY); **Maricao**: Maricao, Nov 1881, *P. Sintenis 461* (US); Maricao, in convalli fluminis, Nov 1884, *J. Urban 461* (GOET, LE); **Patillas**: "Las Tetas" Cayey, 10 Mar 1983, *A.H. Liogier et al. 34006* (NY); **Ponce**: Ponce to Peñuelas, 9 Mar 1913, *N.L. Britton et al. 1785* (NY); between Ponce and Santa Isabel, 9 Feb 1923, *N.L. Britton & E.G. Britton 7344* (NY); **Puerto Rico**: Vieques island, Isabel Segunda to Campo Cielo, 24 Jan 1914, *J.A. Shafer 2361* (CAS, NY); along route 181, eastern Puerto Rico, 9 Sep 1967, *R.J. Wagner 1215* (LE, MO); Vieques, *R.O. Woodbury 1970* (MO); **Salinas**: Camp Santiago, along trail from northern side of Piedras Chiquitas, 18º3'40''N, 66º16'04''W, 240 m, 24 May 2013, *P. Acevedo-Rodríguez & J. Knight 15650* (US); **San Germán**: Inter Amer. Univ., in front of Del Toro, 27 Jun 1966, *W.R. Stimson 3049* (MO,

NY); **San Lorenzo**: Cerro Gregorio (El Jagual), 20 Sep 1983, *A.H. Liogier et al. 34488* (NY); **San Juan**: 3 miles E of Santurce, 3 Feb 1899, *A.A. Heller 227* (NY); Río Piedras, 20 Dec 1914, *J.A. Stevensen 2464* (US); **Yauco**: Bo. Susúa Forest Reserve, trail along Río Loco, 18º05'03''N, 66º54'32''W, 150 m, 23 Mar 1998, *G. Breckon 5574* (US); Bosque Estatal de Susua, along trail and Rio Loco, 18º4'628N, 66º54'326W, 147 m, 9 Feb 2003, *D.K. Christopher et al. 64* (NY).

SABA. Near the Bottom, along the trail from Booby Hill to the Bottom, 18 Mar 1999, *A.S.J. van Proosdij et al. 1057* (NY).

SAINT VINCENT AND THE GRENADINES. **Saint George**: King's Hill, 1-7 Apr 1950, *R.A. Howard 11136* (NY); Dorsetshire Hill, near Kingstown, 4 Apr 1947, *C.V. Morton 4713* (US).

SURINAM. Commewijne: Plantage Bent's Hoop, 5º56.28'N, 54º54.20'W, 15 Mar 2012, *T.J. Heilbron & D. Sanredjo 6* (NY, U); **Sipaliwini**: inter Coppename fluv. Ostium et Coronie oppidum, 16 Dec 1948, *J. Lanjouw & J.C. Lindeman 1462* (CORD, U); **Marowijne/Sipaliwini**: Litani River, old Indian camp, 1 Aug 1937, *H. Rombouts 735* (B, NY).

TRINIDAD AND TOBAGO. Tobago: Charlotteville, 17 Feb 1980, *Y.S. Baksh 10* (NY); in Studley Park, 8 Jul 1913, *W.E. Broadway 4563* (Z); Roxborough, Bloody Bay road, 700 ft, 12 Aug 1958, *J.W. Purseglove 6314* (US).

TURKS AND CAICOS ISLANDS. **North Caicos**: along road north of Kew to Witby, 30 Aug 1974, *D.S. Correll 43340* (NY).

UNITED STATES OF AMERICA. **Arizona**: Pima County, Baboquivari Mts., Fresnal Canyon, 9 Sep 1931, *M.F. Gilman 142* (CAS, NY); Baboquivari Mts., Monoto Canyon, 31 Oct 1945, *L.N. Goodding 405-45* (NY); western side of Mt. Baboquivari, 4000 ft, 6 Oct 1944, *F.W. Gould et al. 2687* (CAS, GH, NY, UC, US); Baboquivari Mts., 19 Sep 1931, *M.E. Jones s.n.* (UC); Maricopa County, Tempe, 614 W 11th St, in old flood-irrigated neighborhood, 7 Nov 1991, *E. Joyal 1931* (NY);

Florida: Lee County, Cayo Costa State Park, on trail to Quarantine Rocks, 26º 41'58"N, 82º14'52"W, 4 Mar 2003, *A. Bishop & B. Holst CC0048* (USF); same locality, 10 Jun 2003, *A. Bishop & B. Holst CC0053* (USF); Volusia County, S side of Turtle Mound, ca. 5 mi. SE of New Smyrna Beach, 26 Dec 1965, *D. Blake s.n.* (USF 95108); Cayo Costa State Park, North Captiva Island, 18 Jan 2003, *S.W. Braem NC0026* (USF); Miami-Dade County, Elliot Key, 1 Apr 1904, *N.L. Britton 379* (NY); Monroe County, Boot Key, 23 Jun 1969, *J. Brockmann 103* (FSU); Fort Myers Beach, Matanzas Pass, near S end of Presgrove, 20 Oct 1984, *E. Brown s.n.* (USF 184100); Sanibel Island, Woodring Point, 27 Nov 1970, *W.C. Brumbach 7357* (GH); same locality, 27 Nov 1971, *W.C. Brumbach 7750* (GH, NY); upper Captiva Island, 30 Nov 1971, *W.C. Brumbach 7754* (GH, US); Point Ybel, eastern Sanibel Is., 17 Dec 1975, *W.C. Brumbach 8915* (NY); Upper Captiva, 30 Dec 1975, *W.C. Brumbach 8917* (NY); Medina County, Rattlesnake cave, near Ney Bat cave, 21 Jul 1950, *P.T. Bryant & G. Bryant 94* (UC); northern Key Largo, 17 Nov 1962, *A.F. Clewell & F.C.*

Craighead 543 (FSU); Citrus County, near Hell's Gate, mouth of Homosassa River, 3 Jul 1936, *D.S. Correll 5814* (FSU); Bay County, Shell islands at the mouth of St. John's River, Aug-Oct [sine year], *A.H. Curtiss 2205* (CAS, CORD, GH, LE, M, NY, US); Key Largo, 5 Jul 1895, *A.H. Curtiss 5458* (GH, LE, NY, UC, US); Lake County, S edge of Lake Dora, 12 May 1979, *R.F. Daubenmire & J. Daubenmire s.n.* (USF 180254); Dade County, along road 205 about 8 mi. north-east of Flamingo, 4 Jan 1941, *C.C. Deam 60321* (NY); Hernando County, left bank of Withlacoochee, 22 Mar 1883, *J. Donnell Smith s.n.* (NY 01422134, US 02826679); Tallahassee hammock, Key Largo, 23 Nov 1903, *A.A. Eaton 421* (GH, NY); Brevard County, Hummock, 29 Oct 1902, *A. Fredholm 5523* (CORD, GH, SI); Kew West, Aug 1877, *A.P. Garber s.n.* (NY 01422137); Cayo Costa Island, Center portion of island at Old Ware Mound, 24 Nov 1992, *B.F. Hansen & R.P. Wunderlin 12049* (USF); Collier County, Gooland, 9 Jun 1960, *C.B. Heiser 4675e* (FSU); Jackson County, Marianna, 3 Aug 1899, *Herb. Biltmore 10175* (UC); Marco, Jul-Aug 1900, *A.S. Hitchcock 242* (GH, US 02826684, NY); Mound Key State Archaeological Site, 12 Jan 2007, *E. Jensen MK0040* (USF); Levy County, Central part of Seahorse Key, an island in the Cedar Keys National Wildlife Refuge, 19 Oct 2012, *W.S. Judd et al. 8388* (FLAS, USF); Big Pine Key, southeast Hammock, 7 Jan 1953, *E.P. Killip 42473* (B, UC); E of Pineola, 3 Dec 1955, *R. Kral 1884* (FSU); Pineaola, Withlacoochee River, 21 Oct 1977, *R. Kral 61242* (NY); Withlacoochee River, E of Pineola, 21 Oct 1962, *O.K. Lakela et al. 25447* (USF); ca. 9 mi. S of New Smyrna Beach, Mosquito Lagoon, 4 Nov 1962, *O.K. Lakela 25539* (FSU, USF); Marco Island, Gulf of Mexico, 23 Oct 1964, *O.K. Lakela 27780A* (USF); Chokoloskee, on the Gulf of Mexico, 20 Aug 1965, *O.K. Lakela & D. Laker 29097* (NY, USF); Chokoloskee, W of FL 29, off road terminus, 31 Aug 1966, *O.K. Lakela & F. Almeda 30154* (USF); Duval County, Fort George Island, 29 Sep 1898, *L.H. Lighthipe 738* (NY); Flager County, Washington Oaks State Park, W side, 6 Jul 1972, *R.W. Long s.n.* (USF 110899); Big Pine Key, 7 Aug 1966, *R.W. Long et al. 2231* (FSU, USF); Turtle Mound Monument, facing Indian River, Shell Mound Hammock, S of New Smyrna Beach, 9 Oct 1966, *R.W. Long et al. 2271* (USF); Bear Creek Road, N of Flamingo, 29 Dec 1973, *R.W. Long & R.P. Wunderlin 4079* (USF); Palm Beach County, Palm Beach Junior College, Singer Island, 9 mi. S of Lost Tree Village, 26 Oct 1969, *W.L. McCart 11158* (USF); Brickell Hammock, Miami, 4 Mar 1930, *H.N. Moldenke 727* (NY); N Captiva Island, 15 Dec 1978, *S. Morrill & J. Harvey 154* (USF); vicinity of Eustis, 1-15 Jun 1894, *G.V. Nash 985* (GH, LE, NY, U, UC); North Key largo, 17 Dec 1974, *J.E. Poppleton & A.G. Shuey s.n.* (FSU 00005156, USF 116312); Turtle Mound, 7 Aug 1976, *J.E. Poppleton 933* (USF); Key West, 1842-1849, *F. Rugel 87* (GH); Manatee County, vicinity of Manatee, 22 Oct 1898, *J.H.Simpson 86* (F, GH, US); between Cutler and Longview Camp, 9-12 Nov 1903, *J.K. Small & J.J. Carter 793* (NY); between Cocoanut Grove and Cutler, 8 May 1904, *J.K. Small & P. Wilson 1773* (NY); Miami, 5-21 May 1904, *J.K. Small & P. Wilson 1892* (NY); near the beach, 19 Nov 1904, *J.K. Small 2121* (NY); pinelands between Miami and Kendall Station, 5 Nov 1906, *J.K. Small & J.J. Carter 2673* (NY); Caldwell's Hammock near Silver Palm School, 8 Nov 1906, *J.K. Small & J.J. Carter 2676* (NY); Lower Matecumbe Key, Aug 1907, *J.K. Small 2772* (NY); Hammocks between Miami and Cocoanut Grove, 21 Nov 1912, *J.K. Small 3867* (NY); same locality, 26 Nov-20 Dec 1913, *J.K. Small 4604* (GH, NY, US); Key West, 30 Nov-3 Dec 1913, *J.K. Small & E.W. Small 4864* (NY); eastern end of Key West, 16 Dec 1913, *J.K. Small & G.K. Small 4983* (NY); Old Rhodes Key, 10 Mar 1915, *J.K. Small & C.A. Mosier 5695* (NY); Rachel Key, 6 Feb 1916, *J.K. Small 7468* (NY); Hammocks Cape Sable, Middle Cape, 2 Apr 1916, *J.K. Small*

7686 (NY); Hammock along Indian River, Coco, 9 May 1918, *J.K. Small 8740* (NY); St. Johns/Flagler County, between south Matanzas River and Ocean, 1882, *J.D. Smith 424* (US); Florida Keys, Plantation Key, 21 Mar 1958, *W.L. Stern 263* (GH, US); N of West Palm Beach, 8 Oct 1967, *V.I. Sullivan 1067* (FSU); Sumter County, Cherry Lake, ca. 1 km E of Morse Blvd. and 0.2 km S of CR 466, 28º55'01''N, 81º57'38''W, 17.7 m, 25 Dec 2011, *M.T. Strong & F. Strong 4071* (NY, USF); above Menehee Flats South of Vanderbilt, 26 Nov 1943, *B.C. Tharp & F.A. Barkley 13A136* (CAS, NY, UC); Perico, 5 Jun 1900, *S.M. Tracy 6831* (F, NY, US); 1 mi. E if Wulferd's Point, Sanibel Island, 21 Nov 1961, *D.B. Ward 2836* (FSU); Turtle Mound, ca. 7 mi. S.E. of New Smyrna Beach, 2 Apr 1966, *D.B. Ward 5555* (NY); Seahorse Key, 1 Aug 1958, *E. West et al. s.n.* (USF 41723); Port Inglis (abandoned settlement) on Chambers Island (Sec. 14, T 17S, R 15 E), near mouth of Withlacoochee River, 4 May 1959, *C.E. Wood et al. 9220* (GH, NY); Martin County, Twin Rivers Park, Rocky Point between Seamark Place and Williams Way, near the confluence of St. Lucie River and Indian River Lagoon, 27º9'46"N, 80º10'58"W, 12 Dec 1999, *S.W. Woodmansee et al. 432* (USF); Pineola Fern Grotto, 18 May 1974, *R.P. Wunderlin 5306* (USF); J.N. "Ding" Darling National Wildlife Refuge, Sanibel Island, ca. 2 mi. ESE of Wulfert (Blind Pass), 28 Oct 1978, *R.P. Wunderlin et al. 6246* (USF); **Georgia**: Jackson County, Carr Lake, 2 Jul 1915, *J.A. Drushel 2810* (P); Glynn County, St. Simon's Island, Taylor´s Fish Camp on old Cooper Plantation, 21 Nov 1983, *T. Schoettle s.n.* (GA 066742); **Lousiana**: Plaquemines, Pointe à la Hache, Jul 1879, *A.B. Langlois s.n.* (CAS 106405); Cameron, north of La. 82, W of the Mermentau River, E of Oak Grove, Sec. 6, *R.D. Thomas et al. 87043* (NY, SP); Cameron, 4 Jul 1903, *S.M. Tracy 8650* (GH, NY, US); **New Mexico**: from Western Texas to El Paso, May-Oct 1849, *C. Wright 537* (F, GH, UC, US); **Texas**: Travis County, 13 Aug 1943, *F.A. Barkley 13434* (NY, UC, US);Bexar County, Río de Medina, prope Béjar, Oct. 1828, *J.L. Berlandier 1907* (BM, G, GH, NY, P00409851, P00409855, P00409956); Texas, 1859, *J.L. Berlandier 3045* (NY, P, US); San Antonio, 2 Oct 1900, *B.F. Bush 1205* (NY); Río Hondo, Aug 1913, *H. Chandler 7044* (GH, NY, UC, US, Z); Cameron County, southside of Resaca del Rancho Viejo, 6 airline miles n.e. of Brownsville, 30 Nov 1945, *V.L. Cory 51407* (NY); Kinney County, twenty miles northeast of Brackettville, along the West Nueces River, 22 Aug 1946, *D.S. Correll & V.L. Cory 14104* (NY); Cameron, 4 Oct 1952, *D.S. Correll 14847* (CORD, MEXU, US); Duval County, San Diego, *M.B. Croft 179* (NY, US); Texas, 1835, *T. Drummond 243* (W); Jackson County, Edna, 20 Aug 1920, *J.A. Drushel 4179* (P); Las Palmas Ranch, vicinity of Brownsville, 1-5 Aug 1921, *R.S. Ferris & C.D. Duncan 3188* (CAS, NY); Calhoun County, Port Lavaca, 25 ft, *G.L. Fisher 41153* (CORD); W. French Place San Antonio, 16 Oct 1942, *B. Freeborn et al. 37* (NY); Refugio County, 17 mi E of Refugio, 9 Jul 1977, *P.A. Fryxell 2953* (CTES, NY); Nueces County, Aransas Pass, 1 Oct 1997, *P.A. Fryxell 5085* (CTES, MEXU); near Bracken, 2 Jul 1903, *B.H.A. Groth 40* (GH, NY, US); Colorado River, eastern Texas, 20 May 1872, *E. Hall 495* (NY, US); Brownsville, 10 miles S.E., 17 May 1919, *H.C. Hanson 468* (NY, US); Goliad County, O'Connor Ranch, Fant Ranch, Fant ranchhouse, cult., 26 Jul 1987, *S.R. Hill 18388* (GH, NY, USF); Bexar, Jun 1904, *G. Jermy s.n.* (NY 1422159, NY 1422170, NY 1422160); Williamson County, San Antonio, *G. Jermy 189* (GH); Uvalde County, west of Uvalde, 26 Apr 1931, *M.E. Jones 28471* (UC); Webb County, Laredo, 26 Jul 1882, *G.W. Letterman 96* (US); Pecos County, Comanche Spring, New Braunfels, 1846, *F. Lindheimer 482* (G, GH, K, LE, M, MO, P, UC, US, W); Comanche Spring, New Braunfels, Jul 1850, *F. Lindheimer 1036* (G, LE, M, NY, UC, W);

Comal County, *B. Matthes 139* (GOET, P, W); San Antonio River, 19 Aug 1931, *M.C. Metz 111* (CAS); Starr County, 6.7 mi N of Rio Grande City, 26º30'N, 98º45'W, 60 m, 15 Oct 1990, *J.S. Miller et al. 5792* (NY); Georgetown, 10 Dec 1879, *E. Palmer 931* (GH, LE, P, NY, US); Atascosa County, Pleasanton, 23 Sep 1916, *E. Palmer 10789* (CAS, US); Santa Rosa Valley, *C.C. Parry et al. s.n.* (NY 1422165 pp); Corpus Christi, 1869, *H.W. Ravenel s.n.* (US 02826705); Palm Grove, below Brownsville, 26 Oct 1947, *H.R. Reed 432* (NY); Brownsville, 1922, *R. Runyon 285* (US); Brownsville, known distribution in The Lower Rio Grande Valley, 15 Jul 1934, *R. Runyon 5449* (M); Lavaca River, 30 Aug 1941, *B.C. Tharp 256* (GH, UC); Galveston County, field E of road that goes N from highway 87 to Bob's Place, 8.7 mi. W of Gilchrist, 27 Jul 1961, *A. Traverse 2523* (A); Texas méridional, Oct 1849, *M. Trécul 1126* (P); Austin County, 5 mi NE of Industry on FM 2502, 21 Feb 1998, *B. Wilke 5* (GH).

VENEZUELA. Venezuela, 1892, *F. Geay s.n.* (P 00481561); **Amazonas**: Yanamami village of Ashidowateri, headwaters of the Shani-shani River, 2º4'N, 64º35'W, 225 m, 31 Aug 1991, *B.M. Boom & C. Brewer-Carias 10105* (NY, VEN); **Aragua**: Prope coloniam Tovar, 1854, *A. Fendler 1018* (G, MO); Maracay, en cerro vecino al hotel Maracay, 19 Oct 1969, *A. Krapovickas 15554* (BAA, CORD, CTES, LP, MO); **Bolívar**: 18 km E of La Paragua-Ciudad Piar Road on road to San Pedro, 300 m, 7 Jul 1975, *A.H. Gentry & P.E. Berry 15038* (MO); Parque de la Represa San Pedro, Tumeremo, 7º18'N, 61º27'W, 150 m, 3 Jul 2000, *C. Knab-Vispo et al. 1288* (NY);); R. Torrecilla, near Turbaco, 150-300 m, 7-19 Nov 1926, *E.P. Killip & A.C. Smith 14251* (GH); 20 km east La Paragua, 6°56'N, 63°15'W, 200 m, 23 Jul 1978, *R.L. Liesner et al. 5417* (MO, P, VEN); San Pedro de las Dos Bocas at junction of Río Paragua and Caroni, 7°00'N, 62°54'W, 200 m, 7 Jul 1978, *R.L. Liesner & A.C. González 5735* (CORD, MO); El Palmar, 31 Aug 1957, *E. Medina 939* (VEN); Serranía Maigualdia, cerca de la ribera del Río Caura, alrededores del caserío Jabillal, 7º5'30''N, 64º57'W, 7 Apr 1989, *B. Stergios & L. Delgado 13640* (NY, US); Kon-quén, along Río Aparurén, 480 m, 7 Jul 1953, *J. Steyermark 76030* (F, NY, VEN); E of Miamo, altiplanicie Nuria, 300-500 m, 8 Jan 1961, *J. Steyermark 88191* (NY, VEN); Carretera El Dorado-Santa Elena de Uairén, Pica de Penetración Km 33-35, 1 Sep 1957, *B. Trujillo 3711* (MY); **Carabobo**: alrededores de El Palito, cerca del Puerto Cabello, 24 Nov 1973, *J.H. Hunziker 9032 & 9034* (CORD); alredores de San Esteban, 29 Oct 1952, *L. Schnee 1183* (MY); matorrales arriba de Sabana Grande, 1150 m, Nov 1938, *L. Williams 10597* (F); **Distrito Capital**: vicinity of Macarao, 31 Aug 1924, *H. Pittier 11561* (G, US, VEN); Turmerito, 900-1000 m, May 1934, *H. Pittier 13543* (US); vicinity of Caracas, 4 Nov 1916, *J.N. Rose 21950* (GH, US); Cerro Naiguatá, vecindad de Quebrada Frontina, 5 km al SO de los tanques de electricidad de Caracas (Cocuizal), 900-1100 m, 2 Nov 1963, *J. Steyermark 91845* (US, VEN); San José Park, Caracas, 28 Sep 1946, *C. Vogl 424* (F); **Falcón**: Zamora, Cerro Mampostal, 4 Aug 1977, *A.C. González 993* (MO); Sierra de San Luis, Piedra de Agua, 11°08'N, 69°40'W, 600 m, 20 Jun 1979, *R.L. Liesner et al. 7613* (MO); Sierra de San Luis, Montaña de Paraguariba, 1300 m, 21 Jul 1967, *J. Steyermark 99428* (US); 2-4 km W of Carrizalito, 1200-1400 m, 11°08'N, 69°47'W, 19 Oct 1985, H. *van der Werff & R. Wingfield 7446* (U); **Guárico**: Dist. Infante, Chaguaramas, Finca "Las Babitas", 19 Oct 1980, *B. Trujillo et al. 17177* (MY); **Lara**: sabanas áridas de Barquisimeto, 26 Jan 1946, *T. Tamayo 2618* (VEN); Crespo District, on the road from Duaca to Barquisimeto 7.2 km SW from El Eneal, 21 Jan 1981, *C. Burandt et al. 27V* (UNAH, USF); **Mérida**: regione El Morro ad El Milagro, 2200-2300 m, 14 Feb 1957, *L. Bernardi 6105* (G); Las Gonzales-San

José road, near the village of Las Gonzales, 8°25’N, 71°20’W, 800-1600 m, 9-10 Nov 1990, *L.J. Dorr & L.C. Barnett 7678* (CAS, NY, US); Ejido La Enfadosa, 1550 m, 9 Jul 1968, *S. López-Palacios 2143* (MO, MY); between San Juan and Las Gonzáles, Jan 1950, *G. Marcuzzi s.n.* (CORD 00088594); Estanques Valley, 1000 m, 2 Feb 1928, *H. Pittier 12837* (F, G, GH, M, MO, US, VEN); El Morro, 13 May 1964, *B. Trujillo 6351* (MY); **Miranda**: carretera Carenero-chirimena, 2 km northwest of Carenero, 0-5 m, 22 Nov 1969, *J. Steyermark & G. Bunting 102309* (M, US, VEN); **Monagas**: col. La Paila, May 1853, *I. Holton 13* (G); a lo largo del riachuelo que corre frente a la Cueva del Guácharo, 23 May 1986, *B. Trujillo et al. 20576* (MY); **Norte de Santander**: Aguacaliente, de San Luis (Cúcuta), *M. de Garganta F. s.n.* (F 1294522); de Bochalema a la Hacienda del Talco, camino de Durania, 2 Jun 1944, *M. de Garganta F. 818* (F); **Nueva Esparta**: Macanao, 400 m, *Bro. Gines 2716* (US); **Sucre**: vicinity of Cristobal Colón, 5 Jan-22 Feb 1923, *W.E. Broadway 366* (GH); Península de Araya, Morro de Chacopata (costa oriental), 17 Sep 1982, *G. Colonnello 522* (MY); Isla de Patos, a 5 km al sureste de la punta oriental de la peninsula de Paria, 10º38,3’N, 61º51,8’W, 80-100 m, 29 Nov 1975, *O. Huber 252* (MO); Península Araya, 20 km NW of Cariaco, 10º38’N, 63º40’W, 0-100 m, 17-18 May 1981, *R.L. Liesner & A.C. González 12024* (COL, MO, NY, VEN); Península de Paria, Cerro Patao, al noreste de Puerto de Hierro, cabeceras del Río Oscuro, 10°40’N, 62°04’W, 10-200 m, 21 Mar 2000, *W. Meier et al. 6621* (MY); Isla Margarita, 7 Dec 1901, *O.O. Miller & J.R. Johnston 82* (F); cerca de Caícara de Maturin, 4 Jan 1941, *H. Pittier 14710* (US, VEN); NE de Güiria, Cerro Patao, Península de Paria, 100-300 m, 23 Jul 1962, *J. Steyermark & G. Agostini 91269* (US); **Táchira**: Carretera de San Antonio a Rubio, 1200-1500 m, 22 Nov 1948, *H. García Barriga 13265* (COL, US); **Yarucuy**: PN Yurubí, sector 2.5 km al NO de San Felipe, 10°15’N, 68°45’W, 300 m, 9 Jul 1984, *G. Aymard et al. 2721* (MO, VEN); **Zulia**: Miranda-Bolívar, vía El Consejo de Ciruma-Palito Blanco, en la vecindad de El Toro, a 8 km. al sur de El Consejo, 18 Nov 1979, *G.S. Bunting 8159* (MO); Dtto. Perijá, en km 38 de la carretera Machiques-La Fría, 22 Sep 1982, *N. Galué 122* (MO); Mara, 4 km N of Corpozulia, campamento Carichuano, 11º2’30’’N, 72º16’30’’W, 150-200 m, 1 Jun 1980, *J. Steyermark et al. 123045* (MEXU, MO); along road in pastured valley of Río Cachirí, 10º48’N, 72º18’W, 150 m, *J. Steyermark 123436* (MEXU, MO).

VIRGIN ISLANDS (US). Saint Croix Is.: Jack Bay, hillside above Rustey's Twist, 23 Jul 1970, *W. D’Arcy 4737* (B, FSU, MO, NY); Caledonia Gut, NW corner of island, 24 Feb 1974, *F.R. Fosberg 55377* (US); near summit of Mt. Eagle, 22 Jan 1980, *F.R. Fosberg 59385* (US); St. Croix, *L.C. Richard s.n.* (P 00410142); Signal Hill, 850 ft, 22 Jan 1896, *A.E. Ricksecker 232* (F, MO, NY, UC, US); **Saint John Is.**: Center line road, 27 Mar 1985, *P. Acevedo-Rodríguez et al. 646* (NY); Coral Bay Quarter, Private road off of Centerline Road (ca. 7.5 km from Cruz Bay), 120 m, 1 Sep 1987, *P. Acevedo-Rodríguez & A. Reilly 2122* (NY); Maho Bay, Centerline road intersection with Rt. 107, 310 m, 19 Jan 1988, *P. Acevedo-Rodríguez 2433* (NY); Coral Bay, road to Ajax Peak, 200 m, 14 Feb 1988, *P. Acevedo-Rodríguez & D. Chinea 2653* (NY); Coral Bay Quarter, Bordeaux Mountain Road, 0.1 km from Center Line Road, 320 m, 20 Aug 1992, *P. Acevedo-Rodríguez et al. 5092* (NY, US); Bethany, 5-7 Feb 1913, *N.L. Britton 350* (NY, US); St. John, mountain, 10-14 Jan 1921, *C.F. Morrow 10 & 15* (US); **Saint Thomas Is**.: Raphune, 18º20’11’’N, 64º5-4’34’’W, 31 Aug 2000, *P. Acevedo-Rodríguez 11344* (US); St. Thomas, *Anonymous 130* (P); St. Thomas, Aug 1881, *H.F.A. von Eggers 322* (LE, W) & *322 b* (M, P); St. Thomas, Apr 1887, *H.F.A. von Eggers 583* (P); St. Thomas, [probably 1840], *E. von*

Friedrichsthal 310 (W); St. Thomas, 26 Feb 1874, *O. Kuntze s.n.* (US, NY 01284132) St. Thomas, *A. Riedlé 1798* (P).

Cultivated

ARGENTINA. Córdoba: Capital, cult. Invernadero IMBIV (UNC), semillas de NMCA 10983, 1 May 2021, *C. Carrizo García 102* (CORD); cult. invernadero IMBIV-Universidad Nacional de Córdoba (UNC), semillas enviadas por P. Bosland, University of New Mexico, 11 Mar 2008, *M. Scaldaferro 9* (CORD); cult. invernadero UNC, semillas enviadas por Llatas Quiroz, de Perú, 25 Oct 2013, *M. Scaldaferro 41* (CORD).

COLOMBIA. Antioquia: Jardín Botánico J. A. Uribe, cult., 15 Nov 1974, *L. Atehostúa G. s.n.* (JAU 001262).

ENGLAND. **Berkshire**: Reading, grown in greenhouse Reading University, seeds from R. Orellana, Tabasco, México, Mar 1973, *B. Pickersgill Cb 4* (Heiser 3702a) (COL);

MEXICO. **Campeche**: Jardín Botánico de Zoh-Laguna, a 10 km al N de Xpujil, 18º35'N, 89º25'W, 200 m, 22 Jul 1996, *P. Alvaro M. 397* (MEXU); **Yucatán**: Jardín Botánico Balancanche, cult., 28 Aug 1989, *S. Escalante 627* (MEXU).

PERU. **Lima**: Lima, cult. en jardín, 15 Feb 1995, *A.T. Hunziker 25481* (CORD); Museo Historia Natural "J. Prado", cult., 2 Mar 1976, *G. Vilcapoma 284* (USM) & 25 Feb 1984, *G. Vilcapoma 327* (USM).

UNITED STATES OF AMERICA. California: grown in Davis, seeds from Mexico, locality unknown, leg. A. N. Prater through Gentry Inc., 1953, *P.G. Smith (Davis Ac. 635)* (UC); **Indiana**: cult. in Indiana University (IU) greenhouse, seeds P. Smith Ac. 1534 from Paxantla (Veracruz, Mexico), 1955, *C.B. Heiser s.n.* (US 02826915); Bloomington, cult.,1949, *C.B. Heiser C19* (CORD); grown in IU greenhouse from seed collected by T. Pizzalto at New Orleans, Lousiana, seed from H. Eshbaugh, 1976, *C.B. Heiser E 1130* (UC); **Missouri**: Saint Louis, Horticulture, Missouri Botanical Garden, Nov 1936, *D. Fairburn s.n.* (UC); **Texas**, Cameron County, Brownsville, Texas 2535 Hipp Ave., 17 Oct 1970, *A. Chávez Jr. s.n.* (GH 01145454).

2. Capsicum baccatum L. var. baccatum

ARGENTINA. Chaco: Gral. José de San Martín, Estancia Varela, camino a Zapallar, ubicado a orillas del Río Guaycurú, 17 Feb 1938, *A.G. Schulz s.n.* (CORD); same locality, *A.G. Schulz 2028* (CTES); Colonia Benítez, Dic 1932, *A.G. Schulz 2050* (CTES); Colonia Benítez, Feb 1935, *A.G. Schulz 2103* (CORD, CTES, F); Colonia Benítez, Dec 1947, *A.G. Schulz 6714* (BAB, CTES, MO); Colonia Benítez, Feb 1951, *A.G. Schulz 8049* (BAB, CTES); Fontana, San Fernando, 19 Apr 1967, *A.G. Schulz 15929* (CTES, F); Dept. Primero de Mayo, Colonia Benítez, 12 Mar 1945, *A.T. Hunziker 7343* (CORD); Colonia Margarita Belén, Guaycurú, 30 Nov 1908, *T. Stuckert 19345* (CORD); **Corrientes**: Capital, 19 May 1972, *B. Benítez & Anzotegui 76* (CTES); Ituzaingó, ruta 38 y Río Aguapey, 25 Feb 1986, *S. Cáceres et al. 343* (CTES); Berón de Astrada, Puerto Yahapé, 14 Dec 1945, *T.S.*

Ibarrola 3911 (LIL); Ita-Ibaté, 9 Jan 1946, *T.S. Ibarrola 4051* (BM, LIL, F); Santa Tecla, 14 Mar 1946, *T.S. Ibarrola 4281* (LIL); Predio Santo Domingo, Mogote E, 19 Mar 2008, *H.A. Keller et al. 5232* (CTES); General Paz, 1 Jan 1966, *A. Krapovickas & C. Cristóbal 11848* (CTES, UC); Rincón Ombú Chico, 3-5 Jul 1974, *A. Krapovickas et al. 25551* (CTES); Estancia Santa Rita, 27°3'S, 56°4'W, 3 Mar 1987, *A. Krapovickas et al. 41070* (CTES, NY, SI); Paso de los Libres, Yapeyú, 1 Feb 1949, *R. Martínez Crovetto 5581 & 5589* (BAB); orillas del Río Paraná, Dec 1976, *R. Martínez Crovetto & A. Schinini 10857* (CTES); ciudad de Corrientes, Oct 1977, *R. Martínez Crovetto 11125* (CTES); San Cosme, near San Cosme, 21 Nov 1959, *T.M. Pedersen 5315* (CTES, E, GH, LP, P, S, US); Mburucuyá, Ea. Santa Teresa, 6 Jan 1966, *T.M. Pedersen 7712* (MO, NY); Rincón Ombú Chico, 25 Apr 1975, *A. Schinini et al. 11282* (CTES); Río Paraná y arroyo San Juan, 25 May 1975, *A. Schinini & C.L. Quarín 11542* (CTES; LIL, MO); 46 km W de Itá Ibaté, Valencia, 15 Jan 1977, *A. Schinini 14061* (CTES); 5 Km E de Guaviraví, costa del Río Uruguay, 13 Feb 1979, *A. Schinini et al. 17024* (CTES, NY); en cultivo de granja, camino a Santa Ana, Laguna Pampin, 25 Sep 2004, *A. Schinini 36578* (CTES); 17 km NW de San Carlos, Ea. Rincón Chico, 5 Mar 1985, *S. Tressens et al. 3116* (CTES, MO); same locality, 14 Feb 1991, *S. Tressens et al. 3873* (CTES, GH); Santo Tomé, Ea. Bertrán (Infrán Cué), 23 Km SW de Virasoro, 7 Apr 1992, *S. Tressens et al. 3991* (CTES, GH, LIL); **Jujuy**: Santa Bárbara, camino al Cedral, 24º14´S, 64º13´W, 910 m, 17 Dec 1998, *O. Ahumada & A. Castellón 9065* (CORD, SI); Ledesma**,** Finca Yuto, sobre RP nº 82 (entrada sur a Yuto), a 1,5 km al E de RN nº34, 21 Mar 2005, *G.E. Barboza et al. 1274* (CORD, MA); PN Calilegua, rumbo a Abra de las Cañas, 6 Apr 2007, *G.E. Barboza et al. 1921 bis* (CORD); entre Santa Clara y Abra de Los Morteros, 18 Mar 1973, A.*L. Cabrera et al. 23364* (LP, SI); Calilegua, camino a los Cafetales, 11 Feb 1980, *A.L. Cabrera et al. 31487* (MO, SI); San Salvador de Jujuy, terrenos baldíos a orillas del ferrocarril, 24º10’58’’S, 65º18’20’’W, 17 Jan 2016, *F. Chiarini 1223* (CORD); Dique las Maderas, 1189 m, 24 Apr 2013, *A.A. Cocucci et al. 5262 & 5263* (CORD); Mesada de las Colmenas, al sud de Río Jordán, 17 Dec 1962, *H.A. Fabris 3454* (BAB, LP); entre Río de Aguas Negras y Calilegua, 580-680 m, 22 Feb 1994, *J.H. Hunziker et al. 12745* (MO, SI); Perico, 1 Mar 1911, *P. Jörgensen s.n.* (BAB 34923); RP 37, 2m del desvío de la RP 1, camino a Vinalito, 23º 43´S, 64º30´W, 540 m, 21 Feb 1998, *O. Morrone et al. 2884* (CORD, SI); just east of entrance to Parque Nacional Calilegua, 8 km W of Hwy. 34 at Calilegua, 23°46'S 064°51'W, 615 m, 14 Apr 2000, *M. Nee & L. Bohs 50775* (MO); Alto La Viña, ruta 56, km al NE de Jujuy, 1250-1300 m, 29 Dec 1989, *L. Novara & S. Bruno 9295* (CORD, G, MCNS, S); Dique Las Maderas, 1300 m, *M.V. Romero 1, 2 & 3* (CORD); Saucelito, 15-17 Feb 1906, *C. Spegazzini s.n.* (LP 18574); Palpalá, camino a Capillitas, 25 Feb 1964, *M. Villa Carenzo & P.R. Legname 3846* (LIL); ruta de Santa Clara a Abra de los Morteros, 1050 m, 28 Feb 1997, *F.O. Zuloaga et al. 6377* (MO, SI); **Formosa**: Pirané, RN 81, 20 km al oeste de la entrada a Pirané, 21 Jul 1986, *M.M. Cerana 823* (CORD); Embocadura Los Pilagás, Río Pilagá, 27 Nov 1900, *E. Kermes 500* (BAB); Formosa, 19 Jun 1975, *A. Krapovickas & A. Schinini 28514* (CTES, G); **Misiones**: Candelaria, en poblado de Candelaria, entrando por la Avda. principal (R. González de Santa Cruz), al fin del asfalto, 7 Dec 2002, *G.E. Barboza et al. 415* (CORD, CTES, SI); Oberá, Arroyo Acaraguá por ruta nº 103, 400 m, 27°30'18"S, 54°53'4"W, 8 Dec 2002, *G.E. Barboza et al. 458* (CORD); Candelaria, en poblado de Candelaria, 28 Mar 2004, *G.E. Barboza et al. 927* (BM, CORD, MO); Capital, Posadas, unas dos cuadras antes de llegar a la costanera, cultivado en cantero, 7 Oct 2011, *G.E. Barboza 3055* (CORD, SI); Parque Nacional Iguazú, CIES, 20 Dec 1996, *G.*

Bernardello 932 (CORD); Garupá, sobre RN 12, 3 Jan 1995, *A. Cardozo et al. 231* (CORD, CTES); camino al Teyu-Cuaré, 27°9'35"S, 55°20'18"W, 17 Apr 2004, *J. Daviña & A.I. Honfi 593* (SI); Posadas, 16 Dec 1907, *E. Ekman 806* (S, US); Puerto Bemberg, 7 Feb 1946, *A.T. Hunziker 7336, 7337 & 7338* (leg. O. Heritier) (CORD); Eldorado, acceso a puerto Yarará, 26 Jan 2008, *H.A. Keller & M. Franco 4940* (CTES); acceso al club del río, 27°16'40,7''S, 55°34'1,3''W, 28 Feb 2010, *H.A. Keller 8340* (CTES); Garupá, próximo al monumento al Indio A. Guacurarí, 17 Feb 1978, *P.R. Legname et al. 5901* (CTES, LIL); Jacutinga, Mar 1965, *R. Martínez Crovetto 100* (CTES); Posadas, 16 Dec 1944, *T. Meyer s.n.* (GH) & 5976 (LIL); Puerto Iguazú, 15 Mar 1944, *T. Meyer 5477* (LIL, GH); Eldorado, 10 Mar 1944, *T. Meyer 6790* (LIL); Puerto Iguazú, 5 Feb 1947, *T. Meyer 11918* (LIL); Puerto Rico-San Martín, 205 m, 28 Mar 1951, *J.E. Montes 15164* (CORD, LIL, SI); Cainguás, Capiovy, 12 Apr 1951, *J.E. Montes 15188* (BAA, CTES, LIL); Monte Carlo, 180 m, 2 May 1951, *J.E. Montes 15202* (CORD, LIL, SI); Teyucuaré, 15 Mar 1952, *J.E. Montes 15612* (CTES, LIL) & 18 Mar 1952, *J.E. Montes 15633* (LIL); Parque Provincial de Faxinal**,** 27°37'57"S, 55°43'32"W, 15 Jan 2007, *J. Paula-Souza 7254* (ESA, CORD, SI); Alto Paraná, bei Piray, Sep 1901, *Ricinel 48* (Herb. T. Stuckert 11414) (CORD); Posadas, Barrio El Laurel, 19 Jun 1998, *M. Rodríguez 168* (CORD, SI); San Ignacio, Guaraní, al oeste del pueblo por el camino hacia Río Uruguay, 29º23'58''S, 56º47'04''W, 62 m, 20 Apr 2014, *M. Scaldaferro 50* (CORD); cerca de Puerto Nuevo, 16 Feb 1948, *A.G. Schulz 7155* (CTES); Avellaneda, 9 Apr 1949, *E. Schwindt 1548* (LIL); Posadas, 16 Jan 1907, *C. Spegazzini s.n.* (BAB 20526); Guaraní, Fracrán, 20 Feb 1907, *S. Spegazzini s.n.* (SI); Santa Ana, alrededores de la curtiembre Santa Ana, próximo al puerto, 14 Jun 1988, *R. Subils & E. Moscone 4273* (CORD); Puerto Libertad, propiedad Bemberg, Ayo. Guatambú, 125 m, 5 Dec 1993, *R.O. Vanni et al. 3289* (CTES, GH, LIL, MO, SPF); **Salta**: Capital, Sierra de Vélez, cerro de la ermita, camino hacia la capilla de la Virgen de Schoenstadt, 1350 m, 14 Apr 2006, *G.E. Barboza 1805 & 1807* (CORD); Rosario de Lerma, Barrio El Tránsito, por el camino viejo que lleva a Campo Quijano, 1400 m, Feb 2010, *G.E. Barboza 2431 bis* (BM, CORD, NY); ascenso por el cerro detrás de Barrio Tres Cerritos, desde el Santuario de la Comunidad de Schöenstatt hacia la cima del cerro donde se encuentra el Santuario de Virgen del Corazón de Jesús, 24º45'40''S, 65º22'57.8''W, ca. 1500 m, 30 Dec 2018, *G.E. Barboza 5038* (CORD, SI); entre La Caldera y km 1614, 1264 m, 24 Apr 2013, *A.A. Cocucci et al. 5258* (CORD); Aguas Blancas, Estación de Aforo, 20 Jan 2014, *A.A. Cocucci & A. Sérsic 5469b* (CORD); Iruya/Orán, entre El Tabacal e Isla de las Cañas, 640 m,16 Jan 2014, *A.A. Cocucci & A. Sérsic 5471* (CORD); General San Martín, 500 m al NW por el camino a Acambuco, desde el cruce de la RN 34 cerca de Yacuy, 500 m, 9 Mar 2001, *R. Fortunato et al. 7013* (BAB); El Oculto, puesto del Sr. Jaime Laime a 500 m del Río Anta Muerta, 12 Mar 2001, *R. Fortunato et al. 7127 & 7128* (BAB); El Oculto, La Bambú, cultivado en el huerto familiar, 12 Mar 2001, *R. Fortunato et al. 7129* (BAB); Pje. El Oculto, finca Madenor S.A., a 20 km al W de la confluencia con RN 50, 12 Mar 2001, *R. Fortunato et al. 7130* (BAB); 3 km al SW del cruce de los caminos Cortadera-I. de Cañas en dirección a Cortaderas, 800 m, 13 Mar 2001, *R. Fortunato et al. 7162* (BAB, CTES, MO); Barrio Tres Cerritos (Las Margaritas al 200), 4 Feb 2010, *J.R. de la Fuente s.n.* (CORD 00006960); Estancia La Despensa, a ± 25km de la Caldera, 16 Apr 1942, *A.T. Hunziker 1579* (CORD, MA); Urundel, 12 May 1942, *A.T. Hunziker 1985* (CORD, MA, LIL) *& 1987* (CORD, LIL); Urundel, Arroyo Nipán, 12 May 1942, *A.T. Hunziker 2010 & 2024* (CORD); Tabacal, 310 m, 2 Apr 1943, *A.T. Hunziker 2776 & 2786* (CORD); Estación Experimental INTA-Cerrillos, 28 Mar 1995, *A.T. Hunziker 25491* (CORD); Castañares,

campos de la Universidad Nacional de Salta, 1200 m, 10 Feb 1993, *F. Juárez de Varela 2076* (CORD); Urundel, en las inmediaciones del Río Colorado, 30 Jan 1945, *A. Krapovickas 1575* (CORD); Las Piletas, 4 km W de Yariguarenda, 7 May 1998, *A. Krapovickas et al. 47263* (NY); Rosario de la Frontera, 7 Jan 1905, *M. Lillo 3850* (LIL); San José, Feb 1873, *P.G. Lorentz & G. Hieronymus 240* (CORD, GOET); San Martín, Río Caraparí, 650 m, 22 Feb 1954, *T. Meyer 18144* (LIL); entre Quisto y Obraje Palo Santo, 19 Mar 1965, *T. Meyer & A.R. Cuezzo 22440* (CTES); J. V. González, 10 km al oeste, 8 Mar 1958, *J. Morello & A.R. Cuezzo 157* (LIL); La Lagunita, en Coronel Ollero, 8 Mar 1958, *J. Morello & A.R. Cuezzo 174* (LIL); Osma (cult. finca de L. Chávez), 1250 m, 10 May 1995, *E. Moscone & R. Neumann 210* (BM, CORD, NY); Estación Experimental INTA-Cerrillos, 11 May 1995, *E. Moscone & R. Neumann 214 & 215* (CORD, MA); Sierra de Vélez, cerro de la ermita, detrás de la capilla de la virgen de Schoenstadt, 1300 m, 15 Jan 2005, *L. Novara et al. 12205* (BM, CORD, MCNS); Vespucio, 22 Nov 1947, *F. Rial Alberti s.n.* (BAB 71882); La Caldera, Mar 1942, *S. Rosa 366* (LIL, MCNS); Territorio de Orán, 19 Mar 1905, *C. Spegazzini s.n.* (BAB 14053, CTES 133283); Sierras de Vélez, laderas de cerros al este del barrio Tres Cerritos, 4-5 km al NE de la ciudad de Salta, 1400 m, 13 Feb 2001, *J. Tolaba 2843* (BAA, CORD, CTES, MCNS); near Rio Las Cañas, Sierra Lumbrera, 38km, NE of Lumbreras, 800 m, 20 Feb 1937, *J. West 8389* (GH, LIL, MO, UC); **Tucumán**: San Pedro de Colalao, 1916, *L. Castillón s.n.* (ex LIL 88480) (GH); carretera Choromoro-quebrada de la Higuera, 25 Jan 1955, *E. de la Sota 104* (LIL); camino a S. Pedro de Colalao, 1100 m, 16 Apr 1926, *S. Venturi 4226* (LIL, US).

BOLIVIA. Beni: Estación Biológica del Beni, comunidad Los Manguitos, 14º30’S, 66º37’W, ca. 200 m, 7 Nov 1994, *E. Rivero 218* (LPB); Ballivian, Rurrenabaque, dooryard garden, cult., 14º30’S, 67º30’W, 227 m, 5 Aug 1989, *D.E. Williams 932* (CTES, NY, US); **Chuquisaca**: 34 km E de Monteagudo, en Cruz Pampa, 19º49'S, 63º48'W, 1160 m, 17 May 1994, *D.G. Debouck et al. 3019* (LPB); Villa Vaca Guzmán (Muyupampa), 19º52,842'S, 63º45,704 W, 1183 m, 23 Feb 2005, *C. Manchego CBP 11* (LPB, USZ); Luis Calvo, Villa Vaca Guzmán (Muyupampa), 19º52,954'S, 63º44,345 W, 1217 m, 24 Feb 2005, *C. Manchego CBP 12* (USZ); en los alrededores de Monteagudo, cerca de la comunidad Las Palmitas, 1148 m, 26 Feb 2005, *C. Manchego CBP 13, 14 & 1*5 (USZ); llegando a San Miguel, en la ruta Monteagudo a Rosario del Ingre, 20º04,799’S, 63º53,216’W, 1001 m, 1 Mar 2005, *C. Manchego CBP 16* (USZ); San Miguel, entre la ruta de Monteagudo hacia Rosario del Ingre, 20º05,095’S, 63º53,161’W, 996 m, 1 Mar 2005, *C. Manchego CBP 17* (USZ); antes de llegar a San Miguel, sobre la ruta Monteagudo-El Ingre, 20º02,760’S, 63º52,947’W, 991 m, 3 Mar 2005, *C. Manchego CBP 18* (LPB, USZ); en la ruta de Boyuibe a Villamontes, en las cercanías de Machareti, en patio de una casa, 20º49,00’S, 63º20,969’W, 638 m, 29 Mar 2005, *C. Manchego CBP* NT2 (USZ); en las cercanías de Villa Vaca Guzmán (Muyupampa), 19º52,846'S, 63º45,214 W/19º53,333'S, 63º44,445 W, 1192-1236 m, 24 Feb 2005, *C. Manchego CBNP 04, 05 & 06* (USZ); 0.2 km E of Camiri-Villa Montes highway on turnoff to Ñancaroinza, 20°42'22"S, 63°17'42"W, 740 m, 19 Mar 2007, *M. Nee & R. Flores S. 54782* (COL, CORD, F, LPB, MO, MEXU, NY); Cantón Pedernal, Comunidad Roldana, pendientes medias de la serranía Cazaderos, 1550 m, 19º48´S, 64º6´W, 11 Feb 1999, *A. Ovando 18* (LPB); Sud Cinti, El Palmar, Quebrada Champial, 21°04'31"S, 64°20'39"W, 1300 m, 6 Mar 2008, *E. Portal et al. 495* (MO); Salvador-Cimboc, El Centro (1 km al sur), 820 m, 7 Apr 1993, *C. Saravia Toledo et al. 11392* (CORD, CTES, LPB, SI); El Salvador, Cimboc, 2 km al sud de Isirenda, 850 m, *C.*

Saravia et al. 11572 (CORD); El Salvador-Cimboc, camino al puesto Huare, 1-2 km S del centro, 10 Apr 1993, *C. Saravia Toledo 11607* (CTES, LPB, MO, NY); El Salvador-Cimboc, El Centro, 800 m, 6 May 1995, *C. Saravia Toledo 13481* (CTES); Hacienda Aperiate, ca. 80 km saliendo de Monteagudo a Rosario del Ingre, 20º19'21"S, 63º51'32"W, 1300 m, 15 Mar 1996, *M. Serrano 1566* (LPB); Hacienda Aperiate, ca. 80 km saliendo de Monteagudo a Rosario del Ingre, 20º19'21"S, 63º51'32"W, 1400 m, 22 Mar 1996, *M. Serrano 1903* (LPB); Belisario Boero, bajando hacia Santa Rosa, en dirección al Río Grande, 18º55'37"S, 64º17'07"W, 1353 m, 2 Mar 2006, *J.R.I. Wood et al. 22339* (LPB); **Cochabamba**: Campero, 29 km S of town square of Aiquile, on road to Sucre, 18º30.711'S, 65º10.685W, 2241 m, 4 Mar 1993, *D. Spooner et al. 6657* (LPB, WIS); **La Paz**: Nor Yungas**,** Yolosa, entrada al campamento de la mina, a unos 200 m antes de Yolosa, bajando de Coroico, 16º13'38"S, 67º44'28'W, 6 Jun 2010, *M. Atahuachi et al. 1666* (LPB); Sud Yungas, unos 36 km de Puente Villa hacia Chulumani-Monton Coro, playa sobre el Río Chajro, 1300 m, cult., 1 Apr 1994, *S. Beck 21356* (CORD, LPB, NY); Yolosa, subiendo 1.2 km hacia San Juan de Miel, 1430 m, 19 May 1996, *S. Beck et al. 22714* (BAB); Franz Tamayo, Parque Nacional Madidi, camino Apolo-Azariamas, arroyo Pintata, 14°28'06"S, 68°32'19"W, 940 m, 20 Feb 2003, *L. Cayola et al. 42* (LPB, MO, NY); same locality, 14°28'23"S, 68°32'17"W, 893 m, 23 Feb 2003, *L. Cayola et al. 127* (MO); from Chullumani to La Paz, 3 km west of Huancané, 17 Mar 1971, *W.H. Eshbaugh E954* (GH); **Santa Cruz**: Santa Cruz de la Sierra, inner ring, between #50 and #84 on south side of Mercado St, 17°47'19"S, 63°10'54"W, 13 Feb 1995, *J.R. Abbott 16182* (MO); Yabaré, ca. 25 km N of Estación Tres Cruces, 300 m, 17 Mar 1995, *J.R. Abbott 16431* (MO, NY, USZ); Bella Vista, sendero Cola de Mono, 18°17'0.22''S, 63°40'37''W, 1250-1300 m, 14 Jun 2006, *L. Arroyo et al. 3313* (USZ); Puente La Negra, sobre la carretera Samaipata-Santa Cruz, 18º08'26"S, 63º41'32"W, 23 May 2010, *M. Atahuachi et al. 1632* (LPB); carretera Cotoca-Las Pailas, desviando por la ruta 29 (hacia el sur) frente a la entrada a la finca San Juanito, 17º44'03"S, 62º54'31"W, 24 May 2010, *M. Atahuachi et al. 1634* (LPB); Hacienda La Esperanza, camino de Cotoca a Montero Hoyos, 17º46'06"S, 63º21'15"W, 24 May 2010, *M. Atahuachi et al. 1635* (LPB); Las Cruces, en el camino entre Santa Cruz y Terebinto, propiedad de don J. Pedrazas, 17º27'28"S, 63º39'21"W, 24 May 2010, *M. Atahuachi et al. 1636* (LPB); Bermejo, en la propiedad de la Sra. Melania, al frente del restaurante de la localidad, 29 Nov 2012, *G.E. Barboza & C. Carrizo García 3646* (CORD); desde Mairana, pasando Yerba Buena rumbo a Comarapa, 17º59'26''S, 64º02'52''W, 1171 m, 12 Dec 2017, *G.E. Barboza et al. 4913* (CORD, LPB, MO, NY, P); unos 5 km antes de Bermejo, viniendo desde Tarumá, apenas pasando el km 428, 18º09'36''S, 63º36'14''W, 12 Dec 2017, *G.E. Barboza et al. 4919* (CORD); Comarapa, Jardín de Cactaceae de Pulquina, 1584 m, 22 Feb 2006, *N. Biggs et al. 38* (K, LPB, USZ); Camiri, Feb 1951, *M. Cárdenas 4701* (US); RN 9, de Camiri hacia Santa Cruz, ca. Km 166, pasando 7 km la entrada a Tatarenda Nuevo, 19º03'30''S, 63º23'22''W, 799 m, 8 Mar 2018, *C. Carrizo García 63* (CORD); Chiquitos, RN 4, entre San José de Chiquitos y Corumbá, 18º37'17''S, 58º59'53''W, 146 m, 4 May 2016, *A.A. Cocucci et al. 5992* (CORD); RN 9, entre Santa Cruz y Basilio, 18º05'26''S, 63º11'40''W, 499 m, 5 May 2016, *A.A. Cocucci et al. 5996* (CORD); Estancia San Miguelito, laguna Marañón, 17°01'S, 61°52'W, 270 m, 8 Dec 1996, *A.F. Fuentes 1385 A* (MEXU, MO, USZ); 12 km de Santa Cruz, en selva de jardín botánico poco explotada, 23 Mar 1994, *J.H. Hunziker et al. 13036* (SI); Vallegrande, Masicurí, a 88 km al sur de Vallegrande (cult. en jardines de la población Masicurí), 18º49'37''S, 63º46'48''W, 714 m, 19 Dec 2005, *R. Hurtado 296* (CORD, LPB);

ciudad de Santa Cruz de la Sierra, en el Zoológico Municipal, 8 Jul 1995, *A. Jiménez L. 50* (USZ); 5 km N de Tatarenda, 19º12’S, 63º32’W, 17 Apr 1977, *A. Krapovickas & A. Schinini 31459* (CORD, CTES); Embocada del Carmen, ca. 40 km SSW de Concepción, 16º38’S, 62º26’W, 550 m, 2 May 1977, *A. Krapovickas & A. Schini*ni *32144* (CTES); San Ignacio, 16º22’S, 60º58’W, 370 m, 5 May 1977, *A. Krapovickas & A. Schinini 32244* (CTES); 27 km S de San Ignacio, camino a San Miguel, 400 m, 7 May 1977, *A. Krapovickas & A. Schinini 32360* (CTES); Gutiérrez, cerca de la comunidad Sauzalito, carretera Abapó-Camiri, 19º24.802’S, 63º31.235’W, 977 m, 21 Feb 2005, *C. Manchego CBP 01* (LPB, USZ); cerca de la localidad El Chorro y Taruma, a 2 km de Gutiérrez, 19º24,996’S, 63º32.585’W, 990 m, 21 Feb 2005, *C. Manchego CBP 03* (LPB, USZ); a 1 km de la población de Gutierrez, sobre el camino a El Chorro, 19º25.124’S, 63º32.245’W, 964 m, 21 Feb 2005, *C. Manchego CBP* 04 (USZ); a 500 m de la carretera a Camiri, en las cercanías de la población de La Herradura, 19º29.659’S, 63º31.752’W, 954 m, 22 Feb 2005, *C. Manchego CBP* 05 (LPB, USZ); Gutiérrez, en la ruta de Ipatí a Charagua, 19º27.511’S, 63º14.417’W, 828 m, 17 Mar 2005, *C. Manchego CBP A1* (USZ); en la localidad de San Isidro (propiedad de Sauces-Rosales), Izozog, 19º34,675’S, 63º13,401’W, 861 m, 24 Mar 2005, *C. Manchego CBP T*1 (USZ); zona Izozog, dentro de la propiedad Guariri, 19º19.255’S, 63º16.979’W, 823 m, 26 Mar 2005, *C. Manchego CBP T2* (LPB, USZ); en la ruta de Bajo Izozog a Boyuibe, 20º17.444’S, 63º15.321W, 855 m, 29 Mar 2005, *C. Manchego CBP T4* (USZ); dentro del parque Kaa-Iya, en el campamento Tucavaca, 18º30,978’S, 60º48,626’W, 276 m, 1 May 2005, *C. Manchego CBP T29* (LPB, USZ); en las proximidades de Cuevo, 20º26.911’S, 63º33.396’W, 1108 m, 7 Apr 2005, *C. Manchego CBP NT5* (LPB, USZ); en Lagunillas, 19º37,119’S, 63º40,270’W/19º40,008’S, 63º40,059’W, 902-921 m, 23 Feb 2005, *C. Manchego CBNP 01 & 02* (USZ); en las cercanías de la población de Aratical, 19º43,094’S, 63º39,434’W, 979 m, 23 Feb 2005, *C. Manchego CBNP 03* (USZ); Conseción Don Mario ubicado a 125 km de San José de Chiquito, 400 m, 22 Feb 1997, *F. Mamaní M. et al. 1435* (CTES, MEXU, USZ); Warnes, Pampa de Viru-Viru, a 17 km al N de ciudad de Santa Cruz, 17º39’46’’S, 3º69’24’’W, 360 m, 2 Apr 1994, *M. Mencho & E. Gutiérrez 586* (MEXU, USZ); Alto Parapetí, 850 m, 8 Jan 1982, *R. de Michel 159* (CORD, LPB); Alto Parapeti, "Hacienda Yabuimbia", 800 m, 18 May 1987, *R. de Michel 714* (LPB); Valle de Tucavaca (29 km NE de Santiago, camino a Santo Corazón, 31 Jan 1995, *B. Mostacedo C. 2699* (MO); Estancia Cachari (Bañados del Izozog), 400 m, 1 May 1993, *G. Navarro Sánchez 1618* (LPB, MO, USZ); 14 km (by air) NW of San Javier, Las Lajas, "El Chupadero, 550 m, 30 Nov 1990, *M. Nee 10103* (MO, NY); 3 km. S of Mataral, broad valley of Rio La Ciénega, 18°08'S, 64°13'W, 1400 m, 1 Feb 1987, *M. Nee & G. Coimbra S. 33969* (MO, NY); 12 km E of center of Santa Cruz, on road to Cotoca, 375 m, 20 Feb 1987, *M. Nee 34184* (CAS, CORD, LPB, MA, MO, NY, P, SI, US); 10 km S of center of Santa Cruz, 1 km N of YPFB oil refinery on dirt road to Lomas de Arena, 17º52’30’’S, 63º11’W, 425 m, 15 Feb 1988, *M. Nee 36304* (LPB, MO, NY); 8 km NW of Mairana, 18°03'S, 63°59'W, 1250 m, 6 Mar 1988, *M. Nee 36514* (CORD, MO, NY, USZ); ca. 15 km. NE of Cotoca along highway to Puerto Pailas, 17°42'S, 62°53'W, 325 m, *M. Nee 37759* (MO, NY, USZ); 10 km ENE of center of Santa Cruz, on dirt road to Monte Cristo, 17º45´S, 63º05´W, 390 m, *M. Nee 42191* (CORD, MO, NY, USZ); 13 km NE of Cotoca, 2-3km N of Quebrada Cotoca on road to “El Pauro”, 17º41´S, 62º55´W, 325 m, 18 Dec 1992, *M. Nee 43167* (CORD, LPB, MO, NY, USZ); 7 km (by air) NW of Puerto Pailas, ca 2.5 km. W of center of Montero Hoyos, 300 m, 10 Feb 1994, *M. Nee 44837* (MO, NY); along Quebrada Peji, vicinity bridge on new highway from

Santa Cruz to Camiri, 440 m, *M. Nee 45842* (MO, NY, USZ); along old dirt road highway and railroad from Cotoca to Puerto Pailas, 2.5 km W of Rio Grande bridge at Puerto Pailas, 17°40'S, 62°48'W, 290 m, 13 Jan 1998, *M. Nee 47937* (CORD, MO, NY, USZ); 3.5 km NE of Montero Hoyos, 17°37'S 062°49'W, 280 m, 24 Feb 1998, *M. Nee 48439* (CORD, LPB, MO, NY); along highway from Santa Cruz to Abapo, 3 km S of crossing of railroad and 2 km S of bridge over Quebrada Peji, 17°58'S, 63°11'W, 450 m, 27 Feb 1998, *M. Nee 48489* (MO, NY, USZ); along dirt road 3 km NW of center of Cotoca, at Quebrada Calleja, 17°44'S 063°00'W, 360 m, 4 Mar 1998, *M. Nee 48553* (MO, NY); along Río Piraí at Santa Cruz-Samaipata highway, bridge at Tarumá, 18°06'29"S, 63°27'25"W, 590 m, 10 Apr 2007, *M. Nee et al. 55030* (MO, USZ); Santa Cruz de la Sierra, *A.D. d'Orbigny 604* (P); a 300 m del Río Santa Elena, 18°55'51''S, 64°06'39''W, 1296 m, 6-7 May 2012, *G.A. Parada et al. 4355* (MO, USZ); Cabezas, 420 m, 3 Mar 1945, *I. Peredo 348* (LIL); San Ignacio, 25 km al SW Guapomocito, 400 m, 24 Jan 1986, *R. Seidel & S. Beck 59* (LPB); San Juancito, 27 km al N de San Ignacio, 1 May 1986, *R. Seidel & S. Beck 361* (LPB); 5,1 Km N de San Ramón, rumbo a Ascensión de Guarayos, 866 m, 15 Jan 2005, *J.G. Seijo et al. 3448* (CORD, CTES, HUEM, LPB); a 126,8 km NE de Concepción y a 38,3 km NE de Santa Rosa, 16º03'10"S, 61º08'34"W, 344 m, 18 Jan 2005, *J.G. Seijo et al. 3580* (CORD, CTES, LPB, SI); Parque Nacional Kaa iya, picada hacia el S del campamento, 18º30'22''S, 60º48'24''W, 284 m, 3 Feb 2005, *J.G. Seijo et al. 3812* (CORD, CTES); Ichilo, Cantón Buenavista, bosquecitos, 450 m, 28 Apr 1916, *J. Steinbach 2044* (LIL); just south of Abapó, 18º37.115'S, 62º16.126'W, 563 m, 11 Apr 2004, *J. Tewksbury 1007* & *1009* (USZ); Villa Fátima, 4 km al SW del centro de la ciudad, 420 m, 3 Feb 1989, *I. Vargas Contreras 79* (MO, NY, USZ); Bella Vista, barbecho, sobre el camino, de retorno del Circuito ecoturístico "Quebrada el Fraile," al de la "Cola de Mono", 1323 m, 14 Jul 2006, *M. Vargas Contreras 141* (MO, NY, USZ); comunidad de Bella Vista, camino hacia el Chorro del Fraile, 18°19'55"S, 64°40'35"W, 1100-1365 m, 28 Apr 2006, *D. Villarroel 483* (MO, NY, USZ); Guadalupe village, c. 8 km of Vallegrande, 2050 m, 25 Dec 1999, *J.R.I. Wood & D.J. Goyder 15579* (LPB); Comunidad "Los Sotos", between Roboré and Limoncito, 18º17'S, 59º49'W, 300 m, 23 Feb 2006, *J.R.I. Wood 22267* (USZ); Pucará, al fondo del valle del Río Grande, 18º41'56"S, 64º16'11"W, 1590 m, 18 Feb 2007, *J.R.I. Wood et al. 22798* (LPB, USZ); Germán Busch, Puerto Suárez, al norte en línea recta de la entrada de El Padrino, 18º54'46,06"S, 58º18'03,86"W, 107 m, 25 Feb 2013, *F. Zenteno-R et al. 12798* (LPB); **Tarija**: Aviles, zona del proyecto PERIT/GTZ en Colón, 31 Jan 1988, *S. Beck & M. Liberman 16298* (B, NY); Cercado, pasando el Río Santa Ana, 21º37'49"S, 64º33'36"W, 1915 m, 24 Apr 2006, *S. Beck 32066* (LPB); Palos Blancos, 800 m, Apr 1978, *M. Coro 1016 & 1126* (LIL); Entre Ríos, a 23 km al norte de Palos Blancos, camino a Huacaya, 21º12'34"S, 63º47'44"W, 522 m, 13 Feb 2014, *R. Hurtado & R. Zenteno* 1636 (LPB); El Sivingal, 10 km N de San Simón, 21º20´S, 64º2´W, 30 Apr 1983, *A. Krapovickas & A. Schinini 39010* (CTES, UC), *39012* & *39018* (CORD, CTES, LPB); 30 Km N de Yacuiba, Campo de la Tapia, cerca de Caiza, 21º45'S, 63º32'W, 600 m, 7 Apr 1977, *A. Krapovickas & A. Schinini 30952* (CTES); cerca de Chocloca, en la Loma cerca del Río Wayco, 1850 m, 4 Feb 1988, *M. Liberman et al. 2051* (LPB); de Tarija a Tolomosa, 1920 m, 20 Mar 1978, *C. Ochoa 11995* (CORD, MOL, NY, US); cerca de la población de Caraparí, 21º49,582'S, 63º44,643'W, 794 m, 2 Apr 2005, *C. Manchego CBP T7 & T8* (USZ); en el límite fronterizo de Bolivia-Argentina, en las cercanías de Yacuiba, 22º01,743'S, 63º40,948'W, 611 m, 2 Apr 2005, *C. Manchego CBP T9* (LPB, USZ); cerca de Villamontes, 21º16,062'S, 63º31,141'W, 393 m, 3 Apr 2005, *C. Manchego CBP T10*

(LPB, USZ); en las afueras de Entre Ríos, 1229-1250 m, 5 Apr 2005, *C. Manchego CBP T16 & T17* (LPB, USZ); Entre Ríos, 21º30,728'S, 64º10,581'W, 1228-1263 m, 5 Apr 2005, *C. Manchego CBP T18, 19 & T20* (USZ); en la población de Carapari, 21°49.664'S, 63°44.634'W, 814 m, 2 Apr 2005, *C. Manchego CBP NT3* (USZ); ruta Yacuiba-Villamontes, 6 Apr 1993, *C. Saravia Toledo et al. 11372* (CTES); Entre Ríos, Reserva Nacional de Flora y Fauna Tariquía, comunidad Salinas**,** 21°45'27"S**,** 64°13'15"W**,** 1188 m, 24 Jan 2006, *M. Serrano et al. 7297* **(**HSB, MA, MO, NY).

BRAZIL. Aguas Bellas, *Anonymous s.n.* (P); Brazil, *W.J. Burchell 1319* (K); habitat in Provinciae, *C.F.P. von Martius s.n.* (M); Brasilia, Jun 1822, *L. Riedel 787* (NY); Brasilia, *F. Sellow 221* (CORD fragment, K, P); **Bahia**. Santo Antônio de Jesús, entrada p/BA-028, Faz. Saveluma, próximo do Benfica, 11 May 2009, *L.Y.S. Aona et al. 1220 A* (RB); Bahia, *D. Duboc s.n.* (P 00409882); Bahia, *M. Guillot s.n.* (P 00410148 & P00409993); Souto Soares, 12 km E of Souto Soares on the road to Várzea do Cerco, 12º05'S, 41º31'W, ca. 800 m, 29 May 1980, *R.M. Harley et al. 22736* (CEPEC, F, K, MBM, RB, SPF); São Gabriel, Estrada para o alto do bode, 11º1'S, 41º39'W, 18 Apr 2009, *R.F. Machado et al. 271* (HUEFS); Morro do Chapéu, Buraco do Posidônio, 11º38'45''S, 41º16'14''W, 970 m, 18 Apr 2008, *E. Melo et al. 5571* (HUEFS); Buraco da Dona Duda ou Buracão, 19 Apr 2008, *E. Melo et al. 5664* (HUEFS); Barra do Mendes, Serra da Catinguinha, 20 Feb 2001, *M.V. Moraes 173* (HUEFS); Ibicaraí, Rod. BR-415, a 2 km W de Ibicaraí, 17 Mar 1979, *S.A. Mori et al. 11603* (CEPEC, NY, RB); Santa Maria da Vitória, ca. 7,7 km S de Santa María da Vitória na estrada para Lagoinha, 13º27'0''S, 44º10'16''W, 13 Feb 2000, *L.P. de Queiroz et al. 5958* (CEPEC, HUEFS, JPB); **Distrito Federal**: Reserva Ecológica do IBGE, cult., 13 Jun 2000, *T.S. Filgueiras 3615* (NY, FURB, RB); Chacara das Araras, cult., 10 Feb 1983, *R.A. Wolff 16* (RB); **Espírito Santo**: Santa Leopoldina, Cachoera do Retiro, 20º09'02"S, 40º26'55"W, *M.O. Crepaldi 59* (RB); Barra de São Francisco, Parque Sombra da Tarde, 230 m, 12 Dec 2000, *L. Kollmann et al. 3497* (RB); Santa Teresa, Nova Lombardia, Reserva Biológica Augusto Ruschi, Tracomal, 7 May 2003, *R.R. Vervloet & W. Pizziolo 2378* (RB); mata do Sr. A. Duca, 15 May 1984, *J.M. Vimercat 124* (RB); **Goiás**: Formosa, Fazenda Sucupira, proximidadesdo povoado Barreiro, 15°21'09.47''S, 47°11'05.80''W, 796 m, 24 Mar 2016, *P.H.A. Melo 5030* (BHCB); Nerópolis, Parque Estadual Altamiro de Moura Pacheco, Trilha do Guatambú (2,900 Km da placa), 16º30'32.7"S, 49º09'52.2W, 750 m, 14 Apr 2005, *Mendonça et al. 5971* (RB); **Mato Grosso do Sul**: Bela Vista, Rod. MS-384, 7 km de Antonio João, borda da Serra de Amambaí, 9 Feb 1993, *G. Hatschbach 58780* (CORD, CTES); Rod. MS-384, Rio Guaviral, 10 Feb 1993, *G. Hatschbach et al. 58827* (CAS, CTES, GH, HUEFS, LIL, MBM, MO, NY, SI); **Minas Gerais**: Lagoa Santa/Matozinho, APA Carste de Lagoa Santa, Sep 1995, *A.E. Brina & L.V. Costa s.n.* (BHCB); Três Corações, Rod. Três Corações-S. Tomé das Letras, 4 Feb 1973, *G. Hatschbach & L.Z. Ahumada 31246* (NY); Curimataí, arredores, 6 Jun 2004, *G. Hatschbach et al. 77668* (MEXU); Caldas, 2 Dec 1918, *S.E. Henschen s.n.* (ex herb. Regnell Ser. III, 1002) (US); São João del Rey, Bengo, 9 Feb 1988, *L. Krieger s.n.* (CESJ 25076, MBM 341919); Marliéria: Parque Estadual do Rio Doce, Lagoa da Carioca, 19º45'09''S, 42º37'59''W, 263 m, 26 Jan 1997, *J.A. Lombardi 1570* (F, MBM); Pains, Fazenda Amargoso, Km 16, 20º23'14''S, 45º38'59''W, 31 Jan 2003, *P.H.A. Melo & J.A. Lombardi 362* (BHCB, HUEFS); Santo Hipólito, cerca de 5 km além de Santo Hipólito em direção a Monjolos, à margem esquerda do Rio Pardo Pequeño, 23 Mar 1997, *R. de Mello-Silva 1275* (K, MBM, NY, SPF); Ouro Preto, Glaura, 20º18'S, 43º38'W, 1020 m, 5

May 2012, *M.C.T. Messias & R. M. Carrillo 2443* (OUPR); Caldas, 12 May 1873, *H. Mosén 984* (S); Juiz de Forá, top of Morro do Cristo, 800 m, 18 Oct 1970, *M. Nee 3424* (COL, RB, US, VEN); Brumadinho, Inhotim, 20°07'37''S, 44°13'31''W, 890 m, 29 Apr 2008, *F.M. Rodrigues & J.G. Oliveira 302* (RB); Santana do Riacho, Serra do Cipó (Serra da Lapa), São José da Cachoeira, 20 Feb 2007, *V.C. Souza et al. 32883* (ESA, RB, SPF); **Paraná**: Cruzeiro do Oeste, Ivaté, Jan 1961, *R. Braga 1536* (UPCB, US); **Rio de Janeiro**: Rio de Janeiro, *Dr. Mertens s.n.* (NY 00656000); Gavea, horto florestal, na vegetação ruderal, 29 Aug 1938, *H. Monteiro 1918* (CORD); Mun. Vassouras, à márgem da estrada de Morro Azul à Sertão, 9 Mar 1940, *H. Monteiro 2276* (CORD); Lagoa de Rodriguez, 1835, *F.C. Raben 7* (C); Rio de Janeiro, Oct 1832; *L. Riedel 1074* (CORD, NY, US); Paraíba do Sul: Fazenda do Sobral, 26-29 Nov 1881, *J. de Saldanha da Gama 6075* (CORD); Barra do Pirai, fragmento do Campus UGB, 20 Mar 2015, *G.R. Souza et al. 1680* (RB); **Rio Grande do Sul**: Porto Alegre, Morro São Pedro, 6 May 1980, *L. Aguiar & L. Martau 339* (CTES); Roça Velha, Baia da Assunção, 28 Aug 2006, *I. Augusto s.n.* (ICN 019084); Pelotas, Pedreira Santa Cecília, 8 Jun 1959, *J. da Costa Sacco 1332* (F, HB); Camaquá, Palanque, 15 Apr 1987, *W. Corvello s.n.* (CEN 10709); Osorio, Estrada entre RS 030 e Lagoa do Horácio, 29°54'54''S, 50°12'57''W, 3 m, 19 Apr 2015, *F. Gonzatti & E. Valduga 1779* (HUCS, RB); Uruguiana, Barragem do Sanchouri, camino a São Borja, km 143, 28 Feb 1979, *A.T. Hunziker et al. 23481* (CORD); Torres, 16 May 2003, *V.F. Kinupp et al. 3169* (ICN); Lami Sítio Capororoca, 11 Jan 2007, *V.F. Kinupp et al. 3229* (ICN); Retiro da Ponta Grossa, 24 Aug 2006, *H. Leister s.n.* (ICN 185540); Fazenda S. Maximiano, BR 116, km 32, 23 Mar 1978, *N.I. Matzenbacher s.n.* (ICN 00033364, P); Tres Passos, a 8/8,5 km de Tres Passos rumbo a Crissiumal, 280 m, 20 Nov 2003, *L.A. Mentz et al. 263* (CORD, ICN); Viamão**,** Parque Estadual de Itapuã, 7 Jul 2004, *L.A. Mentz et al. 309, 400, 401* (ICN); S. Leopoldo, Imagro, Morro Sapucaya, 1 May 1935, *B. Rambo 1896 pp.* (mixed with *Solanum* sp.) (LIL); Fião, 29 May 1949, *B. Rambo 41743* (LIL); ad montem Sapucaia, 18 Jun 1949, *B. Rambo 42062* (LIL); Lagoa dos Quadros, 21 Feb 1950, *B. Rambo 45874* (CORD, LIL) & *45999* (B, LIL); Lagoa dos Quadros, 18 Jan 1951, *B. Rambo 49778* (CTES, LIL); Gravataí, M. Morungava, acesso anterior a ponte-Rio Morungava, a esquerda da RS 020 sentido Gravataí-Taquara, 28 Mar 1979, *Z. Rosa et al. s.n.* (F 1926285, HAS 9131); Fazenda São Maximiliano, 4 Mar 1990, *E.P. Schenkel et al. 447* (ICN); **Santa Catarina**: Florianópolis, Naufragados, 200 m, 17 Nov 1970, *R.M. Klein & A. Bresolin 9216* (FLOR, ICN, US); Tapera, Ribeirão, 14 Oct 1969, *R.M. Klein & A. Bresolin 8346* (US); Araranguá, Sombrío, 5 Feb 1946, *B. Rambo 31580* (CORD, LIL); **São Paulo**: Iracemópolis, mata da Fazenda Bertioga (entrada placa Fazenda), 29 Jul 1993, *K.D. Barreto et al. 980* (RB); entre Mauá e Ribeirão Pires (vía férrea São Paulo-Santos), 8 Mar 1954, *M. Kuhlmann 3026* (CORD, RB, SP); São Paulo, arredores da Cidade Universitaria, 28 Apr 1967, *F. de Oliveira 4* (HUEFS, RB, US).

COLOMBIA. Amazonas: margen izquierda del Río Mirití-Paraná, bocana Guayaca, resguardo Miriti, familia Letuama, 0º59'31,5''S, 70º38'2,5''W, 290 m, *D. Cárdenas et al. 9401* (COAH); margen izquierda del Río Mirití, comunidad Guayabo, chagra Maria Rosa Yucuna y Pedro Yucuna (capitán), 0º58'15,8''S, 70º34'57,2''W, 244 m, *D. Cárdenas et al. 9403* (COAH); margen izquierda del Río Mirití, comunidad Centro Providencia, Puerto Baradero Chagra William-Makuna y Olinda Puinabe, 1º5'25.8''S, 70º15'33.8''W, 244 m, *D. Cárdenas et al. 9423* (COAH); **Antioquia**: Corregimiento de Providencia, cerca a la hidroeléctrica, 200 m, 19 Apr 1977, *J. Santa et al. 155* (COL); **Magdalena**: Santa Marta,

100-250 ft, 1898-1901, *H.H. Smith 1164* (F, G, L, MO, UC, S, US), *1169* (F, G, K, MO, P, US); *1479* (E, F, G, MO, P, U, UC, US); **Sucre**: Hda. La Estanzuela, 15 km al ENE Tolú, 26 May 1974, *P. Warner 376* (FMB); **Vichada**: Cumaribo, margen izquierda del rio Uva, comunidad Manajuare, 03º45'42.6''N, 69º22'42,9''W, 750 ft, *F. Rodríguez 164* (COL).

CUBA. Cuba, *Guio 80* (SEV-H 11309)

PARAGUAY. Paraguay, Dec 1885, *E. Hassler 215* (G, K); Cordillera de Altos, Feb, *E. Hassler 6070* (BM, G, GH, K, MO, NY, P, W, UC, US); **Alto Paraguay**: Cucarani, Dec 1991, *G. Schmeda 1584* (LPB, US); **Amambay**: Arroyo Estrella, Est. Yamborandí, 22°20'S, 56°55'W, 7 May 1989, *N.M. Bacigalupo et al. 1163* (FCQ); Parque Nacional Cerro Corá, Cerro Muralla, 350 m, *F.J. Fernández Casas & J. Molero 6063* (MO, NY); cerca del Parque Nacional Cerro Corá, en peñasco proximo a Gas Ory, 9 II, Jan-Feb 1982, *F.J. Fernández Casas & J. Molero 6131 A* (MO); a pocos km de Bellavista en dirección a San Carlos, 12 Feb 1982, *F.J. Fernández Casas & J. Molero 6248* (MO); afueras de Bellavista, junto al rio Apa, 12 Feb 1982, *F.J. Fernández Casas & J. Molero 6274* (MO); Estancia Errante, ca. 32 km SW de Bella Vista, 24 Aug 1980, *A. Schinini & E. Bordas 20626* (CTES); 13-15 km S de ruta 5, Cerro Corá, Colonia Picada Lorito, 11 Dec 1997, *A. Schinini & M. Dematteis 33747* (CTES, NY); Chiriguelo, 13 km E of PN Cerro Cora, Weaver's ranch, 200 m, 26 Mar 1983, *J.E. Simonis et al. 207* (MO, U); PN Cerro Corá, vicinity of Cerro Muralla, 22°39'S, 56°03'W, 300 m, 7 Feb 1982, *J.C. Solomon et al. 6778* (MO, PY); PN Cerro Corá, 22°39'S, 56°03'W, 300 m, 15 Feb 1982, *J.C. Solomon et al. 6966* (MO, PY); Río Apa, 15 Dec 1983, *R. Vanni et al. 299* (CTES); around Cerro Corá, 27 Feb 2001, *E.M. Zardini & A. Acosta 56060* (MO); **Boquerón**: Gral. Eugenio A. Garay, linea de Hito, frontera con Bolivia, 330 m, 8 May 1988, *A. Charpin & L. Ramella 21481* (CORD, G); Chaco Boreal, Misión Campo Loro, 4 Jul 1993, *T.L. Gragson 124* (MO); Filadelfia, 26 Nov 1982, *W.J. Hahn 802* (MO, PY); Tte. Ochoa, 21°45'S, 60°55'W, *A. Schinini & R. Palacios 25565* (CTES, FCQ); **Caazapá**: Santa Ursula, near a home garde, 5 May 1998, *K.A. Williams et al. 122* (CORD); **Canindiyú**: in rupetribus prope Cerro Pytá, Feb 1885-1895, *E. Hassler 1926* (BM, G, P); **Central**: Itá Enramada, Nov 1971, *A. Schinini 4140* (CTES); Central Paraguay, 1888-1890, *T. Morong 388* (BM, E, MO, NY, US); Estero del Ypoá, Villeta-Puerto Guyratí, 6 km S of Villeta, on trail to Paraguay River, 18 Nov 1992, *E.M. Zardini & L. Guerrero 33706* (AS, MO); **Concepción**: 3 km N Rio Ipamé, 18 Feb 1968, *A. Krapovickas et al. 13996* (BAA, CORD, UC); entre Paso Horqueta y Concepción, 25 Feb 1968, *A. Krapovickas et al. 14230* (CORD, CTES, MO); San Salvador, 21 Feb 1917, *T. Rojas 2436 & 2436 A* (AS, MO); Estancia Garay Cuê, 5.5 km NW of Casa Estancia, 22°38'42"S, 57°22'53"W, 185 m, 21 Mar 2011, *W.D. Stevens 31295* (MO, PY); Estancia Lapuri, between Estancia Arrecife and road to Valle Mi, 300 m, 15 Jan 2000, *E.M. Zardini & D. Quintana 53786* (AS, MO); **Cordillera**: Emboscada, 19 Jan 1987, *E. Bordas & G. Schmeda 4024* (CTES, FCQ); regione lacus Ypacaray, Dec 1913, *É. Hassler 12385* (BM, K, L, MO, NY, S, UC, US, Z); Itacurubí Cordillera, Compañía Pira Yu-í, 2 km from the cross road Valenzuela/Rt 2, 25º28'09.1''S, 56º52'30.8''W, 220 m, 13 May 1998, *K.A. Williams et al. 134* (CORD, FCQ) & *135* (FCQ); Caacupé, Hotel Caacupé, growing spontaneously in the patio, 25º23'10.9''S, 57º08'22.8''W, 250 m, 14 May 1998, *K.A. Williams et al. 136* (CORD, FCQ); eastern side of Río Piribebuy basin, 17 km W of Arroyos y Esteros, 25°08'S, 57°15'W, 23 Dec 1989, *E.M. Zardini & R. Velázquez 17171* (AS, MO); **Guairá**: Villarrica, Oct 1947, *P. Jörgensen 3679* (CAS, GH, LIL, MO, SI US);

Iturbe (Monte de Santa Clara), 8 Mar 1952, *J.E. Montes 15716* (CORD, LIL); monte Santa Clara-Iturbe, 15 Jul 1952, *J.E. Montes 15900* (LIL); Cordillera de Ybytyruzú, Cerro Peró, 1 km W of Destacamento Tororo, 25°55'S, 56°15'W, 17 Dec 1988, *E.M. Zardini & R. Velásquez 8922* (FCQ, MO); same locality, 13 Jan 1989, *E.M. Zardini & A. Aguayo 9522, 9549 & 9729* (FCQ, MO); **Neembucu**: Pilar, Curupayty, 18 Dec 1950, *T. Meyer 15922* (LIL); **Paraguarí**: Guarapi, Mar 1880, *B. Balansa 3131* (G, P); Parque Nacional Ybycui, 26°00'S, 56°50'W, 13 Jan 1983, *W.J. Hahn et al. 1024* (MO, NY, PY); 8 km de Paraguarí, camino a Peribebuy, cerca de una cascada, 6 Feb 1966, *A. Krapovickas 12446* (CTES); Cerro de Acahay, May 1919, *T. Rojas 3368* (CORD); Ibytymí, Mar 1942, *T. Rojas 9529* (AS, CORD, MO); Compañía Yeguarizo, Cerro de Acahay, 25º52'56.5S, 57º09'28.6''W, 280 m, 8 May 1998, *K.A. Williams et al. 129* (CORD); Calistro, Compañía Canete Cué, Carapegua Calistro, 25º50'41.6''S, 57º11'47.8''W, 180 m, 9 May 1998, *K.A. Williams et al. 131* (CORD); Cerro Mbatoví, 25°25'S, 57°07'W, 26 Jan 1989, *E.M. Zardini & M. Velázquez 10019* (FCQ, MO); Acahay Massif, 400 m, 11 Jun 1989, *E.M. Zardini et al. 12774* (MO, PY); Mbatoví Mountain, 25°25'S, 57°07'W, 300 m, 24 Jun 1989, *E.M. Zardini & M. Velázquez 13104* (FCQ, MO); National Park Ybycu'í, southwestern area around La Rosada, 3 Feb 1992, *E.M. Zardini & R. Franco 30370* (MO, PY); **Presidente Hayes**: Puerto Pinasco, Chaco Paraguayo, Oct 1930, *K. Fiebrig 12649* (AS, MO); Puerto Santa Rita, 27 Mar 1917, *T. Rojas 2435 a* (CORD, AS, MO); **San Pedro**: Loma Hoby, 18 Mar 1954, *A. Woolston 165* (SI); Yaguareté forest (Sustainable Forest Systems Site), trail to Río Aguaray, 23°46'16"S, 55°59'37"W, 20 Jun 1995, *E.M. Zardini & C. Balbuena 42915* (MO, PY, MEXU).

PERU. Cuzco: La Convención, Entre Santa Teresa y Santa María, en el pueblo antiguo de Santa María, 13º00'48''S, 72º37'11''W, 1208 m, 25 Jan 2012, *G.E. Barboza et al. 3419* (CORD); Calca, Quebrada, 1000 m, 21 May 1982, *L.v.d. Hoogte & C. Roersch 724* (F); Valle de Santa Ana, above Quillabamba, 4200 ft, 20 Jan 1975, *T. Plowman & E. Wade Davis 4788* (GH); Maranura, Hacienda Mandor, 3500 ft, 17 Jan 1975, *T. Plowman & E. Wade Davis 7407* (GH); Quillabamba, 25 Mar 1966, *P.G. Smith s.n.* (CORD 00090106, USM); Maranura, 12°54'47"S, 72°09'54"W, 1636 m, 22 Mar 2004, *L. Valenzuela et al. 3117* (CUZ, MO); Hacienda Sahuayaco, 820 m, 8 Jan 1940, *C. Vargas C. 1686* (GH); **Pasco**: Oxapampa, 2 mi. from Huancabamba, 1972, *P.G. Smith SA 251* (COL).

VENEZUELA. Sucre: Cumaná, Bordones, 9 Oct 1799, *F.W.H.A. von Humboldt & A.J. A. Bonpland 150* (B-W: 3 sheets).

Cultivated

ARGENTINA. Hort. Argentinens, 1787, *Anonymous s.n.* (M-0171541). **Buenos Aires**: Capital Federal, cult. Facultad de Agronomía de Buenos Aires, semillas de La Despensa (Salta), 29 Jan 1943, *A.T. Hunziker 7347* (CORD), 15 Feb 1943, *A.T. Hunziker 7348, 7349 & 7350* (CORD); cult. Jardín Botánico de la Facultad de Agronomía de Buenos Aires, semillas de Hunziker 1987 (Urundel, Salta), 3 Mar 1943, *A.T. Hunziker 7351, 7352 & 7355* (CORD), 15 Apr 1943, *A.T. Hunziker 7353 & 7354* (CORD); **Córdoba**: Capital, cult. Facultad de Ciencias Exactas Físicas y Naturales, semillas legadas por R. Neumann, 25 Mar 1990, *A.T. Hunziker 25380* (CORD); cult. Facultad de Ciencias Exactas Físicas y Naturales, semillas provenientes de Salta, 9 Feb 1990, *A.T. Hunziker 25381* (CORD); **Corrientes**: Capital, en el Campus Universitario, cult., 31 Mar 1998, *A. Schinini 34466*

(CTES); **Salta**: Capital, cult. invernáculo Sr. E. Lusvarghi, 27 Mar 1998, *G.E. Barboza 163* (CORD).

BRAZIL. Minas Gerais: Belo Horizonte, Jardim Botânico da Universidade Federal de Minas Gerais, 19°53’29.8’’S, 43°54’57.5’’W, 830 m, 15 Jan 2007, *D.F. Félix 71* (BHCB).

PARAGUAY. Central: Jardín Botánico (Asunción), 27 Nov 1944, *T. Rojas 12452* (AS, CORD, MO).

UNITED STATES OF AMERICA. **Indiana**: Cultivated in IU greenhouse, purchased in Coroico, Nor Yungas (Bolivia), 1959, *C.B. Heiser C276a, C279a* & *C280a* (CORD).

2b. Capsicum baccatum L. var. pendulum (Willd.) Eshbaugh

ARGENTINA. Córdoba: Santa María, Alta Gracia, en jardín de la ciudad, 22 Dec 2012, *R. Deanna 51* (CORD); San Alberto, cult. entre San Pedro y el Chocolate, camino a Chancaní, 384 m, 4 Jan 1956, *A.T. Hunziker 11491* (CORD); Capital, Barrio Alto Verde, cult. casa particular, 22 Jan 1991, *E.A. Moscone 192* (CORD); Río Segundo, RN 9, Pilar, cult. en jardín de Fam. Subils Aguirre, 13 Mar 1995, *R. Subils 4569* (CORD); **Corrientes**: Concepción, Tabay, cult., 1 Jan 1972, *M.M. Arbo 358* (CTES); Capital, Escuela de Agricultura, 22 Nov 1974, *L. Ferraro 554* (CTES); Mburucuyá, cerca de casa, resto de antiguo cultivo, 3 Mar 1945, *A. T. Hunziker 7339* (CORD); a ca. 15 km de Mburucuyá, después del puente sobre el Río San Lorenzo, en el monte, 6 Mar 1945, *A. T. Hunziker 7342* (CORD); Paraje Yahaveré, 28°31’15,1’’S, 57°45’55,4’’W, 57 m, 22 Mar 2012, *A. Pirondo et al. 160* (CTES, CORD); **Jujuy**: San Antonio, Vivero Las Petunias, Los Alisos, 24°16’41’’S, 64°16’34’’W, 15 Jan 2019, *N. Palombo 3* (CORD); **Misiones**: San Martín, justo en el desvío hacia Puerto Ouro Verde, 16 May 2004, *G.E. Barboza et al. 1038* (CORD); San Ignacio, camino a Teyú Cuaré, 18 Apr 2014, *M. Scaldaferro 49* (CORD); Candelaria, Santa Ana, alrededores de la localidad, en zona de desmonte, 13 Jun 1988, *R. Subils & E. Moscone 4265* (CORD); **Salta**: Capital, alrededores de la Ciudad Universitaria, 12 Jan 2001, *J.R. de la Fuente s.n.* (CORD 00101756); Los Naranjos, camino a Maroma, 23 Jun 1996, *N. Hilgert 1363* (MCNS); Los Naranjos, 27 Jun 1996, *N. Hilgert 1373 & 1374* (MCNS); Santa Victoria, P.N. Baritú, casa de Josefa Vilte, 3 Dec 1997, *N. Hilgert 2028* (MCNS); Guachipas, cult. Jardín, 21 Apr 1942, *A. T. Hunziker 2052* (CORD); RP 33, Payogasta, Pasaje Bella Vista, 29 Mar 1995, *A. T. Hunziker 25496* (CORD); Osma, 10 May 1995, *E.A. Moscone 211* (CORD); Acoite, 22 Jan 1983, *E.M. Zardini 1739* (MO).

BOLIVIA. Cochabamba: rumbo a Villa Tunari, apenas pasando el puente de Espíritu Santo, 17°03’51’’S, 65°38’45’’W, 488 m, 9 Dec 2017, *G.E. Barboza et al. 4891* (CORD); at Quioma, silver mineson bank of Rio Tintin, 2100 m, 3 Apr 1939, *W.J. Eyerdam 25313* (F, GH, UC); Centro Fitotécnico Pairumani, 18 Mar 1992, *E.A. Moscone 209* (CORD); Bella Vista, 2575 m, 1 Oct 1947, *R. Scolnik & R. Luti 605* (CORD); **Chuquisaca**: Tomina, Comunidad Thiu Mayum 68 km SSE de Padilla, 19º34'S, 64º06'W, 1200 m, 16 May 1994, *D.G. Debouck et al. 3016* (LPB); **La Paz**: Mapiri, 1892, *M. Bang 1474* (CAS, F, US); Villa Marka, rumbo a Caranavi, 16°11’07’’S, 67°44’34’’W, 1009 m, 5 Dec 2017, *G.E. Barboza 4886* (CORD); Sirupaya bei Yanacachi, 1650 m, 21 Nov 1906, *O. Buchtien 337* (US); Polo Polo bei Coroico, 1100 m, Oct-Nov 1912, *O. Buchtien 4698* (US); Franz Tamayo,

Huarutumo, 1000 m, Aug 1959, *M. Cárdenas 5565*; Santa Ana de Mosetenes, 390 m, 3 Apr 1990, *I. Hinojosa & A. Wásra 1133* (LPB); **Santa Cruz**: La Bélgica (25 km N de Santa Cruz de la Sierra), 23 Apr 1977, *A. Krapovickas & A. Schinini 31659* (CTES); Ascensión de Guarayos, 26 Apr 1977, *A. Krapovickas & A. Schinini 31721* (CTES, F, LPB); Embocada del Carmen, ca. 40 km SSW de Concepción, 16°38'S, 62°26'W, 550 m, 2 May 1977, *A. Krapovickas & A. Schinini 32133 & 32141* (CTES, L); Porongo, Reserva privada Urubai, a 7 km de la localidad de Terebinto, 17º41'S, 63º16.3'W, 400-600 m, 26 May 2006, *I. Linneo F. 476* (USZ); Alto Parapetí, hacienda Yapuimbia, 800 m, 8 Feb 1986, *R. de Michel 492* (CORD); Cuarirenda Izozog, 7 Aug 1998, *A. Roca 0689* (LPB); La Miel, 17 km WSW (linea recta) del centro de Santa Cruz, 17º48'S, 63º21'W, 460 m, 24 Sep 1860, *M. Saldias P. 760* (MO); Santa Cruz de la Sierra, cult. in backyard garden in city, 17°46'S, 63°04'W, 375 m, 18 Sep 1988, *D.E. Williams 696* (CTES, LPB, NY, USZ); **Tarija**: Cercado, from home in Tarija, 11 Mar 1971, *W.H. Eshbaugh E 924* (GH); Saladillo, 21º36'S, 63º45'W, 1000 m, 8 Apr 1977, *A. Krapovickas & A. Schinini 31054 & 31056* (CTES); Yacuiba, 7 May 1983, *A. Krapovickas & A. Schinini 39321* (CTES).

BRAZIL. **Amapá**: Macapá, dist. Fazendinha, Pólo Hortifruti, 5 Jul 2008, *L.A. Pereira et al. 1819* & 8 Nov 2008, *L.A. Pereira et al. 1899* (CORD, RB); Vila Ressaca, Rodovia do Curiaú, 14 Jul 2008, *L.A. Pereira & W.M.S. Severino 1851* (RB); Santana, Ilha de Santana, 19 Jul 2008, *L.A. Pereira et al. 1863* (HAMAB, RB); **Goiás**: Jataí, UFG-Campus Jatobá, 1 Jul 2010, *E.V.E. Amaral 63* (CEN, HJ); Niquelandia, Rua 7 de Setembro Nº 12, 6 Apr 1988, 580 m, *L.A. Skorupa 330* (CEN); **Minas Gerais**: Sá Fortes, área cultivada, 22 Oct 2008, *R.R. Vianna s.n.* (OUPR 23117 & 23178); **Paraná**: Morretes, Estação Experimental, May 1988, *L.C. Leitao s.n.* (RB 274863); Campo Mourao, 1978, *J.M. Lima 332* (RB); Maringá, CESUMAR, horto, 555 m, 3 Mar 2010, *C. Rodrigues et al. 03* (HUEM); Campina do Siqueira, 3 Feb 1967, *C. Stellfeld 1677* (CORD, UPCB); **Rio de Janeiro**: Paraty, Praia de Martim de Sá/Reserva Ecológica da Juatinga, 23°19'20''S, 44°24'26''W, 15 Mar 2006, *R. Borges 66* (RB); **Rondonia**: Vilhena, 34.4 km do entroncamiento da BR-364 em direção a Colorado do Oeste, 12°57'S, 60°27'W, 350 m, 8 Oct 1990, *B.M.T.Walter et al. 566* (CEN, HEPH, UFG); **Santa Catarina**: Luis Alves, Vila do Salto, estrada a Maximo, 26°45'S, 48°51'W, 90 m, 26 Apr 1985, *E. Lleras Pérez et al. 1992*, *1993*, *1994* & *1995* (CEN, CORD); **São Paulo**: Piracicaba, ESALQ (Departamento de Horticultura), *G. Alves de Paiva 01* (RB); Parque Estadual de São Paulo, cult., 4 May 1956, *W. Hoehne s.n.* (RB 342486, CEN 026355, NY 656003); Itú, *Padre A. Russel 6140* (US, SP); Piracicaba, plantação de pimenta, 20 Oct 1995, *R. Rodrigues Vidal 03* (RB); Pindorama, 20 Oct 1990, *E.P.M. Shayer s.n.* (RB 619339); Lençoís Paulista, cult., 1991, *J.C. Toniolo s.n.* (RB 619552).

CHILE. Coquimbo: Vicuña, a 62 km al E de La Serena, en las inmediaciones de la bodega pisquera Capel, 22 Feb 1996, *A.T. Hunziker 25552 & 25559* (CORD).

COLOMBIA. **Meta**: Mun. Villavicencio, 500 m, 12 Oct 1938, *J. Cuatrecasas & H. García Barriga 3519* (COL, US).

ECUADOR. Esmeralda: Carretero Santo Domingo de los Colorados-Quinindé, km 171-188, hasta el Río Cócola, 100 m, 17 Feb 1950, *M. Acosta Solís 16208* (F). **Loja**: en el predio de

la Ciudad Universitaria Nacional de Loja, 4°2'6''S, 79°12'8''W, 2162 m, 5 May 2017, *G.E. Barboza 4824* (CORD).

PARAGUAY. Central Paraguay, 1888-1890, *T. Morong 696* (US); **Alto Paraná**: Distrito Naranjal, Compañía Agropeco, 26°03'34.4''S, 55°14'27.6''W, 26 Jul 1998, *F. Mereles et al. 7502* (CORD); **Boquerón**: garden, 25°58'1.7''S, 56°30'45.9''W, 5 May 1998, *K.A. Williams et al. 121* (CORD); **Cordillera**: Itacurubí Cordillera, Compañía Pirá Yu-í, 25°28'21.45''S, 56°52'47.8''W, 200 m, 13 May 1998, *K.A. Williams et al. 135* (CORD); **Guairá**: Colonia Eugenio A. Garay, Compañía Mbocayá, 25°59'42.2''S, 56°10'36.5''W, 23 Jul 1998, *F. Mereles et al. 7499* (CORD); Villarrica, salida de la ciudad, Cerro Polilla, 21 Apr 1999, *F. Mereles & M. Soloaga 7568* (CTES); Cordillera de Ybytyruzú, Cerro Peró, near Destacamento Tororo, camino a Cerro Polilla, 25°55'S, 056°15'W, 23 Jul 1989, *E.M. Zardini & R. Velásquez 13856* (FCQ, MO); **Paraguarí**: cerca de la estación de Paraguarí, 9 Apr 1885, *C. Galander 1877* (CORD); Carapeguá, camino a Acahay, 6 Mar 2006, *A. Honfi et al. 1288* (CORD, MNES); Ybycui, Compañía Ibycui, 26°01'20.8''S, 57°00'48''W, 145 m, *K.A. Williams et al. 127* (CORD) & *128* (FCQ); **Presidente Hayes**: camino a Puerto Nanawa, a 1 km de la intersección con camino a Puerto Falcón, 5 Mar 2006, *A. Honfi et al. 1284* (CORD, MNES); Puerto Falcón, al lado del laboratorio de Salubridad, 5 Mar 2006, *A. Honfi et al. 1287* (CORD, MNES); **San Pedro**: ruta 3, Río Jejuí-Guazú, 24º8'S, 56º25'W, 16 Jan 1979, *A. Krapovickas & C.L. Cristóbal 34271* (CORD, CTES);

PERU. Ancash: Fundo Carrizal, off Pan American Highway, about 2 km SE of the City of Casma, 09°29'S, 78°17'W, May-Jun 1986, *P. Francia 42* (MO); **Ayacucho**: Santa Rosa, 600 m, 26 Oct 1977, *Johns 343* (MOL); Estrella, between Huanta and Rio Apurimac, 500 m, 8-14 May 1929, *E.P. Killip & A.C. Smith 22637* (F, US); **Cajamarca**: alrededores de Huahuaya (ruta a Tabacones), 870 m, 31 Jul 1994, *S. Leiva González et al. 1214 & 1216* (F, NY); **Cuzco**: Cocalpampa, Chaullay, Maranura, Quintalpata +/- 150 km NW from Cusco on the road to Kiteni, 1210-1435 m, 12°49'S, 72°47'W, 29-30 Dec 1986, *P. Núñez V. et al. 6799* (MO, USM); La Convención, ca. 150 km de Cusco, en la vía carretera Cusco-Quillabamba, áreas cultivadas de Chaulley y La Pampa, 12º57'S, 72º40'W, 1100-1300 m, 14 Nov 1987, *P. Núñez V. 8600* (MO, NY); **Junín**: Tarma, Río Ulcumayo, Pomamarca, ca. 12 km NW of S. Ramon, 31 Nov 1962, *H.H. Iltis 257* (MOL); **Lima**: La Molina, 300 m, 30 Mar 1954, *O. Velarde Nuñez 1, 2, 3, 5, 6* & *10* (CORD); La Molina, 300 m, 29 May 1954, *O. Velarde Nuñez 11, 13, 15* & *17* (CORD); same locality, 13 Feb 1954, *O. Velarde Nuñez 23,* 5 Jun 1954, *O. Velarde Nuñez 21* & 30 Jun 1954, *O. Velarde Nuñez 24, 25* & *27* (CORD); **Loreto**: Santa Rosa, lower Río Huallaga below Yurimaguas, 135 m, 1-5 Sep 1929, *E.P. Killip & A.C. Smith 28864* (NY, US); **San Martín.** Distrito San Martín, west side of Rio Huallaga, west of Shapaja, 900 ft, 5 Aug 1937, *C.M Belshaw 3210* (UC, US).

Cultivated

ARGENTINA. Córdoba: Capital, cult. Invernadero IMBIV (UNC), semillas de FECOAGRO-San Juan "cultivar picante", 28 Feb 2018, *G.E. Barboza 4975* (CORD); en el Jardín de la Fac. Cs. Exactas, Físicas y Naturales, 410 m, 7 Jan 1971, *A. T. Hunziker 20762* (CORD); cult. Invernadero Museo Botánico, semillas del Mercado de Salta, 9 Feb 1990, *A. T. Hunziker 25382* (CORD); cult. Invernadero Museo Botánico, semillas de Tarija (Bolivia), 6 Mar 1990, *A. T. Hunziker 25383* (CORD); cult. Invernadero Museo Botánico, semillas de Los Toldos (Salta), 10 Feb 1994, *Menéndez Sevillano s.n.* (CORD 90112 &

90115); cult. Invernadero Museo Botánico, seeds from Cochabamba (Bolivia), 18 Mar 1992, *E.A. Moscone 205* (CORD); cult. Invernadero Museo Botánico, seeds from Mercado Jujuy, 18 Mar 1992, *E.A. Moscone 206* (CORD); Invernadero IMBIV (UNC), seeds from Piura market (Peru), 15 Feb 2013, *M. Scaldaferro 19, 20* & 29 Oct 2013, *M. Scaldaferro 42* (CORD); cult. Invernadero IMBIV (UNC), seeds from Mercado Villamontes-Tarija (Bolivia), 21 Aug 2013, *M. Scaldaferro 37* (CORD); cult. Invernadero IMBIV (UNC), seeds from Dept. Hayes, Puerto Falcon (Paraguay), 17 Sep 2013, *M. Scaldaferro 38* & 25 Oct 2013, *M. Scaldaferro 40* (CORD); cult. Invernadero IMBIV (UNC), seeds from Dept. Paraguarí, Carapeguá (Paraguay), 25 Oct 2013, *M. Scaldaferro 39* (CORD); cult. Invernadero IMBIV (UNC), seeds from Pairumani-Cochabamba (Bolivia), 11 Feb 2014, *M. Scaldaferro 43* (CORD); cult. Invernadero IMBIV (UNC), seeds from Mercado Abasto de Santa Cruz (Bolivia), 3 Apr 2014, *M. Scaldaferro 45* (CORD); **Salta**: Cerrillos, cult. Invernáculo de la Estación Experimental INTA, 28 Mar 1995, *A.T. Hunziker 25485, 25486 & 25488* (CORD).

UNITED STATES OF AMERICA. California: cult. in IU greenhouse, seeds from Monteagudo-Chuquisaca (Bolivia), P. Smith Ac. 1971, 14 Nov 1958, *C.B. Heiser s.n.* (CORD 00101758 & 00101759); Yolo County, University of California, campus, Truck Crops Division, 10 Dec 1948, P. Smith Ac. 771, 1949, *C.B. Heiser C 29* (CORD); cult. in IU greenhouse, seeds from Santa Cruz de la Sierra (Bolivia), 1959, *C.B. Heiser C281a, C282a* & *C290* (CORD); grown in IU greenhouse from seed colected by V. Casali at Campinas, Sao Paulo, Brazil, 29 Jul 1974, *C.B. Heiser BGH 952* (UC); cult. in IU greenhouse from seed bought at Quito market (Ecuador), 3 Jul 1963, *C.B. Heiser 4804, 4806* (CORD); grown in Davis, 1953, *P. Smith Ac. 1519* (seeds from H.C. Landes, through Chili Products, Co. Los Angeles, Chile) (UC).

2c. Capsicum baccatum L. var. umbilicatum (Vell.) Hunz. & Barboza

ARGENTINA. Córdoba: Capital, cult. casa particular Barrio Alberdi, 26 Feb 2021, *G.E. Barboza 5163* (CORD); cult. Invernadero IMBIV (UNC), semillas de frutos comprados en Córdoba (Argentina), 10 Mar 2020, *C. Carrizo García 101* (WU); cult. Jardín Experimental del Museo Botánico, 5 Mar 1990, *A.T. Hunziker 25383* (CORD); cult. propiedad Sr. Munch, 18 Mar 1992, *E.A. Moscone 197* (CORD); Argüello, cult. en casa particular, 12 Apr 1995, *R. Münch s.n.* (CORD 250); ciudad, cult. en un jardín, 4 Apr 1994, *I. Rodríguez s.n.* (CORD 241); cult. Invernadero IMBIV (UNC), seeds from Petrópolis-Rio de Janeiro (Brazil), *M. Scaldaferro 57* (CORD); Calamuchita, Santa Rosa de Calamuchita, casa de Pochi, 16 Feb 2016, *M. Scaldaferro 63* (CORD); **Corrientes**: Dept. Capital, cultivado, semillas provenientes de Salta, 1 Jul 1999, *A. Schinini 35123* (CTES, MBM, NY); **Distrito Federal**: cultivado en huerta familiar, 22 Mar 1995, *P. Melchiore s.n.* (CORD 501); **Salta**: Capital, cult. jardín Sr. Lusvarghi, *G.E. Barboza 164* (CORD); Rosario de Lerma, El Tránsito, por el camino viejo a Campo Quijano, cult. casa particular, 1350 m, 11 Jan 2005, *G.E. Barboza 1123* (CORD); ciudad, Barrio Tres Cerritos, 20 Jan 2007, *G.E. Barboza 1918* (CORD), Cerrillos, cult. en Estación Experimental del INTA-Cerrillos, 28 Mar 1995, *A.T. Hunziker 25487* (CORD); ciudad, semillas de Paraguay, *R. Neumann s.n.*, 13 Dec 1989 (CORD 75).

BOLIVIA. Tarija: Prov. Gran Chaco, Villa Montes, 6 May 1983, *A. Krapovickas & Schinini 39244* (CTES); **Santa Cruz**: Prov. Florida, Bermejo, 29 Nov 2012, *G.E. Barboza & C. Carrizo García 3648* (CORD).

BRAZIL. Amapá: Macapá, Minipolo Hortifruti da Fazendinha, 5 Aug 2008, *L.A. Pereira et al. 1824* (HAMAB, RB); Vila do Trem, Rod. BR 156 km 10, 8 Jul 2008, *L.A. Pereira et al. 1830* (HAMAB, RB); Linha D, Rodovia AP 20, Km 8, 8 Jul 2008, *L.A. Pereira 1834* (CEN, HAMAB, RB); **Goiás**: Jataí, UFG-Campus Jatobá, 1 Jul 2010, *E.V.E. Amaral 65* (CEN, HJ); **Pará**: Banco de Germoplasma de pimenta da Embrapa Amazônia Oriental, Acesso 18, 17 Oct 2011, *S.T. Rodrigues & A.V. Carvalho 483* (IAN); Belém, Banco de Germoplasma de pimenta da Embrapa Amazônia, Acesso 3, 17 Oct 2011, *S.T. Rodrigues & A.V. Carvalho 490* (BHCB); **São Paulo**: Campinas, Parque do IAC, Estufa da Botanica, 22 Dec 1999, *L.C. Bernacci 2816* (F)

COLOMBIA. Amazonas: cult. in dooryard garden in Puerto Nariño, Rio Loreto-Yacu, 14 Oct 1972, *L.L. Glenboski C-87* (COL).

3. Capsicum benoistii Barboza

ECUADOR. Loja: Pueblo Nuevo, 04º05'51"S, 79º11'55"W, 2580 m, 21 May 1978, *F. Vivar C. & Estudiantes 1066* (LOJA); **Tungurahua**: Río Verde Grande, 1500 m, 30 Mar 1956, *E. Asplund 20070* (S).

4. Capsicum caatingae Barboza & Agra

BRAZIL. Alagoas: Santana do Ipanema, Fazenda Curral do Meio, 9º22'13"S, 37º14'54"W, 10 Jul 2007, *G.B. Araujo et al. 205* (MAC); São José da Tapera, 21 Sep 2012, *G.B. Araujo 642* (MAC); Serra da Camonga, 24 Jul 2008, *E.C. Chagas-Mota 828* (MAC); Água Branca, Morro do Craunã, 9º15'58"S, 37º54'54"W, 24 Apr 2009, *E.C. Chagas-Mota & L.M. Leão 3144* (MAC); Inhapi, Serra do Grude, 8 Aug 2009, *E.C. Chagas-Mota 4711* (MAC); Morro do Craunã, 9 Aug 2009, *E.C. Chagas-Mota 4828* (MAC); Palmeira dos Índios, Serra das Pias, Fazenda Barra Nova, 18 Oct 2009, *E.C. Chagas-Mota 6286* (MAC); Minador do Negrão, Fazenda Pedra Talhada, 30 Jan 2010, *E.C. Chagas-Mota 7450* (MAC); Taquarana, Serra da Itapaiuna, 6 Feb 2010, *E.C. Chagas-Mota 7578* (MAC); Coité do Nóia, Pereiro Velho, 20 Aug 2010, *E.C. Chagas-Mota 8184* (MAC); Serra da Itapaiuna, 13 Nov 2010, *E.C. Chagas-Mota 9419* (MAC); Fazenda Pedra Talhada, 19 Aug 2011, *E.C. Chagas-Mota et al. 11047* (MAC); Traipu, Serra das Mãos, 8-21 Jul 2010, *A. Costa 367 & 405* (MAC); Morro do Craunã, 1 Jun 2014, *E. Lins 3* (MAC); Fazena Fortaleza, Serra Velha, 9º24'22"S, 36º37'59" W, 16 Aug 2001, *R.P. Lyra-Lemos & E.M. Duarte 5771* (MAC); Pão de Açúcar, caminho para Ilha do Ferro, 2 Km depois do Riacho Grande, 21 Jun 2002, *R.P.Lyra-Lemos et al. 6827* (MAC); Mata Grande, Sítio Sabonete, área próxima a casa do Sr. José Vieira, 25 Mar 2006, *R.P. Lyra-Lemos et al. 9402* (MAC); Xingó, 9º34'17"S, 36º46'55"W, 13 Sep 2008, *R.P. Lyra-Lemos 11567* (MAC); Serra das Mãos, 10 Aug 2015, *R.P. Lyra-Lemos et al. 13147 & 13177* (MAC); Boqueirão, reserva dos Índios, *R.P.Lyra-Lemos et al. 14027* (MAC); Delmiro Gouveia, 13 Jun 2014, *A.M. Miranda 6695* (BHCB, EAC, HST, HUEFS, MAC, RB, UFRN); Pariconha, Sítio Araticum, 23 Aug 2009, *M.N. Oliveira 7* (MAC); Estação Ecológica Curral do Meio, 13 Jun 2017, *A.B. Silva 1402* (MAC); REVIS do

Craunã e do Padre, Morro do Craunã, 27 Apr 2014, *E.L. Silva et al. 1* (MAC); Olho d´Água do Casado, 28 Jun 2000, *R.A. Silva 1506* (MAC); Fazenda Capelinha, 17 Jul 2000, *R.A. Silva & D. Moura 1586* (RB); Fazenda Morro Grande, 9º31'9,8''S, 37º48'54.5''W, 8 Aug 2000, *R.A. Silva & D. Moura 1632* (RB); Piranhas, Faz. Picus, 15 Jul 2001, *R.A. Silva 1793* (UFP); **Bahia**: Serra Preta, Faz. Santa Clara Nova, 12º09'24"S, 39º22'46"W, 262 m, 2 Apr 2009, *M.F. Agra et al. 7081* (CORD, JPB); Itatim, Morro do Quixaba, 12º44'21,3"S, 39º47'33,9"W, 273 m, 3 Apr 2009, *M.F. Agra et al. 7085* (CORD, JPB); Arataca, Serra do Peito-de-Moça, estrada que liga Arataca a Una, ramal ca. 22,4 km de Arataca, com entrada no Assentamento Santo Antônio, RPPN "Caminho das Pedras", 15º10'25''S, 39º20'30''W, 1000 m, 23 Sep 2006, *A.M. Amorim et al. 6317* (NY); Campo Formoso, 10º30'32''S, 40º25'59''W, 739 m, 13 Apr 2006, *V. Barreto et al. 218* (BHCB, HUEFS); Feira de Santana, Dist. São José da Itapororoca, Serra de São José, 12º38'S, 39º28'W, 18 Jun 1985, *H.P. Bautista & G.C. Pinto 1023* (CEPEC, CORD, HRB, HUEFS); Serra de São José, 12 May 2003, *C. van der Berg 986* (HUEFS); Barro Petro, Serra da Pedra Lascada, 13,7 km de Barro Preto, na estrada que passa pela fazenda São Miguel e sobe até o acesso a Serra, 14º46'13''S, 39º32'10''W, 860 m, 16 Jan 2007, *R.A.X. Borges et al. 517* (NY); Almadina, 13,8 km ao SE de Coaraci na estrada para Almadina, Faz. São José, 14º42'21''S, 39º36'12''W, 650-900 m, 16 Jan 2007, *R.A.X. Borges et al. 525* (NY); Senhor do Bonfim, encosta da Serra da Maravilha, trilha aberta pela Chesf, 650 m, 11 Jul 2005, *D. Cardoso 625* (HUEFS); Conceição do Coité, Serra do Mucambo, 11°30'S, 39°11'W, 3 Feb 2013, *D.N. Carvalho & M.O. Matos 188* (HUEFS); same locality, 26 Jun 2013, *D.N. Carvalho 283* & *308* (HUEFS); same locality, 10 Sep 2014, *D.N. Carvalho 515* (HUEFS); Salgadália Serra do Mucambo, 30 Jan 2016, *D.N. Carvalho 582* (HUEFS); Serra do Maravilha, 10º24'16''S, 40º12'36''W, 739 m, 28 Jul 2005, *R.M. Castro et al. 1229* (HUEFS, JPB); Santa Teresinha, Pedra Redonda, 7 Jul 2017, *G. Costa et al. 2617* (HSTM, HURB); Cruzeiro do Mocó, 173 m, 9 Jun 2014, *J.A. Costa 61* (HUEFS); Dist. Ipuaçu, 12º14'02''S, 39º4'34''W, 19 May 2005, *A.P.L. Couto et al. 89* (HUEFS); Ipuaçu, Face oeste, 12º13'55''S, 39º4'39''W, 30 Jun 2005, *A.P.L. Couto et al. 106* (HUEFS, SP); São Gonçalo dos Campos, Fazenda Azulão, próxima a estrada antiga para Humildes, 19 Jul 2017, *W.O. Fonseca & T.N. Alves 482* (HSTM, HURB, UFP); Ibiquera, Gruta da Lapinha, 12º27'51''S, 40º58'47''W, 610-710 m, 1 May 2004, *F. França et al. 4955* & *4956* (HUEFS); Estação da Embasa, Cachoeira/Bahia, Vale dos Rios Paraguaçu e Jacuipe, Jul 1980, *Grupo P. do Cavalo 331* (ALCB, CEPEC, HRB, HUEFS); mata a NE. da B. Bananeiras, Cachoeira/Bahia- Vale dos Rios Paraguaçu e Jacuipe, 12º32'S, 39º05'W, 40/120 m, Jul 1980, *Grupo P. do Cavalo 438* & *453* (ALCB, CEPEC, HRB, HUEFS, NY); Cachoeira/Bahia-Vale dos Rios Paraguaçu e Jacuipe, 12º32'S, 39º05'W, 40/120 m, Sep 1980, *Grupo P. do Cavalo 694* & *771* (ALCB, CEPEC, HUEFS); Salvador, Reserva de Cotegipe, 22 Sep 1994, *M.L. Guedes s.n.* (ALCB); Euclides da Cunha, Sítio do Jaime, 10º30'S, 39º00'W, 20 Mar 2004, *M.L. Guedes et al. 10847* (ALCB); Miguel Calmon, Piemonte da Diamantina, entorno do Parque Sete Passagens, Ponto 230, 11º19'56''S, 40º35'53''W, 525 m, 23 Dec 2006, *M.L. Guedes et al. 13245 & 13297* (ALCB); Serrote, Faz. do Sr. Oscar, 20 Apr 2018, *M.L. Guedes et al. 30361* (ALCB, HVC); Jacobina-Barracão de Cima, 11º01'38''S, 40º33'38''W, 662 m, 6 Jul 1996, *R.M. Harley et al. 3445* (CEPEC, HUEFS, K, SPF); 4 km north of Senhor do Bonfim, 10º27'S, 40º11'W, 550 m, 24 Feb 1974, *R.M. Harley 16297* (CEPEC, RB); Monte Alto, Caatinga de Grotão, 14º13'55''S, 39º4'34''W, 8 Jul 2006, *C.T. Lima et al. 38* (CEPEC, HUEFS); Estrada de Carrapixel à Estiva, depois do vilarejo Pau Darco, 10º27'17''S, 40º11'15''W, 540 m, 25

Jun 2007, *Lopes et al. 1422* (CEPEC); Monte Alto, 12º13'53''S, 39º4'38''W, 30 Sep 2003, *C.F. Lucca et al. 18* (HUEFS); Fazenda Cruzeiro do Mocó, 208 m, 27 May 2013, *C. Martins 242* (HUEFS); Itatim, interior da mata da base do inselberg, 12º45'12''S, 39º46'W, 300 m, 26 Jan 1997, *E. Melo et al. 1969* (HUEFS, JPB); Morro da Pedra Grande, base do morro, 12º42'57'S, 39º45'46''W, 285 m, 8 Apr 2006, *E. Melo et al. 4336* (ESA, HUEFS); Fazenda da Chapada, 12º33'40"S, 39º49'37"W, 15 Mar 2008, *E. Melo et al. 5440* (HUEFS); Inselbergue Monte Alto, 130 m, 28 Aug 2008, *E. Melo et al. 5926* (HUEFS); Riachão do Jacuípe, 11º8'38"S, 39º1'19"W, 250-275 m, 4-7 Jun 2009, *E. Melo et al. 6230 & 6295* (HUEFS); Rio Ribeirão, 478 m, 14 Dec 2012, *E. Melo 11840* (HUEFS); Riacho Sebastião ou Rio Milagres, divisa Itatim com Milagres, 488 m, 14 Dec 2012, *E. Melo 11878* (HUEFS); Fazenda Chapada, BA-052 Km 8, ramal a esquerda, 190 m, 12 Oct 2015, *E. Melo 13140* (HUEFS); Morro da Queixaba, 12º44'26''S, 39º47'37''W, 290 m, 12 Feb 2006, *A.O. Moraes et al. 159* & *160* (ESA, HUEFS); Parque da cidade, 12º15'S, 39º4'W, 17 May 2007, *M.V. Moraes 790* (HUEFS); Serra de São José, Faz. Boa Vista, 12º15'S, 38º58'W, 11 May 1984, *L.R. Noblick 3207* (CEPEC, CTES, HUEFS, MBM, NY); Serra Preta, 6 km W do Ponto de Serra Preta, Faz. Santa Clara, 12º10'S, 39º20'W, 17 Jul 1985, *L.R. Noblick & Lemos 4227* (HUEFS, JPB); Jacobina, 11º16'19"S, 40º27'48"W, 25 Aug 1980, *R. Orlandi 251* (RB); Caem, Estrada BR 324, km 4, 11º09'15''S, 40º24'19''W, 450 m, 18 Feb 2004, *G. Pereira-Silva et al. 8481* (CEN, HUEFS); Itiúba, Fazenda Experim. da EPABA, 10º43'S, 39º50'W, 27 Jun 1983, *G.C.P. Pinto & H.P. Bautista 103* (RB) & *104* (CORD, HRB, RB); Fazenda Cruzeiro do Mocó, 12°13'12'', 39°2'44" W, 211 m, 14 May 2013, *A.S. Queiroz & F. França 34* (HUEFS); Fazenda Cruzeiro do Mocó, 12°13'17'', 39°2'49" W, 185 m, 28 May 2013, *A.S. Queiroz et al. 55* (HUEFS); Santo Estevão, BR 116, 53 km SW de Feira de Santana, 200 m, 12º30'40''S, 39º22'27''W, 27 May 1987, *L.P. de Queiroz et al. 1520* (CEPEC, HUEFS, NY); Ipirá, Fazenda Nova Fanela, ca. 2.5 km S de Ipirá, 12º10'45''S, 39º46'12''W, 278 m, *L.P. de Queiroz et al. 10598* (BHCB, HUEFS, JPB); Rodovia BA-052 (Estrada do Feijão), próximo ao Galhardo, 181 m, 8 Jun 2012, *L.P. de Queiroz 15495* (HUEFS); Jaguarari, Fazenda Mata Funda, 21 Jul 1972, *F.B. Ramalho 132* (BHCB, HST, IPA); Cruzeiro do Mocó, 7 May 2014, *U.S. Santos et al. 5* (HUEFS 207390); Itatim, Pedra Grande, 12º42'59''S, 39º45'41''W, 320 m, 9 Apr 2005, *M.F.B. da Silva et al. 18* (BHCB, HUEFS); Caraíba, Apr 1994, *M. Sobral & Ganev 7646* (ICN, HUEFS, MBM); Santo Estêvão, próximo à BR 116, 12º29'55"S, 39º20'53"W, 29 Jul 2001, *V.C. Souza et al. 26687* (RB); Jeremoabo, ca. 12 km de Jeremoabo, 10º05'S, 38º09'W, 22 May 1978, *J. de Souza Silva 608* (SP); **Minas Gerais**: Monte Azul, sentido Jaíba, estrada de chão, 15º12'36''S, 42º53'49''W, 775 m, 22 Apr 2006, *D.S. Carneiro-Torres et al. 717* (BHCB, HUEFS, JPB); **Pernambuco**: Inajá, subida da Serra Negra, 1 May 1951, *D. de Andrade-Lima 51-918* (IPA); encosta da Serra Negra, 20 May 1962, *D. de Andrade-Lima 62-4055* (IPA, UFP); Arco Verde, Estação Experimental do IPA, 22 Jul 1971, *D. de Andrade-Lima 71-6395* (IPA); Buíque, Chapada São José, 21 Jun 1975, *D. de Andrade-Lima 75-8086* (HUEFS, IPA); na Reserva Biologica da As. Negra, ca. 1000 m, 20 Aug 1980, *D. de Andrade-Lima et al. 79* (IPA); Garanhuns, 7 Jul 2000, *M.V.B. de M.A. Carvalho 175* (BHCB); Venturosa, Parque Pedra Furada, 8°34'30''S, 36°52'45''W, 783 m, 17 Jan 1998, *K.C. Costa & M.J. Rodal 5* (NY, PEUFR); same locality, 4 Aug 1998, *K.C. Costa et al. 91* (NY); Reserva Biológica de Serra Negra, 21 Jul 1995, *E. Ferraz et al. 273* (NY); Parque Municipal Pedra Furada, 22 Apr 2008, *P. Gomes et al. 826* (RB); Garanhuns, ca. 58 km de Arcoverde, 8°44'2''S, 36°44'5''W, 955m, 19 Apr 2005, *E. Melo et al. 3862* (BHCB, HUEFS); Res. Ecol. Serra Negra, 23 Mar 1994, *A.M. Miranda et al. s.n.* (ALCB

026146, HST 9555, UFRN 0000959); Lagoa dos Gatos, Pau Ferrado, Serra do Urubu, 21 Apr 1994, *A.M. Miranda & Félix 1642* (ALCB, HST, UEC); Garanhuns, nascente do rio, 426 m, 6 Nov 2010, *M. Oliveira et al. 5408* (IPA); Jupi, Povoado de Neves, após a cidade de Jupi, cerca de 20 km para Garanhuns, 11 Oct 1990, *R. Pereira et al. 115* (IPA); Serra das Varas, na trilha da mata da Torre, 915 m, 21 Feb 2006, *R. Pereira et al. 2607* (BHCB, HUEFS, IPA); Ibimirim, estrada Ibimirim-Petrolândia, 3 Jun 1995, *M.F. Sales et al. 604* (JPB); Parque Nacional Serra do Catibau, trilha dos Torres, 8º34'33''S, 37º14'45''W, 740 m, 27 Jun 2007, *R.M. Santos et al. 1574* (ESAL, HUEFS); **Sergipe**: Canindé de São Francisco, Grota do Angico, Parcela A21R5, 20 Jul 2011, *L. Calado s.n.* (ASE 28115); Lagarto, Faz. Tapera do Nico, 7 May 1982, *E.M. Carneiro 371* (ASE); Poço Redondo, Serra da Guia, 17 Oct 2009, *S.M. Costa 606* (ASE); Japaratuba, Povoado Bonito, 24 Nov 2014, *S.A. Damasceno 56* (ASE, BHCB); Poço Verde, Assentamento Santa Maria da Lage, 31 Aug 2010, *E.V. R. Ferreira 150* (ASE, MAC); Mun. Porto da Folha, Porto da Folha, 10 Jul 2014, *F.B. Gonçalves 267* (ASE); Serra da Guia, 30 Sep 2019, *W.J. Machado & J.B. Jesus 738* (ASE); same locality, 23 Oct 2010, *W.J. Machado & J.B. Jesus* 787 & *800* (ASE); Assentamento Che Guevara, 5 Jul 2013, *G.M.A. Matos et al. 308* (ASE, BHCB); Curituba, Fazenda Cana Brava, 6 Dec 1999, *S. Moura 1016* (RB); Povoado Lagoa Grande, Fazenda São Pedro, 10º1'12"S, 37º14'37"W, 19 Apr 2011, *D.G. Oliveira et al. 158* (ASE, MAC); Unid. de Conser. Monumento Natural Grota do Angico, 23 Apr 2010, *A.C.C. Silva 244* (ASE); same locality, 11 May 2010, *A.C.C. Silva 259* (ASE); Monumento Natural Grota do Angico, 22 Jun 2010, *A.C.C. Silva 287* (ASE); Nossa Senhora da Glória, Faz. Olhos d'Água, 8 May 1984, *G. Viana 955* (ASE); same locality, 25 Aug 1987, *G. Viana et al. 1986* (ASE).

Cultivated
BRAZIL. Minas Gerais: Mun. Viçosa, cult. in Horto Botanico next to Herbarium VIC on campus of UF de Viçosa, 20°45'13''S, 42°52'38''W, 650 m, 23 Apr 2010, *M.F. Agra & L. Bohs 7257* (BHCB); Campos da UFV, entre as casas de vegetação da Fitotecnia, 20º45'35''S, 42º52'28''W, 680 m, 17 May 1999, *L. Bianchetti et al. 1560* (CEN); cult. en Viçosa, 13 Apr 1988, *A.T. Hunziker 25233* (CORD: 3 sheets); Campus da UFV, Grade de casas de vegetação ao lado do Agros, 30 May 2016, *D.B.B. Pereira s.n.* (VIC 47041).

5. Capsicum caballeroi M.Nee
BOLIVIA. Chuquisaca: Monteagudo, comunidad San Lorenzo, 19°56'05"S, 64°13'19"W, 1520 m, 26 Dec 2007, *C. Ortiz 3* (HSB, MO); Huacareta, Serranía Los Milagros, 20°20'21"S, 64°02'35"W, 1450 m, 23 Dec 2005, *M. Serrano et al. 6882* (HSB, MO); Tomina, c. 8 km NW of Villa Tomina, 2100 m, 31 Dec 1994, *J.R.I. Wood 9060* (NY); **Cochabamba**: Dianpampa, exposición SW, 17°40'53"S, 64°40'55"W, 2160 m, 4 Sep 2003, *D.C. Daly et al. 21* (BOLV, MO); **Santa Cruz**: La Yunga, subiendo hacia el bosque de Unga por el final del camino que lleva de la Yunga hacia los cultivos que quedan al frente de la comunidad, 18º04'33"S, 63º57'58"W, 23 May 2010, *M. Atahuachi et al. 1629* (LPB); ca. 1,5 km de El Empalme (ruta vieja Santa Cruz-Cochabamba) rumbo a Khara-Huasi, 17º50'3.2"S, 64º42'9.7"W, 1 Dec 2012, *G.E. Barboza et al. 3655* (CORD); a unos 54 m de la ruta, rumbo a La Siberia viniendo desde Comarapa, 17º49'27.4''S, 64º41'20.5''W, 2530 m, 11 Dec 2017, *G.E. Barboza 4907* (BM, CORD, LPB, SI); El Empalme, a 2,3 km del desvío rumbo hacia la comunidad de Khara Huasi, 17º50'03.2''S, 64º42'10''W, 2439 m,

11 Dec 2017, *G.E. Barboza 4908* (CORD); Siberia, entre El Empalme y Locotal, 17º50'38"S, 64º41'34"W, 2100-2850 m, 8 Apr 2004, *E. Calzadilla et al. 111* (NY, USZ); Siberia a 12 Km de Torrecillas, del campamento a unos 2500 metros hacia abajo por camino viejo, 17°49'34"S, 64°40'23"W, 2580 m, 3 Nov 2003, *J.A. Carrasco et al. 147* (MO, NY, RB, USZ); Serranía Siberia, 20-35 km W of Comarapa on the old Cochabamba-Santa Cruz road (Hwy 4), 17°54'S, 64°29'W, 2000 m, 14 Jan 1990, *L.J. Dorr & L.C. Barnett 7041* (CORD, LPB, MO, NY); carretera antigua a Santa Cruz, camino del empalme a Dianpampa y San Mateo, Chua Kocha, 17°49'13"S, 64°36'37"W, 2360 m, 15 Jun 2003, *S. Durán et al. 1771* (BOLV, MO); Siberia, Chua Kocha, 17°49'10"S, 64°42'29"W, 2220 m, 22 Jun 2003, *E. Fernández T. et al. 1896* (MO); Astillero, exposición SW, 17°49'26"S, 64°41'22"W, 2550 m, 14 Aug 2003, *E. Fernández T. et al. 2007* (BOLV, MO); PN Amboró, Cerro Bravo, cerca Comarapa, 17°39'52"S, 64°35'04"W, 2600 m, 17 Jun 1995, *A. Jardim et al. 1995* (MO, NY, USZ); camino a Siberia, 25 km de Comarapa, alrededores del campamento, 17º49'42"S, 64º40'34"W, 2450 m, 5 Nov 2003, *C.G. Jordán et al. 517* (USZ); 1.5 km down from El Empalme (on Comarapa-Cochabamba highway) on road to Khara Huasi, 17°50'24"S, 64°42'12"W, 2475 m, 2 Aug 2003, *M. Nee et al. 52407* (CORD, LPB, MO, NY); La Yunga, 7.5 km (línea recta) NE de Mairana, 18°05'S, 63°55'W, 1880 m, 15 Mar 1997, *M. Saldías P. 4977* (MO); 50 km al norte del Mataral (en la carretera Santa Cruz-Comarapa) pasando por San Juan del Potrero y bajando a la cuenca del alto Río Ichilo, 17°43'S, 64°13'W, 2300-2450 m, 28 May 1989, *D.N. Smith et al. 13470* (BOLV, LPB, MO, NY, USZ); PN Amboró, Siberia-El Empalme, 5 km entrando hacia Khara Huasi; carretera entre Comarapa-Cochabamba, 17°50'S, 64°43'W, 2300 m, 8 May 1992, *I.G. Vargas C. & E. Prado 1282* & *1286* (MO, NY, USZ); PN Amboró, San Juan del Potrero, Naranjos, 17°53'S, 64°26'W, 2150 m, 12-13 May 1992, *I.G. Vargas C. et al. 1343* (MO, NY, USZ); PN Amboró, proximidades del Cerro Bravo a 10 km al N de Comarapa, 17°49'05"S, 64°32'05"W, 2400-2500 m, 7 Apr 1994, *I.G. Vargas C. & J.M. Camacho 3118* (CORD, MO, NY, US, USZ); same locality, 17°49'04"S, 64°33'W, 2400-2500 m, 15 Nov 1995, *I.G. Vargas C. et al. 4151* (MO, NY, USZ); Siberia, 25 km desde Comarapa por la ruta Comarapa-Cochabamba, 17°49'31"S, 64°40'30"W, 2550 m, 4-6 Nov 2003, *I.G. Vargas C. & C.G. Jordán 7000* (BM, CORD, MEXU, MO, NY, RB, USZ); La Yunga de Mairana, zona del Hueco de la palma, 18°03'51"S, 63°55'03"W, 1200 m, 26 May 2005, *I.G. Vargas C. et al. 7233* (LPB, MO, USZ); c. 11-12 km N of Comarapa, on sides of Cerro Bravo, 2200 m, 23 May 1996, *J.R.I. Wood 11102* (LPB); subiendo de Comarapa hacia Cerro Bravo, pasando ca. 1.5-2 km del cruce a Laguna Verde, 17º51'3"S, 64º32'8.4"W, 20 Nov 2005, *J.R.I. Wood & M. Mendoza 22105* (USZ).

6. Capsicum campylopodium Sendtn.

BRAZIL. Espírito Santo: Venda Nova do Imigrante, 3 Nov 1974, *P.L. Krieger 13410* (BHCB, CEN); Conceiçao de Castelo, estrada de terra Castelo-Caxixe, a 2 km do pavimento, 20º36’S, 41º11’W, 600 m, 28 Mar 1986, *E. Lleras Pérez et al. 2161* (CEN, CORD; = *A.T. Hunziker 25135*, CORD); estrada de terra Castelo-Caxixe, a 1 km de Caxixe Quente em direcao a Caxixe Alto, 20º36’S, 41º 05’W, 700 m, 28 Mar 1986, *E. Lleras Pérez et al. 2172* (CEN, CORD; = *A.T. Hunziker 25134*, CORD); **Minas Gerais**: Caratinga, Estacion Biológica Caratinga, Mata do Joaó, 20 Mar 1984, *P.M. Andrade & M.A. Lopes 109* (RB); Mata do Jaó, 5 km da Fazenda Montes Claros, 430 m, 19º47’S, 42º07’W, 27 Mar 1986, *E. Lleras Pérez et al. 2152* (CEN, CORD; = *A.T. Hunziker 25129*) *& 2157* (CEN,

CORD; = *A.T. Hunziker 25130*); Viçosa, mata da Prefeitura (Paraíso), en sub-bosque, 18 Dec 1988, *G.A.R. de Melo s.n.* (RB 338690); Coimbra, 2 km depois do Córrego Estiva, 4 Dec 1997, *A. Salino 3850* (BHCB); Bairro Inácio Martins ("Grota dos Camilos"), 25 Feb 1987, *M.F. Vieira 542* (VIC); **Rio de Janeiro**: Guanabara, Bico do Papagaio, Floresta da Tijuca, 26 Oct 1972, *J. Almeida de Jesus 2064* (CORD, RB); próximo da Restinga, Estrada da Tijuca, 22 Sep 1972, *J. Almeida de Jesus 1959* (CORD, RB); Niteroi, Itaipuaçú, Pico Alto Moirão, 9 Sep 1982, *R. Andreata et al. 522* (CORD, RB: 3 sheets); Petrópolis, Reserva Biológica de Tinguá, km 3/4, a 9.6 km de la Estrada Roscio/Pati do Alferes, 1350 m, 19 Apr 2006, *G.E. Barboza et al. 2057* (BM, CORD, MO, NY); Parque Estadual da Serra da Tiririca, Córrego dos Colibris, parte alta, próximo à pedra da Cortina, 21 Oct 1999, *A.A.M. de Barros et al. 835* (RB); Parque Estadual da Serra da Tiririca, Córrego dos Colibris, parte alta na cumeeira, 200-250 m, 14 Jan 2005, *A.A.M de Barros et al. 2367* (RB); Vista Chineza, 1955, *G. Barroso s.n.* (RB 92768); Magé, BR 116, km 91.5, estrada Teresópolis-Mage, 22º32'S, 43º02'W, 800 m, 7 Apr 1986, *L. Bianchetti et al. 399* & *400* (CEN, CORD); Parque Nacional da Tijuca, trilha do pico da Tijuca, 22º56'53''S, 43º17'30''W, 680 m, 18 May 1999, *L. Bianchetti et al. 1562* (BHCB, CEN); a 7,6 km do entroncamento de Santa Maria Madalena-Concei o do Macabu, 21º59'30''S, 41º58'50''W, 600 m, 21 May 1999, *L. Bianchetti et al. 1566* (CEN); Parque Municipal Ecológico da Prainha, entre o Mirante do Cruzeiro do Sul e o Morro dos Caetés, 28 Oct 2003, *M. Bocayuva & A. Calvente 91* (RB); Mangaratiba, Reserva Ecologica (RE) Rio das Pedras, trilha para a Lagoa Seca, 450 m, 12 Jul 1997, *M.G. Bovini 1211* (RB); RE Rio das Pedras, trilha para o Toca da Aranha, 23 Nov 1998, *M.G. Bovini & J.M. Braga 1566* (RB) & 23 Nov 2001, *M.G. Bovini et al. 2100* (RB); same locality, 108 m, 10 Feb 2009, *M.G. Bovini & C.M. Mynssen 2729* (RB); prope Rio de Janeiro, km 29, Rio-Petropolis, 9 Nov 1815, *C.B. Bowie & A. Cunningham s.n.* (BM 1016665, K); Vista Chineza, 1928, *A.C. Brade s.n.* (GH, R 25857, RB, US 1570363); Açuda da Solidão, 10 Nov 1948, *A.C. Brade et al. 19148* (LIL, MO, NY, RB 69117, UC, US); RE Rio das Pedras, trilha da Toca da Aranha, 20 Oct 1997, *J.M.A. Braga 4361* (RB); Parque Municipal Ecológico da Prainha, entre o Mirante do Cruzeiro do Sul e o morro dos Caetés, ca. 350 m, 13 Nov 2003, *J.M.A. Braga et al. 7273* (CEN, CEPEC, F, MO, NY, RB, SP); Teresópolis, Serra dos Órgãos, 23 Jan 1943, *E.A. Bueno 47* (R); Floresta Nacional da Tijuca, trilha do Bico do Papagaio, 30 Nov 2012, *M.O. Bünger & M.C. Souza 635* (BHCB); Frechal, between Majé and the Organ Mountains, [22°33'S, 43°02'W], *W.J. Burchell 1913* (K); Parque Nacional da Tijuca, caminho das Furnas, ca. 450 m, 23 Oct 1977, *P.P. Carauta & P. Maas 2730* (CORD, GUA); Floresta da Tijuca, Estrada do Excelsior, 9 Oct 1963, *A. Castellanos 24386* (RB); Estrada das Paineiras, 18 Oct 1931, *J. Cruz 27* (F, R); Guanabara, Recreio dos Bandeirantes, Mar 1963, *A.P. Duarte 7666* (RB, NY); Corcovado, 12 Oct 1904, *P. Dusén 5072* (CORD fragment, GH, M, S), same locality and date, *P. Dusén s.n.* (US 01915097); Mesa do Imperador, 8 Oct 1946, *A. Edmundo 360* (CORD, RB); Pico Alto Moirão, 16 Nov 1983, *C. Farney et al. 364* (RB); Parque Estadual Maciço da Pedra Branca, Jacarepaguá, crescendo na beira da trilha para a Casa Amarela, 13 Feb 2016, *C.D.M. Ferreira & P. Feliz 233* (RB); Paineiras, 5 Oct 1879, *R. Galvão* 137 (P); Represa dos Guinle, 1100 m, 31 Jan 1978, *A. Gentry & A.L. Peixoto 913* (CORD, MO, RB, W); Floresta da Tijuca e Petropolis, Jacarepaguá, 29 Dec 1869, *A. Glaziou 4168* (C, K, P: 3 sheets); an Retiro, 21 Jan 1877, *A. Glaziou 8872* (CORD, G, K, P, R); Floresta da Tijuca, 20 Nov 1878, *A. Glaziou 11385* (K, P: 2 sheets); am Alto da Imperatriz, 2 Feb 1880, *A. Glaziou 12108* (CORD, G, K, LE, P); Corcovado, 7 Sep 1915, *F.C. Hoehne s.n.* (SP30097, US1617147); same locality, 7 Nov 1915, *F.C. Hoehne*

s.n. (US 1617147); Corcovado, 15 Nov 1946, *F.W. Hunnewell 18784* (GH, NY); Floresta de Tijuca, ca. 600 m, 6/7 Dec 1967, *A.T. Hunziker 19572* (CORD, P), *19573* (CORD, MO), *19574*, *19575 & 19576* (CORD); PN Serra dos Órgãos, por BR 116, desde Teresópolis a Rio, en el Km 91,5, 800 m, 7 Apr 1986, *A.T. Hunziker 25160* (BM, CORD, NY); a 8 km de la unión de BR 40 (km 77) con la ruta entre Rocio y Pati de Alferes, 1220 m, 9 Apr 1986, *A.T. Hunziker 25166* (CORD, P); Serra do Camborá, 15 Dec 1942, *J.G. Kuhlmann 6288* (CORD, RB); Valença, *J.A. Lira Neto s.n.* (GH, RB, R 25654, US 1570213); RE de Rio das Pedras, trilha para o Cambucá, 19 Oct 1996, *J.A. Lira Neto et al. 444* (RB, RUSU); Maciço da Tijuca, Reserva Florestal da FEEMA, 400 m, 17 Oct 1977, *P.J. Maas & P. Carauta 3275* (CORD, NY, RB); Paraty, mata do lado direito da trilha para Praia de Martim da Sá (em direção ao Cairuçú na APA- CAIRUÇÚ), 200 m, 10 Nov 1990, *R. Marquete et al. 260* (RB); Magé, Estação Ecologica Estadual de Paraiso, Centro de Primatologia do RJ, Rio Paraíso, 190 m, 23 Nov 1984, *G. Martinelli et al. 10405* (CEPEC, NY, RB, SPF); D.F., Pico do Papagaio, 19 Nov 1946, *L.E. de Mello Filho 502* (R, 7 sheets); Madalena, morro atraz do 44 Horto Santos Lima, 19 Jan 1957, *L.E. de Mello Filho 1187* (R); the Organ Mountains [Serra dos Órgãos], *J. Miers 4541 pp* (right branch, K); Alto da Boa Vista, Estrada Vista Chinesa, km 2, 200 m, Dec 1976, *M.S. Moraes 18* (CORD, GUA, MO); D.F., Alto da Boa Vista, Paineiras, Jan 1958, *A.X. Moreira s.n.* (R 103801); Parque Estadual da Serra da Tiririca, Córrego dos Colibris, parte alta da trilha, na clareira antes de entrar na mata do topo, 27 Feb 2002, *H.P. Moreira et al. 84* (RB); Distrito de Caxambú, Represa Caxambú Grande, PARNASO, 22º30'40"S, 43º7'0"W, 1097 m, 13 Jan 2009, *W.M. Nadruz et al. 2251* (RB); Serra da Carioca, 14 Dec 1944, *P. Occhioni s.n.* (CORD 00087794, RB 53049); Sacopan, Morro da Saudade, 14 Nov 1946, *Octavio s.n.* (CORD 00087795, RB 00461398); Nova Friburgo, estrada para Friburgo meio da Serra, 500 m, 25 Dec 1962, *G. Pabst & E. Pereira 7225* (CORD, R, US); matas da Vista Chinesa, 24 Oct 1999, *C.H.R. de Paula & R. Bacelar 203* (RB); Guanabara, Estrada do Cristo, 30 Oct 1958, *E. Pereira et al. 4444* (HB, RB, US); Serra d' Estrella, *J.B. Pohl s.n.* (CORD, G, W); Tijuca, 1872, *T.A. Preston s.n.* (K001071995); Corcovado, 1872, *T.A. Preston s.n.* (K001071996); Itaipuaçu, Pico Alto Moirão, 9 Sep 1982, *J.R. Profice et al. 22* (CORD, GUA); caminho da Pedra do Archel (Alto da Boa Vista), 19 Dec 1946, *J.A. Rente 31* (R, 4 sheets); Serra dos Órgãos, *Richard 528* (CORD, P); prope Mandioca, Jan. 1823, *L. Riedel s.n.* (NY); Serra d'Estrella, in sylva umbrosis humis, Feb 1923, *L. Riedel s.n.* (NY 04141313, NY 0414314); Serra do Mendanha, Estrada para as antenas de Furnas, 13 Nov 2011, *I.M.C. Rodrigues et al. 473* (BHCB); Paulo e Virginia, Tijuca, 11 Dec 1946, *M. Rosa 107 & 111* (R); Rio de Janeiro, 1816/1821, *A. de Saint-Hilaire Cat. A2, N° 58* (P00410019); Rio de Janeiro, 1816/1821, *A. de Saint-Hilaire Cat. A1, N° 651* (P00410020, P00410021); D.F., Paineiras, 28 Nov 1879, *J. de Saldanha da Gama 5373* (R, RB); D.F., Comarim, 19 Dec 1933, *A. Sampaio et al. s.n.* (R 113699); Serra d'Estrella, *H.W. Schott 5409* (F, GH, W); Alto da Boa Vista, Estr. Vista Chinesa, km 2, em frente ao CBRI, do outro lado da estr. numa picada, 11 Nov 1982, *D.S. Souza Pedroza et al. 658* (CORD, GUA, MO); Guanabara, matas do Corcovado, ± 450 m, 22 Oct 1969, *D. Sucre 6114* (BM, CORD, RB); mata rupícola da vertente sul da Serra da Piaba, 50-100 m, 4 Nov 1969, *D. Sucre 6191* (F, MBM, RB); Serra do Mendanha, ± 450 m, 27 Nov 1969, *D. Sucre & P.I. Braga 6402* (CORD, RB); matas da subida do Pico da Tijuca, 750 m, 21 Jan 1971, *D. Sucre 7387* (CORD, RB); Guanabara, grota do Pae Ricardo, 500-550 m, 22 Dec 1971, *D. Sucre 8156* (CORD, RB); Corcovado, ± 490 m, 18 Jan 1972, *D. Sucre 8238* (CORD, RB); Rio de Janeiro, 1893, *E. Ule s.n.* (CORD 00087793, R 25896, US 1570401); Serra dos

Órgãos, 16 Dec 1891, *E. Ule 2416* (R); Walde des Corcovado, *E. Ule 3273* (CORD); Corcovado, *A-C. Vauthier s.n.* (P 00482079); Fazenda Guinle, 23 Jan 1947, *H. Velloso s.n.* (CORD 00087812, R 92935); Fazenda Guinle, 22 Jan 1943, *H. Velloso 556* (CORD, R); Mendes, Reserva Florestal dos Irmãos Maristas, 6 Mar 1980, *Vianna 1509* (CORD, GUA); mata do Horto Florestal, 18 Nov 1922, *Victorio s.n.* (CORD, RB 00722096); Organ Mountains, 1838-1842, *C. Wilkes s.n.* (GH, US); Paineiras, ca. 300-500 m, 11 Oct 1927, *H. Zerny s.n.* (W 15091).

Cultivated
BRAZIL. Minas Gerais: Viçosa, casa de vegetação com coleção de pimentas do Prof. V. Casali (sementes de serra de Petrópolis-RJ, na BR-040 entre Xerém e Belvedere), 649 m, 22 Apr 1986, *L. Bianchetti et al. 511* (CEN, CORD); cult. en Univ. Fed. Viçosa por Prof. V. Casali (semillas de Petrópolis, RJ), 24 Mar 1986, *A.T. Hunziker 25116* (CORD); cult. en Viçosa por Prof. V. Casali (semillas de E. Lleras Pérez et al. 2157), 7-14 Dec 1986, *A.T. Hunziker 25244* (CORD); cult. en Viçosa por Prof. V. Casali (semillas de E. Lleras Pérez et al. 2152), 7-14 Dec 1986, *A.T. Hunziker 25245* (CORD).

7. Capsicum carassense Barboza & Bianch.
BRAZIL. **Minas Gerais**: Catas Altas, Serra do Caraça, near Santa Barbara, trilha a Capela y Gruta de Lourdes, 20º05'39''S, 43º28'45''W, 1430 m, 26 Apr 2010, *M.F. Agra et al. 7268* (BHCB, JPB, RB, UT); Ouro Preto, Mina da Capanema (Mina da Serra Geral), RPPN Vale, near town of Glaura, trail to Cabeza do Macaco, 20º13'04''S, 43º35'05''W, 1691 m, 29 Apr 2011, *M.F. Agra et al. 7340* (BHCB); Barão de Cocais, Parque Natural do Caraça (PNC), a 150 m do monastério, perto da Fonte do Bode, 1250 m, 19º56'S, 43º28'W, 22 Apr 1986, *L. Bianchetti et al. 512* (CEN); Barão de Cocais, PNC, 31 May 1992, *L. Bianchetti et al. 1363, 1364, 1367, 1368* (CEN); estrada da torre-Samarco Mineração-Antonio Pereira, 16 Dec 1996, *M. Brosehel & J. Craig 406* (RB); Santa Barbara, Serra de Gandarela/C2, 20º3'24''S, 43º41'28.60''W, 1637 m, 26 Nov 2008, *F.F. Carmo & L.C. Ribeiro 3527* (BHCB); Serra do Caraça, ca. 70 km suleste de Belo Horizonte, próximo ao Mosteiro do Caraça, 17 Nov 1977, *N.D. da Cruz et al. 6291* (SP, RB, UEC); Santa Barbara, 20º00'52''S, 43º40'13''W, 1497 m, 17 Dec 2014, *F.D. Gontijo et al. 589* (BHCB); PNC, muy cerca del monasterio, en el bosquecillo vecino al Fonte de Bode, ca. 1250 m, 21 Apr 1986, *A.T. Hunziker et al. 25206* (CORD); PNC, muy cerca del monasterio, 1250-1300 m, 10 Dec 1986, *A.T. Hunziker et al. 25256* (CORD, BM); Serra do Caraça, 12-14 Dec 1978, *H.F. Leitão Filho et al. 9546 & 9725* (UEC); habitat in irriguis lapidosis Serra do S. Geraldo, w/d, *C.F.P. von Martius s.n.* (M 0171537); Serra do Caraça, 4 Dec 1999, *R.C. Mota 106* (BHCB); same locality, 18 Dec 2002, *R.C. Mota 2260* (BHCB); same locality, 5 Jan 2005, *R.C. Mota 2653* (BHCB); RPPN Caraça, trilha para Tanque Grande, 03 Dec 2013, *J. Ordones et al. 2229* (BHZB); RPPN Capanema, 19 Oct 2015, *M.O. Pivari et al. 2768* (BHCB); Serra do Caraça, 1600 m, 12 Sep 1990, *J.R. Stehmann et al. s.n.* (ESA 33757); Serra do Caraça, Tanque Grande, 16 Feb 2012, *J.R. Stehmann & F.S. Faria 6269* (BHCB); na trilha para o Tanque Grande, 20º06'05''S, 43º29'33''W, 1249 m, 26 Oct 2014, *J.R. Stehmann et al. 6344* (BHCB, RB); Rio Acima, Serra do Gandarela, 20º05'52''S, 43º41'12''W, 12 Dec 2011, *C.V. Vidal & R.L. de Paula 1157* (BHCB).

8. Capsicum cardenasii Heiser & P.G.Sm.
BOLIVIA. La Paz: Pucuma, antes de llegar a Luribay, entrando por el desvío de Belén**,** 17°7'23''S, 67°35'54''W, 2825 m, 4 Dec 2017, *G.E. Barboza 4881 & 4882* (BM, CORD, LPB); Peña Colorada, a 2,1 km antes de llegar a Luribay, 17°4'27''S, 67°38'50''W, 2623 m, 4 Dec 2017, *G.E. Barboza 4884* (CORD); Loayza, 17º4'S, 67º40'W, 2500 m, 5 Apr 1994, *S. Beck 130 PG94* (CORD, LPB, M); Pucuma, on road between Belen and Luribay, ca. 10 km southwest of Luribay, 2930 m, 10 Mar 1971, *W.H. Eshbaugh 614* (MU); Pisco, 1 km from Luribay on road to Patacamaya, 2800 m, 9 Apr 1987, *W.H. Eshbaugh 2046 C* (CORD); same locality, 1 Aug 1990, *W.H. Eshbaugh 2046 J* (CORD); mitad del camino de Luribay a Ayo Ayo, 17º03'46.8"S, 67º41'13.2''W, 2834 m, 1 Mar 2003, *T. Ortuño* 271 (LPB); **Tarija**: Arce, Padcaya, 5.4 km beyond Padcaya to river crossing called Zaire, 18 Mar 1980, *R. J. Hickey & Eshbaugh, W.H. s.n.* (GH, MU).

Cultivated
ARGENTINA. **Córdoba**: Capital, cult. casa particular I. Rodríguez, semillas obtenidas del Jardín Botánico de Nijmegen, 2 Nov 1994, *I. Rodríguez s.n.* (CORD 00087965); **UNITED STATES OF AMERICA**. **California**: Yolo Co., University of California at Davis, Department of Vegetable Crops greenhouse, seeds sent by M. Cárdenas to P.G. Smith, from Sorata (Bolivia), 1965, *P.G. Smith s.n.* (DAV Ac. 1793) (CORD 00088580-00088583, DAV); **Indiana**: cult. at IU, fruit purchased at street market, La Paz (Bolivia), origin unknown, 8 Mar 1980, *W.H. Eshbaugh 1526* (MU); cult. at IU greenhouse, fruit from market La Paz, said to come from Collana, 1 Aug 1990, *W.H. Eshbaugh 1527* (CORD); Bloomington, cult. at Indiana University (IU) greenhouse, 13 Nov 1955, *C.B. Heiser s.n*. (leg. P. Smith AC 1793) (IND 139343); cult. at IU greenhouse, fruit from Finca de Fadrique Muñoz Reyes, Río Abajo, Murillo (La Paz, Bolivia), 30 Nov 1959 (CORD, US), 30 Dec 1959 & 21 Mar 1960 (CORD), *C.B. Heiser C271*; cult. at IU greenhouse, fruit through M. Cárdenas, 1 Jun 1956 & 3 Feb 1959, *C.B. Heiser 4196* (CORD), 8 Jul 1957 & 28 Jan 1959, *C.B. Heiser 4196* (US, LIL); grown IU greenhouse, collected from La Paz, Bolivia market, said to be from "Rio Abajo", 10 Apr 1962, *P.G. Smith SA 268* (MU), 23 Jan 1962, *P.G. Smith SA 269* (MU); IU greenhouse, from La Paz market, 27 Sep 1963, *P.G. Smith SA 406* (MU); **Missouri**: Saint Louis, cult. at Missouri Botanical Garden, seed from market La Paz (J. Solomon 5205, not vouchered), 1 May 1989, *W. D'Arcy 17716* (MO); cult. at Missouri Botanical Garden, fruit purchased in Mercado Lanza (La Paz, Murillo, Bolivia), 7 Mar 1980, *J.C. Solomon 5304* (LPB, MO).

9. Capsicum ceratocalyx M.Nee
BOLIVIA. La Paz: Huancané, 6.5 km hacia el sud sobre camino nuevo, 2280 m, 8 Mar 1980, *S. Beck 3051* (LPB, CORD); cerca de Chulumani, Apa Apa, subiendo de la Estancia 1,5 km en línea recta hacia el monte, a mano derecha del camino, 16º20'S, 67º30'W, 2040 m, 27 May 2001, *S. Beck 28089* (LPB); frente de Florida, otro lado el Río Unduavi, hacia la finca La Glorieta, 16º22'S, 67º44'W, 1500 m, 11 Apr 2009, *S. Beck 33135* (LPB); Valle de Unduavi, frente a Yanacachi, 2100 m, 1 Apr 1988, *R. Seidel & I. Hinojosa 1267* (CORD, LPB); basin of Rio Bopi, Asunta (near Evenay) , 690-750 m, 27/31 Jul 1939, *B.A. Krukoff 10584* (A, F, G, GH, LIL, LP, LPB, MO, NY, US, S); borde alto del Río Unduavi, 4.6 km below Yolosa, then 19.1 km on road up the Río Huarinilla, 16°12'S, 67°53'W, 1700 m, 12 Nov 1982, *J.C. Solomon 8844* (CORD: 3 sheets, F, MO, SI); 14.3 km SW (above) Yolosa

on road to Chuspipata, 16°14'S, 67°47'W, 2000 m, 23 Mar 1984, *J.C. Solomon 12086* (LPB, MO); **Cochabamba**: Prov. Ayopaya, Rio Tocorani, 2500 m, Jul 1911, *T. Herzog 2289* (CORD, L, S).

10. Capsicum chacoense Hunz.

ARGENTINA. Catamarca: Paclín, San Antonio, 25 Jan 1957, *S.A. de Ance 81* (SI); La Guardia, 1917-1918, *E. Agusti s.n.* (BA 2209); Quebrada de Sébila, a± 15 Km antes del desvío a Pomán, entre Km 1145/1146, 28º15'S, 66º16'W, 22 Apr 1994, *G.E. Barboza et al. 86* (CORD); a 1 km saliendo de San Pablo rumbo a Concepción, 28º41'55''S, 66º02'53"W, 820 m, 23 Feb 2003, *G.E. Barboza et al. 632* (CORD); entrada a San Pablo viniendo de Concepción, 28º41'52''S, 66º02'38"W, 23 Feb 2003, *G.E. Barboza et al. 638* (CORD); subiendo la cuesta de Los Ángeles, entre km 12 y 13, 28º34'56''S, 65º56'07"W, 930 m, 24 Feb 2003, *G.E. Barboza et al. 645* (CORD); desde Andalgalá rumbo a Potrero de Santa Lucía, 27º31'32''S, 66º19'51''W, 1271 m, 31 Mar 2017, *G.E. Barboza et al. 4803* (CORD); San Rafael, 6 Oct 1946, *A. Brizuela 343* (LIL); Puesto de Zabafeld, 4 Jan 1947, *A. Brizuela 627* (LIL); Olta, 15 Jan 1947, *A. Brizuela 664* (LIL); Santo Domingo, 9 Jan 1947, *A. Brizuela 821* (CAS, LIL); La Brea, 24 Feb 1947, *A. Brizuela 837* (CAS, LIL, MO); El Río de la Dorada, 7 Mar 1947, *A. Brizuela 936* (E); El Barrial, 23 Nov 1949, *J. Brizuela 70* (LIL); El Puesto de Pilimon, 26 Jan 1947, *J. Brizuela 196* (LIL, MO); Puestos Los Riojanos, 1 Feb 1947, *J. Brizuela 229* (G, LIL); Monte Redondo, 60 m, 11 Jan 1950, *J. Brizuela 338* (LIL); Santo Domingo, 13 Jan 1950, *J. Brizuela 410* (LIL); La Brea, 14 Jan 1950, *J. Brizuela 475* (CORD, LIL); Las Palomas, 5 Mar 1950, *J. Brizuela 875* (CORD, LIL); Santa Lucía, 2 Apr 1950, *J. Brizuela 1009* (CORD, LIL); La Higuerita, 28 Mar 1950, *J. Brizuela 1066* (G, LIL); El Potrero, 30 Mar 1950, *J. Brizuela 1127* (LIL); Km 981, 4 Apr 1950, *J. Brizuela 1174* (LE, LIL); Puesto de Lobo, 11 Apr 1950, *J. Brizuela 1217* (CORD, LIL); El Bañado Riojano, 14 Apr 1950, *J. Brizuela 1225* (LIL);El Cajón, 18 Apr 1950, *J. Brizuela 1284* (LIL); La Brea, 20 Apr 1950, *J. Brizuela 1312* (CORD, LIL); Santa Lucía, 2 Apr 1960, *J. Brizuela 1609* (LIL); La Banda, 26 Feb 1929, *A.L. Cabrera 1132* (LP); Quebrada de Belén, 4 Mar 1971, *A.L. Cabrera et al. 21831* (LP); 14 km SE of Andalgalá, via the road to Cuesta de la Chilca, 1050 m, 28 Feb 1973, *P. Cantino 698* (CORD, GH, SI); Londres de Quimivil, El Shincal, 13 Feb 2000, *A. Capparelli 158* (LP); Pomancillo, 14 Jan 1940, *A. Castellanos s.n.* (BA 33921, LIL); alrededores de la Capital, 20 Oct 1910, *L. Castillón 24* (SI); Capital, 20 Oct 1910, *L. Castillón 14331* (LIL); Sierra del Ambato, entre Chumbicha y Masa, 28º46'28''S, 66º22'60''W, 2730 m, 22 Feb 1998, *A.A. Cocucci 978* (CORD); Amadores, 17 Nov 1946, *M.J. Dimitri & B. Piccinini 56* (BAB); Colpes, 40 Km vía Singuil, 980 m, 15 Feb 1971, *H. Ellenberg 4501* (CORD, LPB); Valle Los Ángeles, 1350 m, Jan 1953, *Falcone & Castellanos 262 & 5023* (BAF); Sierra de Ambato (falda E), quebrada de San Jerónimo, a unos 5 km al NO de Chumbicha, 700/800 m, 21 Feb 1964, *A.T. Hunziker & E. Di Fulvio 16995* (CORD); Sierras de Ancasti, ruta 66, cerca mitad camino entre Ancasti e Icaño, 700 m, 23 Feb 1964, *A.T. Hunziker & E. Di Fulvio 17129* (CORD); Sierra de Ambato (falda E), subiendo por la cuesta entre Miraflores y Los Angeles, 1100 m, 27 Nov 1965, *A.T. Hunziker et al. 18343* (CORD); Quebrada del Río El Tala, RP 4, rumbo a Rodeo, ca. 1000 m, 23 Feb 1971, *A.T. Hunziker 21016* (CORD); La Estancita, 9 May 1979, *P.R. Legname 5786* (BAA, LIL); La Candelaria, 28º39.696'S, 65º26.499'W, 699 m, 3 Feb 2014, *G.J. Martínez 1185* (CORD); camino de Frías a Infanzón, 4 km de La Chacra, 3 May 1959, *J. Morello & A.R. Cuezzo 1004* (LIL);

Pomancillo, Feb 1941, *L.R. Parodi 14133* (LP); entre La Bajada y Amadores, Ruta 38, 28°20'54.2"S, 65°36'41.1"W, 744 m, 26 Feb 2008, *J. Pensiero et al. 7511* (CTES, SF, SI); La Rinconada, 56 Km de Catamarca, base W de la Sierra de Graciana, 1100-1150 m, 25 Mar 1995, *C. Saravia Toledo 12812* (CTES); RN 60, 350 m antes del cruce de RN 60 y RN 38, camino a Capayán, 28º52'20''S, 66º14'37''W, 28 Mar 2013, *M. Scaldaferro 22* (CORD); saliendo de San Pablo, 1 km camino a Concepción, 28º41'51''S, 66º02'37''W, 28 Mar 2013, *M. Scaldaferro 23* (CORD); sobre ruta 38, saliendo de Catamarca hacia S.M. de Tucumán, 28º28'14''S, 65º38'04''W, 28 Mar 2013, *M. Scaldaferro 24* (CORD); entre la Aguada y el Fuerte de Andalgalá, Nov 1877, *F. Schickendantz 74* (CORD); entre la Aguada y el Fuerte de Andalgalá, Jan 1876, *F. Schickendantz 158* (CORD); Aguada de Andalgalá, *F. Schickendantz 187* & *211* (CORD); Puntilla de Choya, Oct 1873, *F. Schickendantz 299* (CORD, GOET); Capital, *Schunck 9711* (LIL, UC); Andalgalá, 2 Mar 1939, *M.I. Scott de Birabén & M. Birabén 1143* (LP); próximo a Choya, 1451 m, 22 Jan 2007, *A.C. Slanis et al. 194* (LIL); alrededores de Belén, 1300 m, 22 Jan 1952, *H. Sleumer & F. Vervoorst 2349* (G, LIL, US); Catamarca, Jan-Feb 1910, *P.L. Spegazzini s.n.* (BAB 33226); a 10 km de Catamarca, antes del Río Fariñango, 28 Feb 1986, *R. Subils 3876* (CORD); entre Esquiú y Portillo Grande, 20 Feb 1977, *N.S. Troncoso 1741* (SI); falda E de las Sierras de Ambato, Las Rejas (Quebrada del Rio El Tala), 20 Apr 1973, *E.A. Ulibarri 456* (CORD, SI); al costado de la RP 47, 2.6 km al norte de Chuquiago, 27º32'37''S, 66º21'28''W, 1128 m, *J.D. Urdampilleta et al. 693* (CORD, CTES); Saujil, El Potrero, quebrada Río Saujil, 1600 m, 18 Feb 1952, *F. Vervoorst 3535* (LIL, US); **Chaco**: Quitilipi, 2 Jan 1945, *R.M. Aguilar 136* (LIL); Margarita Belén, Dec 1945, *R.M. Aguilar 568* (LIL) & 22 Feb 1946, *R.M. Aguilar 721* (LIL, P, S); Paso Ancho, 5 Feb 1946, *H. Berti & M. Escalante 431* (LP); Colonia José J. Mármol, 28-31 Dec 1946, *F. Buratovich 152* (LIL); La Clotilde (Lote 7), 5 Jan 1947, *F. Buratovich 303* (LIL); Las Cuchillas (lote 7), 5/7 Jan 1947, *F. Buratovich 314* & *396* (LIL); P.R. Saenz Peña, Napalpí, 8 Jan 1947, *F. Buratovich 413 & 477* (LIL); Campo Largo, Napalpí, 11 Jan 1947, *F. Buratovich 580 & 606* (LIL); entre Tres Isletas y Fortín, 17 Jan 1949, *J.A. Castiglioni & A.E. Ragonese 7042* (BAB); entre Colonia Castelli y Fortín Lavalle, 18 Jan 1949, *J.A. Castiglioni & A.E. Ragonese 7109* (BAB); Independencia, cruce de RN 80 con RN 16, 102 m, 25 Apr 2016, *A.A. Cocucci et al. 5969* (CORD); San Lorenzo, Samuhú, 2 Mar 1927, *J.D. Escobar 19* (CORD); La Invernada, a 20 km al W de Sauzalito hacia Tres Pozos, por antigua ruta, 18 Nov 1990, *R. Fortunato et al. 1445* (BAB); 14 km al NE de Castelli, por Ruta Prov. 5 en direcc. a Pt. Lavalle, 19 Nov 1994, *R. Fortunato et al. 4687* (BAB); 15 km de Sauzalito en dirección a Santa Rita, 2 Mar 2000, *R. Fortunato et al. 6520* (BAB, CTES); Campo Silvo-Pastoril del INTA Colonia Benítez, 7 Mar 2000, *R. Fortunato et al. 6667* (BAB); Las Unidas, 13 Jan 1942, *J.B. Gaillard s.n.* (BAB 63778); Colonia Benítez, 1 Apr 1917, *L. Hauman s.n.* (BA 2213); entre Colonia Benítez y Resistencia, 12 Mar 1945, *A.T. Hunziker 7341 & 7344* (CORD); Bermejo, Estancia San Carlos, 7 Apr 1945, *A.T. Hunziker 7357* (CORD); Nueva Pompeya, 4 Apr 1918, *P. Jörgensen 2987* (BA, LIL, SI); Libertad, ruta 16, Km 83, 2 Mar 1980, *P.R. Legname 7218* (CTES, LIL); ruta 16, Km 37, 20 m, 20 Mar 1981, *P.R. Legname 8186* (CTES, LIL); El Colchón, 30 Nov 2004, *G.J. Martínez 136* (CORD); Río Bermejito, 19 Jan 2005, *G.J. Martínez 190* (CORD); Colonia Benítez, Apr 1929, *T. Meyer 92* (LIL); Charata, 24 May 1934, *T. Meyer 858* (LIL); Margarita Belén, Feb 1938, *T. Meyer 2457* (GH, LIL) & *2458* (LIL); Pampa del Infierno, 30 Apr 1945, *T. Meyer 8561* (LIL); Picada Comisión del Bermejo, 30 km al NO de Fuerte Esperanza, 24°58'S, 62°2'W, 18 Jan 1980, *B.G. Piccinini & J. Hilfer 4123* (BAB, CTES); Selvas del Río de Oro,

26°49'S, 58°58'W, 28 Jan 1980, *B.G. Piccinini & J. Hilfer 4293* (BAB); Enrique Urien, Campo Bonazzola, Nov 1940, *A.P. Rodrigo 2637* (LP); Comandante Fernández, 20 km NE de Saenz Peña, ruta 95, 19 Feb 1980, *A. Schinini 19988* (BAB, CTES); General Güemes, 11 km SE de J.J. Castelli, Colonia 44, 26º00'47''S, 60º34'11''W, 19 Feb 2002, *A. Schinini & M. Urbani 35782* (CTES); Quitilipi, Feb 1930, *A.G. Schulz 1* (LP 011395); Colonia Benítez, Feb-Mar 1930, *A.G. Schulz 2* (LP); Colonia Benítez, Sep 1928, *A.G. Schulz 90* (BAB); Enrique Urien, 15 Jan 1946, *C.L. Schulz 845* (B, LIL); Colonia Benítez, Nov 1931, *A.G. Schulz 2030* (CORD, CTES) & Dec 1932, *A.G. Schulz 2032* (BAB, CTES); Charadal, 30 Dec 1942, *A.G. Schulz 3814* (CTES); Colonia Benítez, 15 Apr 1944, *A.G. Schulz 4272* (BAB, CORD, CTES); Colonia Benítez, Dec 1947, *A.G. Schulz 6719* (CTES, F); Enrique Urien, 11 Feb 1949, *A.G. Schulz 7361* (CTES); Enrique Urien, 18 Feb 1949, *A.G. Schulz 7363* (LIL); Enrique Urien, 25 Feb 1951, *A.G. Schulz 8178* (CTES, LIL, MY); Pampa Aguará, 18 Mar 1952, *A.G. Schulz 8284* (CTES, LIL); Pinedo, 15 Mar 1955, *A.G. Schulz 8930* (CTES); Colonia Benítez, 5 Mar 1955, *A.G. Schulz 9032* (BAB, CTES); El Tragadero, 12 Feb 1956, *A.G. Schulz 9064* (CTES, G); Nueve de Julio, Las Breñas, 26 Oct 1959, *A.G. Schulz 10649* (CTES); Ruinas Km 75, Ruta 95, 2 Aug 1965, *A.G. Schulz 15632* (CTES); Ruinas Km 75, Ruta 95, 27 Dec 1966, *A.G. Schulz 15801* (BAB, CTES); Ruinas Km 75, Ruta 95, 11 May 1970, *A.G. Schulz 17367* (BAB, CTES); San Lorenzo, 12 Apr 1972, *A.G. Schulz 18190* (CTES); Capdevilla, 8 Dec 1946, *C.L. Schulz 932* (LIL); Campo del Cielo, Itín, 26 Dec 1946, *C.L. Schulz 1082* (LIL); Fray Justo Santa María de Oro, Santa Sylvina, 27 Apr 1947, *C.L. Schulz 1141* (E) & 29 Jan 1947, *C.L. Schulz 1180* (LIL); Colonia Resistencia, Jan 1886, *C. Spegazzini s.n.* (LP 18570); Colonia Benítez (Nicolas Rojas Acosta), 18 Dec 1908, *T. Stuckert 19510* (CORD) & 2 Sep 1909, *T. Stuckert 20267* (CORD); Chaco austral, 1903, *I. Vila s.n.* (BAB 11055); **Córdoba**: ciudad de Córdoba, cerca de los Cuarteles, rumbo a la Calera, 12 Mar 1959, *L. Ariza Espinar 248* (CORD); Dean Funes, 30 Apr 1963, *L. Ariza Espinar 1626* (CORD); Obispo Trejo, 15 Dec 1946, *B. Balegno 1058* (LIL); Las Peñas, 21 Dec 1946, *B. Balegno 1149* (B, LIL); Cañada Larga, entre la mina de cuarzo y Agua de Ramón, 30º49'17''S, 65º19'44''W, 460 m, 26 Feb 2014, *G.E. Barboza et al. 4078* (CORD); Las Lajas, 3'º42'03''S, 64º36'47''W, 718 m, 1 Apr 2014, *G.E. Barboza et al. 4200* (CORD); desvío por camino a Copacabana (camino a Las Lajas), 30º42'03''S, 64º36'47''W, 718 m, 28 Mar 2017, *G.E. Barboza et al. 4766* (CORD); Lucio V. Mansilla, alrededores de la antena, 28º47'60''S, 64º42'29''W, 207 m, 1 Apr 2017, *G.E. Barboza et al. 4809* (CORD); El Diquecito, 14 Mar 1984, *G. Bernardello 471* (CORD); San Francisco del Chañar, 6 km oeste de la plaza del pueblo, hacia L.V. Mansilla, 13 Jan 1986, *G. Bernardello et al. 525* (CORD, CTES); ruta 60, km 834, pasando Dean Funes, 8 Jan 1990, *G. Bernardello & L. Galetto 726* (CORD); Universidad Nacional de Río Cuarto, márgenes del río, 13 Mar 1980, *C.A. Bianco 651* (RIOC); Cerro Corona-Sierra de Los Condores, 8 Mar 1983, *C.A. Bianco 1652* (RIOC); El Cuero, 30 Apr 1991, *C.A. Bianco 3216* (RIOC); Las Peñas – Potosiorco, 31 Mar 1992, *C.A. Bianco 3465* (RIOC); Icho Cruz, borde de Río San Antonio, 22 Jan 1973, *S. Botta & F. Ruiz de Angulo 115* (SI, CORD); Nono, La Quebrada, 9 Apr 1936, *A. Burkart 7752* (K, SI); Río Tercero, 11 Mar 1943, *A. Burkart 13361* (SI); Quilpo, 30º52'25''S, 64º40'13''W, 708 m, 12 Mar 2010, *J.J. Cantero 6385 & 6423* (CORD); Embalse Río Tercero, 32°18'15"S, 64°23'13"W, 20 Feb 2014, *J.J. Cantero 6976* (CORD); Alpa Corral, 32°41'58"S, 64°43'33"W, 870 m, 19 May 2015, *J.J. Cantero 7102* (CORD); al este de Salsacate, 18 Dec 2018, *J.J. Cantero 7311* (CORD); La Travesía, 8 Feb 1956, *J.A. Caro 1694* (BAF); Córdoba, *A. Castagnino s.n.* (BAA 11140); La Cocha, 15 Feb 1926, *A. Castellanos s.n.* (BA 26/372); San Javier, Jun 1927, *A.*

Castellanos s.n. (BA 11703); Valle de Los Reartes, 24 Apr 1917, *A. Castellanos 217* (SI); a mitad camino sobre la ruta entre S. Francisco del Chañar y Lucio V. Mansilla, torres de alta tensión, 17 Mar 2011, *F. Chiarini 758* (CORD); Salinas Gandes, entre Quilino y L. V. Mansilla, 9 Dec 1990, *A.A. Cocucci 477* (CORD); Quilino, 22 Feb 1998, *A.A. Cocucci et al. 973* (CORD); Chancaní, vado Río Chancaní, -31,399166S, -65472108 W, 373 m, *A.A. Cocucci 4966* (CORD); ruta 20, falda oeste de las sierras, después de los túneles, 13 Dec 1958, *A.E. Cocucci 77* (CORD); Yacanto, 27 Jan 1978, *M.T. Cosa 50* & 1 May 2002, *M.T. Cosa 342* (CORD); Los Ruices, alrededores del pueblo, a casi 3 km de Ischilín, 30º33'29''S, 64º22'33''W, 930 m, 15 Jan 2012, *M.T. Cosa 399* (CORD); Serrezuela, 8 Nov 1945, *A.R. Cuezzo 928* (LIL); Nono, May 1991, *L. Cunill s.n.* (BAA 21919); Salsipuedes, Mar 1938, *G. Dawson 117* (LP); Marull, camino a Playa Grande (orilla sudoeste de Mar Chiquita), 2 Jan 1977, *E. Di Fulvio 455* (CORD); Ongamira, 27 Mar 1940, *J.A. Domínguez & J.R. Domínguez s.n.* (BAF 8690); Tulumba, Jan 1900, *J.A. Domínguez 179* (BAF); Potrero de la escuela de Agricultura, Dec 1901, *A. Fernández s.n.* (BAB 1671, CTES 133281); Achiras, *J. Gillies s.n.* (K); entre Achiras y San Luis, *J.. Gillies 25* (E, GH); Campo los Morteritos, entre Santa Rosa y Yacanto, 750 m, 2/10 Nov 1945, *J. Gutiérrez 75* (CORD); between Cosquín and Bialet Massé, 11km N of turning to Tanti, 650 m, 24 Jan 1966, *J.G. Hawkes et al. 3308* (MO); in der kleinen Sierra sudöste von San Roque, 29 Jan 1876, *G. Hieronymus 356* (CORD, GOET); Sierra Chica, en una quebrada al sudeste de San Roque, 29 Jan 1876, *G. Hieronymus 427* (CORD, P); Capilla del Monte, 13 Apr 1915, *C.C. Hosseus 103* (CORD); al costado del camino, entre Charbonier y Copacabana, 19 Jan 1942, *C.C. Hosseus 340* (CORD); en el Cerro Uritorco, 10 Mar 1917, *C.C. Hosseus 424* (CORD); Lago San Roque, orilla opuesta a la del club de Regatas, 4 Feb 1945, *A.T. Hunziker 5941* (CORD); Lago San Roque, 13 Jan 1945, *A.T. Hunziker 6016* (CORD); Sierra Chica E, en el cerrito de Malagueño, 8 Sep 1946, *A.T. Hunziker 6734* (CORD); entre Pozo de la Olla y el Río Dulce, 24 May 1949, *A.T. Hunziker 7624* (CORD); Sierra Chica, falda O, Cerro Uritorco, ca. 1400 m, 19 Nov 1950, *A.T. Hunziker 8530* (CORD); ciudad de Córdoba, barrancas al NO del observatorio, 21 Jan 1951, *A.T. Hunziker 8764* (CORD); Cumbre de Gaspar, falda O, Cuesta de Las Chacras, sobre ruta 20 entre Arroyo Murrúa y Arroyo Albarracín, ca. 1600 m, 3 Feb 1951, *A.T. Hunziker 8842* (CORD); La Cumbre, Cerro de La Cruz, 18 Feb 1951, *A.T. Hunziker 8915* (CORD); Sierras de Serrezuela (Falda E), entre Tuclame y Serrezuela, 14 Apr 1951, *A.T. Hunziker 9111* (CORD); entre La Higuera y Rumi Huasi, 15 Apr 1951, *A.T. Hunziker 9153* (CORD); Sierras Guasapampa, falda O, cerca de Ojo de Agua, 15 Apr 1951, *A.T. Hunziker 9170* (CORD); Sierra Ischilín, falda O, cerca de Ischilín, 29 Apr 1951, *A.T. Hunziker 9222* (CORD); cerca de Altautina, en el camino a Villa Dolores, 9 Jan 1952, *A.T. Hunziker 9519* (CORD); Sierras de Pocho, falda O al NE de Chancaní, ca. 700 m, 16 Feb 1952, *A.T. Hunziker 9805* (CORD); inmediaciones de Río Primero, 4 Feb 1955, *A.T. Hunziker 10642* (CORD); cerca de El Coro, en el camino que viene desde San Miguel, 11 Feb 1955, *A.T. Hunziker 10700* (CORD); Sierra al O de Villa de María, Casa de Piedra, 6 Mar 1955, *A.T. Hunziker 10816* (CORD); La Travesía, entre la Población y Luyaba, 8 Feb 1956, *A.T. Hunziker 11695* (CORD); a orillas de Mar Chiquita, entre Miramar y la desembocadura de Rio Plujunta, 21 Jan 1957, *A.T. Hunziker 13271 & 13300* (CORD); Sierras de Pocho, falda O sobre ruta 20, cerca del último túnel, 24 Mar 1958, *A.T. Hunziker & J.A. Caro 13495* (CORD); Sierras de Pocho, ruta 20, en la base de la falda O, 16 Jan 1960, *A.T. Hunziker & A.E. Cocucci 14698* (CORD); Sierras de Maza, entre San Antonio y Maza, 5 Feb 1960 m, *A.T. Hunziker 14842* (CORD); Chaján, 9 Mar 1963, *A.T. Hunziker 16516* (CORD); Cerro

Uritorco, falda occidental, 1000-1200 m, 15 Jan 1965, *A.T. Hunziker 17972* (CORD); a ca. 6 km al O de la Iglesia de San Francisco del Chañar, 700 m, 30 Jan 1987, *A.T. Hunziker & R. Subils 24963 & 24964* (CORD); entre ruta 38 y Dique nivelador de Pichanas, hacia el sur de la ruta, 12 Mar 1990, *A.T. Hunziker et al. 25388* (CORD); Campo de San Roque, 22 Feb 1863, *J. Isern 8240* (SI); Almafuerte, 1 Mar 1930, *D.O. King 284* (BAB, LP); Serrezuela, RN 38, 16 Feb [*sine year*], *A. Krapovickas 1215* (CORD); Casabamba, 25 Feb 1945, *A. Krapovickas 1905* (CORD); RN 9, Estación Experimental INTA-Manfredi, 14 Apr 1950, *A. Krapovickas 6671* (BAB, CORD, LIL, SI); Córdoba, Nov 1891, *O. Kuntze s.n.* (G, F, LP 010336 & 010337; US 701470 & 701471); Soto y Cruz del Eje, 28 Jan 1885, *F. Kurtz 192* (CORD); Córdoba, 30 Dec 1884, *F. Kurtz 958* (CORD); del nacimiento del Río Las Tapias, cerca de la Estancia la Vuelta, 18 Feb 1887, *F. Kurtz 4615* (CORD); entre Soto y Cruz del Eje, 23 Feb 1889, *F. Kurtz 6503* (CORD); Quinta Soriano, al N del Bajo Grande, 15/31 Jan 1912, *F. Kurtz 16056* (CORD); Saldán, May 1939, *R. Lahitte s.n.* (BAB 60390); Capilla del Monte, 1 Jun 1939, *R. Lahitte s.n.* (BAB 60391); Quebrada de las Rosas, camino a La Calera, 21 Mar 1953, *A.E. Lanfranchi 1125* (LP); Quebrada de las Rosas, 7 Mar 1954, *A.E. Lanfranchi 1144* (LP); Córdoba, *P.G. Lorentz s.n.* (SI 24395); San Francisco, Feb 1971, *P.G. Lorentz 105* (G, CORD); Córdoba, Jan 1925, *W. Lossen 323* (GH, LE, MO); Chuchira, 22 Dec 2012, *M.C. Luján 327* (CORD); Pozo de los Árboles, 2 Apr 1960, *R. Luti & E. Bücher 4004* (LIL); Quilino, 16 Dec 1947, *T. Meyer 13128* (LIL); Las Maravillas, 900 m, 26 Jan 1948, *T. Meyer 13325* (CORD, LIL); entre Chancaní y Villa Dolores, 6 Dec 1958, *J. Morello et al. s.n.* (LIL); Falda del Sauce, ruta 36,5 km antes de V.Gral Belgrano, 800 m, 13 Feb 1985, *E. Moscone 98* (CORD), 5 Mar 1985, *E. Moscone 104* (CORD) & 15 Mar 1992, *E. Moscone 194* (CORD); Falda de los Reartes, propiedad de Gigena, 15 Mar 1992, *E. Moscone 195* (CORD); Pilar, 18 Mar 1992, *E. Moscone 207* (CORD); Escobar, 2 Jan 1941, *E.G. Nicora s.n.* (SI 070555); Charbonier, 22 Jan 1941, *E.G. Nicora s.n.* (SI 070556); San Marcos a Totoralejos, 7 Feb 1941, *E.G. Nicora s.n.* (SI 17684); San Esteban, 8 Jan 1938, *E.G. Nicora 1648 & 1650* (SI); Nono, camino La Quebrada a las Cumbres, 9 Feb 1973, *E.G. Nicora 7645* (BAA); Arroyo Aguas Dulces, en el cruce camino a los Túneles, 28 Mar 1975, *E.G. Nicora 8044* (BAA); Capilla del Monte, 30 Mar 1944, *C.A. O'Donell 351* (F, LE, LIL) & *901* (LIL); entre Mina Clavero y Niña Pabla, 23 Mar 1944, *C.A. O'Donell & J.M. Rodríguez V. 920* (GH); Copina, 12 Mar 1944, *C.A. O'Donell & J.M. Rodríguez V. 978* (LIL); entre Capilla del Monte y La Cumbre, 20 Jan 1947, *C.A. O'Donell 4436* (LIL); Cruz del Eje, 19 Jan 1947, *C.A. O'Donell 4410* (LIL); San Marco, B. Ville, Dec 1920, *L.R. Parodi 3102* (BAA); Valle Hermoso, 20 Mar 1944, *M. Ruiz de Huidobro 125* (LIL); Villa Candelaria, *M. Sayago B-113* (CORD); RN 60, 3 km antes de Quilino, viniendo desde Lucio V. Mansilla, 30º10'53''S, 64º30'27''W, 376 m, 1 May 2013, *M. Scaldaferro 27 & 28* (CORD); camino de tierra entre Dean Funes e Ischilín, 3 km antes de llegar a Ischilín (cerca de Los Ruices), 30º33'28''S, 64º22'24''W, 868 m, 1 May 2013, *M. Scaldaferro 29* (CORD); sobre ruta que une Las Tapias con Merlo, entre La Población y Luyaba, 32º04'18''S, 65º03'28''W, 711 m, 7 Jul 2013, *M. Scaldaferro 32* (CORD); Amboy, 32º16'06''S, 64º54'74''W, 635 m, 16 Mar 2014, *M. Scaldaferro 44* (CORD); San Marcos, 29 Feb 2016, *M. Scaldaferro 65* (CORD); Los Cocos, Jan 1943, *H. Schwabe 650* (LP); Córdoba, Jan 1887, *C. Spegazzini s.n.* (LP 18568); Puesto del Paraíso, 3 May 1896, *T. Stuckert s.n.* (CORD 00087958); Sacanta, 6 Dec 1898, *T. Stuckert s.n.* (CORD 00006682); Salto, 22 Dec 1896, *T. Stuckert 917* (CORD); Altos Sud, 25 Dec 1896, *T. Stuckert 1008* (CORD); San Vicente, 16 Dec 1896, *T. Stuckert 1120* (G, CORD); San Martín, 20 Dec 1896, *T. Stuckert 1166* (CORD); Alta Córdoba, 7 Jan 1897, *T. Stuckert*

1320 (CORD); Salto, 26 Mar 1897, *T. Stuckert 2405* (G, CORD); San Vicente, 8 Jan 1898, *T. Stuckert 4238* & *4247* (CORD); Altos Sud, 17 Dec 1898, *T. Stuckert 5557* (CORD); Altos Sud, alrededores de la ciudad, 18 Dec 1898, *T. Stuckert 5614* (CORD); Malagueño, 3 Jan 1899, *T. Stuckert 6007* (CORD, G); Sacanta, Dec 1900, *T. Stuckert 9913* (CORD); Estancia San Teodoro, Apr 1902, *T. Stuckert 11559* (CORD); Quinta, en los alrededores de la ciudad, 8 Jan 1903, *T. Stuckert 12551* (CORD); Rio I, Estancia, Nov 1903, *T. Stuckert 13644* (CORD); Sacanta, May 1904, *T. Stuckert 14330* (CORD); Estancia San Teodoro, Jun 1906, *T. Stuckert 15477* (CORD) & 26 Jan 1910, *T. Stuckert 21449* (CORD); La Playosa, Mar 1911, *T. Stuckert 22168* (CORD); un poco al norte de Dean Funes, 7 Dec 1956, *R. Subils & L. Articó 6* (CORD); Los Tres Picos, entre Costa Sacate y Rincón, 2 May 1971, *R. Subils et al. 1229* (CORD); Quebrada de las Rosas, 29 Jul 1969, *R. Subils 1437* (CORD); Cañada de Machado Sud, 30 Nov 1975, *R. Subils 2107* (CORD); Villa Rosario del Saladillo, 13 Dec 1976, *R. Subils 2335* (CORD); Yacanto, Jan 1922, *J. Vattuone 99* (SI); Agua de Oro, 650 m, Jan 1952, *B. Veronesi s.n.* (LIL); Charbonier, 8 Jan 1947, *M. Villafañe 348* (LIL, MO); Capilla del Monte, 10 Jan 1947, *M. Villafañe 400* (LIL); Huerta Grande, 16 Dec 1946, *E. Wall s.n.* (S16-28221); E.E.A. INTA Manfredi, 23 Mar 1990, *D.E. Williams & A. Krapovickas 1122* (CTES, F, GH, LIL, LPB, MO, NY); **Corrientes**: Ruta 6, 2ª Sección, San Cosme, 27 Nov 1981, *R. Carnevali 5290* (CTES); Puerto Valle, Jan 1990, *S. Heinonen et al. 143 & 152* (CTES); Pasaje Cebollas, 2 May 1945, *T.S. Ibarrola 2988* (CORD); 4 km E de Paso de la Patria, 20 Feb 1969, *A. Krapovickas et al. 14900* (CTES, LIL, MO, MY, UC); Estancia La Blanca, 26 km SE de Libertador, 30º20'S, 59º20'W, 12 Mar 1975, *A. Krapovickas et al. 27442* (CTES, G, LIL, MO, UC); Colonia Libertador, arroyo Barrancas, 15 Mar 1975, *A. Krapovickas et al. 27769* (CTES); ruta 12, antes de Itá-Ibaté, 15 Jan 1951, *E.G. Nicora s.n.* (BAA 288); Estancia La Yela, 25 Nov 1957, *T.M. Pedersen 4670* (G, MO, P, UC, S, US); Estancia Santa María, 14 Apr 1960, *T.M. Pedersen 5517* (E, GH, LP, P, S, US); Estancia La Yela, 21 Nov 1972, *T.M. Pedersen 10239* (CORD, NY, S, SI); Río Paraná y Arroyo San Juan, 25 May 1975, *A. Schinini & C.L. Quarín 11524* (MO); Tres Cerros, Co. Nazareno (Co. de Coutinho), 14 Feb 1979, *A. Schinini et al. 17088* (CTES, ICN); **Entre Ríos**: La Paz, alrededores, en sotobosque de Ñandubay, *A. Burkart 21384* (SI); Punta Gorda, *A. Burkart 22299* (MO, SI); Bovril, 16 Mar 1962, *A. Burkart 23492* (SI); Diamante, 15 May 1962, *A. Burkart 25459* (MO, SI); Punta Gorda, *Burkart 26746* (SI); Paraná, 1 Feb 1898, *C.M. Hicken s.n.* (SI-024394); Paraná, *P.G. Lorentz s.n.* (BA 2214); Colonia Hernandarias, 13 Feb 1878, *P.G. Lorentz 1318* (BAF); Paraná, *Naboulet 140* (BA); cercanías de Paraná, 1891-1892, *L. Onetto s.n., Herb. Kurtz 7912* (CORD); **Formosa**: Pozo Nayarán, reducción de Indígenás Pilagás, 18 Jan 1982, *P. Arenas 1956* (CTES, SI); Ing. G. Juárez, Toldería toba, 23º54'S, 64º51'W, 20 Feb 1983, *P. Arenas 2197* (CTES, SI); El Churcal, 16 Nov 1985, *P. Arenas 3043* (CTES); Pozo de Maza, 25 Jan 1989, *P. Arenas 3388* (CTES); Ingeniero Juárez, 14 Jan 1957, *A. Burkart 20300* (SI); Estancia Pai Curuzu, 25°01'02''S, 58°28'19,6''W, 96 m, 21 Mar 2012, *J.P. Coulleri & S.M. Ferrucci 338* (CTES); Reserva "El Bagual de Alparamis", 25 km al W de San Francisco de Laishi, 26°10'S, 58°56'W, 10 Dec 1996, *A. Di Giácomo 230* (CTES); Reserva "El Bagual", 5 Apr 2003, *A. Di Giácomo 607* (CTES); Reserva "El Bagual", 5 Apr 2003, *A. Di Giácomo 607* (CTES); 4 km del cruce de ruta Gral. Belgrano-San Carlos, en dirección a San Ramón, 17 Nov 1991, *R. Fortunato et al. 2399* (BAB); alrededores de San Martín 2, 23 Mar 1992, *R. Fortunato et al. 3030* (BAB); Ea. La Esperanza en La Miseria, alrededores de Sargento Leyes, 24 Mar 1992, *R. Fortunato et al. 3080* (BAB, MO); 3 km del cruce con ruta Pozo de Navagan-Lugones, hacia Estero Chumuco (Paraguay), 26 Mar

1992, *R. Fortunato et al. 3215* (BAB, SI, MO); alrededores de la ciudad Formosa, 5 Jan 1980, *E.R. Guaglianone et al. 309* (CORD); Parque Nacional Pilcomayo, Puesto Algarrobo, 15 Dec 1988, *E.R. Guaglianone 2255* (SI); Puerto, Jan 1928, *S. Horovitz s.n.* (BAA 8590); El Colorado, Jun 1972, *P. Insfrán 765* (CTES); Formosa, Feb 1918, *P. Jörgensen 2206* (LIL, MO, SI); Formosa, 15 Dec 1900, *E. Kermes s.n.* (BAB 377 bis); Comandante Fontana, May 1970, *R. Martínez Crovetto 31* (CTES); Palo Santo, 6 Dec 1972, *V. Maruñak et al. 626* (CTES, LP); Estancia El Ombú, 26°06'06"S, 58°48'06"W, 18 Dec 2004, *H. Maturo 245* (BM, CTES, FCQ, MO, SI, UNR); Pirané, 12 Jan 1946, *I. Morel 452* (E, LIL); Rcho. Pilagá, lote 4, 26 Dec 1945, *I. Morel 722* (LIL); Casco Cué, Jan/Feb 1946, *I. Morel 776* (B, LIL) & *910* (G, MO, LIL); ruta 11, Clorinda a Formosa, Km 4, 3 Dec 1946, *I. Morel 1746* (LIL); Loma Porá, 17 Dec 1946, *I. Morel 1845* (LIL); Riacho Negro, 19 May 1947 (fr), *I. Morel 2867* (LIL, S); norte de Riacho Negro a 2 kms, 11 Jul 1947, *I. Morel 3375* (LIL); Salvación, 17 Nov 1947, *I. Morel 4171* (LIL); Pueblo Laguna Blanca, 16 Jan 1948, *I. Morel 4417* (LIL); Río Pilcomayo, Paso Santa Rita, 3 Dec 1948, *I. Morel 6746* (LIL); Puerto Ramos, 2 Feb 1949, *I. Morel 7127* (LIL); Isla Leena, 9 May 1949, *I. Morel 7691* (CORD, LIL); SO a 12 km de Filipina, 18 Nov 1949, *I. Morel 8909* (LIL); 15 km al norte de Ing. Juárez, 10 Jan 1978, *R.A. Palacios 733* (CORD); ruta 81, 25 km. NW da bifurcaçao para Clorinda na Ruta 11, 25°59'27"S, 58°25'35"W, 65 m, 2 Feb 2007, *J. Paula-Souza 8175* (CTES, SI); entre Pozo de Tigre y Laguna San Luis, 25 Feb 1951, *A.E. Ragonese & J.A. Castiglioni 7991* (BAB, CORD); Monteagudo (Km 10, Ruta 11), 14 Jan 1947, *A. Reales s.n.* (B, LIL); Laguna de los Ríos, 14 Dec 1946, *A.E. Reales 65* (CAS, LIL); Las Lomitas, 130 m, 11 Dec 1984, *A. Schinini & S.N. Pire 24216* (BAB, CTES); ruta 39, 4 km S de Ing. Juárez, cercano al canal de riego del Río Bermejo, 6 Mar 2001, *A. Schinini et al. 35295* (CTES); Bartolomé de las Casas, 14 Dic 1970, *A.G. Schulz 17721* (CTES); Nuevo Porteño, 25 Jan 1972, *A.G. Schulz 17958* (CTES); El Cogoik, 19 Feb 2004, *S. Tressens et al. 6925* (CTES, SI); **Jujuy**: Pampa Blanca, 13 Apr 1984, *O. Ahumada 5013* (CTES, LIL); entre Santa Clara y Abra de los Morteros, 18 Mar 1973, *A.L. Cabrera et al. 23353* (LP, SI); La Estrella, 23º49’41’’S, 64º13’27’’W, 428 m, 6 May 2016, *A.A. Cocucci et al. 6000* (CORD); Palma Sola, 10 Feb 1964, *H.A. Fabris et al. 5278* (LP); Puesto Viejo, Finca Cabeza de Toba, la Cumbre, 24°29’29.5’’S, 64°56’20.3’’W, 1012 m, 13 Mar 2006, *G. Guzmán et al. s.n.* (MCNS 2280); Arroyo del Medio, 15 Feb 1988, *Magnoni 10* (CORD, MCNS); Quebrada Casa de Piedra, por la ruta 34, 2-4 km al NE de la rotonda de ingreso al pueblo, 29 Mar 2006, *L. Novara et al. 12504* (MCNS); Perico, 3 Apr 1911, *P. Jörgensen s.n.* (BAB); Río Perico, 940 m, 28 Mar 1977, *A. Krapovickas & A. Schinini 30655* (CTES); Perico, 17 Jan 1906, *C. Spegazzini s.n.* (LP 18573); **La Pampa**: Rancul, Colonia Lobocó, 8 Feb 1944, *J. Fortuna s.n.* (LIL); entre Rancul y Chamaico, 9 Mar 1976, *H.O. Troiani & P.E. Steibel 4077* (CORD); Ingeniero Foster, 26 Feb 1976, *P.E. Steibel & H.O. Troiani 3835* (CORD); **La Rioja**: Santa Lucía, 15 km SW de Chamical, saliendo del pueblo rumbo a Polco, 25 Mar 1990, *F. Biurrum & E. Pagliari 3271 pp* (CTES); Dique, 1 Mar 1941, *A. Burkart 12553* (MO, SI); Chilecito, 19 Feb 1941, *G. Covas 1184* (LP); Chepes, 11 Nov 1945, *A.R. Cuezzo 976* (LIL); Río Seco, *A. Giacomelli 130* (BAB); Saladillo, Jan 1905, *A. Giacomelli 4806* (LIL); Iliar, cerca de Olta, 4 Feb 1922, *M. Gómez s.n.* (BA 29/191); San Francisco, 14 Jun 1928, *M. Gómez s.n.* (BA 29/191); a ± 15 km de La Rioja, camino a La Ramadita, 17 Feb 1944, *A.T. Hunziker 4795 & 4797* (CORD); La Ramadita, a ± 25 km de La Rioja, 17 Feb 1944, *A.T. Hunziker 4807, 4812* & *4820* (CORD); Río Hondo, camino al Cantadero, 4 Mar 1944, *A.T. Hunziker 5011* (CORD); cerca de Las Cañas, camino al Cantadero, 4 Mar 1944, *A.T. Hunziker 5071* (CORD); cerca

del Cantadero, 5-7 Mar 1944, *A.T. Hunziker 5088* & *5299* (CORD); Sierra de Ulapes, falda E, frente a Ulapes, 25 Mar 1958, *A.T. Hunziker 13520* (BAF, CORD); ruta 79, a ca. 16 km de Ulapes, 26 Mar 1958, *A.T. Hunziker & J.A. Caro 13610* (BAF, CORD); Sierra de los Llanos, entre Olta y el Dique, 19 Feb 1959, *A.T. Hunziker et al. 13912* (CORD); Sierra de los Llanos, Río Totoral, a ca. 5 km de Chelco, 700 m, 5 Mar 1959, *A.T. Hunziker et al. 14160* (CORD); cerca de Patquía, unos 7 km al OSO, rumbo a Paganzo, 3 Feb 1961, *A.T. Hunziker et al. 15834* (CORD); Ulapes, 428 m, 24 Mar 1949, *A. Krapovickas 6021* (BAB); Bajo Hondo, 428 m, 24 Mar 1949, *A. Krapovickas 6093* (BAB, CORD); Patquía, Guayapa, 13 Mar 1933, *R. Lahitte s.n.* (BAB 47702); La Calera, a 27 km de la ciudad, 750 m, 5 Dec 1948, *R. Malme 156* (LIL); Ruta 79, km 219, 30°32'7.6"S, 66°16'48.8"W, 512 m, 24 Feb 2008, *J. Pensiero et al. 7434* (CTES, SF, SI); Sierra de Velazco, camino a Las Cañas, 4 Mar 1944, *A. Soriano 900* (BAB, SI); Ulapes, Jun 1903, *T. Stuckert 13210* (CORD); La Rioja, Jan 1940, *R. de la Vega s.n.* (CORD 00006677); **Misiones**: San Pedro, Laharrague, 1 Aug 1951, *J.E. Montes 15418* (LIL, S); San Juan, 6 Jun 1947, *E. Schwindt 996* (E, LIL); El Alcazar, 208 m, 30 Mar 1949, *E. Schwindt 1362* (LIL, CTES); **Salta**: Rivadavia, Misión La Paz, 22º26'S, 62º22'W, 15 Feb 1982, *P. Arenas 2121* (CTES); Puerta Blanca, 38 km al NE de Salta (por RP 41), 380 m, 2 Feb 1990, *H. Ayarde & M. Sidán 299* (LIL); Rivadavia Banda Norte, El Chañar, 30 Mar 1995, *S. Bianchi & C. Camardelli 951* (MCNS); Cobos, 4 Mar 1958, *A.L. Cabrera & J.M. Marchionni 12781* (LP); Guachipas, Cuesta de el Cebilar y El Lajar, 20-30 km al SE de Guachipas, 1300-1800 m, 3 Apr 1984, *A. del Castillo & R. Neumann 383 p.p.* (MCNS); Las Curtiembres, sobre terrazas del río, 1250 m, 13 Dec 1985, *A. del Castillo & F. de Varela 847* (MCNS); Candelaria, 24 Jan 1964, *A.R. Cuezzo 3379* (LIL); 1 km al S de Guachipas en dirección a El Cebilar, 16 Mar 2001, *R. Fortunato et al. 7184* (BAB); Orán, Yuchán, 22 May 1945, *Herrera 543* (LIL); La Caldera, Estancia La Despensa, a ± 25 Km de La Caldera, 16 Apr 1942, *A.T. Hunziker 1578* (CORD); Joaquín V. González, 9 Feb 1945, *A. Krapovickas 1709* (CORD, CTES); Esteco, 28 Mar 1975, *A. Krapovickas et al. 27991* (CTES); 20 km al E de Rosario de la Frontera, 29 Mar 1975, *A. Krapovickas et al. 28010* (CTES); 8 km de J.V. González, camino a Metán, 6 May 1975, *A. Krapovickas et al. 28162* (CTES) & *28164* (CTES, G, LIL, MO, UC); 20 km al E de Salta, rumbo a Güemes, 22 Mar 1977, *A. Krapovickas & A. Schinini 30421* Copo Quille, 23 Mar 1977, *A. Krapovickas & A. Schinini 30457* (CTES); San Lorenzo, 23 Mar 1977, *A. Krapovickas & A. Schinini 30494* (CTES, G); (CTES, F, MEXU, MO); Coronel Moldes, Apr 1934, *R. Lahitte s.n.* (BAB 49556); camino de Cabeza de Buey a Río Juramento, 790 m, 22 Feb 1977, *P.R. Legname et al. 5322* (LIL); Rosario de la Frontera, 26 Feb 1888, *M. Lillo 610* (LIL); El Dorado, 20 Apr 1946, *F.E. Luna 957* (E, G, LIL); Coronel Mollinedo, 335 m, 15 Jan 1947, *M.R. Málvarez 583* (LIL); Los Blancos, Barrio Mataco, 24 Jan 1983, *A. Maranta 185* (MO); 15 Km O de Hickmann, Pozo El Milagro, *V. Maruñak et al. 564* (CTES); entre gas del Estado y Juramento, km 1374, 26 Mar 1969, *T. Meyer et al. 9170C* (LIL); entre Joaquín V. González y Olleres, 1 Mar 1959, *T. Meyer 20304* (LIL); RN 16,3 km SE de Toloche, 6 Mar 1986, *A.M. Molina & J. Hilfer 2993* (BAB); J. V. González, 10 km al este, Finca San Javier, 5 Mar 1958, *J. Morello & A.R Cuezzo 284* (LIL); J. V. González, 10 km al norte, 4 Mar 1958, *J. Morello & A.R. Cuezzo 402* (LIL); Osma, 1210 m, 10 May 1995, *E. Moscone & R. Neumann 212* & *213* (CORD); La Pedrera, camino a La Higuerilla, 10 km al SE de Salta, 1300 m, 19 Apr 1981, *L. Novara 1936* (G, MCNS); cerro al sur del San Bernardo y El Portezuelo, 26 Jan 1983, *L. Novara 3069* (CORD, MCNS); camino a Lote 5, 30 km al E de Gral. Mosconi, 12 Apr 1983, *L. Novara 3353* (CORD, MCNS, MO); Chachapoyas, Sierra de Vélez, al norte de Tres Cerritos, 14 Jan 1989, *L.*

Novara 8423 (CORD, G, MCNS, S); ruta 10, 4-6 km al NE de Gral. Güemes, 650-700 m, 12 May 1990, *L. Novara & S. Bruno 9898* (CORD, CTES, G, LIL, M, MCNS, S); ruta 5, ca. 5 km. E da Ruta 9, 25°12'13"S, 65°54'29"W, 21 Jan 2007, *J. Paula-Souza 7981* (CTES, ESA, SI); El Naranjo, 22 Jan 1945, *S.A. Pierotti (h) 33* (LIL); entre Gaona y El Quebrachal, 26 Jan 1950, *A.E. Ragonese & J.A. Castiglioni 8036* & *8039* (BAB); San José de Orquera, 3 Sep 1957, *C. Saravia Toledo 103* (LIL); Pozo Largo, 31 Jan 1984, *C. Saravia Toledo 828* (MCNS); Pozo Largo (J.V.Gonzalez), 350 m, 15 Mar 1986, *C. Saravia Toledo 1197* (CTES, SI); Los Colorados, 250 m, 4 Mar 1992, *C. Saravia Toledo 10706* (CORD, G); Misión aborígen La Merced Vieja, 150 m, 25 Feb 2004, *G.F. Scarpa 649* (SI); Represa a Campo Gasano-Tartagal, 500 m, 6 Feb 1925, *R. Schreiter 3791* (LIL); El Morenillo, 26 Mar 1935, *R. Schreiter 10101* (LIL); Q. Naranjito-Tartagal, *A.G. Schulz 5222* (LIL); entrada a Hickmann, 23º12’40”S, 63º33’23”W, 256 m, 13 Feb 2005, *J.G. Seijo et al. 3869* (CORD, CTES, NY, SI); Anta, zona chaqueña, 24°49'35"S, 64°9'24"W, 470 m, 14 Apr 2008, *A.C. Slanis et al. 116* (SI); Pampa Grande, Jan 1897, *C. Spegazzini s.n.* (LP 18572); La Viña, Jan 1897, *C. Spegazzini s.n.* (LP 18571); pocos km antes de Alemania, 12 Nov 1984, *R. Subils et al. 3564* (CORD); Sierras de Metán, Finca Cachari, 18-20 km al W de Lumbreras, 1334 m, 25°12’57.3’’S, 65°06’44’’W, 10 Apr 2006, *J. Tolaba et al. 4090* (MCNS); quebrada del Río Colorado, 26°05’21’’S, 66°01’19’’W, 1800-2300 m, 27 Apr 2014, *R. Troncoso & M. Fabbroni 224* (MCNS); Arenal, Ruta 55 junto a alambrados, 18 Jun 1963, *J.J. Valla 10* (BAA, CORD); ruta 68 from Cafayate to Alemania, km 77, 25°37'S, 65°38'W, 1240 m, 22 Mar 2007, *B.W. Van Ee et al. 669* (SI); San Severo a San Javier, Campo del Norte S.A., 29 Mar 1985, *F. de Varela & A. del Castillo 1197* (MCNS); La Palata, 26 Feb 1958, *M. Villa Carenzo 159* (LIL); Serranía de Las Termas, 27 Feb 1958, *M. Villa Carenzo 202* (CTES, LIL); **San Juan**: Valle Fértil, sierra de Valle Fértil, 15 Feb 1975, *J.A. Ambrosetti 1898* (MERL); San Agustín del Valle Fértil, 22 Apr 1982, *A. Cortéz 156* (SI); 7 km del desvío de Ruta Prov. 510 en dirección E a Los Bretes, 30°45'S, 67°26'W, 900 m, 11 Mar 1998, *R. Fortunato et al. 5959* (BAB, CTES, SI); San Agustín, 7-8 Jun 1933, *K.F. Hayward s.n.* (BAB); N de Usno, 8 Mar 1998, *R. Kiesling 8964* (SI); **San Luis**: Estancia La Unión, a 5 km al norte de Villa Mercedes, 515 m, 13 Dec 1968, *D.L. Anderson 1447* (CORD); Bajo de Velis, 15 Dec 1905, *W. Bodebender s.n.* (BAF 8696); Alto Pencoso, Feb 1914, *C. Bruch & E. Carette s.n.* (LP 010698); Santa Rosa, La Quebrada de la Higuerita, 29 Jan 1944, *A. Burkart 13972* (CORD, SI); Alto Pencoso, Feb 1914, *E. Carette 28* (SI); San Francisco, 12 May 1925, *A. Castellanos s.n.* (BA 25/967); RN 146, km 144, cerca de salinas y Laguna El Bebedero, 33º27’35’’S, 66º25’47’’W, 541 m, 27 Jan 2016, *A.A. Cocucci & A. Sérsic 5877* (CORD); llegando a Merlo, 32º48’31’’S, 65º18’06’’W, 800 m, 6 Feb 2016, *A.A. Cocucci & A. Sérsic 5939* (CORD); Entre Merlo y Cortaderas. Cerro de Oro, 32°21'52"S, 64°57'20"W, 1216 m, 13 Mar 2011, *N.B. Deginani 2093* (SI); Río Papagayos, 1020 m, 13 Dec 1989, *C. Ezcurra & M. Ponce 533* (SI); entre la Villa de Luján y San Francisco, 15 Mar 1882, *C. Galander s.n.* (CORD 00087959); Merlo-Piedra Blanca, 1000 m, 5 Feb 1947, *D. Grassi 2063* (LIL); El Balde, 12 Jan 1927, *H. Greslebin s.n.* (BA 27/47); San Luis, *J.R. Guiñazú s.n.* (BAB 59641); San Martín rumbo al cerro Blanco, 950 m, 15 Jan 1960, *A.T. Hunziker & A.E. Cocucci 14666* (CORD); Sierra de San Luis, Las Masas, ruta 5, entre Quebrada del Tigre y Bañado de Cautana, 500 m, 17-20 Feb 1960, *A.T. Hunziker & A.E. Cocucci 14913* (CORD); Sierra de Socoscora, al E ruta 146, entre San Francisco y Nogoli, 900 m,17-20 Feb 1960, *A.T. Hunziker & A.E. Cocucci 15050* (CORD); Sierra del Gigante, en las inmediaciones de la Calera, 700-750 m, 31 Jan 1963, *A.T. Hunziker & P. Maldonado 16263* (CORD); Alto Pencoso, alrededores de la

estación ferroviaria, 25 Jan 1979, *A.T. Hunziker et al. 23390* (CORD); a ± 3 Km al N de Concarán, rumbo a Santa Rosa, por el viejo camino de tierra, 17 Feb 1989, *A.T. Hunziker et al. 25334* (CORD); San Luis de la Punta (Aguada de Pigrodon), 8 Mar 1863, *J. Isern 8094* (SI); Merlo, Piedra Blanca, 27 May 1990, *H. Juliani s.n.* (CORD 00087963); Sierras de San Luis, Bajo de Velis, 28 Jan 1895, *F. Kurtz 8510* (CORD, LP); Bajo de los Velez, 740 m, 18 Feb 1942, *R. Maldonado B. 1250* (LP); Piedra Blanca, 12 Feb 1958, *T. Meyer 13848* (LIL); 0.5 km. al E del acceso a Papagayos, cerro en faldeo Oeste de la Sierra de Comechingones, 32°40'30"S, 64°58'40"W, 1100 m, 14 Jan 2004, *R. Pozner 207* (SI, CTES); ruta 148, a 5 km de la Escuela de Agronomía, 28 Feb 1995, *E. Rosa 1697* (CTES); Luján, laderas de cerros próximas al Dique, 640-670 m, 26 Mar 1983, *A. Ruiz Leal s.n.* (MERL); RP 1, desde Cortaderas a Papagayos, llegando a El Tala, 32º31’53’’S, 64º59’02’’W, 944 m, 7 Jul 2013, *M. Scaldaferro 33* (CORD); Tilisarao, Mar 2013, *M. Scaldaferro 36* (CORD); ruta prov. 40, 5 km W de Papagallo, 8 Feb 1999, *J.G. Seijo 1833* (CTES, SI); El Portezuelo, Jan 1933, *M.A. Vignati 337* (LP); Luján, entre Pueblo y el Dique homónimos, 630 m, 26 Mar 1983, *L. del Vitto s.n.* (MERL); **Santa Fe**: entre Berna y Malabrigo, 14 Nov 1984, *G. Bernardello et al. 498* (CORD, CTES); Estero La Zulema, entre El Tostado y Fortín Olmos, 10 Nov 1954, *A.T. Hunziker 10376* (CORD); Arroyo Golondrina, entre Fortín Olmos y Fortín Chilcas, 10 Nov 1954, *A.T. Hunziker 10381* (COL, CORD); camino de Reconquista a Nicanor Molina, Jan-Feb 1936, *M.M. Job 874* (S); Lanteri, Estancia Bonazzola, 1 Feb 1936, *M.M. Job 1210* (BAA); Estancia Las Norias, Logroño, FCE, ± 70 m, 29 Dec 1944, *A. Krapovickas 762* (CORD); camino entre Villa Guillermina y El Rabón, 18 Feb 1988, *J. Pensiero & J. Tivano 3261* (CORD); al NW del km 101, 61 m, 15 Mar 1997, *J. Pensiero 5245* (SF); Vera, desvío Cerrito, 13 Dec 1979, *E.F. Pire 520* (CTES); 9 de Julio, camino a María Alicia, *D. Prado 10* (SI); Videla, 12 Nov 1936, *A.E. Ragonese 2227* (US); Villa Rosa (Rafaela), 30 Nov 1946, *M. Terribile 407* (B); **Santiago del Estero**: ruta Nac. 89, Km. 488, desde Quimilí hacia Suncho Corral, 3 Km. al N de Suncho Corral, 27°56'19"S, 63°24'19"W, 146 m, 22 Mar 2006, *S.S. Aliscioni et al. 665* (BAA, CTES, SF, SI); Guasayán, Feb 1939, *J.L. Argañaraz 210* (LP); Campo Verde, 9 Jun 1943, *H.H. Bartlett 20417* (SI); along highway 12, Loreto, 10 Jun 1943, *H.H. Bartlett 20435* (GH, SI, UC, US); Piedra Buena, 23 Feb 1980, *A.L. Cabrera et al. 31896* (SI); Chaco santiagueño, Mar 1914, *R. Castañeda Vega 72* (SI); entre Tacañitas y Simbol Bajo, 19 Jan 1950, *Castiglioni et al. 7324* (US); entre RN 9 y Cajón, 12 Feb 1988, *A.A. Cocucci 248* (CORD); Arraga-La María, 27 Jan 1983, *Crespo s.n.* (LIL); El Retiro, km 80, 4 Jun 1986, *F. Dalla Tea 37* (CTES); 9 km al O de Ojo de Agua, en dirección a Amimán, 29°27'S, 63°46'W , 240 m, 26 Nov 1995, *R. Fortunato & R. Micheli 5090* (BAB); Quebrachos, Sumampa, 20 Apr 1945, *P. García 950* (LIL); Sierra de Guasayán, Quebrada de Maquijata, 570 m, 11 Jun 1982, *A.T. Hunziker et al. 24346* (CORD); Arraga-“La María”, 3 Jan 1988, *N.A. Jaime 23* (CTES); Copo, camino a El Desierto, 14 Nov 1984, *Kuntz et al. 78* (CTES); Río Hondo, Yutuyacu, 15 Jan 1949, *P.R. Legname 49* (LIL); Los Naranjitos, 18 Jan 1949, *P.R. Legname 117* (LIL); Mansupa, 24 Jan 1949, *P.R. Legname 165* (CTES, LIL); Brea Puñuna, 27 Jan 1949, *P.R. Legname 204* (CTES, LIL); camino de El Caburé a Monte Quemado, 2 Mar 1980, *P.R. Legname 7316* (CTES, LIL); La Banda, 14 Apr 1907, *M. Lillo 6206* (GH, LIL, UC); Beltrán, 12 Nov 1940, *R. Maldonado B. 504* (LP); ruta 9, al S de Termas de Río Hondo, 10 Mar 1987, *E. Marchessi et al. 1205* (BAA); ruta 64, entre Luján y San Lorenzo, 12 May 1983, *A.M. Molina et al. 1328* (BAB); RP 5, 17 km N de Tintina, 5 Mar 1986, *A.M. Molina & J. Hilfer 2788* (BAB); RN 16, 7 km SE de El Caburé, 7 Mar 1986, *A.M. Molina & J. Hilfer 3046* (BAB); Pellegrini, Cerro de Remate, 3 Apr 1989, *T.M. Pedersen 15395* (BAB, CTES, NY,

SI); camino a El Desierto, 14 Nov 1984, *K. Pérez & D. Tea 18* (BAB); Mal Paso, *Perrone s.n.* (BAF 8689); Simbol Cañada, Santos Lugares, 27°24'S, 64°11'W, 21 Feb 1977, *B.G. Piccinini & C.A. Petetin 2961* (BAB, CTES); RP 3, 2 km al N de Vinará, 27°22'S, 64°68'W, 21 Feb 1977, *B.G. Piccinini & C.A. Petetin 2980* (BAB, CTES); el Cevilar, *S.A. Pierotti (h) s.n.* (LE, LIL); Arraga-La María, 27 Jan 1982, *R. Renolfi 137* (CTES); Pozo Belbeder, 31 Jan 1984, *R. Renolfi 422* (BAB, CTES); Los Tigres, junto a vías entre Pueblo Nuevo y P. Viejo, 10 Mar 1970, *L.D. Roic 492* (BAB); Matará, Suncho Corral, 1918, *M. Rojas s.n.* (BAA 8589); Campo Gallo, 26 May 1949, *A. Soriano & W. Barrett 3592* (BAB); Choya, El Salvador (clausura Nº 5), 20 May 1961, *E. de la Sota 747* (LIL); Parque Nacional Copo, 50 km al NW de Pampa de los Guanacos, 25°53'41.9"S, 61°55'5.3"W, 1 Nov 1999, *J. Tolaba et al. 1375* (BAA, MCNS); Santiago del Estero, 1943, *L.A. Tortorelli s.n.* (BAB 29); entre Estación Tacanas y El Charco, 6 Mar 1966, *M. Villa Carenzo 2789* (LIL); **Tucumán**: entre Las Cejas y Tucumán, 24 Jan 1950, *J.A. Castiglioni & A.E. Ragonese 8027* (BAB 75599); Tapia, lomas al este, 12 Mar 1914, *L. Castillón 3568* (LIL); ruta 9, 5 km N de Tapia, 27 Mar 1975, *A. Krapovickas et al. 27879* (CTES, G); Graneros, La Madrid, 26 Jan 1886, *F. Kurtz 4221* (CORD); Vipos, 25 Mar 1887, *M. Lillo 325* (LIL) & *327* (P); Vipos, 19 Apr 1887, *M. Lillo 1949* (LIL); La Cañada, Río Tala, 13 May 1900, *M. Lillo 2533* (UC); San Pedro de Colalao, Río Tipas, 10 Mar 1946, *T. Meyer 9795* (LIL); Las Salinas, 270 m, 8 Jun 1913, *L. Monetti s.n.* (GH, LIL 62434); Río del Nío, 12 Feb 1914, *L. Monetti 1975* (GH, LIL); Las Cejas, 25 Jan 1947, *C.A. O'Donell 4328* (B, LIL, MO); Tapia og Vipos, 2 May 1953, *E. Petersen & J.P. Hjerting 947* (S); Trancas, 23 Dec 1913, *F. Rodríguez 1196* (BAB); Vipos, 786 m, 6 Jan 1922, *R. Schreiter 1927* (LIL); ruta 9, Puente sobre Arroyo Mista, 27 Jan 1955, *E. de la Sota 129* (G, LIL); Burruyacú, 20 May 1900, *T. Stuckert 9472* (CORD) & 20 Apr 1903, *T. Stuckert 13092* (CORD); Naranjo, 28 Mar 1944, *L.A. Varela s.n.* (LIL; UC); Piedra Buena, 18 May 1944, *L.A. Varela s.n.* (LIL); 200 m antes de Vipos, 30 Mar 1971, *F. Vervoorst s.n.* (*M.N. Correa 4233*) (BAB); Chañar Pozo, 300 m, Nov 1919, *S. Venturi 737* (LIL, SI); Tapia, 850 m, 14 Nov 1920, *S. Venturi 1066* (LIL, SI, US, UC); Cruz Alta, km 754, 416 m, 1 Dec 1922, *S. Venturi 1598* (BA, LIL, MA, SI, US);cerros del campo, 1200 m, 30 Dec 1928, *S. Venturi 7860* (CAS, GH, SI); Garmendia, 25 Dec 1958, *M. Villa Carenzo 424* (LIL); camino a San Pedro de Colalao, 25 Feb 1961, *M. Villa Carenzo & P.R. Legname 1440* (LIL); camino A. Miscta a Viclos, 25 Mar 1972, *M. Villa Carenzo 3499* (LIL).

BOLIVIA. Chuquisaca: Pampa Grande, cerca de Luis Calvo, 25 Apr 2014, *A.A. Cocucci & A. Sérsic 5488* (CORD); comunidad Thiu mayu, 68 km SSE de Padilla, 19°34'S, 64°06'W, 1200 m, 1 Jan 1953, *D.G. Debouck 3016* (LPB, MO); Salvador Cimboc, Clausura "El Huare", 750 m, 23 Jan 1992, *C. Saravia Toledo & J. Nelson 10343* (CORD, G); El Salvador Cimboc, el Centro, 800 m, 8 Mar 1992, *C. Saravia Toledo & J. Nelson 10815* (CORD); 6 km al E del puesto El Pato (camino de Carandaity a Campo Azul), 450 m, 8 Apr 1993, *C. Saravia Toledo et al. 11495* (CORD, CTES); Los Tamarindos, 430 m, 8 Apr 1993, *C. Saravia Toledo et al. 11496* (CTES); entre Tiguipa y Los Galpones (ruta a Carandaity), 550 m, 12 Apr 1993, *C. Saravia Toledo et al. 11544* (CORD, CTES); El Salvador-Cimboc, Puesto Nuevo I, 800 m, 6/7 May 1995, *C. Saravia Toledo 13472 & 13484* (CORD, CTES); Puesto Nuevo II, 800 m, 6 May 1995, *C. Saravia Toledo 13490* (CORD); desvío hacia el este de Boyuibe hacia Fortín Villazón, a 12,6 km E de Boyuibe, 20º26'42"S, 63º11'12"W, 707 m, 11 Feb 2005, *J.G. Seijo et al. 3864* (CORD, CTES); Prov. Oropeza, camino entre Imilla Huañusca y Puente Arce, 18º39,602'S, 65º10,224'W,

1744 m, 9 Mar 2003, *J.R.I. Wood & A. Carretero 19317* (LPB); **Cochabamba**: Cercado, Cantón Santa Ana de Calacala, Santa Ana, 1800 m, Feb 1980, *Coro-Rojas 1534* (CORD, LPB); **Santa Cruz**: Comarapa, Pulquina Abajo, predio del cactario, 18º06'03''S, 64º25'38''W, 1563 m, 11 Dec 2017, *G.E. Barboza et al. 4910* (CORD, LPB, P); Aguarati Izozog, al borde del camino para ir al chaco de M. Cuellar, desde Aguaratimi, 6 Dec 1998, *G. Bourdy 2000* (LPB); 15 Km E de San Ignacio, 411 m, 16°12'20"S, 60°58'58"W, 2 Apr 2006, *M. Dematteis et al. 2166* (CTES, SI); Tatarenda, 6 Apr 1902, *R. Fries 1547* (S); Ñuflo de Chávez, área de estudio del proyecto "BOLFOR", Las Trancas-95, 16°31'13"S 061°50'47"W, 450 m, 2 Mar 1995, *F. Mamaní M. 583* (MO, USZ); en las proximidades de la población de Cuevo, 20º26,911S, 63º33,396W, 1108 m, 7 Apr 2005, *C. Manchego CCP T25* (LPB, USZ); Fortín Ravelo (en el límite este del ANMI Kaa-Iya), 19º17,743S, 60º37,206W, 275 m, 30 Apr 2005, *C. Manchego CCP T26 & T27* (LPB, USZ); Fortín Ravelo, en el extremo E del parque Nacional Kaa-Iya, 19º17,743'S, 60º37,206W, 275 m, 30 Apr 2005, *C. Manchego CCP T28* (USZ); en la comunidad de Caplatindi, Izozog, 19º37,930S, 62º36,539W, 444 m, 24 Mar 2005, *C. Manchego CCP A1* (LPB); colectado en las cercanías de la población de Gutiérrez, 19º25.348'S, 63º31.434W, 954 m, 21 Feb 2005, *C. Manchego CCP 01* (USZ); en la ruta de Abapo a Camiri, al este de la población de La Herradura, 19º30,312'S, 63º31,025 W, 871 m, 22 Feb 2005, *C. Manchego CCP 03* (LPB, USZ); en la ruta Charagua a Izozog, dentro de la propiedad Mariqui, 19º50,265S, 62º55,853W, 588 m, 19 Mar 2005, *C. Machengo CCNP A1* (LPB, USZ); en la ruta de Charagua hacia Boyuibe, 20º17,444'S, 63º15,321'W, 855 m, 29 Mar 2005, *C. Manchego CCNP NT s.n.* (USZ); zona Izozog, en la ruta de Charagua a Boyuibe, 20º07,629'S, 63º07,124'W, 705 m, 29 Mar 2005, *C. Manchego CCNP T3* (LPB, USZ); close to the town of Yukeriti (or Yuque), off of road from Boyuibe to Charagua, 20.6258S, 63.25031W, 879 m, 19 Mar 2006, *C. Manchego & M. Simon 60062B, 60065B* & *60068B* (GH); same locality, -20.26254S, 63.25068W, 865 m, 19 Mar 2006, *C. Manchego & M. Simon 60076B* (GH); Ibisiriri, -19.56393S, -62.56690W, 21 Mar 2006, *C. Manchego & M. Simon 60087B* (GH); on road from Charagua to Santa Cruz, -19.23761, -63.12205, 380 m, 1 Apr 2006, *C. Manchego et al. 60194B* (GH); on road from Charagua to Santa Cruz, -19.23663, -63.12094, 594 m, 1 Apr 2006, *C. Manchego et al. 60198B* (GH); Ibasiriri Izozog, a 3 km de la Brecha, 300 m, 20 May 1999, *R. de Michel 2568* (LPB, NY, USZ); Pulquina, Jardín Botánico de Pulquina y sus alrededores, 18º04.10S, 64º24.30'W, 1557 m, 11 Jan 2004, *M. Mendoza 788* (LPB); Bañados del Izozog, 5 km al sur de Cachari, 350 m, 10 Mar 1991, *G. Navarro & I.G. Vargas C. 261* (LPB, MO); 3.7 km SW from turnoff the Camiri-Boyuibe highway at sign for Caño Guayrí, 20°12'55''S, 63°30'03''W, 875 m, 24 May 2008, *M. Nee et al. 55779* (USZ); bosque hermoso-Tucumano-Padilla, 20º23'60"S, 63º20'14"W, 1145 m, 27 Apr 2007, *T. Ortuño 769* (LPB); Vallegrande, 18°31'32''S, 64°17'32''W, 1066 m, 4 Feb 2013, *G.A. Parada et al. 5445* (MO, USZ); camino de Tucavaca a San José, salida del parque, 4 Feb 2005, *J.G. Seijo et al. 3820* (CORD, CTES, LPB, SI); Río Mizque, ca. 1 km W del puente del Río Mizque, sobre la carretera Saipina hacia Aiquile, 18º05,29'S, 64º42,04'W, 1486 m, 20 Feb 2003, *J.R.I. Wood & M. Mendoza 19144* (LPB); bajando de Pucará hacia Santa Rosa del Río Grande, 18º42,08'S, 64º16,24'W, 1644 m, 6 Mar 2005, *J.R.I. Wood et al. 21792* (LPB); **Tarija**: Abra del Portillo, a 10 km de Tarija rumbo a Padcaya, 1970 m, 25 Mar 2006, *G.E. Barboza et al. 1793* (CORD); Cerro Gamoneda, 2000 m, 15 Jan 1986, *E. Bastión 407* (LPB) & 16 Feb 1986, *E. Bastión 779* (CORD, LIL); Tarija, 28 Jan 1902, *R. Fries 1224* (S); Strasse v. Palos Blancos, hacia el N rumbo a P. Margerita, 940 m, 22 Feb 1982, *R. Gerold 284* (LPB); 5 km S de Río Pilcomayo, frente a

Villa Montes, camino a Yacuiba, 63º30'W, 21º18'S, 10 May 1977, *A. Krapovickas & A. Schinini 31154* (CTES, SI); 5 km antes de llegar al abra de la Cruz, camino a Padcaya, 1900 m, 30-31 Jan 1988, *M. Liberman et al. 1883* (CORD); a pocos kms de Villamontes, en la localidad de San Antonio, 21°17.346'S, 63°27.365'W, 384 m, 3 Apr 2005, *C. Manchego CCNP T12* (USZ); en la población de Ibibobo, en la ruta de Villamontes al Paraguay, 21º32,545S, 62º59,184W, 322 m, 4 Apr 2005, *C. Manchego CCNP T15* (LPB); San Jacinto, en la ruta hacia la represa del mismo nombre, 21º35.693'S, 64º42.483'W, 1849 m, 6 Apr 2005, *C. Manchego CCNP T23* (USZ); San Jacinto, en la ruta hacia la represa del mismo nombre, 21º35.647'S, 64º42.431'W, 1854 m, 6 Apr 2005, *C. Machengo CCNP T24* (USZ); a pocos kilómetros de la población de Palos Blancos, 21º30.778'S, 63º47.535'W, 837 m, 31 Mar 2005, *C. Manchego CCP T5* (LPB, USZ); a pocos kms de la población de Palos Blancos, dentro de la hacienda Tres Aguadas, 21°37.407'S, 63°46.459'W, 879 m, 31 Mar 2005, *C. Manchego CCP T6* (USZ); a pocos kms de Villamontes, en la localidad de San Antonio, 21°17.346'S, 63°27.365 W, 384 m, 3 Apr 2005, *C. Manchego CCP T11* (USZ); en la población de Ibibobo, en la ruta de Villamontes al Paraguay, 21°32.833'S, 62°59.278'W, 335 m, 4 Apr 2005, *C. Manchego CCP T13* (USZ); en inmediaciones a la población de Ibibobo, 21º30,429S, 62º59,500W, 339 m, 4 Apr 2005, *C. Machengo CCP T14* (LPB, USZ); ca. 20 km, a 20 km de Villa Montes, en inmediaciones de una estación petrolera, 21º17.628'S, 63º18.108'W, 380 m, 3 Apr 2005, *C. Manchego CCP NT 4* (USZ); cerca 18 km de Palos Blancos, camino a Villamontes, 21º25'18''S, 63º45'27''W, 736 m, 12 Feb 2008, *M. Mendoza et al. 2805* (USZ); Villamontes, 50 km camino a Tarija, 28 Mar 1995, *B. Mostacedo C. 2975* (MO); 33.5 km (by highway) and 33 km (air) SE of Villamontes on new highway to Isibobo and Paraguay, 21º22'07''S, 63º09'50''W, 350 m, 20 Mar 2007, *M. Nee & R. Flores S. 54790* (LPB, MO); 3 km by road W of Palos Blancos, at highest point on this part of the road from Palos Blancos to Entre Ríos, 21º24'45''S, 63º48'27''W, 875-915 m, 22 Mar 2007, *M. Nee & R. Flores S. 54863* (LPB, MO, NY, USZ); 6 km E of Palos Blancos on road to Villa Montes, 21°26'10"S, 63°43'58"W, 815 m, 23 Mar 2007, *M. Nee & R. Flores S. 54882* (LPB, MO); 3.5 km N of valley of Río Pilcomayo, along highway (this part still unpaved) from Villa Montes to Isibobo and Paraguay, 37.9 km (by road) and 37 km (by air) SE of Villa Montes, 21°24'01"S, 63°07'53"W, 435 m, 27 Mar 2009, *M. Nee & A. Molina O. 56085* (NY); a 31.5 km E de Caiza, rumbo a D'Orbigni, 21º51'40''S, 63º19'04''W, 561 m, 9 Jan 2005, *J.G. Seijo et al. 3351* (CORD, CTES); a 45,8 km E de Caiza, rumbo a D'Orbigni, 21º51'39"S, 63º11'14"W, 561 m, 9 Jan 2005, *J.G. Seijo et al. 3356* (CORD, CTES, LPB); Colón Norte, cerca de los terrenos del PETT, 21º46'S, 64º36'W, 1800 m, 11 Feb 1994, *M. Subieta 56* (LPB); by side of road between Villa Montes and Palos Blancos, 21º27.370'S, 63º42.555'W, 983 m, 11 Apr 2004, *J. Tewksbury 1020* (USZ); Narvaez, 1700 m, 5 Feb 1937, *J. West 8248* (MO).

PARAGUAY. Pilcomayo River, 1888-1890, *T. Morong 961* (F, GH, MO, NY, U, US); Gran Chaco, 10 Jun 1903, *A. Pride s.n.* (K); Loma Clavel, 5 Oct 1905, *T. Rojas 2605* (K); **Alto Paraguay**: Puerto Diana, 6 km de B. Negra, 8 Jan 1974, *P. Arenas 327* (CTES); Puerto Diana, a 5 km de Bahia Negra, 20°10'S, 58°10'W, Jan 1976, *P. Arenas 1393* (CTES, SI); Chaco septentrionalis, Dec 1907, *K. Fiebrig 1472* (AS, CORD, G, MO, S, Z); Colonia Potrerito, a 17 km SW de Bahía Negra, 23 Feb 1989, *T. Florentín Peña 29* (CTES); Puerto Casado, 19 Feb 1948, *W. Hartley 5H 132* (CAS); Palmar de las Islas, Estancia San José, 500 m al sur del casco, 9 Feb 1999, *R. Insua 513* (FCQ); Palmar de las Islas, Mayor P. Lagerenza, Mar 1989, *F. Mereles 2920* (FCQ, G); San Carlos del Alto Paraguay, 17 Sep

1986, *L. Molas & V. Vera 1067* (PY); Puerto Casado, Chaco Paraguayense, Dec 1916, *T. Rojas 2434* (CORD); Puerto Casado-Chaco Paraguayo (Puerto La Victoria), 26 Jan 1917, *T. Rojas 2440* (AS, CORD, MO); Bahía Negra, 30 Nov 1946, *T. Rojas 13815* (B, CORD, LIL, MO); Mayor Pedro Lagerenza, selva ribereña del Río Timane, 4 Apr 1978, *A. Schinini & E. Bordas 14930* (CTES); same locality, 6 Apr 1978, *A. Schinini & E. Bordas 15041* (CTES, SI); Ea. Alva Mater, km 145, 9 Apr 1996, *R. Vanni & D. Kurtz 3708* (CTES, G); Parque Nacional Defensores del Chaco, Madrejón área, 20º33'24''S, 59º50'28''W, 12 Feb 1999, *E.M. Zardini & J. Godoy 50089* (AS, MEXU, MO); Proposed Biosphere Reserve "Gran Chaco Americano": Cerro León, 20°25'46"S, 60°18'54"W, 9 Feb 2002, *E.M. Zardini & J.C. Rivas 58461* (MO); **Amambay**: Ayo. Estrella, Estancia Yamborandí, 22°20'S, 56°55'W, 7 May 1989, *N.M. Bacigalupo et al. 1136* (BAB, SI); Parque Nacional Cerro Cora, 22°40'S, 56°05'W, 250-300 m, 18 Mar 1983, *W.J. Hahn et al. 1213* (MO, PYO); same locality, 7 May 1984, *W.J. Hahn et al. 2506* (MO, PYO); **Boquerón**: Misión Santa Rosa, 21º45'S, 61º35'W, Feb 1981, *P. Arenas 1688* (CTES, NY, SI); Colonia Fernheim, Filadelfia, Mar 1981, *P. Arenas 1853* (CTES, SI); Colonia Fernheim, Filadelfia, 9 Jan 1984, *L. August 18* (CTES); Fortín E. Garay (prov. Nueva Asunción), 25 May 1984, *F. Billiet 3183* (MO, PY); Fortín Nueva Asunción, 20°43'S, 61°56'W, 24 Mar 1986, *D. Brunner 1569* (G, MO, PY); PN Teniente Enciso, alrededores de "la trinchera", 21°12'S, 61°39'W, 28 Mar 1986, *D. Brunner 1692* (MO, PY); alrededor de Escuela Esperanza Viva, 23º27'46''S, 60º04'26''W, 121 m, 27 Apr 2016, *A.A. Cocucci et al. 5977* (CORD); Filadelfia, 22°20'S, 60°05'W, 8 Jun 1983, *W.J. Hahn 1405* (G, PY); 7 km S de Filadelfia, 7 Dec 1992, *A. Krapovickas & C.L. Cristóbal 44232* (CTES, G, NY); ruta transchaco, 25 km SE de Nueva Asunción, 13 May 1994, *A. Krapovickas et al. 45375* (CTES, G, GH, SI); Campo Vía, a Colonia Neuland, 22°52'05"S, 59°58'08"W, 27 May 1993, *F. Mereles & R. Degen 5136* (CTES, FCQ, MO); Tyto. Fortín Toledo-ruta Trans Chaco, 22°23'31"S, 60°31'27"W, 17 Feb 2002, *F. Mereles 8651* (BAB, FCQ); P.N. Teniente Enciso, 20°54'27"S 61°50'25"W, 23 Feb 2006, *M. Peña-Chocarro 2536* (BM, CTES, FCQ, MO); ruta Trans-Chaco, 21º30'S, 61º15'W, 12 Mar 1979, *A. Schinini & E. Bordas 16524* (CTES); ruta Trans-Chaco, 61 km NW de Tte. Ochoa, 21º28'S, 61º22'W, *A. Schinini & R. Palacios 25726* (CTES, G); Mariscal Estigarribia-Chaco Paraguayo, 1 Jun 1980, *G. Schmeda 167* (CTES, FCQ); Campo Loro, 8 May 1987, *G. Schmeda 863* (CTES, FCQ, US); Gesudi, km 22 N Montania, 28 Oct 1990, *G. Schmeda 1301* (US); col. Neuland, Aeropuerto Boquerón, 13 Sep 1990, *R. Vanni et al. 2081* (CTES, G); Reserva de la Est. Exp. Isla Poí, 26 Feb 1991, *R. Vanni et al. 2282* (CTES); Est. Exp. Filadelfia, 1 Mar 1991, *R. Vanni et al. 2561* (CTES, G); cruce de camino Tte. Montania-Madrejón con el camino a Pitiantuta, 21°26'46.6''S, 59°52'10,4''W, 8 Feb 2005, *C. Vogt & F. Mereles 231* (CTES, FCQ); PN Teniente Agripino Enciso, 21°13'S, 61°39'W, 19 Jan 1994, *E.M. Zardini & L. Guerrero 37883*, *37913* & *37916* (AS, MO); between Mariscal Estigarribia and Cruce to Loma Plata, Route 9, Km 504, 22°13'S, 60°24'W, 25 May 1994, *E.M. Zardini & T. Tillería 39351* (AS, MO); between PN Teniente Agripino Enciso and Nueva Asunción, 20°53'23"S, 61°50'56", 27 Jan 1995, *E.M. Zardini & A. Acosta 42322*, *42328* & *42346* (AS, MO); PN Teniente Agripino Enciso and Nueva Asunción, 20°57'10"S, 61°48'47"W, 270 m, 27 Jan 1995, *E.M. Zardini & A. Acosta 42450* & *42445* (AS, MO); **Caazapá**: Tavai, de Y-hovy, 500 m, 26°10'S, 55°17'W, 18 May 1989, *N. Soria 3900* (CTES, FCQ, MO); **Central**: Tablada Nueva, 28 Jan 1944, *C. Pavetti & T. Rojas 10821* (AS, CORD, MO); Asunción, Tablada Nueva, 23 Nov 1950, *T. Rojas 14325* (BAF, CORD, CTES, LIL); Cerrito de Estansuela-Areguá, Feb 1972, *A. Schinini 4248* (CTES, G, US); **Concepción**: Arroyo

Tagatiya-mí, 22°37'S, 57°32'W, 140 m, 4 Apr 1986, *D. Brunner 1741* (MO, PY); Arroyo Tagatiyá-Mí, 22°37'S, 57°32'W, 16 Mar 1994, *E.M. Zardini 38869* (AS, MO); **Cordillera**: Eastern side of Río Piribebuy basin, 17 km W of Arroyos y Esteros, 25°08'S, 57°15'W, 3 Mar 1990, *E.M. Zardini et al. 19653* (AS, MO); **Presidente Hayes**: Paratodo, Colonia Menno, 13 Mar 1974, *P. Arenas 436* (BAB, CTES); Estancia Loma Pytá, 2 Apr 1974, *P. Arenas 555* (SI); Estancia Loma Pyta, 23º40'S, 59º35'W, 7 Dec 1978, *P. Arenas 645* (CTES, SI); Fortín General Bruguez, 24º45'S, 58º50'W, 3 Jan 1980, *P. Arenas 1546* (CTES, SI); Nanawa, Ing. E. Ayala, 23º24'S, 59º45'W, 1981, *P. Arenas 2377* (CTES, SI); Qemkuket (comunidad Maká), desvío de la ruta 9 a Puerto Falcón, 6 Jan 2010, *P. Arenas 3764* (CTES, FCQ); Estancia Ñ, 1 Feb 2005, *J. De Egea & M. Peña-Chocarro 769* (CTES, FCQ, MO); cercanías de Pozo Colorado, 15 Apr 1994, *F. Mereles 5536* (FCQ, MO); Benjamín Aceual, Compañía colinia Rio Verde, km 49, near a Toba community, 24º56'58.3''S, 57º33'09.8''W, 60 m, 15 May 1998, *K.A. Williams et al. 140* (CORD, FCQ).

Cultivated
ARGENTINA. Buenos Aires: Capital Federal, cult. Jardín Botánico de la Facultad de Agronomía y Veterinaria, UBA, 7 Apr 1945, *J.J. Dauny s.n.* (CORD 00029474); cult. Jardín Botánico de la Facultad de Agronomía, UBA, semillas del Mercado Municipal de Jujuy (Argentina), 1 Jun 1942, *A.T. Hunziker 2104* (CORD); cult. ciudad de Buenos Aires (Seguí 430), semillas de origen desconocido, 27 Mar 1948, *A.T. Hunziker 2452* (CORD); cult. ciudad de Buenos Aires (Seguí 430), semillas de origen desconocido, 13 Jan 1947, *A.T. Hunziker 7315* (CORD); cult. Jardín Botánico de la Facultad de Agronomía y Veterinaria, UBA (Villa Ortuzar), Jan-Feb 1943, *A.T. Hunziker 7356, 7359 & 7360* (CORD); cult. Jardín Botánico de la Facultad de Agronomía y Veterinaria, UBA, semillas de La Despensa (Salta), 4 Mar 1943, *A.T. Hunziker 7358* (CORD); **Córdoba**: Capital, cult. Fac. Ciencias Exactas, Físicas y Naturales, semillas de Saldán (Córdoba, Argentina), Jan 1966, *A.T. Hunziker 18572* (CORD); RN 9, Pilar, leg. *R. Subils*, cult. en jardín, 14 Feb 1988, *A.T. Hunziker 25219* (CORD).

UNITED STATES OF AMERICA. California: grown at Davis (UC), seeds from Argentina, 1953, *P.G. Smith Ac 689* (UC); grown at Davis (UC), seeds from San Luis (Argentina) sent by A. Burkart through C. B. Heiser, 1953, *P.G. Smith Ac 1256* (UC); **Indiana**: Bloomington, cult. IU, seeds from Argentina, 15 Sep 1948, *C.B. Heiser Jr. C 10* (CORD).

11. Capsicum chinense Jacq.
ARGENTINA. Salta: Santa Victoria, PN Baritú, Lipeo, casa de Clemencia Ugarte, 8 Dec 1997, *N. Hilgert 2060* (MCNS).

BELIZE. Corazal: Calcutta, 18º20'N, 88º25'W, 10 m, 18 Nov 1989, *M.J. Balick 2211* (NY).

BOLIVIA. Beni: Ballivián, Rio Chimanes, environs of Fatima, 29 May 1981, *E. W. Davis 1066* (GH); Ballivián & Yacuma, Estación Biológica del Beni (EBB), comunidad Chaco Brasil, 14º30'S, 66º37'W, 200 m, 20 Oct 1995, *I. Guareco 627* (NY); Estación Biológica del Beni, 14 Dec 1994, *E. Rivero 67* (NY); EBB, Tierra Santa, 14°30'S, 66°37'W, 200 m,

19 Jun 1995, *E. Rivero 428* (LPB); Estancia El Porvenir, 50 km E of the Río Maniqui (San Borja), on the road to Trinidad, 14°49’S, 66°25’W, 250 m, cult., 12 Nov 1985, *J.C. Solomon 14672* (LPB); Rurrenabaque, dooryard garden, 14°30’S, 67°30’W, 227 m, cult., 5 Aug 1989, *D.E. Williams 934* (CORD, CTES); Carmen Florida, Tacana Indian village 7 km upstream of Rurrenabaque on Rio Beni, 14°30’S, 67°30’W, 240m, 13 Dec 1990, *D.E. Williams 1177* (CTES, LIL, LPB); **Cochabamba**: Carrasco, Chimoré, comunidad Yuqui de Bia Recuate, 190 m, 22 May 2004, *E. Martínez S. et al. 246* (NY); Chapare, territorio Indígena Parque Nacional Isiboro-Secure, community of San Antonio, 16°24’S, 65°54’W, 240 m, cult., 17 Apr 2004, *E. Thomas 705* (LPB); territorio Indígena Parque Nacional Isiboro-Secure, community of Sanandita, 16°32’S, 65°29’W, 210 m, cult., 30 Sep 2004, *E. Thomas 1092* (NY) & *1093* (LPB); **La Paz**: Franz Tamayo, en la senda entre Buena Vista y Ubito, 14º26’S, 68º28’W, 1350 m, 10 Sep 1993, *N. Helme 80* (NY); Iturralde, San José de Uchupiamonas, 35 km W on foot trail from Tumupasa, 14º15’S, 68º10’W, 630 m, 24 Oct 1988, *D.E. Williams 772 (*LPB); same locality, *D.E. Williams 773* (CORD, CTES, LPB, NY); **Pando**: Abuná, Nacebe sobre el Río Orthón, Tierra Santa, 11 Oct 1989, *S. Beck et al. 19317* (COL, CORD, Q); **Santa Cruz**: Ñuflo de Chávez, Embocada del Carmen, ca. 40 km SSW de Concepción, 16º38’S, 62º26’W, 550 m, cult., 2 May 1977, *A. Krapovickas & A. Schinini 32488* (CORD, CTES); Guarayos, Reserva de Vida Silvestre Rios Blanco y Negro, alrededores del campamento, 14°43.5’S, 63°58.4’W, 250 m, 22 Jun 1993, *I.G. Vargas C. et al. 2666* (F, LPB); same locality, 22 Jun 1993, *I.G. Vargas C. et al. 2667* (F, MEXU).

BRAZIL. Acre: Cruzeiro do Sul, Serra do Moa, 23 Jan 1980, *Penereiro 11062* (UEC); **Amapá**: Macapá, APA da Fazendinha, 26 May 2009, *L. Espindola-Nascimento & L.S. Simoa 04, 05 & 06* (HAMAB, RB); same locality, 28 May 2009, *L. Espindola-Nascimento & J.R. Pinheiro 13* (HAMAB, RB); APA do rio Curiau, Comunidade Curiau de Dentro, 2 May 2008, *L.A. Pereira et al. 1699* (HAMAB); Distr. Fazendinha, Minipolo Hortifruti, 5 Jul 2008, *L.A. Pereira et al. 1816, 1817* & *1821* (HAMAB, RB); Vila do Trem, Rod. BR 156, Km 10, 8 Jul 2008, *L.A. Pereira et al. 1827, 1828, 1831 & 1833* (CEN, HAMAB, RB), *L. A. Pereira et al. 1835* (CORD); Linha D, Rodovia AP 20, Km 8, 8 Jul 2008, *L.A. Pereira 1837* (HAMAB, RB); Santana, Ilha de Santana, 12 Jul 2008, *L.A. Pereira et al. 1839* (CEPEC, HAMAB, HB, RB); *L.A. Pereira et al. 1846* (HAMAB, HB, RB); Vila Ressaca, Rod. do Curiau, 14 Jul 2008, *L.A. Pereira 1851, 1852* (HAMAB, RB); Comunidade Curiau de Fora, 14 Jul 2008, *L.A. Pereira & W.M.S.Severino 1854* (HAMAB, RB); Ilha de Santana, 19 Jul 2008, *L.A. Pereira et al. 1864* (HAMAB, RB); *L.A. Pereira et al. 1868* (HAMAB, HB, RB); *L.A. Pereira et al. 1870* (HAMAB); Horto do IEPA, 20 Jul 2008, *L.A. Pereira 1873* (HAMAB, HB, RB); Vila Ressaca, 5 Nov 2008, *L. A. Pereira et al. 1896* (CORD, RB); Minipolo Hortifruti, 8 Nov 2008, *L.A. Pereira et al. 1901, 1902, 1904, 1905, 1906* & *1908* (CORD, BHCB, HAMAB, RB); Vila do Trem, Rod. BR 156, Km 10, 10 Nov 2008, *L.A. Pereira et al. 1918 & 1920* (BHCB, CORD, HAMAB, RB); **Amazonas**: Tefé, 30 Jul 1988, *L. Krieger 22981 pp*. (BHCB); **Bahia:** Ilheus, Área do CEPEC, km 22 da Rodovia Ilhéus/Itabuna, 23 Oct 1979, *J.L. Hage & E. B. dos Santos 356* (F); Mata de Sâo João, Vila Sauípe, Quintal de D.Mirene, 8 Jul 2002, *E. von S. Medeiros et al. 182* (RB); Vila Sauípe, Quintal de D. Tânia, 10 Jul 2002, *E. von S. Medeiros et al. 196* (RB); Vila Sauípe, Quintal de D. Tânia, 10 Jul 2002, *E. von S. Medeiros et al. 200* (RB); **Distrito Federal**: Reserva Ecológica do IBGE, cult., 13 Jun 2000, *T.S. Filgueiras 3614* (RB); **Goiás**: Caldas Novas, próximo a Corumba, 27 Apr 1994, *Dos Santos et al. 274, 275, 276* & *277* (UB, CEN); Niquelandia, Rua 7 de Setembro Nº 12, 14º04'S, 48º04'W, 580 m, 6

Apr 1988, *L.A. Skorupa & J.N. da Silveira 329* (CEN); Alvorada do Norte, Fazenda Angical, 12º05'S, 49º02'W, 300 m, 9 Apr 1988, *L.A. Skorupa & J.N. da Silveira 378* (CEN); **Mato Grosso:** Nova Lacerda, campamento abandonado da BR-364 junto ao Corrego Dourado, 14º10'59"S, 59º 41'46"W, 290 m, 19 May 1985, *J.F. Montenegro Valls et al. 8782* (CEN, CORD); **Minas Gerais**. cult. in Viçosa, 11 Apr 1988, *A.T. Hunziker 25234* (CORD); **Para**: Estacão Ecologica do Jari, 0º75'S, 52º30'W, 13 Oct 1987, *H.T. Beck et al. 90* & *91* (NY, US); Conceição do Araguaia, ca. 20 km W of Redenção, near Côrrego São João and Troncamento Santa Teresa, 8º3'S, 50º10'W, 350-620 m, cult., 13 Feb 1980, *T. Plowman et al. 8812* (F); **Paraíba**: João Pessoa, Jardim Botanico, 7º60'S, 34º52'W, 10 Aug 2007, *P. da C. Gadelha Neto 1748* (CORD); **Parana**: Antonina, São João, 25 Dec 1975, *G. Hatschbach 34886* (CORD, MBM); **Pernambuco**: Tapera, 15 May 1935, *B. Pickel 3964* (US); **Rio de Janeiro**: Paraty, Praia de Martim de Sá/Reserva Ecológica da Juatinga/APA de Cairuçu, 23°19'20''S, 44°24'26''W, 15 Mar 2006, *R. Borges 67* (RB); cult. Chacara de Turiaçu, 25 Jul 1989, *S.L. Gonçalves & M. Kaketru 1 & 2* (RB); **Rondônia**: Vilhena, 34.4 km do entroncamento da BR-364 em direção a Colorado do Oeste, 12º57'S, 60º27'W, 350 m, 8 Oct 1990, *B.M. Teles Walter et al. 567* (CEN); Cerejeidas, Pimenteiras, na cidade, divisa com a Bolívia, horta de fundo de quintal, 13º28'S, 61º03'W, 230 m, 8 Oct 1990, *B.M. Teles Walter et al. 572, 573* & *576* (CEN); Cerejeidas, casa de moradores da cidade, proprietária Divina/Célio Preto, 13º28'S, 61º03'W, 250 m, 8 Oct 1990, *B.M. Teles Walter et al. 579* (CEN); **Roraima**: Cantá, in open areas of Amazon rainforest, 02º30'52.2''N, 60º47'48.5''W, 87 m, 17 Sep 2014, *M.V. Romero 9* (UEC); **São Paulo**: São Paulo, Parque do Estado de São Paulo, 4 May 1944, *W. Hoehne s.n.* (JPB); Pinheiros, 22 May 1928, *F.C. Hoehne s.n.* (US 1617106); Campinas: IAC, Fazenda Sta. Elisa, CEC-Centro de Hortaliças, cult., -22.9056S, -47.0608 W, 854 m, 3 Apr 2017, *N.S. Lélis et al. 1* (CEN, RB), *2* (RB) & *12* (CEN, RB); **Santa Catarina**, Garuva, ingreso al Parque Acuático Monte Crista, 24 Feb 2006, *G.E. Barboza et al. 1622* (CORD).

COLOMBIA. Amazonas: Leticia, km 12 desde Leticia a Pedrera, 14 Apr 1975, *Cabrera 3365* & *3366* (COL); Puerto Santander, margen derecha del Río Caquetá, 0°37'7.1''S, 72°23'8.4''W, 213 m, *D. Cárdenas et al. 9311* & *9317* (COL, COAH); Río Caquetá, Isla Clemencia, 0°32'56''S, 72°9'54''W, 290 m, *D. Cárdenas et al. 9331* (COL); comunidad Perey, resguardo Aduche, 0°47'18''S, 72°1'39''W, 427 m, *D. Cárdenas et al. 9356* (COL); Chorro del Quinche, 0°55'50''S, 71°46'47''W, 335 m, *D. Cárdenas et al. 9361* (COL); margen derecha del Río Caquetá, San Francisco, 1°6'21''S, 71°6'18.8''W, 183 m, *D. Cárdenas et al. 9377* (COL); Resguardo Miraña Bora, Parque Cahuinarí, Las Palmas, 1°16'8.4''S, 70°57'59''W, 213 m, *D. Cárdenas et al. 9385* & *9393* (COAH, COL); Miriti-Paraná, margen derecha del Río Miriti, Puerto Remanso, comunidad Quebrada Negra, *D. Cárdenas et al. 9431* (COAH, COL); Corregimiento La Pedrera, comunidad Camaritawa, 1°11'27''S, 69°35'14''W, 79 m, *D. Cárdenas et al. 9439* (COL); Puerto Santander, Resguardo indígena Nonuya de Villazul, comunidad de Peña Roja, 100-200 m, 30 Mar 2000, *S. Castro & A. Rodríguez 132* (COL); Puerto Santander, Isla Sumaeta, 100-200 m, 17 Apr 2000, *S. Castro & A. Rodríguez 307, 308, 310, 312, 313 & 314* (COL); same locality, 100-200 m, 17 Apr 2000, *S. Castro & A. Rodriguez 309 & 311* (COL, HUAZ); Meta, alrededores de la casa del Sr. C. Matapí, 100-200 m, 12 Nov 2000, *S. Castro & I. Matapí 563* & *564* (COL); Puerto Santander, Meta, alrededores Chagra de Ramiro Matapí, 100-200 m, 12 Nov 2000, *S. Castro & I. Matapí 561* (HUAZ)& 565 (COL, HUAZ); Río Igará

Paraná, 27 Aug 1987, *C.I. Henao & Buraiño 26* (COL); Reserva Indígena Miraña, 13 Jun 1984, *La Rotta et al. 435* (COL); Amazonas, 24 Jul 1965, *Lozano et al. 577A* (COL); Puerto Nariño, 24 Jul 1965, *G. Lozano et al. 642* (COL); Loretoyacu River, 100 m, 20/30 Oct 1945, *R. E. Schultes 6620* (US); Puerto Nariño at Loreto Yacu River, 200 m, 12 Sep 1963, *D.D. Soejarto & H. Cardozo 813* (GH); **Antioquia**: Puerto Berrío, 125 m, 11 Jan 1931, *W.A. Archer 1404* (US); La Pintada, carretera La Pintada-Valparaíso, 5°40'N, 75°35'W, 640 m, 3 Oct 1987, *O. Marulanda & S. Churchill 490* (COL); **Caquetá**: Solano, inspección Araraquara, Shuchichi, 3°62'28''S, 72°28'27''W, 244 m, *D. Cárdenas et al. 9308* (COL); Milán, Inspección San Antonio de Getucha, Resguardo indígena Gorgonia, 218 m, 1°05'152''N, 75°28'219''W, 29 Aug 2006, *W. Trujillo 130* (HUAZ); **Casanare**: Margen derecha del Río Casanare, caserío indígena Morichito, 100 m, 7 Jul 1977, *C. de Camargo 020* (COL); **Chocó**: Acandi, Bahía El Aguacate, camino hacia la quebrada la Mora, 30 Jun 2005, *S.E. Hoyos et al. 348* (COL); Río Sucio, Parque Natural Nacional Los Katyos, alrededores de Peye, 50 m, 12 Jun 1976, *H.A. León 20* (MO); **Guainía**: Caserío de Karanacoa, en el Río de Guainía, 150 m, 12 Oct 1977, *J. Espina et al. 182* (COL); sitio Santa Helena, en el Río Negro, 75 m, 28 Oct 1977, *J. Espina et al. 276* (COL); Caserío Santa Rita, 55 m, 5 Oct 1977, *M.A. Pabón E. 257, 258, 259 & 277* (COL). **Meta**: jardines y prados del municipio Villavicencio, 500 m, 20 Apr 2011, *F.L. Quevedo et al. 1816* (COL); Villavicencio, 19 Jan 2005, *L. G. Velásquez Castillo MA-8* & *9* (COL); **Nariño**: Tumaco, Resguardo Indígena Alto Albí, 6 May 1992, *González 89* (COL), Buesaco, Vereda El Cortijo, a 13 km de la vía de Villamoreno, 2500 m, 29 Apr 1984, *O. de Benavides 4672* (PSO); **Norte de Santander**: frontera Colombia-Venezuela, 30 km de Río de Oro por el Caño del noreste, 300-500 m, 14 Oct 1964, *P. Solange 8* (COL); **Putumayo**: Río San Miguel o Sucumbios, Conejo y los alrededores, frente de la Quebrada Conejo, 300 m, 2-5 Apr 1942, *R.E. Schultes 3497* (GH); **Santander**: Suaita, corregimiento San José de Suaita, a la cascada, por la vía de Guadalupe, 27 Sep 2003, *J.L. Fernández-Alonso et al. 20758* (COL); Piedecuesta, km 45A, 9 Nov 2011, *N. Rosado 01* (COL); **Valle del Cauca**: Candelaria, entre Gorgona y Cabuyal, 1000 m, cult., 2 Jun 1943, *J. Cuatrecasas 14479* (COL, CORD, F, US); Mondomo, Angel, *M. Torres & F. Rodriguez 2032* (FMB); **Vaupés**: Mitu, comunidad Garrafa, margen derecha del caño Cuduyari, 1º17'42''N, 70º17'1.4''W, 180 m, *F. Rodriguez 79* (COL); Yavaraté, Piracuara, 27 Nov 1952, *R. Romero Castañeda 3797* (COL, GH); Río Macaya, vicinity of Cachivera del Diablo and mouth of river, 300 m, May 1943, *R. E. Schultes 5518* (COL); Río Kananarí, c. 250 m, 25 Jul 1951, *R.E. Schultes & I. Cabrera 13138* (COL); **Vichada**: Mun. Cumario, Cumaribo, región del bajo Mucu, inspección de S. José de Ocune, 140 m, 2 Sep 2001, *J.E. García Guzmán s.n.* (COL 468677).

COSTA RICA. **Heredia**: Finca La Selva, the OTS Field Station on the Rio Puerto Viejo just E of its junction with the Río Sarapiquí, 100 m, 14 Nov 1982, *T. McDowell 796* (F).

CUBA. **Matanza**: Ad Jagüey, 600 m, Apr 1889, *H.F.A. von Eggers 5302* (K); **Villa Clara**: Aromao River, Soledad, Aug 1941, *R.A. Howard 6278* (GH, P).

ECUADOR. Esmeralda: km 170-175, vía Santo Domingo-Quinindé, 300 m, 12 Sep 1949, *M. Acosta Solís 13989* (F); Río Cayapa, Zapallo Grande, 0º49'N, 78º57'W, 100 m, 30 Jun 1982, *L. P. Kvist 40566* (QCA, QCNE) & *40586* (QCNE); **Guayas**: San Ignacio, near Tenguel, 23 Feb 1919, *I. Holmgren & O. Heilborn s.n.* (S); **Morona-Santiago**: Macas, N

of the village, c. 900 m, 14 Mar 1956, *E. Asplund 19746* (S); alrededores de Mutinza, SW de Makuma, 2º12'S, 77º44'W, 650-750 m, 4 Dec 1996, *S. Báez et al. 28* (QCA); Centro Shuar Yukutais, 10 km S Sucua, Rio Upano, 2º31'S, 78º09'W, 25 Sep 1988, *B. Bennet & P. Gómez A. 3441* (QCNE); Palora, Sangay, huerto casero de la comunidad de Chinimp, 1º48'S, 77º55'W, 980 m, *V. Caballero Serrano 006* (CHEP); cantón Macas, 2º17'S, 78º60'W, 1070 m, 1 Jul 2004, *D. A. Herrera 16* (QCA); alrededores de la Misión Salesiano, Bomboiza, 800 m, 24 Jul 1985, *J.O. Pujupet RBAE 1005* (NY); **Napo**: Puerto El Carmen del Putumayo, 0º5'N, 75º52'W, ca. 200 m, 1 Aug 1980, *R. Andrade 33001B* (QCA); Comuna San José de Payamino at Río Payamino, 0º30'S, 77º18'W, 300 m, cult., 1-7 Dec 1983, *H. Balslev & D. Irvine 4582* & *4598* (QCA, QCNE); San Pablo de los Secoyas, on the Rio Aguarico, 0°18'S, 76°20'W, 300 m, cult. by the Secoya-tribe, 21 Feb 1984, *H. Balslev 4890, 4892, 4894, 4895* & *4904* (QCA, QCNE); Jatun Sacha Biol. Station, 23 km W of Puerto. Napo, 1º04'S, 77º39'W, 12 Jul 1990, *B. Bennet et al. 4276* (QCNE); Cantón Aguarico, Chiro Isla community, north bank of Rio Napo, ca. 195 km downstream from Puerto Francisco Orellana (Coca), 00º36'S, 75º52'W, 200 m, 13-19 Dec 1990, *R. Bensman 310* (QCNE); Shiripuno, 1º01'S, 77º05'W, 280 m, 31 Jul 1993, *D. Bolotin 21* (QCA); San Pablo de los Secoyas, 0º15'S, 76º21'W, 300 m, 13 Aug 1980, *J. Brandbyge & E. Asanza 32813* (MO); Reserva Biológica Jatun Sacha, 8 km al E de Misahualli, 1º04'S, 77º36'W, 450 m, 24 Apr-5 May 1987, *C.E. Cerón et al. 1292 & 1299* (QCNE); the same locality, 24 Aug 1988, *C.E. Cerón & M. Cerón 4712* (QCNE); Dureno, comunidad Cofán, al sur del Río Aguarico, 20 km al E de Lago Agrio, 0º05'N, 76º40'W, 350 m, 27 Dec 1988, *C.E. Cerón et al. 5849* (QCNE); confluence of Quiwado and Tiwaeno Rivers, Waorani, 1º50'S, 77º40'W, 18 Apr 1981, *E.W. Davis & J. Yost 993* (GH, QCA); Río Aguarico, 40 km downstream from San Pablo de los Secoyas, 0º16'S, 76º11'W, 230 m, 14 Feb 1980, *L. Holm Nielsen et al. 21109* (QCA, QCNE); Río Aguarico, N of bank of river at San Pablo de las Secoyas, 0º17'S, 76º24'W, 230 m, 21 Feb 1980, *L. Holm-Nielsen et al. 21696* (AAU, NY, MO, QCA); carretera Hollín-Loreto, km 40-50, alrededores de la comunidad Huamani y del Rio Pucuno, 00º43'S, 77º36'W, 1200 m, 10-22 Oct 1988, *F. Hurtado 715* (QCNE); village of Dureno, c. 300 ft, Aug 1963, *R. T. Martin 75* & *168* (GH); Río Shiripuno, Quehueiri-ono, 1º12'S, 77º9'53''W, 250 m, 15 May 1995, *J. Miller & P. Yépez 651, 652 & 667* (QCA, QCNE); along Rio Napo, ca. 20 km E of Puerto Misahualli, 1º01'S, 77º30'W, 450 m, 5 Jul 1986, *J.S. Miller et al. 2404* (QCNE); on Rio Aguarico, cult., 6 Jul 1966, *H.V. Pinkley 230, 256, 257* & *258* (GH); same locality, 6 Dec 1966, *H.V. Pinkley 259* (GH, S); same locality, 22 May 1966, *H.V. Pinkley 544* (GH), Rio Aguarico, Dureno, environs of village, cult, 1 Aug 1974, *T. Plowman et al. 4054* & *4055* (COL, GH); cercanías de Archidona, 0º56'S, 77º50'W, 610 m, 13 Jan 2001, *F. Prieto 21* (QCA); Ahuano, 1º6'S, 77º35'W, 310 m, 7 Jun 1993, *M. Ríos 429* (QCA, QCNE); same locality, 7 Jun 1993, *M. Ríos & A. Oña 430* (QCA, QCNE); Tena, alrededores del pueblo, cult. jardines, 19 Aug 1995, *V. Tafur 219* (F); Río Aguarico, Shushufindi, 244 m, in house garden, 14 Feb 1975, *W.T. Vickers 115* (CHEP); same locality, 27 Feb 1975, *W.T. Vickers 178* (CHEP); **Napo-Pastaza**: Vera Cruz, at paht in forest, c. 900 m, 18 Feb 1956, *E. Asplund 19430* (S); **Orellana**: Comunidad Indillama (etnia Kichwa), a 20 minutos en canoa desde el Puerto Itaya, 0°26'S, 76°31'W, 250 m, 11 May 2004, *L. Carrillo & D. Reyes 434* (MO, QCNE); Aguarico, Parroquia El Edén, comunidad El Edén, 15 Feb 2005, *L. Carrillo & D. Reyes 591* (QCNE); Reserva Étnica Huaorani, Comunidad Bataburo, 01º09'49''S, 76º55'54''W, 290 m, 27 Mar 2004, *B. Freire & D. Naranjo 612* (QCNE); same locality, 27 Mar 2004, *D. Naranjo & B. Freire 329* (QCNE); Avila Viejo, 0º38'S, 77º25'W, 600-800 m, 6 Oct 1996,

E.O. Kohn 1611, 1612 & 1613 (QCNE); same locality, 29 Mar 1997, *E.O. Kohn 1896* (QCNE) & 31 Mar 1997, *E.O. Kohn 1910* (QCNE); Comunidad Indillama, a 0:30 horas en deslizador del Puerto Pompeya, aguas abajo por el Rio Napo, 0°26’S, 76°31’W, 280 m, 6-20 May 2004, *D. Reyes & L. Carrillo 502* (QCNE, MO); Parroquia El Edén, comunidad El Edén, 0º29’41’’S, 76º04’37’’W, 200 m, 15 Feb 2005, *D. Reyes & L. Carrillo 773* (QCNE); **Pastaza**: Kapawí (Amuntai), Río Pastaza, 2°31’S, 76°48’W, 235 m, 14-20 Jul 1988, *W.H. Lewis et al. 13607* (QCNE, MO); Captaine Chiriboga, Río Pastaza, 2º32’S, 76º49’W, 235 m, 25-29 Jul 1988, *W.H. Lewis et al. 13960* (QCNE); Kapawí (Amuntai), 25-29 Jul 1989, *W.H. Lewis et al. 14010* (QCNE, MO); alrededores del Pozo Villano “B” (AGIP) y en el sendero al Río Uniquino, 1º27’10’’S, 77º26’32’’W, 320-450 m, 12 Mar 2008, *A.J. Pérez et al. 3833* (QCA); **Sucumbíos**: Gonzalo Pizarro, Parroquia Puerto Libre, comunidad Cofán de Sinangüe, 0º08’N, 77º27’W, 700-800 m, Sep 1993, *C.E. Cerón 20848* (QCNE); Shushufindi, comunidad Secoya San Pablo de Cantesiayá, sendero Sehuayaha, 0º15.58’S, 76º27.33’W, 300 m, 6 Apr 2008, *C.E. Cerón & C. Reyes 62013* (QAP); Anangu, Río Napo, cult. in and around the village, 0º31’S, 76º23’W, 260 m, 28 Jun 1983, *J.E. Lawesson et al. 39639 & 39640* & 39646 (QCA, QCNE); Cuyabeno, Parroquia Tarapoa, comunidad Siona de Sototsiaya, río debajo de Poza Honda, 0º14’27’’S, 76º26’15’’W, 230 m, 25 Feb 2005, *N. Miranda & G. Moya 436 & 437* (QCNE); Shuchufindi, Comunidad Santa Elena, 8 km de la vía principal a Puerto Itaya, 0°20’46’’S, 76°33’14’’W, 250 m, 2-6 Feb 2004, *G. Moya & D. Reyes 206 & 207* (QCNE); same locality, 2 Feb 2004, *D. Reyes & G. Moya 234* (QCNE); **Zamora-Chinchipe**: Nangaritza, comunidad Chumbias, 4°16’32’’S, 78°43’56’’W, 1020 m, 28 May 2001, *F. Santín et al. 102* (LOJA).

FRENCH GUIANA: Acarouany, cult., *Herb. Sag. 465* (GOET); Cayenne, 5 Apr 1986, *M. Fleury 124* (NY).

GUYANA. Cuyuni-Mazaruni: Kubinang village, W bank of Mazaruni R, between Kukuni R and Kako R, ca. 460 m, 14 Sep 1960, *S.S. Tillett & C.L. Tillett 45424* (NY); Morowda villagen, near Kako mouth, 470 m, 4 Oct 1960, *S.S. Tillett & C.L. Tillett 45627* (NY).

HONDURAS. Morazán: El Zamorano, Yeguare Valley river, 800 m, 6 Sep 1969, *A. Molina R. 24525* (US).

MEXICO. Campeche: Calakmul, Ej. Narciso Mendoza, km 33 al S de Xpujil, 18º13’40’’N, 89º27’12’’W, 240 m, cult., 12 Oct 1997, *S. Ramírez A. 56* (MEXU); **México**: Temascaltepec Dist., Acatitlan, 18 Aug 1933, *G. B. Hinton 4336* (UC, US); Tlahuac Dist., chinampas at San Andrés Mixquic, 19º13’30’’N, 98º57’49’’W, 2250 m, 30 Jul 1987, *J.J. Jiménez-Osornio 106* (F); **Michoacán**: Zitácuaro, San Francisco Coatepec, 19.421ºN, 100.374ºW, 1900 m, cult., 18 Nov 2011, *R. Bye et al. 89* (MEXU); Huetamo, 375 m, 23 Mar 1982, *J.C. Soto Núñez & G. Silva R. 3842* (MEXU); **Querétaro**: Querétaro, 1910, *G. Arsène 10587* (US); **Quintana Roo**: Felipe Carrillo Puerto, Chancah Veracruz, 10 m, huerto familiar, 23 Apr 1983, *E. Gutiérrez 24* (MEXU); same locality, 23 Nov 1984, *E. Gutierrez 388* (MEXU); X-Hazil, 28 Mar 1985, *J. Palma Gutiérrez 85-24* & *85-25* (MEXU); Rancho Las Palmas, ca. 40 km al S de F. Carrillo Puerto, 15 m, 19 Dec 1985, *J. Palma Gutiérrez 85-69* (MEXU); **Sonora**: Guaymas, 1887, *E. Palmer 135* (GH, MEXU) & *136* (UC, MEXU); **Tabasco**: Huimanguillo, Ocuapan, 9 km al oeste de Huimanguillo, rumbo a Mecatepec, Km 2 rumbo a Fco. Rueda, 16 Jan 1979, *C. Cowan 1805* (MEXU);

Yucatán: Pixoy, 20º42’53’’N, 88º15’45’’W, huerto, 22 m, 19 Aug 1986, *E. Ucan Ek 4652* (MEXU).

PANAMA. Panamá: en el Llano de Chepo, 16 Dec 1972, *L. Carrasquilla et al. 281* (F); **Los Santos**: vic. of Río Pedregal, 25 mi. al SW de Tonosí, 2500/3000 ft, 7 Dec 1980, *W.H. Lewis 2925 & 2980* (MO).

PERU. Amazonas: Condorcanqui, Rio Cenepa, Quebrada Huampami, garden, 3 May 1973, *E. Ancuash Atsut 296* (MEXU); Luya, Camporredondo-Anexo Tullanya, 9 Dec 2001, *J. Campos de la Cruz & W. Vargas 6945* & *6947* (USM); Jaipe-Camporredondo, 11 Dec 2001, *J. Campos de la Cruz & W. Vargas 6981* (USM); Bagua, Imaza, Rio Cenepa región, comunidad Yamayakat, 4º55’S, 78º19’, 300 m, Jan 1995, *V. Hodges & J. Gorham 96* (HUT); Rio Cenepa, Quebrada Kayamas, 820 ft, 17 Apr 1973, *R. Kayap 652* (GH, US); Nazareth, camino a Bagua en el Río Marañón, alrededor de la escuela, 13 Mar 1966, *P.G. Smith s.n.* (USM 81628); **Cajamarca**: San Ignacio, alrededores de Namballe, 720 m, 4 Aug 1994, *S. Leiva González et al. 1320* (HAO†, NY); **Cuzco**: Convención, Quempire, caserío Campa, 460-480 m, 24 Jul 1963, *R. Ferreyra 16384* (CORD); **Huánuco**: Leoncio Prado, Tingo María, left bank of Rio Huallaga, 700-800 m, 5 Apr 1976, *T. Plowman 5822* (GH); 3 km north of Tingo María, 700 m, 19 Oct 1938, *H.E. Stork & O.B. Horton 9462 & 9463* (UC, F); **Lambayaque**: Lambayeque, cult. en avenidas, jardines, 28 m, 22 Mar 1994, *S. Llatas Quiroz 3454* (F); **Lima**, La Molina, 300 m, cult., 30 Mar 1954, *O. Velarde Núñez 4*, 29 May 1954, *O. Velarde Núñez 9, 12 & 16* (CORD); **Loreto**: Quebrada Orejón Pucaurquillo, Pevas, 100 m, 29 Oct 1980, *F. Ayala et al. 2792* (F, MO, NY) *& 2793* (NY); Maynas, Rio Yuvineto, affluent du Putumayo, territoire des indiens Secoya, 18 Aug 1978, *G. Haxaire 5143* (NY); Yurimaguas, cult., 14 Sep 1984, *K. Hormia 2194* (USM); Washintsa and vicinity, Río Huasaga, 3º20’S, 76º20’W, 185 m, 16-26 Jun 1986, *W.H. Lewis et al. 11207* (USM); Mariscal Ramón Castilla, Rio Ampiyacu, above Pucu Orquilla 2 km, in Indian garden, 11 Feb 1969, *T. Plowman 2457* & *2458* (GH); Rio Yaguasyacu, affluent of Rio Ampiyacu, Brillo Nuevo and vicinity, 2º40’S, 72º00’W, 16 Apr 1977, *T. Plowman et al. 6902* (GH); Río Ampiyacu, Pebas and vicinity, ca. 3º10’S, 71º49’W, 27 Apr 1977, *T. Plowman et al. 7077* (GH, USM); Brillo Nuevo and vicinity, 1 May 1977, *T. Plowman et al. 7138* (GH); Lower Rio Huallaga, Yurimaguas, 155-210 m, 11 Nov 1929, *L. Williams 5225* (F); Iquitos, 4 Sep 1930, *L. Williams F-8202* (F); **Madre de Dios**: Tambopata, Río Tambopata, comunidad nativa de Infierno, 12º50’S, 69º17’W, 260 m, 19 Feb 1991, *V. Pesha Baca 95* (MO); zona reservada de Tambopata, 12º49’S, 89º18’W, 280 m, 19 Aug 1990, *C. Reynel et al. 5235* (MO); **Pasco**: Palcazu, comunidad nativa Santa Rosa de Palcazú, sector Santo Domingo, 10º26’50’’S, 75º03’25’’W, 470 m, 13 Oct 2008, *M. Haumán et al. 0317* & 320 (USM); **San Martín**: Mariscal Cáceres, Tocache Nuevo, Río Cañuto, Curarelandia, ca. 8º06’S, 76º36’W, 475 m, cult., 19 Dec 1981, *T. Plowman & J. Schunke V. 11497* (USM, MO); same locality, ca. 8º11’S, 76º30’W, 500 m, 24 Dec 1981, *T. Plowman & J. Schunke V. 11695* (CORD, USM); San Martín, Tarapoto, 400 m, 25 Oct 1998, *J. Schunke V. 14518* (NY).

PUERTO RICO. Bayamón: Prope Bayamón, 22 Mar 1885, *P. Sintenis 866* b (GH).

SURINAM. Kwamala, 2º20’N, 55º47’W, Indian garden, Jan 1983, *M.J. Plotkin 139* (US).

VENEZUELA. Bolívar: Kon-quén, along Río Aparurén, 480 m, 7 Jul 1953, *J. A. Steyermark 76028* & *76029* (F, NY).

Cultivated
ARGENTINA. Buenos Aires: San Isidro, cult. en Jardín de San Isidro, Feb 1943, *A.T. Hunziker s.n.* (CORD 00032052); **Córdoba**. Capital, cult. en invernáculo del IMBIV (ciudad Universitaria), semillas de Lima (Perú), 3 Jul 2008, *G.E. Barboza 1901* (CORD); cult. ciudad de Córdoba, 25 Mar 1995, *A.T. Hunziker 25502* (CORD); **Salta.** Cerrillos, cultivado en invernáculo de Estación Experimental INTA, 28 Mar 1995, *A. T. Hunziker 25490* (CORD).

BRAZIL. Amazonas: Manaus, cult. Jardín Botánico de Ducke, 21 Sep 2010, *G.E. Barboza 2504, 2505* & *2506* (CORD); **Goiás**: Jatai, UFG-Campus Jatobá (casa de vegetação), 1 Jul 1970, *E.V.E. Amaral 139* (CEN); **Rio de Janeiro**: Rio de Janeiro, cult. no Horto do Jardim Botânico do Rio de Janeiro, 28 Mar 1990, *L. D'A. Freire de Carvalho s.n.* (RB 285505).

UNITED STATES OF AMERICA. Indiana: cult. in IU greenhouse from seed collected in Brazil, Bahia, Itamotingo, Rio São Francisco between Juazeiro and Curaça, 23 Jul 1974, *B. Pickersgill RU72-99* (UC); cult. IU greenhouse from seed collected in Brazil, Piauí, Colonia, 33 km S of Oeiras, 22 Jul 1974, *B. Pickersgill RU72-175* (UC); cult. at Indiana University greenhouse, seeds from Perú, Reque (leg. C. Rick, 1958), *P. Smith AC 1905* (US).

12. Capsicum coccineum (Rusby) Hunz.
BOLIVIA. Bolivien, 600 m, 13-20 Apr 1892, *O. Kuntze s.n.* (NY 00138595, NY 00138596); **Beni**: Ballivián, Gegend von Reyes, Rurrenebaque am Río Beni, Oct-Dec 1930, *E. Fleischmann 143* (S); Estación Biológica del Beni, Charaton, barbecho de Benjamín Cari, 14º30'S, 66º37'W, 200 m, 26 May 1995, *I. Guareco 457* (LPB, NY); Estación Biológica del Beni, ca. 25 km noreste del San Borja por el Río Manique, 14º35'S, 66º35' W, 250 m, 23 Aug 1991, *T. Killeen et al. 3415* (BOLV, NY); **Cochabamba**: Carrasco, Sehuencas, proximo al río y el puente de madera, 416 m, 9 Oct 2006, *S. Altamirano et al. 3711* (BOLV, MO); Chapare, Territorio Indígena Parque Nacional Isiboro-Secure, community of San Antonio, 16º24'S, 65º54'w, 240 m, 19 Nov 2004, *E. Thomas & R. Berdeja 1428* (BOLV, LPB); Carrasco, 12 de Julio, ca. 9 km S of Israel on E bank of Rio Sajta, 400 m, 24 Jul 1999, *J.R.I. Wood 14892* (LPB); **La Paz**: Woods of Macharia, 5/6000 ft, Dec 1864, *R. Pearce s.n.* (K); Colonia Tupiza, 615 m, 15 Dec 1994, *R. Seidel & D. Vaquiata 7689* (CORD, LPB, NY); Larecaja, NE de la Comunidad Muchanes, 250 m, 28 Jan 1995, *L. Vargas et al.1310* (LPB); Prov. Abel Iturralde, San Buena Ventura, 1400 ft., 30 Nov 1901, *R.S. Williams 623* (BM, CORD, K, MO, NY, US); Pilon Lajas Park, logging road from Campamento Bella Vista near La Cascada, 600 m, 4 Aug 1998, *J.R.I. Wood & D. Wasshausen 13893* (LPB); **Santa Cruz**: La Chonta, a 1 km antes de llegar al poblado La Chonta, 440 m, 2 May 2006, *G.E. Barboza et al. 1916* (CORD); a unos 1.5 km antes de llegar al poblado de La Chonta, 17º39'03''S, 63º41'13''W, 402 m, 14 Dec 2017, *G.E. Barboza et al. 4921* (CORD, LPB, MO); trail from Río Yapacaní into Campamento Mataracú of PN Amboró, 1-2 km N of Campamento, 17º32'S, 63º52'W, 350 m, 31 May 1998, *L. Bohs & M. Nee 2754* (USZ); Río Surutú, 340 m, 25 Jan 1957, *T. Meyer 19927*

(LIL); Parque Nacional Amboró (PNA), slopes along Río Saguayo, 1 km NE of entrance into first Andean foothills, 17º39'S, 63º43'W, 400 m, 18 Jan 1988, *M. Nee & M. Saldías P. 35956* (CORD, LPB, MO, NY, USZ); PNA, trail along Rio Cheyo and Rio Agua Blanca, 4-10 km SW of Huaytu, 17º36'S, 63º39' W, 400 m, 22 Jan 1988, *M. Nee 36049* (LPB, MO, NY, USZ); ca. 3-4 km S of San Rafael and 0.5 km N of San Salvador, 11 km (by air) SW of Villa German Bush, ca. 17º29', 63º56'W, 600-650 m, 19 Nov 1988, *M. Nee & M. Saldías P. 36874* (CORD, LPB, MO, NY, USZ); PNA, slopes along Río Saguayo, 1 km NE of entrance into first Andean foothills, 17º39'S, 63º43'W, 400 m, 18 Dec 1988, *M. Nee 37233* (CORD, MO, NY, USZ); along Rio Saguayo, 1.5-3 km NE of entrance into first Andean foothills, 17º38' to 39'S, 63º43'W, 375 m, 21 Dec 1988, *M. Nee 37305* (CORD, G, LPB, MO, NY, US, USZ); PNA, along Rio Saguayo, 0-2 km upstream from the Quebrada Yapoje, 17°34'S, 63°44'W, 350 m, 14 Dec 1989, *M. Nee 38180* (LPB, MO, NY, USZ); trail parallel to Rio Cheyo, 7 km SW of Terminal de Huaytu, 17°38'S, 63°40'W, 375 m, 23 Sep 1990, *M. Nee 38899* (LPB, MO, NY, USZ); PNA, 5 km SSW of Buena Vista, W side of Rio Sururtu, 17°29'30"S, 63°40'30"W, 320 m, 20 Oct 1990, *M. Nee 39360* (LPB, MO, NY); PNA, Río Saguayo near mouth of Quebrada Yapojé, 17º34'S, 63º44'W, 350 m, 11 Jun 1991, *M. Nee 40886* (F, LPB, MO, NY, USZ*);* intersección de los Ríos Surutú y Pitisama, 17º39'5''N, 63º25'W, 400 m, 19 Sep 1987, *M. Saldías P. 110* (MEXU, USZ); Sara, monte alto del Río Guendá, 440 m, 22 Aug 1916, *J. Steinbach 2625* (CORD, K, LIL, SI, UC); submonte del Río Surutú, 400 m, 26 Oct 1916, *J. Steinbach 3038* (LIL, SI) & *3044* (SI); raluras en el bosque del Río Surutú, 500 m, 28 Sep 1917, *J. Steinbach 3475* (CORD, GH, K, LIL, NY); Buena Vista, raluras del bosque Surutú, 400 m, 1-26 Apr 1921, *J. Steinbach 5550* (A, CORD, F, G, GH, MO, US); 1-2 km below Campamento Chonta, 500 m, 4 Oct 1997, *J.R.I. Wood & M. Menacho 12642* (LPB); Vallegrande, Pucará, al fondo del valle del Río Gande, ca. 2 km pasando el puente, camino hacia Pucará, 18º42'01''S, 64º15'49''W, 18 Feb 2007, *J.R.I. Wood et al. 22801* (USZ).

PERU. Ayacucho: Río Apurimac Valley, near Kimpitiriki, 400 m, 10/11 May 1929, *E.P. Killip & A.C. Smith 23023* (CORD, US); **Cuzco**: Quispicanchi, hills around Río Araza between Pan de Azucar and Quince Mil Airport, 13º13'S, 70º45'W, 643 m, 10 Aug 1991, *P. Núñez V. 14114* (MO, USM); Camanti, Maniri, camino desde represa Yanamayo en dirección al Cerro Camanti, 13º71'S, 70º48'W, 720 m, 25 Aug 1990, *M. Timaná 758* (MO); Cerro Camanti, vertiente del Yanamayo, 13º71'S, 70º45'W, 720 m, 1 Sep 1990, *M. Timaná 818* (MO, USM); entre la represa del Río Yanamayo a lo largo del camino paralelo a la quebrada Toquimayo, 13º17'S, 70º48'W, 720 m, 15 Oct 1990, *M. Timaná 1007* (MO, USM); **Huánuco**: Tingo María, 24 Jul 1940, *E. Asplund 1*2491 (CORD fragment ex S, S); same locality, 10 Aug 1940, *E. Asplund 12940* (CORD fragment ex S, S); Tingo María, Valle of Río Huallaga, ca. 7000 ft, 11-14 Jul 1937, *C.M. Belshaw 3084* (CORD, GH, K, NY, UC, US); Tingo María, Río Huallaga, 4 Oct 1972, *T.B. Croat 21043* (MO); Centro Ganadero Tournavista, Nov 1974, *F. Encarnación 750* (CORD, USM); cerca de Tingo María, km 138, carretera Huánuco-Pucallpa, 600-700 m, 11 Aug 1946, *R. Ferreyra 922* (CORD, LE, M, US, USM); alrededores de Tingo María, 600-650 m, 10 Jul 1958, *R. Ferreyra 13163* (USM); Pachitea, Sungaro, along highway construction 10 km S of Sungaro River crossing, W of Puerto Inca, 9º22'S, 75º00'W, 250-300 m, 14 Sep 1982, *R.B. Foster 8802* (F, MO, USM); Tingo María, in woods at edge of chacra, 14 Jan 1981, *C.B. Heiser 7802* (CORD); Tingo María, Oct 1977, *Johns et al. 142* (MOL); Tingo María, left bank of Rio Huallaga, 780-800 m, 5 Apr 1978, *T. Plowman 5821* (F, GH, USM); in low

forest at Aucayacu, 589 m, 6 Sep 1965, *J. Schunke V. 821* (COL, CORD, F, G, NY, US); Rupa Rupa, al E de Tingo María, 680 m, 29 Nov 1971, *J. Schunke V. 5199* (CORD, F, G); Quebrada de Aucayacu, 590 m, 14 Aug 1964, *J. Schunke V. 6554* (CORD, F, MO, US, USM); José Crespo y Castillo, Quebrada de Sangapilla, cerca a Aucayacu, 600 m, 4 Mar 1978, *J. Schunke V. 9984* (CORD fragment, MO); Rupa Rupa, al noreste de Tingo María, cerca a Mapresa, 672 m, 2-4 Oct 1978, *J. Schunke V. 10620* (MO); Cucharas, 500 m, 10 Mar 1954, *F. Woytkowski 1176* (CORD, USM); Pendencia, 900 m, 28 Sep 1962, *F. Woytkowski 7516* (GH, MO, UC, US); **Junín**: Puerto Yessup, 400 m, 10-12 Jul 1929, *E.P. Killip & A.C. Smith 26351* & *26386* (F, NY, US); **Loreto**: Contamana, trail to Aguas Calientes, 160-200 m, 27 Jul 1970, *F. Mc Daniel & L. Santiago 2556* (F, US); Contamana, trail to Aguas Calientes, 160-200 m, 27 Jul 1970, *S. Mc Daniel 14075* (F, US); **Madre de Dios**: Manu, Cocha Cashu Biological Station, 11º52'S, 71º22'W, 400 m, 24 Sep 1982, *L. Emmons 86* (MO); Rio Manu, playa 16 above the Boca, 15 Aug 1976, *R. Foster & C. Augspurger 3164* (US); Cocha Cashu, forest in vicinity ox-bow lake of Rio Manu, between Panagua & Tayakome, 17-24 Aug 1974, *R.B. Foster et al. 3484* (US); Rio Manu, Cocha Cashu Station, 20 Sept 1976, *R.B. Foster & J. Terborgh 5043* (USM); same locality, 350 m, 31 Jul 1979, *R.B. Foster 6849* (F, MO); Parque Nacional Manu (PNM), Rio Sotileja, 11º40'S, 71º55'W, 400-500 m, 4 Oct 1986, *R.B. Foster & B. d'Achille 11630* (USM); PNM, Rio Cumerjali, 11º49'S, 71º32'W, 350-400 m, 24 Oct 1986, *R.B. Foster & B. d'Achille 12017* (F, INPA, LPB, MO, NY, USM); PNM, alrededores de la estación Cocha Cashu, 11º53'S, 71º24'W, 350 m, 24 Dec 1985, *R. Kalliola et al. P3-051* (USM); Manu Park, Cocha Cashu uplands, 11º45'S, 71º0'W, 400 m, 22 Sep 1986, *P. Núñez V. 6203* (MO, USM); alrededores del Albergue turístico Cusco Amazónico, margen izquierdo del Río Madre de Dios, ca 35 km de Puerto Maldonado, 200 m, 29 Oct 1988, *P. Núñez V. et al. 10024* (GH, MO); Tambopata, Cuzco Amazónico, 12º05'S, 69º03'W, 200 m, 1 Jun 1989, *O. Phillips & P. Núñez 163* (MO, USM); Las Piedras, Cuzco Amazónico, en el borde del Río Madre de Dios, 200 m, 23 Jul 1991, *M. Timaná 1930* (MO); Las Piedras, en bosque cerca quebrada Madama, 12º29'S, 69º03'W, 200 m, 30 Oct 1991, *M. Timaná & N. Jaramillo 2878* (MO); Las Piedras, Cuzco Amazónico, 12º29'S, 69º03'W, 200 m, 16 Nov 1991, *M. Timaná & N. Jaramillo 3224* (MO); same locality , 6 Dec 1991, *M. Timaná 3670 & 3673* (MO); Puerto Maldonado, 13º08'S, 69º36'W, 270-300 m, 24 Nov 1013, *L. Valenzuela & I. Huamantupa 1013* (MO); **San Martín**: Mariscal Cáceres, 69 km NE of Tingo María on road to Tocache, Huallaga Valley, 550 m, 16 Jul 1982, *A.H. Gentry et al. 37623* (MO, USM); Juan Jui, Alto Río Huallaga, 400-800 m, Feb 1936, *G. Klug 4248* (BM, CORD, F, GH, K, MO, U, UC, US, USM, S); Río Cañuto, "Curarelandia", propiedad of J. Schunke V., near km 23 along road from Tocache Nuevo to Puerto Pizana, 8º06'S, 76º36'W, 475 m, 19 Dec 1981, *T. Plowman & J. Schunke V. 11523* (CORD, F, GH, MO, NY, P, US, USM); Maynas Alto, Tocache, Jun 1830, *E.F. Poeppig 1799* (CORD, GH, W); Tocache, Jun 1830, *E.F. Poeppig 1845* (G); Fundo La Campiña, 2 km abajo de Tocache Nuevo, margen derecha del Río Huallaga, 400 m, 10 Sep 1969, *J. Schunke V. 3415* (COL, CORD, F, G, NY, US); Puerto Pizana (margen derecho del Río Huallaga), 350-380 m, 26 Jul 1973, *J. Schunke V. 6442* (MO, USM); Tocache Nuevo, Quebrada de Challuayacu, 480-500 m, 2 Feb 1979, *J. Schunke V. 10777* (MO); Tocache Nuevo, Quebrada Cachiyacu de Huaquisha, 500-650 m, 13 Dec 1980, *J. Schunke V. 12469* (FSU, IBE, MISS, MO, UNA); **Ucayali**: Bosque Nacional A. von Humboldt, Pucallpa -Tingo María Road, Quebrada Capirona, 08°40'S, 75°00'W, 270 m, 30 Jan 1978, *C. Froehner 169* (MO); Iparia, cuenca del Río Iparia, afluente del Rio Ucayali, cerca de la comunidade nativa Ashaninka de

Miraflores, 9º21’11’’S, 74º28’50’’W, 200 m, 8 Sep 2007, *J.G. Graham & J. Schunke V. 4633* (NY); desembocadura de la quebrada de Huacamayo, norte de Aguayti, cerca al Fundo Julio Cesar, 9º2’S, 75º30’W, 250 m, 11 Oct 2004, *J. Schuncke V. & J.G. Graham 16612* (F); Fundo ganadero L. & G., 8 km abajo del Puerto Esperanza, margen derecho del Río Purús, 10°12'S, 70°57'W, 150-190 m, 12 Mar 2002, *J. Schunke V. & J.G. Graham 15004* (MO, NY, USM); Cordillera Azul, km 15 on Tingo María-Pucallpa road, 1 km on dirt road that begins at Puente Pumahuasi, 700 m, 5 Jun 1981, *K. Young & G. Sullivan 713* (MO).

BRAZIL. **Acre**: Tarauacá, basin of Rio Juruá, Rio Tarauacá, Reserva Indígena Praia do Carapaná, Seringal Universo, 8º26’58’’S, 71º20’57’’W, 21 Nov 1995, *D.C. Daly et al. 8711* (MO, NY); Santa Rosa, Rio Purus, Seringal Santa Helena, 09°07'49"S, 70°10'37"W, 23 Mar 1999, *D.C. Daly et al. 9979* (MO, NY); Seringal Universo, Colocação Maceió, 8º15’20’’S, 70º59’10’’W, 16 Jun 1995, *C. Figueiredo et al. 892* (MO, NY); Feijó, Rio Muru, Seringal Lancha, 8º31’5’’S, 70º51’57’’W, 30 Nov 1995, *A.R.S. Oliveira et al. 675* (MO, NY); Sena Madureira, Bacia do Rio Purus, Faz. Nova Olinda, Carreador do Poti, ca. 8 km da sede, 10º07’S, 69º13’W, 21 Oct 1993, *M. Silveira et al. 551* (CAS, CTES, INPA, MEXU, MO, NY); Bacia do Rio Purus, Faz. Nova Olinda, margem direta do Rio Iaco, Carreador do São Bento I, ca. 15 km da sede, 10º06’S, 69º12’W, 26 Oct 1993, *M. Silveira et al. 650* (INPA, NY); bacia do Alto Juruá, Rio Tarauacá, Reserva Indígena Praia do Carapanã, Colocação Vista Alegre, 8º26’57’’S, 71º20’57’’W, 21 Nov 1995, *M. Silveira 1080* (INPA, MO, NY); Assis Brasil, Río Acre, Seringal Auristella, Sep 1911, *E. Ule 9732* (CORD, G, K, L, U, US); **Amazonas**: Boca do Acre, Río Acre, Seringal São Francisco, -9.08333, -67.4, Apr 1911, *E. Ule 9738* (CORD, K, G, US); **Rondonia**: Ariquemes, ca. 6 km from crossing of DER-RO, TB-65, BR-364, 2 Jun 1984, *D. Frame et al. 217* (INPA).

Cultivated
UNITED STATES OF AMERICA. California: Yolo Co., University of California, Department of Vegetable Crops greenhouse, seeds from near village of Quincemil, Cuzco, Peru, 1959, *P.G. Smith SA 393* (CORD, DAV).

13. Capsicum cornutum (Hiern) Hunz.
BRAZIL. **Rio de Janeiro**: Paraty, Corisção, APA-Cairuçu, 600 m, 25 Nov 1994, *M.G. Bovini & L.C.S. Giordano 363* (CEN, CORD, RB); Itatiaia, Tres Picos, 1933, *A.C. Brade 12668* (RB); Taquaral, 22 Feb 1936, *A.C. Brade 15095* (R, RB); Parque Nacional do Itatiaia (PNI), camino para o Véu de Noiva, 24 Mar 1972, *P.I.S. Braga 2441* (CORD, RB); PNI, trilha para Cachoeira Itaporani, 22°25’36’’S, 44°37’14’’W, 1152 m, 23 May 2017, *Y.F. Gouvêa 215* (BHCB); Caixa d’Agua (Alto), 4 Oct 1932, *J.G. Kuhlmann s.n.* (CORD 00086172, R 29010; RB 53641); Engenheiro Paulo de Frontin, Morro Azul, acesso esquerdo à fazenda do Pau Ferro, Sítio do coronel Mário, 26 Jan 1998, *L.J.S. Pinto et al. 92* (CORD, RB); **São Paulo**: Santo André, Subprefeitura de Paranapiacaba e Parque Andreense, parque América, trilha travessa Marechal Rondon, 23º46'34,41"S, 46º24'37,24"W, 13 Sep 2007, *R.J. Almeida-Scabbia et al. 5113* (SP); Reserva Biológica (RB) do Alto da Serra de Paranapiacaba, en la trilha 17 próximo a trilha 4, 800 m, 24 Sep 2010, *G.E. Barboza & M.T. Cosa 2517* (CORD); Paranapiacaba, por el camino de tierra rumbo al poblado de Paranapiacaba, 23º46’08’’S, 46º18’33’’W, 775 m, 25 Sep 2010, *G.E.*

Barboza & M.T. Cosa 2525 (CORD); Estrada Velha (Camino do Mar) São Paulo-Santos, a 5.7/6 km do complexo da Petrobras, 23º51'S, 46º27'W, 510 m, 16 Apr 1986, *L. Bianchetti et al. 491* (CEN, CORD); Estrada Velha (camino do Mar) São Paulo-Santos, a 6 km do complexo da Petrobras, 23º51'S, 46º27'W, 570 m, 16 Apr 1986, *L. Bianchetti et al. 492 & 493* (CEN, CORD); Estrada Velha (Camino do Mar) São Paulo-Santos, 27 May 1992, *L. Bianchetti et al. 1333* (CEN); arredores da Estação Biológica de Boracéia, a 1,4 km da guarita 1 da SABESP, sentido Salesópolis, 23°38'02''S, 45°57'35''W, 820 m, 7 May 1999, *L. Bianchetti et al. 1542* (CEN); in the upper part of the ascent up to the Serra de Cubataõ, summit do Pico da Serra, 22 Dec 1826, *W.J. Burchell 3705* (K, P); RB do Alta da Serra de Paranapiacaba, Estação Experimental, 26 Oct 1982, *S.A.C. Chiea 223* (SP); Estação Biológica (EB), Paranapiacaba, 23°47'S, 46°19'W, 750-900 m, 20 Nov 1981, *A. Custódio Filho et al. 678* (SP); same locality, 26-27 Oct 1982, *A. Custódio Filho et al. 1004* (SP); same locality, 7 Dec 1982, *A. Custódio Filho et al. 1144* (SP); Estação Biológica (EB), Paranapiacaba, trilha 1, ca. 50 m do mirante, 23°46'58''S, 46°19'12''W, 856 m, 23 Jan 2014, *B.F. Falcao et al. 10* (BHCB); Marsilac, Camping Ana Paula Capivari-Monos, 17 Jan 1996, *R.J.F. García et al. 726* (BHCB, PMSP, SP); RB do Alta da Serra de Paranapiacaba, margen da estrada que leva da entrada à Casa do pesquisador, 838 m, 19 Nov 2013, *L. Giacomin et al. 2018* (BHCB); Parelheiros, Capivari, Fazenda da SABESP ao final da Estrada do Capivara, trilha á dereita da Casa da Guarda, 23°58'08"S, 46°40'49"W, 13 Feb 1995, *S.A.P. Godoy et al. 352* (SP); Paranapiacaba, mata da Estação Biológica, 17 Nov 1955, *O. Handro* 552 (CORD; SP); Alto da Serra, Estação Biológica, 2 Nov 1922, *F.C. Hoehne s.n.* (SP 7980); Serra do Mar, EB de Paranapiacaba, ca. 850 m, 1 Dec 1967, *A.T. Hunziker 19557* (CORD, NY); Estrada Velha (camino do Mar), a 6 km do complex de Petrobras, 570 m, 16 Apr 1986, *A.T. Hunziker 25197* & *25198* (CORD) & *25199* (BM, CORD, NY); EB da Serra de Paranapiacaba, 5 May 1982, *M. Kirizawa et al. 771* (CORD, SP); same locality, picada 1, 1 Nov 1988, *M. Kirizawa 2087* (SP); same locality, 5 Nov 1991, *M. Kirizawa et al. 2551* (SP); Piassaguera, 2 Oct 1922, *J.G. Kulhmann s.n.* (RB 00721997); Alto da Serra, Estação Biológica, 31 Dec 1942, *M. Kuhlmann s.n.* (CORD 00086174; SP 43590) & 8 Nov 1943, *M. Kuhlmann s.n.* (CORD 00086171, SP 49475, SPF 67423); Paranapiacaba, Estação Biológica, 30 Jan 1958, *M. Kuhlmann 4321* (CORD, SP); Alto da Serra, Estação Biológica, 2 Oct 1931, *C. Lemos s.n.* (CORD 00086170 & 00086175, SP 28314, US1617120); Paranapiacaba, via férrea São Paulo-Santos, 28 Oct 1965, *J.R. Mattos 12778* (CORD, SP); Parque Estadual da Serra do Mar, Núcleo Curucutu, trilha da cachoeira do Banquinho, 23º59'07"S, 46º44'35"W, 29 Oct 1999, *M.A. Mayworm et al. 135* (SPF); Santos, about 42 km from São Paulo, on road to Santos, Apr 1972, *B. Pickersgill 463* (CORD); Núcleo Curucutu, trilha para o rio Embú-Guaçu, 2 Dec 1998, *L. Rossi et al. 2037* (SP); between Santos and São Paulo, May 1942, *C. Sandeman 2038* (K); Paranapiacaba, Estação Biológica, 750-900 m, 28 Jan 1983, *M. Sugiyama et al. 230* (SP); São Bernardo do Campo, Parque Caminhos do Mar, Calçada do Lorena, Setor A, 12 Nov 1992, *M. Sugiyama & S.A. Chiea 1094* (SP); trilha construida pela CESP, estrada da Torre, camino para o Vale do Quilombo, próximo à Vila de Paranapiacaba, 31 Jan 1996, *M. Sugiyama et al. 1399* (BHCB, SP, SPF).

14. Capsicum dimorphum (Miers) Kuntze

COLOMBIA. Without locality, 1760-1808, *J.C. Mutis 2010* (US); **Antioquía**: Santa Elena, 1500-1700 m, 28 Dec 1930, *W.A. Archer 1170* (CORD, GH, MEDEL, US) & 1 Jan 1931,

W.A. Archer 1273 (GH, US); cerca de Santa Helena, camino entre Medellín y Río Negro, 2500 m, 2 Oct 1947, *F.A. Barkley et al. 6* (COL, US) & *F.A. Barkley et al. 10* (MEDEL); La Ceja, 2400-2500 m, 21 Aug 1948, *F.A. Barkley 262* (MEDEL); Urrao, Carretera Urrao-Caicedo, 6º28'N, 76º10'W, 3151 m, 5 Dec 1986, *R. Callejas 3151* (HUA, MO, NY); Sonsón, Vía Sonsón-La Soledad, 1.1 km de la Troncal Principal, Vereda Manzanares, 2800 m, 8 Apr 1988, *R. Callejas et al. 6373* (NY); San José de la Montaña, carretera 13 km de la cabecera en la vía al municipio de Toledo, 6º52'00"N, 75º43'00"W, 2550 m, 11 Nov 2003, *F.A.Cardona & H. David 1224* (HUA, NY); Envigado, Vereda El Escobedero, margen izquierdo frente a la "Morena", 2000-2150 m, 18 Jul 1996, *M.A. Correa Múnera 858* (JAUM); Vereda El Escobero, costado occidental Cerro San Luis, nacimiento de la Quebrada La Cachona, 6º8'11"N, 75º33'0"W, 2350-2450 m, 16 Aug 1996, *M.D. Correa A. 1124* (COL); Entrerríos, Vereda El Zancudo, 2300 m, 1 Sep 1998, *Estudiantes herbario MEDEL 158* (MEDEL); Caldas, Vereda La Clara, 6º02'50"N, 75º37'10"W, 1800-1900 m, 4 Mar 2005, *Estudiantes herbario MEDEL 630* (MEDEL); La Ceja, Jul 1934, *Hno. Daniel 397* (COL, US); Laguna de Guarne, 27 Sep 1935, *Hno. Daniel 1464* (MEDEL, US); Páramo de Sonsón, 2700-2850 m, 26 Jan 1945, *Hno. Daniel 3429* (COL, CORD, MEDEL, US); Medellín, Carretera Santa Elena, km 14, 2500 m, 14 Mar 1989, *R. Fonnegra G. 2672* (COL, HUA, MO, NY); Parque Ecológico Piedras Blacas, 6º18'N, 75º29' W, 2400 m, 13 Aug 1994, *R. Fonnegra G. & F.J. Roldán 4952* (COL, NY); Medellín, Parque Ecológico Piedras Blancas, sector Lajas, 75º29'W, 6º18'N, 2350 m, 10 Dec 1994, *R. Fonnegra G. et al. 5315* (COL, HUA, MO, NY); Cocorná, Vereda El Viaho, lado oriental torre 105, 2200 m, 29 Oct 1999, *A. Gil et al. 310* (JAUM); Corregimiento de Santa Elena, vereda el Cerro, cabeceras de la Q. Santa Elena, finca de Pineda, 2700 m, 5 Mar 1996, *L.F. Giraldo et al. 667* (JAUM); Medellín, vereda Santa Elena Central, Quebrada La Avícola, 2500 m, 17 Jul 1996, *L.F. Giraldo et al. 900* (JAUM); Corregimiento Altavista, monte El Encanto, quebrada El Barcino, 6º13'44"N, 75º39'33"W, 2350-2380 m, 16 Jun 1997, *L.F. Giraldo et al. 1631* (JAUM); bosque de La Sierra, 2800 m, 18 Sep 1947, *R. González M. et al. 13* (COL, MEDEL, US); along rd from Medellín to Río Negro, 2500 m, 17 Nov 1948, *E.P. Killip et al. 39889* (US); Morrogol, S of Medellín, 6º15'N, 75º32'W, ca. 2400 m, 23 Oct 1983, *A. Juncosa & G. Misas 1071* (JAUM, MO); Carolina del Príncipe, Represa Miraflores, 26 Nov 1980, *G. Lozano C. 3270* (COL); Carmen del Viboral, Vereda La Milagrosa, vía El Canada, Finca la Soledad, 2400-2600 m, 27 Oct 1987, *J.L. Luteyn & R. Callejas 11796* (NY); Jardín, road between Jardín and Río Sucio, ca. 9 km from Jardín, 05°33'N, 75°49'W, 2300-2400 m, 29 Jan 1989, *J. MacDougal & F.J. Roldán 3557* (MO); Carretera Sonsón-Los Medios, 05º41'N, 75º21'W, 2420 m, 8 Jul 1987, *O. Marulanda 395* (COL); Medellín, Alto de Boquerón, margen derecha de la via Medellín-San Jerónimo, 06º18'N, 75º40'W, 2600-2650 m, 23 Oct 2000, *J.C. Marrugo G. & J.M. Vélez P. 55 & 265* (MEDEL); Fusagasugá, La Aguadita, Finca La Carbonera, 4º24'37,7"N, 74º18'40,9"W, 2300-2400 m, 26-31 May 2009, *H. Mendoza 17394* (UDBC); Amalfí, vereda Guayabito, 6 Jul 2005, *J.A. Navarro et al. 288* (COL, UDBC); Medellín, Corregimiento San Antonio de Prado, vereda El Astillero, La Mayordomía, Piedra La Galana, 6º15'N, 75º39'30"W, 2330-2630 m, 19 Jul 2011, *P. Pedraza-Peñalosa et al. 2201* (NY); Corregimiento Altavista, Vereda Aguas Frías, cabeceras quebrada La Picacha, 6º13'57"N, 75º39'45"W, 2380-2415 m, 23 Apr 1997, *W. Rodríguez et al. 589* (JAUM); Parque Ecológico Piedras Blancas, Parajes La Soledad y Piedras Blancas, 6º18'N, 75º29'W, 2350 m, 18 Jun 1995, *F.J. Roldán et al. 2332* (HUA, MO, NY) *& 2350* (NY); Caramanta, 2000 m, Sep 2012, *C. Sánchez 981* (MEDEL); Corregimiento Nutibara, Frontino, cuenca alta del Rio Cuevas, 1800-2000 m,

18 Mar 1984, *D. Sánchez S. et al. 125* (MEDEL; NY); Urrao, Carretera a Caicedo, 2000-2900 m, 12 Jun 1991, *D. Sánchez et al. 1457* (MEDEL); Bolívar, Sitio La Mansa, 1975 m, 15 Nov 1996, *D. Sánchez et al. 3176* (MEDEL); Envigado, Quebrada la Carriqui, 6º10'N, 75º34'W, 2250 m, 4 Nov 2004, *L.F. Vera-Sánchez 987* (MEDEL); ca. 5 km WSW of Yarumal, just W of El Peñol, 6º57'N, 75º28'W, 2420 m, 30 Nov 1986, *J. Zarucchi & D. Cárdenas L. 4366* (CORD, COL, MO); **Boyacá**: Duitama, Corregimiento El Carmen, carretera El Carmen-Virolin, 2300-2500 m, *J. Betancur et al. 6187* (COL, FMB); Santa María, Cordillera Oriental, vereda Caño Negro, de Santa Maria hacia Cuchilla Negra, 4º51'27"N, 73º16'60"W, 1400-1900 m, 3 Nov 2004, *C. Granados-Tochoy et al. 411* (COL); Quebrada El Pato, El Cidro, Río Pomera (Cordillera Oriental), 11 km SE of Moniquirá, 2355 m, 20 Aug 1944, *H. St. John 20655* (C, NY, S, UC, US); **Caldas**: Florencia, de Rancho Quemado por vía a Pensilvania, 05º23'13"N, 75º09'48"W, 1800-200 m, 27 Sep 1993, *C. Barbosa 14924* (FMB); Pinares, above Salento (Cordillera Central), 2400-2700 m, 2-10 Aug 1922, *F.W. Pennell 9318* (NY); Manizales, Reserva Río Blanco, debajo de las Palomas, 2590 m, 5 Feb 2005, *D. Sanín 1048* (JAUM); **Caquetá**: Quebrada del Río Hacha, debajo de Gabinete, 2100-2250 m, 23 Mar 1940, *J. Cuatrecasas 8572* (COL, F); límite entre los Dptos. Caquetá y Huila, 1º52'49"N, 75º40'55,2"W, 2385 m, 17 Apr 2016, *A. Orejuela R. et al. 2685* (COL, CORD); **Cauca**: Munchique, 2500 m, 21 Apr 1939, *A.H.G. Alston 8157* (BM, COL, F, S, US); La Cumbre, near Pico de Aguila, W of Morales, 2750 m, 27 Aug 1944, *E.L. Core 1105* (COL, F, US); El Tambo, camino a Santa Ana, desde la cabaña del Parque Nacional Natural Munchique hasta la estación de policía, 2850-3046 m, 16 Aug 2011, *C.I. Orozco et al. 3838* (COL, CORD); El Tambo, PN Munchique, por la carretera que conduce al Km 51, vía a la Gallera, sector de El Sopladero, 2130-2347 m, 17 Aug 2011, *C.I. Orozco et al. 3863* (COL, CORD); El Tambo, por el sendero de Las Tangaras, hacia el Mirador, 2130-2347 m, 18 Aug 2011, *C.I. Orozco et al. 3865* (COL, CORD); **Cundinamarca**: Albán, Finca del Padre Luna, 2300 m, 10 Feb 2010, *G. Beltrán et al. 140* (COL); Albán, Vereda Los Alpes, Río Dulce, Fundación Granjas Infantiles del Padre Luna, 4º53'35"N, 74º25'17"W, 2140 m, 30 Mar 1998, *S. Castro & C. Bernal 32* (COL); between Fusagasuga and Sibate, at Rio Blanca near La Guaytla, 9 km N of Fusagasuga, 24 km S of Sibate, 04°23'N, 74°20'W, 2000 m, 23 Mar 1983, *T.B. Croat 55459* (MO); San Miguel a Aguabonita, 2200-2800 m, 20-25 Apr 1946, *J.M. Duque Jaramillo 3330* (COL); Soacha, Santandercito, Parque de Chicaque, 2200-2600 m, *J.L. Fernández-Alonso et al. 23798* (COL); Facatativá a Sasaima, Río Gualivá, 2300-2900 m, 1 Sep 1954, *H. García Barriga 15258* (COL); Sasaima, Vereda San Bernardo, 1700-1900 m, 3 Nov 2007, *H. García Barriga 21269* (COL); Tena, Laguna de Pedro Palo, 2060 m, 10 Jun 1967, R. *Jaramillo et al. 2698* (B, COL, CORD, Q); bei Cipacan, 2700 m, Jan 1883, *F.C. Lehmann 2603* (K, US); Fundación Granjas Infantiles del Padre Luna, granja El Gran Ciudadano, 4º53'58"N, 74º25'41"W, 2000 m, 26 Feb 2001, *S. Obando et al. 46* (COL); Albán, Vereda Las Marías, Granja "El Buen Ciudadano", 2000 m, 24 Apr 2000, *C.I. Orozco et al. 3371* (COL); Soacha, Parque Chicaque, 2000-2600 m, 1 Apr 2001, *C.I. Orozco et al. 3675* (COL, FMB); Bojacá, Vereda Roble Hueco, Finca Honduras Cucharal, 2030-2230 m, 17 Feb 2004, *C.I. Orozco et al. 3717* (COL, UDBC); Junín, Reserva Biologica Carpanta, 4º35'5"N, 73º39'54"W, 2620 m, 21 Dec 1988, *L. Pavajeau 60* (COL); Junín, Reserva Biologica Carpanta, 19 Jun 1989, *L. Pavajeau 174* (COL); Granja El Gran Ciudadano, 4º53'35"N, 74º25'17"W, 2140 m, 30 Mar 1998, *P. Pedraza-Peñalosa et al. 59* (COL); San Francisco, Vereda Sabaneta, 2450 m, 21 Feb 1990, *R. Sánchez & E. Linares 1479* (COL); Albán, 6.7 km de Albán por la carretera a Sasaima, Granja del Gran

Ciudadano, 1850-2100 m, 5 Oct 1998, *K. Poveda et al. 19* (COL); Carpanta Biological Reserve, 20 km due west of Junín, 4º35'N, 73º40'W, 2550 m, Aug-Dec 1990, *A. Repizzo & Z. Calle 217* (COL, MO, NY); Soacha, en áreas relictuales de bosque andino, Granja Ecológica El Porvenir, 04º34'36,3"N, 74º18'09,5"W, 2534 m, 26 Aug 2009, *O. Rivera Díaz et al. 4217* (COL); Soacha, en el sendero ecológico Granja Ecológica El Porvenir y en las manchas de bosque anexos a la casa, 2534 m, 15 Mar 2010, *O. Rivera Díaz et al. 4270* (COL); al S de Santandercito, ca. 2000 m, Jun 1951, *L. Uribe U. 2117* (COL, US); San Francisco, vereda de Sabaneta, 2600 m, 21 Jan 1968, *L. Uribe U. 6059* (COL, US); **Huila**: Cordillera Oriental, debajo de Gabinete en la hondonada del abra de San Andrés, 1900-2100 m, 24 Mar 1940, *J. Cuatrecasas 8655* (COL, F, US); forest around Mehrenberg road from Popoyán, 2350 m, 6 Jul 1984, *W. D'Arcy et al. 15644* (CORD, MO); La Plata, Vereda Agua Bonita, Finca Mehrenberg, 1200-1300 m, 20 Jul 1975, *S. Díaz P. et al. 717* (COL, RB); La Plata, Vereda de Agua Bonita, finca Meremberg, Km 106 de la vía La Plata-Popayán, 2º 23'11"N, 76º26'15"W, 2200 m, 18 Apr 1982, *J.H. Torres R. 965* (COL); **Meta**: Quetame, Inspección de Policía de Guayabetal, carretera al Calvario, 2000-2450 m, 11 Jun 1979, *G. Lozano C. 4036* (COL); **Nariño**: Reserva Natural La Planada, cerro Inciensal, sendero a la quebrada El Basal, 01º10'18"N, 78º00'09"W, 2148 m, *G. Herrera & J. Bittner 9479* (FMB); **Quindío**: Salento, Reserva Natural Alto Quindío, Acaime, 4º 37'45"N, 75º27'50"W, 2680 m, 11 May 1991, *C.A. Agudelo et al. 1596* (COL); Alto Río Quindío, camino a la reserva La Marina, 2600-2700 m, 8-9 May 1990, *R. Bernal 1828* (COL); Portachuelo, 1844, *J. Goudot s.n.* (P); Las Tapias, *J. Goudot s.n.* (K, P); Génova, Vereda Río Gris Alto, finca el Jardín, 2400 m, 15 Sep 1993, *C.A. López 99* (COL); Filandia, Vda. Cruces, Finca el Paraíso, 4º41'34"N, 75º37'12"W, 2000 m, 7-11 Jun 2002, *J.E. Mendoza & L. Quevedo 407 & 525* (FMB); Salento, Reserva Natural Acaime, 13 km E of Salento and 5 km E of Cocora, 04°37'N, 75°27'W, 2650-2800 m, 28-29 Jul 1995, *L.M. Renjifo 253* (MO); Laguneta, Salento, 2800 m, 21 Mar 1942, *K. von Sneidern 3044* (GH, S) & 11 Apr 1942, *K. von Sneidern 3214* (S); Génova, Vereda San Juan Alto, finca La Caucásica, 2500-2780 m, 19 Mar 1995, *C. Vélez et al. 5014* (COL); **Risaralda**: Santuario-Apia, orillas del Río San Rafael, Hacienda de Molina, 05º03'27"N, 75º56'03"W, 6 Dec 1989, *C. Barbosa 14060* (FMB); Pereira, Parque Natural Regional Ucumarí (PNRU), camino entre La Pastora y Peña Bonita, 4º42'30"N, 75º29'31"W, 2300-2600 m, 22 Nov 1989, *G. Galeano et al. 2127* (COL); Pereira, PNRU, 2460-3000 m, 21 Jun 1989, *F. González G. et al. 1553* (COL); Pereira, PNRU, ca. 22 km ESE of Pereira, 4º40'N, 75º35'W, 2100-2670 m, 25 May 1989, *J.L. Luteyn & O. Rangel 13146* (COL, NY); Pereira, PNRU, Cordillera Central, 04°45'N, 75°35'W, 1750-2300 m, 9-15 Apr 1994, *C. Múrcia 129* (MO) & 26-27 May 1994, *C. Múrcia 191* (MO); La Pastora, 04°45'N, 75°35'W, 2100-2550 m, 8-10 Aug 1995, *C. Múrcia 470* (MO); Río San Rafael, below Cerro Tatama, 2200-2400 m, 7-11 Sep 1922, *F.W. Pennell 10316* (NY); La Pastora, Reserva Ucumari, 4º42'34"N, 75º29'3"W, 2610 m, 12 Oct 1989, *O. Rangel Ch. et al. 5511* (COL); La Pastora, 4º42'54"N, 75º28'51"W, 2610 m, 10-12 Oct 1989, *O. Rangel Ch. et al. 5336 & 5539* (COL); **Santander**: San Joaquín, Vereda San Ignacio, camino entre "Loma negra" y "Morro Pardo", 06º23'23,9"N, 73º53'56,4"W, 2754 m, 23 Mar 2010, *S. Angel et al. 465* (COL); Charalá, Vereda Santa Helena, predio la Sierra, margen izquierdo aguas abajo del Río La Rusia, Santuario de Fauna y Flora Guanentá Alto Río Fonce, 06º01'N, 73º09'W, 2500 m, 8 Oct 1998, *J.J. Cadena-M. et al. 143* (COL); Encino, Reserva Biológica Cachalú, Vereda Río Negro, camino desde la horqueta hasta la Cascada romántica, 6º05'N, 73º08'W, 2000 m, 19 Mar 2008, *R. Cortés et al. 2432* (COL, UDBC); Floridablanca, Sitio desde El

Mortiño, camino al km 12, 7º6'54"N, 73º3'30"W, 1800 m, 23 Apr 2004, *C. Granados-Tochoy & J. Garzón 691* (COL); Corcova, 2380 m, 15 Aug 1977, E. *Rentería A. et al. 601* (COL, MO); Charalá, Carretera a Virolín, 3,5 km, 1350 m, 29 Nov 1989, *R. Sánchez et al. 1323* (COL); **Tolima**: Roncesvalles, a la orilla de la trocha hacia San José de las Hermosas, 2750 m, 1 Nov 1980, *L.A. Camargo 7781* (COL); La Portezuela, La Palmita, 1844, *J. Goudot s.n.* (P); Santa Isabel, Vereda Purima, 1 Aug 1980, *J.M. Idobro 10379* (COL); **Valle del Cauca**: Hoya del Río Cali, lado derecho del Río Pichindé, cuchilla de Los Cárpatos, 2300 m, 24 Jul 1946, *J. Cuatrecasas 21664* (CORD, F, US); Monte La Guarida (Cordillera Occidental), sobre La Carbonera, entre Las Brisas y Albán, 2000 m, 16-24 Oct 1946, *J. Cuatrecasas 22216* (CORD, F, US); Argelia, Vereda Las Brisas, 2140 m, 21 Jan 1983, *M.P. Franco Rosselli et al. 1645* & *1649* (COL, MO); Finca Zíngara, Km 18, carretera al Mar o S. Bolívar, vía a Dapa, 2100 m, 28 Feb 1994, *J.G. Gensini 103* (CUVC, NY); bosque de San Antonio, W of Cali, near television tower, 03°30'N, 76°37'W, 1950-2050 m, 15 Jul 1984, *A.H. Gentry 48174* (MO); La Elvira, Finca Zíngara, ca. 25 km W of Cali at km 18, 1600-1700, 20 Apr 1989, *J.L. Luteyn 12554* (CAS, NY); La Cumbre, Corregimiento de Bitaco, Vda. Chicoral, 3º34'13"N, 76º35'11"W, 2093 m, 23 Jul 2003, *H. Mendoza et al. 14946 & 15269* (FMB); Torremolinos, Km 22 de la carretera Cali-Buenaventura, 1900 m, 1990, *G. Múrcia 77* (MO); Finca San Pablo, Km 15 of Cali-Buenaventura Road, 1900, 26 Oct 1990, *G. Múrcia 77 B* (MO); La Cumbre, Cordillera Occidental, 1800-2100 m, 14-19 May 1922, *F.W. Pennell & E.P. Killip 5761* (GH, NY, US); Cuesta de Tocotá, Western Cordillera, 1500-1900 m, Dec 1905, *H. Pittier 737* (GH, US); La Cumbre, Vda. Chicoral, 2093 m, 21 Jul 2003, *M. Ruiz et al. 139* (FMB); Palmira, Corregimiento Potrerillo, La Nevera, finca El Orisol, carretera Potrerillo-Ataco, 2550 m, 25 May 1990, *R. Ruiz & E. Correa 929* (PSO); Cali, Finca Zíngara, 4 km hacia Dapa desde Km 18 de la carretera Cali-Buenaventura, 2000 m, 4 Mar 1994, *P. Silverstone-Sopkin et al. 6641* (CUVC, MO, NY) *& 6643* (CTES, NY); Palmira, Vda. San Nicolás, Finca San Nicolás, 3º31'57,47"N, 76º9'21,65 W, 2100 m, 26 Feb 2005, *W.G. Vargas et al. 4238* (FMB).

ECUADOR. **Napo**: Tena, Cordillera de los Huacamayos, entre Sisahua y Chacana Loma, 00°48'S, 78°07"W, 2100-2310 m, 10 Aug 1995, *J. Jaramillo & I. Tapia 18525* (QCA); **Zamora-Chinchipe**: Bombuscaro, vertiente derecha, sendero por el otro lado del río a las fincas, 04º06'43"S, 78º57'55''W, 960 m, 30 Jul 1998, *O. Cabrera et al. 291* (QCNE); Zamora, buffer zone near the eastern border of Parque Nacional Podocarpus (PNP), 04º05'34"S, 78º57'38" W, 984 m, 4 Jun 2007, *J.L. Clark 9965* (NY); along road between Los Encuentros and El Sarsa, 13.7 km S of bridge over Río Zamora at Los Encuentros, 03°48'40"S, 078°36'28"W, 1455 m, 15 Jul 2004, *T.B. Croat 91102* (MO); PNP, Bombuscaro entrance, 04º07'S, 78º58'W, 1050 m, 30 Aug 2008, *J. Homeier & N. Cumbicus 4060* (QCNE); limit of PNP, Quebrada del León, 4º7'S, 78º58'W, 1100-1150 m, 31 Aug 1988, *J.E. Madsen & L. Ellemann 75101* (QCA); Quebrada del León, affluent of Río Bombuscara S of Zamora, 4º7'S, 78º58'W, 1100 m, 5 Feb 1989, *B. Øllgaard et al. 90357* (QCA); Zamora, 1000 m, 4°05'S, 78°57'W, Jan 1995, *W. Palacios & M. Tirado 13256* (MO, QCNE).

PERU. **Pasco**. Oxapampa, Distr. Huancabamba, PN Yanachaga-Chemillen, desde Centro Yanachaga (antes Hospedaje PROSOYA), unos 6 km hacia arriba, entrando en el bosque primario, 9 May 2019, *G.E. Barboza 5047* (CORD); en la Quebrada Yanachaga,

10º23’40’’S, 75º29’18’’W, 2317 m, 23 Jan 2020, *G.E. Barboza & S. Leiva González 5053* (CORD); Parque Nacional Yanachaga-Chemillén (PNYCh), sector Tunqui, camino hacia María Puñis, 10º16'31.36"S, 75º30'58.72"W, 1895 m, 18-21 Oct 2008, *M. Cueva 218* (HOXA, MO, USM) & 6-18 Feb 2009, *M. Cueva 460* (HOXA, USM, MO); sector Daniel, 10º26'27.3"S, 75º26'26.29"W, 2240 m, 24-28 Feb 2009, *M. Cueva 462* (HOXA, MO, USM); Distr. Oxapampa, PNYCh, Sector San Alberto, alrededores del refugio el Cedro, 10º32'43,3"S, 75º21'29,5"W, 2483 m, 26-27 Apr 2009, *M. Cueva 628* (HOXA, USM, MO); sector San Daniel, 10º26’28’’S, 75º26’45,3’’W, 2200-2350 m, 15 Aug 2005, *A. Monteagudo et al. 9371* (HOXA, MO); cercanías del Hito del Sector San Daniel hacia la trocha Erica, en la cordillera Yanachaga, 10º26’46’’S, 75º26’18’’W, 2260 m, *A. Monteagudo et al. 13706* (HOXA, MO, USM); Chotabamba, La Suiza Vieja, antigua carretera hacia cerro de Pasco, 10º40'23"S, 75º31'28.6"W, 1935-2415 m, 8 Aug 2005, *E. Ortiz V. et al. 752* (HOXA, MO, USM); sector Grapanazu, 10º26'12"S, 75º23'13"W, 2310 m, 11 Oct 2003, *R. Rojas et al. 1649* (HOXA, MO); sector Grapanazu, 10º25'55"S, 75º27'33"W, 2410 m, 12 Oct 2003, *Rojas, R. et al. 1744* (HOXA, MO); sector Huampal, trocha Robin Foster, 10º10'59"S, 75º34'17"W, 1210 m, 20 Aug 2009, *L. Valenzuela et al. 13438* (HOXA, MO, USM); sector Pan de Azúcar, 10º11'43’’S, 75º35'05’’W, 1500 m, 18 Apr 2012, *L. Valenzuela 20885* (HOXA, MO); Quebrada Yanachaga, 10º23'44"S, 75º28'56"W, 2250 m, 13 Jun 2003, *R. Vásquez et al. 28196* (HOXA, MO); same locality, 10º24’13’’S, 75º29’04’’W, 1900 m, 11 Jan 2005, *R. Vásquez et al. 30563* (HOXA, MO); sector San Daniel, 10°26’35’’S, 75°26’16’’W, 2200-2500 m, 10 Mar 2006, *R. Vásquez et al. 31010* (HOXA).

15. Capsicum eshbaughii Barboza
BOLIVIA. Cochabamba: Carrasco, camino Cochabamba-Comarapa, Copachuncho, ca. 3000 m, 29 Mar 1987, *D. Flores 89* (CORD, LPB, NY); **Santa Cruz**: Florida, Samaipata, ca. 650 m desde la ruta, 18º10’23’’S, 63º52’03’’W, 1635 m, 9 Mar 2018, *C. Carrizo García 67* (CORD); El Sauce, west of Samaipata, 1730 m, 30 Mar 1987, *W.H. Eshbaugh 1943 d* (CORD), 7 Feb 1989, *W.H. Eshbaugh 1943 C* (CORD) & 1 Aug 1990, *W.H. Eshbaugh 1943 a* (CORD); ca. 2-5 km del pueblo de Samaipata sobre el camino hacia al Mina, 18°09'37"S, 63°52'32"W, 19 Dec 2004, *M. Mendoza & D. Vidal 1265* (LPB, MO, USZ); 5 km (by air) SE of Mairana, on road to Samaipata, at “Quebrada Seca”, 18º09’S, 63º56’W, ca. 1550 m, 4 Feb 1988, *M. Nee 36164* (CORD, G, LPB, MO, NY, SI, USZ); 0.5-1 km N of Samaipata, 18°10'S, 63°52'00"W, 1600 m, 31 Dec 1992, *M. Nee & I. Vargas C. 43483* (LPB, MO, NY, USZ); at Achira Camping resort, 18°10'S, 63°49'W, 1370 m, 18 Apr 2002, *M. Nee 52197* (MO, NY); on descent from Samaipata to Mairana, 18º09'20"S, 63º55'47"W, 13 Nov 2006, *J.R.I. Wood et al. 22449* (USZ).

Cultivated
ARGENTINA. Córdoba: cult. Invernadero IMBIV (UNC), seeds from Carrizo García 67 (Bolivia), 21 Mar 2021, *N. Palombo 19* (CORD).

UNITED STATES OF AMERICA. California: Yolo County, University of California at Davis, Department of Vegetable greenhouse, seeds from Yerba Buena, Mairana area, 158 km W of Santa Cruz, Bolivia, 1950, *P.G. Smith SA 281* (CORD, DAV, UC); **Indiana**: Bloomington,

cult. in IU greenhouse, seeds from 158 km W of Santa Cruz, Bolivia, on road to Cochabamba (P. Smith C 281), 1300 m, 14 Jun 1960, *C.B. Heiser C 301* (UC).

16. Capsicum eximium Hunz.

ARGENTINA. **Jujuy**: ca. 7.3 km de San Francisco rumbo a Valle Grande, 23°36'23"S, 64°57'19"W, 1421 m, 16 Feb 2012, *G.E. Barboza et al. 3543* (CORD); Santa Clara, 24°20'S, 64°33'W, 1400 m, 16 Feb 1995, *N.B. Deginani et al. 918* (MO, NY, SI); Cachipunco, 13 Feb 1964, *H.A. Fabris et al. 5229* (LP); 6 km SW de La Mendieta, 1,4 km E de RP 56, 13 May 1998, *A. Krapovickas 47426* (CTES, LPB, MEXU); RP 6, 6 km E de Santa Clara, 14 May 1998, *A. Krapovickas 47449* & *47466* (CTES, GH); RP 6, 7 km NE de Aguas Blancas, 14 May 1998, *A. Krapovickas et al. 47484* (CTES); camino a Valle Grande, entre Refugio San Francisco y Arroyo Yerba Buena, 4 Dec 1969, *P.R. Legname & A.R. Cuezzo 7204 C* (LP); ca. 4 km by road N from San Francisco toward Valle Grande, 1420 m, 17 Apr 2000, *M. Nee & L. Bohs 50807* (MO, NY); camino a El Fuerte desde Santa Clara, 24°19'6"S, 64°30'46"W, 1186 m, 12 Apr 2008, *A.C. Slanis 72* (SI); **Salta**: Cuesta de El Cebilar y El Lajar, 20-30 km al SE de Guachipas, 1300-1800 m, 3 Apr 1984, *A. Del Castillo & R. Neumann 383 p.p.* (MCNS); PN Baritú, Lipeo, 3 Dec 1997, *N. Hilgert 2027* & 8 Dec 1997, *N. Hilgert 2061* (MCNS); Quebrada de San Antonio, Pampa Grande, 1600 m, 6 May 1942, *A.T. Hunziker 1907* (BM, CORD, G, MCNS, MO, NY, SI, US); La Candelaria: Potrero El Nogalito, 26 Apr 1964, *Z.A. de Jeréz et al. 4919 C* (LIL); La Ollada, Apr 1934, *R. Lahitte s.n.* (BAB 49846); El Cebilar, 1200-1400 m, 7 Feb 1983, *L. Novara & R. Neumann 3121* (CORD, MCNS, MO); Los Toldos, entre la Municipalidad y El Arranay, 3-4 km al sur del pueblo, 1600 m, 18 Mar 1985, *L. Novara 5102* (MCNS); alrededores del pueblo de Santa Victoria, 2500-2800 m, 13-16 Dec 1988, *L. Novara 8346* (CORD, G, M, MCNS); Capital, Sierra de Vélez, Cerro de la ermita, detrás de la capilla de la virgen de Schoenstadt, 1300-1400 m, 15 Jan 2005, *L. Novara et al. 12206* (CORD, MCNS); Los Toldos, camino al Rio Lipeo, 2 Jun 1993, *G. Roitman s.n.* (BAA 22608); Sierras de Metán, cerro El Cobre, 18-20 km al W de Lumbreras, 25º13'00.3"S, 65º07'14.7"W, 1450 m, 7 Apr 2006, *J. Tolaba 3918* (MCNS); Orán, Cordillera Oriental, sierra de Zenta, San Andrés, 23°05’22’’S, 64°52’03,8’’W, 1700-1800 m, 23 Apr 1998, *A. Schinini et al. 34778* (CTES); Santa Victoria, Lizoite, 20 Jan 1983, *E.M. Zardini et al. 1641* (GH, MO); Acoite, 22 Jan 1983, *E.M. Zardini et al. 1740* (MO); Pucará, 28 Jan 1983, *E.M. Zardini 1870* (MO); **Tucumán**: Burruyacu, 1400 m, 1918, *E. Bailetti 207* (LIL); Trancas, Cerro de Medina, 1000 m, 28 Mar 1914, *L. Monetti 2257* (LIL).

BOLIVIA. **Chuquisaca**: Tarabuco, ca. 30 km hacia Sudañez, 2500 m, 7 Mar 1981, *S. Beck 6255* (CORD, LPB, SI); 3 km S of Mojotoro, 32 km NE of Sucre on hillside opposite Rio Chico, 2030 m, 8 Mar 1971, *W.H. Eshbaugh 588* (IND, MU); Yotala, retén de Yotala, sobr el cerro al frente del retén, 19º09.25’S, 65º15.54’W, 2505 m, 17 Jan 2004, *M. Mendoza & R. Lozano 827* & 828 (USZ); below the gorge between Tarabuco and Zudañez, 2700 m, 18 Nov 1994, *J.R.I. Wood 8774* (NY); on descent from Tarabuco to Zudañez, 6-7 km above Lamboyo, 2700 m, 21 Mar 1998, *J.R.I. Wood & M. Serrano 13331* (USZ); 1 km al N de Abra San Miguel, carretera Sucre-Potosí, 19º22'25.4’’S, 64º11'20,3"W , 2216 m, 21 Feb 2004, *J.R.I. Wood & J. Gutiérrez 20218* (LPB, USZ); bajando Lamboyo hacia Zudañes, 19º04.29’S, 64º48.21’W, 2500 m, 29 Jan 2005, *J.R.I. Wood & H. Huaylla 21532* (LPB);

Cochabamba: camino de Pasorapa a Peña Colorada, 2060 m, 8 Feb 1994, *C. Antezana 721* (BOLV, LPB); camino Aiquile-Peña Colorada, en la subida del cerro antes de bajar hacia Peña Colorada, 18º11'17"S, 64º55'67"W, 2155 m, 26 Jan 2004, *M. Atahuachi et al. 773* (LPB); Vic. Cochabamba, 1891, *M. Bang 1126* (F, GH, K, MO, US, W, WIS); Comunidad Hoyadas, desde Totora rumbo a Aiquile, ca. 40 km antes de llegar a Aiquile, en los alrededores de la casa del Sr. Sergio Rojas Moreira, 17°55'59''S, 65°9'49''W, 2419 m, 10 Dec 2017, *G.E. Barboza 4895 & 4896* (BM, CORD, LPB); Aiquile, camino Totora-Aiquile, Mesa Rancho (en casa Sr. Cirilo), 18°6'15''S, 65°10'25''W, 2115 m, 10 Dec 2017, *G.E. Barboza 4902* (CORD, SI) *& 4903* (CORD); Mizque, Tako-Tako, 2050 m, 21 Dec 1949, *M.A. Brooke 5934* (F, NY); Comunidad Suero Mayu, 2230 m, 11 Jan 1990, *R. Caballero Pardo 103* (LPB); Puente de Pojo, 2200 m, *M. Cárdenas s.n.* (CORD 00087957; L); near Río Caine, 2000 m, Jan 1942, *M. Cárdenas 2270* (CORD fragment, US); Río Caine, 1180 m, Jan 1949, *M. Cárdenas 4237* (US); Pojo, 2900 m, *M. Cárdenas 5114* (GH, US); pasando Totora, rumbo a Aiquile, 17°50'42.1"S, 65°10'01.6"W, 2760 m, 14 Feb 2017, *C. Carrizo García & L. Amarilla 93* (CORD); between Alfamayo and Tincomayo, 17º 57'S, 65º 55'W, 6 Mar 1980, *J. Hawkes et al. 6558* (C); camino del cruce Raqay pampa Molineros hacia Novilleros, 2750 m, 30 Jan 1994, *A. López & E. Saravia 203* (LPB); cerca a Tinku-Mayu, camino Anzaldo a Viña, 6 Mar 1980, *E. Meneces & W. Terceros 1004* (LPB); Aiquile, camino a Raqaypampa, 2249 m, 18 Mar 1998, *M. Mercado & R. Navia 1811* (LPB); Capinota, camino entre las Comunidades Azary-Siguayo, 19°06'36"S, 65°13'51"W, 2735 m, 8 Apr 2007, *J.A. Peñaranda 458* (HSB, MO); Apillapampa, 17º48.894'S, 66º12.278'W, 2840 m, 2 Mar 2003, *E. Thomas 291* (LPB); Pasorapa, 600 m del Pueblo de Pasorapa, en la salida sobre el camino hacia Peña Colorada, 18º19.15'S, 64º11.14'W, 2098 m, 21 Feb 2003, *J.R.I. Wood & M. Mendoza 19165* (LPB); same locality, 18º19.21'S, 64º40.41'W, 2352 m, 21 Feb 2003, *J.R.I. Wood & M. Mendoza 19166* (LPB); Totora, sobre camino Totora a Omereque, 17º52.46'S, 65º02.65'W, 2471 m, 26 Dec 2004, *J.R.I. Wood et al. 21229* (LPB); sobre el camino Cochabamba-Capinota, entre Charamoco y Ucuchi, 2500 m, 5 Feb 2005, *J.R.I. Wood & M. Atahuachi 21581* (LPB); **La Paz**: Inquisivi, ca. 10 km desde el cartel de bienvenida a Inquisivi, rumbo a Circuata, 2250 M, 28 Apr 2006, *G.E. Barboza et al.1860* (CORD); Inquisivi, 3 km hacia Circuata, ca. 2200 m, 20 Feb 1981, *S. Beck 4465* (CORD, F, LPB, SI); Loayza, pasando Luribay rumbo a La Paz (salida por Patacamayo), 17°3'48''S, 67°41'16''W, 3001 m, 4 Dec 2017, *G.E. Barboza 4885* (BM, CORD, LPB, MO, NY,); debajo de Inquisivi, 2150 m, 29 Dec 1989, *S. Beck 17259* (CORD, LPB); Sud Yungas, Lambate, 74 km hacia La Plazuela, 16º36'S, 67º30' W, 1900 msm, 5 Mar 2000, *S. Beck 25261* (CORD); valley separating Inquisivi and Sita, 16°58'S, 67°10'W, 2150-2430 m, 22 Dec 1989, *L.J. Dorr 6801* (CAS, CTES, LPB, MO, SI); San Pedro, vic. Sorata, 2550 m, Jan-Mar 1861, *G. Mandon 427* (B, BR, CORD fragment, G, GH, GOET, K, LE, P, S, W); between Yamora and Micayani, headwaters of Rio Khokhoni, 3.5 km (by air) SSE of Inquisivi, 16º56'S, 67º07'W, 2850 m, 14 Jan 1989, *M. Nee 37575* (CORD, LPB, MO, NY, USZ); between Yamora and Micayani, 4 km (by air) SE of Inquisivi, 16º56'S, 67º07'W, 2550-2650 m, 14 Jan 1989, *M. Nee 37585* (MO, NY); **Potosí**: Potosí, 2450 m, Nov 1911, *O. Buchtien 3262* (US); Chayanta, Pocoata, al frente de Tacarani, 18º30'46"S, 66º08'30"W, 2888 m, 2 Feb 2016, *I. Jiménez et al. 7517* (LPB); Cruz Kasa, 2525 m, Sep 1996, *V.H. Zamora 193* (LPB); **Santa Cruz**: entre el Bello y San Gerónimo, alrededores de una quebrada, 18°25'55"S 064°11'22"W, 1780 m, 4 Mar 2011, *L. Arroyo et al. 5252* (MO, USZ); Quinales, desvío al norte desde Comarapa rumbo a la Laguna Verde, 17º52'26.5''S, 64º32'14''W, 2090 m, 30 April 2006, *G.E. Barboza et al.*

1905 (CORD); Santa Rosita, al sur de Vallegrande, a unos 2 km del desvío de la ruta, 18°31'14''S, 64°5'50''W, 2014 m, 13 Dec 2017, *G.E. Barboza 4914* (CORD, LPB); Camiri, 67 km hacia Monteagudo, 1150 m, 27 Oct 1983, *S. Beck & M. Liberman 9789* (CORD, LPB); Comarapa, road from Pulquina to Santa Cruz, Quilco, c. 10 km from Empalizada, 18°84'82''S, 64°38'46''W, 1685 m, 3 Mar 2006, *N. Biggs et al. 118* (LPB, K, USZ); Mataral, pasando San Isidro, ca. 2 km antes de Tambo, 18°05'42.1"S, 64°20'38.3"W, 1960 m, 15 Feb 2017, *C. Carrizo García & L. Amarilla 31 & 32* (CORD); alrededores de Moro Moro, camino del basural y cementerio, a la vera del camino, 18°22'20.2"S, 64°19'17.1"W, 2354 m, 16 Feb 2017, *C. Carrizo García & L. Amarilla 33* (CORD); saliendo de Vallegrande hacia Pucará, zona urbana, antes de llegar a Guadalupe y tomar la Ruta del Che, 18°31'14.6"S, 64°05'24.1"W, 1989 m, 16 Feb 2017, *C. Carrizo García & L. Amarilla 36, 37, 38 & 39* (CORD); Abra de Quine, at top of hill 200-210 km west of Santa Cruz on the road to Comarapa, 1900-1950 m, 2 Mar 1971, *W.H. Eshbaugh 575* (IND, MU); Pulquina, camino a BuenaVista, entre Buitron y San José, 18°19'49''S, 64°27'46''W, 1908 m, 6 Apr 2008, *M. Garvizu et al. 1156* (USZ); Santa Rosita, a 50 m de la carretera principal siguiendo por el camino angosto a Huasa Cañada, 18º32,56'S, 64º05,53W, 1993 m, 16 Jan 2003, *M. Mendoza & E. Calzadilla 362 & 363* (LPB); ca. 3-4 km N de Comarapa, 17°53'28"S, 64°33'05"W, 2215 m, 11 Jan 2004, *M. Mendoza & E. Calzadilla 801* (LPB, MO, USZ); Pulquina, ca. 4-6 km de Pulquina, Estancia La Isla de Vivianas y sus alrededores, 18º07'04''S, 64º24'06''W, 26 Feb 2006, *M. Mendoza & M. Balcazar 2021* (NY, USZ); 6.5 km NNW of center of Vallegrande, 18º26'S, 64º07'W, 1950 m, 9 Mar 1988, *M. Nee & J. Solomon 36558* (CORD, LPB, MO, NY); on road from El Trigal to Mataral, 6 km NW of Estancia Cochabambita, 18º12'S, 64º12'W, 1725 m, 9 Mar 1988, *M. Nee & J. Solomon 36578* (CORD, LPB, MO, NY, USZ); 5.5 km S of Vallegrande, vic. Santa Rosita, Quebrada Hausa Cañada, 18º32'S, 64º06'W, 2050 m, 31 Dec 1988, *M. Nee & I. Vargas C. 37448* (CORD, MO, NY, USZ, WIS); 1 km S of highest point on Mataral to Trigal road, 18º13'S, 64º12'W, 1700 m, 24 Dec 1989, *M. Nee & I. Vargas C. 38323* (CORD, MO, NY, P); Quebrada del Zorro, 2.5 km S of center of Vallegrande, 18º30'S, 64º06'W, 2040 m, 25 Dec 1994, *M. Nee 38361* (CORD, LPB, MO, NY); valley of the Río Grande, on road from Pucará to the new bridge over the Río Grande, 18º41'30'' S, 64º15'45''W, 1850 m, 29 Jan 1994, *M. Nee & I. Vargas C. 44747* (CORD, LPB, MO, USZ); along highway from Comarapa to Mairana, 3.9 km E and down from Abra del Quiñe, 18º06'22''S, 64º19'35''W, 1780 m, 13 Apr 2007, *M. Nee et al. 55070* (NY, USZ); camino hacia la localidad de Arenales (Río Mizque), 18°18'12"S 064°24'38"W, 1934 m, 18 Jan 2011, *G.A. Parada et al. 2760* (MO, USZ); Valle Grande, zona SW del centro, 18°29'19"S, 64°06'44"W, 2048 m, 11 May 2008, *J.A. Peñaranda et al. 817* (HSB, MO); Cochabambita, 1700 m, 27 Jan 1993, *C. Saravia Toledo 10325* (CORD); Valle del Trigal (Finca El Pugro), 1680 m, 27 Jan 1994, *C. Saravia Toledo 12126* (CORD); 15,5 km de El Trigal, camino a Tembladeral, 1800 m, 29 Jan 1994, *C. Saravia Toledo 12245* (CORD, CTES, SI); Um Vallegrande, 1900 m, 28 Jan 1928, *C. Troll 1292* (B, CORD fragment, M); San Antonio, 2,5 km al S de Vallegrande, 18º30'S, 64º6'W, 2030 m, 10 Jan 1989, *I. Vargas C. 36* (MO, NY, USZ); Huasacañada, 5 km al S de Vallegrande, 18º31.5'S, 64º5.7'W, 2050 m, 3 Nov 1990, *I. Vargas C. 816* (CTES, LPB, MEXU, MO, NY, USZ) & 4 Jan 1991, *I. Vargas C. 906* (LPB); Parque Nacional Amboró, San Juan del Potrero, trayecto entre La Huerta y Yunguillas, 17°57'S, 64°23'W, 1700-2000 m, 12 May 1992, *I. Vargas C. 1382* (MO, USZ); Pucará, bajando de Pucará rumbo a Santa Rosa de Río (ca. media bajada), 18º42.25'S, 64º65.93'W, 1370 m, 25 Jan 2005, *J.R.I. Wood & M. Mendoza 21490* (LPB,

USZ); **Tarija**: entre Tarija y Tomatitas, cerca del camino, 1920 m, 2 Feb 1986, *E. Bastión 681* (CORD, LPB, SI); Bermejo, 1400 m, 15 Feb 1903, *K. Fiebrig 2072* (CAS, CORD, GH, GOET, K, M, P, S, US, W); Tolomosa, 1 Feb 1917, *R. Fries 1110* (S); El Sunchal, por la quebrada del Río Negro, 22º05’15’’S, 64º38’02’’W, 1600 m, 10 Feb 2006, *S. Gallegos 318 A* (LPB); Cercado, PERTT, Campo Experimental, 1950 m, 9 Feb 1982, *R. Gerold 175* (CORD; LPB); cerca de Padcaya, entrando a mano izquierda de la quebrada Wayco, 28 Jan 1988, *M. Liberman et al. 1833* (CORD, LPB); cerca de Chocloca, en la Loma cerca del Río Wayco, 1850 m, 4 Feb 1988, *M. Liberman et al. 2052* (LPB, NY); en los alrededores de la localidad de San Lorenzo, 21º25.068’S, 64º45.568’W, 2031 m, 6 Apr 2005, *C. Manchego CEP T21* (LPB); same locality and date, *C. Manchego CENP T22* (USZ); Campamento Río Negro, 1500 m, 5 Feb 1953, *T. Meyer 17527* (CORD, LIL, W); between Padcaya and La Momora, 22º06.071'S, 64º40.421'W, 1425 m, 14 Jan 2001, *J.R.I. Wood & D.J. Goyder 16736* (LPB); Pampa Redonda, 21º43'11"S, 64º51'03"W, 2045 m, 8 Feb 2006, *F. Zenteno et al. 3608* (LPB).

Cultivated
ARGENTINA. **Buenos Aires**: Capital Federal, cult. Jardín Botánico de la Fac. de Agronomía y Veterinaria de Buenos Aires, semillas de A. T. Hunziker 1907, 15 Feb 1943, *A.T. Hunziker 7345* (CORD); **Salta**: Capital, cult. Jardín particular Bº Tres Cerritos, 20 Jan 2007, *G.E. Barboza 1919* (CORD); cult. en casa particular Bº Universitario (semillas de Dept. Güemes), 14 Apr 1998, *E. Lusvarghi s.n.* (CORD 500) & 9 Apr 2002, *E. Lusvarghi s.n.* (CORD 00087955);

UNITED STATES OF AMERICA. Indiana: Bloomington, cult. in Bloomington greenhouse, seeds from Pojo, near Comarapa, Bolivia (through Cárdenas AC 1863), 18 Feb 1957, *C.B. Heiser 4197* (IND, US); cult. at IU Experimental Field, 8 Jul 1957, *C.B. Heiser 4197* (CORD, IND); cult. in the experimental garden at IU, 26 Aug 1958, *C.V. Morton 10907* (US); cult. in IU greenhouse, seeds from Cochabamba, 1 km south Pojo bridge, 27 Feb 1963, *P.G. Smith SA 272* (IND, MU); grown IU experimental field, seed collected from around a home at Pulquina, Chaco Boreal area of Bolivia, 10 Jun 1960, *P.G. Smith SA 276* (IND, MU); grown IU greenhouse, seeds from 235 kilometers east of Sucre, 23 Jan 1962, *P.G. Smith SA 325* (IND); cult. in IU experimental field, from seed collected in market in Tarija, 13 Sep 1960, *P.G. Smith PS* 350 (IND, MU); cult. in IU greenhouse, seeds from market in Tarija, said to be from San Ana, 17 Sep 1963, *P.G. Smith SA 351* (IND, MU).

Hybrids *C. eximium* x *C. pubescens*
BOLIVIA. Chuquisaca: Tomina, Campo Redondo, km 496, rumbo a Sucre, ingresando unos 700 m de la ruta (granja Sr. R. Chenaud), 19º18’08’’S, 64º19’46’’W, 2066 m, 16 Dec 2017, *G.E. Barboza et al. 4924, 4925 & 4926* (CORD); **La Paz**: Sud Yungas, a ca. 6,5 km de Huancané rumbo a San Isidro, 2900 m, 26 Apr 2006, *G.E. Barboza et al. 1849* (CORD).

17. Capsicum flexuosum Sendtn.
ARGENTINA. **Corrientes**: Santo Tomé, Ea. San Juan Bautista, 28º10’S, 55º38’51’’W, 100 m, 17 Apr 2005, *G.E. Barboza et al. 1487* (CORD); Capital, ciudad, *A.J.A. Bonpland s.n.* (P); Santo Tomé, Ea. Garruchos, potrero Puente, 11 Feb 1972, *A. Krapovickas et al. 21558* (CTES); Costa del Río Uruguay, 13 Apr 1974, *A. Krapovickas et al. 25312* (CTES, GH);

Ituzaingó, Ea. Rincón Chico, 17 km NE de San Carlos, 1-5 Mar 1985, *S. Tressens et al. 3092* & *3097* (BAA, CTES, MO); **Misiones**: Cuartel Yacutinga, sobre camino Yaguarete, 8 May 1996, *L. Amarilla et al. 31* (CTES); pasando el pueblo de Santa Ana, rumbo hacia el Río, 27º21'33,4''S, 55º35'14,5''W, 7 Dec 2002, *G.E. Barboza et al. 426* (CORD); Parque del Salto Encantado, a ca. 7 km del INTA de Aristóbulo del Valle, rumbo a Jardín América, 27º05'40''S, 54º56'22''W, 550m, 15 May 2004, *G.E. Barboza et al. 1027* (CORD); 20 km del desvío sobre RN 14, hacia el Predio Guaraní, 15 May 2004, *G.E. Barboza et al. 1034* (CORD); Predio UNLP: Valle del arroyo Cuña Pirú, borde del arroyo Cuña Pirú, 21 Sep 1999, *F. Biganzoli et al. 544* (SI); San Antonio, 25 Apr 1955, *M. Buchinger & Rodríguez 3222* (CORD); Campo Viera a Campo Grande, 14 Feb 1978, *A.L. Cabrera & A.A. Sáenz 29195* (SI); a ± 22 km de El Soberbio, al costado del camino que va a Saltos del Moconá, 27°11'27''S, 54°0'44''W, 3 Mar 2012, *F. Chiarini & G. Wahlert 875* (CORD); San Ignacio, Dec 1914, *R.H. Chodat 47* (G); Parque Nacional Iguazú, catarata inferior, 31 Oct 1982, *L. Cusato 1667* (BAA); 6 km del cruce entre RP 4 y camino de Bonpland hacia Colonia Almafuerte, 8 May 2004, *J. Daviña & A. Honfi 597* (CORD); camino a puerto Paraíso, 10 km pasando el desvio, 27°15'S, 54°02'W, 150 m, 27 Feb 2000, *N. Deginani et al. 1523* (MO); Parque Provincial Piñalito, alrededores del Salto Merri, 26º26'S, 53º49'W, 5 Mar 2000, *N. Deginani et al. 1701* (CORD, SI); Bonpland, 3 Jan 1908, *E.L. Ekman 808* (CORD fragment, G, S, US); Circuito Superior, 8 Mar 2006, *F.E. Gatti 24* (CTES); Sierra Morena, *S. Heinonen 59* (SI); Bonpland, 20 Aug 1909, *P. Jörgensen s.n.* (BAB, CORD); Paraje Paraíso, comunidad aborigen Guabyrá Poty, 7 Sep 2000, *H.A. Keller 288* (CTES); Predio Guaraní, tramo I, 26°54-59'S, 54°12-18'W, 26 Jul 2001, *H.A. Keller 1144* (CTES); Paraje Paraíso, 16 Oct 2001, *H.A. Keller 1349* (CTES); Predio Guaraní, camino a arroyo Soberbio, 26°54'S, 54°12'W, 8 Aug 2002, *H.A. Keller et al. 1945* (CTES); Paraje Paraíso, aldea aborigen Guavirá Poty, 5 Jun 2003, *H.A. Keller & A. Ferreira 2135* (CTES); Ruiz de Montoya, aldea aborigen Takuapi, 17 Jan 2005, *H.A. Keller 2957* (CTES); Concepción, aldea aborigen Yraka Miri, 15 Jul 2008, *H.A. Keller 5570* (CTES); San Ignacio, acceso al Club del río, 152 m, *H.A. Keller & N.G. Paredes 7798* (CTES, SI); aldea guaraní Teko' a Miri, 26°53'24.1''S, 55°07'38.5''W, 13 Apr 2011, *H.A. Keller et al. 10099* (CTES); Iguazú, Puerto Península, 17 Feb 1971, *A. Krapovickas et al. 18340* (BAA, CTES, P, US); Leandro N. Alem, Cerro Azul, *S. Kummritz & D. Ohashi 99* (CTES); Ruta Eldorado a San Pedro, márgenes del Ao. Piraý Guazú, *P.R. Legname et al. 7356* (CTES); in vicin. Coloniae Bonpland, *W. Lillieköld s.n.* (S); Santa Ana, 2 Dec 1904, *A. de Llamas 729* (BAB); Santa Ana y alrededores, Ver. 1907, *A. de Llamas s.n*. (SI); Jardín América, Mar 1965, *R. Martínez Crovetto 135* (CTES); Eldorado, 3 Mar 1944, *T. Meyer 5506* (GH); Oberá, 25 Mar 1944, *T. Meyer 6694* (LIL); Arroyo San Juan, 14 Jan 1947, *T. Meyer 11550* (LIL); Loreto, 220 m, 27 May 1947, *J.E. Montes 398* (BAB, CORD); Santa Ana, 12 Nov-20 Dec 1945, *J.E. Montes 1635* & *1689* (LIL); Loreto, 14-21 Mar 1946, *J.E. Montes 2139* (CORD, LIL); Puerto Gisela, 240 m, 31 Mar 1946, *J.E. Montes 2140* (CORD, LIL); Loreto, *J.E. Montes 2155* (C, LIL), 12 May 1946, *J.E. Montes 2213* (F, US), La Pastora, 220 m, 30 May 1946, *J.E. Montes 2305 b* (B, CORD, LIL, W), San Juan, 28 Dec 1946, *J.E. Montes 2353* (SI); La Pastora, 18 Jun 1946, *J.E. Montes 2375* (LIL) & 11 Jul 1946, *J.E. Montes 2411* (C, CORD, SI); Loreto, 3 Jun 1943, *J.E. Montes 2411b* (SI), 2 Sep 1946, *J.E. Montes 2476* (US); Puerto Rico, 27 May 1949, *J.E. Montes 4026* (LP); Piray-Guazú, 6 Jul 1949, *J.E. Montes 4277 & 4289* (CORD, LP); Loreto, 18 Feb 1951, *J.E. Montes 12314* (CORD, LP); Monte Carlo, 205 m, 8 Feb 1955, *J.E. Montes 14727* (F, MO, MY, NY); Obraje El Caburei, 450 m, 19-20 Jul 1957, *J.E. Montes 27474 & 27476* (F, MO); Parque Provincial Cruce

Caballero, ruta Nac. 14, 14 km de San Pedro camino a Tobuna, 26º31'S, 53º59'W, 540 m, 14 Feb 1996, *O. Morrone 780* (MO, NY, SI); RN 101, Parque Provincial Urugua-í, 10 km de Deseado camino a Bernardo de Irigoyen, 25º50'S, 53º59'W, 360 m, 17 Feb 1996, *O. Morrone et al. 932* (CORD, MO, SI); Reserva Nac. Estricta San Antonio, a 6 km de San Antonio, 26º01'S, 53º47'W, 15 Oct 1996, *O. Morrone et al. 1444* (CORD, MO, SI); Arroyo Paranaí Guazú, por RN 12, en propiedad privada "Recreo Paranaí Guazú, 24 Mar 1998, *E. Moscone 229* (CORD); PP Cruce Caballero, 12 Dec 1997, *M. Múlgura et al. 1779* (CORD, MO, SI); Finca Osonunú, camino de acceso, 19 Sep 2000, *M. Múlgura et al. 2112* (SI); Reserva de Vida Silvestre Urugua-í, alrededores de la casa, sendero, 25°58'S, 54°7'W, 400 m, *M. Múlgura et al. 3322* (SI); Parque Provincial Urugua-í, Sendero Dr. L.H. Rolón, 25º51'S, 54º10'W, 300 m, 16 Mar 2002, *M. Múlgura et al. 3391* (CORD, SI); Reserva Biológica de Biosfera Yabotí, arredores da comunidade indígena Caramelito, 500 m, 17 Jan 2007, *J. Paula-Souza et al. 7336* (BHCB); from Posadas to Iguazú, by the bridge on the Aguaray-miní, 19 Feb 1992, *T.M. Pedersen 15791*(C, CTES); Picada zona Aeroclub, 11 Jul 1989, *M.E. Rodríguez & R.D. Aranda 171* (CTES); Capital, Establecimiento Santa Inés, Jul 1926, *A.C. Scala s.n.* (F 931931, LP); Establecimiento La Plantadora, Aug 1926, *A.C. Scala s.n.* (LIL, LP 011434); Santiago de Liniers, 10 Jul 1972, *A. Schinini 4901* (CTES); 52 km E de Eldorado, RP 17, 20 Jan 1973, *A. Schinini & A. Fernández 5952* (CONC, CORD fragment, CTES, MO, Z); RP 17, 80 km E de Eldorado, 22 Jan 1973, *A. Schinini & A. Fernández 5967* (CTES); J.J. Lanusse, Tribu Guaraní, 24 May 1973, *A. Schinini & A. Fernández 6058* (CTES, MO, RB); ruta 15, junto a la casa del guardaparques, 26°54'S, 54°12'W, 7 Sep 1994, *A. Schinini et al. 28711* (CTES); límite del predio Guaraní con el arroyo Paraíso, 8 Sep 1994, *A. Schinini et al. 28756* (CTES); Santa Ana, 27°21'2.7''S, 55°35'6.7''W, 118 m, 25 Oct 2006, *A. Schinini 36777* (CTES); cerca del Puerto, 16 Feb 1948, *A.F. Schulz 7155* (LIL); Santo Pipó, 21 Feb 1948, *A.G. Schulz 7204* (CTES, LIL); Cerro San Pedro, 27 Jun 1946, *G.J. Schwarz 2875* (B, LIL, W); Makako, 5 Jul 1946, *G.J. Schwarz 2938* & *2939* (MO, P); Cerro Haselback, 12 Jul 1946, *G.J. Schwarz 2986* (LIL, S); Corpus, 20 Sep 1946, *G.J. Schwarz 3414* (CORD, LIL); San Javier, Alba Posse, 30 Jan 1947, *G.J. Schwarz 4023* (CORD, LIL, P, W); Arroyo Pindaití, 21 Feb 1947, *G.J. Schwarz 4160* (LIL, W); Soberbio, 19 Mar 1947, *G.J. Schwarz 4389* (MO, P); Oberá, 13 Apr 1947, *G.J. Schwarz 4458* (MO, P); Santo Pipó, 3 Jul 1947, *G.J. Schwarz 4690* (LIL, P); Gob. Roca, 16 Apr 1948, *G.J. Schwarz 5763* (LIL); Km 101, ruta 12, 20 May 1948, *G.J. Schwarz 5991* (LIL, W); Puerto Delicia, Km 23, 13 Dec 1948, *G.J. Schwarz 6900* (CORD, CTES, LIL); Aº Uruguay, 17 Dec 1948, *G.J. Schwarz 7037 & 7038* (CORD, LIL); Puerto Mineral, 15 Mar 1949, *G.J. Schwarz 7589* (LIL); Arroyo Cuña-pirú, 20 Apr 1949, *G.J. Schwarz 7755* (LIL); Salto Tabaý, 18 Jun 1949, *G.J. Schwarz 7882* (CORD, LIL); San Juan, 28 May 1947, *E. Schwindt 305* (LIL); Tabay, 29 Oct 1948, *E. Schwindt 947* (G, P); Santa Teresa, 160 m, 12 Jun 1949, *E. Schwindt 1871* (LIL); Santa Teresa, 5 Jul 1949, *E. Schwindt 1911* (LIL); Santa Teresa, 13 Jul 1949, *E. Schwindt 1948* (G, P); cercanías Pirayguazú (arroyo), 15 Jul 1949, *E. Schwindt 1966* (LIL); Campo Viera, 4 May 1950, *E. Schwindt 4217* (CORD, LIL); Campo Grande, 235 m, 9 May 1950, *E. Schwindt 4310* (LIL); Salto Encantado, 24 Jun 1950, *E. Schwindt 4581* (CORD, LIL); Oberá (Colonia), 21 Jul 1950, *E. Schwindt 4790* (CORD, LIL); Arroyo Urugua-í y ruta 12, selva desde el puente hasta 3 km sobre la ruta 12 rumbo a Puerto Iguazú, 29 May 1987, *R. Subils & E. Moscone 4133* (CORD); rumbo a Bosetti, a ± 5 km del límite del Parque Nacional, 29 May 1987, *R. Subils & E. Moscone 4154* (CORD); Santa Ana, alrededores de la curtiembre, próxima al puerto, 14 Jun 1988, *R. Subils & E. Moscone 4271* (CORD, NY);

Predio Guaraní, ruta 15 y ayo. Yaguareté-ruzú, 26°54'S, 54°12'W, *S. Tressens et al. 4694* (CTES); Predio Guarani, Ayo. Caramelito y limite con el Predio del Instituto de Prevision Social, 26°54'59"S, 54°12'18"W, 17 Mar 1994, *S. Tressens et al. 4977* (CTES, MO); Predio Guaraní, 1 Dec 1994, *S. Tressens et al. 5131* (CTES); Predio Guaraní, Tramo I, 7 May 1997, *S. Tressens et al. 5802* (CTES); Predio Guaraní, Picada hacia el ayo. Paraíso, cruzando el ayo. Itapyrú, 26º54-59' S, 54º12-18'W, 8 May 1997, *S. Tressens et al. 5881* (CTES, GH, MEXU, NY); Arroyo Ñacanguazú, col. Ñacanguazú, 27º5'S, 55º25'W, 24 Sep 1993, *R. Vanni & A. Schinini 3057* (CTES, GH, MO); PN Iguazú, Sendero Jacaratia, 3 Apr 1997, *R. Vanni et al. 3945* (CTES); ruta Nac. 101, entre Piñalito y Deseado, 390 m, 3 Mar 1995, *F. Zuloaga et al. 5171* (MO, SI); Parque Prov. Salto Encantado, Salto Encantado, picada a la base, 390 m, 12 Apr 1996, *F. Zuloaga et al. 5431* (NY, SI); RP 21, de Paraíso a Moconá, 26º50'S, 54º08'W, 430 m, 27 Mar 1998, *F. Zuloaga et al. 6722* (CORD, MO, SI).

BRAZIL. **Minas Gerais:** Caldas, estrada para Pedra Branca, a 1,2 km da trevo da sa da de Pocinhos do Rio Verde para a Cachoeira Cascata, 21º56'42"S, 46º24'59"W, 1050 m, 16 Mar 2011, *L. Bianchetti et al. 1552* (CEN); area rural Pedra Branca, 1300 m, *V.W.D. Casali s.n.* (CEN 60625); Poços de Caldas, Morro das Camisinhas, próximo a Estação Ferroviária, 21°50'20''S, 46°33'53''W, 7 Mar 1983, *H.F. Leitão et al. 1997* (BHCB); Cidade de Caldas, 6 Jan 1860, *A.F. Regnell Ser. III, 1001* (CORD fragment, UPS, US); same locality, 11 Mar 1860, *A.F. Regnell Ser. III, 1001* (CORD fragment, S, UPS, US); Río Verde, 7 Apr 1868, *A.F. Regnell Ser. III, 1001* (CORD fragment, UPS); Mun. Lagoa Santa, Mar 1866, *E. Warming s.n.* (C); **Paraná**: Querência do Norte, Caiuá-Ilha Grande, RPPN Fazenda da Mata, 30 Sep 2008, *A.M.D. Amancio et al. 188* (MBM); arredores Turvo, 25º2'16"S, 51º31'27" W, 989 m, 13 Nov 2009, *M.G. Caxambu & E.L. Siqueira 2890* (HCF, MBM); Guaraqueçaba, Río Serra Negra, 25º11'22"S, 48º24'58"W, 15 m, 25 Mar 2014, *M.G. Caxambu et al. 5146* (HCF); RPPN Hilva Jandrey Marques, 23º53'27"S, 51º58'44" W, 374 m, 5 Dec 2014, *M.G. Caxambu et al. 5902* (BHCB, HCF); Lobato, Fazenda Remanso Irmãos Ferraz, 21 Jul 1962, *J. Corrêa Gomes & F. Mattos 1029* (RB); 9 km S of Jardinopolis along the road to Medianeira, 300 m, 13 Mar 1976, *G. Davidse & W. D'Arcy 11227* (MO, SP); Tibagi, Faz. Barra Grande, 11 Nov 1999, *M.C. Dias et al. s.n.* (BHCB 53459, CEN 35136, FUEL 26687); Curitiba, Parque Barigui, 13 Dec 1996, *V.A.O. Dittrich & C. Kozera 298* (BHCB, UPCB); Ponta Grossa, 18 Jan 1909, *P. Dusén 7613* (GH, NY, S); same locality, 28 Feb 1909, *P. Dusén 7825* (F, GH, NY, S); Jaguarinhyva, 720 m, 13 Jan 1915, *P. Dusén 16337* (GH, S); Foz do Iguaçú, 19 May 1949, *J. Falcão 190* (CTES, LIL, RB); Serra do Benjamin, 2 May 1957, *G. Hatschbach 3952* (MBM, US); Guarapuava, Palmeirinha, 22 Oct 1960, *G. Hatschbach 7352* (CTES, MBM, RB, US); Parque Nacional Foz do Iguaçú, 18 Apr 1965, *G. Hatschbach 12593* (US); Passo do Pupo, 5 Dec 1990, *G. Hatschbach 18030* (C, CORD, CTES, MBM, Z); Imbituva, Rod. BR-277, 21 Jan 1968, *G. Hatschbach 18412* (MBM); Cataratas del Iguazú, unos 200 m al sur del Hotel, 25 Jan 1970, *A.T. Hunziker 20232* (CORD); Parque Nacional do Iguaçú, en el km 20 de la ruta de entrada al Parque, 25º05'S, 53º36'W, 160 m, 18 Apr 1985, *A.T. Hunziker 24992* (CORD, MO); Parque Nacional Iguaçú, a ca. 30 km al sur de Medianeira, rumbo a Capanema, 285 m, 19 Apr 1985, *A.T. Hunziker et al. 24993* (CORD); sitio do Macanha, 6 Nov 1977, *N. Imaguive 5276* (MBM); Jaguariahyva, 15 May 1914, *G. Jönsson 330 a* (GH, S); Parque Barigui, 23 Apr 1996, *C. Kozera et al. 128* (BHCB); Parque Iguaçú, 27 Dec 1979, *R. Kummrow 1307* (CORD, MBM); Porto Amazonas, Rod. BR 277, 18 Nov 1983, *R. Kummrow 2449* (ALCB, CESJ, MBM, NY, RB); Pinhão, Rio Jordão, 8 Mar 1996, *P.*

Labiak & S.R. Ziller 403 (EFC, MBM); Cianorte, Reserva florestal do CMNP, ca. 30 km E of Cianorte, ca. 300 m.s.m, 16 Mar 1966, *J.C. Lindeman & J.H. de Haas 578* (CORD, MBM); Matelândia, Río Floriano, 1 km from the river, 3 Dec 1966, *J.C. Lindeman & J.H. de Haas 3581*(CORD, MBM, NY, RB); Pinhalzinho, 20 Mar 1967, *J.C. Lindeman & J.H. de Hass 5029* (CTES, K, NY); Parque Nacional do Iguaçú, en el km 20 de la ruta de entrada al Parque,, 18 Apr 1985, *E. Lleras Pérez et al. 1947* (CEN, RB); a ca. 30 km al sur de Medianeira, rumbo a Capanema, 19 Apr 1985, *E. Lleras Pérez et al. 1956* (CEN); Piraí do Sul, em campo na beira da rodovia para Curutiba, 8 Oct 2005, *H. Lorenzi et al. 5656* (BHCB, IAC); Faz. Palmeirinha, 25º19'14,5''S, 51º33'05.2''W, 1010 m, 28 Nov 2003, *L. Mentz et al. 290* (CORD, ICN); Parque Estadual Villa Rica do Espírito Santo, 23º55'S, 51º58'W, Apr 1995, *S.B. Mikich s.n.* (NY, UPCB 33190); Londrina, Santa Helena, 7 Feb 1975, *T.M. Pedersen 10996* (CORD, CTES); a 5 km de Laranjeiras do Sul, em direção a Cascavel, BR 277, 25º22'S, 52º28'W, 900 m, 3 Nov 1989, *G. Pedralli et al. 3045* (CEN, MO, NY, RB); Parque Nacional Iguaçú, 14 Feb 1960, *E. Pereira 5305* (CORD, US); Campo Magro, Morro da Palha, 22 Jan 2002, *J.M. Silva et al. 3559* (MBM); Castro, Río Cunhaporanga, Nov. 1988, *S.M. Silva & R.M. Britez 1714* (MBM); 5 km de Sengés em direção a Itararé, estrada para Fazenda Rio Bonito/Transfada, ca. 17 km da entrada, 13 Feb 1995, *J.P. Souza et al. 30* (RB); Prudentópolis, 25º24'33.3''S, 50º46' 31.9''W, 1 Dec 1995, *J.R. Stehmann & A. Ippolito 1694* (BHCB, UEC); Río Branco do Sul, Caverna de Bromado, 26 Jun 1996, *A.C. Svolenski & G. Tiepolo 236* (EFC, MBM); RPPN Moreira Sales, 7 Feb 2014, *F.Y. Tanaka et al. s.n.* (HCF); Quatro Barras, 25°22'47''S, 49°02'16''W, 970 m, 12 Dec 2009, *V. Thode 298* (BHCB); Fazenda Santa Helena, 4 Sep 1986, *A.O.S. Vieira s.n.* (RB 329247); Parque Nacional Iguaçú, 7 Feb 1965, *S. Vogel 573* (US); **Rio Grande do Sul**: São Jerônimo, Pólo Carboquímico, Porto do Conde, próximo à Fazenda do Conce, 30 Mar 1982, *M.L. Abruzzi 576* (CTES, F, HAS); Arraio da Tigre, Barragem da Itaúbe, 12 Apr 1978, *O. Bueno et al. 628* (CORD fragment, CTES, F); Neu-Württemberg, 450 m, 10 Oct 1905, *A. Bornmüller 585* (C, G, GH, U); Vale Real, 15 Oct 1998, *S. Diesel 1655* (US); Jaraguá do Sul, Garibaldi, 26º33'08''S, 49º10'33.40''W, 180 m, 26 Jan 2010, *S. Dreveck & F.E. Carneiro 1620* (BHCB, FURB); Montenegro, S. Salvador, 2 Oct 1945, *S. Friedrichs s.n.* (LIL, PACA 032925); Parecí Novo, 12 Feb 1945, *E. Henz s.n.* (LIL 156196, PACA026608) & 15 Oct 1945, *E. Henz s.n.* (LIL, PACA032709); São Leopoldo, São Leopoldo, 13 Oct 1946, *E. Henz s.n.* (PACA 36963, SI 024380); Dos Irmãos, 10 Oct 1946, *E. Henz 35360* (SI); Bom Principio, cerca del puente sobre el Rio Cai (ruta RS-122, km 22), a 9 km de Feliz, 70 m, 29º29'S, 51º21'W, 22 Apr 1985, *A.T. Hunziker 24995* (CORD, MO); Vale do Sol, Linha XV de Novembro, 13 Oct 1992, *J.A. Jarenkow 2158* (FLOR, MBM, PEL); Caixas do Sul, Criúva, 780 m, 29 Jan 2002, *A. Kegler 1256* (BHCB, HAMAB, HUCS); Ana Rech, 780 m, 17 Mar 2002, 17 Mar 2002, *A. Kegler 1354* (BHCB, HAMAB, HUCS); Fazenda Souza, 780 m, 28 Jan 2003, *A. Kegler 1426* (BHCB, ICN); Villa Oliva, 780 m, 17 Jan 2003, *A. Kegler 1565* (HUCS, US); Salvador do Sul, São Salvador, Dec 1941, *J.E. Leite 745* (NY); Seberi, Km 76 da BR-386, 42 km ao sul de F. Westphalen, 500 m, 27º44'S, 53º16'W, 21 Apr 1985, *E. Lleras Pérez et al. 1973 a* & *1974* (CEN, CORD); Sarandí, km 135 da BR-386, 300 m ao Sul da Patrhula Rodoviaria, 28º04'S, 52º55'W, 550 m, 21 Apr 1985, *E. Lleras Pérez et al. 1975* (CEN, CORD); Soledade, Km 228 da BR-386, 5 km ao Norte do arroio Farpado, 28º50' S, 52º29'W, 540 m, 21 Apr 1985, *E. Lleras Pérez et al. 1976* (CEN, CORD); cerca del puente sobre el Rio Cai (ruta RS-122, km 22), a 9 km de Feliz, 22 Apr 1985, *E. Lleras Pérez et al. 1981* (CEN, CORD, RB, SP); São Lorenço, ca. 4 km, caminho para Ruinas, 28º25'26''S,

54º42'15''W, 26 Feb 2010, *E. Melo et al. 7813* (HUEFS); Canela, 4 Feb 1986, *H. Neubert 51* (B); São Francisco de Paula, 4 Mar 1986, *H. Neubert 216* (B); Bento Gonçalvez, Mato da Univ. Federale Rio Grande do Sul, 10 Nov 1980, *G. Pedralli 87* (ICN); Vacaria, proximidade do antigo Encanados, 28º6'7" S, 50º52'43"W, 670 m, 3 Sept 2006, *G. Pereira-Silva et al. 10880* (CEN); margem esquerda do lago da UHE Barra Grande, 700 m, 11 Jan 2007, *G. Pereira-Silva et al. 11175* (CEN); Augusto Pestana, 6 Sep 1953, *Pivetta 938* (HRCB, PACA); a 30 km de Cruz Alta, hacia Ijuí, 3 Feb 1971, *M.L. Porto & P.L.Oliveira s.n.* (ICN 00033440); São Sebastião do Caí, Alto Feliz, 5 Mar 1933, *B. Rambo s.n.* (LIL, PACA 000331); Caí, Mariquinhas, 26 Jul 1933, *B. Rambo s.n.* (LIL, PACA 000634, W); Passo do Inferno, 12 Feb 1941; *B. Rambo 4880* (LIL); Vila Oliva, 6 Jan 1946, *B. Rambo s.n.* (LIL, PACA 031114, W); Bom Jesus, Faz. Bernardo Velho, 1000 m, 13 Jan 1947, *B. Rambo s.n.* (LIL, PACA 34642, S, SI 024379); Ferrabraz, 12 Jan 1949, *B. Rambo s.n.* (B, CORD, LIL, PACA 39953); Farroupilha, Santa Rita, 29 Jan 1949, *B. Rambo s.n.* (B, LIL, PACA 40352); Ivotí, pr. S. Leopoldo, 23 Jun 1949, *B. Rambo* s.n. (LIL, PACA 42101); Pr. Caí, 17 Jul 1949, *B. Rambo s.n.* (C, LIL, PACA 042581); Ivotí, pr. S. Leopoldo, 22 Aug 1949, *B. Rambo s.n.* (C, L, LIL, PACA 43008 & 43018); prope Taquara, 2 Sep 1949, *B. Rambo s.n.* (CORD, LIL, PACA 43200 & 43238); S. Francisco de Paula, in araucarieto, 900 m, 18 Dec 1949, *B. Rambo s.n.* (LIL, PACA 044827); Sta. Rita, 700 m, 7 Feb 1950, *B. Rambo s.n.* (LIL, PACA 45758); Hamburgo, Travessão, 25 Jun 1949, *B. Rambo s.n.* (CORD, LIL, PACA 42165); prope Novo Hamburgo, 12 Aug 1949, *B. Rambo s.n.* (LIL, PACA 42911); Butterberg, 22 May 1950, *B. Rambo s.n.* (LIL, PACA 047120) & 13 Nov 1950, *B. Rambo s.n.* (B, CORD, LIL, PACA 049133); Parecí, 26 Nov 1950, *B. Rambo s.n.* (CTES, PACA 049214); Osorio, Morro Grande, 10 Jan 1952, *B. Rambo s.n.* (PACA 051797, S, US 2102206); Viadutos, 13 Oct 1971, *E. Santos et al. 2869* (CTES, R); Linha Julio de Castilhos, 8 Sep 1949, *A. Sehnem 3824* (SI) & *A. Sehnem 3828* (F, INPA, NY, US); Linha Bonita, 400 m, 19 Oct 1949, *A. Sehnem 3927* (NY, SI, US); Igrejinha, Nova Aurora, acceso secundario, 11 Apr 2005, *E.L.C. Soares 83* & *84* (ICN, RB, NY); Josafá, *M. Sobral 2989* (ICN); Candelaria, Cerro Botucaraí, Aug 1986, *M. Sobral et al. 5161* (ICN, MBM, NY, SP, UEC); Derrubadas, Parque Estadual Do Turvo, estrada para Porto García, May 1995, *M. Sobral & I.S. Almeida 7926* (F, ICN, MBM, SP); subida da trilha Morro do Forno-Josafá, 500-600 m, Dec 1995, *M. Sobral & J.A. Jarenkow 8021* (F, ICN, MBM, PEL, SP); Barra do Ouro, Estrada para Riozinho, 2 Nov 1994, *J.R. Stehmann et al. 1520* (BHCB, UEC); São Martinho, Agua Negra, Cerro de São Martinho, 31 Apr 1947, *J. Vidal 01490* (CTES, R); Carapina, 950 m, 21 Feb 1987, *V. Wasum s.n.* (HUCS 2494 & 2531, US); José Velho, 750 m, 26 Nov 2000, *R. Wasum 783* (HUCS, US); **Santa Catarina:** Curitibanos, debajo del Puente Río Marombas (del lado izquierdo) viniendo desde Curitibanos hacia Campos Novos, km 270/271, 27°29'33''S, 50°58'22''W, 920 m, 25 Mar 2004, *G.E. Barboza et al. 905* (CORD); São Cristovão do Sul, próximo a Unidade Penitenciária, 27º16'11''S, 50º24'1''W, 993 m, 25 Feb 2008, *S. Dreveck et al. 268* (ESA, FUEL, FURB); Lageado Cruzeiro, 27º8'8"S, 51º23'22"W, 762 m, 20 Nov 2008, *S. Dreveck et al. 403* (BHCB, FURB); São Joaquim, Vila Luizinho, 1132 m, 10 Mar 2014, *G. Felitto & V. Ariati 804* (ICN, LUSC, MBM, SMDB); Ponte Serrada, 100 m, *P. Floss s.n.* (BHCB 128311); Uribici, Cascata do Avencal, 28º02'42''S, 49º37'03''W, 1184 m, 15 Jan 2013, *L.A. Funez & A.E. Zermiani 1394* (FURB, RB); Major Vieira, Rio Novo, 26º33'5" S, 50º18'46"W, 861 m, 23 Nov 2007, *A.L. de Gasper et al. 1018* (FURB); Rio das Antas, Retiro Saudoso, 26º54'33''S, 51º1'58''W, 970 m, 23 Jan 2008, *A.L. Gasper et al. 1363* (ESA, FURB); Caçador, Serra Azul, 26º43'55''S, 51º07'08''W, 1348 m, 26 Jan 2008, *A.L.*

de Gasper 1394 (FURB); fragmento de FOM a cerca de 8 km de distancia de Capão Alto, no sentido do rio Pelotas, 27º57'20"S, 50º32'51"W, 958 m, 10 Feb 2006, *E. de S. Gomes Guarino et al. 978* (CEN); Río Canoas, São João, 900 m, 17 Mar 1995, *G. Hatschbach & O. Ribas 61697* (BHCB, MBM); Rod. SC-430, 10 km S de Vaca Gorda, 7 Dec 2000, *G. Hatschbach et al. 71589* (MBM); Estrada Paraiso-São Miguel d'Oeste, 2 km ao sul de Paraiso, 20 Apr 1985, *A.T. Hunziker 24994* (CORD); Lajes, Encruzilhada, 900 m, 5 Dec 1962, *R.M. Klein 3190* (B, CORD, HBR, US); Mondaí, 250 m, 28 Aug 1964, *R.M. Klein 5628* (CORD, HBR, FLOR, US); Florianópolis, Isla de Santa Catarina, Morro Costa da Lagoa, 490 m, 18 Dec 1968, *R.M. Klein 8050* (RB, US); Rio do Campo, Tamanduá, 26º54'31''S, 50º07'45''W, 733 m, 25 Feb 2010, *A. Korte & A. Kniess 2051* (BHCB, FUEL, FURB, SAMES); Morro dos Conventos, 26 Nov 1980, *A. Krapovickas & A. Schinini 36971* (C, CTES, MBM); Estrada Paraiso-São Miguel d'Oeste, 2 km ao sul de Paraiso, 26º38'S, 53º40'W, 450 m, 20 Apr 1985, *E. Lleras Pérez et al. 1957* & *1961* (CEN); Estrada Ipora-Mondai, 3 km ao suleste de Ipora, 27º00'S, 53º32'W, 450 m, 20 apr 1985, *E. Lleras Pérez et al. 1964* (CEN, CORD); Estrada Ipora-Mondai, 12 km suleste de Ipora, 27º04'S, 53º28'W, 485 m, 20 Apr 1985, *E. Lleras Pérez et al. 1970* (CEN); Praia Grande, descendo a Serra de Itaimbezinho a Praia Grande a 13 km do Resturante do IBDF (P. N. Aparados da Serra), 630 m, 29º10'S, 50º04'W, 630 m, *E. Lleras Pérez et al. 1989* (CEN); Puente del Río Marombas, por ruta BR 470 rumbo Curitibanos-Campos Novos, 850 m, 25 Nov 2003, *L. Mentz et al. 278* (CORD, ICN); Anita Garibaldi, Santa Rosa, 27º37'42''S, 51º07'14''W, 854, 8 Jan 2016, *A.A. de Oliveira 2881* (CRI, FURB, RB); Ibiam, Santo Copéuro, 27º10'53''S, 51º17'58''W, 788 m, 27 Jan 2016, *A.A. de Oliveira 2911* (FURB, RB); Serra Azul, 26º43'55''S, 51º06'32''W, 1348 m, 29 Jan 2016, *A.A. de Oliveira 2916* (FURB); Campo Belo do Sul, Fazenda Gateados, 27º 59' 20"S, 50º48'13"W, 900 m, 3 May 2005, *G. Pereira-Silva 9995* (CEN, CNMT); Capão Alto, Estrada de acesso ao encanadinho, ca. 1 km a oeste da BR 116, próximo ao Passo do Socorro, 28º11'44''S, 50º45'13''W, 700 m, 16 Jan 2007, *G. Pereira-Silva et al. 11207* (CEN); Nova Teutonia, 27 Nov 1943, *F. Plaumann 229* (CTES, RB); Alto Uruguay, 15 Feb 1934, *B. Rambo s.n.* (LIL, PACA 001176); Itapiranga, 7 Oct 1957, *B. Rambo s.n.* (B, PACA 061246); Bom Retiro, Riosinho, 1000 m, 24 Dec 1948, *R. Reitz 2776* (HBR, LIL, UC, S, US); Anitápolis, Serrinha, 30 Dec 1951, *R. Reitz 4543* (CORD, HBR, FLOR, NY, US); São José, Serra da Boa Vista, 1200 m, 4 Feb 1953, *R. Reitz 5484* (HBR, US); Lauro Müller-Urussanga, Pinhal da Companhia, 300 m, 25 Oct 1958, *R. Reitz* & *R.M. Klein 7542* (HBR, US); Rio do Bugre, 800 m, 8 Jan 1962, *R. Reitz & R.M. Klein 11724* (HBR, US); Canoinhas, Rio dos Pardos, 750 m, 25 Feb 1962, *R. Reitz & R.M. Klein 12487* (HBR, FLOR, US); Morro do Pinheiro Sêco, 950 m, 18 Dec 1962, *R. Reitz* & *R.M. Klein 14102* (CORD, FLOR, HBR, NY, US); Guaraciaba, São Luis, 700 m, 3 Jan 1964, *R. Reitz & R.M. Klein 16945* (CORD, HBR, US); Cerro Negro, Fazenda do Sr. J. Pedro do Amaral Rodrigues, 27º54'14''S, 51º02'32''W, 787 m, 6 Aug 2005, *A.A. Santos et al. 2663* (CEN) & 5 Dec 2005, *A.A. Santos et al. 2829* (CEN); Santa Terezinha, baía do Itajaí, Rio Hercílio, 26º40'56''S, 49º50'19''W, 200 m, 23 Jan 2016, *P. Schwirkowski 1519* (CRI, FURB); Parque Municipal Lagoa do Peri, 27°43'03''S, 48°31'39''W, 226 m, 10 Mar 2010, *A. Silva-Santos et al. 1990* (BHCB); Canoinhas, bog and pinheiral west of Canoinhas on the road to Pôrto União, 750 m, 17 Jul 1956, *L.B. Smith & R. Reitz 8603* (CORD, HBR); Pinheiral by new airport east of Pôrto União, ca. 750 m, 19 Dec 1956, *L.B. Smith & R. Reitz 8833* (CORD, HBR); Treinta-tres, 33 km west of Caçador, 900-1000 m, 23 Jul 1956, *L.B. Smith & R.M. Klein 9108* (HBR, US); Santo Antonio, near Passo de Socorro (Estrada de Rodagem Federal km 67-71, S of Lajes),

800-900 m, 14 Jan 1957, *L.B. Smith* & *R.M. Klein 9968* (CORD, HBR, R); Itapiranga, 4 km west of Popí, on the road to Sant'Antonio, 200-350 m, 24 Feb 1957, *L.B. Smith et al. 11764* (HBR, ICN, US); Rio Peperi-guaçú, Peperi, ca. 26º32'S, 53º44'W, 300-600 m, 13 Nov 1964, *L.B. Smith & R.M. Klein 13238* (NY, P, R, US) & *13240* (GH, NY, R, UC, US); Mata Borda da Mata, 29°10'15''S, 49°58'50''W, 118 m, 6 Nov 2009, *V. Thode 277* (BHCB); Campos Novos, Florão da Serra, 27º19'12''S, 51º31'33''W, 585 m, 19 Nov 2008, *M. Verdi et al. 961* (BHCB, FURB); Santa Rosa, 27º37'41''S, 51º07'14''W, 854 m, 20 Jan 2009, *M. Verdi et al. 1321* (BHCB, FURB); São Francisco, 27º37'45''S, 51º15'37''W, 707 m, 19 Mar 2009, *M. Verdi et al. 2*640 (BHCB, FURB); São Bento, 28º37'12''S, 49º35'24''W, 50 m, 5 Nov 2009, *M. Verdi et al. 3003* (FURB); Morro Grande, Três Barras, 28º42'36''S, 49º46'12''W, 311 m, 23 Nov 2009, *M. Verdi et al. 3103* (FURB); **São Paulo**: Riberão Grande, Parque Estadual Intervales, trilha da caçadinha, 24°16'S, 48°25'W, 780 m, 20 Apr 2003, *D.F. Araki et al. 116* (BHCB); a 19,8 km al N de Monteiro Lobato, por SP 50, rumbo a Campos do Jordão, 1050 m, 27 Feb 2006, *G.E. Barboza et al. 1657* (BM, CORD, MO, NY, RB); ca. 19 km al N de Monteiro Lobato, rumbo a Campos do Jordao, entre km 142-144, 22º51'01''S, 45º46'16''W, 1022 m, 6 May 2012, *G.E. Barboza et al. 3631* (CORD); de Monteiro Lobato, rumbo a Campos do Jordao, por SP 50, 22°51'14,2''S, 45°46'32,6''W, 1009 m, 4 Apr 2018, *G.E. Barboza & R. Deanna 5021* (CORD); Atibaia, Fazenda Grota Funda, 18 Apr 1988, *L.C. Bernacci et al. 21468* (SJRP); Porto Ferreira, Parque Estadual, 17 Mar 1999, *J.E. Bertoni 400* (BHCB, RB); Amparo, Estrada para Fazenda 20 Palmos, a 3.4 km do entroncamento com a rodovia SP 360, 22º38'01''S, 46º46'11''W, 830 m, 5 Apr 1999, *L. Bianchetti et al. 1533* (CEN); Monte Alegre do Sul, 3 km da saída de Monte Alegre do Sul para Pinhalzinho, 300 m entrando na estrada para Pousada Cachoeira, 22°41'59''S, 46°39'51''W, 770 m, 5 Apr 1999, *L. Bianchetti et al. 1534* (CEN); Campinas, May-Jun 1918, *J. de Campos Novaes 1941* & *2179* (US); Campinas, Estufa Instituto Agronomico, 11 Oct 1938, *A.S. Costa 4882*; Paranavaí, 10 Feb 2004, *C.N.G. Costa et al. 1650* (BHCB); Estação Experimental Ubatuba, 24 Nov 1938, *C. Franco et al. s.n.* (BHCB 76891); Cosmópolis, 652 m, 16 May 2008, *L.C. García 133* (UEC); Fazenda Santa Genebra, 1 Oct 1978, *C. Heiser 7801* (CORD); entre Monteiro Lobato y el Hotel Estancia, ruta SP 50, unos 19 km al N de M. Lobato, 950-1000 m, 30 Apr 1985, *A.T. Hunziker 25013* (BM, CORD, NY); Jundiaí, Estação Experimental do IAC, 5 Apr 1995, *S.L. Jung-Mendacolli et al. 1421* (BHCB, IAC, SP); Monte Alegre, proximidades da Fazenda Santa Isabel, 8 Apr 1943, *M. Kuhlmann 665* (CORD); Rodovia SP-50, 19 km ao norte de Monteiro Lobato, 22º50'S, 45º45'W, 960 m, 30 Apr 1985, *E. Lleras Pérez et al. 2036* & *2037* (CEN, CORD, RB); Araraquara, camino ao Rio Jacaré, 26 Mar 1899, *A. Loefgren 4321* (CORD, SP) & *15430* (US); São José do Rio Pardo, 18 Sept 1919, *A. Loefgren 15427* (US); Reserva do Parque Estadual das Fontes do Ipiranga, 3 Feb 1983, *I.C.C. Macedo 5* (MO, SP); Souzas, mata particular das Três Pontes, 31 Oct 1990, *P.L R. de Moraes et al. 23685* (CORD, UEC); Rio Claro, Horto florestal Navarro de Andrade, 26 Jan 1993, *L. Moura s.n.* (BHCB 126631); without locality, *A.F. Regnell III 1001* (CORD 00087946 fragment, UPS, US 208484); Capivary, 1821-1824, *L. Riedel 1073* (MO); Parque Estadual do Jaraguá, trihla do Mauro, 23°27'11''S, 46°46'07''W, 981 m, *F.M. Souza et al. 1073* (BHCB); Jardim São Luís, Parque Guarapiranga, 6 Nov 1987, *V.C. Souza & M.O. Pedraz 1116* (PMSP); de Monteriro Lobato rumo a Campos do Jordão, pela SP 50, 1009 m, 4 Apr 2018, *J.R. Stehmann et al. 6467* (BHCB); Reserva do Parque Estadual das Fontes do Ipiranga, 26 Mar 1985, *C.B. Toledo et al. 69* (COL, SP); Reserva Ecológica da Serra do Japi, 5 Mar 2005, *J. Vasconcelos Neto 05/15* (UEC); Faz. Santa

Genebra, mata proxima à Cidade Universitaria, 5 Jan 1978, *J. Vasconcelos Neto 6813* (SP); Serra do Japí, 1 May 1989, *J. Vasconcelos Neto 21594* (UEC); in itinere Cerqueira Cesar-"Fazenda bella vista", Jul 1901, *R. v. Wettstein 975* (C, CORD, WU, Z);

PARAGUAY. **Alto Paraná**: Colonia 13 Tujutí, 31 km N Hernandarias, 25º08'S, 54º38'W, 14 Oct 1984, *D. Brunner et al. 913* (MO, PY); Centro Forestal Alto Paraná, 25º28'S, 54º42'W, 16 Apr 1986, *D. Brunner 1804* (PY); Monumento Natural Kuri'y, 1 Sep 2001, *F. González Parini 170* (FCQ); Limoy, 60 km N of Hernandarias, 24º50'S, 54º20'W, 360 m, 15 Dec 1982, *W. Hahn et al. 937* (NY); Ruta Puerto Stroessner-Salto del Guairá, 32 km N de Hernandarias, 275 m, 16 Dec 1982, *A. Schinini 23074* (C, F); Reserva Biológica Itabo, 35 km W Rio Paraná, 25°5'S, 54°5'W, 9 Oct 1990, *A. Schinini & G. Caballero Marmori 26990* (CTES); Puerto Stroessner, *L.C. Stutz 364* (MO); Puerto Stroessner, km 16, Mar 1983, *L.C. Stutz 1348* (G); Puerto Stroessner, km 12, May 1983, *L.C. Stutz 1720* (G, MO); same locality, 21 Dec 1984, *L.C. Stutz 2095* (MO); Hernandarias, Itaipú Forest Nursery, 25º26'S, 54º38'W, 265 m, 11 May 1998, *K.A. Williams et al. 133* (CORD, FCQ); Estancia Río Bonito, Forest III, 25°37'49"S, 54°48'50"W, 26 Dec 1994, *E.M. Zardini 41733* (MO, PY); Estancia Río Bonito, Forest II (around Rio Bonito), 25°37'55"S, 54°48'17"W, 240 m, 30 Aug 1994, *E.M. Zardini 41772* (MO, PY); **Amambay**: Pedro Juan Caballero, 740 m, 14 Feb 1951, *G.J. Schwarz 11868* (CTES, LIL); **Caaguazú**: Distrito de Tojao, 25º14'39''S, 56º10'26''W, 27 Jul 2002, *F. González Parini 1176* (CTES, FCQ); Coronel Oviedo, camino Estancia Morurú, 24 Jan 1951, *T. Rojas 14415* (CORD); **Caazapá**: PN Caaguazú, cuenca del Arroyo Guazú, 21 Jul 1986, *L. Molas 792* (PY); Tavaí, Compañía Toranso, 18 May 1989, *M. Ortiz 1283* (FCQ); San Agustín, Jun 1932, *T. Rojas 6019* (CORD, PY); PN Caaguazú, 26º05'49''S, 55º28'58''W, 450 m, 25 Nov 1997, *E.M. Zardini & A. Benítez 47532* (AS, MEXU, MO); **Canindiyú**: Estación Biológica Mbaracayú, ca. 10 km E of Villa Ygatimí, c. 3 km from field station headquarters, 24º07'53.6''S, 55º29'43.5''W, 180 m, 22 Nov 2003, *L. Bohs & C. Vogt 3167* (FCQ) & *3171* (FCQ, NY); in silva pr. Yeruti, in regione Yerbalium de Maracayu, Dec 1898-1899, *E. Hassler 5742* (BM, G, NY); Reserva Natural del Bosque Mbaracayú, La Morena, 16 Oct 1996, *B. Jiménez & G. Marín 1651* (BM, CTES, MO, PY) & *5893* (BM); Aguara Ñu, Valinotti-cue, 11 Oct 1997, *G. Marín et al. 776* (BM, CTES, PY); Colonia Alborada, 23 Nov 1948, *J.E. Montes 3280* (CORD fragment, SI, US); Reserva Natural del Bosque Mbaracayú, 3.5 km del Puerto Yeui-mi, 2 Dec 1997, *A. Schinini & M. Dematteis 33242* (CTES, F, G, GH, MO, P); Mbaracayú Natural Reserve, 24°08'00"S, 55°31'41"W, 13 Jan 1998, *N. Soria 3890* (CTES, FCQ, MO); Tavai, a 500m de Y-hovy, 26°10'S, 55°17'W, 500 m, 18 May 1989, *E.M. Zardini 47751* (AS, MO); Mbaracayú Natural Reserve, around Jejuí-Mí, 24°07'59"S, 55°31'40"W, 25 May 1999, *E.M. Zardini 50492* (AS, MO); Estancia Tapytá of Shell Forestry Ltd., 26°17'15"S, 55°46'14"W, 16 Dec 1999, *E.M. Zardini 53141* & *53151* (AS, MO); **Guairá**: bords de l'arroyo Guazú, à lést de la Cordillera Villa-Rica, 21 Sep 1874, *B. Balansa 2107* (K, P); Cordillera de Mbocaïtí, 5 Mar 1883, *B. Balansa 4704* (CORD, G, K, P); prope Villarrica, Jan 1905, *É. Hassler 8633* (G); Villarrica, Dec1931, *P. Jörgensen 4370* (A, CAS, F, GH, LP, LIL, MO, S, SI, US); Colonia Independencia, trayecto a Colonia Carlos Pfannl, 25º42'39.5''S, 56º10'0.7''W, 23 Jul 1998, *F. Mereles et al. 7498* (CORD) ; Borja, 15 Dec 1952, *J.E. Montes 16310* (LIL); Costa Jhú Cerro, 24 Jan 1953, *J.E. Montes 16536* (LIL); Cerro Acati, 22º55'S, 56º15'W, 12 Dec 1988, *N. Soria 2901* (FCQ, G, MO); Colonia Independencia, 25º42'S, 56º10'W, 200 m, 7 May 1998, *K.A. Williams et al. 125* (CORD, FCQ); Cordillera de Ybytyruzú, Cerro Acatí, 25°55'S, 56°15'W, 700 m, 17 Feb 1989, *E.M.*

Zardini 11076 (FCQ, MO); **Itapuá**: ruta 1, 15 km NW de Encarnación, 16 Nov 1978, *M.M. Arbo et al. 2038* (CTES); Cordillera San Rafael, Alto Vera, 21 Oct 2001, *F. González Parini et al. 324* (FCQ) ; Natalio 25, 9 May 2003, *F. González Parini et al. 1706* (FCQ); Distrito Alto Vera, Compañía San Rafael, 26º34'S, 55º45'W, 26 Jul 1998, *F. Mereles et al. 7503* (CORD); **Paraguarí**: Parque Nacional Yby cu'í, sendero al Salto Guaraní, 26º03'S, 56º50'W, 15 Jan 1989, *A. Aguayo 75* (MO, PY); Pirayu-hi, entre Villarrica et Paraguari, 11 Feb 1876, *B. Balansa 2110* (G, P); Paraguarí, dans les bois, 7 May 1878, *B. Balansa 2120* (K, P); Macizo Acahay, Compañia Yeguariso, 21 Apr 1994, *B. Benítez B. 585* (MO); Pr. Sapucay, Dec 1885-1895, *É. Hassler 1607* (BM, CORD fragment, G, K, P); Serranía Chararà, 6 Dec 1943, *C. Pavetti & T. Rojas 10716* (AS, CORD); Carapeguá, Calistro, 27 Mar 1919, *T. Rojas 3356* (AS, CORD); Cerro Acahay, 6 Jun 1919, *T. Rojas 3363* (AS, CORD); Macizo Acahay, 26º54'S, 57º09'W, 500 m, 14 Jun 1988, *E.M. Zardini 5771* (MO, PY); Cerro Palacios, 25º25'S, 57º10'W, 250 m, 8 Sep 1988, *E.M. Zardini 7166* & *7200* (FCQ, MO); Administración-Arroyo Corrientes-La Rosada, 26º07'S, 56º53'W, 4 Jun 1989, *E.M. Zardini & R. Velásquez 12460* (MO, PY); Acahay Massif, 25°54'S, 057°09'W, 400 m, 11 Jun 1989, *E.M. Zardini & R. Velásquez 12920* (MO, PY); along tributary of arroyo Minas, 4 km N of Administration-Arroyo Corrientes-La Rosada, 31 Oct 1989, *E.M. Zardini & U. Velásquez 15600* (PY); **San Pedro**: Centro Forestal Capi' ivary, 24º49'S, 55º56'46''W, 1 Dec 2002, *F. González Parini 1533* (CTES, FCQ); Alto Paraguay, Colonia Primavera, 9 Sep 1956, *A.L. Woolston 729* (C, CORD, MO, NY, SI, U, UC, US); Yaguarete Forest Systems (Sustainable Forest Systems site), 23°47'49"S, 56°10'28"W, 31 Jul 1996, *E.M. Zardini 45402* (AS, MO).

Cultivated
ARGENTINA.**Tucumán**: Capital, cult. at Horto Botanico Inst. Lillo, 2 Nov 1954, *A.T. Hunziker 10320* (BM, COL, CORD, MO, NY, P); Instituto M. Lillo (cultivado), 8 May 1987, *E.A. Moscone 148* (CORD); Parque del Inst. M. Lillo, 28 May 1952, *H. Sleumer s.n.* (LIL); cultivado en jardines Inst. M. Lillo, 450 m, 2 Feb 1970, *M. Villa Carenzo 3373* (LIL).

AUSTRIA. **Wien**: cult. HBV (Universität Wien), semillas de origen desconocido (Brasil), 15 Jul 2019, *C. Carrizo García 84* (WU).

18. Capsicum friburgense Bianch. & Barboza
BRAZIL. **Rio de Janeiro**: Mun. Nova Friburgo, RPPN Bacchus, Macaé da Cima, near Nova Friburgo, trilha do Telefone Rio de Janeiro, 1555 m, 29 Apr 2010, *M.F. Agra et al. 7293* (BHCB); RPPN Bacchus, Macaé da Cima, near Nova Friburgo, trilha da Aguada, 22°22'33''S, 42°30'06''W, 29 Apr 2010, *M.F. Agra et al. 7299* (BHCB); rumbo al camino hacia la torre de TV en el cerro Caledonia, a unos 1.3 km del desvío a São Bernardo, 22º20'43''S, 42º35'08''W, 1717 m, 18 Apr 2008, *G.E. Barboza et al. 2048* (CORD, MO); subindo o Morro da Caledônia, a 6.5 km do Camping Club do Brasil (RJ.2), 22°17'S, 42°32'W, 1820 m, 6 Apr 1986, *L. Bianchetti et al. 391* (CEN, CORD); Morro da Caledônia, a 6.6 km do Camping Club do Brasil, 1750 m, 22 May 1992, *L. Bianchetti et al. 1299* (CEN); estrada do Morro da Caledônia, a 600 m do portão de entrada da torre telefônica, na beira da estrada, 22°21'04''S, 42°35'05''W, 1920 m, 21 May 1999, *L. Bianchetti et al. 1565* (CEN); Parque Estadual dos Três Picos, trilha para a Cabeça-do-

Dragão, 22°19’38’’S, 42°43’10’’W, 1803 m, *M.G. Bovin et al. 4229* (RB); Pico Nova Caledônia, 14 Jan 1985, *H.C. de Lima et al. 2526* (BHCB, CEN, F, RB: 3 sheets); Parque Estadual dos Três Picos, Vale dos Deuses, trilha para a Caixa de Fósforo, 22º20’4’’S, 42º44’9’’W, 18 Oct 2018, *F.H. Nadal et al. 28* (RB).

19. Capsicum frutescens L.

BELIZE. Cayo Distr: east of Terra Nova Forest Reserve, W of Valley of Peace, 17º21’N, 88º55’W, 100 m, 6 Jul 1995, *D.E. Atha et al. 1022* (NY); San Antonio village, 17º05’N, 89º02’W, 8 Nov 1988, *R. Arvigo et al. 148* (NY); Nabatunich, near Sukkotz, 17º06’N, 89º00’W, 24 Jan 1990, *M.J. Balick et al. 2272* (NY); San Antonio, 17º05’N, 89º04’W, 25 Jan 1990, *M.J. Balick et al. 2391* (NY); **Corozal** Distr.: Corozal-San Andres road, Jul 1933, *P.H. Lundell 4766* (MICH, NY); **Orange Walk** Distr., Honey Camp, Sep-Dec 1928, *C.L. Lundell 87* (US); **Toledo** Distr.: Columbian Forestry Station, 14 Jul 1971, *K. Cosentino 98 & 99* (F);

BOLIVIA. **Beni**: Prov. Ballivian, Isla de Espiritu, en la zona de influencia del rio Yacuma, 200 m, 13 Apr 1981, *S. Beck 5362* (CORD); Itenez, Huacaraje, 13º31’S, 63º45’W, 11 Apr 1979, *A. Krapovickas & A. Schinini 34819* (CTES); Estación Biológica del Beni, Limoncito, Canchón de Juan Mayer, 14º30'S, 66º37'W, 200 m, 20 Jun 1995, *E. Rivero 437* (CTES); Baurez, 29 Nov-14 Dec 1947, *R. Scolnik & R. Luti 672* (CORD, US); Rurrenabaque, dooryard garden, 14°30’S, 67°30’W, 227 m, 5 Aug 1989, *D.E. Williams 933* (CTES, USZ); **Cochabamba**: Villa Tunari, Yungas, 8 Jan 1958, *A. Krapovickas 8435* (LIL); Entre Ríos, along the highway from Buena Vista to Villa Tunari (carretera nueva Santa Cruz-Cochabamba), 17°11’55’’S, 64°32’10’’W, 795 m, 30 Apr 2007, *M. Nee et al. 55227* (NY, USZ); Villa Tunari, hotel near main Villa Tunari-Cochabamba highway, 16°58’23’’S, 65°24’59’’W, 305 m, 16 May 2008, *M. Nee 55645* (USZ); **La Paz**: A. Iturralde, Parque Nacional y Area Natural de Manejo Integrado Madidi, Chalalan, sector Comunidad San José de Uchupiamonas, 14°14’S, 68°05’W, 450 m, 29 Nov 2004, *A. Araujo-M. et al. 1638* (CTES, USZ); Nor Yungas, canyon of Río Coroico, road from Coroico to Caranavi, 2 km NE of Chorro, ca. 16°01’S, 67°37’W, 900 m, 30 Oct 1984, *M. Nee & Solomon 30265* (UC); Tumupasa, 50 km NW of San Buenaventura on Road to Ixiamas, 14°10’S, 67°55’W, 560 m, 22 Oct 1988, *D. Williams 764* (USZ); **Pando**: Nicolás Suárez, Cobija, 260 m, 17 Sep 1988, *S. Beck 17097* (CORD); **Santa Cruz**: Ichilos, Ocorotú, ca 10 km de Buena Vista, hacia el SE, camino a Guaytú, 5 Dec 2012, *G.E. Barboza et al. 36*65 (CORD); Ñuflo de Chávez, región de Lomerío, en los alrededores de La comunidad El Puquio, 300-500 m, 14 Nov 1994, *M. Del Aguila et al. 662* (USZ); Ascensión de Guarayos, 26 Apr 1977, *A. Krapovickas & A. Schinini 31689* (CTES); embocada Del Carmen, ca. 40 km SSW de Concepción, 16°38’S, 62°26’W, 550 m, 2 May 1977, *A. Krapovickas & A. Schinini 32132* (CTES); Chiquitos, Roboré, 24 Apr 1980, *A. Krapovickas & A. Schinini 36348* (CTES); Ascención de Guarayos, 15º30’S, 62º45’W, 250 m, 20 Apr 1997, *A. Miranda 8* (CORD, NY); Velazco, Estancia Flor de Oro, W side of the Rio Guapore (= Rio Itenez), cult., 13°33’S, 61°00’30’’W, 190 m, 20 Jun 1991, *M. Nee 41131* (USZ); Sara, Buena Vista, 500 m, 25 Feb 1921, *J. Steinbach 5373* (F, GH), same locality, 500 m, 27 Mar 1921, *J. Steinbach 5484* (A).

Brazil. **Acre**: Vila Assis, 180 m, 20 Jul 1995, *P. Núñez V. et al. 16906* (USM); **Amapá:** Macapá, APA do Rio Curiaú, comunidade Curiaú de Dentro, 1 May 2008, *L.A. Pereira et al. 1672* (HAMAB, RB); comunidade Curiaú de Fora, 7 May 2008, *L.A. Pereira et al. 1809* (HAMAB, RB); distr. Fazendinha, Minipolo Hortifruti da Fazendinha, 5 Jul 2008, *L.A. Pereira et al. 1820* (HAMAB, RB); Vila do Trem, Rod. BR 156, km 10, 8 Jul 2008, *L.A. Pereira et al. 1829* (RB); Linha D, Rod. AP 20, km 08, 8 Jul 2008, *L.A. Pereira et al. 1836* (HAMAB, RB); comunidade Curiaú de Fora, 14 Jul 2008, *L.A. Pereira & W.M. Severino 1855* (HAMAB, RB); Santana, Ilha de Santana, 19 Jul 2008, *L.A. Pereira et al. 1865* & *1866* (HAMAB, RB); **Amazonas**: E bank of rio Abacaxis, 4°10'S, 58°41'W, 12 Jul 1983, *S.R. Hill et al. 12996* (F, GH, RB, US); Tefé, 20 Feb 1981, *L. Krieger 34* (BHCB) & 30 Jul 1988, *L. Krieger 22976 & 22981 pp*. (BHCB); Manaus, vicinity of INPA Ecologia campus, 23 Jul 1992, *M. Nee 43027* (NY); **Bahia:** Salvador, feria de São Joaquim, 5 Apr 2009, *G.E. Barboza 2271* (CORD); Jussari, Reserva Particular Parque Natural, entrada a 7.5 km na Rod. Jussari/Palmira, 15°09'16.2''S, 39°31'52.2''W, 6 May 2000, *A.M.V. de Carvalho et al. 6863* (NY, RB); estrada que liga São José da Vitória a Uma, 15°6'37.57''S, 39°16'0.59''W, 108 m, 22 Sep 2008, *L. Giacomin et al. 200* (BHCB, CORD); Ilhéus, area do CEPEC, km 22 da Rodovia Ilhéus/Itabuna, 10 Jun 1981, *J.L. Hage 945* (RB); Vila Sauípe, 10 Jul 2002, E. v*on S. Medeiros et al. 176, 198* & *199* (RB); 11.2 km W of BR 101 & Aurelio Leal on dr to Lage do Banco, 14º20'S, 39º23'W, 3 May 1992, *W.W. Thomas et al. 9080* (NY); **Ceará**: Antonina do Norte, 3 Apr 2015, *F.F.S. Sousa 17* (RB); **Espírito Santo**: Reserva Florestal de Linhares, 14 Apr 1997, *D.A. Folli 2995* (BHCB); Cachoeiro de Itapemirim, estrada para Itabira, 90-110 m, 25 Apr 1972, *D. Sucre 8934* (CORD, RB); **Goiânia:** 15 Dec 1980, *Dalafene 2* (UB); **Goiás**: Vila Buriti, rio Tocantins, 13°32'24''S, 48°08'33'W, 310 m, 28 Nov 2001, *G. Pereira- Silva et al. 5761* (CEN); Niquelândia, rua 7 de Setembro, n° 12, 580 m, 14°04'S, 48°04'W, 6 May 1988, *L.A. Skorupa & J.N. da Silveira 328* (CEN); Padre Bernardo, Taboquinha, 15°18'S, 48°01'W, 23 Apr 1991, *R.F. Vieira 709* (CEN); estrada Jacira-Antiga estrada enre Mincau e o canteiro de obras da Usina, 13°45'S, 48°22'W, 19 Mar 1996, *B.M.T. Walter et al. 3249* (CEN, RB); Colinas do Sul, Reservatório do AHE Serra da Mesa em formação, região do rio Tocantinzinho, 14°17'S, 48°08'W, 450 m, 8 Jul 1997, *B.M.T. Walter et al. 3851* (CEN); **Mato Grosso**: Santo Antonio do Leverger, cerca de 32 km a leste de Cuiabá, na BR-163, 15°42'12''S, 55°46'51''W, 180 m, escapado de cultivo, 3 Jun 1985, *J.F.M. Valls et al. 9014* (CEN); **Mato Grosso do Sul**: Corumbá, Albuquerque, 4 Jun 1998, *I.M. Bortolotto & D.P. Rodrigues B-615* (BHCB); Fazenda Santa Filomena, Nhecolãndia, Pantanal, 19°11'S, 56°43'W, 19 Apr 1990, *A. Pott et al. 5562* (CEN); **Minas Gerais**: Santo Antônio do Itambé, southeastern drainage of Pico de Itambé, about 5 km directly W and N of Santo Antônio do Itambé, 950 m, 9 Feb 1972, *W.R. Anderson et al. 35734* (LP, NY, UB, US, UEC); without locality, 13-V-1985, *Cortese Neto s.n.* (VIC); Belo Horizonte, cult. horto, *T.S.M. Grandi 261* (BHCB); camino a Diamantina, 29 Apr 1961, *Gregory et al. 10508* (LIL); Grão Mogol, 29 Jun 1981, *S.M. Pereira 3219* (BHCB); Belo Horizonte, 7 Apr 1980, *TSMG 261* (BHCB); **Pará**: Estacão Ecologia do Jari, 00º75'S, 52º30'W, 14 Oct 1987, *H.T. Beck et al. 116* (GH); Belem, Mar-May 1929, *B.E. Dahlgren & E. Sella 564* (F, GH); **Paraíba**: João Pessoa, 26 Sep 1978, *M.F. Agra 74* (JPB); **Rio de Janeiro**: Paraty, Praia de Martim de Sá/Reserva Ecológica da Juatinga, 23°19'20''S, 44°24'26''W, 15 Mar 2006, *R. Borges 65* (RB); cult. Chácara de Turiaçu, 25 Jul 1989, *S.L. Gonçalves & M. Koketru 3* (RB); Cerro Pan de Azúcar, 14 Jul 1948, *J.H. Hunziker 2532* (CORD); JBRJ, canteiro 28, 4 Mar 2015, *J.R. Matos 32* (RB); Pedra de Guaratiba, 22°59'30''S, 43°38'59''W, 25 Oct

2000, *R. Patzlaff 15* (RB); **Rio Grande do Sul**: Torres, 29 Jun 2015, *M. D'Avila s.n.* (BHCB 178208); **Rondonia**: Vilhena, 8 Oct 1990, *B.M.T. Walter et al. 570* (CEN); Cerejeiras, Pimenteiras-na cidade; divisa com a Bolivia, in horta de fundo de quintal, 13°28'S, 61°03'W, 230 m, 8 Oct 1990, *B.M.T. Walter et al. 575* (CEN); Cerejeiras, casa de moradores da cidade, Sr. Preto, 22°30'S, 61°03'W, 250 m, 8 Oct 1990, *B.M.T. Walter et al. 581 & 582* (CEN); Porto Velho, P.S.L., próximo a cantina, 9 Apr 2005, *E.M. Ferreira 31* (RB); Porto Velho, Libertade, 9 Apr 2005, *E.M. Ferreira 60* (RB); Porto Velho, Parque Ecológico, 4 Jun 2005, *E.M. Ferreira 64 & 65* (RB); **Santa Catarina**: Mun. Florianópolis, Saco Grande, 10 m, 25 May 1966, *R.M. Klein & A. Bresolin 6447* (US); **São Paulo**: Parque do Estado de São Paulo, 13 Apr 1948, *W. Hoehne s.n.* (BHCB 36775, JPB 24266, NY, SPF 13539); Parque do Estado, Capital, *W. Hoehne 3539* (CORD); Ubatuba, 20 Mar 1981, *Rodrigues et al. 12335* (UEC); **Tocantins**: Itacajá, Reserva indígena Krahó, aldeia Pedra Branca, 9 May 2000, *A. Amaral Santos et al. 721* (CEN); Araguaina, at "Acampamento do Gaúcho", on Belém-Brasilia highway, 7°12'S 48°14'W, 1 Jan 1970, *G. Eiten & L.T. Eiten 10140* (BHCB, UB); Babaculandia, margem esquerda do rio Corrente, 7°14'22''S, 47°45'48''W, 140 m, 21 Apr 2008, *G. Pereira-Silva 13172* (CEN).

COLOMBIA. Amazonas: Corregimiento de Araracuara, margen derecha del Río Caquetá, 13 Sep 1977, *L.E. Aguirre-Galviz 1099* (COL); Puerto Santander, margen derecha del Río Caquetá, resguardo Miraña Bora, Parque Cahuinari, 1º42'20''S, 73º38'56''W, 305 m, *D. Cárdenas et al. 9394* (COAH, COL); margen derecha del Río Miriti, Comunidad Guayabo, 0º58'15.8''S, 70º34'57.2''W, 244 m, *D. Cárdenas et al. 9405* (COAH); Puerto Nuevo, 1º00'30.1''S, 70º27'1.9''W, 305 m, *D. Cárdenas et al. 9412* & *9413* (COAH); Comunidad Puerto Lago, 1º15'44''S, 69º58'37''W, 214 m, *D. Cárdenas et al. 9434* (COAH); Corregimiento La Pedrera, comunidade Camaritawa, 1º11'27''S, 69º35'14''W, 79 m, *D. Cárdenas et al. 9441* (COAH, COL); Puerto Santander, Meta, alrededores de la cas del sr. C. Matapí, 100-200 m, 12 Nov 2000, *S. Castro & I. Matapí 562* (COL); Aduche, Chagra de I. Macuna, 100-200 m, 22 Nov 2000, *S. Castro & N. Andoke 597* (COL); same locality, 24 Nov 2000, *S. Castro & N. Andoke 606* & 608 (COL, HUAZ); Igará-Paraná, Santa María, chagra C. Z+ueche, 28 Feb 1988, *C.I. Henao 167* (COL); Caño Anduche, chagra de I. Andoke, 18 Mar 1982, *C. La Rotta 147* (COAH); Santa Isabel, chagra de T. Miraña, 12 Jul 1982, *C. La Rotta 273* (COAH); Puerto Ñariño, 24 Jul 1965, *G. Lozano C. et al. 575* (COL); Corregimiento La Chorrera, vereda Cordillera, bajo Río Igaraparaná, 100 m, 30 Sep 1992, *O. Montenegro 137* (COL); Puerto Narino, mouth of Río Loretoyacu, ca. 100 m, 8 May 1972, *T. Plowman 3205* (COL, GH); Siete de Agosto, *A. Posada 2603* (COAH); Naranjales, *A. Posadas 2606* (COAH); Río Igara-Parana (afl. Rio Putumayo), territoire des indiens Witoto Jitomagaro, 11 Jun 1974, *C. Sastre 3298* (COL); Puerto Leguizamo, Río Putumayo, carretera entre Caucayá (Puerto Leguizamo) y La Tagua, 225 m, 17 May 1942, *E.E. Schultes 3794* (COL); Leticia, km 6, *M. Torres & F. Rodríguez 2020* (COAH); margen izquierda del Igara (Puerto Rico), 1º42'47''N, 72º34'08.9''W, 162 m, *M. Torres et al. 4015* (COAH); margen derecha del Río Putumayo, Tarapacá, 2º53'17.3''N, 69º44'37.3''W, 33 m, *M. Torres et al. 4055* (COAH); Araracuara, sede Experimental Hortalizas, 14 Sep 1984, *M.E. Vásquez 002* (COAH); **Antioquia**: Medellín, valle de Aburrá, 1500 m, 24 Oct 1970, *D.D. Soejarto et al. 2539* (COL, GH); **Arauca**: Arauca, inmediaciones de la UN de Colombia, hacienda El Cairo, carretera Arauca-Tame, km 9, 200-300 m, 12 Jun 2003, *D. Giraldo-Cañas et al. 3485* (COL); **Atlántico**: cerca de Usiacurí, 2 Jan 1949, *J. Araque Molina & F. Barkley 19 A053* (MEDEL); **Bolívar**:

Turbaco, fundación Jardín Botánico “G. Piñeres”, 28 Aug 1980, *J. Espina 571* (COL); fundación Jardín Botánico “G. Piñeres”, 28 Aug 1980, *J. Espina 586* (COL); vicinity of Cartagena, 1919, *Bro. Heriberto 15 & 231* (US); **Boyacá**: carretera Santa María-Bogotá, cercanias del embalse de Chivor, 1200-1300 m, 4º55’04’’N, 73º18’51’W, 26 Apr 2005, *J. Betancur et al. 11615* (COL); **Caquetá**: Solano, Puerto Arturo, Huerta C. Huirillo, 0º33’45’’S, 72º24’5.9’’W, 335 m, *D. Cárdenas et al. 9300* (COL); Inspección Araracuara, Shuchichi, 3º62’28’’S, 72º28’27’’W, 244 m, *D. Cárdenas et al. 9307* (COAH, COL); resguardo Aduche, Chorro de la Sardina, 0º35’17’’S, 72º13’32’’W, 366 m, *D. Cárdenas 9325* (COAH, COL); Puerto Banano, abajo del Chorro de la Sardina, 0º34’52’’S, 72º12’46’’W, 311 m, *D. Cárdenas 9328* (COAH); Comunidad Perey, resguardo Aduche, chagra C. López, 0º47’18’’S, 72º1’39’’W, 427 m, *D. Cárdenas et al. 9354* (COAH, COL); margen derecha del Río Caquetá, en frente a Luis Angel, 00º56’48’’S, 71º36’37’’W, 244 m, *D. Cárdenas et al. 9362* (COL); margen derecha del Río Caquetá, Resguardo Miraña Bora, Parque Cahuinarí, Las Palmas, 01º42’20’’S, 73º38’56’’W, 305 m, *D. Cárdenas et al. 9384* (COAH); Florencia, vereda Bajo Caldas, Finca La Turbina, 1º39’07’’N, 75º39’04’’W, 688 m, 12 Feb 2002, *J. Díaz et al. 106* (COAH, UDBC); Vereda El Canelo, Finca Cataluña, margen izquierdo del Río Orteguaza, 01º29'58.7"N, 75º31'16.6"W, 330 m, 20 Jan 2002, *C. Marín et al. 2482* (UDBC); Milán, Inspección San Antonio de Jetuchá, resguardo indígena Gorgonia, 1°05’152’N, 75°28’219’’W, 218 m, 29 Aug 2006, *W. Trujillo 208* (HUAZ); **Cauca**: Guapí, Isla de Gorgona, zonas cercanas a las instalaciones del Inderena, 0-150 m, 10-14 Sep 1987, *J.L. Fernández Alonso et al. 7542* (COL); Parque Nacional Natural de Isla Gorgona, camino a Pablo VI, 3 Jun 1986, *G. Lozano et al. 5167* (FMB); **Cundinamarca**: Ubalá B, Vereda Boca de Monte, 1000-1050 m, 29 Jun 1998, *J.L. Fernández Alonso et al. 16057* (COL); Girardot, Isla del Sol, a orillas del Río Magdalena, 10-15 Jan 1972, *H. García Barriga 20315A* (COL, US); **Guainía**: Puerto Inírida, Comunidad El Coco Nuevo, distante 6 km NE de la cabecera municipal, 3º54’33’’N, 67º54’10’’W, 2 Mar 2000, *J.C. Arias-G & J. Garzón J. 667* & *668* (COAH); Comunidad Matraca, margen izquierda Río Inírida, 02º52’23’’N, 69º05’36’’W, 5 Mar 2000, *J.C. Arias-G. et al. 682* (COAH); Comunidad Paujil, Huerto Tucan Miguel Lizardo Puinave, 3º52’42.6’’N, 67º53’38’’W, 120-180 m, *C. Marín & F. Rodríguez 504* (COAH); Puerto Inírida, 3º51’56.6’’N, 67º55’56’’W, 180 m, *C. Marín & F. Rodríguez 508* (COAH); Vereda comunal, margen derecha, Tomasa Betancur, *C. Marín & F. Rodríguez 509* (COAH, COL); Comunidad Curripacos, 3º59’34’’N, 68º21’13’’W, *F. Rodríguez 2* (COAH, COL); Comunidad Curripacos, 3º51’56.6’’N, 67º55’56’’W, *F. Rodríguez 505* (COL); Caño Bocón, afluente del Río Inírida, aldea indígena Barranco Tigre, 25 Aug 1979, *G. Triana 18* (COL); **Guaviare**: San José de Guaviare, Laguna El Recreo, Aug 1998, *G. Garzón et al. 3214* (COL); El Retorno, inspección La Unilla, caserío San Lucas, en cercanias de caños El Tablazo y Morichera, 2º11’43.8’’N, 72º47’03.5’’W, 220 m, 20 May 1996, *R. López et al. 1756* (COL); Calamar, límite entre los munícipios del Retorno y Miraflores, Vereda Agua Bonita baja, 01º54’17.3’’N, 72º25’50’’W, 5 Jan 2000, *R. López et al. 5943* (COAH); same locality, 01º51’03.6’’N, 72º27’25.9’’W, 18 Sep 2000, *R. López et al. 5997* (COAH); Calamar, Barrio O. Vargas Cuellas, Fam. Tovar, 01º58’06.1’’N, 72º39’14.3’’W, *F. Rodríguez & M. Coy s.n.* (COAH); **Magdalena**: Ciénaga, 15 Sep 1949, *M. de Romero 72* (COL); **Meta**: Villavicencio, a orillas del Río Guatiquía, 350 m, 20 Nov 1948, *J. Araque Molina & F.A. Barkley 18M.050* (COL, MEDEL); cerca de Peralonso, 24-28 Jul 1946, *R. Jaramillo et al. 351* (COL, US); Restrepo, alrededor de la Quebrada Caño Seco, límite entre las veredas Alto Caney y Salinas, 570 m, 28 Jun 1979, *D. Pinto et al. 48* (COL);

Villavicencio, *M. Torres & F. Rodríguez 2025* (COAH); Villavicencio, 19 Jan 2005, *L.G. Velásquez Castillo MA-4 & MA-7* (COL); **Nariño**: Tumaco, km 63, Carretera Tumaco-Pasto, Llorente, 260 m, 6 May 1984 *O. de Benavides 4692* (PSO); Maguí, trayecto Payán-Barbacoas, 130 m, 8 Jul 1987, *O. de Benavides 8312* (PSO); Taminango, Corregimiento de El Remolino, El Manzano, 1000 m, 20 Oct 1988, *O. de Benavides 10527* (PSO); La Unión, 29 Mar 1963, *M.L. Bristol 702* (COL); Río Sanquianga, Vereda Loma Grande, 1º51'N, 78º19'W, 20 m, 16 Oct 1992, *R. Caballero 40* (MEDEL); **Putumayo**: Orito, comunidad Paez Aguadita, *F. Rodríguez 48 & 49* (COAH); San Miguel, Vereda Puerto Limón, *F. Rodríguez 53* (COAH); **Tolima**: Mariquita, 600 m, 25 Sep 2001, *C. Bernal et al. 1098* (COL); **Valle del Cauca**: Cali, Felidia, margen derecha del Río de Las Nieves, finca La Tulia, 1700 m, 19 Jul 1945, *J. Cuatrecasas 19689* (F, COL, US); Palmira, prédio UN de Colombia, sede Palmira, *F. Rodríguez s.n.* (PSO 038195); Jamundí, 1000 m, 24 May 1967, *M.E. Sánchez 7* (COL); **Vaupés**: Carurú, alrededor del casco urbano, 200 m, 1º00'45.5''N, 71º17'44.7''W, 15 Nov 2009, *D. Cárdenas 24632* (COAH); Pacoa, comunidade Embera-Katio, Cañabravita, *F. Rodriguez 59* (COAH); Mitú, Comunidad indígena Cotán-Yarinal, *F. Rodríguez 68* (COAH); Inspección Santa Ana, *F. Rodríguez 71* (COAH); Comunidad Trubon, margen derecha del Río Caupes, 1º12'31''N, 70º04'4.8''W, 184 m, *F. Rodríguez 82* (COAH); Comunidad Yacayaa, Cunamia, 01º0.6'16.2''N, 70º30'20''W, 200 m, *F. Rodríguez 112* (COAH); Comunidad Murutinga, margen derecha del Río Murutinga, 01º00'38''N, 70º05'39.8''W, 180 m, *F. Rodríguez 118* (COAH); Comunidad Betania, margen izquierda del caño Timbo, km 50 carretera Mitú-Bogotá, Cachibera, *F. Rodríguez 120* (COAH); Soratama, Río Apaporis, entre el Río Pacoa y el Río Kananarí, 250 m, 17 Jul 1951, *E.E. Schultes & Cabrera 13046* (A, US); same locality, 27 Aug 1951, *R.E. Schultes & I. Cabrera 13752* (COL, GH); Mitú, 9 Oct 1976, *J.L. Zarucchi 2173* (COL, GH).

COSTA RICA: Puntarenas: Santo Domingo de Golfo Dulce, Mar 1896, *J. Donnell Smith 7255* (GH); **San José**: San José, 1160 m, Apr 1932, *M. Valerio 151* (F).

CUBA. Isla de Pinos, Nueva Gerona, in tickets, 15 Nov 1920, *E. Ekman s.n.* (S 12226).

ECUADOR. Bolívar: Parroquia Telimbela, Chimbo, 01º41'S, 79º10'W, 2200 m, 3 May 1991, *C. Cerón 14561* (QCNE); **Chimborazo**: San José de Prisipamba, a 800 m de Pallatanga, 1630 m, 11 Jan 2003, F. *Angulo 007* (CHEP); Cantón Cumandá, en el Recinto San Pablo, 2000 m, 19 Jan 2005, *P. Ordóñez 016* (QCNE); **El Oro:** Pénjamo, 23 Nov-25 Dec 1976, *L. A. de Escobar 828* (QCA); Casacay, Dukus, 03º18'30''S, 79º42'28''W, 270 m, 2 Mar 1996, *V. van den Eynden & E. Cueva 654* (QCA, QCNE); **Esmeralda**: Río Cayapa, Zapallo Grande, 500 m upstream the village on the opposite bank to the forest, 00º48'N, 78º54'W, 100 m, 26 Jun 1982, *L.P. Kvist & E. Asanza 40356* (COL); Río Cayapa, Zapallo Grande, farmland close to the village, 0º48'N, 78º54'W, 100 m, cult., 1 Jul 1982, *L.P. Kvist & E. Asanza 40456* (AAU, QCA, QCNE); Zapallo Grande, 5 km downstream from the village, 0º49'N, 78º57'W, 100 m, cult., 30 Jun 1982, *L.P. Kvist 40565* (AAU, NY, QCA, QCNE); Cantón Eloy Alfaro, Comuna de Corriente Grande, 00º41'N, 78º50', 150-200 m, 6 Aug 1993, *A.P. Yañez et al. 1407* (QCNE); **Galápagos: Albemarle** Is. (Isabela Is.), Santo Tomás, 350 m, 31 Jul 1977, *A. Adsersen & H. Adsersen 2361* (C); near Santo Tomas, 29 Apr 1932, *J.T. Howell 9009* (CAS); **Chatham Is.** (San Cristobal Is.), Finca La Esperanza near Cerro Verde, 26 Feb 1986, *P. Bentley 456* (QCA); San Cristóbal, 450 ft, 10 Oct 1905, *A. Stewart 3355* (CAS); Chatham Island, Wrick Bay, 350 ft, 29 Jan 1906, *A.*

Stewart 3356 & *3357* (CAS, US); at the village of Progress, 18 Apr 1932, *M. Williams s.n.* (CAS 389187); **Floreana** Is. (Charles Is.), hab. in insula Charly, *Andersson s.n.* (S); Charles Is., 450-1000 ft, 11 Oct 1905, *A. Stewart 3354* (CAS); **Indefatigable Is**. (Santa Cruz Is.), Santa Cruz's farm, Floreana, 1 Apr 1977, *A. Adsersen & H. Adsersen 1517* (QCA); Santa Cruz, Charles Darwin Research Station, 0°45'S, 90°17'W, 13 Apr 1982, *P.S. Bentley 312* (QCA, QCNE); Charles Darwin Research Station, 13 Apr 1982, *P.S. Bentley 312-a* (QCNE, US) & 26 May 1983, *P.S. Bentley 312-b* (QCNE, US); 4-5 mi N Academy Bay, 775 ft, 13 Feb 1953, *R.I. Bowman 63* (UC); on trail to El Chato, 30 Jul 1966, *P.A. Colinvaux 440* (CAS); two-thirds the way to Bella Vista, along the old trail, 120-130 m, 15 Feb 1964, *L.A. Fournier 218* (CAS, US); N slope of Mount Crocher, N of Cerro Colorado, 510-555 m, 4 Mar 1972, *M. & O. Hamann 645*; Santa Cruz, S of the farm "Flor del Bosque", central part of the island, 320-330 m, 8 Jun 1972, *M. & O. Hamann 1418* (C); in the Scalesia forest, northeast of Bella Vista, 150-200 m, 5 Mar 1981, *O. Hamann & S. Serberg 1700* (C); near Fortuna, 8 May 1932, *J.T. Howell 9175A* (CAS); between Bella Vista and Mt. Crocker, 560 m, 2 Feb 1964, *S. Itow 136* (CAS); Los Gemelos, 0º37'38.1''S, 90º23'20.1''W, 600 m, 16 Apr 2001, *P. Jaramillo et al. 1775* (QCNE); Santa Cruz, 220 m, 8 Jun 1966, *I. von Rentzell de Atkinson 11* (CORD); Isla Santa Cruz, in evergreen forest along the "old trail" from Puesto Ayora to Bella Vista, 110-150 m, 15 Mar 1981, *O. Serberg 1753* (C); 0.5 km N of Caseta, in 'mora' (Caesalpinia bonduc) field, 10 Aug 1970, *R. Silberglied s.n.* (GH); along a trail leading inland NW from Academy Bay, 150 ft, 10 Feb 1939, *R.G. Taylor G.9* (CAS); along trail near Bella Vista, 520 ft, Apr 1974, *H.H. van der Werff 909* (AAU, CAS, QCA); Horneman's Ranch, near Bella Vista, 225 m, 8 Feb 1964, *I.L. Wiggins 18643* (CAS); **Guayas**: Guayaquil, patio de la Universidad, 2º11'S, 79º53'W, 5 m, 12 May 1994, *X. Cornejo S. & C. Bonifaz 2584* (QCNE); Isla Puná, Puná Nueva, 02º44'S, 79º54'W, 4 Jun 1987, *J.E. Madsen 63565* (QCA, QCNE); **Loja**: desde Macará, La Guar, carretera Macará-Sapotillo, km 12-13, 4º19'03''S, 80º01'10''W, 517 m, 6 May 2017, *G.E. Barboza & S. Leiva González 4829* (CORD); Rd. Quilanga-Cariamanga, Km 6-10, 4º21'S, 79º24'W, 1200-1400 m, 22 Jun 2000, *J.E. Madsen et al. 7040* (LOJA, QCNE); Río Pindo, 3º49'38''S, 79º42'58''W, 9 Dec 1992, *F. Vivar C. 4003* (LOJA, QCNE); **Los Ríos**: Río Palenque Biological Station, km 56, Quevedo-Santo Domingo, 150-220 m, 7 Oct 1976, *C.H. Dodson & A.H. Gentry 6576* (MO); Hacienda Clementina, 30 m, 23 Feb 1947, *G. Harling 316* (MO, S); **Manabí**: Cantón Puerto López, cult. en jardín de un hostal, 30 Dec 2012, *R. Deanna 53* (CORD); **Morona-Santiago**: Macuma, Shuar gardens, 7 Mar 1982, *W. van Asdall 82-59* (QCA); Centro Shuar Yukutais, 03º30'S, 78º10'W, 21 Apr 1989, *B. Bennett & P. Gomez Andrade 3776* (QCNE); Morona, bosque Domono, a 12 km de Macas, 2º15'S, 78º8'W, 1070 m, 2-4 Dec 1994, *I. Padilla et al. 3088* (QCNE); **Napo**: Cantón Tena, Estación biológica Jatun Sacha, a 8 km al E de Misahuallí, 01º04'S, 77º36'W, 450 m, Jun 1989, *B.C. Bennett & School for Field Studies s.n.* (QCNE 119241); Cantón Lago Agrio, Dureno, comunidad indígena Cofán, 00º02'S, 76º42'W, 350 m, 5-15 Feb 1986, *C. Cerón 194* (MO, NY, QCA, QCNE); Pompeya, 19 Apr 1983, *L. Siquihua 4* (QCA); **Orellana**: Aguarico, Parroquia Nuevo Rocafuerte, comunidade Martinica (Kichwa), s.f, *D. Reyes & L. Carrillo 1049* (QCNE); **Pastaza**: Kapawi (Amuntai), Río Pastaza, 2º31'S, 76º48'W, 235 m, 25-29 Jul 1989, *W.H. Lewis et al. 14011* (QCNE); **Pichincha**: Km 170-175, vía Santo Domingo-Quinindé, 300 m, 11 Sep 1949, *M. Acosta Solís 13927* (F); Santo Domingo de los Colorados, centro científico Río Palenque, 0º35'S, 79º21'W, 220 m, 15 Mar 1992, *T.B. Croat 73052* (QCNE); Santo Domingo, Luz de América, Hacienda Esmeralda Molestina, 650 m, 14 Feb 1999, *P. Gómez 006* (CHEP);

Santo Domingo de Los Colorados, via Quinindé, Km. 8, Hac. El Salvador, 540 m, 17-18 Jan 2004, *D. Guilcapi 19* (CHEP); in the Colorado community "Congoma Grande" at km 23 on the Santa Domingo-Puerto Limón road, 00º21'S, 79º22'W, 100 m, 9 Jun 1982, *L.P. Kvist 40201* (NY, QCA); Pedro Vicente Maldonado, población rural del cantón Quito, al NO de Alvaro Pérez Itriago, a 3.5 km, 00º05'N, 79º02'W, 700 m, 4 Jul 2003, *P. Santacruz 20* (QCA); **Sucumbíos**. Shushufindi, Comunidad Secoya de San Pablo, en la reivera del Río Aguarico, 00º15'25.4''S, 76º25'31.7''W, 230 m, 6-14 May 2004, *N. Miranda-Moyano 225* (QCNE); Río Aguarico, Shushufindi, in house garden, 4 Mar 1975, *W.T. Vickers 208* (CHEP, QCA); 7 Mar 1975, *W.T. Vickers 226* (QCA, CHEP); **Zamora-Chinchipe**: Cantón el Pangui, comunidad shuar Michanunka, 3º54'74.50''S, 77º21'58''W, 776 m, 17 Jun 2007, *A. Ortega et al. 51* (LOJA); Gira Zamora-Yanzatza-El Padmi, 3º49'30''S, 78º46'3''W, 1-2 Jul 1987, *F. Vivar C. & B. Merino 3027* (LOJA).

EL SALVADOR. Santa Ana: entre la Peña del Cuervo y el Cerro El Yupe, ca. 3 km al NO de Candelaria de la Frontera, 1100 m, 22 Jul 1993, *J.L. Linares 556* (MEXU); **Sonsonate**: vicinity of Santa Emilia, 135 m, 22-25 Mar 1922, *P.C. Standley 22237* (GH, US); **San Vicente**: San Vicente, 23 Sep 1978, *W. D'Arcy 12130* (NY); **San Salvador**, San Salvador, 17 Sept 1922, *S. Calderón 1198* (NY, US); San Salvador, Jun 1906, *L.V. Velasco 8925* (US).

GUATEMALA. **Chiquimula**: Camotán, Jucotán, Dispensario Bethania, 14°4.0019'N, 89°23.241'W, 450 m, 16 Oct 2000, *J. Kufer 100* (MEXU); **Izabal**: Puerto Barrios, 20 Mar 1947, *J.F. Brenckle 47-418* (CORD); Livingston, 18 Feb 1905, *C.C. Deam 24* (MICH); vicinity of Quiriguá, 75-225 m, 15-31 May 1922, *P.C. Standley 23755 & 24297* (GH); vicinity of Puerto Barrios, at sea level, 2-6 Jul 1922, *P.C. Standley 25055* (US); **Petén**: La Libertad and vicinity, Aug-Nov 1933, *M. Aguilar H. 199* (MEXU, MICH); Tikal, in forest, 16 Jul 1959, *C.L. Lundell 16480* (CAS); **El Progreso**: San Agustín Acasaguastlán, 300 m, 26 Oct 2003, *M. Véliz 14613* (MEXU).

GUADELOUPE: Along side road off N7, ca. 5 km WNW of Moule, 21 Apr 1969, *M.R. Crosby & C.A. Crosby 4694* (MEXU).

GRENADA: **Saint Andrew Parish**: Grand Etang, 18 Mar 1924, *G.S. Miller 149* (US).

GUYANA. **Demerara-Mahaica**: campus of the University of Guyana and surrounding fields, 7º49'N, 58º08'W, 10 m, 15 Dec 1988, *W. Hahn 4830* (NY, US); **Mahaica-Berbice**: Distr., Campagne, 3 km S of coastal highway between Mahaicony & Abary Rivers, 6º30'N, 57º50'W, 0-5 m, 15 Aug 1995, *S.R. Hill et al. 27189* (US); **Upper Takutu-Upper Essequibo**: Konashen-area, Essequibo River, 1º34'N, 58º41'W, 250 m, 25 Sep 1989, *M.J. Jansen-Jacobs et al. 1809* (US).

HAITI: Grand'Anse: Cir. Haití, Ile La Navase, northern section, in savannas apparently native, 20 Oct 1928, *E. Ekman s.n.* (S 10824); **Nord**: vicinity of Pilate, 325 m, 11 Feb 1926, *E.C. Leonard 9700* (US); **Nord-Ouest**: Cir. Haití, Ile La Tortue, La Vallée, 25 Mar 1928, *E. Ekman s.n.* (S 9805b); **Ouest**: Port-au-Prince, Montfleury, 23 Nov 1924, *E. Ekman s.n.* (S); between St. Marc and L'Arcahaie, 8 Apr 1925, *G.S. Miller Jr. 199* (US).

HONDURAS. Atlántida: Lancetilla Valley, near Tela, 20-600 m, 6 Dec-20 Mar 1928, *P.C. Standley 53392* (F); **Comayagua**: barrio arriba Comayagua, 850 m, 13 Apr 1985, *H.J. Ramos 164* (NY); **Morazán**: drainage of the Rio Yeguare, área de la N.A.P., Zamorano, 800 m, 6 Jul 1948, *A. Molina R. 1054* (GH, US); Jicarito, camino entre Jicarito y San Antonio del Mico, 950 m, 29 Sep 1948, *A. Molina R. 1177* (F).

JAMAICA. Vicinity of Constant Spring, 23 May 1904, *W.R. Maxon 2160* (US).

MEXICO. Campeche: Escárcega, Felipe Angeles a Nva. Rosita, 10 m, 10 Oct 1984, *C. Chan 4149* (MEXU); km 17 de la carr. Escarcega-Champotón sobre la terracería a Venustiano Carranza, 5 Feb 1983, *E. Martínez S. & F. Martínez 6316* (MEXU); Calakmul, a 2 km al SE de Dos Naciones, 17º57’41’’N, 89º20’33’’W, 170 m, 27 Oct 1997, *E. Martínez S. 29417* (MEXU); **Chiapas**: Chiapa de Corzo, El Chorreadero, 5.6 miles E of Chiapa de Corzo, 2500 ft, 3 Jul 1965, *D.E. Breedlove 10689* (CAS, F); Solosuchiapa, 2-4 km below Ixhuatán along road to Pichucalco, 1200 m, *D.E. Breedlove 19922* (CAS, MEXU); Venustiano Carranza, near Rancho Carmen along the road from Acala to V. Carranza, 2500 ft, 7 Jan 1967, *R.M. Laughlin 2018* (CAS); Ocosingo, en campamento COFOLASA, a 24 km al SE de Crucero Corozal, camino Boca Lacantum, 220 m, 25 Feb 1985, *E. Martínez S. 11240* (MEXU, NY); en el vértice del Río Chixoy camino a Chajul, 130 m, 12 Jan 1986, *E. Martínez S. M-16073* (MEXU); Escuintla**,** Esperanza, 23 Feb 1948, *E. Matuda 17642* (MEXU); **Oaxaca**: Tuxtepec, Rancho “Los Caracoles”, cerca de Tuxtepec, Aug 1963, *J.M. Alcocer & C. Morales s.n.* (CAS 615429); Comaltepec, Distr. Ixtlan, Soyalapam, ca. 300 m, 17º45’N, 96º30’W, 15 Dec 1989, *R. Lopez Luna 0579* (NY); Juchitán de Zaragoza, Itsmo de Tehuantepec, La Ventosa, 16º33’27’’N, 94º56’48’’W, 34 m, 8 Jun 2014, *F. Sánchez L. et al. 953* (MEXU); Matías Romero Avendaño Ubero, 30-90 m, Jun 1937, *L. Williams 9538* (F); San Mateo del Mar, 5 m, 20 Jan 1978, *D. Zizumbo & P. Colunga 145* (CAS); **Quintana Roo**: Othón P. Blanco, Zoológico y Jardín Botánico PAYO OBISPO, 29 Jan 1990, *S. Torres 127* (MEXU); **Tabasco**: Centro, Ranch. Tumbulushal, carretera Vhsa-Teapa, km 20, 17º50’N, 92º56’W, 15 m, 5 May 1993, *P. Alegría O. 47* (MEXU); Cunduacán, 14 Aug 2003, *S. Becerril Pérez & N.L. Ortiz Cornejo 7* (MEXU); Teapa, en la escuela de Veterinaria de la UJAT, Km 25 de Villahermosa a Teapa, 14 Apr 1988, *M.A. Magaña 1957* (MEXU); poblado de Nacajuca, 20 m, 3 Oct 1978, *R. Ortega et al. 876* (NY, UC); Ranch. Dos Montes, km 12, carr. Vsa.-Escárcega, atrás del aeropuerto de Villahermosa, 17º45’N, 92º45’W, 15 m, 21 Sep 1988, *A. Sol S. 134* (MEXU); **Veracruz**: Orizaba, *M. Botteri 904* (US); Coatzacoalcos, Laguna Ostión, camino Pajapan-San Juan Volador, 18º11’N, 94º36’W, 6 Nov 1986, *J.I. Calzada 12662* (MEXU); San Lorenzo Tenochtitlan, 29 Nov 1967, *M.A. Martínez A. 346* (MEXU); **Yucatán**: Izamal, *G.F. Gaumer 1019* (CAS, F); Chichén Itzá, Jun 1932, *W.C. Steere 7404* (MEXU).

NICARAGUA. **Zelaya**: Monkey Point, lado S del campo de aterrizaje abandonado, 11º36’N, 83º39’W, 10 m, 22 Oct 1981, *P.P. Moreno & J.C. Sandino 12134* (MEXU); **Matagalpa**: along road between Waswalí Abajo and Waswalí Arriba, ca. 12º55-56’N, 85º57’W, 620-660 m, 24 May 1981, *W.D. Stevens et al. 20300* (MEXU).

PANAMA. **Canal Zone**: Balboa, Canal Zone, Nov 1923-Jan 1924, *P.C. Standley 25504* (US); Sosa Hill, Balboa, 27 Nov/10 Dec 1923, *P. Standley 26493* (A, C); **Panamá**: along

Corazal, road near Panamá, *P. Standley 26862* (US); between Matías Hernández and Juan Díaz, *P. Standley 32004* (US).

PERU. **Amazonas**: Condorcanqui, Galilea, 11 Jul 1991, *Salaün 177* (USM); **Cajamarca**: San Ignacio, Huarango, Mechinal, 05º19'S, 78º43'W, 600 m, 2 Feb 1996, *J. Campos de la Cruz & O. Díaz 2339* (HUT, USM); alrededores de Namballe, 720 m, 4 Aug 1994, *S. Leiva González et al. 720* (F); sector de Huito, 2 km al NO de la ciudad de Jaén, 870 m, 21 Dec 2004, *I. Sánchez Vega et al. 12745* (NY); **Huánuco**: Bosque Nacional de Iparia, a lo largo del Río Pachitea, cerca del campamento Miel de Abeja (1 km arriba de Tournavista), 300-400 m, 23 Dec 1966, *J. Schunke V. 1397* (COL); **Junín**: Tarma, cerca de Oxapampa, unos 5 o 6 Km al sur, 23 Apr 1959, *P.G. Smith s.n.* (USM); 3 millas al E de San Ramón, 26 Apr 1959, *P.G. Smith s.n.* (USM); **Lambayeque**: Chiclayo, Reque, 25 m, 7 Mar 1994, *S. Llatas Quiroz 3452* (F); same locality, 20 Apr 1997, *S. Llatas Quiroz 4147* (HUT); **Loreto**: Rio Napo, Tamarico?, 14 Aug 1966, *R.T. Martin & C.A. Lau-Cam 1242* (GH); Alto Amazonas, Distr. Balsapuerto, Soledad, 05º40'18''S, 76º37'28''W, 8 Nov 2007, *G. Odonne 0522* (USM); same locality, 24 May 2008, *G. Odonne 0641* (USM); Maynas, Río Amazonas, Isla Rondiña, opposite Leticia, Caño Gamboa, 18 Mar 1977, *T. Plowman et al. 6418* (GH, USM); **Madre de Dios**: Puerto Maldonado, 2 Dec 1978, *G. Müller & P. Gutte 8422* (USM); **Piura**: Huancabamba, camino de Piura-Canchaque, 13 Mar 1966, *P.G. Smith s.n.* (USM); **San Martín**: Huallaga, cerca de Bellavista, 250-350 m, 16 Sep 1954, *R. Ferreyra 10086* (USM); Mariscal Cáceres, Tocache Nuevo, 8º11'S, 76º30'W, 500 m, *T. Plowman & J. Schunke V. 11694* (CORD, USM).

PUERTO RICO. **Bayamón**: San Juan, Buen Consejo, Río Piedras, 19 May 1914, *Bro. Hisram s.n.* (US 759716); prope Bayamón, 28 Mar 1885, *I. Urban 866b* (CORD, LE); **Camuy**: Camuy, 10 Dec 1937, *F.H. Sargent B69* (MO, US).

SURINAM. Coronie, 20 Oct 1933, *J. Lanjouw 1040* (CORD).

TRINIDAD AND TOBAGO: **Tobago**, Scarborough, 12 Apr 1913, *W. Broadway 4498* (F, US, S).

VENEZUELA. **Amazonas**: selvas pluviales y áreas perturbadas del Río Pasimoni, a la altura de Buridajao, 10-12 Feb 1989, *B. Stergios et al. 13316* (US); **Anzotegui**: Pekin, 100 m, *H. Peñaloza 4856* (VEN); **Apure**: Mun. Mantecal, Módulo de la UNELLEZ Fernando Corrales, 10 Sep 1981, *G. Aymard 473* (VEN); **Aragua**: cerca de La Trilla, km 35, carretera Maracay-Ocumare de la costa, ca. 400 m, 25 Aug 1973, *V. Badillo 6379* (MY); alrededores de la Facultad de Agronomía, Maracay, 450 m, 18 Aug 1966, *C. Benítez de Rojas 13* (MY); Maracay, El Limón, 6 Nov 1967, *G. Ferrari 185* (MY); hills above Guayas, Tuy valley, 12 Sep 1926, *H. Pittier 12199* (VEN); **Bolivar**: ciudad de Bolívar, 35 m, 4-25 Nov 1929, *E.G. Holt & W. Gehriger 109* (NY); 3 km W of El Paujil and 0-4 km S of road, along Rio Paujil, 4°30'N, 61°36'W, 800-900 m, 13 Nov 1985, *R. Liesner 20013* (VEN); **Carabobo:** Hacienda Cura San Joaquín, 430 m, 20 Jul 1968, *C. Benítez de Rojas 379* (MY); alrededores de El Palito, cerca de Puerto Cabello, 24 Nov 1973, *J.H. Hunziker 9031* (CORD); Hacienda de Cuara, cerca de San Joaquín, en lugares frescos y sombreados, 8 Jul 1918, *H. Pittier 7909* (GH, US, VEN); silvestre en relicto de selva en galería del Río Guataparo, aledaña al pastizal Calicanto en la Hacienda Guataparo, alrededores de

Valencia, 29 Jan 1982, *B. Trujillo & Rodríguez 18026* (MY); **Delta Amacuro:** alrededores de Tucupita, 27 Aug 1959, *B. Trujillo 4643* (MY)**; Distrito Federal**: Caracas, a few miles N of Consejo, 4 Feb 1954, *Anonymous s.n.* (GOET); Colinas de Bello Monte, 10º23'N, 66º55'W, 1100 m, 8 Jun 1981, *A. Castillo 1079* (CORD); El Valle, Hacienda Sosa, , 20 Sep 1949, B. *Trujillo & M. Trujillo 388* (MY); **La Guaira (Vargas)**: Vargas, Todosana, 10°38'N, 66°27'W, 120 m, 20 Apr 1992, *A. Castillo s.n. (CEE-45)* (VEN); Vargas, plantas cultivadas en alrededores de la residencia de la Est. Experimental de la Fac. de Agronomía UCV, vecindades de Petaquire, 1730 m, 18 Aug 1979, *B. Trujillo & M. Trujillo 15605* (MY); **Sucre**: Isla Margarita, 7 Dec 1901, *O.O. Miller & J.R. Johnston 81* (F); **Trujillo**: alrededores de Cuicas, 9°42′0.86″N 70°17′9.28″W, 950 m, 27 Apr 1968, *C. Benítez de Rojas 298* (MY); Valle del Río Momboy, entre Valera y Mendoza cerca de El Cucharito, 900-1000 m, 14 Sep 1988, *J. Bono 6896* (MY).

Cultivated
ARGENTINA. Buenos Aires: Tigre, 20-IV-1953, *Anonymous s.n.* (LP *898103*).
Córdoba: Capital, cult. en invernadero de IMBIV (Ciudad Universitaria), 31 Jan 2008, *M. Scaldaferro 13* (CORD); cult. cult. en invernadero de IMBIV (Ciudad Universitaria), semillas compradas en mercado Rio de Janeiro (Brasil), 18 Mar 1992, *E.A. Moscone 200* (CORD); Río Segundo, Pilar, 5 Apr 2001, *R. Subils 4680* (CORD); **Salta**: Cerrillos, Estación Experimental INTA Cerrillos, 28 Mar 1995, *A.T. Hunziker 25489* (CORD).

BRAZIL. **Minas Gerais**: Belo Horizonte, comprado en mercado, 8 Jul 2003, *G.E. Barboza 795* (CORD); **Paraíba**: Campus da U.F.P.B., 27 Mar 1988, *M.F. Agra 625* (JPB); Jardím Botânico, 7º60'S, 34º52'W, 22 Aug 2007, *P. da C. Gadelha Neto 1751* (CORD, JPB); **Paraná**: Itaperica da Serra, a 2 km de Itaperica, rumbo a Embu Guazú, comprada en vivero, 15 Apr 2008, *G.E. Barboza et al. 2022* (CORD); Morretes, Estação Experimental, May 1988, *L.C. Leitão s.n.* (RB 274865); Estação Experimental IAPAR, 20 Feb 1989, *L.C. Leitão s.n.* (RB 280074); **Pernambuco**: Recife, cult. en predio UFPE, 13 Jul 1999, *E. Moscone 237* (CORD); **Rio de Janeiro**: comprado en el mercado de Humaitá (Cospal de Humaitá), 18 Apr 2008, *G.E. Barboza et al. 2041* (CORD); comprado en Mercado de Rio de Janeiro, Jun 1991, *A.T. Hunziker 25505* (CORD); Guanabara, adquirido en mercado, 6 Feb 1973, *A. Krapovickas et al. 23427* (CTES); **Santa Catarina**. Garuva, Parque Aquatico Monte Crista, en la Pousada de Monte Crista, 14 Apr 2008, *G.E. Barboza et al. 2019* (CORD); **São Paulo**, comprado en el mercado Municipal, 18- IX- 2008, *G.E. Barboza 2064bis* (CORD).

CUBA. **La Habana**: cultivado en el Hotel Palco, 14 Sep 2009, *G.E. Barboza s.n.* (CORD 1325).

PARAGUAY. **Central**, San Lorenzo, 2 Jun 1985, *A. Schinini & E. Bordas 24511* (CTES).

PUERTO RICO. Yabucoa: Yabucoa, in hortis cultis, 8 Sept 1886, *I. Urban 5055* (CORD).
UNITED STATES OF AMERICA. California: grown in Davis, from Mexico, 1953, *P.G. Smith s.n.* (Davis Ac 1433) (UC); grown in Davis, from El Salado, Margarita Isl. Venezuela (leg. C.S. Alexander through F.C.O. Sauer), 1953, *P.G. Smith s.n.* (Davis Ac 1495) (UC); grown in Davis, from La Rinconada, Margarita Isl. Venezuela (leg. C.S. Alexander through F.C.O. Sauer), 1953, *P.G. Smith s.n.* (Davis Ac 1498) (UC); grown in Davis, from El Pabado,

Margarita Isl. Venezuela (leg. C.S. Alexander through F.C.O. Sauer), 1953, *P.G. Smith s.n.* (Davis Ac 1500) (UC); **Florida**: 7 miles northeast of Plant City, 14 Oct 1930, *F.S. Banton 6838* (CAS); **Illinois**: Chicago, 21 Sep 1987, *T. Plowman 14539* (CORD); **Indiana**: Bloomington, cult. at IU greenhouse, seeds from Saipan, Mariana Islands (leg. P. *Smith AC 965*), 1955, *C.B. Heiser s.n.* (CORD); cult. at IU Experimental Field, seeds from La Penconadu, Is. Margarita, Venezuela (leg. P. Smith Ac 1498), 1955, *C.B. Heiser s.n.* (CORD); cult. at IU greenhouse, seeds from Burpee Seed Co. B5298, 1958, C.B. *Heiser s.n.* (CORD, LIL); cult. Indiana University, seeds from Pichilingue, Ecuador (leg. C. Rick; P. Smith Ac 1929), 1958, *C.B. Heiser s.n.* (CORD); cult. at IU greenhouse, seeds from Piura, Perú, 1958, *C.B. Heiser s.n.* (LIL); cult. at IU greenhouse, (leg. P. Smith AC 1973), 1959, *C.B. Heiser s.n.* (CORD); cult. in Bloomington, Aug 1948, *C.B. Heiser Jr. C6* (CORD); cult. IU Experimental Field, wild form used by Totonac Indians, El Tapin, Paxantla, Venezuela (leg I. Kelly 3), *C.B. Heiser C36* (CORD); cult. at IU greenhouse, 1956, *C.B. Heiser C229 & 230* (CORD); cult. at IU greenhouse, seeds from Trinidad (leg. C. Hagen), 24 Sep 1958, *C.B. Heiser C256* (CORD); cult. at IU greenhouse, seeds from Trinidad (leg. C. Hagen), 1958, *C.B. Heiser C259 & C260* (CORD); cult. at IU Experimental Field, seeds from weed in yard, Quinindé, Ecuador, 6 Sep 1963, *C.B. Heiser 5041* (CORD). cult. in IU greenhouse, seeds from market at Guayaquil, Ecuador, 3 Dec 1963, *C.B. Heiser 6097* (CORD); cult. IU Experimental Field, seeds from market at Guayaquil, Ecuador, 6 Sep 1963, C.B. *Heiser 6100* (CORD); IU Experimental Field (seeds from market Quito, Ecuador, 1962), 6 Sept 1963, *C.B. Heiser 6247* (CORD); grown in IU greenhouse from seed collected in Brazil, Bahia, market in Remanso, 9º40'S, 42º5'W, 380 m, 22 Aug 1974, *B. Pickersgill RU72-118* (UC); grown in IU greenhouse from seed collected in Brazil, Piauí, Fazenda Serra Talhada Veneza, 28 km NE of São Raimundo Nonato, 8º20'S, 42º40'W, 350 m, 23 Jul 1974, *B. Pickersgill RU72-140* (UC); grown in IU greenhouse from seed collected in Brazil, Ceará, Quincunca, 6º53'S, 39º37'W, 550 m, 23 Jul 1974, *B. Pickersgill RU72-244* (UC); grown in IU greenhouse from seed collected in Brazil, Rio Grande do Norte, market in Currais Novos, 6º17'S, 36º32'W, 350 m, 22 Jul 1974, *B. Pickersgill RU72-35*7 (UC); IU Experimental Field, seed from Tarapoto, Perú, 6 Sept 1963, *P.G. Smith SA 374* (CORD); **Louisiana**. Avery island, Iberia Parish, grown for preparation of McIlhaney Tobasco Sauce, in cult., 7 Jun 1969, *F.G. Meyer & P.M. Mazzeo 11988* (CAS); **Nuevo Mexico**: Las Cruces, Instituto Chile Pepper, 12 Feb 2009, NMCA 40011, NMCA 40016 & NMCA 40025 (CORD).

20. Capsicum galapagoense Hunz.

ECUADOR. Galápagos: Indefatigable Is. (Santa Cruz Is.), Cerro Colorado II, at the marked trail, 630 m, 29 Jun 1977, *A. Adsersen & H. Adsersen 2062* (CDS, QCA); Narborough Is. (Fernandina Is.), 19 Sep 1974, *H. Adsersen 2742* (CDS); Jervis Is. (Rábida Is.), 24 Oct 2010, *M.R. Gardener 49155* (CDS); Indefatigable Is., on the north slope by the road between Santa Rosa and the land, 500-540 m, 30 Mar 1981, *O. Hamann & O. Seberg 1808* (C); Albemarle Is. (Isabela Is.), near the geyser, 870-890 m, 16 Jul 1972, *M. Hamann & O. Hamann 1839* (C); James Is. (San Salvador or Santiago Is.), "La Trágica", E part of the island, 350-360 m, 19 Aug 1972, *M. Hamann & O. Hamann 2094* (C); Indefatigable Is., Academy Bay, 2 May 1932, *J.T. Howell 9027* (B, CAS, G, US); Bartolomé Is., 18 Jul 1997, *P. Jaramillo 8628* (CDS); Abingdon Is. (Pinta Is.), 26 Jul 1990, *C.K. Mc. Mullen 7033* (CDS); Santa Cruz Is., 150 m, 28 May 1932, *H.J.F. Schimpf 20* (BM, CAS, CORD,

G, GH, M, MO, NY, P, US, Z); Albemarle Is., Villamil, bushes in woodland, 450–600 ft, 24 Aug 1905, *A. Stewart 3351* (CAS, GH, NY, MO, US); James Bay, occasional bushes above 1600 ft, *A. Stewart 3353* (CAS); Indefatigable Is., Academy Bay, 50-350 ft, 10 Apr 1930, *H.K. Svenson 248* (F, GH); Albemarle Is., Volcán Alcedo, 11-16 Apr 1984, *L. Tan Tuoc s.n.* (CDS); Indefatigable Is., along trail in Croton forest, 100 ft, 10 Feb 1939, *R.G. Taylor G11* (CAS, K, NY); Albemarle Is., W slope above Bahia Urvina, 1460 ft, 19 Jun 1974, *H.H. van der Werff 1255* (CAS, GH, QCA); Indefatigable Is., below C. Maternidad, mixed Pisonia forest, 1800 ft, Dec 1974, *H.H. van der Werff 1728* (CAS).

Cultivated
UNITED STATES OF AMERICA. California: Yolo Co., University of California, Department of Vegetable Crops greenhouse, seeds from Indefatigable Island, Galapagos Is., Ecuador (leg. C.M. Rick, Ac 1501), 1959, *P.G. Smith s.n.* (CORD 00088591, DAV).

21. Capsicum geminifolium (Dammer) Hunz.
COLOMBIA. New Grenada, May 1851, *J. Triana 3864* (BM); **Antioquia**: Betania, Vereda Pedral Arriba, orilla quebrada la Bramadora, 1700-1850 m, 4 Oct 2003, *J.C. Marrugo G. et al. 1021* (MEDEL); El Jardín, 5-6 km south of Jardín on Jardín-Riosucio road, 5º45'N, 75º50'W, 2390 m, 18 Jul 1995, *N.W. Sawyer 720* (CONN); Santa Fe de Antioquia, Sector San Judas (zona periurbana), via aledaña a la laguna de Oxidación, 6º32'42,11N, 75º49'00,60"W, 533 m, 28 Dec 2014, *D.A. Zapata C. 827* (JAUM); **Boyacá**: Chiquinquirá, 20 Jul 1909, *Frère Félix s.n.* (CORD 00086184, E, G, L, M) & 2 Oct 1907, *Frère Félix s.n.* (E); **Caquetá**: Florencia, en Gabinete, 2300-2450 m, 22 Mar 1940, *J. Cuatrecasas 8456* (COL, F, US); límite entre los Dptos. Caquetá-Huila, 1º52'49.7''N, 75º40'55.2''W, 2385 m, 17 Apr 2016, *A. Orejuela et al. 2688* (COL, CORD); **Cundinamarca**: La Vega-Facatativá Highway, 2100 m, 3 Sep 1947, *O. Haught 6134* (COL); Supatá, 19 km vía Pacho, 2400 m, 17 Jan 2007, *G. Morales 2914* (COL); Carretera a La Vega, 2200 m, 12 Nov 1949, *M. Schneider 893* (COL); Patasia en los bosques del Cerro Traga-arepas, 2100 m, 10 Feb 1948, *L. Uribe U. 1655* (COL); **Huila**: La Plata, Vereda Agua Bonita, Finca Merenberg, 1200-1800 m, 17 Jul 1975, *S. Díaz et al. 659* (COL); **Risaralda:** Mistrató, Corregimiento San Antonio del Chamí, 5º23'N, 75º54'W, 1500-1600 m, 26 Apr 1992, *J. Betancur et al. 3417* (COL).

ECUADOR. Azuay: The eastern Cordillera, 1-8 km N of the village of Sevilla de Oro, 8000-9000 ft, 27 Jul-21 Aug 1945, *W.H. Camp E-4312* (COL, CORD, F, MY, NY, US); Cuenca, road from Cuenca to Guayaquil (vía Molleturo/El Cajas), Tambaloma, 2º43'49"S, 79º24'46"W, 2560 m, 30 May 2007, *J.L. Clark 9806* (QCNE); Sevilla de Oro, old road 10-12 km N of the village, 2750-2850 m, 11 Sep 1976, *B. Øllgaard & H. Balslev 9348* (AAU, MO, NY); **Cañar**: Ventanillas, above Shoray on east slope of Cerro Yanguang Río Masar, 2º36'S, 78º34'W, ca. 2900 m, 5 Feb 1945, *F.R. Fosberg & F. Prieto 22763* (NY, P, US); **Chimborazo**: Cañon of the Río Chanchan, about 5 km, north of Huigra, 5000-6000 ft, 19-28 May 1945, *W.H. Camp E-3366* (CORD, MO, P, S, UC); vicinity of Huigra, mostly on the Hacienda de Licay, 4200 ft., 20 Aug 1918, *J. N. Rose & G. Rose 22258* (GH, NY, US); Carretero partidero de Pallatanga-San Juan-Llimbe, 2300-2720 m, 26 Feb 1987, *V. Zak 1752 & 1759* (QCA); **Cotapaxi**: road between Quevedo and Latacunga, 76.0 km E of Quevedo, 00°57'S, 79°01'W, 2300 m, 5 Apr 1983, *T.B. Croat 55803* (AAU, MO, NY);

around Pilalo, 00º57’S, 79º2’W, 2400 m, 3 Jul 1968, *L. Holm-Nielsen & S. Jeppesen 1240* (AAU, QCA, S); Cantón Sigchos, Triunfo Grande, 35 km de Sigchos, vía Sigchos-Las Pampas, finca del Sr. Galo Roballo, 00°32'04"S, 78°58'43"W, 2427 m, 2 Aug 2003, *J.E. Ramos et al. 6826* (CHEP, CUVC, MO, NY, QCNE) & *6834* (CUVC, NY); por la carretera en Triunfo Grande, 00º32'25"S, 78º58'18"W, 2500 m, 3 Aug 2003, *J. Ramos et al. 6909* (CUVC, NY); Bajo Triunfo Grande, bosque a mano izquierda de vía Triunfo Grande-Las Pampas, 00°30'55"S, 78°59'53"W, 2321 m, 9 Aug 2003, *J.E. Ramos et al. 7172* (CHEP, CUVC, MO, QCNE); **El Oro**: 10 km west of Piñas on road to Sta. Rosa, 950 m, 8 Oct 1979, *C.H. Dodson et al. 9157* (F); between Paccha and Puente Grande, passing over Montaña de Pueblo Viejo, 1830-2430 m, 26 Aug 1943, *J.A. Steyermark 54146* (F, VEN) *& 54149* (F, VEN); **Loja**: El Bosque, Quebrada Romerillos, 4º14'S, 79º10'W, Mar 1993, *A. Balcazar 74* (LOJA); Celica-Alamor road, c. 3 km W of Celica, 2200 m, 18 Feb 1985, *G. Harling & L. Andersson 22180* (NY); estribaciones de Celica, c. 3 km NE Guanchanamá, 2800 m, 21 Feb 1985, *G. Harling & L. Andersson 22344* (GB); 4 km S of Utuana, road to Tacamoros, "La Cruz Blanca", 2400 m, 16 Feb 1993, *G. Harling & B. Ståhl 26441* (QCA); Carretera Sopotepamba-Celica, Guanchanama, 1600-2600 m, 26 Feb 1988, *J. Jaramillo 10265* (NY); El Cisne-Zaruma, Km 2.9, 3º50’15’’S, 79º25’41’’W, 2340 m, 12 Dec 1994, *P.M. Jørgensen et al. 1401* (LOJA, QCA, QCNE, MO); Catamayo-Catacocha, Km 25, turnoff at Las Chinchas towards Piñas, Km 1.6, 3º57’08’’S, 79º28’53’’W, 2490 m, 13 Dec 1994, *P.M. Jørgensen et al. 1476* (MO, QCNE); El Cisne-Zaruma, Km 5.2, 3º50’16’’S, 79º26’14’’W, 1800 m, 14 Dec 1994, *P.M. Jørgensen et al. 1501* (LOJA, MO, QCA, QCNE); montains of Rio Campana, border of the Podocarpus National Park, 2250 m, 4º10'19"S, 79º08'59"W, 4 Jan 2001, *J.E. Madsen et al. 7601* (LOJA); Reserva El Bosque, 04º13'S, 79º09"W, 2200 m, 24 May 2002, *B. Merino & T. Delgado E-1569* (LOJA); Las Juntas, Feb 1883, *H.A.-C. Poortman 520* (P); Shucos, 03º55'43,6"S, 79º12'20,8"W, 2313 m, 6 Aug 2007, *V.M. Rivas & E. Guanín A. 113* (LOJA); PN Podocarpus, in disturbed area at base of El Sendero a Mirador, 4º15'S, 79º10'W, 2005 m, 24 Jun 1995, *N.W. Sawyer & J. Burneo 694* (LOJA, QCNE); ca. 3 km guard (reten) post at W end of Celica, 500 m W of rd jct to Zapotillo, 4º05’S, 79º50’w, 2120 m, 2 May 1991, *M. Spooner et al. 5036* (CORD); **Los Ríos**: Hacienda Mónica, 12 km E San Carlos, ca. 180 m, 23 Oct 1967, *B. Sparre 19319* (S); **Morona-Santiago**: Cima de la Cordillera del Cóndor, Centro Shuar Numpatkaim, 03º16'8"S, 78º19'6"W, 2700-2820 m, 22 Jul 2005, *T. Katán 349* (LOJA, QCNE); **Napo**: Quijos, faldas occidentales de la Cordillera de Guacamayos, margen derecha del Río Cosanga, 2400 m, 17 Oct 1990, *W.A. Palacios 6318 b* (MO); **Pichincha**: Mindo, *E.F. André 3839* (K); valley of Río Pilatón, near the bridge below Carretas, 7 Nov 1939, *E. Asplund 9713* (S); Mindo Loma Cloud Forest Reserve, km 73.5 via Calacali-La Independencia (3 km past the entrance to the village of Mindo), 00º0'44"S, 78º44'29"W, 1800 m, 23 May 2011, *J.L. Clark & C. Aulestia 12193* (NY, UNA); Quito, Reserva Orquideológica El Pahuma, carretera Calacalí-Los Bancos, Km 22, 00°01'42"N, 78°37'50"W, 2000 m, 19 Oct 1999, *T. Delgado & Grupo Post-Grado MO-QCNE 38* (MO, QCNE); road Santo Domingo-Quito, Cornejo Astorga (Tandapi), 1800 m, 7-10 May 1968, *G. Harling et al. 9226* (GB, MO); Aloag to Santo Domingo de los Colorados Hwy. near km 36, 2100 m, Jan 1990, *T. Mione 466* (CORD, MO); crescit in declivibus montis Carazou [Monte Corazón], pr. Miligally, May 1882, *L. Sodiro 114/82* (F fragment); between Km 37 and 50 along Río Saloya (between Volcán Atacaso and Volcán Pichincha), 1830-2430 m, Apr 1943, *J.A. Steyermark 52482* (VEN); **Sucumbíos**: Along road from Lumbaquí to La Bonita, 68.6 km N of main Baeza-Lago Agrio Road, 1 km S of Rosa Florida, 00°23'44"N

077°31'40"W, 1200 m, 21 Aug 2004, *T.B. Croat & G. Ferry 93747* (MO, QCNE); **Zamora-Chinchipe**: Tambo de Savanilla, 18 Dec 1876, *E.F. André 4570* (CORD, K); Estación Biológica San Francisco (EBSF), en los alrededores del casco de la Estación, 3°58'17,5''S, 79°04'45,2''W, 1865 m, 17 Aug 2017, *G.E. Barboza & S. Leiva González 4819* (CORD); EBSF, a 200 m del casco de la Estación, antes de cruzar el Río San Francisco, rumbo al camino del Atajo, 3°58'19.9''S, 79°04'43.1''W, 1810 m, 17 Aug 2017, *G.E. Barboza & S. Leiva González 4845* (CORD, CTES, HAO, LOJA, SI,); EBSF, a 300 m del casco de la Estación, antes de llegar al río, 03º58'20''S, 79º04'43''W, 1830 m, 17 Aug 2017, *G.E. Barboza & S. Leiva González 4852* (CORD); same locality, 17 Aug 2017, *S. Leiva González & G.E. Barboza 6576* (CORD, HAO); near cabin of S. León, W of Escuela Byron Jiménez, just S of Las Pircas, region of Guaramizal, 4º46'31"S, 79º11'50"W, 2100 m, 29 Mar 2005, *L. Bohs et al. 3346* (QCNE); Reserva Ecológica Tapichalaca (Fundación Jocotoco) between Yanganá and Valladolid, along Sendero Tangano starting from Jocotoco station, 04º29'42"S, 79º07'55"W, 2500 m, 3 Apr 2005, *L. Bohs et al. 3401* (QCNE); Fundación Arco Iris, between Loja and Zamora, trail from field station to Rio San Francisco, 3º59'20"S, 79º0535"'W, 2200 m, 5 Apr 2005, *L. Bohs et al. 3421* (NY, QCNE); ruta Yanganá-Valladolid, base de Reserva Joco-Toco, 4º28'08''S, 79º08'44''W, 2602 m, 15 Jul 2012, *R. Deanna & S. Leiva González 3* (CORD); límite de Parque Nacional Podocarpus (PNP), a 600 m de desvío de la ruta Loja-Zamora, 3º59'12''S, 79º03'22''W, 2251 m, 16 Jul 2012, *R. Deanna & S. Leiva González 11* (CORD); PNP, sector El Trigal, 03º58'43''S, 79º08'27''W, 2694 m, 22 Jan 2013, *R. Deanna & S. Leiva González 99* (CORD); carretera nueva Loja-Zamora, 3°58'S, 78°06'W, ca. 2070 m, 19 Mar 1989, *A. Freire Fierro 1243* (QCA); Estación Biológica San Francisco, 1830 m, 19 Apr 2000, *A. Freire Fierro & M. Asanza 3087* (QCNE); road Loja-Zamora, Km 25, 2200 m, 18 Apr 1974, *G. Harling & L. Andersson 13680* (GB, MO); Reseva San Francisco, road Loja-Zamora, ca. 35 km from Loja, 3º58'S, 79º04'W, 1790 m, 14 Apr 2007, *J. Homeier 2555* (LOJA, GOET, NY); road between Loja & Zamora, ca. Km 16-18, 2500-2650 m, 31 Dec 1978, *J.L. Luteyn et al. 6595* (NY); San Francisco, 3º59'08"S, 79º04'23"W, 1900 m, 17 Apr 2002, *B. Merino & T. Delgado E-1340* (LOJA); límite del PNP, desvío de la ruta principal en el límite entre Prov. Zamora Chinchipe/Loja, 2750 m, 15 Nov 2011, *C.I. Orozco et al. 3922* (COL, CORD, QCA); pasando Yanganá rumbo a Valladolid, 2400 m, 16 Nov 2011, *C.I. Orozco et al. 3931* (COL, CORD, QCA) & *3934* (COL); rumbo a Valladolid, desde Yanganá, 2573 m, 16 Nov 2011, *C.I. Orozco et al. 3944* (COL, CORD); area of Estacion Científica San Francisco, 3º58'S, 79º04'W, 1830 m, 15 May 2008, *F.A. Werner 2594* (LOJA).

PERU. Amazonas: Luya, Distr. Camporredondo, Tullanya, 1700-2000 m, 26 Nov 1996, *J. Campos de la Cruz et al. 3031* (MO, MEXU, NY, USM); Bongará, carretera hacia Fernado Belaunde Terry, 5º41'47''S, 77º47'51''W, 2152 m, 25 Apr 2014, *S. Leiva González & G.E. Barboza 5647* (CORD, HAO); Bagua, Aramango. Catarata Numparket, 5°29'33"S, 78°19'59"W, 2000 m, 13 Dec 2001, *R. Vásquez et al. 27256* (MO); **Cajamarca**: San José de Lourdes, alrededores de Camaná, 1850 m, 22 Mar 1997, *J. Campos de la Cruz & S. Corrales 3603* (MO, USM); carretera Cutervo-La Capilla, km 1550m, 6º16'54''S, 78º50'18''W, 2463 m, *R. Deanna & S. Leiva González 77* (CORD); San Andrés de Cutervo, carretera entre San Andrés y Santo Tomás, Km 12, 2320 m, 7 Aug 1987, *C. Díaz S. et al. 2548* (MO); Santuario Nacional Tabaconas-Namballe, Pampa Limón, 1980 m, 23 Nov 1998, *C. Díaz et al.10120* (MO, USM); Hualgayoc, Hacienda Taulis, just below

Palmito, 2575 m, 31 Aug 1964, *P.C. Hutchinson & K. von Bismarck 6396* (USM); Santa Cruz, ruta Monteseco-Chorro Blanco, 1480 m, 20 Jan 1996, *S. Leiva González et al. 1740* (CORD fragment, HUT, NY); San Miguel, Monte Seco, 1800 m, 10 Feb 1986, *S. Llatas Quiroz 1745* (F, NY); Huarango, Nuevo Mundo, Caserío Rey del Oriente, 1800 m, 26 Jul 1997, *E. Rodríguez R. & J. Campos de la Cruz 1811* (HUT, MO); Nuevo Mundo, Caserío Pisaguas, 1700-1800 m, 12 Nov 1997, *E. Rodríguez R. 1928* (HUT, MO); Tabaconas, caserío La Bermeja, entre camino La Bermeja-Huaquillo, 1700-1940 m, 20 Nov 1997, *E. Rodríguez R. & R. Cruz A. 2046* (HUT, MO, USM); Chorro Blanco (Bosque Monteseco), 2000 m, 24 May 1987, *A. Sagástegui A. et al. 12998* (HUT); **Cuzco**: Urubamba, San Miguel, Urubamba Valley, 1800 m, 9 Jun 1915, *O.F. Cook & G.B. Gilbert 1165* (US); La Convención, Echarati Tingkanari, Camp. III, trocha Paititi, 12º15’46’’S, 72º05’53’’W, 2179 m, 11-12 May 2004, *N. Salinas et al. 7249* (USM); **Huánuco**: Huánuco, Carpish, ca. 2700 m, 16 Aug 1940, *E. Asplund 13115* (S); Pachitea, Cushi, 5000 ft, 19-23 Jun 1923, *J.F. Macbride 4831* (F); Churubamba, trail Puente Durand to Éxito, Mt. Santo Toribio, 1500-1800 m, 26 Sep 1926, *Y. Mexia 8246* (F, NY); Gasa, 12 km west of Puente Durand, N of Huánuco, Valley of Chinchao Río, 1300 m, 5 Nov 1938, *H.E. Stork & O.B. Horton 9852* (F, GH); road from Huánuco to Tingo María, Abra Carpish just north of the tunel, 2720-2800 m, 22 Mar 2001, *M. Weigend et al. 5285* (NY); **Lambayeque**: Ferrenafe, Bosque de Chinama, 2300-2700 m, 15 Aug 1988, *A. Cano 2278* (F); **Pasco**: Oxapampa, Parque Nacional Yanachaga-Chemillen (PNYCh), Sector Tunqui, riberas del Río Muchuymayo, alrededores del hito PNYCh, 10°17'22"S, 75°31'05"W, 1790 m, 22-24 Oct 2008, *M. Cueva 270* (HOXA, HUSA, MO, USM); PNYCh, Sector San Daniel, 10°26'27"S, 75°26'30"W, 2240 m, *M. Cueva 480 & 485* (HOXA, HUSA, HUT, MO, USM); PNYCh, camino entre el Refugio Abra Esperanza y la tercera miniestación metereológica, 10°31'53,7"S, 75°20'41,9"W, 2690 m, 21-22 Apr 2009, *M. Cueva 569* (HOXA, HUSA, HUT, MO, USM); PNYCh, San Daniel, camino a laguna San Daniel, 10º25’40’’S, 75º26’09’’W, 2000-3100 m, 23 Feb 2009, *P. Gonzáles 341* (CORD, E, USM); PNYCh, sector San Alberto, camino del refugio El Cedro al Abra Esperanza, 10º32’43’’S, 75º21’30’’W, 2420-2700 m, 3 Oct 2007, *L. Hernani A. & A. Peña C. 379* (USM); PNYCh, quebrada San Alberto, Refugio El Cedro, 10°32'S, 75°21'W, 2450 m, 27 Jul 2002, *A. Monteagudo et al. 3475* (HOXA, MO, USM); sector San Daniel, cerca del Hito del PNYCh, 10°26'46.5"S, 75°26'17.8"W, 2200 m, 17 Aug 2005, *A. Monteagudo et al. 9470* (HOXA, MO); PNYCh, zona de amortiguamiento, parte media de la Quebrada San Luis, 10°33'55"S, 75°20'43"W, 2200-2360 m, 18 Sep 2007, *A. Monteagudo et al. 15087* (HOXA, MO); PNYCh, Abra Yanachaga, 10°22'46,3"S, 75°27'42,9"W, 2930 m, 3 Aug 2005, *E. Ortiz & R. Francis J. 736* (HOXA, MO); PNYCh, Sector Grapanazu, 10°26'12"S, 75°23'13"W, 2310 m, 11 Oct 2003, *R. Rojas et al. 1642* (HOXA, MO); PNYCh, Sector San Alberto, 10º32'45"S, 75º21'26"W, 2510 m, 26 Nov 2007, *E. Tepe et al. 2318* (NY, USM, UT); Huancabamba, PNYCh, la Colmena-trocha Erica, 10°25'43"S, 75°26'08"W, 3460 m, 20 Aug 2008, *L. Valenzuela et al. 11575* (HOXA, MO, USM); PNYCh, Sector San Alberto, 10°32'S, 75°21'W, 2600 m, 20 Jan 2003, *R. Vásquez & R. Francis 27853* (HOXA, MO) & 14 Mar 2003, *R. Vásquez & R. Francis 28032* (HOXA, MO); PNYCh, sector San Daniel, 10°26'35"S, 75°26'16"W, 2200-2500 m, 10 Mar 2006, *R. Vásquez et al. 31019* & *31021* (HOXA, MO, USM); same locality, 10°26'46"S, 75°27'01"W, 2095 m, 25 Feb 2009, *R. Vásquez & L. Valenzuela 35377* (HOXA, MO, USM); **Piura**: Huancabamba, Canchaque, "Chorro Blanco", 1600 m, 18 Jan 1988, *C. Díaz S. et al. 2767* (MO); Ayabaca, alrededores

del Pueblo de Huamba, 2310 m, 3 Jun 1997, *S. Leiva González et al. 2018* (F, MO, NY); base del Bosque Cuyas, 1900 m, 19 Jan 2004, *S. Leiva González et al. 2788* (NY).

22. Capsicum hookerianum (Miers) Kuntze

ECUADOR. Without locality, *J. Miers* s.n. (BM 000072904); **Guayas**: Guayaquil, shady ticket on hillside W of the town, 26 Jan 1955, *E. Asplund 15241* (B, NY, S, Z); near the highway to Daule W of Guayaquil, 30 Jan 1955, *E. Asplund 15280* (S); Cerro Azul W of Guayaquil, 8 Feb 1955, *E. Asplund 15363* (B, NY, S); Naranjal, Parroquia Taura, Reserva Ecológica Manglares-Churute, Cerro Perequetre Chico, 2°27'S, 79°40'W, 160-300 m, 26 Feb 1992, *C.E. Cerón et al. 18258* (MO, NY, QCNE); Guayas, 2º09’57’’S, 80º07’58’’W, 200-300 m, 21 Jan 1997, *J.L. Clark et al. 3831* (MO, QCNE); Bosque Protector Paraíso, 200 m, 2°12'S, 79°57'W, 23 Jan 1994, *X. Cornejo S. & C. Bonifaz 1353* (GUAY); Bosque Protector Cerro Blanco, 300 m, 2°10'S, 79°58'W, 20 Feb 1994, *X. Cornejo S. & C. Bonifaz 1778* (GUAY); Urbanización Capeira, 50 m, 2°02'S, 79°57'W, 25 Jun 1994, *X. Cornejo S. & C. Bonifaz 2958* (GUAY); Cerro Azul, enter at Chongon, Km 24 Guayaquil-Salinas, 7 km N in tropical dry forest, 2°09'S, 79°59'W, 100 m, 18 Mar 1980, *C.H. Dodson 9617* (MO); Capeira, km 21, Guayaquil to Daule, 2°00'00"S, 79°52'48"W, 20-200 m, 11 Feb 1982, *C.H. Dodson 12331* (MO); Cerro Azul, 50-300 m, 2º15’S, 80º00’W, 17 Jan 1991, *A.H. Gentry & C. Josse 72356* (MO, QCNE); comienzo del camino de la Hacienda Barcelona, Km 12 de Guayaquil, 2°10'S, 79°58'W, 2 Nov 1962, *A. Gilmartin 554* (GUAY); Pedro Carbo, 100 m, 14 Jan 1940, *O. Haught 3041* (AA, F, S, US); hillsides of Guayaquil, 11 Jan 1943, *O. Haught 3514* (US); Cerro Azul, near summit, just below microwave towers, 2°10'S, 79°57'W, 420 m, 26 Feb 1996, *D. Neill & T. Núñez 10491* (BM, MO, MY, QCNE); Guayaquil, Cerro Blanco, vía a la Costa, Km 15, 2°10'S, 79°58'W, 400 m, 17 Mar 1992, *W.A. Palacios & D. Rubio 9956* (MO, QCNE); Guayaquil, carretera a Salinas, Km 15, 2°10'S 79°58' W, 400 m, 21 Jan 1992, *D. Rubio & G.A. Tipaz 2361* (CHEP, MO, QCNE,); Cerro Azul, carretera a Salinas, Km 13, 2°22'S 80°19'W, 100 m, 7 May 1992, *G.A. Tipaz et al. 865* (MO, QCNE); **El Oro**: Bosque Petrificado Puyango, sendero del Petrino, 280 m, 3°52'S, 80°04'W, 13 Apr 1996, *X. Cornejo S. & C. Bonifaz 5009* (GUAY); **Loja**: Macará, desde Macará, carretera Sabanilla-Saucillo, km 631.5-632, 4º02'32"S, 80º09'50"W, 692 m, 6 May 2017, *G.E. Barboza & S. Leiva González 4826* (CORD); desde Macará, desvío Relleno Sanitario, carretera Macará-Sapotillo, km 10, 4º19'56"S, 79º59'34"W, 540 m, 6 May 2017, *G.E. Barboza & S. Leiva González 4831* (CORD); Celica-Zapotillo Road, c. 5-6 km S of Sabanilla, 600 m, 14 Apr 1980, *G.W. Harling & L. Andersson 18283* (MO); Macará-Zapotillo road, km 10-13, 500-600 m, 16 Apr 1980, *G.W. Harling & L. Andersson 18316* (MO); 2 km W Tambo Negro on Macará-Sozoranga road, 4°23'S, 79°52'W, 600 m, 8 Mar 1991, *M. Kessler 2712* (QCA); **Manabí**: Bahía de Caráquez, frente a la Isla Corazón, 180-200 m, 00°40'S, 80°22'W, 18 Feb 1994, *X. Cornejo S. 1727* (GUAY); Cerro Montecristi, 300-400 m, 18 Jul 1986, *T.C. Plowman & P.W. Alcorn 14335* (CORD, F, MO, QCA).

PERU. Without locality, *H. Ruiz & J.A. Pavón s.n* (MA); **Tumbes**: Zarumilla, Pampas de Hospital El Caucho, quebrada "Faical", entre "Est. Biol. El Caucho" y Campo Verde, 370 m, 21 Jan 1989, *C. Díaz S. et al. 3201* (MO, USM); Contralmirante Villar, Distr. Casitas, Parque Nacional "Cerros de Amotape", Quebrada del Plátano, 2 May 1990, *C. Díaz S. et al. 4032* (MO, USM); Zarumilla, borde de la carretera entre campamento de evaluación

permanente y parcela V "Campo Verde", 03°50'29"S, 80°15'30"W, 500 m, 10 Feb 1993, *C. Díaz S. et al. 6242* (MO, USM); Matapalo, El Caucho-Campo Verde, 700 m, 16 Jan 1995, *C. Díaz S. et al. 7417* (MO, USM); carretera entre "Naranjal" y "Campo Verde", 03°51'S, 80°10'W, 650 m, 21 Jan 1995, *C. Díaz S. et al. 7474* (MO); carretera entre "El Caucho" y Quebrada Naranjal, 03°50'39"S, 80°18'53"W, 650 m, 25 Jan 1995, C. *Díaz S. et al. 7512* (MO, USM); Cerros de Amotape, Quebrada Los Conejos, SE of Cherrelique, 4º09'S, 80º37'W, 820-850 m, 9 Jun 1987, *A.H. Gentry & C. Díaz S. 58262* (MO); Contralmirante Villar, Huásimo, Quebrada Ucumares, 550 m,12 Feb 1976, *T.C. Plowman 5439* (MO); Matapalo, Bosque Nacional de Tumbes, about 120 km of Campo Verde, 600-800 m, 23 Dec 1967, *D.R. Simpson & J. Schunke V. 469* (COL, CORD fragment, F, G, NY, US); región de Pampa Hospital, 400 m, 10 Feb 1947, *O. Velarde Núñez 326* (US); Tumbez, mountains SE of Hacienda La Choza, 900-1000 m, 27-28 Feb 1927, *A. Weberbauer 7684* (CORD, F);

23. Capsicum hunzikerianum Barboza & Bianch.

BRAZIL. **São Paulo**: Cunha, Parque Estadual (PE) da Serra do Mar-Núcleo Cunha, trilha do Rio Bonito, 12 Nov 1996, *L.B. Alburquerque et al. 49* (ESA, SP, UEC); Salesópolis, Estación Biológica de Boracéia, a unos 300 m del casco de la EBB, 23º39'19''S, 45º53'24''W, 840 m, 30 Mar 2019, *G.E. Barboza 5041* (CORD); Estação Biológica da Boracéia-trilha da Bomba, 840 m, 23°39'15''S, 45°53'23''W, 6 May 1999, *L. Bianchetti et al. 1537* (CEN); Biritiba Mirim, Estação Biológica de Boracéia, 23°38'-23°39'S, 45°52'-45°53'W, 890-950 m, 14 Nov 1983, *A. Custódio Filho 1849* (CEN, CEPEC, SP, SPSF); Biritiba Mirim, Estação Biológica de Boracéia, 23°38'-39'S, 45°52'-53'W, 890-950 m, 14 Nov 1983, *A. Custódio Filho 1860* (CEN, SP), 24 Nov 1983, *A. Custódio Filho 1914* (CEN, SP, SPSF) & 9 Dec 1983, *A. Custódio Filho 2056* (CEN, COL, SP, SPSF); Casa Grande, Reserva Florestal, Guaratuba, 23°39'S, 45°52'W, 890-950 m, 4 Feb 1988, *F. Custódio Filho & A. Custódio Filho 448* (SPSF); Parque Estadual da Serra do Mar, estrada intermediária km 45, 23°38'16.7''S, 45°41'56.6'', 25 Apr 2000, *N.M. Ivanauskas et al. 4631* (BHCB, RB); Estação Biológica de Boracéia, 22 Nov 1957, *M. Kuhlmann 4311* (CEN, SP 79300 & 79300a, SPSF); Boracéia, Estação Experimental de Quina (atual Estação Biológica de Boracéia), 27 Nov 1940, *A.S. Lima & L. da Silva s.n.* (BHCB 77035 & 77039, IAC 585 & 5870, SP 5870, LIL 429724); Salesópolis/São Sebastião, estrada da Petrobras, SP-090, 23°35'57.8''S, 45°42'21.6''W, 1075 m, 16 Jan 2006, *J.A. Lombardi et al. 6217* (BHCB); Estação Biológica de Boracéia, perto do Rio Coruja, 29 Nov 1966, *J. Mattos & M. Mattos 14254* (CORD, SP); Estação Experimental Boracéa, 21 Nov 1940, *L. da Silva s.n.* (BHCB 77035, CORD 00003943, SP 48598 & 48603); PE da Serra do Mar-Núcleo Cunha, trilha da Pedra ao Indalá, 23º14'45"S, 44º59'36"W, 1070 m, 16 Dec 1996, *J.P. Souza et al. 970* (ESA, SP, UEC); Estação Biológica de Boracéia, trihla para o mirante do Guaratuba, 818-848 m, 24 Oct 2007, *J.R. Stehmann et al. 4830 & 4831* (BHCB, RB).

24. Capsicum lanceolatum (Greenm.) Morton & Standley

GUATEMALA. Alta Verapaz: montains along road between Tactic and the divide on road to Tamahu, 1500-1600 m, 1-7 Apr 1941, *P.C. Standley 91469* (F, G, GH); **Baja Verapaz**: Unión Barrios, west of Km 160/161, 31 Aug 1975, *C.L. Lundell & E. Contreras 19773* (CAS, MO, S); Niño Perdido, Cerro Verde, 15º10'38''N, 90º11'02''W, 3 Dec 1976, *C.L.*

Lundell & E. Contreras 20417 (CAS, MO, S); Niño Perdido, on San José road bordering Arroyo El Coracol, 24 May 1977, *C.L. Lundell & E. Contreras 20971* (CAS, F, MEXU, MO); Biotopo del Quetzal, 1630 m, 22 Jul 1988, *E.M. Martínez S. et al. 23039* (MEXU); Sierra de las Minas, 3 km southeast of Purulhá, 1800, 2 Jan 1974, *L.O. Williams et al. 43125* (EAP, F) & *L.O. Williams et al. 43612* (EAP, F); **Chimaltenango**: slopes of Volcán Fuego, 1200-1600 m, 20 Sep 1942, *J.A. Steyermark 52093* (F, G, GH); **Huehuetenango**: Sierra de los Cuchumatanes, Xoxlac, 1650-2500 m, 15º59'01''N , 91º21'58''W, 17 Jul 1942, *J.A. Steyermark 48958* (F, MO, US); **Izabal**: Morales, Sierra Caral, along trail heading south from research station (15.40695, -88.69598) to summit of Sierra Caral (15.38749, -88.69357), 700-1000, 14 Jun 2011, *K. Watson et al. 39* (NY); **Quezaltenango**: Palmar, 14 Oct 1934, *A.F. Skutch 1450* (F, GH, US); Finca Pirineos, below Santa María de Jesús, 1350 m, 11 Mar 1939, *P.C. Standley 68235, 68244 & 68294* (EAP, F, US); between Finca Pirineos and Patzulín, 1200-1400 m, 9 Feb 1941, *P.C. Standley 86930* (F, US); along Quebrada San Gerónimo, 1-2 Jan 1940, *J.A. Steyermark 33357* (F); Finca Pirineos, below Santa María de Jesús, 1350 m, 1-2 Jan 1940, *J.A. Steyermark 33429* (F); **Sacatepequez**: Barranco above Dueñas, 1600-1800 m, 21 Jan 1939, *P.C. Standley 63153* (F, NY); **San Marcos**: near Aldea Fraternidad, between San Rafael Pie de la Cuesta and Palo Gordo, 1800-2400 m, 10-18 Dec 1963, *L.O. Williams et al. 25674* (EAP, F, GH) & *25950* (GH); outer slopes of Volcán Tajumulco, 8-10 km E of San Marcos, 2300 m, 1964/1965, *L.O. Williams et al. 26924* (EAP, NY, US, W); **Solala**: Volcán Santa Clara, 1250-1400 m, 8 Jun 1942, *J.A. Steyermark 47328* (F); slopes of Volcán Atitlán, 20 Jun 1942, *J.A. Steyermark 47911* (F); **Suchitepequez**: SW lower slopes of Volcán Zunil, in vicinity of Finca Montecristo, 1200 m, 31 Jan 1940, *J.A. Steyermark 35237* (F, MO); Volcán Zunil, vic. Finca Las Nubes, 500-800 m, 2 Feb 1940, *J.A. Steyermark 35422* (US); 1 ½- 2 miles west of Finca El Naranjo, 1250 m, 1 Jun 1942, *J.A. Steyermark 46797* (F); **Zacapa**: summit of Sierra de las Minas, 2500 m, 13 Oct 1939, *J.A. Steyermark 29933* (F).

HONDURAS. **Comayagua**: cumbre de la montaña Reserva Biológica Cordillera de Montecillos, al lado del camino el Danto a Jesús de Otoro, San Juanillo, 2020 m, 14°30'N, 87°53'W, 2 Mar 1991, *P.R. House 866* (EAP, HEH, MO, TEFH); **Cortés**: ca. 30 m above Río de Cusuco, along trail from new park station building to Cerro Cusuco, 15°30'N, 88°13'W, 1640 m, 19 Mar 1993, *R.J. Evans 1469* (EAP, MO); Cuenca Qbr. Cantiles, nuclear zone of the Cusuco National Park, 1940 m, 22 Mar 1993, *T. Hawkins & D. Mejía 702* (EAP, MO, NY); **La Paz**, Olancho, La Unión, Montaña La Muralla, 200 km al noroeste de Tegucigalpa, 1320 m, 17 Mar 2000, *J. Araque 462* (EAP); Montaña Verde, Aldea las Marías, Cordillera Guajiquiro, 2100 m, 23 May 1964, *A. Molina R. & A.R. Molina 14053* (EAP, F, NY); Aldea Las Marías, Cordillera Guajiquiro, 23 May 1964, *P.C. Standley 14077* (EAP).

MEXICO. Chiapas: 45 km NE of Huiztla, road to Motozintla, 1900 m, 17 Nov 1971, *D.E. Breedlove & A.R. Smith 22629* (CAS, ENCB, MO); Selva Negra, 10 km above Rayón Mezcalapa, along road to Jitotol, 1700 m, 13 Jul 1972, *D.E. Breedlove 26097* (CAS, ENCB, MO); 45-50 km NE of Huixtla along road to Motozintla de Mendoza, 1900 m, 28 Dec 1972, *D.E. Breedlove & R.F. Thorne 31031* (CAS, MO); Siltepec, above Siltepec on the road to Huixtla, 2000-2400 m, 18 Jan 1973, *D.E. Breedlove & A.R. Smith 31992* (CAS); La Independencia, third ridge along logging road from Las Margaritas to Campo Alegre, 16°28'31"N, 91°49'25"W, 2300 m, 6 May 1973, *D.E. Breedlove 34836* (CAS, MO); east of

Laguna Tzikaw, Monte Bello National Park, 1300 m, 13 May 1973, *D.E. Breedlove 35237* (CAS, MO); La Trinitaria, 4 km E of Laguna Tsiskaw [Tziscao] near Dos Lagos, 16°05'35"N, 91°38'12"W, 1300 m, 19 Oct 1974, *D.E. Breedlove 38789* (CAS, MO); La Independencia, from Las Margaritas to Campo Alegre, 2300 m, 24 Oct 1976, *D.E. Breedlove 41082* (CAS, MEXU, MO); La Trinitaria, near Cinco Lagos, 16°05'56"N, 91°40'03"W, 1372 m, 5 Oct 1981, *D.E. Breedlove 53338* (CAS, MO); La Trinitaria, 10 km ENE of Dos Lagos above Santa Elena, 16°06'16"N, 91°33'40"W, 1170 m, 28 Dec 1981, *D.E. Breedlove 56499* (CAS, MO); Cinco Lagunas, Lagos de Montebello National Park, 1600 m, 24 May 1988, *D.E. Breedlove 68656* (CAS); La Trinitaria, a 5 km al oeste de lago Tziscao, 23 Jun 1982, *E.F. Cabrera C. & H. de Cabrera 2939* (MEXU); 1 km al E de Tziscao, sobre el camino a Santa Elena, Parque Nacional Lagos de Montebello, 16°04'48"N, 91°39'00"W, 1489 m, 1 Oct 1983, *E.F. Cabrera C. & H. de Cabrera 5819* (MEXU, MO); El Rosario, 8 miles S of Motozintla, 1800 m, 15º19'12''N, 92º17'24''W, 10 Jul 1977, *T.B. Croat 40738* (MO); Rayón, a orillas del cerro asociada con *Lobelia* y *Calceolaria*, en el Mirador el Caminero en la Selva Negra, 8 Jul 1988, *M.A. Magaña 2006* (MEXU); Ocosingo, a 5 km al S Frontera Echeverría sobre la orilla del Río Usumacinta, 80 m, 4 Dec 1984, *E. Martínez S. 9066* (MEXU); Mt. Tacaná, 1000-2000 m, Aug 1938, *E. Matuda 2438* (GH, MEXU, MICH); Cascada Siltepeg, 1600 m, 1 Mar 1945, *E. Matuda 5177* (MEXU, MO); Trinitaria, Ejido Benito Juárez, anexo Tzicao, 10 Jul 1984, *A. Méndez Girón 7745* (MO); Rayón, 1900 m, 8 Jul 1988, *A. Miguel 2006* (MEXU); Rayón, 9 miles northwest of Pueblo Nuevo Solistahuacán along the road between Rincón Chamula and Rayón, 17°30'N, 93°40'W, 5800 ft, 30 Sep 1980, *H. Mill 614* (CAS); Chihuahua, Finca Irlanda, Aug 1913, *C.A. Purpus 6976* (BM, F, GH, K, NY, MO, UC, US); San Cristóbal de las Casas, 10 Oct 1978, *W. Schwabe & W. Kailing s.n.* (MEXU); a 4-5 km al E de la Laguna de Tziscao, camino a Las Margaritas, 23 Sep 1983, *O. Téllex 7154* (MEXU); Rayón, 9 miles northwest of Pueblo Nuevo Solistahuacán along the road between Rincón Chamula and Rayón, 5800 ft, Sep 1971, *R.F. Thorne & E. Lathrop 41699* (CAS); Ejido Cuauhtémoc, Km 17, 5 Aug 1984, *A.S. Ton 7803* (MEXU, MO); Rancho Nuevo, 4 Km al oeste de El Rosarie, 5 Aug 1984, *E. Ventura V. & E. López 4208* (MEXU, MO); Motozintla, Llano Grande, 4 km al noroeste, 2900, 4 Mar 1987, *E. Ventura V. & E. López 4381* (F, MEXU, NY); Unión Juárez, 8 km al sur de Unión Suárez, 3000 m, 9 Aug 1988, *E. Ventura V. & E. López 5472* (F); **Oaxaca**: Tuxtepec, Presa Temazcal, camino a los vertederos, 90 m, 8 Aug 1985, *L. Cortés & R. Cortés C. 30* (MEXU); San Miguel, Distrito Juchatan, Benito Juárez rumbo a cerro Salomón, 10 Jul 2008, *J. Lucas 135* (IEB); San Miguel Chimalapa, filo de La Culebra, entre Cerro Guayabitos y Cerro Salomón, 16°45'N, 94°11'W, 1700 m, 25 Nov 1985, *J. Solomón Maya 2570* (MEXU); Santa María, Mpio San Miguel Chimalapa, Cabecera del arroyo de paso por El Mamey, al ONW del Cerro El Reten, ca. 24 km en línea recta al NNE de Zanatepec, 1600-1800 m, 2-4 Jul 1986, *J. Solomón Maya 3521* (MEXU); cabecera del cañón Hierba Santa, faldas del Cerro Guayabitos, ca. 4 km en línea recta al NO de Benito Juárez, 16°44'N, 94°10'W, 1500-1700 m, 2 Aug 1986, *J. Solomón Maya J. 3704* (MEXU); San Miguel, Cerro El Retén, cerca del paraje palmero "El Progreso" y la vereda El Progreso-El Salto, ca. 23 km al NE de Zanatepec, 10 Sep 1986, *J. Solomón Maya 3867* (MEXU); Totontepec, Mixe, Mirador de Amatepec, 7 Km al N de Totontepec, carr. a Choapan, 1930 m, 27 Oct. 1987, *R. Torres C. & L. Cortes A. 10360* (CAS, MEXU, MO); Cerro Sabinal, ca. 2 km al SO de cerro Guayabitos, al O de la cima del cerro, 16°44'N, 94º11'30" W, 1500 m, 21 Dec 1984, *T. Wendt et al. 4685* (NY); Cerro Salomón, ca. 2 km en línea recta al NNO del Cerro

Guayabitos, ca. límite con el municipio de Santa María Chimalapa, 16°45'N, 94°11'30"W, 1850 m, 23 Dec 1985, *T. Wendt et al. 5173* (NY); **Veracruz**: Catemaco, Cerro Pipiapan, 17 May 1986, *R. Acosta P. 1405* (MEXU, XAL, WIS); Soteapan, lado oeste de la Sierra de Santa Marta, 18º26'N, 94º58'W, 1100 m, 28 May 1972, *J.H. Beaman 6014* (CORD, F, MEXU); Soteapan, Sierra de Santa Maria, al norte de Ocotal Chico, 4 Nov 1986, *J.I. Calzada 12646* (IEB, MEXU, XAL); San Pedro Soteapan, 3 km N a Santa Martha, 1350 m, 12 Nov 1980, *F. Vázquez B. & O. Hernández 45* (UC); Atzalan, La Calavera, 1100 m, 2 Apr 1970, *F. Ventura A. 798* (CORD, F, MEXU); Atzalan, La Calavera, 1000 m, 7 Jul 1975, *F. Ventura A. 11600* (CORD, ENCB, F, MEXU).

Cultivated

AUSTRIA. **Wien**: cult. HBV (Universität Wien), semillas compradas a La Palma Co. (España), 10 Mar 2020, *C. Carrizo García 73* (WU).

25. Capsicum longidentatum Agra & Barboza

BRAZIL. **Bahia**: Itatim, cerca de Pedra Resoles, Morro detrás del Restaurant Resoles, 12°44′21.8″S, 39°47′01.8″W, 295 m, 3 Apr 2009, *M.F. Agra et al. 7083* (CORD, JPB); Morro do Quixaba, 12°44′21.3″S, 39°47′33.9″W, 273 m, 3 Apr 2009, *M.F. Agra & G.E. Barboza 7086* (CORD, JPB); Boa Nova, PARNA Boa Nova, Lagoa de D'Anta, acceso pela BR 030 em direção a BR 116, 14°21'25''S, 40°12'46''W, 900 m, 8 Feb 2013, *A.M. Amorim et al. 8292* (CEPEC, RB); Ibiquera, 12°27'57''S, 40°58'54''W, 709 m, *S.F. Conceição et al. 507* (HUEFS); Irecê, CETEP, 25 Feb 2016, *G. Costa & A.C.S. Moraes 1744* (HUEFS); Milagres, Fazenda Bastião (cerca de 114 km antes de Milagres) na BR 116, 18 Dec 1996, *Equipe Projeto Abelhas s.n.* (HUEFS 240347); Morro do Chapéu, entorno oeste do Parque Estadual de Morro do Chapéu, 11°20'34''S, 41°21'45''W, 726 m, 1 Dec 2006, *F. França et al. 5556* (BHCB, HUEFS); Lagedo Bordado, 11°15'28''S, 41°9'40''W, 690 m, 5 May 2007, *J.M. Gonçalves et al. 175* (HUEFS); Lapão, Morro Pelado, 11°24'22''S, 41°49'22''W, 787 m, 27 Oct 2009, *M.L. Guedes et al. 16183* & *16187* (ALCB, HUEFS); Serra de Itiúba, about 6 km E of Itiúba, 10°41'S, 39°48'W, 500 m, 19 Feb 1974, *R.M. Harley 16184* (CEPEC, RB); Morro do Chapeu, APA Gruta dos Brejões/Vereda do Romão Gramacho, 31 Oct 2009, *R.F. Machado 368* (HUEFS); Morro do Quixaba, 12°44'S, 39°47'W, 276 m, 14 Dec 1996, *E. Melo et al. 1859* (HUEFS); Morro do Bastião, 12°45′12″S, 39°46'W, 282 m, 25 Jan 1997, *E. Melo et al. 1920* (HUEFS, JPB); Fazenda São João Brejões, 11°16'1''S, 41°5'21''W, 720m, 14 Apr 2007, *E. Melo et al. 4693* (BHCB, HUEFS); Iaçu, inselbergue Morro da Garrafa, 12°45'18''S, 39°51'48''W, 330 m, 26 Dec 2008, *E. Melo & B. Marques da Silva 6081* (HUEFS); Morro Pelado, próximo à Torre, 11°24'22''S, 41°49'21''W, 790 m, 25 Oct 2009, *E. Melo et al. 6909* (HUEFS); Curaçá, Serra Canabrava, 9º56'38"S, 39º 37'30"W, 559 m, 13 Mar 2011, *E. Melo 9364* (HUEFS); inselbergue Morro da Quixaba, 12°44′26″S, 39°47′37″W, 290 m, 12 Feb 2006, *A.O. Moraes et al. 161* (BHCB, HUEFS); Brumado, Fazenda Lagoa Redonda, 14°17'S, 41°33'W, 500 m, 26 Mar 1984, *L.C. de Oliveira Filho & J.C.A. Lima 131* (CEPEC, CORD); São Gabriel, 11°12'4''S, 41°54'16''W, 17 Jan 2007, *J.F. Pastore & R.M. Harley 2603* (CEN, HUEFS, RB); 3 km após Irecê, estrada p/Xique-Xique, 700 m, 18 Feb 1981, *M. do Socorro & G. Ferreira 150* (MO); Urandi, Olhos d'Água, 16 Dec 2017, *F.R.S. Tabosa et al. 55* (BHCB); **Minas Gerais**: Matías Cardoso, 14°53'09''S, 43°45'33.7''W, 485 m, 23 Jan 2017 m, *P.H.A. Melo & T.R. Peixoto 6080* (BHCB);

Pernambuco: Caruaru, E.E. do IPA, 10 Apr 1992, *F. Guedes 98* (IPA); Mirandiba, Serra do Tigre, 08°03′35″S, 38°43′07″W, 495 m, 18 Apr 2007, *M. Pinheiro 253* (CORD, JPB, UFP).

26. Capsicum longifolium Barboza & S.Leiva
PERU. Amazonas: Omia, entre la Cordillera y Quebrada de Agua Sal, 06º26'18"-06º25'4.4"S, 77º10'30.8"-77º10'2.3"W, 2457-2118 m, 22 Sept 2008, *V. Quipuscoa S. et al. 4374* (HUSA, HUT, F, USM); **Cajamarca**: Prov. San Ignacio, Huarango, Quebrada El Mirador, 05°16'12"S, 78°40'01"W, 2200 m, 13 Mar 2000, *J. Campos de la Cruz et al. 6607* (CORD fragment; MO); **Junín:** Chanchamayo, at Puyusacha, a private conservation area, on road starting from the outskirts of San Ramon, 11º05'29''S, 75º26'24''W, 2000 m, 29 May 2013, *T. Särkinen et al. 4805* (BM, E, USM); same locality, 30 May 2013, *T. Särkinen et al. 4813* (E, USM). **Piura**: Huancabamba, Río Samaniego, zona de amortiguamiento del Santuario Nacional Tabaconas-Namballe, 2150-2200 m, 25 Apr 2003, *S.M. Baldeón Malpartida & J. Campos 5316* (USM).

ECUADOR. Zamora-Chinchipe: Estación Biológica San Francisco (EBSF), camino hacia la antena, pasando el Río San Francisco, 3º58’22.5’’S, 79º04’40.9’’W, 1830 m, 2 May 2017, *G.E. Barboza & S. Leiva González 4821* (CORD); EBSF, a unos 300 m después del cruzar el Río San Francisco, por el camino del Atajo, 3°58’21.6’’S, 79°04’41.4’’W, 1839 m, 17 Aug 2017, *G.E. Barboza & S. Leiva González 4846 & 4851* (CORD, HAO, LOJA); EBSF, después de cruzar el Río San Francisco, 3°58’22.5’’S, 79°04’39.3’’W, 1888 m, 17 Aug 2017, *G.E. Barboza & S. Leiva González 4849 & 4850* (CORD, LOJA); same locality, 2 May 2017, *S. Leiva González 6531* (HAO) & 17 Aug 2017, *S. Leiva 6580* & *6581*(CORD, HAO); road Loxa-Zamora, 5 km W of Tambo, 2100 m, 14-19 Jul 1959, *G. Harling 5867* (S); above Valladolid on road to Yanganá, 2700 m, 2 Feb 1985, *G. Harling & L. Andersson 21464* (GB).

27. Capsicum lycianthoides Bitter
COLOMBIA. Nuelle Grenada, 1844, *J. Goudot 55* (P); **Antioquia**: Caldas, Finca La Oculta, 2020 m, 14 Oct 1983, *L.K. Albert de Escobar & A. Uribe 3645* (HUA, MO); Fredonia, Cerro Bravo, Finca de los Betancur, camino hacia la toma de agua, 1770-1900 m, 7 Jun 1992, *R. Fonnegra et al. 4417* & *4442* (COL, HUA, MO, NY); Jardín, road between Jardín and Río Sucio, ca. 9 km from Jardín, 05°33'N, 75°49'W, 2300-2400 m, 29 Jan 1989, *J.M. MacDougal & F.J. Roldán 3547* (MO); **Caldas**: Manizales, vía a Villamaría, 2000 m, 11 Jun 1985, *F. Escobar FM 122* (NY); **Huila**: La Plata, Finca Merenberg, 2300 m, 3 Aug 2010, *A. Orejuela R. 109* (COL); **Nariño**: Ricaurte, trail from La Planada to Pielapi, 01º04'N, 78º02'W, 1600-1800 m, 22 Jul 1988, *A.H. Gentry et al. 63614* (MO, PSO); Reserva Natural La Planada, trocha Centro de Científicos-Mirador, 15 May 1994, *H. Mendoza 661* (FMB) & 15 Mar 1995, *H. Mendoza 814* (FMB); Ricaurte, 1300 m, 12 Apr 1941, *K. von Sneidern s.n.* (S); **Risarlada**: Parque Natural Ucamarí, trail behind the station at La Suiza, 4º42'N, 75º32'W, 2000 m, 20 Feb 1992, *B. Eriksen & B. Ståhl 19* (COL); Pereira, Vereda la Suiza, Santuario de Fauna y Flora Otún-Quimbaya Pereria, 04º45'00"N, 75º46'00"W, 1850 m, *S. Estrada V. 11* (ANDES); Parque Regional Ucumarí, 04°45'N, 75°35'W, 1600 m, 5-7 Aug 1995, *C. Múrcia 371* (MO); **Putumayo**: Mun. Mocoa,

Corregimiento de San Santonio, vereda Alto Campucana, finca La Mariposa, 01°12'N, 76°38'W, 1350-1420 m, 20 Apr 1994, *J.L. Fernández Alonso et al. 10725* (COL, MO); **Quindío**: Calarca, Corregimiento Quebrada Negra, Vda. Vista Hermosa, Finca La Floresta, 4°27'35"N, 75°38'41"W, 1650-2150 m, 2 Mar 1991, *C.A. Agudelo et al. 899* (COL); Circasia, Sector Bremen, 1930, 7 Sep 1985, *G. Arbeláez S. et al. 1136* (COL); Génova, Vereda Varsovia, Hda. Varsovia, 1820 m, 17 Mar 1988, *G. Arbeláez S. et al. 2466* (COL); Vereda Membrillal, desde la Pizarra bordeando el río por la carretera destapada, 1700 m, 13 Feb 2010, *G.D. Beltrán 85* (COL); Circasia, Vereda Barcelona baja, 1830 m, 26 Jun 1979, *F. Maya et al. s.n.* (COL 000032890); Génova, Oct 1996, *W.G. Vargas 3546* (MO); Filandia, carretera el Roble-Circasia, 1780 m, 24 Jan 1990, *M.C. Vélez et al. 1008* (COL); **Valle del Cauca**: Cali, Kilómetro 18, vía Buenaventura por la carretera antigua, 3 km cerca Finca Mi Universo, 1900-2000 m, 14 Feb 2010, *G.D. Beltrán 87* (COL); Tuluá, along road between Tuluá and Santa Lucia (vía La Marina and Venus), 2.1 km SE of Venus, 04°00'N, 76°05'W, 1700 m, 16 Feb 1990, *T.B. Croat 70657* (MO); La Cumbre, along road between main Cali-Loboguerrero road and Bitaco, 03°36'42"N, 76°37'17"W, 1650 m, 24 Jul 1997, *T.B. Croat & J.F. Gaskin 80291* (CUVC, MO); Bugalagrande, Hoya del Río Albán, Quebrada Robada, Alto Bonito, 1800-1900 m, 20-21 Oct 1946, *J. Cuatrecasas 22375* (F); filo de la Cordillera Occidental al sur de Las Brisas, 1850 m, 27 Oct 1946, *J. Cuatrecasas 22665* (CORD, F, US); Dagua, Hoya del Río Dígua, entre Queremal y La Elsa, ca. 1200 m, 27-29 Mar 1947, *J. Cuatrecasas 23996* (CORD, F); Dapa, vía Cali-Dagua, Reserva “El Refugio”, 3º32’09’’/3º32’02’’N, 76º36’56’’/76º36’57’’W, 1913/1975 m, 1 Feb 2016, *R. Deanna 164, 168* & *170* (CORD); Argelia, Vereda Las Brisas, Finca San Jorge, 1850-1960 m, 22 Jan 1983, *S. Díaz P. 3883* & *3900* (COL); Finca Zingara, Km 18 Carretera al Mar, vía a Dapa, 2100 m, 24 Jan 1994, *J.G. Gensini 136* (NY) & 19-27 Feb 1994, *J.G. Gensini 180 & 199* (NY); Corregimiento Bitaco, Hacienda Himalaya, 1800 m, 11 May 1988, *M.D. Heredia et al. 563* (NY); La Cumbre, 1900-2200 m, 21-25 May 1922, *E.P. Killip 5911* (NY); El Silencio, Yanaconas, 1900-2200 m, 28 Feb 1939, *E.P. Killip & H. García 33781* (US); Río Dígua Valley, dense forest along Río Engaño, 675 m, 2-4 Apr 1939, *E.P. Killip 34823* (COL, US); La Elvira, Finca Zingara, ca. 25 km W of Cali, at km 18, 03°28'N, 76°37'W, 1600-1700 m, 20 Apr 1989, *J.L. Luteyn et al. 12547* (CAS, MO, NY); Vereda La Quisquina, Finca Casa Blanca, 03º33'55.9"N, 76º09'51.8"W, 1914 m, 25 Feb 2005, *J.E. Mendoza et al. 4202* (FMB); vía Cali-Dagua, km 23, Reserva Privada “El Refugio”, 4 Nov 2011, *A. Orejuela R. & E. Calderón Sáenz 166* (COL); Corregimiento de Pance, vía Pance-Hato Viejo, PNN Farallones de Cali, ca. 2000 m, 6 Nov 2011, *A. Orejuela R. 174* (COL); hills of Miraflores above Palmira, 1200-1600, Jan 1906, *H. Pittier 872* (NY, US); La Cumbre, Chicoral Alto, detrás de Dapa, en los nacimientos del Río Bitaco, 1970 m, 20 Jan 1989, *J.E. Ramos 1794* (NY); Calima, Vereda Campo Alegre, "Alto Calima" trocha paralela al Río Calima, cerca desembocadura Río Bravo, 900 m, 29 Aug 1981, *P.A. Silverstone-Sopkin 556* (MO, TULV);

ECUADOR. Azuay: Molleturo, Santa Cruz, 1200-1500 m, 14 Jan 1991, *A. Castellanos 212* (QCA); Coop. Luchadores del Litoral (El Sillado), 1250 m, 21 Jan 1991, *A. Castellanos 239* (QCA, QCNE); road from Cuenca to Guayaquil, 02°30'S, 79°18'W, 9200-9600 ft, 17 Jul 1984, *W. D'Arcy 15777* (MO); Azuay, by stream, 03°04'48"S, 79°19'48"W, 7 Jul 1986, *W. D'Arcy 16423* (MO); between Río Gamolotal and Río Huigra on road to Molleturo, 1220-1520 m, 10 Jun 1943, *J.A. Steyermark 52914* (F). **Bolívar**: Limón, estribaciones inferiores de la Cordillera Occidental, 800 m, 19 Oct 1943, *M. Acosta Solís 6435* (F); Atio

de Telimbela, 1500 m, 18 Nov 1943, *M. Acosta Solís 6899* (F); camino de Sarapata, sec. Tres Puentes, 2200-3000 m, 22 Nov 1943, *M. Acosta Solís 6968* (F); Hda. Changuil, Sector la 47, 02°10'W, 79°10'W, 400 m, 10 Aug 1995, *C. Bonifaz & X. Cornejo 3249* (GUAY); Guaranda, recorrido entre el recinto Chazo Juan hasta el km 3 del sendero a la Chorrera de Moras, 01°23'36"S, 79°08'59"W, 1030 m, 18 Feb 2005, *H. Vargas L. 5065* (MO, NY, QCNE); **Cañar**: Gualleturo, La Abundancia junto a la Quebrada San Juan, 2000 m, Oct 1990, *A. Castellanos 54* (QCA, QCNE); along road between Azoques and El Triunfo (road to Guayaquil, Machala, Riobamba), 1 km S of La Delicia, 02°27'S, 79°10'W, 22 Oct 1980, *T.B. Croat 50879* (MO); Km 110 from Durán, 1300 m, 15 Jan 1962, *C.H. Dodson & L.B. Thien 2101* (MO, QCNE) *& 2131* (QCNE, MO, WIS); Manta Real, Río Patul, camino entre Zhucay y Río Patul, 02º33'S, 79º20'W, 1200 m, 11-12 Jul 1991, *A. P. Yáñez & R. Foster 262* (QCA); **Carchi**: Mira, Parroquia Gualchan, along road between El Chical and Gualchan, 14 km S of Río Gualpi Bridge, 00º49'46"N, 78º12'38"W, 1671 m, 16 Oct 2012, *T.B. Croat 104378* (MO, QCNE); trail Maldonado-Tobar Donoso, 3-4 km NW of Maldonado, 1500 m, 27 Feb 1974, *G. Harling & L. Andersson 12250* (MO); trail from Rafael Quindís mountain finca to Río Verde and short distance up Río Verde, 1890 m, 28 Nov 1987, *W.S. Hoover 1896* (MO); further ascent of Río Verde approaching headwaters of river at base of Cerro Golondrinas, 1900 m, 30 Nov 1987, *W.S. Hoover 2093* (MO); Medium Cerro Golondrinas beginning at principal stream division, 2030 m, 2 Dec 1987, *W.S. Hoover 2227* (MO); norte del Carmen, camino a Chical, 2000-2200 m, 10 Feb 1992, *W. Palacios et al. 9746* (MO, QCNE); El Carmen, Cerro Golondrinas, 2000-2400 m, 18-25 Aug 1994, *M. Tirado et al. 1309* (MO, QCNE); Maldonado, 1500 m, 8 Oct 1981, *L. Werling & S. Leth-Nissen 397* (QCA); **Cotopaxi**: along road between Quevedo and Latacunga, 23.5 km E of La Maná, 00°53'S, 79°04'W, ca. 950 m, 10 Oct 1983, *T.B. Croat 57028* (MO); Tenefuerste, Río Pilalo, km 52-53, 750-1300 m, 7 Feb 1982, *C.H. Dodson & A.H. Gentry 12269* (MO); Río Pilaló, km 52-53, Quevedo-Latacunga, 750-1300 m, 21 Feb 1982, *C.H. Dodson & L.B. Thien 12778* (MO, QCNE); San Francisco de la Pampa, desde Naranjito rumbo a la cabaña de la Reserva de Otonga, 00º25'08,8''S, 79º00'12,7''W, 1905 m, 19 Nov 2011, *C.I. Orozco et al. 3960* (COL, CORD) & *3972* (COL, CORD, QCA, QCNE); desde la cabaña de la Reserva de Otonga rumbo a la base del bosque, 20 Nov 2011, *C.I. Orozco et al. 3989* (QCA); La Maná, Reserva Ecológica (RE) Los Ilinizas, sector El Oriente, 1570 m, 12 Jul 2003, *P.A. Silverstone-Sopkin et al. 9069* (NY, QCNE); RE Los Ilinizas, sector Brasil, 00°40'28"S, 79°05'03"W, 1525 m, 17 Jul 2003, *P.A. Silverstone-Sopkin et al. 9249* (NY, QCNE); RE Los Ilinizas, sector Guadual Grande arriba del alto Río Guadal, 00º39'14"S, 79º03'49"W, 1300 m, 20 Jul 2003, *P.A. Silverstone-Sopkin et al. 9377* (QCNE); Pujilí, RE Los Ilinizas, Sector II (Sector Sur), sector Chuspitambo, al occidente de Choasillí, 00°58'42"S, 79°06'22"W, 1900 m, 5 Aug 2003, *P.A. Silverstone-Sopkin et al. 9790* (CHEP, CUVC, MO, NY, QCNE); RE Los Ilinizas, Sector II (Sector Sur), sector Paloseco, 00°58'34"S, 79°06'58"W, 1700 m, 12 Aug 2003, *P.A. Silverstone-Sopkin et al. 10070* (CUVC, NY, QCNE); **El Oro**: camino a Limón-Playa, cerca al Río Dumarí, 03°29'S, 79°45'W, 600 m, 12 Oct 1993, *X. Cornejo 421* (GUAY); Santa Rosa, 11 km west of Pinas on new road to Sta. Rosa, 850 m, 8 Oct 1979, *C.H. Dodson et al. 9149* (MO); 10 km west of Piñas on new road to Sta. Rosa, 950 m, 8 Oct 1979, *C.H. Dodson et al. 9161* (MO); new road Saracay-Balsas-Velacruz, c. 8 km SE of Saracay, 750 m, 30 Apr 1980, *G.W. Harling & L. Andersson 18756* (MO); Bosque Petrificado Puyango, close to the quebrada which crosses the forest, 03º52'30"S, 80º05'01"W, 450 m, 26 Feb 1997, *B.B. Klitgaard et al. 411* (QCNE); along road near Piñas toward Santa Rosa, 3º09'S, 79º08'W,

3000 ft, 8 Dec 1965, *D.H. Knight 641* (MO, WIS); **Guayas**: Naranjal, Reserva Ecológica Manglares-Churute, Cerro Cimalón, 100-670 m, 02°24'S, 79°35'W, 27 Feb 1992, *C.E. Cerón 18324* (MO, NY, QCNE); **Imbabura**: Cotacachi, Parroquia Intag, Comuna Santa Rosa de Pucurá, Reserva Privada Intag, 1850 m, 6 Mar 2002, *J. Caranqui et al. 517* (CHEP, MO, QCNE); Reserva Siempreverde, a lo largo del sendero "La Cascada", 00º22'39'N, 78º25'45''W, 2380-2550 m, 4 Jul 2012, *A.J. Pérez et al. 5386* (QCA); Parroquia García Moreno, Reserva Biológica Los Cedros, sendero El Oso, 00°19'11"N, 78°47'08"W, 1900 m, 27 Oct 2005, *H. Vargas L. 6292* (MO, QCNE); **Loja**: Biological station, El Bosque about 10 miles N.E. of town of Podocarpus, 3500 m, 04°15'S 079°15'W, 26 May 1991, *R. Bensman 480* (MO, NY); along road from Balsas to Piñas, 6.7 km NNW of Balsas, 03°43'46"S, 79°50'19"W, 818 m, 31 Jul 2004, *T.B. Croat & L.P. Hannon 92746* (MO, QCNE); **Los Ríos**: Clementina Farms, Cerro Samama, 5.7 km S and W of main Pueblo-Viejo-Caluma road, 5.2 km W from bridge over Río Pita, 01°38'51"S, 79°19'52"W, 370-600 m, 14 Aug 2004, *T.B. Croat et al. 93341* (BM, MO, NY, QCNE) & *93366* (MO, NY, QCNE); Cerro Samana, SE of Potosí, SW of Caluma, vicinity of village of Pita, between Pita and Escuela 18 de Deciembre, 165-400 m, 18 Mar 2006, *T.B. Croat 96081* (MO); Clementina, 20 Mar 1953, *F. Fagerlind & P-G. Wibom 2632* (S); Hacienda Clementina, Cerro Samama, trail Pita and La Torre, 500-700 m, 17-19 Sep 1999, *C. Gustafsson & C. Bonifaz 412* & *477* (S); Hacienda Clementina, 750 m, 18 Mar 1947, *G. Harling 477* (S), 24 Mar 1947, *G. Harling 519* (S) & 12 Aug 1947, *G. Harling 1680* (S); Hacienda Clementina, Cerro Samama, trail Pita and La Torre, 400-600 m, 4 Jun 1995, *J.T. Knuden & B.Ståhl 381* (S) & 23 Oct 1995, *J.T. Knuden & B.Ståhl 439* (S); Hacienda Clementina, cerro Samama, 01º39'S, 79º19'W, 780 m, 8 Jul 1995, *S. Roponen & Å. Johannessen 85* (S) & 13 Dec 1996, *S. Roponen & Å. Johannessen 229* (S); road Chillogallo-Chiriboga, between the village and Km 48, 1600-2000 m, 12 Jun 1967, *B. Sparre 16984* (S, US); Haciendad Clementina, Cerro Samama, on trail from Casa Pita, ca. 38 km NE of Babahoyo, 01º39'S, 79º22'W, 400-600 m, 19-20 May 1994, *B. Ståhl & J.T. Knudsen 1065* & *1090* (S) & 25 May 1994, *B. Ståhl & J.T. Knudsen 1224* (NY); Hacienda Clementina, Cerro Samama, above Destacamento Pita on trail to La Torre, 01º39'S, 79º20'W, 750 m, 27 Feb 2009, *B. Ståhl & S. Báez 7061* (S); **Napo**: Archidona, Parque Nacional Napo-Galeras, sendero hacia Huamaní, 1500-1650 m, 15 Mar 1997, *A. Álvarez et al. 1584* (MO, QCNE); about 2 km NW of Santa Rosa de Quijos, 1500 m, 12 Feb 1980, *G.W. Harling & L. Andersson 16487* (GB, MO); Cantón Tena, Cordillera de Huacamayos, entre Carachupa-Chacana, 00º48'S, 78º07'W, 1940-2200 m, 7 Aug 1995, *J. Jaramillo & I. Tapia 18452* (QCA); **Pichincha**: Reserva Forestal Endesa, Río Silanche, "Corpación Forestal Juan Manuel Durinini", km 113 de la carretera Quito- Puerto Quito, 650-700 m, 15-16 Dec 1985, *M. Bedoya 5* (MO, QCA); Nono-Nanegal road, 15 km W of Nono, 4 Jul 1978, *J.D. Boeke 2327* (MO, NY); El Pahuma Orchid Reserve, 17 km east of Nanegalito, trail from "La Guarida del Oso" to "Sendero de Los Yumbos", 00º00'S, 78º38'W, 2200-2650 m, 19 Apr 2003, *J.L. Clark et al. 7671* (QCNE, US); along road between Tandayapa and Mindo, 00°01'00"S, 78°46'00"W, 1930 m, 16 Dec 1979, *T.B. Croat 49390* (MO); road from Calacalí to Tandayapa, at Km 22 (west of junction into town of Calacalí), 00°01'03"N, 78°36'25"W, 1000 m, 20 Jul 1998, *T.B. Croat & J. Whitehill 82726* (MO); Maquipucuna Reserve, vicinity of Nanegal, 6.7 km NE of Nanegalito-Nanegal road (turnoff ca. 3 km S of Nanegal), 1330-1430 m, 11 Mar 2006, *T.B. Croat et al. 95685* (BM, G, MO, NY, QCNE, US); along road from Pacto and Cielo Verde on Río Guayabamba (Imbabura Province), 23.8 km to Pacto, 00°11'47"N, 78°52'07"W, 844 m, 25 Mar 2006, *T.B. Croat et*

al. 96413 (MO, QCNE); along road from Mindo to Tandayapa departing main asphalt road from Nanegalito to Dto. Quito just east off to Mindo, 00º01'36"S, 78º44'56"W, 1741 m, 16 Oct 2007, *T.B. Croat et al. 99984* (MO, QCNE); Quito, Reserva Mashpi, along road leading into reserve, 14 km N of plaza in Pacto, then 6.3 km into reserve, 00°09'57"N, 78°52'17"W, 1045 m, 7 Dec 2008, *T.B. Croat et al. 100944* (MO, QCNE); Reserva Mashpi, along road leading into reserve, 13.8 km N of central plaza in Pacto, then 7-8 km into reserve, 00º09'53"N, 78º52'46"W , 910-1000 m, 8 Dec 2008, *T.B. Croat 101027* (MO, QCNE); Bosque Protector "Río Guajalito", km 59 de la carretera antigua Quito-Santo Domingo de los Colorados, a 3 1/2 km NE de la carretera, 00º14'19"S, 78º48'15"W, 2095 m, 27 Jan 2013, *R. Deanna et al. 133* (CORD, QCNE); área Maquipucuna, 6 km al este de Nanegal, 00º07'29"S, 78º38'55"W, 1219 m, 29 Jan 2013, *R. Deanna et al. 144* (CORD); Dos Ríos, Km 90, old road via Chiriboga, Quito-Santo Domingo, 1100 m, 4 Apr 1984, *C.H. Dodson et al. 14163* (MO, QCNE); Cerro Guarumos-La Y del sendero al Cerro El Castillo, derecho de vía del Oleoducto de Crudos Pesados, 00º03'S, 78º38'W, 2600-2700 m, 7 Sep 2001, *A. Freire Fierro et al. 3181* (MO, QCNE); 11 km W of Tandapi, trail along Chictoa River, tributary of Río Pilatón, 00°20'38"S, 78°51'36"W, 1350-1550 m, 26 Oct 1974, *A.H. Gentry et al. 12095* (MO); a 3,5 km al NE de la carretera antigua Quito-Santo Domingo de los Colorados, estribaciones occidentales del volcán Pichincha, 2200 m, 13 Feb 1987, *R. Grijalva 259* (QCA); Estación Los Faisanes, c. 12 km from Río Pilatón, 1400 m, 18 Mar 1985, *G. Harling & L. Andersson 23121* (NY, QCA); Reserva Endesa, ca. 6 km WNW of P. Vicente Maldonado, 800 m, 23 Mar 1985, *G. Harling & L. Andersson 23265* (QCA); Reserva Forestal Endesa, Río Silanche, "Corporación Forestal Juan Manuel Durini", Km 113 de la carretera Quito- Puerto Quito, 0º05'N, 79º02'W, 650-700 m, 23 Feb 1984, *J. Jaramillo 6425 (6423)* & *6426* (QCA), 16 Aug 1984, *J.L. Jaramillo 6774* (MO, QCA, QCNE) & *6965-B* (MO, QCA), 21 Oct 1985, *J.L. Jaramillo* 8209 (QCA); Reserva Florística-Ecológica "Río Guajalito, Km 59 de la carr. Antigua Quito-Santo Domingo de los Colorados, 0º13'53''S, 78º48'10''W, 1800-2200 m, 29 Jun 1985, *J.L. Jaramillo & V. Zak 7856* (CHEP, MO, NY, QCA); Parroquia San José de Minas, sector Las Palmas, margen derecha del Río Cambugán, 00º11'N, 78º31'W, 1800-2200 m, 1 Apr 1999, *E. Jiménez et al. 1126* (Q); Hacienda San Luis, Km 96, carretera vieja Quito-Santo Domingo, Feb 1975, *F.J. Ortiz 11* (MO, QCA); vía Calacalí-Nanegalito, 0º03'N, 78º35'W, 2100 m, 24 Jan 1989, *W. Palacios & H. van der Werff 3591* (MO, QCNE); Bellavista Cloud Forest Reserve near Nanegalito, trail H, 00°00'57"S, 78°40'48"W, 2250 m, 23 Dec 2002, *S.D. Smith 203* (MO, QCNE); road Alóag-Santo Domingo, Tandápi (M. Cornejo Astorga), 1500 m, 19 Jan 1967, *B. Sparre 14042 & 14365* (S); road Alóag-Santo Domingo, San Ignacio, km 23, 2000 m, 4 Mar 1967, *B. Sparre 14591* (RB, S); Tandápi (M. Cornejo Astorga), 27 May 1967, *B. Sparre 16714* (S); Cerro de Sosa, c. 7 km airline SE of Nanegal, 0º07'N, 78º38'W, 1700-1750 m, 29 Aug 1989, *L. Webster 27143* (QCA); Reserva Maquipucuna, trail from Hacienda Espárragos to Cerro de Sosa, 0°07'N, 78°38'W, 1500-1600 m, 12 Sep 1989, *L. Webster & L. Hebert 27493* (QCA); Reserva Maquipucuna, el Pacchal, 0º08'N, 78º38'W, 1300-1500 m, 9 Jul 1990, *G.L. Webster & UREP participants 27868* (MO, NY, QCA, QCNE); same locality, 10 Jul 1990, *G.L. Webster & T. Thompson 27964* (CORD, DAV); slopes of Gregoire´s Hill (W ridge of Cerro Campana), 1300-1500 m, 3 Jul 1991, *G.L. Webster et al. 28749* (MO, QCNE); Bosque Protector Maquipucuna, along trail from Guava plantation to Rio Tulambi, 00º07.3' N, 78º38'W, 1300-1400 m, 10 Jul 1992, *G.L. Webster & R. Rhode 29283* (QCNE); Cerro Campana, 5-6 km E of Nanegal, ridge between Quebrada Cariyacu & Q. Loreto, 0º09'N, 78º37'W, 1750 m, 1 Sep 1993, *G.L. Webster et*

al. 30044 (CORD, DAV); Parroquia Nanegalito, western slopes of cerro Negro, 00º04'N, 78º39'W, 2075-2100 m, 8 Sep 1993, *G.L. Webster et al. 30501* (QCNE); Bosque Protector Maquipucuna, sendero de las Palmitas, 00º07,5'N, 78º38'W, 1300-1350 m, 18 Nov 1998, *G.L. Webster et al. 32907* (QCNE); Reserva Florística-Ecológica (RFE) "Río Guajalito, Km 59 de la carr. Antigua Quito-Santo Domingo de los Colorados, 0º13'53''S, 78º48'10''W, 1800-2200 m, 16 Feb 1986, *V. Zak 836* (MO, NY, QCA) & 9 Nov 1986, *V. Zak 1433* (MO, QCA); carretera Quito-Lloa- Mindo, Hacienda "El Pedregal", 1650-1850 m, 10 Jul 1987, *V. Zak & J.L. Jaramillo 2147* (MO); RFE "Río Guajalito, estribaciones occidentales del Volcán Pichincha, 00°13'S, 78°48'W, 1800-2200 m, 22 Jul 1987, *V. Zak & J.L. Jaramillo 2220* (BM, MO, MEXU); carretera Quito-Alóag- Sto. Domingo de los Colorados, Km 94, sector "La Esperie", 00°20'S, 78°50'W, 1500-1800 m, 2 Dec 1987, *V. Zak & J.L. Jaramillo 3099* (MO); carretera antigua Quito-Santo domingo de los Colorados, km 59, "Bosque Protector Río Guajalito", 00°12'S, 78°48'W, 1900 m, 13 Jun 2003, *V. Zak 9804* (QCA); **Santa Elena**: Cordillera Chongón-Colonche, Bosque Protector Loma Alta, 600 m, Jul 1995, *C. Bonifaz & X. Cornejo 3126* (GUAY).

28. Capsicum minutiflorum (Rusby) Hunz.
Bolivia. Cochabamba: Chapare, Espíritu Santo, Antahuacana, 160 km al noroeste de Cochabamba, 750 m, Jun 1909, *O. Buchtien 2231* (GH, US, Z); Ayopaya, San Cristóbal, 16°41'45"S, 66°42'41"W, 2800 m, 27 Oct 2005, *E. Fernández 3998* (BOLV, MO); **Santa Cruz**: Buena Vista, Hotel Flora y Fauna, 17º30'57.4"S, 63º38'07.1"W, 422 m, 9 Dec 2007, *S. Abrahamczyk s.n.* (LPB); Florida, Comunidad de Bella Vista, sendero Chorro del Fraile, 18°18'76"S, 63°40'38"W, 1240 m, 18 May 2005, *L. Arroyo et al. 2880* (LPB, MO, USZ); cerca del Chorro el Fraile, 18°18'43"S, 63°40'23"W, 1200 m, 5 Nov 2005, *L. Arroyo et al. 2930* (MO, USZ); sendero Chorro del Fraile, 18°17'38.50"S, 63°40'24, 29"W, 1250 m, 14 Jun 2006, *L. Arroyo et al. 3317* (BOLV, MO, USZ) & *3333* (MO, USZ); Vallegrande, Alto Seco, alrededor de la propiedad el Sausal, en la localidad de Peñones (propiedad de la familia Farel), 18°59'23''S, 64°01'52''W, 1726 m, 29 Sep 2011, *L. Arroyo et al. 6053* (MO, USZ); unos 5 km antes de Bermejo, viniendo desde Tarumá, apenas pasando el km 428 (a unos 80 m de la ruta, entrando en una quebrada hacia la derecha), 18º09'36"S, 63º36'14"W, 28 Nov 2012, *G.E. Barboza & C. Carrizo García 3644* (CORD); Las Negras, entre Las Cuevas y Bermejo, por el camino viejo Cochabamba-Santa Cruz, alrededor de un puente, 18º08'07''S, 63º41'14''W, 995 m, 13 Dec 2017, *G.E. Barboza et al. 4918* (CORD); La Guardia, 5 km hacia el sur, 630 m, 9 Jan 1998, *S. Beck 23451* (LPB); Jardín Botánico de Santa Cruz, 12 km E of center of Santa Cruz on road to Cotoca, 17°46'S, 63°04'W, 375 m, 23 Jun 1998, *L. Bohs et al. 2823* (USZ); RN9, de Camiri hacia Santa Cruz, ca. Km 166, pasando 7 km la entrada a Tatarenda Nuevo, 19º03'30''S, 63º23'22''W, 799 m, 8 Mar 2018, *C. Carrizo García 61 & 62* (CORD); RN7, desde La Guardia hacia Samaipata, pasando Bermejo, después del puente La Negra I, 18º08'07''S, 63º41'14''W, 1035 m, 8 Mar 2018, *C. Carrizo García 64* (CORD, LPB); El Torno, Reserva El Jardín de las Delicias, próximo al Río Blanco, 17°51'58"S, 63°30'49"W, 1500 m, 2 Nov 2008, *I. Linneo F. et al. 1558* (MO, USZ); San José, 4-5 km al oeste de San José, entrando por camino a Espejillos, 17°55'45.49"S, 63°24'21.35"W, 17 Apr 1993, *B. Mostacedo 495* (NY, USZ); along road from Santa Cruz to Samaipata, 1 km SW of Angostura, in gorge of Rio Pirai, 650 m, 18°09'S, 63°31'W, 13 Jan 1987, *M. Nee 33493* (LPB, MO, NY); 12 km E of center of Santa Cruz, on road to Cotoca, 17°46'S, 63°04'W, 375 m, 21 Jan 1987, *M. Nee*

33715 (LPB, NY); 3 km SW of Estancia San Rafael de Amboro, 17 km. (by air) SSE of Buena Vista, 17°36'S, 63°37'W, 350 m, 29 Jul 1987, *M. Nee 35427* (LPB, MO, NY); E side of Rio Surutú at crossing on road to El Carmen, 17º31'S, 63º40'30''W, 320 m, 29 Oct 1990, *M. Nee 39545* (CORD, LPB, MO, NY, USZ); Ñuflo de Chávez, 14 km (by air) NW of San Javier, Las Lajas, "El Chupadero", 16º10'S, 62º36'W, 550 m, 30 Nov 1990, *M. Nee 40110* & *40103* (CORD, NY, USZ); 4 km W of highway bridge over Río Piraí, 18º06'S, 63º30'W, 750 m, 11 Dec 1991, *M. Nee 42027* (CORD, MO, NY, U, USZ); "Puente Las Cruces" along highway from Santa Cruz to Samaipata, 6 km W (by air) of Bermejo, along Río Vicoquín, 18º08'S, 63º41'W, 1000 m, 21 Dec 1991, *M. Nee 42218* (LPB, MO, NY); 6 km NW of Terebinto on road to El Hondo, 17º41'S, 63º25'15''W, 450 m, 16 Jan 1994, *M. Nee 44503* (CORD, LPB, NY, USZ); along new highway from Santa Cruz to Abapó, 9 km S of Río Peji bridge, 500 m, 13 Jan 1987, 18°03"S, 63°12'00"W, 2 May 1998, *M. Nee 49201* (MO, NY); 6 km NW of highway at Tarumá and bridge over Río Piraí, along Quebrada Salada and old mule trail and current pipeline from Santa Cruz to Bermejo and Samaipata, 18º06'S, 63º30.6'W, 700 m, 27 Mar 2002, *M. Nee & M. Sundue 51994* (LPB, MO, NY, USZ); vic. Hotel Flora y Fauna, 5 km SE of Buena Vista, 17°31'S, 63°38'W, 375 m, 24 Apr 2005, *M. Nee 52917* (MO); camino entre Mosquera-Petacas, 18°42'10''S, 63°39'29''W, 720 m, 10 May 2012, *G.A. Parada et al. 4456* (MO, USZ); Bermejo, Centro de Investigación de Ecología y Conservación Los Volcanes, 18º06'S, 63º36'W, 1100 m, 8 Jul 2003, *D. Soto et al. 95 A* (USZ); Parque Nacional Amboró (PNA), Río San Rafael, 10 Km al NE de Mairana, pasando La Yunga, 18°02'03"S, 63°52'03"W, 1500 m, *I. Vargas C. 2131* (BOLV, MO, NY, USZ); PNA, La Playa, 15-20 km al N de Santa Rosa de Lima, Riveras del Agua Dulce y juntas con el Río Moija, 17º46.5'S, 64º13.5'W, 1300-1400 m, 3-7 Apr 1993, *I. Vargas C. et al. 2173* (BOLV, CORD, NY, USZ); Bella Vista, el "Novillo", 18°16'17.2"S, 63°39'22.3"W, 1558 m, 25 Jan 2007, *M. Vargas Contreras 254* (MO, USZ); Bella Vista, Sendero ecológico el Chorro del Fraile, 18º18'72.7"S, 63º40'38.9" W, 1210 m, 12-13 Dec 2007, *D. Villarroel 1677* (USZ) & *1712* (MO, USZ).

29. Capsicum mirabile Mart.

BRAZIL. Bahia: Fazenda Serra Bonita, 9.7 km W de Camacã na estrada para Jacarecí, daí 6 km SW na estrada para a RPPN e Torre da Embratel, 15°23'30"S, 39°33'55"W, 835 m, 8 Dec 2004, *A.M. Amorim et al. 4429* (BHCB, CEPEC, NY, MO) & 28 Oct 2005, *A.M. Amorim et al. 5371* (BHCB, CEPEC); Arataca, Serra das Lontras, 900 m, 30 Mar 2008, *A.M. Amorim et al. 7235* (BHCB, CEPEC, RB); Fazenda Serra Bonita, 900-1000 m, 9 Dec 2006, *R.A.X. Borges et al. 298* (HUEFS, NY, RB, SP) & 23 Jan 2007, *R.A.X. Borges et al. 707* (BHCB, CEPEC, NY, RB); PARNA Serra das Lontras, acesso (BR 101), entrada no Distrito de Pratas, Faz. Da Dra. K. Serra do Mangue, 15º11'22''S, 39º23'7''W, 700-900 m, 24 Nov 2011, *L. Daneu et al. 632* (CEPEC, MBML); Fazenda Serra Bonita, 15º23'30''S, 39º33'55''W, 850 m, 8 Jan 2006, *M. Lópes et al. 416* (BHCB, CEPEC, HUEFS, SPF); Serra das Lontras, ca. de 7 km no ramal que liga o distrito de Itatingui a Serra, 31 Mar 2006, *J.L. Paixão 951* (NY); **Espírito Santo**: Domingo Martins, BR 262, Km 98, 100 m da entrada para Granja Etina, 20º20'S, 41º02'W, 800 m, 30 Mar 1986, *L. Bianchetti et al. 330* (CEN, CORD), *331, 332 & 335* (CEN); Ibitirama, Santa Marta, -20.4911, -41.7544, 12 Jun 2012, *H.M. Dias et al. 783* (BHCB, VIES); Divino de São Lourenço, Parque Nacional do Caparaó, Córrego do Limo Verde, 20º33'01''S, 41º45'45''W, 1179 m, 10 Feb 2011, *L.L. Giacomin et al. 1248* (BHCB, CORD, ICN, RB); en camino lateral hacia granja Etina, al

norte de la ruta BR 262 (km 98) a Vitoria, a unos 100 m del límite con el municipio Conceição do Castelo, ca. 800 m, 30 Mar 1986, *A.T. Hunziker 25140* (CORD); **Minas Gerais**: Araponga, Pension Serra d'Agua at foot of Parque Estadual Serra do Brigadeiro, near town of Araponga, 20º41'34''S, 42º29'54''W, 1020 m, 20 Apr 2010, *M.F. Agra et al. 7244* (BHCB, CORD); Ouro Preto, Parque Estadual Pico do Itacolomi, mata do Morro do Cachorro, 20º25'20''S, 43º30'25''W, 1410 m, 26 Apr 2011, *M.F. Agra et al. 7323 & 7331* (BHCB); Parque Nacional do Caparaó (PNC), 4 km do posto do IBDF caminho Cachoeira Bonita, 20º28'S, 41º49'W, 1700 m, 1 Apr 1986, *L. Bianchetti et al. 346, 347* & *350* (CEN, CORD); PNC, 3.5 km do posto do IBDF caminho Cachoeira Bonita, 20º28'S, 41º49'W, 1610 m, 1 Apr 1986, *L. Bianchetti et al. 348, 349, 351* & *352* (CEN, CORD); caminho Lima Duarte-Reserva Florestal Estadual da Serra de Ibitipoca, a 8 km da Praca Central de Lima Duarte, 21º47'S, 43º50'W, 700 m, 19 Apr 1986, *L. Bianchetti et al. 496* (CEN, CORD), *499*, *500* & *501* (CEN); Barbacena, BR 265, a 1 km do entrocamento com a BR 040, estrada Barbacena-Río Pomba, 21º18'S, 43º37'W, 1000 m, 22 Apr 1986, *L. Bianchetti et al. 510* (CEN, CORD); María da Fé, Mata da EPAMIG, próximo do antigo poço, 1290 m, 25 Jul 1998, *L. Bianchetti & P.G. Bustamante 1518* (CEN); same locality, 11 May 1999, *L. Bianchetti et al. 1554* (CEN); PNC que leva até o Pico da Bandeira, 1710 m, 23 May 1999, *L. Bianchetti et al.* 1568 (CEN); São Francisco do Prata, Serra das Flores, 23 Mar 1991, *M.C. Brugger et al. s.n.* (BHCB 015236, CEN 31556, CESJ 24628); Parque Estadual Pico do Itacolomi, mata do Morro do Cachorro, 1504 m, 11 Feb 2010, *E.S. Cândido et al. 317, 320 & 333* (OUPR); same locality, 1352 m, 14 Jul 2012, *E.S. Cândido et al. 750* (OUPR); Camanducaia, Mata do Altair, 22º42'39''S, 45º55'54''W, 1900 m, 21 Apr 2001, *G.S. França & R. Stehmann 289* (BHCB); Delfim Moreira, Fazenda Boa Esperança, trilha do Marlon, 22º34'31"S, 45º19'18"W, 1400 m, 16 Mar 2011, *A.L. de Gasper et al. 2610* (BHCB, FURB); Fazenda Boa Esperança, trilha Dos Romeiros, 22º35'02''S, 45º19'02''W, 1626 m, 19 Mar 2011, *L.L. Giacomin et al. 1481* (BHCB, CORD); PNC, a unos 3.5-4 km de la entrada al parque, un poco más debajo de Cachoeira Bonita, 1600-1700 m, 1 Apr 1986, *A.T. Hunziker 25142* & *25143* (CORD); camino hacia Ibertioga y a unos 8 km de la Plaza Central de Lima Duarte, ca. 700 m, 19 Apr 1986, *A.T. Hunziker 25200* & *25201* (CORD, NY; = *L. Bianchetti et al. 495* & *494*, CEN, CORD); Espera Feliz, en el camino de Paraíso a Forquilha, ca. 8 km de la plaça de Paraíso, 20º31'S, 41º48'W, 1300 m, 1 Apr 1986, *A.T. Hunziker et al. 25239* (CORD; = *L. Bianchetti et al. 354*, CEN, CORD); Santa Rita de Jacutinga, 3 Mar 1987, *L. Krieger 21364* (BHCB, CEN); Serra da Araponga, Fazenda Neblina, 1300 m, 10 Mar 1997, *L.S. Leoni 3625* (RB); Dist. Estouro, Morro do Brigadeiro, Faz. Brigadeiro, ca. 700 m da casa da fazenda, 20º41'S, 42º28'W, 1200 m, 25 Mar 1986, *E. Lleras Pérez et al. 2140* (CEN, CORD); Faria Lemos, Fazenda Santa Rita, 550 m, 23 Nov 2006, *E.J. Lucas et al. 667* (BHCB, ESA, RB); PNC, estrada para Macieira, 20º29'S, 41º49'W, 1642 m, 1 Nov 2010, *T.M. Machado et al. 297* (BHCB, RB); Mariana, Estrada para o distrito de Camargos, 9 Feb 2009, *V.R. Scalon et al. 628* (OUPR); Serra da Grama, estrada Araponga-Fervedouro, km 2, 20°39'S, 42°25'W, 960 m, 8 Feb 1989, *A.O. Scariot et al. 381* (IAC, CEN, SPF); divisa entre Camanducaia e Gonçalves, 22º42'39''S, 45º55'54''W, 1900 m, 3 Feb 2001, *J.R. Stehmann et al. 2710* (BHCB); Reserva Florestal Uaimi, trihla para a Serra do Batatal, 20º15'48.5''S, 43º32'0.6''W, 1385 m, 12 Apr 2008, *J.R. Stehmann et al. 5037* (BHCB, HSTM); Alto Jequitibá, Parque Nacional do Caparaó, Estrada para Cachoeira Bonita, 1388 m, 3 Dec 2010, *A.K.L. Venda et al. 54* (BHCB, CEPEC, CESJ, FLOR, MBML, RB); **Rio de Janeiro**: Nova Friburgo, Reserva Macaé de Cima, estrada de terra do Hotel São João para o Sítio dos Miller, 19 Jan 1999, *L.O.*

Anderson et al. 99/36 (UEC); Petrópolis, Reserva Biológica de Tinguá, km 3/4, a 9.6 km de la Estrada Roscio/Pati do Alferes, 1350 m, 19 Apr 2006, *G.E. Barboza et al. 2058* (CORD); subindo o morro da torre de TV a 600 m da entrada do Panorama Park, 22º17'S, 42º32'W, 1230 m, 6 Apr 1986, *L. Bianchetti et al. 359* (CEN, CORD); subindo o morro da TV a 750 m da entrada do Panorama Park, 1250 m, 6 Apr 1986, *L. Bianchetti et al. 363* (CEN, CORD); subindo o morro da TV a 2-2.2 km da entrada do Panorama Park, 22º17'S, 42º32'W, 1500 m, 6 Apr 1986, *L. Bianchetti et al. 366, 379* & *385* (CEN, CORD); subindo o morro da TV, a 2.1 km da entrada do Panorama Park, 1450 m, 6 Apr 1986, *L. Bianchetti et al. 380, 381, 382, 383* & *384* (CEN); subindo o morro da Caledonia, a 5 km do Camping Club do Brasil(RJ. 2), 22º17'S, 42º32'W, 1760 m, 6 Apr 1986, *L. Bianchetti et al. 394* (CEN); Teresópolis, Parque Nacional da Serra dos Órgãos, trilha do Pico do Sino, entre a cachoeira Véu da Noiva e o antigo abrigo 2, 1210 m, 20 May 1999, *L. Bianchetti et al. 1564* (CEN); PN da Serra dos Órgãos, trilha para a Pedra do Sino, 1200 m, 27 Sep 2006, *M.G. Bovini et al. 2605* (CORD, RB); PN da Serra dos Órgãos, entre Barragen e Toca dos Caçadores,18 Dec 1980, *J. Cardoso 150* (CORD, GUA, R); PN da Serra dos Órgãos, trilha para a Pedra do Sino, 22º27'06''S, 43º00'04''W, ca. 1600 m, 12 Mar 2001, *C.B. Costa et al. 494* (CORD, MBML, SP); Jacarepaguá, Parque Estadual Maciço da Pedra Branca, 13 Feb 2016, *C.D. Ferreira & P. Feliz 241* (RB); PN da Serra dos Órgãos, acima do Abrigo 1, 10 Jan 1960, *B. Flaster 49* (R); PN da Serra dos Órgãos, acima do Abrigo 2, 28 Nov 1964, *B. Flaster 1165* (R: 7 sheets) & *1166* (R: 6 sheets); Macaé de Cima, RPPN Bacchus, 22º22'28''S, 42º30'0.8''W, 1501 m, 19 Apr 2009, *L.L. Giacomin et al. 899* (BHCB); Retiro, Nov 1943, *C. Goes & D. Constantino s.n.* (CORD 00087954 fragment, RB 51789); Cerro Caledonia, subiendo la montaña hacia la torre de TV, 1200-1250 m, 6 Apr 1986, *A.T. Hunziker 25155* & *25156* (BM, CORD); subiendo la montaña hacia la torre de TV, 1450 m, 6 Apr 1986, *A.T. Hunziker 25157* (BM, CORD, CTES, MO, NY, P, US, SI); subiendo al cerro de la torre de televisión, 14 Apr 1988, *A.T. Hunziker 25260* (BM, CORD, MO, NY); camino de Nova Friburgo al Pico Altissimo de Caledonia, hacia la torre de TV, ca. 1800 m, 15 Apr 1988, *A.T. Hunziker 25264* (BM, CORD); subiendo por el hermoso camino hacia la torre de TV, en el cerro Caledonia, muy cerca de la torre, 1600/1800 m, 16 Apr 1988, *A.T. Hunziker 25267* (BM, CORD, NY); PN da Serra dos Órgãos, hygrophiler Wald an der Estrada impedida unterhalb. des obersten Parkplatzes geg. Pedra do Sino, ca. 1080 m, 1 May 1960, *H. Hürlimann 6032* (Z); prope Rio de Janeiro, 1823, *L.B. de Karwinski s.n.* (BR 8254160); PN da Serra dos Órgãos, trilha para alojamento 04 e a Pedra do Sino, ca. 22º25-32'S, 42º59'-43º07'W, ca. 1190 m, 12-13 Apr 2011, *J.A. Lombardi et al. 8333* (BHCB, UNOP); Nova Friburgo, 1 Jan 1936, *B. Lutz 1007* (R); Morro do Caledonia, 1400-1600 m, 8 Jun 1977, *G. Martinelli et al. 2461* & *2472* (RB) & *G. Martinelli et al. 2492* (F); the Organ Mountains [Serra dos Órgãos], *J. Miers 4541* pp (left branch, K); PN Serra dos Orgaos, trihla para a travessia, 14 Mar 2014, *C.M. Mynssen et al. 1404* (RB); PN Serra dos Orgaos, trilha Cartão Postal, 16 Nov 2011, *I.M.C. Rodrígues et al. 502* (BHCB); Serra dos Orgaos, 15 Dec 1952, *J. Vidal II-308* & *II-5672* (R: 3 sheets); Miguel Pereira, Retiro das Palmeiras, 22°28'35''S, 43°28'27''W, *M.S. Wängler & V.S. Ferreira 1027* (BHCB, RB); P.E. Três Picos, Trilha Cabeça do Dragão, 1797 m, 28 Jan 2015, *M.S. Wängler 1569* (RB);

São Paulo: PN Serra do Bocaina, from park administrative headquarters to alojamento at entrance to park, 22º41’14’’S, 44º37’55’’W, 1366 m, 2 May 2011, *M.F. Agra et al. 7360* (BHCB); Guarulhos, Sítio Costa, 716 m, 11 Dec 2012, *R.J. Almeida Scabbia et al. 910* (SP); Santo Antonio do Pinhal, Eugenio Lefévre, rumbo a Campos do Jordão, a 200 m de la estación de tren, 22º49’40’’S, 45º37’45’’W, 1202 m, 26 Sep 2010, *G.E. Barboza & M.T. Cosa 2533* (CORD); de Campos do Jordão hacia la estación Lefévre, sobre la izquierda del camino, 22º49’14’’S, 45º37’30’’W, 1150 m, 6 May 2012, *G.E. Barboza et al. 3632* (CORD); Est. Eugênio Lefevre, apenas a 100 m del cruce de la vía de ferrocarril, 22°49’40,2’’S, 45°37’45,1’’W, 1167 m, 4 Apr 2018, *G.E. Barboza & R. Deanna 5024* (BM, CORD); Salesópolis, Estação Biológica de Boracéia (EBB), estrada interna a 6.9 km do alojamento da USP, no sentido USP-guarita, 23º38’49’’S, 45º56’24’’W, 5 Jun 1999, *L. Bianchetti et al. 1538* (CEN); na 35 km da Estrada Taubate-Campos do Jordao, 11 May 1999, *L. Bianchetti et al. 1550 & 1551* (CEN); Piquete, Estrada Piquete-Itajuba, Km 49, 1350 m, 14 May 1999, *L. Bianchetti et al. 1556* (CEN); Estação Biológica de Boracéia, 28 Dec 1983, *A. Custódio Filho 2099* (SP); Casa Grande, EBB, 890-950 m, 17 Dec 1986, *A. Custódio Filho 2807* (UEC); Eugenio Lefévre, rodovia para Campos do Jordão, 14 Jan 1965, *O. Handro 1106* (CORD, SP, US); Campos do Jordão, Dec 1945, *J.E. Leite 3792* (A, GH); Rodovia SP-50, 5 km ao sul de Campos do Jordão, 22º46’S, 45º41’W, 1450 m, 30 Apr 1985, *E. Lleras Pérez et al. 2050* (CEN, CORD); Santa Izabel, perto do km 71 da rodovia Santa Izabel-Igaratá, 13 Dec 1964, *J. Mattos 12165* (CORD, SP); Nazaré Paulista, Estrada em torno da Represa Atibainha, 23º08’33.4’’S, 46º21’28.4’’W, 800 m, *V.C. Souza 11168 (*ESA).

Cultivated
BRAZIL. Mina Gerais: Mun. Viçosa, cultivated in Viçosa by Prof. V. Casali, 1-14 Dec 1986, *A.T. Hunziker 25235* (seeds from L. Bianchetti et al. 335), *25236* (seeds from L. Bianchetti et al. 332), *25237* (seeds from L. Bianchetti et al. 348) & *25238* (seeds from L. Bianchetti et al. 349), *25239* (seeds from *L. Bianchetti et al. 354*), *25243* (seeds from L. Bianchetti et al. 331), *25250* (seeds from L. Bianchetti et al. 380), *25251* (seeds from L. Bianchetti et al. 381), 25252 (seeds from L. Bianchetti et al. 382), *25253* (seeds from L. Bianchetti et al. 383), *25254* (seeds from L. Bianchetti et al. 384) & *25255* (seeds from L. Bianchetti et al. 394) (CORD).

30. Capsicum mirum Barboza
BRAZIL. São Paulo: Mun. Bananal, a unos 17 km al sur de Bananal, por ruta SP 247, rumbo a Sertão do Bocaina, 22º45’50’’S, 44º23’35’’W, 1150 m, 26 Feb 2006, *G. E. Barboza et al. 1649* (fl, fr) (CORD); do cruzamento de Bananal rumo à Estacão Ecológica de Bananal, 22°46’55.8’’S, 44°22’41.1’’W, 1333 m, 5 Apr 2018 (fl, fr), *J.R. Stehmann et al. 6474* (= *Barboza & R. Deanna 5028*) (BHCB).

31. Capsicum muticum (Sendtn.) Barboza
BRAZIL. Brésil, *Anonymous s.n.* (CORD 00087944, P); Brazil, *F. Sellow 79* (photo F 2874 ex B; CORD 00006954, fragment ex B); Brasilien, *J. Widgreen 429* (S16-29236) & 597 [527?] (S16-29232); **Rio de Janeiro**: Petrópolis, Jan 1939, *Atamp 7659* (R 65590); Alto da Serra, subiendo el cerro de la torre de TV, ca. 2,5 km del desvío hacia las antenas, 1240 m,

1 May 2013, *G.E. Barboza & C. Carrizo García 3946* (CORD, photo); Alto da Serra, ingresando por la Estrada Torres do Morim, a 1.8 km del desvío hacia la antena de TV, 22°32'08.7''S, 43°09'32.8''W, 1064 m, 6 Apr 2018, *G.E. Barboza et al. 5032* (CORD); subiendo o morro da TV a 6.5 km da fabrica de tecidos Santa Helena, 22º32'S, 43º11'W, 1300 m, 8 Apr 1986, *L. Bianchetti et al. 406* (CEN); subiendo o morro da TV a 6 km da fabrica de tecidos Santa Helena, 22º32'S, 43º11'W, 1260 m, 8 Apr 1986, *L. Bianchetti et al. 407* & *408* (CEN, CORD); Petrópolis, 10 Feb 1915, *J. Diogo 420* (CORD, R, US); Alto da Serra, subiendo al cerro de la torre de TV, ca. 1260 m, 8 Apr 1986, *A T. Hunziker et al. 25161* (CORD); Serra d'Estrella, Mar 1823, *L. Riedel s.n.* (NY 001184742), same locality, Apr 1823, *L. Riedel s.n.* (MO, NY 00656032); caminho da Mosela, 1877, *J. de Saldanha da Gama 5163* (CORD, R); Serra dos Orgãos, *A-C. Vauthier 526* (CORD, fragment; G); Petrópolis, 1859-1860, *H. Wawra & F. Maly 413* (CORD, W); Rio de Janeiro, 1844, *J. Widgreen s.n.* (S16-29225).

Cultivated
BRAZIL. Minas Gerais: Mun. Viçosa, cult. en Viçosa por Prof. V. Casali (semillas de *L. Bianchetti et al. 406*), 7-14 Dec 1986, *A.T. Hunziker 25246* (CORD).

32. *Capsicum neei* Barboza & X.Reyes
BOLIVIA. Chuquisaca: Hernando Siles, Km 631 + 800 m, yendo desde Monteagudo rumbo a Sucre, sobre mano derecha, 19°48'11.0"S, 64°01'08.2"W, 24 Jan 2019, *G.E. Barboza & X. Reyes 5040* (CORD); Luis Calvo, Ticucha, serranía del Iñao, 12 km al NO de la comunidad de Ticucha, 19°35'0.4''S, 63°53'12.7''W, 1431 m, 11 Apr 2003, *A. Carretero et al. 824* (HSB, MO, NY); Entierrillos, aprox. a 5 km de la escuela de Entierrillos, serranía del Iñao, 19°31'S, 63°52'W, 1700 m, 18 Dec 2003, *A. Carretero et al. 939* (HSB, MO); Serranía del Iñao, pasando la Laguna, 19°31'S, 63°52'W, 18 Dec 2003, *A. Carretero et al. 998* (HSB, MO, NY); Las Frías, ca. a la cima de la serranía de Ñahuañanca, 19°09'30.6"S, 63°50'40.6"W, 1930 m, 22 Dec 2003, *A. Carretero et al. 1067* (HSB, MO, NY); Las Frías, ca. 1/2 km de la vivienda de Sr. Severino Daza, hacia la cima de la serranía de Yahuañanca, 19°09'31"S, 63°50'23"W, 1600 m, 23 Dec 2004, *A. Carretero et al. 1085* (HSB, MO, NY); Tomina, aprox. 800 m. antes de llegar a Llantoj, de La Florida subiendo hacia el E de la Serranía de Kaska Orcko, 19°09'46"S, 64°03'42"W, 1750 m, 11 Oct 2004, *J. Gutiérrez R. 1004* (HSB, MO); Llantoj, aprox. 800 m antes de llegar a Llantoj, de la Florida subiendo hacia el E de la Serranía de Kaska Orcko, 19°09'46"S, 64°03'42"W, 1750 m, 15 Dec 2004, *J. Gutiérrez R. 1072* (HSB, MO); ca. 7 km de Monteagudo, inicio del cañón Heredia, 19°47'17"S, 64°02'08"W, 1127 m, 13 Dec 2006, *H. Huaylla et al. 2178* (HSB, MO); Sud Cinti, ca. 3 horas en caballo al NW de la comunidad de Orocote entre los Ríos Limonal y Cochayo, 20°47'S, 64°21'W, 1650 m, 29 Apr 2005, *R. Lozano 1207* (HSB, MO); Parque Nacional y área natural de manejo integrado de la Serranía del Iñao, cuenca del Río Limón, 19°44'01"S, 63°54'52"W, 1247 m, 15 Dec 2006, *E. Portal et al. 108* (HSB, MO); foot of Cerro Urkhal path before 2nd river crossing, 19°48'S 63°57'W, 1300 m, 4 Oct 2000, *K. Wendelberger 170* (HSB, MO); Rio Limón Valley between Padilla and Monteagudo, 1500 m, 1 Jan 1995, *J.R.I. Wood 9104* (NY); **Santa Cruz**: Vallegrande, Alto Seco, cima de Peña Blanca, 19°00'03''S, 64°01'30''W, 1925 m, 29 Sep 2011, *L. Arroyo P. et al. 6108* (MO, USZ); parcela temporal de muestreo N° 5, 18°41'58''S, 63°43'37''W, 1640 m, 28 Jul

2011, *G.A. Parada et al. 3523* (MO, USZ); Mairana, La Yunga de Mairana, 18°04'13''S, 63°55'08''W, 2190 m, 15 Nov 2004, *M. Serrano et al. 5482* (NY).

33. Capsicum parvifolium Sendtn.

BRAZIL. **Bahía**: Anguera, Fazenda Retiro, ca. 18 km de Feira de Santana na Estrada do Feijão sentido Ipará, 12º9'42''S, 39º11'2''W, 300-600 m, 22 May 2007, *D. Cardoso & R.M. Santos 1922* (NY, UEC, HUEFS); Maracas, km 6–7 de la route Maracas-Contendas da Sincorá, 25 Nov 1986, *A. Chautems et al. 165* (CEPEC); Morro do Chapéu, Chapada Diamantina, 29 Apr 2006, *M.L. Guedes et al. 12268* (ALCB); Camacã, RPPN Serra Bonita, 9,7 km W de Camacã na estrada para Jacarecí, daí 6 km SW na estrada para a RPPN e Torre da Embratel, 15º23'30''S, 39º33'55''W, 835 m, 13 Feb 2005, *J.G. Jardim 4435* (NY); Serra Preta, camino para Serra Preta, 12°31′55″S, 41°35′14″W, 800 m, 28 Mar 2003, *E. Melo et al. 3646* (HUEFS); Buraco da Duda ou Buracao, 19 Apr 2008, *E. Melo et al. 5658* (EAC, HUEFS); Barra do Mendes, encosta rochosa do Río Verde, 11º48'30''S, 42º11'28''W, 763 m, 17 Dec 2009, *E. Melo et al. 7695* (HUEFS); Jequié, Morro da Torre, 13°53′27″S, 40°07′20″W, 671 m, 13 Apr 2007, *L.P.* de *Queiroz et al. 12964* (HUEFS); **Ceará**: Ceará, F. *Allemão 1229* (US); Santa Quitéria, Fazenda Itatiaia, 26 Apr 1984, *A. Fernandes et al. s.n.* (EAC 12511, JPB); Pedra Branca, Pedra Branca, cong. 227, sub.3, 5º24'0"S, 39º57'36"W, 11 Mar 2014, *M. Mayer 24* (EAC, RB); Crateús, Serra das Almas, around plot 1, Mar 2017, *P.W. Moonlight & T. Särkinen 709* (HUEFS); Meruoca, distr. de Palestina, Sítio São Gonçalo, 23 Feb 2018, *A.F.B. Silva 139* (EAC); Serra das Almas, 26 Feb 2007, *E. Silveira s.n.* (EAC 39778, UFRN 5820); General Sampaio, 15 Mar 2008, *E. Silveira s.n.* (HUEFS 138496); Sobral, Maciço da Meruoca. Sítio Santa Cruz, 7 Apr 2016, *E.B. Souza 3924* (HUEFS); Santa Quitéria, Serra dos Quirino ou dos Pajeú, ca. 8,3 km SW (em linha reta) de Itatira, 04º33'42.34"S, 39º41'27,73"W, Apr 2012, *J.P. Souza et al. 11012* (RB); at same locality, 4º32'60"S, 39º40'60"W, 26 Apr 2012, *J.P. Souza et al. 11029* (EAC, ESA, RB); **Minas Gerais**: Manga, Gleba B, 2 Dec 1989, *M.B. Horta et al. 126* (BHCB); **Paraíba**: Maturéia, Serra de Teixeiros, Pico do Jabre, 28 Feb 1994, *M.F. Agra et al. s.n* (JPB); Campina Grande, São José da Mata, Fazenda Pedro da Costa Agra, estrada para Soledade, 7°46'S, 35°52'W, 500–510 m, 2 Apr 1988, *M.F. Agra 658* (JPB); Pico do Jabre, 25-27 Mar 1994, *M.F. Agra et al. 2569* & *2639* (JPB); Pico do Jabre, 07°11'10"N, 37°25'53"W, 800-1010 m, 18-21 Jan 1998, *M.F. Agra et al. 4776, 4903* & *5171* (JPB, MO); Pico do Jabre, ao Norte da sede da Telpa, em direção Leste, 07°11′10″N, 37°25′53″W, 800–1010 m, 10-23 Apr 1998, *M.F. Agra et al. 5249* (JPB, MO, P) & *5297* (JPB, MO); same locality, 7-8 Apr 1999, *M.F. Agra et al. 5479* (JPB); Pico do Jabre, 30 Mar 2009, *M.F. Agra & G.E. Barboza 7075* (CORD, JPB); São José dos Cordeiros, RPPN-Fazenda Almas, Area I, trilha para casa de Comadre Florzinha, 07°28′37″S, 36°53′16″W, 15 Feb 2003, *M.R. Barbosa et al. 2674* (JPB); RPPN-Fazenda Almas, camino para Pedra da Bola, 3 Mar 2004, *M.R. Barbosa et al. 2895* (JPB); RPPN-Fazenda Almas, trilha para parcela, 10 Mar 2007, *M.R. Barbosa et al. 3127* (JPB); Distr. São José da Mata, 20 May 1992, *L.P. Félix 4994* (JPB); Aguiar, Serra de Santa Catarina, 7º1'37"S, 38º10'23"W, 20 Apr 2015, *P. da C. Gadelha Neto & I.B. Lima 3993* (JPB, NY, RB); Rio do Cardoso, 600 m, 19 Apr 2005, *A.V. Lacerda & F.V. Barbosa 415* (JPB); same locality, 21 Jul 2005, *A.V. Lacerda & F.M. Barbosa 471 & 486* (JPB); RPPN-Fazenda Almas, Manga da Cabeça dos Cachorros, 600 m, 12 Apr 2007, *I.B. Lima & J.R. Lima 526* (JPB); Soledade, Comunidade Cachoeira, 521 m, 2 Jun 2006, *R.F. Lucena & A.C. Silva 227 & 228* (PEUFR); Queimadas, Sítio Lucas, próximo à subestação, LT GAR-CG3, T 193-3, 27 May 2014, *M. Oliveira*

5795 (IPA); RPPN Fazenda Almas, Parcela (Diversidade), 29 Apr 2008, *M.C. Pessoa & J.R. Lima 323* (JPB); Puxinanã, em uma cerca viva, 24 Jun 1935, *B.J. Pickel 3892* (IPA, US); **Pernambuco**: Sertânia, Estrada vicinal saindo da PE indo para Olho D'Agua dos Silva, 7°54'06.20''S, 37°15'03.20''W, 672 m, 13 Jan 2010, *J.G. Carvalho-Sobrinho et al. 2518* (BHCB); Ipubi, Estrada Serra Branca-Serrolândia, 15 Feb 1984, *G. Fotius & I.B. Sá 3769* (HTSA, IPA, MAC); Centro da Caprino-Ovino-Cultura do I.P.A., 8°4'25″S, 37°12′24″W, 610 m, 4 Apr 2001, *R.M. Harley & A.M. Giulietti 54171* (HUEFS, K); Buíque, 27 Feb 2004, *R.F. Lucena & U.P. Alburquerque 91* (JPB) & *92* (PEUFR); Triunfo, Estrada para Santa Cruz, Fazenda Bom Jesus, 26 Feb 1986, *V.C. Lima et al. 75* (IPA); Alagoinha, no Km 8 da BR 232, faz. Cajueiro Seco, 14 Jan 1998, *G.F.A. Melo et al. 221* (CEPEC, SP, UFP); Ouricuri, bota-fora da Mina de gipsita da Voltarantin, 14 Apr 2010, *A.M. Miranda et al. 6148* (HCDAL, HTSA, HST, HUEFS, JPB, RB); Serra Talhada, PE-418, divisa PE-PB, Serra Pintada, 647 m, 8 Apr 2015, *M. Oliveira & J.R. Silva 5862* (HUEFS); Gravatá, Tapéra, Mount "Cruzeiro," Jul 1926, *B.J. Pickel 2492* (IPA, US); **Piauí**: Cocal, Pinga, 15 Mar 2003, *E.M.F. Chaves et al. 182* (IPA, JPB); São José do Piauí, Morro do Baixio, 21 Feb 2000, *M.R.A. Mendes et al. 292* (JPB); encosta do Morro do Baixio, 20 Dec 2001, *M.R.A. Mendes & R.S. Albino 509* (JPB); Morro do Baixio, 10 Feb 2002, *M.R.A. Mendes et al. 539* (JPB); Buriti do Montes, Serra da Lagoa, Jan 1907, *E. Ule 7475* (L); **Rio Grande do Norte**: Cerro Corá, south of Cerro Cora, 700 m, Mar 1972, *B. Pickersgill RU72–366* (CORD, IPA, MBM); Acari, nas proximidades do Km 17 da BR 427, sentido Currais Novos-Acari, 6º19'53"S, 36º37'29"W, 378 m, 26 Feb 2011, *A.A. Roque et al. 885* (HUEFS, RB, UFRN); Jucurutu, RPPN Stoessel de Britto, Serra do Estreito, 6 Jun 2008, *A.A. Roque 1061* (ASE); São Tomé, Conglomerado RN 93 04-10-3F8, 6º07'12''S, 36º10'48''W, 23 Aug 2014, *L.A.S. Santos 1237* (RB, UFRN).

COLOMBIA. **Atlántico**: Barranquilla and vicinity, Oct 1928, *Bro. Elías 576* (US); Savanilla, 31 May 1874, *O. Kuntze 1811* (NY). **Magdalena**: Santa Marta, near Mamatoca, 100 ft, 23 Aug 1898-1899, *H.H. Smith 1176* (BM, CORD, E, GH, K, L, MO, NY, P, UC, S, US); Santa Marta, hill 3 miles inland from Playa Brava, 500 ft, 6 Jun 1898/1899, *H.H. Smith 1183* (F, K, MO, NY, US).

VENEZUELA. **Aragua**: M. Briceño Iragorry, 4–5 km al NE de Bahía de Cata, 150 m, 13 Apr 1981, *G. Carnevali et al. 531* (VEN); Sendero Cata-Catica, PN H. Pittier, 10 May 1992, *M. Castro & A. Magallanes 39* (MY, VEN); Ocumare de la Costa, 10 Jul 1937, *H. Pittier 14032* (US, VEN); **Carabobo**: Distrito Valencia, margen derecha del Río Las Marías, Carretera La Belén-Las Marías desde Trincheras, 350 m, 25 Aug 1991, *C. Benítez de Rojas & F. Rojas 4202* (VEN, MY, UOJ); Porto Cabello, [year] 1917, *H.M. Curran & M. Haman 1181* (GH, US); **Distrito Federal**: Vargas, Parroquia Catia La Mar, Esc. Naval, ca. 10°36'N, 67°02'W, 5 m, 10 Oct 1988, *N. Ramírez 2548* (MY, NY, VEN); **Lara**: Iribarren, arriba del caserío Simara, ca. 8 km de Bobare, 20 May 1978, *C. Burandt et al. 193* (VEN); Urdaneta, 8 km vía desde Aguada Grande vía Siquisique, 15 Jul 1980, *R.F. Smith V. 9349* (MY, VEN); **Sucre**: Isla Los Venados, south of Playa Las Barracas Azules, 0–50 m, 10 Sep 1973, *J.A. Steyermark et al. 108086* (US, VEN); Playa Manzanillo, west of Hotel Cumanagoto and Cumaná, 0–2 m, 8 Sep 1973, *J.A. Steyermark et al. 108194* (MO, US, VEN); **Nueva Esparta**: Is. Margarita, El Valle, river trail, 15 Jul 1901, *O.O. Miller & J.R. Johnston 255* (GH, MO, NY, P, US).

34. Capsicum pereirae Barboza & Bianchetti

BRAZIL. Bahía: Boa Nova, PN de Boa Nova, setor sul, Fazenda Liberdade, 12°41’25’’S, 38°57’31’’W, 1 Mar 2013, *L.Y.S. Aona 2046B* (RB); Arataca, Serra das Lontras, ca. 7 km no ramal que liga o povoado de Itatinguí à Serra das Lontras, ca. 15º10’25’’S, 39º20’30’’W, 600-700 m, 12 Feb 2005, *J.G. Jardim et al. 4345* (NY); Rod. Arataca/una entrada a direta no assentamento Santo Antônio 9,5 km, vicinal para Fazenda Palmeira 8,9 km da entrada, serra do Peito de Moça, RPPN Palmeira/IESB, 15º10’27’’S, 39º20’22’’W, 450 m, 18 Dec 2005, *J.G. Jardim et al. 4860* (BHCB, CAS, MO, NY); Almadina, Serra do Concovado, Rod. Almadina/Coaraci, ca. 5 km, 14º42’13’’S, 39º36’9’’W, 300-900 m, *J.L. Paixão et al. 863* (NY); Fazenda Cotermaia, entrance 1.2 km E of Boa Nova on road to Dario Meira, 14º22.419’S, 40º11.305’W, 810 m, 18 May 2001, *W.W. Thomas & S. Sant’Ana 12496* (NY); **Espírito Santo**: Castelo, Domingos Martins, a 11.5 km do entroncamento da BR 262/Vargem Alta, estrada para Caxixe Alto, 20º29’S, 40º57’W, 18 May 1992, *L. Bianchetti et al. 1270* & *1273* (CEN); beira da estrada de terra que liga Castelo à Venda Nova do Imigrante, 20º26’43’’S, 41º05’32’’W, 1000 m, 22 May 1999, *L. Bianchetti et al. 1567* (CEN); Santa Teresa, Estação Biológica de Santa Lúcia, 27 Jan 2000, *V. Demuner & E. Bausen 628* (RB); Alfredo Cháves, São Bento de Urânia, 8 Oct 1994, *G. Hatschbach 61141* (MBM); Caxixe Alto, cerca del Morro Forno Grande (en la propiedad de R. Uliana, vecina a la Fazenda L. Campos), a 11 km de la ruta BR-262, 1000 m, 28 Mar 1986, *A.T. Hunziker 25137* (CORD); próximo do Morro Forno Grande, a 1 km de Caxixe Alto em entrada de terra a 11 km da BR-262, 20°32’S, 41°07’W, 1000 m, 28 Mar 1986, *E. Lleras Pérez et al. 2179* & *2181* (CEN, CORD); Iúna, Serra do Valentim, Floresta do Senhor Aristides, próximo ao transecto 1, 21.8572ºS, 42.0483W, 25 Feb 2014, *J.P. Zorzanelli 957* (BHCB, VIES); Serra do Valentim, Floresta do Senhor Aristides, 27 Mar 2014, *J.P. Zorzanelli 968* (BHCB, VIES); **Minas Gerais**: Lima Duarte, Conceição do Ibitipoca, Reserva Florestal do Ibitipoca, Gruta do Pião, 21º42’S, 43º53’W, 29 May 1992, *L. Bianchetti et al. 1346, 1347, 1348, 1349* & *1350* (CEN); Parque Estadual (PE) do Ibitipoca, mata da Gruta do Pião, 21º42’11’’S, 43º52’18’’W, 16 May 1999, *L. Bianchetti et al. 1558* (CEN); PE do Ibitipoca, 20 Apr 2010, *B. Carvalho et al. 24* (RB); PE do Ibitipoca, Cachoeirinha, 21º40'19"S, 43º52'28"W, 1522 m, 31 Feb 2004, *R.C. Forzza et al. 3329* (RB); PE do Ibitipoca, trilha da mata grande, 21º42'S, 43º00’W, 1600 m, 22 Nov 2004, *R.C. Forzza et al. 3610* (RB); PE do Ibitipoca, 30 Jun 2006, *R.C. Forzza et al. 4209* (MBM); Gruta dos Moreiras, 23 Nov 2006, *R.C. Forzza et al. 4366* (BHCB, CEPEC, K, NY, RB, SPF); Santa María do Salto, Talismã, RPPN Loredano Aleixo (Fazenda Duas Barras), 16º24’47’’S, 40º02’50’’W, 816 m, 30 Oct 2013, *L.L. Giacomin et al. 1946* (BHCB, HUEFS, NY, RB); same locality, 16º24’10’’S, 40º03’23’’W, 864 m, 31 Oct 2013, *L.L. Giacomin et al. 1959* (BHCB); PE do Ibitipoca, Gruta dos Três Arcos, 21º40’40.1’’S, 43º53’00.2’’W, 1666 m, 26 Jan 2010, *J.C. López et al. 78* (SPF); Nanofloresta Nebular, Gruta dos Moreiras, 15-20 m da entrada da gruta, 1650 m, 18 Feb 2017, *G.B. Oliveira 360* (CESJ); Gruta dos Três Arcos, 15-20m da entrada da gruta, 18 Feb 2017, *G.B. Oliveira 371* (CESJ); Gruta dos Moreiras, 21 Mar 2017, *G.B. Oliveira 387* (CESJ); PE do Ibitipoca, Gruta da Cruz, 15-20 m da entrada da gruta, 1660 m, 21 Mar 2017, *G.B. Oliveira 395* (CESJ); Caldas, Pocinhos do Rio Verde, Pedra Branca, 28 Mar 2008, *M.G. Rezende et al. 100* (BHCB); Conceição de Ibitipoca, circuito do Pico do Pião, á beira da trilha, à direita subindo para o Pico do Pião, pouco antes da entrada da Gruta do Pião, 21 Dec 2010, *I.M.C. Rodrigues & M.M. Vieira 446 & 447* (BHCB); PE de Ibitipoca, trilha para a Gruta das Bromélias, 21º42'33.3’'S, 43º55'00"W, 1450 m, 30 Nov 2004, *E. von Sohsten Medeiros et*

al. 353 (CEN, RB); PE do Ibitipoca, 1400 m, 17 Dec 1986, *H.O. Souza et al. 9081* (BHCB, RB); Serra de Ibitipoca, Pico do Pião, 1500–1600 m, 15 May 1970, *D. Sucre & Pe. L. Krieger 6863* (CEN, CORD, RB: 3 sheets); Felício dos Santos, APA Felício, 18º10'S, 43º17'W, 1000-1400 m, 8 Oct 2004, *P.L. Viana et al. 2253* (BHCB); **São Paulo**: Bananal, a unos 25 km al sur de Bananal, por Ruta SP 247, rumbo a Sertão do Bocaina, 26 Feb 2006, *G.E. Barboza et al. 1651* (CORD).

Cultivated
BRAZIL. Minas Gerais: Viçosa, plants cultivated at Universidade Federale de Viçosa by V. Casali (seeds from Caxixe Alto, ES), 7–14 Dec 1986, *A.T. Hunziker 25247, 25248* & *25249* (CORD).

35. Capsicum piuranum Barboza & S.Leiva
PERU. Piura: Huancabamba, carretera Canchaque-Huancabamba, km 98-99 (ca. 20 km de Canchaque rumbo a Huancabamba), subiendo a Cuello del Indio, 05º22'43''-05º22'44''S, 79º33'34''-79º33'37''W, 2341-2346 m, 8 May 2017, *G.E. Barboza & S. Leiva González 4841* & *4842* (CORD); Morropon, Chalaco, Bosque Mijal, 05°03'51.1"S, 79°43'25.9"W, 2800 m, 1 Nov 2015, *M. Cueva et al. 2655* (USM); same locality and date, *M. Cueva et al. 2656, 2657 & 2658* (USM); Huancabamba, carretera Huancabamba-Canchaque, a una hora del Abra Cruz Blanca, 05°22'25"S, 79°34'02"W, 2860 m, 13 Apr 2017, *M. Cueva 2912* (USM); distrito Canchaque, km 98-99 (ruta Canchaque-Huancabamba), 5º 22'44.1"-5º 22'42.7"S, 79º 33'37.2"-79º 33'34.1" W, 2341-2346 m, 8 May 2017, *S. Leiva González & G.E. Barboza 6561* & *6562* (HAO);

36. Capsicum pubescens Ruiz & Pav.
ARGENTINA. Jujuy: Gral Belgrano, Yala, cult. patio de vivienda, 24 Mar 2009, *H. Keller et al. 6943* (CTES); **Salta**: Capital, cult. en Jardín de A. Cuvei, 28 Mar 1995, *A.T. Hunziker 25484* (BM, CORD).

BOLIVIA. Without locality, Jul 1941, *I. von Rentzell s.n.* (SI 14485 pp); **Beni:** Gral. Ballivián/Yucuma, Estación Biológica del Beni, comunidad Charatón, en el chaco de Francisco Bie, 14º30'S, 66º37'W, 200 m, 27 May 1995, *I. Guareco & J. Balderrama 464* (LPB); **Chuquisaca**: Tomina, Campo Redondo, km 496, rumbo a Sucre, ingresando unos 700 m de la ruta (cult. Sr. Romelio Chenaud), 19º18'08''S, 64º19'46''W, 2066 m, 16 Dec 2017, *G.E. Barboza et al. 4923* (CORD); **Cochabamba**: Chapare, Espíritu Santo, 1891, *M. Bang 1185* (CORD, E, F, GH, K, LE, M, MO, US, Z); on new road to Todos Santos, about 115 km NE of Cochabmaba, near Chimoré, 800 m, 10 Mar 1939, *W.J. Eyerdam 24801* (UC); Carrasco, trail from Estancia Sehuencas along Río Fuerte, ca. 1.2 km E of Rio Monte Puncu, cult. or a remnant cultivar, 2200 m, 13 May 1994, *N. Ritter 989* (LPB, NY, GH, MO, WIS); **La Paz**. vic. La Paz, 10,000 ft, 1890, *M. Bang 200* (CORD, E, F, G, GH, K, L, LE, M, MO, R, UPS, US, Z); Yungas, 1890, *M. Bang 727* (F, K, MO, US); Sud Yungas, Salto Velo de la Novia, a 19 km del desvío hacia Chulumani viniendo desde Unduavi, 14º12'07,2''S, 73º52'56.9''W, 2160 m, 26 Apr 2006, *G.E. Barboza et al. 1847* (CORD); Nor Yungas, Coroico, a una cuadra de la terminal de ómnibus, cult., 16º11'27''S, 67º43'35''W, 6 Dec 2017, *G.E. Barboza et al. 4889* (CORD); Huancané, justo en el desvío

hacia Apa-Apa, 16º22’38’’S, 67º31’56’’W, 1823, 6 Dec 2017, *G.E. Barboza et al. 4890* (CORD); frente de Florida, otro lado del Río Unduavi, finca La Glorieta, 16º22’S, 67º44’W, 1700 m, 11 Apr 2009, *S. Beck 33151* (LPB); Sirupaya bei Yanacachi, 2100 m, cult., 14 Dec 1906, *O. Buchtien 338* (G, US); Polo Polo bei Coroico, 1100 m, Oct/Nov 1912, *O. Buchtien 4033* (GH); Milluguaya, 1300 m, Dec 1917, *O. Buchtien 4033* (GH, US); Larecaja, Hacienda Simaco, sobre el caminho a Tipuani, cult. Feb 1920, *O. Buchtien 5542* (US); Inquisivi, Quimé, Jardín Botánico, 17º02’S, 67º15’W, cult., 3100 m, 20 Dec 1989, *L.J. Dorr 6729* (CAS, CTES, LPB, MO, NY, SI); Triananear Coroico, 1 May 1974, *J.A. Duke & H.F. Winters 17330* (MO); Mercado Belzu, said to be from the Yungas, 21 Feb 1971, *W.H. Eshbaugh 765* (GH); Coroico, a 1 km camino a Caranavi, 16º10'S, 67º43'W, cult., 20 Nov 1999, *F. Fonturbel FR-17* (LPB); along the trail between Hospital San Antonio de Quime and the Rio Chichipata, 16º58’S, 67º13’W, 3000-3100 m, cult., 15 May 1988, *M. Lewis 88629* (LPB, MO); Sorata, in hortis colitur, 2200 m, Apr 1898, *G. Mandon 428* (P); Huancané, 16º22’S, 67º32’W, 2000 m, cult. or perhaps ruderal, 29 Sep 1985, *M. Nee & J. Solomon 32018* (LPB, MO, NY); cultivated at hotel in Chulumani, 16°25'S, 67°32'W, 1750 m, 11 May 2001, *M. Nee 51786* (LPB, MO, USZ); Quime, 115 km (línea recta) del centro de la ciudad de La Paz, en terrenos baldíos, 16º58’30’’S, 67º13’W, 3150 m, 12 Jan 1989, *M. Saldías P. 606* (CTES, MEXU, NY, USZ); Chulumani, camino a Granja “Loma Linda”, 1740 m, 1 Sep 1947, *R. Scolnik & R. Luti 526* (CORD 00006959); Tusihuaya, 1740 m, 2 Sep 1947, *R. Scolnik & R. Luti 526* (CORD 00006957 & 00006958); Murillo, 16°30'S, 68°08'W, 3600 m, 7 Mar 1980, *J.C. Solomon 5305* (LPB, MO); 45.5 km below the dam at Lago Zongo, cult., 16º03’S, 68º01’W, 1200-1400 m, 23 Dec 1984, *J.C. Solomon 12991* (LPB, MO); 36 km below the dam at lago Zongo, vicinity of Chururaqui hidroelectric plant, cult., 16º06’S, 68º04’W, 1900-2100 m, 20 Jan 1985, *J.C. Solomon 13104* (LPB, MO); La Florida, 16º31’S, 68º05’W, 3300 m, 13 Aug 1986, *J.C. Solomon 15508* (LPB, MO); Quime, 16º58’S, 67º13’W, 3000 m, cult., 13 Mar 1988, *J.C. Solomon & M. Nee 18181* (LPB, MO, USZ); Muñecas, Camata, Charazani, 15º14'S, 68º43'W, 3000 m, 16 Jun 2004, *J. Terrazas 1*, *2*, *4*, *5* & *6* (LPB); Coroico, 1891, *M. Weddell s.n.* (P00452435); **Santa Cruz**: Vallegrande, alrededores de Moro Moro, camino del basural y cementerio, a la vera del camino, 18°22'32.4"S, 64°19'16.1"W, 2323 m, 16 Feb 2017, *C. Carrizo García & L. Amarilla 35* (CORD); Loma Larga, a 60 km al sur de Vallegrande, a 300 m al este de Loma Larga, 18º45'26"S, 63°53'41"W, 1901 m, cult., 8 Apr 2006, *R. Hurtado 698* (LPB); Huasacañada, 5 km al S de la ciudad de Vallegrande, 18º31’S, 64º5’W, 2050 m, 31 Dec 1988, *M. Saldías P. 563* (NY, USZ); Florida**,** Alto de Mairana, 16 Oct 1928, *J. Steinbach 8288* (CORD, GH, K); Huasacañada, 7 Jan 1989, *I. Vargas C. 30* (LPB, USZ); Huasacañada, 18º31.5’S, 64º5,8’W, 2050 m, márgenes de terrenos cultivados, 10 Feb 1991, I. *Vargas C. 932* (CTES, LPB, NY, USZ) & 19-22 Feb 1993, *I. Vargas C. et al. 1979* (CTES, F, MO, USZ).

COLOMBIA. **Antioquia**: La Ceja, Vereda El Tambo, 6º00’N, 75º26’W, 2200 m, 22 Mar 2005, *Estudiantes Herbario MEDEL 709* (MEDEL); planicie de selva abierta y esparcida, 2400 m, 17 Apr 1949, *F. Silva et al. 19An404* (CORD, MEDEL, US); **Cundinamarca**: Salto de Tequendamama, al lado norte de La Planta, 2200 m, 1-3 Oct 1938, *J. Cuatrecasas 173* (F, US); **Huila**: San Agustín, alrededores del Parque Arqueológico, 1600-1700 m, 5 Dec 1957, *R. Romero Castañeda 6674* (COL); **Nariño**: Corregimiento de Chachaguí, 1800 m, 22 May 1980, *O. de Benavides 2246* (PSO); Mallama-Chucunés, 1500 m, 24 Jun 1980, *G. López Jurado & J.S. Riascos 613* (PSO); Potosí, Cuaspud, 3000 m, cult., 5 Aug 1993,

B.R. Ramírez P. 5639 (PSO); Pasto, Volcán El Galeras, ca. 3200-3500 m, 4 Jun 1946, *R.E. Schultes & M. Villarreal 7958* (COL, GH, NY, US); **Putumayo**: Sibundoy, 2200 m, 8 Sep 1979, *O. de Benavides 1986* (PSO); Santiago-Colón, 2250 m, 26 Feb 1986, *O. de Benavides 6190* (PSO); Valle de Sibundoy, 5 km S Sibundoy (Indian garden), ca. 2200 m, 12 Jun 1963, *M.L. Bristol 1115* (COL, PSO, US) & 22 Aug 1963, *M.L. Bristol 1335* (COL, GH, PSO, US); alrededores de Sibundoy, 2250 m, 18 Feb 1942, *R.E. Schultes 3269* (GH); Valle de Sibundoy, 2225-2300 m, 29 May 1946, *R.E. Schultes & M. Villarreal 7598* (CAS, COL, F, GH, US).

COSTA RICA. Guanacaste: Puntarenas, PN Corcovado, Península de Osa, Sendero El Mirador, 20 Sep 1995, *E. Fletes & L. Angulo 239* (BM); Alajuela, San Ramón, Cordillera de Guanacaste, Monteverde, San Gerardo Biological Station, road to Río Caño Negro, 24 May 1995, *D. Penneys 502* (BM).

ECUADOR. Without locality, 1800-2400 m, *F.C. Lehmann 4949* (CORD, K); **Azuay**: in a Cholo Garden, about 3 km north of Sevilla de Oro, 8,000 ft, 28 Aug 1945, *W.H. Camp E-5031* (CORD); Paute, Parroquia G. Ortega, 2º49’02’’S, 78º45’28’’W, 2440 m, cult., 5 Jul 1991, *C.E. Cerón 15577* (MO, QCNE); Carretera Cuenca-Paute-Sevilla de Oro, entre Paute-Sevilla de Oro, 2490 m, 8 Aug 1986, *J. Jaramillo 8926* (AAU, F, MO, NY, QCA); Loma de la Plata, slopes bordering Río Putucay, at Chacanceo, 1430-1465 m, 22 May 1943, *J.A. Steyermark 52690* (CORD fragment, F, VEN); **Canar**: Azoques, Parroquia L. Cordero, 02.44S, 78.44 W, 2880 m, cult., 15 Jun 1991, *C.E. Cerón 15237* (MO, NY, QCNE); **Carchi**: Maldonado-Tulcán road, ca. Km 20, 0º55’N, 78º68’W, 2400-2600 m, 7 Oct 1981, *L. Werling & S. Leth-Nissen 371* (L, MO, QCA); **Chimborazo**: Penipe, Bayushig, carretera a Matus, 01º33’S, 78º33’W, 2800 m, 19 Jan 2002, *G. Echeverria 915* (CHEP); Alausí (la Moya), a 15 m de la Escuela E. Alfaro, 1365 m, 4 Jan 2004, *S. Moina Z 23* (CHEP); **Imbambura**: cantón Otavalo, Hacienda “Rosa Pamba”, 2850-3000 m, 15 May 1944, *M. Acosta Solís 8047* (F); Hacienda Pinsaquí, near Otavalo, cult., 7 Jul 1966, *H.V. Pinkley 359* (GH); **Loja**: Matupe (6 km N of Loja), 2150 m, 8 Oct 1955, *E. Asplund 18050* (MO, S); 2 km South of Saraguro, 03º38’S, 79º14’W, 2500 m, 14 Nov 1988, *L. Ellemann 66689* (AAU, LOJA, QCA, QCNE, MO); Namanda, 1600 m, 24 Nov 1946, *R. Espinosa 1080* (CORD); cultivated plants of the R. Espinosa Gardens, University of Loja, 4º2’1’’S, 79º12’24’’W, 2300 m, 23 Jul 1977, *J. Hart 953* (GH, LOJA, US); **Napo-Pastaza**: Tena, bank of Rio Tena, 400 m, 8 Apr 1935, *Y. Mexia 7195* (UC); **Pichincha**: along the Road from Quito to Santo Domingo de Los Colorados, 15 Jan 1945, *W.H. Camp E-1739* (S); Urb. Miraflores, 2830 m, 9 Mar 1979, *P. Gómez 3* (QCA); cantón Sangolqui, Barrio Selva Alegre, huerta particular, *F. Mejía 002* (QAP); **Tungurahua**: Baños, 20 Jul 1987, *J. Cascante 6* (CHEP); **Zamora Chinchipe**: Quebrada San Francisco, ca. 25 km E of Loja (12 km E of pass) on Loja-Zamora road, edge of PN Podorcarpus, 3º58.82’S, 79º06.20’W, 2000-2100 m, probably cult., 30 Oct 1994, *S. Knapp et al. 9103* (MO, QCA, QCNE).

GUATEMALA. Alta Verapaz: vicinity of Cobán, cult., 1300 m, 23 Mar-19 Apr 1941, *P.C. Standley 91227* (F); **Chimaltenango**: Finca La Alameda, near Chimaltenango, cult., 1830 m, 11-22 Dec 1940, *P.C. Standley 80793* (F); **Quetzaltenango**: 24 km al S de la desviacíon a Sololá y Ponajache, 4 Apr 1978, *M.T. Germán R. et al. 736* (MEXU, MO); **San Marcos**: vicinity of town of Tajumulco, northwestern slopes of Volcán Tajumulco, around house of

Rio Cusuchimá, 2300-2800 m, 28 Feb 1940, *J.A. Steyermark 36930* (F); **Sololá**: shores of Lago de Atitlán by San Pedro, 1800 m, 7 Jun 1942, *J.A. Steyermark 47273* (F, US).

HONDURAS. Morazán: Zamorano, 800 m, 30 May 1945, *J. Valerio R. 3039* (F); Zamorano, 800 m, 22 Aug 1945, *J. Valerio R. 3237* (F).

MÉXICO. Chiapas: Tenejapa, slope with Pinus and Quercus, paraje of Kulak'tik, 6000 ft, cult., 11 Aug 1965, *D.E. Breedlove 11696* (CAS, US); Venustiano, Carranza, wooded slope at Rancho Carmen along the road from Acala to V. Carranza, 2600 ft, 18 Jul 1966, *R.M. Laughlin 1324* (CAS); Zinacantán, below Zinacantán Center along the trail to Ixtapa, 6,200 ft, 5 Dec 1966, *R.M. Laughlin 2937* (CAS); Mt. Tacana, 1000-2000 m, Aug 1938, *E. Matuda 2406* (GH, K, MEXU, MICH, NY); Oxchuc, cultivado en huerto familiar, 2000 m, 5 Jul 1981, *V.M. Sánchez León 1139* (MEXU); paraje of Kulak'tik, 5800 ft, Jan-Mar 1964, *A.S. Ton 125* (CAS); at Colonia Ach'lum, cult., 8,700 ft, 27 Dec 1965, *A.S. Ton 398 A* (CAS, MEXU); **Distrito Federal**: El Rosario, cult., 23 Aug 1936, *L.H. McDaniels 698* (CORD fragment, F, K); Mexico city, cult., Sep 1940, *B.P. Reko 41* (GH); **México**: Tezcoco, Texcoco, cult. en invernadero de Fitotecnia, 12 May 1992, *R. Bonilla Baes & E. Monsalvo G. 346* (MEXU); Temascaltepec, Parque Universitario "Las Orquídeas", km 67.5, carretera Toluca-Tejupilco, cult., 19º2'37.23''N, 100º3'5,33''W, 1679 m, 8 Dec 2012, *C.B. Monsalvo J. 12* (MEXU); Tezcoco, ciudad, cult. para ornato, 7 Mar 1981, *M.T. Pulido 511* (CAS, MEXU); Teotihuacán, en el Jardín Botánico del Museo Arqueológico, 14 Oct 1998, *R. Subils & G.E. Barboza 4612* (CORD); **Michoacán**: Zitácuaro, San Francisco Coatepec, Quinta Diana Kennedy, cult. en invernáculo, 19.421ºN, 100.374ºW, 1900 m, 28 Aug 2010, *R. Bye et al. 13* (MEXU); Hidalgo, El Caracol, 13 km al SE de Mil Cumbres, cult., 2000 m, 23 Apr 1984, *J. Soto Núñez et al. 6369* (MEXU); 4 km al NE de San Felipe de Los Alzati, cult. en huerto familiar, 1940m, 16 May 1986, *J.C. Soto Núñez & F. Solórzano G. 12592* (MEXU); **Morelos**: Hueyapan, parcelas de cultivo, 3200 m, 30 Sep 1986, *L. Aguilar P. s.n.* (MEXU 945006); **Oaxaca**: Ixltán de Juárez, Tiltepec, 17º31'34''N, 96º19'17''W, 1450 m, 16 Mar 1999, *J. García R. & I. Montaño M. 348* (MEXU, NY); Comaltepec, S. Comaltepec, 17º33'N, 96º31'W, 2000 m, 7 Dec 1987, *L. Hernández García & G.J. Martín 166* (MEXU, MO); S. Comaltepec, 17º33'N, 96º31'W, 2000 m, 19 Jun 1988, *L. López L. & G.J. Martín 135* (MEXU, MO, NY); **Veracruz**: Coatepec, congregación de Zoncuantla, por la carretera vieja Xalapa-Coatepec, cult., 19º30'N, 96º57'W, 1100 m, 1 Nov 1984, *J.I. Calzada 10856* (MEXU); Tlaltetela, Los Bejucos, cerca de los Baños de Carrizal, cult., 180 m, 14 Nov 1977, *G. Castillo C. et al. 214* (F, UC); Juchique de Ferrer, cerro de Villa Rica cerca de la hacienda de La Flor, cult., 19º48'N, 96º46'S, 1130 m, *G. Castillo C. et al. 1757* (F); Xalapa, El Haya, por la carretera antigua a Coatepec, cult., 1350 m, Oct 1982, *M. Chazaro B. 2558* (MEXU); Capilla de Tonayán, hacia el cementerio, 19º41'02''N, 96º55'06''W, 1820 m, 12 Feb 2009, *D. Jimeno Sevilla et al. 967* (MEXU, MO); Tonayán, poblado de Tonayán, 1790 m, 6 Dec 1975, *W. Marquez R. 472* (MEXU, UC, XAL); Ayahualulco, *M. Nee & Taylor 25938* (F); Tlilapan, Tlilapan, 18º48'N, 97º06'W, 1170 m, 28 Mar 1976, *C. Velázquez L. 104* (UC); Zapata, 1300 m, 16 Mar 1974, *F. Ventura A. 9739* (CORD).

NICARAGUA. Sierra de Managua, 600-900 m, *A. Garnier 665* (US).

PERU. Vita [Vitoc?], *Mc. Lean s.n.* (K000585921, CORD 00006969); Peru, *J.A. Pavón s.n.* (BM 000992132, BM 000992133). **Amazonas**: Chachapoyas, vicinity of Leimebamba, upstream along creek flowing through edge of town into the Rio Utcubamba, escaped? but not near habitations, 2250 m, 4 Apr 1964, *P.C. Hutchison & J.K. Wright 4892* (F, MO, UC, US, USM); **Ancash**: Recuay, Marca, cult., 8 Aug 1994, *P. Gamarra 439* (USM); **Apurimac**: Andahuaylas, Andahuaylas, cult. in garden of Dr. O. Usandivaras, 2950 m, 4 Nov 1935, *J. West 3739* (GH, UC); **Cajamarca**: San Ignacio, Huarango, Nuevo Mundo-Nuevo Progreso, margen de la Quebrada Las Juntas, 5º18'30''S, 78º43'W, 1350 m, cult., 23 Jul 1997, *J. Campos de la Cruz & S. Nuñez 4266* (MO, USM); Santa Cruz, Monte Seco, above the village on trail to Charro Blanco, 1500-1800 m, 7 Mar 1998, *N. Dostert 98/162* (F, M); Chota, a 1 km de Paraguay (Ruta Qurocoto-La Granja), huerta de casa, 2350 m, 10 Aug 1994, *S. Leiva González et al. 1427* (F, MO) & *1429* (F, HAO†); Cajamarca, Mun. Jesús, Municipal nursery, cult., 2500 m, 15 Apr 2000, *O.L. Orozco 122* (MO, NY); **Cuzco**: Urubamba, San Miguel, Valley Urubamba, 1800 m, 31 May 1913, *O.F. Cook & G.B. Gilbert 1017* (US); ca. 80 km WNW of Cuzco, Machu Picchu, at Puente Ruinas, ca. 2500 m, 3 Jan 1963, *H.H. Iltis 1067* (MOL); Aguas calientes, ruinas de Machupicchu, a 112 km de Cusco, en el camino ferroviario Cusco-Quillabamba, 13°09'21"S, 72°31'22"W, 2000 m, cult., 24 Mar 1987, *P. Núñez V. 7537* (MO); a 69 km de Cusco, entre Tancacc y Ollanta, 13°16'S, 72°16'W, 2750-2800 m, cult., 5 Dec 1987, *P. Núñez V. 8720* (MO); La Convención, Valle de Santa Ana above Quillabamba, 5800 ft, cult.?, 20 Jan 1975, *T. Plowman & E.W. Davis 4809* (F, GH, USM); Hacienda Amaibamba, 1650 m, 18/19 Jul 1949, *R. Scolnik 821* (CORD) & *822* (BM, CORD, NY, P, SI); **Huánuco**: Huamalíes, Punchao, 3534 m, cult., 19 Apr 1999, *C.O. Adrián 84* (USM); L. Prado, Tingo María, across the Huallaga on trail to Monson, Oct/Feb 1949-1950, *H.A. Allard 21850* (US); Tingo María, cult., 600 m, 2 Jun 1977, *J. Hart 621* (GH); **Junín**: Huancayo, pueblo de Pariahuanca, *Mathews 854* (OXF); Jauja, en un jardín de Concepción, cult., 3300 m, Nov 1948, *C. Ochoa 602* (GH, MOL); **Lima**: Lima, San Isidro, 157 m, cult., 1 Aug 1979, *E. Cerrate de Ferreyra 7644* (MO, USM) & 18 Oct 1980, *E. Cerrate de Ferreyra 7673* (USM); **Loreto**: Alto Amazonas, Puerto Arturo, below Rio Huallaga, below Yurimaguas, 135 m, 24-25 Aug 1929, *E.P. Killip & A.C. Smith 27820* (US); San Antonio, Alto Río Itaya, 145 m, Sep-Oct 1929, *L. Williams 3405* (F); **Pasco**: Oxapampa, cult. en huerto, 10º23'58''S, 75º29'12''W, 2200 m, 28 Oct 2007, *L. Chuck 137* (HOXA, MO, USM); PN Yanachaga-Chemillén, sector San Daniel, 10º26'27''S, 75º26'30''W, 2240 m, 24-28 Feb 2009, *M. Cueva & R. Rivera 484* (HOXA, HUT, MO, USM); PN Yanachaga-Chemillén, sector San Daniel, 10º25'40''S, 75º26'09''W, 2000-3100 m, 23 Feb 2009, *P. Gonzalez 344* (E, USM); ex Pozuzu, 1896, *H. Ruiz & J.A.Pavón s.n.* (MA 815153, MA 815155). **Puno**: Carabaya: Ollachea-San Goban road, just north of Camatane, cult., 15 Aug 1980, *J.D. Boeke & S. Boeke 3027* (F, NY); **San Martín**: Huallaga, abajo de La Morada, en chacras de cultivo, 6°57'S, 77°32'W, 2000-2200 m, 11 Aug 1997, *V. Quipuscoa & L. Bardales 979* (HAO†, HUT).

VENEZUELA. **Mérida**: Palmira, Justo Briceño, 1636 m, 3 Oct 1973, *S. López-Palacios & J.A. Bautista B. 3473* (MO); Miranda, Timotes, 23 Jun 1928, *H. Pittier 12707* (NY, US);

Cultivated

ARGENTINA. Córdoba: Capital, cult. en Córdoba (semillas ex Bolivia cedidas por M.J. Frezzi), May 1949, *A.T. Hunziker 7605* (CORD); cult. en Fac. C.E.F. y Nat. (UNC),

semillas de Santa Cruz-Bolivia, Feb 1992, *E. Moscone 198* (CORD); cult. en Fac. C.E.F. y Nat. (UNC), semillas de Región Central Chanchamayo, Perú, 18 Mar 1992, *E. Moscone 202* (CORD); cult. Invernadero IMBIV (UNC), semillas de Barboza et al. 4889 (Bolivia), 21 Mar 2021, *N. Palombo 21* (CORD); cult. Invernadero IMBIV (UNC), semillas de frutos comprados en Cochabamba (Bolivia), 29 Mar 2021, *N. Palombo 22* (CORD); cult. Invernadero IMBIV (UNC), semillas de frutos comprados en Camiri-Santa Cruz (Bolivia), 29 Mar 2021, *N. Palombo 23* (CORD).

BRAZIL. Rio Grande do Sul: Pelotas, Embrapa Clima Temperado. Estufa. Acesso P303, 29 Jul 2013, *J.C. Villela s.n.* (ECT0003448); **Sao Paulo**: Salto, Fazenda Ituaú - Terra de Gigantes, Estrada Velha Salto Capivari, km 7 (origen Peru), 13 Jan 2011, *P.T.Souza* 21 (UEC).

UNITED STATES OF AMERICA. California: San Marino, cult. Huntington Botanic Garden, 3 Oct 1973, *E. McClintock s.n.* (CAS 569551); Santa Cruz, cult. in the Garden at Santa Cruz (seeds from Chile), 1921, *C.A. Reed s.n.* (CAS 23829); cult. in the Garden at Santa Cruz, (seeds from Peru), Oct. 1923, *C.A. Reed s.n.* (CAS); Davis, grown at University of California, (seed from Guatemala, leg. O.W. Norvell), 1953, *P.G. Smith* (Davis Ac. 1425) (UC); **Indiana**: Bloomington, cult. in Indiana University (IU) greenhouse, seeds P. Smith Ac. 1932 from Tacna (Peru), 1959, *C.B. Heiser s.n.* (CORD 00088578, LIL 471205); cult. in IU greenhouse, seeds from market La Paz, Bolivia (leg. I. Kelly), 1959, *C.B. Heiser C272* (CORD); cult. in IU greenhouse, seeds from patio del Hotel, Nor Yungas, Bolivia (leg. I. Kelly), 1959, *C.B. Heiser C273* (CORD); cult. at IU Experimental Field, seeds from Trujillo market, Peru (leg. P. Smith PS 149), 6 Oct 1960, *C.B. Heiser s.n.* (CORD 00088599); cult. Bloomington (seed originally from San Juan Ixcoy, Guatemala), 15 Sep 1948, *C. B. Heiser Jr. #C1* (CORD 00091452); **Missouri**: cult. greenhouse Missouri Botanical Garden, seeds from Tlatlauhuitepec, Veracruz-Mexico (leg. Carl O. Sauer), Jul 1948, *J. Sauer s.n.* (CORD 00088598, LIL 363174)

37. Capsicum rabenii Sendtn.

BRAZIL. Without locality, 16 Dec 1889, *A. Glaziou 1002* (CORD, P); **Bahía**: Porto Seguro, Parque Nacional Monte Pascoal, 26 Apr 1967, *J. Lanna & A. Castellanos 26542* (HB); Bonito, Buraco da Duda ou Buracão, 19 Apr 2008, *E. Melo et al. 6545* (HUEFS); **Distrito Federal**: Chapada da Contagem, limestone outcrop (Companhia Fercal), near Córrego Landím, ca. 25 km NE of Brasilia, ca. 1000 m, 2 Feb 1968, *H.S. Irwin et al. 19438* (F, G, UB, UC, US); Brasilia, Parque rural, 15° 43'0" S, 47° 53'60" W, 1110 m, 1 Feb 1983, *M.J. Pires 392* (CEN); same locality, 15º43'44"S, 47º54'7" W, 1110 m, 10 May 1983, *M.J. Pires 433* (CEN); Brasilia, Fercal, 15º31'60"S, 47º51'0"W, 9 Apr 2008, *T.S. Vilar & A.A. Santos 3* (CEN); Brasilia, Campus do Cenargen, na borda da mata do Córrego do Bananal, 15°43’42’’S, 47°54’05’’W, 1005 m, 9 Nov 2010, *B.M.T. Walter et al. 6014* (CEN); **Espírito Santo**: Cachoeiro de Itapemirim, Pacotuba, Reserva Florestal do Bananal do Norte, 110 m, 27 Apr 1972, *D. Sucre & T. Soderstron 8987* (CEN, RB); **Goiás**: Anapolis, 28 km a NW do Trevo Rodoviario Anapolis-Goiania-Brasilia da Rod. BR 153, rumbo a Jaragua (km 1196,5), 16º09’S, 49º05’W, 990 m, 19 Mar 1986, *A. Allen & Werneck 3505* (CEN, JPB); Jataí, UFG, Campus Jatobá, 1 Jul 2010, *E.V. Amaral 38* & *62* (CEN); Rod. GO-050, 8-10 km N de Catalão, 800 m, 29 Nov 1992, *G. Hatschbach et al. 58241* (CORD,

CTES, MBM, Z); Corumbá de Goiás, 50 km N of Corumbá de Goiás on road to Niquelândia, Goiás in valle of Rio Maranhão, ca. 800 m, 24-25 Jan 1968, *H.S. Irwin et al. 19117* & *19158* (F, GH, UB, US); 75 km N of Corumbá de Goiás on road to Niquelândia, Goiás in valle of Rio Maranhão, ca. 700 m, 25 Jan 1968, *H.S. Irwin et al. 19189* (F, K, MO, UB); Catalão, 20 km NE of Catalão, 875 m, 23 Jan 1970, *H.S. Irwin et al. 25203* (LIL, MO, NY, UB); Caldas Novas, margen direita do Rio Corumbá, ca. 4 km da estação de pesca da Fazenda Jacuba, 17°50'S, 48°35'W, 600 m, 12 Dec 1993, *G. Pereira-Silva et al. 2100* (CEN, MO, NY, RB); Minacu, ca. 200 m a montante da barragem, na margem esquerda do rio Tocantins, 13º23'35"S, 48º08'43"W, 310 m, 20 Feb 2001, *G. Pereira-Silva et al. 4678* (CEN); Alexânia, Fazenda Cafundó, 16º18'35''S, 48º34'54''W, 880 m, 19 Feb 2003, *G. Pereira-Silva et al. 7216* (CEN); Goyas, 1816/1821, *A. Saint Hilaire 764* (CORD fragment, P00409994, P00409995, P00409996); São Domingos, Fazenda Flor de Ermo, 13º37'01"S, 46º46'09"W, 495 m, 25 Mar 2000, *A.C. Sevilha & A.O. Scariot 1945* (CEN); fragmento perturbado da Fazenda Flor do Ermo, 13º36'29"S, 46º46'23"W, 460 m, 15 Apr 2002, *A.C. Sevilha & A.O. Scariot 2137* (CEN); Padre Bernardo, Taboquina, 15º18'S, 48º10'W, 23 Apr 1991, *R.F. Vieira 700* (CEN); Hidrolândia, Fazenda Barreirão, 2 Jan 1997, *B.M.T. Walter 3628* (CEN); **Mato Grosso**: Barra do Garças, Vale de Sonhas, ca. 93 km S from Xavantina on Aragarças road, 14°38'S, 52°14'W, 9 Nov 1968, *R.M. Harley et al. 10986* (K, NY, P, U, UB); **Minas Gerais**: Visconde do Rio Branco, 23 Mar 1979, *E.C. Almeida 48* (VIC); Viçosa, 12 Jan 1949, *P. de T. Alvim 310* (CORD, F, LIL, P); Juiz do Fora, 2 Mar 1985, *Anonymous s.n.* (RB 01366938); Passa Quatro, beira da rodovia para Cruzeiro, 27 Feb 2008, *L.C. Bernacci et al. 4467* (ESA, IAC); Lima Duarte, caminho Lima Duarte-Reserva Florestal da Serra de Ibitipoca, a 19 km da praça central de Lima Duarte, 20º44'S, 43º53'W, 800 m, 19 Apr 1986, *L. Bianchetti et al. 504* & *505* (CEN, CORD); Caldas, biera da estrada para Pedra Branca, a 3,3 km do trevo da saída de Pocinhos do Río Verde para la Cachoeira da Cascata, 21º57'15"S, 46º24'09"W, 1100 m, 13 May 1999, *L. Bianchetti et al. 1553* (CEN); Maria da Fé, estrada para o Pedro, perto da cachoeira, 4 km da entrada da Fazenda Pomária, 22º18'51"S, 45º22'27"W, 1280 m, 14 May 1999, *L. Bianchetti et al. 1555* (CEN); Santos Dumont, São Sebastião da Boa Vista, 12 Apr 2013, *B.E. Conde 186* (CESJ); Entre Rios de Minas, Fazenda da Pedra Branca, 30 Jan 1977, *M.P. Coons 77-162* (VIC); Entre Rios, Apr 1833, *H.C. Dent 90* (BM); Campus Dom Bosco, *T.N. Dias s.n.* (HUFSJ 609); Bairro de Lourdes, Rua Papa João XXIII em terreno baldio, 5 Mar 1999, *A.M. Fontes s.n.* (VIC 23032); Lagoa Santa, *A. Glaziou s.n.* (CORD 86141, P); Paraopeba, Lagoa Preta, 18 Nov 1958, *E.P. Heringer 6472* (UB, US); Serra da Mantiqueira, cerca de Conceiçao do Ibitipoca, rumbo al Parque Estadual de Ibitipoca, 800 m, 19 Apr 1986, *A.T. Hunziker 25202* (BM; CORD); Entre Rios de Minas, Faz. da Pedra, 7 Nov 1969, *P.L. Krieger 7470* (CESJ, HUFU, MBM, SPF); Juiz do Fora, 5 Feb 1977, *P.L. Krieger s.n.* (VIC 5212); Viçosa, 14 Jan 1935, *J.G. Kuhlmann s.n.* (CORD 00088590, VIC 2603); Caldas, May 1854, *G.A. Lindberg 176d* (S); Caldas, Jun 1854, *G.A. Lindberg 177* (BR); Araponga, Estrada entre Estêvão de Araújo e Araponga, a 4 km de Estêvão de Araújo, 20º38'S, 42º31'W, 810 m, 25 Mar 1986, *E. Lleras Pérez et al. 2142* (CEN, CORD); Arcos, Fazenda Faroeste, margem direita do Rio São Miguel, 20º15'05.7''S, 45º39'46.7''W, 1 Feb 2003, *P.H.A. Melo & J.A. Lombardi 381* (BHCB; HUEFS); Viçosa, road to São Miguel, near Km 4, 3 Jan 1930, *Y. Mexia 4205* (MBG, NY); Caldas, 16 Dec 1859, *A.F. Regnell III 1002* (S, UPS) & 18 Feb 1867, *A.F. Regnell, ser. III, nº 1002* (UPS); Belo Horizonte, 1970, *L. Rennó 3027* (BHCB); São João del Rei, Rodovia BR-265, Lavras-Barbacena, Km 82, 21º11'S, 44º20'W, 950 m, 5 Feb 1989, *A.O. Scariot et al. 340* (CEN);

Pedro Leopoldo, 9 Feb 1980, *H. Sick & L.A. Pereira Gonzaga s.n.* (RB); Lagoa Dourada, Estrada secundária para fazenda junto à BR-383, 20º58'S, 44º7'W, 19 Apr 2011, *M. Sobral et al. 13870* (RB); Santa Rita do Sapucai, ca. 4 km de Santa Rita en direção a São Sebastião da Bela Vista, arredores da reserva, 22°12’27.26’’S, 45°44’15.29’’, 29 Jan 2016, *V.C. Souza 40126* (ESA, RB); Aiuruoca, estrada para o Matutu, 30 Jan 1996, *J.R. Stehmann s.n.* (BHCB 31734); Ouro Preto, Estrada para Mendes, após a Fazenda da Brígida, 20º20’45.5’’S, 43º30’32.5’’W, 8 Mar 2012, *B.V. Tavares et al. 17* (OUPR); Rodrigo Silva, perto do Sítio Araguaia de indo para a Bocaina, 20º24’15.9’’S, 43º36’39.4’’W, 8 Mar 2012, *B.V. Tavares et al. 23* (OUPR); Monte Belo, Fazenda Lagoa, Agua Escondida, 19 Jun 2007, *M.C.W. Vieira 2177 & 2179* (RB); Caldas, beira do Rio Verde, cult., 21º55’S, 46º22’W, 1200 m, 26 Mar 1991, *R.F. Vieira & I. Costa 683* (CEN, RB); **Paraná**: Paranaguá, Alexandra, in cultis, 5 Sep 1910, *P. Dusén 10200* (F, GH, K, L, NY, P, S); São José da Boa Vista, Río Jaguariaiva, 19 Nov 1970, *G. Hatschbach & O. Guimarães 25528* (F, MBM); Maringá, km 10 da BR 369 entre Maringá e Campo Mourao, 23º30’S, 52º00’W, 440 m, 17 Apr 1985, *E. Lleras Perez et al. 1942* (CEN, CORD) & *1944* (CEN); Campo Mourão, 31 May 1978, *J. Marques de Lima 273* (RB); **Rio de Janeiro**: Itatiaia, a unos 3 km de la entrada principal al Parque Nacional Itatiaia, justo a la entrada del Hotel Cabañas, 27 Feb 2006, *G.E. Barboza et al. 1654* (CORD); circa Rio de Janeiro, Jun-Jul 1767, *P. Commerson 160* (P); São Christovão, 26 Sep 1876, *A. Glaziou 8841* (G, K, LE, P00410009, P00410010, R); Rio de Janeiro, *C.F. von Martius s.n.* (BR); Paraíba do Sul, Fazenda de Sobral, 26-29 Nov 1881, *J. da Saldanha da Gama 6075* (R); Sobral, prope Parahyba do Sul, Nov 1881, *C.A. Schwacke s.n.* (R); Rio de Janeiro, 1881, *C.A. Schwacke s.n.* (US 1570311); Rio de Janeiro, ad Belem cult., 1888, *C.A. Schwacke s.n.* (R 92791); **Rio Grande do Sul**: Viamão, Lamí, 3 Jan 1949, *B. Rambo s.n.* (LIL, PACA 39416); Osorio, Lagoa dos Barros, 14 Dec 1949, *B. Rambo s.n.* (CORD, LIL, PACA 44769); Lagoa da Pinguela, prope Osorio, 27 Mar 1950, *B. Rambo s.n.* (CORD, ICN, LIL, PACA 46466); prope Osorio, 1 May 1950, *B. Rambo s.n.* (CORD, LIL, PACA 46998); **Santa Catarina**: Garuva, Mina Velha, en el camino desde Joinville a Garuva, unos 10 km antes del límite con Paraná, 19 Jan 1970, *A.T. Hunziker 20229* (CORD); Itajaí, Luis Alves, Braço Joaquim, 300 m, 24 May 1956, *R.M. Klein 2063* (HBR, UC, US); Brusque, Azambuja, 30 m, 6 Dec 1961, *R. Klein 2774* (FLOR, HBR, US); Jaraguá do Sul, Corgo de Jaraguá, *J.B. Pohl 5173* (CORD, W); Ibirama, Hôrto Florestal I.N.P., 100 m, 21 Sep 1956, *P.R. Reitz & R.M. Klein 3760* (HBR, US); Azambuja, 35 m, 2 May 1952, *P.R. Reitz 4749* (HBR, US); São Bento do Sul, Rio Natal, linha férrea próxima à igreja, 23 Feb 2011, *P. Schwirkowski s.n.* (FPS); same locality, 17 Jan 2015, *P. Schwirkowski 885* (FPS); Rio Natal, linha férrea, próximo a igruja de Rio Natal, 26º20’49’’S, 49º18’14’’W, 450 m, 24 Mar 2018, *P. Schwirkowski 2713* (FURB); **São Paulo**: Campinas, Parque Jambeiro, próximo à lagoa, 19 Feb 1995, *P.R. Andrade & R.M. Chagas 1188* (IAC, SP); Mogi das Cruzes, em cultivo, 7 Mar 2005, *Anonymous* 05/17 (CORD); rumbo a Paraibuna, desde Salesópolis, por SP 077, en el km 108.5, 450 m, 25 Feb 2006, *G.E. Barboza et al. 1640* (CORD); Bananal, a unos 10 km al sur de Bananal, por ruta SP 247, rumbo a Sertão do Bocaina, 22°44’57’’S, 44°22’53’’W, 600 m, 26 Feb 2006, *G.E. Barboza et al. 1646, 1646b* & *1647* (CORD); Monteiro Lobato, 19 km al norte de Monterio Lobato, por ruta SP 50, rumbo a Campos do Jordao, 1050 m, 27 Feb 2006, *G.E. Barboza et al. 1656* (CORD); Parelheiros, 23°52’27.3’’S, 46°46’32’’W, 771 m, 1 Apr 2018, *G.E. Barboza & R. Deanna 5003* (CORD); Monterio Lobato, a 3 km de Monteiro Lobato, rumbo a Campos do Jordao, 22°54’58.4’’S, 45°48’12.6’’W, 732 m, 3 Apr 2018, *G.E. Barboza & R. Deanna 5020* (BM, CORD); desde cruce de Bananal rumbo

a la Estación Ecológica Bananal, 22°45’31.5’’S, 44°23’07.8’’W, 681m, 5 Apr 2018, *G.E. Barboza & R. Deanna 5027* (CORD); rodovia SP 66 (Rod. Dos Tropeiros), km 303, Estrada Resende-Bananal, 500 m, 22º37’S, 44º29’W, 12 Apr 1986, *L. Bianchetti et al. 435* (CEN, CORD); Barra do Turvo, Parque Estadual de Jacupiranga, Núcleo Cedro, 24º57'46"S, 48º24'59"W, 570 m, 5 Jan 1999, *L. Bianchetti et al. 1524* (CEN); Monte Mor, 7 Oct 1995, *E.G. Briske s.n.* (ESA 30840); Santos, in the forest above the Benedectine Monastery, 1 Dec 1826, *W.J. Burchell 3460* (CORD, K, P); Tremembé, Parada Sete, 26 Jan 1948, *J.P. Coelho s.n.* (SPSF); Pereira Barreto, Ilha Secca, 2 Aug 1936, *F.C. Hoehne & A. Gehrt s.n.* (SP 35755, US); Pindamonhangaba, Piracuama, Fazenda Magalhaes, 1300 m, 5 Dec 1967, *A.T. Hunziker 19563* & *19564* (BM, CORD); 19 km ao norte de Monteiro Lobato, SP 50, 970 m, 22º50’S, 45º45’W, 30 Apr 1985, *E. Lleras Pérez et al. 2047* (CEN, CORD); Itapetininga, Fazenda French, 10 Dec 1887, *A. Loefgren 438* (SP); Santa Izabel, perto do km 71 da Rodovia Santa Izabel-Igaratá, 13 Dec 1964, *J. Mattos 12156* (SP); Botucatu, Parque Ecológico da Pavuna, da sede até a lago pela trilha principal, 22º50’30’’S, 48º30’47’’W, 761 m, 22 Apr 2009, *L.B. Santos 160* (VIES); Campinas, 8 Nov 1873, *A.E. Severino s.n.* (Herb. Regnell 177/III, 1002) (CORD, UPS); Río Claro, arrededores da UNESP, 6 Feb 1985, *F.S. Torres s.n.* (CEN 30532); Jundiaí, Reserva Ecológica da Serra do Japi, 5 Mar 2005, *J. Vasconcelo Neto 05/16* (UEC); Sumaré, Sumaré, 14 Jan 1977, *J. Vasconcelo Neto 6707* (F, UEC); Monte Alegre do Sul, Estação Experimental do IAC, 21 Jan 2000, *L.C. Vernacci 2820* (F); Mogi Mirim, Fazenda Emilio Spina, 27 Feb 1941, *A.P. Viegas & A.S. Costa s.n.* (CORD, IAC 6201, SP 48628); IAC, Faz. Santa Elena, 17 Mar 2003, *E. Zuchiwschi 55* (ESA); **Tocantis**: Babaçulândia, margen esquerda do rio Corrente, chácara Paraíso, 07º14'22"S, 47º45'48"W, 140 m, 21 Apr 2008, *G. Pereira-Silva et al. 13175* (CEN).

PARAGUAY. **Amambay**: Weaver´s ranch, 13 km E of PN Cerro Cora, Arroyo Capiivary, 200-300 m, 27 Mar 1983, *J.E. Simonis et al. 216* (F, NY, MO, U); **Caazapá**: Santa Ursula, 55 Km NE de Yuty, 180 m, 23 Mar 1993, *A. Schinini et al. 27787* (CTES, LIL); **Central**: L’Assomption, à la lisière des bois, Jan 1877, *B. Balansa 2079* (P); Laguna Ypacaray bei Patiño-cué, 3 Mar 1903, *K. Fiebrig 952* (GH, GOET, M); **Guairá**: Villa Rica dans les haies, Feb 1879, *B. Balansa 2079a* (P); Villarrica, *P. Jörgensen 7679* (NY); Cordillera de Ybytyruzú, Cerro Peró, 1 km W of Destacamento Tororo, 25º55’S, 56º15’W, 17 Dec 1988, *E.M. Zardini & A. Aguayo 9508* (FCQ) & 17 Feb 1989, *E.M. Zardini 10972* (FCQ, MO); **Paraguarí**: Cerro Hû, 25 Nov 1950, *B. Sparre & F. Vervoost 552* (LIL, MO, US, S); Acahay Massif, 25º54’S, 57º09’W, 400 m, 11 Jun 1989, *E.M. Zardini et al. 12744* (PY) & 26 Feb 1992, *E.M. Zardini 30803* (MO, PY); **Presidente Hayes**: Gran Chaco, ad ripam occidentalem flum. Paraguay, Jan 1903, *É. Hassler 2891* (G, P).

Cultivated

BRAZIL. Mun. Viçosa: UFV, pastagem da Zootecnia, 21 Mar 1997, *M.G. Bovini 1142* (VIC); Viçosa, mata da Garagem, Campus da UFV, 15 Nov 1997, *M.G. Bovini 1234* (HUSU, RB, VIC); Viçosa, campus da Universidade, 3 Mar 1977, *M.P. Coons 77-181 & 77-182* (VIC); Visconde do Rio Branco, Estação Experimental de Rio Branco, da Universidade Federal de Viçosa, 24 Mar 1986, *E. Lleras Perez 2135* (CEN, CORD, VIC); Viçosa, Agricultural College lands, 680 m, 22 Dec 1929, *Y. Mexia 4148* (CAS, CORD, G, K, MO, NY, S, UC, US, Z); Viçosa, Agricultural College lands, depression behind Director’s house, 680 m, 20 Mar 1930, *Y. Mexia 4493* (BM, CAS, F, GH, K, LIL, MO, NY,

S, UC, US, Z); UFV, estrada da caixa d'agua, próximo às plantações, 8 Mar 1986, *H.C. Morais s.n.* (VIC 9582); UFV, estrada dos cavalos, 30 jan 1987, *I.C.F. Santos & E.A. Myasato s.n.* (VIC 9946); UFV, Belvedere, 4 Feb 1987, *I.C.F. Santos s.n.* (VIC 10044 & 10045); **São Paulo**: Piracicaba, Departamento Hoticultura ESALQ, 3 Nov 2007, *C.A. Abe s.n.* (ESA 119669); São Roque, Estação Experimental do IAC, 24 Apr 1995, *L.C. Bernacci et al. 1449* (HRCB, IAC); Mata da Pedreira, ESALQ, 19 Mar 1986, *E.L. Catharino 720* (ESA); Jardim Botânico de São Paulo, cult., 13 Jan 1948, *O. Handro s.n.* (CORD 00086148, SP); cult. em São Paulo, 28 May 1959, *O. Handro 860* (CORD, SP); Parque do Estadual São Paulo, *W. Hoehne s.n.* (CEN 26355, SP 15640); in cultis São Paulo, Dec 1833, *L. Riedel s.n.* (NY 04140846, US 1574115); no Horto Florestal de Loreto, Apr 1926, *A.J. de Sampaio 4314* (R); Horto do Departamento de Botânica ESALQ/USP, 12 Jan 1993, *V.C. Souza 2140* (ESA).

38. **Capsicum recurvatum Witasek**

BRAZIL. Minas Gerais: Camanducaia, Monte Verde, Serra da Mantiqueira, 22 Apr 2002, *L.D. Meireles et al. 1047* (UEC); São Gonçalo do Rio Abaixo, São Gonçalo do Rio Abaixo, 11 Jan 1989, *J.R. Stehmann s.n.* (SPF 92819); **Paraná**: Morretes, por el camino de la Graciosa, viniendo desde Quatro Barras rumbo a Morretes, 25º19’57’’S, 48º54’03’’W, 860 m, 26 Mar 2004, *G.E. Barboza et al. 915* (CORD, MO) & 24 Feb 2006, *G.E. Barboza et al. 1629* & *1632* (CORD); por el camino de la Graciosa, justo en una bajada al lado del cartel que indica Camino da Graciosa, 25º19’57’’S, 48º54’03’’W, 860 m, 14 Apr 2008, *G.E. Barboza et al. 2021* (BM, CORD, MO, NY); La Graciosa, 25º 19’56’’S, 48º53’59’’W, 851 m, 31 Mar 2018, *G.E. Barboza & R. Deanna 5000* (BM, CORD); Serra da Graciosa, 6 Nov 1982, *A.C. Cervi & F.C. Silva 2039* (MBM, NY, UPCB); Curitiba/Joinville, banks of the Rio do Santa near the bridge of Highway 101, at km 70, 600 m, 5 Jan 1974, *J. Conrad & W. Dietrich 2041* (CORD, MO); Piraquara, Mananciais da Serra, Jan 1968, *L.T. Drombrowski & Y.S. Kuniyoshi 2817* (BHCB); Ipiranga, 1 Feb 1904, *P. Dusén 3360* (CORD fragment, GH, R: 3 sheets, US); Serra do Mar, inter Roça Nova et Banhado, 30 Dec 1908, *P. Dusén 7418* (CORD, F, GH, K, L, MBM, MO, P, S); Bocaiuva do Sul, Volta Grande, 18 Feb 1914, *P. Dusén 14527* (GH, NY, S); desvío Ypiranga, 18 Dec 1914, *P. Dusén 16139* (F, GH, NY, S); Quatro Barras, Deodoro, Morro do Anhangava, 21 Nov 1943, *G. Hatschbach 52* (MBM, RB); Campina Grande do Sul, Serra da Lapinha, 4 Jul 1963, *G. Hatschbach 10180* (B, LIL, MBM, S, US); Guaraqueçaba, Río do Cedro, 50 m, 19 Oct 1967, *G. Hatschbach 17491* (CORD, MBM, Z); Colônia Floresta, 50-100 m, 23 Jan 1969, *G. Hatschbach & C. Koczicki 20077* (C, MBM, MEXU); Bocaiuva do Sul, Serra Virgem Maria, 11 Nov 1968, *G. Hatschbach 20262* (CORD fragment, CTES, G, MBM, SPF, Z); Colônia Floresta, *G. Hatschbach & C. Koczicki 20877* (MBM); Veú de Noiva, 850 m, 1 Dec 1970, *G. Hatschbach 25714* (C, CORD, G, MBM, MO); Serra Marumbi, Picada ao Olimpo, 1000 m, 19 Jan 1971, *G. Hatschbach 26000* (C, CORD, MBM, MEXU); Rio dos Padres, 1 Dec 1972, *G. Hatschbach 30892* (MBM); Paranaguá, Morro do Inglês, 150 m, 18 Feb 1976, *G. Hatschbach 38096* (ALCB, C, G, MO); Viaduto do Padre, 29 Oct 1977, *G. Hatschbach & L. Landrum 40412* (CORD, MBM, MO); Serra San Miguel, extr. para Parque Estadual das Lauraceas, 30 Oct 1990, *G. Hatschbach & D.D. Guimarães 54794 & 54797* (MBM, NY); Guaratuba, Río Cubatãozinho, 50-100 m, 16 Nov 1992, *G. Hatschbach & A.R. Campos 58235* (CORD, MBM); inmediaciones del límite con S. Paulo, en Río Pardinhas, sobre la ruta de Curitiba a S. Paulo, a unos 60 km de Cuatro Barras, 650

m, 23 Jan 1970, *A.T. Hunziker 20230* (BM, CORD); vista Engeniero Lacerda, por Morretes, 880 m, 24 Jan 1971, *A.T. Hunziker 20775* (BM, CORD, MBM, MO, NY, RB, SI, UEC, US); via Graciosa, Grota Funda, 25º20'S, 48º50'W, 13 Dec 1977, *L.R. Landrum 2881* (NY); Estrada da Graciosa (PR-410), Recanto curva da Ferradura, a 19 km de Morretes-Parque Estadual de Marumbi, 380 m, 25º20'S, 48º54'W, 28 Apr 1985, *E. Lleras Pérez et al. 2029* (CEN, CORD; = *A.T. Hunziker 25003*, CORD); por el camino de la Graciosa, viniendo desde Quatro Barras rumbo a Morretes, 860 m, 25º19'57.1''S, 48º54'02.8''W, 27 Nov 2003, *L. Mentz et al. 289* (CORD, ICN); Estrada da Graciosa, Recanto Mãe Catira, 25º22'S, 48º51'W, 27 Nov 1998, *M.F.R. Paula et al. 18* (NY, UPCB); Sítio Santa Bárbara, 14 Feb 2004, *O.S. Ribas et al. 6004* (MBM); Antonina, Usina Hidrelétrica Parigot de Souza, 800 m, 12 Jan 2006, *O.S. Ribas et al. 7150* (MBM); São José dos Pinhais, Castelhanos, 7 Feb 1998, *J.M. Silva et al. 2264* (MBM); Adrianópolis, Fazenda Mato Limpo, Berneck, 23 Nov 2004, *J.M. Silva et al. 4218* (MBM); same locality, 14 Dec 2005, *J.M. Silva & L.M. Abe 4553* (HCF, HUFU, MBM, RB, SMDB, UNOP); Adrianópolis, 12 Dec 2007, *J.M. Silva & J. Cordeiro 6187* (HUCP, MBM, RB); Guarapuava, Serra Geral, 650 m, 11 Feb 1982, *S. Sohn 98* (CORD, HRB); Graciosa, 31 Mar 2018, *J.R. Stehmann et al. 6447* (BHCB); Alto da Serra do Mar (Corvo), 48 km de Curitiba na estrada Curitiba-Paranaguá, 935 m, 8 Apr 1948, *G. Tessmann 3432* (MBM); **Rio de Janeiro**: Paratí, Estrada Cunha (SP)-Parati (RJ), a 1 km da divisa SP-RJ, 23º10'S, 44º50'W, 1450 m, 13 Apr 1986, *L. Bianchetti 461* (CEN, CORD); Estrada Paratí (RJ)-Cunha (SP), a 1,4 km do entrocamento com a BR-101, 23º 12'S, 44º48'W, 750 m, 14 Apr 1986, *L. Bianchetti et al. 473* (CEN, CORD); Paraty, estrada Paraty-Cunha, a 12,6 km do entroncamento da BR 101 com SP 171, 23º12'27''S, 44º49'22''W, 610 m, 5 Aug 1899, *L. Bianchetti et al. 1546* (CEN); Mangaritiba, Reserva Ecológica Rio das Pedras, 1 Dec 1996, *M.G. Bovini 1119* (RB: 3 sheets); Reserva Ecológica Rio das Pedras, trilha do Corisquinho, 26 Apr 1997, *J.M.A. Braga 3973* (RB); Teresópolis, along the road due northward about a mile and a half from the Fazenda do Pacaqué [Paquequer], 21 Feb 1826, *W.J. Burchell 2443* (K 000585892, K 001073030, P); Itatiaia, Parque Estadual da Pedra Selada, Fazenda Marimbondo, 22°21'41''S, 44°35'22''W, 1468 m, 5 Feb 2015, *L.J.T. Cardoso et al. 1130* (BHCB, JPB, RB); Floresta da Tijuca, 19 Dec 1868, *A. Glaziou 3074 pp* (R, K); Serra do Mar (= Bocaina), en el camino entre Cunha y Paratí, a unos 2 km del límite con el Est. Sao Paulo, a ± 1350 m, 13 Apr 1986, *A.T. Hunziker 25186* (BM, CORD; = *L. Bianchetti et al. 462*, CEN, CORD); Serra do Mar (= Bocaina), en el camino entre Cunha y Paratí, a 2,5 km del límite interestatal y a unos 19 km antes de Paratí, 1200 m, *A.T. Hunziker 25187*, 13 Apr 1986 (CORD; = *L. Bianchetti et al. 464*, CEN, CORD); Angra dos Reis, Fazenda Japuhyba, 19 Mar 1951, *M. Kuhlmann 2643* (CORD, SP); Parque Nacional da Serra dos Órgãos, trilha para o Alojamento 04 e a Pedra do Sino, ca. 22°25-32'S, 42°59'-43°07', ca. 1190-2130 m, 12 Apr 2011, *J.A. Lombardi et al. 8320* (BHCB, UNOP); Parque Nacional da Serra da Bocaina, Trilha do Telégrafo, acesso pelo Sítio Vista Alegre, 30 Jan 2019, *F.H. Nadal et al. 43* (BHCB); Parque Nacional da Serra dos Orgaos, 18 Feb 1962, *O. Solbrig & G.F. Pabst 6884* (CORD, GH, HB, US); **Santa Catarina**: Joinville, Río Manso, 26º16'48"S, 49º8'24"W, 744 m, 25 Feb 2010, *S. Dreveck et al. 1832* (BHCB, FURB); Massaranduba, Guaraní, 26º38'4"S, 48º57'36" W, 7 Mar 2011, *A. Forte 6154* (FURB); Blumenau, Sede Parque Nacional da Serra do Itajaí, 27º3'25"S, 49º5'12"W, 287 m, 21 Jan 2013, *L.A. Funez 1561* (FURB, RB); Corupá, RPPN Emilio Fiorentino Battistella, 26º23'37"S, 49º21'15" W, 660 m, 17 Jan 2015, *L.A. Funez 3325* (FURB, RB); São Bento do Sul, próximo a comunidade de Rio Natal, arredores do CEPA Rugendas (área pertencente a

Univille), 26º19'30"S, 49º18'25"W, 599 m, 10 Feb 2012, *L.L. Giacomin et al. 1709* (BHCB, RB); a unos 9 km después de Pres. Nereu rumbo a Apiuna, ca. 825 m, 27 Apr 1985, *A.T. Hunziker 24999* (CORD; = *E. Lleras Pérez et al. 2015*, CEN, RB, SP); Vidal Ramos, Sabiá, 600 m, 26 Nov 1957, *R. Klein 2232* (FLOR, HBR, L, NY, UC, US); Presidente Nereu, Estrada Madeireira a 7 km de Pres. Nereu indo a Apiuna, 27º12'S, 49º27'W, 770 m, 27 Apr 1985, *E. Lleras Pérez et al. 2009* & *2010* (CEN; = *A.T. Hunziker 24997* & *24998*, CORD); Estrada a Sabiá, a 3 km de interceção a estrada Pres. Nereu-Apiuna, 27º14'S, 49º28'W, 660 m, 27 Apr 1985, *E. Lleras Pérez et al. 2020* (CEN, ICN, RB, SP; = *A.T. Hunziker 25001*, CORD); Estrada Pres. Nereu-Apiuna, 3 km ao norte do entroncamento com a estrada a Sabiá, 27º12'S, 49º28'W, 550 m, 27 Apr 1985, *E. Lleras Pérez et al. 2023* (CEN, ICN; = *A.T. Hunziker 25002*, CORD); Biguassú, Fachinal, 500 m, 18 Jan 1945, *P.R. Reitz C 924* (CORD, HBR, LIL, RB); San Francisco do Sul, Três Barras, Garuva, 100 m, 19 Dec 1957, *P.R. Reitz & R. Klein 5766* (FLOR, HBR, L, UC, US); Sabiá, 750 m, 30 Dec 1957, *P.R. Reitz & R. Klein 5943* (FLOR, L, US); Benedito Novo, Alto Forcação, Doutor Pedrinho, 600 m, 9 Dec 1965, *P.R. Reitz & R. Klein 17345* (B, F, FLOR, P, US); São Bento do Sul, Rio Natal, próximo a CEPA, 26º19'28"S, 49º18'46"W, 14 Dec 2013, *P. Schwirkowski 139* (FPS, FURB); Serra Dona Francisca, trilha do Castelo dos Bugres, 26º13'15"S, 49º3'13"W, 1 Jan 2016, *P. Schwirkowski 1425* (FPS, FURB, RB, UEC) & *1433* (FPS, FURB, MBM); Rio Natal, linha férrea, após o CEPA Rugendas, 26º19'29"S, 49º18'48"W, 600 m, 16 Jan 2016, *P. Schwirkowski 1470* (FPS, FURB); Serra Dona Francisca, trilha do Castelo dos Bugres, 26º13'16"S, 49º3'29"W, 600 m, 30 Oct 2017, *P. Schwirkowski 2534* (FURB); Brusque, Azambuja, 35-135 m, 26 Nov 1956, *L.B. Smith 7974* (CORD, HBR, R, US); **São Paulo**: Ribeirão Grande, Parque Estadual Intervales - (manhã) trilha da caçadinha, com início na estrada do carmo, 780 m, 20 Apr 2003, *D.F. Araki et al. 105* (ESA, SPSF, UEC); Reserva Estadual das Fontes do Ipiranga, 30 Sep 1982, *M.C.B. Attié et al. 59* (ESA, RB, SP); desde Parelheiros rumbo a Eng. Marsilac, en la Estrada Ponte Seca, 23º54'01''S, 46º43'36''W, 750 m, 15 Apr 2008, *G.E. Barboza et al. 2023* (BM, CORD, MO, NY, P, SI); desde Parelheiros rumbo a Eng. Marsilac, en km 49, Estrada Ponte Seca, 23º54'00''S, 46º43'37''W, 766 m, 16 Apr 2018, *G.E. Barboza & R. Deanna 5004* (CORD); São José do Barreiro, desde São José do Barreiro hacia Parque Nacional Serra do Bocaina, 22º40'27''S, 44º36'53''W, 1079 m, 4 Apr 2018, *G.E. Barboza & R. Deanna 5026* (CORD); Sete Barras, Núcleo Saibadela (Fazenda Intervales), 24º13'31"S, 48º12'51"W, 12 Jan 1999, *E.R. Batista et al. 129* (ESA, RB, SPSF, UEC); São Miguel Arcanjo, Parque Estadual (PE) de Carlos Botelho, 10 Mar 2005, *R. Belinelo 05/18* (CORD, UEC); Mun. Tapiraí, Tapiraí, 9 Jan 1995, *L.C. Bernacci et al. 957* (CORD, IAC, SP, UEC); Cunha, Estrada Cunha (SP)-Paratí (RJ), Pedra Marcela, a 300 m da torre de Transmissao de Furnas, 23º04'S, 44º50' W, 1760 m, 13 Apr 1986, *L. Bianchetti et al. 445, 446, 456, 457* (CEN) & *459* (CEN, CORD; = *A.T. Hunziker 25183*, CORD); Cubatão, Estrada velha (caminho do Mar) Sao Paulo-Santos, a 5,5 km do complexo da Petrobras, 23º51'S, 46º27'W, 500 m, 16 Apr 1986, *L. Bianchetti et al. 490* (CEN, CORD); PE Carlos Botelho, a 4.6 km da sede do parque, 24º05'36''S, 47º55'31''W, 810 m, 20 Jul 1998, *L. Bianchetti et al. 1517* (CEN); PE Carlos Botelho, beira da estrada interna, a 6.6 km da sede, 24º4'11''S, 47º57'49''W, 830 m, 29 Apr 1999, *L. Bianchetti et al. 1520* (CEN); Barra do Turvo, Parque Estadual de Jacupiranga, Núcleo Cedro, trilha do aríete, próximo a sede, 24º57'46''S, 48º24'59''W, 570 m, 1 May 1999, *L. Bianchetti et al. 1523* (CEN); Pedro de Toledo, Estrada para Colina Verde a 18,2 km do entroncamento com a Estrada Pe. Manoel da Nóbrega, 24º21'17''S, 47º19'43''W, 380 m, 5 Feb 1999, *L. Bianchetti et al. 1526*

(CEN); Cubatão, Estrada velha do Mar Santos-São Paulo (SP 148), a 3,1 km do monumento Cruzeiro quinhentista, 23º51’23’’S, 46º26’38’’W, 260 m, 5 Mar 1999, *L. Bianchetti et al. 1527* (CEN); near Cubatão, at Rio das Pedras, [23°52’S, 46°28’W], 8 Dec 1826, *W.J. Burchell 3472* (K); Iporanga, Fazenda Intervales, trilha da Figueira, 20 May 1996, *A.M. Corrêa et al. 93* (UEC: 3 sheets, SP); Ubatuba, Parque Estadual da Serra do Mar, trilha do Corisco, ca. 2 km após a Casa da Farinha (sentido Picinguaba-Paraty), 23º19’58’’S, 44º49’57’’W, 71 m, 25 Jan 2012, *L.L. Giacomin & T.E. Almeida 1688* (BHCB, CORD, RB); Parque Estadual da Serra do Mar, Núcleo Picinguaba, trilha saindo da Base Cambucá para a sede da fazenda (Base de Pesquisa), 23º19’18’’S, 44º56’26’’W, 63 m, 26 Jan 2012, *L.L. Giacomin & T.E. Almeida 1691* (BHCB, CORD); São Paulo, Horto Botânico, caminho para chapada, 12 Dec 1901, *A. Hammar s.n* (CORD 00087815, SP 15403); Agua Funda, nativa no Jardim Botãnico, 28 Nov 1971, *O. Handro 2184* (CORD: 3 sheets, GH, HB, MO, SI, SPF, US, W); Alto da Serra, Estrada do Vergueiro, 23 Dec 1920, *F.C. Hoehne s.n.* (CORD 00087819, SP 4703, US 1616747); Parque do Estado de São Paulo, 14 Feb 1945, *W. Hoehne s.n.* (CEN, CORD 00087817, G, NY, SPF 13821, U 1736364); Parque do Estado de São Paulo, perto do Córrego do Pouso dos Urubús, 9 Mar 1960, *W. Hoehne s.n.* (CORD 00087814, NY, RB 342488, SP 15859); Pozo do Urubús, en la “mata” o selva vecina al Jardín e Instituto, 700 m, 9 Dec 1967, *A.T. Hunziker 19546* (BM, CORD, NY, SI); camino entre Cunha y el cerro Pedra Marcela, a 300 m de la Torre de transmisión de Furnas, 23º04’S, 44º50’W, 1760 m, 13 Apr 1986, *A.T. Hunziker 25184* (BM, CORD, MO, NY, RB, SI, US; = *L. Bianchetti et al. 458*, CEN, CORD); Cubatão, Estrada velha (caminho do Mar) Sao Paulo-Santos, a 5,5 km do complexo da Petrobras, 16 Apr 1986, *A.T. Hunziker 25196* (BM, CORD, NY, SI, US); Reserva Biológica Parque Estadual das Fontes do Ipiranga, 28 Nov 1980, *S.L. Jung & A. Barros 374* (RB, SP); Núcleo Picinguaba, mata de encosta, 23 May 1989, *M. Kirizawa & J.A. Correa 2164* (SP); margem do Rio Juquiá, 25 Jun 1941, *M. Kuhlmann s.n.* (CORD 00087818, 00088592 & 00088593, SP 45749); nativa no Jardim Botãnico, 14 Mar 1944, *M. Kuhlmann s.n.* (CORD 00087816, SP 52077); Jardim Botãnico SP, 28 Mar 1955, *M. Kuhlmann 272* (CORD, SP); Cantareira, Horto Florestal, 20 Dec 1951, *M. Kuhlmann 2809* (CORD, RB, SP); Parque Estadual das Fontes do Ipiranga, nativa no Jardim Botânico, 28 Mar 1955, *M. Kuhlmann 3556* (SP); Eldorado, Parque Estadual de Jacupiranga - Núcleo Caverna do Diabo, 24º38’47.9’’S, 48º23’31.5’’W, 9 Feb 1995, *H.F. Leitão Filho et al. 33146* (ESA, HRCB, SP, UEC); Açude da trilha Tucano-Parque Estadual de Carlos Botelho, 24°4’S, 47°44’W, 17 Nov 2002, *R.A.F. de Lima 85* (ESA, RB); Parque Estadual de Carlos Botelho, 400 m, 13 Jun 2004, *R.A.F.Lima 420* (ESA); Parque Estadual de C. Botelho, 19 Jan 1995, *P.L. de Moraes et al. 1164* (CEN, ESA, RB); Santos-Sororocaba, 1 Feb 1875, *H. Mosén 3443* (CORD, P, S); Pindamonhangaba, Fazenda São Sebastião do Riberão Grande, 22 Feb 1996, *S.A. Nicolau 1050* (SP); Parque Natural Municipal da Fazenda do Carmo, 17 Jan 2012, *F.G. Pereira et al. 406* (HSTM, PMSP); about 42 km from São Paulo, on road to Santos, Apr 1972, *B. Pickersgill 462* (CORD); Miracatu, FazendaItereí, BR 116, km 350, 24º03’S, 47º13’W, 500 m, 19 Apr 1994, *J.R. Pirani & R. García 3105* (BHCB, SP, SPF); Reserva Carlos Botelho, entre S. Miguel Arcanjo e Sete Barras, 18 Jan 1978, *G.T. Prance et al. 25919* (UEC); Riberão Grande-Fazenda São Sebastião do Riberão Grande, 900 m, 31 Mar 1994, *L. Rossi et al. 1474* (BHCB, ESA, UEC, SP); Mairiporã, Serra da Cantareira, região do Pinheirinho, 8 Dec 1988, *E.L. Silva 40* (CEN, CORD, UEC, RB, SP); nativa no Jardim Botânico e Parque do Estado, 9 Apr 1974, *J.S. Silva 272* (CORD, RB, SP); 10 km de Barra do Turvo em direção a Pariquera-Açú, 14 Feb 1995, *J.P. Souza et al. 60* (ESA, SP); Área

da Cia. Votorantim, próximo ao início da Trilha do Rio Dezembro, 1 May 2013, *V.C. Souza & T.B. Flores 35055* (BHCB, ESA, RB, UB); Distrito Parelheiros, desde Parelheiros rumo a Marsilac, no km 49, Estrada da Ponte Seca, 1 Apr 2018, *J.R. Stehmann et al. 6451* (BHCB); Jundiaí, Serra do Japí, 14 Nov 1988, *J. Vasconcelos Neto et al. 20857* (K, UEC) & 23 Jan 1989, *J. Vasconcelos Neto et al. 20989* (CORD); Reserva Particular da Votorantim (CBA), trilha dezembro entre a UHE Alecrom e UHE Porto raso, -24.077444, -47.449694, 403 m, 27 Mar 2013, *C.B. Virillo et al. 63* (RB).

39. Capsicum regale Barboza & Bohs
COLOMBIA. Caquetá: Florencia, Corregimiento El Caraño, Km 20, finca Las Brisas, propiedad de Isauro Trujillo, 01°44'11.80"N, 75°40'37.8"W, 1002 m, 7 Oct 2017, *D. Hoyos et al. 118* (COAH, COL); San José del Fragua, vereda La Peneya-camino hacia El Jardín, zona amortiguadora PNN Alto Fragua Indi Wasi, 01°17'31"N, 76°08'0.64"W, 700–850 m, 23 Oct 2017, *D. Hoyos et al. 127* (COAH, COL); Corregimiento El Caraño, Km 20, finca Las Brisas, 9 Dec 2017, *D. Hoyos et al. 146* (COL); Finca de don Isauro, camino al río, en interior de bosque fuertemente inclinado, 01°44'01.4"N, 75°40'35.4"W, 1000 m, 16 Apr 2016, *A. Orejuela et al. 2640* (COL); same locality, 01°44'09.1"N, 75°40'40.3"W, 932 m, 22 Aug 2019, *A. Orejuela et al. 3035* (COL); finca Las Brisas, debajo de la casa, vereda La Cascada, 01°37'5"N, 75°40'50"W, 1000 m, 7 Nov 2015, *D. Sanín 6236* (COL).

ECUADOR. Morona-Santiago: along new road Mendez-Morona, km 30–35, 800 m, 18 Aug 1989, *H. van der Werff & E. Gudiño 11196* (BM, MO, QCNE). **Napo**: Archidona Cantón, Reserva Ecológica Antisana, Comunidad Shamato, entrada por km 21-Shamato, 00°44’S, 77°48’W, 1700 m, 27 Apr 1998, *J.L. Clark et al. 5337* (BM, MO); Parroquia Ahuano, Estación Biológica Jatun Sacha, 8 km E of Misahuallí, Finca Acaro, 01°17'17"S, 77°52'54"W, 910 m, 17 Aug 2005, *J.L. Clark et al. 9403* (BM, US). **Sucumbíos**: Río Bermejo to Cerro Sur Pax, Cofan community of Alto Bermejo, NW between Lumbaqui and Cascales, vicinity of Oso Ridge Camp, 00°19'17.7"N, 77°25'10"W, 1700–1920 m, 2 Aug 2001, *R. Aguinda et al. 1537* (F).

PERU. Loreto: Datem del Marañón, Morona District, Pongo Chinim, valley between the eastern and western ridges of the Kampankis range, ca.14 km south of the Peru-Ecuador border, 3 Aug 2011, *I. Huamantupa 15251* (V0387079F color photo, F).

40. **Capsicum rhomboideum (Dunal) Kuntze**
COLOMBIA. Plantae Expeditionis Botanicae Mutisii, Vice-Regmi Novae-Granatae, 1783-1808, *J.C. Mutis 3590* (COL, US); Nouvelle-Grenade, *J.J. Triana 346* (P);

Antioquia: Turbo, Guapa, 29 May 1876, *E.F. André K 701* (F, GH, K, NY); Medellín, 1500 m, Jun 1930, *W.A. Archer 64* (COL, US); Santa Bárbara, La Planta, 800 m, 7 Sep 1930, *W.A. Archer 605* (NY, US); Medellín, 5 Dec 1930, *W.A. Archer 761* (MEDEL, US); Rionegro, alrededores de Antioquia, 550 m, 25 Oct 1947, *F.A. Barkley W. et al. 17C403* (COL, CORD, MEDEL, UC, US) & 8 Feb 1948, *F.A. Barkley* & *L.L. Arboleda R. 14* (MEDEL, US); Santa Fe de Antioquía, Jul 1962, *S. Espinal T. 521* (COL, MEDEL); San Jerónimo, Río Cauca Valley, near San Jerónimo, 730 m, 7 Oct 1977, *A.H. Gentry & H.A. León 20187* (COL, MO); dos kilómetros al norte de Antioquia, 550 m, 9 Oct 1947, *G. Gutierrez V. & F.A. Barkley 17C080* (MEDEL); Mun. Sopetrán, 500-600 m, 7 Sep 1930, *E.P. Killip 605* (MEDEL); Olaya, Remolino, carretera entre Ulaya y Liborina, 6º41'N, 75º49'W, ca. 800 m, 20 Apr 2002, *J.C. Marrugo G. et al. 709* (COL, MEDEL); ½ km NW of Antioquía on road to Turbo, 700 m, 4 Jul 1971, *M. Nee & S. Mori 4270 & 4271* (COL, US); Finca Cotové, 6º34'N 75º50' W, 500-550 m, 26 May 2001, *J.A. Pérez Z. et al. 1661* (COL, MEDEL); 1 km antes del puente de Occidente, 700 m, 26 Mar 1949, *R. Scolnik et al. 19An322* (CORD, MEDEL); Toledo, Corregimiento de El Valle, Carretera entre San Andres de Querquía y el corregimiento de Toledo, 07º01'36"N, 75º39'32"W, 520 m, 9 Feb 2009, *J.M. Vélez-Puerta et al. 3164* (MEDEL); Vereda el Tunal, Finca El Rodeo, 500 m, 30 Dec 2011, *J.M. Vélez-Puerta et al. 4181* (MEDEL); along road Santa Fé de Antioquia-Anzá, ca 1 km W of crossing of Río Tonuzco at its confluence with Río Cauca, near Finca Cotové, 615 m, 19 Sep 1987, *J.L. Zarucchi et al. 5476* (COL, HUA, MO, NY) & *5491* (COL, MEXU, MO); **Caldas**: Caldas, 3000 m, *M.T. Dawe 774* (K, NY, US); **Cauca**: Mercaderes, Nojarras, Granja U. de Nariño, 700 m, 20 Oct 1988, *O. de Benavides 10603* (PSO); Toribio, Hoya del Río Palo, La Tolda, 2000 m, 18 Dec 1944, *J. Cuatrecasas 19436* (F, GH); Mercaderes, 1110 m, 20 Oct 1946, *O. Haught 5148* (COL, US); Inzá, Río Negro bei Inzá, 1000-1500 m, *F.C. Lehmann 4745* (F, K); Patía, Galíndez, 500 m, Jun 1949, *S. Yepes-Agredo 1172* (COL); **Cundinamarca**: Tocaima, Viota, 550 m, 19 Feb 1876, *E.F. André 1780* (K, NY); El Colegio, Mesitas del Colegio, junto a carretera en el puente de la quebrada Santa Marta, 1800 m, 12 Sep 2006, *G. Beltrán et al. 24* (COL); San Antonio de Tequendama, Vereda Caicedo, Finca Bosque Dormido, 1300 m, 11 Nov 1999, *C. Bernal & A. Pico 350* (COL, PSO); Cachipay, Corregimiento Peña Negra, 1450 m, 14 May 2000, *C. Bernal 642* (COL); Fusagasugá, carretera hacia Arbeláez, 1600 m, 14 Mar 1997, *J. Calle A. et al. 70* (COL); Tena, alrededores Puente San Antonio de Tena, 1500 m, 10 Mar 1940, *J. Cuatrecasas 8254* (COL); La Mesa, San Javier, 1000 m, 20 May 1946, *J.M. Duque Jaramillo 20* (COL); Nariño, Poblado de Nariño, zona del Río Apauta, 340 m, 16 Feb 1986, *J.L. Fernández Alonso & R. Jaramillo 5401* (COL); desvío de Tena a Cachipay, en La Esperanza, 25 Feb 1999, *J.L. Fernández-Alonso & G. López 18335* (COL); Laguna Verde, al S de La Mesa, 700-750 m, 15 Apr 1952, *A. Fernández Pérez & L.E. Mora 1362* (COL); La Esperanza, 1 Apr 1935, *H. García Barriga 3119* (COL, US) & 10 Apr 1935, *H. García Barriga 4708* (COL, US); Guadas, alrededores y orillas del Río San Francisco, 1000-1040 m, 5 Nov 1945, *H. García Barriga 11750* (COL, CORD, US); camino de herradura de La Mesa a San Javier, 950-1320 m, 2-20 Jan 1947, *H. García Barriga 12164* (COL, CORD, MEDEL, US); La Palma, 1100 m, a orillas del Río Negro, 8 Oct 1970, *H. García Barriga 20144* (COL, US); km 52, Carretera La Mesa-Anapoima, 27 Apr 1981, *M. Gil & R.D. López 21* (COL); Pandi, Vereda El Guarumo, 550 M, 4 May 1998, *E. Gordillo & L. Forero 41* (COL); Tena, May 1844, *J. Goudot 4* (P); Granja cafetera "E. Soto", 1280 m, 22 Oct 1943, *G. Gutierrez V. 487* (COL, CORD, GH, MEDEL); Apulo, along trail to Anapoima, hillside east of Apulo, 460-600 m, 4 May 1944, *E.P. Killip et al. 38142 &*

38146 (COL, P, US); Pacho, Finca La Cristalina, 1890 m, 11 Mar 1978, *G. Nates P. 225* (COL, MEXU); Fusagasuga to Pandi, 1000-1300 m, 30 Nov 1917, *F.W. Pennell 2715* (GH, NY, US); Tocaima, Nov 1932, *E. Pérez Arbeláez 2085* (COL, US); Villeta, Quebrada Cune, bosque protector del acueducto de Villeta, 950 m, 14 Nov 1997, *J. Premauer & M.I. Moreno 57* (COL); alrededores del Salto de Tequendama, 14 Dec 1950, *R. Romero Castañeda 2250* (MEDEL); Girardot, 350-400 m, 19 Aug 1917, *H.H. Rusby & F.W. Pennell 111* (F, GH, K, MO, NY, US); La Mesa, Apr 1948, *C. Sandeman s.n.* (K); Tocaima, entre Viotá y Girardot, 320-560 m, Aug 1964, *C. Saravia Toledo 4641* (COL); El Colegio, 1250 m, 5 Jan 1950, *M. Schneider 912* (COL, S); Villeta, 5 Feb 1973, *W. Schwabe s.n.* (B); Santandercito (S. Claver), 1600 m, 7 Mar 1949, *H.S.J. Silva & M. Hernández 343* (COL); Villeta, casco urbano por la vía al Salto, bosque conservado del Acueducto, 985 m, 21 Nov 1997, *A. Suárez M. & J. Jácome 32* (COL); Tena, 1200 m, 1851-1857, *J.J. Triana 2284* (BM, P); Santandercito, 1600 m, 14 Nov 1946, *L. Uribe U. 1387* (COL); San Antonio de Tena, 1700 m, 20 Apr 1964, *L. Uribe U. 4762* (COL); Pandi, Vereda El Yarumo, puente sobre el Río Negro en el cruce con el Río Sumapaz, 900 m, 23 Nov 1998, *S. Vejarano & G. Galeano 28* (COL); **La Guajira**: Albania, Reserva Forestal Protectora Montes de Oca, Cuchilla de Montebello, 500 m, Feb 2009, *A. Ávila et al. 817* (UDBC); Uribia, Corregimiento Nazareth, Parque Nacional Natural Serrenía de Makuira, cerro Keichiwou, 12º09'28.6" N, 71º20'15.3"W, 300-615 m, 3 Jan 2005, *J. Bentancur 11315* (COL); **Huila**: Altamira, desde Florencia por el camino viejo hacia Guadalupe, 02º00’38’’N, 75º45’09’’W, 913 m, 21 Aug 2019, *G.E. Barboza et al. 5050* (CORD); Rivera, Vereda del Pedregal, 21-26 Feb 1947, *L.A. Bermúdez 34950* (F, UC, MO); Baraya, along road from Neiva to Baraya, 3 km SW of Baraya, 46 km NE of Neiva, 745 m, 21 Mar 1983, *T.B. Croat 55349* (MO); El Agrado, Quebrada La Yaguilga, ca. 700 m, Sep 1986, *J.L. Fernández Alonso & G. Morales 6777* (COL); Altamira, 6.4 km SE of Altamira near Guadalupe, ca. 880 m, 9 Jan 1974, *A.H. Gentry et al. 8998* (COL, MO, NY, SI); La Plata, 1000-1400 m, *F.C. Lehmann K 233* (K); Villavieja, Quebrada Las Lajas, 10 May 1985, *F. Llanos 421* (COL); La Plata, Hacienda La Limonada, 1020, 8 Oct 1984, *G. Lozano et al. 4820* (COL); Neiva/Campoalegre, 500-675 m, 19 Mar 1940, *E. Pérez Arbelaez & J. Cuatrecasas 8297* (COL, CORD, F, US); margen nor-oriental del Río Magdalena, Vereda La Escalera, 600 m, 23 Nov 1996, *O. Rivera Díaz 102* (COL, MEDEL); Cabrera Lajas, ca. 11 km E of Villavieja on road to Baraya, 460 m, 4 Jul 1950, *S.G. Smith 1133* (COL, UC, US); **Magdalena:** Santa Marta, Carretera Troncal de Caribe, Cuesta Rodríguez, camino al cerro Las Bóvedas, 250 m, 2 May 2001, *E. Carbonó 4128* (COL); Supra Honda, Río Seco, Dec 1852, *I.F. Holston 569* (K); **Nariño**: Tangua, El Pedregal, 1850 m, 10 Sep 1981, *O. de Benavides 2974* (PSO); Sandoná, a 5 km de la población, vía a Pasto, 1100 m, 2 Oct 1981, *O. de Benavides 3169* (PSO); Pasto, carretera Panamericana Punete Juanambú, 1000 m, 22 Nov 1983, *O. de Benavides 3636* (PSO); Imués, El Pedregal, 2000 m, 2 Mar 1985, *O. de Benavides 5088* (PSO); Albán, cercanías de San José de Albán, 1800 m, 27 Mar 1985, *O. de Benavides 5308* (PSO); Taminango, Km 77 Panamericana Pasto-Popayán, 1300 m, 10 Apr 1985, *O. de Benavides 5406* (NY, PSO); Buesaco, Juanambú, 1400 m, 23 Feb 1986, *O. de Benavides & C. Paredes 6238* (PSO); Corregimiento del Pedregal, Pilcuán, 1800 m, 16 Apr 1986, *O. de Benavides 6463* (NY, PSO); Ancuay-Sandoná, Puente del Guaitara, 1150 m, 25 Oct 1986, *O. de Benavides 7157* (PSO); a 1 km de Buesaco, 2000 m, 21 Nov 1986, *O. de Benavidez 7332* (PSO); Tangua, 2200 m, 6 Mar 1963, *S. Espinal T. 1059* (COL, MEDEL, PSO); Buesaco, alrededores de la población, 2000 m, 27 Jun 1995, *O.D. Guerrero 408* (PSO); Provincia de los Pastos, *F.W.H.A. von Humboldt s.n.* (GH); Consacá,

Secc. Cariaco-Carretera que conduce a la ciudad de Pasto, 1900 m, 5 Aug 1972, *G. López Jurado & J. Riascos 511* (PSO); Túqueres/Guachavés, Carretera Túqueres a Samaniego, 1800-3200 m, Jan 1952, *L.E. Mora 332* (COL, PSO); Pasto, km 5, via a Nariño, 2600 m, 15 May 1985, *B.R. Ramírez P. 295* (PSO); 2 km antes de Buesaco, 2000 m, 4 May 1989, *B.R. Ramírez P. 1399* (PSO); Puente del Juanambú Panamericana Pasto-Popayán, 1000 m, 1 Dec 1989, *B.R. Ramírez P. 1905* (PSO); km 68 Panamericana Pasto-Popayán, 1300 m, 24 Mar 1990, *B.R. Ramírez P. 2174* (PSO); Veredas Bellavista-El Cebadero, 1800-2000 m, 19 Nov 1993, *B.R. Ramírez P. 5891* (PSO); El Rosario, orillas del Río Patía, frente al Remolino, 1º40'N, 77º20'W, 680 m, 18 May 1995, *B.R. Ramírez P. 7476* (PSO); Pasto-Túquerres, carretera Pasto-Túquerres, 1820-2600 m, 30 Nov 1962, *C. Saravia Toledo & R. Jaramillo M. 1806* (COL); Ancuya, region of Pedregal, south of Yacuanquer, between Pasto and Tuquerres, 2 Jun 1946, *R.E. Schultes & M. Villareal 7863* (COL, US); near Tangua, just N of the bridge of main rd over Río Guáitara, ca. 1800 m, *M. Weigend & B.R. Ramírez 3528* (PSO, M);

Norte de Santander: Toledo, La Cabuya, región El Sarare, 1300 m, 14 Oct 1941, *J. Cuatrecasas et al. 12209* (COL, CORD, F, GH, US); Chinácota, La Nueva Donjuana, 3 Jun 1944, *M. de Garganta F. 857* (F); between Chinácota and La Esmeralda, 1000-1300 m, 19 Mar 1927, *E.P. Killip & A.C. Smith 20853* (GH, US); **Santander**: Sur de Bucaramanga, 500 m, 26 Aug 1948, *J. Araque Molina & F.A. Barkley W. 308* (COL, CORD, MEDEL, US); Los Santos, Vereda El Pozo, 6º44.466N, 73º7.836'W, 1310 m, Sep 2004, *J. Bentancur 11063* (COL); Puerto Santander, La Esmeralda, Apr 1935, *E. Pérez Arbeláez 3119* (COL); escarpas de la meseta de Bucaramanga, Quebrada Chapinero, 2 Apr 1968, *J. Rivera C. L-166* (COL, MEDEL); **Tolima**: Espinal, between Espinal and Girardot, 350-500 m, 26 Mar 1939, *A.H.G. Alston 7688* (COL, S, US); Venadillo, Hacienda El Condado, 300 m, 14 Aug 1980, *A.M. Cleef et al. 11091* (COL); Saldaña, 1844-1845, *J. Goudot s.n.* (K, P00410196); Piedras, Guataguicito, 300 m, 2 Nov 1938, *O. Haught 2413* (COL, US); Chicoral, 450 m, 18 Feb 1949, *O. Haught 6307* (COL, NY, US); Ibagué, 800 m, 2 Aug 1952, *M.E. Køie 5149* (US); Espinal to Cuamo, 400 m, 21 Jul 1917, *F.W. Pennell & H.H. Rusby 187* (NY); Cajamarca, Carretera Ibagué-Armenia, antes de Cajamarca, 4º26’N, 75º23’W, 1600 m, 30 Jul 1991, *F.J. Roldán et al. 1626* (COL, HUA, MO, NY, US); Flandes, 330 m, 14 Dec 1946, *M. Schneider 216* (COL); San Sebastián de Mariquita, 535 m, 27 May 1961, *W. Schwabe s.n.* (COL 000032798); **Valle del Cauca**: Dagua, Naranjo, vic. Dagua, 1 Apr 1876, *E.F. André 2520* (GH, K, NY); Yumbo, La Calera, Mulaló, 1050 m, 22 May 1979, *H. Cuadros V. 774* (COL, MO); Bolívar, Cordillera Occidental, Hoya del Río Sanquinini, 1200 m, 8 Dec 1943, *J. Cuatrecasas 15354* (CORD, F); Dagua, Lobo Guerrero, 610-650 m, 9-10 Sep 1944, *J. Cuatrecasas 17782 a* (CORD, F, US); Sevilla, Las Alegrías, Hoya del Río Bugalagrande, 2300 m, 27 Mar 1946, *J. Cuatrecasas 20456* (CORD, US); Vijes, Plana del Valle, Portachuelo de Vijes, 1060-1080 m, 22 Feb 1947, *J. Cuatrecasas 23690* (CORD, F, US); entre Jumbo y Vijes (Cord. Occ.), 1000-1500 m, 16 Feb 1969, *J. Cuatrecasas et al. 27335* (COL, F, US); El Cerrito, Hacienda El Trejo, entre El Cerrito y Palmira, 1050 M, 18 Dec 1938-5 Jan 1939, *H. García Barriga 6460-6462* (COL); abajo de Dagua, 9,1 kms de la unión con la carretera privada del Oleoducto del Pacifico, cruzando el Río Dagua, 19 Nov 1963, *P.C. Hutchinson et al. 3078* (COL); above El Queremal, 3º30'N, 76º45'W, 1450-1500 m, 6 Feb 1984, *A. Juncosa 2018* (MO); below Dagua, 6/7 Sep 1922, *E.P. Killip et al. 11069* (GH, US); Caldas, Papagayeros, 900 m, 4 Sep 1899, *F. Langlassé 16* (G, GH, K, P); Dagua

Thal, Jun-Jul 1881, *F.C. Lehmann 234* (K); Boquerón del Dagua, Andes of Cali, 500 m, *F.C. Lehmann 4732* (GH, US); Guadalajara de Buga, Corregimiento El Vínculo, Estación Biológica, 1000 m, 25 Feb 1986, *G. Parra V. 26* (MO); Tuluá, 1000 m, 10 Mar 1986, *G. Parra V. 089* (COL, CORD, MO); Restrepo, 1000 m, 9 Jun 1985, *J.E. Ramos 418* (MO); Corregimento de Mulaló, near La Calera, 1000 m, 3 Nov 1985, *J.E. Ramos 476* (MO, QCA); entre Dagua y Loboguerrero, 1050 m, 10-12 Nov 1962, *C. Saravia Toledo1421* (COL); Loboguerrero (Valle del Río Dagua, carretera nueva Cali-Buenaventura), 680-720 m, 21 Apr 1983, *P.A. Silverstone-Sopkin 1451* (MO, NY); Valle del Río Dagua, al oeste de los Andes, carretera nueva Cali-Buenaventura, entre km 59 (desde Cali) y Río Bitaco, ca. 720 m, 23 Jun 1986, *P.A. Silverstone-Sopkin et al.* 2322 (CTES, MEDEL); Hacienda San Gerardo, muy cerca a frontera con Municipio Guacarí, cerca de pueblo de Guabas, 950 m, 10 Mar 1988, *P.A. Silverstone-Sopkin 3689* (MO); Hacienda El Milagro, a 4 km, 1000 m, 27 May 1991, *P.A. Silverstone-Sopkin 6108* (COL, CTES).

COSTA RICA. Guanacaste: Abangares, Cebadilla, 10º30'N, 85º5'W, 1400 m, 21 Jul 1985, *W.A. Haber & E. Bello C. 2260* (MO); **Puntarenas**: Cordillera de Tilaran, San Luis, El Callado, Monteverde, 800 m, 6 Sep 1993, *Z. Fuentes & E. Fuentes 480* (CR, MO); Cordillera de Tilarán, 10º18'N, 84º48'W, 900-1200 m, 20 Oct 1990, *W.A. Haber & W. Zuchowski 10105* (CR, MO).

ECUADOR. Without locality, 8000-9000 ft, *R. Pearce s.n.* (K); in Andibus Ecuadorensibus, 1857–1864, *R. Spruce 5050* (C, E, MO); **Azuay**: just N of Oña in valley of Rio Cachihuaycu/Rio León, 3º25'57"S, 79º09'20"W, 2000 m, 6 Apr 2005, *L. Bohs 3446* (NY, QCNE); road Pasaje-Santa Isabel-Girón, valley of Río Jubones, 600-1600 m, 7 May 1974, *G. Harling & L. Andersson 14414* (GB, MO, NY); road Cuenca-Loja, Valle del Río León, ca. 1800 m, 6 Feb 1977, *G. Harling et al. 15092* (GOET, MO, NY); Km 89 S of Cuenca on Pan American Highway, 3º25'S, 79º10'W, 1950 m, 4 May 1973, *L. Holm-Nielsen et al. 4905* (AAU, F, GB, MO, S, U); 5 km WSW Santa Isabel on Cuenca-Machala road, 1500 m, 16 Feb 1991, *M. Kessler 2496* (QCA); Río León Valley on road Cuenca-Loja Dessert, 3º25'S, 79º09'W, 1800-2200 m, 11 Mar 1990, *J.E. Madsen 87010* (AAU, LOJA, QCA, QCNE); **Bolívar/Guayas**: between Chimbo River and Babapampa, 2500 m, Dec 1933, *A. Rimbach 170* (F, NY); **Carchi**: Montúfar, Parroquia La Paz, Río Chamiza, 00°31'N, 77°52'W, 2690 m, 16 Jul 1990, *C.E. Cerón 11092* (MO, QCNE); between Ibarra-Tulcan, 00º35'N, 77º50'W, 2400 m, 27 Jul 1989, *H. van der Werff & E. Gudiño 10652* (MEXU, MO); Montúfar, sector la gruta de la Paz, 2480 m, 22 May 2003, *K.A. Williams et al. 210* (QCNE); **Chimborazo**: Penipe, Parroquia El Altar, 2425 m, 18 Jul 1991, *C.E. Cerón 15714* (MO, QCNE); entre Guasuntus-Alausi, 2°13'S, 78°49'W, 3500 m, 1 Jan 2008, *J. Jaramillo 26136* (QCA); camino de Quito a San Antonio, 2800-2500 m, 3 Feb 1949, *R. Scolnik 1594* (CORD, LIL); **El Oro:** Zaruma, 1150 m, 19 Mar 1955, *E. Asplund 15822* (S); **Guayas**: 10 km N of Guayaquil, 90 m, 20 Jan 1962, *C.H. Dodson & L.B. Thien 2144* (MO); **Imbabura**: El Olivo, Ibarra, 2200 m, 5 Jun 1949, *M. Acosta Solís 12925* (F); Shanshipamba, 2700 m, 12 Nov 1949, *M. Acosta Solís 14234* (F); Pimán, al "Partidero" a Chota, 1700-2000 m, 29 Dec 1949, *M. Acosta Solís 14862* (F); Ibarra, 31 Dec 1930, *M.R. Benoist 3580* (P); Ibarra, Parroquia Ambuqui, 2200 m, 15 May 1992, *C.E. Cerón 18856* (MO, NY, QCNE); Ybarra, weedy open lot, 2200 m, 22 Aug 1982, *W. D'Arcy 14822* & *14823* (MO); Ibarra, 0-2 km along the rd. towards Apacocha, 0º21'N, 78º7'W, 22 Nov 1981, *P. Filskov et al. 37318* (AAU, QCA); dry valley of Río Mira SE of Lita, 600 m, 13 May 1990, *A.H. Gentry et al.*

70189 (MO, QCNE); Ibarra, hills east of town, 2200 m, 21 Feb 1959, *G. Harling 4272* (S); Ibarra, 2225 m, 31 Jan 1962, *C.B. Heiser 4828* (NY); between Salinas and Carchi, 0º33'N, 78º8'W, 1400-1600 m, 5 Dec 1980, *L.B. Holm-Nielsen & J.L. Jaramillo 28836* (AAU, COL, MO, QCA) *& 28876* (AAU, COL, MO, QCA, U); N of Salinas, 0º37'N, 78º8'W, 1500-1550 m, 21 Feb 1984, *A. Juncosa 2228 & 2236* (QCA, MO); Hacienda Perafán, ca. 4 km NW of village of Cotocachi, foothills of Volcán Cotocachi, 0º21'N, 78º10'W, ca. 2500 m, 17 Jan 1984, *S. Knapp 6164* (QCA, QCNE, US); La Toma, vía a Cariamanga, entrada al predio Santa Rita, 1600 m, 20 Feb 1998, *P. Lozano et al. 942* (LOJA); San Luis de Imbaya, Rio Chorlavi, 4 km NW of Ibarra, 1960 m, 29 Sep 1988, *E.B.Madsen 84312* (AAU); Urcuquí, Carretera Salinas –Lita, frente a Tulquizán, 1500 m, 15 Jan 1991, *W.A. Palacios 6837* (CHEP, MO, QCNE); vicinity Lake Yaguarcocha, 2300 m, 5 Jun 1939, *C.W. Penland & R.H. Summers 841* (F, GH, US); Cabuyal, 4 km de la parroquia Pablo Arenas, 1964 m, 18 Nov 2009, *O. Sánchez & C. Aguirre 358* & *373* (LOJA); km 2 vía Urcuquí, 2200 m, 18 Jun 1995, *V. Tafur 237* (F, MO); carretera Ibarra-Mariano Acosta-Pimampiro-El Juncal, Sector El Arenal, 2500-2700 m, 11 Mar 1986, *V. Zak 1423* (MO, NY, QCA); **Imbabura/Pichincha**: Otavalo to Malchingui, 2400-3000 m, 21 Aug 1923, *A.S. Hitchcock 20841* (GH, NY, US); **Loja**: Malacatus, 1800 m, 19 Dec 1946, *R. Espinosa 1120* (CORD); Cerro Villonaco, 1800-2100 m, 9 Apr 1974, *G.W. Harling & L. Andersson 13320* & *13334* (MO); road Catacocha-Macará, ca. 12 km SW of Catacocha, ca. 1400 m, 8 Feb 1977, *G. Harling & L. Andersson 15209* (GB, GOET); road Macará-Cariamanga, 10-15km E of Macará, 11 Feb 1977, *G.W. Harling et al. 15351* (MO); Cerro Villonaco, 2000 m, 7 Feb 1982, G. *Harling et al. 20329* (GB); between Catamayo (La Toma) and Gonzanamá, 1800 m, 12 Feb 1982, *G. Harling et al. 20630* (GB); between Chuquiribamba and Taquil, 2200 m, 14 Nov 1982, *G. Harling et al. 20725* (GB); 3 km from Malacatos on road to Vilcabamba, 1850 m, 3 Feb 1985, *G. Harling & L. Andersson 21478* (GB, QCA); 4-6 km N of Yanganá on road to Vilcabamba, 1800-1900 m, 5 Feb 1985, *G. Harling & L. Andersson 21665* (GB, QCA); road Cariamanga-Yambaca-El Toldo-Chaco, km 10-20, 04°17'S, 79°31'W, 1900-2100 m, 17 Feb 1993, *G. Harling & B. Ståhl 26470* (MO, QCA); east of San Pedro de Vilcabamba, track to Hda. El Bosque, 2020 m, 30 Oct 1984, *P.M. Jørgensen 56290* (QCA); 3 km on dirt track off road Catamayo-Loja, km 7, 1900 m, 31 Jan 1997, *B.B. Klitgaard & G.P. Lewis 386* (LOJA, QCNE); Catamayo Valley, near La Toma, 4º0'0''S, 79º4'30''W, 1300 m, 14 Feb 1965, *D.H. Knight 446* & *447* (WIS); 2 km N of Loja, 3°11'S, 79º2'70''W, 2100 m, 8 Jul 1965, *D.H. Knight 764* (MO, WIS); Catamayo, km 7, track to the left, km 3, 1950 m, 11 Dec 1997, *G.P. Lewis & B.B. Klitgaard 3730* (LOJA, MO, QCNE); Loja, km 3 from La Toma, 3º59'S, 79º20'W, 1400 m, 18 Feb 1984, *J.E. Madsen 50214* (AAU, MO, QCA, QCNE); La Toma-Cariamanga, about km 5, 4º01'S, 79º22'W, 1500 m, 21 Jan 1989, *J.E. Madsen 85610* (AAU, LOJA, QCA, QCNE); El Chaupe –Vilcabamba, 1720 m, *F.A. Vivar C. s.n.* (MO-1263583); **Pichincha**: Otón, 2500-2600 m, 29 Nov 1945, *M. Acosta Solís 11252* (F); Valle de Guayllabamba, 2400 m, *M. Acosta Solís 11295* (F); Cauce del Guayllabamba, 1700-2000 m, 28 Dec 1949, *M. Acosta Solís 14819* (F); Puéllaro, 2100 m, 16 Apr 1950, *M. Acosta Solís 16452* (F); Tumbaco, 29 May 1939, *E. Asplund 6548* (S, US); Pomasqui, 2500 m, 3 Apr 1939, *E. Asplund 6701* (NY, S, US); Pifo, between the village and Los Corrales, 2800 m, 17 Apr 1939, *E. Asplund 6992* (CAS, G, S, US); San Antonio, 2400 m, 5 Nov 1955, *E. Asplund 18272* (S); Carretera antigua Guápulo-Cumbayá, a 4 km de la población de Guápulo, margen izquierdo del Río Machañgara, 2600 m, 5 Nov 1988, *M. Bedoya 316* (MO); Calderón, *M.R. Benoist 3478* (CORD, P, S); Quito, Parroquia Guayllabamba, 00°03'S, 78°22'W, ca. 2100 m, 19 Jun

1989, *C.E. Cerón & 3er Curso Biología U. Central 6953* (MO, QCNE); Guayllabamba, al este del puente del Pisque vía Guayllabamba-Tabacundo, 00°01'S, 78°19'W, 2100 m, 14 Jan 1991, *C.E. Cerón & M. Montesdeoca 13138* (MO, QCNE); Parroquia Guayllabamba, puente del Río Guayllabamba, antes del pueblo, 00°05'S, 78°22'W, 2100 m, 26 Jan 1991, *C.E. Cerón & M. Montesdeoca 13171* (MO, QCNE); San Antonio de Pichincha, camino secundario a Culebrillas, 00°04'38"S, 78°26'15"W, 2069 m, 28-30 Jun 2011, *L. Delgado 23* (QCA); Tababela, faldas del lado noreste del Ilalo, 00°15'98"S, 78°27'99"W, 2500 m, 29 Feb 2012, *L. Delgado 251* (QCA); Sigsipampa vía Pifo, 00°14'27.81"S, 78°19'05.05"W, 2813 m, 17 Mar 2012, *L. Delgado 359* (QCA); Tababela, al noroeste del Nuevo Aeropuerto de Quito, 2415 m, 24 May 2012, *L. Delgado* 505 (QCA); Guayllabamba, sector Loma de Inga Cocha, 2073 m, 16-17 Dec 2006, *E. Freire 7152* (QCNE); 3 km N of Guallabamba, ca. 38 km N of Quito on road to Malchinguin, 2800 m, 10 Nov 1974, *A.H. Gentry 12638* (MO, QCNE); Quito, 1843, *K.T. Hartweg 1296* (BM, E, F, G, K, P); between Quito and Cayambe, 2200 m, 30 Jan 1962, *C.B. Heiser 4813* (NY); Guayllabamba, 20 km NE of Quito, 2200 m, 31 Oct 1986, *F. Hekker & W.H. Hekking 10014* (U); Puellaro, 20 km NNE of Quito, 0°0'N, 78°32'W, 2200 m, 31 Oct 1987, *F. Hekker & W.H. Hekking 10023* (U); Río Guayllabamba, 1 km from the Pan-American Highway upstream, 0º03'S, 78º23'W, 2200 m, 4 Apr 1979, *L. Holm-Nielsen 16643* (AAU, QCA) & *16676* (MO, QCA); 1 km north of the addition La Vicentina, 2750 m, 2 Feb 1973, *J.E. Humbles 6146* (F, MO, NY); Sive Andium Quitensium, *W. Jameson s.n.* (NY, US 534694); plains of Pomasqui and San Antonio, *W. Jameson 328* (G, K); Santo Domingo de los Colorados, along Río Toachi near Santo Domingo, 700 m, 20 Jul 1963, *C. Játiva & C.C. Epling 552* (NY); carretera Atahualpa hasta Y de San José de Minas, 2166 m, 12 Apr 2009, *J. Jaramillo & F. Cohello 29118* (QCA); Valley of Guayallabamba, 0º04'S, 78º22'W, 2000-2150 m, 17 May 1986, *P.M. Jørgensen et al. 61307* (AAU, QCA, QCNE); Parque Recreacional Jerusalem, km 8 carretera Guayllabamba-Puéllaro, 0º01'S, 78º22'W, 1900-2500 m, 28 Aug 1990, *C. Josse 480, 487* & *539* (QCA, QCNE); Quito-Ibarra rd, ca. 3 km NE of Guayllabamba, 0º03'S, 78º19'W, 2300 m, 2 Jun 1979, *B. Løjtnant & U. Molau 14076* (AAU, QCA); vía San Antonio de Pichincha-San José de Minas, Hacienda Tanlahua, 00°06'N, 78°29'W, 2220 m, 20 Sep 1994, *T. Núñez et al. 39* (MO, QCNE); Santo Domingo de los Colorados, 61 m, 29 Oct 1960, *T.D. Pennington 60SD* (NY); Quebrada del Chiche, 0°8'55"S, 78°28'W, 2200-2400 m, 12 Dec 2004, *C. Quintana & S. Valencia 441* (QCA); Quebrada del Chiche, 00°10'53"S, 78°22'34"W, 2200-2400 m, 3 Nov 2005, *C. Quintana & S. Valencia 520 ½* (QCA) & 14 Sep 2006, *C. Quintana & S. Valencia 669* (QCA); Parque Recreacional, Bosque Protector Jerusalén, 00°00'14.7"S, 78°23'50.5"W, 2301 m, 11 Mar 2009, *C. Quintana et al. 843* (QCA); Machachi, Reserva Ecológica Los Ilinizas, Hacienda Buenos Aires, 00º28'24"S, 78º40'10"W, 3378 m, 19 Aug 2003, *J.E. Ramos et al. 7438* (QCNE); vicinity of San Antonio and Pomasqui, 29 Oct 1918, *J.N. Rose & G. Rose 23566* (NY, US); Pomasqui, in coll. silvat. asper, Oct 1870, *L. Sodiro s.n.* (AAU); San Antonio, 41 km N of Quito, 2600 m, 4 Sep 1964, *D.D. Soejarto & E. Hernández 1393* (COL, ECON, GH, US); Río Guallabamba, 1900 m, 2 Mar 1967, *B. Sparre 14653* (S, US); Tabacundo, near bridge across Río Pisque, lower slopes of Loma de Asuajato, 21.5 km W of Tabacundo, 1975 m, 1 Jul 1996, *G.L. Webster et al. 32023* (MO); carretera Quito-Mitad del Mundo-Perucho-Guayllabamba, 2500 m, 17 Apr 1987, *V. Zak 1970* (AAU, C, GB, MEXU, MO, NY, QCA, UC); **Santa Elena**: cerca a Salinas (via Ibarra-San Lorenzo), 1 Mar 1980, *J. Inca s.n.* (Q 0015971); **Tungurahua**: de Cunchibamba a Tangaleo, 2550-2700 m, 23 Oct 1944, *M. Acosta Solís 8597* (F); entre Pelileo y "La Tranquilla", 2200-2250 m, 14 Nov 1944, *M.*

Acosta Solís 8916 (F); Ambato, 9 Jul 1939, *E. Asplund 7576* (NY, P, S); vicinity of Patate, hacienda Leito, 2450 m, 6 Aug 1939, *E. Asplund 8104* (NY, S, US); Baños, 1800 m, 24 Mar 1956, *E. Asplund 19939* (S); above Ambato, 8,000 ft, 9 Jul 1939, *E. Balls 7153* (E, F, UC, US); Baños, hacia el sur de la ciudad, en la sendero de La Virgen, 00°24'05''S, 78°25'23,4''W, 1847, 18 Aug 2017, *G.E. Barboza & S. Leiva González 4854* (CORD, LOJA); Baños, margin of road in town, 6 Dec 1982, *L. Bohs 2026* (GH, QCA, QCNE); Baños, in center of town, 8 Aug 1983, *L. Bohs 2190* (F, GH, QCA, QCNE); Ambato, Quinta La Liria, 21 Aug 1984, *E. Bravo V. 615* (QCA); along the Río Pastaza just west of Baños, 6000 ft, 16 Apr 1945, *W.H. Camp E-2373* (CORD, NY, S); Cantón Ibarra, Parroquia Ambuquí, 1800 m, 8 Dec 1990, *C.E. Cerón & M. Montesdeoca 12519* (MO, QCNE); Rio Ambato valley a little W of Ambato, Nov 1952, *F. Fagerlind & P.-G. Wibom 1016* (S); Ulba, 1820 m, 1 Aug 2005, *L. Flores 23* (CHEP); trail to Hacienda El Refugio above Baños, 01°25'48"S, 78°25'12"W, 1800-1960 m, 24 Apr 1980, *A.H. Gentry & C. Bonifaz 28785* (MO); Ambato, 2608 m, 12 Feb 1952, *B. González C. s.n.* (QCA); Baños, 5 Mar 1920, *O. Heilborn 426* (S); Weg von Baños nach Pelileo, am Derrumbo bei Lligua, 1800 m, 10 Jan 1933, *E. Heinrichs 182* & *184* (G, Z); Baños, path to Buena Vista Café, 27 Jun 2003, *L.R. Landrum et al. 10868* (NY); bei Baños am Tunguragua, 2000 m, Dec 1880, *F.C. Lehmann 438* (BM, US); alrededor de Baños, hacia el mirador, por camino a la La Virgen, 1°24'05''S, 78°25'23.4''W, 1847, 18 Aug 2017, *S. Leiva González & G.E. Barboza 6585* (CORD, HAO); Río Chambo, 30 Apr 1969, *H. Lugo S. 1249* (GB, MO); Illusca, in the vicinity of Baños, 21 Apr 1972, *H. Lugo S. 2444* (GB, MO, QCA); Baños, 1820 m, 14 Jan 2005, *V. Montenegro 1576* (CHEP); vicinity of Ambato, Dec 1918, *A. Pachano 78* (NY, US); vicinity of Baños, 1800 m, 11 Mar 1939, *C.W. Penland & R.H. Summers 44* (F, GH, US); Baños, 24 Nov 1974, *T.C. Plowman & E.W. Davis 4572* (GH, US); eastern Cordillera, slope of Mt. Tungurahua, 1900 m, Feb 1933, *A. Rimbach 391* (MICH, S); Baños, steep slopes S of town, 01º24'09,8"S, 78º25'11.9"W, 1950 m, *E.J. Tepe & M.P. Moreno 3014* (QCNE).

EL SALVADOR. Ahuachapán: San Francisco Menéndez, El Corozo, Mariposario, zona alta "Los Sánchez", 350 m, 7 Jul 2000, *J.M. Rosales 1044* (B, LAGU, MO); El Imposible, San Benito al S de La Cumbre, 16 Mar 1992, *E.A. Sandoval 308* (MO); San Benito, N del Recibidero, Cerro León, 29 Jul 1992, *E.A. Sandoval et al. 550* (MO); al pie del Cerro La Leoncita, 650 m, 15 Jan 1998, *E.A. Sandoval 1743* (MO); **La Libertad**: Finca La Hiralda, 1100 m, 26 Jan 1999, *K.J. Sidwell et al. 695* (MO); **Santa Ana**: camino a Cerro Verde, 1000 m, 30 Sep 1959, *M.G. de Menjivar & E.A. Montalvo 3846* (MO); **San Miguel**: El Pacayal, 4 Feb 1999, *R. Villacorta et al. 2819* (B, BM, EAP, ITIC, LAGU, MO, NY).

GUATEMALA. Baja Verapaz: Salamá, Puente Barranca, 820 m, 24 Jul 1988, *P. Tenorio L. et al. 14756* (MEXU, MO, NY); **Escuintla**: NW slopes of Volcán de Pacaya, road to San Vicente Pacaya, 14º24'16''N, 90º39'74''W, 1225 m, 9 Sep 1997, *M. Nee et al. 47215* (CORD, MO, NY); San Antonio Jute, 780 m, 9 Feb 1939, *P.C. Standley 64892* (F, GH, US); **Guatemala**: Guatemala, 1939, *I. Aguilar 46* (F, GH); Lake Amatitlán (south shore), 19 Apr 1905, *H. Pittier 105* (US); **Huehuetenango**: Cuilco, Sosí Chuiquito, 1129 m, 2 Jul 2003, *A. López c14* (MO, USCG); Aguacatán, 5600 ft., 13 Dec 1934, *A.F. Skutch 1936* (G, GH); Santa Ana Huista, Puente Selegua II, 1500 m, 7 Dec 2006, *A. Soto et al. 1472* (MEXU, MO, USCG); along Río Cuilco, between Cuilco and Aldea of San Juan, 1200-1300 m, 18 Aug 1942, *J.A. Steyermark 50903* (F, GH); **Sacatepéquez**: 2.3 miles SW of

Alotenango on road from Antigua to Escuintla, 1300 m, 26 Jul 1977, *T.B. Croat 41934* (CORD, MO); carretera de terracería a Escuintla, about 4 km down (S) from Alotenango, 14º27'37''N, 90º49'21''W, 1300 m, 14 Sep 1997, *M. Nee et al. 47257* (CORD, MEXU, MO, NY); 3 km S of Alotenango on highway 14, 1500 m, 1-2 Aug 1965, *K.E. Roe et al. 789* (MO); near Pastores, 1600 m, 14 Dec 1938, *P.C. Standley 59930* (F, NY); Alotenango, Astillero municipal, 1100 m, 20 Feb 1997, *M. Véliz 975011* (MO); **Santa Rosa**: Santa Rosa de Lima, collected in village by stream, 947 m, 7 Aug 1994, *J.J. Castillo Mont 2235* (F, MO); Santa Rosa, Jun 1892, *E.T. Heyde & E. Lux 3436* (F, GH, K, M, US); same locality, May 1903, *E.T. Heyde & E. Lux 4545* (F, G, GH, NY, US).

HONDURAS. **Morazán**: Tegucigalpa, quebrada La Orejona, 1000 m, 10 Sep 1979, *R. Andino U. 69* (MO); Santa Lucía, 10 km NE de Tegucigalpa, 1500 m, 16 May 1982, *I. Cambar 173* (MO); alrededores de La UNAH, por la quebrada La Orejona, 1000 m, 27 Oct 1980, *M.E. Castañeda 79* (MO); Tegucigalpa, alrededores Colonia Miraflores, 1000 m, 22 Oct 1980, *G. Cruz R. 92* (MO); Colonia Miraflores, 900 m, 15 Oct 1978, *A.L. Díaz 246* (MEXU, MO); orilla Quebrada La Orejona, N de la UNAH, 950 m, 22 May 1985, *S. Gómez 190* (NY); Quebrada La Orejona, 950 m, 2 Jun 1986, *M.E. Lara 191* (MO); Olancho, cañón del río del Monumento Natural El Boquerón, ca. 28 km al noreste de Juticalpa, 800 m, 15 Oct 1994, *J.L. Linares 1840* (MEXU); orillas del Río Las Canoas, ca. 3 km al E de Tegucigalpa, 1020 m, 30 Oct 1996, *J.L. Linares 3856* (MEXU); Quebrada Las Anonas, km 1 carretera Tegucigalpa-Valle de Ángeles, frente al Establo El Molino, 1050 m, 7 Jul 2002, *J.L. Linares 6186* (MEXU); alrededores de Nueva Suyapa, 1000 m, 3 Oct 1983, *J.F. Martínez Gallegos 79* (MO); Teguciglapa, UNAH, 900 m, 10 Oct 1979, *O. Martínez 85* (MO); along quebrada Suyapa, near Suyapa, 1100 m, 10 Nov 1947, *A. Molina R. 561* (GH, MO, US); camino viejo entre Tegucigalpa y Suyapa, 1100 m, 6 Nov 1948, *A. Molina R. 1457* (F, GH); Quebrada de las Burras, entre Suyapa y Tegucigalpa, 1050 m, 11 Dec 1948, *A. Molina R. 1820* (F, GH); entre la Travesía y Villa San Roque, 1200 m, 1 Aug 1950, *A. Molina R. 2784* (GH, US); Quebrada Suyapa, 1200 m, 19 Aug 1950, *A. Molina R. 3200* (F) *& 3212* (F, GH, US); Tegucigalpa, orillas del Río Chiquito, 939 m, 28 Sep 1950, *A. Molina R. 3248* (GH, US); Puente Colorado, al N de Tegucigalpa, 1000 m, 16 Jan 1951, *A. Molina R. 3875* (GH, US); Tegucigalpa, alrededores Ciudad Universitaria UNAH, 950 m, 10 Feb 1977, *C. Nelson 3908* (MO); Distr. Central, alrededores de Colonia Venezuela, 950 m, 11 Sep 1982, *V.M. Piñeda s.n.* (MO 153547); Quebrada la Orejona, predios de la UNAH, 950 m, 21 May 1985, *D. Ruiz 185* (NY); camino viejo entre Tegucigalpa y Suyapa, 1100-1200 m, 6 Nov 1948, *P.C. Standley 14190* (US); vicinity of Tegucigalpa, Barrio Concordia near Río Choluteca, 900-1000 m, 7 Oct 1949, *P.C. Standley 24095* (GH, US); vicinity of Suyapa, Villa San Roque, 1100-1200 m, 1 Aug 1950, *P.C. Standley 26247* (GH).

MÉXICO. Without locality, *M. Sessé & J.M. Mociño 1508* (F); without locality, 1937, *L.A. Kenoyer s.n.* (MO 561617); **Chiapas**: Tenejapa**,** salida del Río Cruz Pilal, 21 Jun 1984, *G. Alonso Méndez 7709* (MEXU, NY); La Trinitaria, 30 miles south of La Trinitaria, 4500 ft, 27 Jun 1965, *D.E. Breedlove 10591* (CAS, F, US); 13 km N of Arriaga, 830 m, 18 May 1972, *D.E. Breedlove 25238* (CAS, ENCB, MO); Ocozocoautla de Espinosa, along rd. to Mal Paso, 900 m, 25 Jun 1972, *D.E. Breedlove 25667* (CAS, ENCB, MO); Berriozábal, 6-8 km N of Berriozábal along road to Pozo Turipache and Finca el Suspiro, 1100-1150 m, 24 Jul 1972, *D.E. Breedlove 26362* (CAS, MO); Cintalapa, 5 km W of Rizo de Oro, 900 m, 26

Aug 1974, *D.E. Breedlove 36720* (CAS, MEXU, MO); Ocozocoautla de Espinosa, canyon at the head of the Río de la Venta at the Chorreadero near Derna, 800-1000 m, 1 Sep 1976, *D.E. Breedlove 39818* (CAS, MO); Motozintla, outwash plain below Motozintla, 15°22'55"N, 92°12'19"W, 1250 m, 4 Oct 1976, *D.E. Breedlove 40592* (CAS, MEXU, MO); on the Llano de San Juan above Ocozocoautla NE of the new Tuxtla Gutierrez Airport, 1020 m, 3 Oct 1979, *D.E. Breedlove 44367* (CAS); San Fernando, at the tunnel on the road from Tuxtla Gutiérrez to the Chicoasen Dam., 950 m, 14 Jul 1981, *D.E. Breedlove 51520* (CAS, MEXU, MO); above Ocozocoautla near Llano de San Juan, 800 m, 12 Sep 1988, *D.E. Breedlove 69942* (CAS); 10-15 km W of Ocozocoautla on road to El Ocote, 880 m, 25 Oct 1988, *D.E. Breedlove 70605* (CAS); Berriozabal, above El Suspiro, 1080 m, 1 Nov 1988, *D.E. Breedlove 70888* (CAS); Cintalapa, oeste del Cacahuatal "Cañada del Diablo", 700 m, 10 Jul 1994, *J. Castillo et al. 165* (MEXU); Mt. Male, near Porvenir, 3200 m, 6 Jul 1941, *E. Matuda 4656* (GH, MEXU, MO, NY,); between Mazapa and Motozintla, 1200 m, 19 Jul 1941, *E. Matuda 4868* (GH, MEXU); Finca Irlanda, 1 Aug 1913, *E. Matuda 6976* (MO, US); Berriozábal, Las Vistas, 12 Jun 1950, *F. Miranda 6374* (MEXU); Mts. west of Monserrate, *C.A. Purpus 124* (UC, US); Tzimol, 5 km al SW de Tzimol, 1150 m, 14 Sep 1988, *A. Reyes García & G. Urquijo 753* (MEXU, MO); Ocozocoautla, 1 km al NW del entronque Aeropuerto-Ocozocoautla-México, 940 m, 17 Sep 1988, *A. Reyes García & G. Urquijo 982* (MEXU, MO); same locality, 1000 m, 11 Nov 1988, *A. Reyes García & G. Urquijo 1237* (MEXU, MO); Comitán, 8 km al SE de Tzimol, sobre la desviación a Uninajab, 7 Jul 1990, *A. Reyes García et al. 1828* (MEXU, MO); Laguna Coilá, 2 km al E de Uninajab y 500 m al S de Santa Rita (Trapichito), 1140 m, 7 Jul 1990, *A. Reyes García et al. 1844* (MEXU); 1 km al NW del entronque Aeropuerto-Ocozocoautla-México, ca. 1000 m, 16 Jul 1990, *A. Reyes García & R. Hampshire 1933* (MEXU); Jiquipilas, Ejido Tiltepec, 16°20'43"N, 93°54'15"W, 1070 m, 6 Jun 2002, *A. Reyes García & C. Chavarria 5066* (MEXU, MO); San Juan Mixtepec, Yúu nii (Cañada Antigua), a 9 km al E de San Juan Mixtepec, 17°18'N, 97°17'W, 1900 m, 30 Aug 1988, *J. Reyes Santiago & F. Reyes S. 594* (MEXU); Tenejapa, Jul 1982, *A.S. Ton 4395* (MEXU); **Durango**: San Dimas, 5 km de Mala Noche por el camino a La Desmontada, 1280 m, 7 Mar 1990, *M. González 2398* (MEXU); Tepehuanes, Los Cedros al SW de Tabahueto, La Hacienda-Topia, 900-960 m, 5 Sep 1983, *P. Tenorio L. et al. 4204* (MEXU); Tabahueto, 196 km al W de Tepehuanes, 1330 m, 29 Aug 1983, *E. Torrecillas 75* (MEXU); Tamazula, Tabahueto (al catorce), 196 Km al W de Tepehuanes, 29 Aug 1983, *R. Torres C. et al. 3549* (MEXU, MO); **Guanajato**: Guanajato, 1500 m, 4 Jul 1988, *J.G. Maldonado Y. s.n.* (SLPM 24534) (MEXU); El Blanco, 10 km al suroeste de Atarjea, 2000 m, 9 Nov 1988, *E. Ventura V. & E. López 6332* (MEXU); Llano Grande, 10 km al E de Xichú, 1500 m, 14 Sep 1989, *E. Ventura V. & E. López 7328* (MEXU); Atarjea, Mina de La Liga, 1600 m, 11 Oct 1990, *E. Ventura V. & E. López 8925* (MEXU); Xichú, El Tanque, 1300 m, 4 Dec 1991, *E. Ventura V. & E. López 9887* (MEXU); **Guerrero**: S. Chilpancingo, 22 Jun 1935, *O.M. Clark 7135* (MO, NY); Mina Zihuaqueo, 1050 m, 21-22 Aug 1936, *G.B. Hinton 9311* (CAS, GH, LIL, MEXU, U, US); **Hidalgo**: Jacala, 4500 ft, 23 Jun 1939, *V.H. Chase 7075* (F, GH, MO); Zimapan, *T. Coulter 1237* (K, GH); 15 km al NO de Jacala rumbo a Pacula, 1725 m, 20 Aug 2007, *A. Frías-Castro et al. 673* (MEXU); Mextitlan, 4000 m, Aug 1840, *H.G. Galeotti 1189* (BR, G); 5 Km al SE de Venados, ca. 800 m, 5 Jul 1972, *F. González Medrano et al. 4148* (MEXU, MO); Xilotla, al S de Meztitlán, 20 km al NW de Venados, Jul 1972, *F. González Medrano 4181* (MEXU, MO); Cardonal, Tolantongo, cañada de la Piedra Cerrada, 1300 m, 28 Aug 1976, *F. González Medrano 9454* (MEXU); Atotonilco el

Grande, Barranca de Venados, 1900 m, 4 Nov 1980, *R. Hernández Magaña 5302* (MEXU, MO, USCG); Atotonilco el Grande, Barranca de Venados, 1900 m, 8 May 1981, *R. Hernández Magaña et al. 6045* (MEXU, MO, USCG); Cardonal, Barranca de Tolantongo, 10 Jun 1976, *P. Hiriart V. & G. Ortiz C. 22* (MEXU, MO); Barranca Tolantongo, 1 Nov 1946, *F. Miranda 4021* (MEXU); Pacula, Jacala, between Hilo Juanico and Pacula, 1400 m, 30 Oct 1946, *H.E. Moore Jr. 1820* (GH, UC); between Hilo Juanico and Barranca Seca, on trail from Jacala to Pacula, 1400-1600 m, 9 Jul 1948, *H.E. Moore Jr. 3831* (CORD, GH, MEXU); 7 km al NE de Mezquititlán, sobre la carretera a Zacualtipán, 1750 m, 17 Nov 1974, *J. Rzedowski 32523* (CORD, ENCB, MEXU); Jacala, 10 Aug 1937, *M. Taylor E. 108* (CAS, F, MO); **Jalisco**: Casimiro Castillo, 7-8 km al NNE de C. Castillo, Arroyo La Calera, 19°40'16"N, 104°25'04"W, 800-1000 m, 14 Jul 1988, *R. Cuevas & D. Deniz 3081* (MEXU); Tonalá, Barranca d Huentitan, 2 km al E de Tonalá, 1200 m, 29 Jul 1990, *A. Flores M. & P. Ramos G. 2413* (MEXU, MO); 16 km al SSW de Autlán, 8-9 km al NE de Casimiro Castillo, La Calera, 19°38'37"N, 104°22'07"W, 600-800 m, 17 Aug 1988, *L. Guzmán & F.J. Santana 303* (MEXU); Guadalajara, ladera SW de la Barranca del Río Santiago (Oblatos), 1165m, 18 Jul 1997, *M. Harker et al. 2432* (MEXU); Jocotepec, Barranca de Sayula, al oeste de San Pedro Tesistán, 1750 m, 5 Jul 1992, *J.A. Machuca N. 6889* (MEXU); Tonala, Colimilla, Barranca del Río Santiago, 1400 m, 1 Sep 1989, *R. Ramírez Delgadillo & L. Portillo M. 1497* (MEXU); ca. 20 km NE of Antlán, 1200 m, 27 Mar 1965, *R. Mc Vaugh 23267* (ENCB); **México**: Temascaltepec, Acatitlan, 18 Jul 1933, *G.B. Hinton 4339* (UC, US); **Michoacán**: Los Reyes/Periban, Los Chorros del Varal, 900-1000 m, 29 Sep 2005, *I. García & H. Cortéz 7235* (MEXU); Zitácuaro-La Florida, 21 Jun 1938, *G.B. Hinton 11977* (CAS, GH, LIL, US); Tlalpujahua, 1829, *F.W. Keerl s.n.* (BR); **Morelos**: above Cuernavaca, 6500 ft, 24 Sep 1896, *C.G. Pringle 6505* (E, MEXU); **Nueva León**: Linares, near Ebanito, 750 m, 24 Sep 1979, *J.C. Hinton 17588* (CORD, GBH, MEXU); below Ebanito, 680 m, 5 Aug 1980, *J.C. Hinton 17943* (CORD, GBH, MO); Iturbide, Lampazos, 920 m, 21 Sep 1991, *G.S. Hinton 21500* (GBH); Sierra de la Silla, frente a El Mezcal, 28 May 1978, *J.G. Moya 84* (MEXU); Cañón Diente, 12 Aug 1939, *C.H. Müller 2674* (GH, UC); Sierra de la Silla, 28 May 1889, *C.G. Pringle 2544* (BKL, E, F, G, K, MEXU, MO, NY, PH, UC, UPS, US); near Monterrey, 29 May 1908 (fr), *C.G. Pringle 15620* (CAS, F, GH, MO, UC, US); side of Cerro de la Silla, just above km NW Guadalupe, near Monterrey, 18 Dec 1990 , *D. Seigler et al. 13144* (MEXU, MO); 1 km east of dam at Presa de la Boca, 12 May 1991, *D. Seigler et al. 13353* (MEXU); along hwy 85, 2 miles south of Allende (jct. with cut-off to El Centro), 13 Oct 1980, *J. Wussow & G. Landry 253* (MEXU); **Oaxaca**: Santos Reyes Tepejillo, 4 km al N de Santos Reyes Tepejillo, Rio Boquerón, 17°27'N, 97°56'W, 1525 m, 15 Jul 1996, *J.I. Calzada 21036* (MEXU); a 3 km al N de Santos Reyes Tepejillo, senda para Río Boquerón y San Miguel Tlacotepec, 17°27'17,60"N, 97°57'22"W, 1275 m, 16 Jun 1998, *J.I. Calzada 22605* (MEXU); Pluma Hidalgo, cerro Espino, 4 km al E de Concordia, brecha a Finca cafetalera Monte Cristo, 15º52'N, 96º 25'W, 920 m, 21 Jan 1988, *A. Campos V. 1176* (MEXU); San Juan Coatlán, Cañada de San Jerónimo Coatlán (Río Trapiches), 28 Jun 1990, *A. Campos V. 3203* (MEXU); Dist. Teposcolula, ruta 190, ca. 10 km al N de Tamazulapán, a Río del Oro, 2200 m, 11 May 1981, *R. Cedillo Trigos et al. 794* (F); Pochutla**,** Finca Montecristo, 19 km al Ne de la desv. a Chacalapa, 26 Apr 1983, *R. Cedillo Trigos & R. Torres C. 2309* (MEXU, MO); Oaxaca, Jun 1901, *C. Conzatti & V. González 1221* (GH); Pochutla, a 9 km al NE de Chacalapa por camino a Finca Monte Cristo, 1000 m, 1 Jul 1984, *H.M. Hernández M. & R. Torres C. 413* (CAS, MEXU, MO); 3-4 km west of Ixtaltepec, 50 m,

10 Jul 1959, *R.M. King 1525* (US); 4-5 km east of Juchitán, along the Pan-american highway (Routes 185 & 190), 50 m, 12 Jul 1959, *R.M. King 1613* (UC, US); Tehuantepec, recorrido de El Limón a El Milagrito, 8 Jul 1985, *C. Martínez R. 44* (MEXU); Coxtlahuaca, Concepción Buenavista, base del Cerro Pluma, 18°08'24.3''N, 97°19'16.8''S, 1350 m, 4 Jul 1994, *J.L. Panero & I. Calzada 4029* (CTES); Pochutla, San Miguel del Puerto, la Payla, 15°59'44"N, 96°07'28"W, 1381 m, 4 Jun 2005, *J. Pascual 1535* (MEXU, MO); Macuiltianguis, en la orilla de Río Culebra, 1250 m, 2 Aug 1980, *G. Pérez Cruz 245* & *250* (MEXU); San Juan Mixtepec, Cañada de Cerros de Metates, a 20 km NO de San Juan Mixtepec, 1900 m, 8 Jul 1988, *S.J. Reyes 309* (MEXU); ladera Río Mixtepec, a 4 km SE de San Juan Mixtepec, 1900 m, 8 Oct 1988, *S.J. Reyes 972* (MEXU); Dto. Juxtlahuaca, Yúu nii, a 9 km al E de San Juan Mixtepec, 1780 m, 23 Jul 1989, *S.J. Reyes 1782 & 1782a* (MEXU); San Jerónimo Sosola, El Progreso Sosola, 17°25'N, 97°01'W, 1500 m, 26 Jul 1993, *A. Salinas T. & E. Martínez-Correa 7357* (CAS); San Sebastián Abasolo, Santa Rosa Buenavista, 1580 m, 7 May 1985, *A. Saynes Vásquez 657* (MEXU); Coyula, 13 Aug 1895, *L.C. Smith 659* (GH); Santa María Chimalapa, Arroyo Chirimoyal, afluente del Río Portamonedas, a ca 5 km en línea recta al NNE de Benito Juárez, 16º45'N, 94º08' W, 800 m, 28 May 1985, *J. Solomon Maya 1689* (MEXU); San Miguel Chimalapa, cerros al S de El Trebol, ca. 1 km al SE de Benito Juárez, 16º43'N, 94º08'W, 1000 m, 7 Jun 1985, *J. Salomón Maya 1734* (MEXU); Arroyo de las Señoritas, ca. 3-4 km al N de Benito Juárez, 1100 m, 22 Mar 1986, *J. Solomon Maya 3039* (MEXU); Cerro de la Piña, el Cordón El Baúl, ca. 6 km al N de cerro Baíl, 16°38' N, 94°10' W, 1400-1500 m, 6 Jun 1986, *J. Solomon Maya 3430* (MEXU); Santa María Chimalapa, El Hondo del Diablo del Río Portamonedas, ca. 6 km en línea recta al NNE de Benito Juárez, 800 m, 7 Jul 1986, *J. Solomon Maya 3551* (MEXU); Concepción Buenavista, Cerro Cedro, al W de El Enebro, 2260 m, 20 Jul 1985, *P. Tenorio L. 9414* (MO); Santa María Guienagati, arroyo de Caracoles, 16 km al NW de La Chiviza, 1050 m, 15 Mar 1986, *P. Tenorio L. et al. 11156* (MEXU); Tecomavaca, 1 km al W de Pochotepec, 1310 m, 25 Sep 1990, *P. Tenorio L. et al. 17209* (CAS); Pochutla, 9 km al NE de Chacalapa, camino a la Finca Montecristo, 1 Jul 1984, *R. Torres et al. 5457* (F, MEXU, MO); Juchitán, Torre de Microondas, Palma Sola, 11.4 km al W de Almoloya, 760 m, 24 Sep 1984, *R. Torres C. & E.F. Cabrera C. 6173* (CAS, MEXU, MO); Buenos Aires, Tehuantepec, El Carrizal o La Cañada, 960 m, 1 Jul 1987, *R. Torres C. et al. 9826* (F, MEXU, MO); **Puebla**: Coatapec, in the vicinity of San Luis Tultitlanapa, Jul 1907, *C.A. Purpus 2709* (G, UC); Los Naranjos, 1940 m, May 1908, *C.A. Purpus 3362* (E, F, GH, MO, NY, UC, US); Tlacuiloltepac, Jun 1909, *C.A. Purpus 3993* (UC); Caltepec, Callejón del Cosahuico al S de Caltepec, 1730 m, 24 Jun 1984, *P. Tenorio L. & C. Romero de T. 6631* (MO) & 11 Aug 1984, *P. Tenorio L. & C. Romero de T. 6821* (MO); **Querétaro**: Jalpan, 750-800 m, 11 Oct 1977, *E. Argüelles 889* (MEXU); Landa, Río Moctezuma, al S de Tilaco (paso a Los Baños), 500 m, 5 Jul 1988, *E. Carranza 736* (MEXU); Jalpan, 3 km al SW de Soyapilca, 1100 m, 11 Jul 1988, *E. Carranza 772* (MEXU); Pinal de Amoles, 1 km al SW de Escanelilla, 1290 m, 9 Jul 1988, *E. Carranza 766* (MEXU); Landa, 2 km al N de Landa de Matamoros, "Cañada de las Víboras", 1100 m, 30 Jul 1988, *E. Carranza 937* (F); 5 km al S de Arroyo Seco, 950 m, 24 May 1990, *E. Carranza 2455* (MEXU); Arroyo Seco, Río Atarjea, 4-6 km al SW de El Limón, 840-880 m, 23 Jun 1991, *E. Carranza 3212* (GH, MEXU, US); Jalpan, ca. 2 km al WSW de Valle Verde, 1550 m, 1 Jul 1998, *E. Carranza & S. Zamudio 5519* (MEXU); 7 km SE de Tancoyol, 750 m, 30 Apr 1985, *R. Fernández Nava 2946* (G, NY); 3 km al S de Escanelilla, 1100 m, 8 Jun 1986, *R. Fernández Nava 3379* (U); Landa, 7 km al W de

Tilaco, 1050 m, 9 Jun 1986, *R. Fernández Nava 3395* (MEXU); Landa, 2 km al noreste de La Lagunita, 1150 m, 21 Aug 1989, *E. González 874* (MEXU); Jalpan, ca. 5 km debajo de Tanchanaquito, 330 m, 23 Feb 1994, *E. Pérez C. & E. Carranza 2888* (CAS, MEXU); Pinal de Amoles, a 1 km de Escanelilla, Río Escanela, 20°24.13'N, 100°11.76'W, 1980 m, 14 Nov 1996, *M. Martínez 3431* (MEXU); Landa, 2 km al sureste de Jaguey Colorado, 900 m, 7 Aug 1989, *H. Rubio 949* (CAS); Landa, 1 km al suroeste de Tangojo, 750 m, 31 Aug 1989, *H. Rubio 1037* (MEXU); Landa de Matamoros, 2-5 km al noroeste de El Jagüey Colorado, 700 m, 9 Oct 1990, *H. Rubio 2026* (MO); Jalpan, a 5 km al SE de Jalpan, por la carretera hacia San Juan del Río, 1400 m, 1 May 1982, *P. Tenorio L. & R. Hernández M. 327* (F, MEXU); Arroyo Seco, 4 km al E de Arroyo Seco, 840 m, 20 Oct 1982, *P. Tenorio L. & C. Romero de T. 2279* (MEXU, NY); Querétaro, 790 m, 6 Aug 1992, *I. Trejo 2424* (MEXU); **San Luis de Potosí**: near Los Canoas, Oct 1902, *E. Palmer 272* (F, GH, MO, NY, UC, US); Las Canoas, 900-1000 m, 6-7 Aug 1934, *F.W. Pennell 17941* (US); same locality, 17 Jun 1891, *C.G. Pringle 4057* (BKL, G, GOET, MEXU); Minas de San Rafael, Nov 1910, *C.A. Purpus 4893* (F, MO, UC, US); Minas de San Rafael, Nov 1910, *C.A. Purpus 5051* (UC, US); W slope of Sierra Madre Oriental, 5 km W of jct of highway 86 with roadas to Rayon and Cardenas, ca. 1000 m, 21º50'N, 99º40'W, 30 Sep 1965, *K.E. Roe & E. Roe 2223* (LE); Tamasopo, 500 m, Jan 1956, *J. Rzedowski 6849* (ENCB); El Zapote, 20 km al SSW de Río Verde, 1300 m, 7 Jun 1956, *J. Rzedowski 7663* (ENCB); unos 10 km al W del ejido D. Carmona, 700 m, 27 May 1959, *J. Rzedowski 10641* (ENCB); San Luis de los Montes, 800 m, 28 May 1959, *J. Rzedowski 10668* (ENCB, MEXU); unos 25 km al W de Ciudad Valles, 350 m, 11 Sep 1967, *J. Rzedowski 24373* (ENCB); 2 km al NNE de Cárdenas, 1300 m, 15 Aug 1967, *J. Rzedowski 24631* (CAS, ENCB); Villa de Zaragoza, 8 km al E de Las Rusias Carretera a Río Verde, 1570, 10 Oct 1982, *P. Tenorio L. & C. Romero de T. 2229* (MEXU, NY); Los Aguajitos, 11 km al NE de Guadalcázar, hacia Pozo de Acuña, 22º37'30"N, 100º19'0" W, 17 Nov 1996, *R. Torres C. 148709* (MEXU); **Tamaulipas**: vicinity of Marnolejo, 3 Aug 1930, *H.H. Bartlett 10806* (CAS, F, MEXU, US); between Ciudad Mante and Ciudad Valles, 80 m, 12 Jan 1987, *T.B. Croat & D.P. Hannon 62946* (MEXU, MO); W of Antiguo Morelos, 6.1 mi W of Antiguo, 13 Jan 1987, *T.B. Croat & D.P. Hannon 62988* (CAS, MO); Gómez Farías, 2 km al NO de Gómez Farías, brecha a Rancho del Cielo, 650 m, 6 May 1986, *A. García 2203* (MEXU); Tula, 3 km al sureste de Las Cruces, 26 May 1977, *F. González Medrano 10492* (MEXU); Ejido El Carrizal, 18 km al N de Manuel Veg., 300 m, 1 May 1982, *F. González Medrano 12578* (MEXU); Ciudad Victoria, 3 km al SE del Rancho Joya Verde, a 26 km al SW de Cd. Victoria, 900 m, 11 Sep 1982, *F. González Medrano 12872* (MEXU); 3 km al S del Ejido El Sabinito, en la Carr. Soto La Marina-Cd. Victoria, al sur de Tamaulipas, 9 Sep 1984, *L. Hernández 1206* (MEXU); camino de San José a los Diez Millones y la Joya Oscura, 23°2'N, 99°13'W, 1250 m, *L. Hernández 3099* (MEXU); 3 km al oeste de Cd. Victoria, 350 m, 21 Sep 1984, *P. Hiriart 404* & *417* (MEXU); 16 miles west of highway 85 on road A70 to Ocampo, 30 Dec 1977, *N.F. McCarten & R.L. Bittman 2585* (UC); Llera, 5 Km al E de Congregación Garza en el camino al Picacho, 400 m, 12 Oct 1986, *M. Martínez 1329* (MEXU, MO); San Carlos, 2 km al E de Marmolejo, en el camino al Rosario, 24°37'N, 99°1'W, 700 m, 18 Mar 1994, *M. Martínez 2183* (MEXU); San Nicolás, a 2 km del inicio de la brecha a González, 24°33.1'N, 98°41.8'W, 420 m, 28 Nov 1998, *M. Martínez 5243* (MEXU); Victoria, vicinity of Victoria, 320 m, Feb-Apr 1907, *E. Palmer 173* (F, GH, K, MO, NY, UC, US) & May-Jun 1907, *E. Palmer 456* (F, US); Nogales, Jaumave, Jul 1932, *H.W. von Rozynski 467* (B, F, G, K, P, US); Mt. Cañón, near Victoria, 400 m, 4 Apr 1976,

R. Runyon 948 (US); Las Flores, 22°48'N, 99°24'W, 450 m, 24 Jul 1992, I. *Trejo 2321* (MEXU); Gómez Farías, ca. 2 km al NW de Gómez Farías, 450 m, 11 May 1982, *A. Valiente B. 98* (MEXU); ladera de orientación W sobre la Sierra Chiquita, 450 m, 12 Jun 1982, *A. Valiente B. & J.L. Viveros S. 179* (MEXU); 2 km al Este del poblado de Gómez Farías, 450 m, 16 Jul 1982, *A. Valiente B. 272* (CTES, MEXU) & 14 Sep 1982, *A. Valiente B. & J.I. Solís 360* (MEXU); Juamave, 1 May 1930, *H.W. Viereck 350* (US);Tamaulipas, Dec 1930, *H.W. Viereck 1011* (US); Victoria, Cañón al W de Ciudad Victoria atras de La Sierra Gorda, 13 May 1986, *M. Yáñez 783* (MEXU, MO); **Veracruz**: Actopan, Cerro de La Cruz, (sierra Manuel Díaz), 19°34'N, 96°27'W, 650 m, 21 Jul 1985, *R. Acosta P. & F. Vazquez B. 753* (MEXU); Zacuapan, Dec 1906, *J. Brandegee 2294* (F); E. Zapata, Lomas de Rogel, 19°33'34"N, 96°47'37"W, 640 m, 30 Jun 1999, *G. Castillo Campos et al. 19219* (MEXU); unos 4 km antes de San Nicolás, 650 m, 4 Jul 1972, *J. Dorantes et al. 1216* (CAS, MEXU, MO); Comapa, Barranca de Panoaya, 2.5 km al NE de El Coyol, 19°12'N, 96°41'W, 350 m, 26 Aug 1985, *M.E. Medina A. & F. Vazquez B. 542* (MEXU); unos 3 km al E de Río Blanco, rumbo a Acultzingo, 1560 m, 29 Jul 1971, *L.I. Nevling & A. Gómez Pompa 2317* (F); Noalinco, 6 km ESE of San Antonio Paso del Toro, 19º34'N, 96º47'W, 650 m, 14 Jan 1984, *M. Nee & K. Taylor 28811* (CORD, F, MO, NY, UC, XAL); Actopan, 3 km antes de la desviación al Coyolillo rumbo Actopan, 500 m, 19 Aug 1976, *R.V. Ortega 494* (F, MEXU); Zacuapan and vicinity, Barranca de Tenampa, Nov 1906, *C.A. Purpus 2294* (F, GH, MO, NY, UC, US); Zacuapan, Corral de Piedra, Dec 1914, *C.A. Purpus 7562* (G, M, UC, Z); Barranca de Panoya, Dec 1919, *C.A. Purpus 8424* (UC); Hidalgo, Los Baños, ca. 25 km al WSW de Huayacocotla, cerca de San Nicolás Atecoxco, 1500 m, 6 Jul 1990, *J. Rzedowski 49555* (MEXU); Acultzingo, 2 km west of Acultzingo, 1900 m, 22 Jun 1984, *S.L. Solheim 1654* (MEXU, NY); El Trapiche, 400 m, 15 Feb 1971, *F. Ventura A. 3117* (CAS, ENCB); Dos Ríos, Los Reyes, 670 m, 24 Jun 1971, *F. Ventura A. 3945* (CAS); Actopan, Trapiche, 470 m, 6 Oct 1971, *F. Ventura A. 4367* (CAS); Alto Lucero, Providencia, 600 m, 6 Dec 1973, *F. Ventura A. 9378* (G, MEXU); Dos Ríos, Corral Falso, 650 m, 3 Oct 1974, *F. Ventura A. 10619* (CORD); Actopan, San Nicolás, 500 m, 13 Oct 1976, *F. Ventura A. 13411* (G, MEXU); Seis de Enero, 750 m, 2 Dec 1976, *F. Ventura A. 13678* (CAS, ENCB); Xalapa, San Antonio, 650 m, 27 Aug 1977, *F. Ventura A. 14331* (CORD, ENCB, MEXU).

NICARAGUA. **Boaco**: Boaco, 600-700 m, 12º29'N, 85º41'W, 30 Jul 1981, *P.P. Moreno 10196* (MO); **Estelí**: Reserva Natural de Miraflores (RNM), comunidad de San José del Rodeo, entre la casa de Bayardo Moreno y la Quebrada Grande, 13°13'N, 86°17'W, 800-840 m, 16 Jun 2003, *I. Coronado G. & I. Velázquez 24* (MO); RNM, comunidad de San José del Rodeo, de la casa de Bayardo Moreno hacia el sur de la Quebrada Pequeña, 958 m, 19 Jun 2003, *I. Coronado G. & I. Velázquez 149* (MO); Miraflor Moropotente, parcela 3, 100 m al oeste de la casa de habitación del Sr. F. Castillo, 910 m, 23 Oct 2007, *D.I. Moreno V. 34* (HULE; MO); El Chayote, 23 km al N de Estelí, 1200-1300 m, 1 Apr 1983, *P.P. Moreno 21241* (MEXU, MO); El Chayote, 1100-1200 m, 31 Jul 1983, *P.P. Moreno 21761* (MEXU, MO); Kukamonga, Km 167, Portal de Belén, 660-800 m, 1 Aug 1983, *P.P. Moreno 21824* (MEXU, MO); La Gavilana, 940-1000 m, 2 Aug 1983, *P.P. Moreno 21875* (MEXU, MO); Paisaje Terrestre Protegido Miraflor Moropotente, parcela 2 en la Comunidad El Coyolito, 910 m, 23 Oct 2007, *N. Pinell R. 27* (HULE, MO); Miraflor-Moropotente, Comunidad Las Palmas, 1100 m, 24 Dec 2007, *N. Pinell R. 61* (HULE, MO); RNM, Comunidad El Terrero, 900 m, 22 Oct 1999, *R.M. Rueda 12112* (MO); Miraflor

Moropotente, parcela 2 en la Comunidad El Coyolito, 13 Oct 2007, *R.A. Rugama 19* (HULE, MO); Reserva paisaje protegido Miraflor Moropotente, Comunidad El Coyolito, 910 m, 23 Oct 2007, *E.L. Siles G. 30* (HULE, MO, NY); RNM, comunidad de Saca de Agua, 700-800 m, 26 Jan-2 Feb 2008, *E.L. Siles G. 58* & *68* (HULE, MO); Salto de Estanzuela, al S de Esteli, 1000 m, 8 Aug 1984, *D. Soza et al. 141* (MO, US); Km 168 on Hwy 1, 16 km N of Estelí, Cuesta de Kukamonga, 800-850 m, 17 Jun 2008, *W.D. Stevens et al. 27284* (HULE, MO); **Granada**: Volcán Mombacho, Hacienda UPE-Pancasán (antes El Cráter), 600 m, 2 Jul 1983, *A. Grijalva et al. 2712* (MO); Volcán Mombacho, 600 m, 11º45'20''N, 85º58'30''W, 7 Aug 1983, *A. Grijalva et al. 2921* (MEXU, MO); **Jinotega**: along trail between Jinotega and Las Mesitas, west of Jinotega, 1100-1400 m, 21 Jun 1947, *P.C. Standley 9714* (F) *& 9810* (F, US); vicinity of Jinotega, 1030-1100 m, Jun-Jul 1947, *P.C. Standley 10058* (F, US); Cerro de la Cruz, 1190 m, 8 Feb 2010, *W.D. Stevens & O.M. Montiel 29552* (HULE, MO); Km 138 along new road to Jinotega, 1160 m, 11 May 2010, *W.D. Stevens & E. Duarte M. 29871* (HULE, MO); middle E slopes of Cerro de la Cruz above (W of) Jinotega, 1210 m, 22 May 2011, *W.D. Stevens & O.M. Montiel 31749* (HULE, MO); **Madriz**: Somoto, Cerro Quisuca, 3035 ft, 21 Aug 2010, *I. Coronado G. et al. 5331* (HULE, MO); Cerro Quisuca, 1100-1250, 22 Nov 1979, *W.D. Stevens & O.M. Montiel 16042* (MEXU, MO) & 31 May 1980, *W.D. Stevens & O.M. Montiel 17325* (MEXU, MO); Cerro Quisuca, summit and upper slopes, 1100-1250 m, 12 Jul 1983, *W.D. Stevens & O.M. Montiel 22220* (CAS, MEXU, MO); **Matagalpa**: between Simon de Palcella and Mesa La Cruz, 1150 m, 30 Nov 1980, *W.D. Stevens & O.M. Montiel 18532* (MEXU, MO); **Nueva Segovia**: 1 km S of Mozonte-San Fernando road (at Km 245.1), 715 m, 22 May 2010, *W.D. Stevens et al. 30086* (HULE, MO).

PERU. Amazonas: Luya, Camporredondo, camino Ishangas-Cedro-El Toche, 1420-1680 m, 30 Mar 1997, *J. Campos de la Cruz et al. 3732* (MO); Chachapoyas, on road to Caclic, 13 km below and best of Chachapoyas, 1725 m, 13 Oct 1964, *P.C. Hutchison & J.K. Wright 6905* (F, MO, NY, UC, US, USM); from Pte. Ingenio toward Chachapoyas, 1600 m, 25 Feb 1976, *T.C. Plowman 5566* (GH, MO, USM); roadside between Hierba Buena and Tingo, along Rio Utcubamba, 6º25'59"S, 77º52'25"W, 1855 m, 12 Dec 2007, *S. Stern et al. 134* (NY); Bagua, Pongo de Rentema, 400 m, 4 Feb 1999, *R. Vásquez et al. 25940* (MO, USM); **Cajamarca**: San Ignacio, San José de Lourdes, camino a Crucero, 800 m, 8 Jan 1999, *J. Campos de la Cruz & L. Campos 5988* (MO, NY); Cajamarca, prope Caxamarca, *F.W.H.A. von Humboldt s.n.* (GH); Jaén, vicinity of Pucará on the Río Huancabamba, 950 m, 12 Jan 1964, *P.C. Hutchinson & J.K. Wright 3549* (F, M, MO, NY, UC, US, USM); Santa Cruz, Chancay, 1900 m, 18 Nov 1986, *J. Mostacero et al. 1768* (HUT); Jaén, San Antonio (km 81-Pucará), 990 m, 12 Oct 1965, *A. Sagástegui A. 5846* (CORD); Celendin, Las Chirimoyas, 1500 m, 3 Feb 1985, *A. Sagástegui A. et al. 12502* (MO); Contumazá, Andaloy, 1600 m, 27 Mar 1985, *A. Sagastegui A. & S. Leiva González 12515* (HUT, MO); Andaloy (San Benito-Yetón), 2000 m, 23 May 1988, *A. Sagástegui A. 13036* (NY, US); Corlás Cascas-Contumazá, 1650 m, 13 Apr 1995, *A. Sagástegui A. 15559* (MO, NY, QCA); San Pablo, San Pablo-Sangal, 2000 m, 15 Apr 1995, *A. Sagástegui A. 15612* (F, QCA); 44 km after Chota on road to Santa Cruz, 3 May 2003, *M. Weigend et al. 7528* (USM); Pucará, 900 m, 14 Apr 1960, *F. Woytkowski 5678* (G, GH, MO, US); **Piura**: Huancabamba, ruta a Salala, 2100 m, 1 May 1990, *I. Sánchez Vega et al. 5138* (F); Huacabamba, 1900 m, Apr 1912, *A. Weberbauer 6059* (CORD fragment, F, GB, GH, MO);

VENEZUELA. Without locality, 1865, *J.W.K. Moritz s.n.* (BM), Andes, 26 Feb 1930, *C.J. Rutten-Peckel Harig 4* (U); **Anzoategui**: La Soledad, 500 m, Aug 1953, *Bro. Ginés (P. Mandazen Soto*) *3901* (US); Isla Los Monos, N Canoma, N Pertigalete, 10º16'N, 64º33'W, 0-15 m, 2 Sep 1973, *J.A. Steyermark & B.J. Manara 107966* (MO, VEN); **Aragua**: prope Coloniam Tovar, 1854-1855, *A. Fendler 982* (K, MO); Chuao, 28 Jan 1965, *F. Matos 1076* (VEN); PN H. Pittier, 10-15 km E de Cata, 440-560 m, 12 Apr 1981, *G. Morillo et al. 8448* (VEN); east of Tejerías, 545 m, 30 May 1944, *J.A. Steyermark 56884* (F); **Carabobo**: entre Borburata y Patanemo, Jun 1961, *L. Aristeguieta 4442* (VEN); carretera Trincheras –El Cambur, 70 m, 23 Jun 1968, *C. Benítez de Rojas 330* (MY); **Distrito Federal**: Carretera Vieja Caracas-La Guaira, Jan 1965, *L. Aristeguieta 5415* (MO, NY, VEN); in hills above the Universidad Católica Andres Bello, Montalbán, 900-950 m, 13 Sep 1975, *P.E. Berry 1341* (MO, VEN); Cordillera de la Costa, Caraballeda, 20 Dec 1984, *K.S. Brown Jr. s.n.* (CORD, UEC 16612); Tierra Caliente, near Topo, *S.W.? Buschell s.n.* (K); El Valle, 25 Jun 1891, *H.F.A. von Eggers 13177* (CORD fragment, L, US); valley above Los Caracas, 2 Jul 1975, *A.H. Gentry & P.E. Berry14774* (MO, U, US); La Guayra, 7 May 1874, *O. Kuntze 1235* (K, NY); vicinity of Macarao, ca. 20 km west of Caracas, 20 Aug 1974, *A.A. Lasseigne 4450* (MO, NY); 32 km best of La Guaira, 4 km best of Anare, 10º38'N, 66º39'W, 6 Apr 1982, *R.L. Leisner & V. Medina 13444* (MO, MY, U, VEN); El Avila, entre Quebrada Quintero y Quebrada Paraíso, 1600 m, Aug 1976, *B. Manara s.n.* (VEN 113426); Bosque de Catuche, 1000 m, Jun 1917, *H. Pittier 7124* (VEN); Sabanas de Gambia, around Caracas, 950 m, 10 Jul 1921, *H. Pittier 9623* (NY, US, VEN); vicinity of Las Tricheras, road Caracas-La Guaira, 20 Jul 1922, *H. Pittier 10399* (GH, NY, US); vicinity of Macarao, 31 Aug 1924, *H. Pittier 11542* (G, NY, P VEN); hills above Antimano, 11 Dec 1927, *H. Pittier 12585* (G, GH, M, NY, US, VEN); Road to La Guaira, between Catia and Blandin, 30 May 1929, *H. Pittier 13366* (G, GH, M, MO, US, VEN); above Quebrada Ocumarito, SW of Catia La Mar, 67º1'20''N, 10º32'30''W, 700-730 m, 30 Jan 1980, *J.A. Steyermark & P. Colvée 121161* (MY, VEN); Barrancas, Jan 1941, *F. Tamayo 2006* (UC, US, VEN); El Valle, Hacienda Sosa, 11 Nov 1949, *Trujillo & Fernández 296* (MY, VEN); Avila, 1200 m, 15 Sep 1940, *C. Vogl 709* (F); **Falcón**: alrededores de Churuguara, 800 m, Dec 1953, *G. Losser & L. Aristeguieta 3438* (VEN); Bolívar, W of Carrizalito, 1200-1400 m, 19 Oct 1985, *H. van der Werff & R.C. Wingfield 7444* (MO, U); **Guarico**: Paso Real, selvas de Tamanaco, 11/12 Jun 1966, *L. Aristeguieta 6139* (MY, NY, VEN); **Lara**: Carretera entre Quíbor y El Blanquito, 2 km antes del Parque Nacional Yacumbú, 500 m, 31 Oct 1981, *C. Burandt Jr. V. 1003* (MY); Mun. Crespo, carretera Casa de Alto-La Trilla, 800 m, 10 Aug 1993, *C. Benítez de Rojas & M.J. Baldizán 4976* (MY); Duaca, bosque Barro Negro, 600 m, 11 Aug 1993, *C. Benítez de Rojas & M.J. Baldizán 4980* (MY); Carretera Sanare a El Tocuyo, 4 km de Sanare, 1200 m, 11 May 1982, *J. Casadiego 248* (MY); Carreta Barquisimeto-Duaca, paso de Tacarigua, cerca de Eneal, 25 Jul 1976, *G. Morillo et al. 4297* & *4299* (MY, VEN); Barquisimeto, Jan 1924, *J. Saer 109* (US); Serranías de Terepaima, 800-1000 m, Aug 1930, *J. Sauer s.n.* (F 690864); Agua Viva, Cerro Seroche, Mamonal, 15 Nov 1977, *R.F. Smith V. 8617* (VEN); Distr. Jiménea, Paso de Angostura, Sitio de represa de Yacambú, 500 m, 28-31 Jul 1973, *J.A. Steyermark & V. Carreño Espinoza 107636* (MO, VEN); Paso de Angostura, Represa de Yacambú, 9º41'N, 69º31'W, 500 m, Dec 1973, *J.A. Steyermark 108787* (US, VEN); vecindades de Puricaure, 500 m, 27 Mar 1975, *J.A. Steyermark 111696* (US, VEN); límite entre Lara y Yaracuy, Aug 1937, *F. Tamayo 281* (US, VEN); confluencia de Quebrada Honda con el Río Yacumbú, alrededores del "Paso de Angostura", 700 m, 2 Sep 1964,

Trujillo 6885 (MY); **Lara/Portuguesa**: Serranías de Terepaima, 800-1000 m, Aug 1930 m, *J. Saer 627* (F); **Mérida**: cerca de Bailadares, 1700-1800 m, 30 Apr 1976, *V.M. Badillo 7159* (MY); carretera Estanquez-Las Nieves, 850 m, 20 May 1993, *C. Benítez de Rojas et al. 4821* (MY); 30 km SW of Mérida along rd. to San Cristóbal, 1100 m, 23 Oct 1963, *F.J. Breteler 3207* (MO, NY, U, US, WAG) & 30 Jul 1964, *F.J. Breteler 4059* (MO, US, Z); road from Ejido (South of Merida) to El Morro, El Morro, Quebrada Mucusabache, 1680 m, 21 May 1988, *L.J. Dorr et al. 5275* (CAS, MEXU, VEN); Savannas de Lagunillas, 1000 m, Oct 1921, *A. Jahn 667* (US, VEN); Pueblo Nuevo, 16 Jun 1966, *S. López-Palacios 1302* (MO); La Enfadosa, 1550 m, 9 Jul 1968, *S. López-Palacios 2137* (MY); San Cristobal de Torondoy, 10 Dec 1973, *S. López-Palacios & J.A. Bautista B. 3557* (MO); Estanquez, Jan 1950, *G. Marcuzzi s.n.* (CORD, NY); carretera Palmarito/Cerro Verde, 1-3 km al S de Palmarito, 500-600 m, 5 May 1977, *G. Morillo & R. Smith 6537* (MY, VEN); Valle de Caracas, 10º30'N, 66º53'W, 1100 m, *N. Ramírez 1067* (MO, MY, VEN); Campo Elías, 1200 m, 6 May 1975, *L.E. Ruiz Terán et al. 12210* (MO); afluente de la Quebrada Baruta, 10º28'N, 66º51'40'' W, 900 m, 3 Aug 1975, *J.A. Steyermark & P. Berry 111788* (MO, VEN); alrededores de Baruta, 1100 m, 28 Aug 1949, *Trujillo et al. 247* (MY); Chiguará, 23 Mar 1964, *Trujillo 6244* (MY); El Morro, 13 May 1964, *Trujillo 6353* (MY); **Miranda**: Morros de la Guairita, 900-950 m, 16 Aug 1975, *P.E. Berry 1044* (MO, VEN); SE Valle de Caracas, 10º29'10''N, 66º52'30''W, 1100 m, 8 Jun 1994, *C. Grases 10* (MY); Dist. Sucre, Parque Recreacional "Cuevas del Indio, 10º26'30''N, 66º48'30''W, 1000-1200 m, 15 Aug 1984, *R. Ortiz et al. 591* (M, VEN); Guarenas valley, 4 Oct 1925, *H. Pittier 11905* (MO, US, Z); Colinas de Bello Monte, 1100 m, 10 Jun 1992, *N. Ramírez & M. López 3293 & 3294* (MY); Parque Nacional El Avila; vertiente sur, nor noreste de Caucagü, a lo largo de la quebrada Tacamahaca, 1000-1200 m, 3 Aug 1977, *J.A. Steyermark et al. 114061* (MO, NY, VEN); entre Sábana Grande y Baruta, 1100 m, *L. Williams* 10595 (US, VEN); **Monagas**: Piar, Aparicio, 300-580 m, 10 Nov 1995, *J.J. Calzadilla 2136* (UOJ); del mismo lugar, 9 Jun 1996, *J.J. Calzadilla 2704* (UOJ); alrededores de la Cueva del Guácharo, cerca de Caripe, 18 Jul 1972, *K. Dumont et al. 7778* (NY, VEN); PNEG, Cerro La Cueva, 1000-1100 m, 1990/1991, *M.E. de Vásquez 385* (UOJ); **Nueva Esparta**: Is. Margarita, El Valle, 27 Jul 1901, *O.O. Miller & J.R. Johnston 35* (BM, K, MO, NY, P, US); **Portuguesa**: Puente sobre el Río Saguaz, 450 m, 3 Jun 1993, *C. Benítez de Rojas et al. 4898* (MO, MY); **Sucre**: above Las Piedras, 900 m, 14 May 1945, *J.A. Steyermark 62772* (CORD, F, VEN); along Río Guagua tributary to Río Manzanares, above valley of Cumanacoa, 230-300 m, 18 May 1945, *J.A. Steyermark 62814* (CORD, F, MO); **Táchira**: cerca de Pericos, unos 7 kms al sur-oeste de San Cristóbal, 850-900 m, 22 Aug 1985, *J. Bono 5167* (MO); Independencia, distrito Capacho, 15 Jul 1992, *L. Cárdenas 3936* (MY); 7 km W of Rubio, 900-1000 m, 18 Mar 1981, *R.L. Liesner & Á.C. González 10728* (MO, VEN); between Las Dantas and Las Adjuntas, 13km northwest of Ruio, 920-950 m, 27 Jul 1979, *J.A. Steyermark et al.118828* (MO, VEN); Sra. El Casadero, 13 km N of rubio, between Las Dantas and Las Adjuntas, 7º43'N, 72º23'W, 900-1050 m, *J.A. Steyermark et al. 120128* (MO, VEN); **Trujillo**: Jajó, Aug 1958, *L. Aristaguieta 3372* (US, VEN); entre Biscucuy y Campo Elías, km 48-49, 1000 m, 22 May 1971, *C. Benítez de Rojas 970* (MY); Mun. Urdaneta, ca. 3 km antes de Jajó, 30 Apr 1993, *C. Benítez de Rojas & F. Rojas 4786* (MO, MY, NY); Caserío Carmania, El Cucharito, 900 m, 16 May 1988, *J. Bono 6707* (MY); Distr. Bocono, La Morita, ca. 9º22'N, 70º02'W, 2300 m, 4 Jun 1988, *L.J. Dorr et al. 5390* (CAS, MEXU, MY, NY, VEN); Vic. of Escuque, 10 Jan 1929, *H. Pittier 13132* (US, VEN).

Cultivated

ARGENTINA. Córdoba: Capital, cult. invernadero IMBIV (Ciudad Universitaria-UNC), semillas de *Y. Sánchez García 20*, Táchira (Venezuela), *M. Scaldaferro 73* (CORD).

41. Capsicum schottianum Sendtn.

BRAZIL. Without locality, *C.F.P. von Martius 3074* (BR, CORD fragment); Brasilia, *H.W. Schott 5426* (CORD, F 874709, MO, W 0074667; W 0074665); **Minas Gerais**: São Sebastião, Parque Estadual da Serra do Mar, estrada da Limeria, 23º44’95’’S, 45º32’71’’W, 600 m, 19 Apr 2000, *N.M. Ivanauskas et al. 4532* (BHCB); Caldas, Pocinhos do Rio Verde, 21 Jan 1980, *A. Krapovickas & C. Cristóbal 35425* (CTES, MO); Ouro Preto, 25 Jan 1951, *A. Macedo 3079* (CORD fragment, LIL); Juiz de Fora, Morro do Imperador, 11 Jan 2002, *D.S. Pifano & A.S.M. Valente 266* (BHCB, CESJ, RB, SPF); Monte Belo, Fazenda Lagoa, 1981, *M.C.W. Vieira 117* (RB); **Rio de Janeiro**: Petrópolis, 6.3 km do entroncamento BR 40 (Km 77), 22º28’S, 43º18’W, 1180 m, 9 Apr 1986, *L. Bianchetti et al. 409* & *410* (CEN, CORD); a 8.3 km do entroncamento BR 40 (km 77)-Estrada Roscio/Pati do Alferes, 22º28’S, 43º18’W, 1220 m, *L. Bianchetti et al. 411* (CEN, CORD); a 9,3 km do entroncamento BR 40 (km 77)-estrada Roscio/Pati do Alferes, 22º28’S, 43º18’W, 1200 m, 9 Apr 1986, *L. Bianchetti et al. 415* (CEN, CORD); 12.8 km do entroncamento da BR 40 (Km 77)-Estrada Roscio/Pati do Alferes, 22º27’S, 43º20’W, 1070 m, 9 Apr 1986, *L. Bianchetti et al. 416* (CEN, CORD); Parque Nacional de Tijuca, trilha do Bico do Papagaio, 22º56’53’’S, 43º17’30’’W, 680 m, 18 May 1999, *L. Bianchetti et al. 1561* (CEN); Petropolis, Estrada para Paty de Alferes (via Roscio), a 10,6 km da BR 040, na divisa Miguel Pereira-Petropolis, vizinho à Reserva Biológica do Tinguá, 22º29’15’’S, 43º18’11’’W, 1210 m, 19 May 1999, *L. Bianchetti et al. 1563* (CEN); Guapimirim, Granja Monte Olivete, afluente do rio Bananal, trilha das Andorinhas, 300-350 m, 18 Jan 1995, *M.G. Bovini et al. 695* (BHCB, RB); Granja Monte Olivete, trilha das Andorinhas, 270 m, 20 Dec 1995, *M.G. Bovini et al. 959* (CORD, RB); Nova Friburgo, Macaé de Cima, Sítio Bacchus, trilha próxima a casa que possui o encanamento, 1300-1600 m, 22º22’28’’S, 42º29’40’’W, 1 May 2001, *M.G. Bovini et al. 1993* (CEN, RB); Granja Monte Olivete, margen do rio Bananal, 400-500 m, 17 Nov 1993, *J.M.A. Braga et al. 867* (CORD, RB); Teresópolis, near a rivulet on the eastern side of the Fazenda do Pacaqué [Paquequer] in the Serra dos Orgaõs, [20 Feb 1826], *W.J. Burchell 2428* (K); Barra Mansa, Fazenda Paraizo, 18 Jan 1961, *A.P. Duarte 5491* (CORD, RB); Tijuca, 19 Dec 1868, *A. Glaziou 3074 pp* (CORD, P); Serra dos Orgãos, May 1839, *M. Guillemin 939* (CORD, P); Corcovado, ca. 600 m, *A.T. Hunziker 19577* (CORD: 3 sheets, P); Petropolis, a casi 6 km de la unión de BR 40 (km 77) con la estrada entre Rocío y Pati de Alferes, ± 1200 m, *A.T. Hunziker 25165* (CORD, NY); 9,3 km de la unión de BR 40 (km 77) con la ruta entre Rocio-Pati do Alferes, 1200 m, 9 Apr 1986, *A.T. Hunziker 25167* (BM, CORD); ca. 12,8 km de la unión de BR 40 (km 77) con la estrada entre Rocío y Pati de Alferes, ± 1070 m, *A.T. Hunziker 25168* (CORD); Açude do Camorim, Jacarepaguá, 3 Jan 1933, *J.G. Kuhlmann s.n.* (CORD 00087798, RB 61773); São José do Barreiro, Parque Nacional da Bocaina, 1700 m, 5 Mar 1977, *G. Martinelli 1107* (CORD, RB); Corcovado, 10 Nov 1947, *A. Pereira Duarte 979* (CORD, RB); Mun. Nova Friburgo, Macaé de Cima, margem do Rio das Flores, 1150 m, 25 Nov 1986, *S. de V.A. Pessôa et al. 127* (F, RB, P); Mun. Resende, Faz. Sáo Damiao, R. Pirapitinga, ca. 450 m, 25 Feb 1987, *R.B. Pineschi 42* (CORD, GUA); Parque Nacional de Tijuca, trilha do Bico do Papagaio, 800 m, 23 Nov 1984, *R. Ribeiro 563* (CORD, GUA);

Corcovado, Oct 1832, *L. Riedel 1080* (NY, US); Bom Retiro ao Pico da Tijuca, 7 Nov 1946, *M. Rosa 75* (R) & 19 Dec 1946, *M. Rosa 118* (R: 3 sheets); Camorim, 19 Dec 1933, *A. Sampaio et al. s.n.* (CORD 000087799, R 113699, hoja B); Therezopolis, Apr 1917, *A. Sampaio 2363* (R); alagado da Estrada Teresópolis-Friburgo, 800 m, 4 Apr 1970, *D. Sucre & P.I.S. Braga 6511* (CEN, RB); Tijuca, Trapicheiro, 1944, *M.H. Valle 90* (CORD fragment, R); Teresopolis, Fazenda da Boã Fé, 10 Jan 1943, *H.P. Velloso 158* (CORD, R); Nova Friburgo, Macaé de Cima, Sítio de David Miller, trilha do bambu, 22º22'S, 42º30'W, 1450 m, 5 May 2008, *P.L. Viana & A.R. Barbosa 3500* (BHCB); **São Paulo**: Bertioga, desde Bertioaga, por SP 098, rumbo a Moji das Cruzes, en el km. 86, hacia mano derecha, 23º42'53''S, 46º02'03''W, 450 m, 25 Feb 2006, *G.E. Barboza et al. 1638* (CORD); Bananal, a unos 20 km al sur de Bananal, por SP 247, rumbo a Sertão do Bocaina, 22º48'20''S, 44º20'20''W, 1190 m, 26 Feb 2006, *G.E. Barboza et al. 1650* (CORD); Ubatuba, desde Ubatuba rumbo a Taubaté, entre Km 84/85, 23º22'35''S, 45º07'07''W, 286 m, 17 Apr 2008, *G.E. Barboza et al. 2032* (BM, CORD) & *2033* (CORD, MO, NY); desde Ubatuba rumbo a Taubaté, entre Km 82/83, 290 m, 17 Apr 2008, *G.E. Barboza et al. 2034* (CORD, SI) & *2035* (CORD); Ubatuba rumbo a Taubaté, al lado de un Puente, unos kms antes del inicio de la cuesta, 17 Apr 2008, *G.E. Barboza et al. 2036* & 2037 (CORD, NY); Santo André, Reserva Biológica do Alto da Serrra de Paranapiacaba, justo detrás del casco principal de la reserva, 23º46'S, 46º19'W, 756 m, 24 Sep 2010, *G.E. Barboza & M.T. Cosa 2519* (CORD); Paranapiacaba, por el camino de tierra rumbo al poblado de Paranapiacaba, 23º46'14''S, 46º18'41''W, 775 m, 25 Sept 2010, *G.E. Barboza & M.T. Cosa 2526* (CORD); Reserva Paranapiacaba, sobre trilha 17, hacia la 4 de la casa interna, 23º46'47''S, 46º18'37''W, 845 m, 7 May 2012, *G.E. Barboza et al. 3633, 3634* & *3635* (CORD); camino de tierra desde la Reserva Biológica do Alto da Serra da Paranapiacaba hacia Paranapiacaba, 23º46'15''/23º46'17''S, 46º18'13''/46º18'12''W, 784/810 m, 7 May 2012, *G.E. Barboza et al. 3636* & *3637* (CORD); Paranapiacaba, salida del pueblo, 23°46'22,7''S, 46°18'16,3''W, 799 m, 2 Apr 2018, *G.E. Barboza & R. Deanna 5006* & *5007* (CORD); desde Paranapiacaba rumbo a Moji das Cruzes, vía subestación Tijuco Preto, 23°46'31,6''S, 46°17'45,4''W, 884 m, 2 Apr 2018, *G.E. Barboza & R. Deanna 5009* (CORD); desde Ubatuba rumbo a Taubaté, ca. Km 85, 23°22'36,0''S, 45°07'02,9''W, 312 m, 3 Apr 2018, *G.E. Barboza & R. Deanna 5014* (CORD); desde Ubatuba rumbo a Taubaté, Km 82,650, 23°22'15,7''S, 45°07'08,3''W, 607 m, 3 Apr 2018, *G.E. Barboza & R. Deanna 5015* (CORD); desde Ubatuba rumbo a Taubaté, ca. Km 81.7, 23°22'05,1''S, 45°07'26,9''W, 723 m, 3 Apr 2018, *G.E. Barboza & R. Deanna 5017* (CORD); Monterio Lobato, rumbo a Monteiro Lobato desde Caçapava, 22°59'36,6''S, 45°47'36,1''W, 736 m, 3 Apr 2018, *G.E. Barboza & R. Deanna 5019* (CORD); Biritiba Mirim, entre primer y segundo puente, antes de llegar a la Estação Biológica de Boracéia, 23º38'37.4''/23º38'49.6''S, 45º54'52.9''/45º54'46.3''W, 826-859 m, 30 Mar 2019, *G.E. Barboza 5042 & 5043* (CORD); Natividade da Serra, na encosta interior da Serra do Mar, 14 Dec 1985, *F. de Barros 1230* (CORD fragment, RB, SP); Bananal, SP 247 (Bananal-Bocaina do Bananal), a 12,8 km da prefectura Mun. de Bananal, 22º45'S, 44º21'W, 1220 m, *L. Bianchetti et al. 438* (CEN; = *A.T. Hunziker 25179*, CORD); SP 247 (Bananal-Bocaina do Bananal), a 24 km da prefeitura, 22º49'S, 44º26' W, 1200 m, 12 Apr 1986, *L. Bianchetti et al. 439* (CEN, CORD; = *A.T. Hunziker 25180*, BM, CORD); Ubatuba, BR-125 (Ubatuba-Taubate), km 82, 23º23'S, 45º08'W, 585 m, 14 Apr 1986, *L. Bianchetti et al. 476* (CEN; = *A.T. Hunziker 25190*, CORD); Caraguatatuba, estrada Caraguatatuba-São Jose dos Campos (SP 99), km 72,5, 23º29'S, 45º35'W, 500/570 m, 15 Apr 1986, *L.*

Bianchetti et al. 480 & *481* (CEN, CORD); Bertioaga, SP 98, km 88 Estrada Moji das Cruzes-Bertioga, 330 m, 14 Apr 1986, *L. Bianchetti et al. 489* (CEN, = *A.T. Hunziker 25192*, CORD); Salesópolis, trilha dos Pilões, 23º39'15''S, 45º53'23''W, 840 m, 5 May 1999, *L. Bianchetti et al. 1535* (CEN); EB Boraceia, trilha da estrada interna, a 5,8 km do alojamento da USP, no sentido USP-guarita, 23º38'49''S, 45º56'23''W, 840 m, 6 May 1999, *L. Bianchetti et al. 1540* (CEN); Bairro Alto, Estrada para Natividade da Serra, a 200 m da Rodovia dos Tamoios, no sentido Tamoios-Bairro Alto, 23º34'53''S, 45º27'30''W, 780 m, 7 May 1999, *L. Bianchetti et al. 1544* (CEN); São Luís do Paraitinga, Rodovia O. Cruz-São Luís do Paraitinga-Ubatuba (SP 125), na altura do km 71, 23º30'09''S, 45º05'52''W, 920 m, *L. Bianchetti et al. 1545* (CEN); Estação Experimental de Boracéa, 26 Apr 1940, *N.G. Blanco s.n.* (BHCB 77019, IAC 557, SP); Paranapiacaba, trilha dos Jipeiros, 22 Jan 2008, *F. Búgola-Silva 8041* (NY, SP); Campinas, May 1918, *J. de Campos Novaes s.n.* (US 1616682); Campinas, Jun 1918, *J. de Campos Novaes 170* (US); Cunha, ao lado da EFE, 23º18'S, 45º09'W, 1180 m, Jul 1982, *J.E. Collares 45* (RB); Estacão Experimental Ubatuba (EEU), 27 Sep 1938, *A. Costa & Viegas 4904* (SP); Estação Biológica de Boracéia, 23º38'S, 45º53', 890-950 m, 10 May 1983, *A. Custodio Filho 1327* (FLOR, NY, SP); Santo André, Reserva Biológica do Alto da Serra de Paranapiacaba, 23º47'64''S, 46º18'78''W, 996 m, 23 Feb 2005, *E.R. Forni-Martins et al. 05/01, 05/03* & *05/10* (CORD, UEC); Estacão Experimental Ubatuba, 4 Nov 1985, *C. Franco et al. 4390* (CORD, SP); Estrada de Itamambuca, km 35 da rodovia Rio/Santos, 14 Apr 1994, *A. Furlan et al. 1527* (SP, UEC); Paranapiacaba, trihla do Rio Mogi, que permeia os limites da Estação Biologica, 23º46'49,16''S, 46º18'32,74''W, 794 m, 13 Oct 2009, *L.L. Giacomin et al. 1107* (BHCB, CORD); Reserva Biológica do Alto da Serra de Paranapiacaba, margens da estrada que leva da entrada à Casa do pesquisador, 23°46'41''S, 46°18'44''W, 800 m, 19 Nov 2013, *L.L. Giacomin et al. 2014* (BHCB); EB de Boracéia, trilha dos Pilões, 1 Apr 2000, *S.A.P. de Godoy et al. 2047* (RB); Serra do Mar, Estación Biológica de Paranapiacaba, ca. 850 m, *A.T. Hunziker 19558* (CORD, MO); Salesópolis, Estação Biológica de Boracéia (EBB), Boracéia, 27 Nov 1948, *M. Kuhlmann 1710* (CORD, SP); Estação Experimental Boracéa, 27 Nov 1940, *A.S. Lima & L. da Silva 5894* (BHCB); Serra da Bocaina, 22º48'S, 44º26'W, 1200 m, 14 Dec 2006, *L.R. Lima et al. 389* (SP); Paranapiacaba, Estação Biológica, 5 Mar 1962, *J. Mattos 11459* (SP); São Paulo, 25 Feb 1874, *H. Mosén 1563* (P, S); Paranapiacaba, 8 Jan 1967, *T.M. Pedersen 7787* (CORD); São José do Barreiro, PN da Bocaina, divisor de águas entre o Rio Mambucaba e o Rio Veado, 21 Jul 1994, *L. Rossi & E.L.M. Catharino 1605* (SP, UEC); Eldorado, Parque Estadual de Jacupiranga, Núcleo Caverna do Diablo, trilha do Bugio, 24°38'43''S, 48°23'36''W, 400 m, 23 Mar 2005, *M.B. dos Santos et al. 6* (RB); Estação Experimental Boracéa, 21 Nov 1940, *L. da Silva s.n.* (BHCB 77043, IAC 5832, SP 48601); Canelinha, EEU, 1 Aug 1940, *C. Smith s.n.* (CORD 00087797, SP 43647); Biritiba–Mirim, Estação Biológica de Boracéia, 23°39'10''S, 45°53'07''W, 861 m, 24 Oct 2007, *J.R. Stehmann et al. 4813* (BHCB); EB do Alto da Serra de Paranapiacaba, Picada 9, 24 Mar 1995, *M. Sugiyama et al. 1293* (SP); Estacão Experimental Ubatuba, *A.P. Viegas 3509* (SP); Cunha, P.E. da Serra do Mar-Núcleo Cunha, beira de Córrego/trilha do Rio Bonito, 12 Dec 1996, *L.F. Yamamoto et al. 2* (ESA, SP, SPSF, SPF);

Cultivated

BRAZIL. Minas Gerais: Viçosa, cultivado en Viçosa por Prof. V. Casali, 7-14 Dec 1986, *A.T. Hunziker 25240* (seeds from *L. Bianchetti et al. 499*), *25241* (seeds from *L. Bianchetti et al. 500*) & *25242* (seeds from *L. Bianchetti et al. 501*) (CORD).

42. Capsicum tovarii Eshbaugh, P.G.Sm. & Nickerent
PERU. Huancavelica: in chacra and on scree slopes around Huanchuy, 8 km upstream from bridge where Huanta road crosses the Río Mantaro, 28 Mar 1971, *B. Pickersgill 401* (RNG); Mantaro valley between Pampas and Salcabamba, 1800 m, 16 Apr 1962, *P.G. Smith Ac 2017* (leg. O. Tovar) (DAV, MU); Tayacaja, arriba de Chejyacc, entre Salcabamba y Surcubamba, margen izquierda del Mantaro, 1800 m, 18 Apr 1962, *O. Tovar 3750* (USM); Quintabamba, Huachocolpa, valle del Mantaro, 850 m, 21 Apr 1963, *O. Tovar 4114* (MU, US); Huanchuy, cerca de Mayoc, 2450 m, Apr 1965, *O. Tovar 5012* (USM); same locality, 8 Apr 1966, *O. Tovar 5363* (USM); **Junín**: Huancayo, Dist. Santo Domingo de Acobambo, Potrero, -12.042814S, -74.803880W, 8 May 2019, *G.E. Barboza 5044* (CORD); abajo de Pariahuanca, 2000 m, 5 May 1979, *O. Tovar 7865* (USM); Huancayo, May 1999, *O. Tovar s.n.* (CORD 00087964).

Cultivated
ARGENTINA. **Córdoba**: Capital, cult. Museo Botánico, semillas de Perú, Junín, Huancayo (leg. O. Tovar, May 1999), 23 Jan 2001, *A.T. Hunziker 25653, 25654* & *25655* (CORD).

UNITED STATES OF AMERICA. **Ohio**: Oxford, grown MU (Miami University) greenhouse, seeds from Peru, Ayacucho, on slopes of hills north of Huanta, 2000 m (leg. H.G. Marshall, Apr 1971), 28 Aug 1975, *W.H. Eshbaugh E-1137* (MU, US).

43. Capsicum villosum Sendtn.
BRAZIL. Espírito Santo: Venda Nova do Imigrante, Mata Fria, 1800 m, 17 Jan 1995, *G. Hatschbach & J.M. Silva 61582* (MBM); **Minas Gerais**: Itamonte, PNI, outskirts of park on road toward Agulhas Negras, turning off from BR-485 to Itamonte, 22º22’25’’S, 44º45’16’’W, 1728 m, 6 May 2011, *M.F. Agra et al. 7400* (BHCB, CORD); Itamonte, BR-354, km 778, 22º22’S, 44º48’W, 1500 m, 11 Apr 1986, *L. Bianchetti et al. 429* (CEN, CORD); same locality and date, *L. Bianchetti et al. 430* & *431* (CEN, CORD; = *A.T. Hunziker 25174* & *25175*, CORD); Itamonte, Serra da Mantiqueira, BR-354, Km 774, 1330 m, *L. Bianchetti et al. 432* (CEN, CORD); BR-354, km 775, 1380 m, 11 Apr 1986, *L. Bianchetti et al. 433* (CEN, CORD; = *A.T. Hunziker 25176*, CORD); Ouro Preto, Parque Estadual do Itacolomi, Lagoa Seca, 1612 m, 27 may 2009, *E.S. Cândido et al. 178* (OUPR); same locality, 1437-1549 m, 13 Jan 2010, *E.S. Cândido et al. 290 & 298* (OUPR); Sertão, 20º25.741S, 43º27.460’W, 1447, 23 Apr 2010, *E.S. Cândido et al. 404* (OUPR); Itamonte, margens da BR-354, 5 Nov 2008, *L.L. Giacomin et al. 373* (BHCB); Ouro Preto, região da Lagoa Seca, ca. 20º26’9’’S, 43º29’31’’W, 1600 m, 5 Feb 2009, *F.O.A. Lemes & G.P. Freitas 602* (ORPR); Araponga, Fazenda da Gramma, trail to Araponga, about 12 km, 1000 m, 6 Feb 1930, *Y. Mexia 4328* (A, BM, CAS, F, G, GH, K, MO, NY, U, UC, US, Z); Itamonte, Serra de Pivú, 1900 m, 9-13 Apr 1879, *Netto et al.* s.n. (Glazoiu, Rangel & Schwacke) (R 25775, US 1570320); Province de Minas Gerais, *A. de Saint-Hilaire 584* & *621* (CORD fragments, P); Minas Gerais, 1879, *C.A.W. Schwacke s.n.* (RB, US 02827210);

São Gonçalo do Río Abaixo, 11 Jan 1989, *J.R. Stehmann & Perdigaõ s.n.* (BHCB 21176, SPF); Bandeiras, Fazenda Boi Rajado, 5.9 km E of Macarani-Bandeiras road on farm road ca. 12 km N of Bandeiras (at Faz. Canada), 15º48'23''S, 40º31'05''W, 830-850 m, 30 Jan 2004, *W.W. Thomas et al. 13660* (CEPEC, NY); Ouro Preto, região da Lagoa Seca, 16 Dec 2010, 1600 m, *A.C. Tonaco & J.M. Lanna 137* (OUPR); Araponga, Serra da Grama, estrada para Bom Jesus da Madeira, veira de estrada à ca. 100 m da casa sede da Fazenda da Neblina, 9 Oct 1986, *M.F. Vieira et al. 475* (CORD, VIC); Estouro, Morro do Brigadeiro, próximo a fazenda, 13 Feb 1985, *W.N. Vidal et al. 633* (CORD, VIC); **Rio de Janeiro**: Santa Maria Madalena, Pedra Dubois, 1100 m, 27 Feb 1976, *W.R. Anderson 11718* (CAS, MBM, NY); Itatiaia, 1972, *D. de Andrade-Lima 72-7202 & 72-7203* (IPA); Parque Nacional Itatiaia (PNI), a unos 2 km de la entrada principal, 26 Feb 2006, *G.E. Barboza et al. 1653* (BM, CORD); PNI, en los alrededores de la Cachoeira Véu da Noiva, 27 Feb 2006, *G.E. Barboza et al. 1655* (CORD); PNI, 18 Feb 1960, *O.M. Barth I 214* (US); Cachoeira Veu de Noiva, 1050 m, 22º25'S, 44º37'W, 11 Apr 1986, *L. Bianchetti et al. 417* & *418* (CEN, CORD); a 1-1,5 km antes de chegar a Cachoeira Veu de Noiva, 22º25'S, 44º37'W, 1040 m, 11 Apr 1986, *L. Bianchetti et al. 420* (= *A.T. Hunziker 25169*, CORD) & *422* (CEN, CORD); PNI, BR 354, km 2,5 estrada via Dutra-Caxambu, 22º24'S, 44º45'W, 1450 m, 11 Apr 1986, *L. Bianchetti et al. 425* (CEN); Parque Nacional Itatiaia, a 2,5 km na trilha para a torre de televisão, a partir da parada Maromba, 22º26'06''S, 44º37'28''W, 1120 m, 15 May 1999, *L. Bianchetti et al. 1557* (CEN); Itatiaia, km 2-3 do Registro, na Nova Estrada, 25 Mar 1942, *W.D. Barros 728* (RB); Mangaratiba, Reserva Rio das Pedras, trilha do Cambucá, 70-310 m, 23 Nov 1999, *M.G. Bovini et al. 1721* (RB); PNI, Estrada Nova, km 1, 25 Mar 1942, *A.C. Brade 17263* (RB) & *17463* (LIL, RB); PNI, trilha do Hotel Simon para o Tres Picos, 22º15'S, 44º34'W-22º28'S, 44º45'W, 1200 m, 23 Nov 1994, *J.M.A. Braga et al. 1613* (RB); PNI, estrada para Pico das Agulhas Negras, 22º15'-22º28' S, 44º34'-44º45' W, 1900-1950 m, 15 Feb 1995, *J.M.A. Braga et al. 2005* (HRB, MO, RB); PNI, trilha para Cachoeira de Itaporani, rio Campo Belo, 1050 m, 18 Feb 1995, *J.M.A. Braga et al. 2134* (RB); PNI, trilha do Hotel Simon para o Tres Picos, 7 Dec 1995, *J.M.A. Braga et al. 3076* (NY, RB); PNI, Cachoeira Poranga, 22º15'/22º28'S, 44º34'/ 44º45' W, 900 m, 4 Dec 1996, *J.M.A. Braga et al. 3730* (RB); PNI, trilha do Hotel Simon para o Tres Picos, 14 Jan 1997, *J.M.A. Braga et al. 3801* (BHCB, JPB, RB); PNI, proximidades do Abrigo III, 22º15'S, 44º34'W/22º28'S, 44º45'W, 1000 m, 23 Jan 1997, *J.M.A. Braga et al. 3898* (NY, RB); Itatiaia, 950 m, 20 Dec 1996, *C.H.W. Flechtmann s.n.* (ESA 87139); PNI, trilha para Serra da os Três Picos, 22º26'7''S, 44º36'27''W, 1113 m, 19 May 2010, *C.N. Fraga et al. 2877* (NY, RB); PNI, Pico das Agulhas Negras, 25 Jan 1978, *L. Freire de Carvalho s.n.* (RB 427524); PNI, trilha para Cachoeira Itaporani, 22°25'38''S, 44°37'14''W, 1145 m, 23 May 2017, *Y.F. Gouvêa 213* (BHCB); PNI, beira do início da trilha "Três Picos", 22°22'22''S, 44°45'16''W, 1055 m, 27 May 2017, *Y.F. Gouvêa 227* (BHCB); PNI, trilha do Véu da Noi, 1129 m, 16 Oct 2009, *E.K.O. Hattori et al. 917* (BHCB); Mantiqueira, PNI, en Maromba, ca. 1100 m, 5-6 Dec 1967, *A.T. Hunziker 19566* (BM, BHCB, CORD, MBM, MO, NY, P, SI, UEC) & *19567* (BM, CORD, MO, NY); PNI, ca. 1 km antes de llegar a Cachoeira Veu de Noiva, 1040 m, 11 Apr 1986, *A.T. Hunziker 25173* (BM, CORD); Lago Azul, 1938, *L. Lanstyak 49* (RB); Itatiaia, km 11, 28 Nov 1938, *F. Markgraf & A.C. Brade 3753* (CORD, RB); Marombas, 14 Mar1967, *J. Mattos & M. Mattos 14776* (CORD, RB, SP); Maromba, Apr 1972, *B. Pickersgill 464* & *464-3* (CORD, MBM); PNI, inicio da Estrada para o Pico das Agulhas Negras, 22º15'S, 44º34'W/22º28'S, 44º45'W, 1700 m, 20 May 1999, *A. Quinet et al. 231* (BHCB, NY, RB); PNI, Estrada para

o Abrigo Macieiras, 22º15'-22º28'S, 44º34'-44º45' W, 1300 m, 31 May 1996, *S.J. Silva Neto et al. 736* (BHCB, JPB, RB); PNI, trilha para Cachoeira Itaporani, 18 Feb 2003, *S.J. Silva Neto et al. 1795* (BR, NY); Serra do Itatiaia, na subida da Serra, 1500 m, 5 Jan 1896, *E. Ule 635* (CORD, R); im Walde des Abhanges der Serra do Itatiaia, 1600 m, Jan 1896, *E. Ule 3744* (CORD); Itatiaya-Gebiet, Subtropischer Regenwald am Wege Maromba-Macieiras, ca. 1200-1800 m, 23 Oct 1927, *H. Zerny s.n.* (W); Mun. Teresópolis, Sierra des Orgues, *A-C. Vauthier s.n.* (P 00482080); **São Paulo**: inter São Paulo & Barbacons, *A. André 3317* (K, NY); Cunha, Parque Estadual da Serra do Mar, estrada do rio Bonito e Paraibuna, 29 Mar 1994, *J.B. Baitello 547* (CEN, UEC, SP); São José do Barreiro, Parque Nacional Serra do Bocaina, en la trilha du Ouro rumbo hacia la cachoeira San Isidro, 22°44'28,6''S, 44°36'58,3''W, 1519 m, 4 Apr 2018, *G.E. Barboza & R. Deanna 5026 bis* (BM, CORD, MO, NY); Bananal, SP 247 (Bananal-Bocaina do Bananal), a 24 km da Prefeitura Municipal de Bananal, 22º49'S, 44º26'W, 1200 m, 12 Apr 1986, *L. Bianchetti et al. 444* (CEN, CORD; = *A.T. Hunziker et al. 25181*, CORD); Paraibuna, Estrada SP 88, Km 108,5, 23º24'S, 45º40'W, 870 m, 15 Apr 1986, *L. Bianchetti et al. 487* (CEN, CORD); Salesópolis, Estação Biológica de Boracéia, estrada interna a 6.9 km do alojamento da USP, no sentido USP-guarita, 23º38'49''S, 45º56'23''W, 7 May 1999, *L. Bianchetti et al. 1539* (CEN); Estação Biológica de Boracéia, beira da estrada a 1.4 km da guarita 1 SABESP, sentido Salesópolis, 23º38'02''S, 45º57'35''W, 820 m, 7 May 1999, *L. Bianchetti et al. 1543* (CEN); Parque Nacional da Serra da Bocaina, trilha do poço, próximo a sede, 22º44'04''S, 44º36'59''W, 1420 m, 9 May 1999, *L. Bianchetti et al. 1549* (CEN); São Paulo, Butantã, Parque Previdência, 9 Mar 1984, *M. Bittar & J. Bessa 90* (PMSP); São José do Barreiro, 17 Feb 1998, *L. Freitas 268* (CORD); beira do Rio Mambucaba, próximo a Cachoeira Sto. Isidro, 16 Mar1999, *L. Freitas 615* (CORD, UEC); Itaquaqueçetuba, 1 Mar 1939, *A. Gehrt s.n.* (CORD 00086115 & 00086116, CTES, RB 318112, SP 39983, U 0115626); núcleo Cunha, trilha para o rio Bonito, 23º13'28"-23º16'10"S, 45º02'53"-45º05'15" W, 1100 m, 19 Mar 1996, *M. Kirizawa et al. 3288* (PMSP, SP, UEC); Guararema, 11 Dec 1951, *M. Kuhlmann 2741* (SP); Alto Tieté, camino para Rio Claro, 25 Oct 1901, *A. Loefgren 5878* (SP, US); Estação Biológica de Boracéia, area seguindo o curso do Tieté, abaixo das Nascentes, 27 Dec 1996, *S.A. Nicolau et al. 1237* (SP); Barra do Turvo, Bela Vista, 6 Apr 2002, *O.S. Ribas et al. 4624* (MBM, SP); núcleo Cunha, trilha da Barra do Rio, 12 Dec 1996, *J.P. Souza et al. 793* (SP); Reserva Florestal da Bocaina, 1600 m, 5 May 1968, *D. Sucre et al. 2907* (RB); Sertão, 3 Oct 1940, *A.P. Viégas s.n.* (IAC 5741); Reserva do Florestal, 11 Feb 1981, *M.G.L. Wanderley 264* (CAS, MBM, NY, SP).
